# Synchronized age-related gene expression changes across multiple tissues in human and the link to complex diseases

**DOI:** 10.1038/srep15145

**Published:** 2015-10-19

**Authors:** Jialiang Yang, Tao Huang, Francesca Petralia, Quan Long, Bin Zhang, Carmen Argmann, Yong Zhao, Charles V. Mobbs, Eric E. Schadt, Jun Zhu, Zhidong Tu, The GTEx Consortium, Kristin G. Ardlie, David S. Deluca, Ayellet V. Segrè, Timothy J. Sullivan, Taylor R. Young, Ellen T. Gelfand, Casandra A. Trowbridge, Julian B. Maller, Taru Tukiainen, Monkol Lek, Lucas D. Ward, Pouya Kheradpour, Benjamin Iriarte, Yan Meng, Cameron D. Palmer, Wendy Winckler, Joel Hirschhorn, Manolis Kellis, Daniel G. MacArthur, Gad Getz, Andrey A. Shablin, Gen Li, Yi-Hui Zhou, Andrew B. Nobel, Ivan Rusyn, Fred A. Wright, Tuuli Lappalainen, Pedro G. Ferreira, Halit Ongen, Manuel A. Rivas, Alexis Battle, Sara Mostafavi, Jean Monlong, Michael Sammeth, Marta Mele, Ferran Reverter, Jakob Goldmann, Daphne Koller, Roderic Guigo, Mark I. McCarthy, Emmanouil T. Dermitzakis, Eric R. Gamazon, Anuar Konkashbaev, Dan L. Nicolae, Nancy J. Cox, Timothée Flutre, Xiaoquan Wen, Matthew Stephens, Jonathan K. Pritchard, Luan Lin, Jun Liu, Amanda Brown, Bernadette Mestichelli, Denee Tidwell, Edmund Lo, Mike Salvatore, Saboor Shad, Jeffrey A. Thomas, John T. Lonsdale, Christopher Choi, Ellen Karasik, Kimberly Ramsey, Michael T. Moser, Barbara A. Foster, Bryan M. Gillard, John Syron, Johnelle Fleming, Harold Magazine, Rick Hasz, Gary D. Walters, Jason P. Bridge, Mark Miklos, Susan Sullivan, Laura K. Barker, Heather Traino, Magboeba Mosavel, Laura A. Siminoff, Dana R. Valley, Daniel C. Rohrer, Scott Jewel, Philip Branton, Leslie H. Sobin, Liqun Qi, Pushpa Hariharan, Shenpei Wu, David Tabor, Charles Shive, Anna M. Smith, Stephen A. Buia, Anita H. Undale, Karna L. Robinson, Nancy Roche, Kimberly M. Valentino, Angela Britton, Robin Burges, Debra Bradbury, Kenneth W. Hambright, John Seleski, Greg E. Korzeniewski, Kenyon Erickson, Yvonne Marcus, Jorge Tejada, Mehran Taherian, Chunrong Lu, Barnaby E. Robles, Margaret Basile, Deborah C. Mash, Simona Volpi, Jeff Struewing, Gary F. Temple, Joy Boyer, Deborah Colantuoni, Roger Little, Susan Koester, NCI Latarsha J. Carithers, Helen M. Moore, Ping Guan, Carolyn Compton, Sherilyn J. Sawyer, Joanne P. Demchok, Jimmie B. Vaught, Chana A. Rabiner, Nicole C. Lockhart

**Affiliations:** 1 Institute of Genomics and Multiscale Biology, Icahn School of Medicine at Mount Sinai, NY, 10029, USA; 2 Department of Genetics and Genomic Sciences, Icahn School of Medicine at Mount Sinai, NY, 10029, USA; 3 Department of Neuroscience, Icahn School of Medicine at Mount Sinai, NY, 10029, USA; 4 Department of Geriatrics and Palliative Medicine, Icahn School of Medicine at Mount Sinai, NY, 10029, USA; 5 Department of Medicine, Endocrinology, Diabetes and Bone Disease, Icahn School of Medicine at Mount Sinai, NY, 10029, USA; 6 The Broad Institute of Massachusetts Institute of Technology and Harvard University, Cambridge, Massachusetts 02142, USA; 7 Analytic and Translational Genetics Unit, Massachusetts General Hospital, Boston, Massachusetts 02114, USA; 8 MIT Computer Science and Artificial Intelligence Laboratory, Massachusetts Institute of Technology, Cambridge, Massachusetts 02139, USA; 9 Department of Genetics, Boston Children’s Hospital, Boston, Massachusetts 02115, USA; 10 Cancer Center and Department of Pathology, Massachusetts General Hospital, Boston, Massachusetts, 02114, USA; 11 Center for Biomarker Research and Personalized Medicine, Virginia Commonwealth University, Richmond, Virginia 23298, USA; 12 Department of Statistics and Operations Research and Department of Biostatistics, University of North Carolina, Chapel Hill, North Carolina 27599, USA; 13 Bioinformatics Research Center and Departments of Statistics and Biological Sciences, North Carolina State University, Raleigh, North Carolina 27695, USA; 14 Department of Environmental Sciences and Engineering, University of North Carolina, Chapel Hill, NC 27599; 15 Department of Veterinary Integrative Biosciences, Texas A&M University, College Station, Texas 77843, USA; 16 Department of Genetic Medicine and Development, University of Geneva Medical School, 1211 Geneva, Switzerland; 17 Institute for Genetics and Genomics in Geneva (iG3), University of Geneva, 1211 Geneva, Switzerland; 18 Swiss Institute of Bioinformatics, 1211 Geneva, Switzerland; 19 Department of Genetics, Stanford University, Stanford, California 94305, USA; 20 New York Genome Center, New York, New York 10011, USA; 21 Department of Systems Biology, Columbia University Medical Center, New York, New York 10032, USA; 22 Wellcome Trust Centre for Human Genetics Research, Nuffield Department of Clinical Medicine, University of Oxford, Oxford, United Kingdom OX3 7BN; 23 Department of Computer Science, Stanford University, Stanford, California 94305, USA; 24 Department of Computer Science, Johns Hopkins University, Baltimore, Maryland 21218, USA; 25 Centre for Genomic Regulation (CRG), Dr. Aiguader 88, 08003 Barcelona, Spain; 26 Universitat Pompeu Fabra, 08003 Barcelona, Catalonia, Spain; 27 Human Genetics Department, McGill University, H3A 0G1 Montréal, Canada; 28 National Institute for Scientific Computing, Petropolis 25651-075 Rio de Janeiro, Brazil; 29 Department of Stem Cell and Regenerative Biology, Harvard University, Cambridge, Massachusetts 02138, USA; 30 Universitat de Barcelona, 08028 Barcelona, Catalonia, Spain; 31 Radboud University Nijmegen, Netherlands; 32 Institut Hospital del Mar d’Investigacions Mèdiques (IMIM), 08003 Barcelona, Spain; 33 Oxford Centre for Diabetes, Endocrinology and Metabolism, University of Oxford, Churchill Hospital, Oxford, United Kingdom OX3 7LJ; 34 Oxford NIHR Biomedical Research Centre, Churchill Hospital, Oxford, United Kingdom OX3 7LJ; 35 Section of Genetic Medicine, Department of Medicine and Department of Human Genetics, University of Chicago, Chicago, Illinois 60637; 36 Department of Human Genetics, University of Chicago, Chicago, Illinois 60637, USA; 37 INRA, Department of Plant Biology and Breeding, AGAP, Montpellier, 34060, France; 38 Department of Biostatistics, University of Michigan, Ann Arbor, Michigan 48109, USA; 39 Department of Statistics, University of Chicago, Chicago, Illinois 60637, USA; 40 Department of Genetics and Biology, Stanford University, Stanford, California 94305, USA; 41 Howard Hughes Medical Institute, Chicago, Illinois, USA; 42 Department of Statistics, Harvard University, Cambridge, Massachusetts 02138; 43 National Disease Research Interchange, Philadelphia, Pennsylvania 19103, USA; 44 Roswell Park Cancer Institute, Buffalo, New York 14263, USA; 45 Science Care, Inc., Phoenix, Arizona, USA; 46 Gift of Life Donor Program, Philadelphia, Pennsylvania 19103, USA; 47 LifeNet Health, Richmond, Virginia 23227, USA; 48 UNYTS, Buffalo, New York 14203, USA; 49 Virginia Commonwealth University, Richmond, Virginia 23298, USA; 50 Department of Public Health, Temple University, Philadelphia, Pennsylvania 19122, USA; 51 Van Andel Research Institute, Grand Rapids, Michigan 49503; 52 Biorepositories & Biospecimen Research Branch, National Cancer Institute, Bethesda, Maryland 20892, USA; 53 National Institutes of Health, Bethesda, Maryland 20892, USA; 54 Biospecimen Research Group, Clinical Research Directorate, Leidos Biomedical Research, Inc., Rockville, Maryland 20852, USA; 55 Sapient Government Services, Arlington, Virginia 22201-2909; 56 Brain Endowment Bank, Department of Neurology, Miller School of Medicine, University of Miami, Miami, Florida 33136, USA; 57 Division of Genomic Medicine, National Human Genome Research Institute, Bethesda, Maryland 20892, USA; 58 Division of Genomics and Society, National Human Genome Research Institute, Bethesda, Maryland 20892, USA; 59 Office of Science Policy, Planning, and Communications, National Institute of Mental Health, Bethesda, Maryland 20892, USA; 60 Division of Neuroscience and Basic Behavioral Science, National Institute of Mental Health, Bethesda, Maryland 20892, USA; 61 Cancer Diagnosis Program, National Cancer Institute, Bethesda, Maryland 20892, USA

## Abstract

Aging is one of the most important biological processes and is a known risk factor for many age-related diseases in human. Studying age-related transcriptomic changes in tissues across the whole body can provide valuable information for a holistic understanding of this fundamental process. In this work, we catalogue age-related gene expression changes in nine tissues from nearly two hundred individuals collected by the Genotype-Tissue Expression (GTEx) project. In general, we find the aging gene expression signatures are very tissue specific. However, enrichment for some well-known aging components such as mitochondria biology is observed in many tissues. Different levels of cross-tissue synchronization of age-related gene expression changes are observed, and some essential tissues (e.g., heart and lung) show much stronger “co-aging” than other tissues based on a principal component analysis. The aging gene signatures and complex disease genes show a complex overlapping pattern and only in some cases, we see that they are significantly overlapped in the tissues affected by the corresponding diseases. In summary, our analyses provide novel insights to the co-regulation of age-related gene expression in multiple tissues; it also presents a tissue-specific view of the link between aging and age-related diseases.

Aging is a certainty in our largely uncertain lives. It is a process in which multiple organs and tissues gradually lose physiological integrity, followed by functional impairment and eventually death of the individual^1^. The molecular mechanisms underlying aging are not fully understood, despite the enormous amount of findings and theories that have emerged in the past decades. The current hypotheses encompass genetic predisposition, calorie restriction, mitochondrial dysfunction, telomere attrition, genomic instability, and many others^2^,^3^,^4^,^5^,^6^. As there is also no unanimous agreement on fundamental issues such as whether aging is genetically programmed^7^,^8^, the ultimate cause of aging and the interconnections among various aging mechanisms remain to be established. On the contrary is the fact that aging is a major risk factor for many complex diseases such as cardiovascular disease, cancer, Type 2 diabetes, Alzheimer’s disease, and Parkinson’s disease^9^,^10^,^11^,^12^,^13^. Given the rapidly expanding aging population world-wide^14^, aging research is increasingly important as it holds the promise for unravelling the secrets of longevity and for bringing new solutions to the treatment of age-related diseases.

With the advent of various high throughput technologies, it is now feasible to measure an individual’s panomics (including transcriptome, metabolome, epigenome, etc.) at a reasonable cost^15^. The rich information in panomic data brings enormous opportunities to the aging research field. For example, using methylation data, Horvath *et al.* defined a molecular clock composed of 353 CpG sites that could accurately predict the human age^16^. By examining the transcriptome changes in the aging neocortex and cerebellum in mice, Lee *et al.* observed genes associated with inflammatory responses, oxidative stress, and reduced neurotrophic support in both brain regions^17^. The AGEMAP project which profiled gene expression in 16 tissues in mice also identified age-associated genes and revealed tissue specific aging patterns^18^. By comparing the transcriptional profiles in mice to those of other species (human, flies, and worms), genes involved in the electron transport chain showed common age regulation in all four species. A large number of human tissue age-gene expression association studies have been performed in various tissues (e.g., brain, muscle, blood, and kidney)^19^,^20^,^21^,^22^,^23^,^24^,^25^. However, the previous gene expression based studies only examined a rather limited number of tissue types. Due to difference in sample collections, platforms used for profiling, and data processing procedures, it is difficult to compare and combine the findings from these studies. The GTEx project provides RNA-Seq based transcriptome profiles in more than 40 tissues from hundreds of human donors of various ages, making it one of the largest single data sets with the most comprehensive tissue types for studying the genetics of human tissue gene expression and age-associated gene expression^26^. Particularly, since multiple tissues are collected from the same individuals, cross-tissue analysis of age-associated gene expression changes becomes feasible. For simplicity we subsequently refer to such genes as “aging genes”.

In this work, we first identify aging gene signatures in nine GTEx tissues and explore their functional characteristics. We then study the synchronization of age-related gene expression changes across different tissues. We also study the connections between tissue aging and complex diseases. In addition, we examine the aging gene signatures across different species, and compare our results with other related aging studies.

## Results

### Identification of tissue specific age-associated genes in the GTEx data

**Figure 1:**
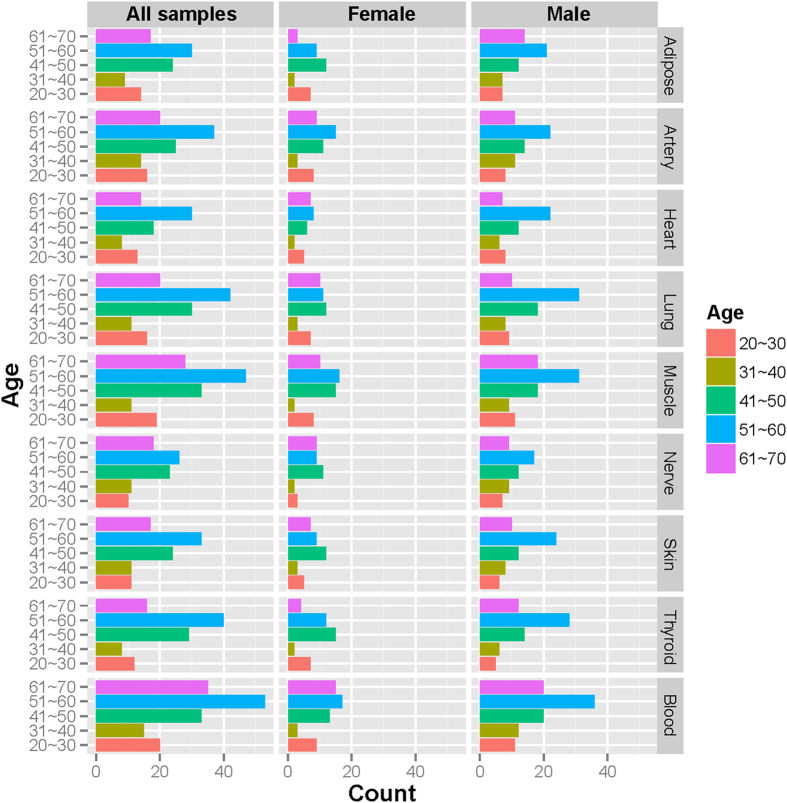
Age distribution of donors in nine tissues. The histograms of donor age distribution for all samples, females only, and males only.

The GTEx project (v3, accessed in December 2012) provided 1,641 whole transcriptome profiles in more than 40 tissues from nearly two hundred post-mortem human donors^26^. Nine tissues had sample sizes of greater than 80, namely, the subcutaneous adipose, tibial artery, left ventricle heart, lung, skeletal muscle, tibial nerve, skin, thyroid, and whole blood. We considered these nine tissues in our study and omitted other tissue types which had fewer samples. The sample age and gender distributions were plotted in Fig. 1, in which the age distributions of all samples, female samples, and male samples were shown in the left, middle, and right columns, respectively and each row corresponds to a tissue type. Overall, donors’ chronological ages ranged from 20 to 70. A customized regression model combined with bootstrapping was used to define age-gene expression associations (see Methods).

Briefly, we followed the GTEx consortium’s practice of pre-processing the gene expression data with a slight modification^26^. We corrected gender, the top three genotype principal components (PCs) to reduce the impact of population structure, and a few top gene expression PCs that did not significantly correlate with age (p-value > 0.05). Correcting gene expression PCs allows us to remove potential confounding factors such as the batch effect^27^. Multiple alternative models of correcting expression PCs were considered and compared (see details in Methods). Since low expressed genes are usually more vulnerable to measurement errors^28^, we removed 20% low expressed aging genes. We bootstrapped the samples for 100 times and compiled our final aging gene list by including genes whose expression levels were significantly associated with age in at least 50 runs. The readers are referred to Methods for the details.

We summarized the numbers of age-associated genes in all nine tissues in Table 1 and the detailed information for all the 41,298 genes was provided in Supplementary Data S1. As shown in Table 1, the number of age-associated genes in the nine tissues ranged from 3 to 3,287. The largest number (n = 3,287) was observed in whole blood; while only 12 and 3 age-associated genes were identified in skin and thyroid, respectively. The large difference in the number of age-associated genes observed in the GTEx data is consistent with previous observations in mouse^18^ and human^22^. To estimate the number of false positives that could be contained in our aging genes, we permuted sample ages for 1,000 times and repeated the aging gene identification procedure on the permutated data (see Methods). The results were summarized in the column named “Permutation” in Table 1. As shown in Table 1, the frequencies of identifying equal or more age-associated genes in the permuted datasets are very small (no greater than 5 times except for skin and thyroid), and the numbers of false positives are small compared to the number of findings in the real data in most tissues (skin and thyroid are the two exceptions), indicating that majority of the identified gene-age associations in the seven tissues are not due to random chance.

**Figure 2:**
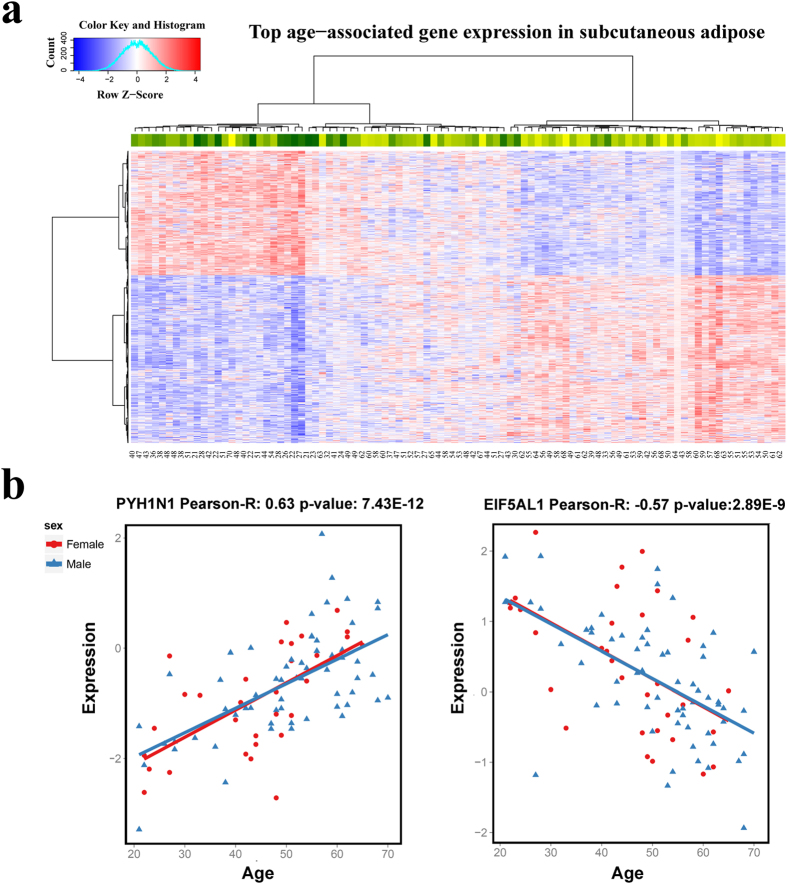
Age-associated gene expression in subcutaneous adipose tissue. (**a**) Heatmap of 1,134 age-associated genes (row) on 94 samples (column). Colors represent normalized gene expression values with blue for low expression and red for high expression. The age of each individual is displayed at the bottom and also illustrated in color bar at the top with dark green for young and yellow for old. (**b**) Scatter plot of 2 representative age-associated gene expression patterns PYH1N1 and EIF5AL1 in adipose tissue. Pearson-R value in the title represents the Pearson correlation coefficient between gene expression and age across all samples. The solid blue triangles plot male samples and solid red circle female samples. Similarly, the blue and red lines denote the regression lines for male and female samples, respectively.

Because of the small number of age-associated genes and high false positive rates in the skin and thyroid tissues, we excluded them and only performed further analysis on seven tissues. To visualize the expression pattern of the inferred aging genes, a heatmap was generated for each tissue (Supplementary Fig. S1). As shown in Fig. 2a, for the adipose tissue, the samples were clearly clustered into two groups based on the Euclidian distance with “Ward” measurement^29^. The left-side group contained many younger individuals compared to the right-side group. The apparent separation of “young” and “old” group was observed in all seven tissues and it is of note that the groupings were different in different tissues (Supplementary Fig. S1). A student’s t-test on the age groups, “young” and “old”, generated significant results (p-values less than 3.59 × 10^−3^) in all seven tissues (Supplementary Table S1). The age distributions of “young” and “old” samples in the seven tissues were provided in Supplementary Fig. S2.

In addition to the grouping on the age axis, the aging genes were also grouped into up-regulated and down-regulated genes, which correspond to positive and negative signs of the coefficient of “age” term in the regression model. In four out of the seven tissues, we saw more up-regulated aging genes than down-regulated ones (heart, lung, and blood are exceptions) (Table 1). To visualize the age-gene expression association at single gene level, we selected two genes with either strong positive or negative age-association (PYH1N1 and EIF5AL1) in adipose tissue and showed the scatter plots in Fig. 2b. The Pearson correlation coefficients of these two genes are 0.63 (p-value = 7.43 × 10^−12^) and −0.57 (p-value = 2.89 × 10^−9^), respectively. As can be seen in Fig. 2b, the age-gene expression association is evident for these two genes and there is no apparent difference between male (blue) and female (red). The scatter plots of the top 100 age-associated genes in all seven tissues are provided in Supplementary Data S2.

### Functional annotation of aging genes points to a large collection of biological processes

To obtain a functional overview, we annotated the up- and down-regulated aging genes separately, using David tools^30^. Due to space limitation, we showed a subset of the top representative annotations in Table 2, and provided a complete list in Supplementary Data S3. The aging gene signatures are significantly enriched for a wide spectrum of Gene Ontology (GO) terms and pathways. The most frequently appeared category is mitochondrion. In five out of seven tissues (adipose, artery, heart, lung, and blood), the enrichment was all seen in the down-regulated aging genes. This result supports the central role of mitochondria in human aging. Mitochondria dysfunction in aging has been observed in multiple model organisms and is among the most recognized aging theories^1^,^31^,^32^,^33^. For example, a systemic RNA interference (RNAi) screen for gene inactivation that increases lifespan in worms showed a 10-fold overrepresentation of genes encoding mitochondrial proteins^34^. Many other functions known to associate with aging were also observed, e.g., down-regulation of electron transport chain (in adipose and heart) and up-regulation of cell death and inflammation response (both in artery). It is of note that several disease pathways, e.g., “hsa05016: Huntington’s disease”, “hsa05012: Parkinson’s disease”, and “hsa05010: Alzheimer’s disease” are significantly enriched in aging gene signatures in multiple tissues, and these neurodegenerative diseases are also known to be age-dependent^12^,^13^,^35^.

Besides the well-known age-related functions, we also saw multiple processes that are less known for their involvement in aging. For example, we observed the up-regulation of cell adhesion in nerve, and up-regulation of ion binding in adipose. All these significantly over-represented biological functions suggest that human aging is an extremely broad and complex process, with both common and specific, up- and down-regulated collections of biological activities in various tissues.

### Synchronization of multi-tissue age-related gene expression changes in human

As shown in Fig. 2a and Supplementary Fig. S1, although the chronological ages of the tissue donors are largely consistent with the “young” or “old” clustering, there are some apparent “outliers”. For example, the 15^th^ adipose sample (from the left) in Fig. 2a is from an individual with an old chronological age of 70, but is clustered with the “young” group where most donors range in age from 20 to 50. This indicated that the molecular age of adipose tissue from this individual is about 30 years younger than its chronological age. Sample mislabelling can be excluded as a possible cause for such “outliers”, due to the accurate sample matching using variants called from RNA-Seq data and DNA genotype data. In fact, this finding is consistent with previous observations in the mouse data from the AGEMAP project^18^. Although AGEMAP concluded that different tissues in the same mouse tend to have coordinated aging, they also observed “outliers” or mouse tissues that clustered into different aging patterns. They hypothesized that individual mice could be composed of a mosaic of tissues with different physiological ages.

One question we asked is whether age-related expression changes in different tissues are coordinated. It is of note that our study design is not longitudinal, therefore we are unable to study the coordinated aging gene expression changes among tissues in single individuals. Instead, we studied this at the population level with the assumption that common patterns of age-related expression changes exist in human population and can be observed in our samples. To answer the aforementioned question, we estimated tissues’ apparent ages or their relative ages (ranks) in the population, and then we calculated their covariation in the population for each tissue-pair. Specifically, we considered two methods: (1) an unsupervised method based on principal component analysis (PCA) and (2) a supervised learning method using the Elastic Net regression^36^.

### PCA analysis

Briefly, we calculated the PCs of aging gene expression in each tissue. In all seven tissues, the first PC (PC1) captured the dominant variance of the aging gene expression and sample coordinate on PC1 was highly correlated with the sample’s chronological age with a mean Spearman correlation coefficient of 0.55 (Supplementary Fig. S3). Therefore we only considered PC1 to estimate the relative rank of tissue’s apparent age.

**Figure 3:**
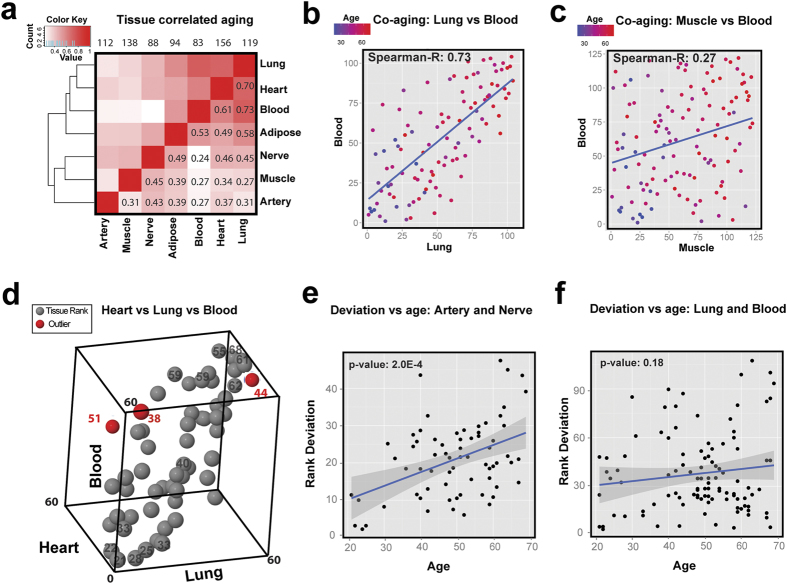
Aging synchronization in multiple tissues. (**a**) Heatmap of tissue co-aging in adipose, artery, heat, lung, muscle, nerve, and blood tissues. The number in each square in the lower triangle indicates the co-aging coefficient between a tissue pair; and the sample number for each tissue is also presented in the top of the sample column. (**b**) Scatter plot of age rank correlation for two highly correlated tissues lung and blood and the regression line. Spearman-R value represents the Spearman correlation coefficient between the ranks defined by the two tissues across all samples. (**c**) Scatter plot of age rank correlation for two relatively uncorrelated tissues muscle and blood and the regression line. (**d**) Scatter plot of age rank correlation for heart, lung, and blood; the axis ranges from 0 to 60 indicating the rank of each sample (in total 59 donors with data in all these 3 tissues); the chronological ages are marked for some representative samples and the outliers are highlighted in red. (**e**) Correlation between age rank deviation and age in artery and nerve. The shaded area indicates the confidence interval of the regression line. “p-value” indicates the p-value for regression coefficient being deviated from 0. (**f**) Correlation between rank deviation and age in lung and blood.

As we performed such calculation in each tissue separately, different tissues from the same individual may have different age ranks among donors. We then calculated the Spearman correlation coefficient of two tissues’ apparent ages across all the individuals, named as “co-aging coefficient”. A high co-aging coefficient indicates that the tissue pair has tight synchronized age-related expression changes in the population, so that if we randomly select an individual and profile the tissue pair, when one tissue appears to be young, the other tissue has a high chance of being young too, or vice versa. We plotted all pair-wise tissue correlations in Fig. 3a. As shown in Fig. 3a, high correlations were seen among lung, blood, and heart (e.g., the correlation between lung and blood was 0.73), whereas much less correlations were observed among other tissues (e.g., the correlation between muscle and blood was only 0.27) (Fig. 3b,c).

One potential cause for observing the synchronization among tissue specific age-related gene expression changes is cross-tissue contamination, in which case gene expression profiled from one tissue is a mixture of gene expression from multiple tissues (e.g., heart tissues might be contaminated with blood). To evaluate the level of contamination, we performed a clustering of samples based on gene expression using (1) all genes and (2) all aging genes. We observed that the samples collected from the same tissue were grouped tightly together in either case (see Supplementary Fig. S4a and S4b). This indicated that cross-tissue contamination may not be the main cause for the strong correlation of age-related gene expression changes amongst certain tissues.

The tight co-aging amongst the lung, heart, and blood tissues is better visualized in Fig. 3d, in which we mapped each overlapping sample (across the three tissues) into a ball in 3 dimensional space according to its rank (based on the coordinate of PC1 projection) in each tissue. It is clear that most samples showed synchronized aging in these three tissues (ball points in grey color), as they are aligned with the diagonal line connecting (0, 0, 0) and (60, 60, 60) and positioned closely to their chronological ages. A couple of outliers are also seen, as they are far off the diagonal line indicating large deviation from donors’ chronological ages. We labelled samples with deviation p-values less than 0.05 as outliers and colored them in red (see Methods for details). We also observed that deviation has a positive correlation with chronological age (see Supplementary Table S2), as we saw greater age deviations in old individuals than those in young individuals. This is particularly true for tissue pairs with relatively weak synchronization. For example, for the artery and nerve tissues (Fig. 3e), the deviation of sample age rank increases significantly as age increases (p-value = 2.04 × 10^−4^); but for the lung and blood tissues (Fig. 3f), although the correlation remains positive, it is not significant (p-value = 0.18).

### Predicted age using Elastic Net

As an alternative approach to PCA, we predicted tissue age using Elastic Net (EN) and performed tissue co-aging analysis using the predicted ages. Briefly, we randomly divided samples into 10 subgroups of equal size and predicted sample age in each subgroup using data from the other 9 subgroups. To reduce bias due to random sampling, we repeated the process for 100 times and the mean of the predicted age in these runs was used as sample’s predicted age (see Supplementary Methods for details). The tissue co-aging patterns based on EN were plotted in Supplementary Fig. S5. As can be seen, the pattern is different from the one obtained by PCA method. Using EN, the most correlated tissue pair is nerve and artery (Spearman correlation coefficient of 0.76), while in the PCA analysis, apparent ages of heart, lung, and blood are highly correlated. As a supervised learning method, Elastic Net is designed to minimize the difference between the predicted age and chronological age in the training samples. As a consequence, we observed that the Spearman correlation between Elastic Net predicted age and chronological age was consistently higher than that in the PCA results for all tissues (Supplementary Table S3). The apparent “outliers” seen in the PCA analysis were predicted to be less dramatic by the EN. For example, the 70 years old adipose tissue was ranked at 13^th^ position in all the 94 samples based on its PC1 projection (smaller rank corresponds to younger age), while based on EN, it was ranked more to the donor’s chronological age (36^th^ position). In Supplementary Fig. S5, we also listed the rooted mean square error (RMSE) of EN prediction for each tissue. With current small sample size, EN showed large errors, e.g., artery and nerve showed RMSEs of 6.75 and 7.20 respectively, while blood tissue showed the largest RMSE of 11.94.

Despite the difference in co-aging patterns observed in PCA and EN analyses, both methods showed that the aging of artery and nerve is strongly correlated with chronological age and this correlation is much smaller for blood, suggesting different tissues may have different levels of deviation from the individual’s chronological age (Supplementary Table S3).

### Tissue specific link between aging genes and complex disease genes

Aging is a known risk factor for many diseases, such as cardiovascular disease, cancer, arthritis, Alzheimer’s disease, Parkinson’s disease, and Type 2 diabetes^37^,^38^,^39^. The functional enrichment in the previous section indicated that aging gene signatures have clear connections with some age-related diseases like Alzheimer’s disease. To comprehensively evaluate the tissue specific connections between aging and diseases, we compiled a large disease gene list containing 234 disease/trait categories by merging two datasets, the NIH GWAS^40^ and OMIM^41^ catalog (see Methods for details).

**Figure 4:**
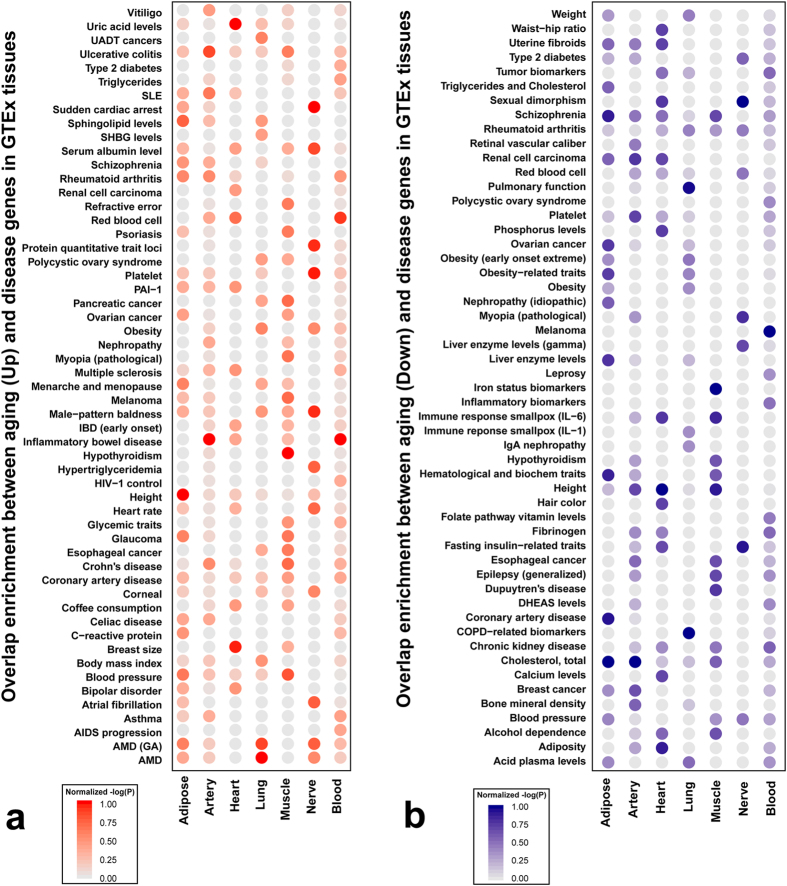
Correlation between age and common diseases in multiple tissues. (**a**) Overlap enrichment between up-regulated aging genes and disease genes in GTEx tissues and (**b**) Overlap enrichment between down-regulated aging genes and disease genes in GTEx tissues. The color depth indicates the normalized negative logarithm of the p-value of the Fisher’s exact test for overlapping between disease and aging genes in specific tissue.

Using this gene set, we determined the enrichment of the disease genes in tissue specific aging gene signatures considering the up- and down-regulated gene signatures separately. A large number of diseases showed tissue specific connections with aging genes. To visualize the result, we selected the top 10 diseases/traits that showed the most significant enrichment for aging genes in each tissue. By considering the unique diseases/traits in all seven tissues, we obtained 55 diseases that showed significant over-representation in up-regulated aging genes, and 53 diseases that showed significant over-representation in down-regulated aging genes. The results for up-regulated aging genes and down-regulated aging genes were shown in Fig. 4a,b, respectively, and the full enrichment analysis results were provided in Supplementary Data S4. As shown in Fig. 4, in some cases, the over-representation of disease genes in tissue specific aging gene signatures was observed in the tissue types that are commonly considered as the disease affected tissue. For example, the down-regulated aging gene signature in the lung shows most significant enrichment in genes associated with chronic obstructive pulmonary disease (COPD)-related biomarkers and pulmonary function, with p-values of 2.51 × 10^−5^ and 3.40 × 10^−5^, respectively. The total cholesterol associated genes and obesity-related traits are significantly over-represented in the adipose tissue down-regulated aging genes with p-values of 8.32 × 10^−3^ and 2.84 × 10^−2^, respectively. It is of note that some of these p-values may not survive the multiple testing correction, and could represent false positive results. In addition, a large number of immune-mediated inflammatory disease genes are over-represented in the blood and artery aging gene signatures, including Crohn’s disease, inflammatory bowel disease, multiple sclerosis, rheumatoid arthritis, and ulcerative colitis. Chronic low-grade systemic inflammation is a common manifestation of aging^42^,^43^, and our results further support the strong connection between aging and inflammatory diseases in the human population. On the other hand, some enrichment results are less intuitive, for example, disease genes of age-related macular degeneration (AMD), a disease of the eye, appeared to be over-represented in the up-regulated aging genes of the lung (Fig. 4a). Overall our results suggest that the connections between aging and diseases are very complex. Although we observed some direct connections between disease and tissue type, many connections could be indirect and thus undetectable from simple enrichment analysis. Aging and complex diseases could also be fundamentally different, as some individuals can have a long period of disease free aging life.

### Aging genes in mouse are different from human aging genes from GTEx

Mouse models have been widely used to study human diseases. Although multiple studies have employed various mouse models to study aging^18^,^44^, it is not clear whether at the molecular level, mouse aging is comparable to human aging. To address this question, we compared the human aging genes derived from the GTEx data with mouse aging genes obtained from the AGEMAP project^18^. The AGEMAP project identified mouse age-associated genes in nine tissues: adrenals, cerebellum, eye, gonads, heart, lung, spleen, spinal cord, and thymus, among which two tissues (i.e., heart and lung) were also profiled by GTEx.

To do a cross species comparison, we first obtained the homologous mapping from homologene database in NCBI (released in 12/14/2012) ( ftp://ftp.ncbi.nih.gov/pub/HomoloGene/). Based on this mapping, 6,454 and 6,576 homologous (human/mouse) gene pairs were identified in the heart and lung data, respectively from both GTEx and AGEMAP (Supplementary Data S5). Among the 6,454 homologous genes, there are 346 aging genes (p-value < 0.001, similar to^18^) in the human heart (Supplementary Data S5) and 18 aging genes in the mouse heart^18^. Similarly, there are 324 aging genes in the human lung (Supplementary Data S5) and 66 aging genes in the mouse lung^18^. There is only one common age-associated gene DAZAP1 in both human and mouse heart tissue (p-value = 0.63, one-tail Fisher’s exact test). DAZAP1 (DAZ Associated Protein 1) is required for normal growth and spermatogenesis in mice^45^,^46^ and is deleted in many azoospermic and severely ligospermic men^47^. A recent study showed that DAZAP1 regulates the splicing of Crem, Crisp2, and Pot1a transcripts^48^. Similarly, there are only three common age-associated genes PTPN6, CDKN1C, and CLIP1 (mapped to Hcph, Cdkn1c, and Rsn in mouse) in lung tissue, the p-value for the one-tail Fisher’s exact test is 0.64.

Overall, our results suggest a very large difference in aging genes between the human and mouse, a finding consistent with results from previous studies^18^,^49^. The large difference in the aging gene pattern has also been observed in other animals like chimpanzee^50^, suggesting the aging process is indeed less well conserved across species as compared to some other biological processes^51^. It is of note that the study designs including sample size, gender distribution, age distribution, and experiment conditions are quite different between the AGEMAP and GTEx studies. This might contribute in part to the observed large difference and further investigation is required to ensure that the dissimilarity of the two species is not due to design artefacts.

### Comparison with other human age-focused gene expression studies

Glass *et al.* performed an analysis on the Multiple Tissue Human Expression Resource (MuTHER) data to identify aging genes in skin, blood, brain, and adipose tissues^22^. Three tissues are related to GTEx tissues, namely, adipose (subcutaneous fat), skin, and lymphoblastoid cell lines (LCLs) from blood.

We applied our model to the MuTHER dataset, and compared the identified aging genes with those from GTEx. As shown in Supplementary Table S4, 157 common aging genes were identified in adipose tissue (enrichment p-value = 1.51 × 10^−4^). In contrast, only 4 common aging genes were identified in skin and blood tissues, respectively (enrichment p-values were both 0.17). The common aging genes were listed in Supplementary Data S6.

We also compared aging genes we derived from the MuTHER dataset using our method with those reported by GLASS *et al.*^22^. They showed a significant overlap with p-values of 1.39 × 10^−177^, ≈0 (< 4.9 × 10^−324^), and 2.06 × 10^−7^ respectively in adipose, skin, and blood (see Supplementary Data S7). This implies that the large difference of aging genes in the skin and blood between MuTHER and GTEx data is inherent to the data themselves, rather than driven by the difference in the analytic methods. It is of note that in MuTHER project, peripheral blood samples were collected, and LCLs were generated through EBV-mediated transformation of the B-lymphocyte component, while in GTEx, the whole blood tissues were used for gene expression profiling. Therefore, it is likely that the difference in aging genes from LCLs vs GTEx whole blood is at least partially due to the sample difference. In addition, the skin tissues were collected from different parts of the body in GTEx (sun exposed lower leg) and MuTHER (relatively photo-protected infra-umbilical skin). It indicates that even the same tissue may have different aging patterns given a different environment such as sun light exposure. Other factors, such as the different gene expression platforms may also contribute to the gross differences in aging genes observed in the different human studies.

## Discussion

In the present study, we present a holistic view of human aging in multiple tissues by analysing the GTEx data. From GO and pathway enrichment analyses, mitochondrion biology is highlighted as the most commonly regulated biological process associated with age. We also observed many other biological processes associated with age such as DNA repair, electron transport chain, and apoptosis. With extensive involvement of a large number of biological processes during aging, an important task is to identify the connections amongst these aging components and reveal the causal relationship among them and identify the key driver genes as candidates for anti-aging drug development.

Through tissue co-aging analysis, we show that tissue aging as reflected by the age-related gene expression changes is potentially synchronized at different levels. In PCA analysis, vital tissues like lung, heart, and whole blood showed tighter co-aging pattern compared to other tissues like muscle. The different levels of tissue age-related expression change synchronization may reflect the different selection pressures on the functional connections among tissues in the early developmental stage which extends into the late stage of our lifespan.

The high correlation between blood and heart/lung provides a hint that circulation system could play a role in synchronizing tissue functions with respect to aging. Recent studies have shown that blood from young mice and possibly GDF11, a circulating transforming growth factor–β (TGF-β) family member can reverse cardiac hypertrophy^52^, stimulate brain vascular remodelling and increase neurogenesis in aging mice^53^.

It is of note that the co-aging pattern derived from PCA and Elastic Net are different. This difference may be caused by the limited sample size that is currently available for EN prediction. A more robust evaluation is possible when GTEx project completes with more than 900 individuals being profiled.

Our study identifies some apparent connections between aging and complex diseases. However, there are a large number of connections between tissue specific aging and diseases that are much less obvious and some are hard to interpret. Clearly, a more detailed study is required to fully understand the mechanistic details of all the connections between aging and diseases.

## Methods

### GTEx data

GTEx data (v3, December 2012 release) provides expression levels of 41,298 genes in nine human tissues: subcutaneous adipose, tibial artery, left ventricle heart, lung, skeletal muscle, tibial nerve, skin (from sun exposed lower leg), thyroid, and whole blood. The sample size of each tissue ranges from 83 to 156 (see Table 1). Since GTEx consortium paper^26^ provides full information on sample collection, RNA collection, RNA-Seq experiment, gene expression estimation, quality control, and gene expression normalization, we did not reproduce such information here.

### Linear regression model for aging gene detection

In each tissue, we modelled gene expression using the following linear regression model:


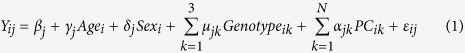


where, *Y*_*ij*_ is the expression level of gene *j* in sample *i, Age*_*i*_ denotes the age of sample *i, Sex*_*i*_ denotes the sex of sample *i, Genotype*_*ik*_(1 ≤ *k* ≤ 3) denotes the value of the *k*-th principal component value of genotype profile for the *i*-th sample, *PC*_*ik*_(1 ≤ *k* ≤ *N*) denotes the value of the *k*-th principal component value of gene expression profile for the *i*-th sample, *N* is the total number of top PCs under consideration, *ε*_*ij*_ is the error term, *β*_*j*_ is the regression intercept (for gene *j*), *γ*_*j*_ is the age regression coefficient, *δ*_*j*_ is the sex regression coefficient, *μ*_*jk*_(1 ≤ *k* ≤ 3) is the regression coefficient for the *k*-th genotype PC, and *α*_*jk*_(1 ≤ *k* ≤ *N*) is the regression coefficient for the *k*-th gene expression PC. For each gene *j*, a least square approach was used to estimate the regression coefficients. If *γ*_*j*_ was significantly deviated from 0, gene *j* was considered to be age-associated. Gene *j* was up-regulated with age if *γ*_*j*_ > 0 and down-regulated if *γ*_*j*_ < 0. We performed the false discovery rate (FDR) adjustment on the p-values using Benjamini Hochberg method^54^ and an FDR less than 0.05 was used as the significance threshold throughout the paper unless otherwise specified.

In addition, we also removed 20% low expressed aging genes. Specifically, for each aging gene, we ranked samples based their expression levels in descending order and calculated the mean expression levels in the top 25% samples. We then ranked all the aging genes according to this mean expression value in descending order and removed the bottom 20% aging genes.

### Correcting for confounding factors based on principal component analysis

Correcting confounding factors is usually indispensable in revealing the true relationship between gene expression change and aging. Besides sex, major principal components (PCs) of genotype and gene expression profile in sample space are also frequently used as confounding factors in gene expression analysis to boost true signal detection. For example, removal of the top PCs has led to a significant increase in the number of expression quantitative trait loci (eQTLs) identified^55^,^56^. To remove potential confounding factors such as the batch effect, we adopted an approach similar to Pickrell *et al.*^27^. It is of note that we only considered top 3 genotype PCs (similar to GTEx eQTL study^26^) and top 5 gene expression PCs throughout this study.

Since age could be one of the top gene expression PCs or correlate with them, directly correcting these PCs is not suitable for our study. Thus, we tested nine models (namely, M1–M9) to correct possible confounding factors: (1) M1: no correction of any confounding factors; (2) M2: correcting gender and the top 3 genotype PCs; (3) M3-M7: correcting gender, top 3 genotype PCs, and the gene expression PCs correlated with age with the Pearson correlation coefficient less than a threshold of 0.1, 0.2, 0.3, 0.4, and 0.5, respectively; (4) M8: correcting gender, the top 3 genotypes, and the combination of gene expression PCs that delivered the largest number of age-associated genes; and (5) M9: correcting gender, the top 3 genotype PCs, and gene expression PCs that do not significantly (p-value > 0.05) correlate with age. The Pearson correlation between the top 5 gene expression PCs and age was listed in Supplementary Table S5 and the number of aging gene identified after removing 20% low expressed genes was listed in Supplementary Table S6. It can be seen that the chronological age was significantly correlated with top PCs in a few tissues (e.g., PC1 in adipose, artery, heart, lung, and blood) (Supplementary Table S5). In addition, confounding factors correction had some influence on the number of inferred aging genes especially for heart, lung, and blood (Supplementary Table S6).

To help us evaluate these models, we performed an enrichment analysis with GenAge genes^57^ using 20,059 GTEx protein coding genes as background (Supplementary Data S8). GenAge provides a manually curated list of 298 genes that presumably regulate the human aging process (accessed on Feb 20th, 2015). The enrichment analysis results of the nine models were shown in Supplementary Table S7, in which we also demonstrated the effect of removing 20% low expressed age-associated genes. In all models, age-associated genes were more significantly overlapped with GenAge genes after removing low expressed genes, suggesting that this filtering may help to refine the true age-associated genes. The model generating the most significant p-value is M9, by which we inferred a total of 7,925 unique protein-coding age-associated genes from all nine GTEx tissues. The number of overlap genes between these aging genes and GenAge genes is 173 with a p-value of 2.69 × 10^−11^ (Supplementary Table S7).

### Bootstrapping, permutation analysis, and the effect of sample size

To further ensure our age-associated genes are not sensitive to a particular input sample set, we bootstrapped the samples (with replacement) for 100 times. In each run, we identified age-associated genes from the bootstrapped samples using M9. A gene is in the final age-associated gene list if: (1) it is an age-associated gene in more than 50 bootstrap runs and (2) it is an age-associated gene using the full sample set.

In addition, we performed permutation analysis to estimate the fraction of false positives in our findings. Specifically, we randomly permuted the ages of samples and repeated the test using M8 for 1,000 times. We counted the number of tests in which more age-associated genes were identified, and removed the tissues whose number was larger than 5. It is of note that M8 delivers greater number of aging genes than M9, therefore provides an upper bound value of FDR.

To estimate the impact of sample size, we randomly selected samples of sizes 10 to 150 with 10 additional samples added each time for each tissue and repeated this process 100 times using M8 (see Supplementary Fig. S6). As expected, large sample sizes increases the power of identifying age-associated genes (e.g., more than 30 fold increase in the number of detected age-associated genes in whole blood when sample size increases from 10 to 150).

### Synchronization pattern of multi-tissue aging in humans

To study the co-aging of multiple tissues, a PCA method was performed to reduce the dimensionality of the gene expression data. We compared different tissues based on the first PC of gene expression. The co-aging coefficient of two tissues was defined as the Spearman correlation coefficient between the coordinates of samples on the first PC (for the two tissues) (see Fig. 3a).

To estimate the aging deviation of a sample in *n* tissues, we mapped each sample into an *n* dimensional Euclidean space with each coordinate being its rank (based on its coordinate on PC1) among the overlapping samples in a tissue. Similarly, we also ranked the samples by their chronological age. Let (*r*_1_, *r*_2_,···, *r*_*n*_) be the rank vector of a sample on *n* tissues and *r* be its rank on chronological age. We defined rank deviation *d* of a sample as 
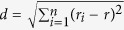
.

### Visualizing tissue co-aging in 3D space

We choose heart, lung, and blood as an example for visualizing tissue co-aging in 3D space. After rank deviation was calculated for each sample, we estimated the corresponding p-value assuming that the distance has a normal distribution and considered samples with p-values less than 0.05 as outliers. Jmol ( http://www.jmol.org/) was used for the visualization (see Fig. 3d).

### Assembly of disease gene list and disease-aging gene link detection

The disease genes were retrieved from two sources: NIH Genome-Wide Association Studies (GWAS) Catalog^40^ (accessed on Aug 13, 2014) and OMIM (Online Mendelian Inheritance in Man, accessed on Aug 13, 2014)^41^. We only considered genes in the GWAS Catalog with p-value < 5 × 10^−8^, a generally accepted threshold for genome-wide significance. Clustering and manual curation were used to merge genes in GWAS and OMIM (See Supplementary Data S9 and S10). We only considered diseases with at least 5 genes. We then performed a Fisher’s exact test between the disease genes and aging genes in each tissue. Aging genes with FDR ≤ 0.02 were used for testing age-disease overlap enrichment (p-values less than 0.05 were considered significant). In addition, we separated the up- and down-regulated genes with age. To visualize the result, we selected the top 10 most significant diseases in each tissue, which resulted in 55 unique diseases for up-regulated or 53 for down-regulated aging genes. The normalized 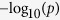 for each disease-tissue pair was plotted in Fig. 4.

### Data access

The GTEx genotype and gene expression data were downloaded from dbGap under dbGaP Study Accession number phs000424.v3.p1. MuTHER gene expression data was obtained from http://www.ebi.ac.uk/arrayexpress/experiments/E-TABM-1140/, accessed on 2-26-2015.

## Additional Information

**How to cite this article**: Yang, J. *et al.* Synchronized age-related gene expression changes across multiple tissues in human and the link to complex diseases. *Sci. Rep.* **5**, 15145; doi: 10.1038/srep15145 (2015).

## Supplementary Material

Supplementary Information

Supplementary Data S1

Supplementary Data S2

Supplementary Data S3

Supplementary Data S4

Supplementary Data S5

Supplementary Data S6

Supplementary Data S7

Supplementary Data S8

Supplementary Data S9

Supplementary Data S10

## Figures and Tables

**Table 1:** Number of age-associated genes in 9 human tissues and permutation test.

Tissues	Sample Size	Aging gene^a^			Permutation^b^	
		Up	Down	Overall	Frequency	Number
Adipose	94	652	482	1134	0	2.16
Artery	112	2258	824	3082	0	5.11
Heart	83	374	454	828	0	2.43
Lung	119	329	547	876	0	2.95
Muscle	138	766	121	887	0	1.53
Nerve	88	116	67	183	0	1.47
Skin	96	6	6	12	36	7.40
Thyroid	105	2	1	3	10	0.34
Blood	156	1381	1906	3287	5	82.60

^a^Number of significant genes after adjusting for 3 types of confounding factors: (1) gender, (2) the top three genotype PCs, and (3) among the top 5 gene expression PCs, the PCs not significantly (p-value > 0.05) correlated with age, and removing 20% low expressed genes in the original expression data. Columns “Up”, “Down”, and “Overall” list the number of up-regulated, down-regulated, and overall aging genes, respectively.

^b^Results of aging genes from 1,000 permutation tests. The column “Frequency” lists the frequencies of identifying equal or more significant genes in 1,000 permuted datasets than those in the original one. The column “Number” lists the average number of aging genes in 1,000 permutation runs.

**Table 2:** Function enrichment of up- and down-regulated age-associated genes in 7 tissues.

Tissue	Up-regulated gene set			Down-regulated gene set		
	ID^a^	Description	Adj p-val^b^	ID	Description	Adj p-val
Adipose	GO:0005578	proteinaceous extracellular matrix	1.52 × 10^−3^	GO:0005739	mitochondrion	5.77 × 10^−47^
	GO:0005815	microtubule organizing center	2.05 × 10^−2^	hsa05016	Huntington’s disease	5.12 × 10^−8^
	GO:0043167	ion binding	3.85 × 10^−2^	hsa05012	Parkinson’s disease	9.22 × 10^−8^
				GO:0022900	electron transport chain	2.68 × 10^−6^
				GO:0006631	fatty acid metabolic process	2.72 × 10^−6^
				hsa05010	Alzheimer’s disease	2.03 × 10^−5^
Artery	GO:0060589	nucleoside-triphosphatase regulator activity	3.34 × 10^−10^	GO:0030163	protein catabolic process	5.96 × 10^−14^
	GO:0030695	GTPase regulator activity	5.00 × 10^−10^	GO:0005739	mitochondrion	1.27 × 10^−6^
	GO:0006952	defense response	6.26 × 10^−8^	GO:0019787	small conjugating protein ligase activity	2.20 × 10^−5^
	GO:0006954	inflammatory response	2.35 × 10^−4^	GO:0000278	mitotic cell cycle	1.57 × 10^−3^
	GO:0008219	cell death	1.38 × 10^−3^	hsa05016	Huntington’s disease	1.25 × 10^−2^
Heart				GO:0005739	mitochondrion	1.29 × 10^−36^
				GO:0006099	tricarboxylic acid cycle	3.20 × 10^−10^
				hsa05012	Parkinson’s disease	8.79 × 10^−8^
				hsa05016	Huntington’s disease	7.10 × 10^−7^
				GO:0022900	electron transport chain	5.46 × 10^−5^
				GO:0006511	ubiquitin-dependent protein catabolic process	2.81 × 10^−4^
Lung				GO:0005739	mitochondrion	9.57 × 10^−5^
				GO:0005764	lysosome	1.00 × 10^−3^
				GO:0005773	vacuole	1.29 × 10^−3^
Muscle	GO:0016071	mRNA metabolic process	1.83 × 10^−4^	GO:0000786	nucleosome	3.23 × 10^−2^
	GO:0006281	DNA repair	3.87 × 10^−3^			
	GO:0046930	pore complex	7.85 × 10^−3^			
Nerve	GO:0048812	neuron projection morphogenesis	1.30 × 10^−2^			
	GO:0048666	neuron development	1.47 × 10^−2^			
	GO:0007155	cell adhesion	4.99 × 10^−2^			
Blood	GO:0006986	response to unfolded protein	1.88 × 10^−4^	GO:0015031	protein transport	1.10 × 10^−11^
	GO:0007049	cell cycle	8.76 × 10^−4^	GO:0016192	vesicle-mediated transport	1.64 × 10^−8^
	GO:0005524	ATP binding	6.13 × 10^−3^	GO:0007264	small GTPase mediated signal transduction	2.41 × 10^−4^
				hsa04722	Neurotrophin signaling pathway	1.71 × 10^−3^
				GO:0006915	apoptosis	9.28 × 10^−3^
				hsa05010	Alzheimer’s disease	1.88 × 10^−2^
				GO:0005739	mitochondrion	4.71 × 10^−2^

^a^Top representative gene set categories.

^b^Adj. p-val denotes the Benjamini false discovery rate.
