# Corrigendum: Synchronized age-related gene expression changes across multiple tissues in human and the link to complex diseases

**DOI:** 10.1038/srep19384

**Published:** 2016-01-21

**Authors:** Jialiang Yang, Tao Huang, Francesca Petralia, Quan Long, Bin Zhang, Carmen Argmann, Yong Zhao, Charles V. Mobbs, Eric E. Schadt, Jun Zhu, Zhidong Tu, The GTEx Consortium, Kristin G. Ardlie, David S. Deluca, Ayellet V. Segrè, Timothy J. Sullivan, Taylor R. Young, Ellen T. Gelfand, Casandra A. Trowbridge, Julian B. Maller, Taru Tukiainen, Monkol Lek, Lucas D. Ward, Pouya Kheradpour, Benjamin Iriarte, Yan Meng, Cameron D. Palmer, Wendy Winckler, Joel Hirschhorn, Manolis Kellis, Daniel G. MacArthur, Gad Getz, Andrey A. Shablin, Gen Li, Yi-Hui Zhou, Andrew B. Nobel, Ivan Rusyn, Fred A. Wright, Tuuli Lappalainen, Pedro G. Ferreira, Halit Ongen, Manuel A. Rivas, Alexis Battle, Sara Mostafavi, Jean Monlong, Michael Sammeth, Marta Mele, Ferran Reverter, Jakob Goldmann, Daphne Koller, Roderic Guigo, Mark I. McCarthy, Emmanouil T. Dermitzakis, Eric R. Gamazon, Anuar Konkashbaev, Dan L. Nicolae, Nancy J. Cox, Timothée Flutre, Xiaoquan Wen, Matthew Stephens, Jonathan K. Pritchard, Luan Lin, Jun Liu, Amanda Brown, Bernadette Mestichelli, Denee Tidwell, Edmund Lo, Mike Salvatore, Saboor Shad, Jeffrey A. Thomas, John T. Lonsdale, Christopher Choi, Ellen Karasik, Kimberly Ramsey, Michael T. Moser, Barbara A. Foster, Bryan M. Gillard, John Syron, Johnelle Fleming, Harold Magazine, Rick Hasz, Gary D. Walters, Jason P. Bridge, Mark Miklos, Susan Sullivan, Laura K. Barker, Heather Traino, Magboeba Mosavel, Laura A. Siminoff, Dana R. Valley, Daniel C. Rohrer, Scott Jewel, Philip Branton, Leslie H. Sobin, Liqun Qi, Pushpa Hariharan, Shenpei Wu, David Tabor, Charles Shive, Anna M. Smith, Stephen A. Buia, Anita H. Undale, Karna L. Robinson, Nancy Roche, Kimberly M. Valentino, Angela Britton, Robin Burges, Debra Bradbury, Kenneth W. Hambright, John Seleski, Greg E. Korzeniewski, Kenyon Erickson, Yvonne Marcus, Jorge Tejada, Mehran Taherian, Chunrong Lu, Barnaby E. Robles, Margaret Basile, Deborah C. Mash, Simona Volpi, Jeff Struewing, Gary F. Temple, Joy Boyer, Deborah Colantuoni, Roger Little, Susan Koester, NCI Latarsha J. Carithers, Helen M. Moore, Ping Guan, Carolyn Compton, Sherilyn J. Sawyer, Joanne P. Demchok, Jimmie B. Vaught, Chana A. Rabiner, Nicole C. Lockhart

Scientific Reports 5: Article number: 1514510.1038/srep15145; published online: 10192015; updated: 01212016

The original version of this Article contained a typographical error in the spelling of the author Jakob Goldmann which was incorrectly given as Jakob Goldman. This has now been corrected in both the PDF and HTML versions of the Article.

