# Supplementary material for: Synchronized age-related gene expression changes across multiple tissues in human and the link to complex diseases
Source: Sci Rep. 2015 Oct 19;5:15145. doi: 10.1038/srep15145 (PMC4609956; doi:10.1038/srep15145)
Supplement: Supplementary Information [file srep15145-s1.doc]

**Synchronized age-related gene expression changes across multiple tissues in human and the link to complex diseases**

Running Title: Age-related gene expression changes in multiple tissues

**Jialiang Yang^1,2^, Tao Huang^1,2,¶^, Francesca Petralia^1,2^, Quan Long^1,2^, Bin Zhang^1,2^, Carmen Argmann^1,2^, Yong Zhao^1,2^, Charles V Mobbs^3,4,5^, GTEx Consortium, Eric E Schadt^1,2^ , Jun Zhu^1,2^, Zhidong Tu^1,2*^**

^1^ Institute of Genomics and Multiscale Biology, Icahn School of Medicine at Mount Sinai, NY, 10029, USA

^2^ Department of Genetics and Genomic Sciences, Icahn School of Medicine at Mount Sinai , NY, 10029, USA

^3^ Department of Neuroscience, Icahn School of Medicine at Mount Sinai, NY, 10029, USA

^4^ Department of Geriatrics and Palliative Medicine, Icahn School of Medicine at Mount Sinai , NY, 10029, USA

^5^ Department of Medicine, Endocrinology, Diabetes and Bone Disease, Icahn School of Medicine at Mount Sinai , NY, 10029, USA

¶ Current: Institute of Health Sciences, Shanghai Institutes for Biological Sciences, Chinese Academy of Sciences, Shanghai 200031, People's Republic of China

^*^Corresponding author: Zhidong Tu

Icahn Institute for Genomics and Multiscale Biology, Department of Genetics and Genomic Sciences, Icahn School of Medicine at Mount Sinai, One Gustave L. Levy Place - Box 1498, 1425 Madison Ave, IMI 3-70F, New York, NY 10029-6574

PH: 212-659-8508 Email addresses: [zhidong.tu@mssm.edu](mailto:zhidong.tu@mssm.edu)

# Supplementary Methods

### Elastic Net regression

The age prediction problem can be formulated as the following Elastic Net (EN) regression problem:

where $\mathrm{Age}_{i}$ is the chronological age of the donor of sample $i$ with $1\leq i\leq M,$ and $M$ is the number of samples in a particular tissue; $x_{ij}$ is the expression of gene $j$ with $1\leq j\leq N$ for sample $i$, here $N$ is the number of pre-selected genes in the tissue;$w_{0}$ is the intercept, $w_{j}$ is the weight for gene $j$; $\alpha$ with $0\leq\alpha\leq1$ is a parameter to balance $L_{1}$ (e.g., lasso) and $L_{2}$ (e.g., ridge regression) penalties, and $\lambda$ is the lasso parameter.

Specifically, for each tissue we first randomly divide the samples into 10 subgroups of equal size (10-fold), and predict the sample age in each fold using the data (expression and age) of other 9 folds. To build a prediction model using the data from 9 folds, we first filter the genes by requiring that the Pearson correlation coefficient between the gene expression and sample age is greater than or equal to 0.2 (similar to[^1^](#_ENREF_1)). Using the tuned model parameters *α* and *λ* (see next paragraph), we build the prediction EN model based on the expression profile of the filtered genes and sample’s age. This model is then used to predict the age of the samples in the other fold and a local prediction rooted mean square error (RMSE) between the predicted sample age and the chronological age is calculated. This process is repeated for 10 times until all the sample ages have been predicted once, and the RMSE is calculated as the mean local RMSEs. To avoid biasness in a particular sampling, we repeat the sampling for 100 times and average the predicted age for each sample (which is predicted 100 times) and the prediction RMSE.

### Parameter tuning

We perform a grid search on the two parameters *α* and *λ* with *α* varying from 0 to 1 with an interval of 0.1 and *λ* varying from 2^-10^ to 2^10^ with a multiplication of 2. The parameters corresponding to the lowest prediction RMSE are selected as the tuned parameters.

### The MuTHER Study

The MuTHER dataset contains 48,638 probe-level gene expression in 825 adipose samples, 48,646 probe expressions in 705 skin samples, and 48,638 probe expressions in 825 LCL samples. To identify age-associated genes from this dataset, Glass et al. excluded probes mapping to genes of uncertain function (LOC symbols) and those encompassing common SNPs (1000G release June 2010)[^2^](#_ENREF_2). We adopted Glass et al.’s filtering criteria on the MuTHER data, which resulted in 23,227 probes and 825 samples in adipose, 23,172 probes and 705 samples in skin, and 23,108 probes and 825 samples in LCLs. We then performed our analyses only using these probes and samples.

# Supplementary Figures

**Supplementary Fig. S1 - Age-associated gene expression in (a) artery, (b) heart, (c) lung, (d) muscle, (e) nerve, and (f) whole blood**

**
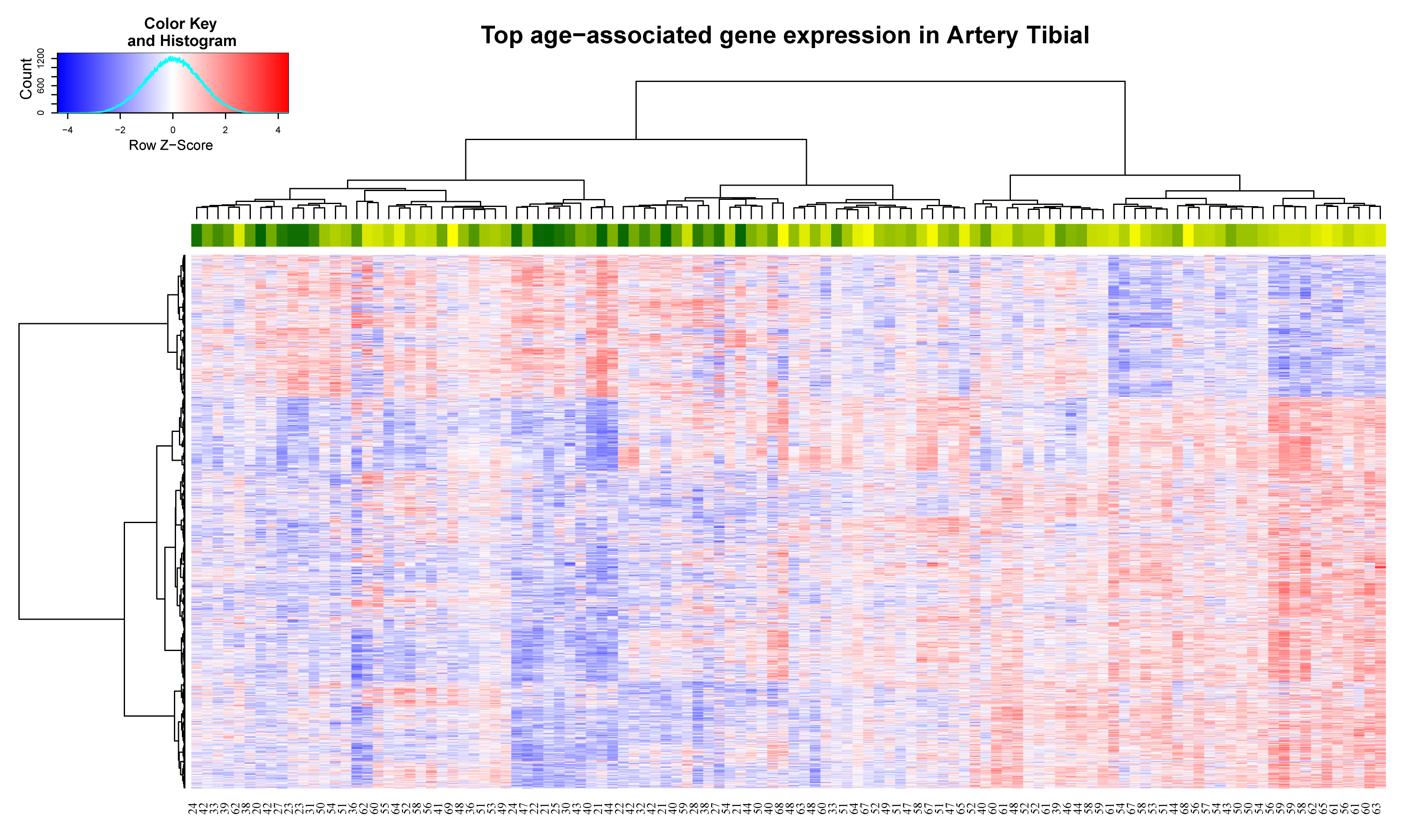
**

**(a)**

**
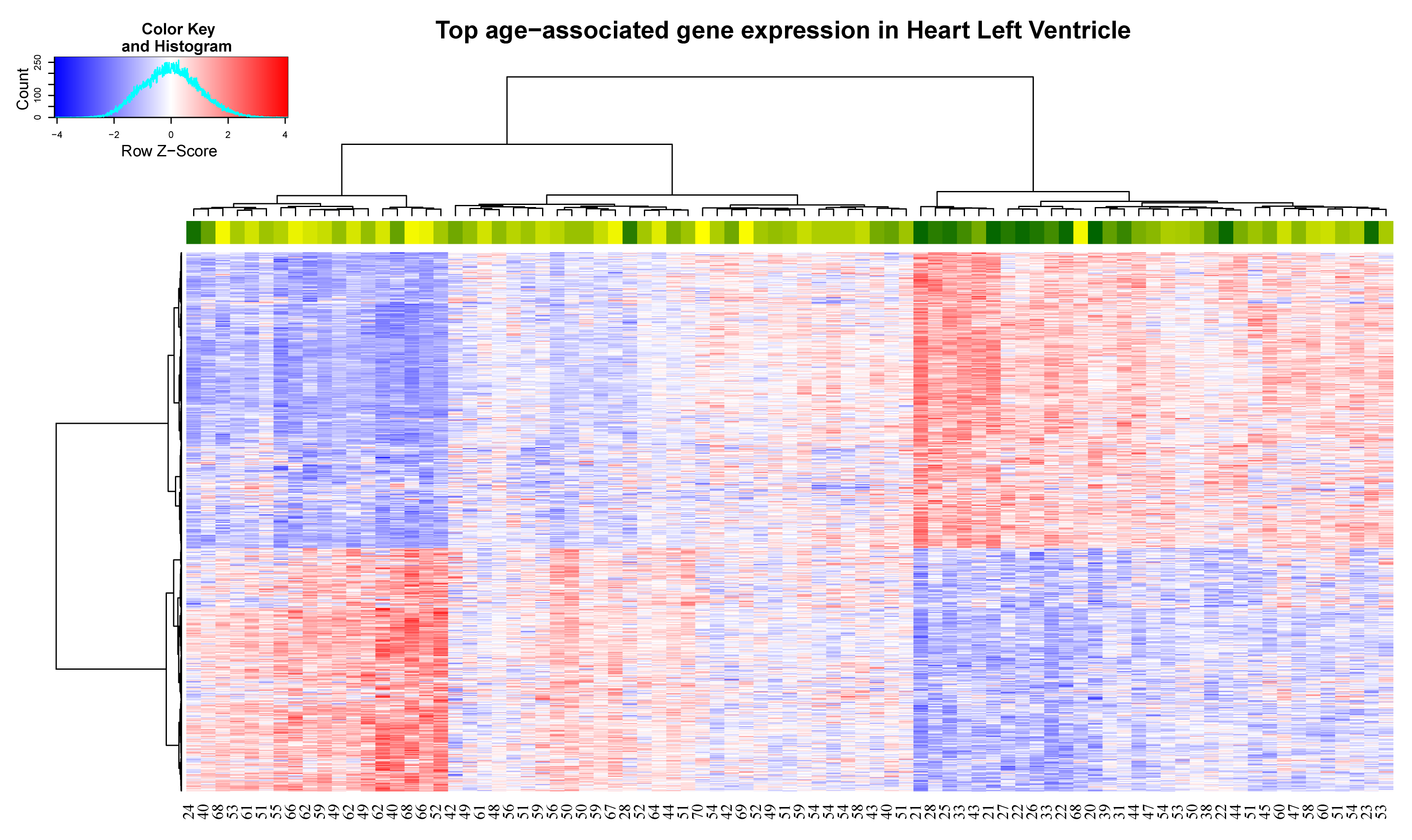
**

**(b)**

**
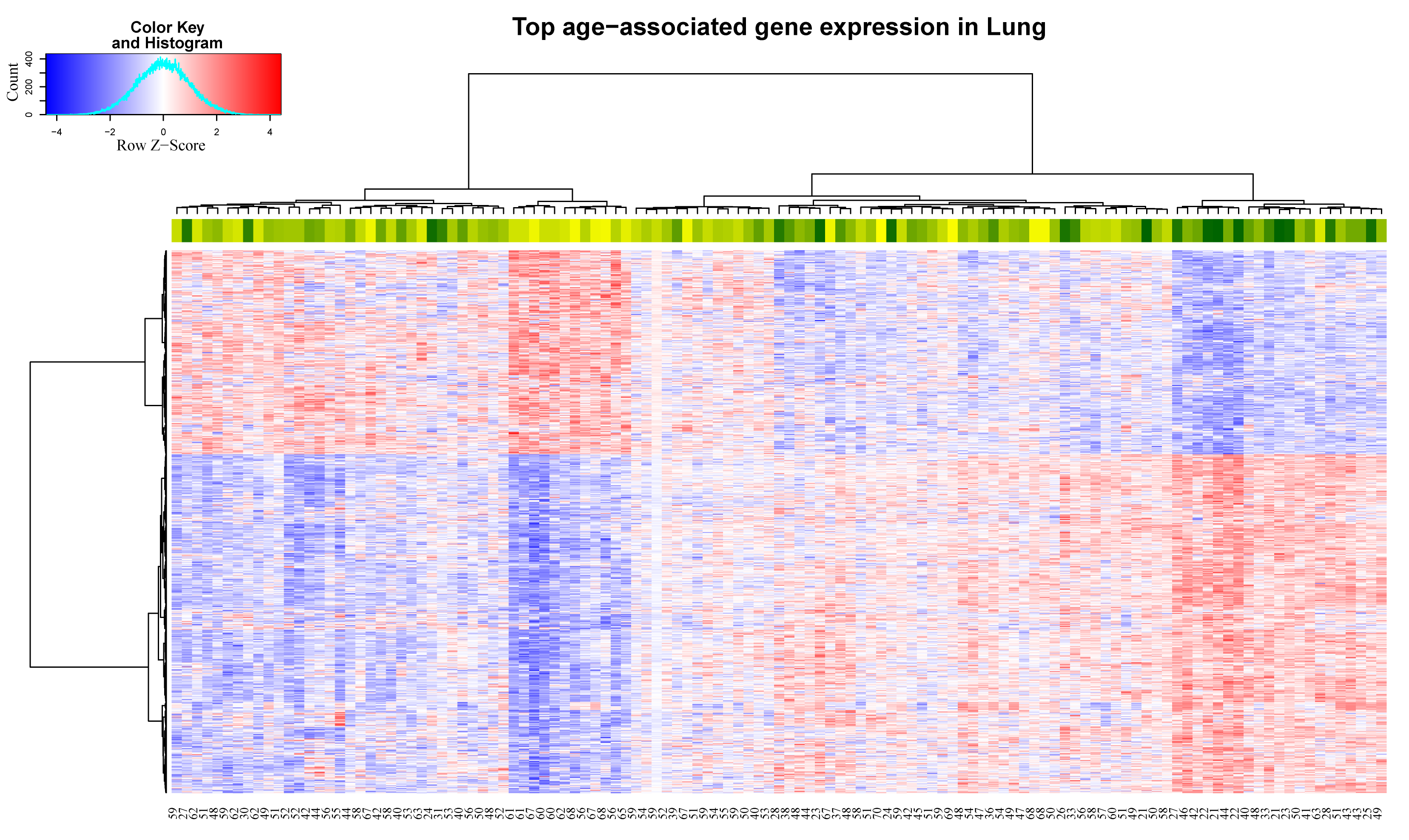
**

**(c)**


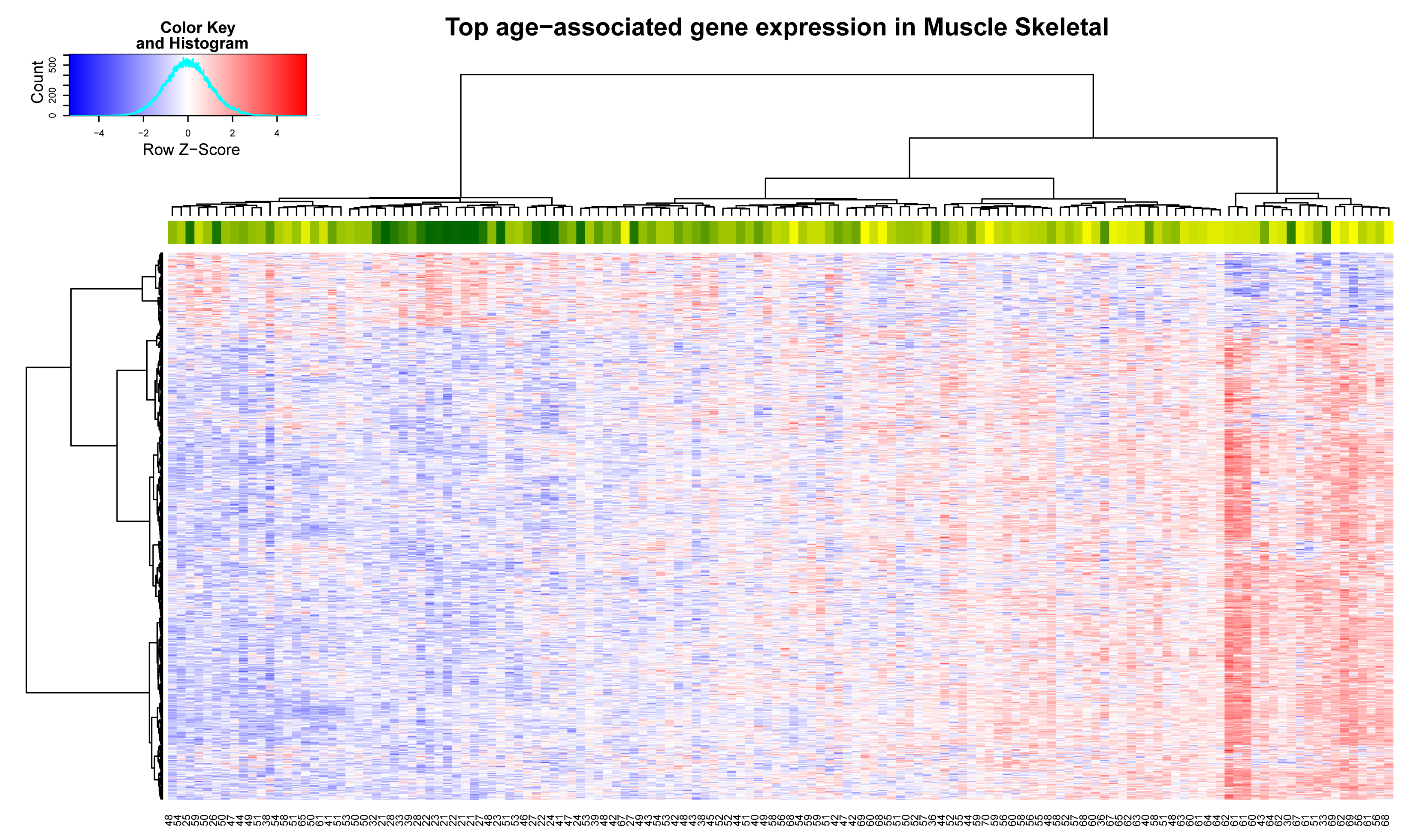


**(d)**


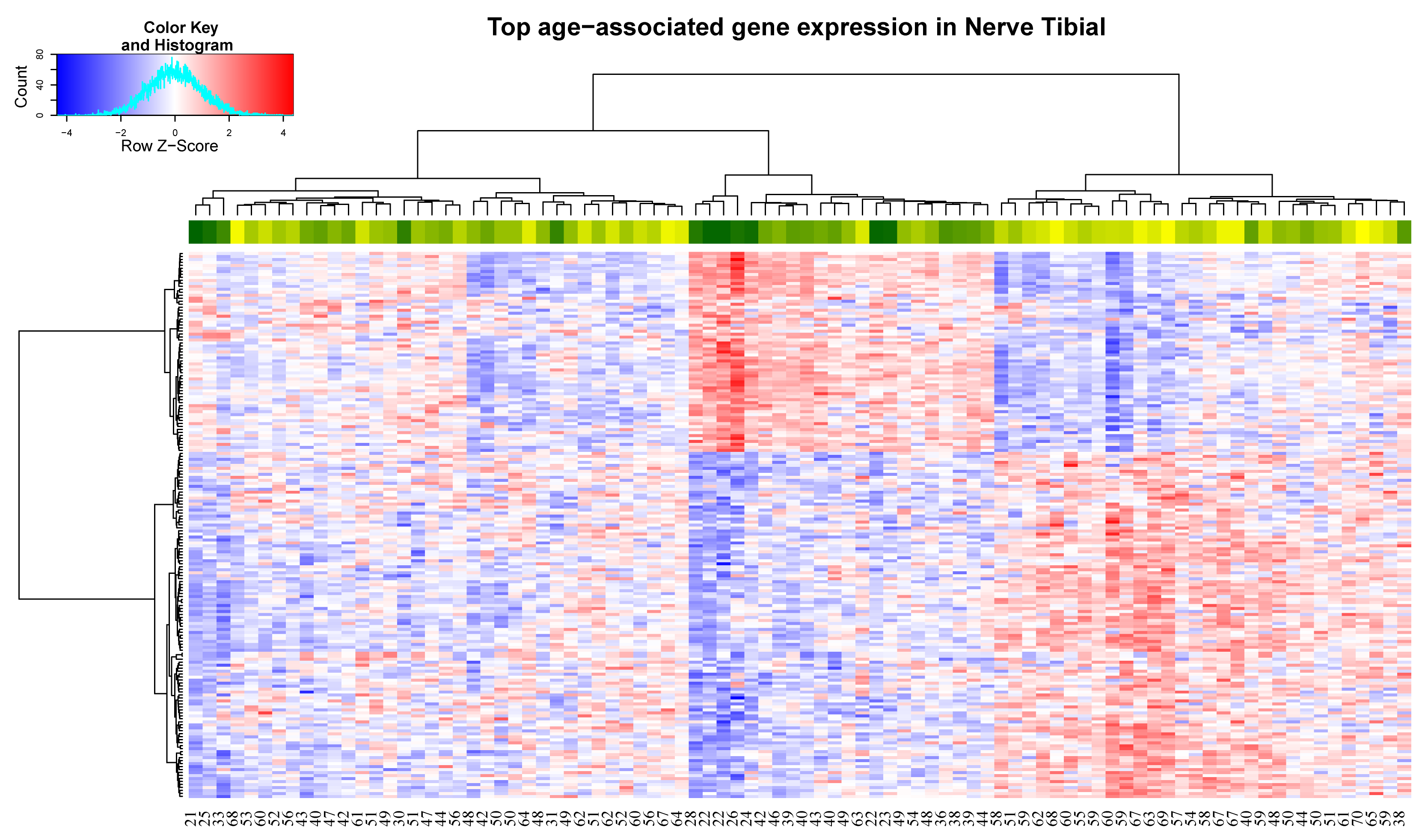


**(e)**


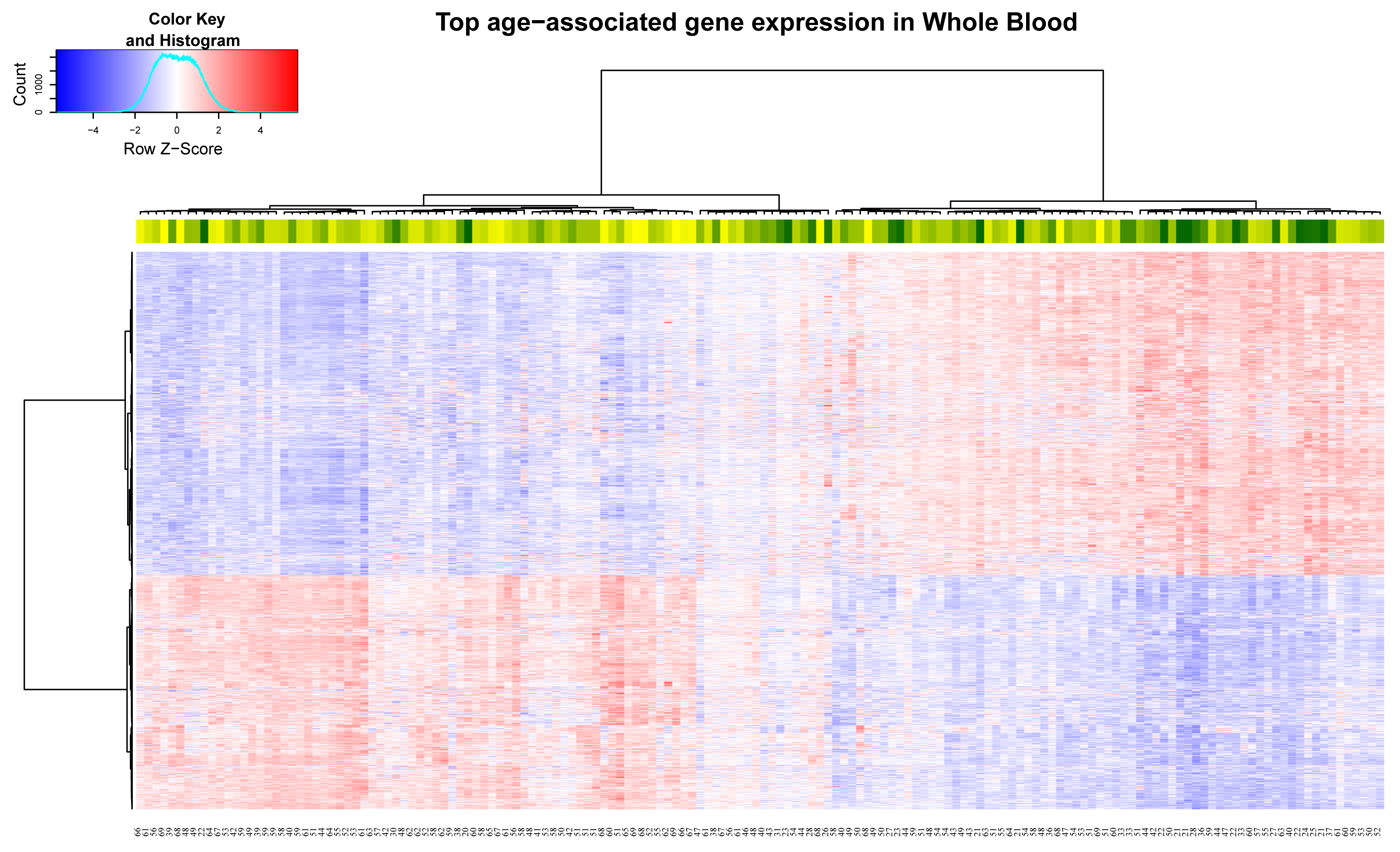


**(f)**

Rows indicate aging genes and columns indicate samples. Heatmap colors represent normalized gene expression values with blue for low expression and red for high expression. The age of each individual is displayed at the bottom and also illustrated in color bar at the top with dark green for young and yellow for old.

**Supplementary Fig. S2 - Age distributions of two groups in adipose, artery, heart, lung, muscle, nerve, and whole blood**

The age distribution of young group is illustrated in blue and that of old group is in red.

**Supplementary Fig. S3 - Variance captured by the top 10 principal components, the correlation between sample chronological ages and coordinates on the first PC, and clustering results by the top 2 PCs for all 7 tissues (a-g) tested**

The left figure shows the variance captured by top 10 PCs; the middle figure shows the correlation between sample ages and coordinates on PC1; and the right figure shows the clustering using the top 2 PCs, each blue point represents one sample clustered to be “young” in Fig. 2a, while the red points are old samples.

**Supplementary Fig. S4 - Cluster of all 991 samples by (a) aging genes (b) all genes**


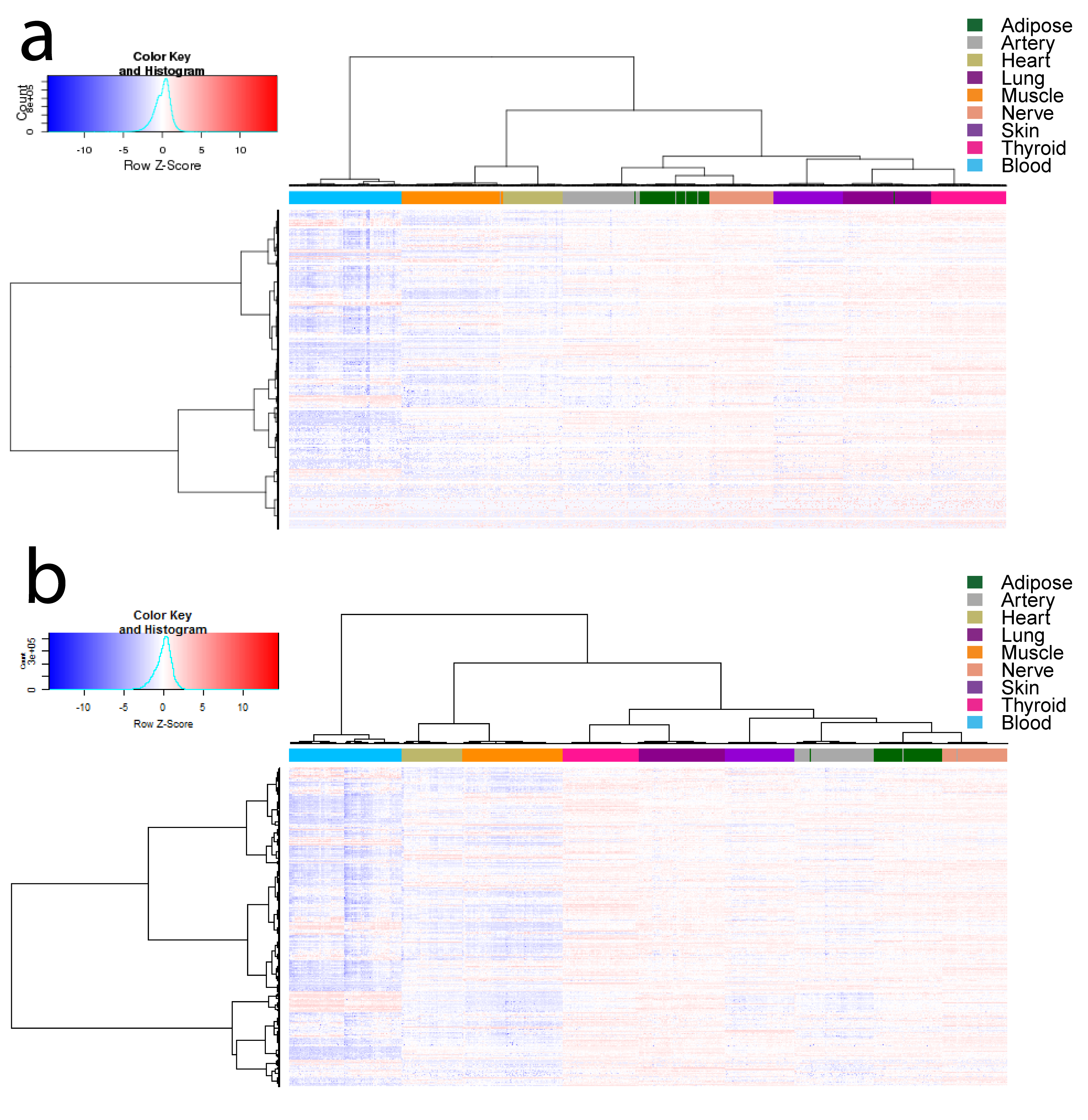


Rows indicate genes and columns indicate samples. Heatmap colors represent normalized gene expression values with blue for low expression and red for high expression. The tissue type of each sample is displayed with different colors in the side bar on the top, where adipose, artery, heart, lung, muscle, nerve, skin, thyroid, and whole blood are represented in darkgreen, darkgrey, darkkhaki, darkmagenta, darkorange, darksalmon, darkviolet, deeppink, and deepskyblue respectively.

**Supplementary Fig. S5 - Tissue correlation inferred by age prediction using the Elastic Net approach**

**
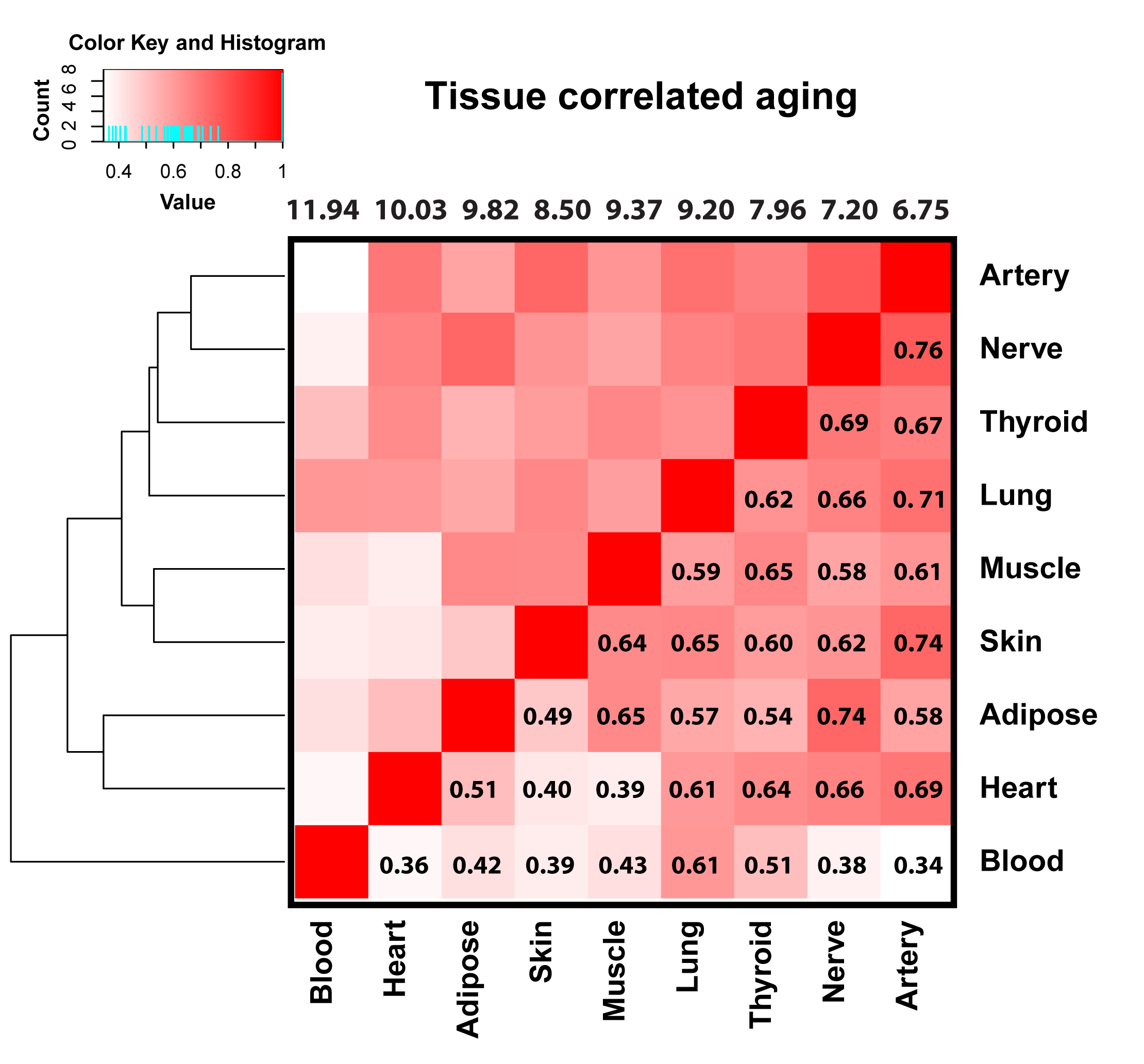
**

The number in each square in the lower triangle indicates the co-aging coefficient between a tissue pair; and the prediction rooted mean square error (RMSE) in 10-fold cross validation for each tissue is also presented in the top of the sample column.

**Supplementary Fig. S6 - Effect of sample size on inferring age-associated genes**


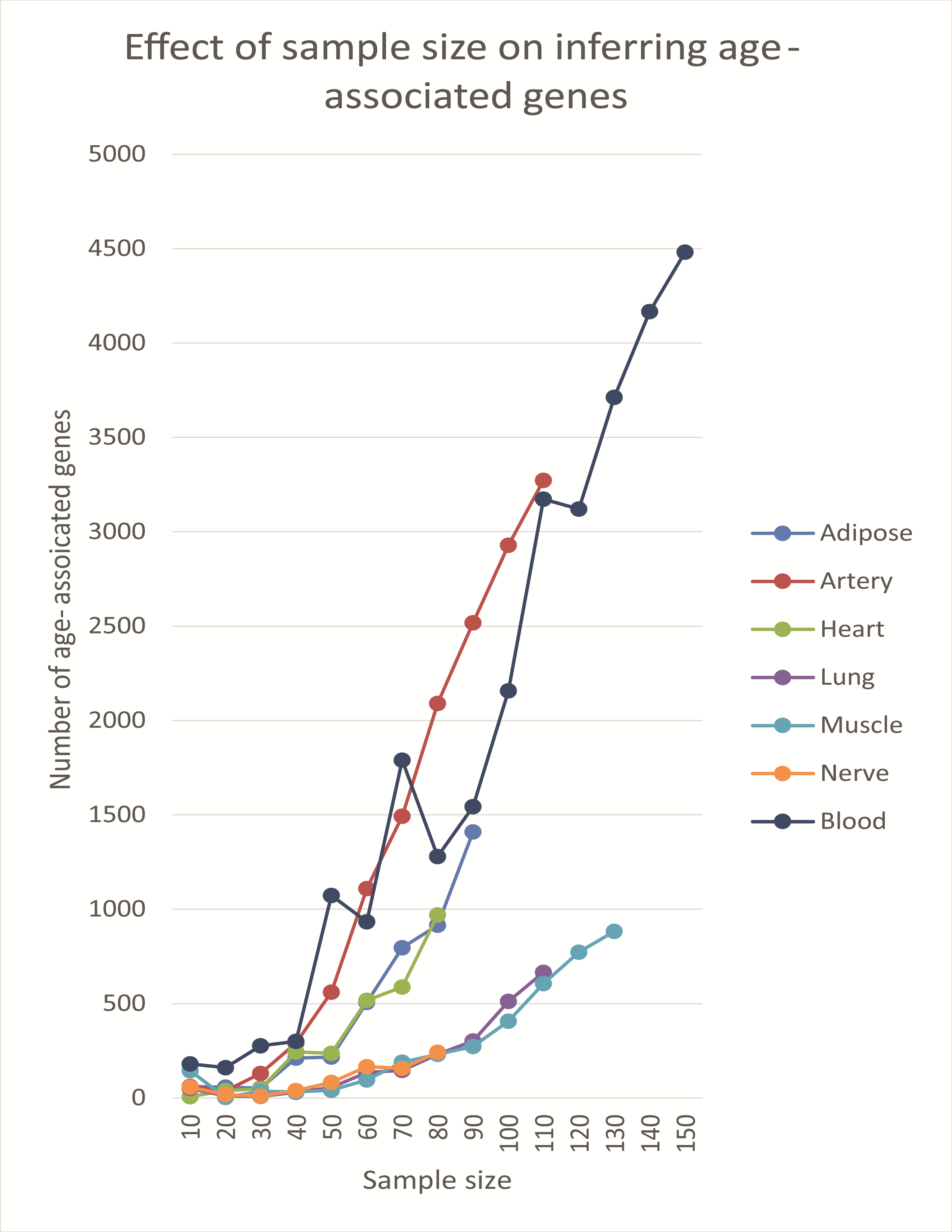


x-axis indicates the sample size and y-axis indicates the average number of age-associated genes in 100 bootstrap runs.

# Supplementary Tables

**Supplementary Table S1 - Difference of young and old expression groups in samples of seven tissues by the student’s** t-test

| **Tissue** | **Young** | | **Old** | | **P-value^b^** |
| --- | --- | --- | --- | --- | --- |
|  | **Mean age^a^** | **Sample size** | **Mean age** | **Sample size** |  |
| Adipose | 43.54 | 54 | 54.60 | 40 | 6.44E-06 |
| Artery | 44.04 | 73 | 55.18 | 39 | 2.04E-07 |
| Heart | 39.79 | 33 | 53.48 | 50 | 1.24E-05 |
| Lung | 46.41 | 74 | 53.13 | 45 | 3.59E-03 |
| Muscle | 40.11 | 46 | 53.76 | 92 | 6.39E-08 |
| Nerve | 45.21 | 58 | 57.60 | 30 | 4.24E-07 |
| Blood | 45.43 | 69 | 53.14 | 87 | 3.56E-04 |

^a^ Mean age of donors in the young group classified in Fig. 2a and Supplementary Fig. S1 by using the Ward’s method[^3^](#_ENREF_3).

^b^ The p-value is calculated by the student t-test on the sample ages in young and old group.

**Supplementary Table S2** – Correlation between rank deviation and age in pair-wise tissues

|  | **Adipose** | **Artery** | **Heart** | **Lung** | **Muscle** | **Nerve** | **Blood** |
| --- | --- | --- | --- | --- | --- | --- | --- |
| Adipose | 1 | 4.99E-02 | 2.81E-01 | 2.25E-01 | 3.12E-01 | 6.59E-02 | 2.84E-01 |
| Artery | 0.23 | 1 | 1.70E-02 | 7.37E-02 | 6.96E-02 | 2.04E-04 | 3.60E-02 |
| Heart | 0.14 | 0.29 | 1 | 2.33E-01 | 4.16E-01 | 3.70E-02 | 4.32E-01 |
| Lung | 0.11 | 0.13 | 0.12 | 1 | 1.94E-01 | 1.56E-02 | 1.83E-01 |
| Muscle | 0.04 | 0.12 | 0.07 | 0.09 | 1 | 2.34E-01 | 5.27E-01 |
| Nerve | 0.17 | 0.36 | 0.3 | 0.27 | 0.17 | 1 | 1.65E-01 |
| Blood | 0.11 | 0.14 | 0.09 | 0.04 | 0.01 | 0.17 | 1 |

The values in lower triangle list the Spearman correlation coefficients between rank deviation and age in all tissues pairs, and the values in the upper triangle list the corresponding p-values.

**Supplementary** Table S3 – The Spearman correlation between chronological age of samples and predicted age rank of samples by both PCA and Elastic Net

|  | **Adipose** | **Artery** | **Heart** | **Lung** | **Muscle** | **Nerve** | **Blood** |
| --- | --- | --- | --- | --- | --- | --- | --- |
| PCA | 0.57 | 0.63 | 0.52 | 0.49 | 0.57 | 0.74 | 0.36 |
| Elastic Net | 0.63 | 0.84 | 0.61 | 0.68 | 0.71 | 0.82 | 0.42 |

**Supplementary Table S4 - Comparison of the aging genes by our regression model on MuTHER and GTEx data, and those identified by Glass et al**[**^2^**](#_ENREF_2) **in adipose, skin, and blood**

|  | **All tested unique gene symbols^a^** | | | **Unique aging gene symbols** | | | **Overlap gene count (p-value^b^)** | | | |
| --- | --- | --- | --- | --- | --- | --- | --- | --- | --- | --- |
|  | **GTEX** | **MuTHER** | **Overlap** | **GTEX** | **MuTHER** | **Glass** | **MuTHER & GTEx** | **overlap%** | **GTEx & Glass** | **overlap%^c^** |
| Adipose | 31,120 | 16,425 | 13,372 | 1,462 | 1,773 | 189 | 157 (1.51E-4) | 83% | 25 (1.37E-3) | 13% |
| Skin | 32,357 | 16,404 | 13,518 | 12 | 3,034 | 1530 | 4 (0.17) | 33% | 4 (2.1E-2) | 33% |
| Blood | 28,998 | 16,350 | 12,897 | 4,858 | 11 | 2 | 4 (0.17) | 36% | 0(1) | 0% |
|  | **All tested gene IDs^d^** | | | **Aging gene IDs** | | |  | **Overlap gene count**  **(p-value)** | | **overlap%** |
|  | **MuTHER** | **GLASS** | | **MuTHER** | **Glass** | | |  |  |  |
| Adipose | 23,227 | 23,227 | | 1,922 | 199 | | | 182 (1.39E-177) | | 0.91 |
| Skin | 23,172 | 23,172 | | 3,355 | 1,617 | | | 1,585 (< 4.9E-324) | | 0.98 |
| Blood | 23,108 | 23,108 | | 11 | 2 | | | 2 (2.06E-7) | | 1 |

^a^ Notice that we can only compare the overlap of unique gene symbol between GTEx and MuTHER (Glass et al.) since they are not using the same gene ID. We used M9 model to infer aging genes in both GTEx and MuTHER data.

^b^ P-value indicates the p-value on the Fisher’s exact test for overlapping of two sets.

^c^ Overlap% is calculated using the number of overlapping genes divided by the smaller number of genes in the two comparison sets.

^d^ We compared gene ID between MuTHER and GLASS et al.

**Supplementary Table S5 – The Pearson correlation between the top 5 gene expression PCs and chronological age in nine tissues**

|  | **PC1** | | **PC2** | | **PC3** | | **PC4** | | **PC5** | |
| --- | --- | --- | --- | --- | --- | --- | --- | --- | --- | --- |
|  | **Cor^a^** | **p-value^b^** | **Cor** | **p-value** | **Cor** | **p-value** | **Cor** | **p-value** | **Cor** | **p-value** |
| Adipose | 0.41 | **4.47E-05** | 0.19 | 6.73E-02 | 0.11 | 2.87E-01 | -0.14 | 1.78E-01 | 0.02 | 8.33E-01 |
| Artery | -0.25 | **7.45E-03** | -0.05 | 5.66E-01 | -0.20 | **3.07E-02** | -0.03 | 7.18E-01 | -0.29 | **1.82E-03** |
| Heart | 0.26 | **1.82E-02** | -0.03 | 7.83E-01 | -0.15 | 1.88E-01 | -0.04 | 7.04E-01 | 0.18 | 1.11E-01 |
| Lung | -0.29 | **1.61E-03** | 0.06 | 5.00E-01 | 0.03 | 7.59E-01 | 0.07 | 4.40E-01 | -0.19 | **4.18E-02** |
| Muscle | -0.03 | 7.05E-01 | 0.22 | **9.67E-03** | -0.05 | 5.74E-01 | 0.03 | 7.61E-01 | -0.13 | 1.23E-01 |
| Nerve | 0.00 | 9.97E-01 | 0.18 | 9.67E-02 | 0.18 | 8.80E-02 | 0.15 | 1.66E-01 | 0.18 | 8.70E-02 |
| Skin | 0.02 | 8.50E-01 | -0.19 | 5.80E-02 | 0.03 | 7.96E-01 | 0.00 | 9.88E-01 | 0.05 | 6.32E-01 |
| Thyroid | -0.01 | 9.49E-01 | 0.10 | 3.22E-01 | 0.06 | 5.38E-01 | 0.00 | 9.81E-01 | 0.07 | 4.63E-01 |
| Blood | 0.28 | **3.67E-04** | -0.14 | 8.69E-02 | 0.02 | 7.83E-01 | -0.10 | 2.24E-01 | -0.12 | 1.25E-01 |

^a^ Cor indicates the Pearson correlation coefficient between the gene expression PC and age.

^b^ p-value indicates the significance of the correlation between the gene expression PC and age. The significant p-values (<= 0.05) are shown in bold fonts.

**Supplementary Table S6 - Number of age-associated genes inferred** by correcting different confounding factors

| **Tissue** | **Sample size** | **M1**^a^ | **M2** | **M3** | **M4** | **M5** | **M6** | **M7** | **M8** | **M9** |
| --- | --- | --- | --- | --- | --- | --- | --- | --- | --- | --- |
| Adipose | 94 | 1214 | 732 | 938 | 1464 | 1464 | 1464 | 4 | 1464 | 1464 |
| Artery | 112 | 2594 | 2368 | 3175 | 3175 | 1822 | 1822 | 1822 | 3367 | 3175 |
| Heart | 83 | 0 | 12 | 886 | 1124 | 21 | 21 | 21 | 1124 | 1124 |
| Lung | 119 | 115 | 76 | 944 | 521 | 9 | 9 | 9 | 944 | 944 |
| Muscle | 138 | 189 | 274 | 1060 | 1076 | 200 | 200 | 200 | 1076 | 1076 |
| Nerve | 88 | 76 | 73 | 148 | 236 | 236 | 236 | 236 | 296 | 236 |
| Skin | 96 | 0 | 0 | 20 | 12 | 12 | 12 | 12 | 20 | 12 |
| Thyroid | 105 | 2 | 4 | 3 | 3 | 3 | 3 | 3 | 4 | 3 |
| Blood | 156 | 1972 | 1854 | 2499 | 4865 | 58 | 58 | 58 | 4865 | 4865 |

^a^ M1: no correction of any confounding factors; M2: correcting gender and the top 3 genotype PCs; M3-M7: correcting gender, the top 3 genotype PCs, and the gene expression PCs correlated with age with the Pearson correlation coefficient less than a threshold of 0.1, 0.2, 0.3, 0.4, and 0.5, respectively; M8: correcting gender, the top 3 genotypes, and the combination of gene expression PCs that delivered the largest number of age-associated genes; M9: correcting gender, the top 3 genotype PCs, and gene expression PCs that do not significantly (p-value > 0.05) correlate with age. The number of aging genes shown was after removing 20% low expressed genes.

**Supplementary Table S7 – Overlap enrichment between aging genes listed in GenAge and aging genes derived from M1-M9**

|  | **All aging genes** | | | **Remove 20% low expressed aging genes** | | |
| --- | --- | --- | --- | --- | --- | --- |
|  | **GTEx aging gene^a^** | **Overlap^b^** | **p-value^c^** | **Aging Gene** | **Overlap** | **p-value** |
| M1**^d^** | 5080 | 93 | 1.02E-02 | 4393 | 86 | 2.23E-03 |
| M2 | 4498 | 85 | 6.48E-03 | 3880 | 79 | 1.19E-03 |
| M3 | 7288 | 145 | 4.87E-06 | 6298 | 137 | 4.87E-08 |
| M4 | 8948 | 183 | 1.52E-09 | 7743 | 170 | 3.17E-11 |
| M5 | 3386 | 69 | 2.62E-03 | 2906 | 66 | 1.86E-04 |
| M6 | 3386 | 69 | 2.62E-03 | 2906 | 66 | 1.86E-04 |
| M7 | 2218 | 43 | 3.78E-02 | 1903 | 41 | 8.91E-03 |
| M8 | 9240 | 184 | 1.51E-08 | 7999 | 171 | 2.97E-10 |
| M9 | 9152 | 185 | 3.10E-09 | 7925 | 173 | 2.69E-11 |

^a^ The number of unique protein coding aging genes inferred from all nine tissues

^b^ The number of overlapping aging genes between GTEx and GenAge

^c^ The significance of the overlap between GTEx and GenAge aging genes using the Fisher’s exact test

^d^ M1-M9 are the same as Supplementary Table S6

# Supplementary Datasets

**Supplementary Data S1 - Aging coefficients, p-values, and FDR for 41,298 genes in nine human tissues**

“NA” indicates that the gene's expression data are not tested for the corresponding tissue; “NULL” indicates that the Entrez ID does not exist.

**Supplementary Data S2 - Expression change of the top 100 age-associated genes in each tissue**

Pearson-R value in the title represents the Pearson correlation coefficient between gene expression and age across all samples. The solid blue triangles plot male samples and solid red circle female samples. Similarly, the blue and red lines denote the regression lines for male and female samples, respectively.

**Supplementary Data S3 - Function annotation on up-regulated and down-regulated aging genes**

*****Gene sets marked in yellow are selected as representatives in Table 2.

**Supplementary Data S4 - Correlation between diseases and aging in multiple tissues**

*****Each entry denotes the p-value for the Fisher's exact test on overlapping genes

*****The disease-tissue pairs mentioned in the manuscript are marked in yellow

**Supplementary Data S5 - Homologous genes between human and mouse in heart and lung**

*****The genes marked in yellow are aging genes at p-value less than 0.001

**Supplementary Data S6 - Comparing the aging genes identified by GTEx and MuTHER data using our regression model**

**Supplementary Data S7 - Comparing age-associated genes identified by our regression method and GLASS on MuTHER Data**

*****Genes in light green are overlapping aging gene between GLASS et al. and our study using MuTHER data.

*****Genes in yellow are aging genes in GLASS et al. but not in our study.

*****Genes in light blue are aging genes in our study but not in GLASS et al.

**Supplementary Data S8 - Overlapping genes between GTEx aging genes and GenAge genes**

*****M9: correcting gender, top 3 genotype PCs, and gene expression PCs not significantly (p-value 0.05) correlated with age

**Supplementary Data S9 - Merge GWAS trait genes**

**Supplementary Data S10 - Merge GWAS and OMIM disease genes**

# References

1. Barretina J*, et al.* The Cancer Cell Line Encyclopedia enables predictive modelling of anticancer drug sensitivity. *Nature* **483**, 603-607 (2012).

2. Glass D*, et al.* Gene expression changes with age in skin, adipose tissue, blood and brain. *Genome Biol* **14**, R75 (2013).

3. Ward JH. Hierarchical Grouping to Optimize an Objective Function. *J Am Stat Assoc* **58**, 236-244 (1963).
