# Supplementary material for: Synchronized age-related gene expression changes across multiple tissues in human and the link to complex diseases
Source: Sci Rep. 2015 Oct 19;5:15145. doi: 10.1038/srep15145 (PMC4609956; doi:10.1038/srep15145)
Supplement: Supplementary Data S2 [file srep15145-s3.pdf]

Synchronized age-related gene expression changes across multiple tissues in human and the link to complex diseases

Running Title: Age-related gene expression changes in multiple tissues

Jialiang Yang[1,2], Tao Huang[1,2,¶], Francesca Petralia[1,2], Quan Long[1,2], Bin Zhang[1,2], Carmen Argmann[1,2], Yong Zhao[1,2], Charles V Mobbs[3,4,5], GTEx Consortium, Eric E Schadt[1,2], Jun Zhu[1,2], Zhidong Tu[1,2*]

[1] Institute of Genomics and Multiscale Biology, Icahn School of Medicine at Mount Sinai, NY, 10029, USA

[2] Department of Genetics and Genomic Sciences, Icahn School of Medicine at Mount Sinai , NY, 10029, USA

[3] Department of Neuroscience, Icahn School of Medicine at Mount Sinai, NY, 10029, USA

[4] Department of Geriatrics and Palliative Medicine, Icahn School of Medicine at Mount Sinai , NY, 10029, USA

[5] Department of Medicine, Endocrinology, Diabetes and Bone Disease, Icahn School of Medicine at Mount Sinai , NY, 10029, USA

¶ Current: Institute of Health Sciences, Shanghai Institutes for Biological Sciences, Chinese Academy of Sciences, Shanghai 200031, People's Republic of China

[*]Corresponding author: Zhidong Tu

Icahn Institute for Genomics and Multiscale Biology, Department of Genetics and Genomic Sciences, Icahn School of Medicine at Mount Sinai, One Gustave L. Levy Place - Box 1498, 1425 Madison Ave, IMI 3-70F, New York, NY 10029-6574

PH: 212-659-8508 Email addresses: zhidong.tu@mssm.edu

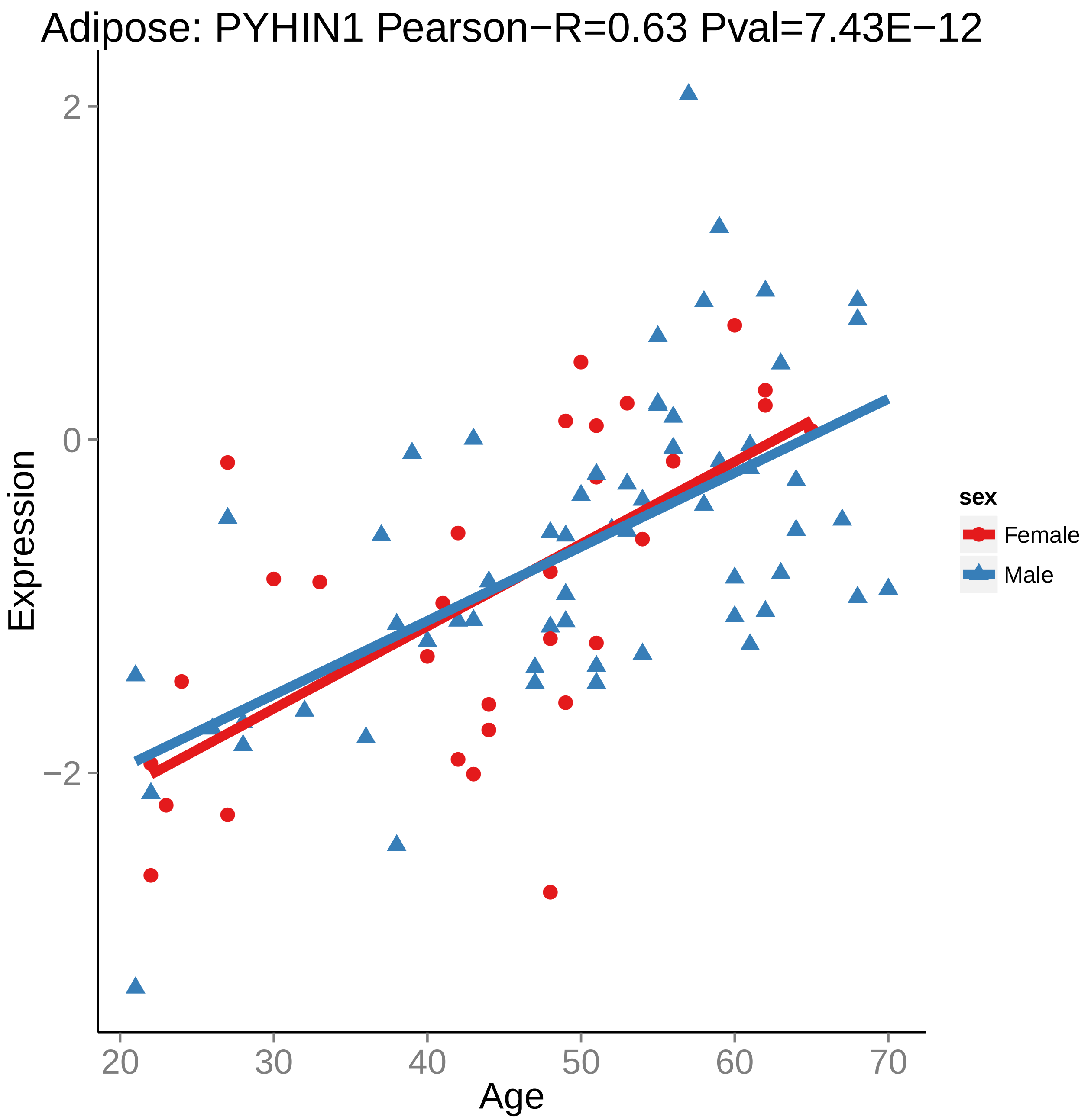

Adipose: PYHIN1 Pearson−R=0.63 Pval=7.43E−12
Expression
Age
2
0
−2
20
30
40
50
60
70
sex
Female
Male

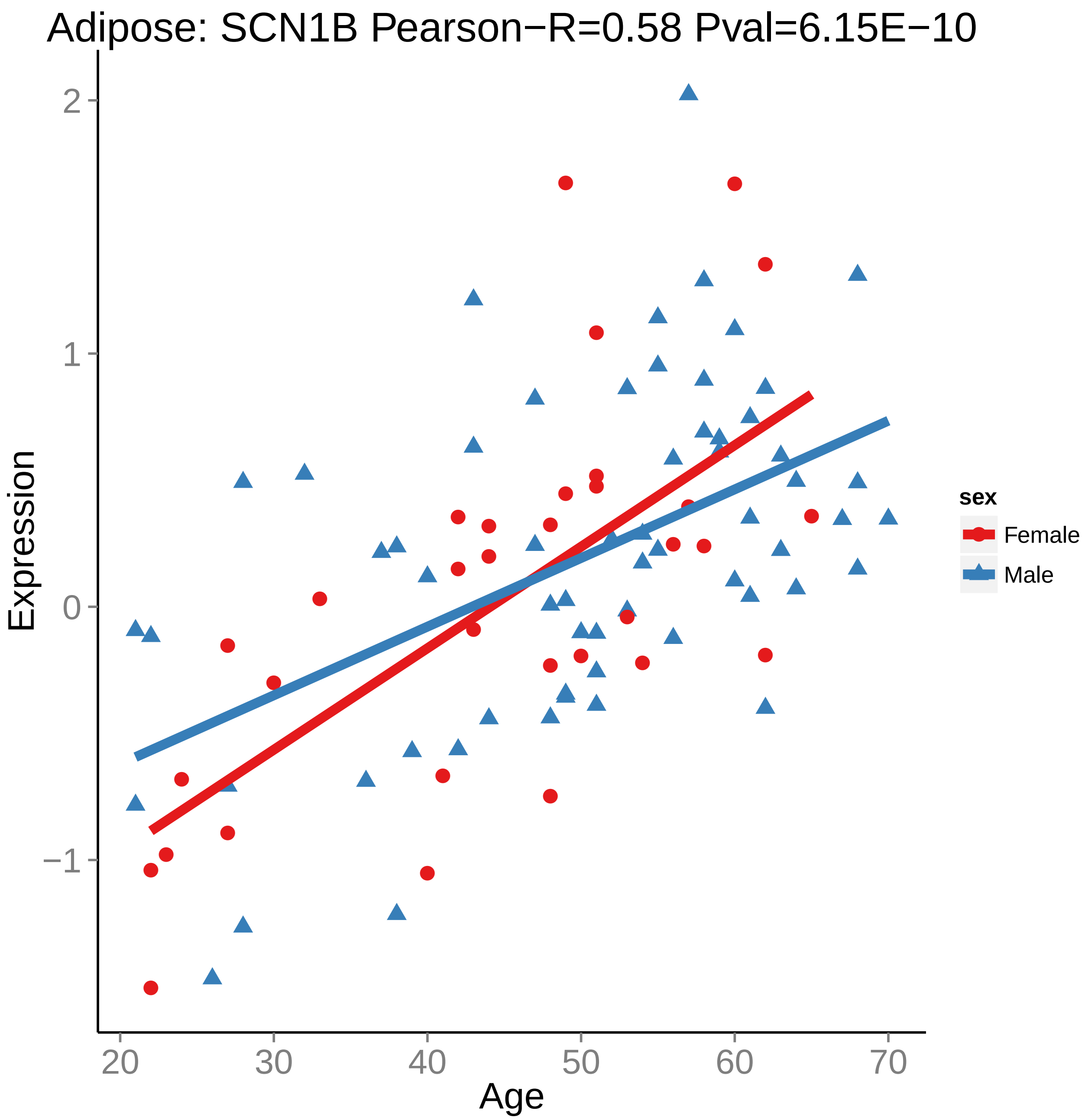
Adipose: SCN1B Pearson−R=0.58 Pval=6.15E−10
Expression
2
1
0
−1
20
30
40
50
60
70
Age
sex
Female
Male

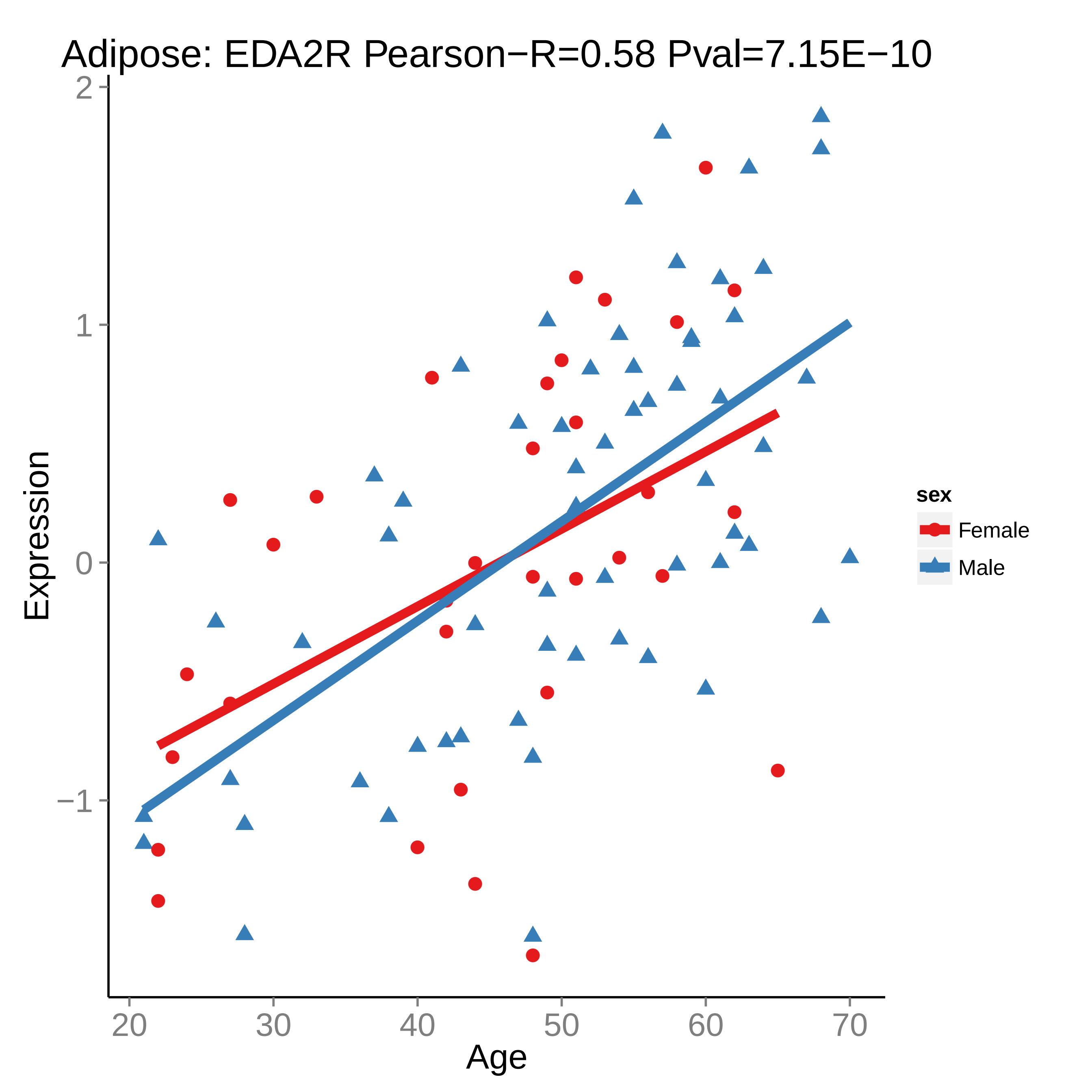

Adipose: EDA2R Pearson−R=0.58 Pval=7.15E−10
2
1
0
−1
Expression
20
30
40
50
60
70
Age
sex
Female
Male

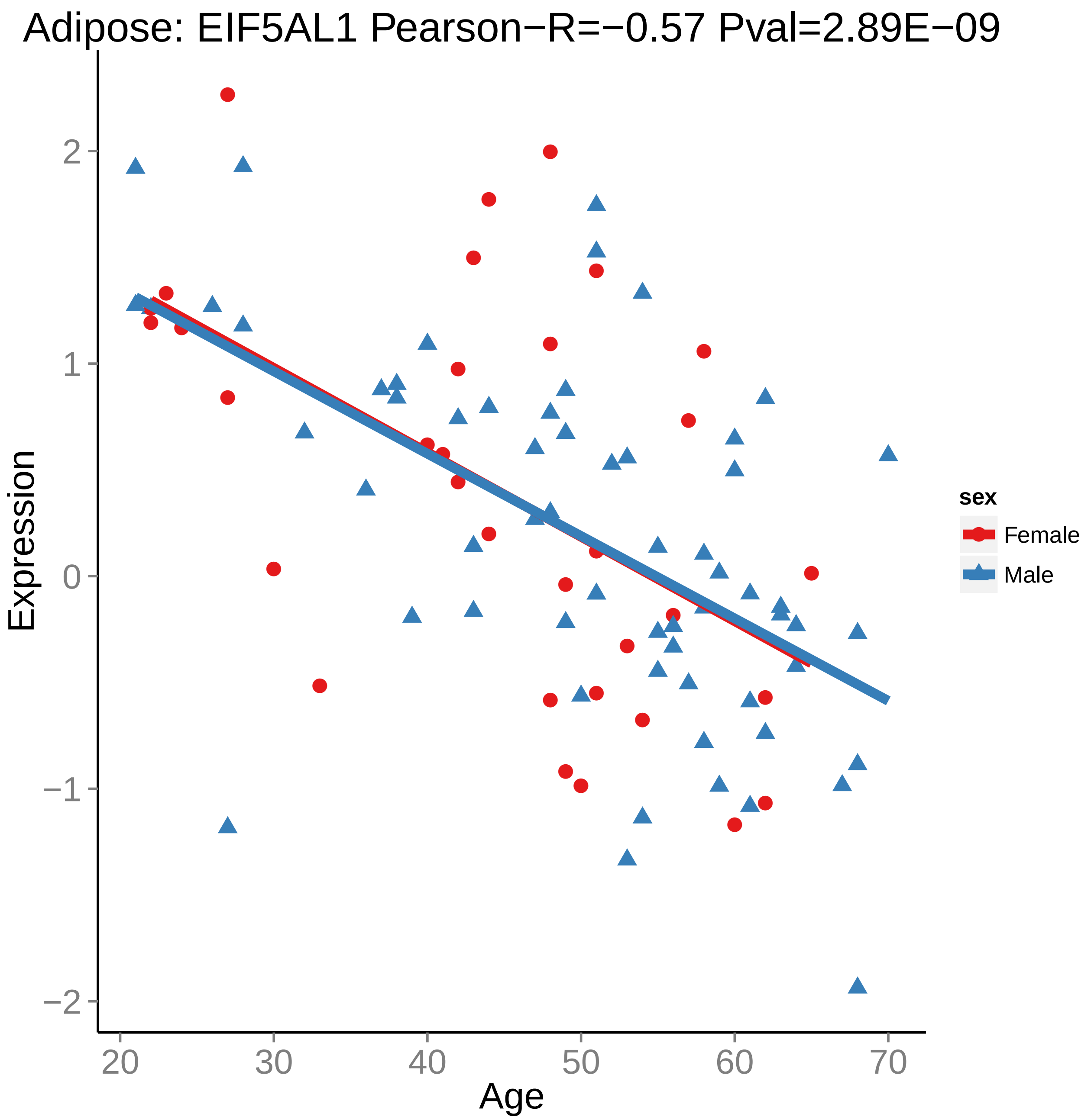

Adipose: EIF5AL1 Pearson−R=−0.57 Pval=2.89E−09
Expression
Age
2
1
0
−1
−2
20
30
40
50
60
70
sex
Female
Male

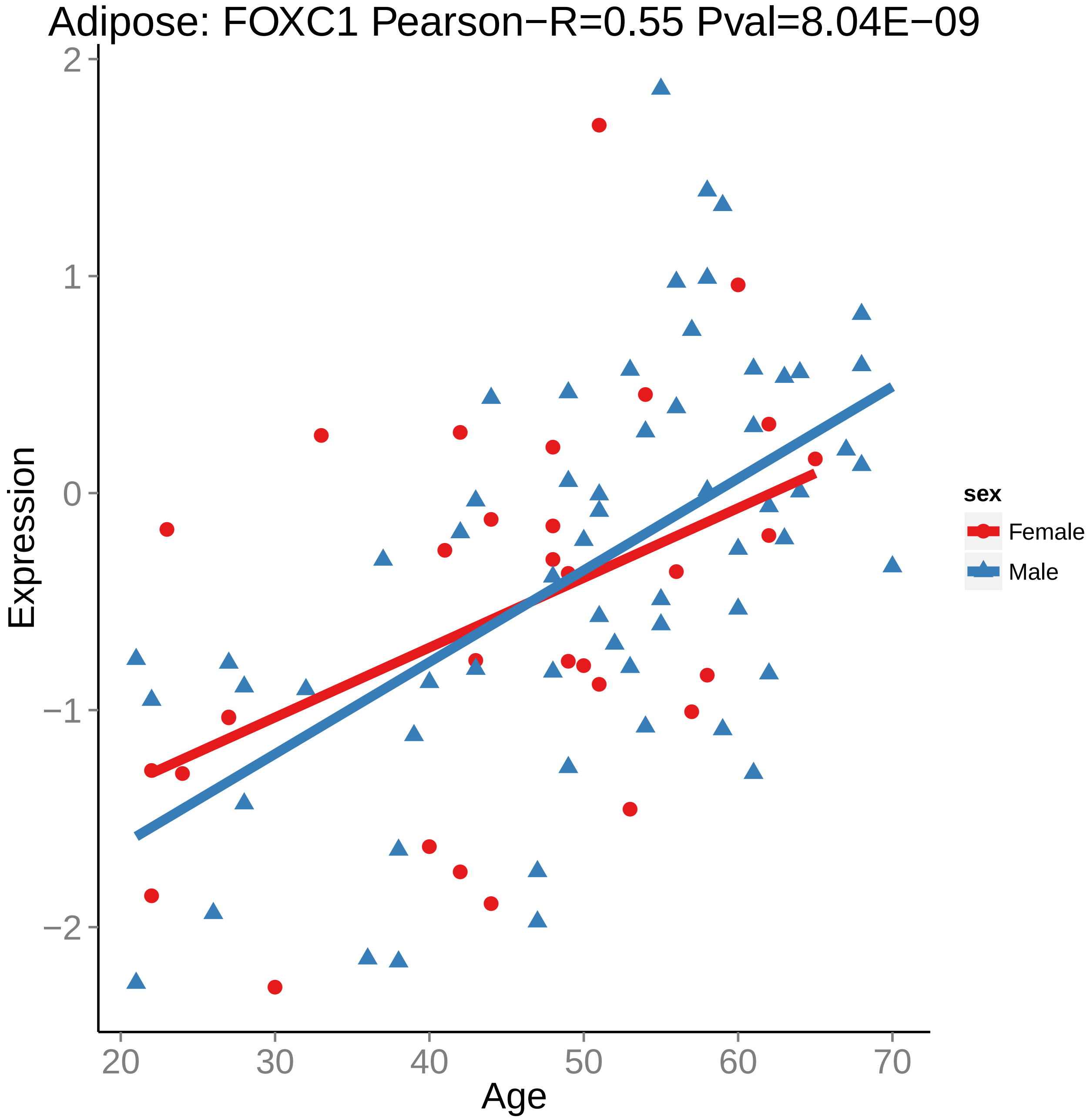

Adipose: FOXC1 Pearson−R=0.55 Pval=8.04E−09
Expression
Age
2
1
0
−1
−2
20
30
40
50
60
70
sex
Female
Male

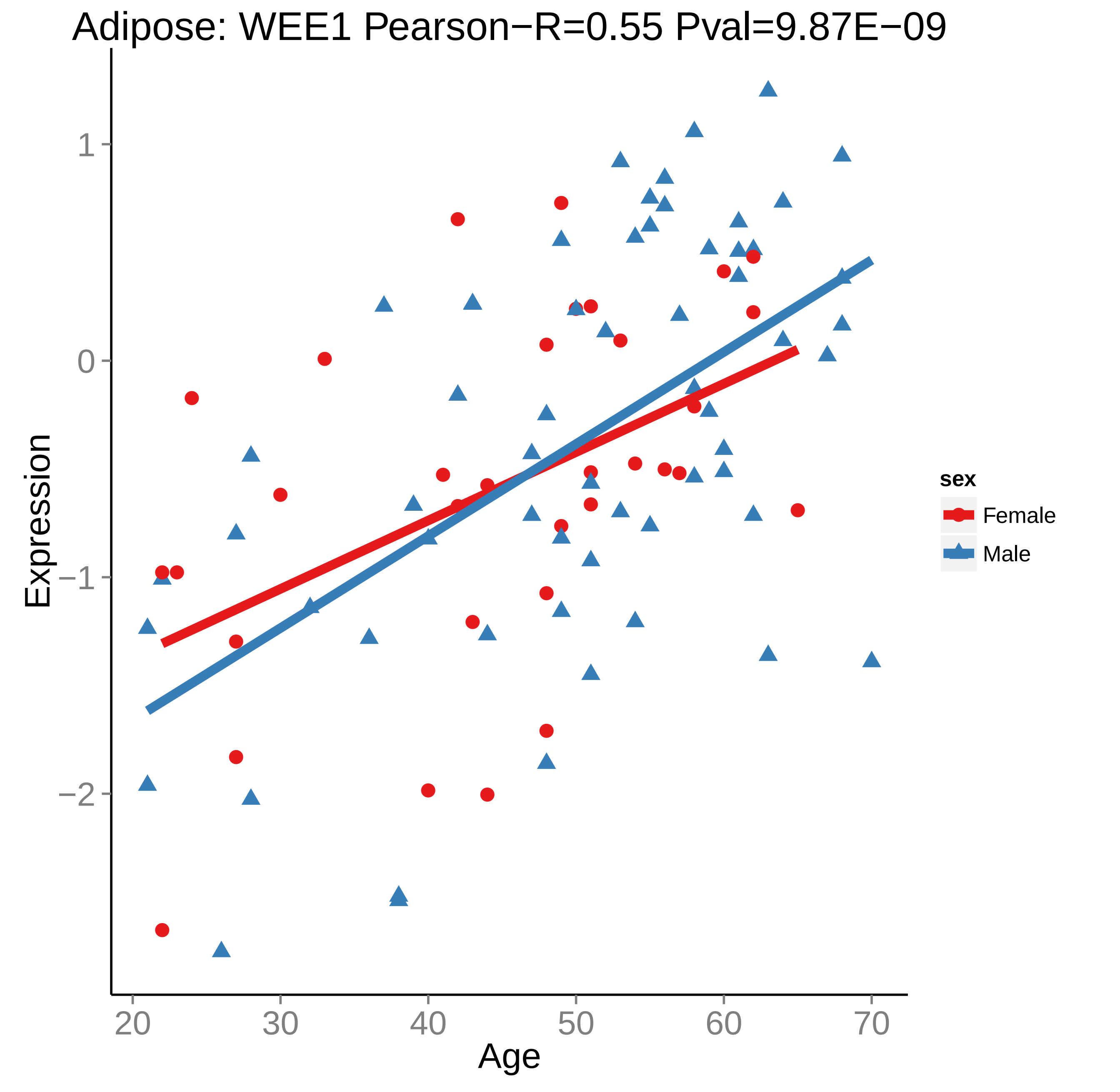
Adipose: WEE1 Pearson−R=0.55 Pval=9.87E−09
Expression
1
0
−1
−2
20
30
40
50
60
70
Age
sex
Female
Male

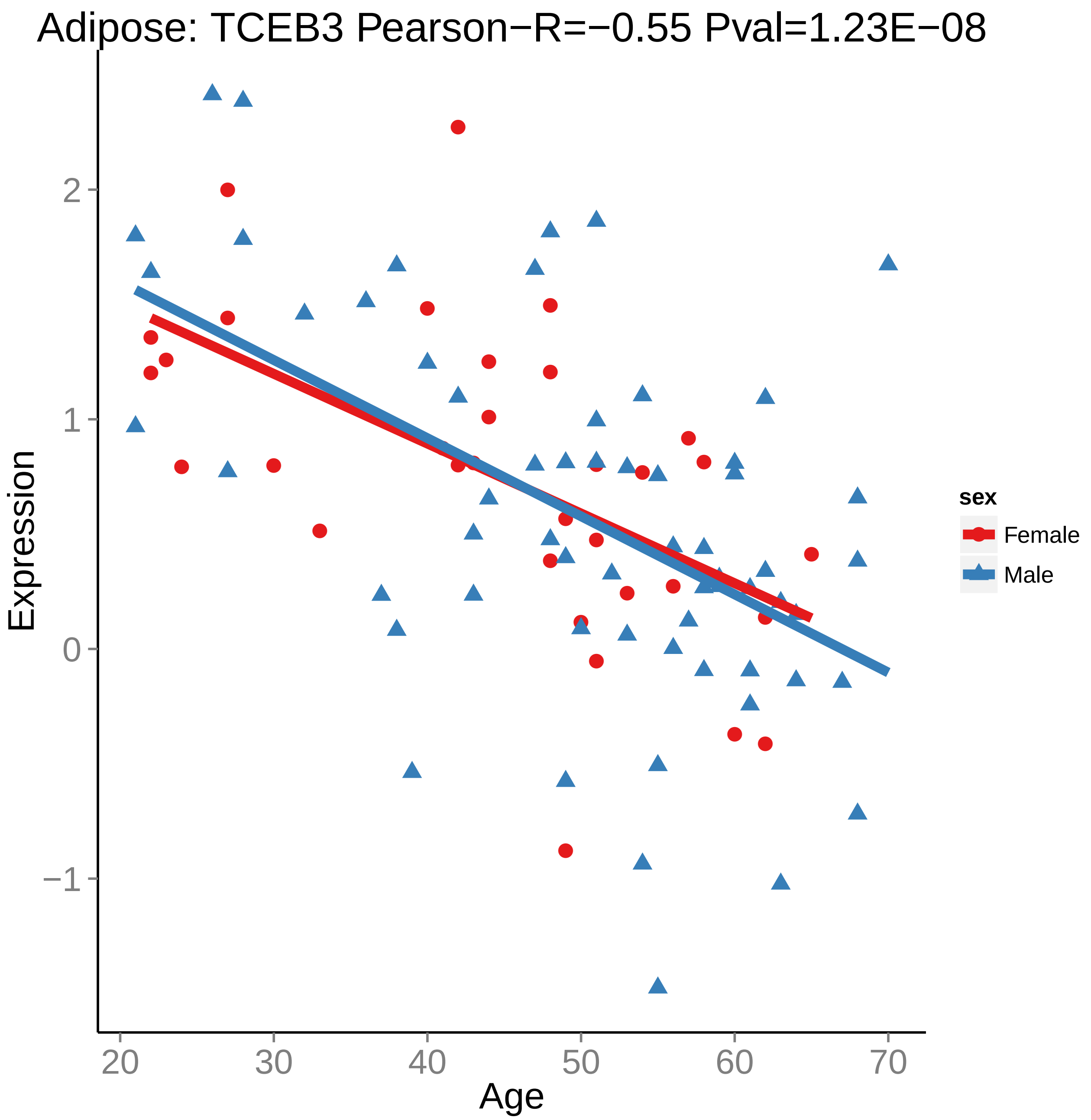
Adipose: TCEB3 Pearson−R=−0.55 Pval=1.23E−08
Expression
2
1
0
−1
20
30
40
50
60
70
Age
sex
Female
Male

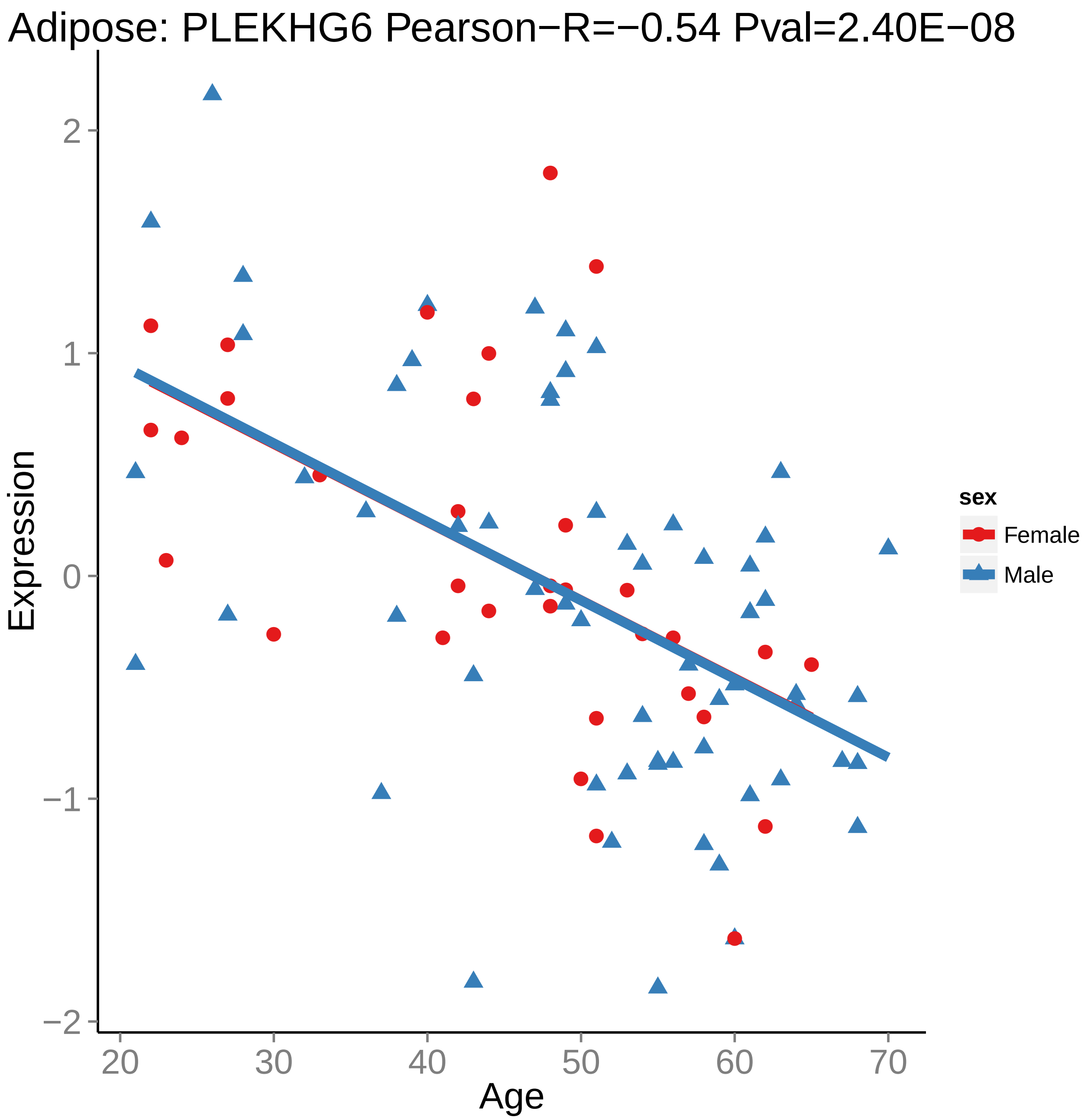

Adipose: PLEKHG6 Pearson−R=−0.54 Pval=2.40E−08
Expression
Age
2
1
0
−1
−2
20
30
40
50
60
70
sex
Female
Male

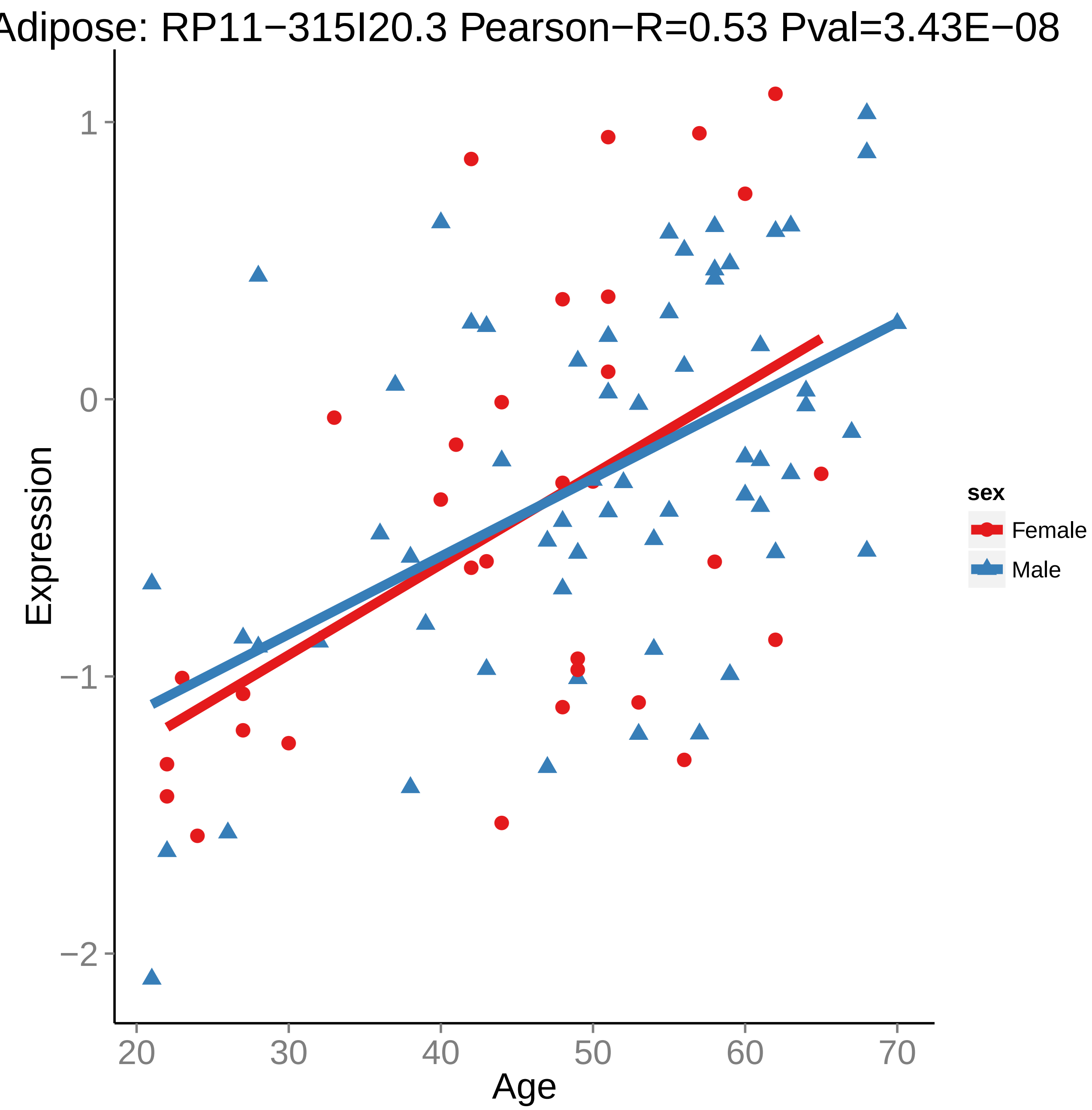

Adipose: RP11−315I20.3 Pearson−R=0.53 Pval=3.43E−08
Expression
1
0
−1
−2
20
30
40
50
60
70
Age
sex
Female
Male

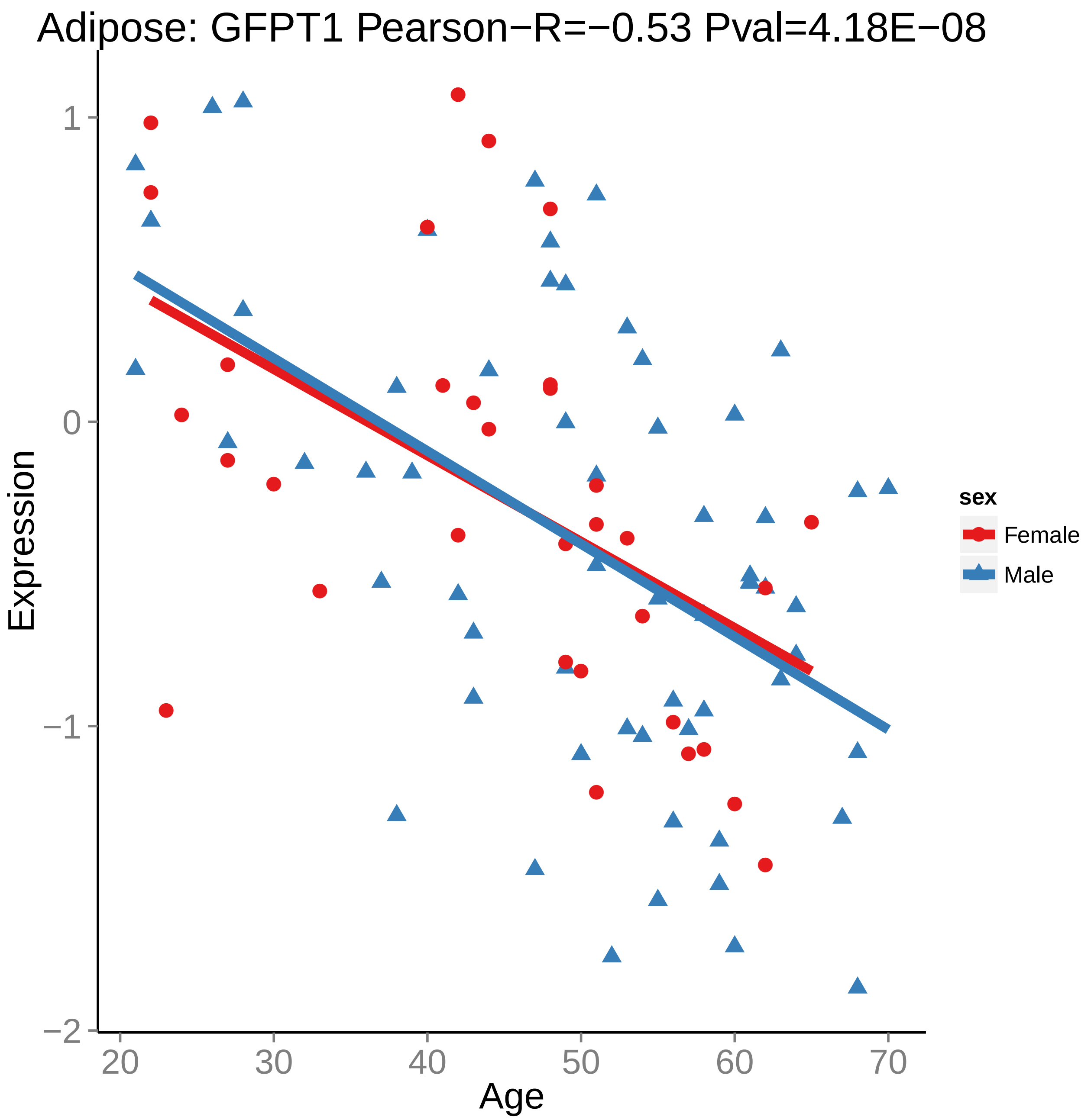

Adipose: GFPT1 Pearson−R=−0.53 Pval=4.18E−08
Expression
Age
1
0
−1
−2
20
30
40
50
60
70
sex
Female
Male

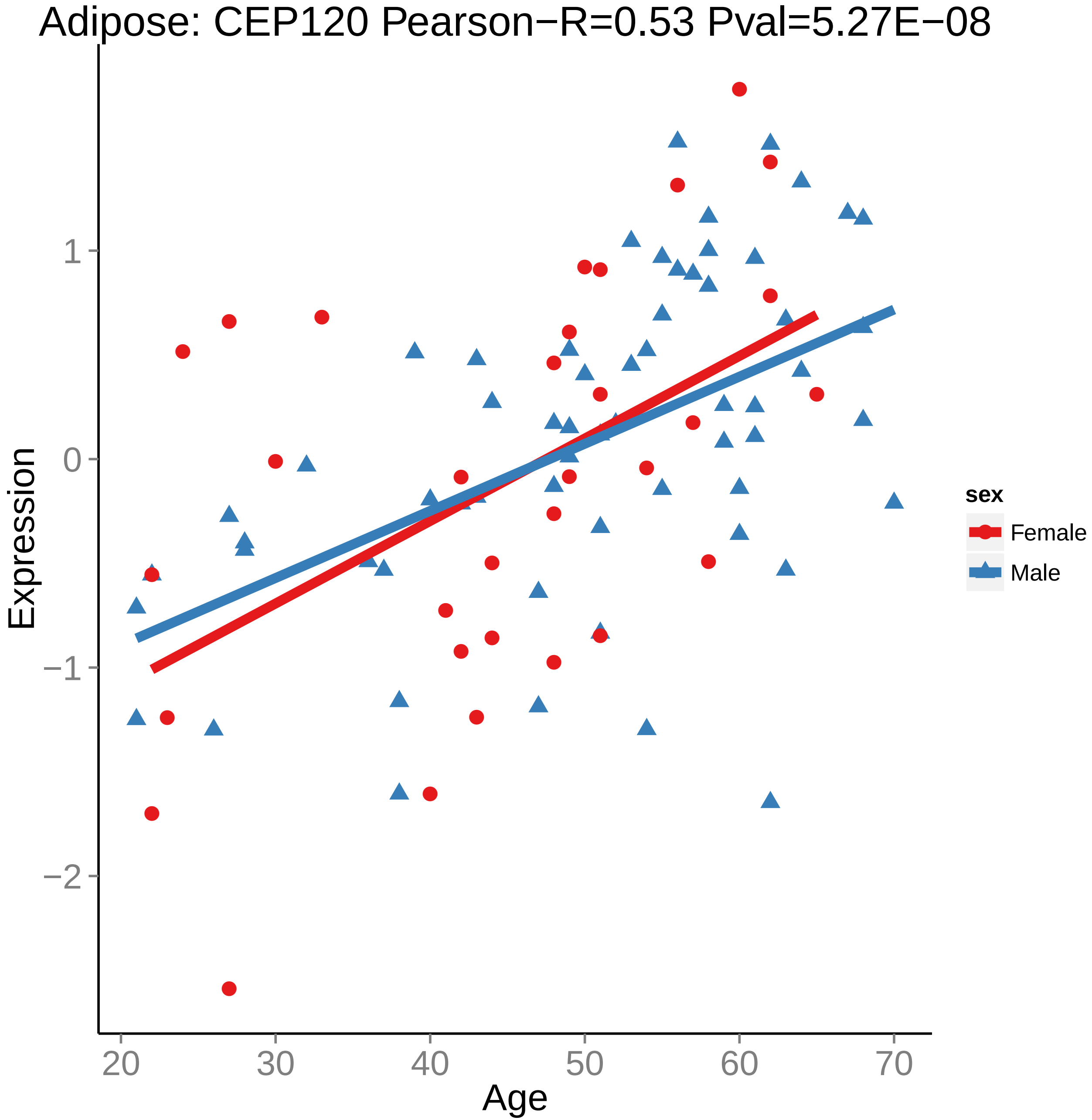

Adipose: CEP120 Pearson−R=0.53 Pval=5.27E−08
Expression
1
0
−1
−2
20
30
40
50
60
70
Age
sex
Female
Male

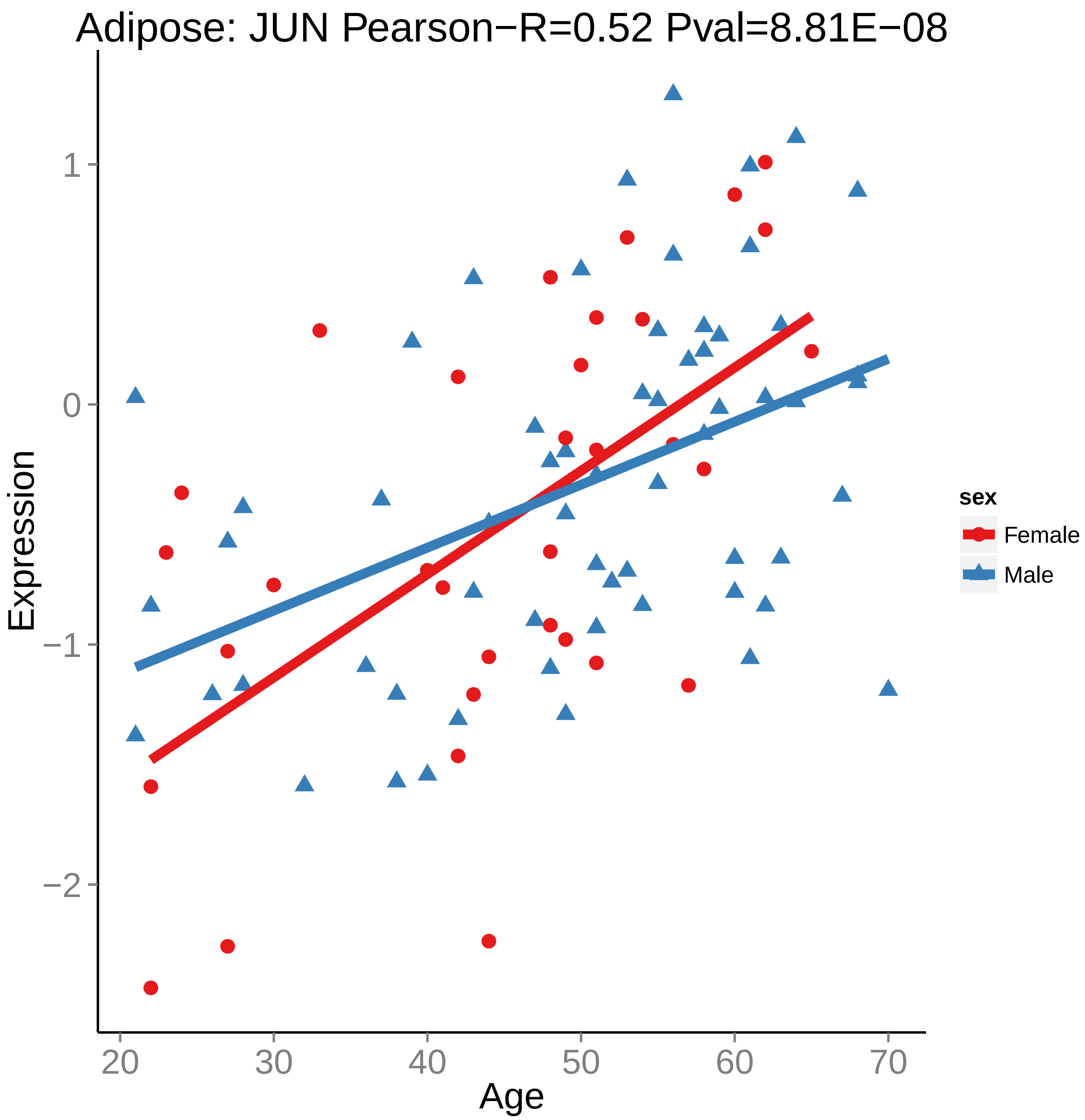

Adipose: JUN Pearson−R=0.52 Pval=8.81E−08
Expression
1
0
−1
−2
20
30
40
50
60
70
Age
sex
Female
Male

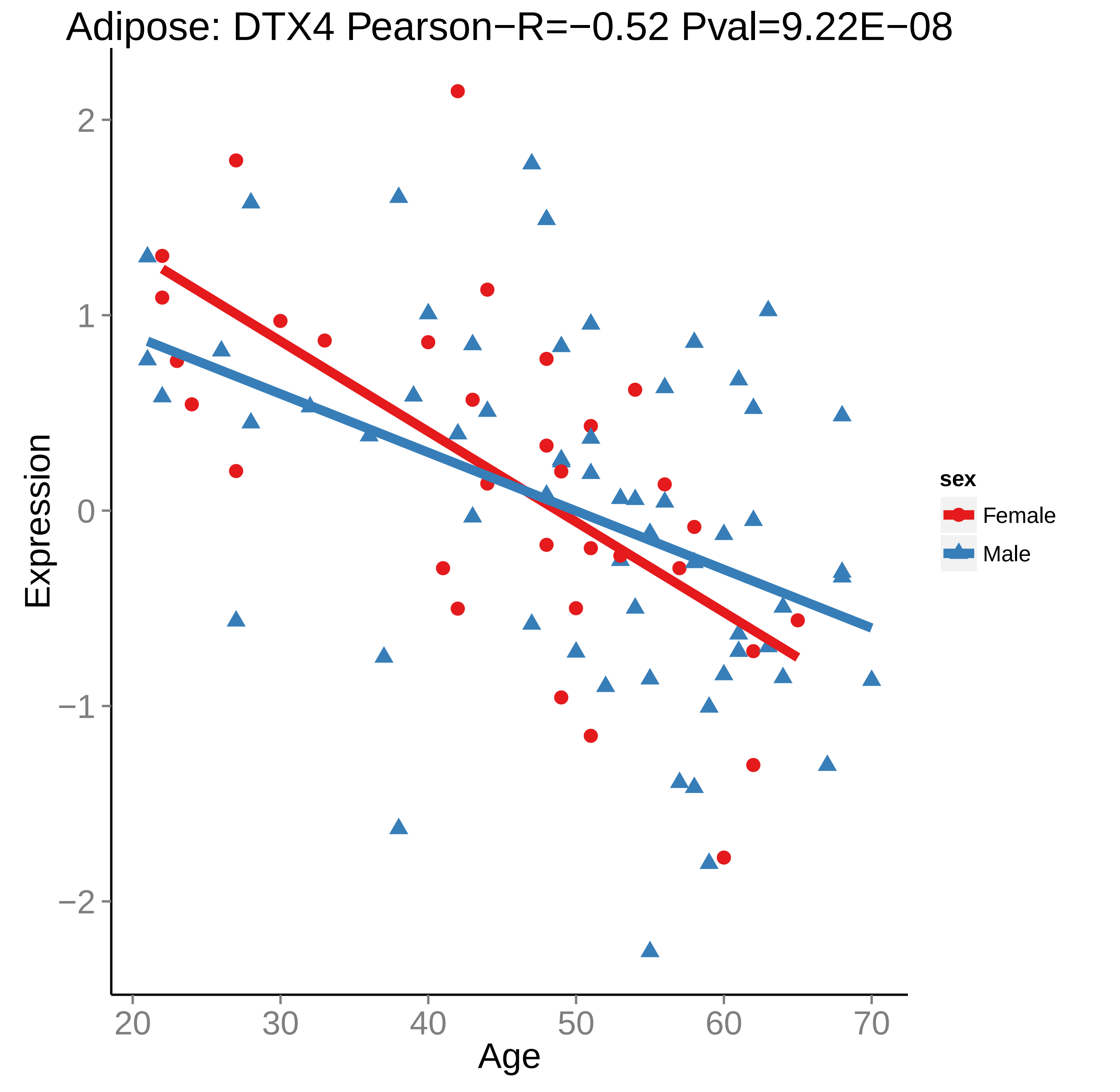

Adipose: DTX4 Pearson−R=−0.52 Pval=9.22E−08
Expression
2
1
0
−1
−2
20
30
40
50
60
70
Age
sex
Female
Male

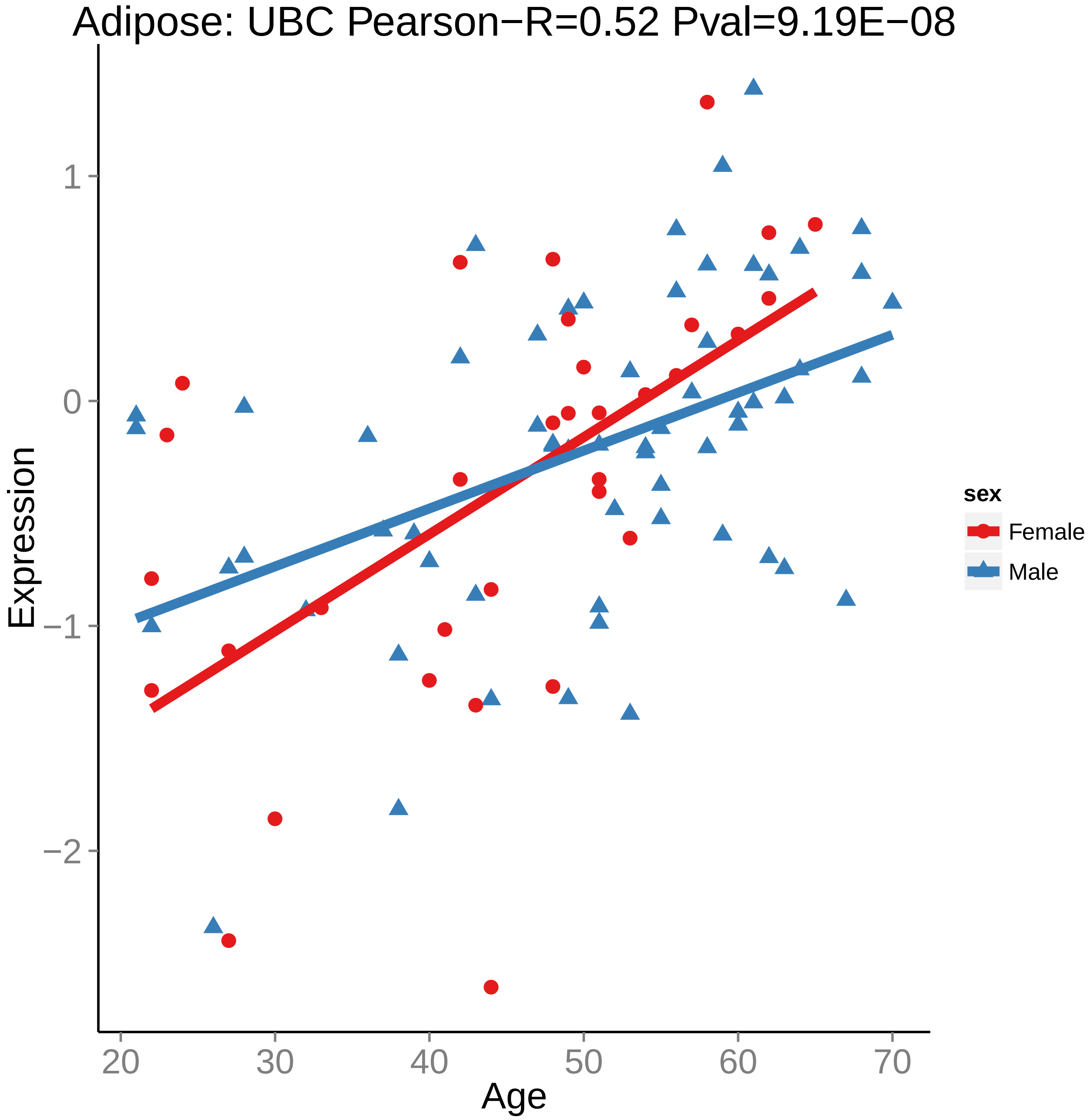

Adipose: UBC Pearson−R=0.52 Pval=9.19E−08
Expression
1
0
−1
−2
20
30
40
50
60
70
Age
sex
Female
Male

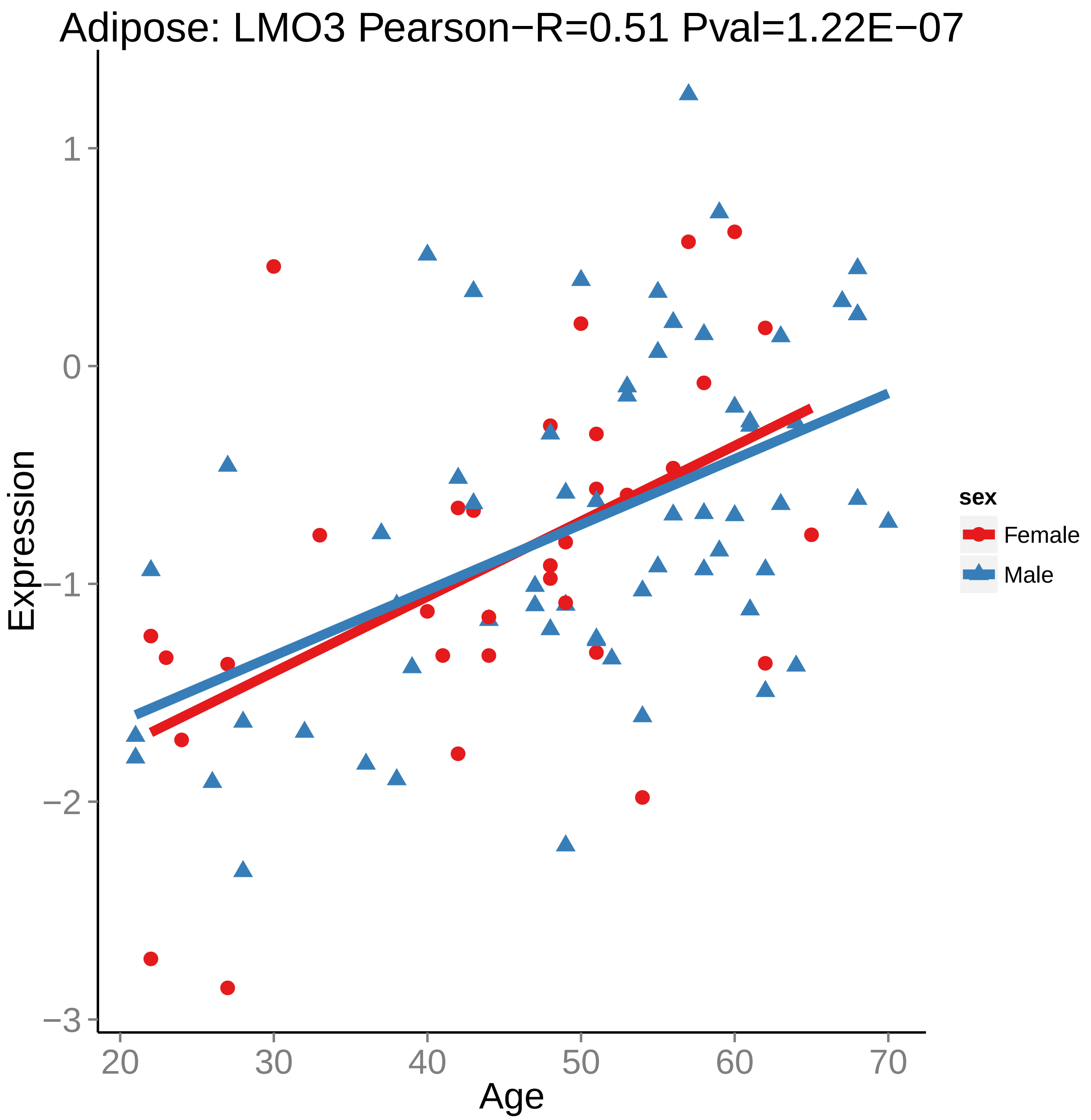

Adipose: LMO3 Pearson−R=0.51 Pval=1.22E−07
Expression
1
0
−1
−2
−3
20
30
40
50
60
70
Age
sex
Female
Male

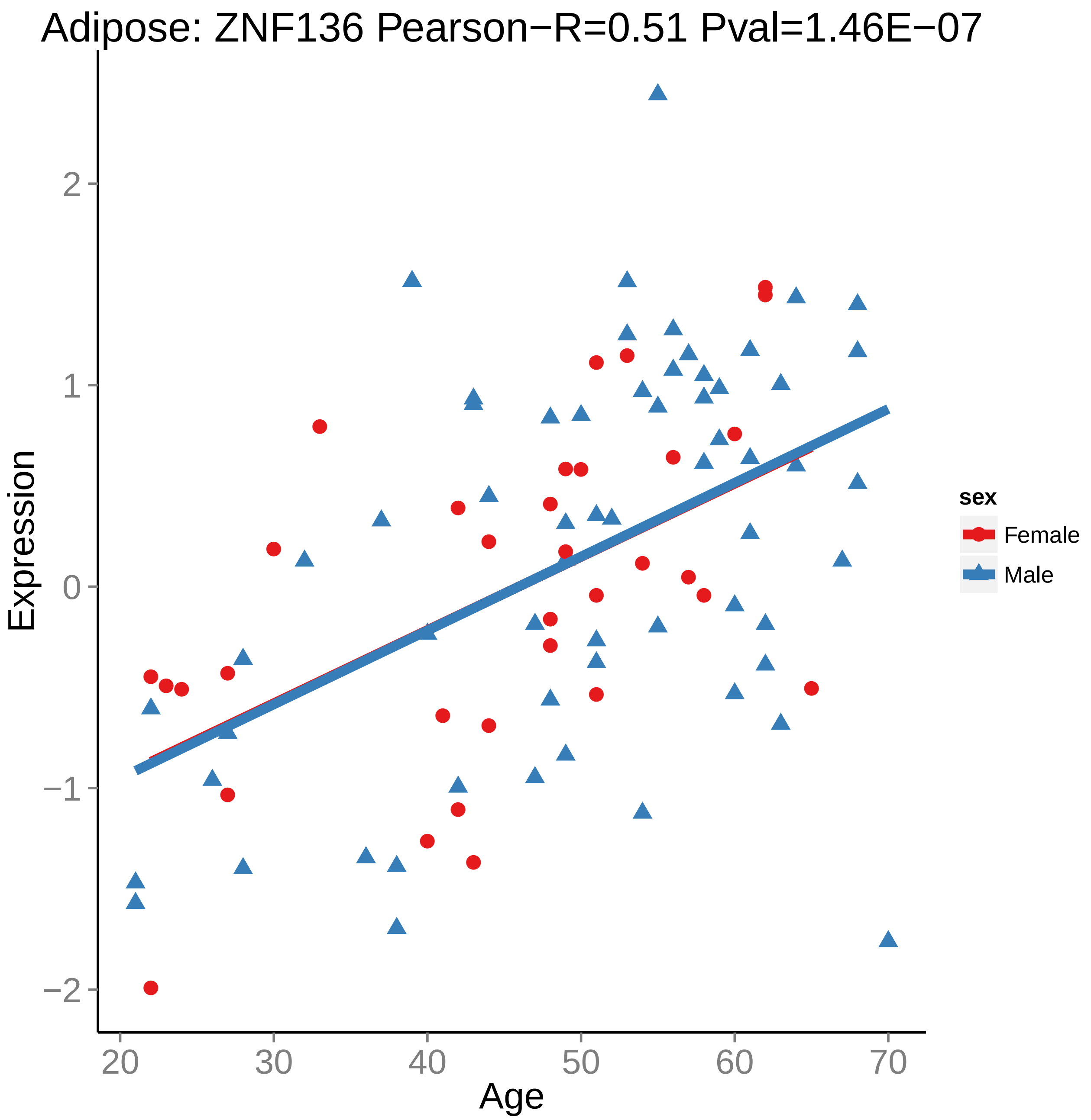

Adipose: ZNF136 Pearson−R=0.51 Pval=1.46E−07
Expression
Age
2
1
0
−1
−2
20
30
40
50
60
70
sex
Female
Male

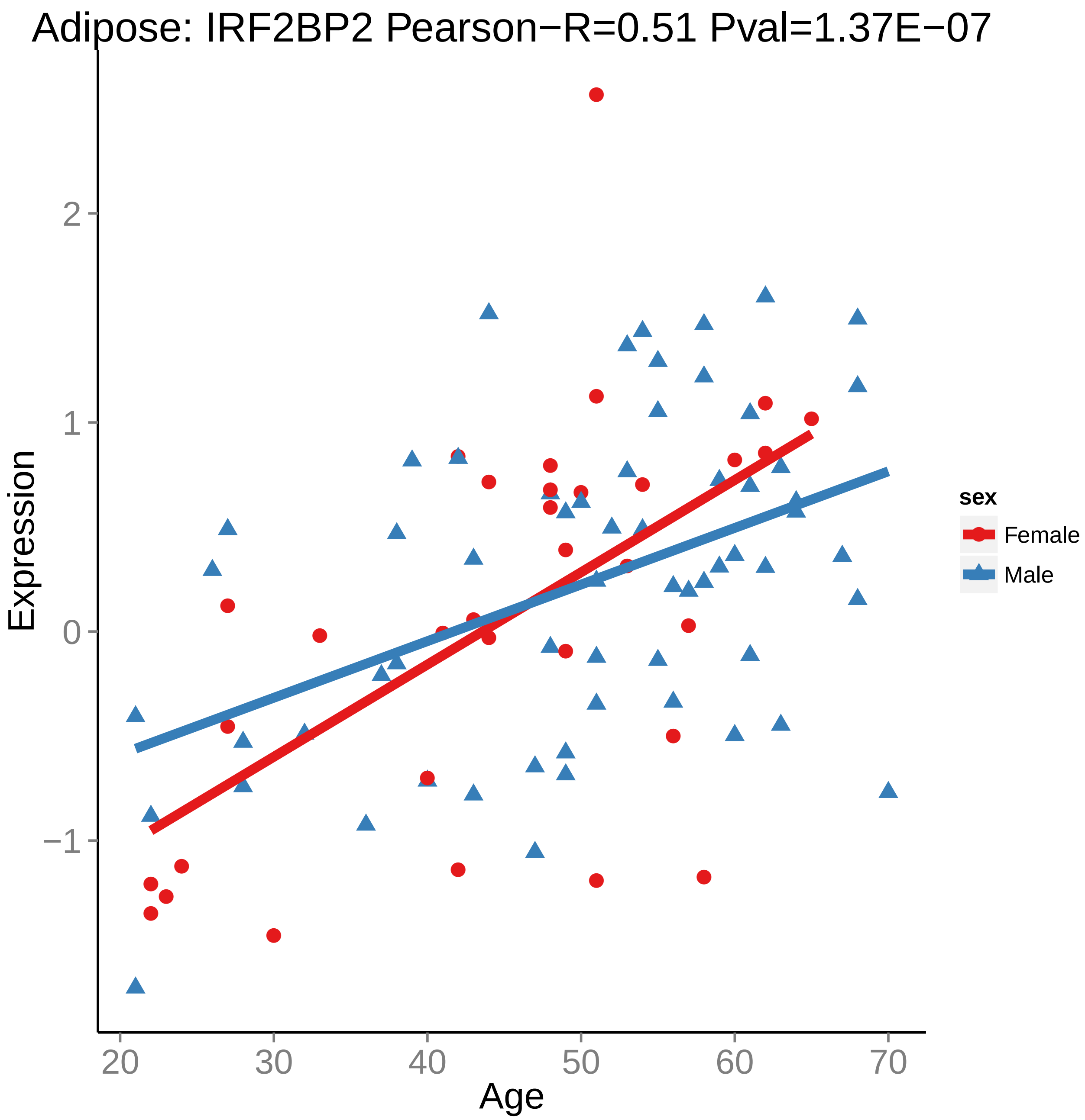

Adipose: IRF2BP2 Pearson−R=0.51 Pval=1.37E−07
Expression
2
1
0
−1
20
30
40
50
60
70
Age
sex
Female
Male

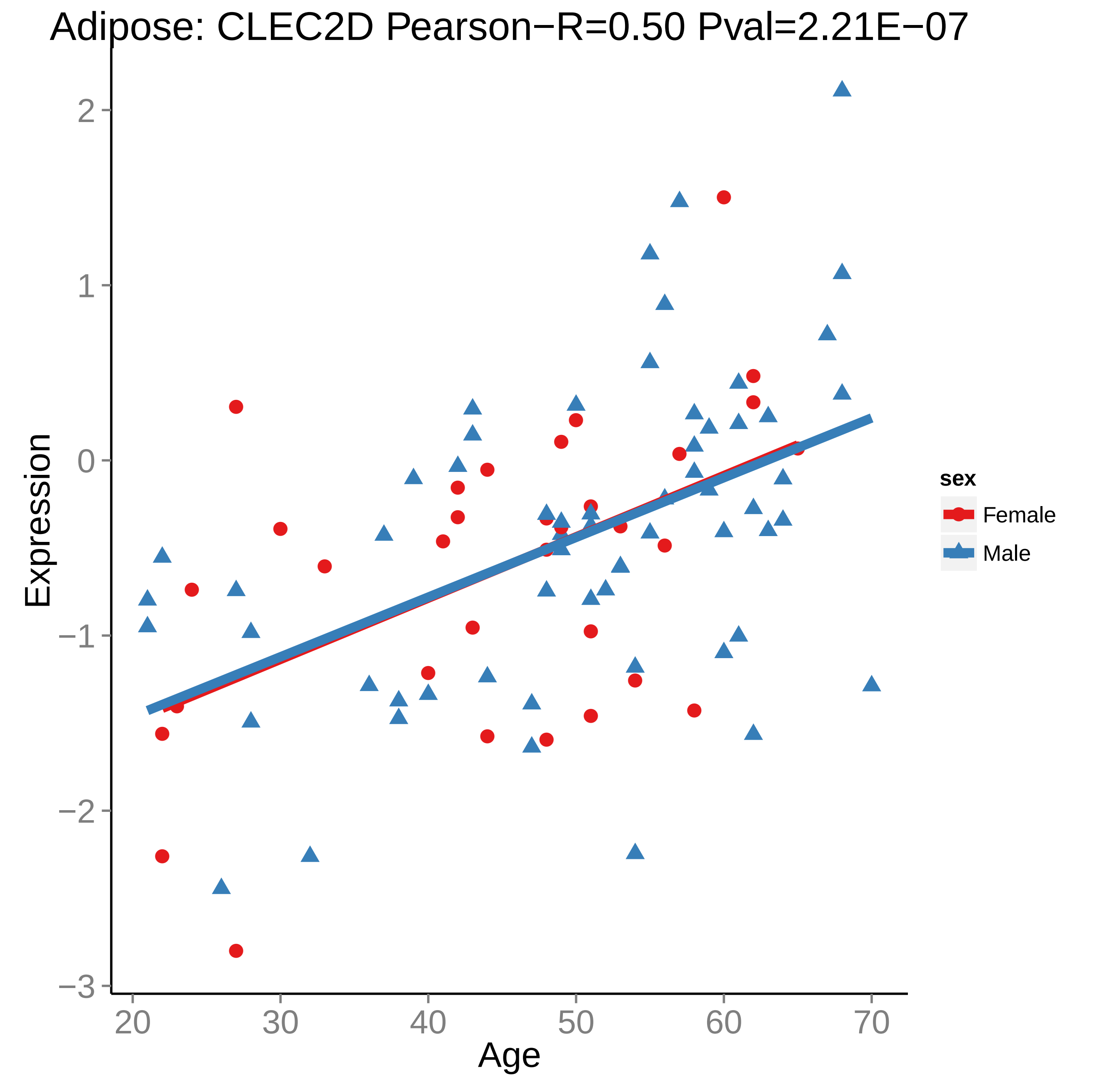

Adipose: CLEC2D Pearson−R=0.50 Pval=2.21E−07
Expression
Age
2
1
0
−1
−2
−3
20
30
40
50
60
70
sex
Female
Male

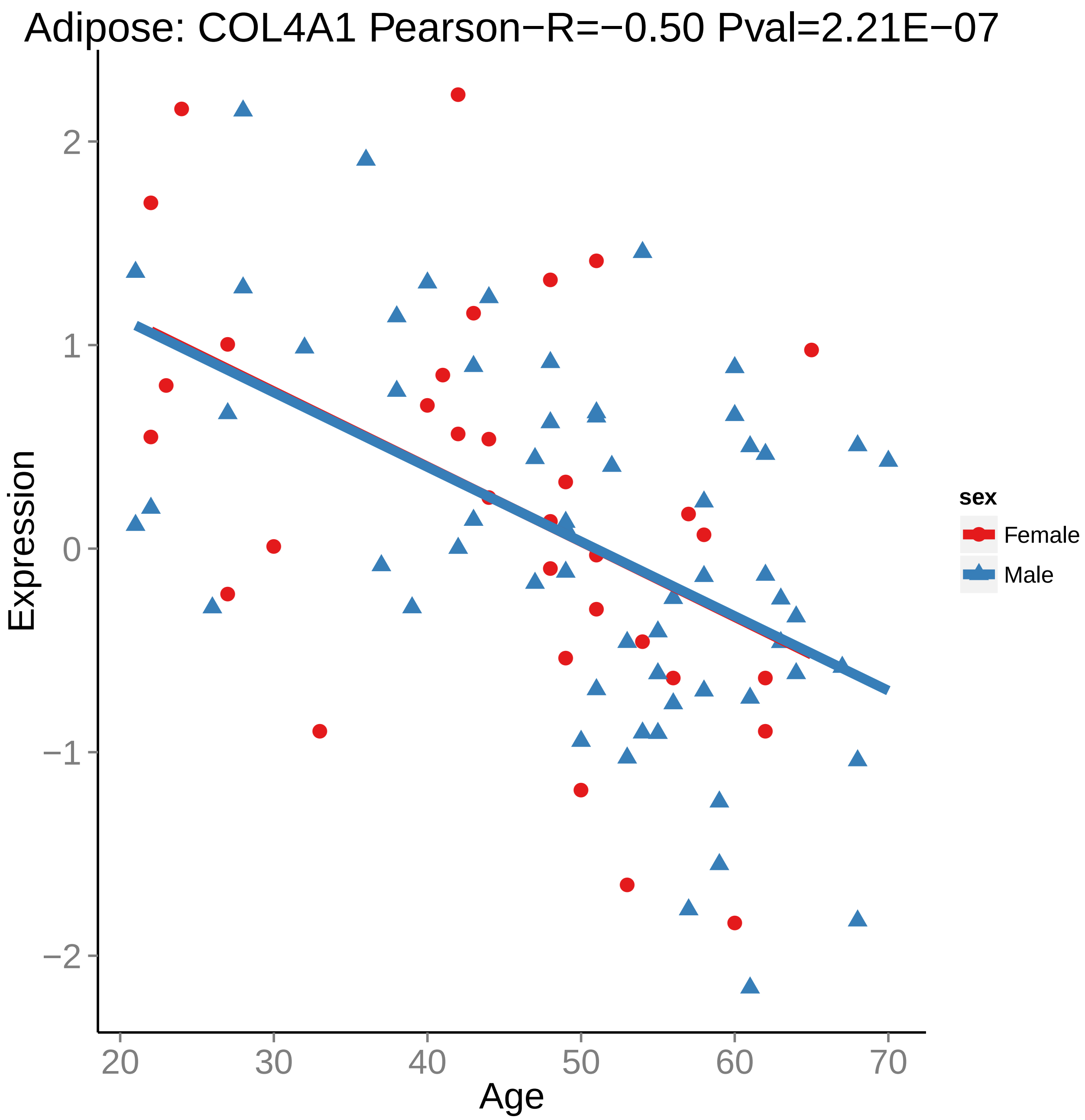

Adipose: COL4A1 Pearson−R=−0.50 Pval=2.21E−07
Expression
Age
2
1
0
−1
−2
20
30
40
50
60
70
sex
Female
Male

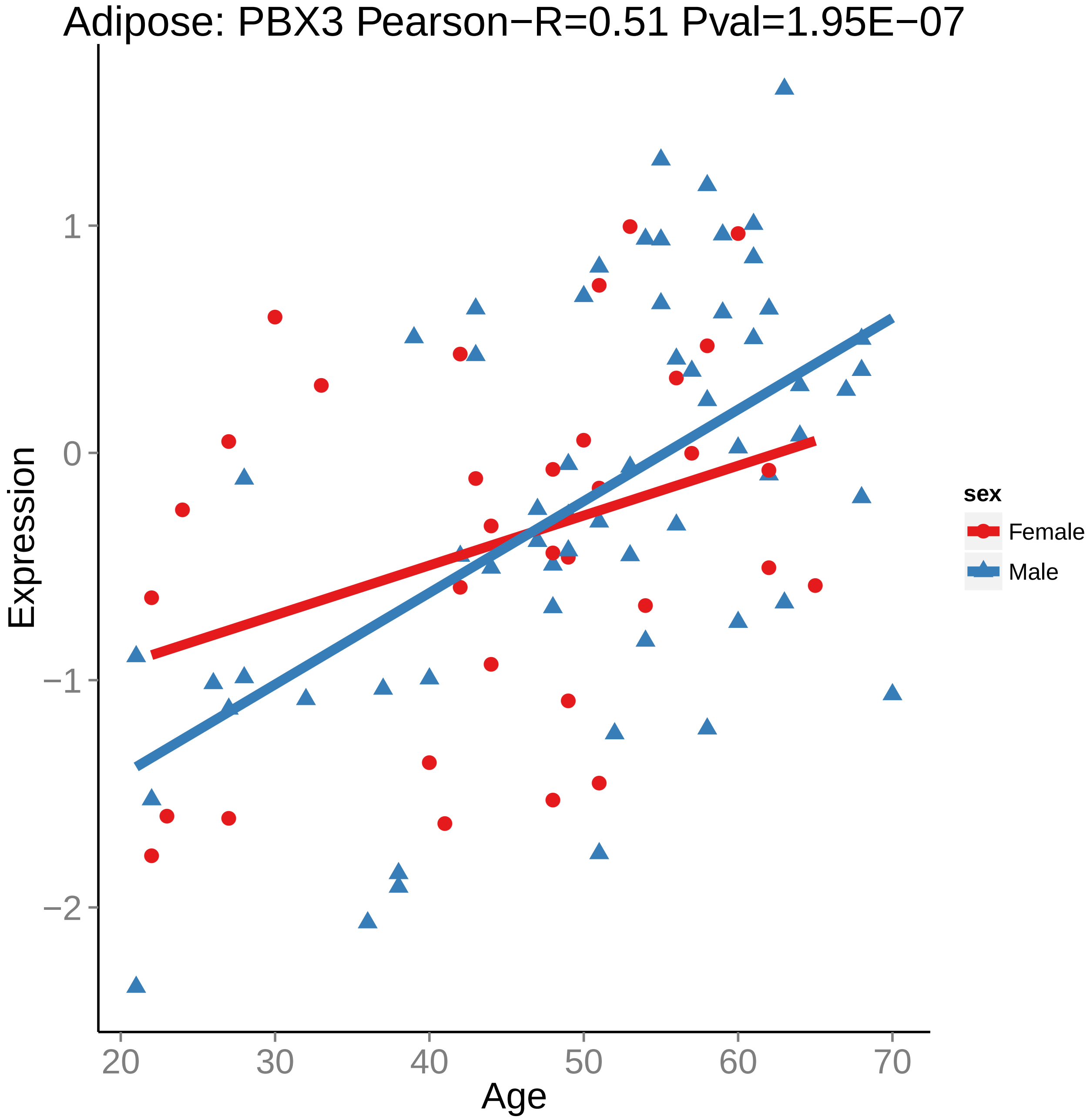

Adipose: PBX3 Pearson−R=0.51 Pval=1.95E−07
Expression
1
0
−1
−2
20
30
40
50
60
70
Age
sex
Female
Male

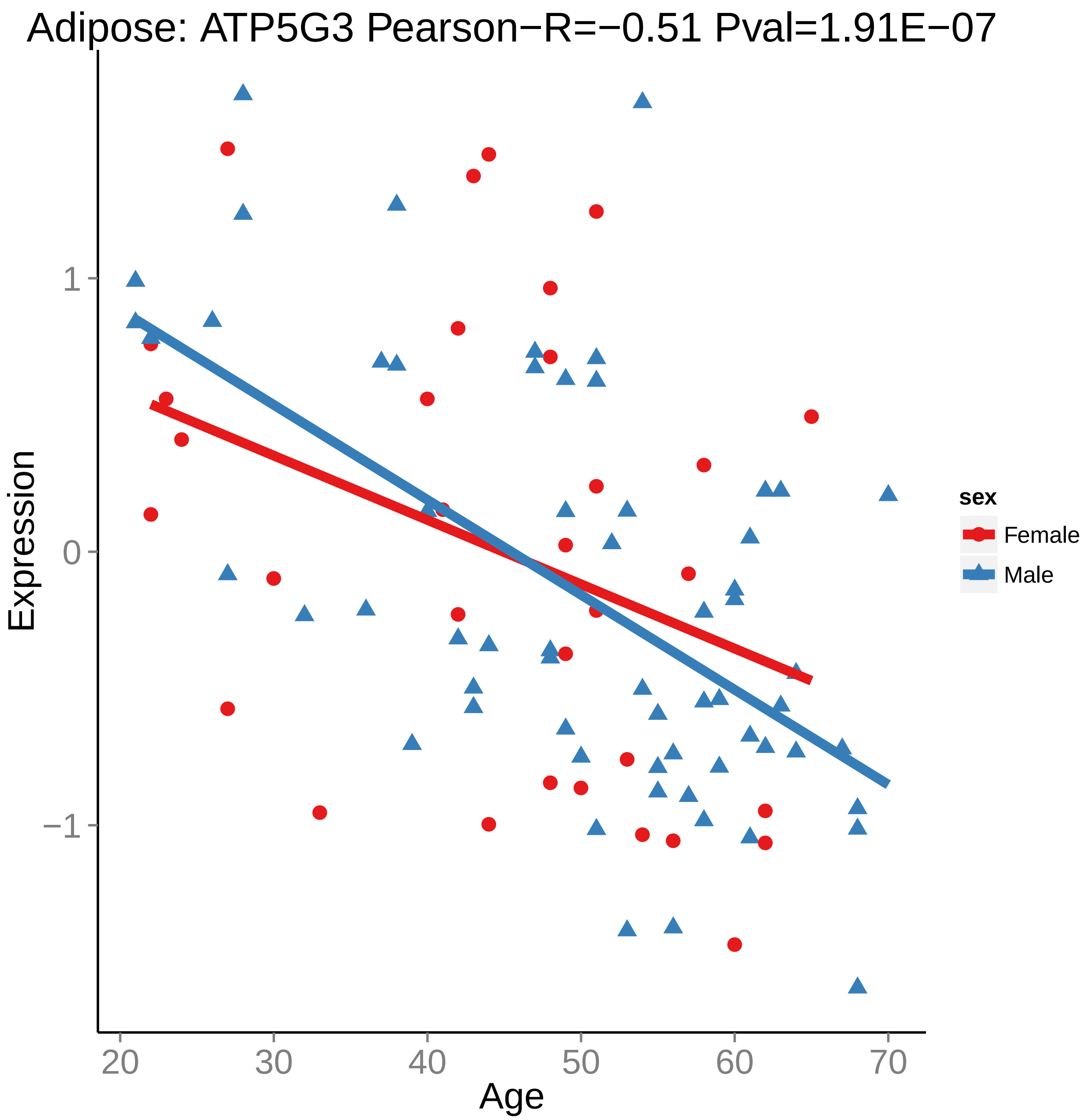

Adipose: ATP5G3 Pearson−R=−0.51 Pval=1.91E−07
Expression
1
0
−1
20
30
40
50
60
70
Age
sex
Female
Male

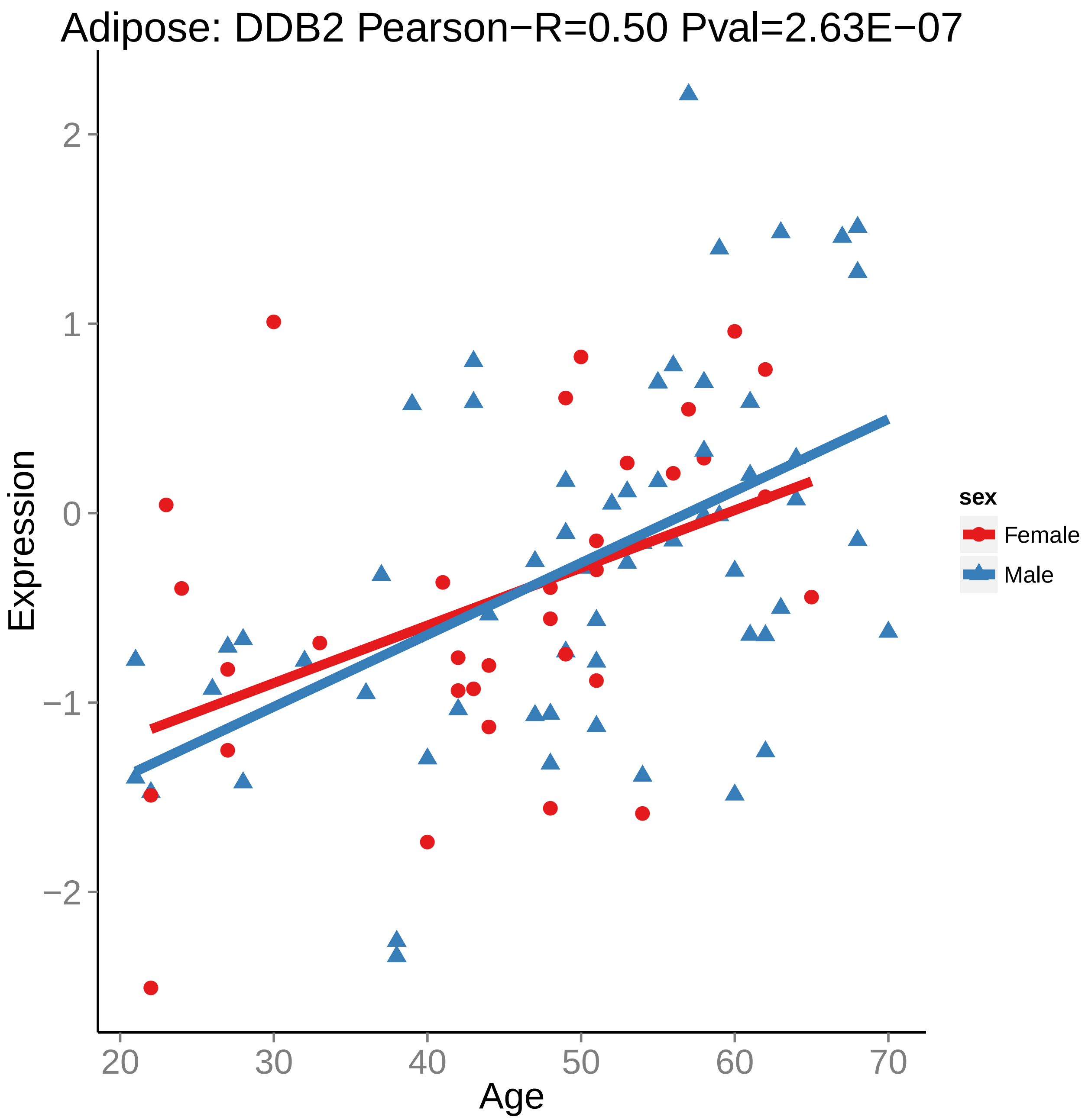
Adipose: DDB2 Pearson−R=0.50 Pval=2.63E−07
Expression
Age
2
1
0
−1
−2
20
30
40
50
60
70
sex
Female
Male

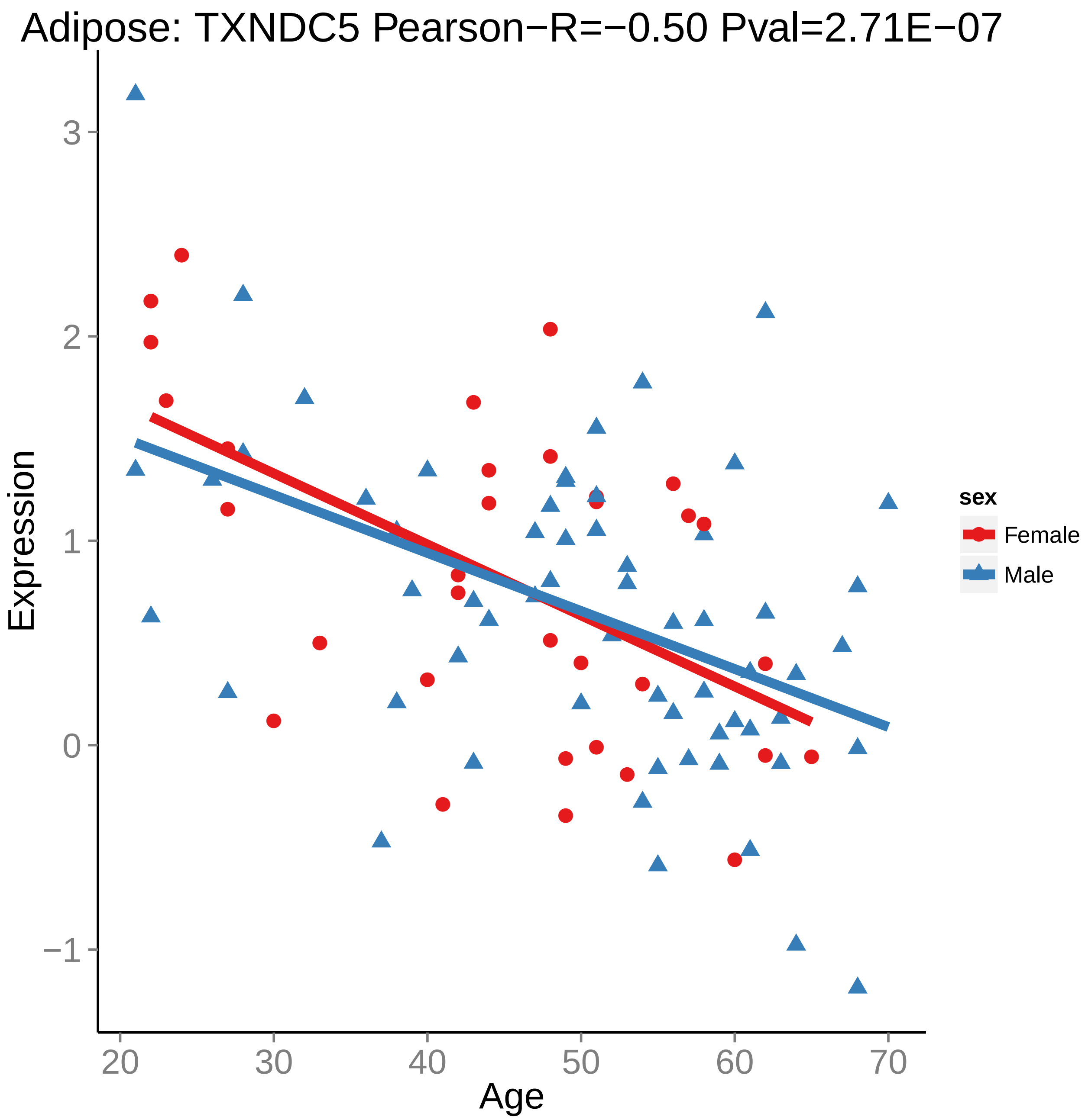

Adipose: TXNDC5 Pearson−R=−0.50 Pval=2.71E−07
Expression
Age
3
2
1
0
−1
20
30
40
50
60
70
sex
Female
Male

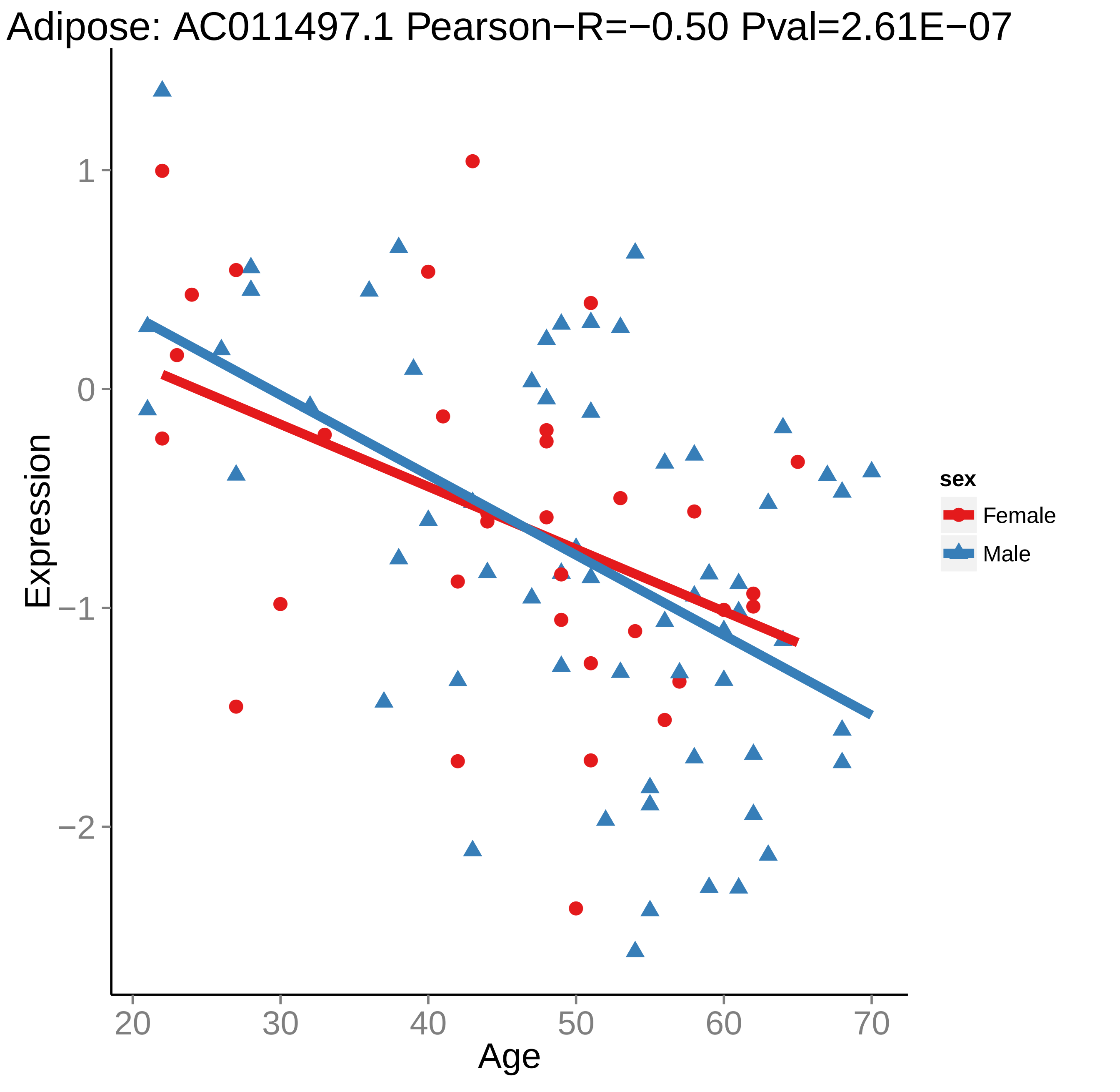

Adipose: AC011497.1 Pearson−R=−0.50 Pval=2.61E−07
Expression
1
0
−1
−2
20
30
40
50
60
70
Age
sex
Female
Male

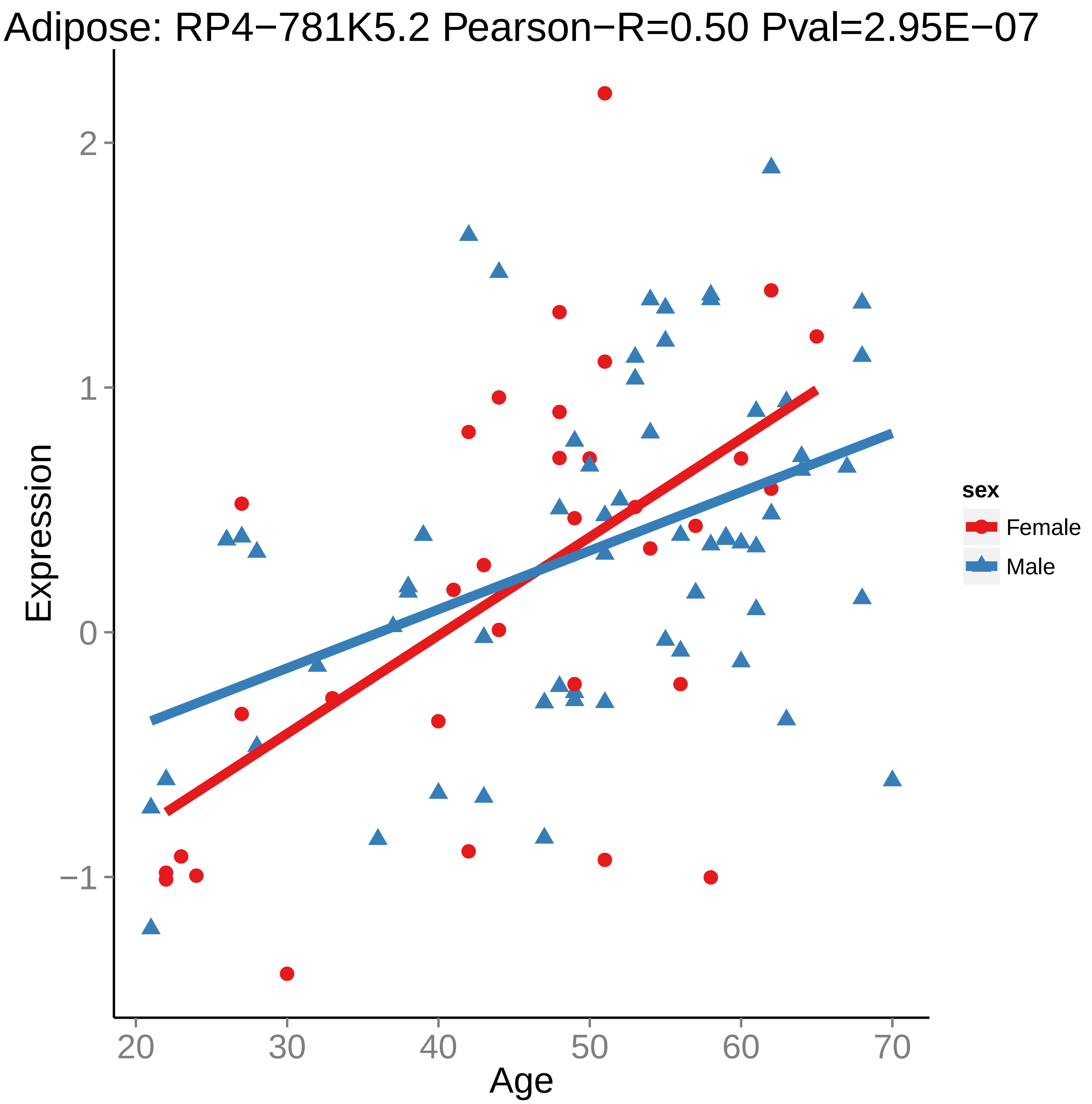
Adipose: RP4−781K5.2 Pearson−R=0.50 Pval=2.95E−07
Expression
Age
2
1
0
−1
20
30
40
50
60
70
sex
Female
Male

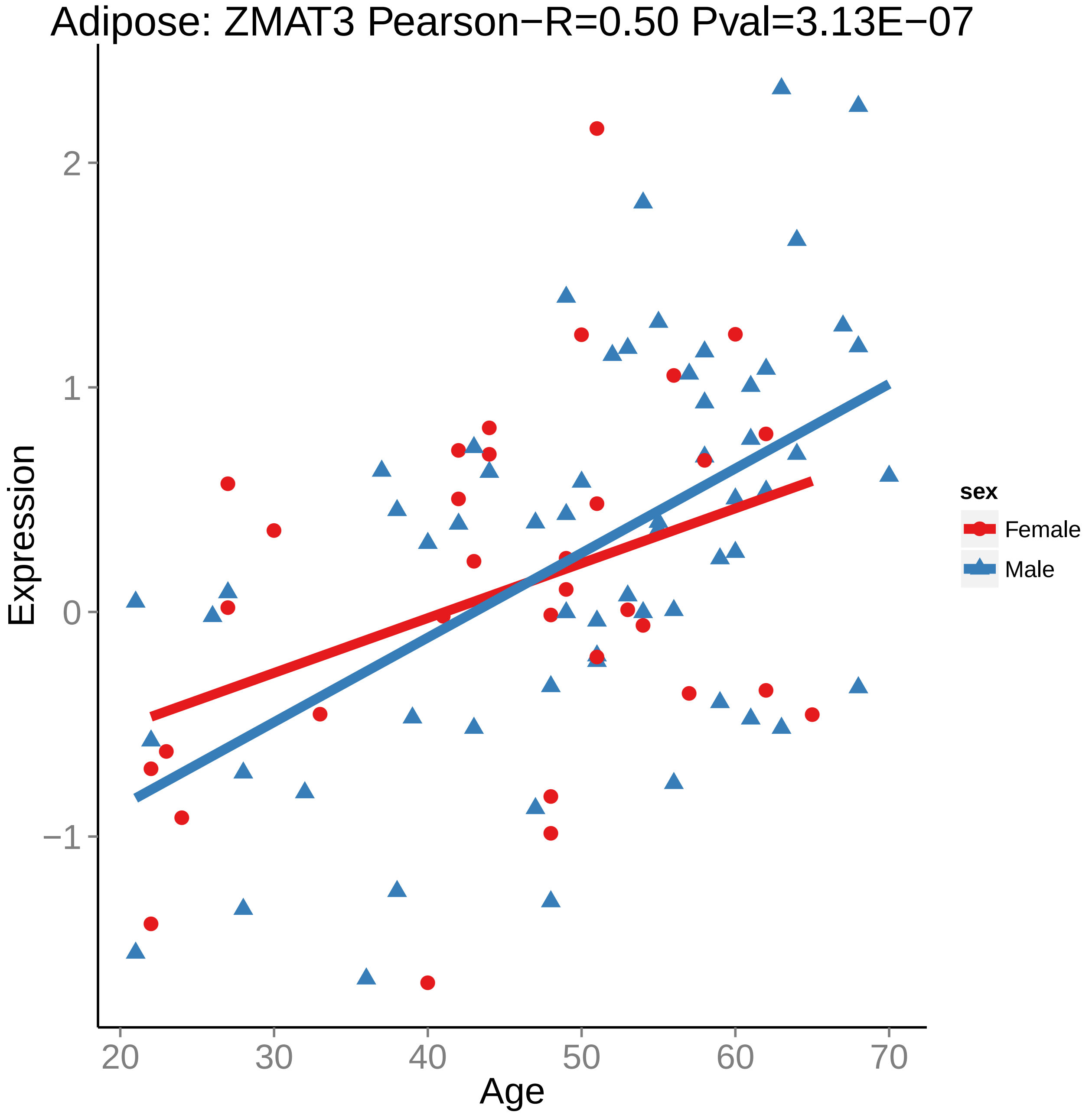

Adipose: ZMAT3 Pearson−R=0.50 Pval=3.13E−07
Expression
2
1
0
−1
20
30
40
50
60
70
Age
sex
Female
Male

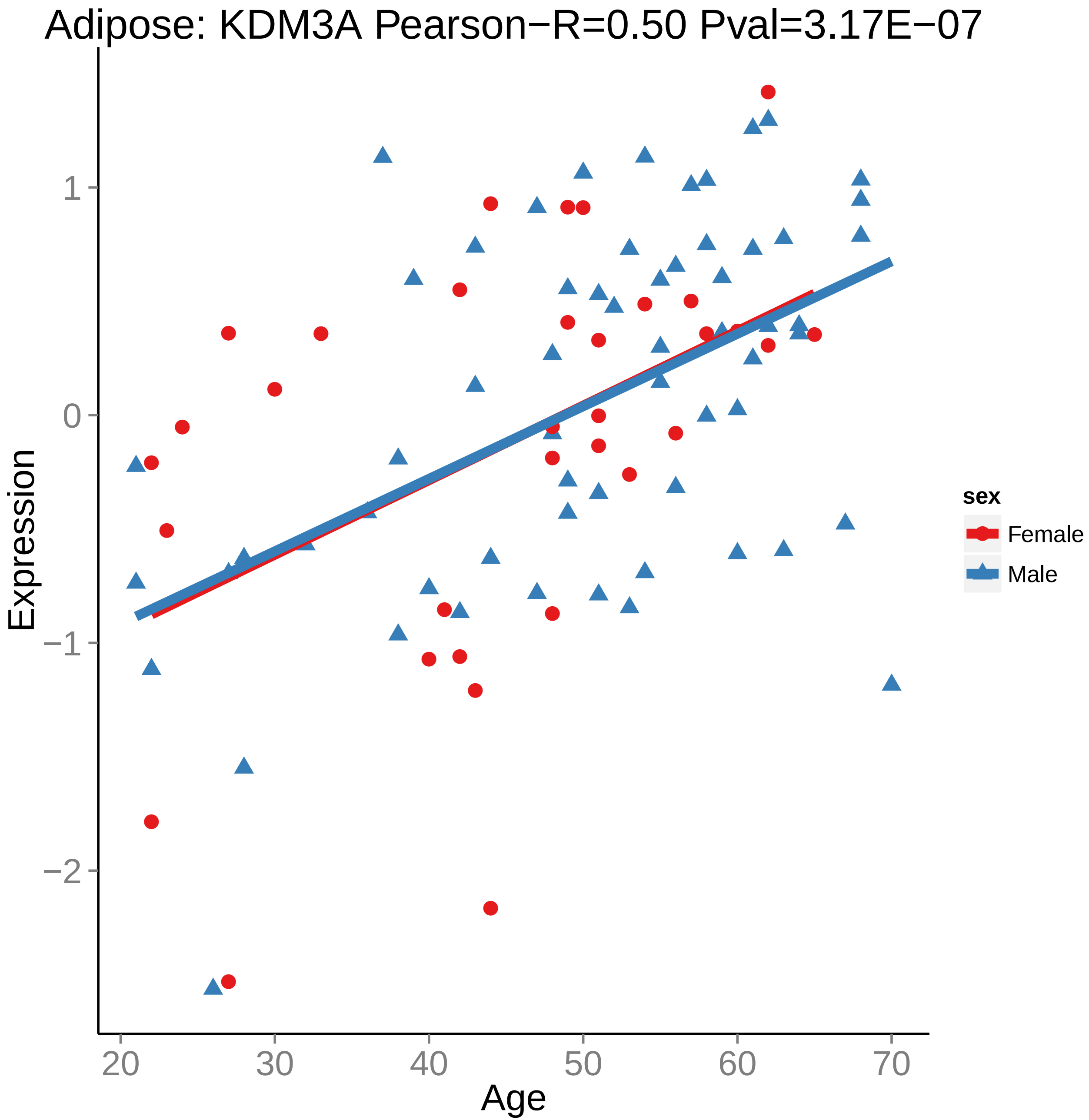

Adipose: KDM3A Pearson−R=0.50 Pval=3.17E−07
Expression
Age
1
0
−1
−2
20
30
40
50
60
70
sex
Female
Male

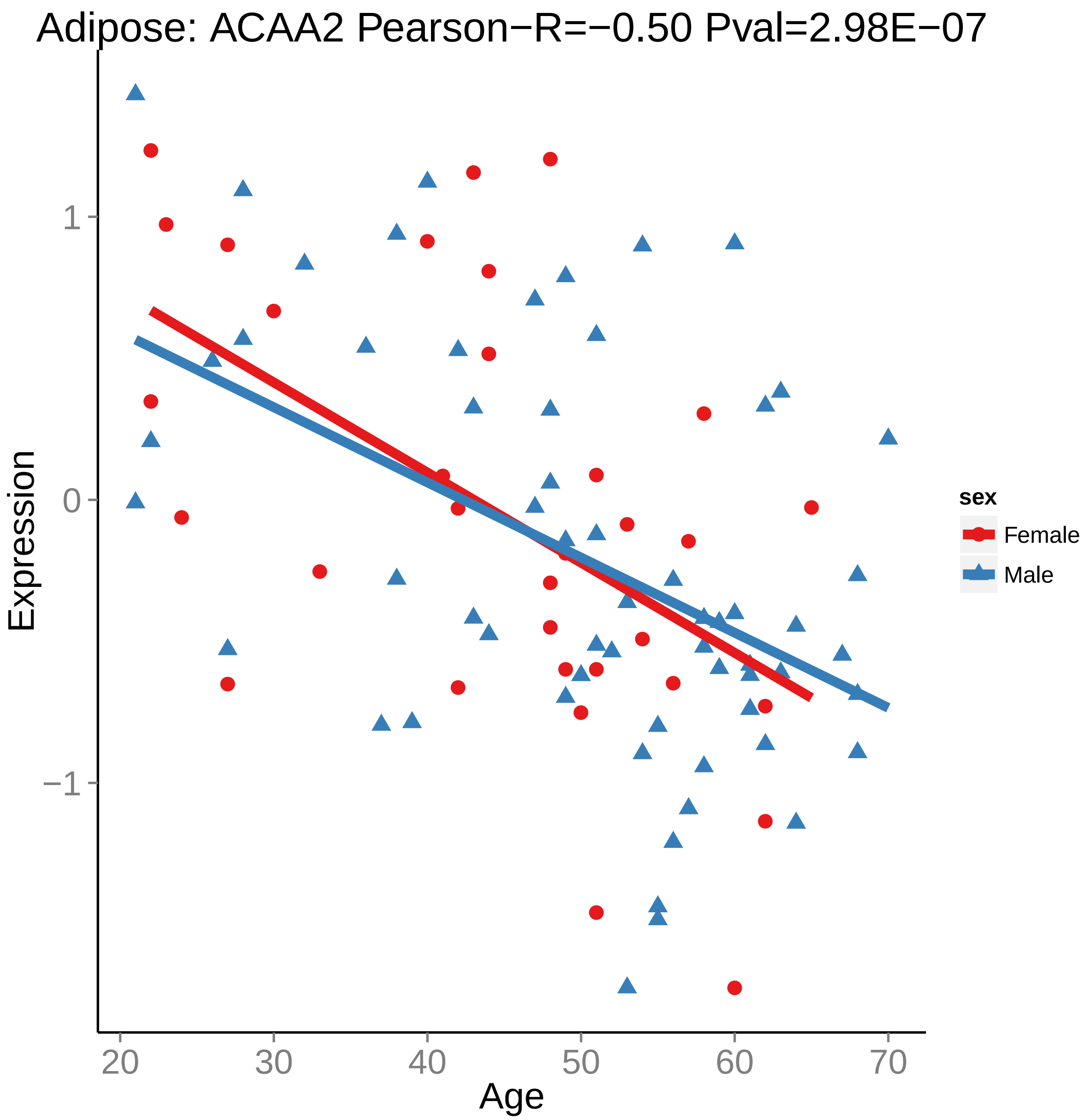

Adipose: ACAA2 Pearson−R=−0.50 Pval=2.98E−07
Expression
1
0
−1
20
30
40
50
60
70
Age
sex
Female
Male

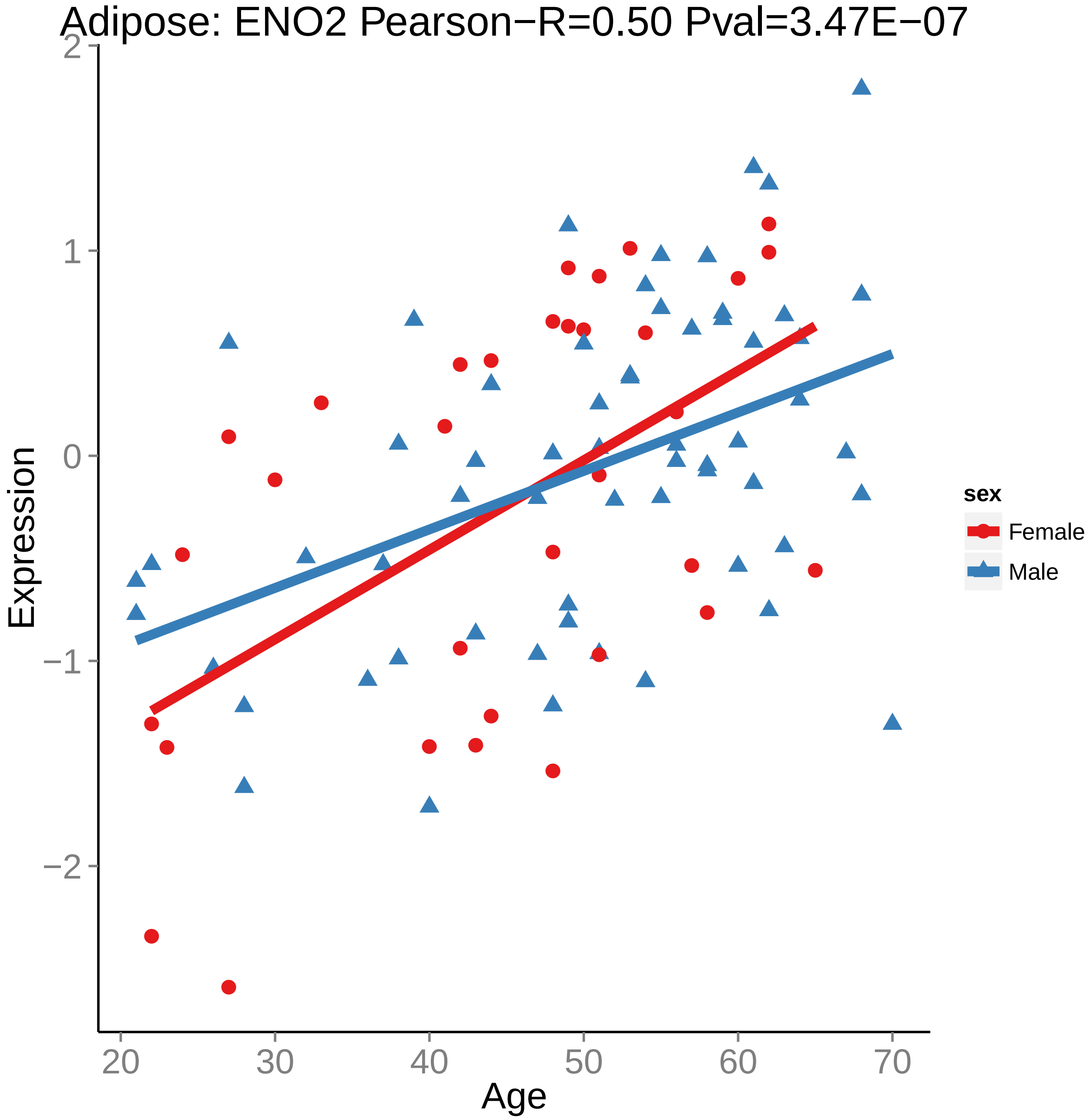

Adipose: ENO2 Pearson−R=0.50 Pval=3.47E−07
Expression
Age
2
1
0
−1
−2
20
30
40
50
60
70
sex
Female
Male

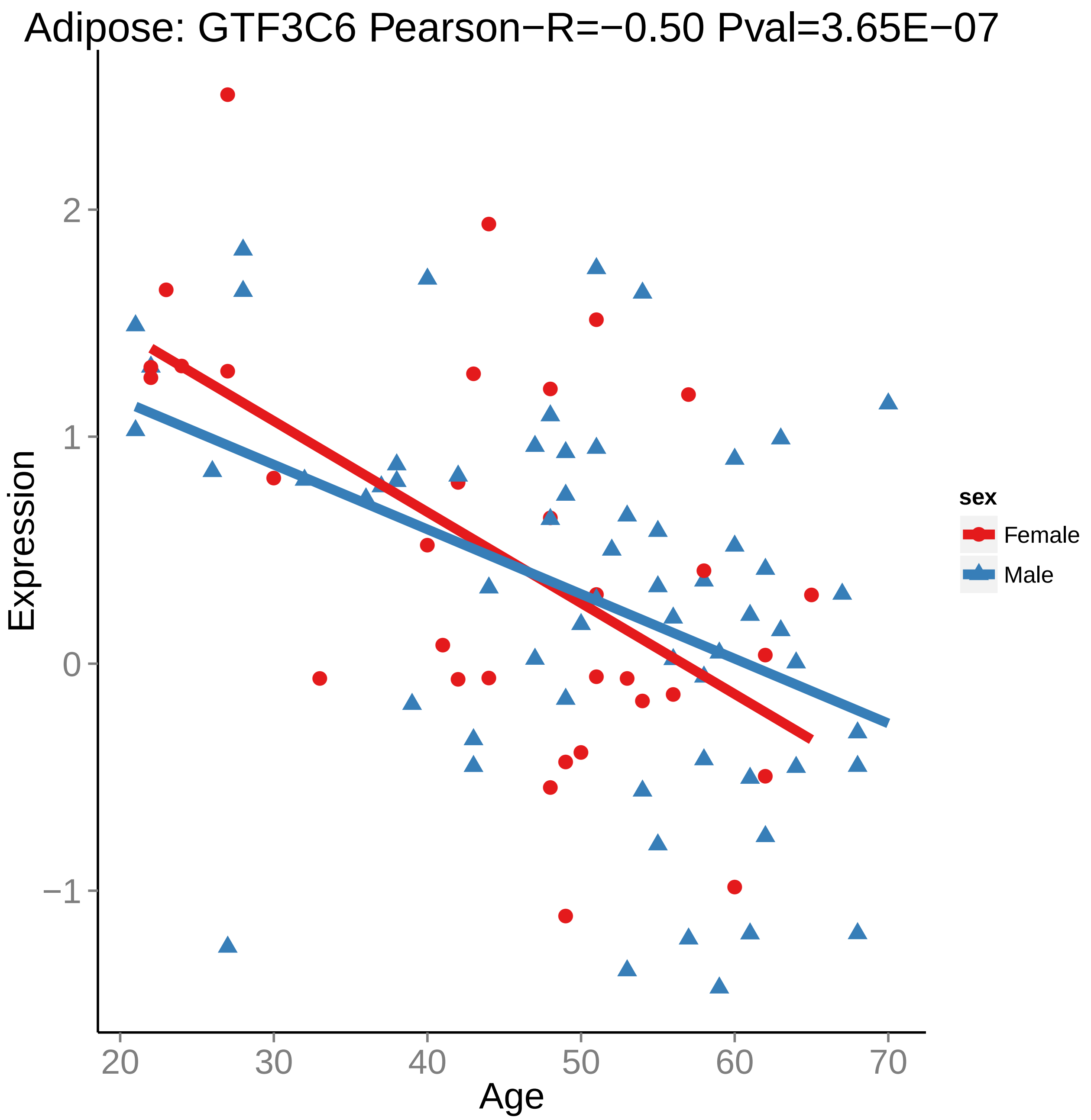
Adipose: GTF3C6 Pearson−R=−0.50 Pval=3.65E−07
Expression
2
1
0
−1
20
30
40
50
60
70
Age
sex
Female
Male

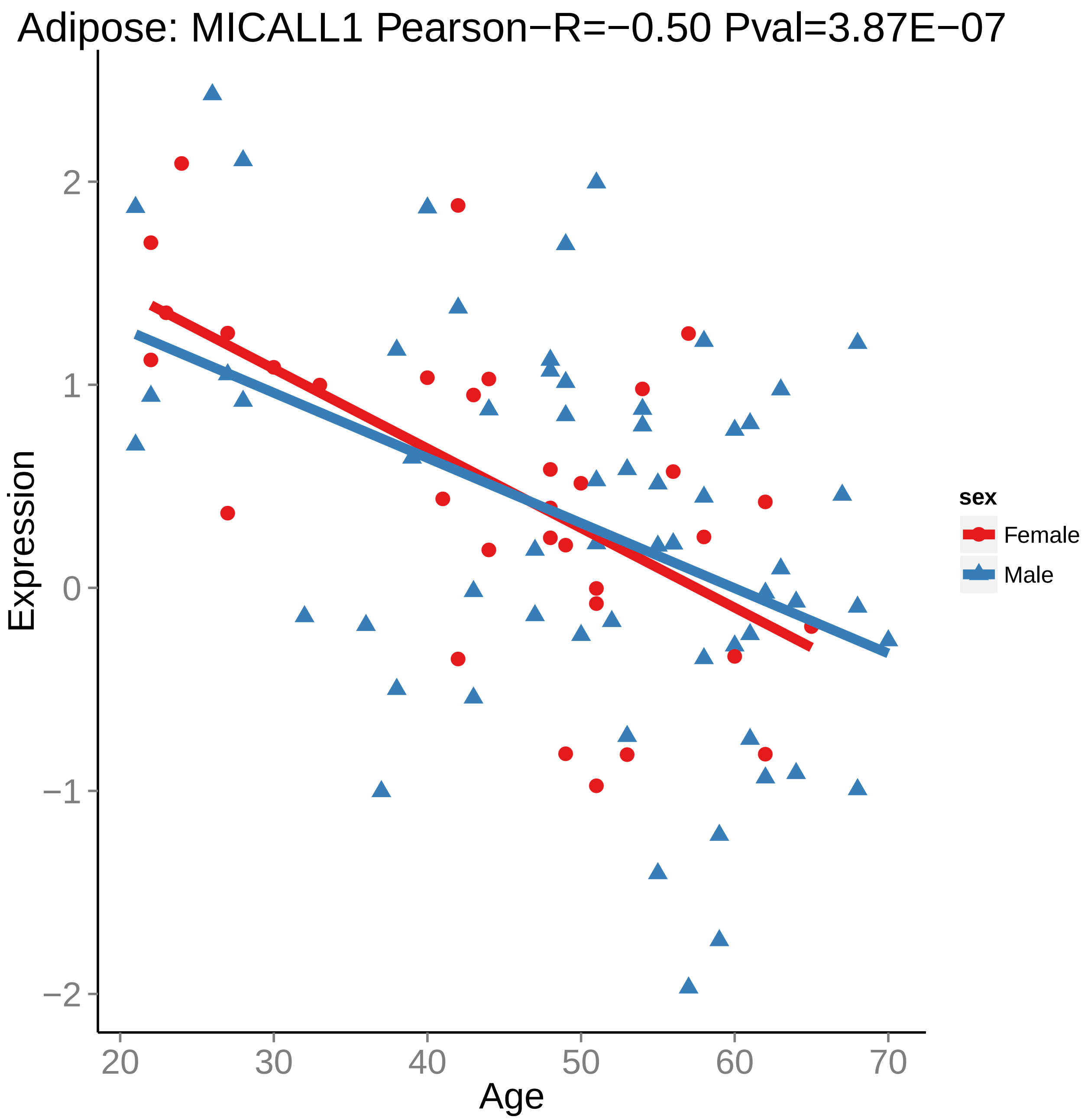

Adipose: MICALL1 Pearson−R=−0.50 Pval=3.87E−07
Expression
2
1
0
−1
−2
20
30
40
50
60
70
Age
sex
Female
Male

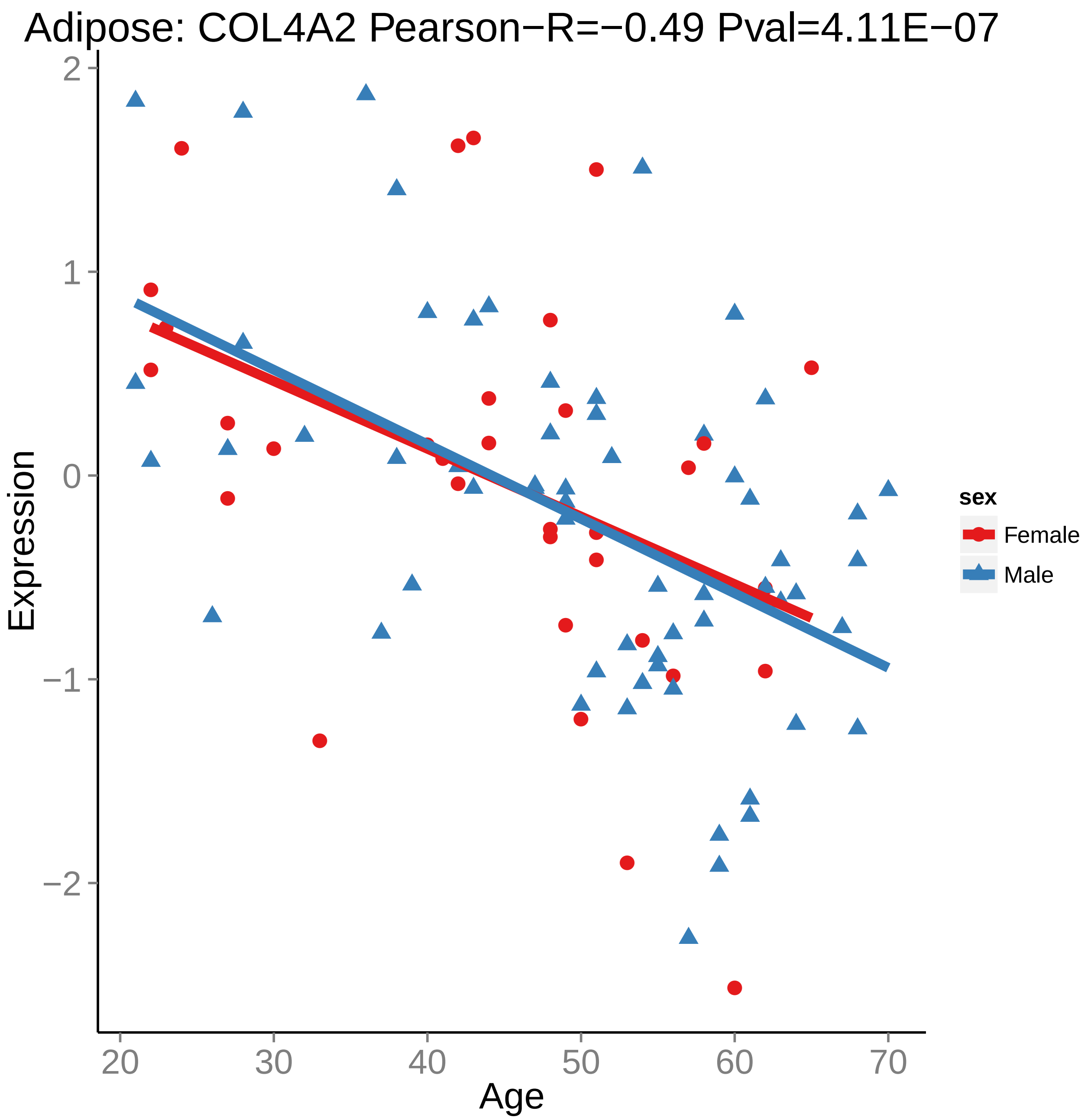

Adipose: COL4A2 Pearson−R=−0.49 Pval=4.11E−07
Expression
Age
2
1
0
−1
−2
20
30
40
50
60
70
sex
Female
Male

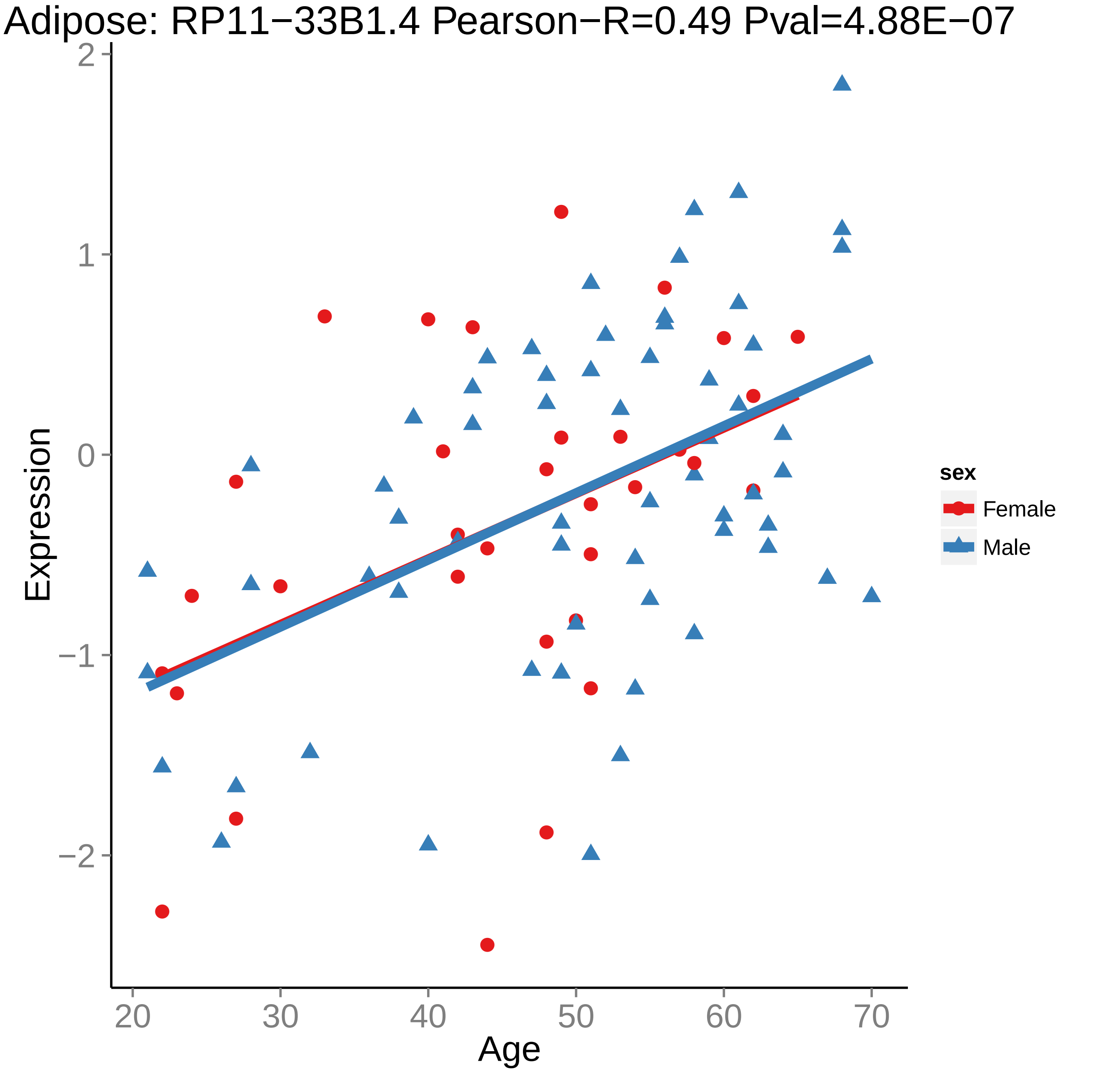
Adipose: RP11−33B1.4 Pearson−R=0.49 Pval=4.88E−07
Expression
Age
2
1
0
−1
−2
20
30
40
50
60
70
sex
Female
Male

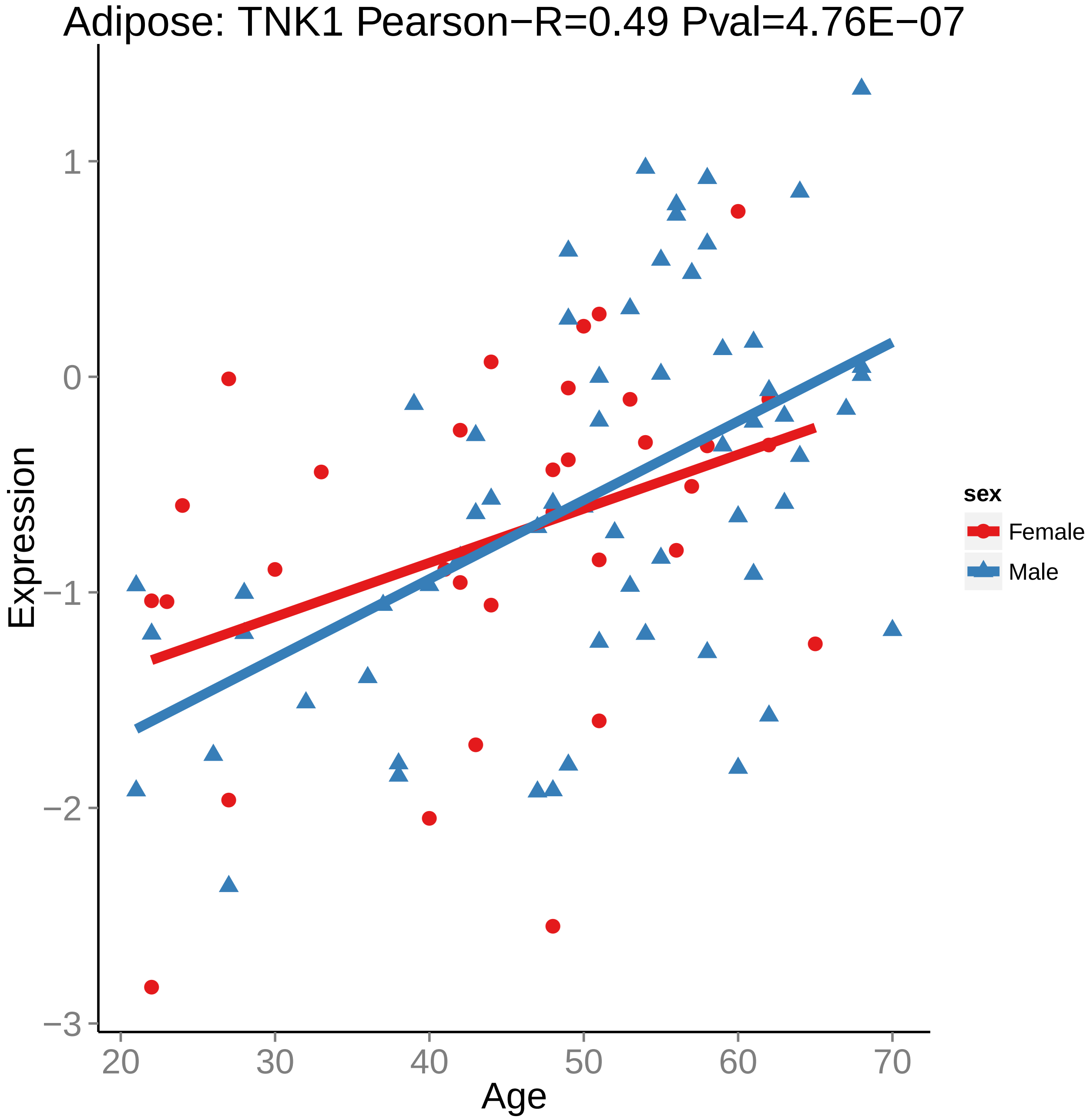
Adipose: TNK1 Pearson−R=0.49 Pval=4.76E−07
Expression
1
0
−1
−2
−3
20
30
40
50
60
70
Age
sex
Female
Male

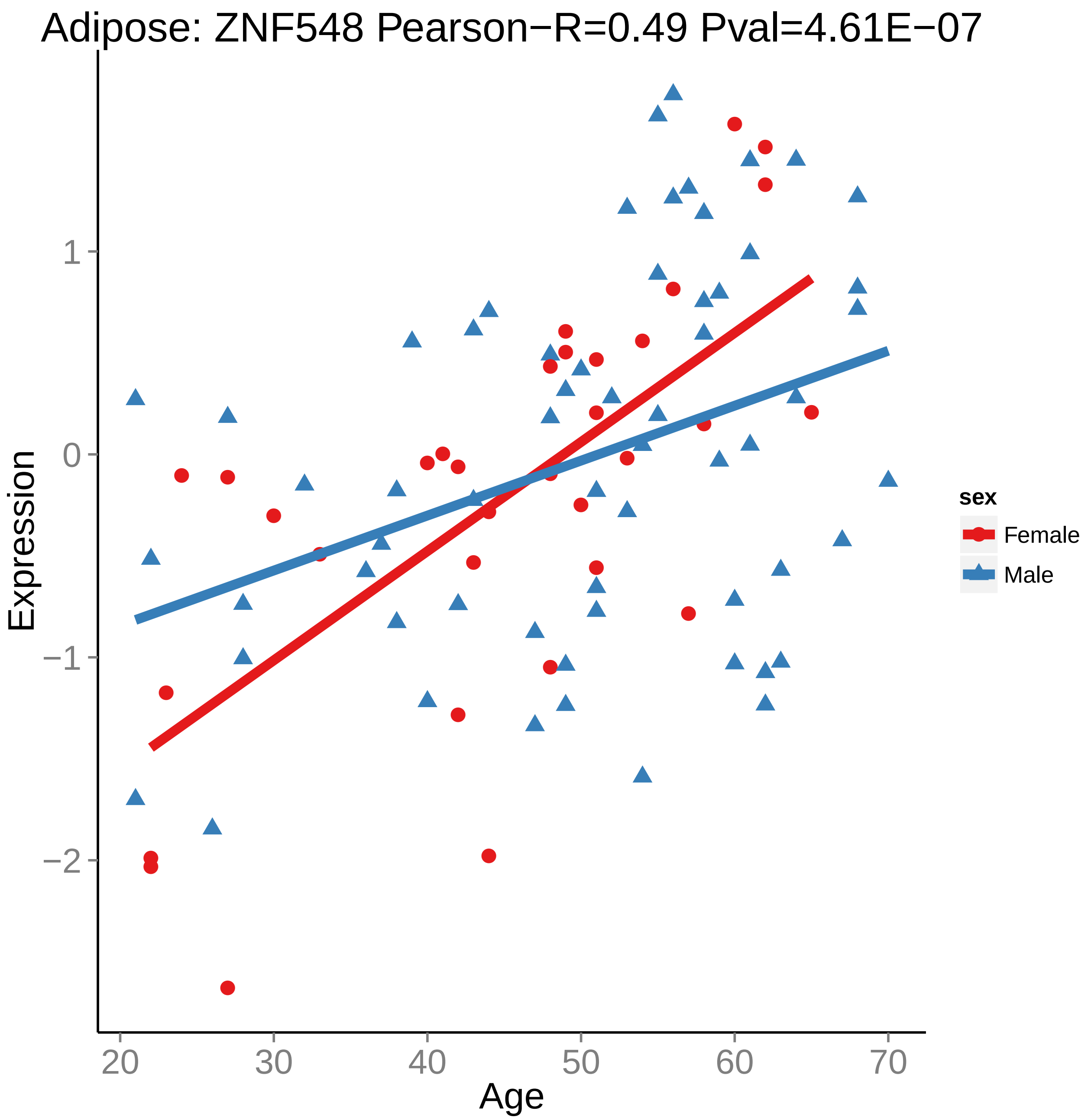
Adipose: ZNF548 Pearson−R=0.49 Pval=4.61E−07
Expression
1
0
−1
−2
20
30
40
50
60
70
Age
sex
Female
Male

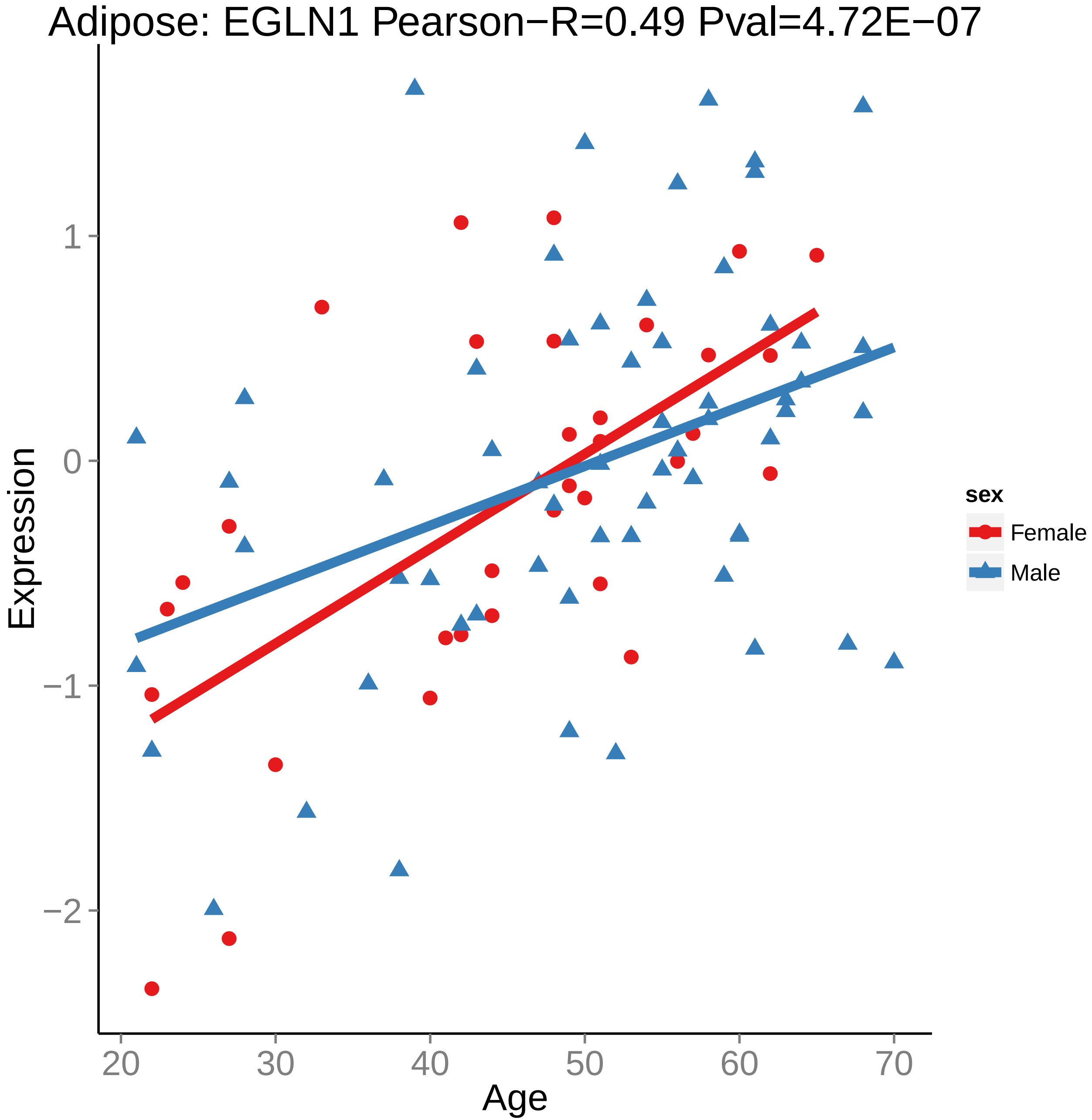

Adipose: EGLN1 Pearson−R=0.49 Pval=4.72E−07
Expression
1
0
−1
−2
20
30
40
50
60
70
Age
sex
Female
Male

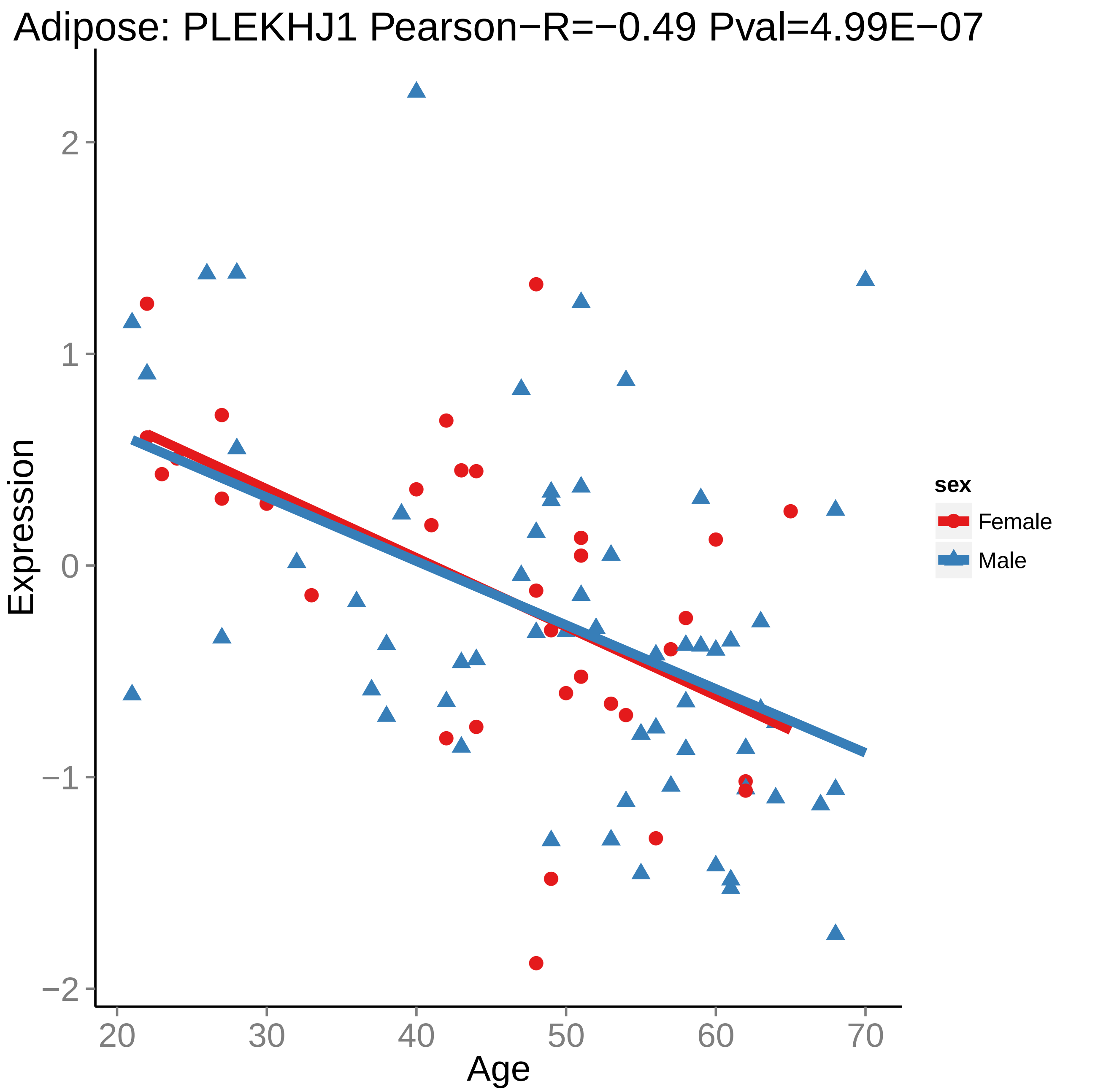
Adipose: PLEKHJ1 Pearson−R=−0.49 Pval=4.99E−07
Expression
Age
2
1
0
−1
−2
20
30
40
50
60
70
sex
Female
Male

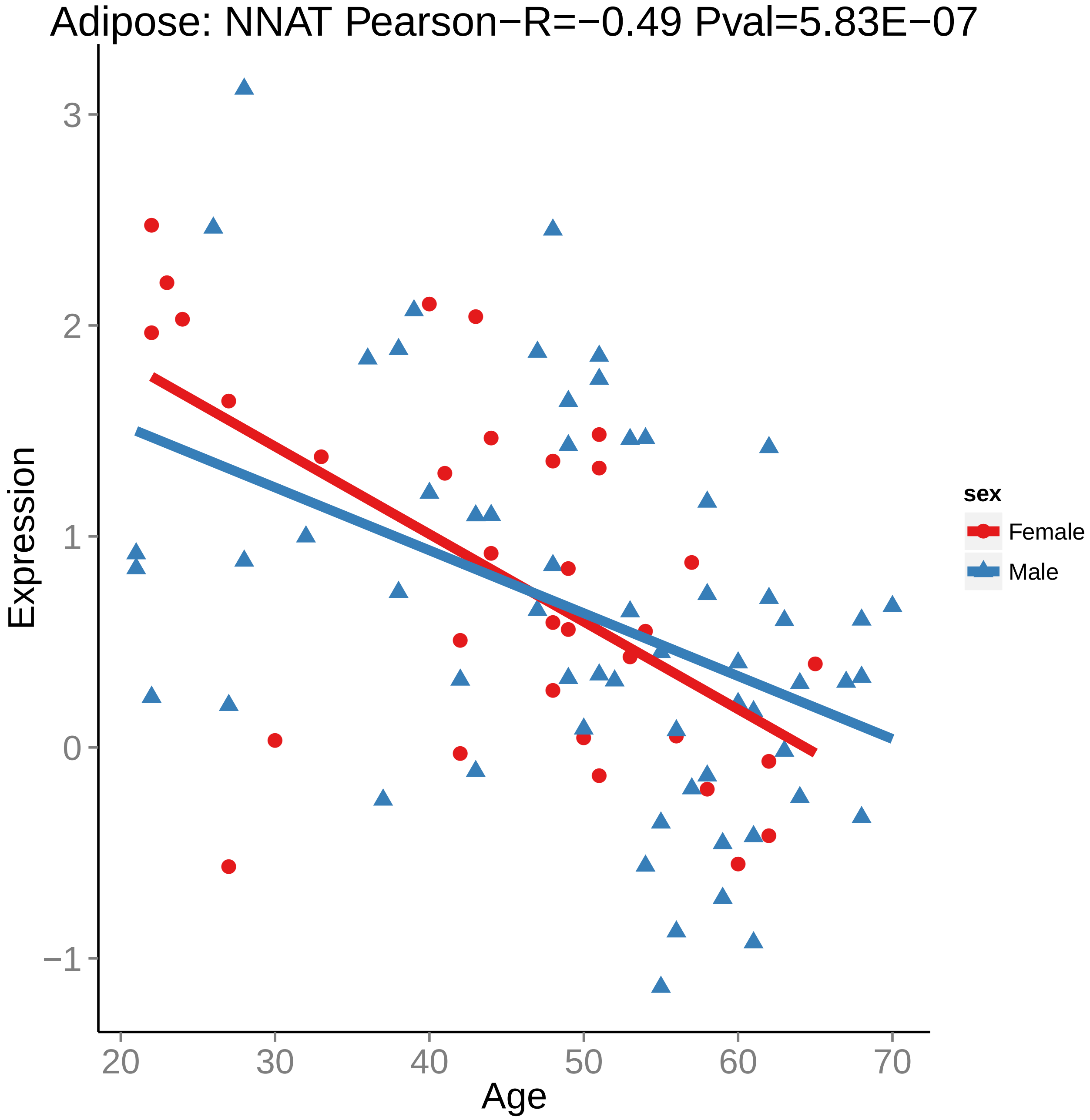

Adipose: NNAT Pearson−R=−0.49 Pval=5.83E−07
Expression
3
2
1
0
−1
20
30
40
50
60
70
Age
sex
Female
Male

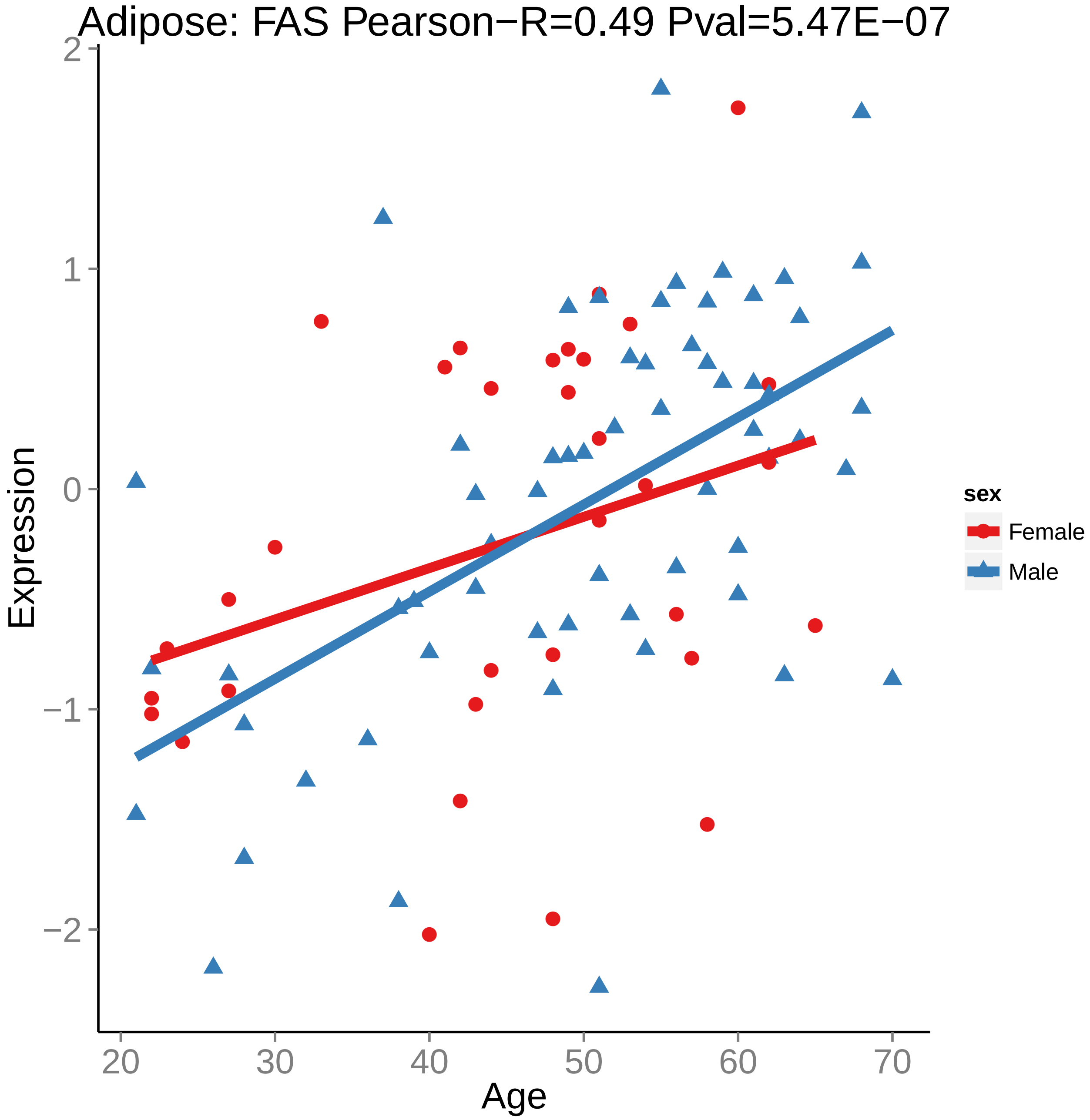
Adipose: FAS Pearson−R=0.49 Pval=5.47E−07
Expression
Age
2
1
0
−1
−2
20
30
40
50
60
70
sex
Female
Male

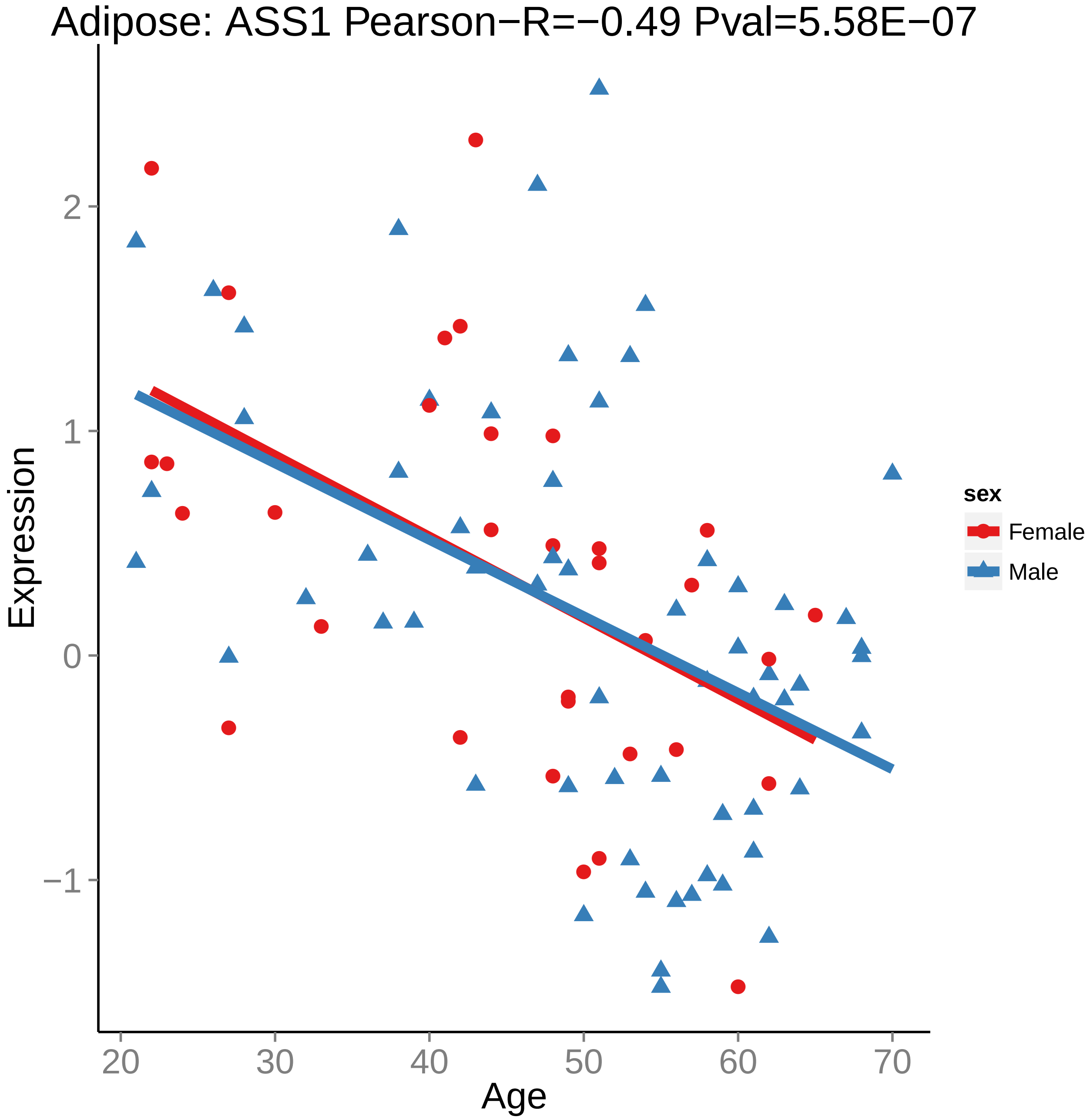

Adipose: ASS1 Pearson−R=−0.49 Pval=5.58E−07
Expression
2
1
0
−1
20
30
40
50
60
70
Age
sex
Female
Male

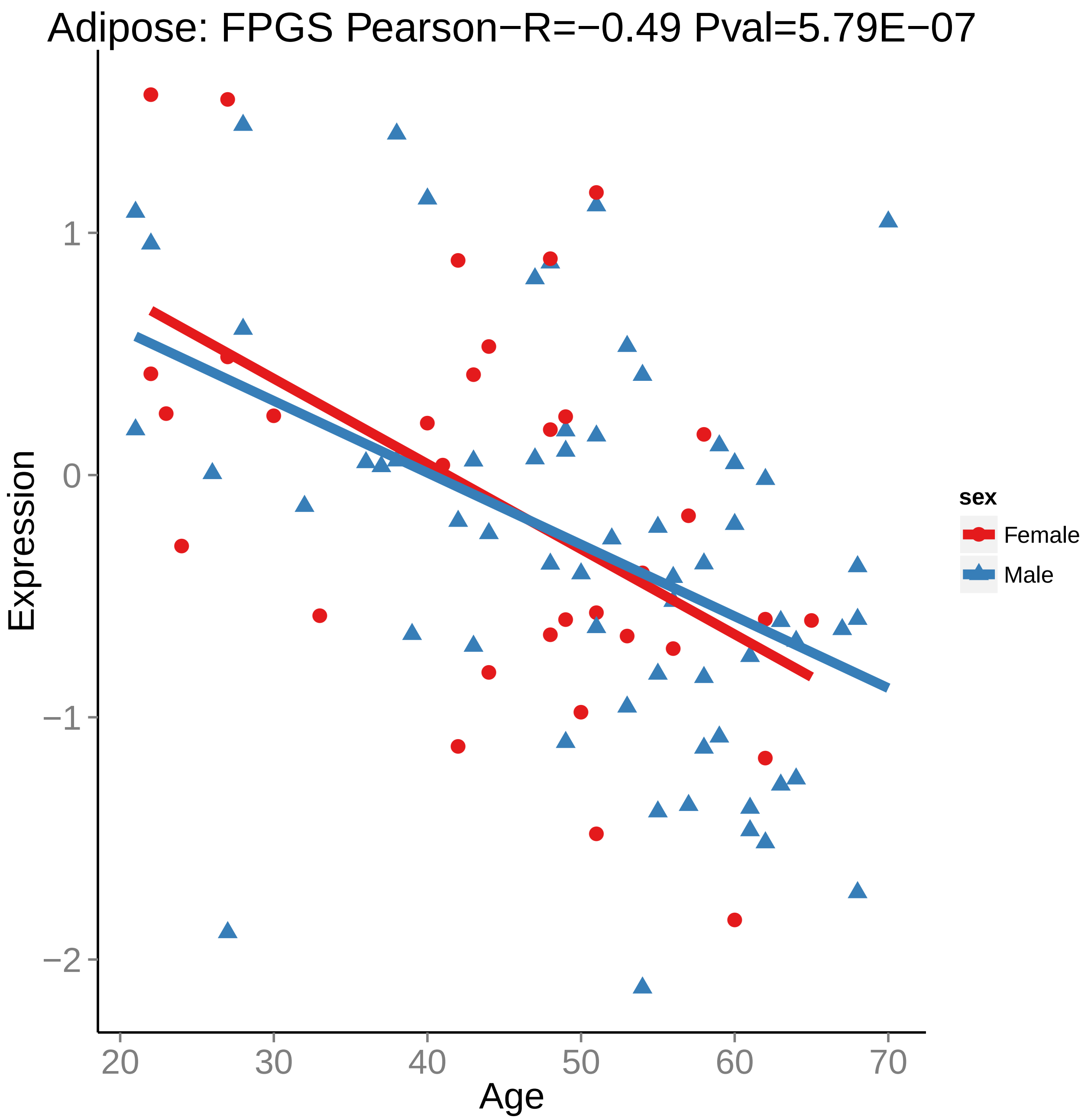

Adipose: FPGS Pearson−R=−0.49 Pval=5.79E−07
Expression
1
0
−1
−2
20
30
40
50
60
70
Age
sex
Female
Male

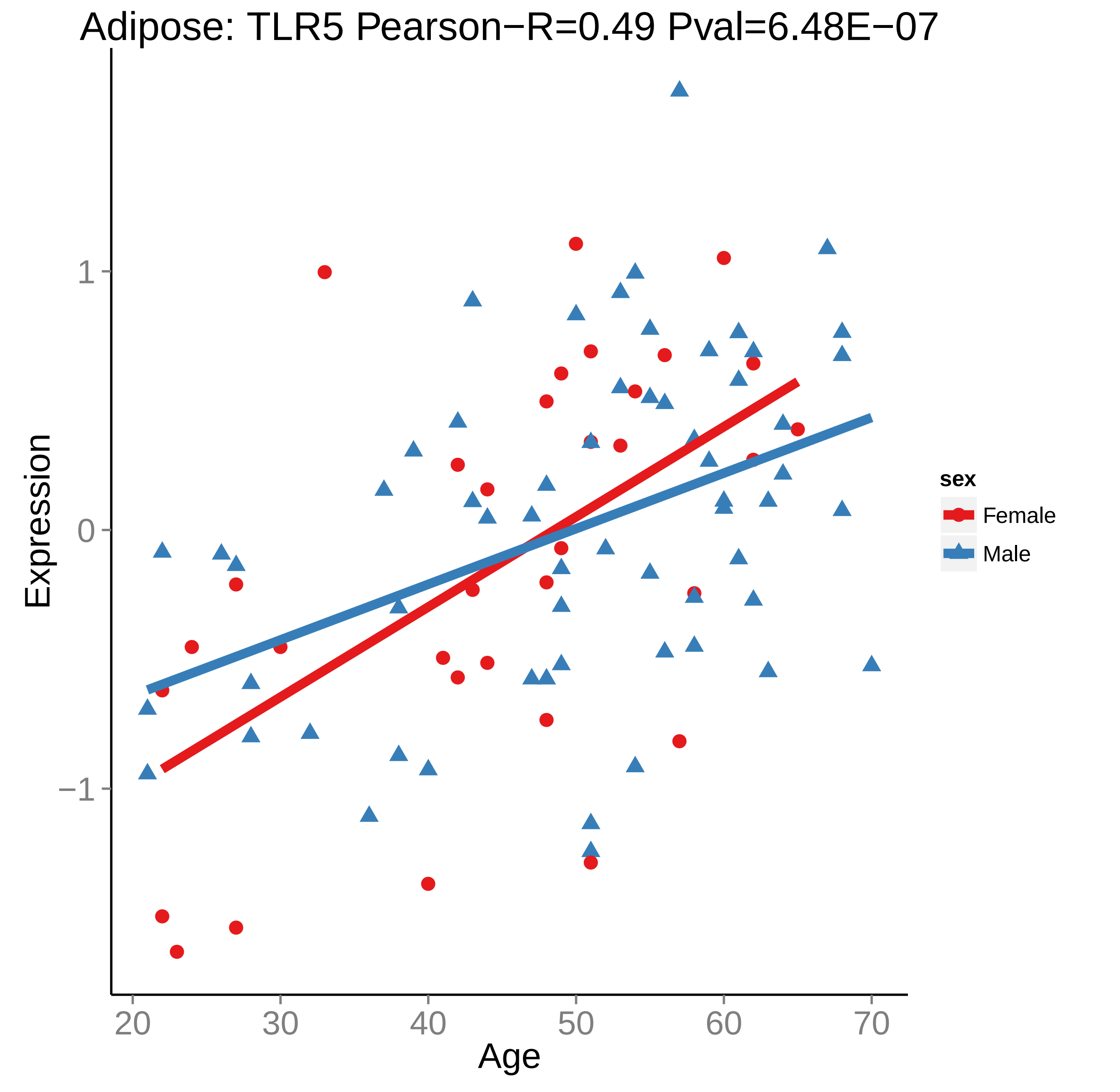

Adipose: TLR5 Pearson−R=0.49 Pval=6.48E−07
Expression
1
0
−1
20
30
40
50
60
70
Age
sex
Female
Male

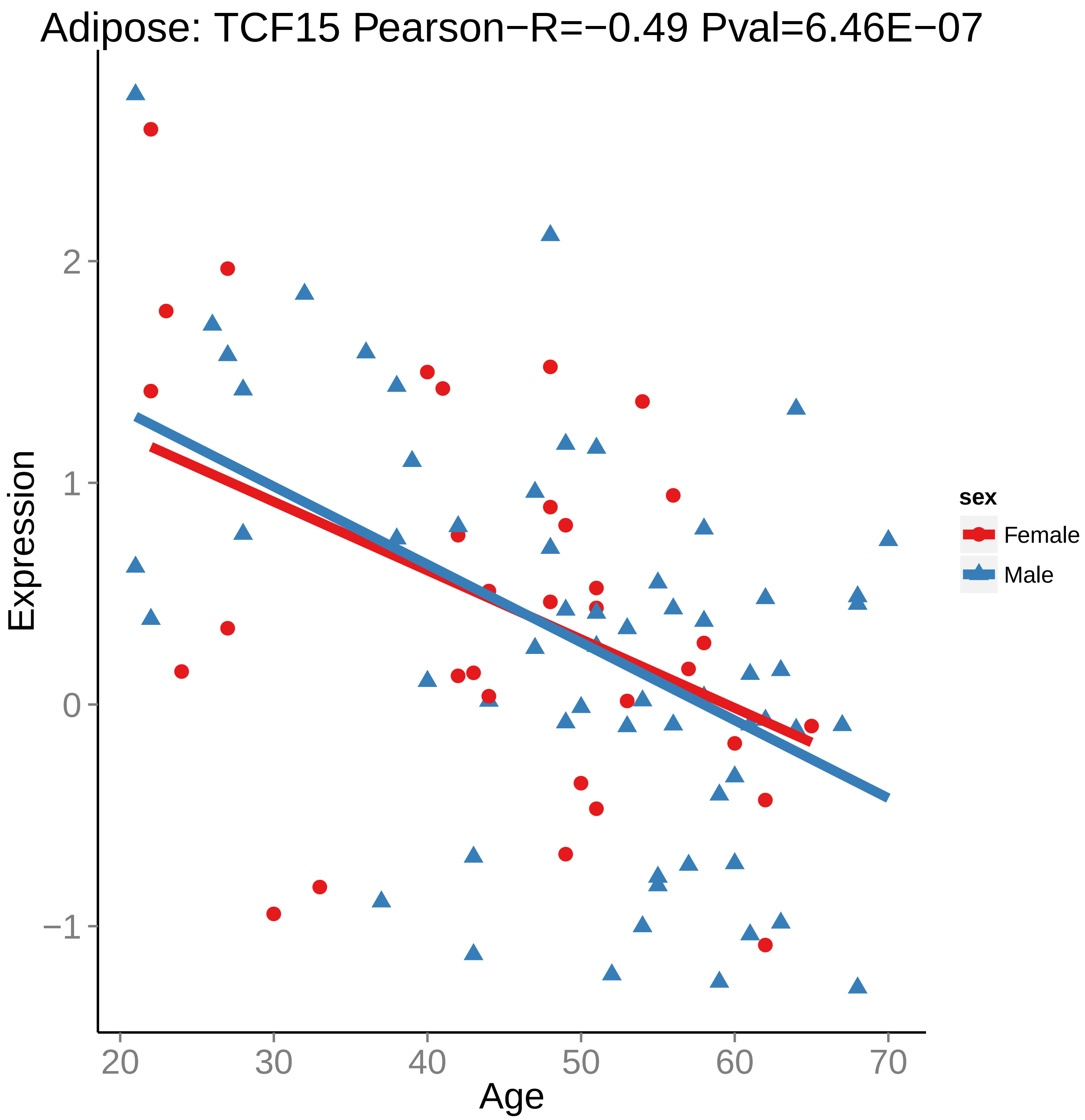

Adipose: TCF15 Pearson−R=−0.49 Pval=6.46E−07
Expression
Age
2
1
0
−1
20
30
40
50
60
70
sex
Female
Male

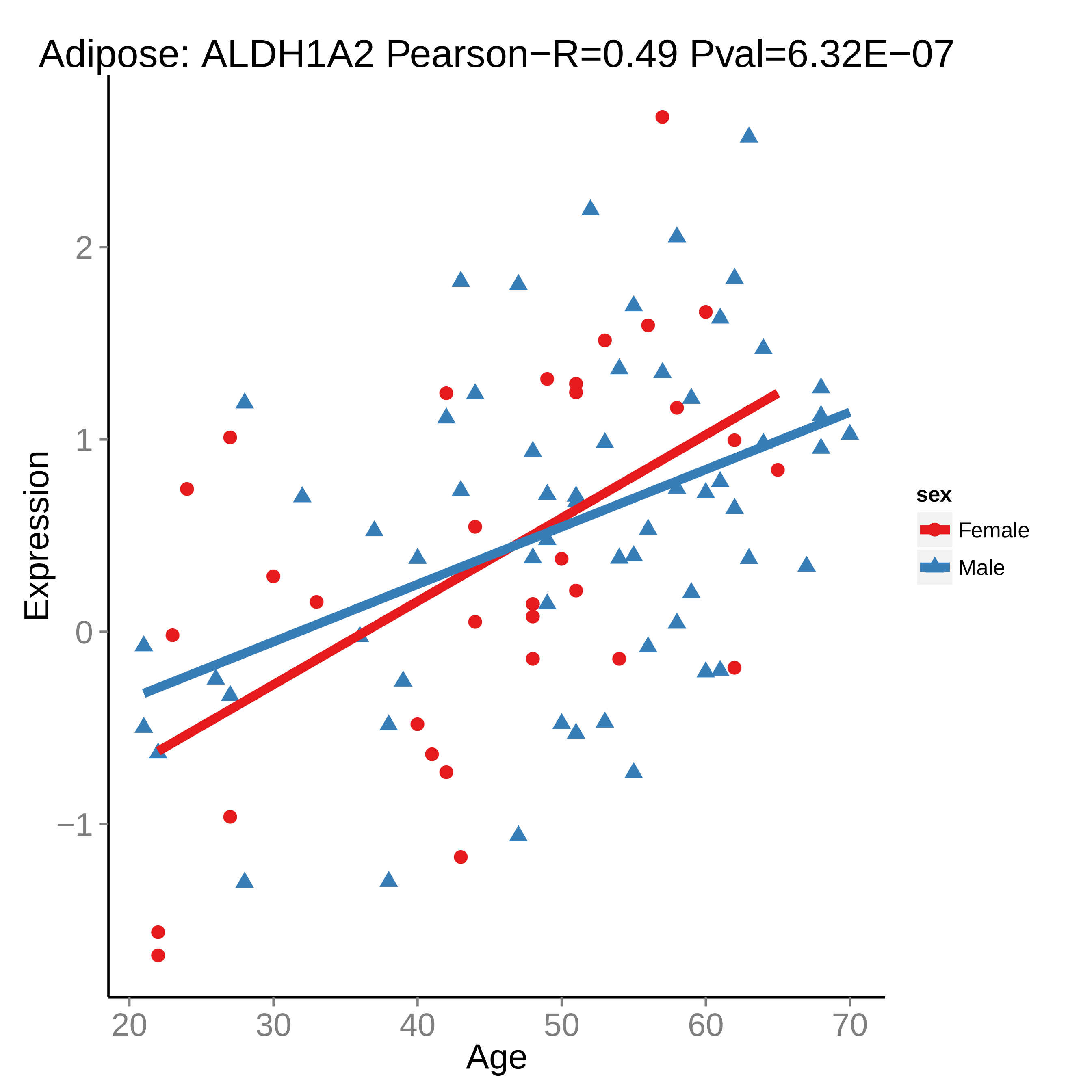

Adipose: ALDH1A2 Pearson−R=0.49 Pval=6.32E−07
Expression
2
1
0
−1
20
30
40
50
60
70
Age
sex
Female
Male

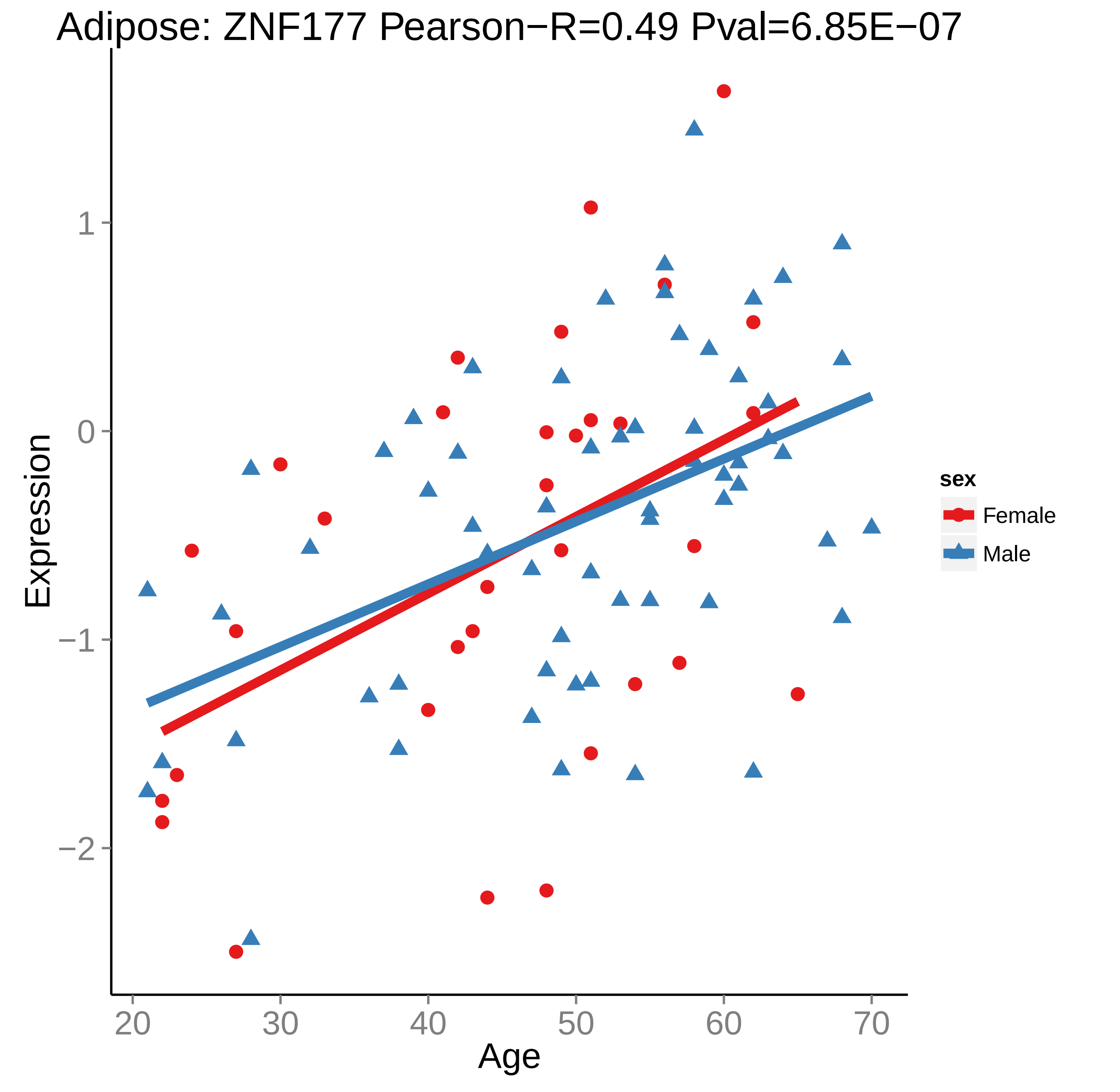
Adipose: ZNF177 Pearson−R=0.49 Pval=6.85E−07
Expression
1
0
−1
−2
20
30
40
50
60
70
Age
sex
Female
Male

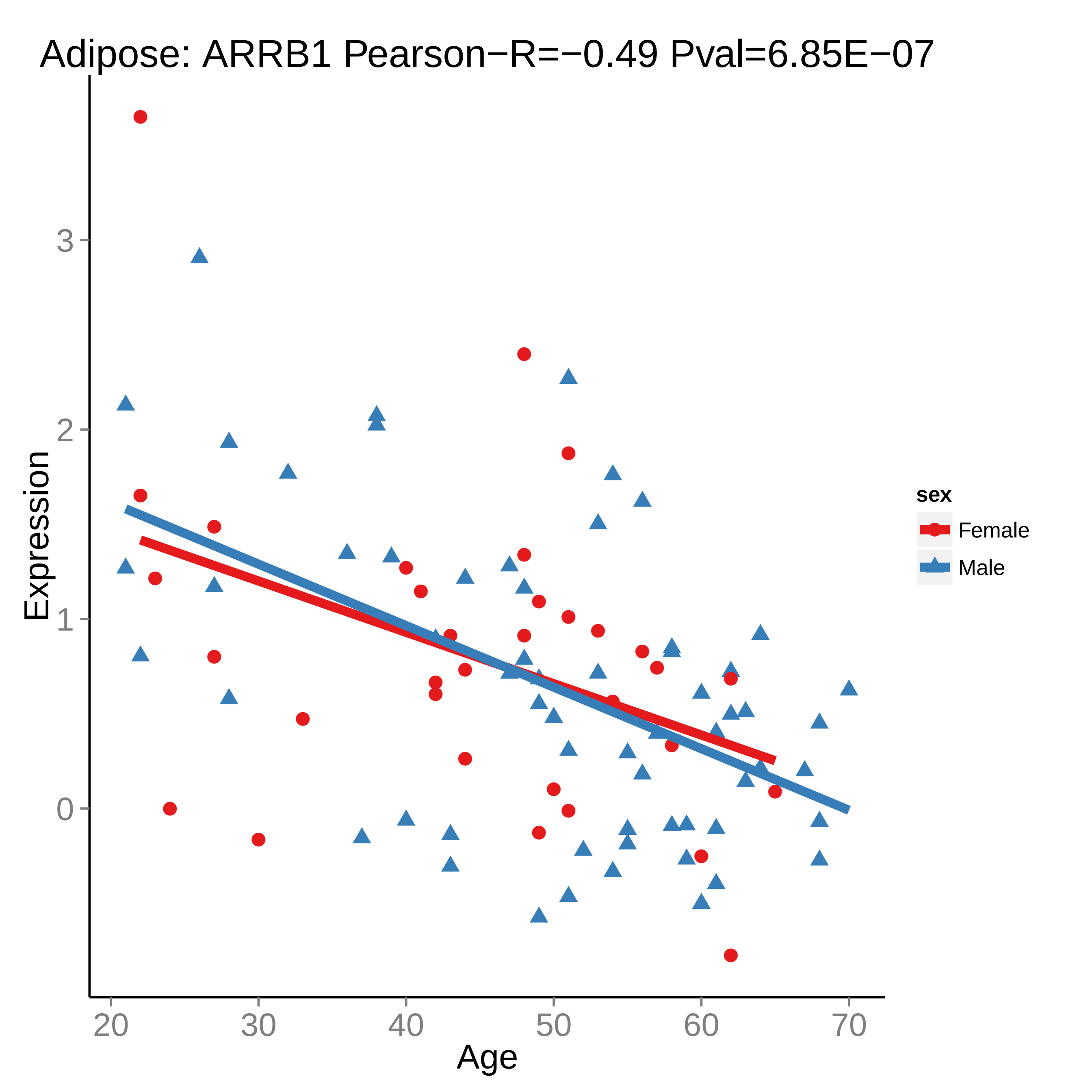

Adipose: ARRB1 Pearson−R=−0.49 Pval=6.85E−07
Expression
Age
0
1
2
3
20
30
40
50
60
70
sex
Female
Male

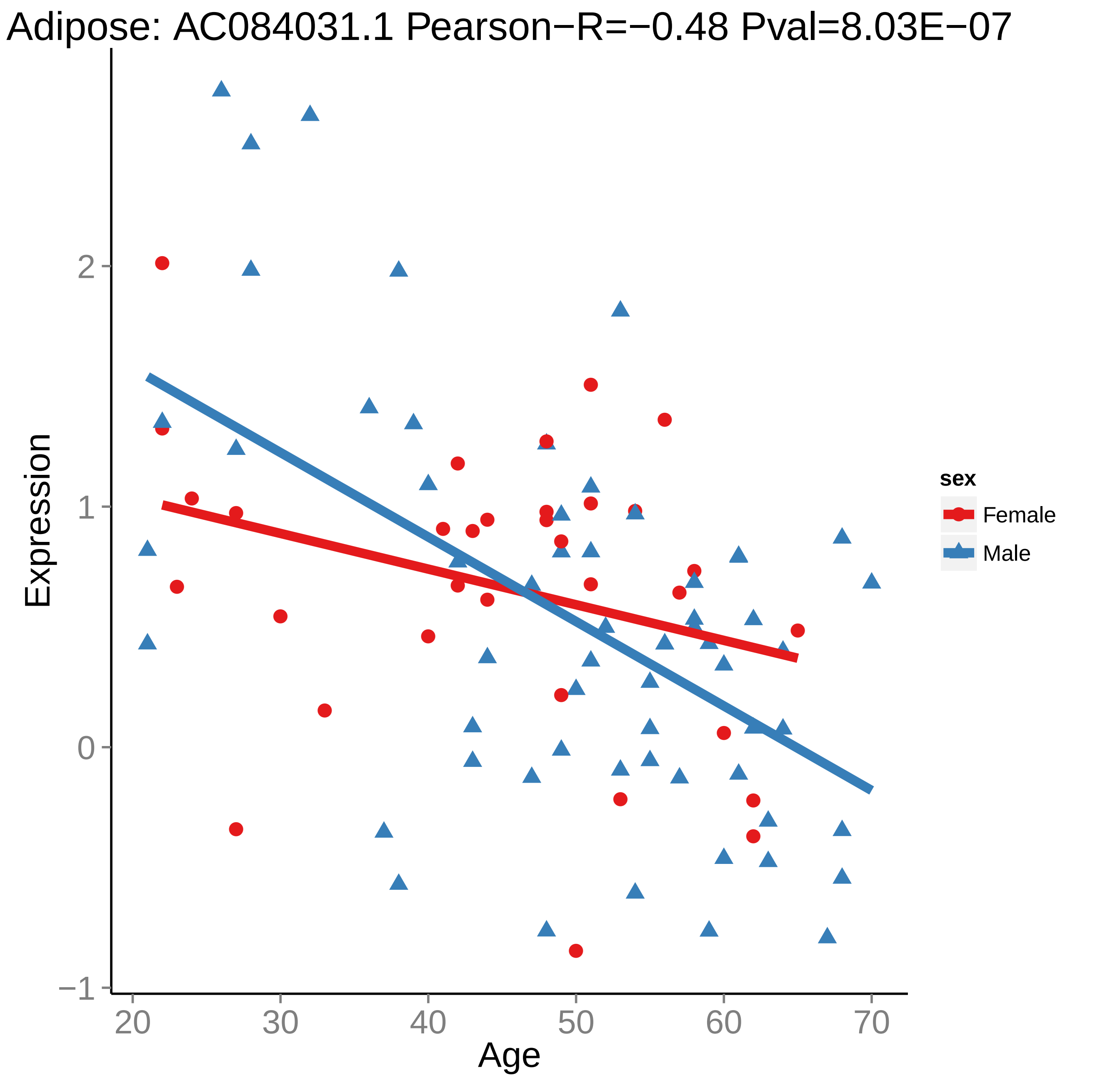

Adipose: AC084031.1 Pearson−R=−0.48 Pval=8.03E−07
Expression
Age
2
1
0
−1
20
30
40
50
60
70
sex
Female
Male

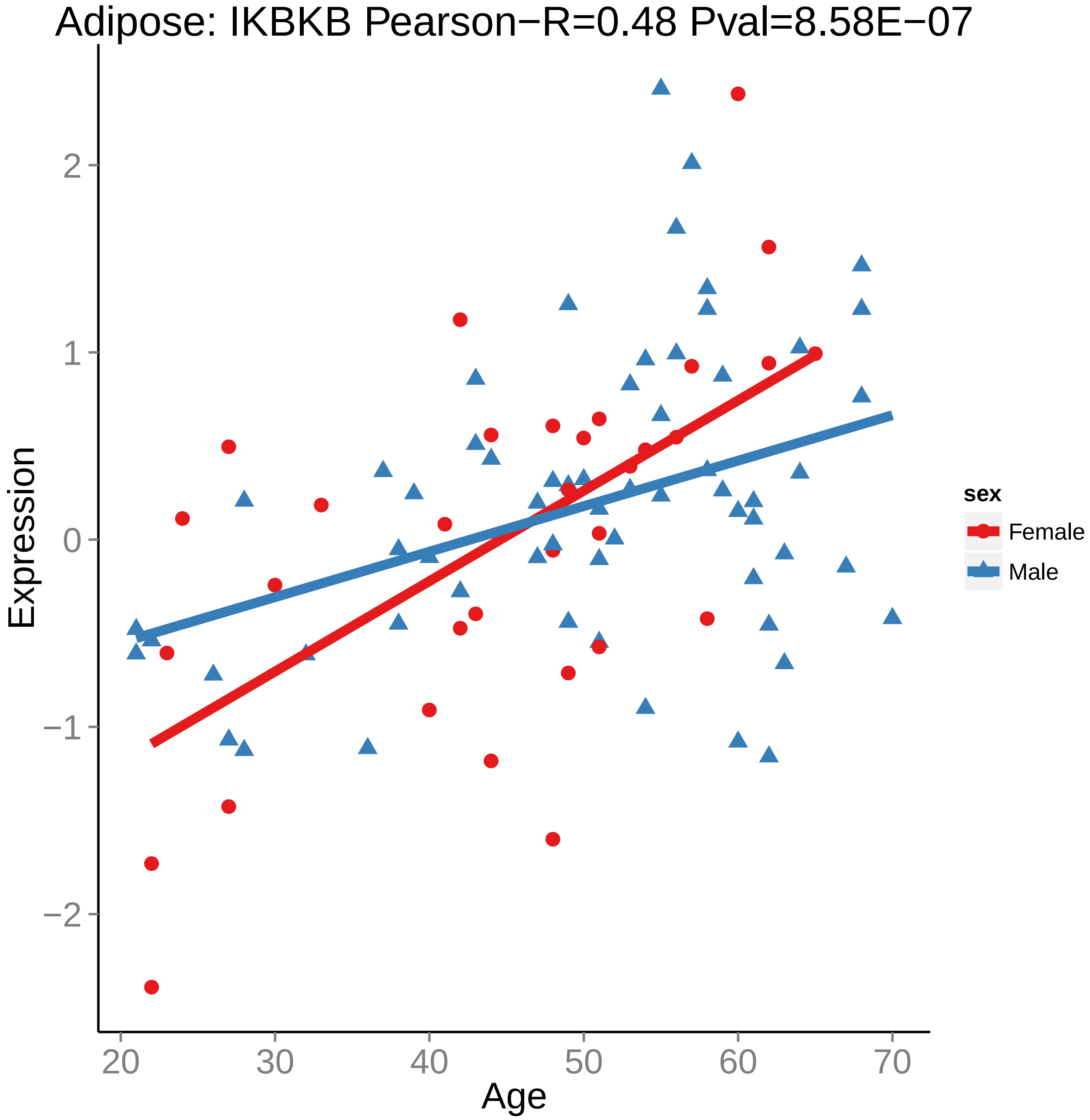

Adipose: IKBKB Pearson−R=0.48 Pval=8.58E−07
Expression
Age
sex
Female
Male
2
1
0
−1
−2
20
30
40
50
60
70

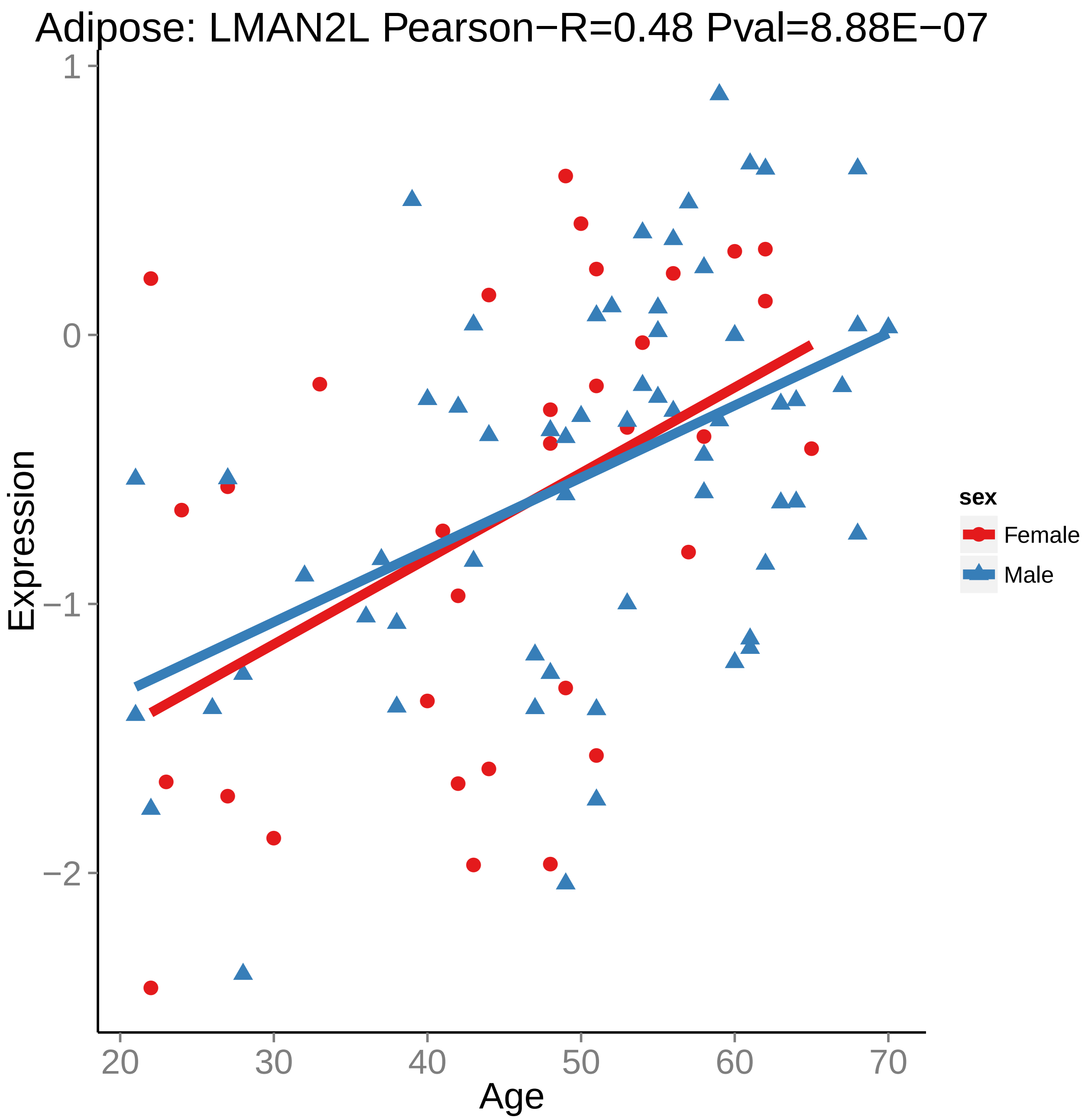

Adipose: LMAN2L Pearson−R=0.48 Pval=8.88E−07
Expression
Age
1
0
−1
−2
20
30
40
50
60
70
sex
Female
Male

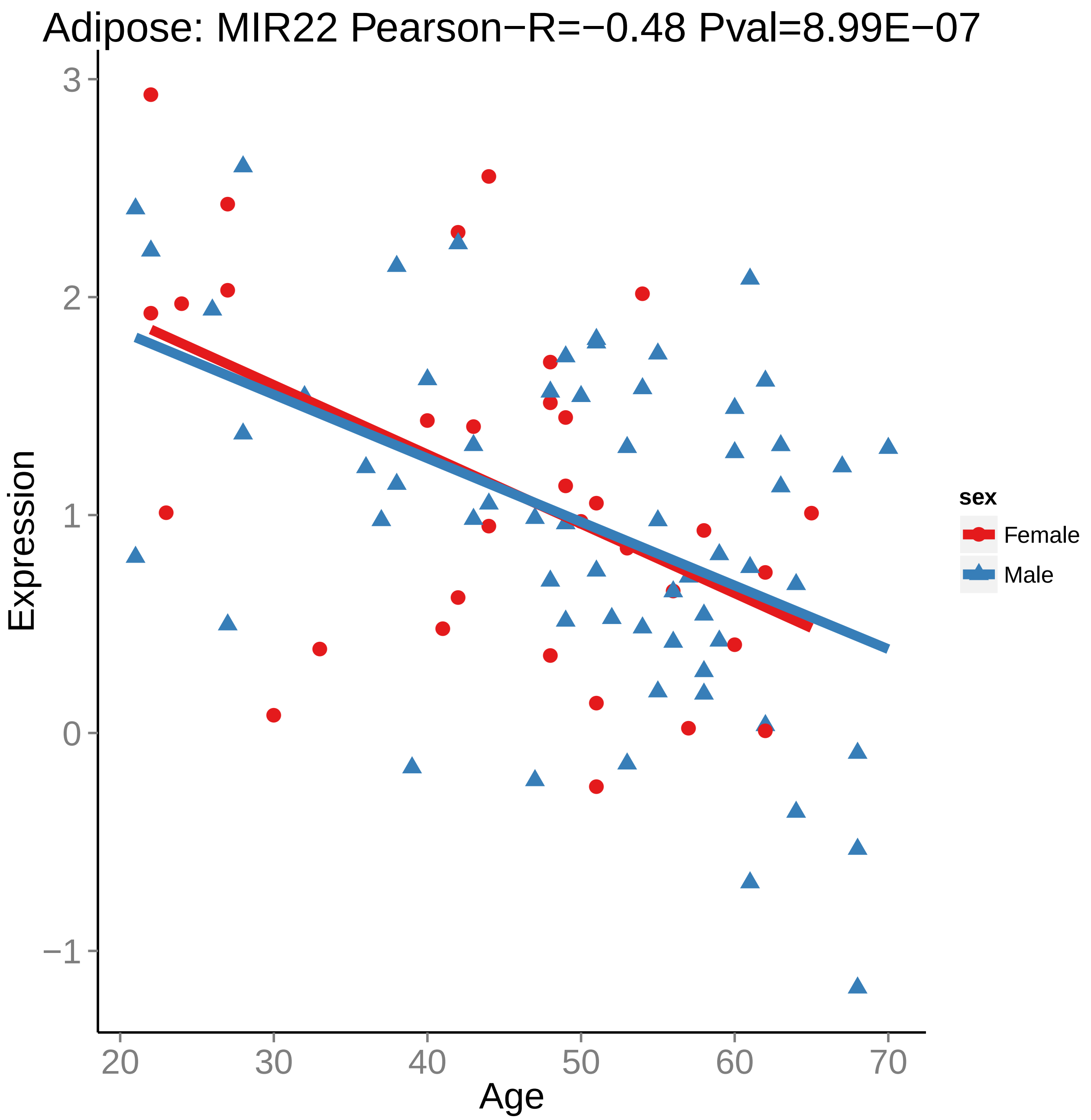

Adipose: MIR22 Pearson−R=−0.48 Pval=8.99E−07
Expression
Age
3
2
1
0
−1
20
30
40
50
60
70
sex
Female
Male

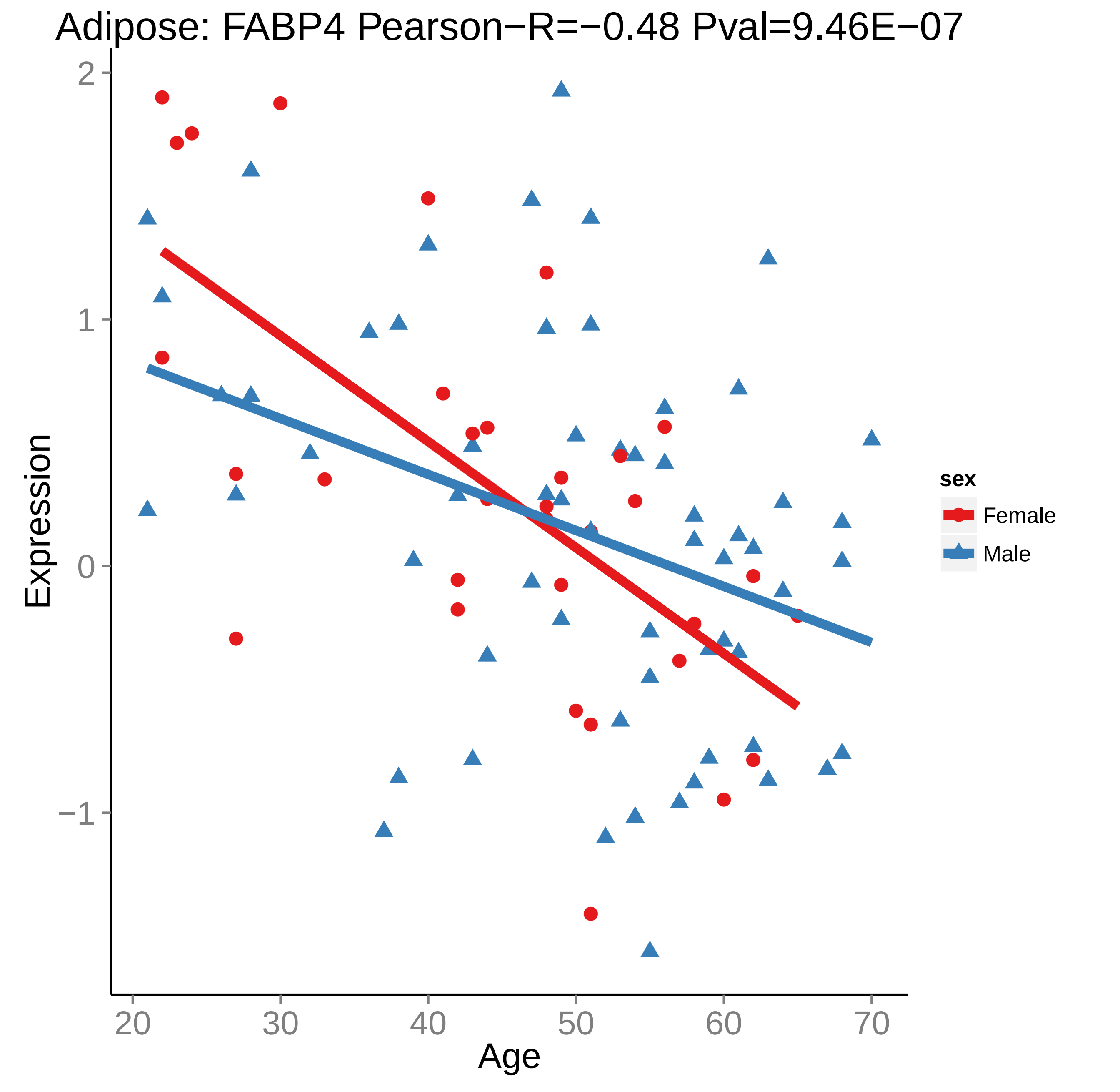

Adipose: FABP4 Pearson−R=−0.48 Pval=9.46E−07
Expression
Age
2
1
0
−1
20
30
40
50
60
70
sex
Female
Male

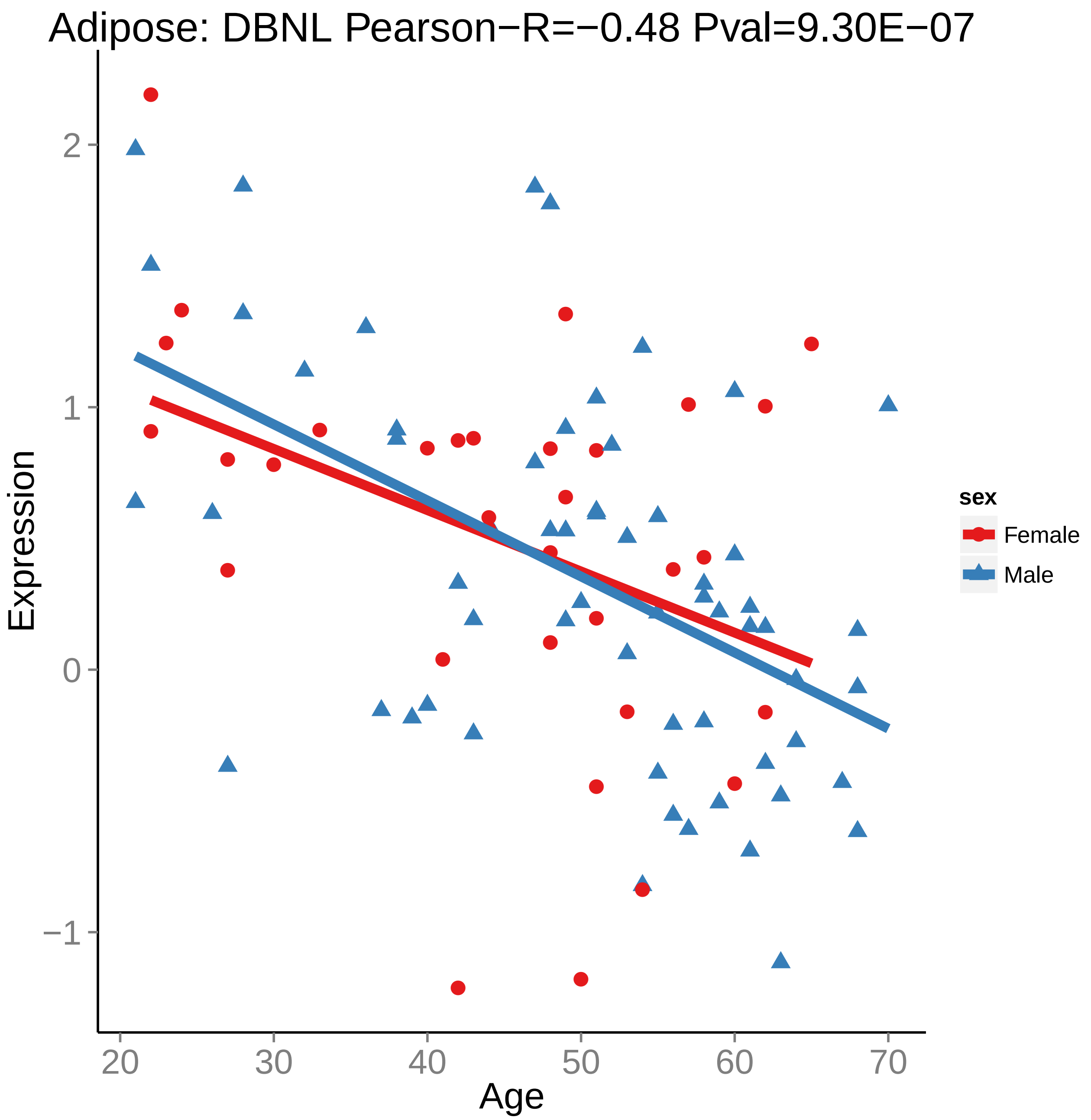

Adipose: DBNL Pearson−R=−0.48 Pval=9.30E−07
Expression
Age
2
1
0
−1
20
30
40
50
60
70
sex
Female
Male

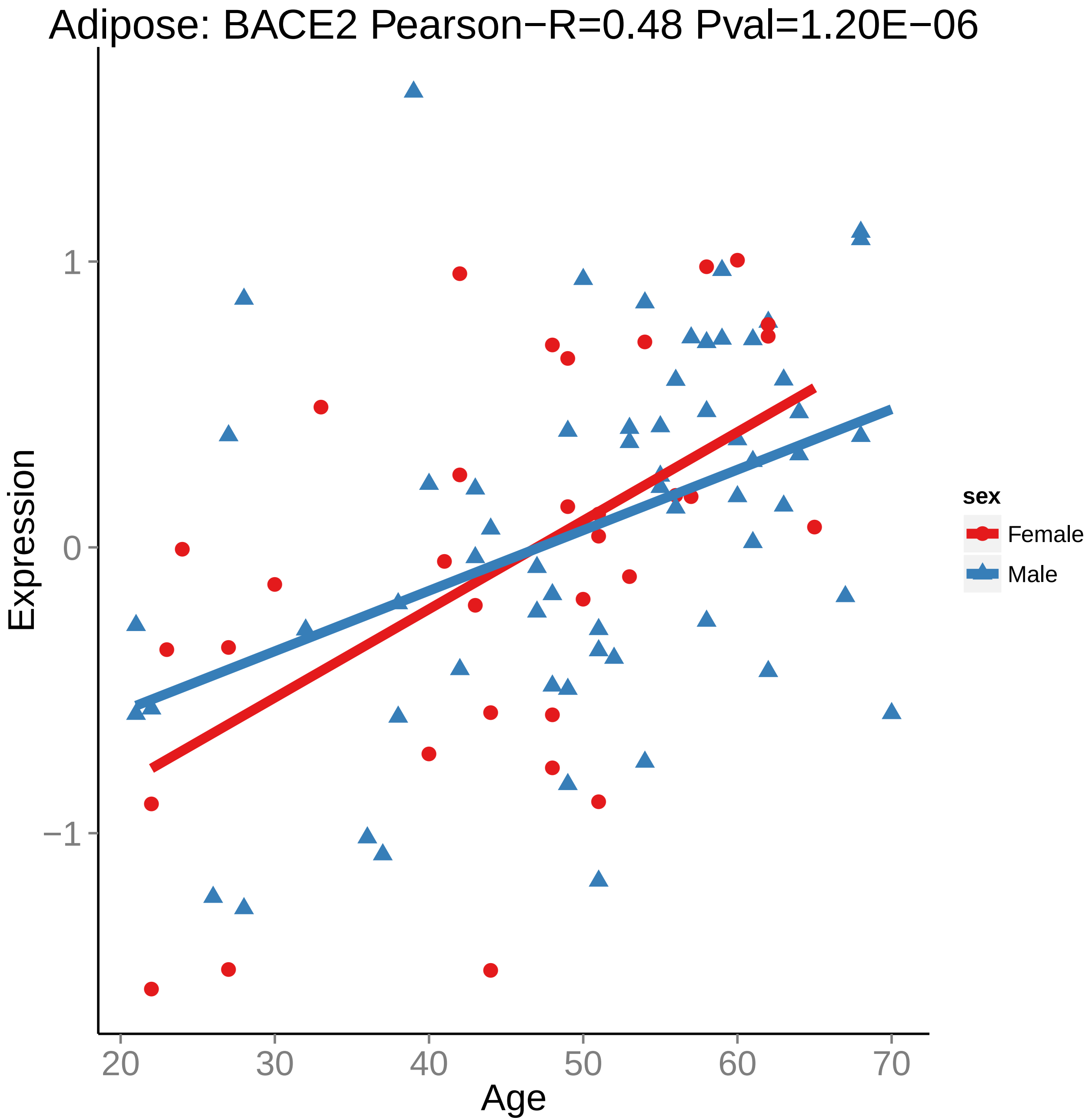
Adipose: BACE2 Pearson−R=0.48 Pval=1.20E−06
Expression
1
0
−1
20
30
40
50
60
70
Age
sex
Female
Male

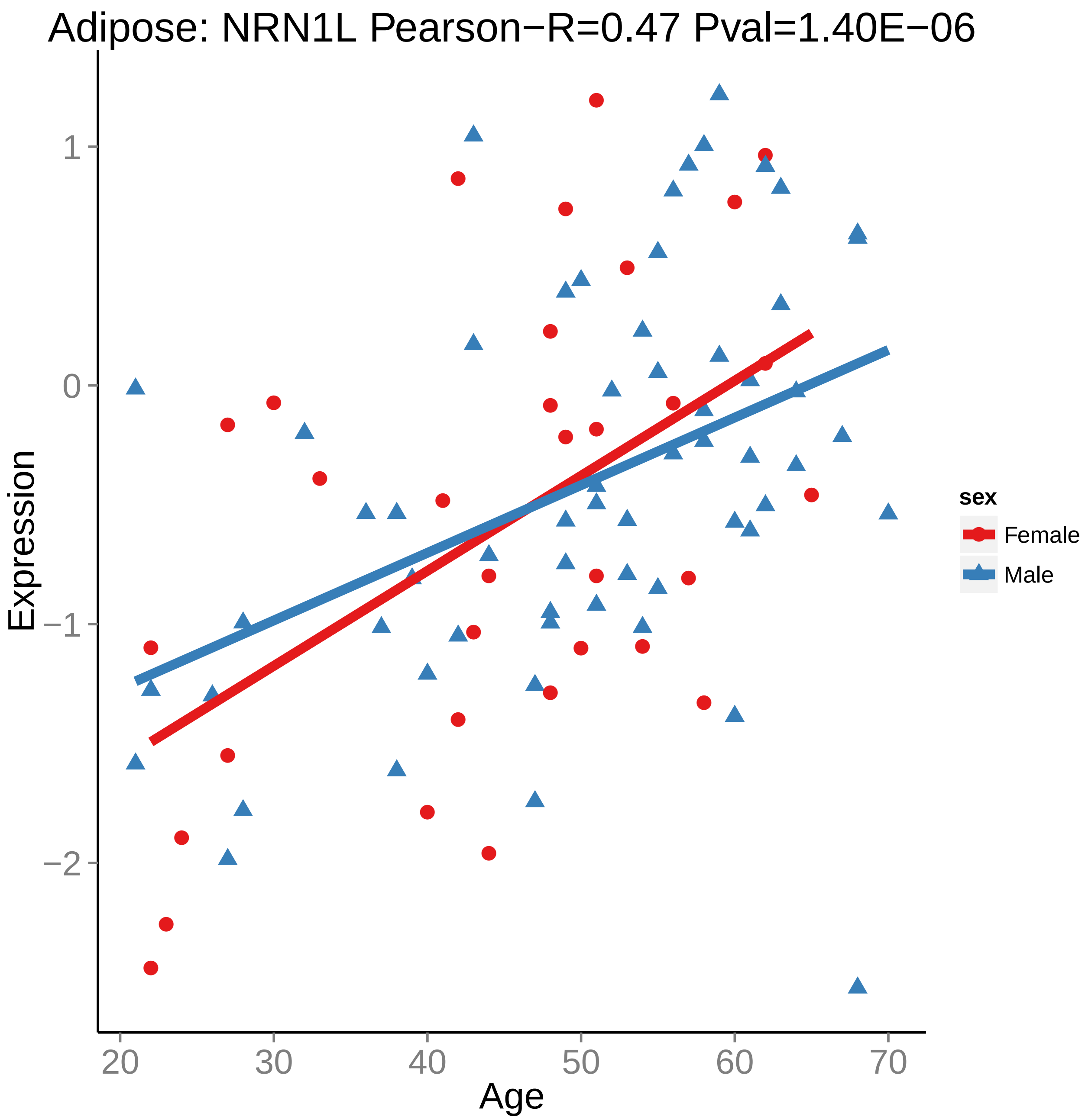
Adipose: NRN1L Pearson−R=0.47 Pval=1.40E−06
Expression
Age
1
0
−1
−2
20
30
40
50
60
70
sex
Female
Male

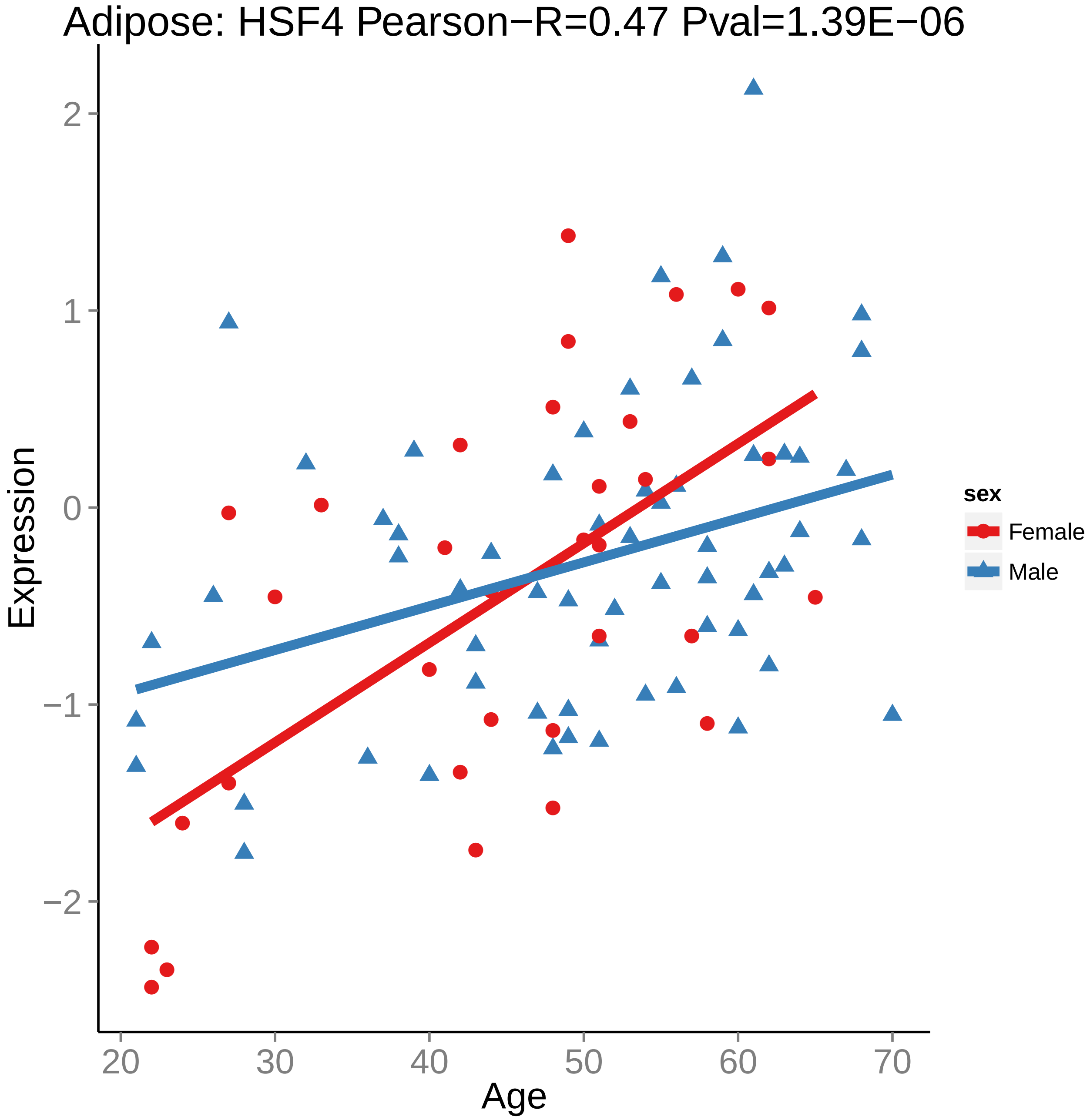
Adipose: HSF4 Pearson−R=0.47 Pval=1.39E−06
Expression
2
1
0
−1
−2
20
30
40
50
60
70
Age
sex
Female
Male

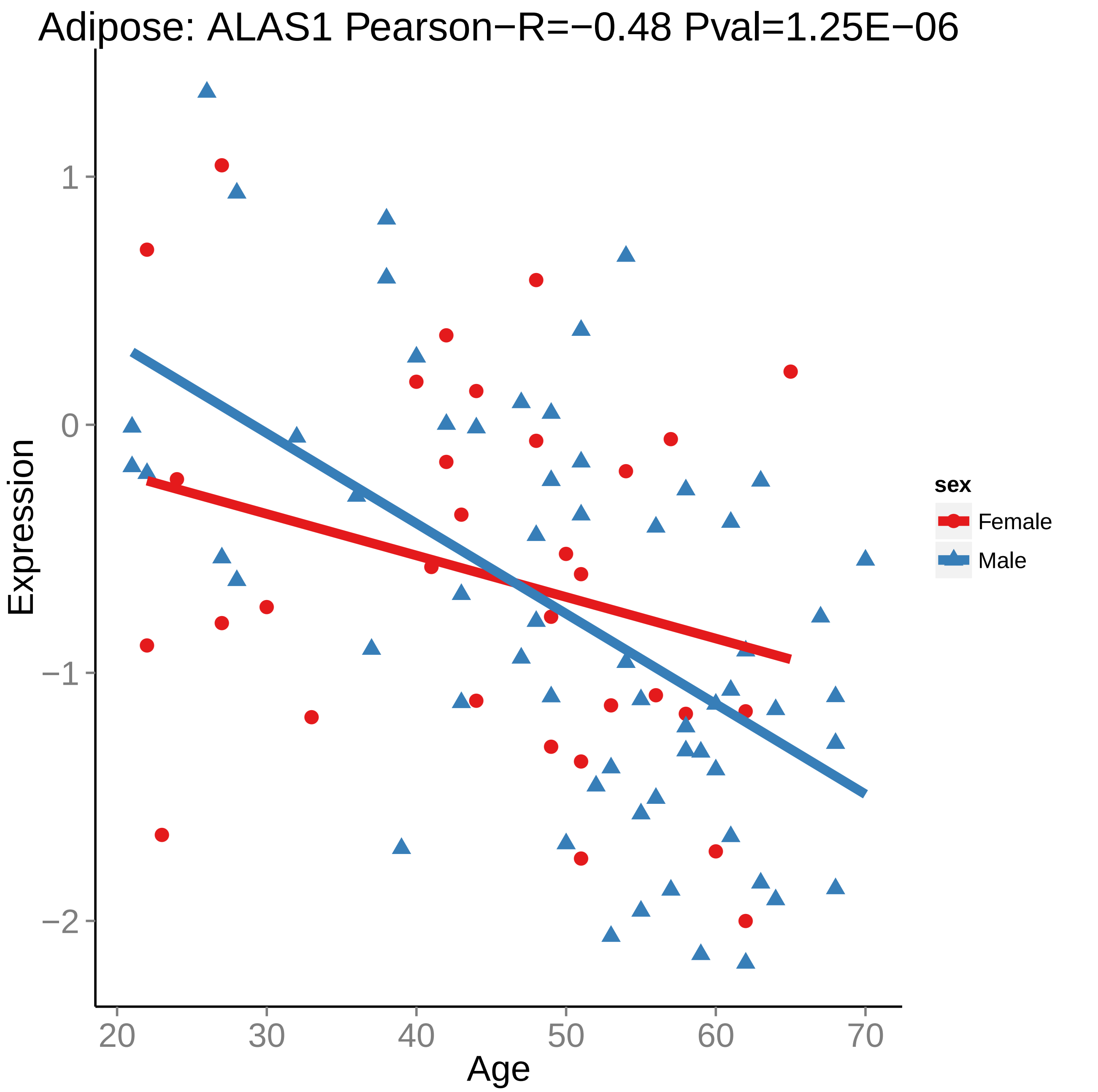

Adipose: ALAS1 Pearson−R=−0.48 Pval=1.25E−06
Expression
1
0
−1
−2
Age
20
30
40
50
60
70
sex
Female
Male

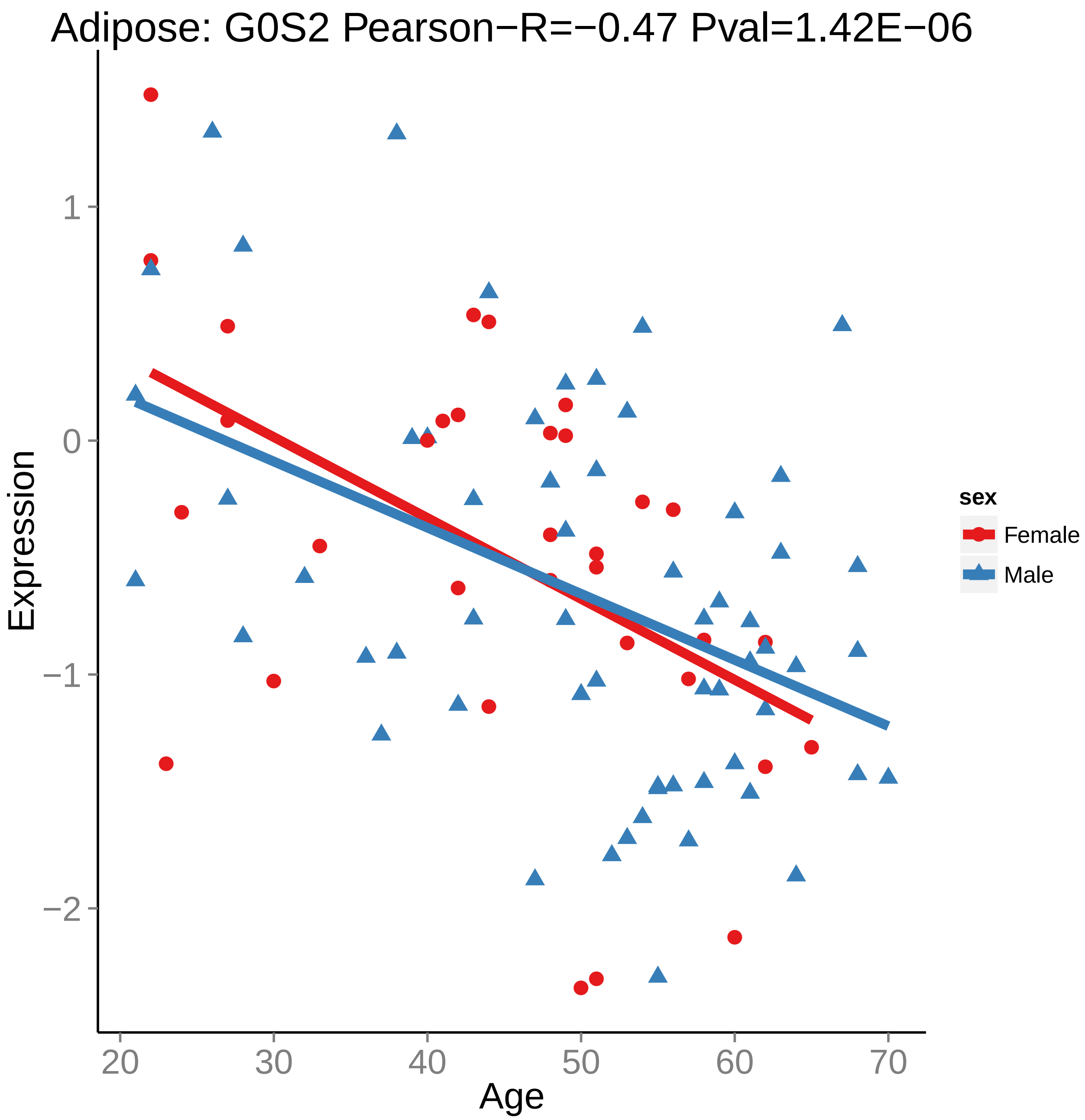
Adipose: G0S2 Pearson−R=−0.47 Pval=1.42E−06
Expression
1
0
−1
−2
20
30
40
50
60
70
Age
sex
Female
Male

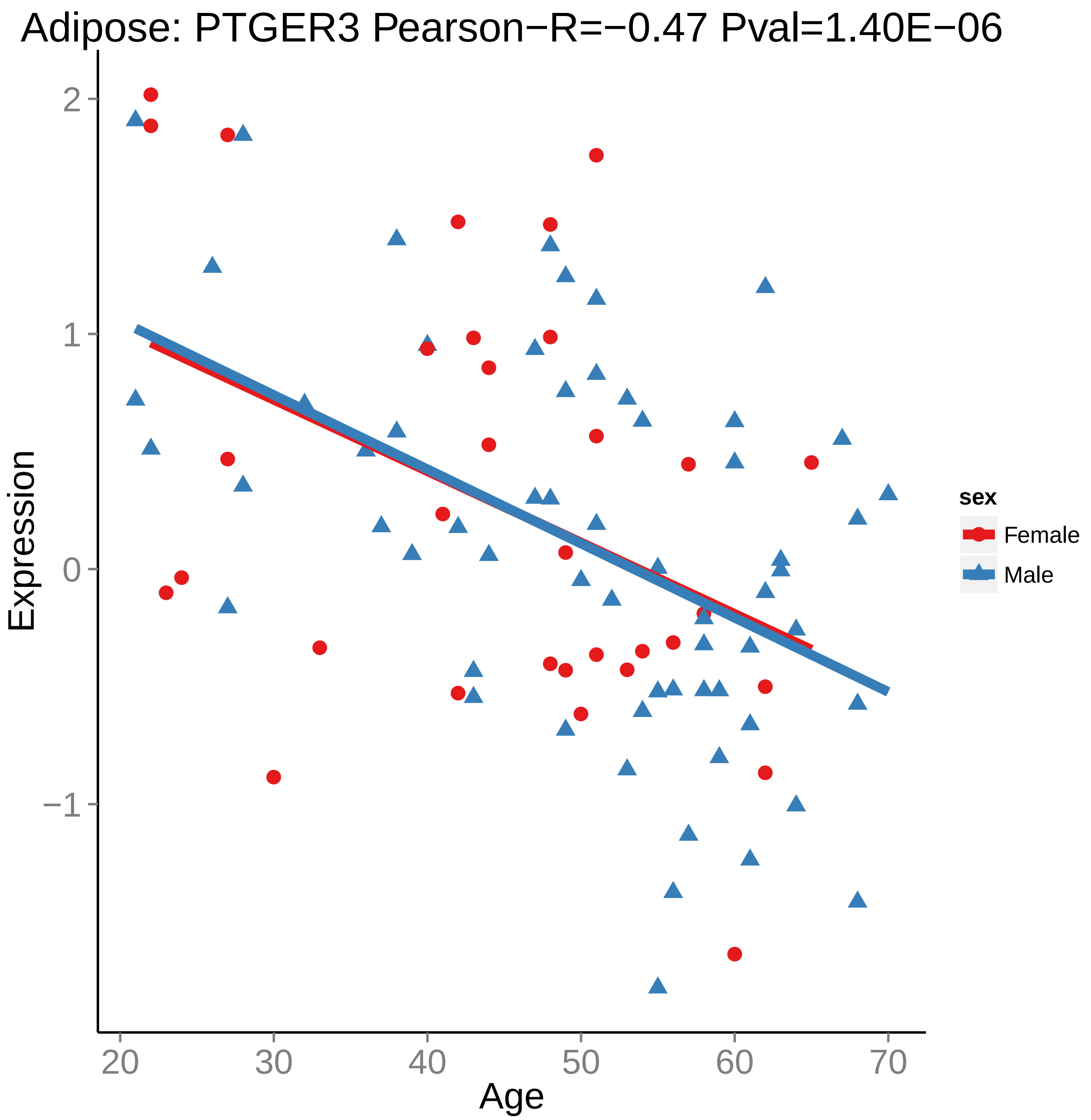
Adipose: PTGER3 Pearson−R=−0.47 Pval=1.40E−06
Expression
Age
2
1
0
−1
20
30
40
50
60
70
sex
Female
Male

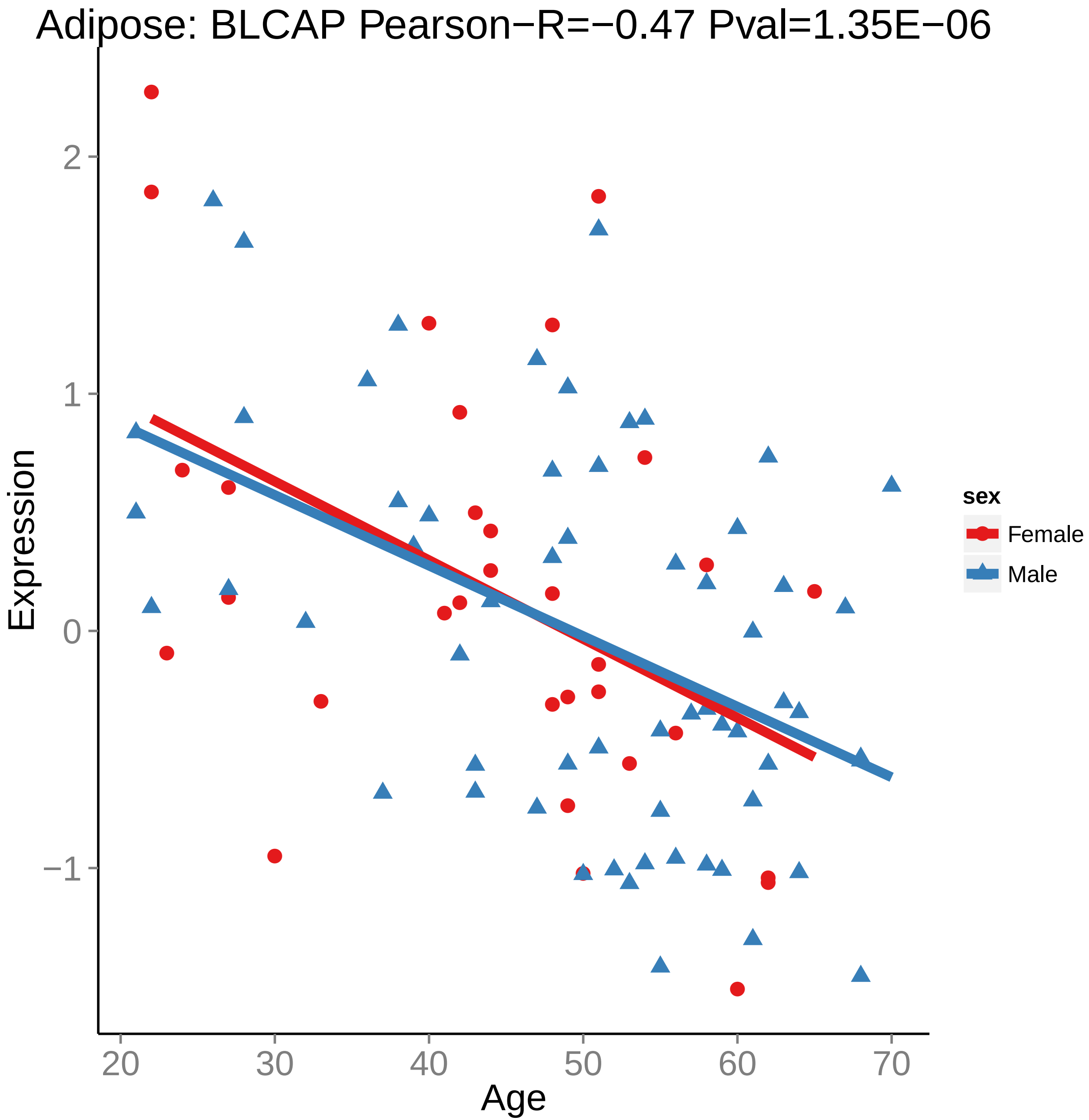
Adipose: BLCAP Pearson−R=−0.47 Pval=1.35E−06
Expression
Age
2
1
0
−1
20
30
40
50
60
70
sex
Female
Male

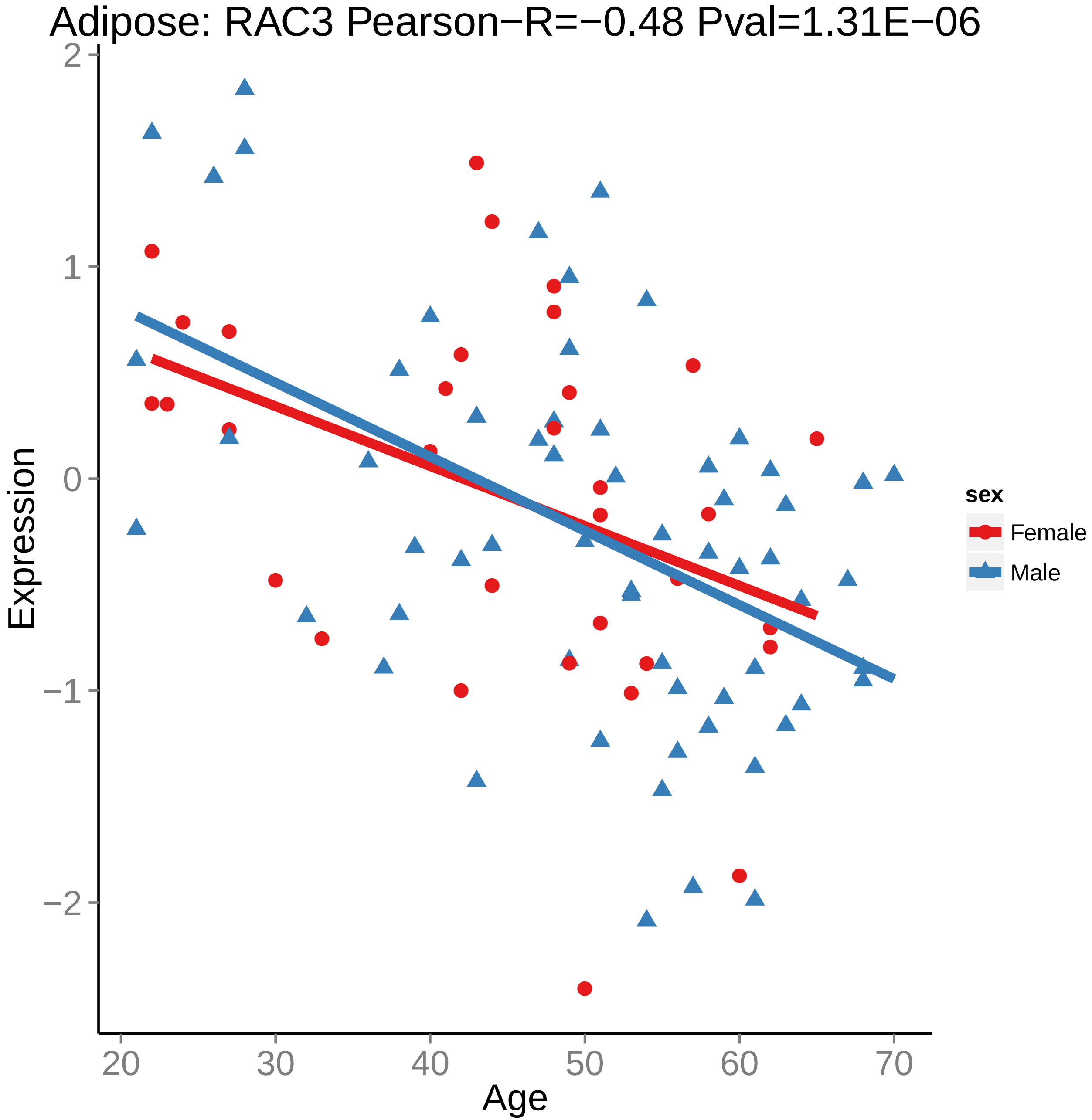

Adipose: RAC3 Pearson−R=−0.48 Pval=1.31E−06
Expression
Age
2
1
0
−1
−2
20
30
40
50
60
70
sex
Female
Male

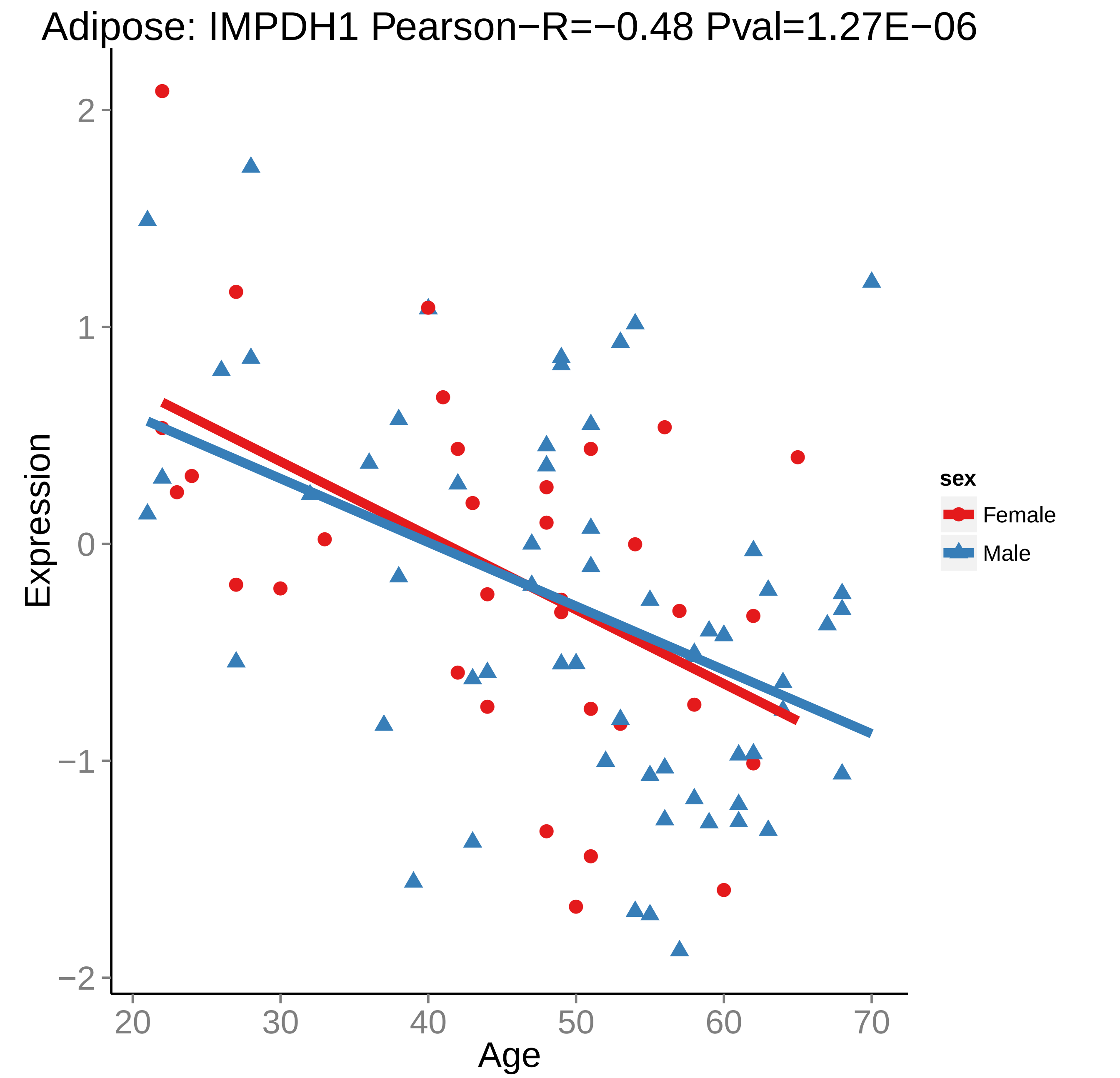

Adipose: IMPDH1 Pearson−R=−0.48 Pval=1.27E−06
Expression
Age
2
1
0
−1
−2
20
30
40
50
60
70
sex
Female
Male

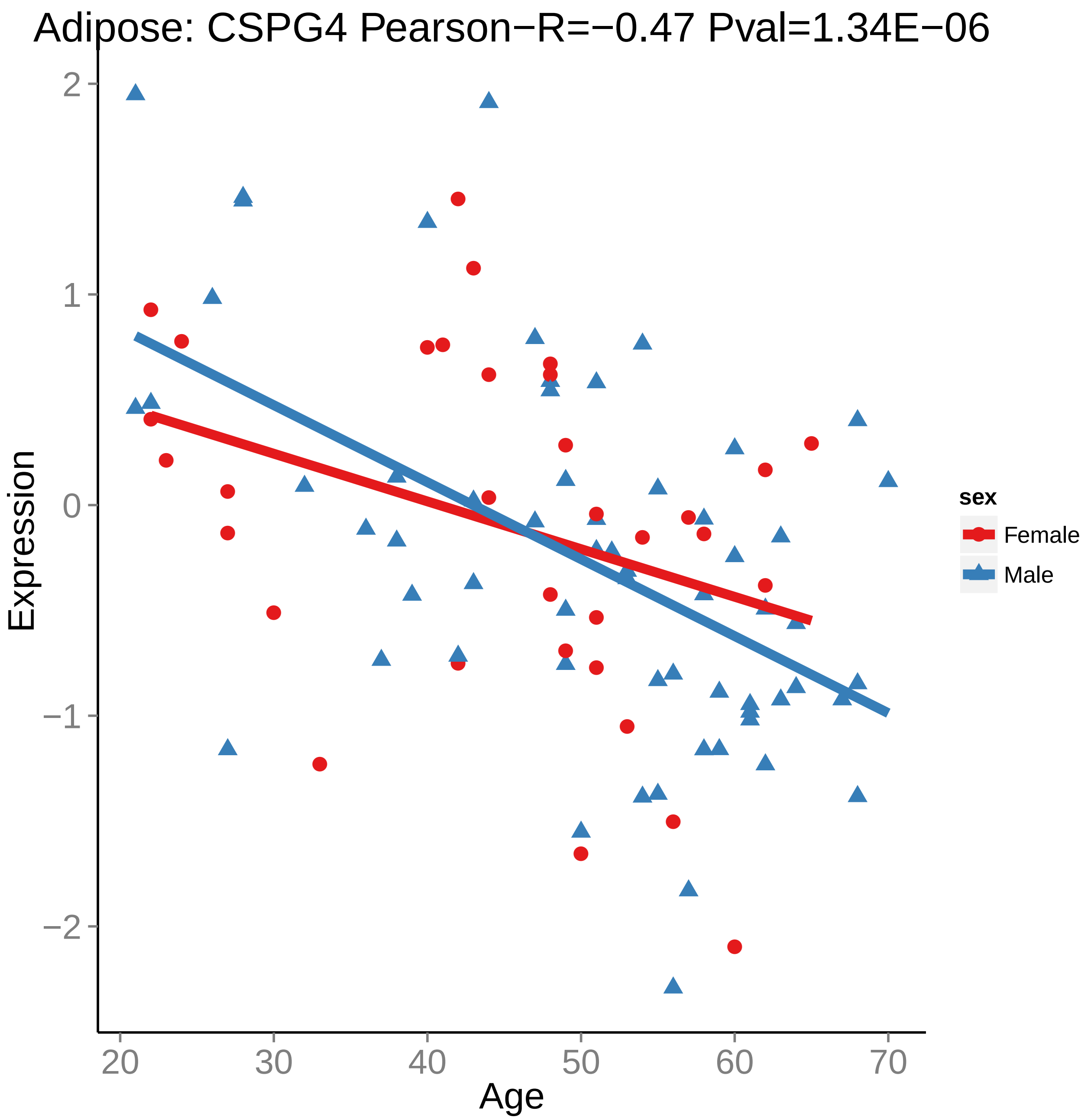

Adipose: CSPG4 Pearson−R=−0.47 Pval=1.34E−06
Expression
Age
2
1
0
−1
−2
20
30
40
50
60
70
sex
Female
Male

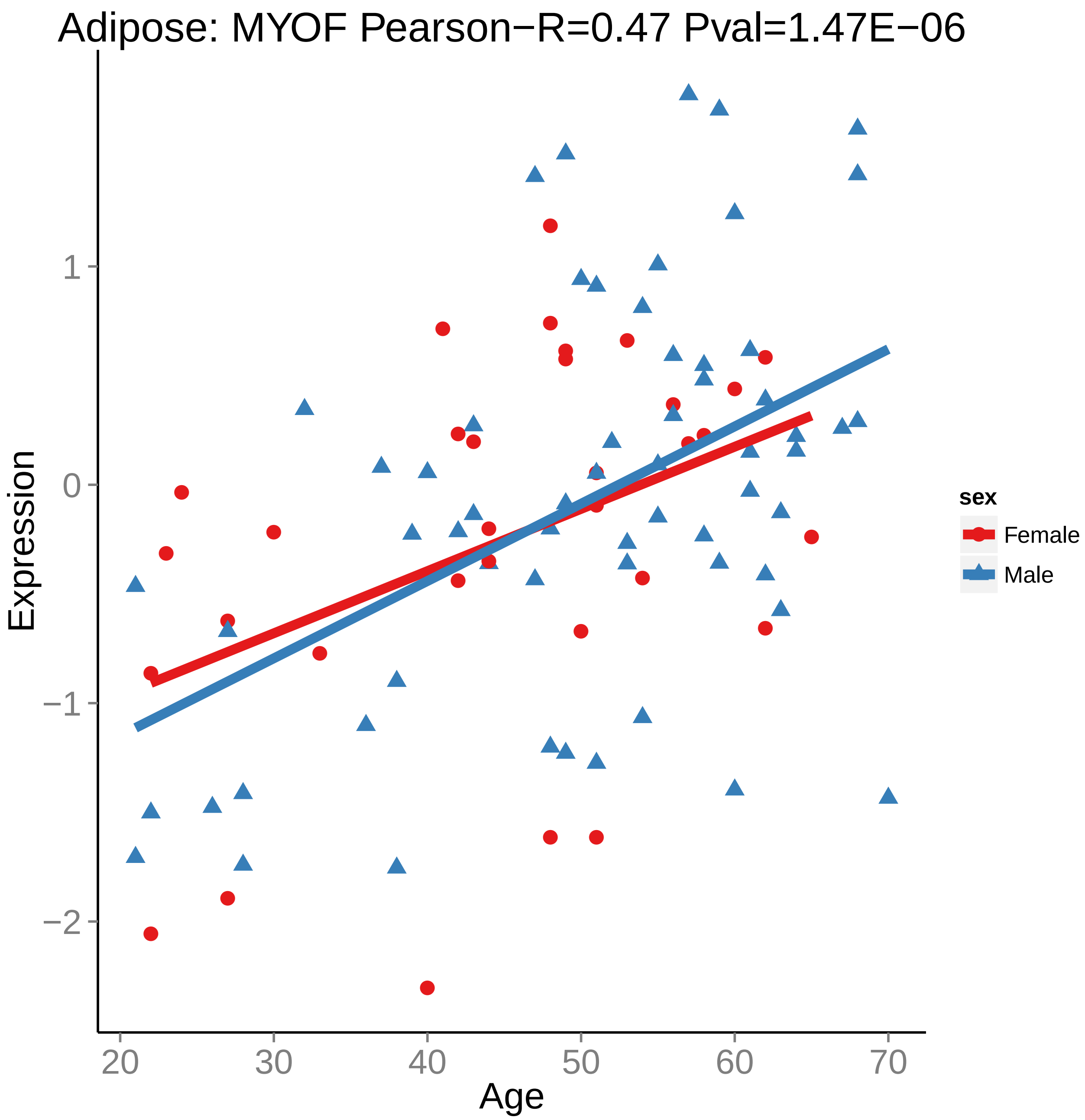

Adipose: MYOF Pearson−R=0.47 Pval=1.47E−06
Expression
1
0
−1
−2
20
30
40
50
60
70
Age
sex
Female
Male

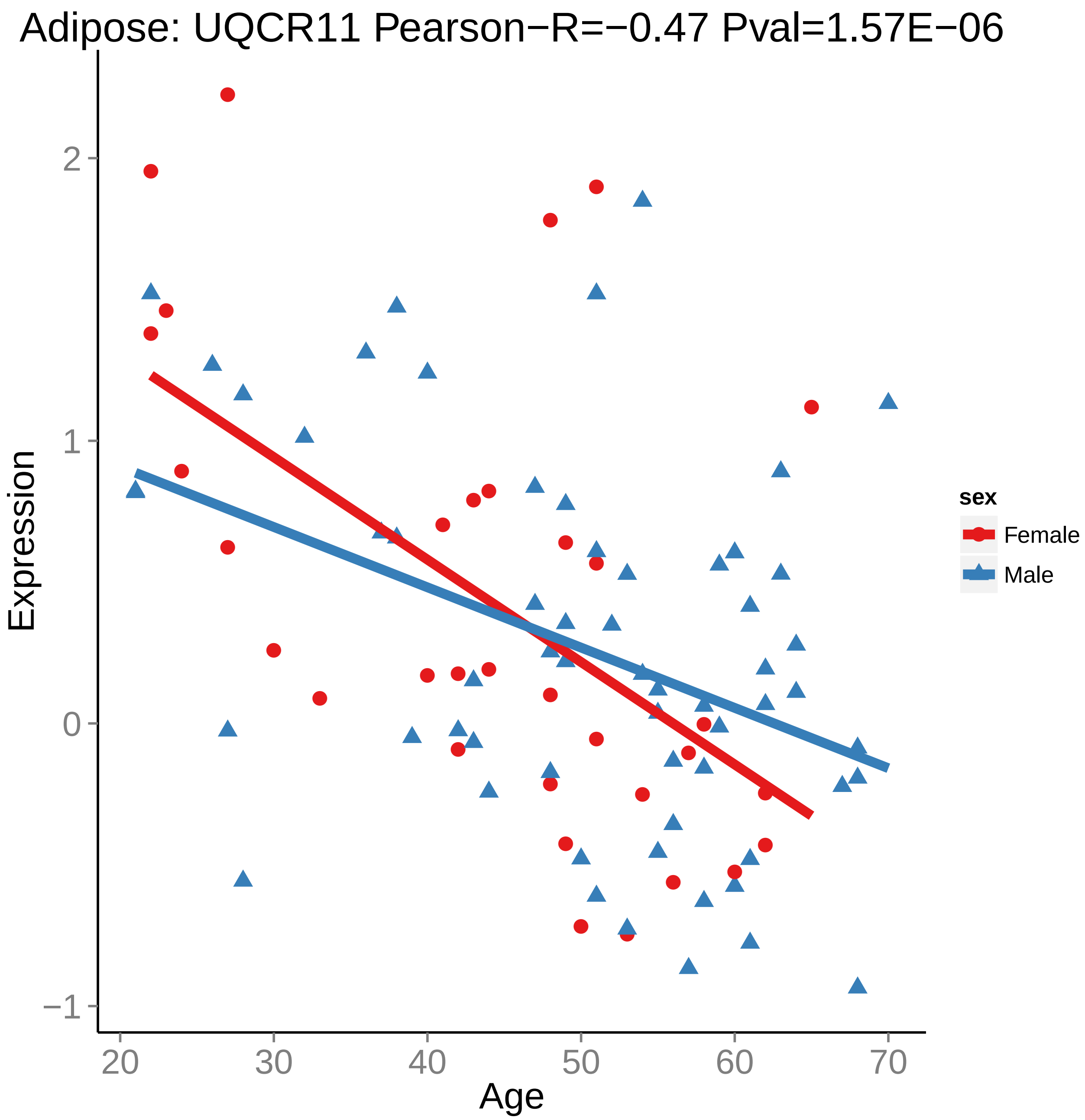

Adipose: UQCR11 Pearson−R=−0.47 Pval=1.57E−06
Expression
2
1
0
−1
20
30
40
50
60
70
Age
sex
Female
Male

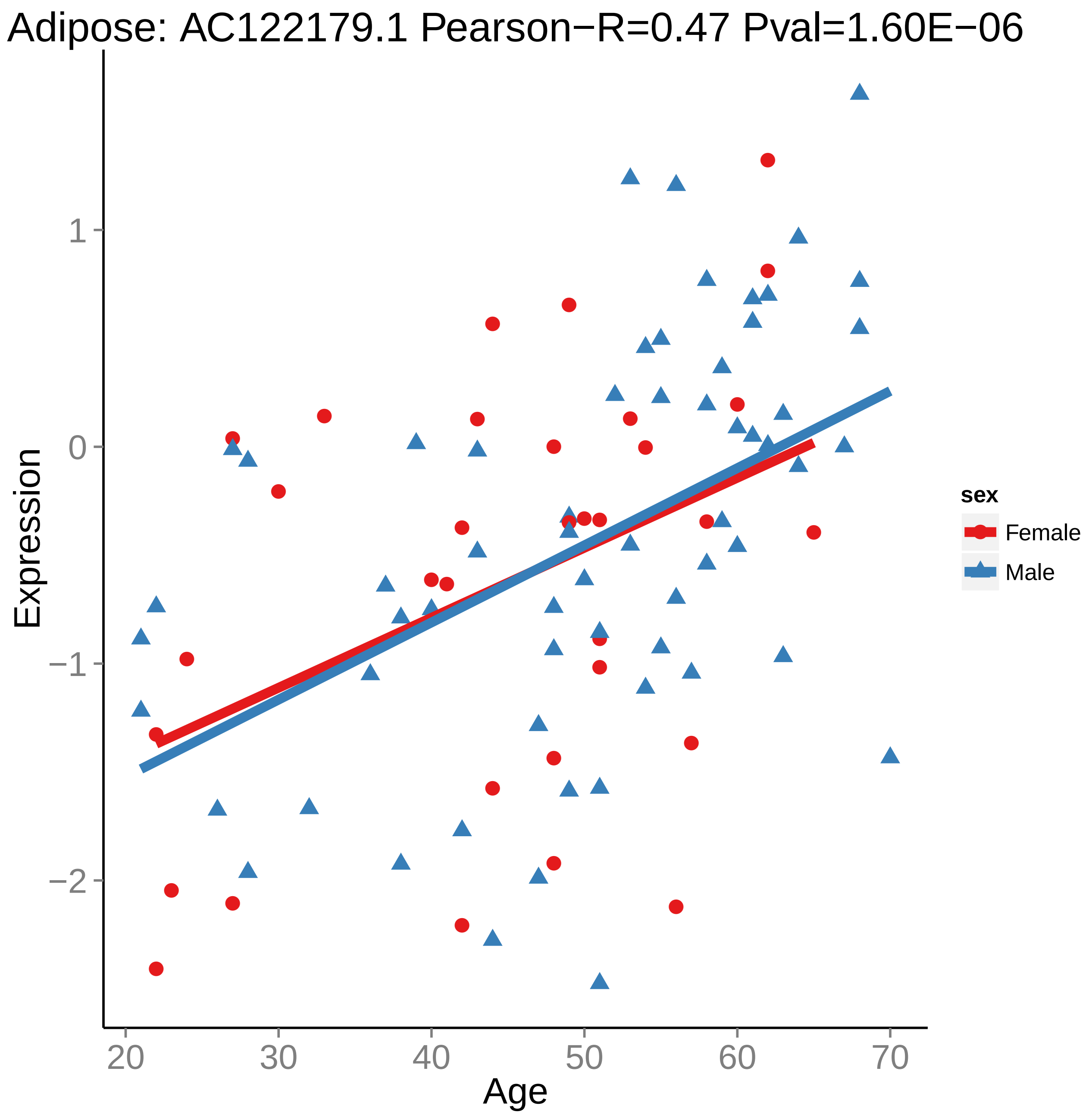

Adipose: AC122179.1 Pearson−R=0.47 Pval=1.60E−06
Expression
1
0
−1
−2
20
30
40
50
60
70
Age
sex
Female
Male

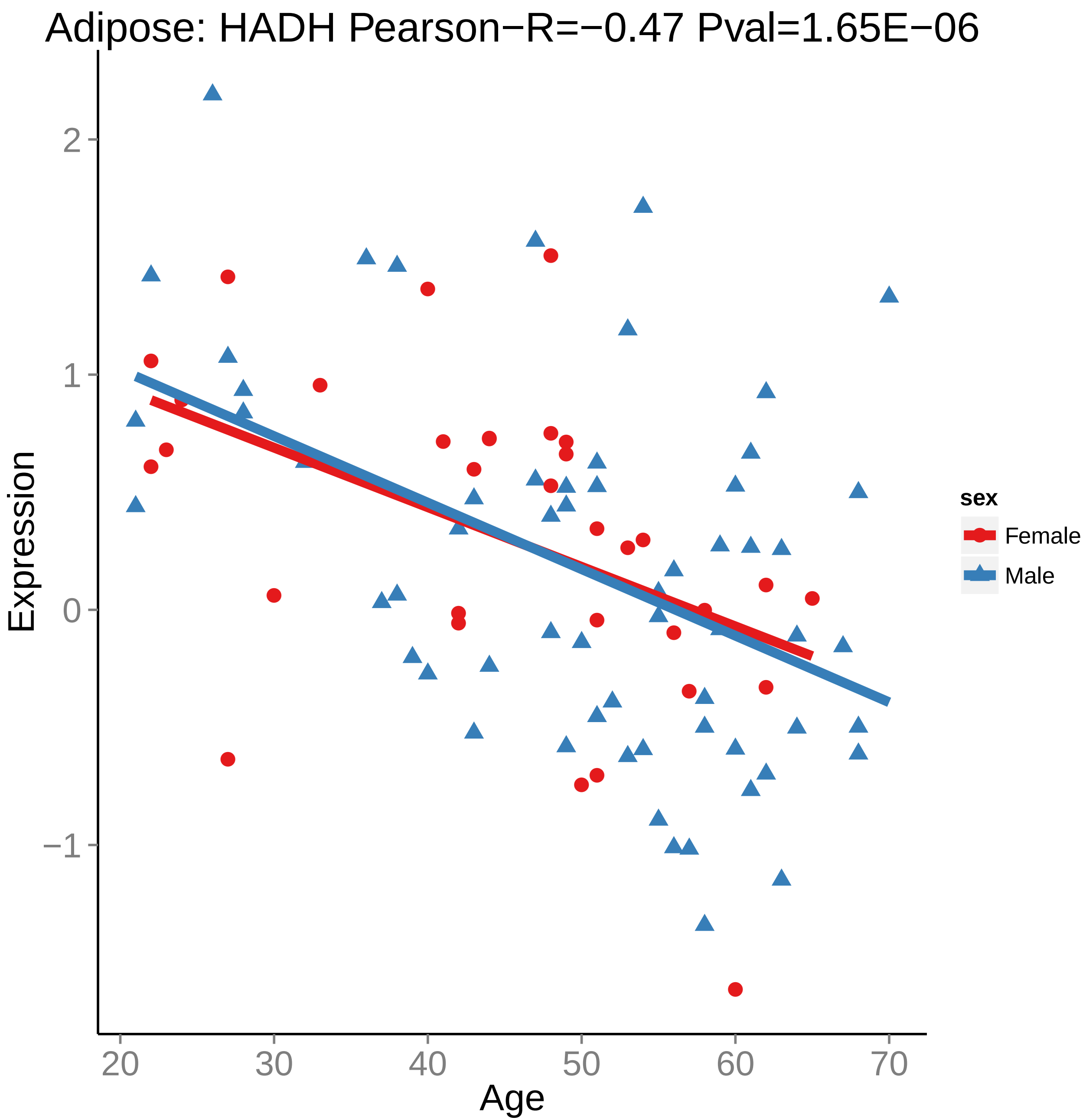

Adipose: HADH Pearson−R=−0.47 Pval=1.65E−06
Expression
2
1
0
−1
20
30
40
50
60
70
Age
sex
Female
Male

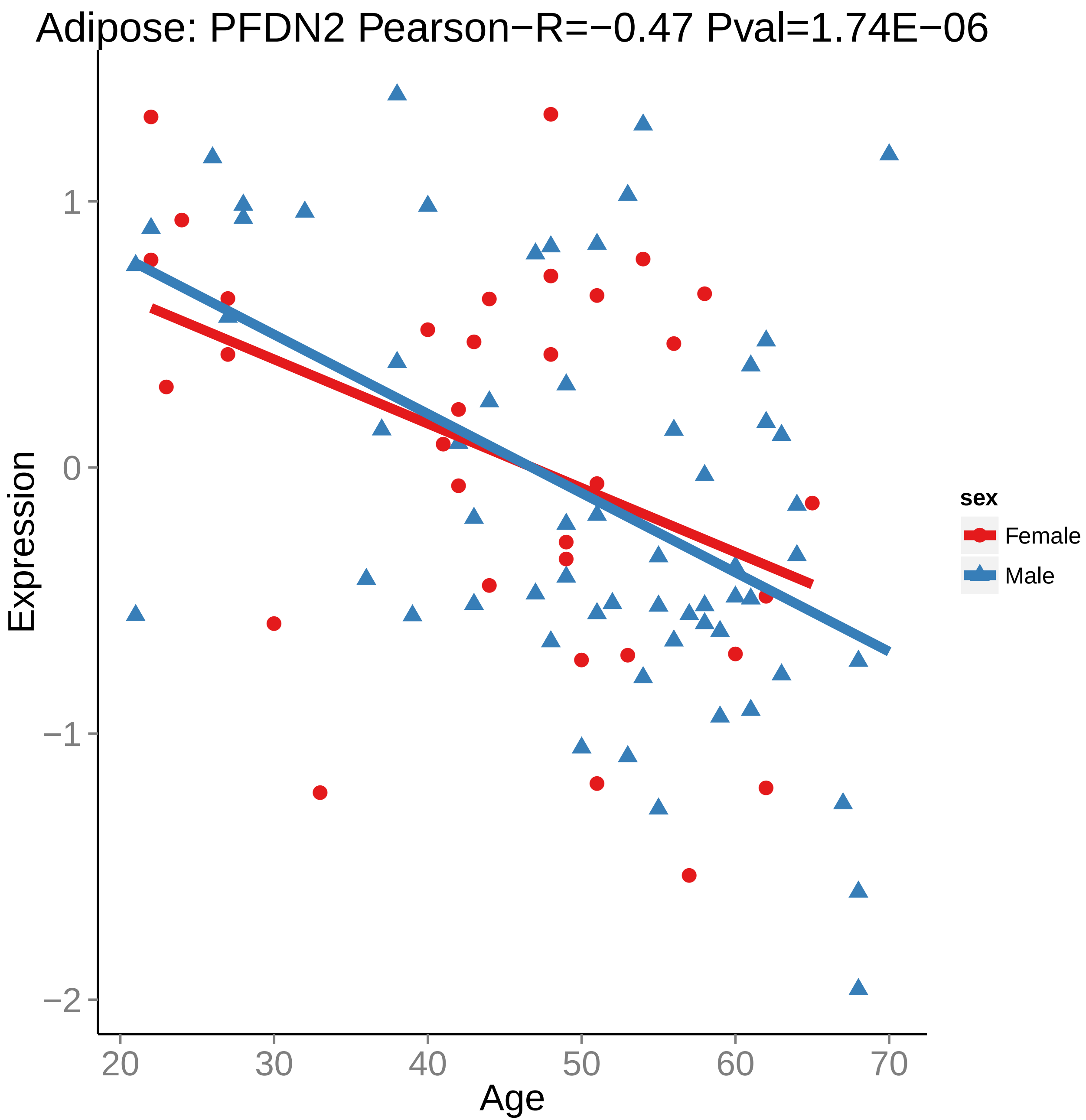
Adipose: PFDN2 Pearson−R=−0.47 Pval=1.74E−06
Expression
1
0
−1
−2
20
30
40
50
60
70
Age
sex
Female
Male

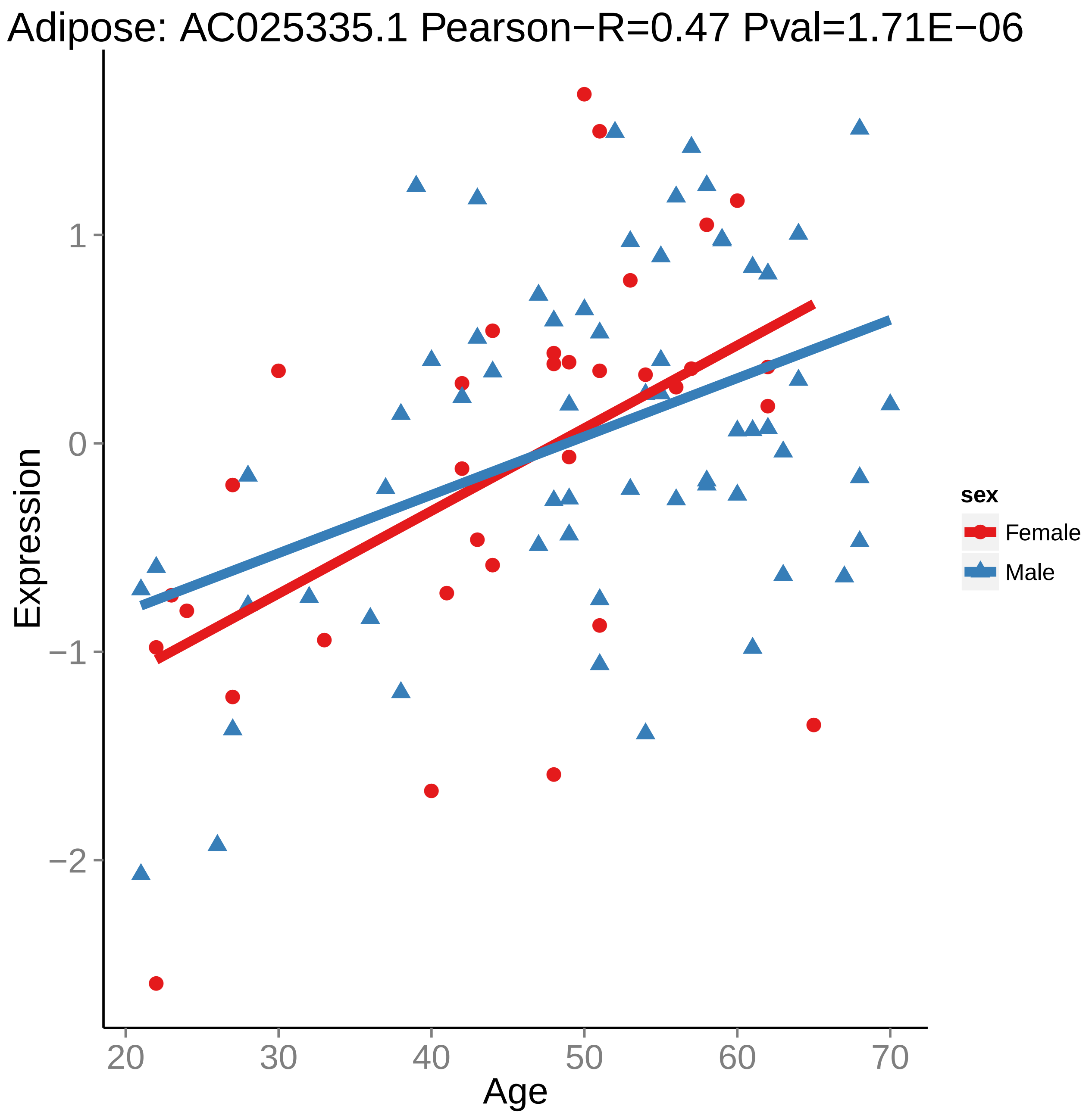

Adipose: AC025335.1 Pearson−R=0.47 Pval=1.71E−06
Expression
1
0
−1
−2
20
30
40
50
60
70
Age
sex
Female
Male

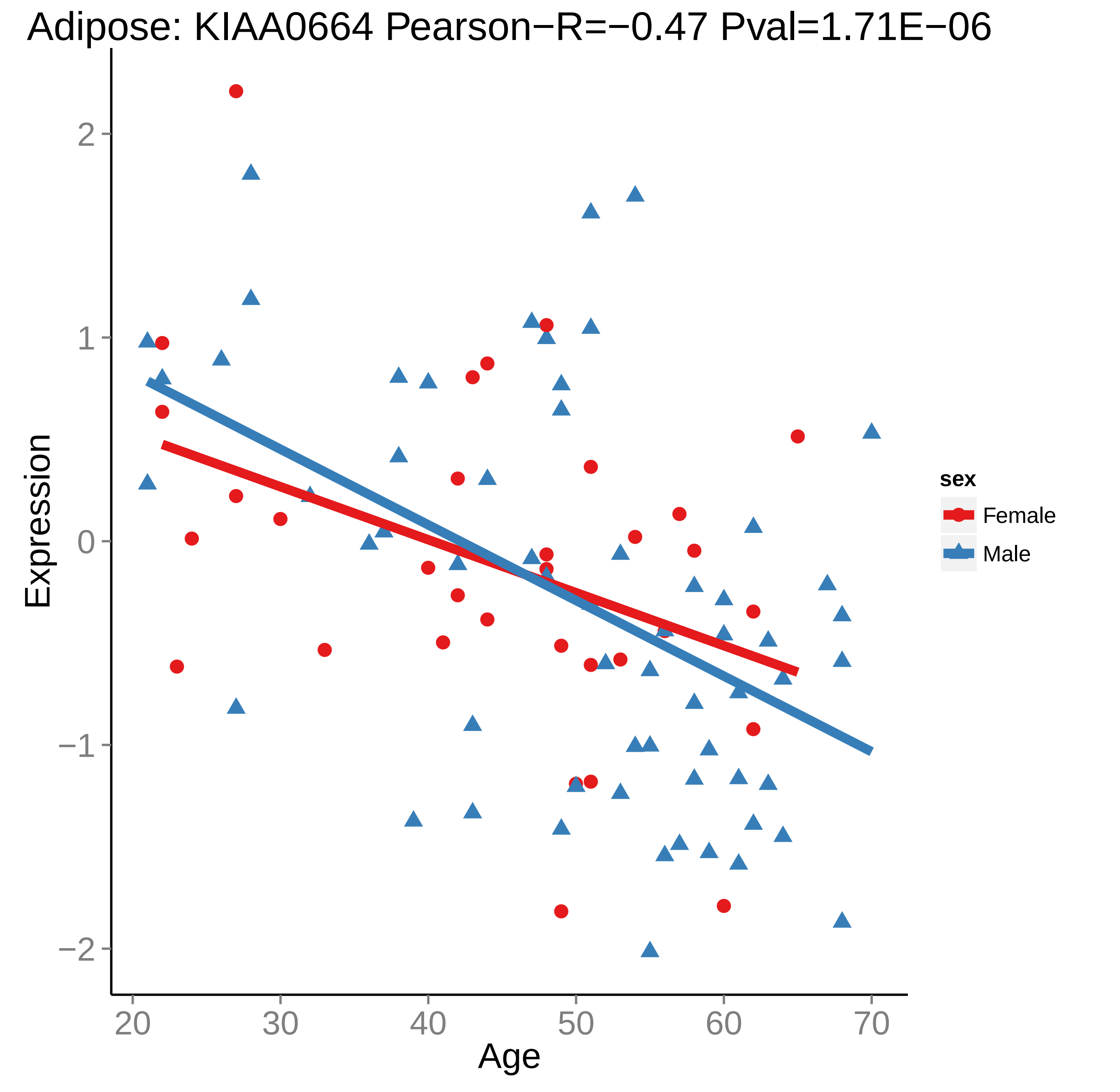
Adipose: KIAA0664 Pearson−R=−0.47 Pval=1.71E−06
Expression
2
1
0
−1
−2
20
30
40
50
60
70
Age
sex
Female
Male

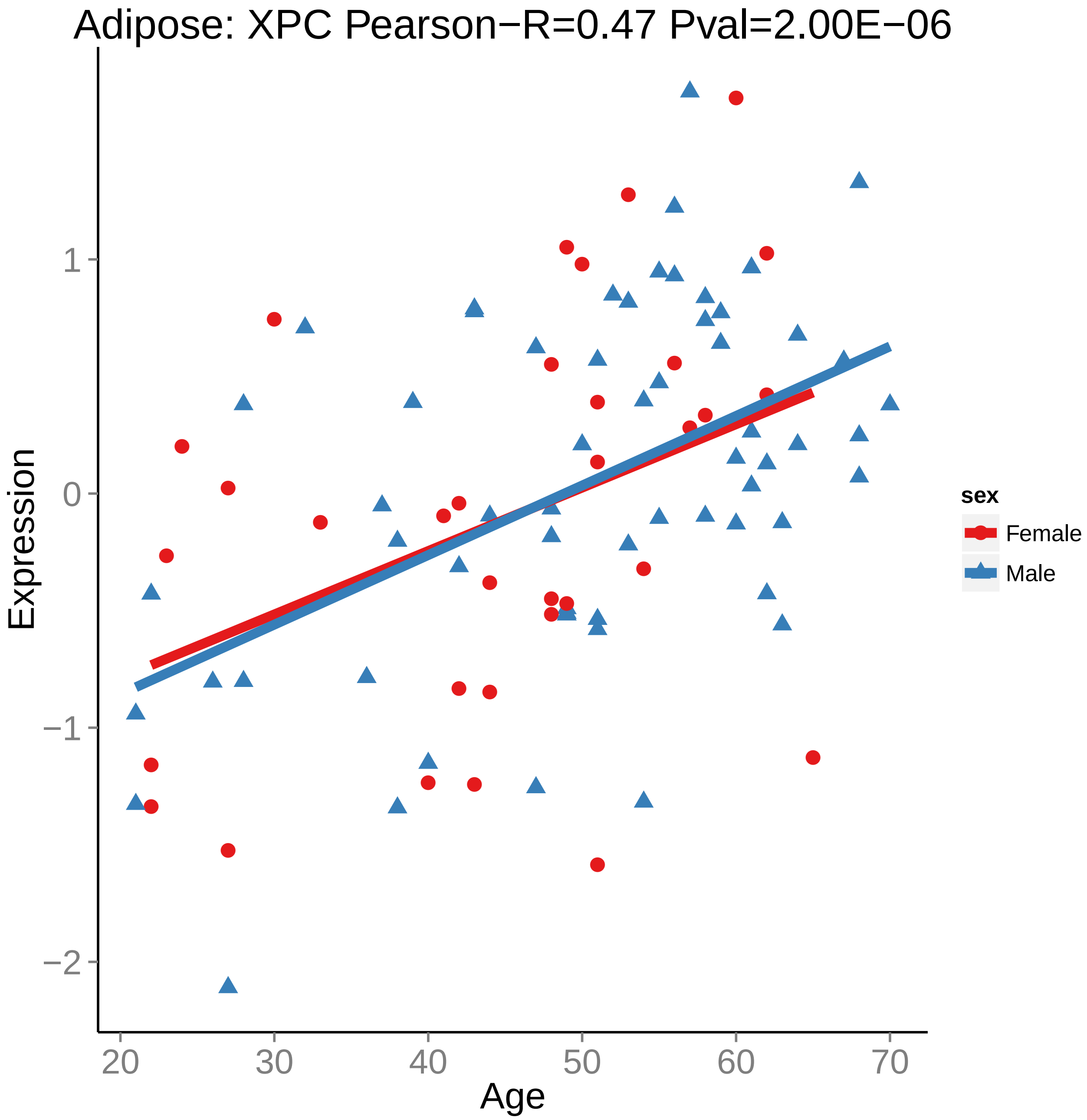

Adipose: XPC Pearson−R=0.47 Pval=2.00E−06
Expression
1
0
−1
−2
20
30
40
50
60
70
Age
sex
Female
Male

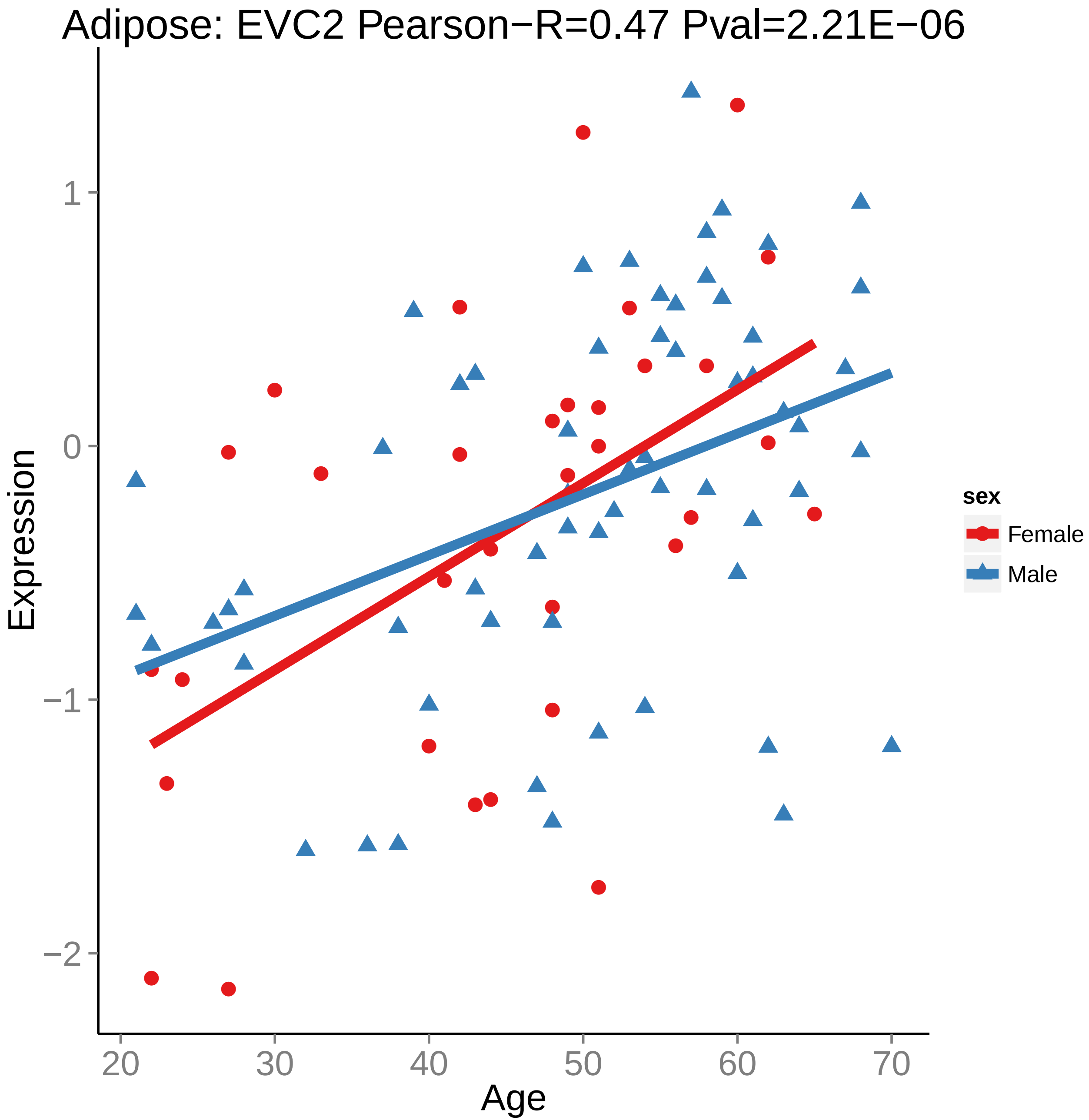
Adipose: EVC2 Pearson−R=0.47 Pval=2.21E−06
Expression
1
0
−1
−2
20
30
40
50
60
70
Age
sex
Female
Male

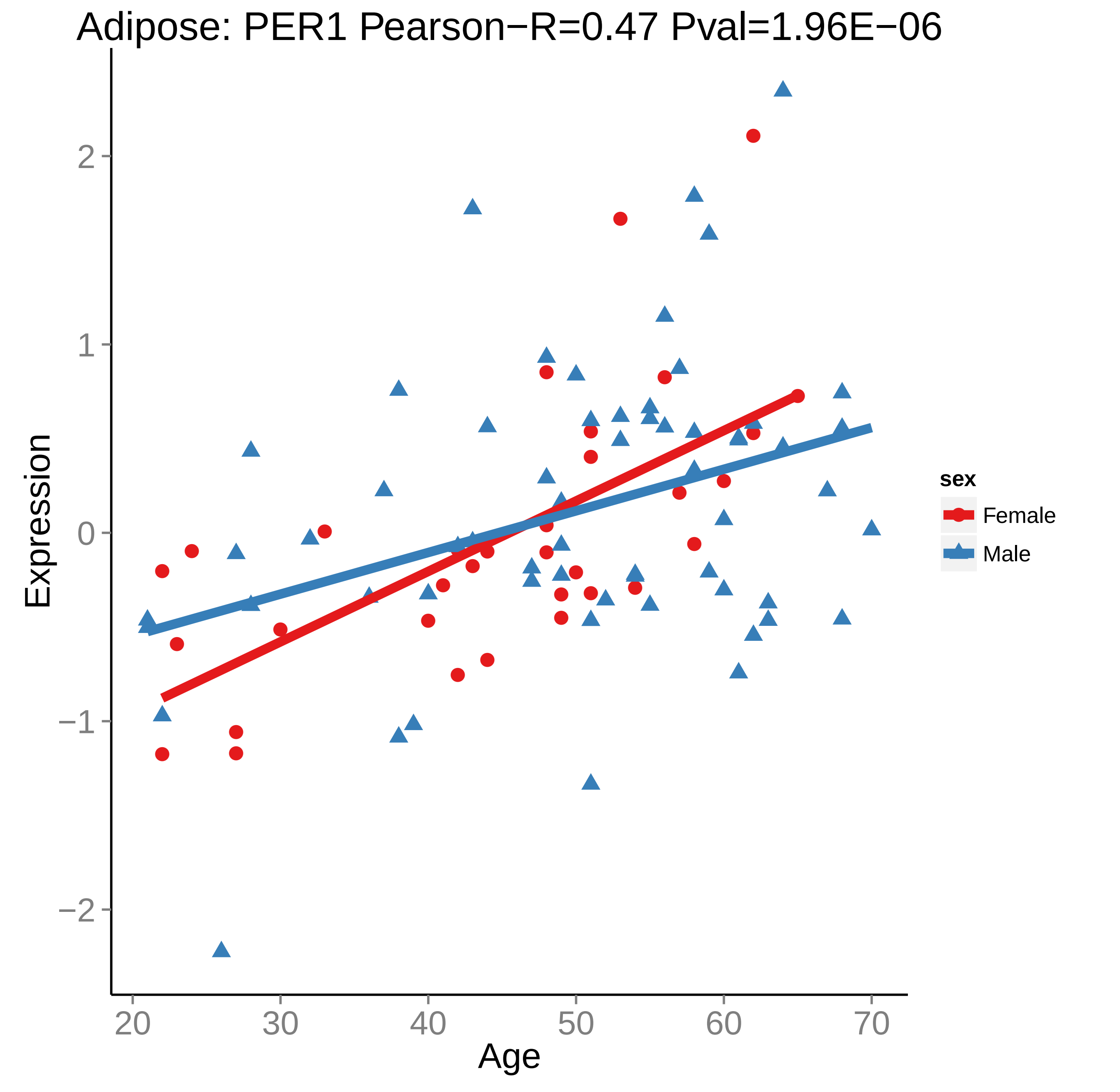
Adipose: PER1 Pearson−R=0.47 Pval=1.96E−06
Expression
2
1
0
−1
−2
20
30
40
50
60
70
Age
sex
Female
Male

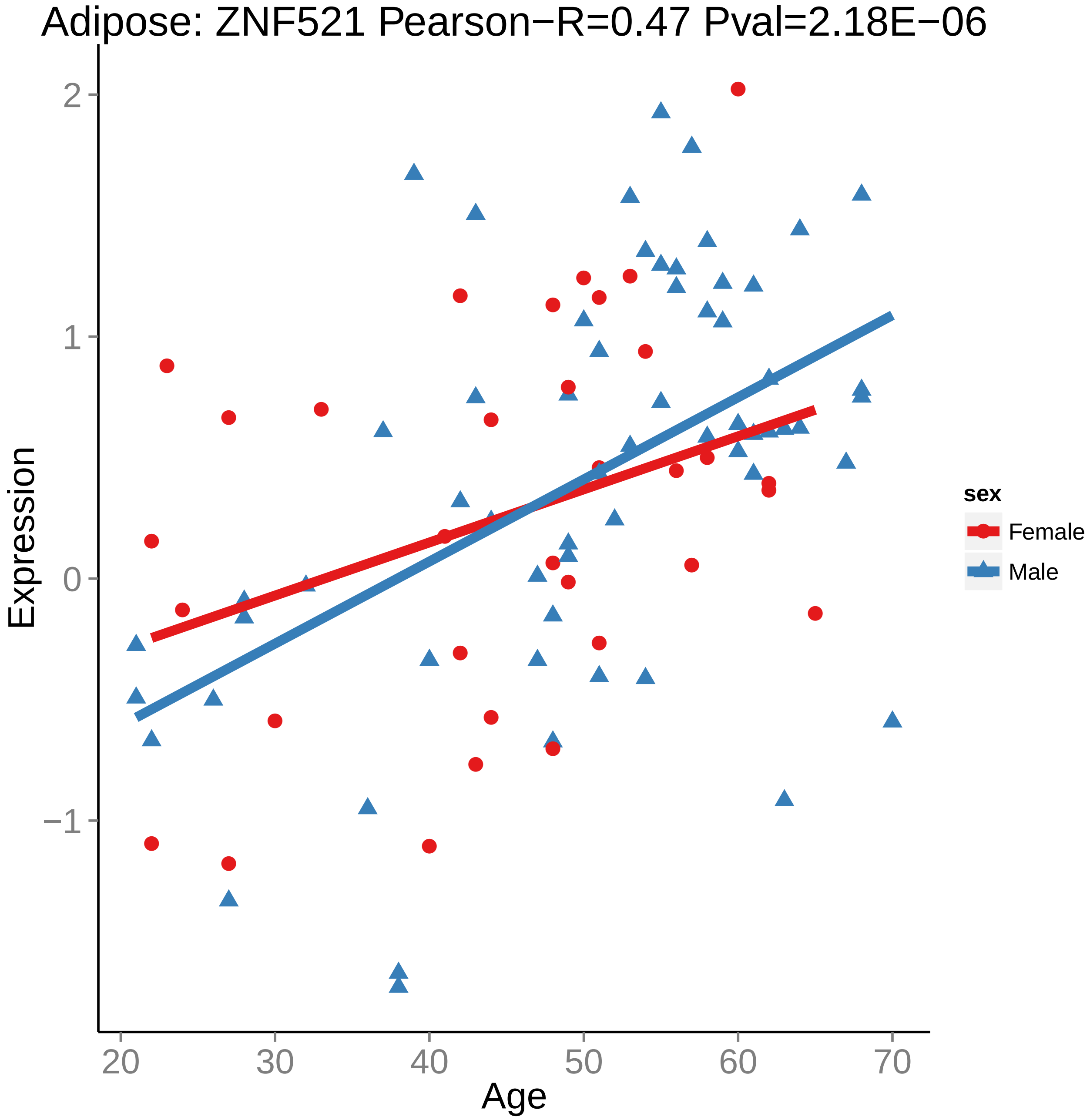
Adipose: ZNF521 Pearson−R=0.47 Pval=2.18E−06
Expression
2
1
0
−1
20
30
40
50
60
70
Age
sex
Female
Male

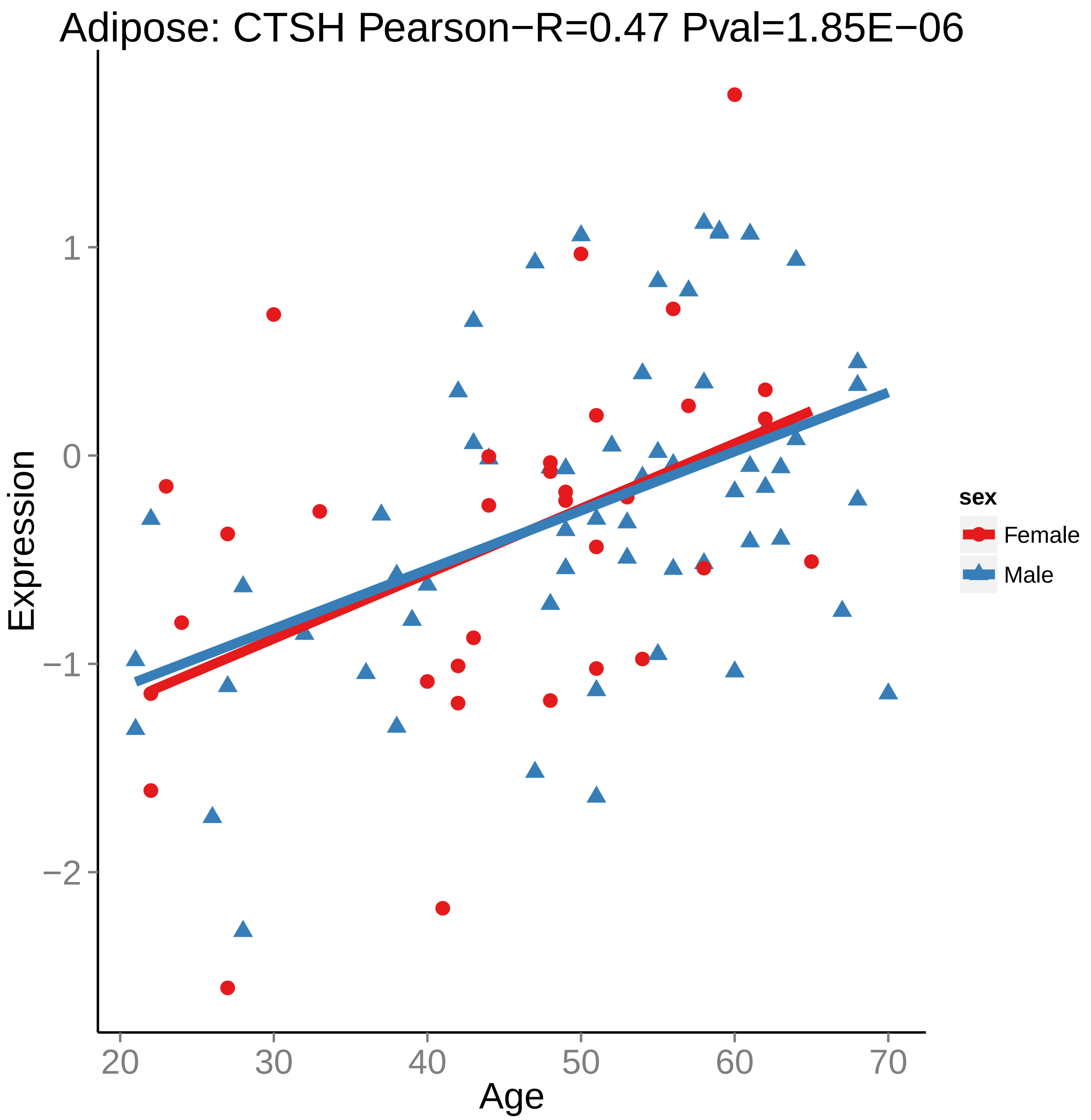
Adipose: CTSH Pearson−R=0.47 Pval=1.85E−06
Expression
Age
sex
Female
Male
1
0
−1
−2
20
30
40
50
60
70

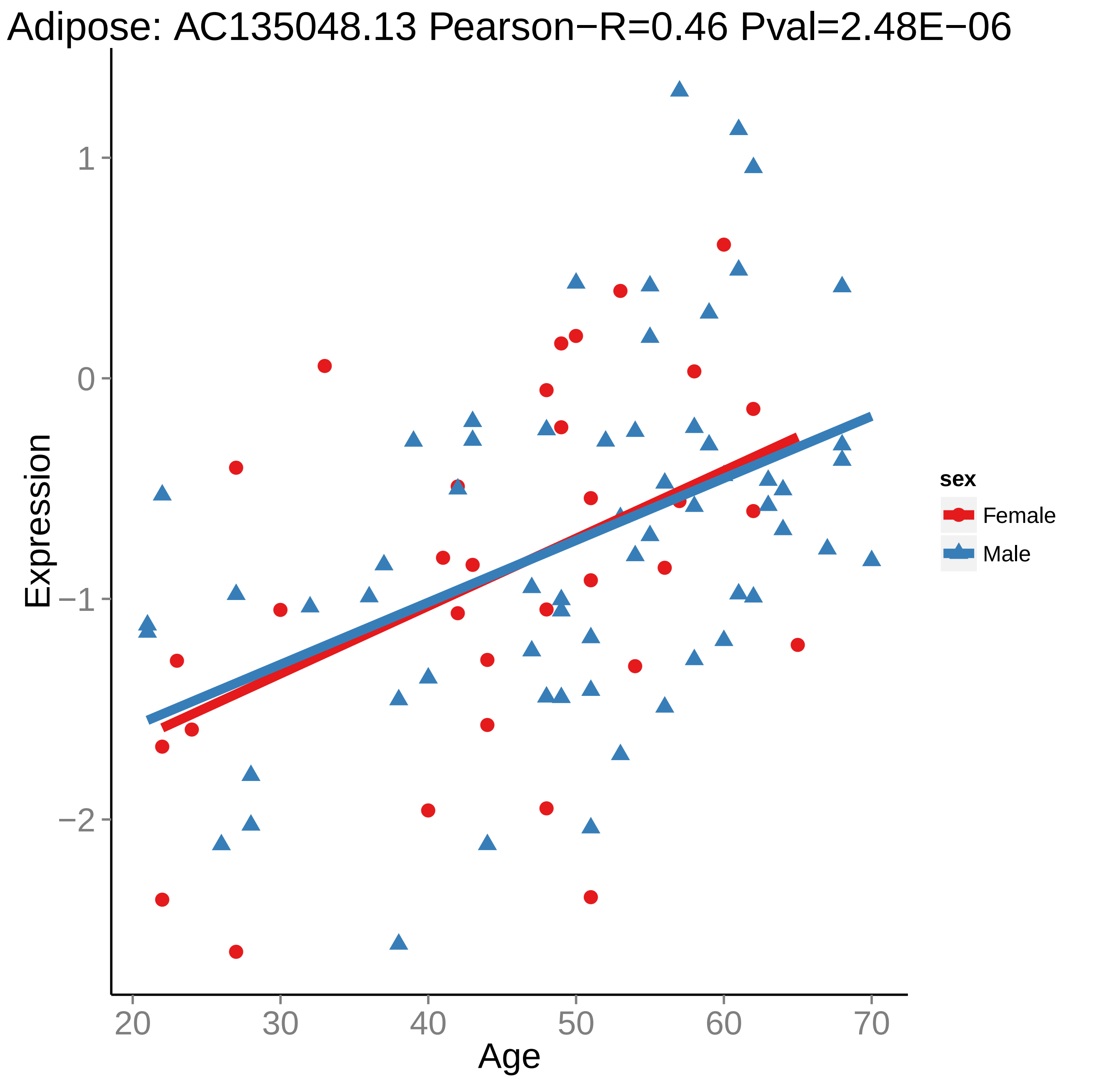
Adipose: AC135048.13 Pearson−R=0.46 Pval=2.48E−06
Expression
Age
1
0
−1
−2
20
30
40
50
60
70
sex
Female
Male

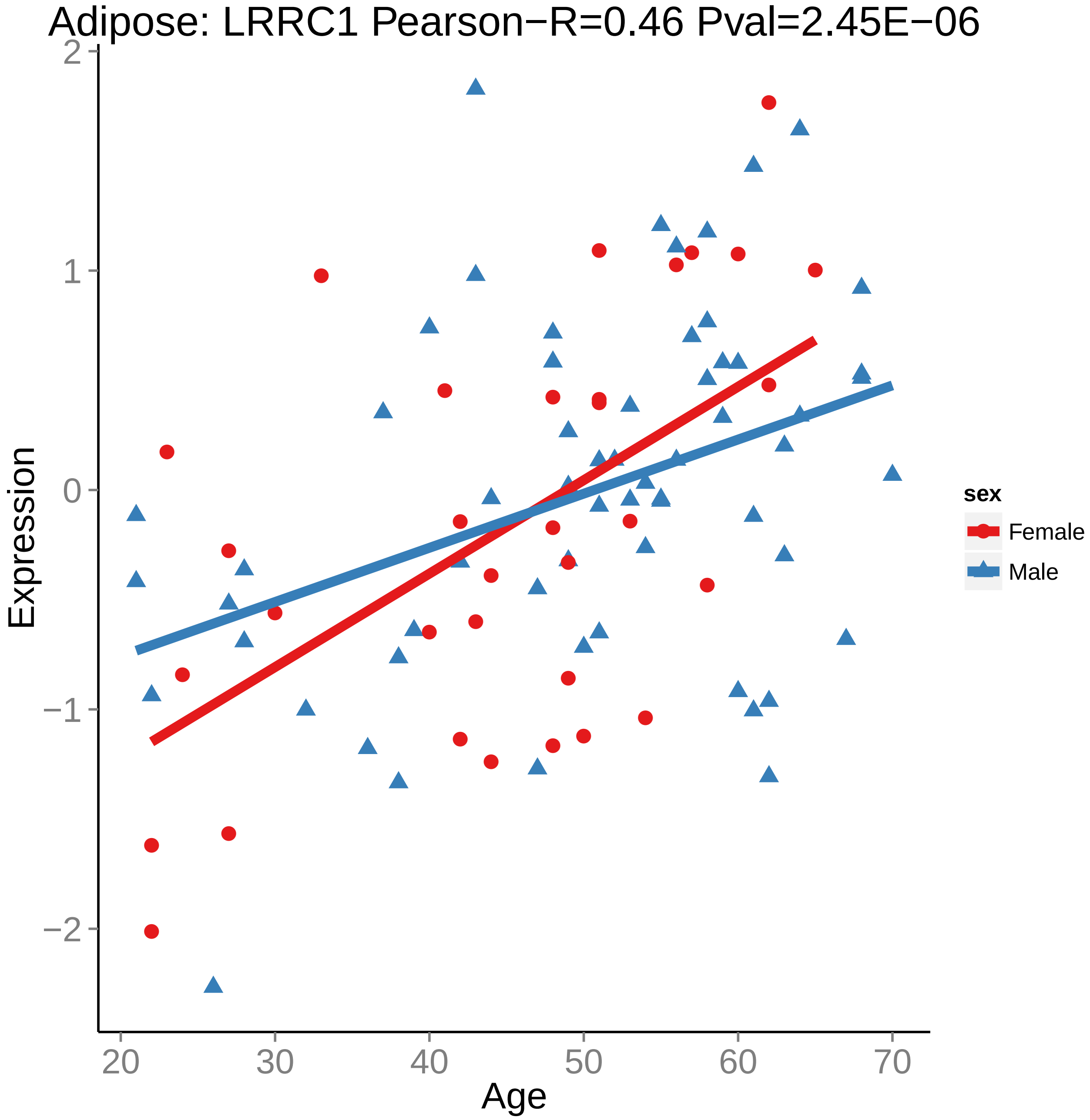

Adipose: LRRC1 Pearson−R=0.46 Pval=2.45E−06
Expression
Age
sex
Female
Male
2
1
0
−1
−2
20
30
40
50
60
70

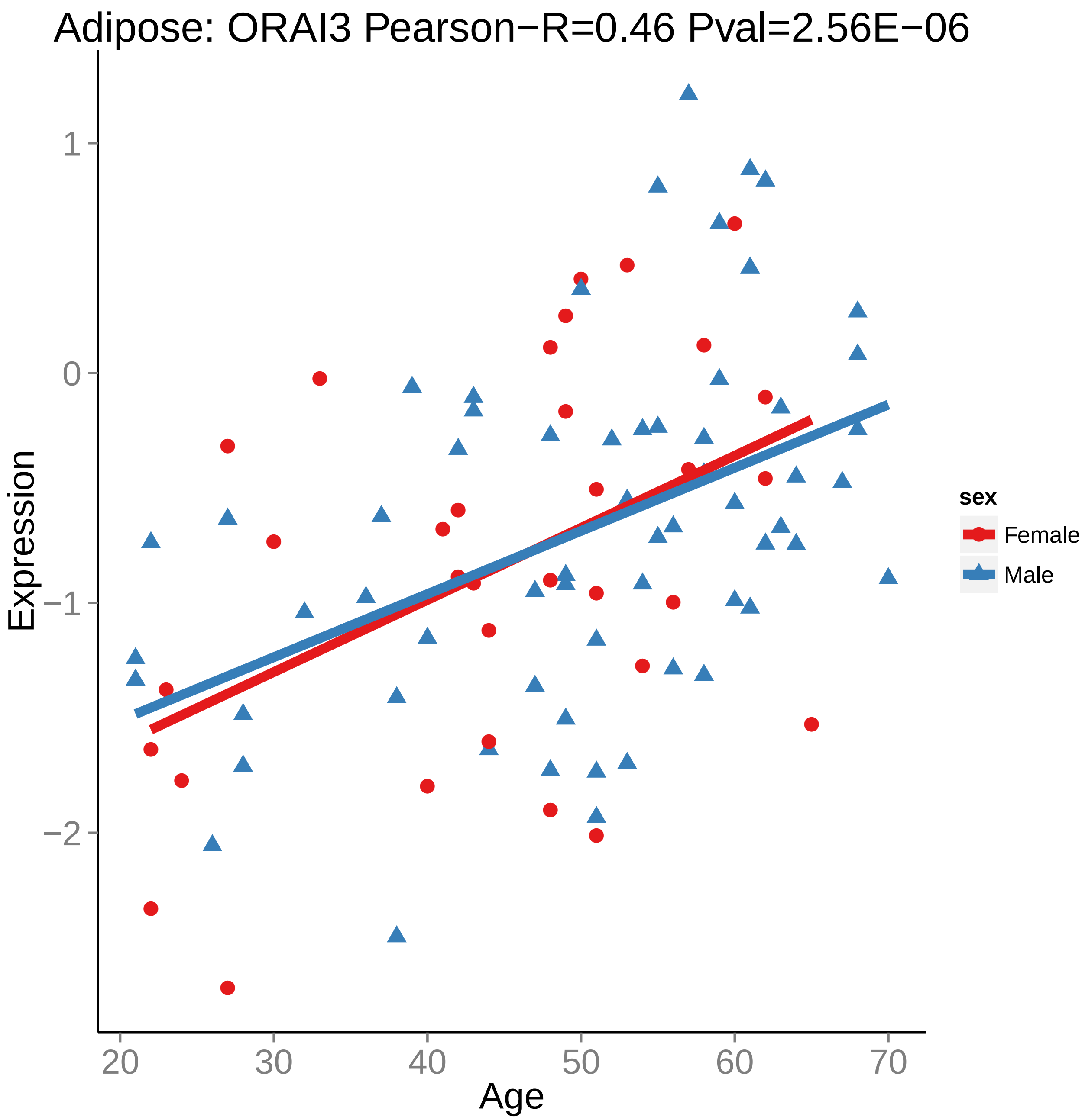

Adipose: ORAI3 Pearson−R=0.46 Pval=2.56E−06
Expression
1
0
−1
−2
20
30
40
50
60
70
Age
sex
Female
Male

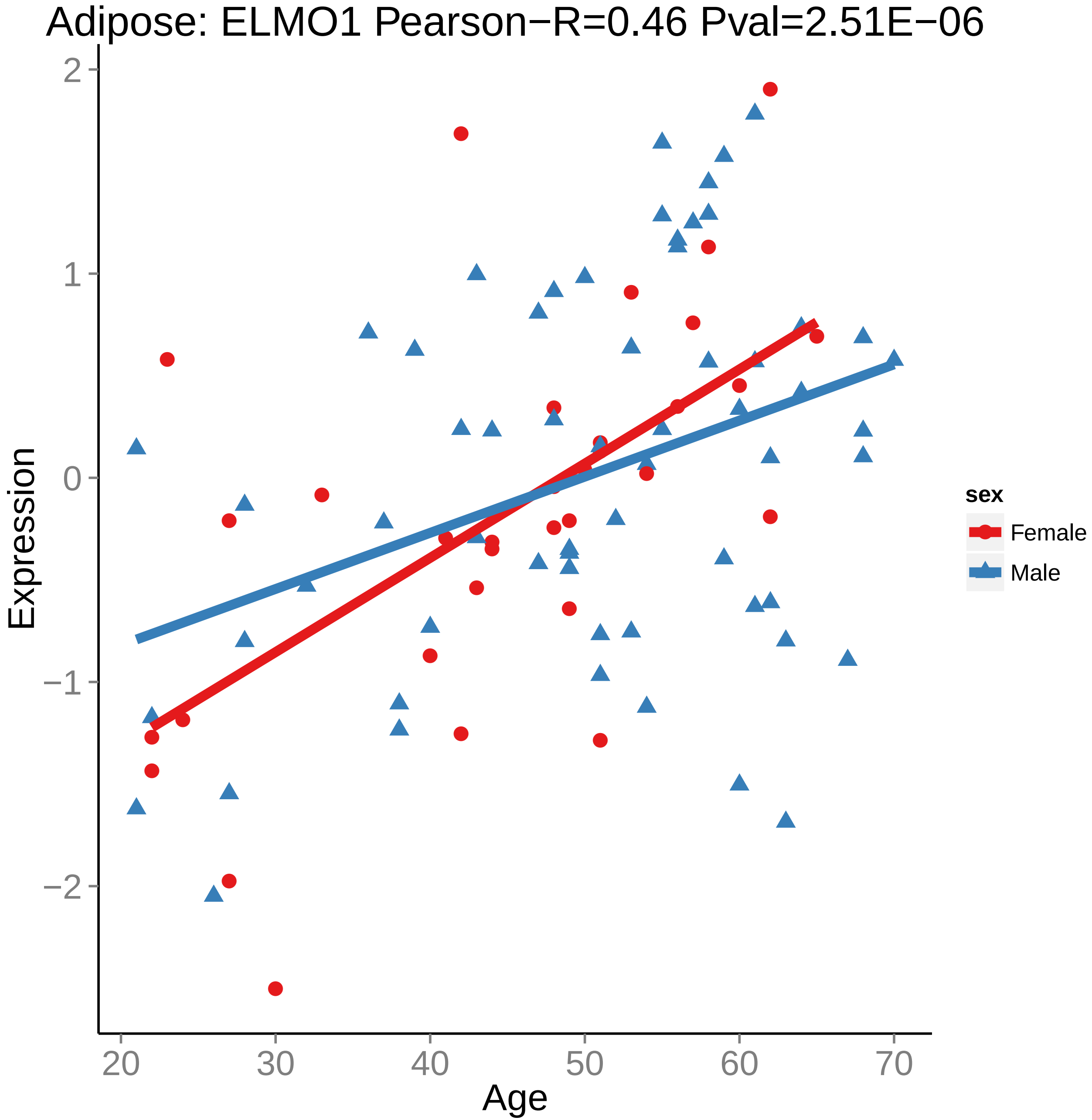
Adipose: ELMO1 Pearson−R=0.46 Pval=2.51E−06
2
1
0
−1
−2
Expression
20
30
40
50
60
70
Age
sex
Female
Male

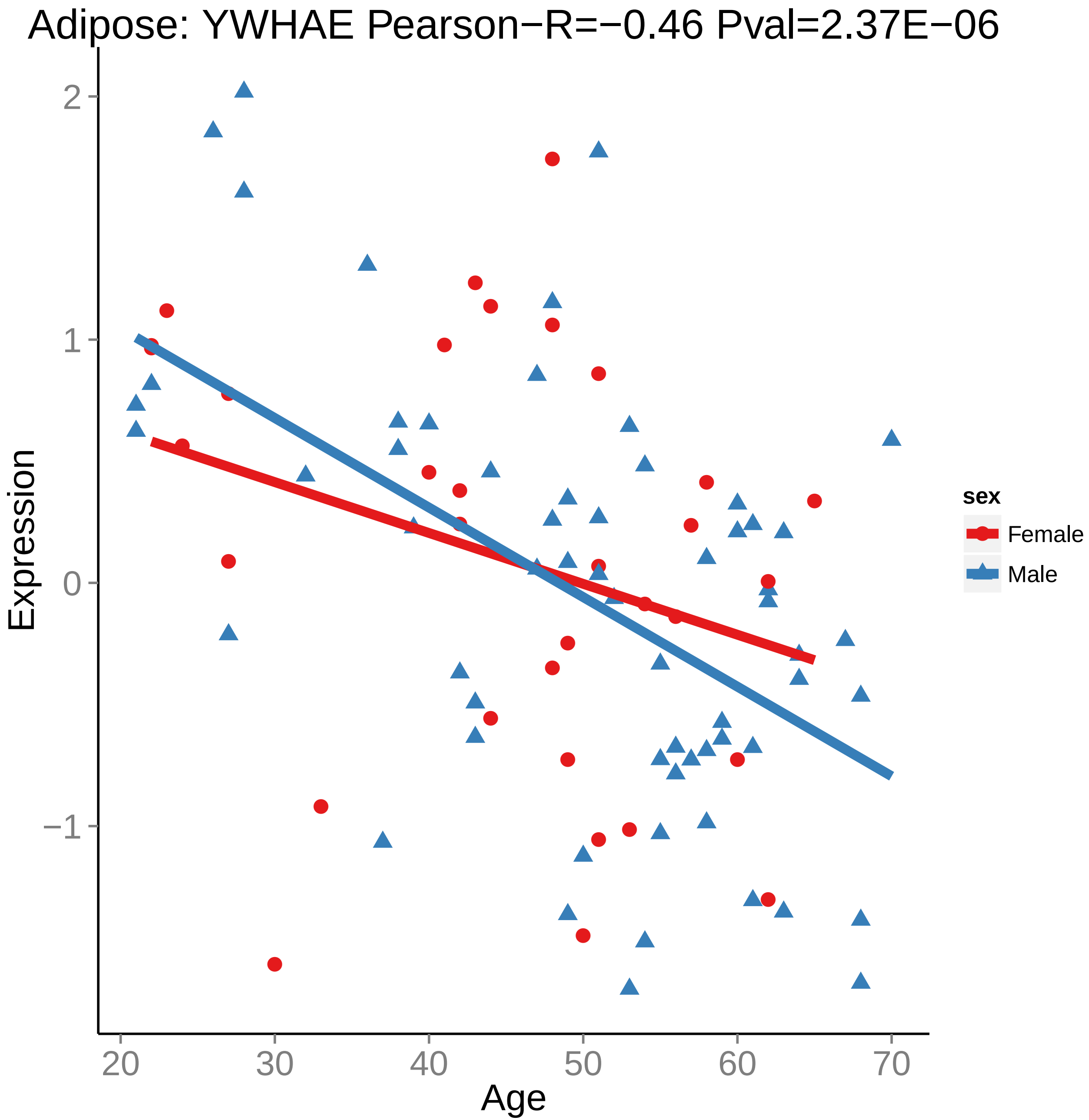

Adipose: YWHAE Pearson−R=−0.46 Pval=2.37E−06
Expression
Age
2
1
0
−1
20
30
40
50
60
70
sex
Female
Male

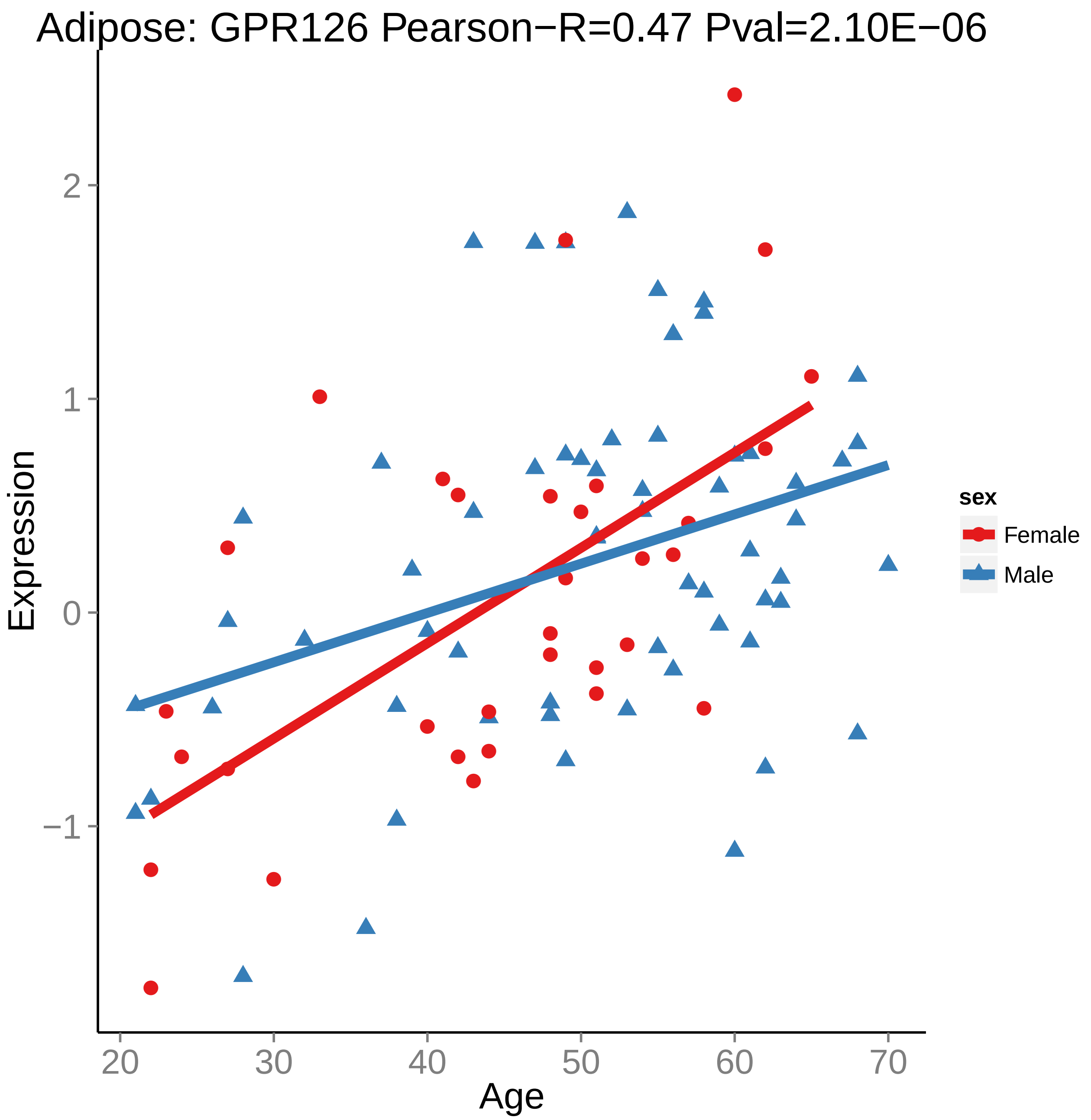

Adipose: GPR126 Pearson−R=0.47 Pval=2.10E−06
Expression
Age
2
1
0
−1
20
30
40
50
60
70
sex
Female
Male

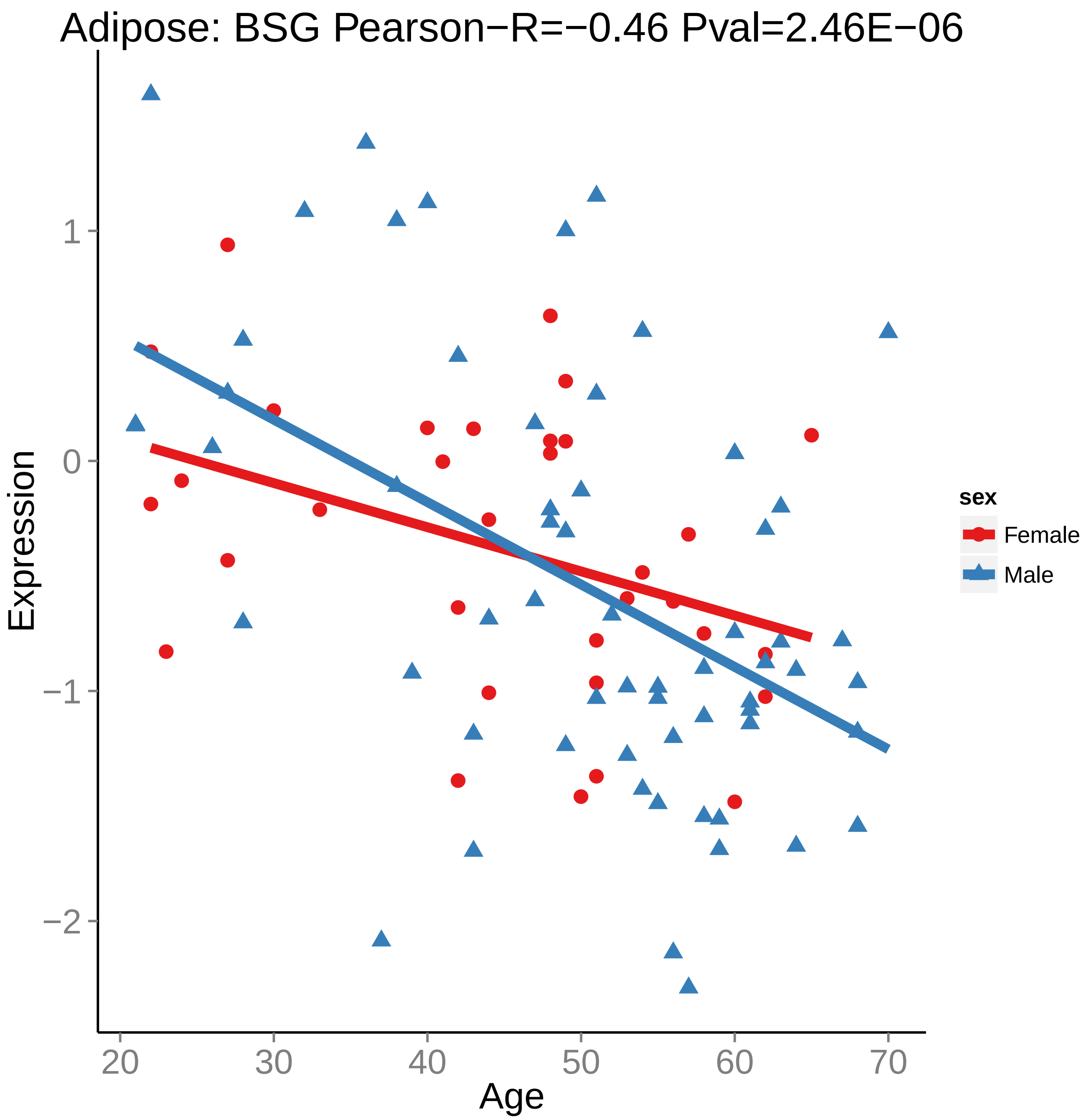
Adipose: BSG Pearson−R=−0.46 Pval=2.46E−06
Expression
Age
1
0
−1
−2
20
30
40
50
60
70
sex
Female
Male

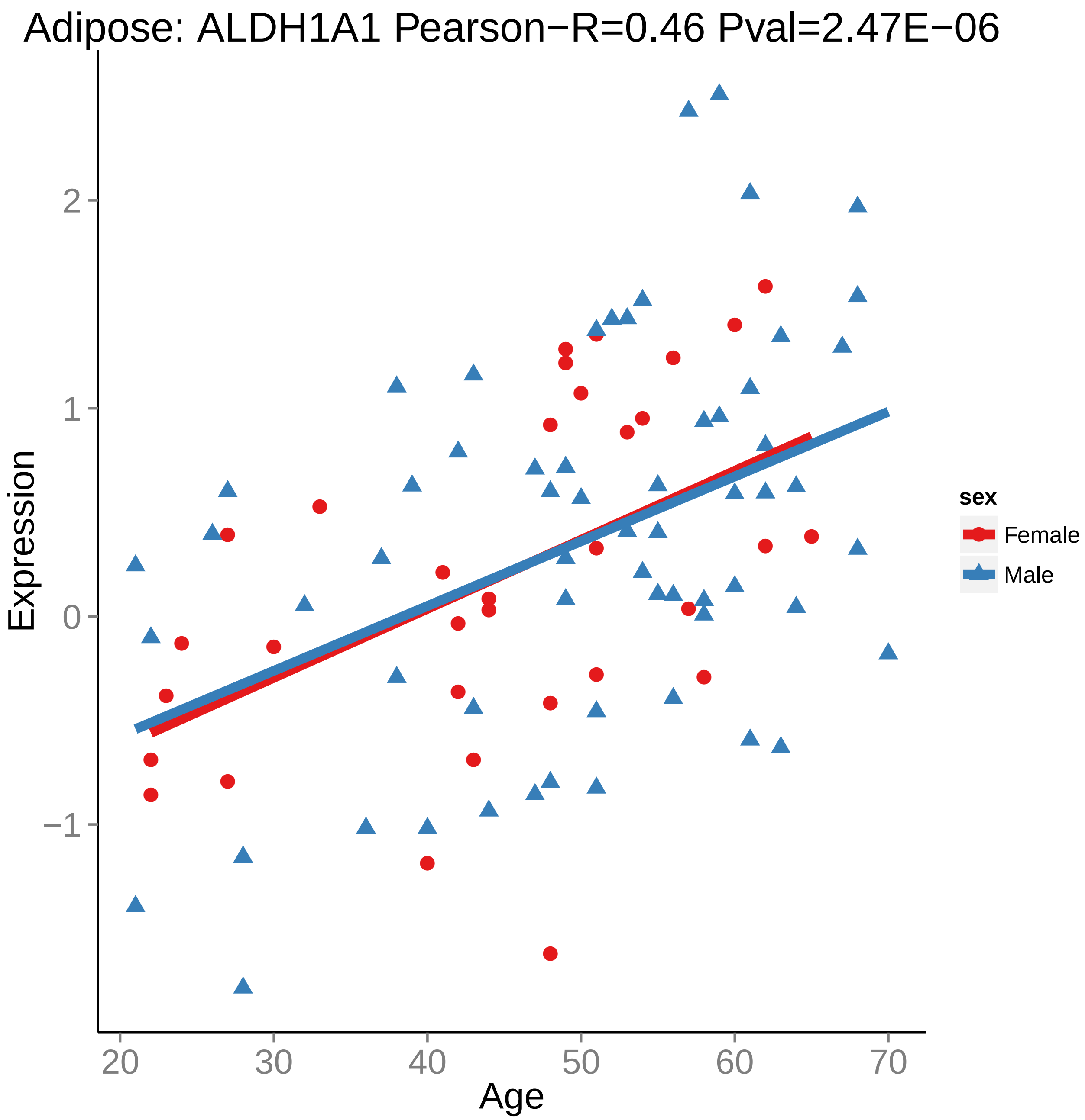
Adipose: ALDH1A1 Pearson−R=0.46 Pval=2.47E−06
Expression
Age
2
1
0
−1
20
30
40
50
60
70
sex
Female
Male

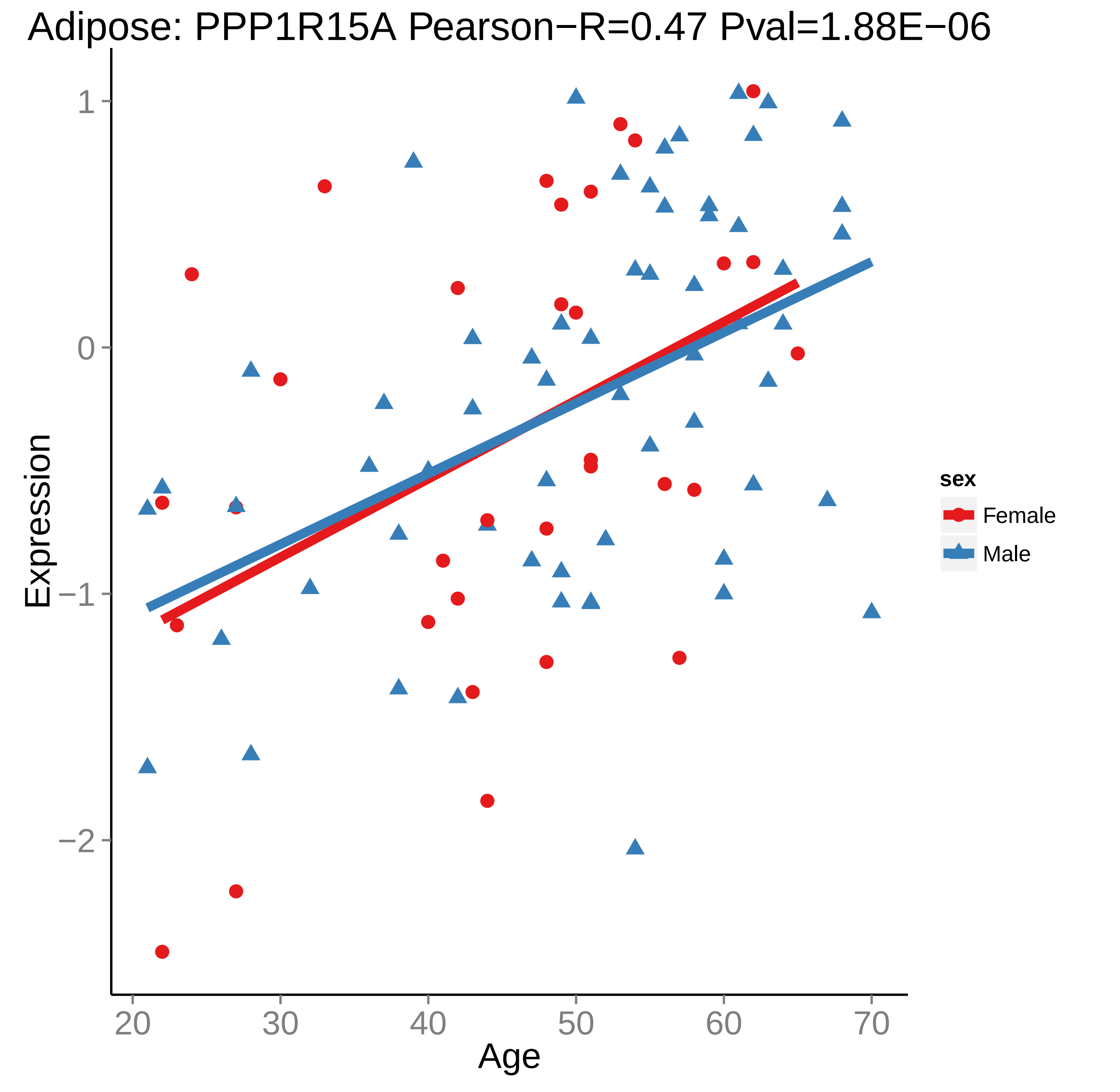
Adipose: PPP1R15A Pearson−R=0.47 Pval=1.88E−06
Expression
1
0
−1
−2
Age
20
30
40
50
60
70
sex
Female
Male

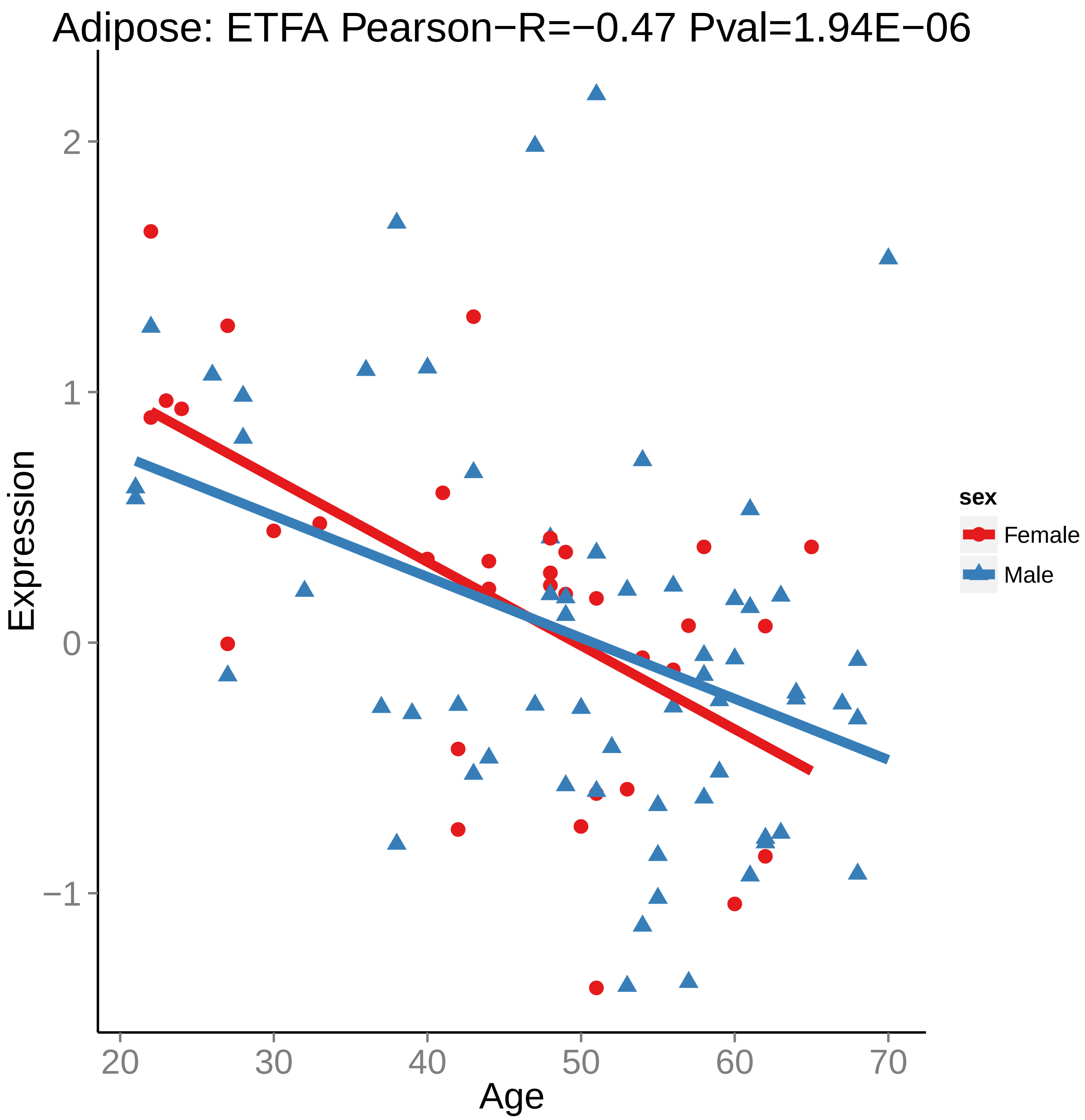

Adipose: ETFA Pearson−R=−0.47 Pval=1.94E−06
Expression
Age
2
1
0
−1
20
30
40
50
60
70
sex
Female
Male

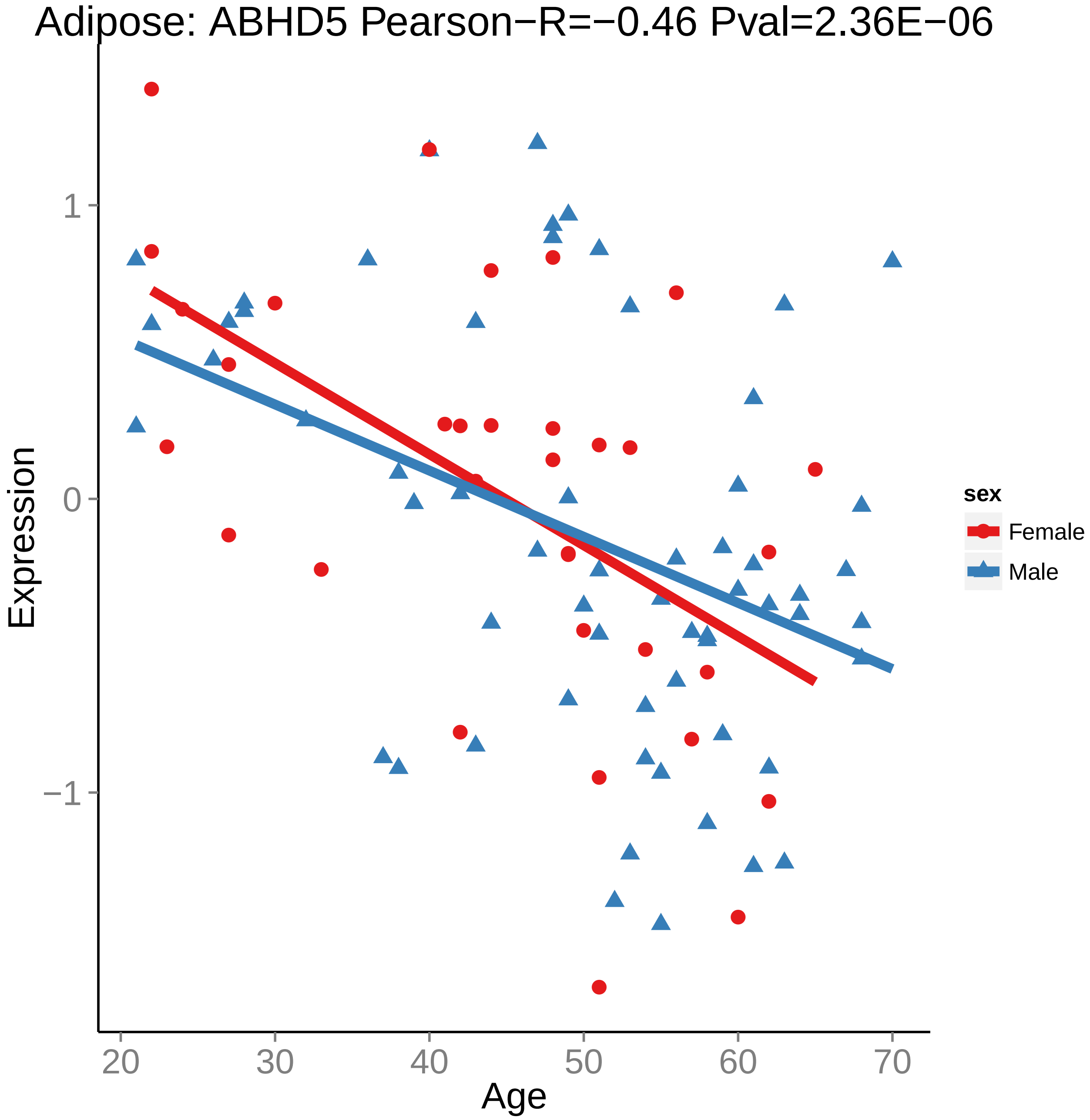

Adipose: ABHD5 Pearson−R=−0.46 Pval=2.36E−06
Expression
1
0
−1
20
30
40
50
60
70
Age
sex
Female
Male

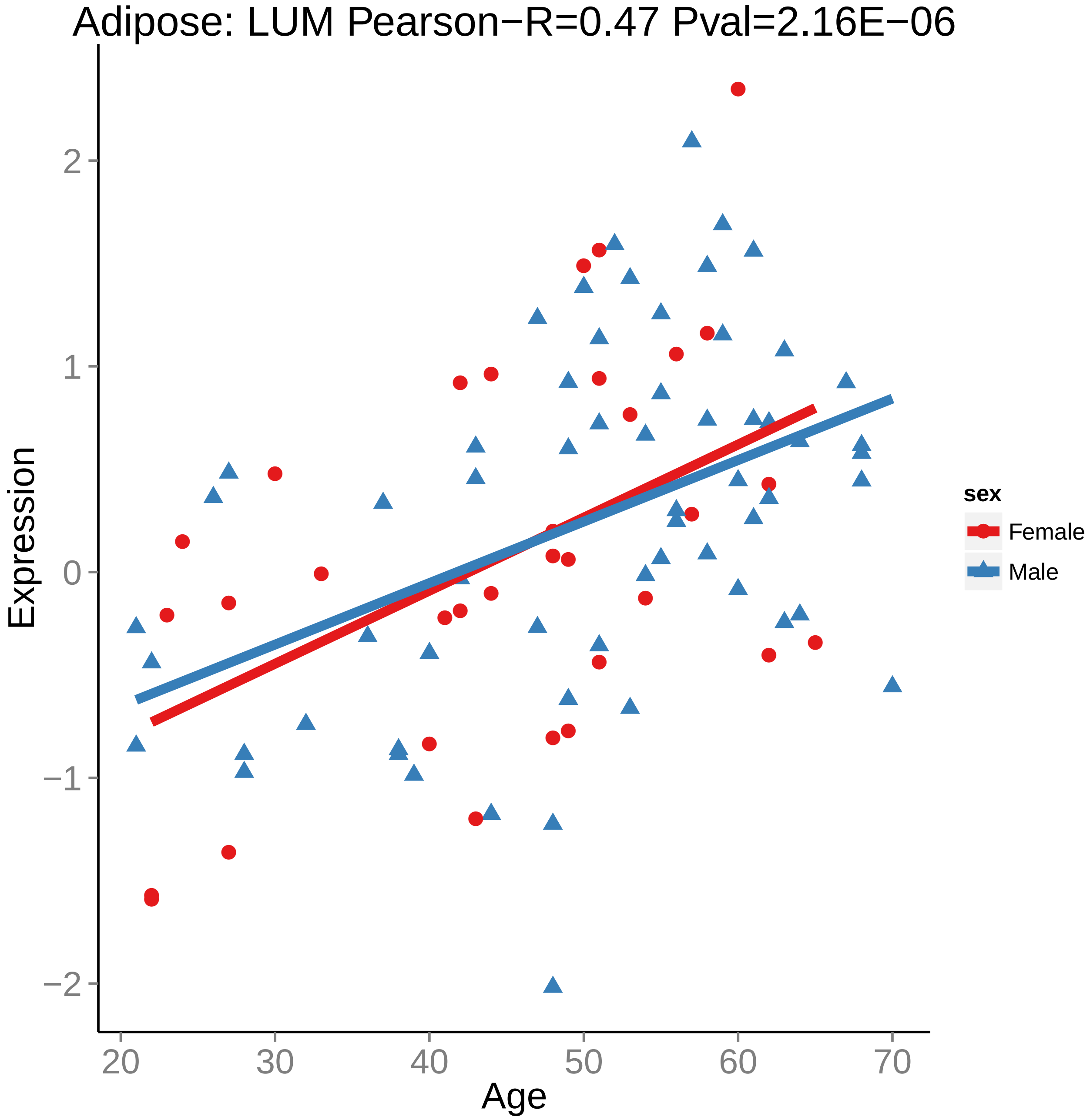

Adipose: LUM Pearson−R=0.47 Pval=2.16E−06
Expression
2
1
0
−1
−2
20
30
40
50
60
70
Age
sex
Female
Male

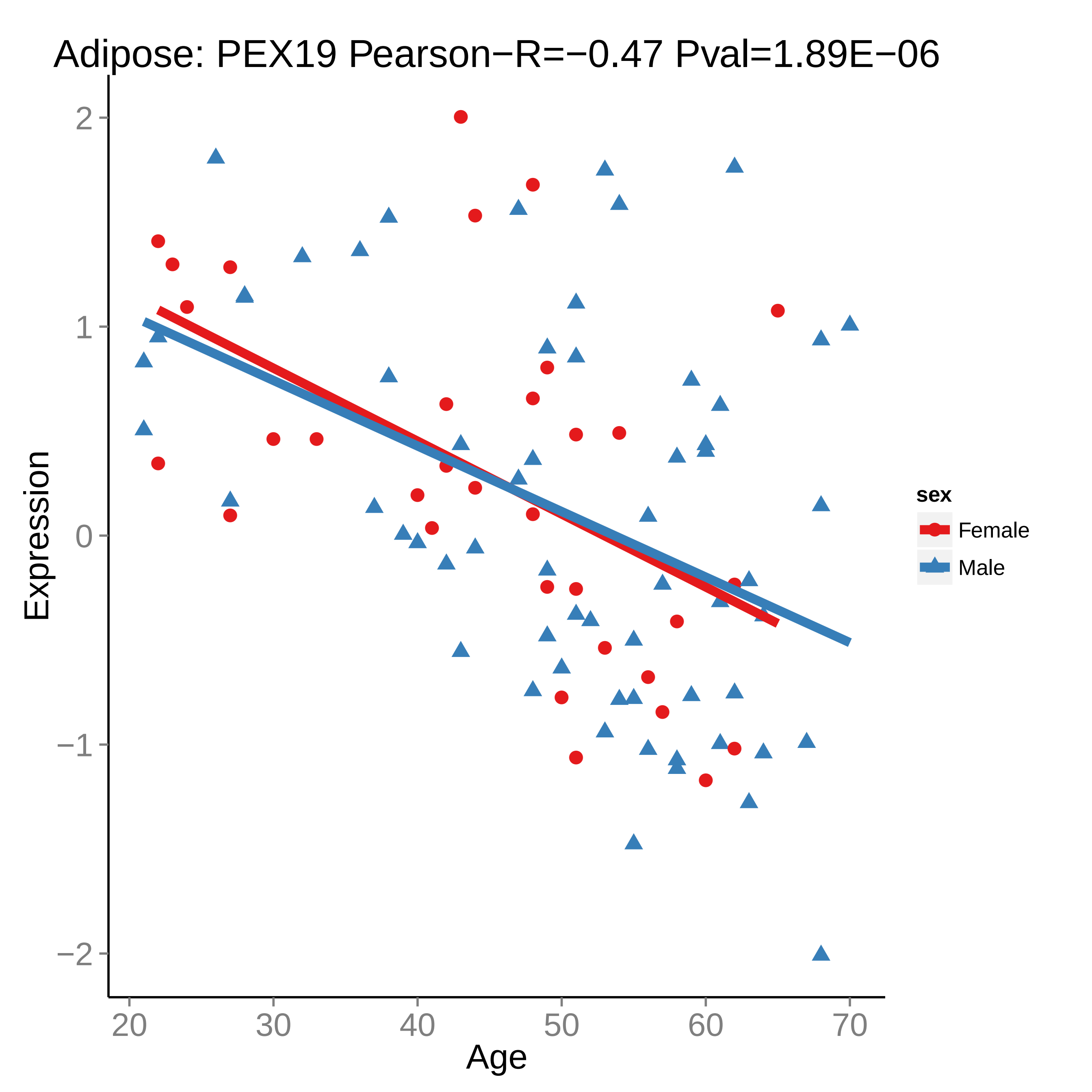

Adipose: PEX19 Pearson−R=−0.47 Pval=1.89E−06
Expression
2
1
0
−1
−2
20
30
40
50
60
70
Age
sex
Female
Male

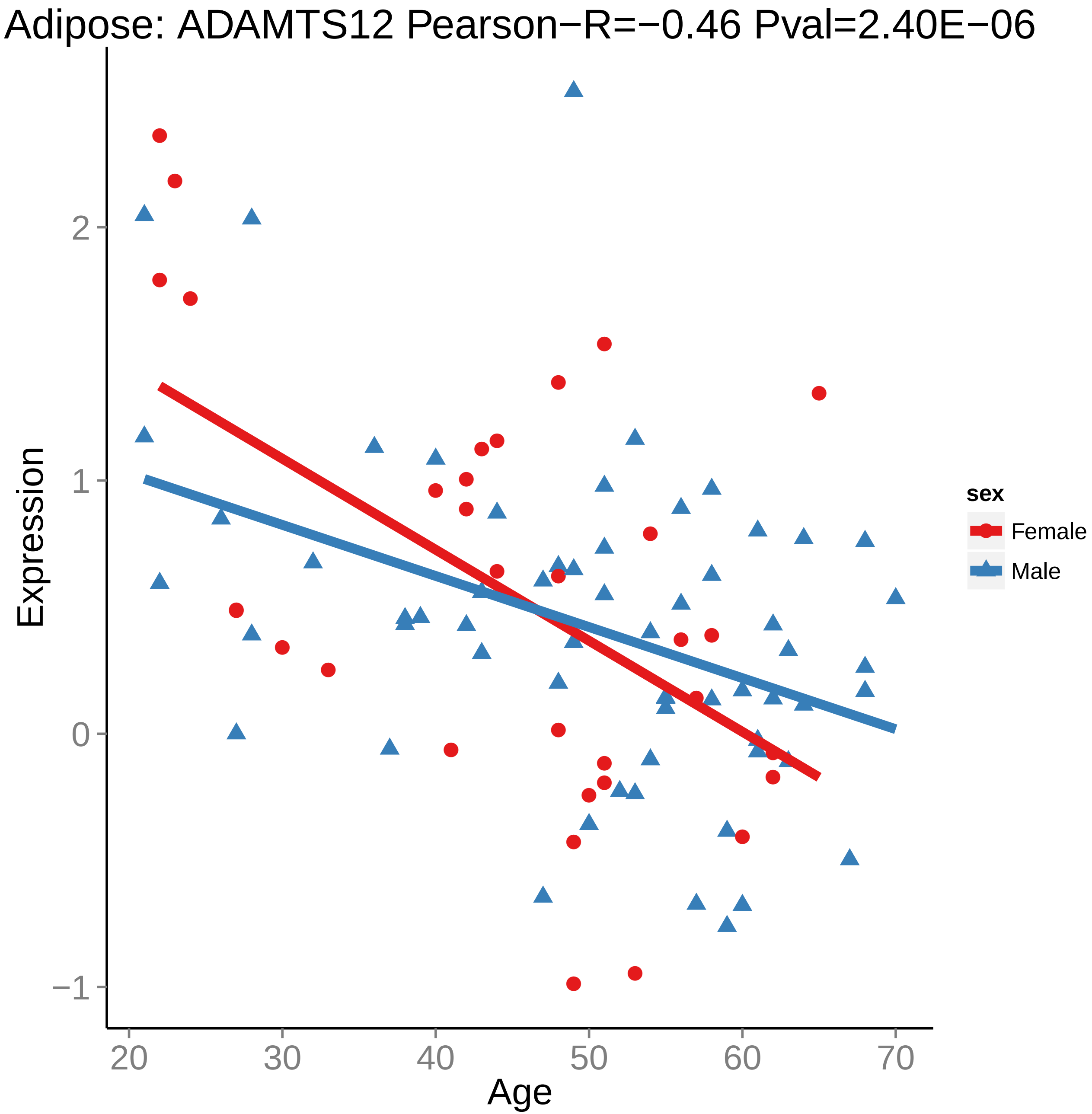

Adipose: ADAMTS12 Pearson−R=−0.46 Pval=2.40E−06
Expression
Age
2
1
0
−1
20
30
40
50
60
70
sex
Female
Male

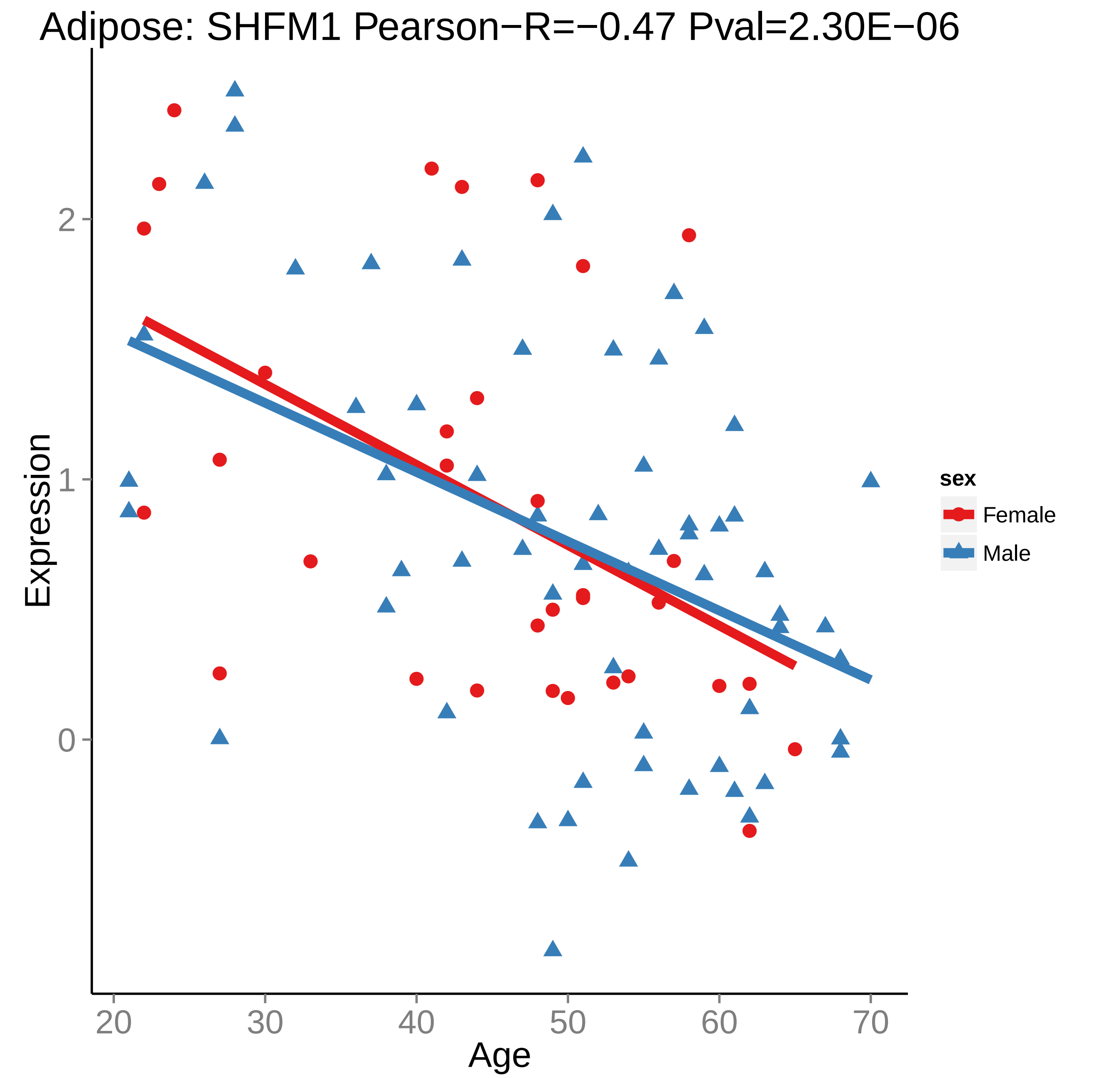

Adipose: SHFM1 Pearson−R=−0.47 Pval=2.30E−06
Expression
2
1
0
20
30
40
50
60
70
Age
sex
Female
Male

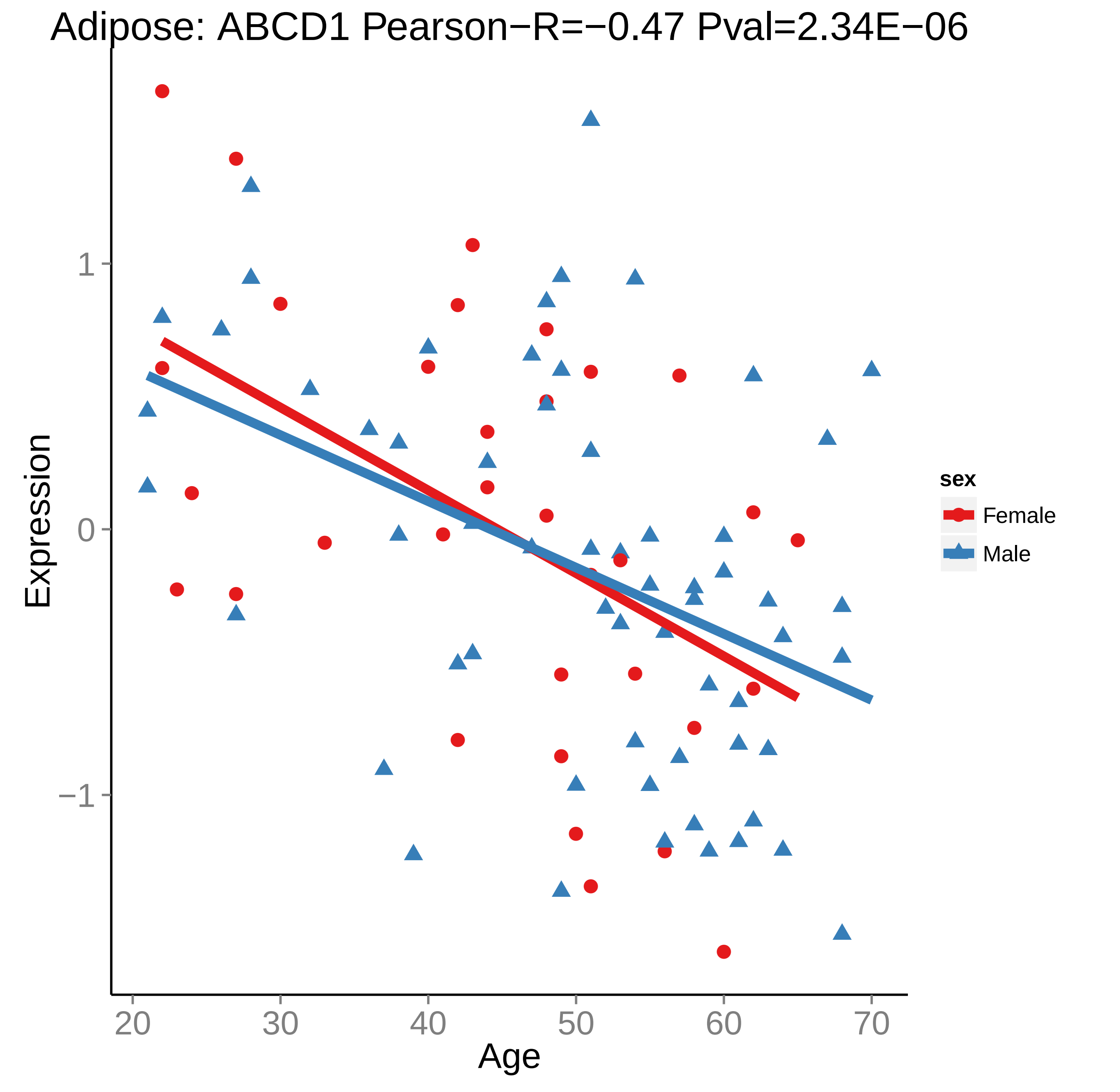
Adipose: ABCD1 Pearson−R=−0.47 Pval=2.34E−06
Expression
1
0
−1
20
30
40
50
60
70
Age
sex
Female
Male

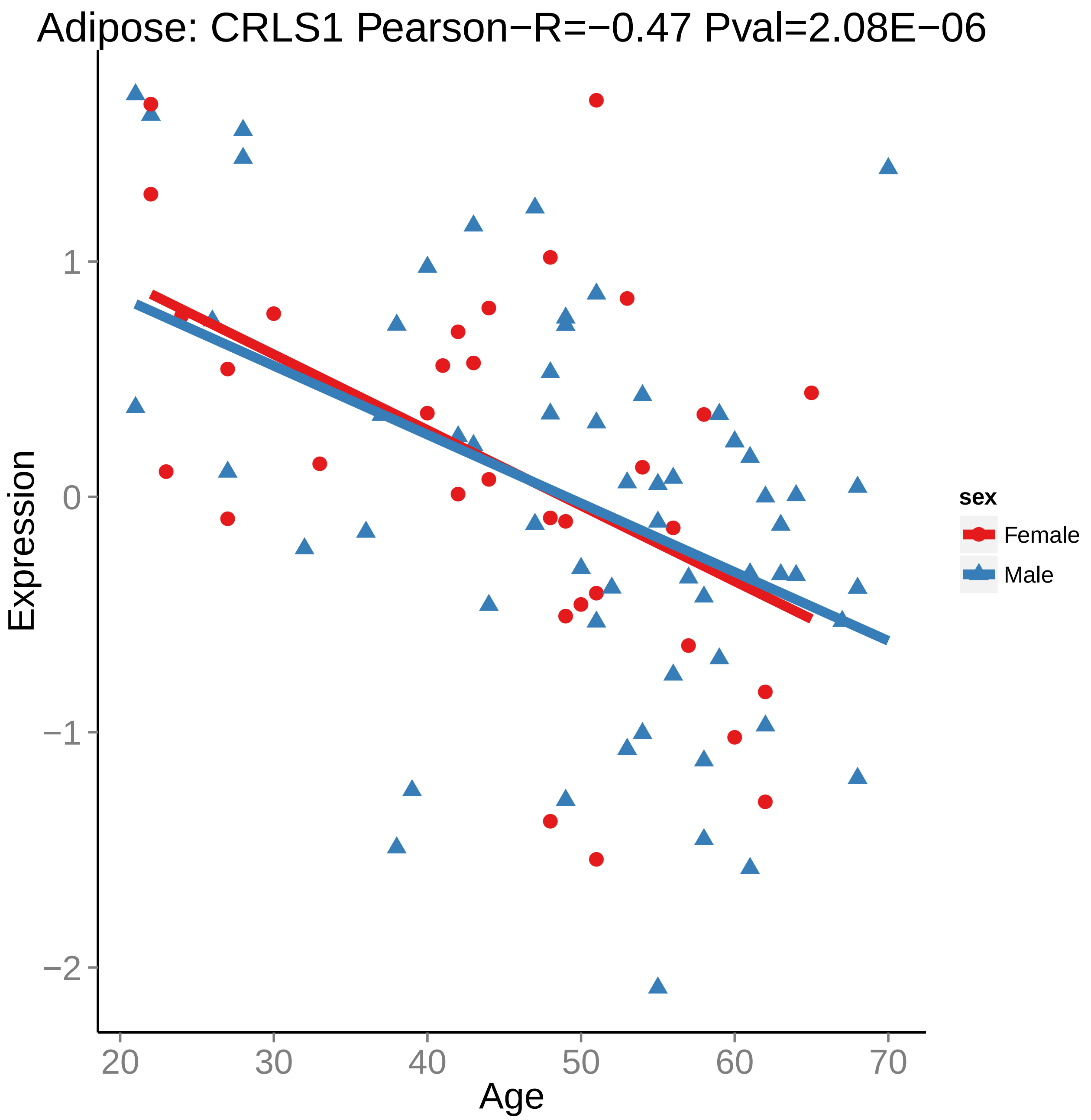

Adipose: CRLS1 Pearson−R=−0.47 Pval=2.08E−06
Expression
Age
1
0
−1
−2
20
30
40
50
60
70
sex
Female
Male

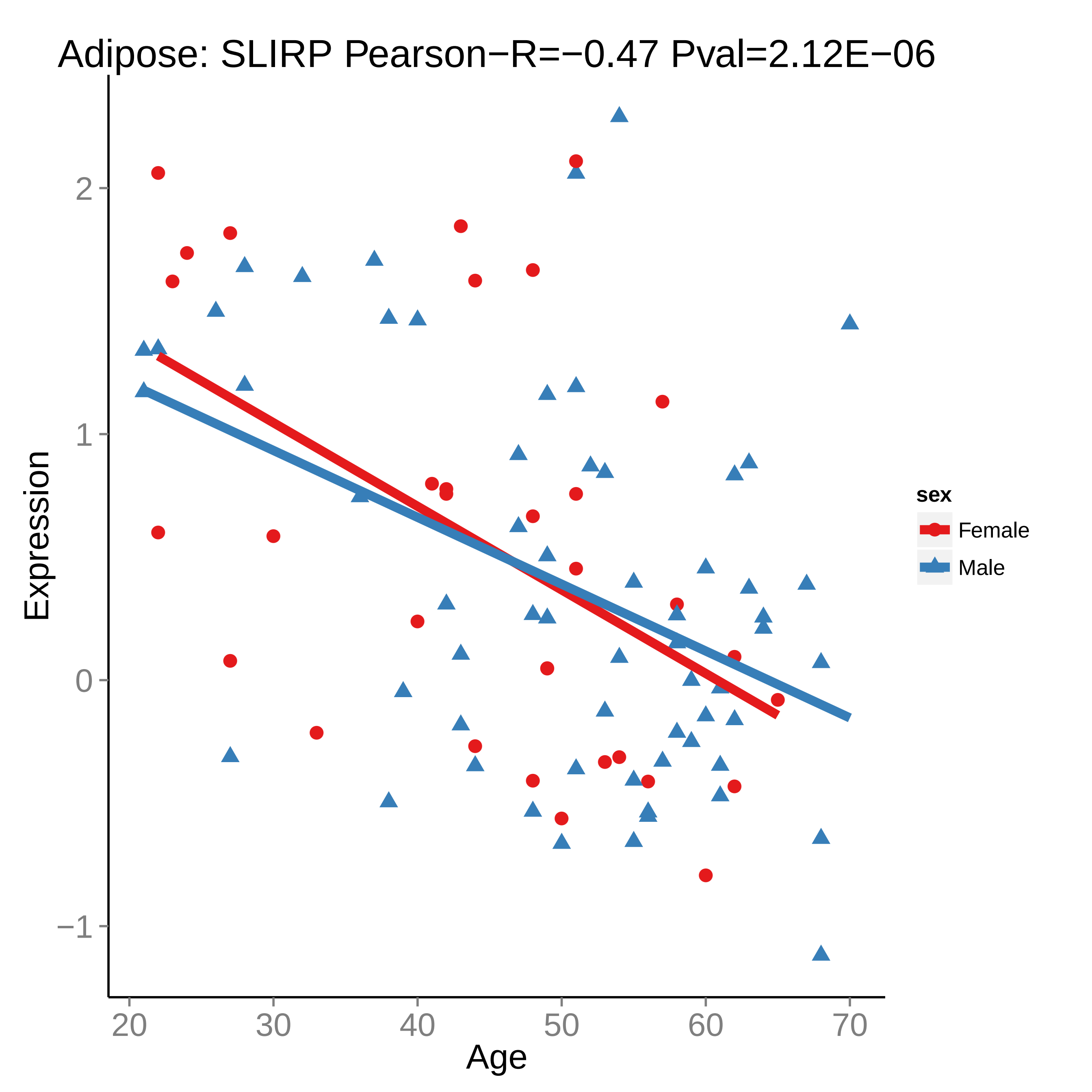
Adipose: SLIRP Pearson−R=−0.47 Pval=2.12E−06
Expression
2
1
0
−1
20
30
40
50
60
70
Age
sex
Female
Male

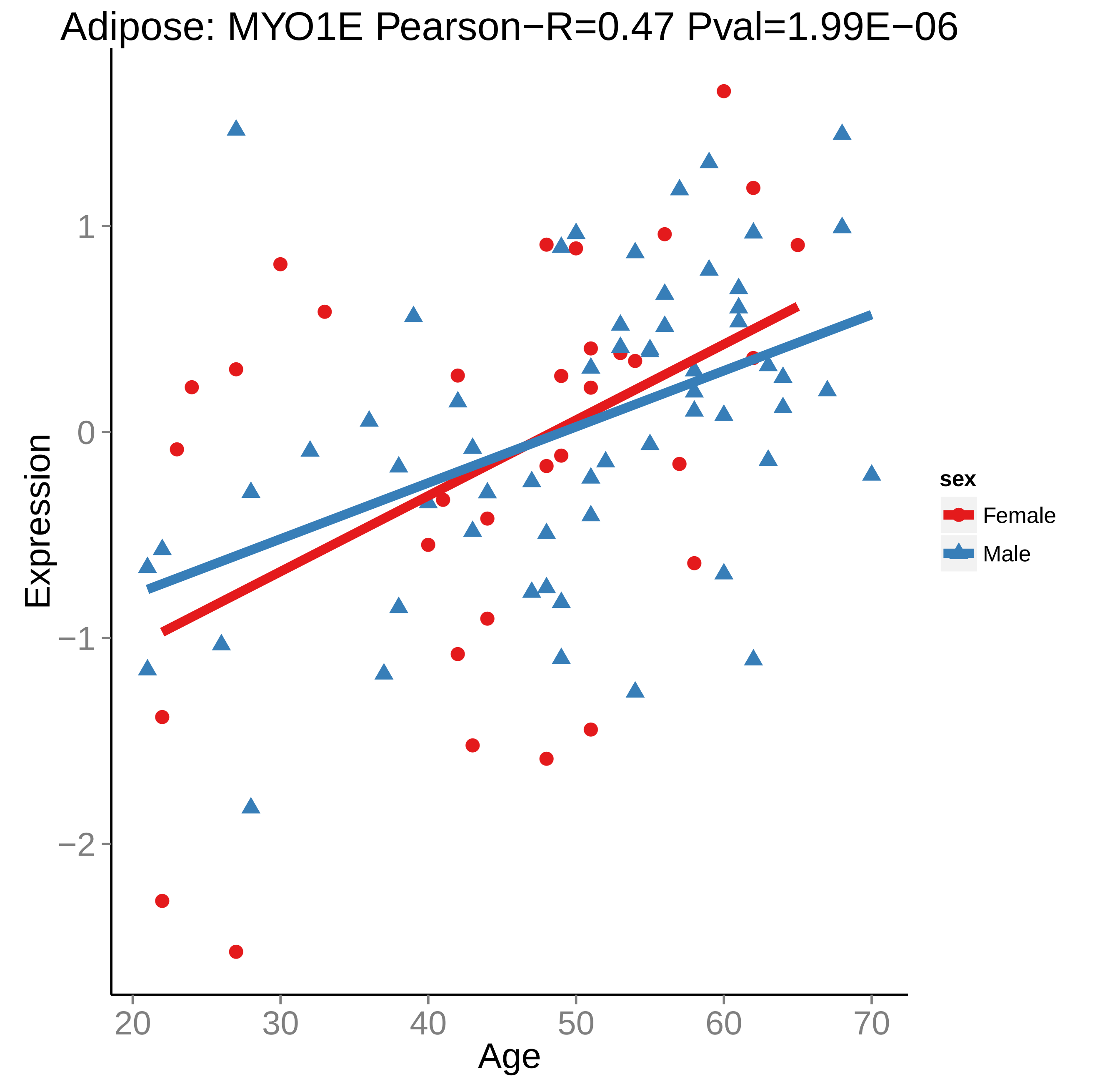

Adipose: MYO1E Pearson−R=0.47 Pval=1.99E−06
Expression
1
0
−1
−2
20
30
40
50
60
70
Age
sex
Female
Male

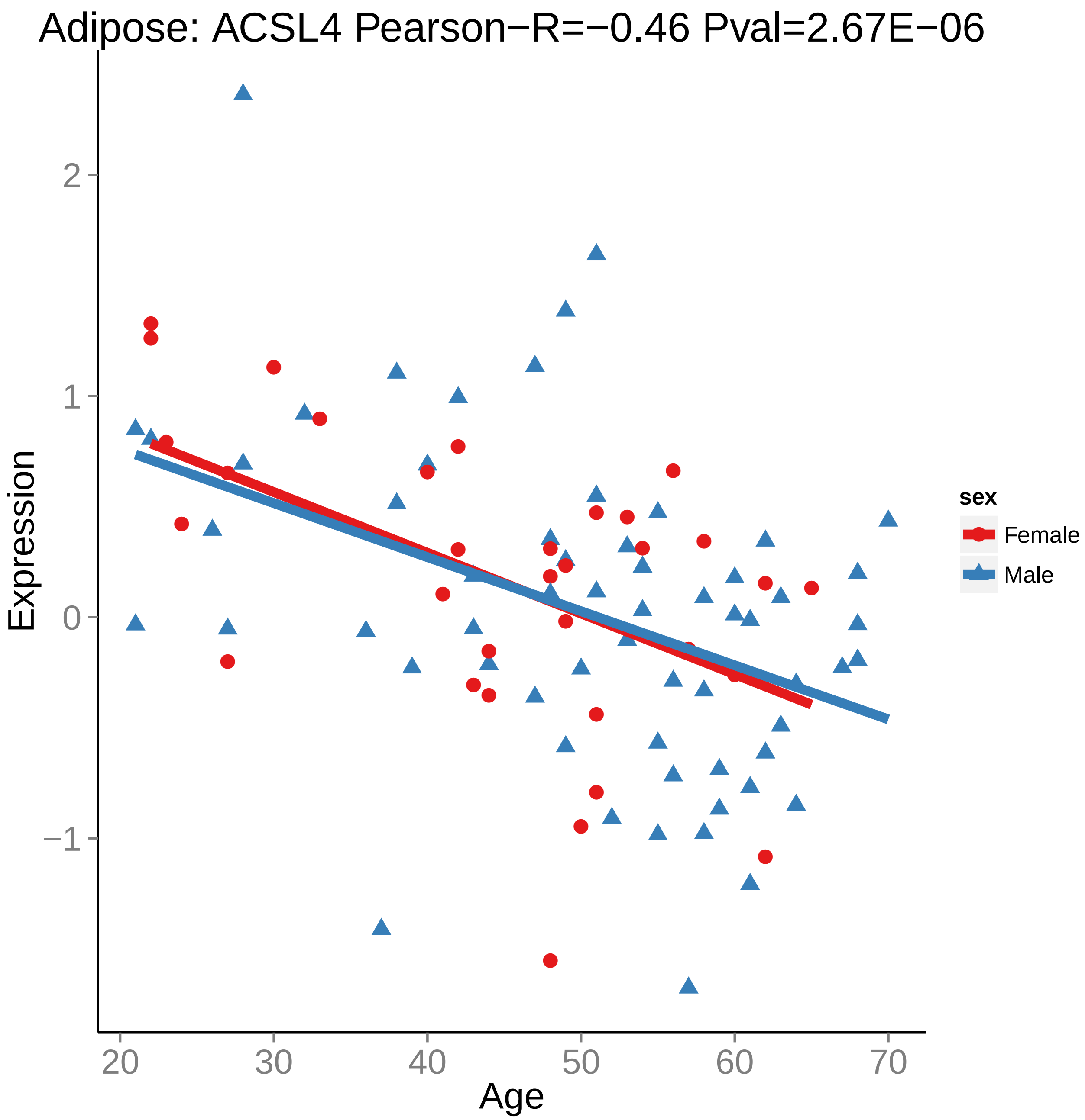
Adipose: ACSL4 Pearson−R=−0.46 Pval=2.67E−06
Expression
2
1
0
−1
20
30
40
50
60
70
Age
sex
Female
Male

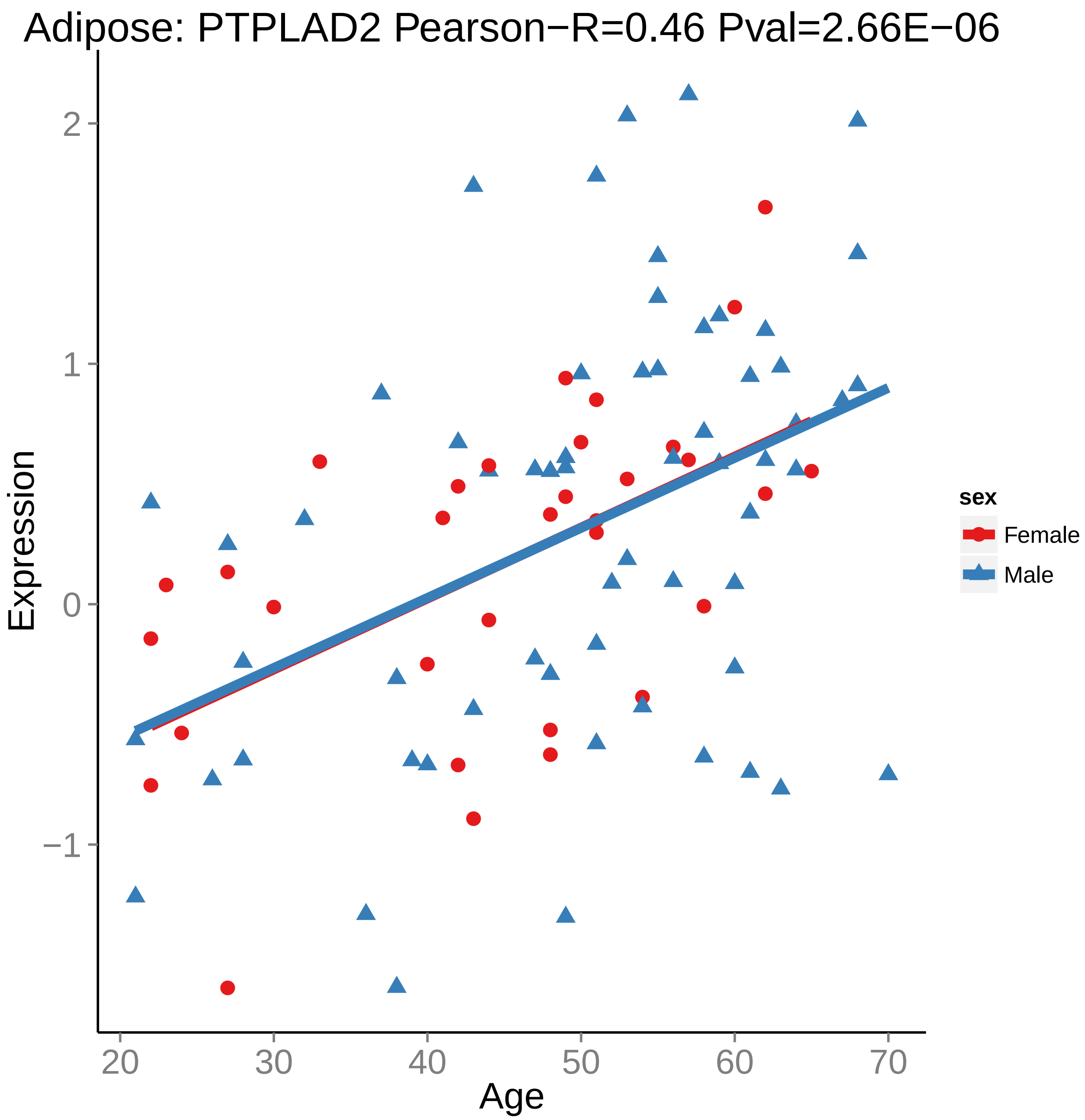

Adipose: PTPLAD2 Pearson−R=0.46 Pval=2.66E−06
Expression
Age
2
1
0
−1
20
30
40
50
60
70
sex
Female
Male

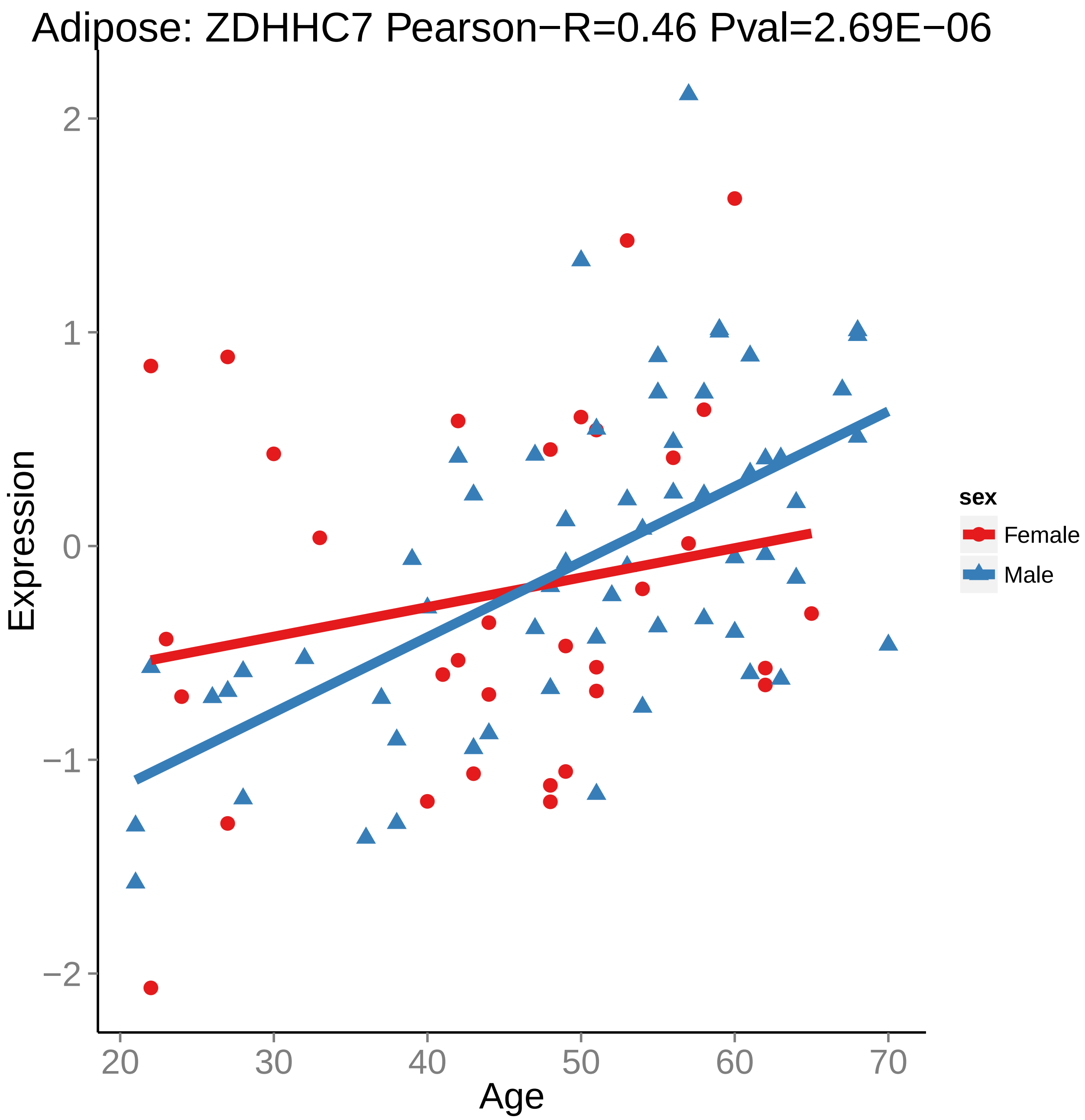

Adipose: ZDHHC7 Pearson−R=0.46 Pval=2.69E−06
Expression
Age
2
1
0
−1
−2
20
30
40
50
60
70
sex
Female
Male

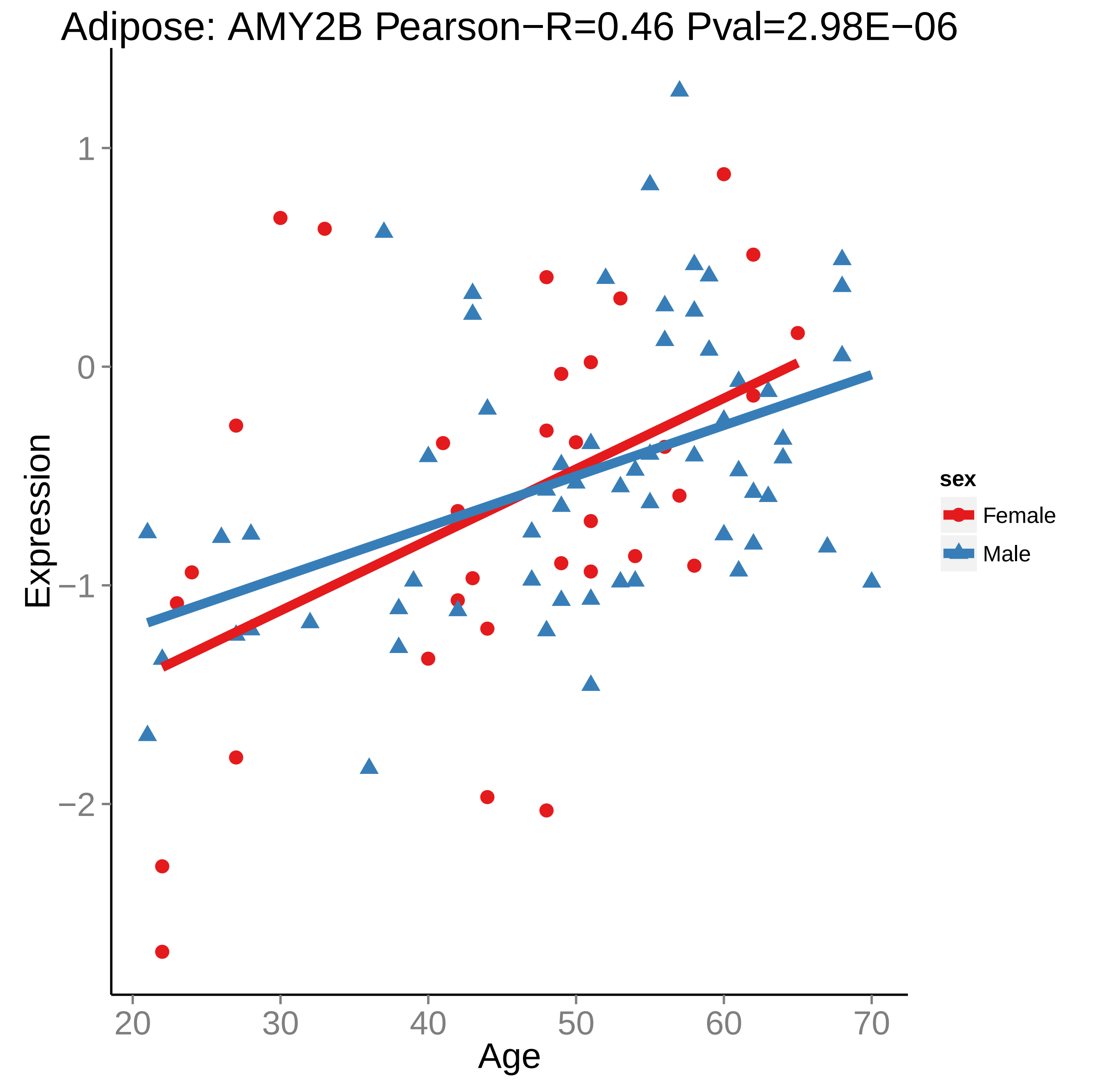

Adipose: AMY2B Pearson−R=0.46 Pval=2.98E−06
Expression
1
0
−1
−2
20
30
40
50
60
70
Age
sex
Female
Male

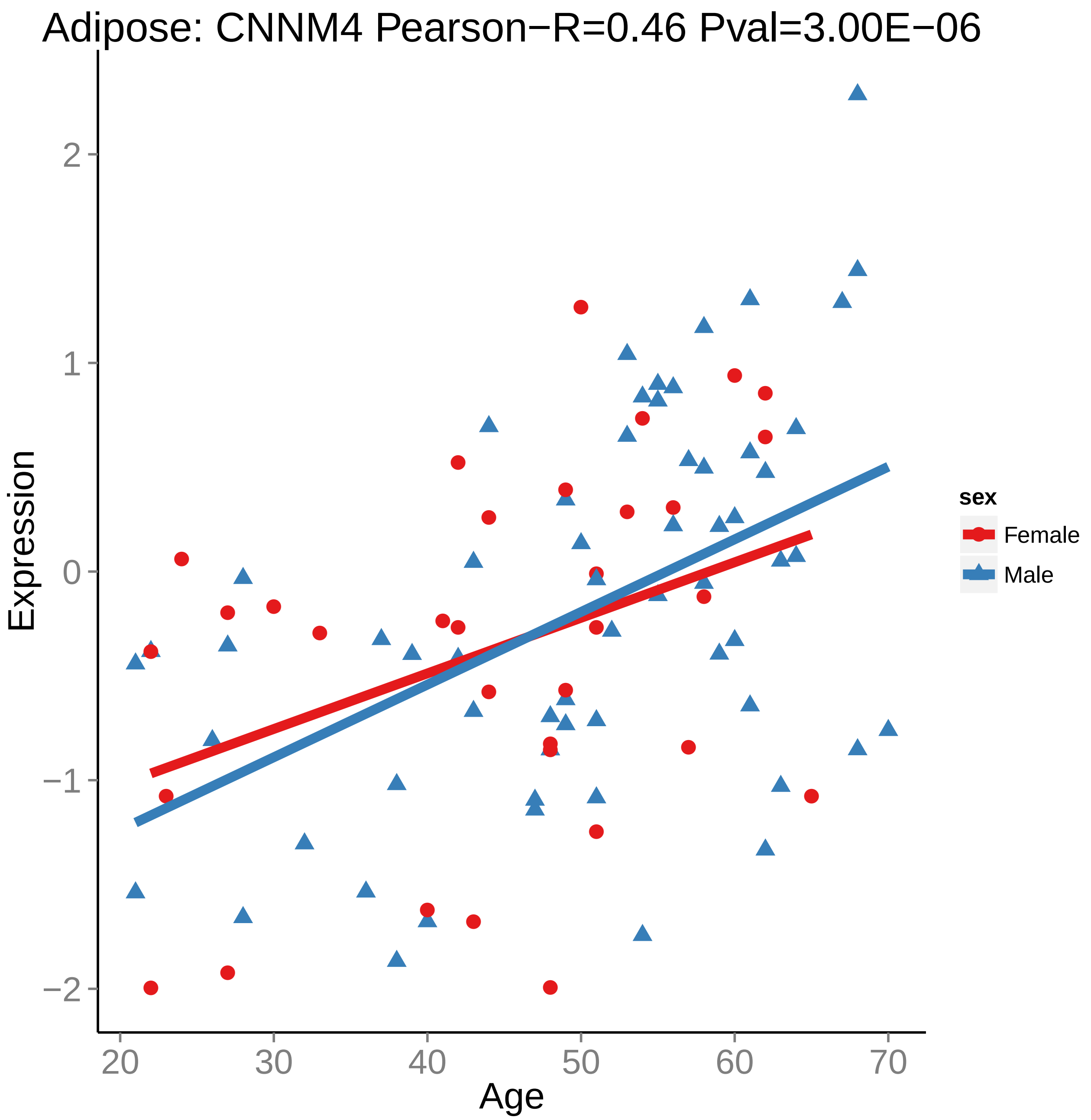
Adipose: CNNM4 Pearson−R=0.46 Pval=3.00E−06
Expression
Age
sex
Female
Male
2
1
0
−1
−2
20
30
40
50
60
70

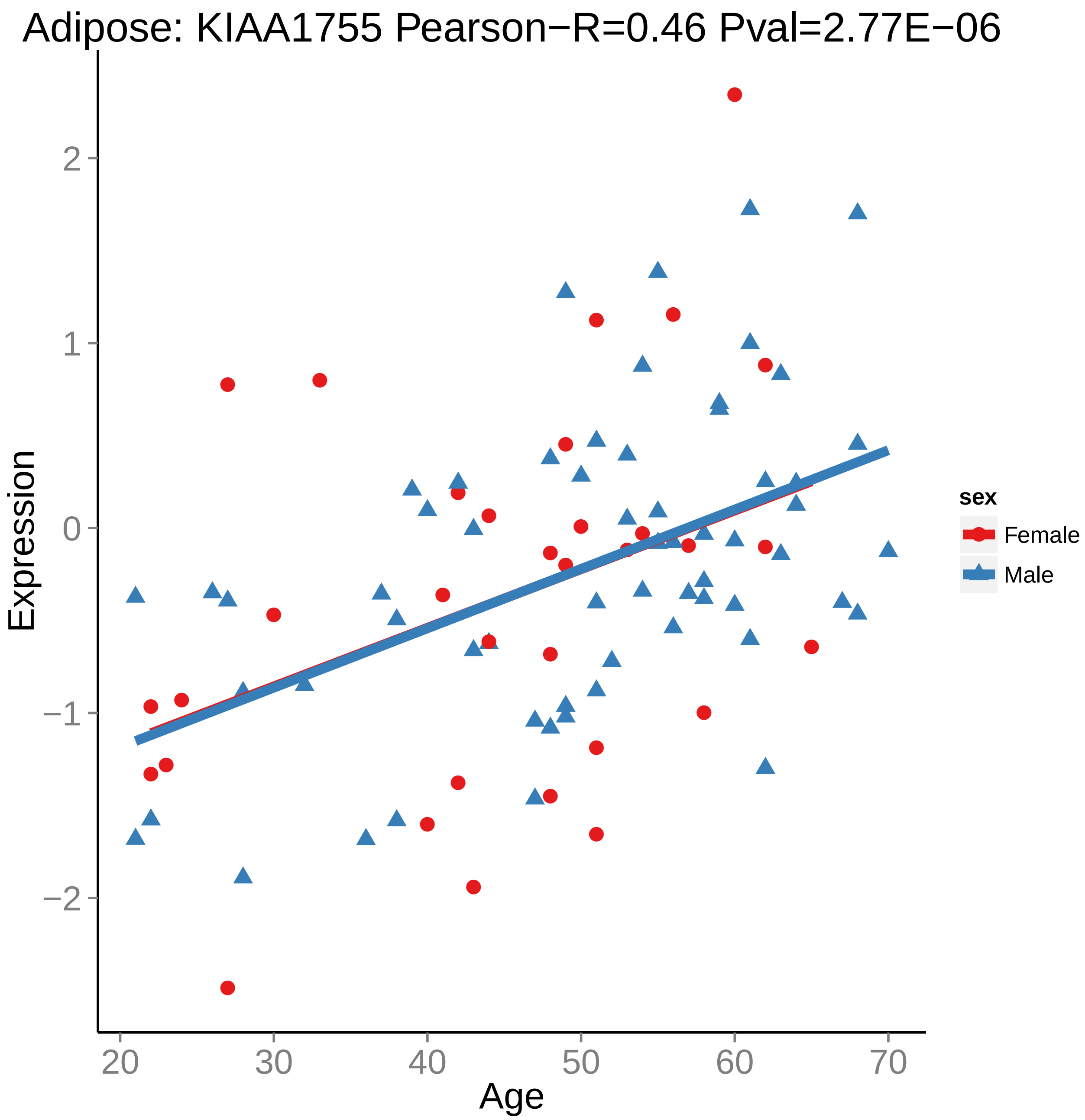

Adipose: KIAA1755 Pearson−R=0.46 Pval=2.77E−06
Expression
2
1
0
−1
−2
20
30
40
50
60
70
Age
sex
Female
Male

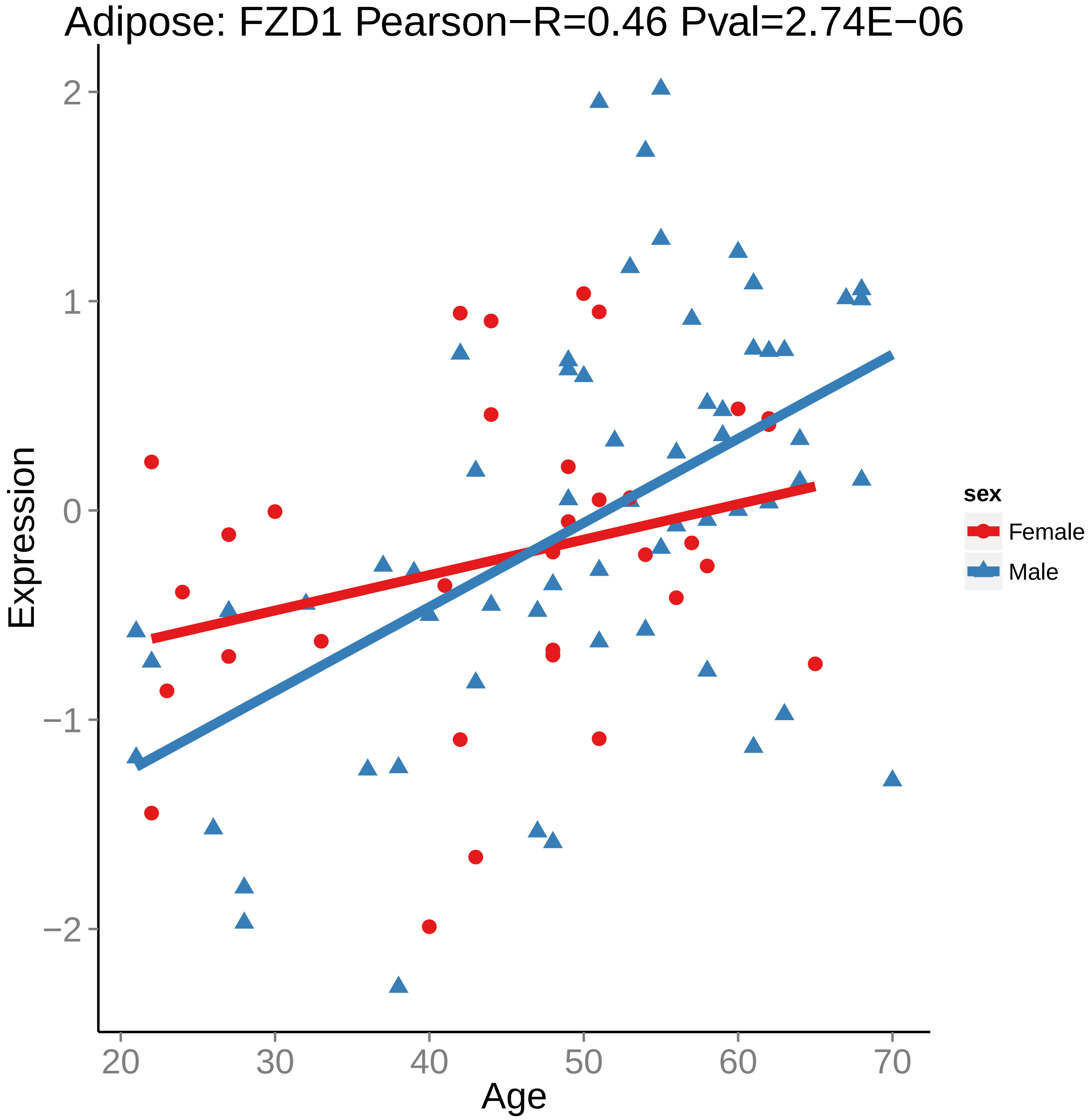

Adipose: FZD1 Pearson−R=0.46 Pval=2.74E−06
Expression
Age
2
1
0
−1
−2
20
30
40
50
60
70
sex
Female
Male

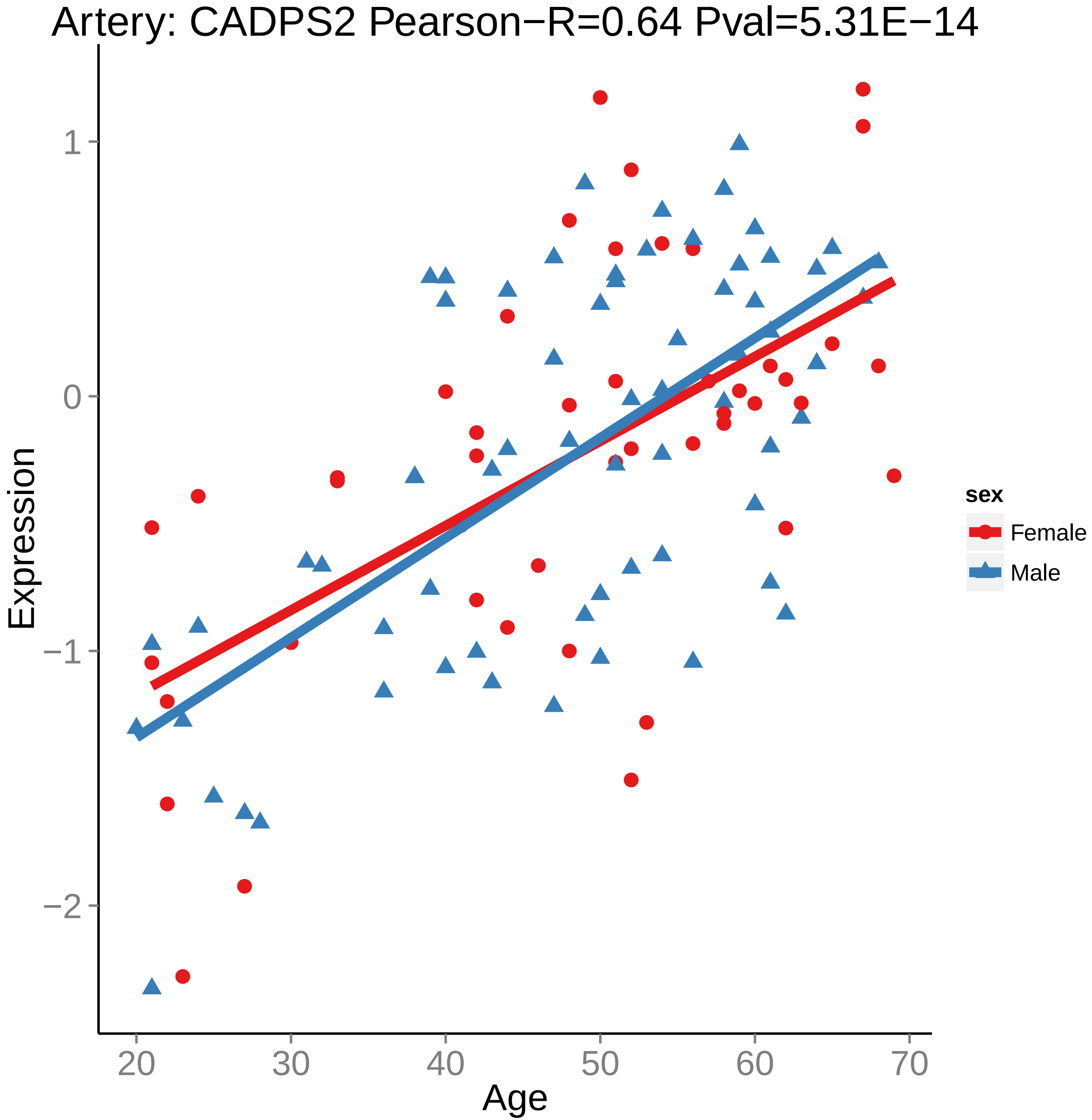

Artery: CADPS2 Pearson−R=0.64 Pval=5.31E−14
Expression
Age
1
0
−1
−2
20
30
40
50
60
70
sex
Female
Male

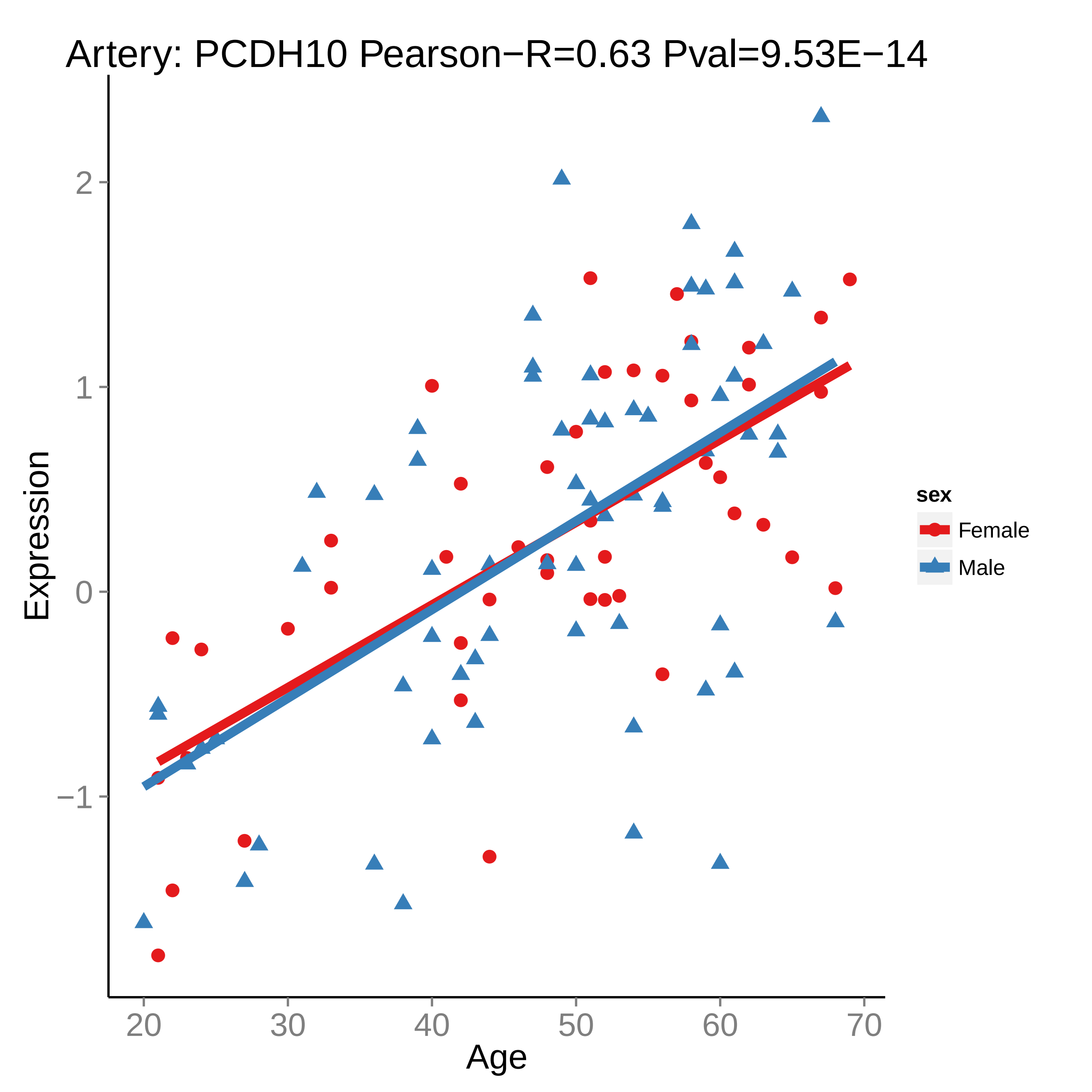
Artery: PCDH10 Pearson−R=0.63 Pval=9.53E−14
Expression
2
1
0
−1
20
30
40
50
60
70
Age
sex
Female
Male

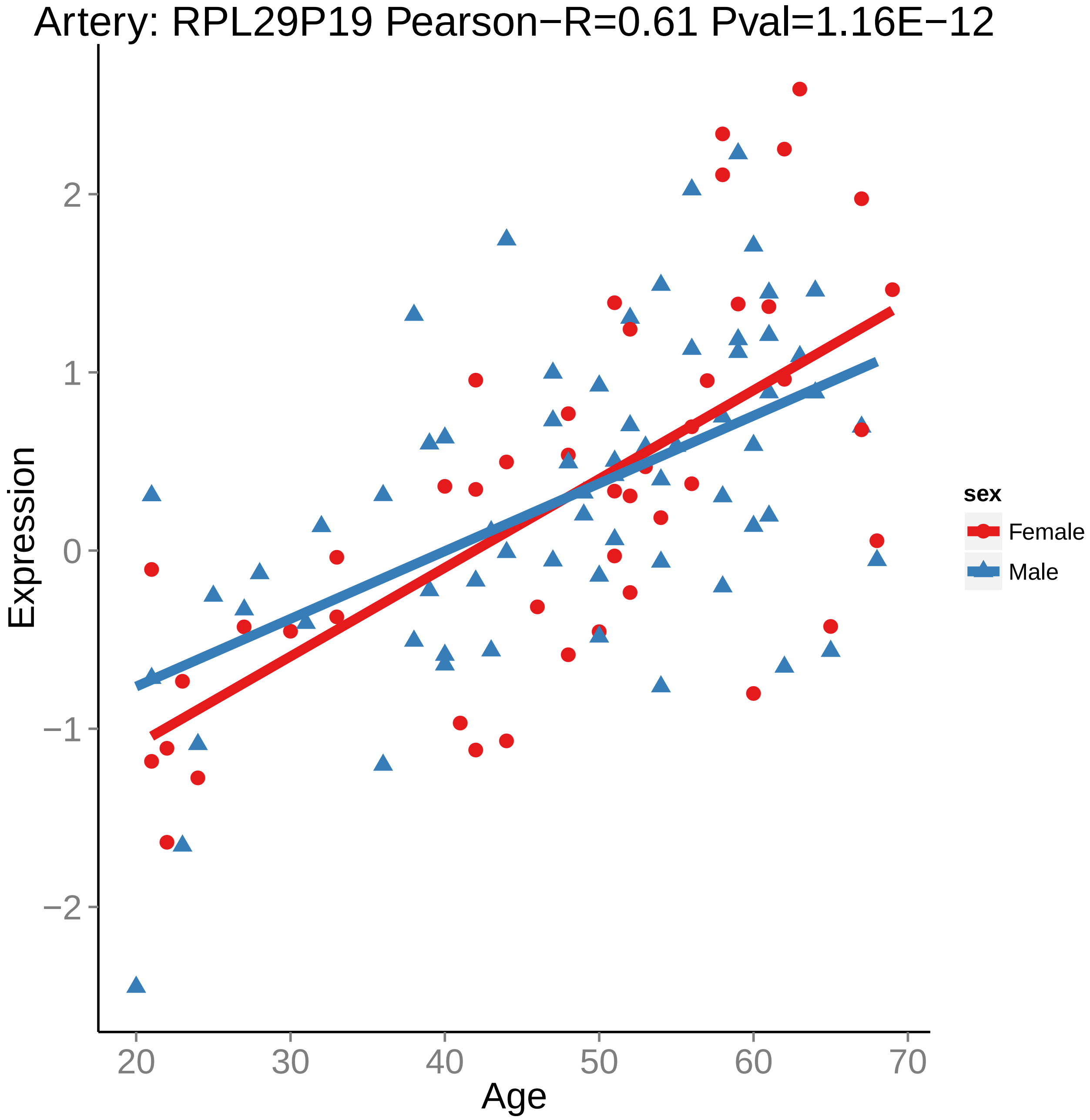

Artery: RPL29P19 Pearson−R=0.61 Pval=1.16E−12
Expression
Age
2
1
0
−1
−2
20
30
40
50
60
70
sex
Female
Male

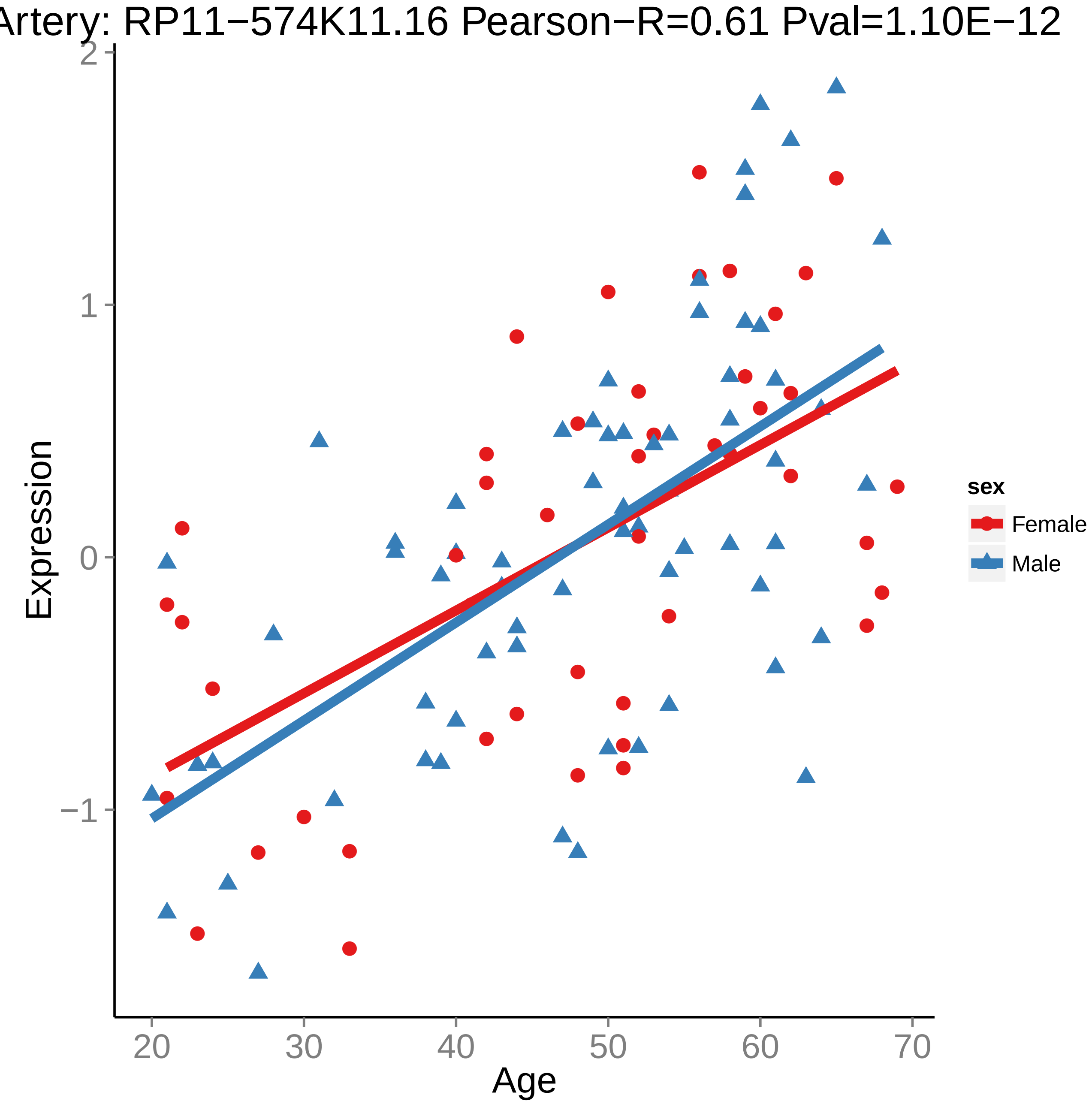

Artery: RP11−574K11.16 Pearson−R=0.61 Pval=1.10E−12
Expression
Age
2
1
0
−1
20
30
40
50
60
70
sex
Female
Male

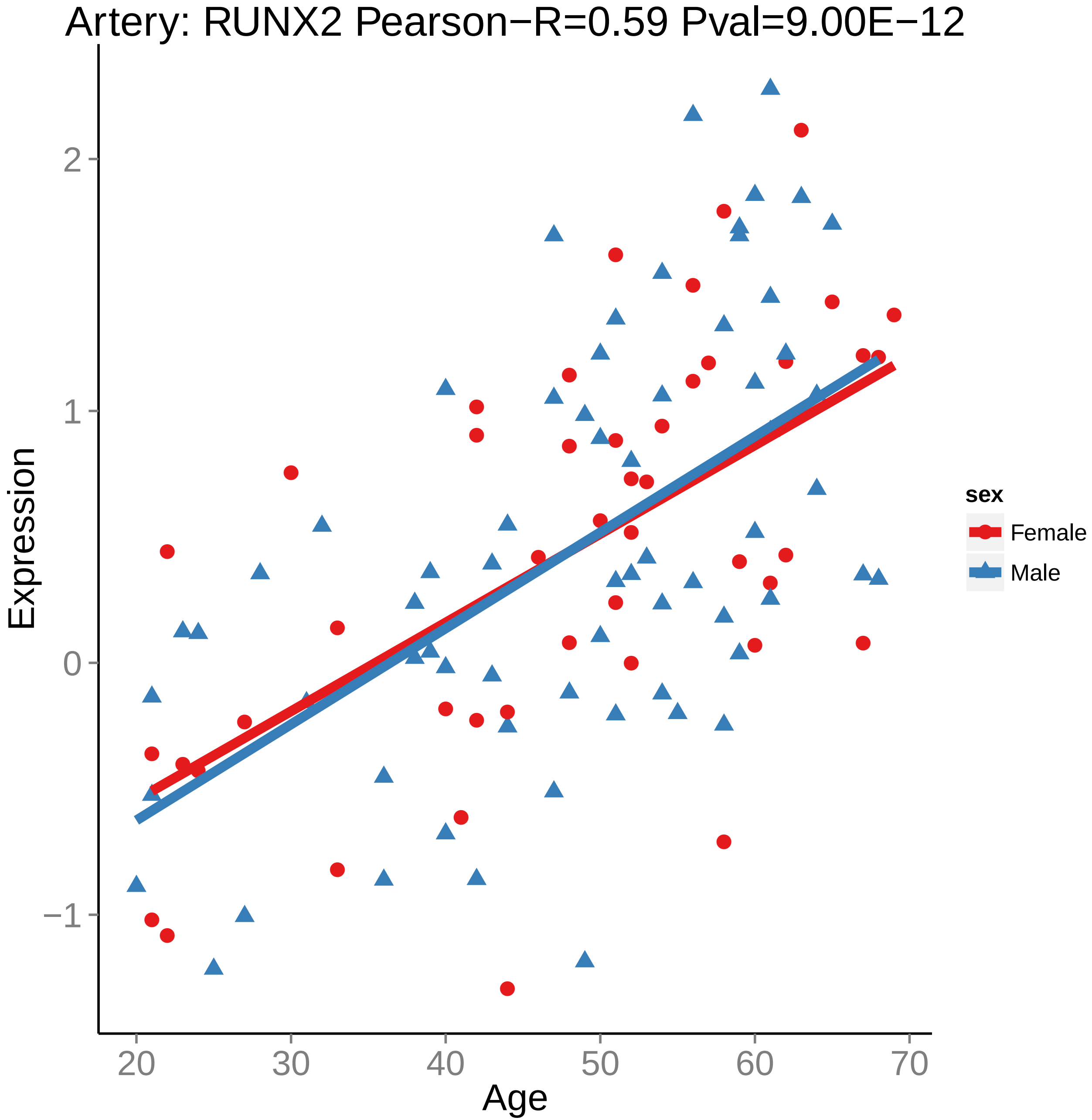

Artery: RUNX2 Pearson−R=0.59 Pval=9.00E−12
Expression
2
1
0
−1
20
30
40
50
60
70
Age
sex
Female
Male

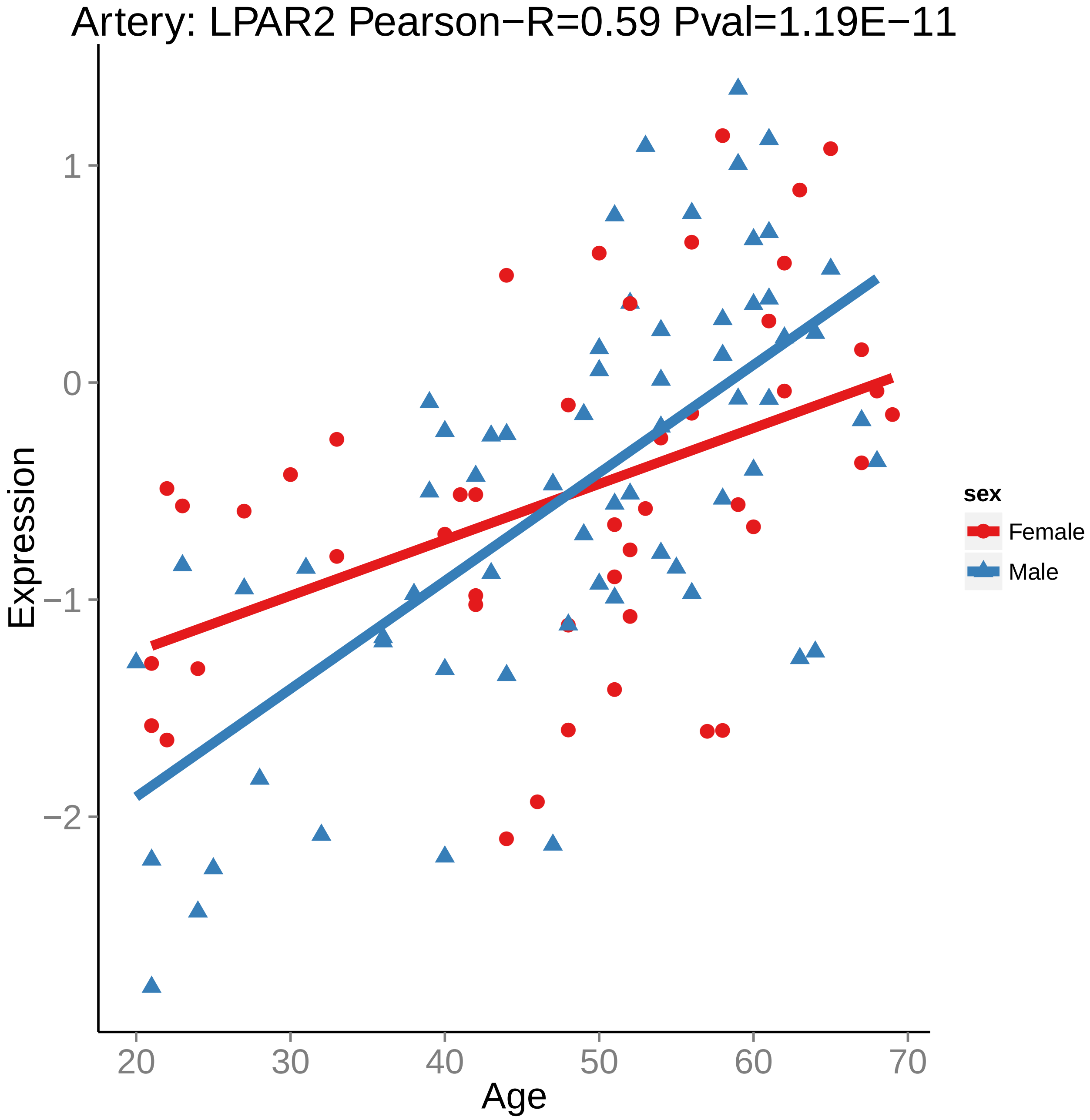

Artery: LPAR2 Pearson−R=0.59 Pval=1.19E−11
Expression
Age
1
0
−1
−2
20
30
40
50
60
70
sex
Female
Male

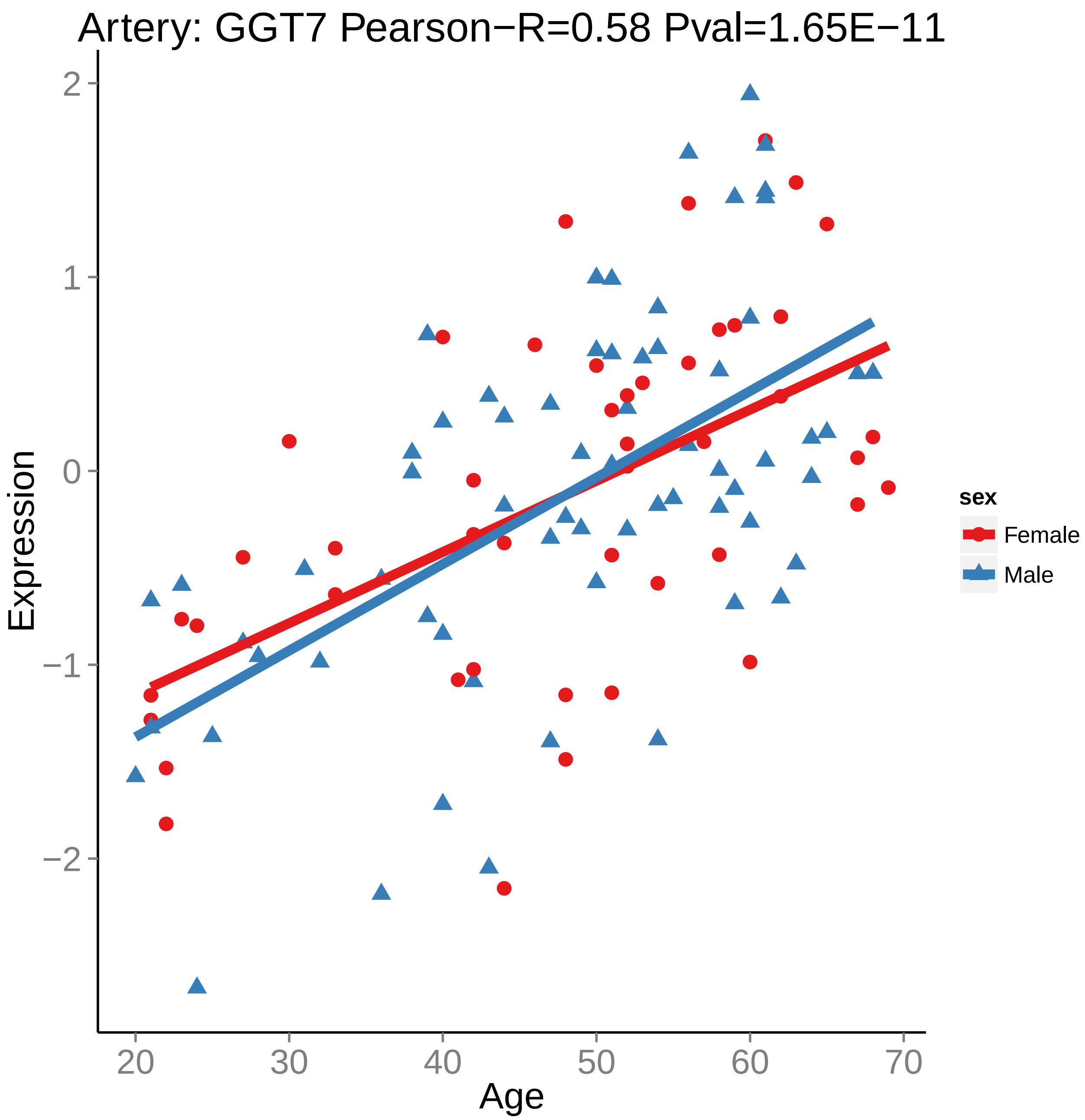

Artery: GGT7 Pearson−R=0.58 Pval=1.65E−11
Expression
Age
2
1
0
−1
−2
20
30
40
50
60
70
sex
Female
Male

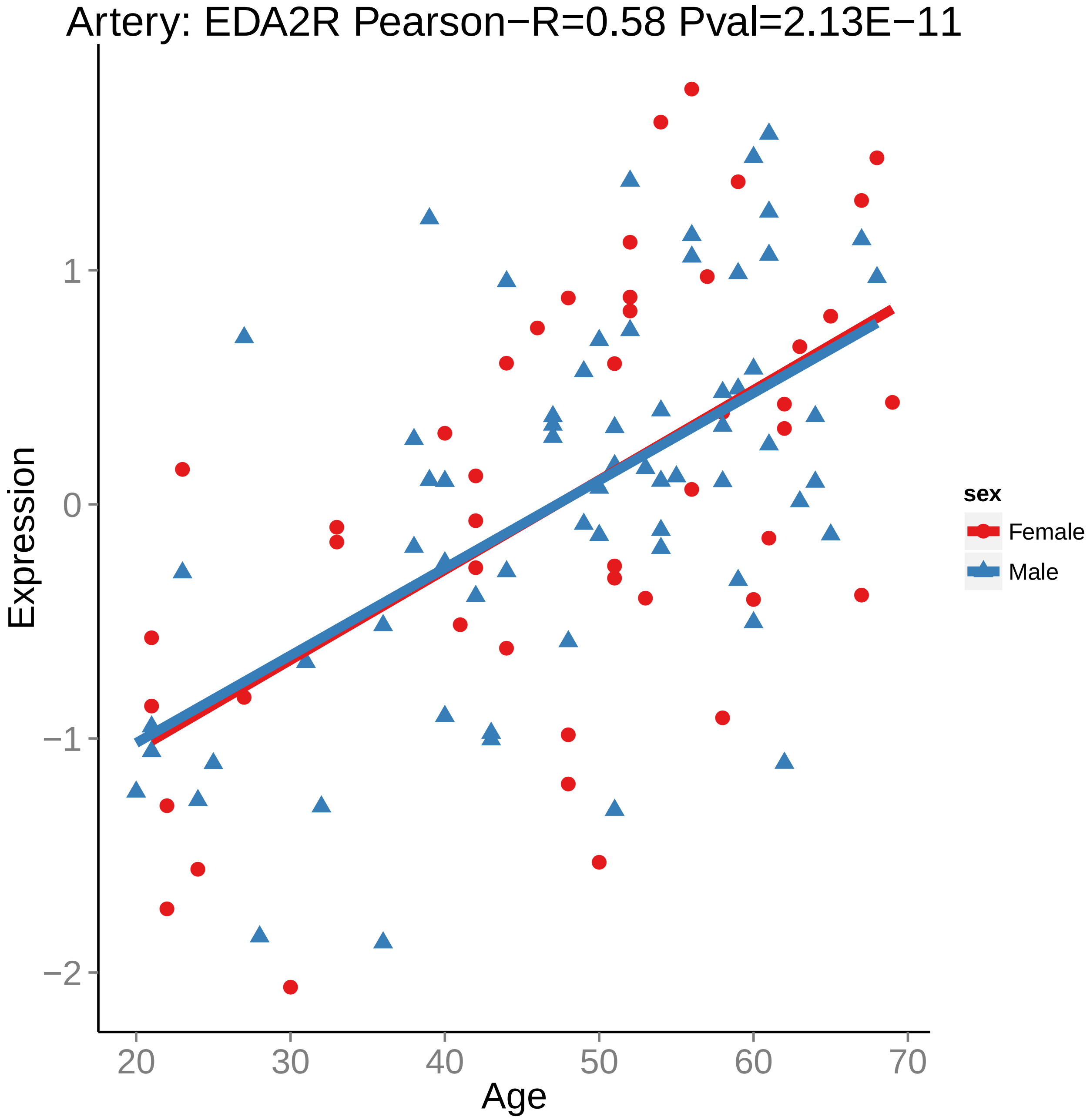
Artery: EDA2R Pearson−R=0.58 Pval=2.13E−11
Expression
Age
1
0
−1
−2
20
30
40
50
60
70
sex
Female
Male

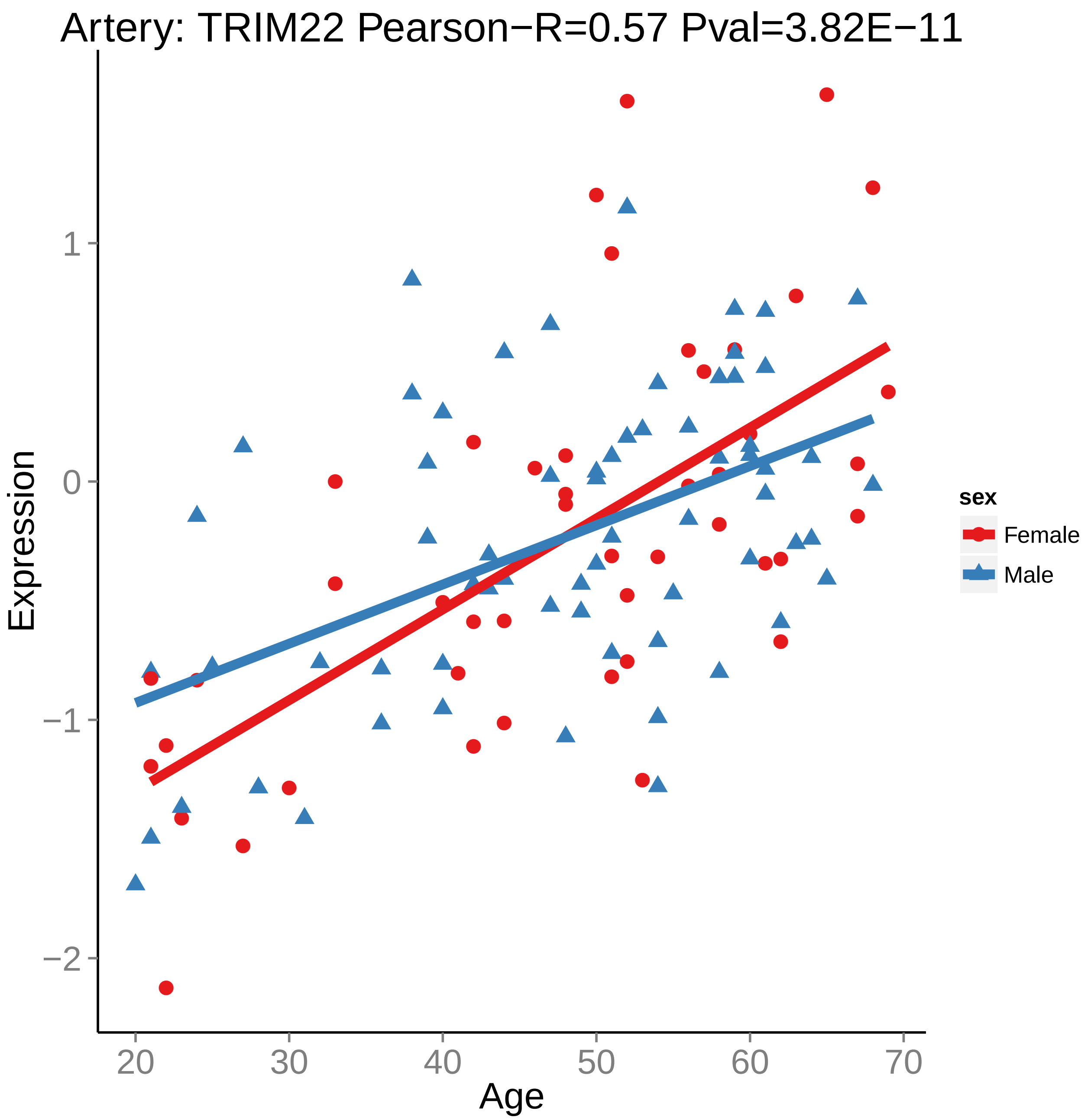
Artery: TRIM22 Pearson−R=0.57 Pval=3.82E−11
Expression
1
0
−1
−2
20
30
40
50
60
70
Age
sex
Female
Male

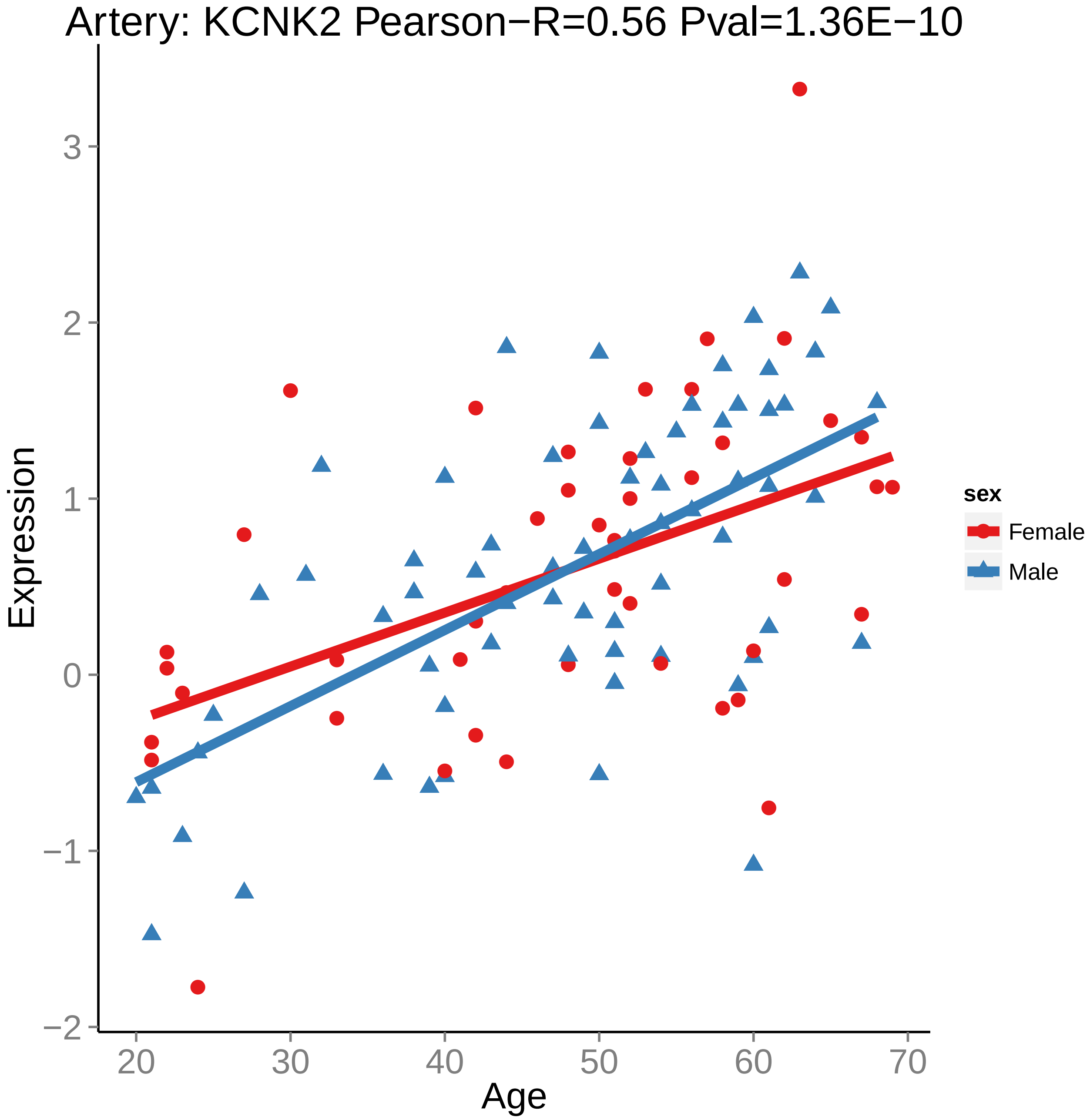

Artery: KCNK2 Pearson−R=0.56 Pval=1.36E−10
Expression
Age
3
2
1
0
−1
−2
20
30
40
50
60
70
sex
Female
Male

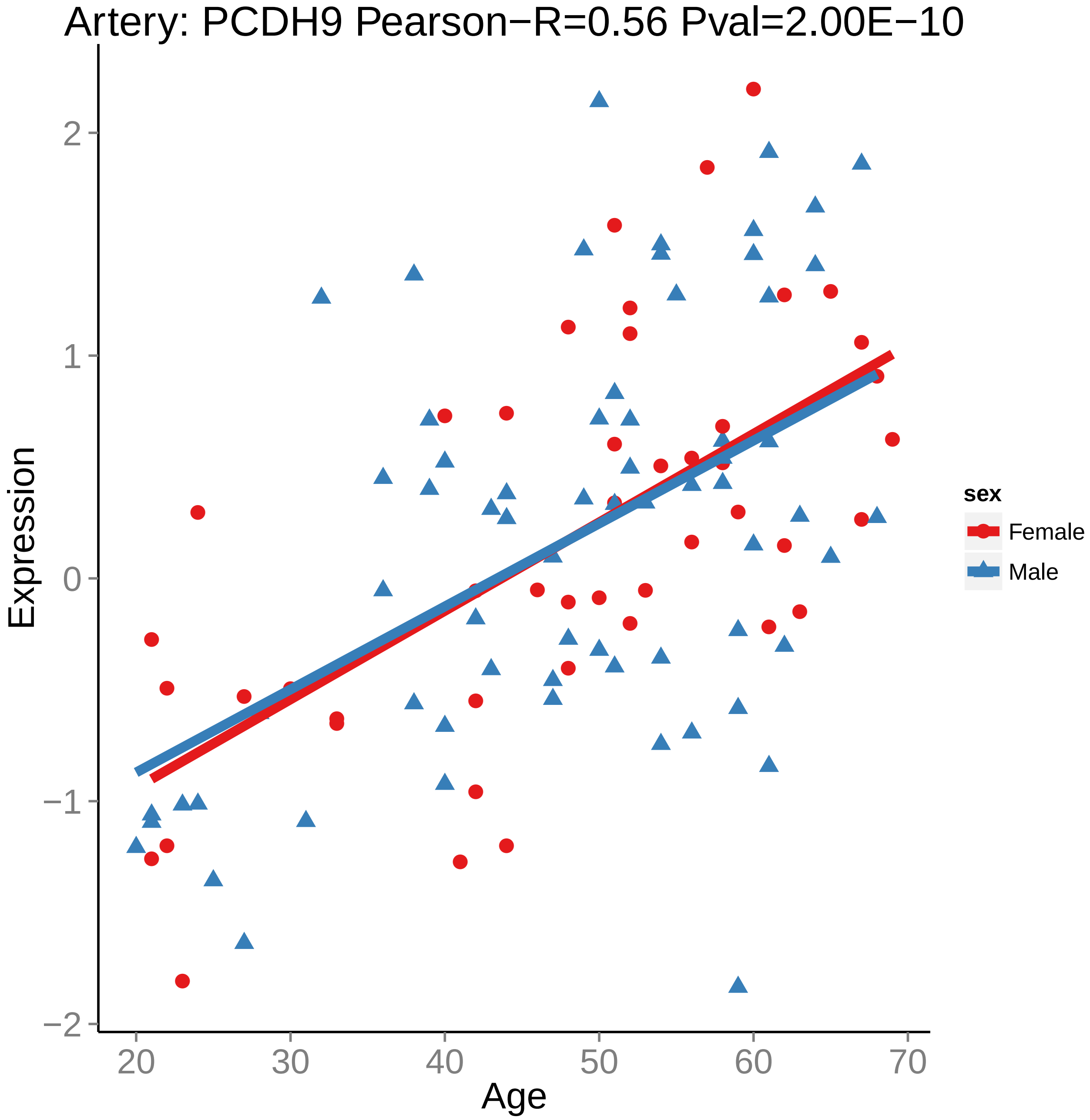

Artery: PCDH9 Pearson−R=0.56 Pval=2.00E−10
Expression
2
1
0
−1
−2
20
30
40
50
60
70
Age
sex
Female
Male

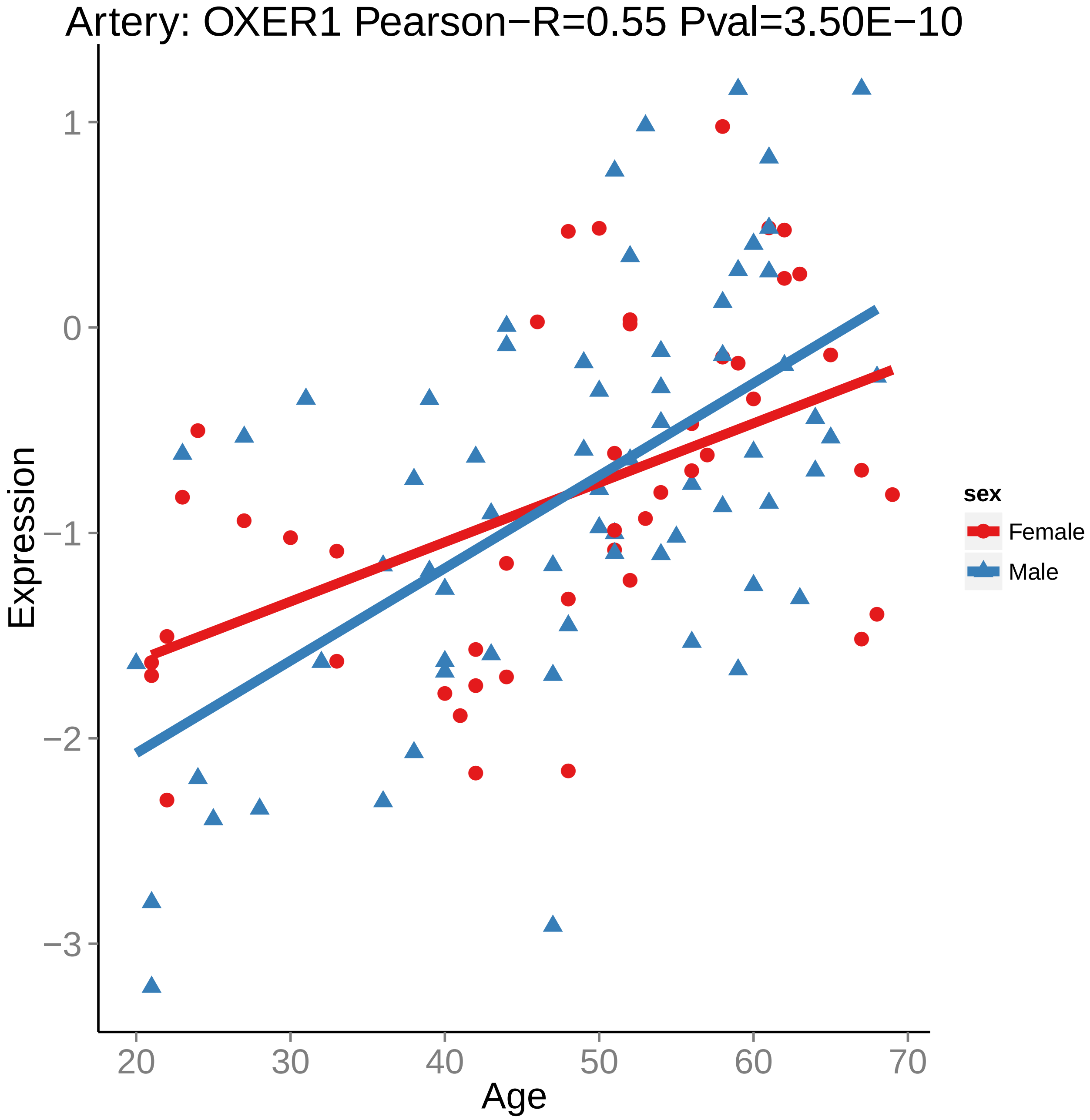

Artery: OXER1 Pearson−R=0.55 Pval=3.50E−10
Expression
1
0
−1
−2
−3
Age
20
30
40
50
60
70
sex
Female
Male

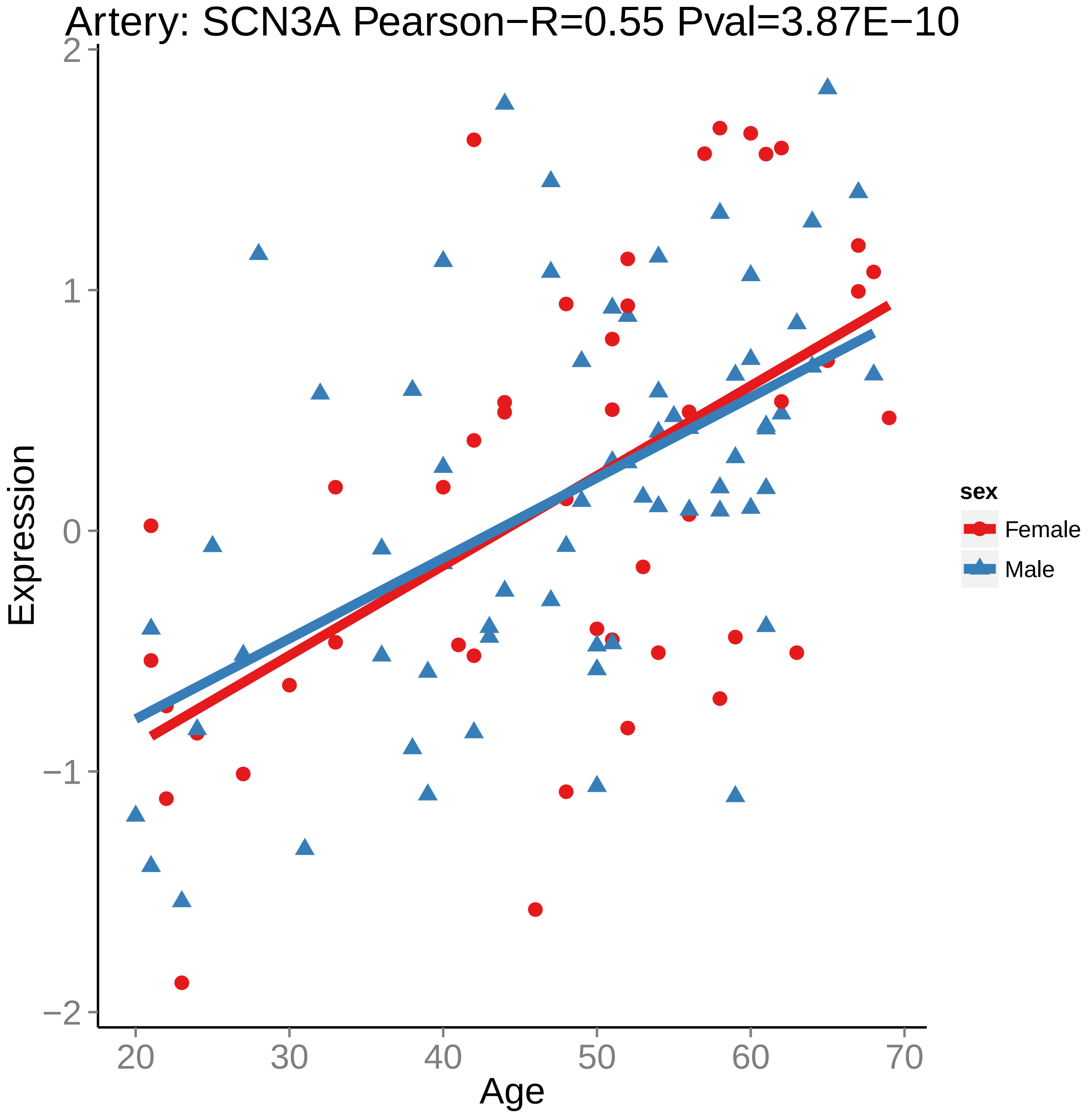
Artery: SCN3A Pearson−R=0.55 Pval=3.87E−10
Expression
Age
2
1
0
−1
−2
20
30
40
50
60
70
sex
Female
Male

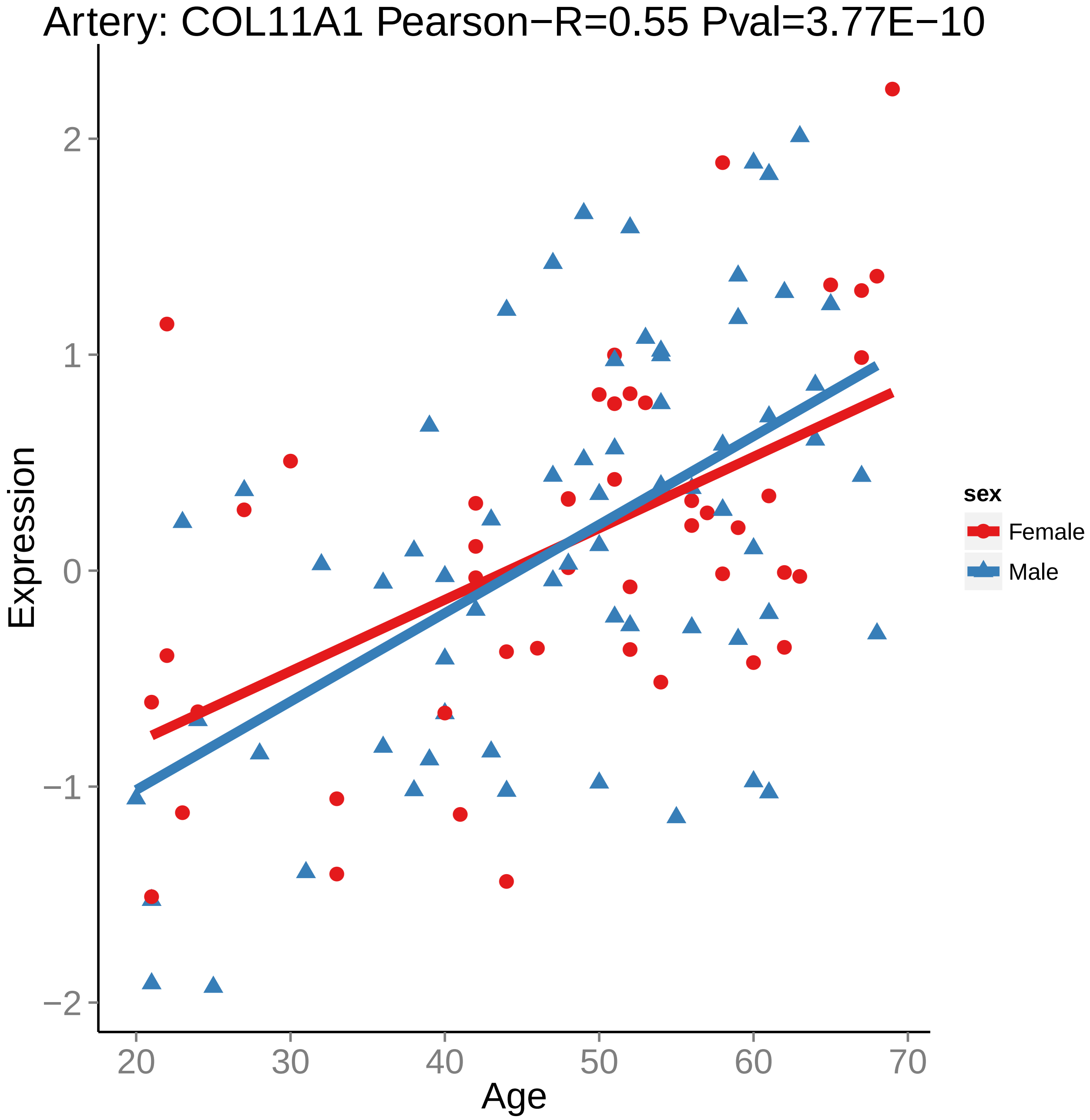

Artery: COL11A1 Pearson−R=0.55 Pval=3.77E−10
Expression
Age
2
1
0
−1
−2
20
30
40
50
60
70
sex
Female
Male

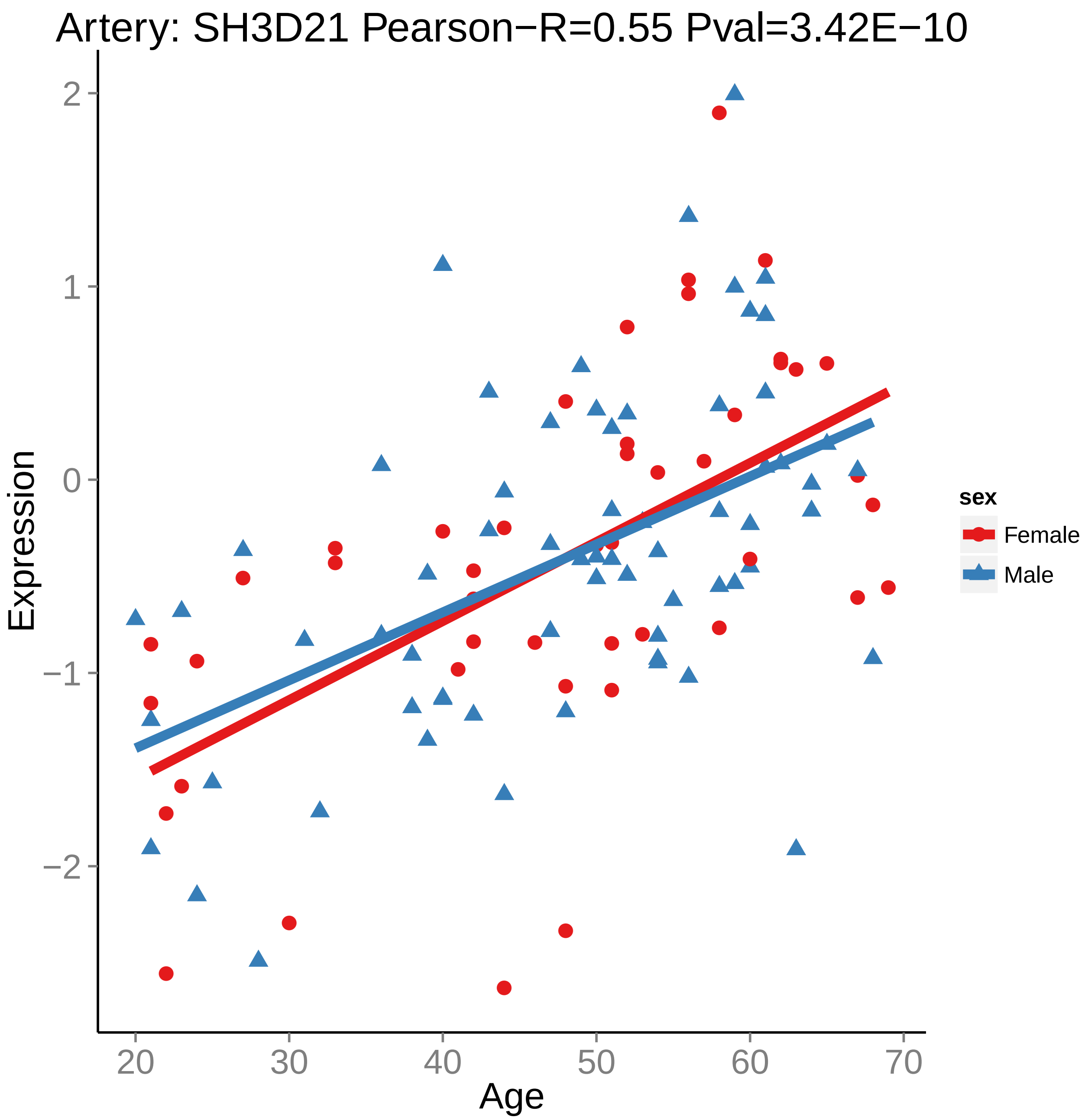

Artery: SH3D21 Pearson−R=0.55 Pval=3.42E−10
Expression
Age
2
1
0
−1
−2
20
30
40
50
60
70
sex
Female
Male

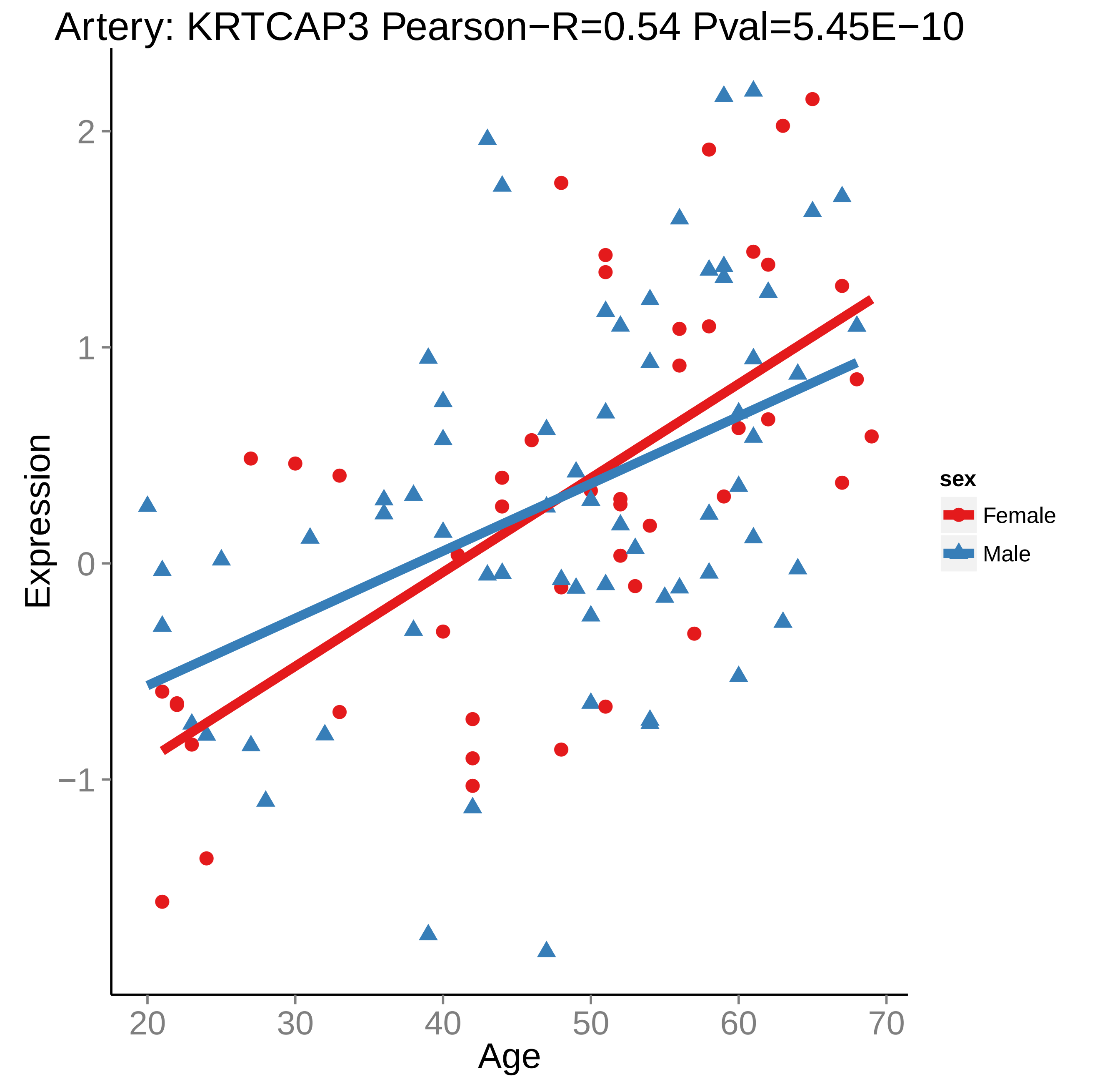

Artery: KRTCAP3 Pearson−R=0.54 Pval=5.45E−10
Expression
Age
2
1
0
−1
20
30
40
50
60
70
sex
Female
Male

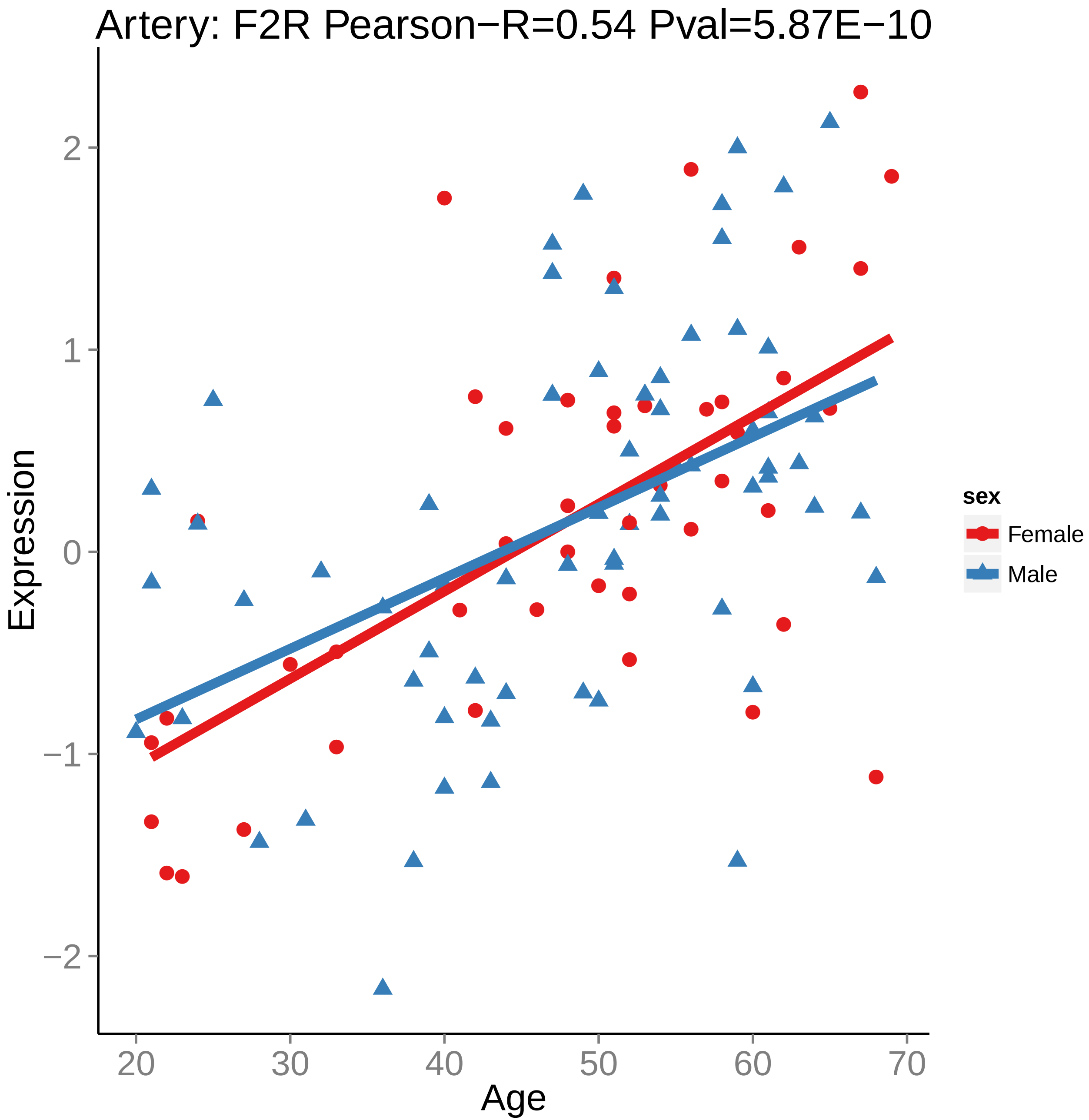

Artery: F2R Pearson−R=0.54 Pval=5.87E−10
Expression
Age
2
1
0
−1
−2
20
30
40
50
60
70
sex
Female
Male

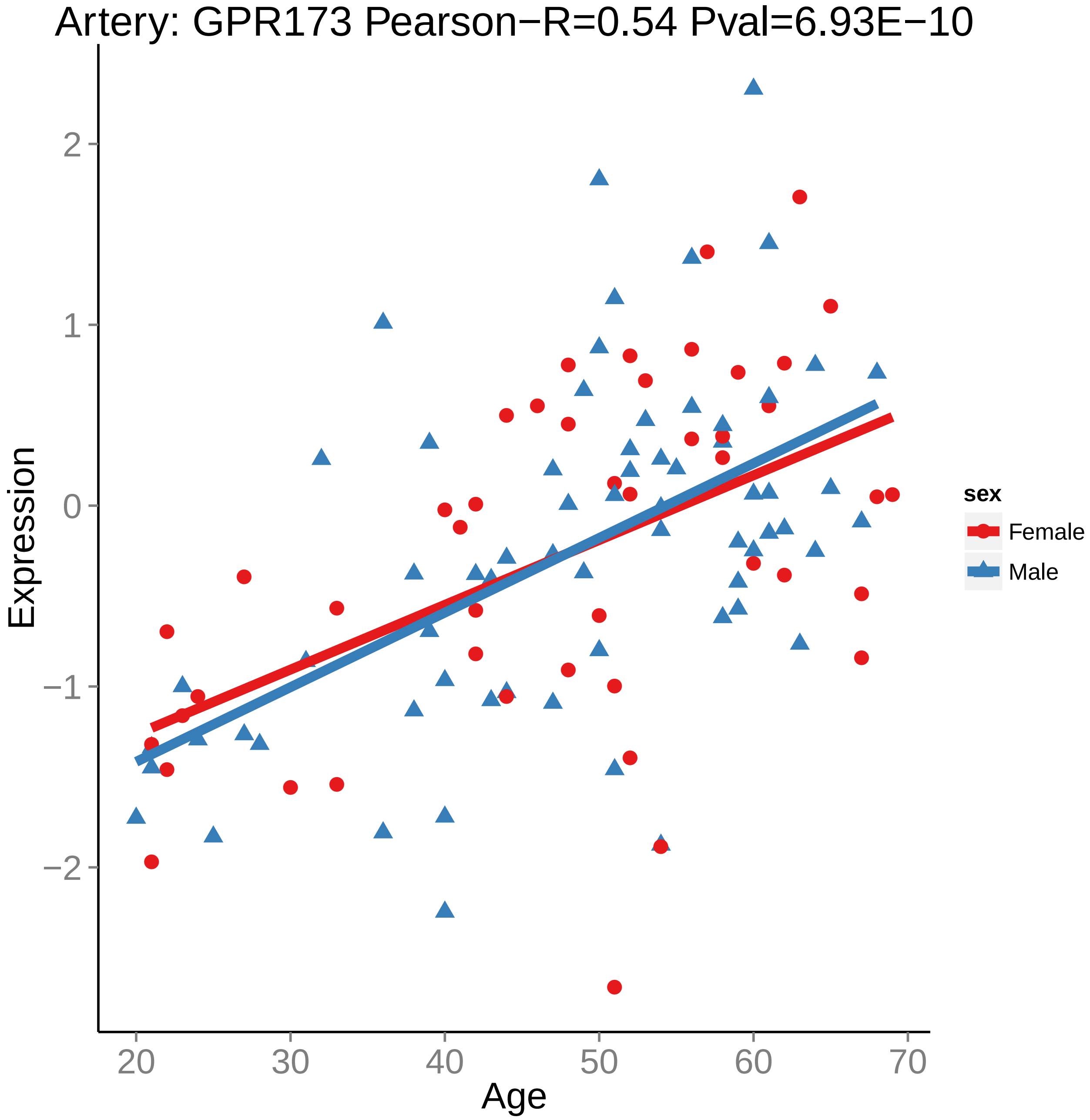

Artery: GPR173 Pearson−R=0.54 Pval=6.93E−10
Expression
Age
2
1
0
−1
−2
20
30
40
50
60
70
sex
Female
Male

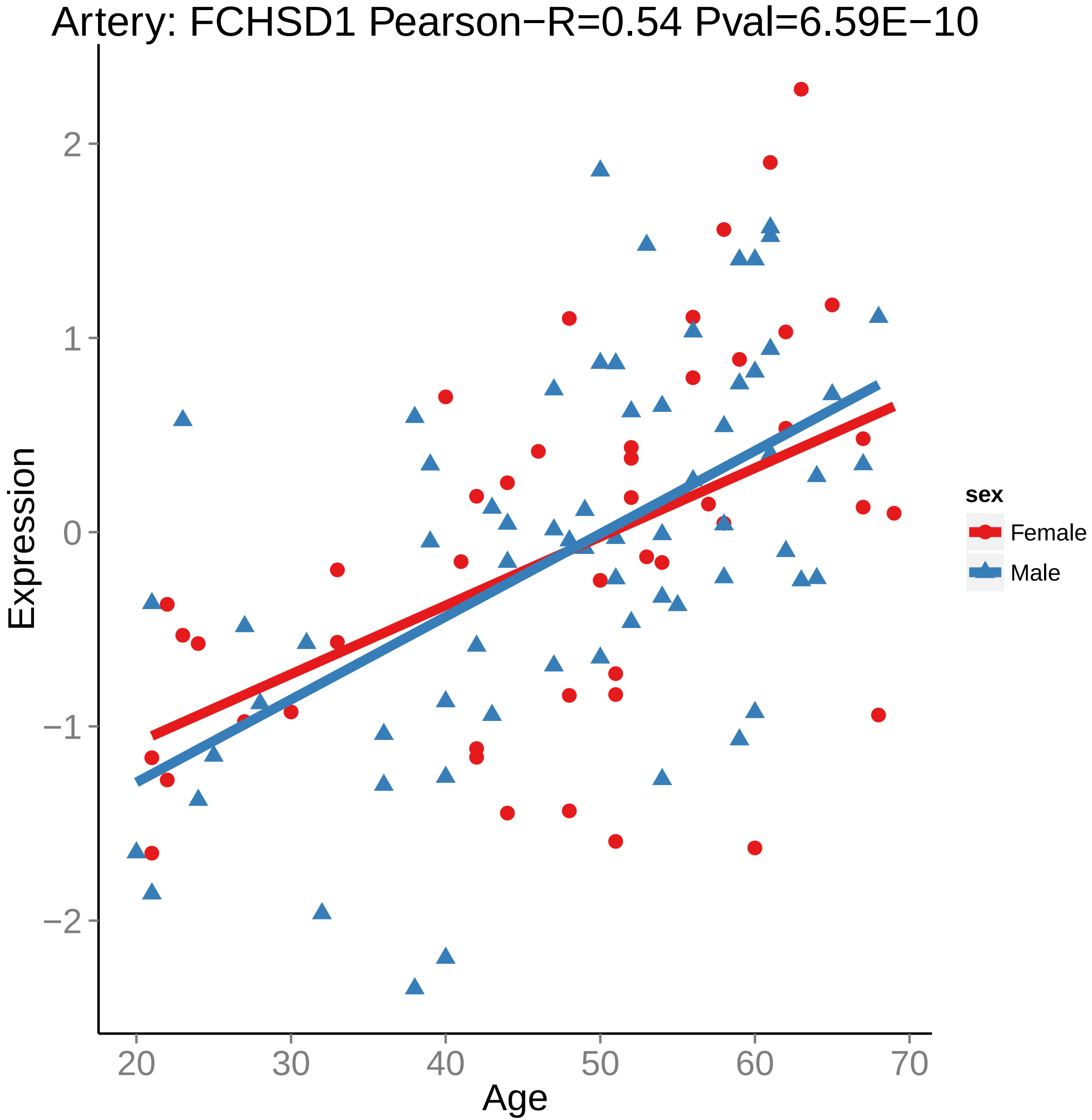

Artery: FCHSD1 Pearson−R=0.54 Pval=6.59E−10
Expression
Age
2
1
0
−1
−2
20
30
40
50
60
70
sex
Female
Male

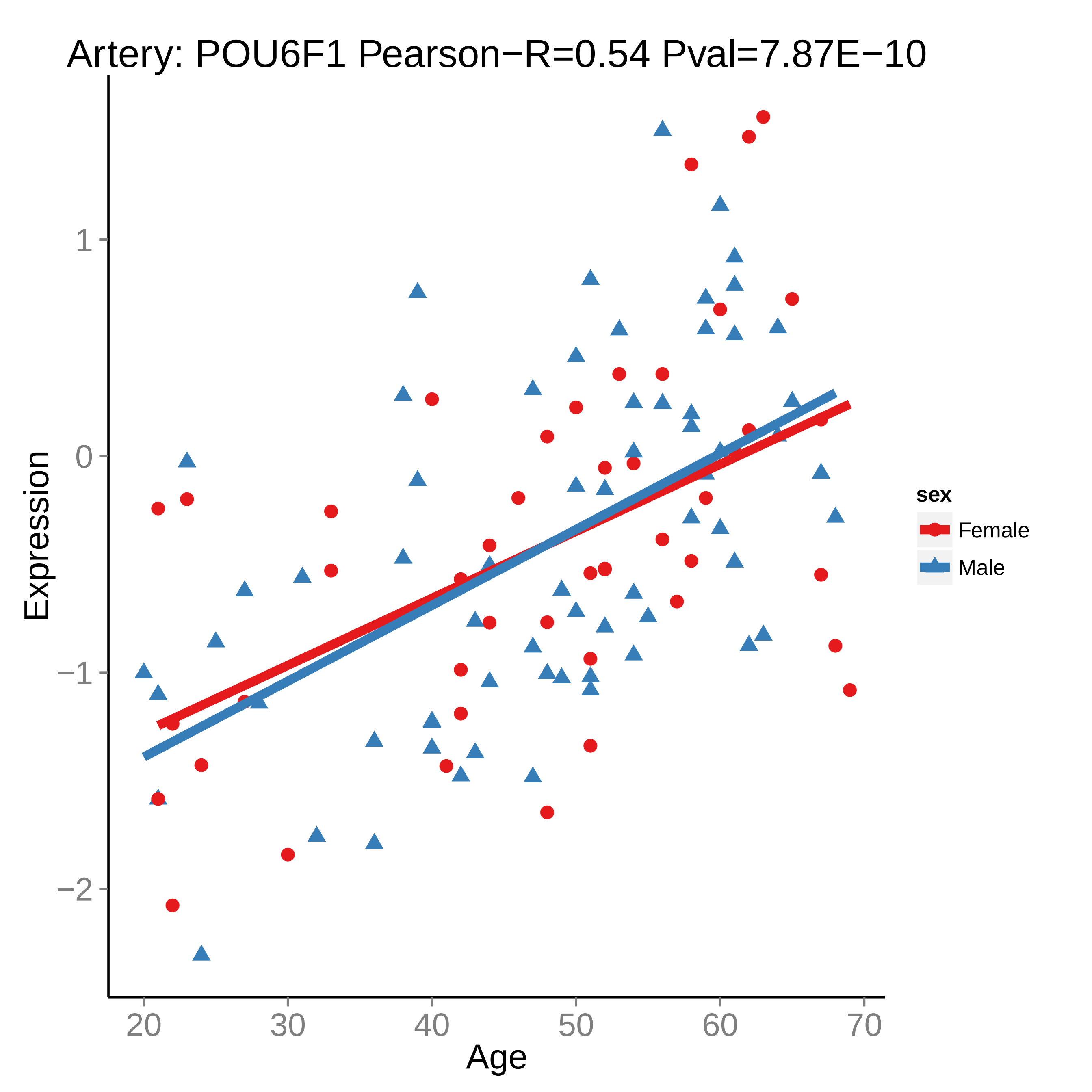

Artery: POU6F1 Pearson−R=0.54 Pval=7.87E−10
Expression
1
0
−1
−2
20
30
40
50
60
70
Age
sex
Female
Male

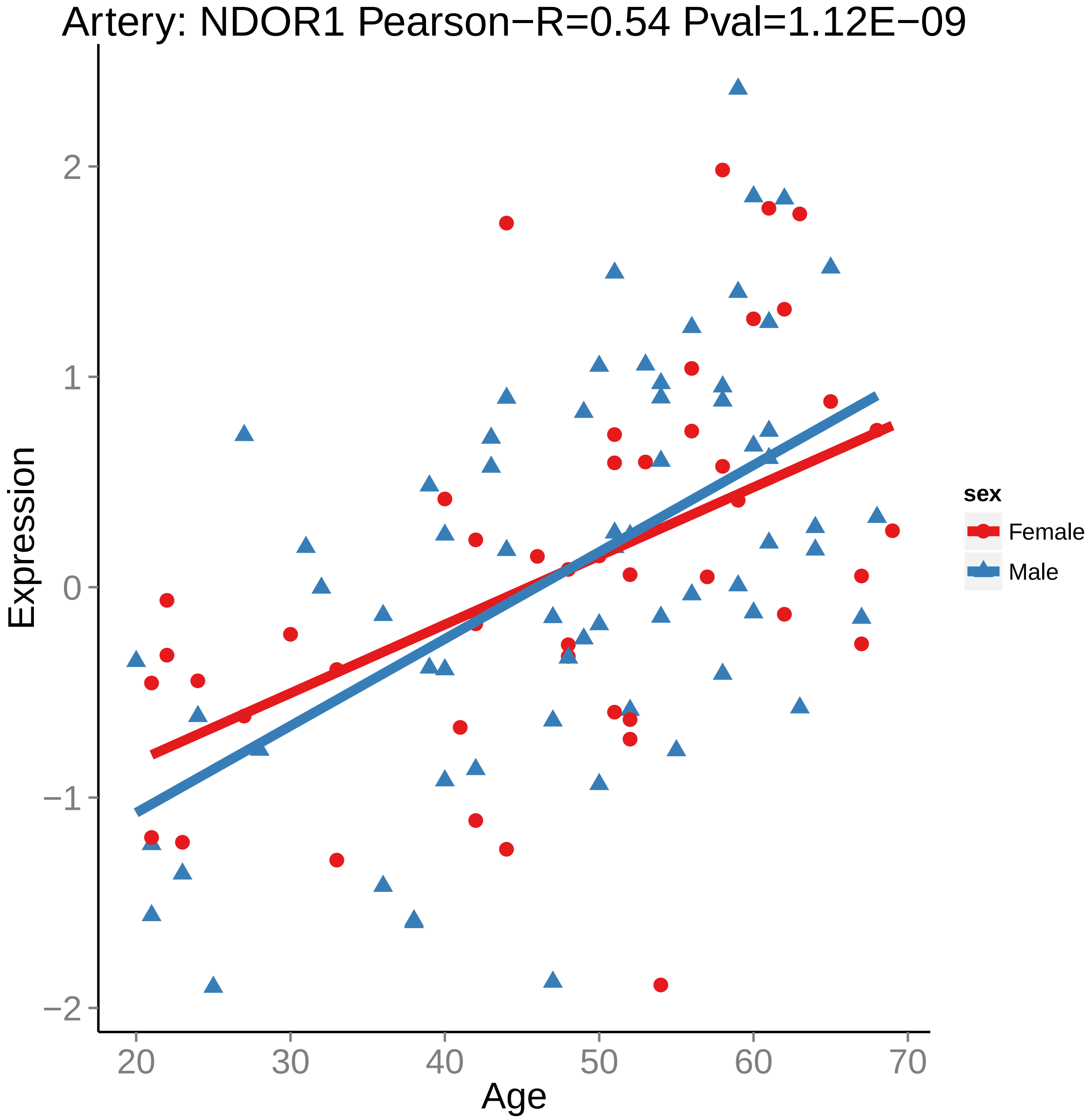

Artery: NDOR1 Pearson−R=0.54 Pval=1.12E−09
Expression
Age
2
1
0
−1
−2
20
30
40
50
60
70
sex
Female
Male

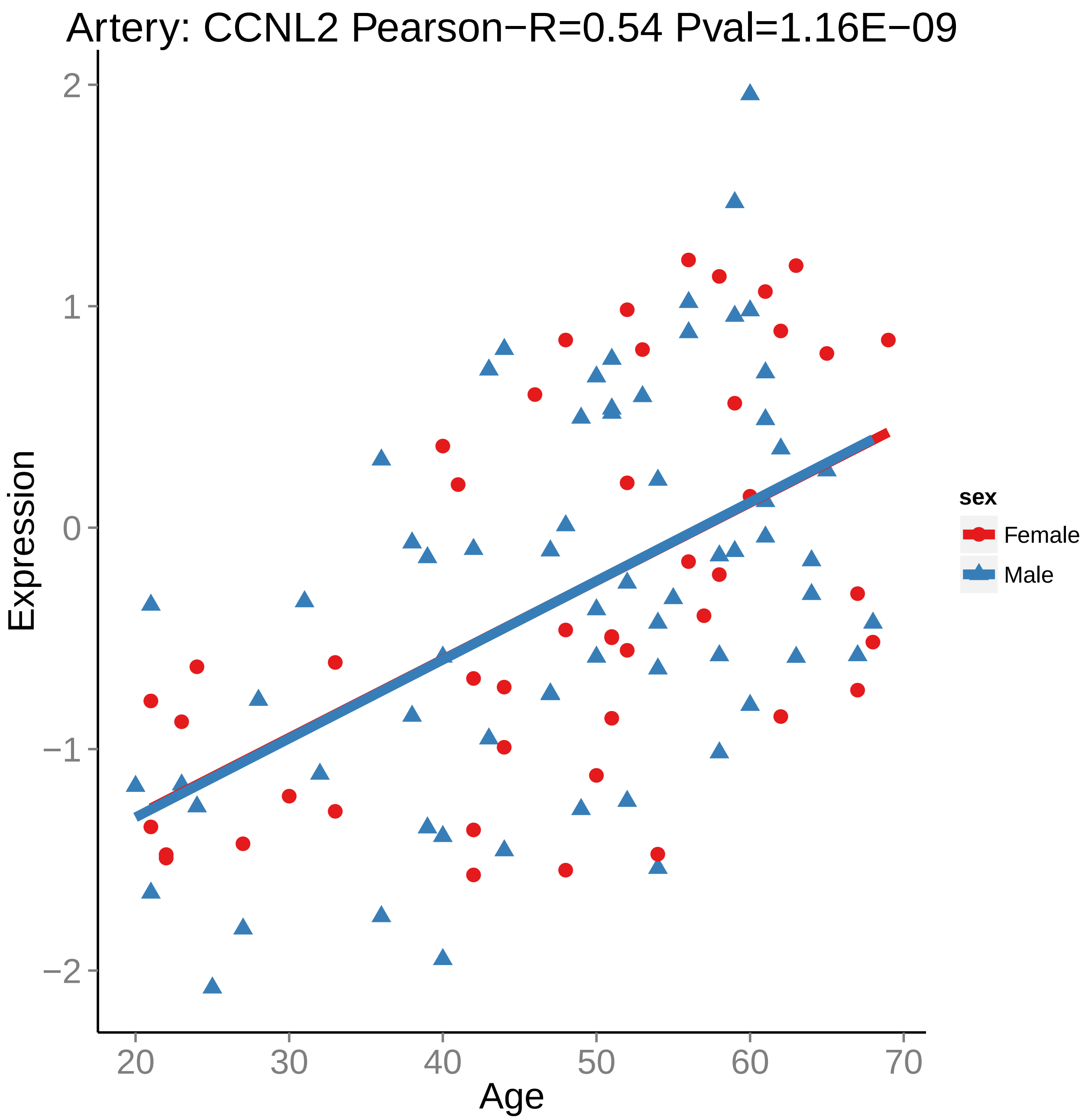

Artery: CCNL2 Pearson−R=0.54 Pval=1.16E−09
Expression
Age
2
1
0
−1
−2
20
30
40
50
60
70
sex
Female
Male

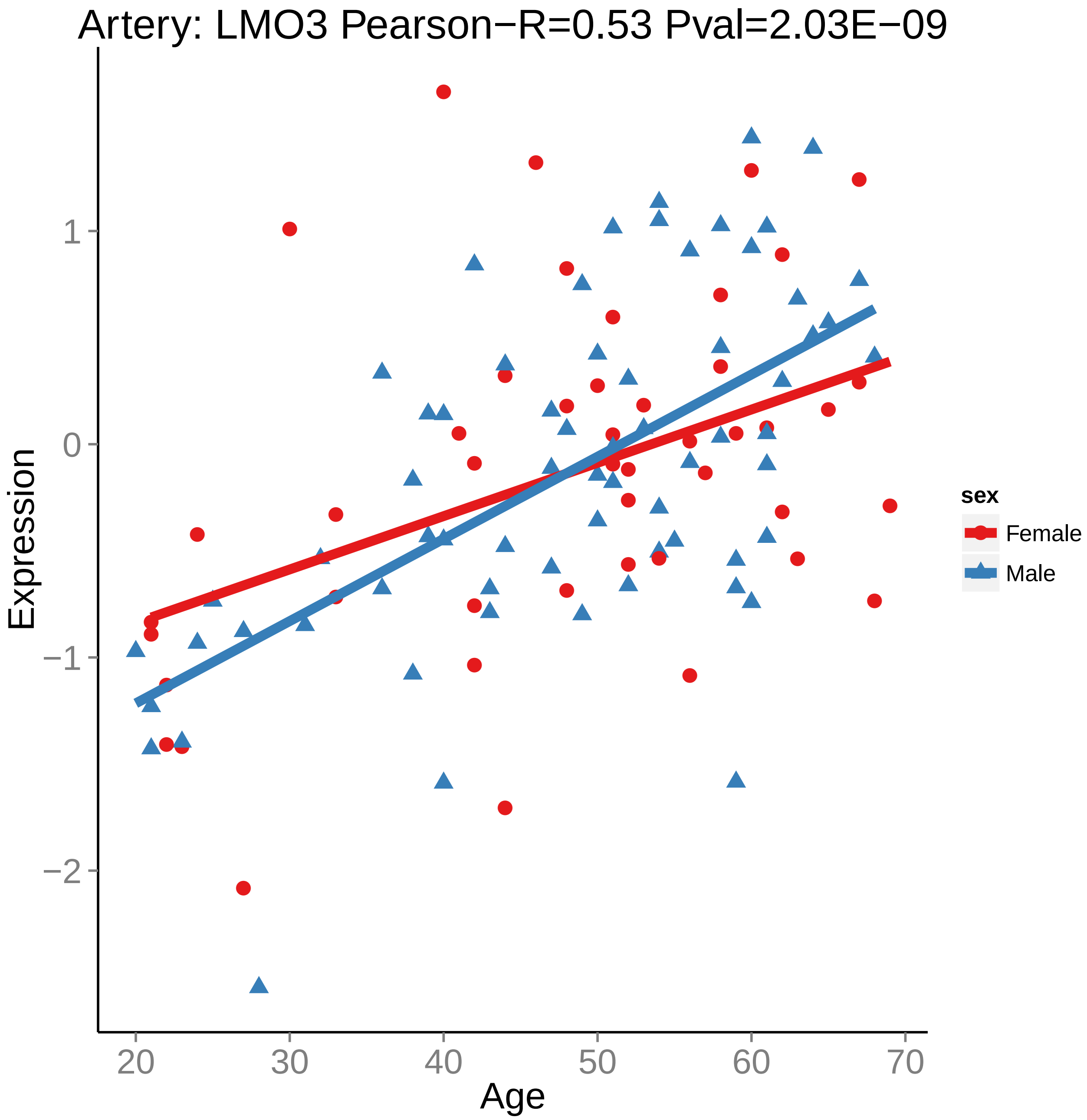
Artery: LMO3 Pearson−R=0.53 Pval=2.03E−09
Expression
Age
1
0
−1
−2
20
30
40
50
60
70
sex
Female
Male

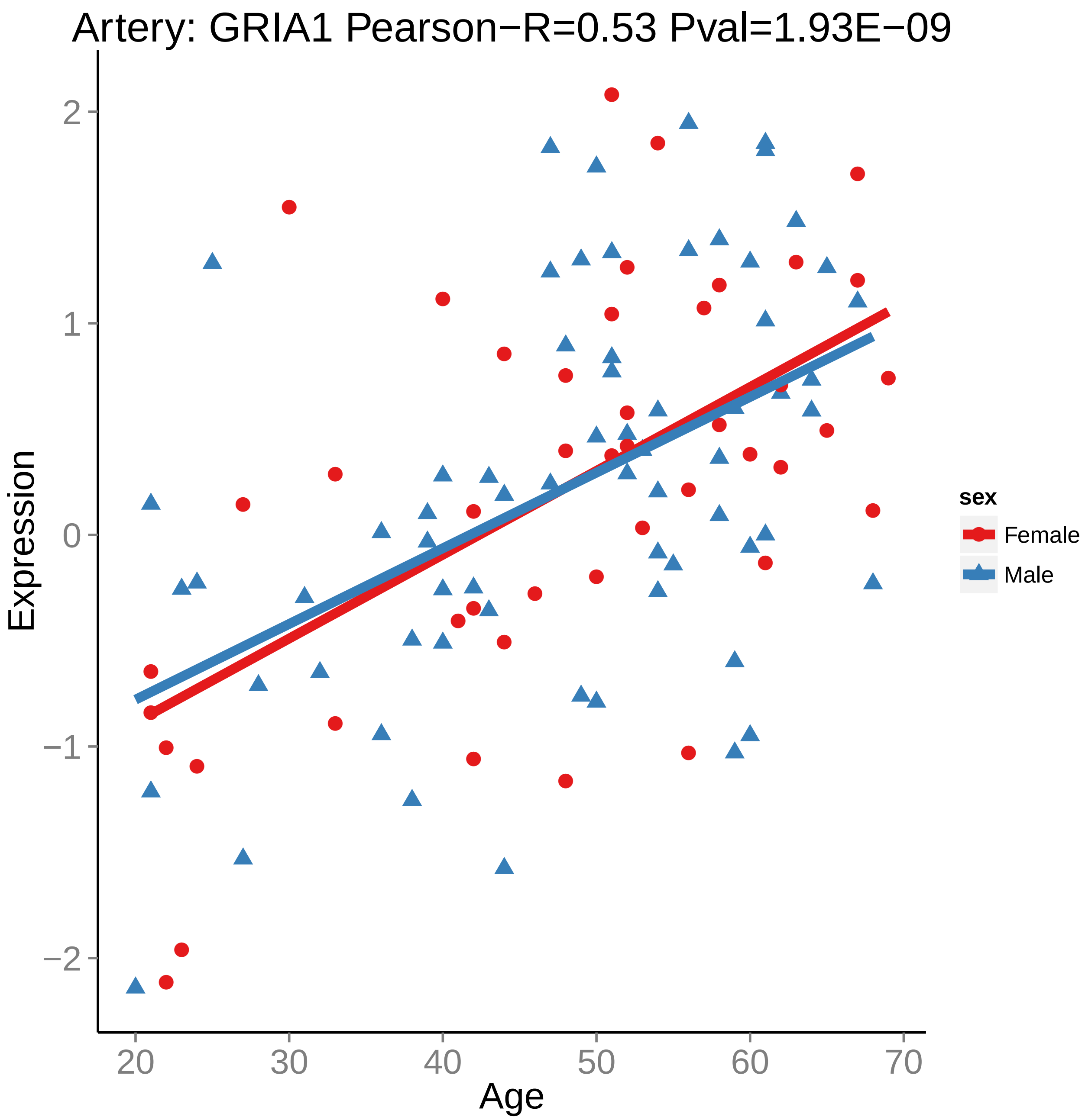

Artery: GRIA1 Pearson−R=0.53 Pval=1.93E−09
Expression
Age
2
1
0
−1
−2
20
30
40
50
60
70
sex
Female
Male

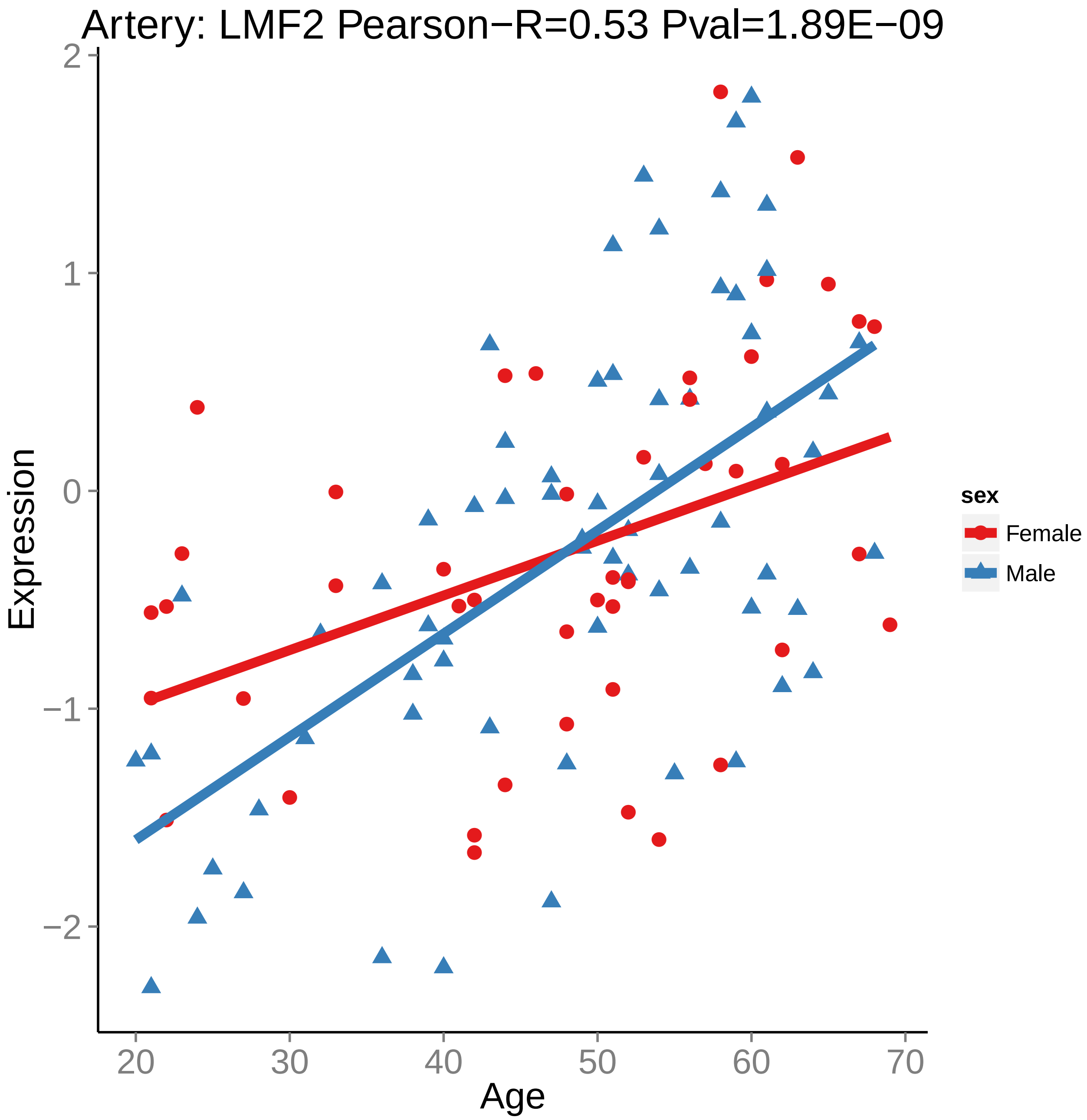

Artery: LMF2 Pearson−R=0.53 Pval=1.89E−09
Expression
Age
2
1
0
−1
−2
20
30
40
50
60
70
sex
Female
Male

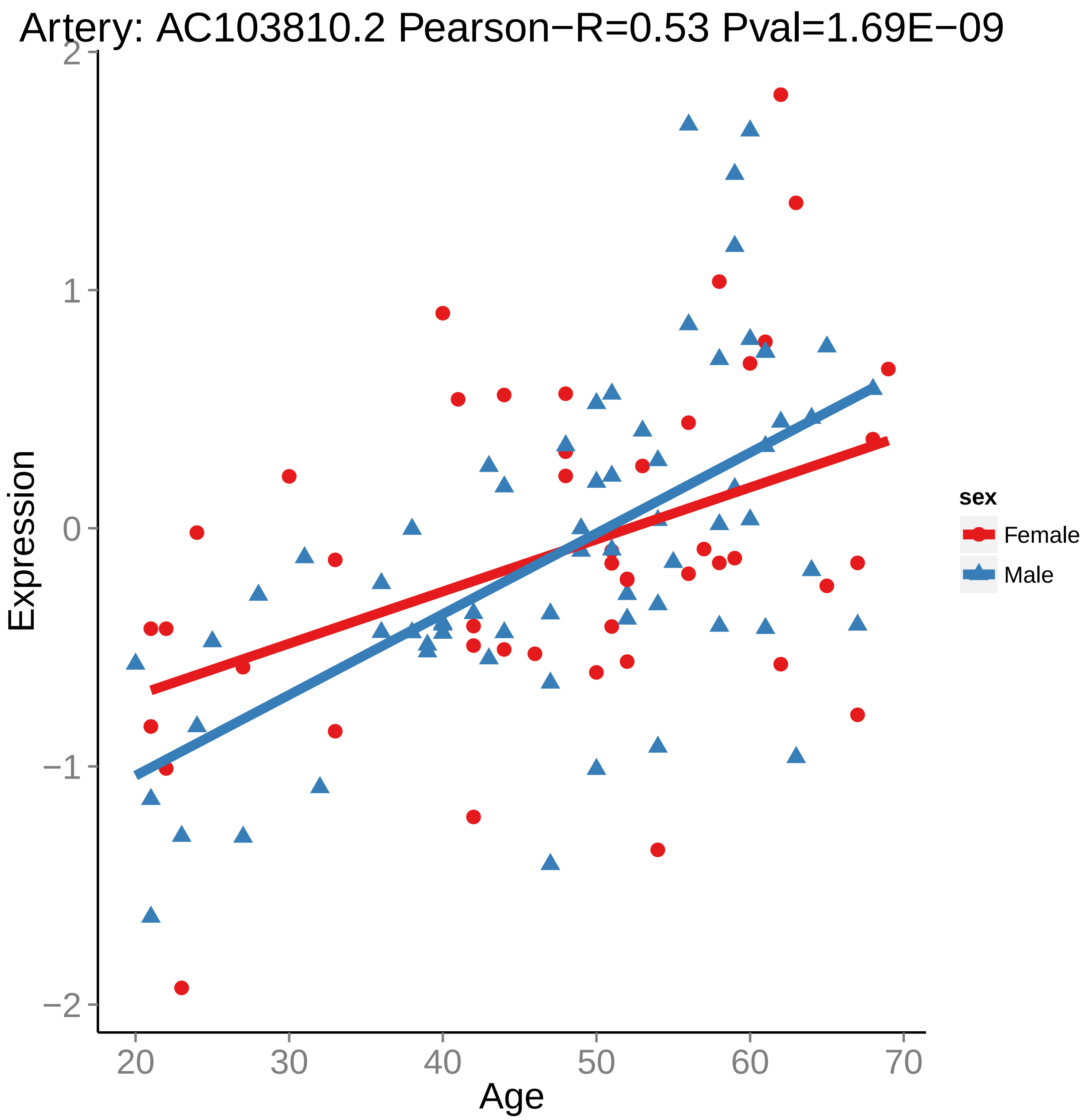
Artery: AC103810.2 Pearson−R=0.53 Pval=1.69E−09
Expression
Age
2
1
0
−1
−2
20
30
40
50
60
70
sex
Female
Male

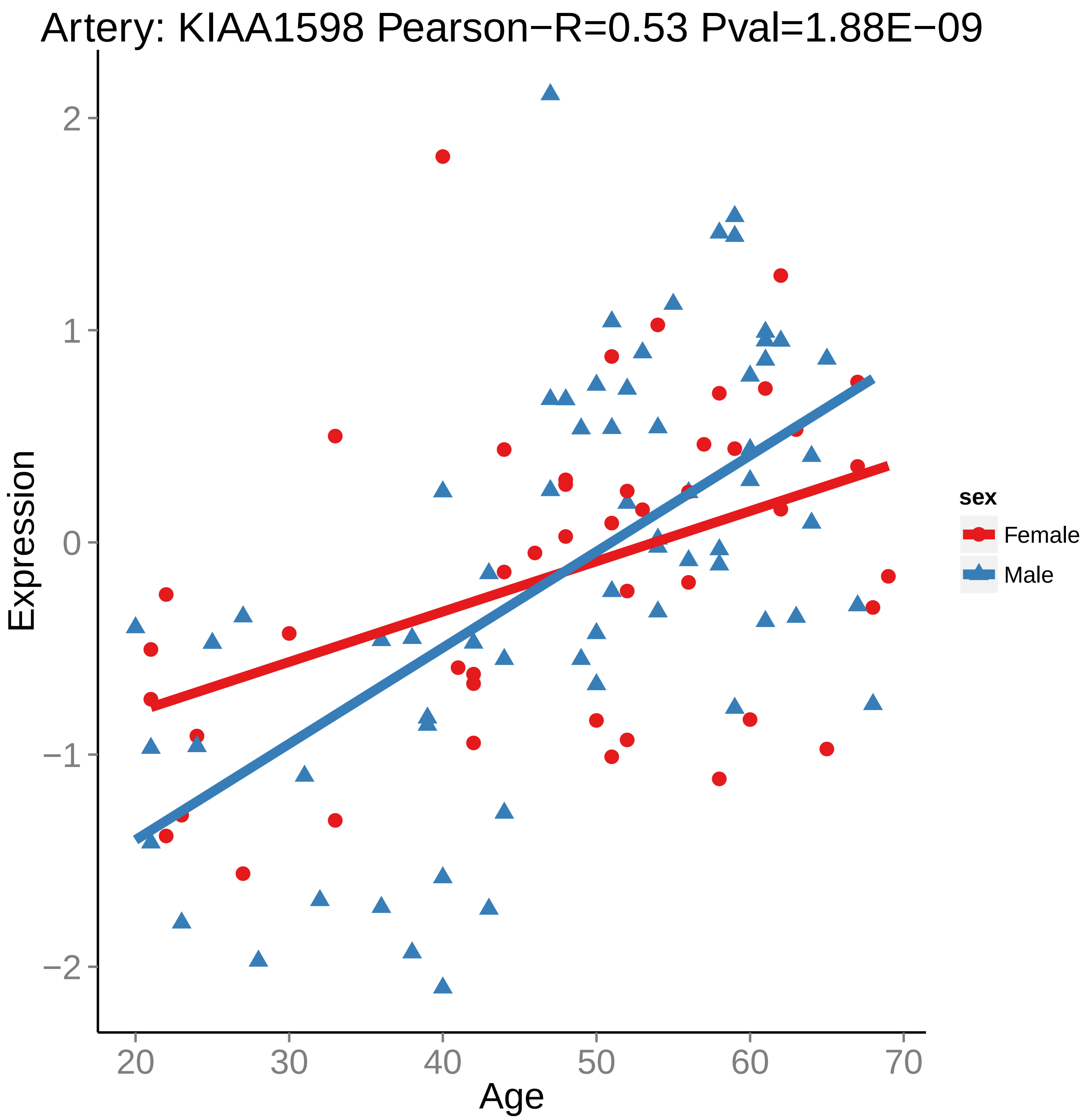

Artery: KIAA1598 Pearson−R=0.53 Pval=1.88E−09
Expression
Age
2
1
0
−1
−2
20
30
40
50
60
70
sex
Female
Male

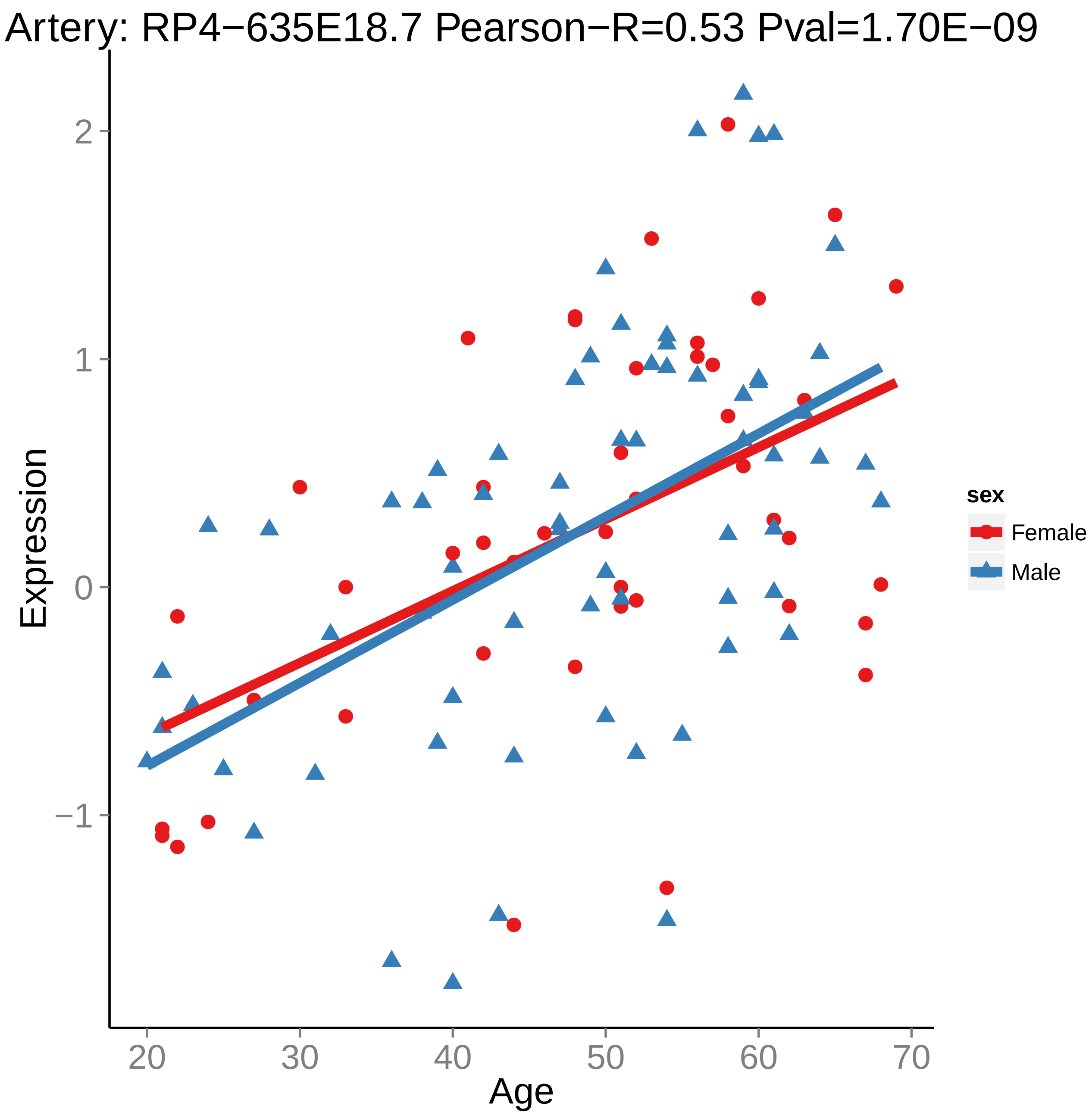

Artery: RP4−635E18.7 Pearson−R=0.53 Pval=1.70E−09
Expression
Age
2
1
0
−1
20
30
40
50
60
70
sex
Female
Male

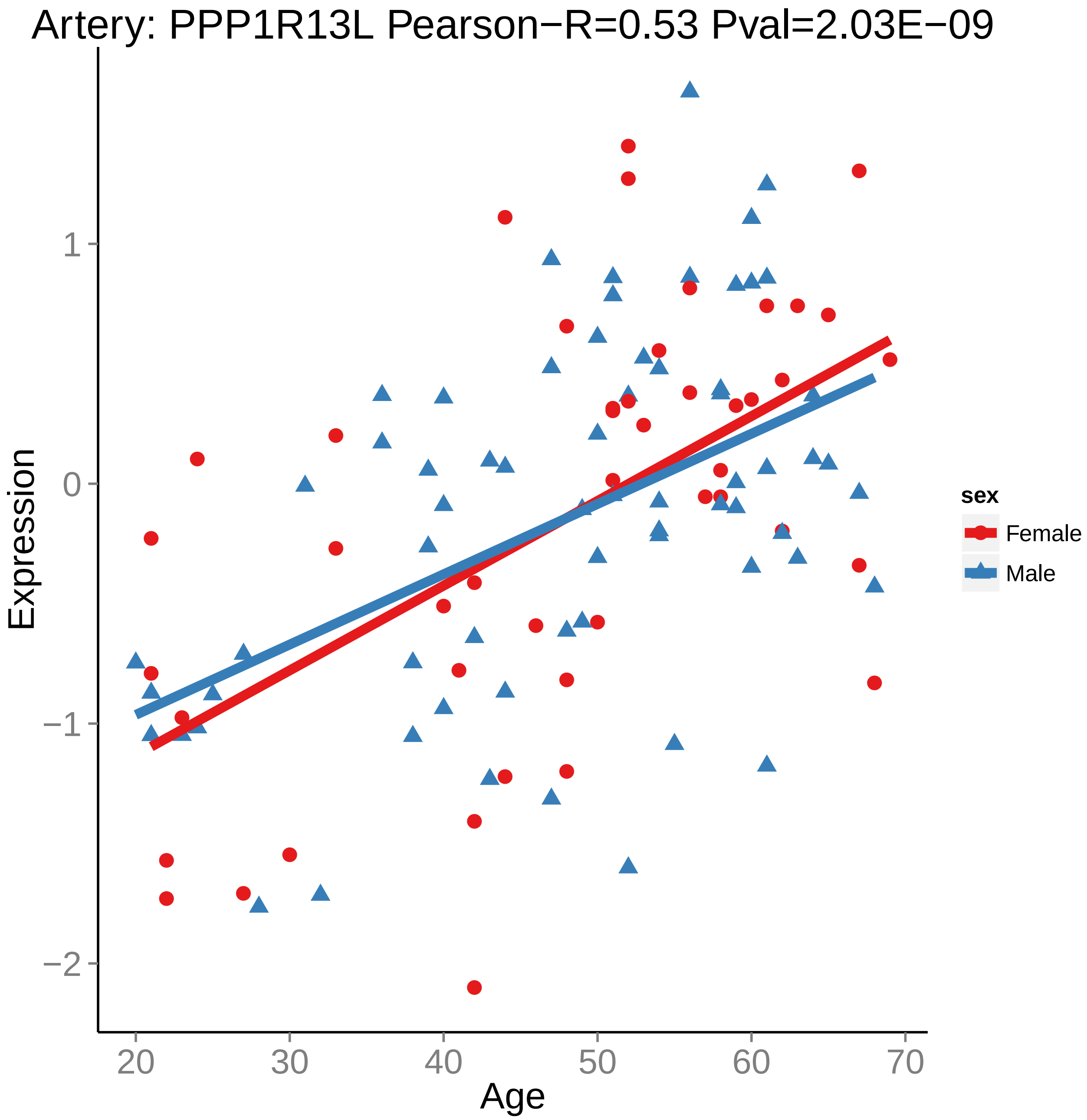

Artery: PPP1R13L Pearson−R=0.53 Pval=2.03E−09
Expression
1
0
−1
−2
Age
20
30
40
50
60
70
sex
Female
Male

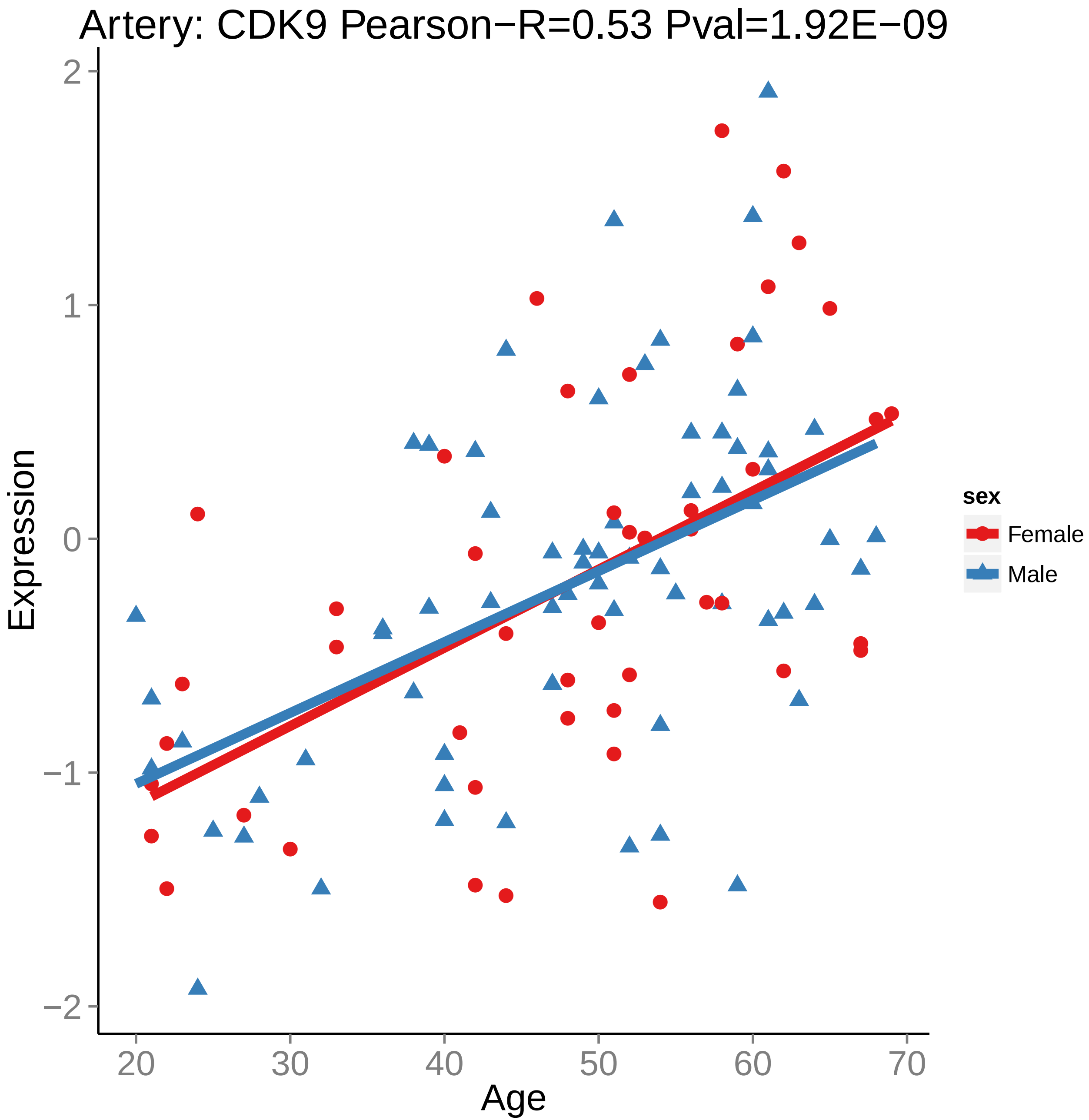
Artery: CDK9 Pearson−R=0.53 Pval=1.92E−09
Expression
2
1
0
−1
−2
20
30
40
50
60
70
Age
sex
Female
Male

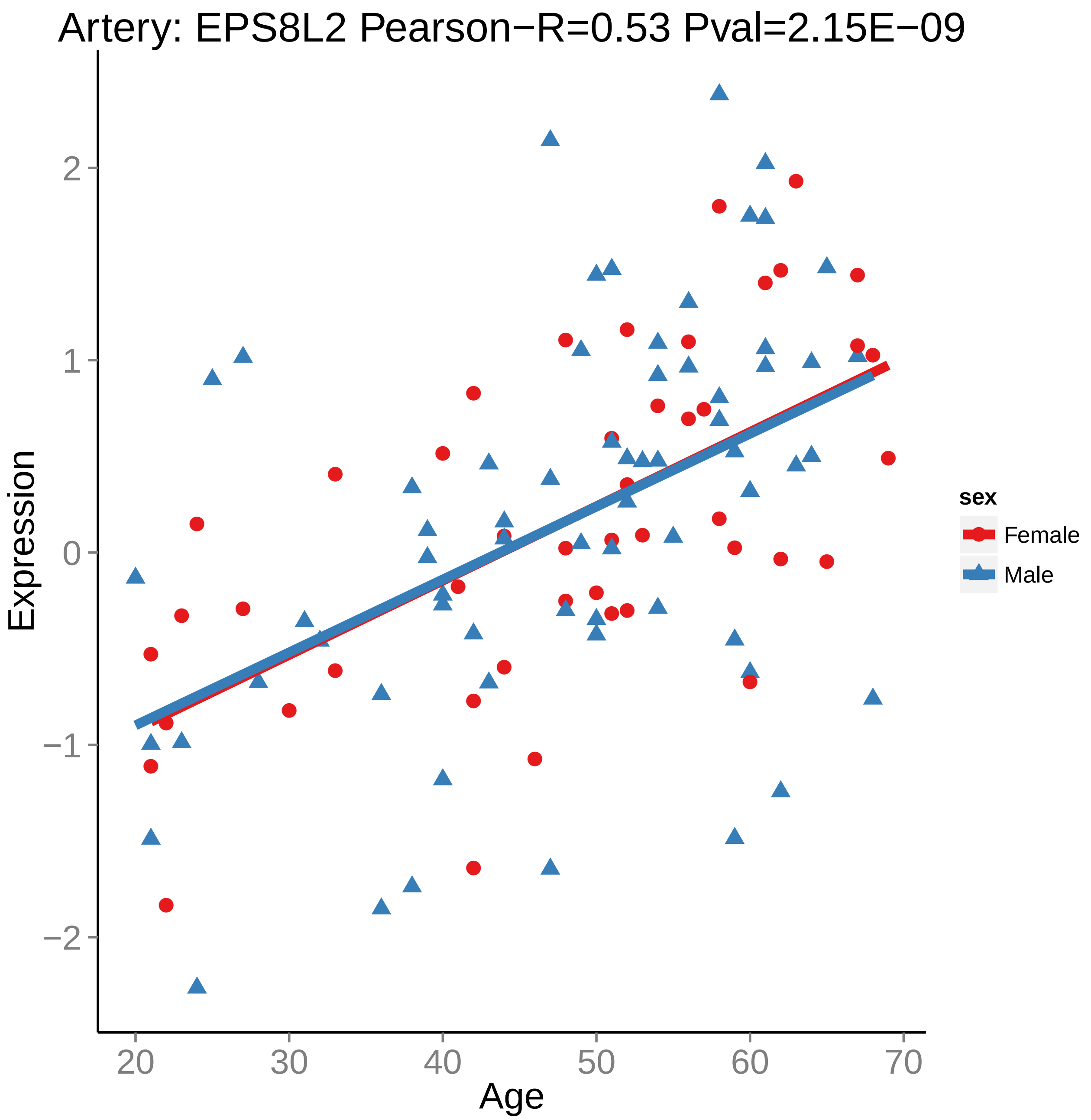

Artery: EPS8L2 Pearson−R=0.53 Pval=2.15E−09
Expression
Age
2
1
0
−1
−2
20
30
40
50
60
70
sex
Female
Male

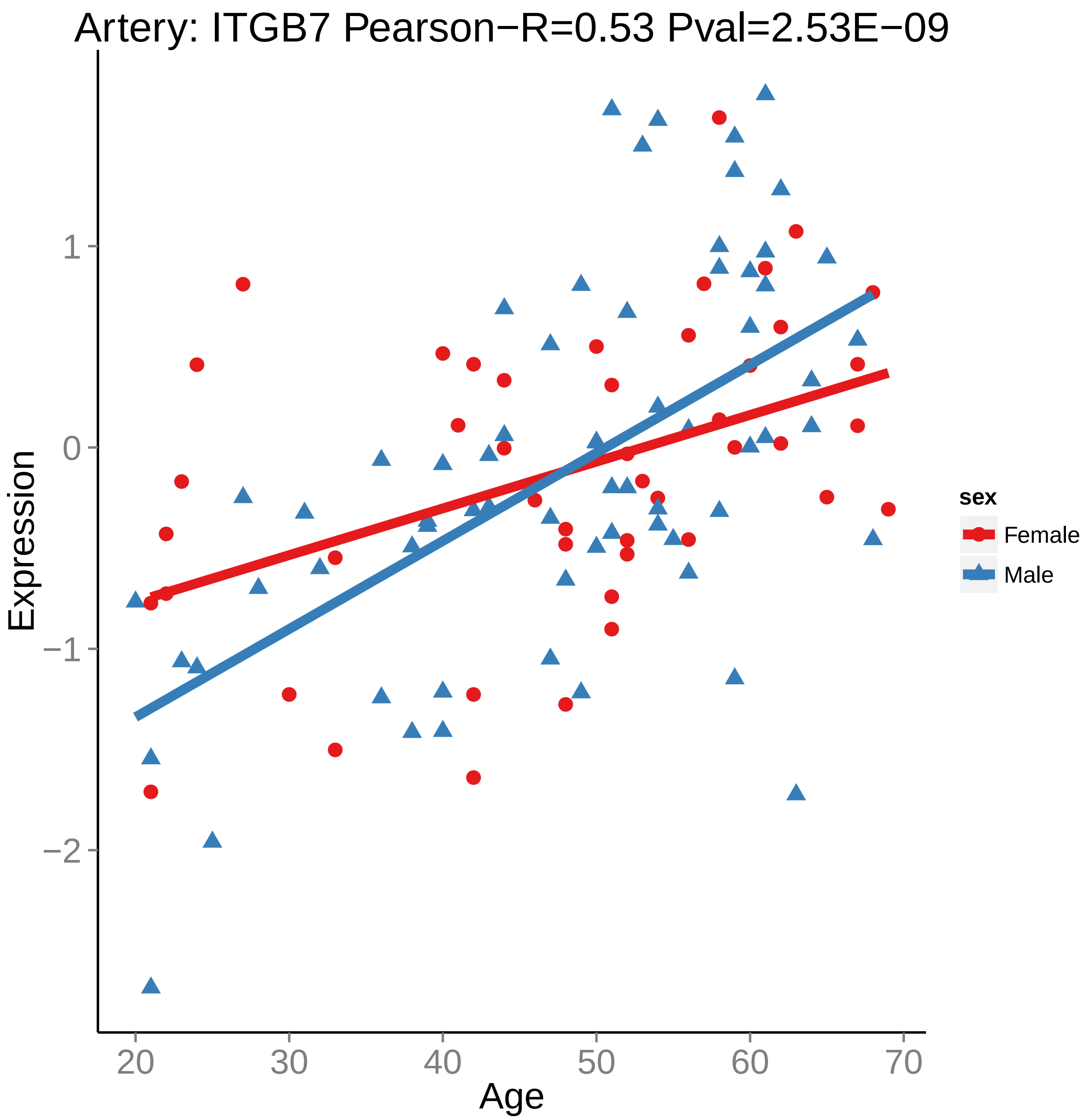

Artery: ITGB7 Pearson−R=0.53 Pval=2.53E−09
Expression
Age
1
0
−1
−2
20
30
40
50
60
70
sex
Female
Male

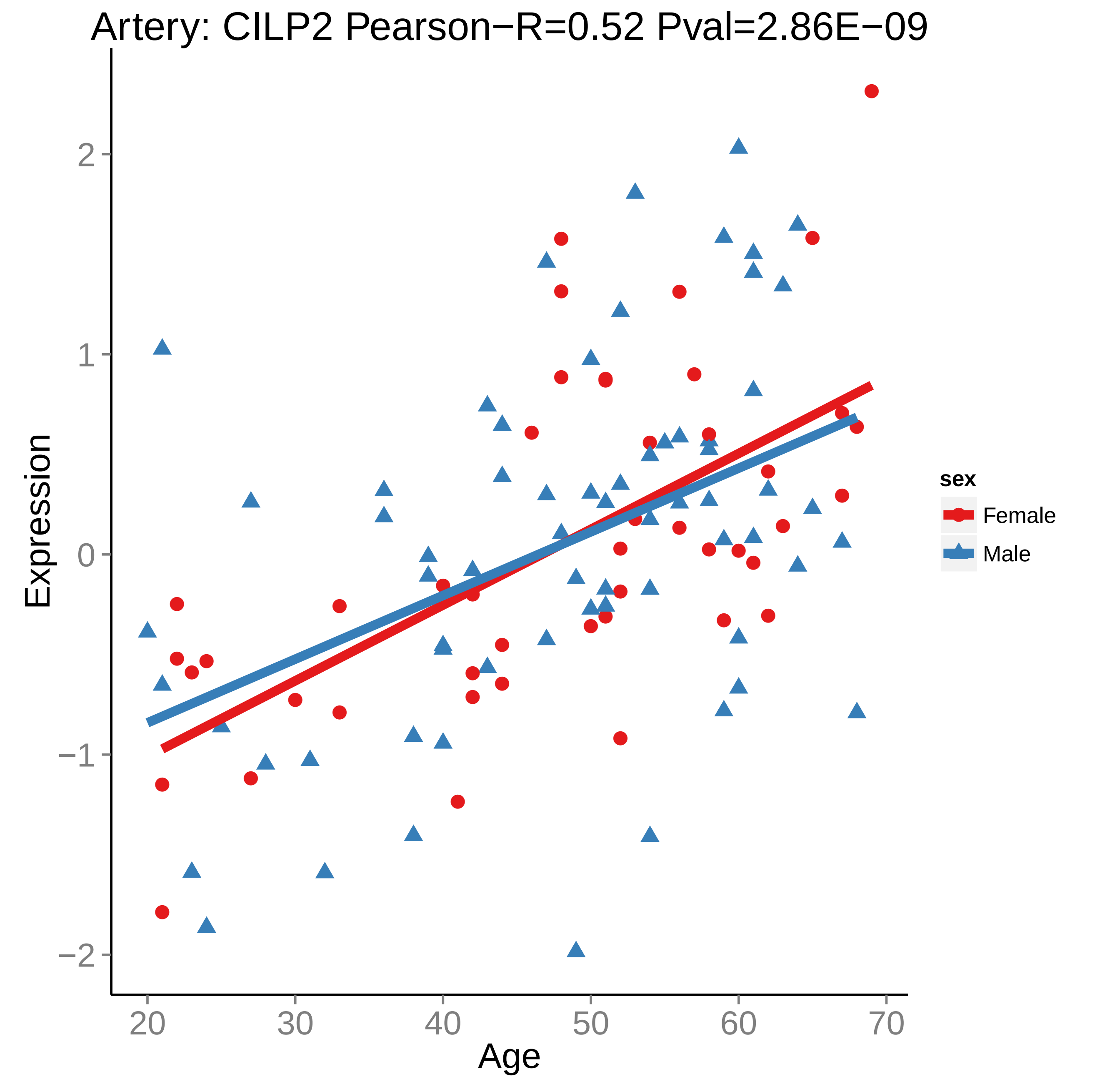
Artery: CILP2 Pearson−R=0.52 Pval=2.86E−09
Expression
2
1
0
−1
−2
20
30
40
50
60
70
Age
sex
Female
Male

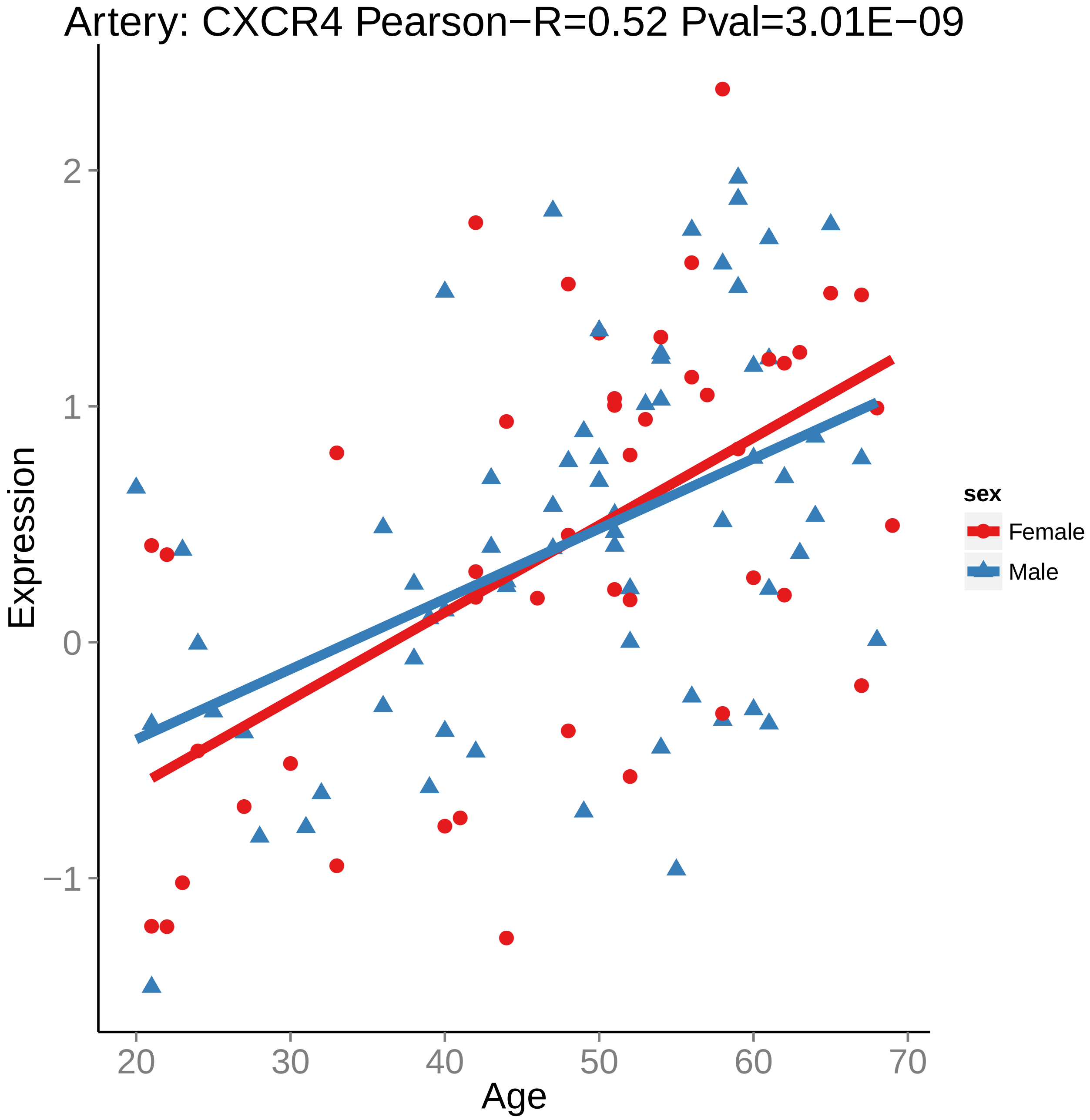

Artery: CXCR4 Pearson−R=0.52 Pval=3.01E−09
Expression
2
1
0
−1
20
30
40
50
60
70
Age
sex
Female
Male

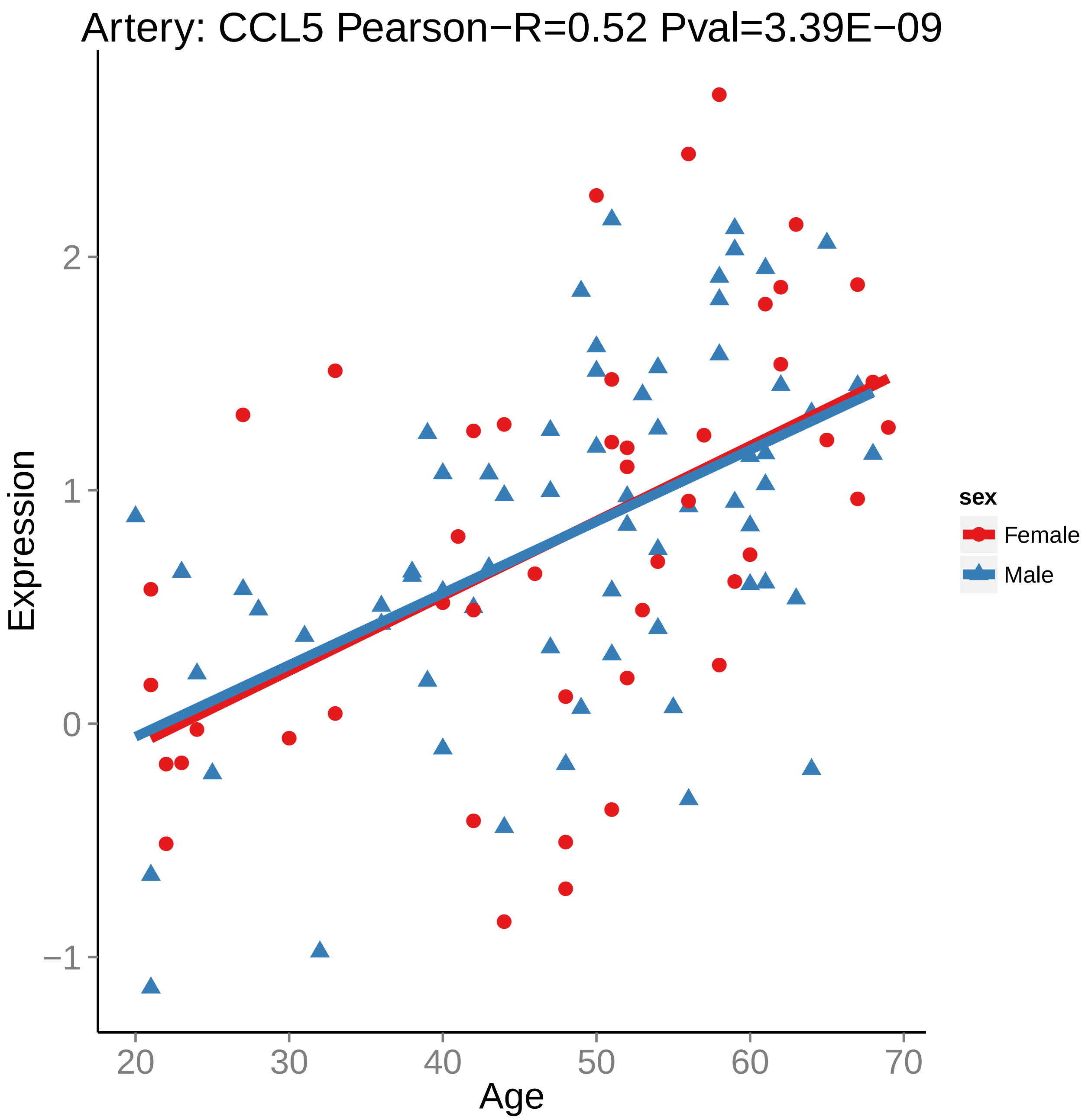

Artery: CCL5 Pearson−R=0.52 Pval=3.39E−09
Expression
2
1
0
−1
20
30
40
50
60
70
Age
sex
Female
Male

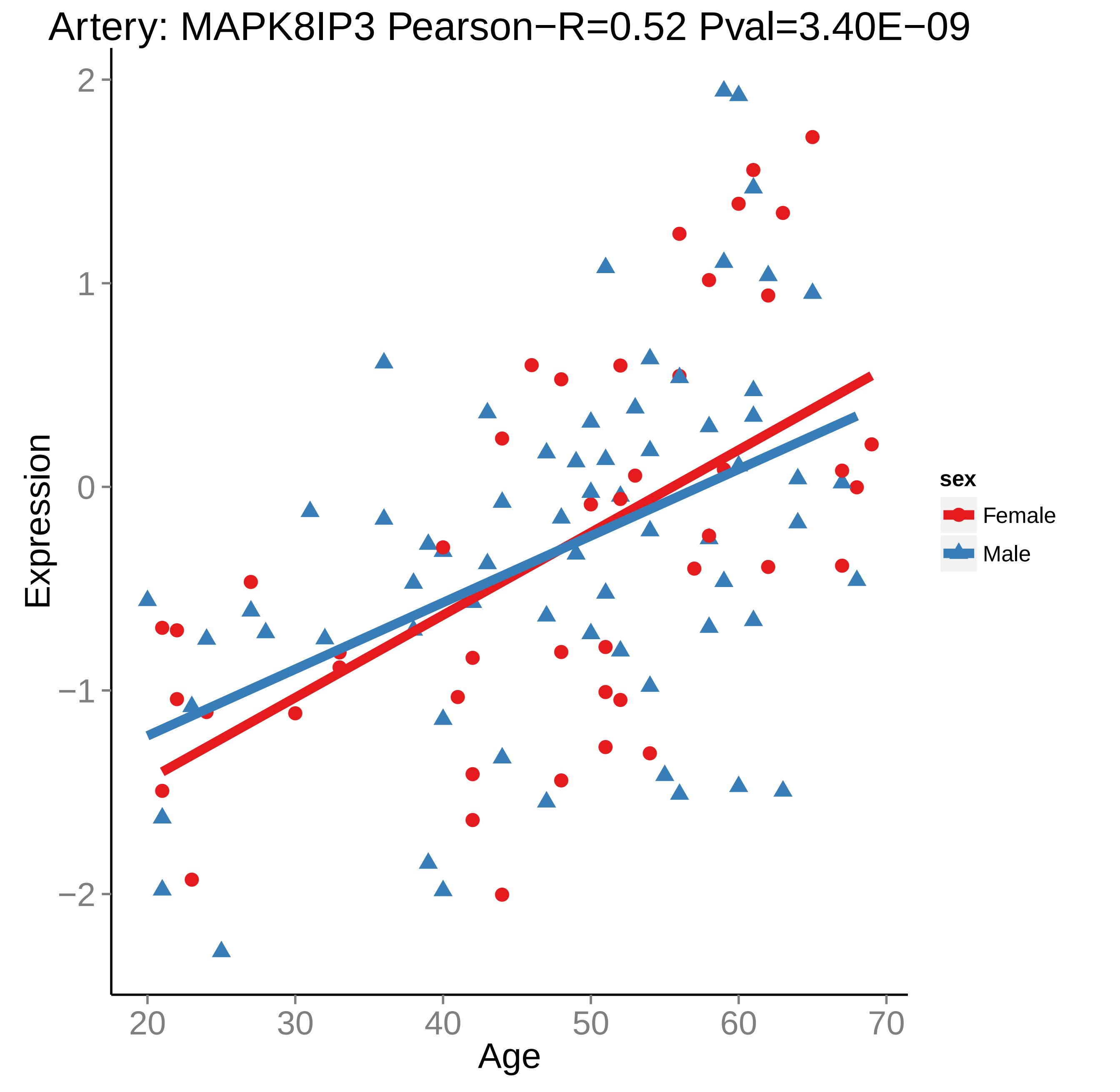

Artery: MAPK8IP3 Pearson−R=0.52 Pval=3.40E−09
Expression
Age
2
1
0
−1
−2
20
30
40
50
60
70
sex
Female
Male

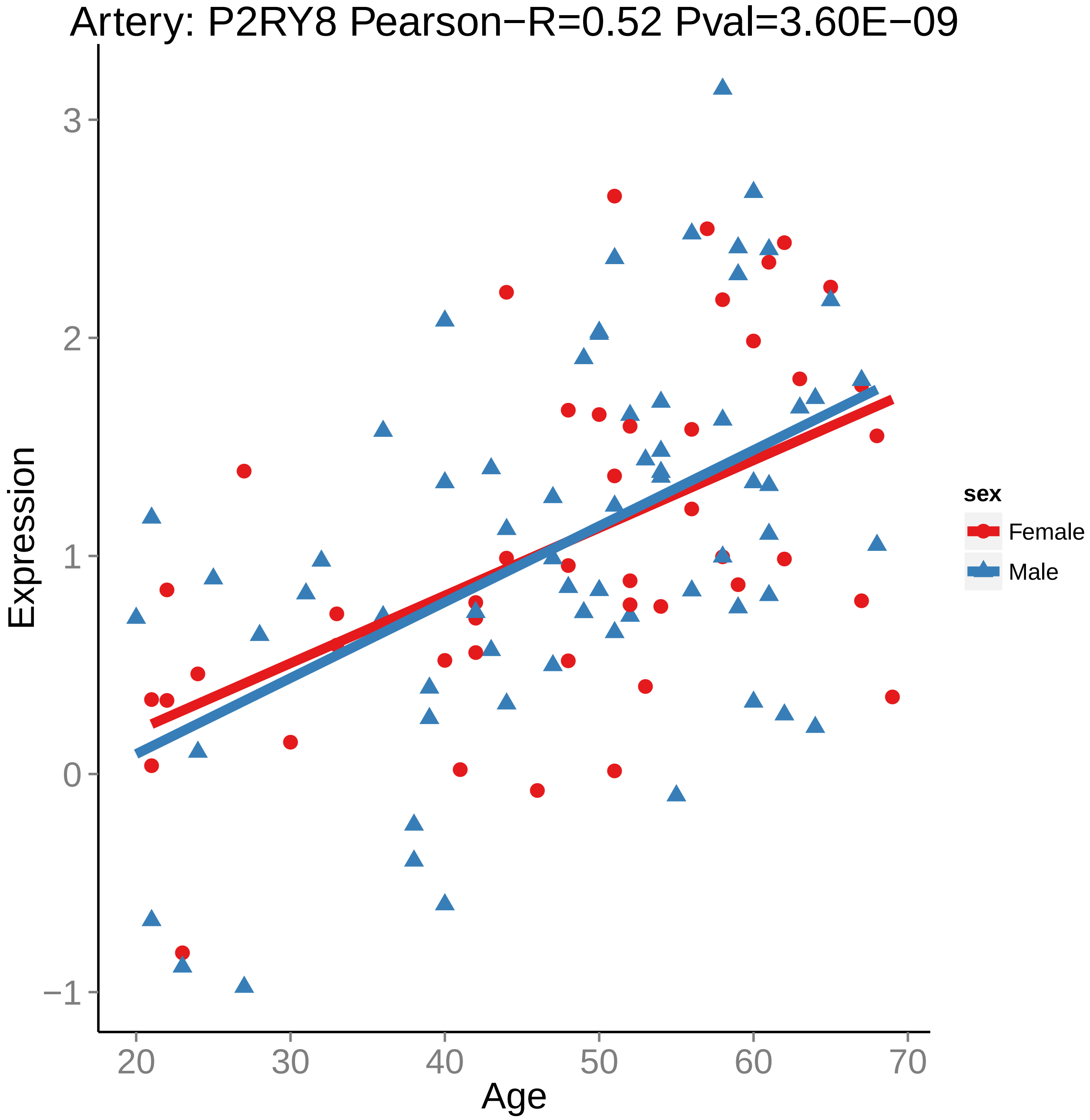

Artery: P2RY8 Pearson−R=0.52 Pval=3.60E−09
Expression
Age
3
2
1
0
−1
20
30
40
50
60
70
sex
Female
Male

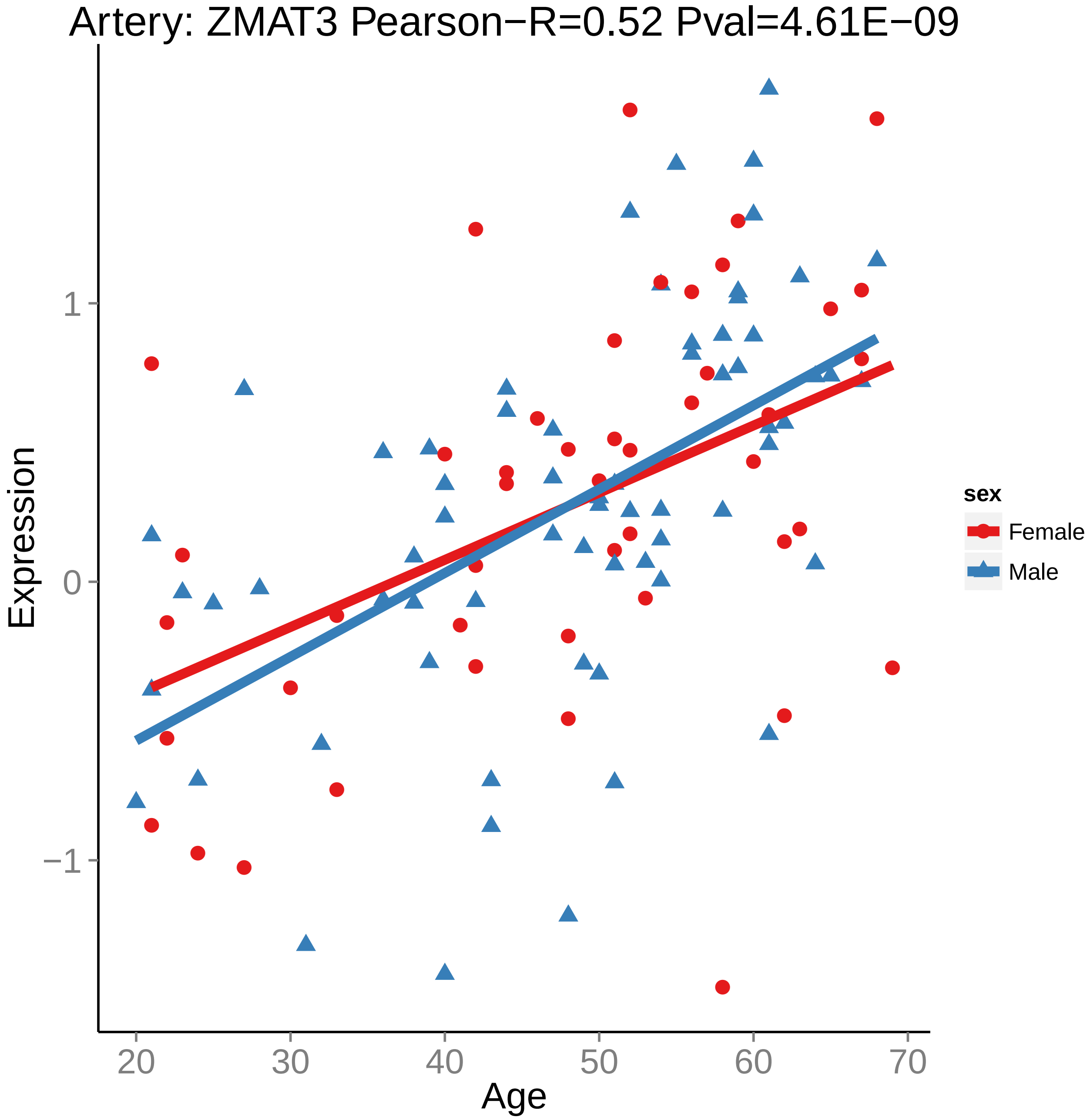

Artery: ZMAT3 Pearson−R=0.52 Pval=4.61E−09
Expression
1
0
−1
20
30
40
50
60
70
Age
sex
Female
Male

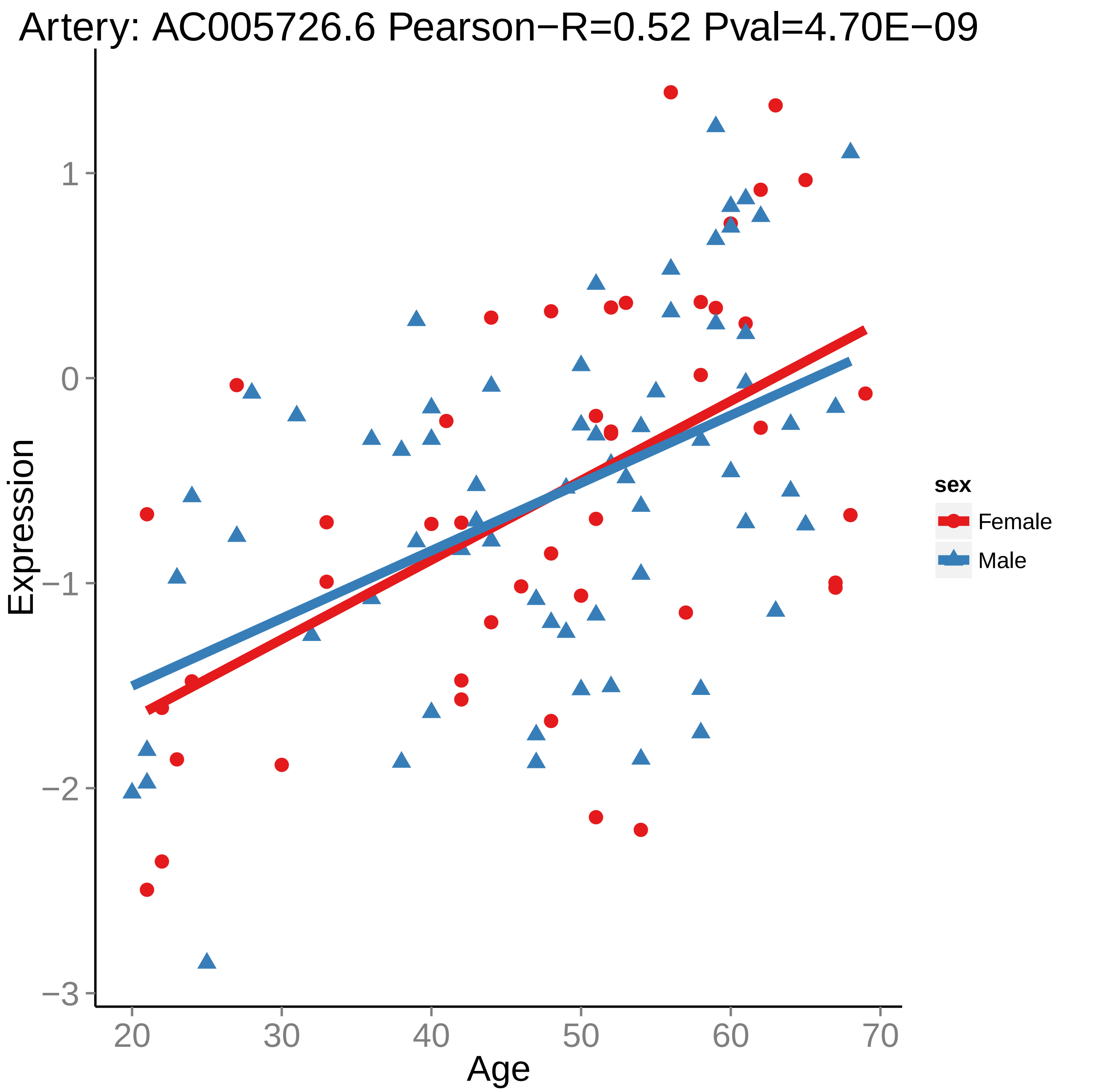

Artery: AC005726.6 Pearson−R=0.52 Pval=4.70E−09
Expression
1
0
−1
−2
−3
20
30
40
50
60
70
Age
sex
Female
Male

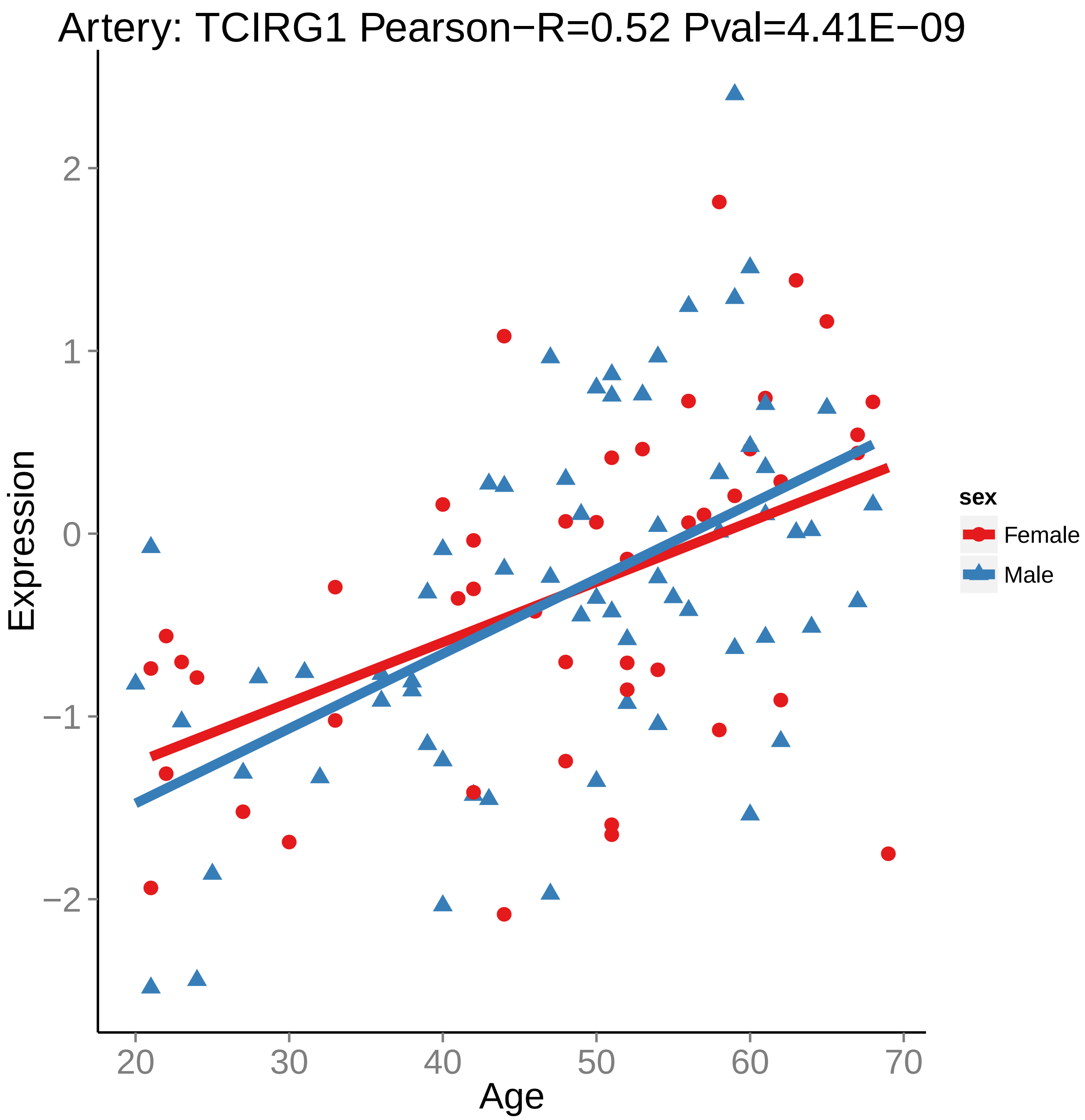

Artery: TCIRG1 Pearson−R=0.52 Pval=4.41E−09
Expression
Age
2
1
0
−1
−2
20
30
40
50
60
70
sex
Female
Male

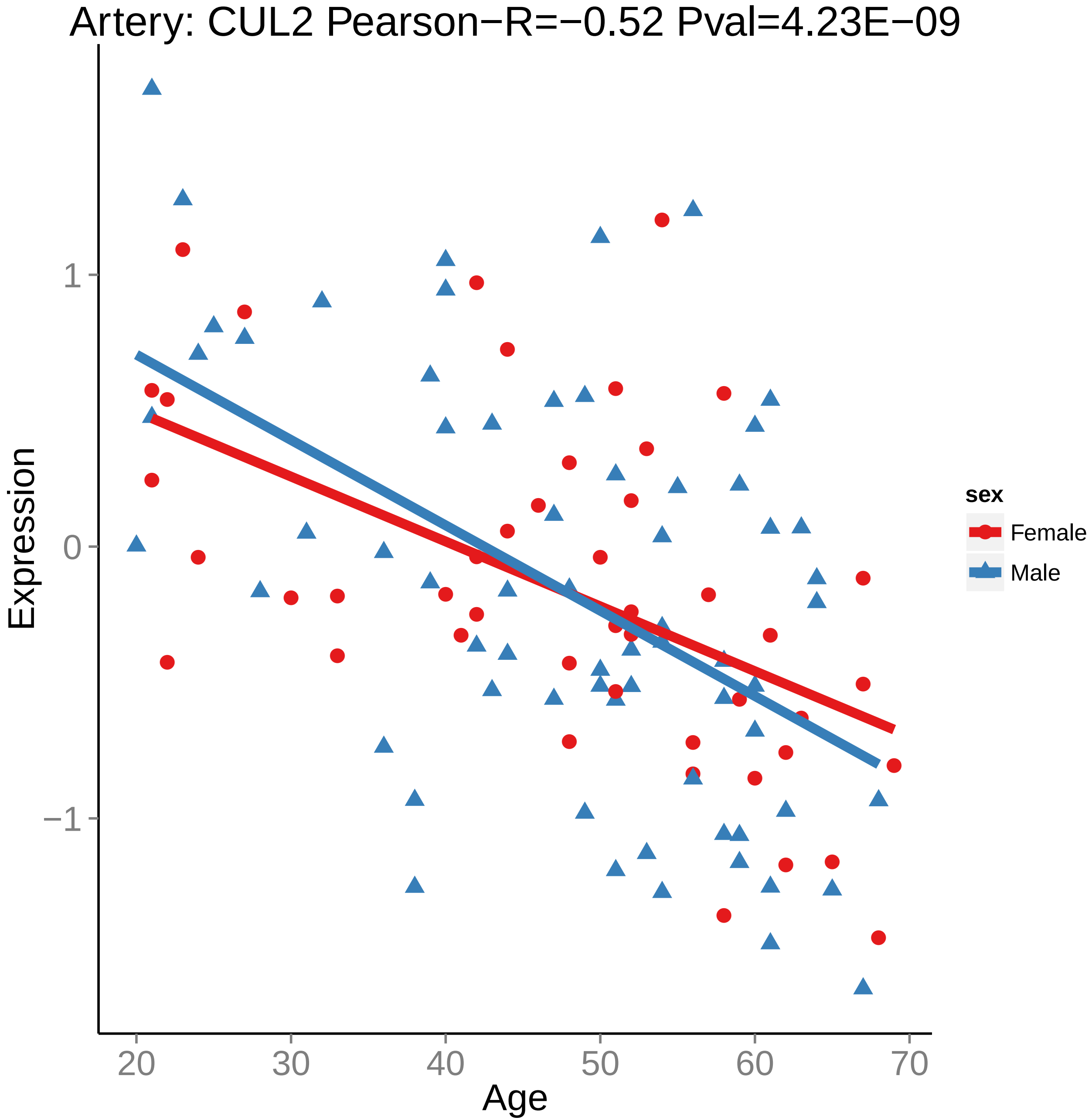
Artery: CUL2 Pearson−R=−0.52 Pval=4.23E−09
Expression
1
0
−1
20
30
40
50
60
70
Age
sex
Female
Male

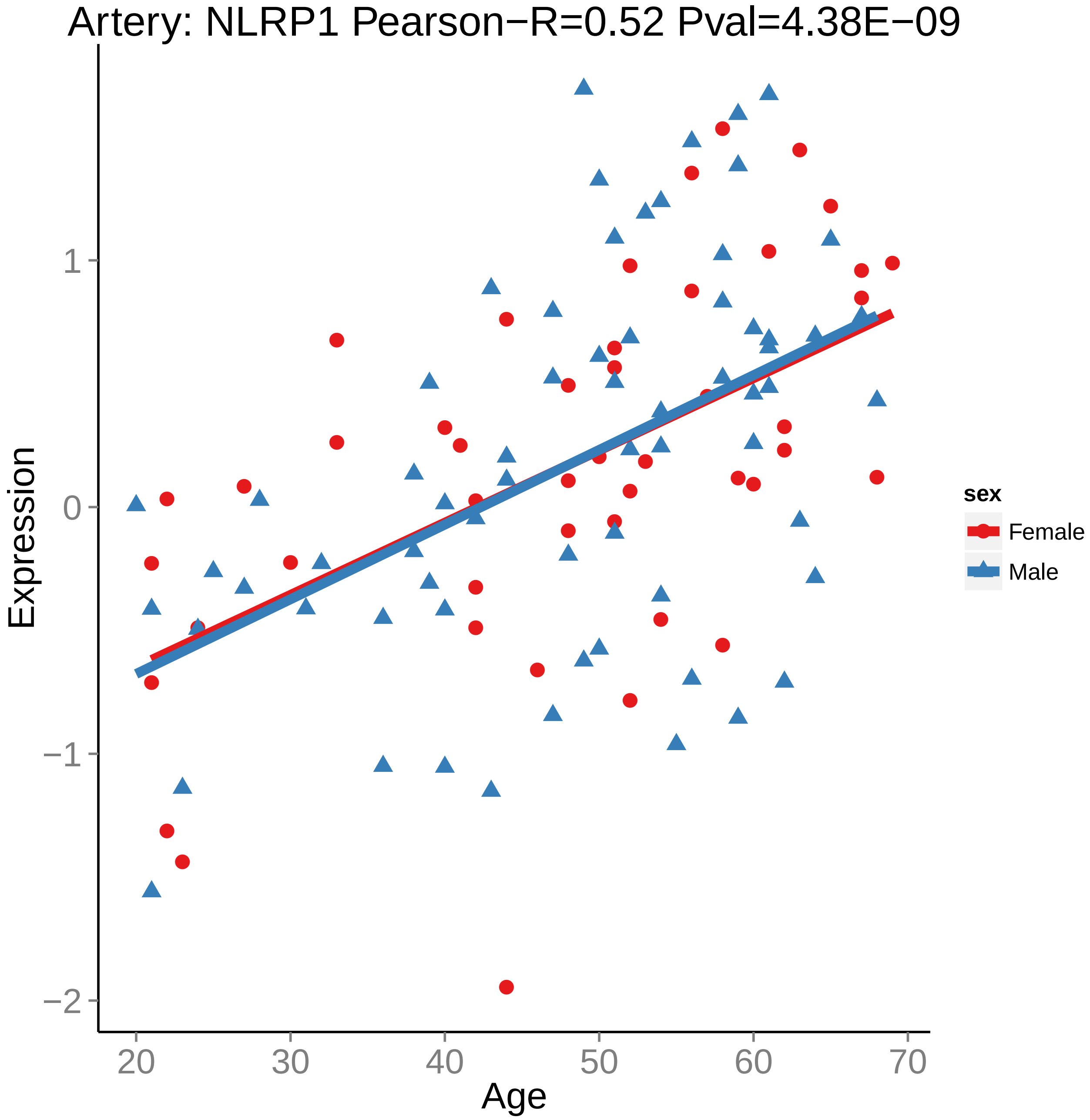

Artery: NLRP1 Pearson−R=0.52 Pval=4.38E−09
Expression
1
0
−1
−2
20
30
40
50
60
70
Age
sex
Female
Male

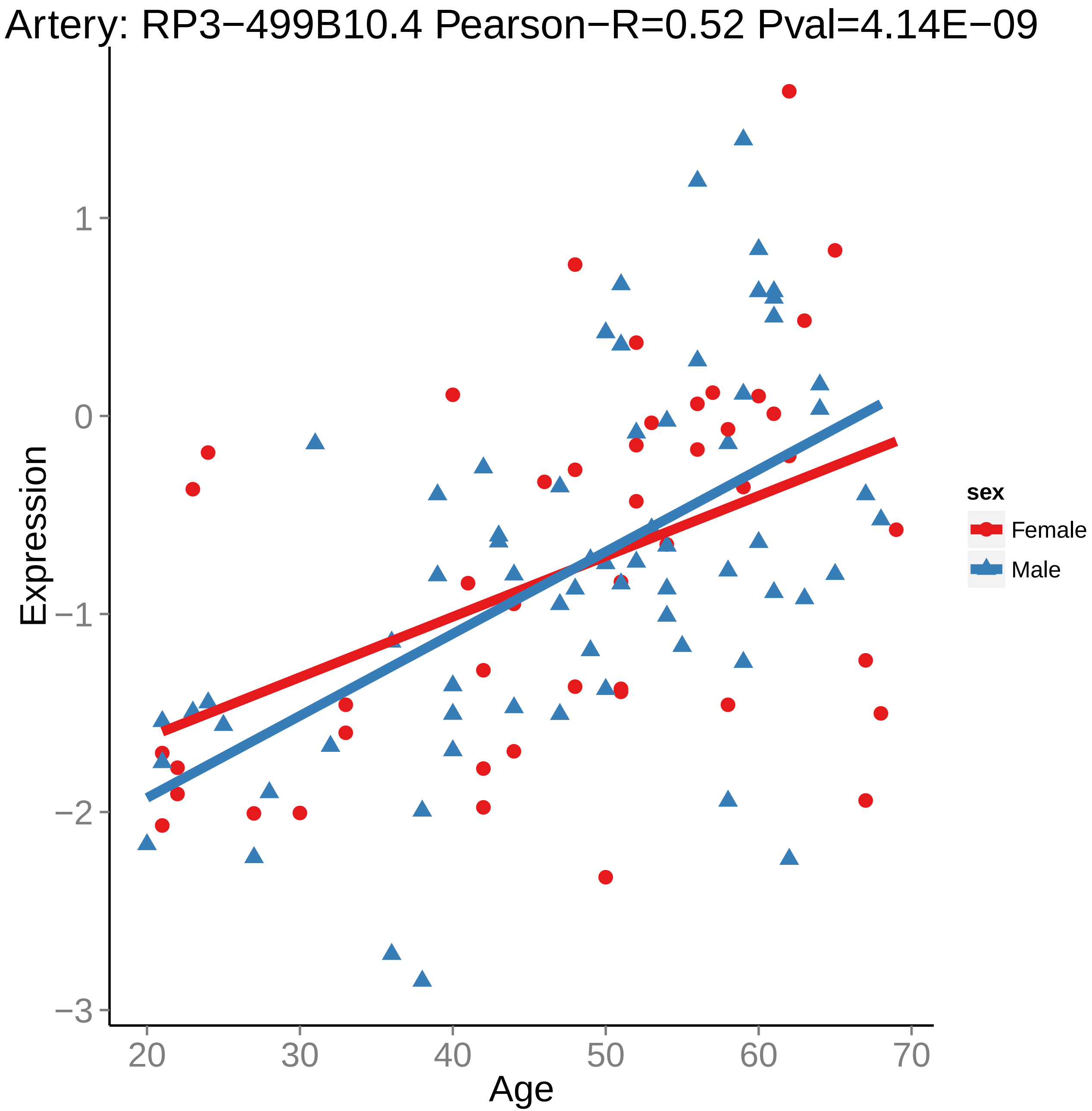

Artery: RP3−499B10.4 Pearson−R=0.52 Pval=4.14E−09
Expression
1
0
−1
−2
−3
20
30
40
50
60
70
Age
sex
Female
Male

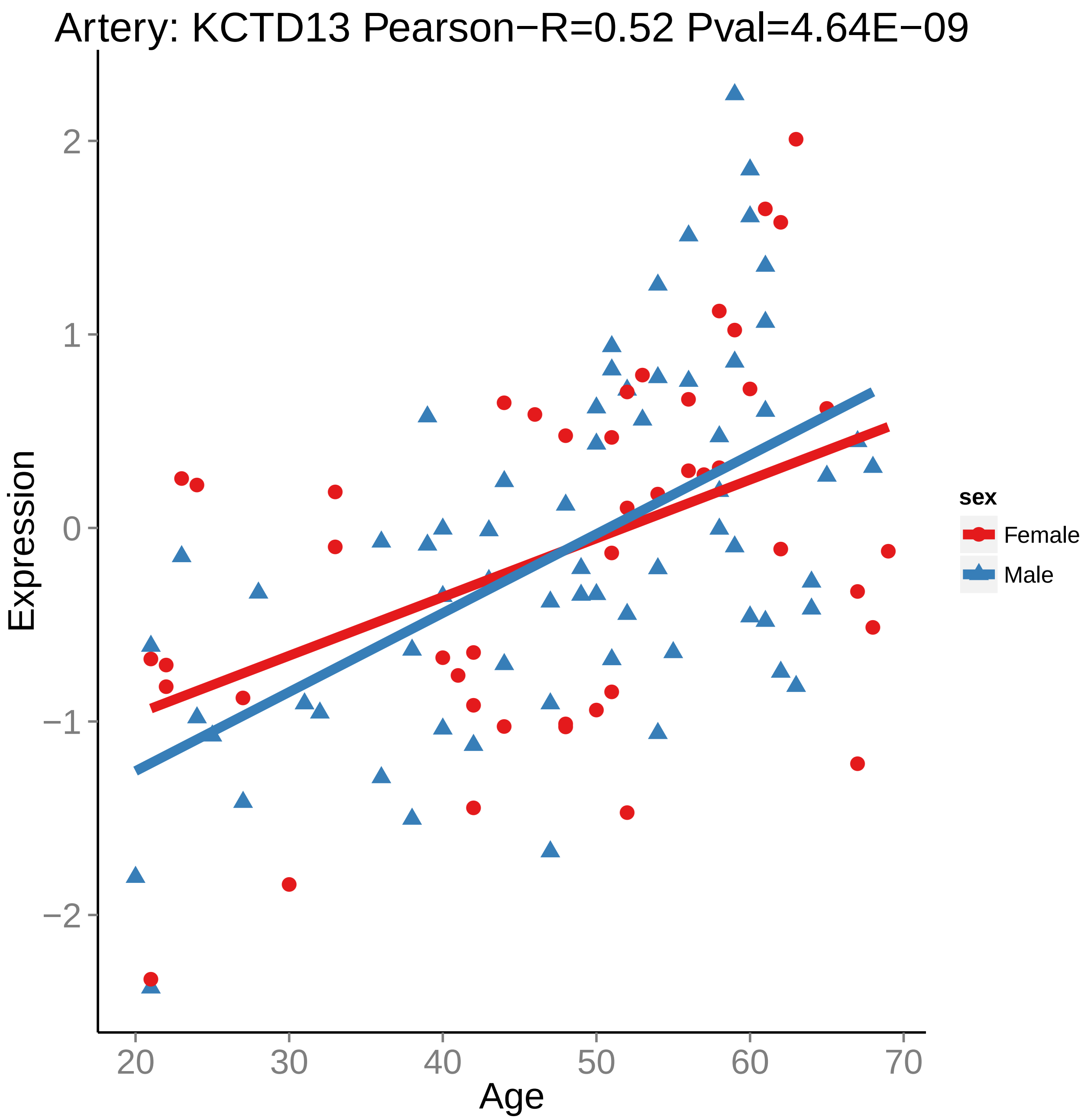
Artery: KCTD13 Pearson−R=0.52 Pval=4.64E−09
Expression
Age
2
1
0
−1
−2
20
30
40
50
60
70
sex
Female
Male

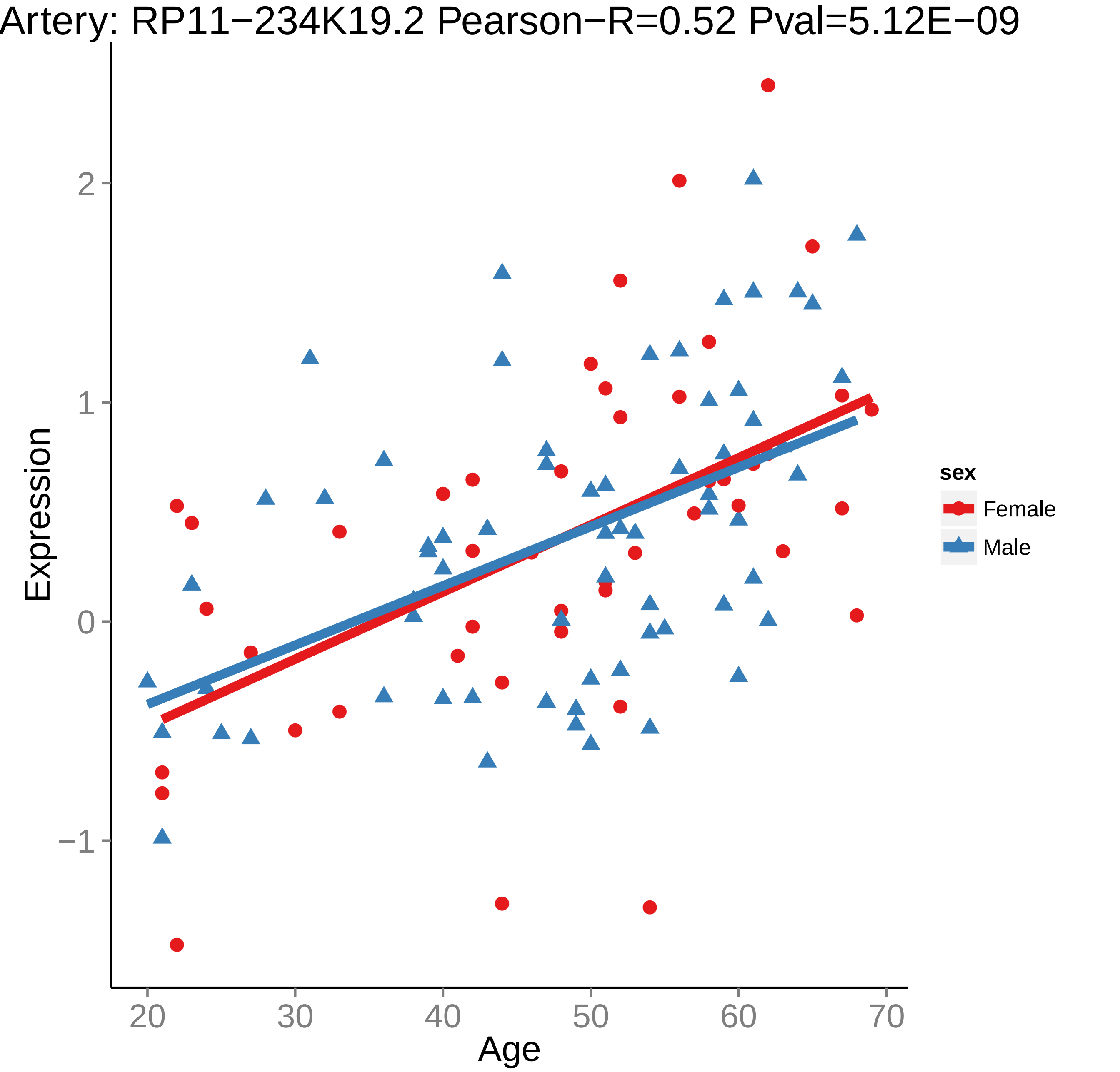

Artery: RP11−234K19.2 Pearson−R=0.52 Pval=5.12E−09
Expression
Age
sex
Female
Male
2
1
0
−1
20
30
40
50
60
70

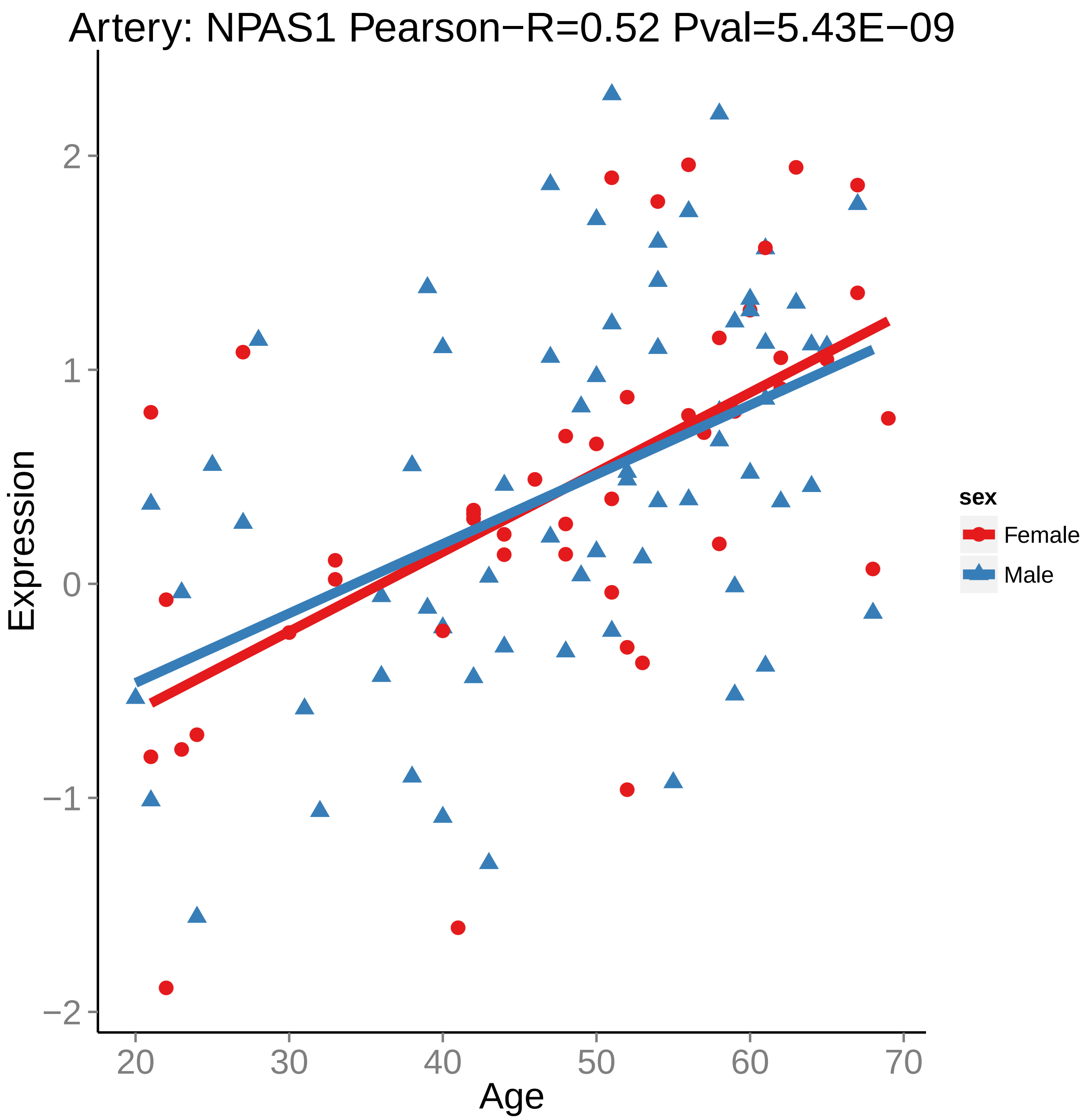
Artery: NPAS1 Pearson−R=0.52 Pval=5.43E−09
Expression
Age
2
1
0
−1
−2
20
30
40
50
60
70
sex
Female
Male

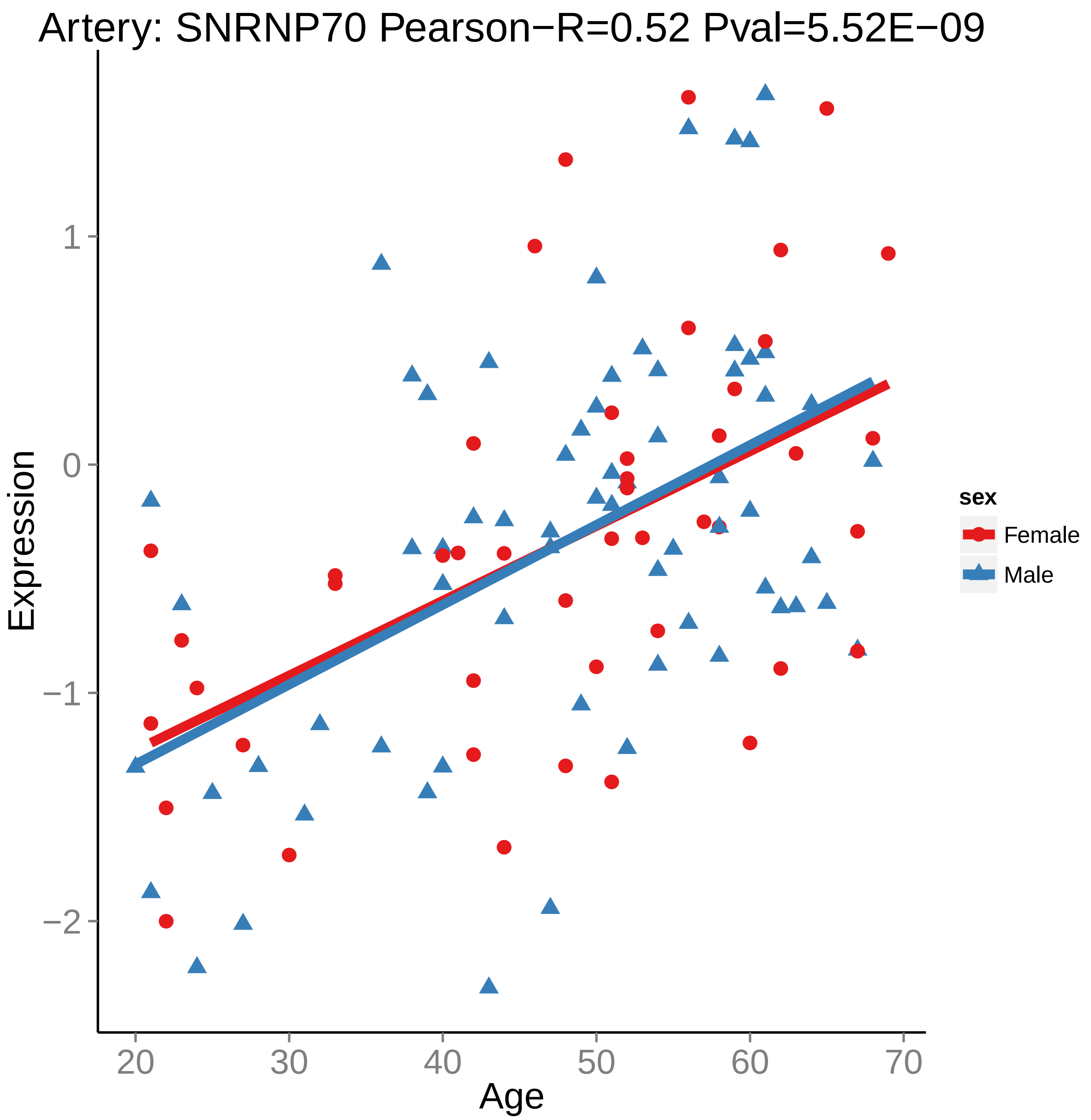

Artery: SNRNP70 Pearson−R=0.52 Pval=5.52E−09
Expression
Age
1
0
−1
−2
20
30
40
50
60
70
sex
Female
Male

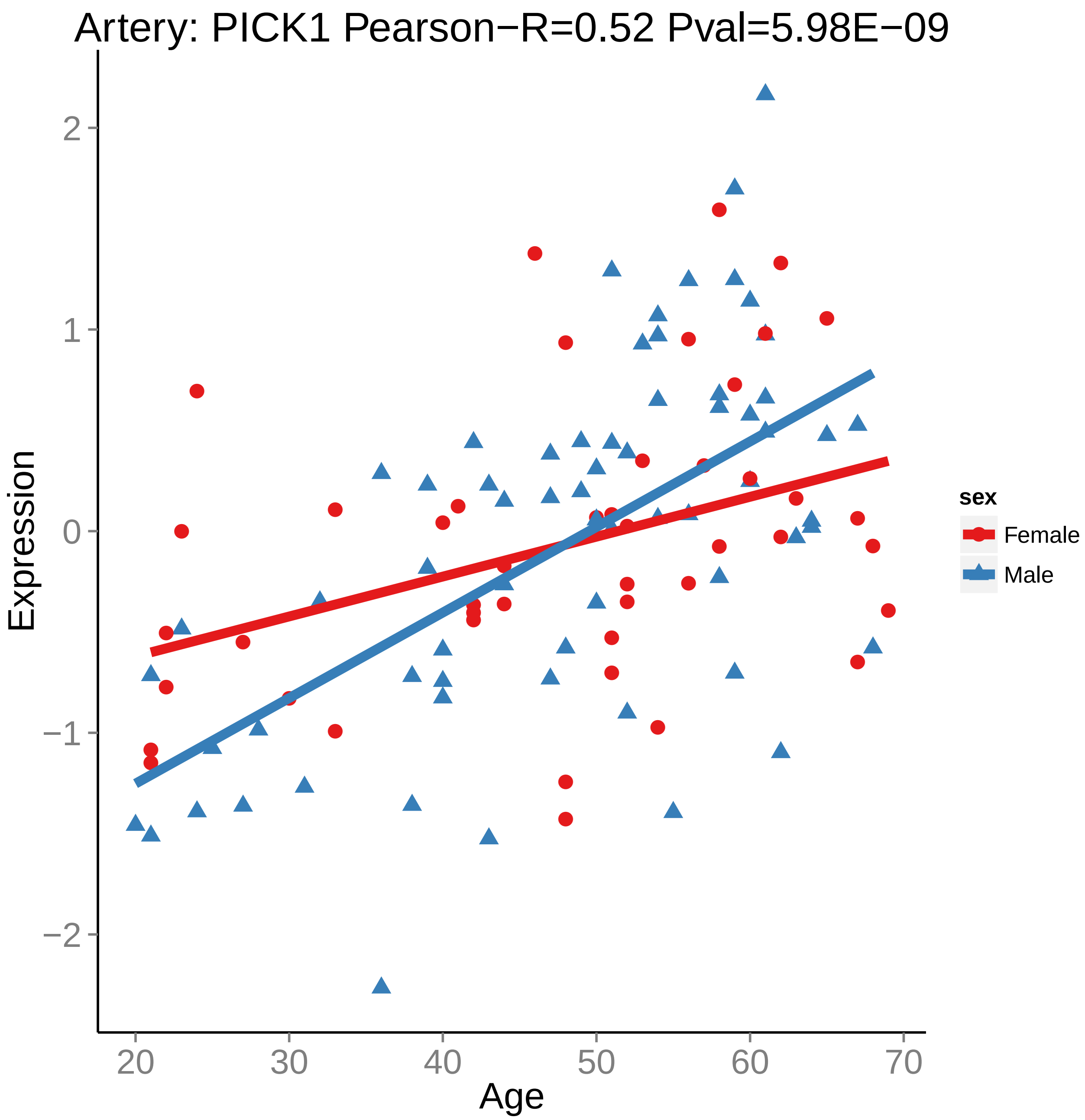

Artery: PICK1 Pearson−R=0.52 Pval=5.98E−09
Expression
2
1
0
−1
−2
Age
20
30
40
50
60
70
sex
Female
Male

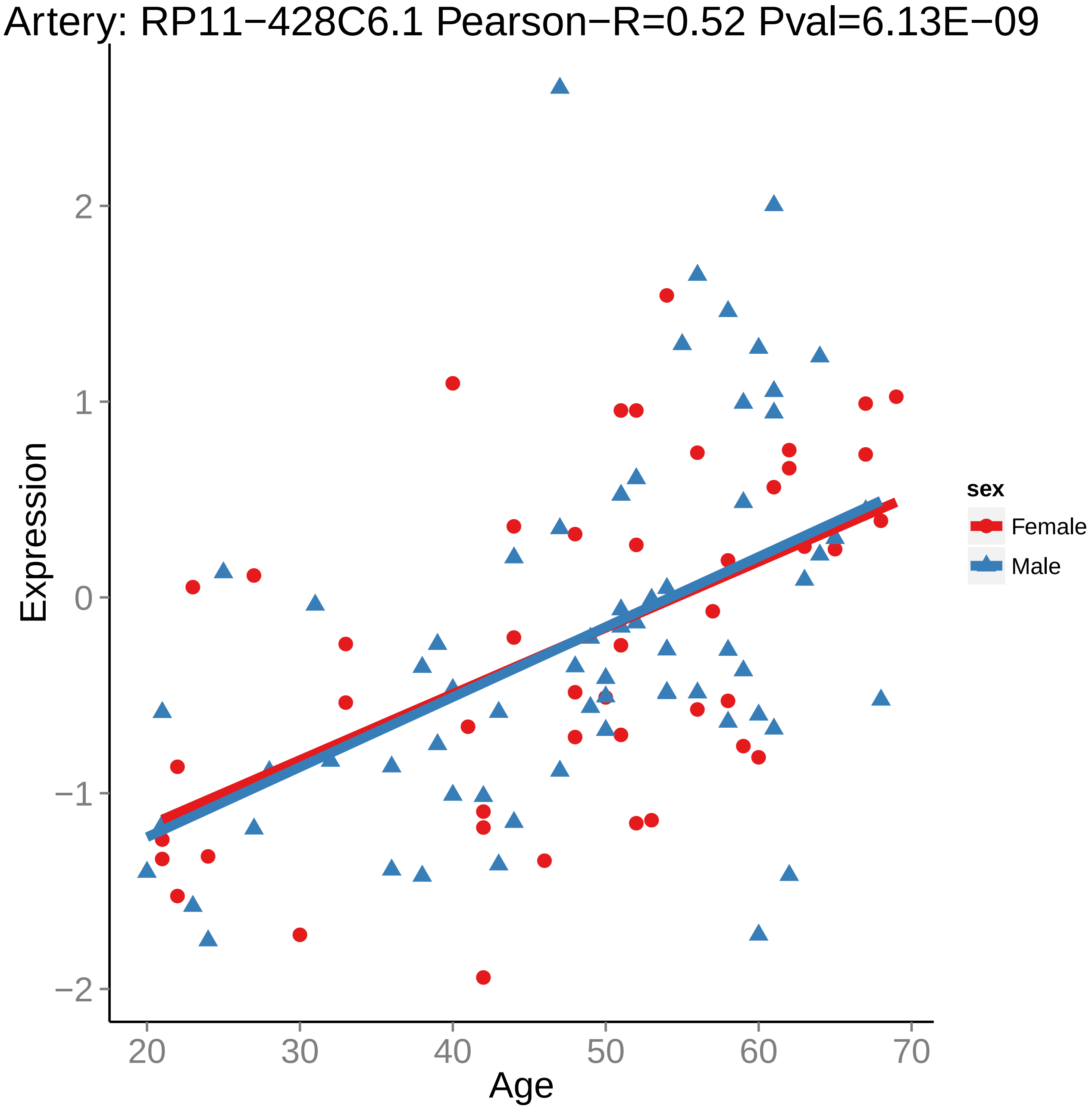

Artery: RP11−428C6.1 Pearson−R=0.52 Pval=6.13E−09
Expression
Age
sex
Female
Male
2
1
0
−1
−2
20
30
40
50
60
70

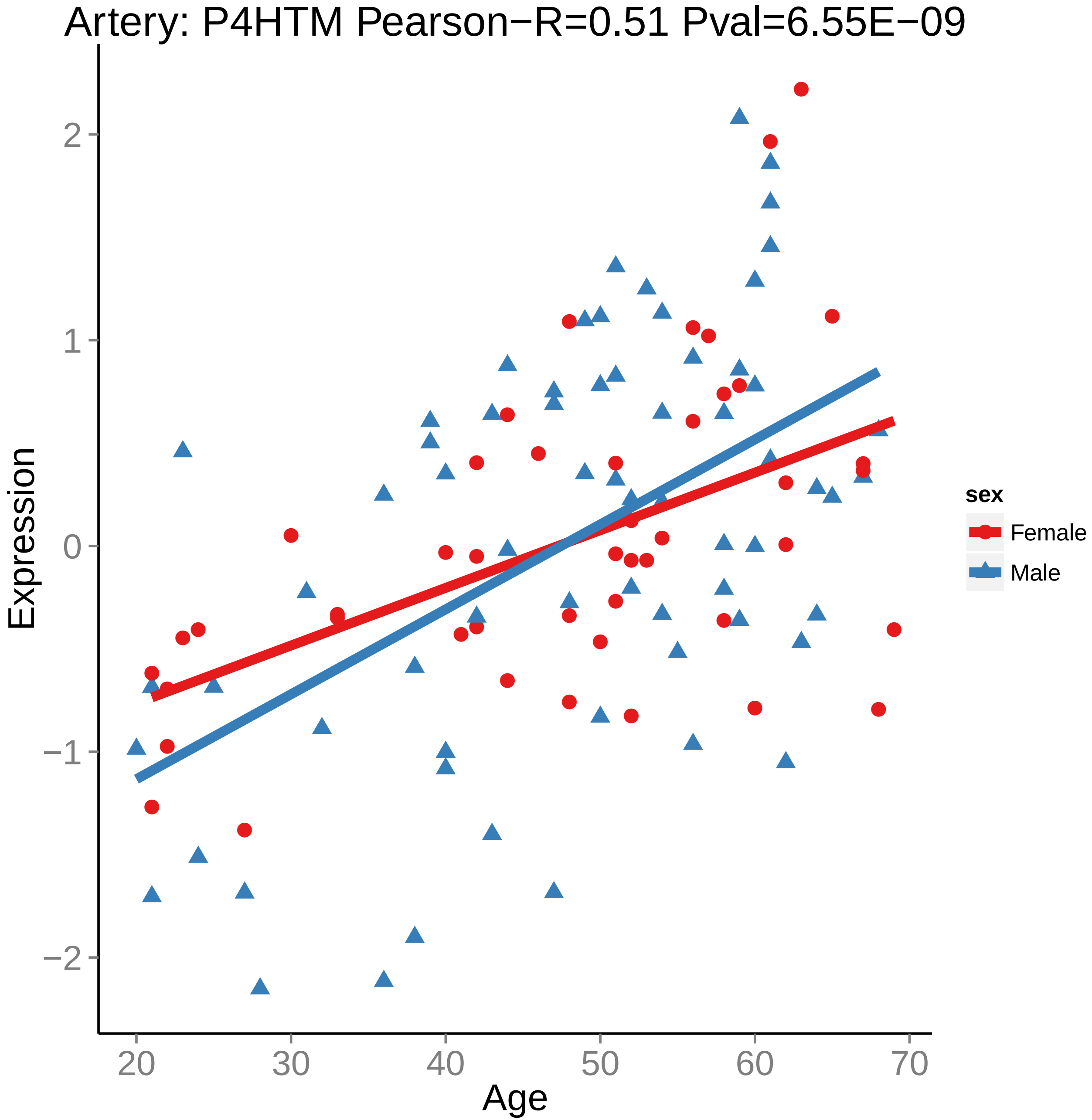

Artery: P4HTM Pearson−R=0.51 Pval=6.55E−09
Expression
Age
2
1
0
−1
−2
20
30
40
50
60
70
sex
Female
Male

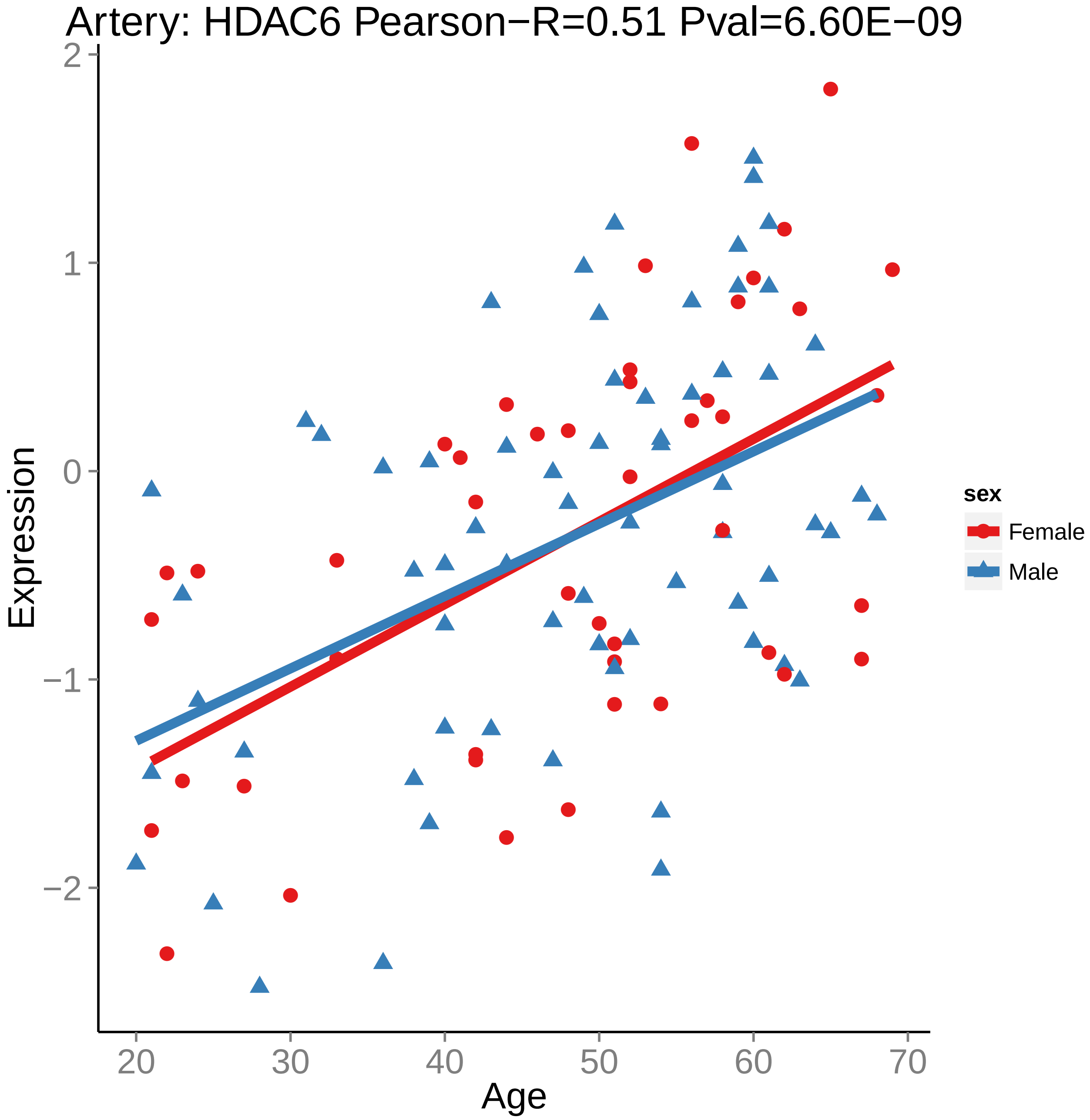

Artery: HDAC6 Pearson−R=0.51 Pval=6.60E−09
Expression
2
1
0
−1
−2
20
30
40
50
60
70
Age
sex
Female
Male

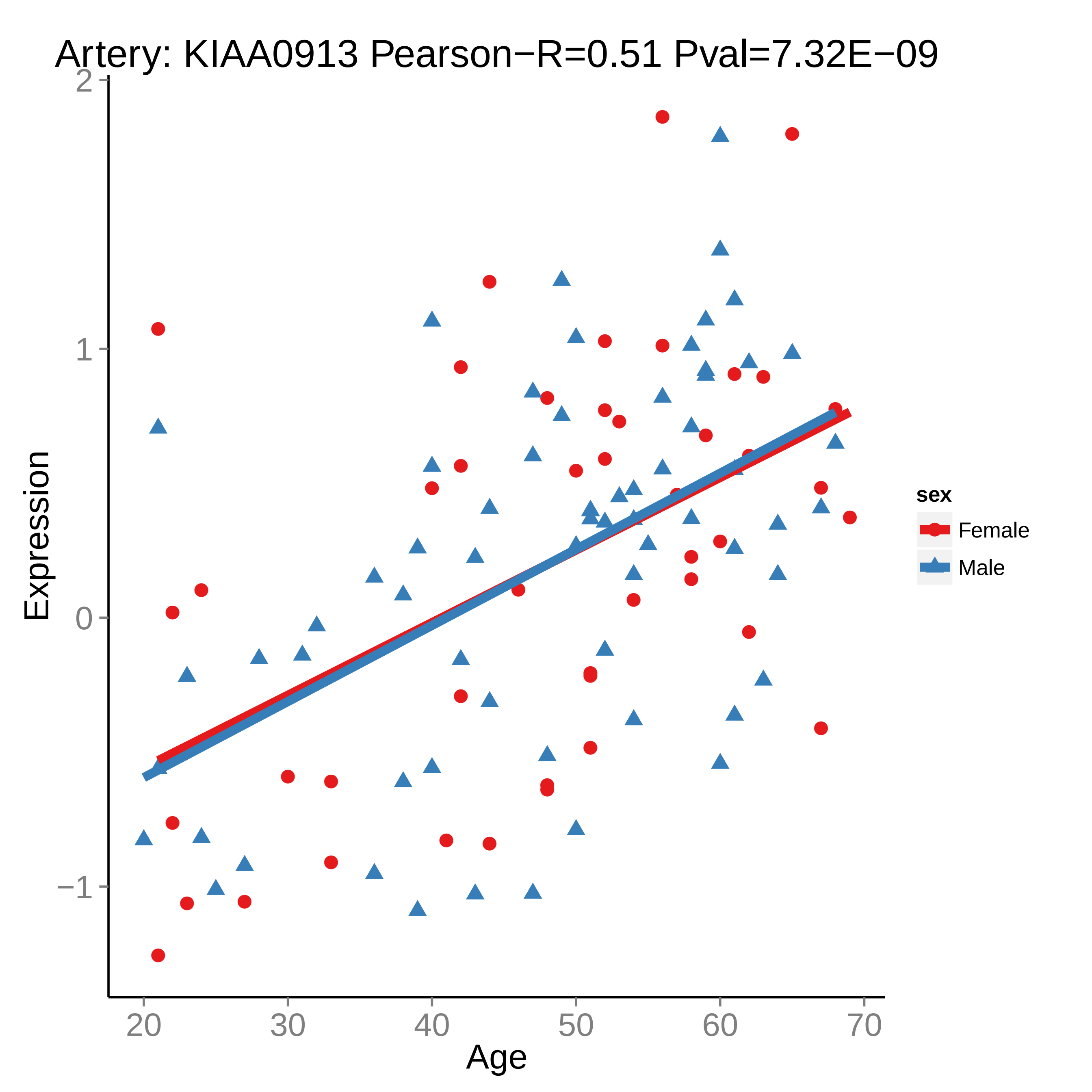

Artery: KIAA0913 Pearson−R=0.51 Pval=7.32E−09
Expression
Age
2
1
0
−1
20
30
40
50
60
70
sex
Female
Male

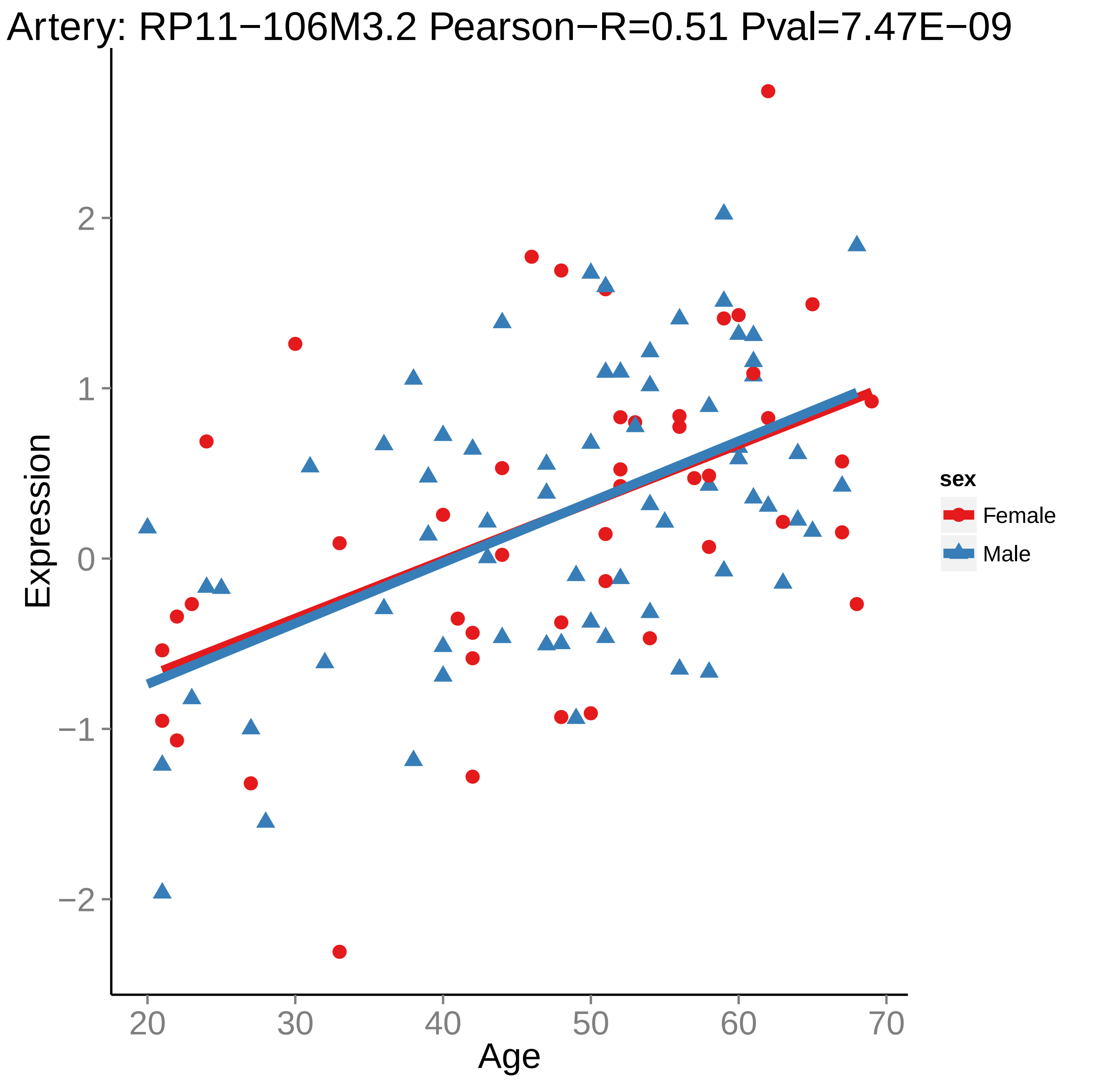
Artery: RP11−106M3.2 Pearson−R=0.51 Pval=7.47E−09
Expression
Age
2
1
0
−1
−2
20
30
40
50
60
70
sex
Female
Male

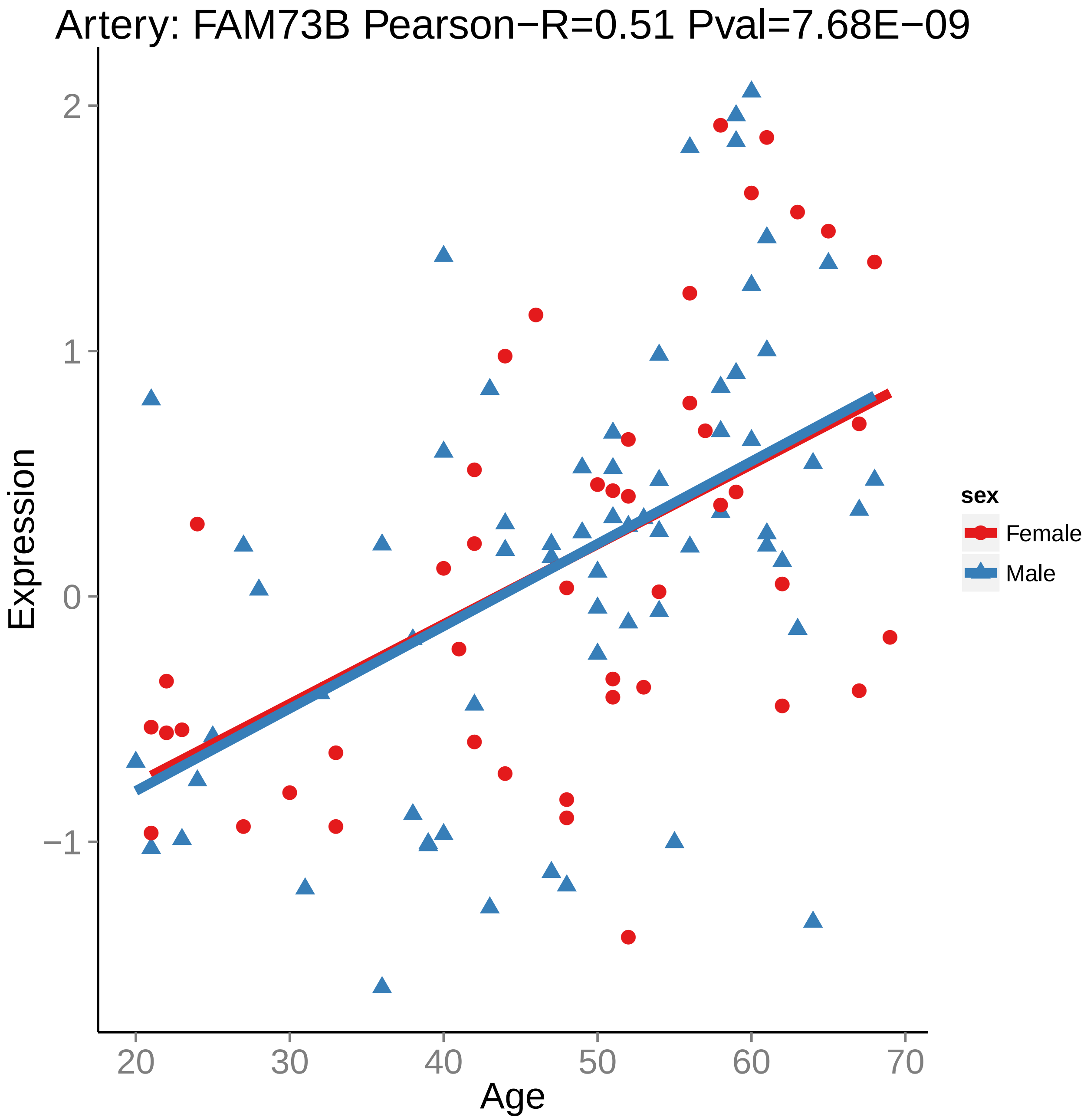

Artery: FAM73B Pearson−R=0.51 Pval=7.68E−09
Expression
Age
2
1
0
−1
20
30
40
50
60
70
sex
Female
Male

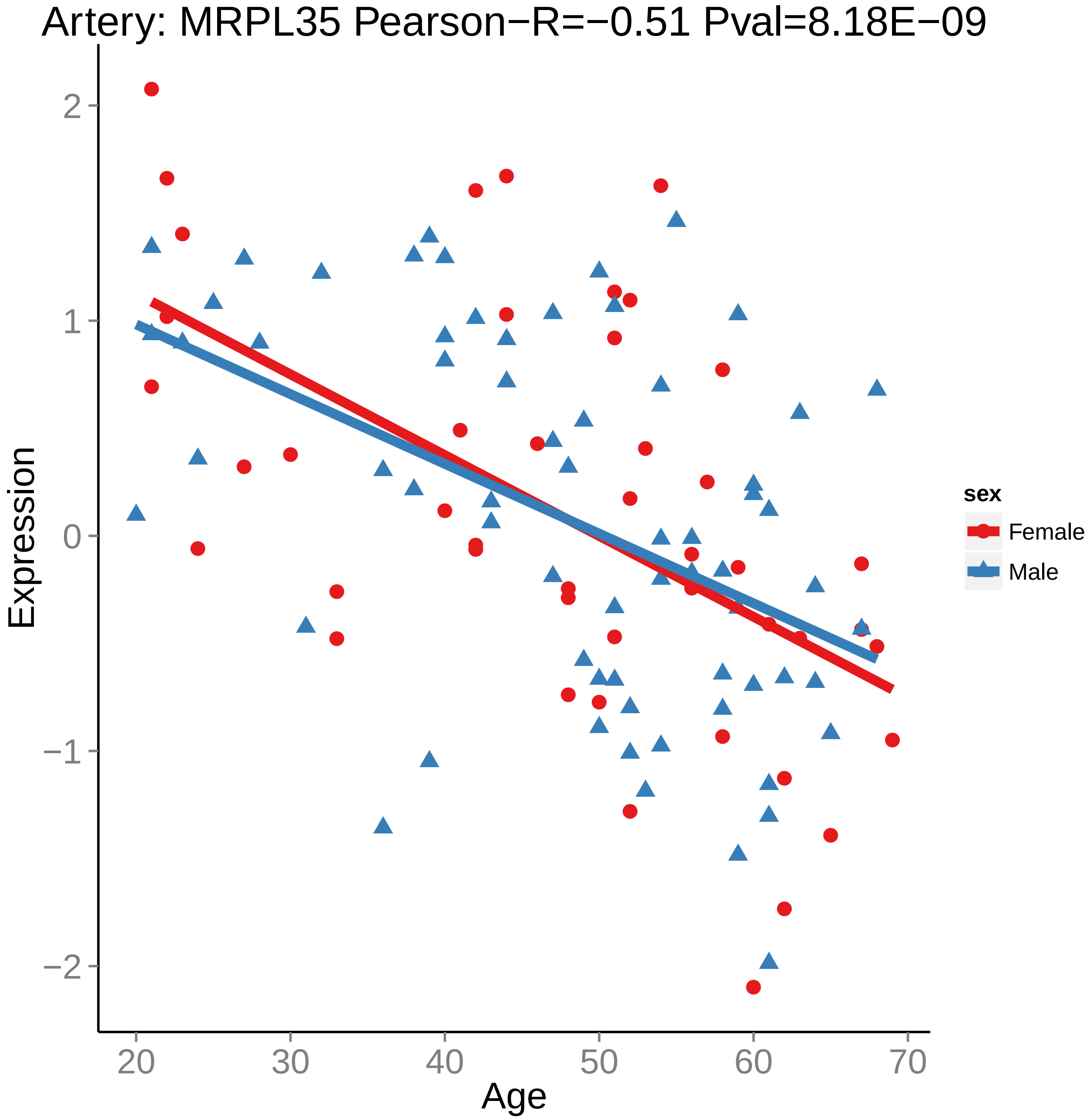

Artery: MRPL35 Pearson−R=−0.51 Pval=8.18E−09
Expression
Age
2
1
0
−1
−2
20
30
40
50
60
70
sex
Female
Male

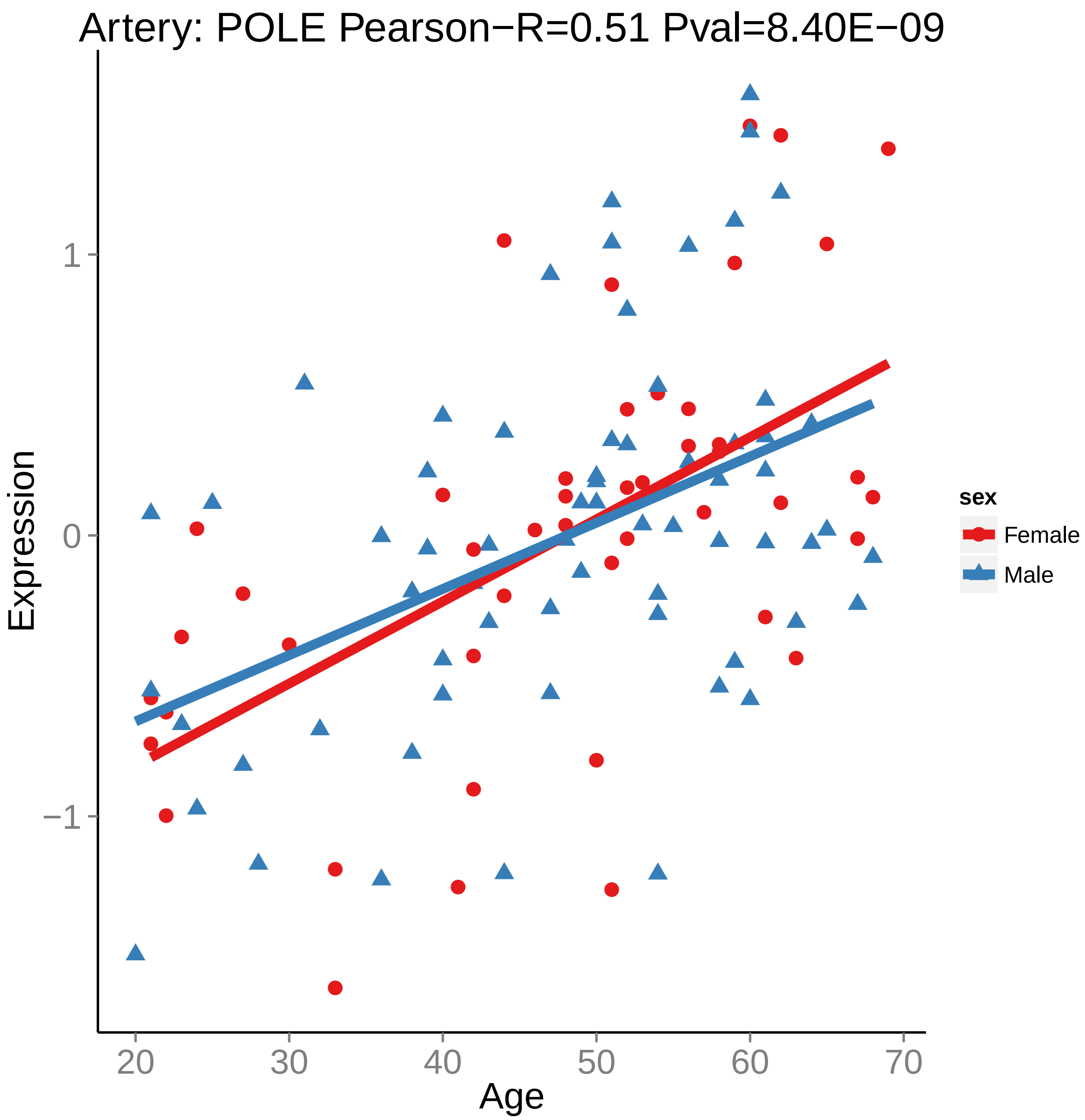

Artery: POLE Pearson−R=0.51 Pval=8.40E−09
Expression
1
0
−1
20
30
40
50
60
70
Age
sex
Female
Male

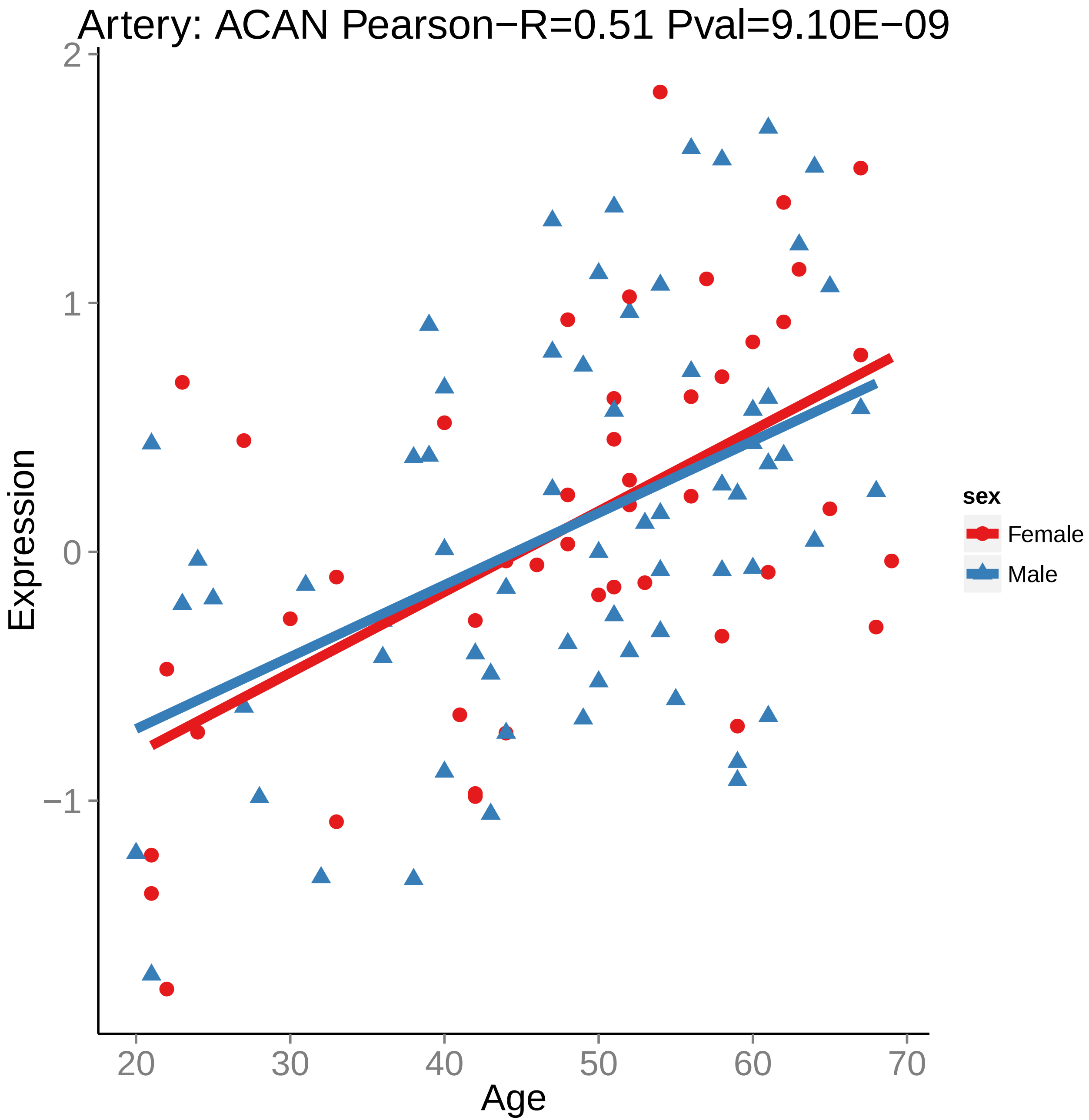
Artery: ACAN Pearson−R=0.51 Pval=9.10E−09
Expression
Age
2
1
0
−1
20
30
40
50
60
70
sex
Female
Male

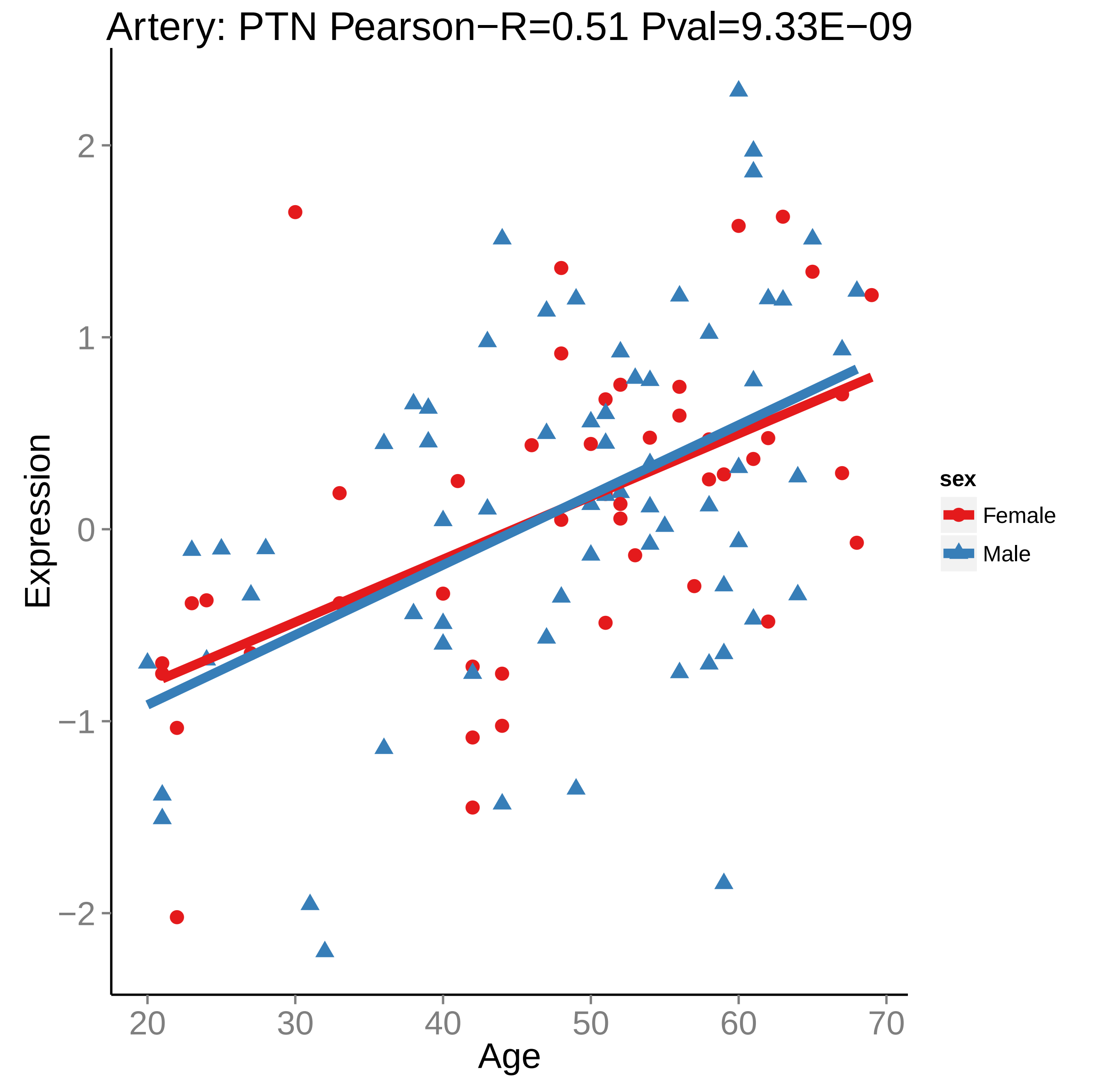

Artery: PTN Pearson−R=0.51 Pval=9.33E−09
Expression
Age
2
1
0
−1
−2
20
30
40
50
60
70
sex
Female
Male

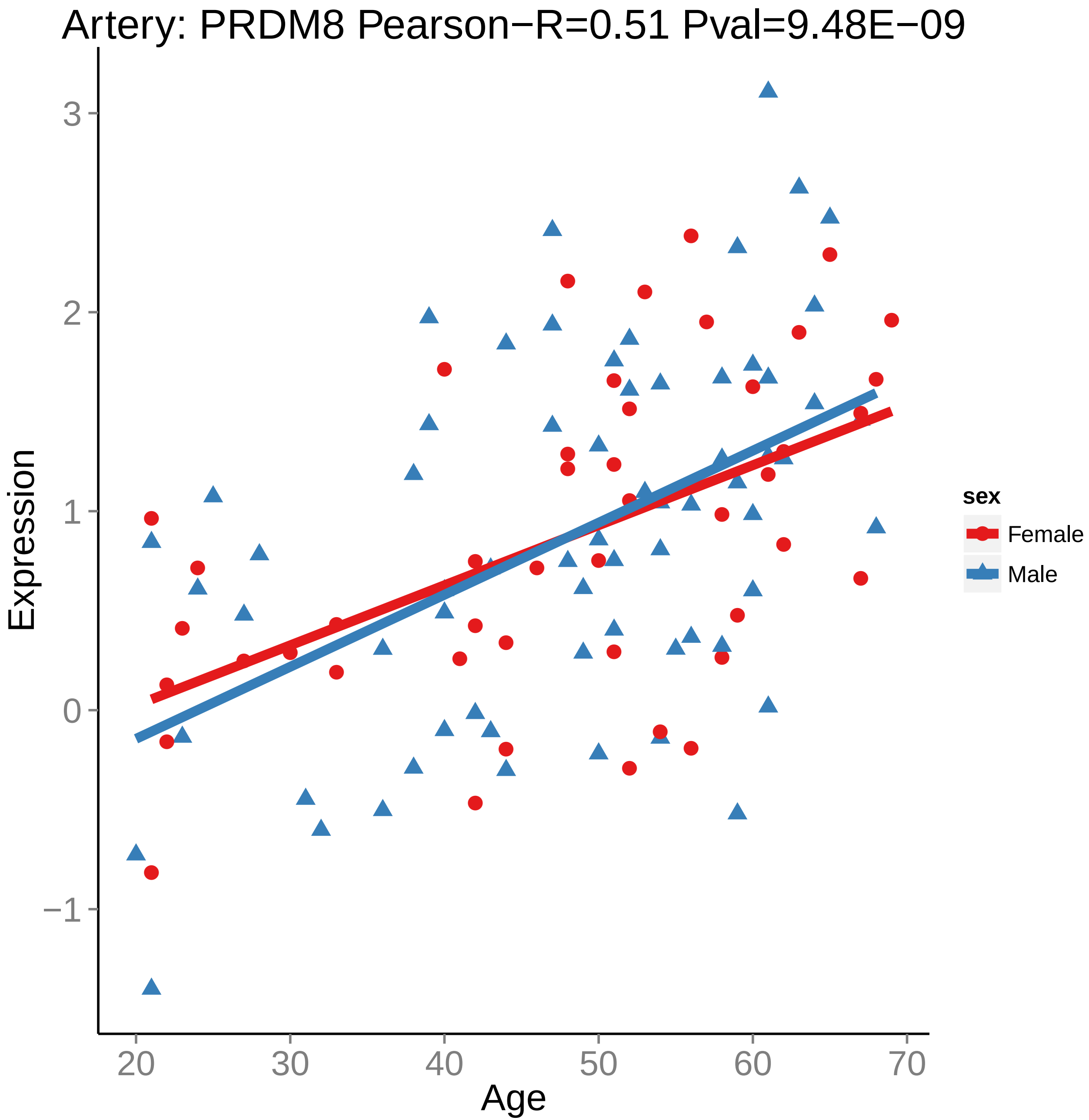

Artery: PRDM8 Pearson−R=0.51 Pval=9.48E−09
Expression
3
2
1
0
−1
20
30
40
50
60
70
Age
sex
Female
Male

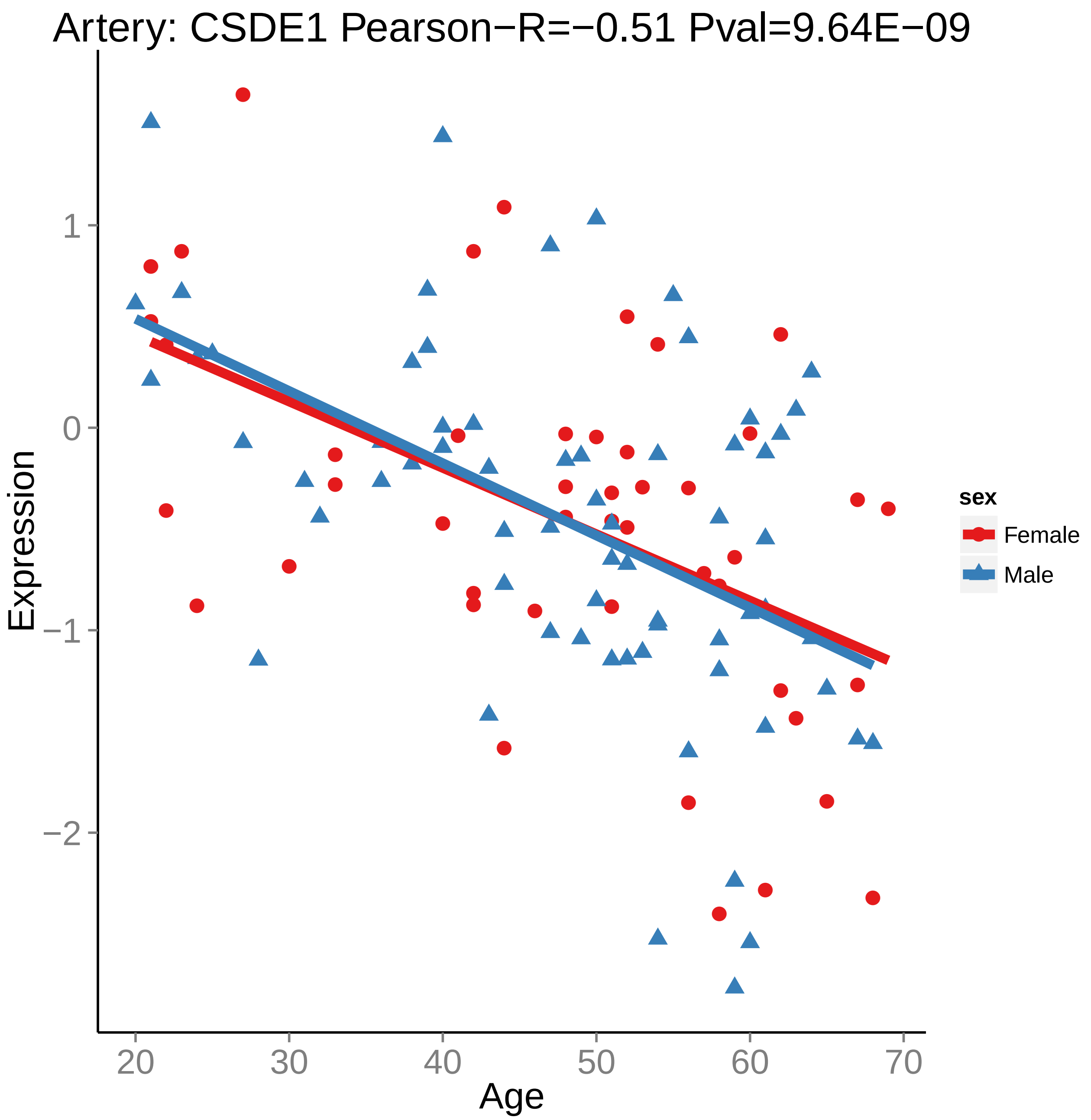

Artery: CSDE1 Pearson−R=−0.51 Pval=9.64E−09
Expression
1
0
−1
−2
20
30
40
50
60
70
Age
sex
Female
Male

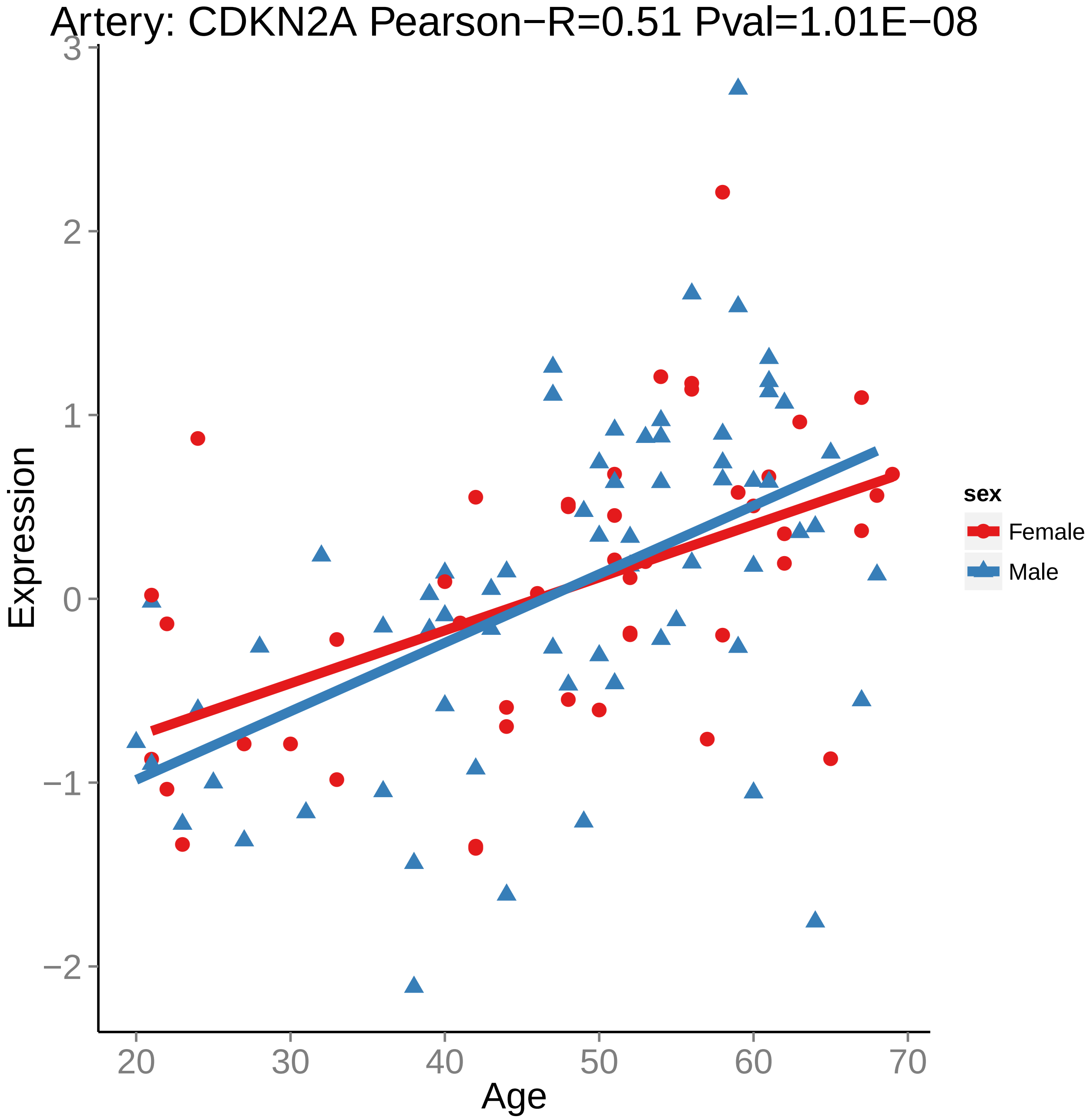

Artery: CDKN2A Pearson−R=0.51 Pval=1.01E−08
Expression
Age
sex
Female
Male
3
2
1
0
−1
−2
20
30
40
50
60
70

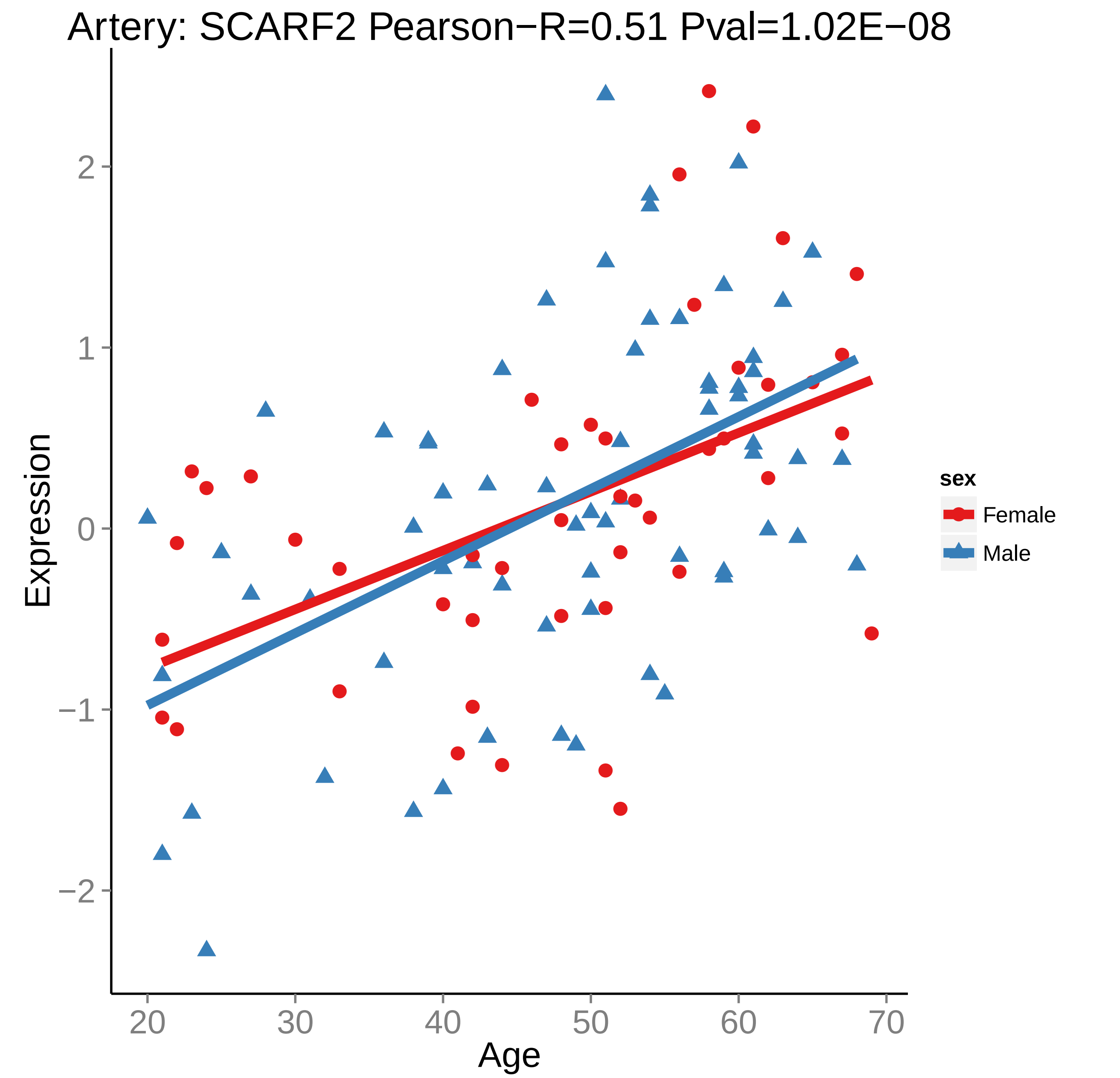

Artery: SCARF2 Pearson−R=0.51 Pval=1.02E−08
Expression
2
1
0
−1
−2
20
30
40
50
60
70
Age
sex
Female
Male

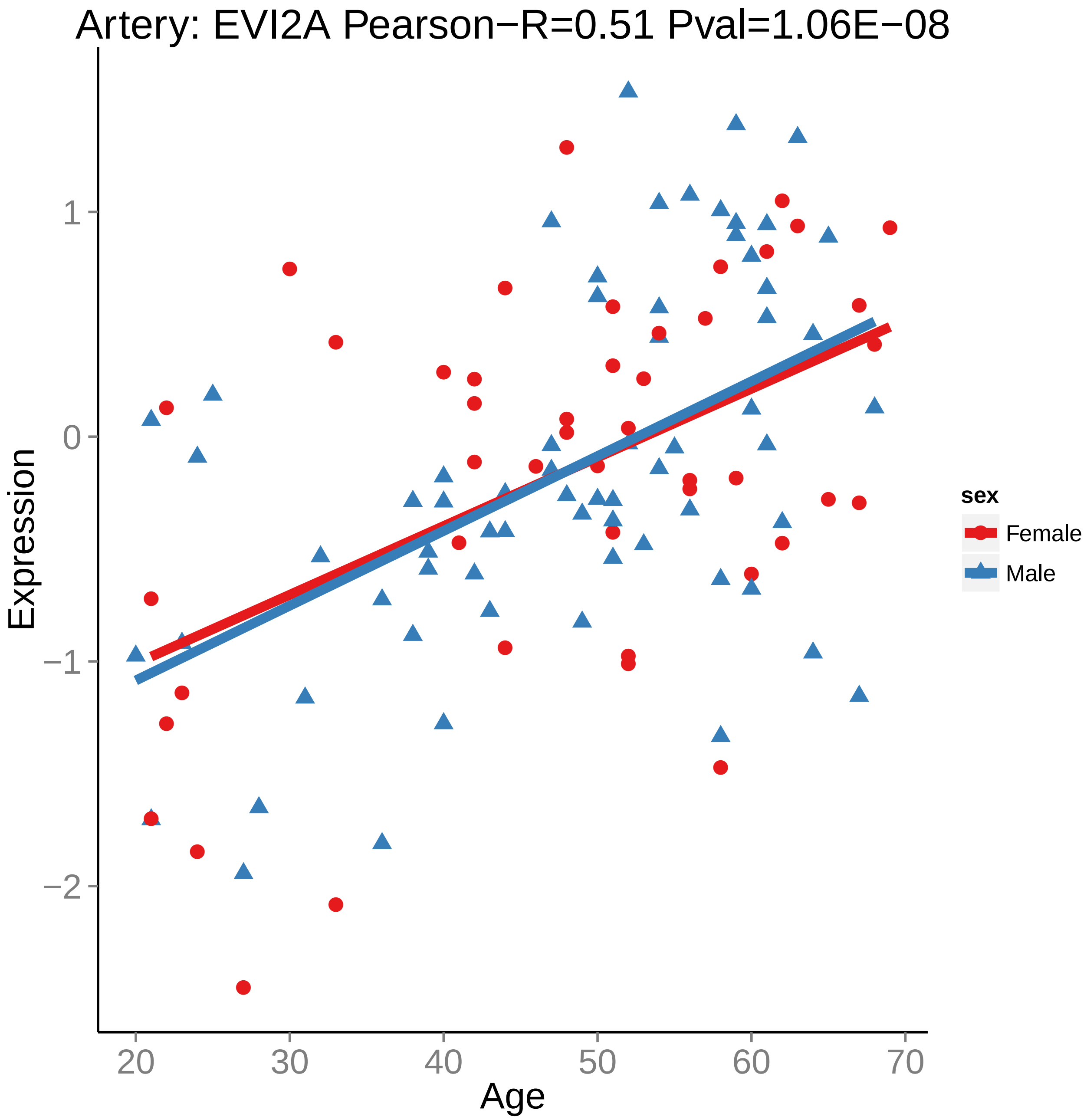

Artery: EVI2A Pearson−R=0.51 Pval=1.06E−08
Expression
1
0
−1
−2
20
30
40
50
60
70
Age
sex
Female
Male

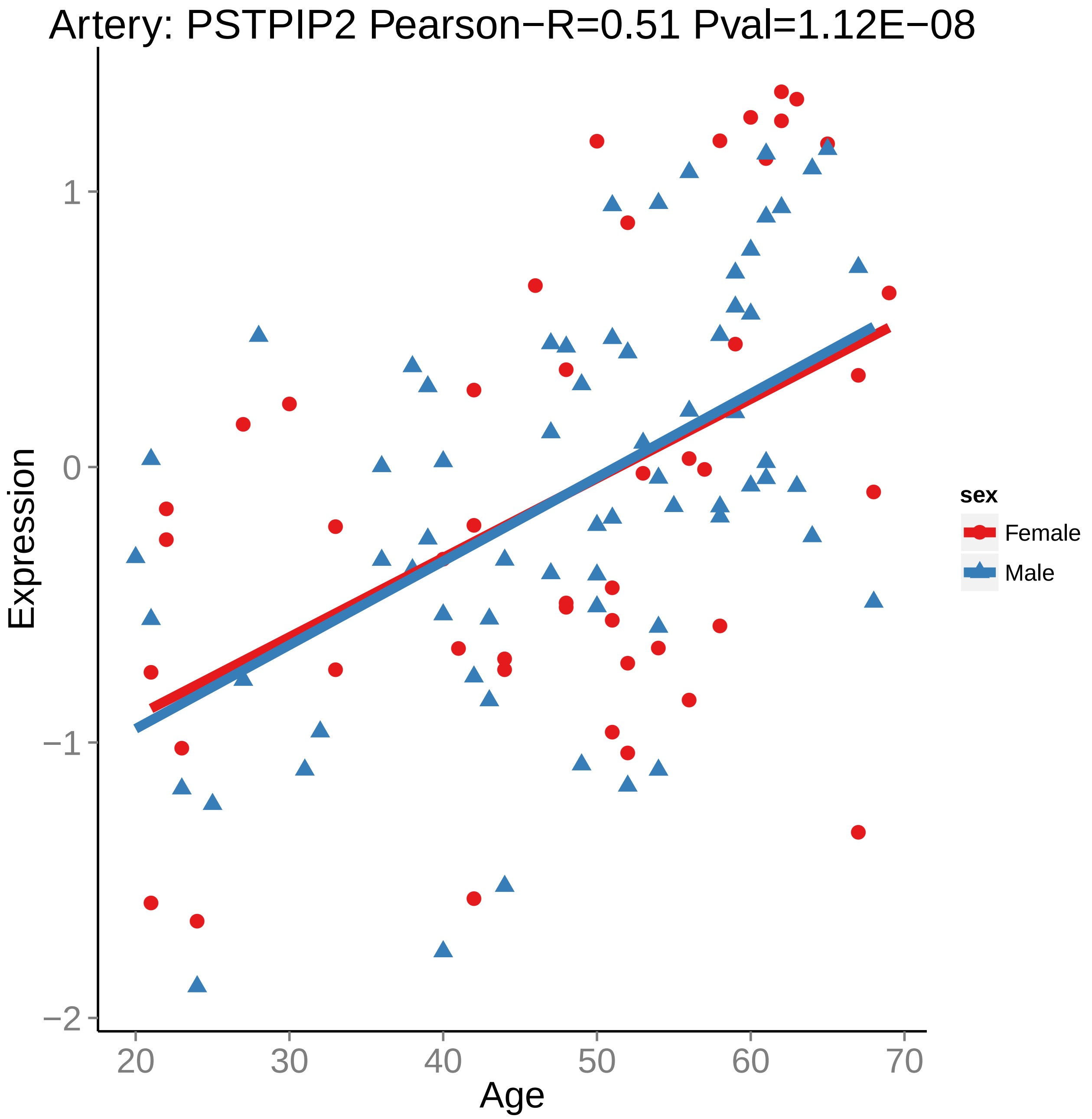

Artery: PSTPIP2 Pearson−R=0.51 Pval=1.12E−08
Expression
Age
1
0
−1
−2
20
30
40
50
60
70
sex
Female
Male

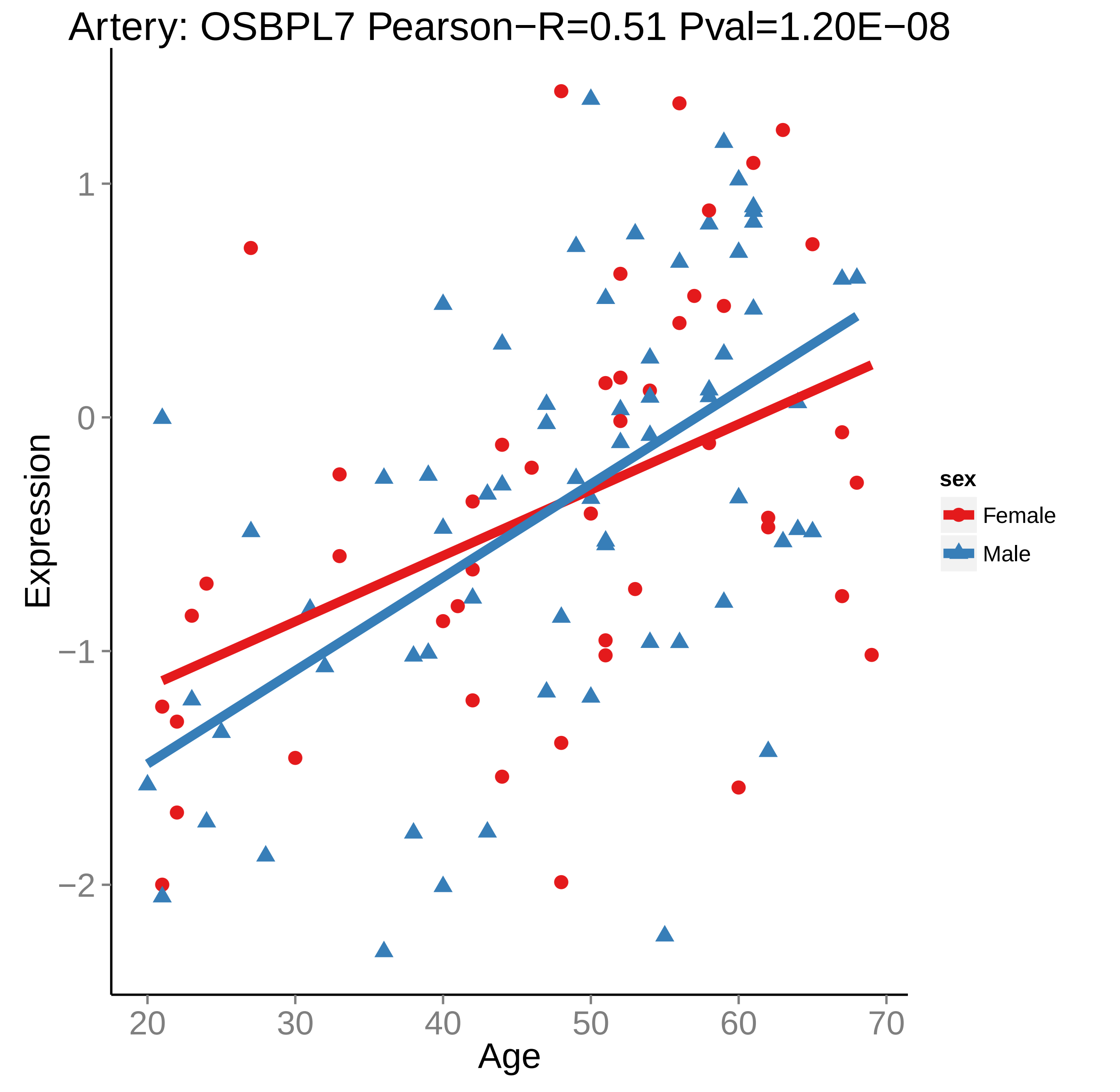

Artery: OSBPL7 Pearson−R=0.51 Pval=1.20E−08
Expression
1
0
−1
−2
Age
20
30
40
50
60
70
sex
Female
Male

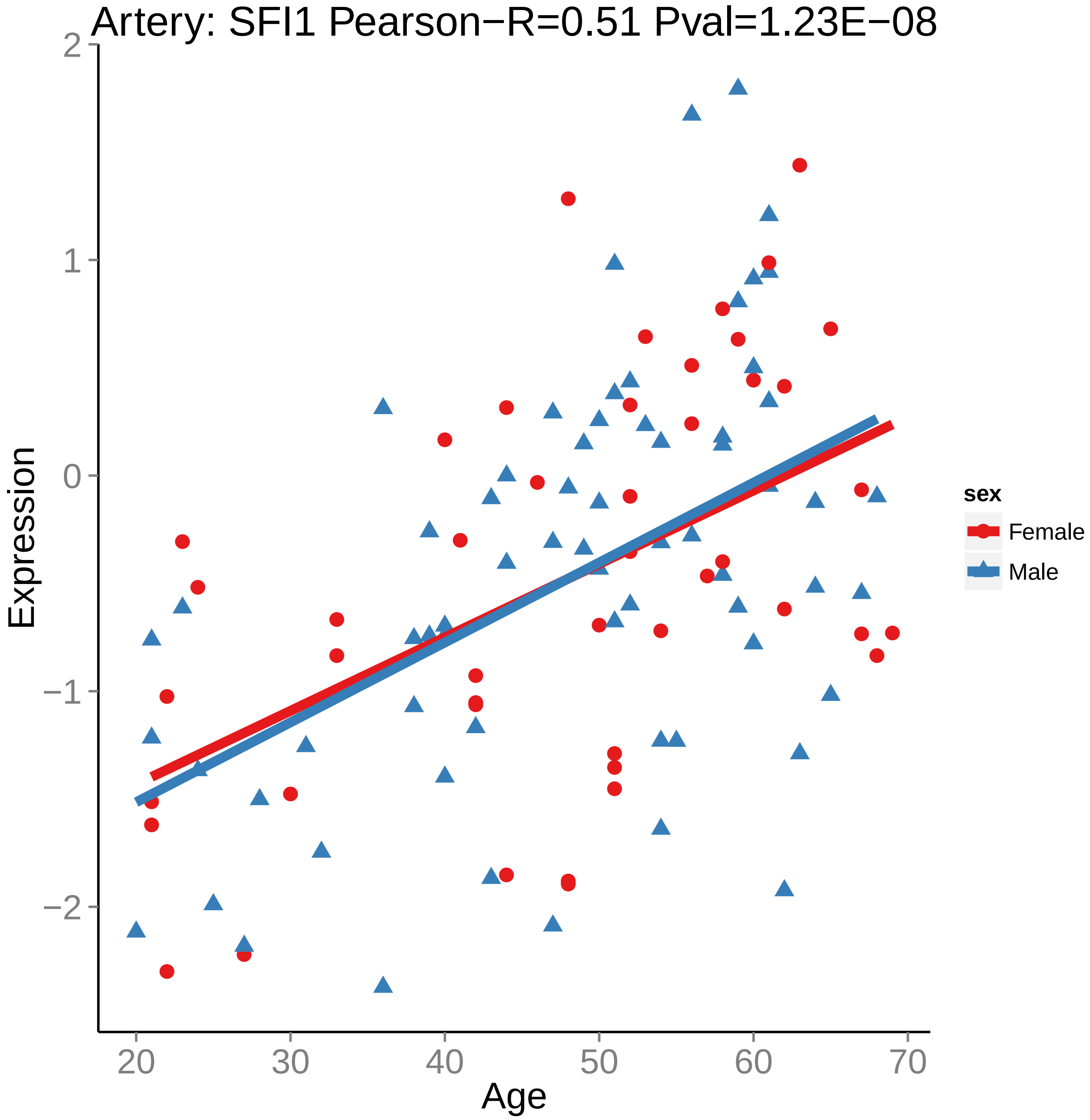

Artery: SFI1 Pearson−R=0.51 Pval=1.23E−08
Expression
Age
2
1
0
−1
−2
20
30
40
50
60
70
sex
Female
Male

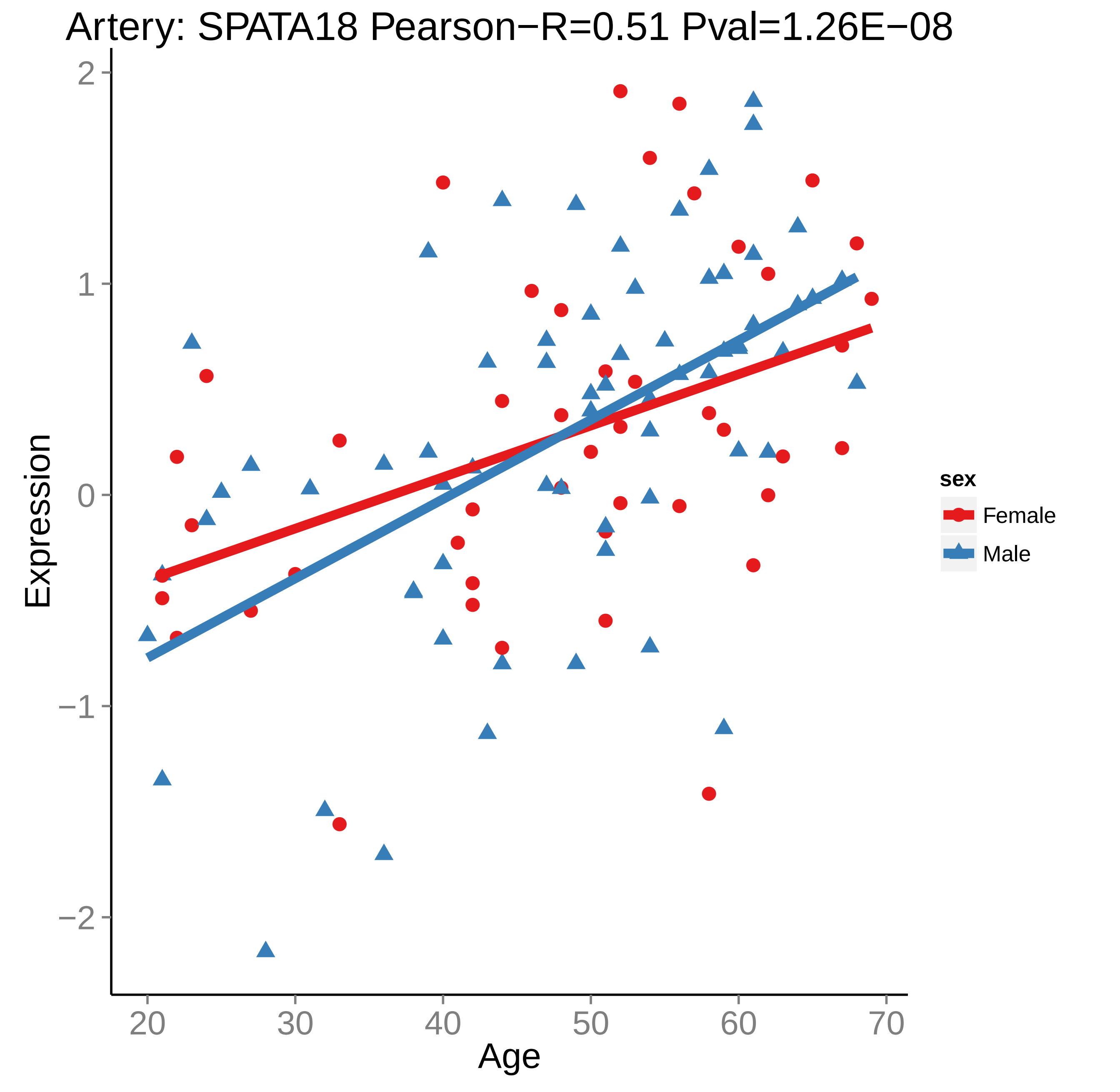

Artery: SPATA18 Pearson−R=0.51 Pval=1.26E−08
Expression
Age
2
1
0
−1
−2
20
30
40
50
60
70
sex
Female
Male

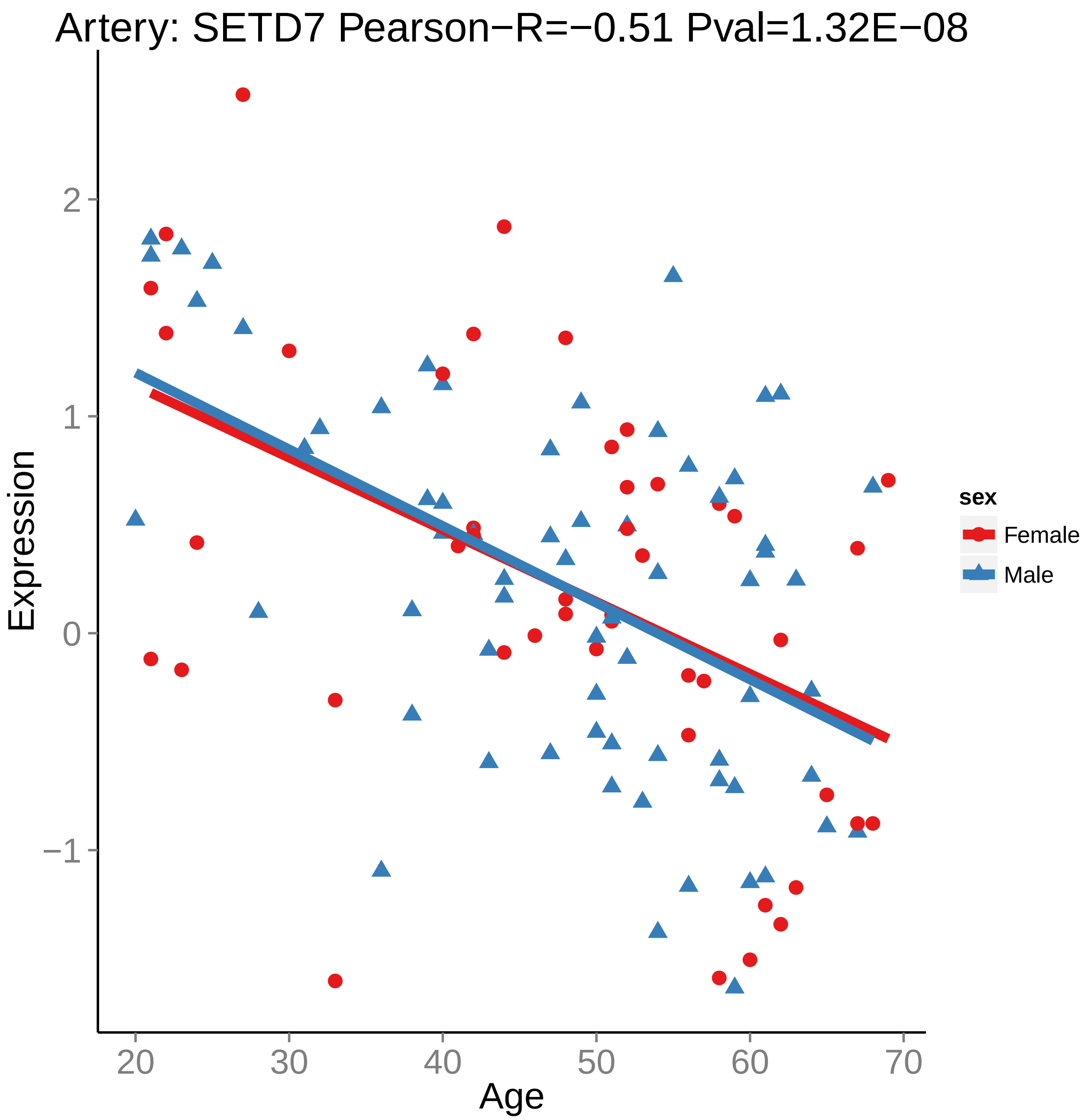

Artery: SETD7 Pearson−R=−0.51 Pval=1.32E−08
Expression
2
1
0
−1
20
30
40
50
60
70
Age
sex
Female
Male

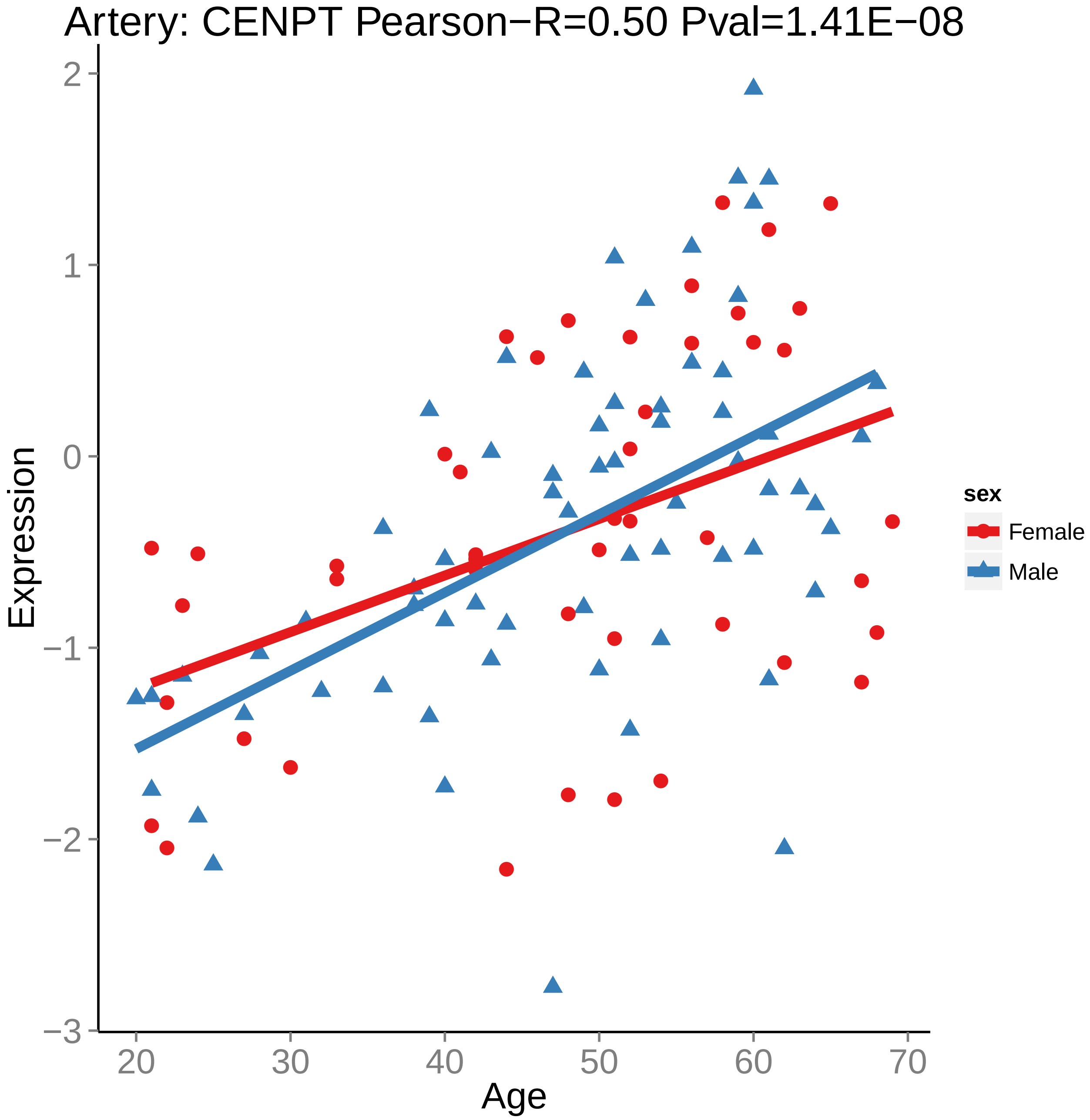

Artery: CENPT Pearson−R=0.50 Pval=1.41E−08
Expression
Age
2
1
0
−1
−2
−3
20
30
40
50
60
70
sex
Female
Male

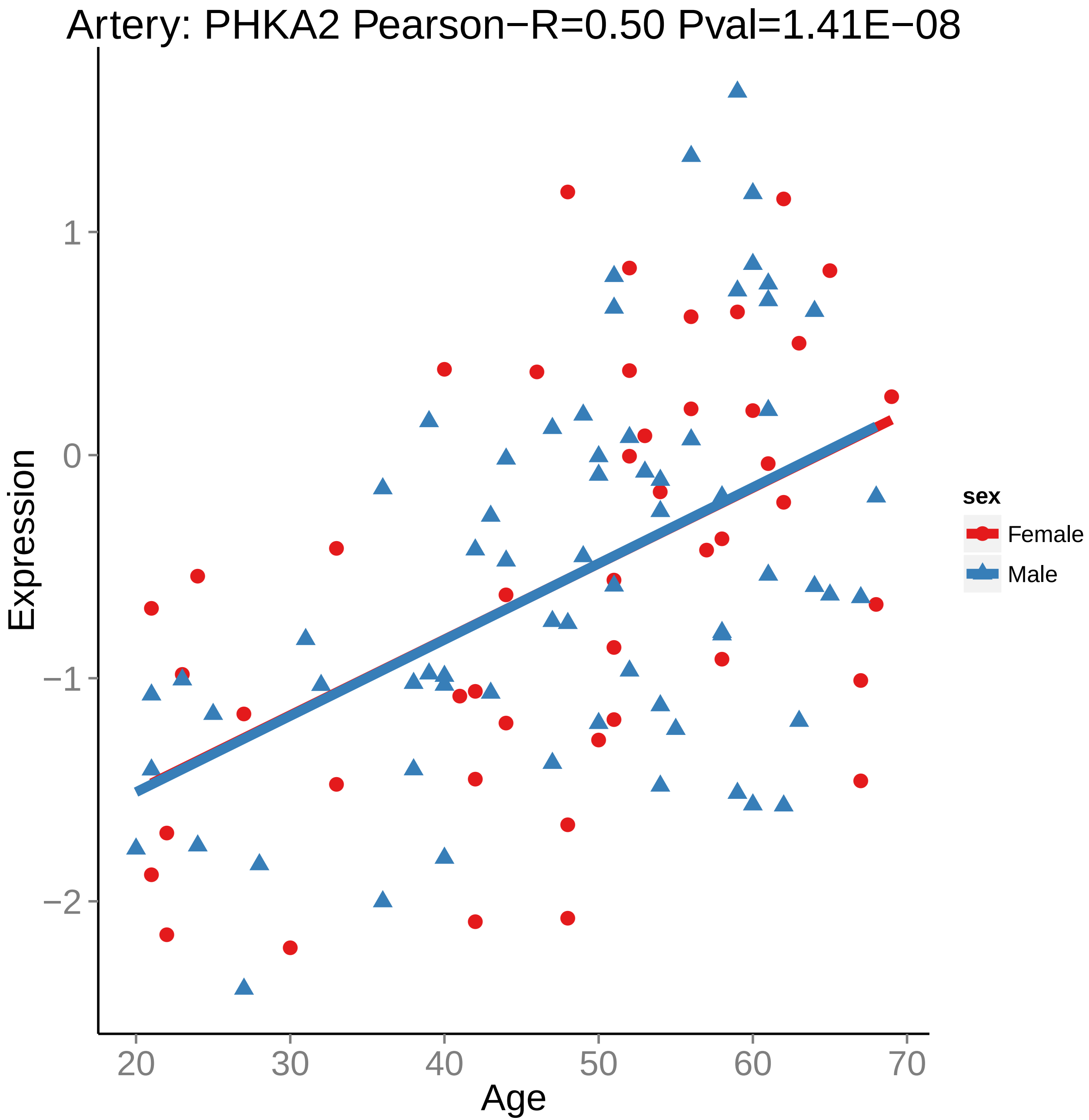
Artery: PHKA2 Pearson−R=0.50 Pval=1.41E−08
Expression
Age
1
0
−1
−2
20
30
40
50
60
70
sex
Female
Male

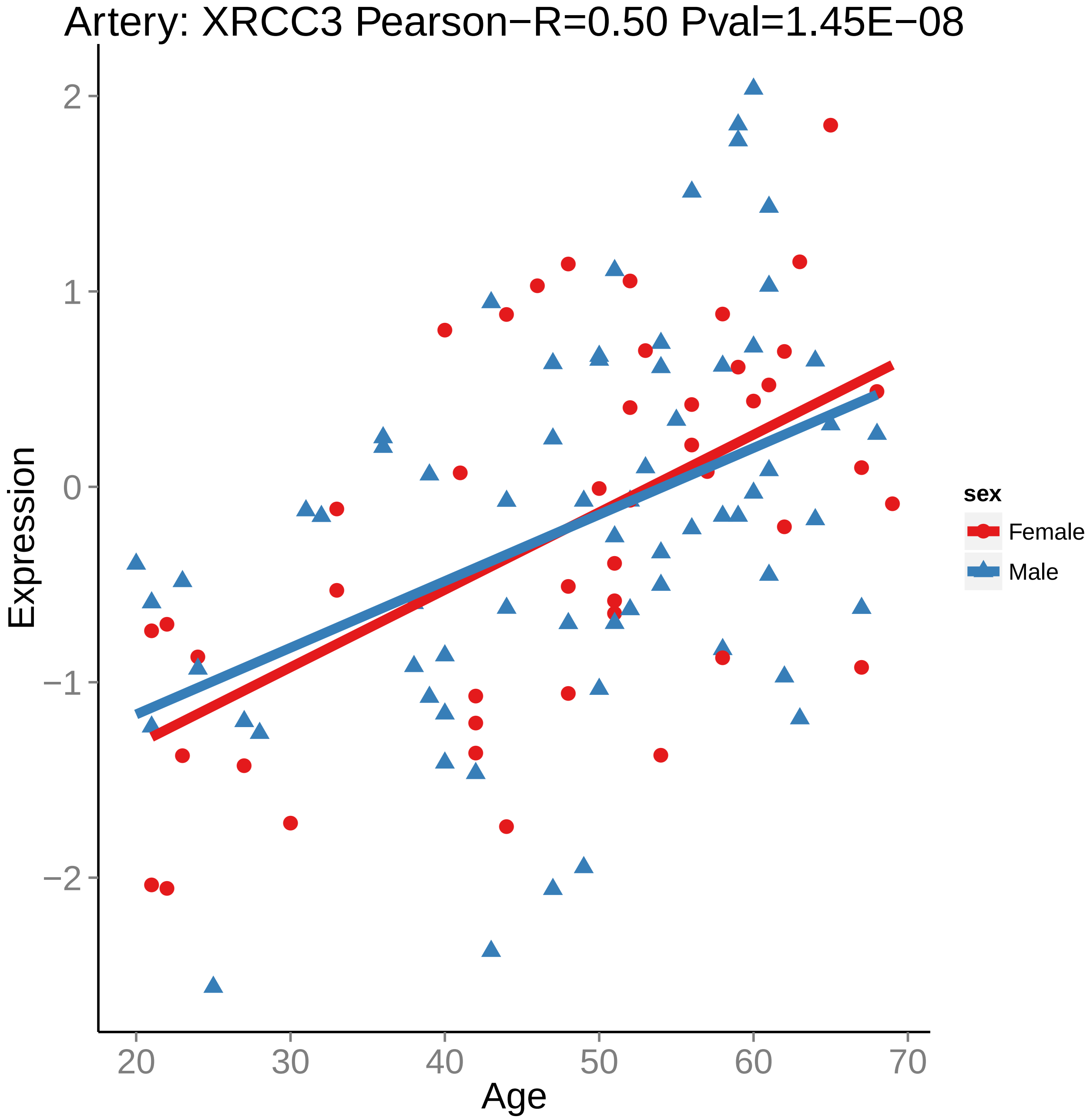

Artery: XRCC3 Pearson−R=0.50 Pval=1.45E−08
Expression
Age
2
1
0
−1
−2
20
30
40
50
60
70
sex
Female
Male

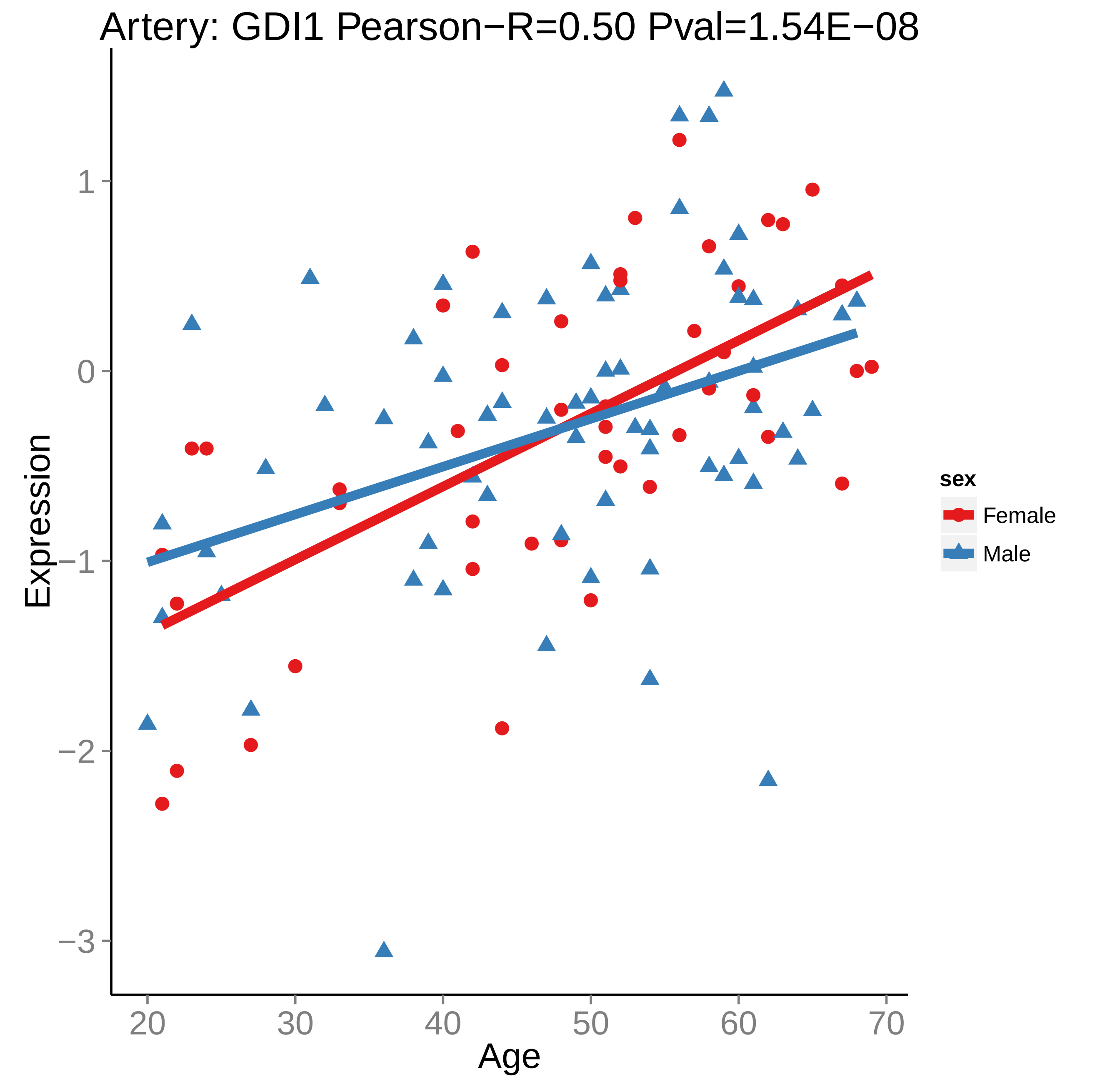

Artery: GDI1 Pearson−R=0.50 Pval=1.54E−08
Expression
Age
1
0
−1
−2
−3
20
30
40
50
60
70
sex
Female
Male

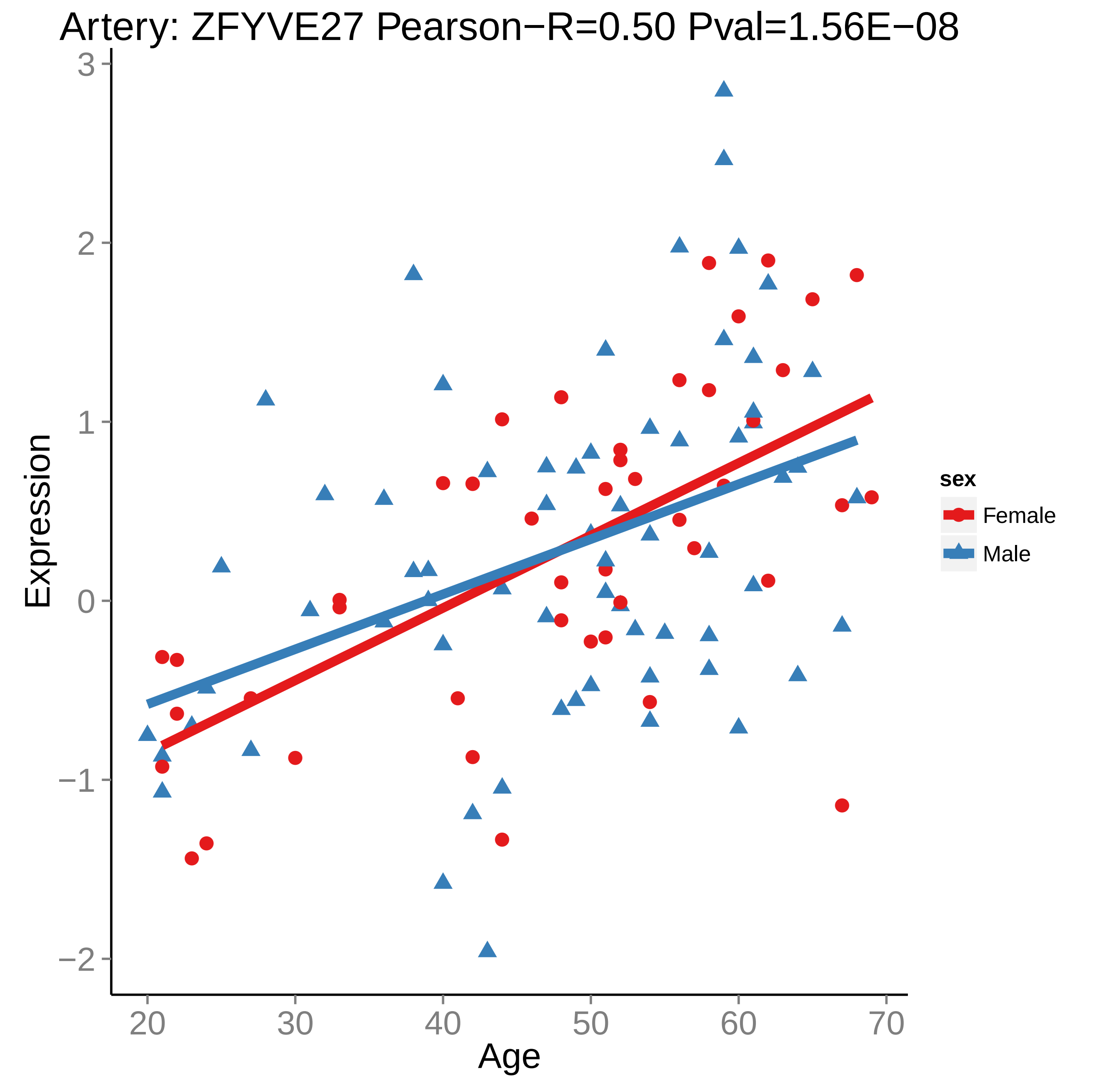

Artery: ZFYVE27 Pearson−R=0.50 Pval=1.56E−08
Expression
Age
3
2
1
0
−1
−2
20
30
40
50
60
70
sex
Female
Male

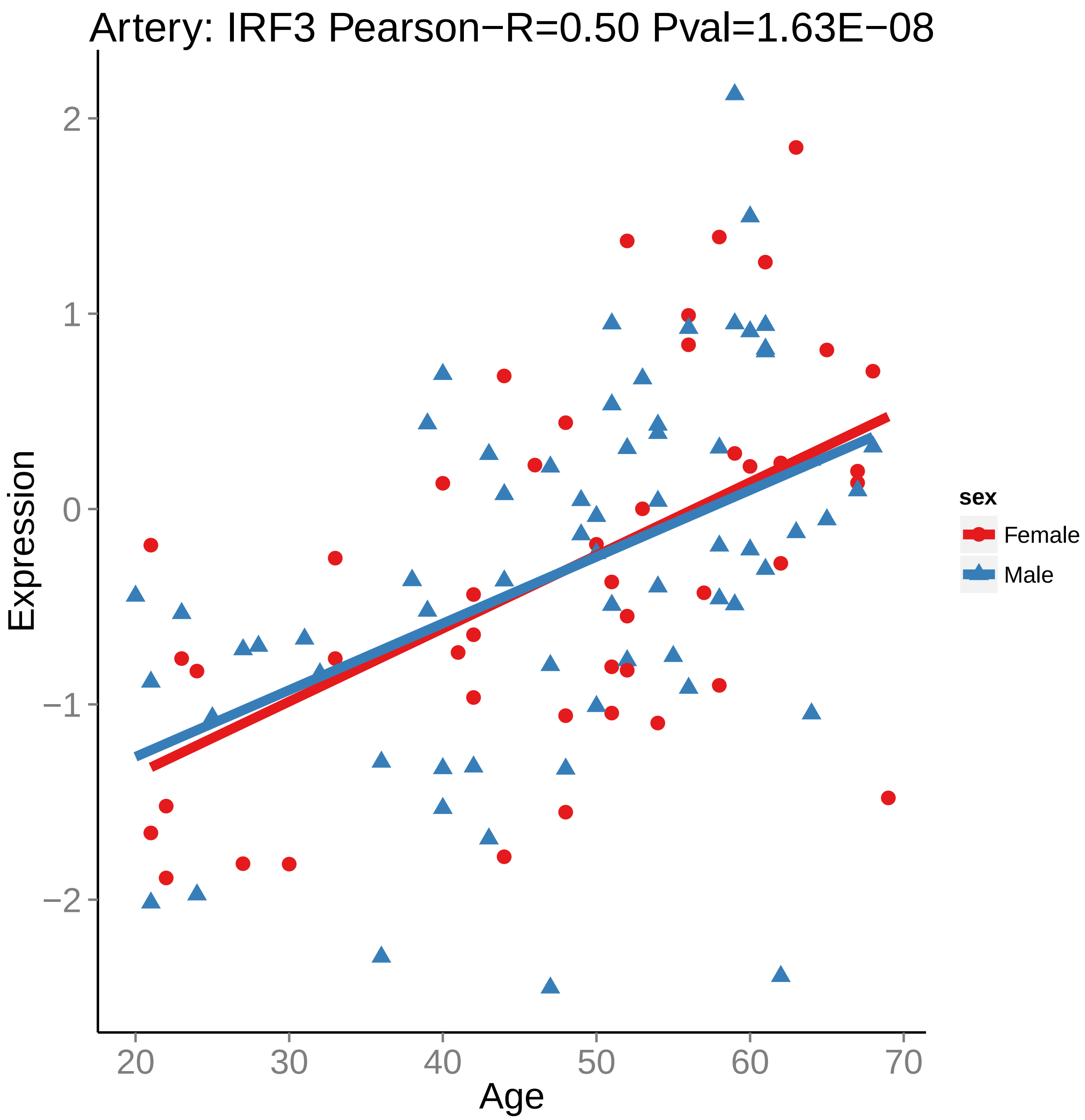

Artery: IRF3 Pearson−R=0.50 Pval=1.63E−08
Expression
2
1
0
−1
−2
20
30
40
50
60
70
Age
sex
Female
Male

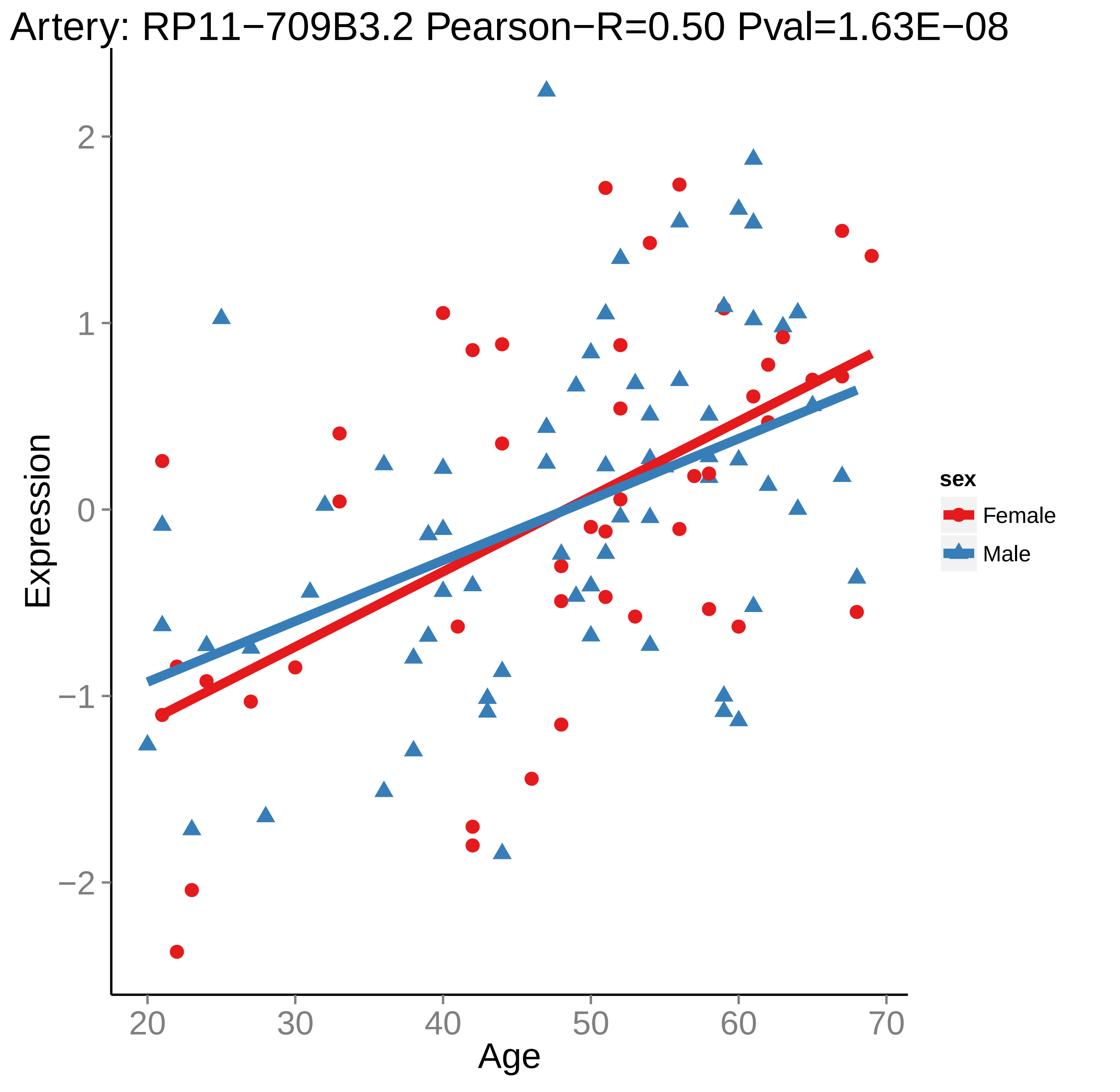

Artery: RP11−709B3.2 Pearson−R=0.50 Pval=1.63E−08
Expression
2
1
0
−1
−2
20
30
40
50
60
70
Age
sex
Female
Male

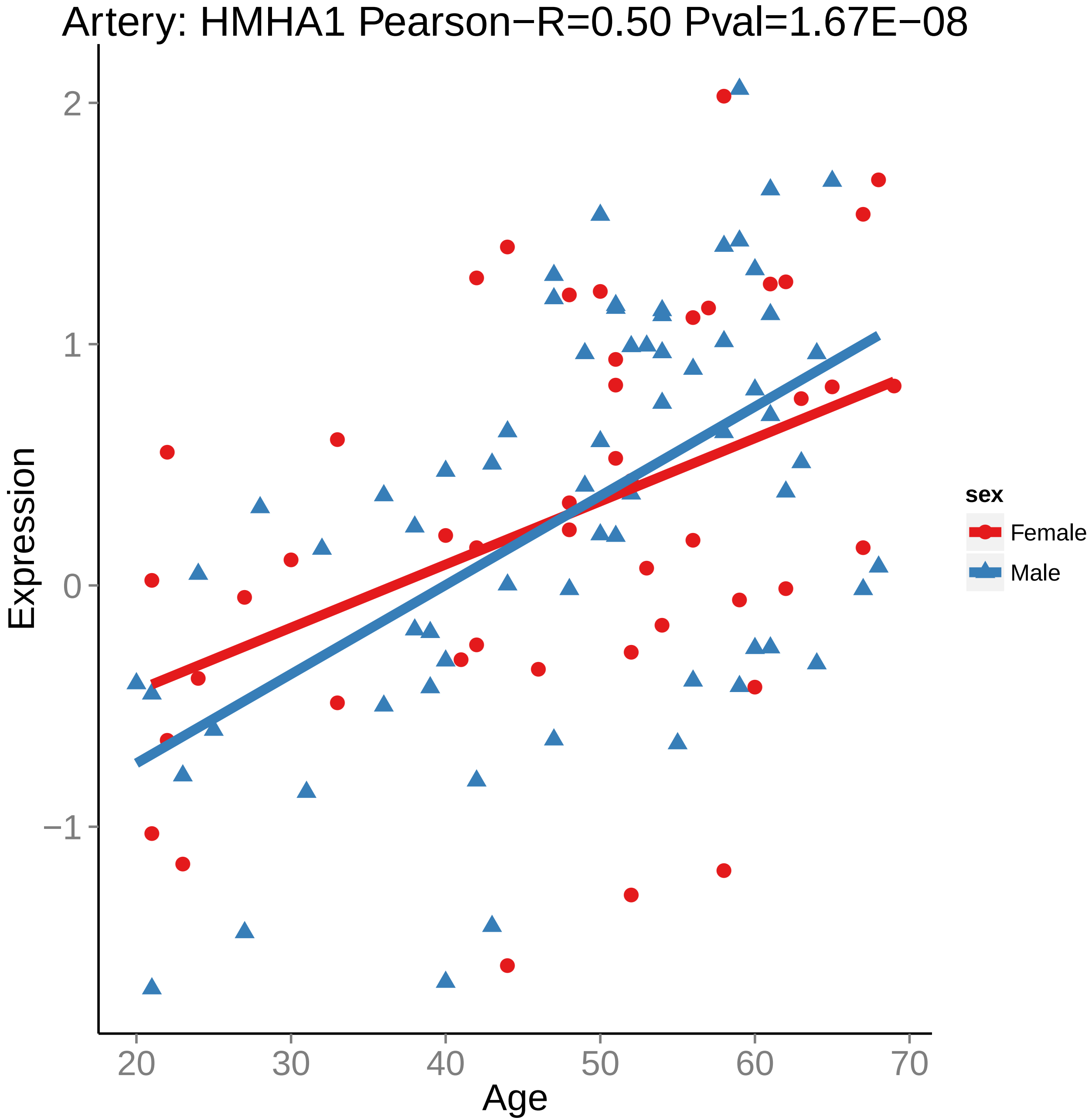

Artery: HMHA1 Pearson−R=0.50 Pval=1.67E−08
Expression
2
1
0
−1
20
30
40
50
60
70
Age
sex
Female
Male

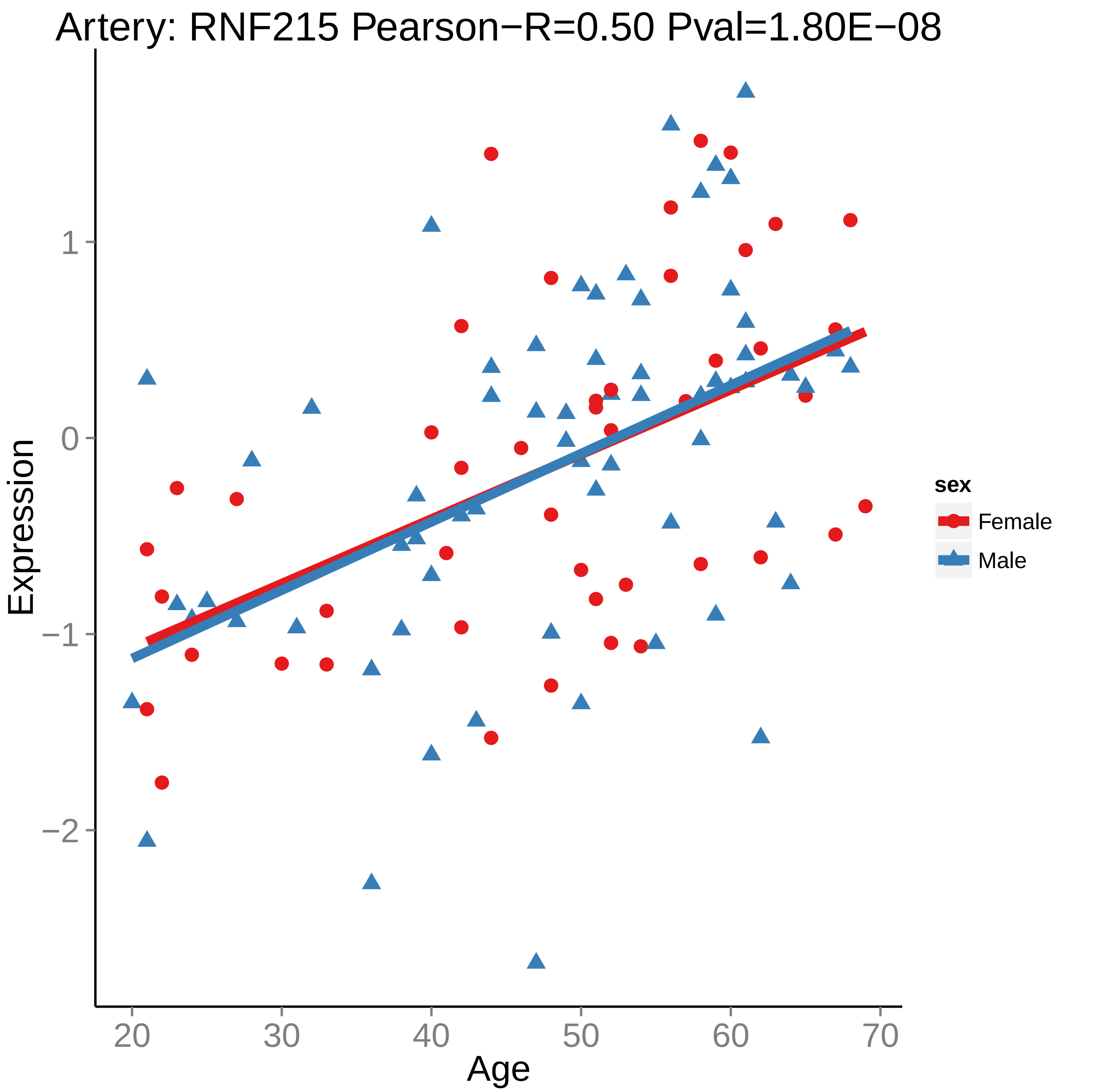
Artery: RNF215 Pearson−R=0.50 Pval=1.80E−08
Expression
Age
1
0
−1
−2
20
30
40
50
60
70
sex
Female
Male

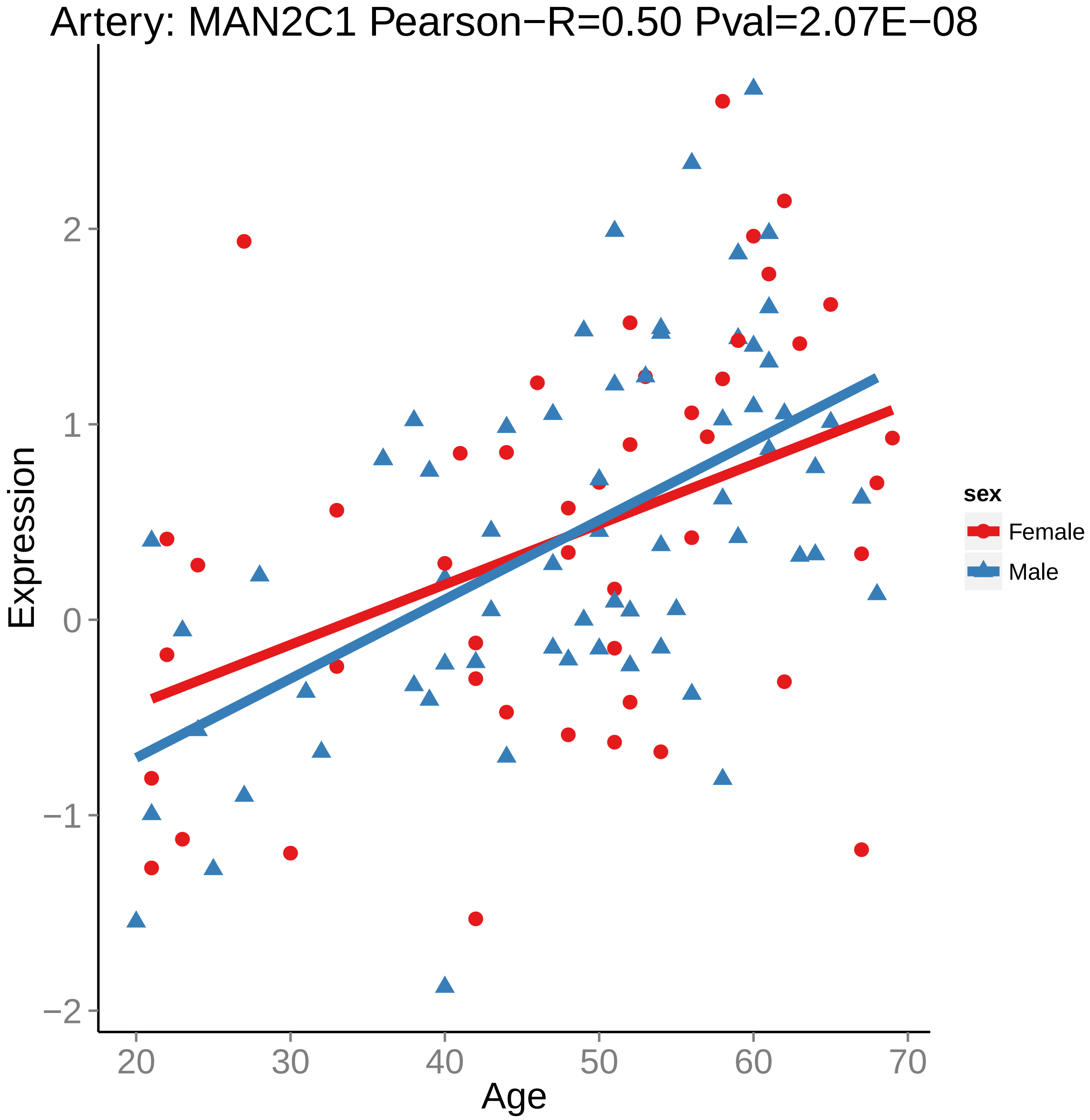

Artery: MAN2C1 Pearson−R=0.50 Pval=2.07E−08
Expression
Age
2
1
0
−1
−2
20
30
40
50
60
70
sex
Female
Male

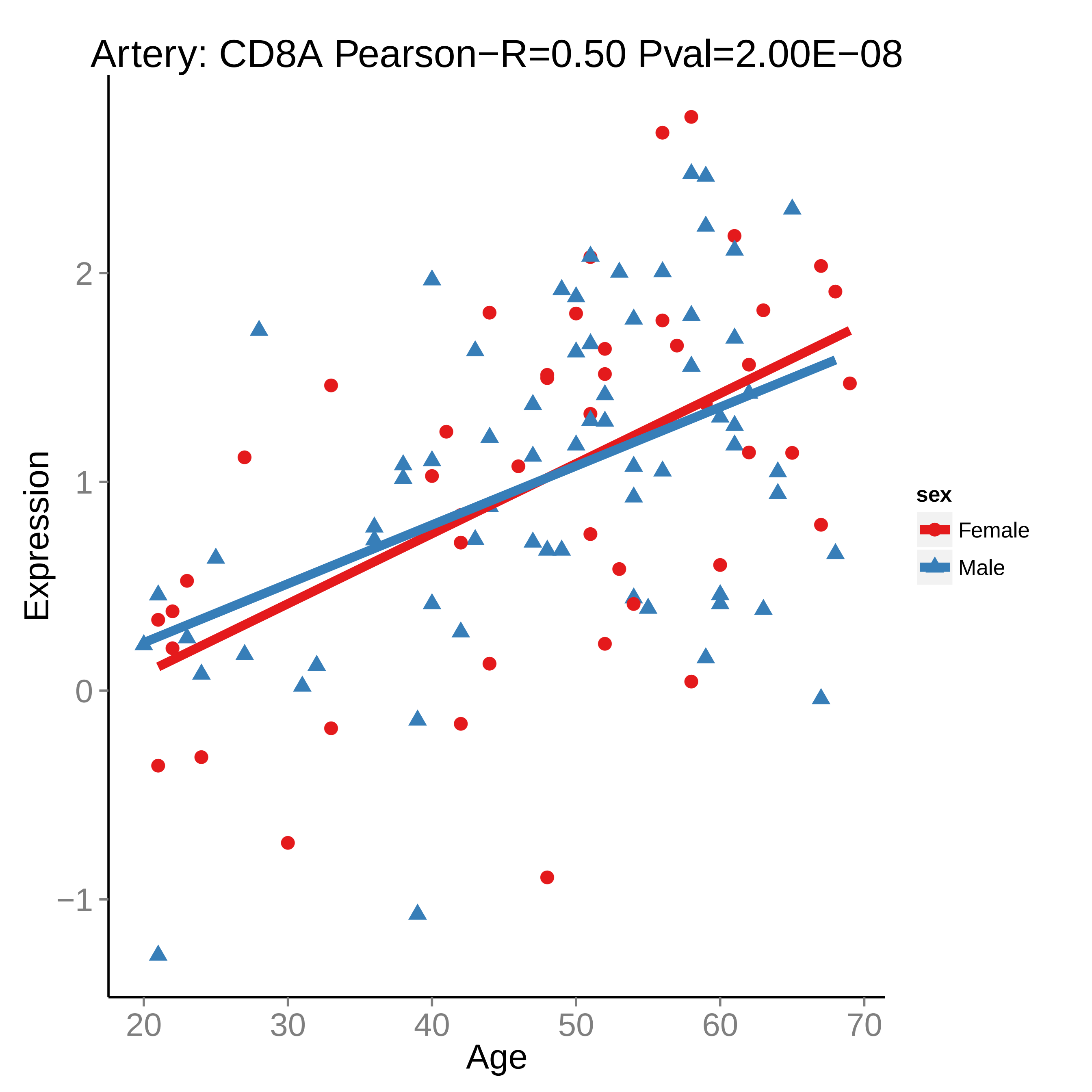

Artery: CD8A Pearson−R=0.50 Pval=2.00E−08
Expression
Age
2
1
0
−1
20
30
40
50
60
70
sex
Female
Male

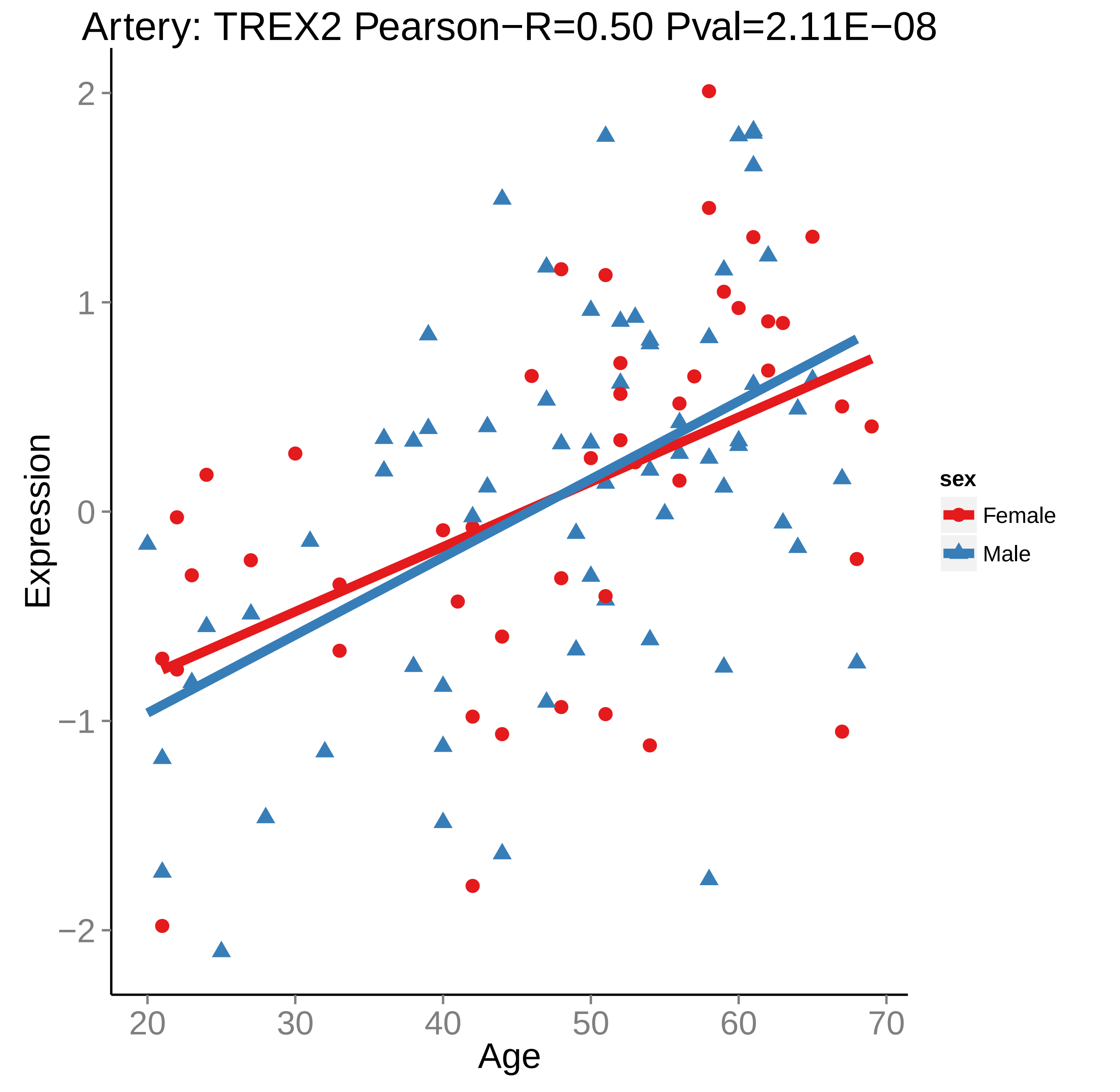

Artery: TREX2 Pearson−R=0.50 Pval=2.11E−08
Expression
Age
2
1
0
−1
−2
20
30
40
50
60
70
sex
Female
Male

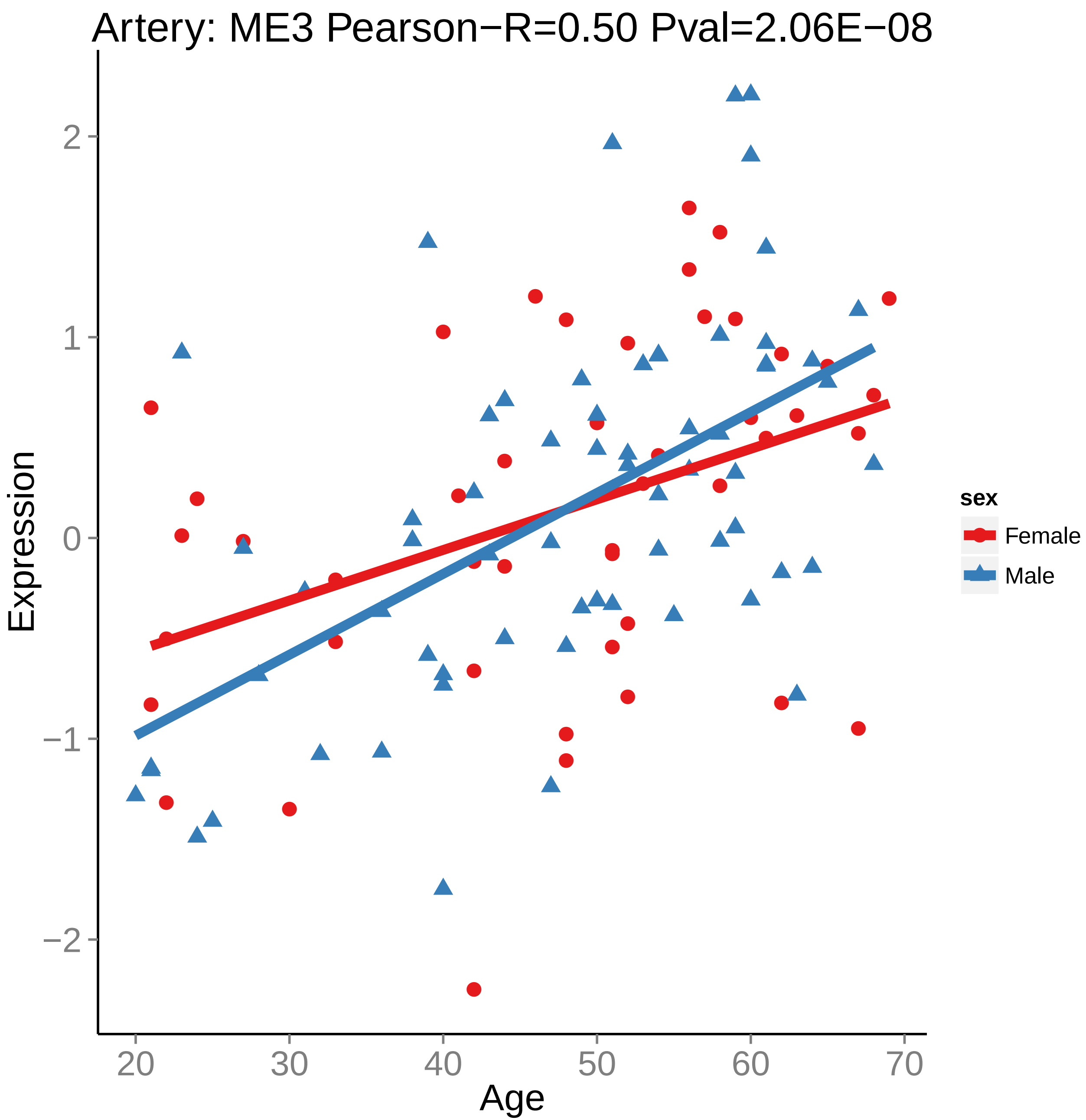

Artery: ME3 Pearson−R=0.50 Pval=2.06E−08
Expression
Age
2
1
0
−1
−2
20
30
40
50
60
70
sex
Female
Male

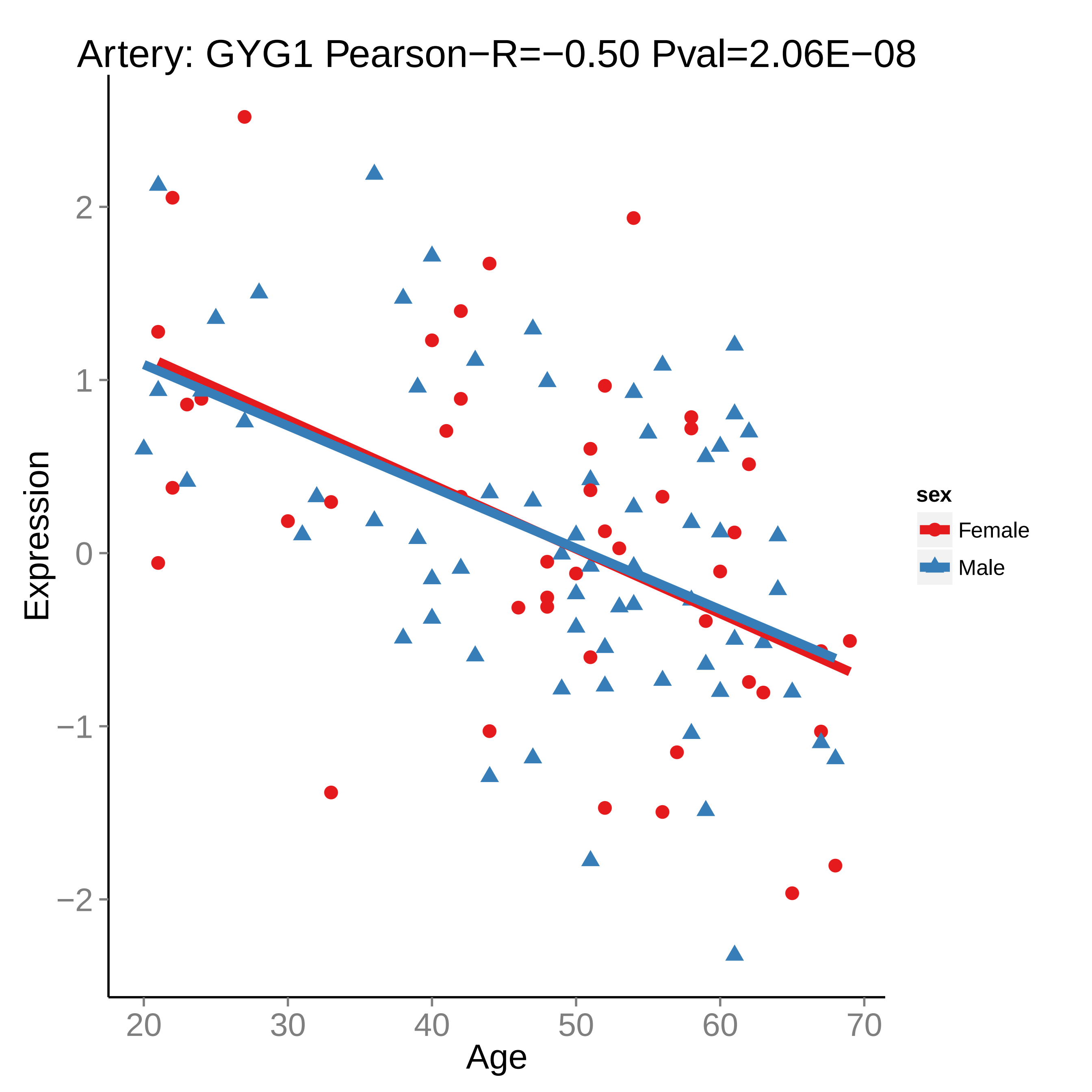

Artery: GYG1 Pearson−R=−0.50 Pval=2.06E−08
Expression
2
1
0
−1
−2
20
30
40
50
60
70
Age
sex
Female
Male

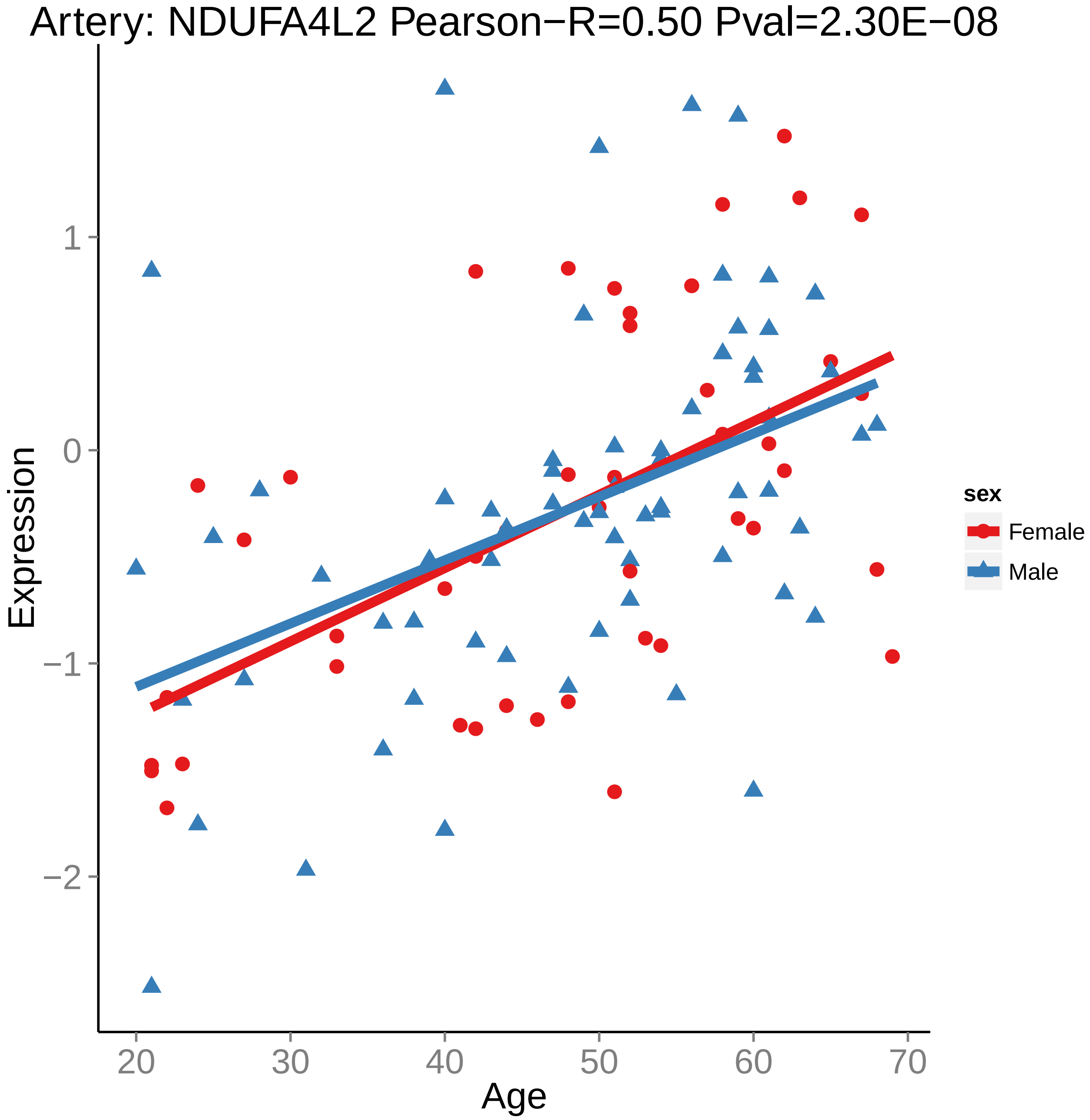
Artery: NDUFA4L2 Pearson−R=0.50 Pval=2.30E−08
Expression
1
0
−1
−2
20
30
40
50
60
70
Age
sex
Female
Male

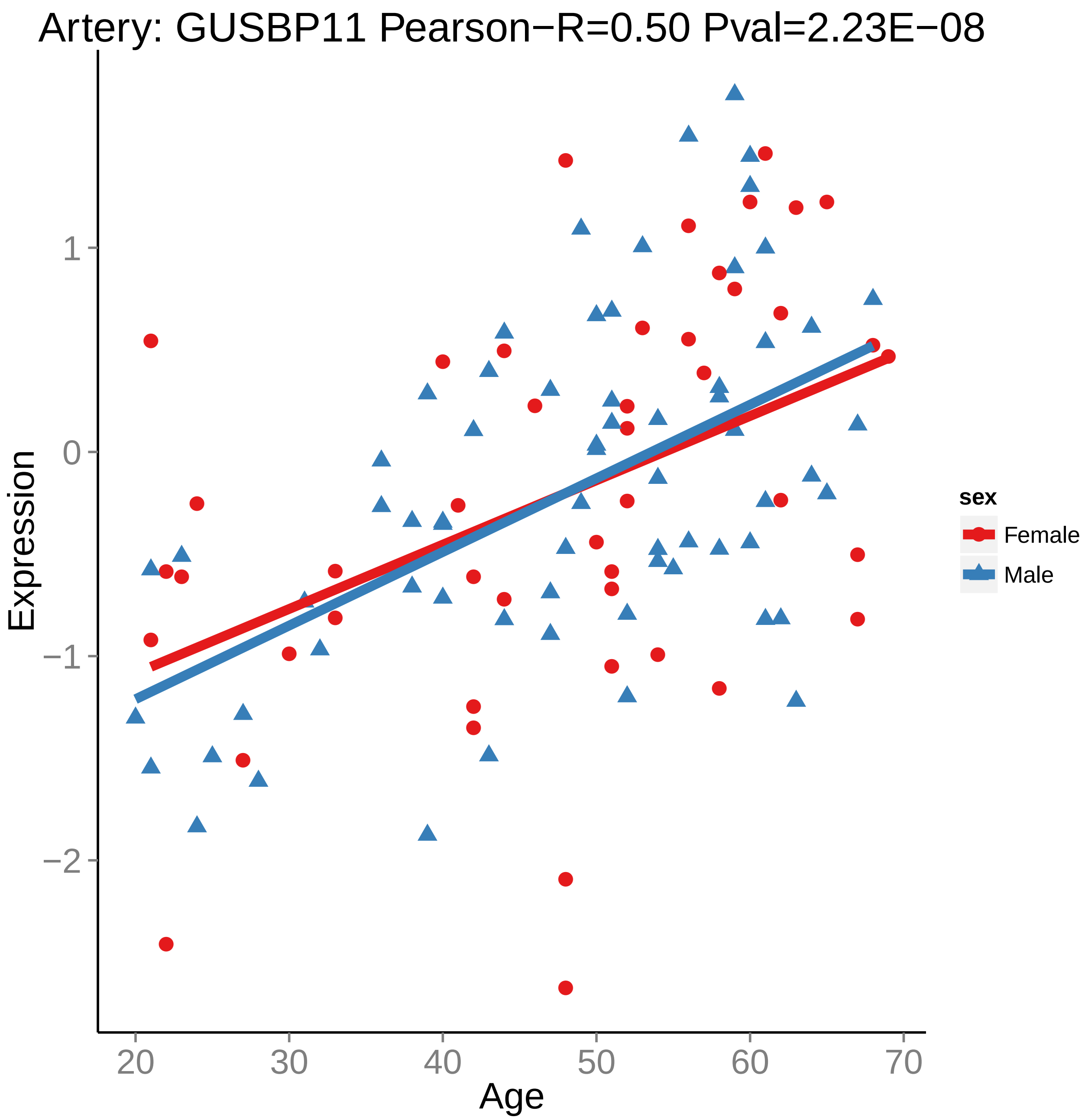

Artery: GUSBP11 Pearson−R=0.50 Pval=2.23E−08
Expression
1
0
−1
−2
20
30
40
50
60
70
Age
sex
Female
Male

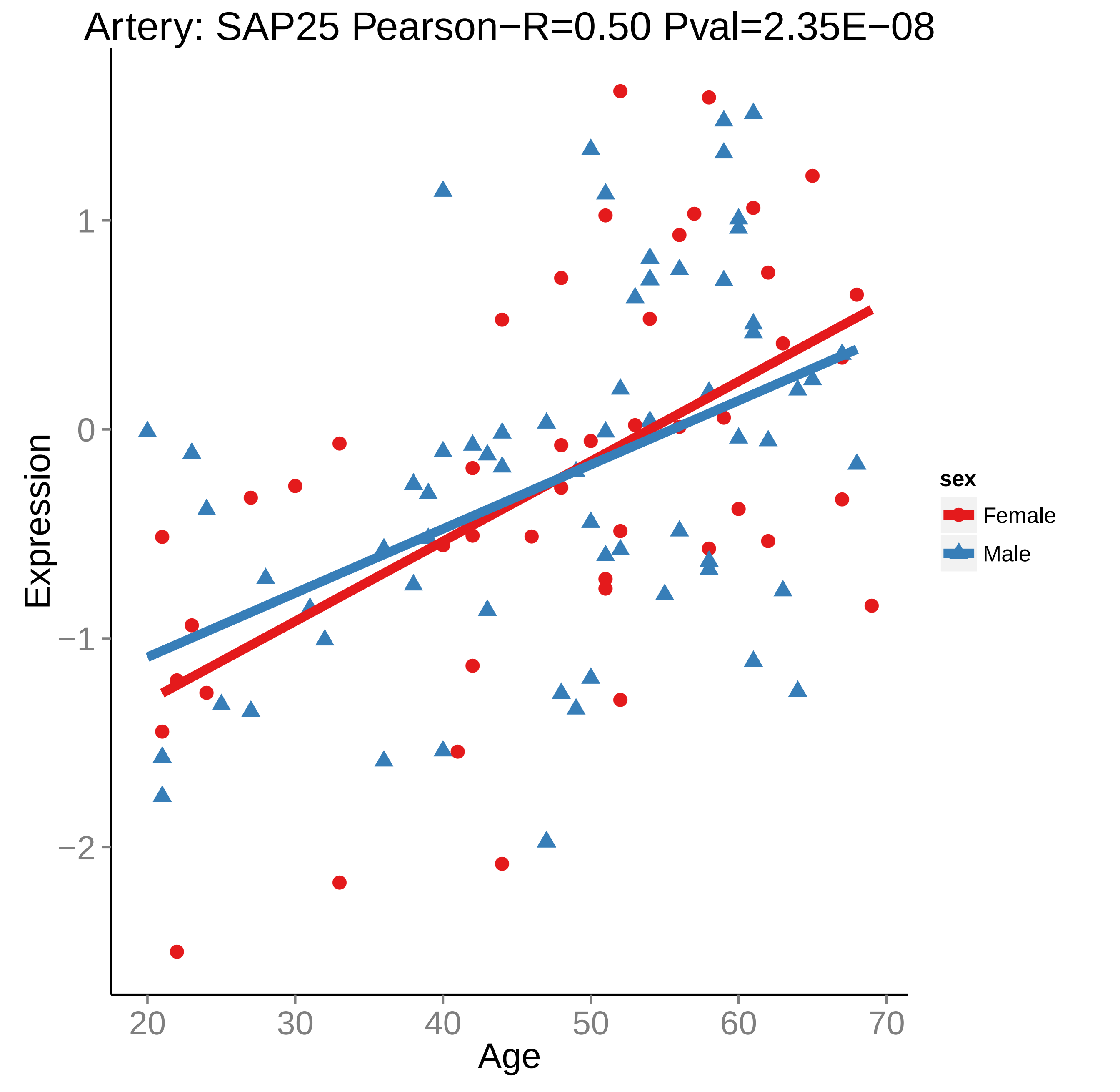
Artery: SAP25 Pearson−R=0.50 Pval=2.35E−08
Expression
1
0
−1
−2
20
30
40
50
60
70
Age
sex
Female
Male

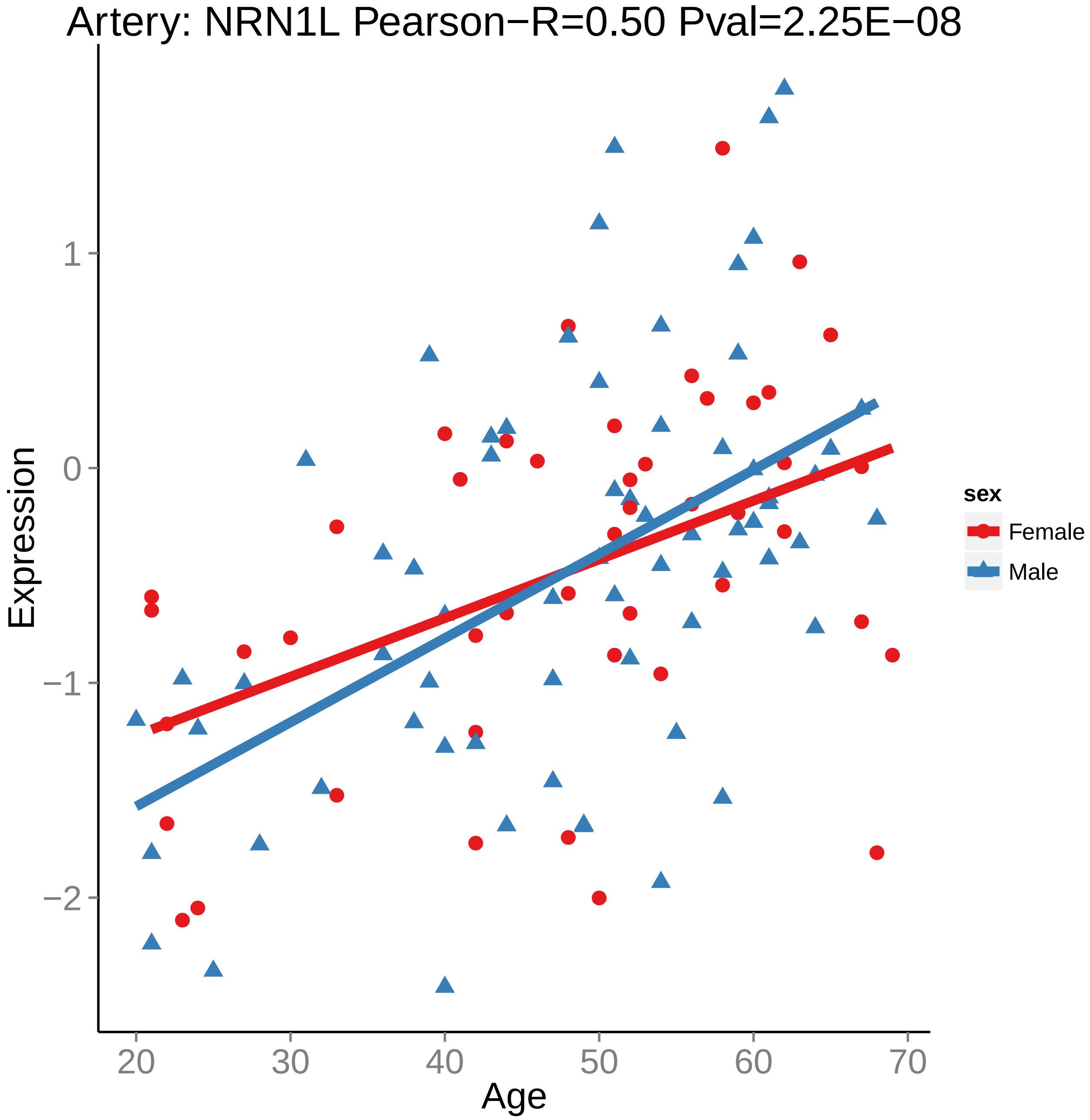

Artery: NRN1L Pearson−R=0.50 Pval=2.25E−08
Expression
1
0
−1
−2
20
30
40
50
60
70
Age
sex
Female
Male

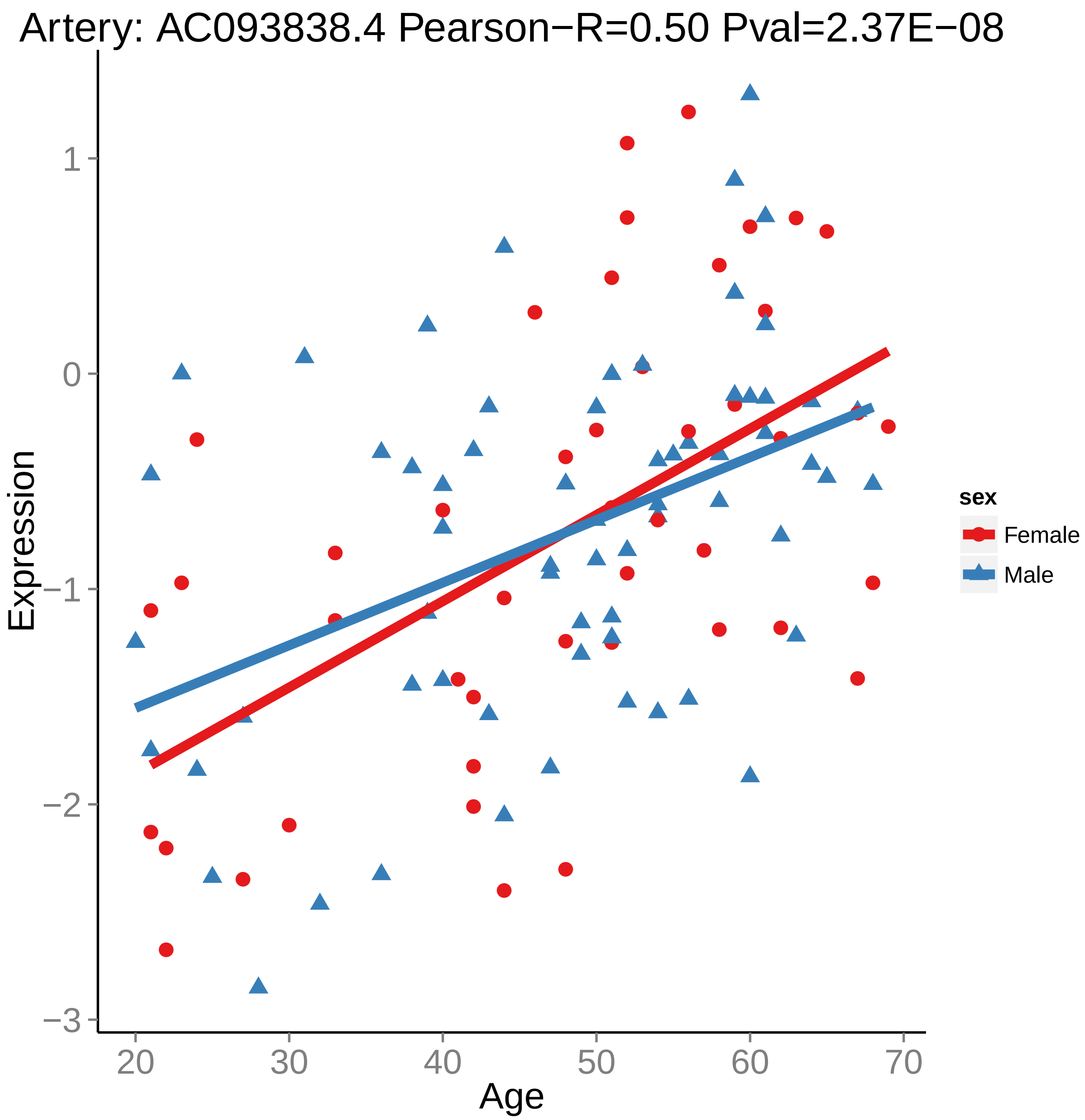

Artery: AC093838.4 Pearson−R=0.50 Pval=2.37E−08
Expression
1
0
−1
−2
−3
20
30
40
50
60
70
Age
sex
Female
Male

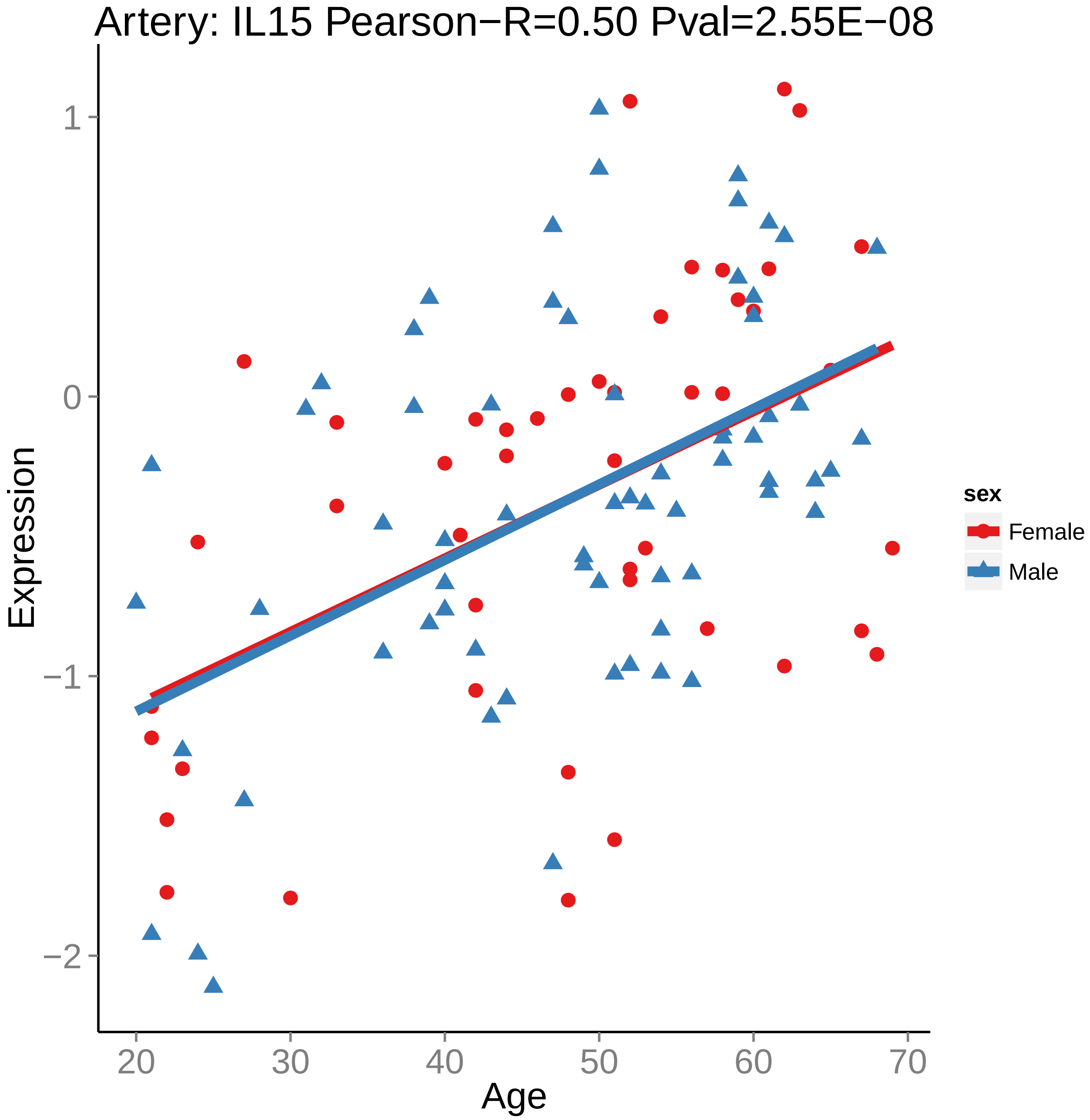

Artery: IL15 Pearson−R=0.50 Pval=2.55E−08
Expression
Age
1
0
−1
−2
20
30
40
50
60
70
sex
Female
Male

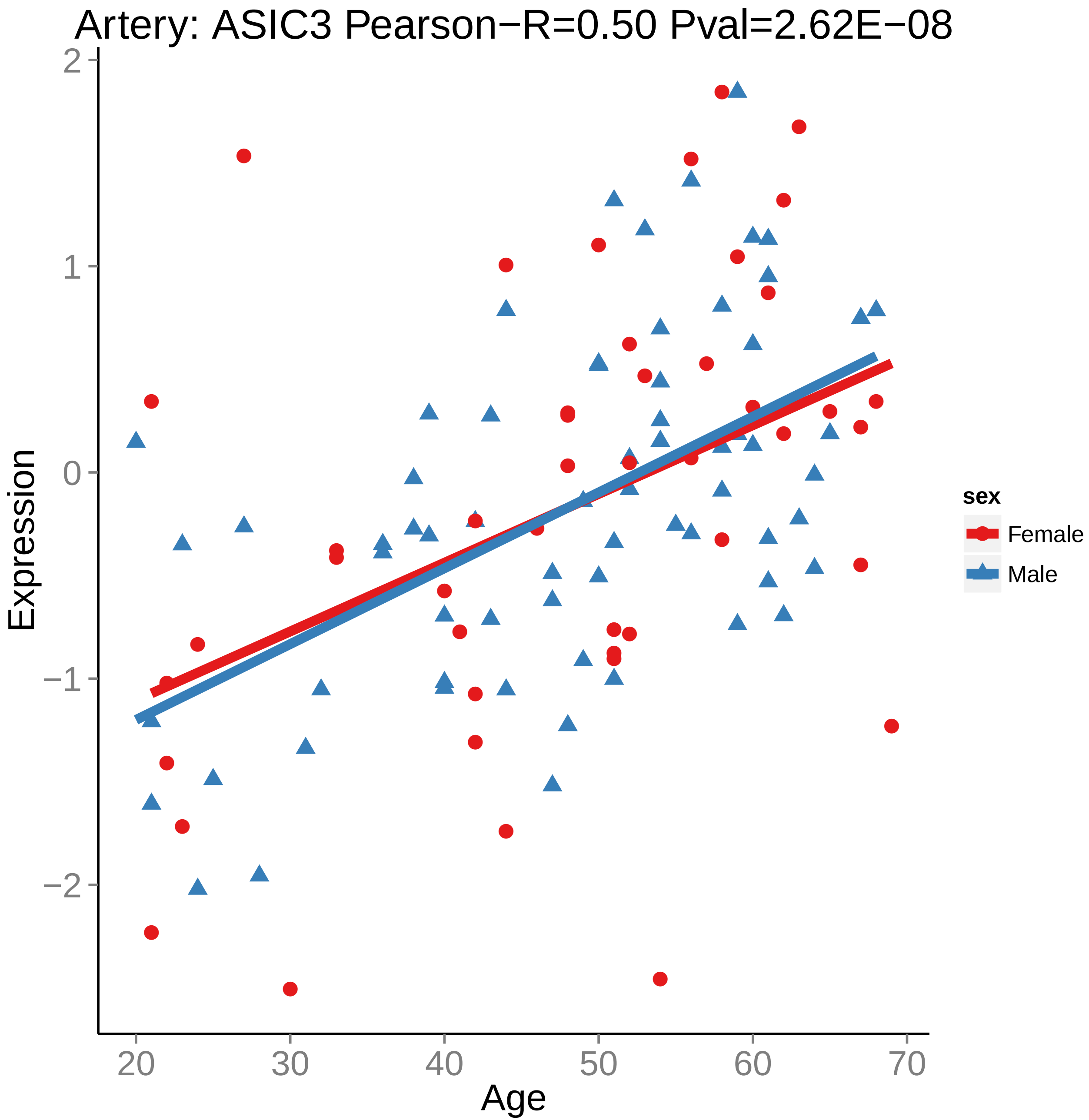

Artery: ASIC3 Pearson−R=0.50 Pval=2.62E−08
Expression
Age
2
1
0
−1
−2
20
30
40
50
60
70
sex
Female
Male

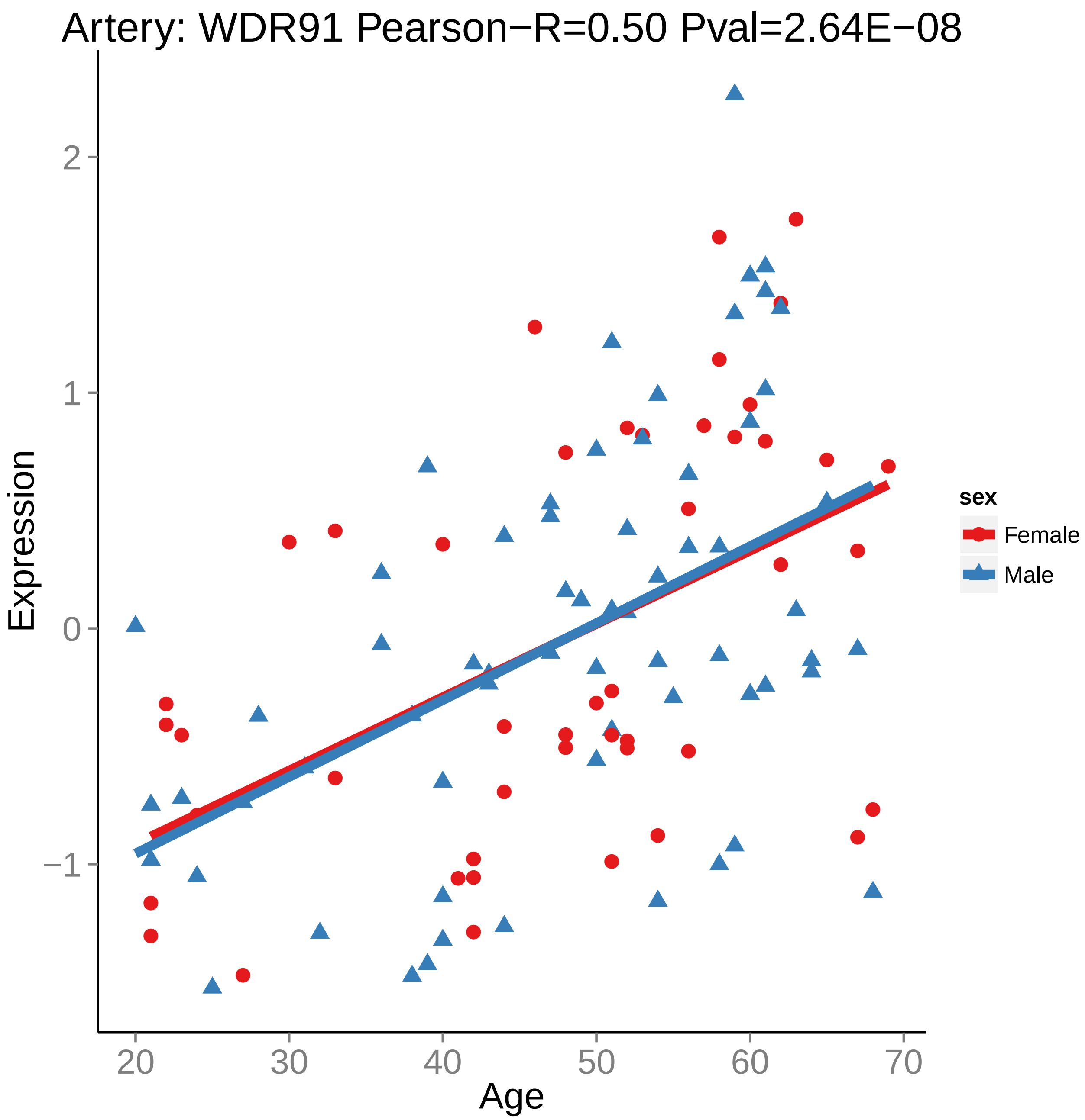
Artery: WDR91 Pearson−R=0.50 Pval=2.64E−08
Expression
Age
2
1
0
−1
20
30
40
50
60
70
sex
Female
Male

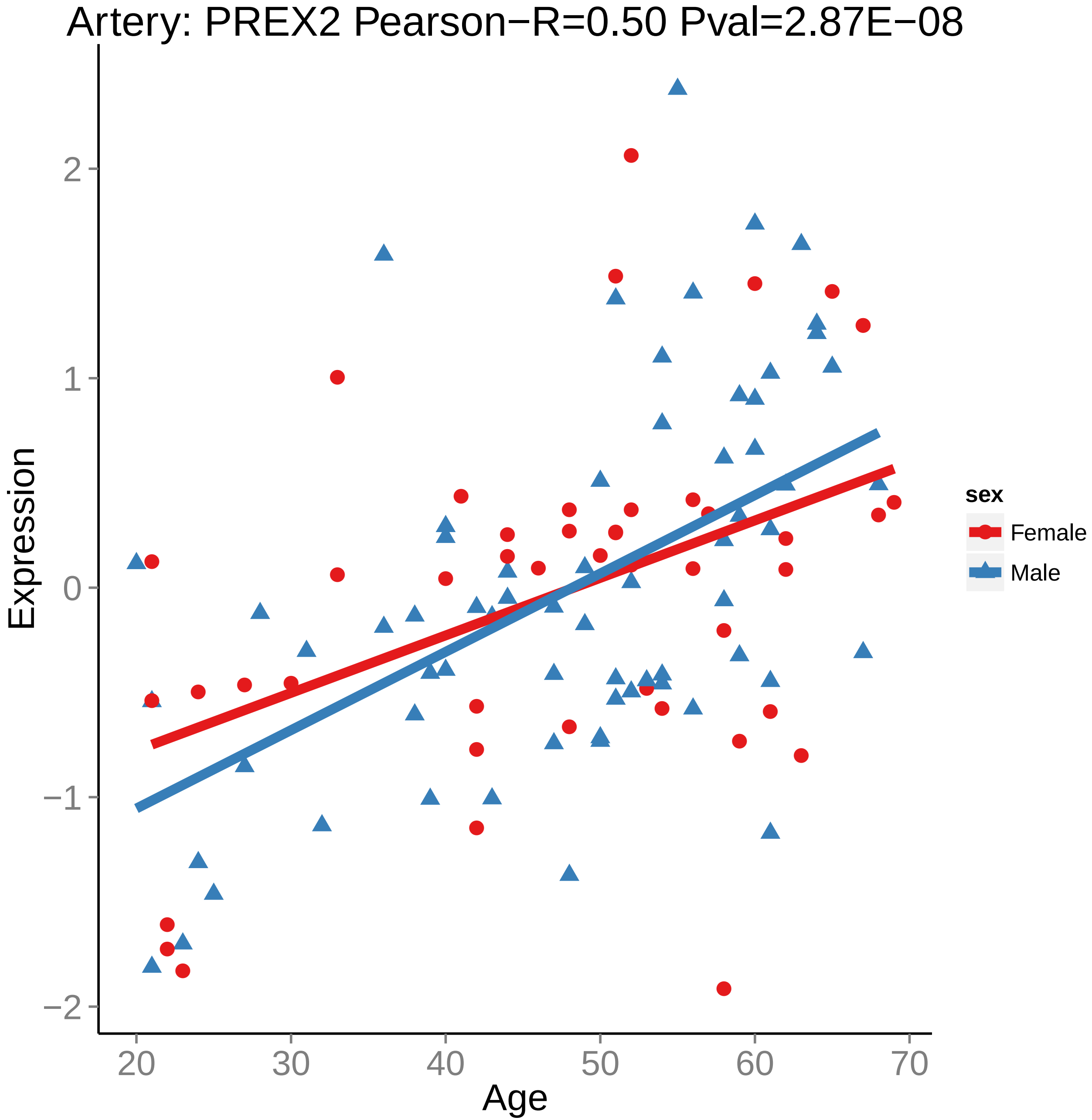

Artery: PREX2 Pearson−R=0.50 Pval=2.87E−08
Expression
2
1
0
−1
−2
20
30
40
50
60
70
Age
sex
Female
Male

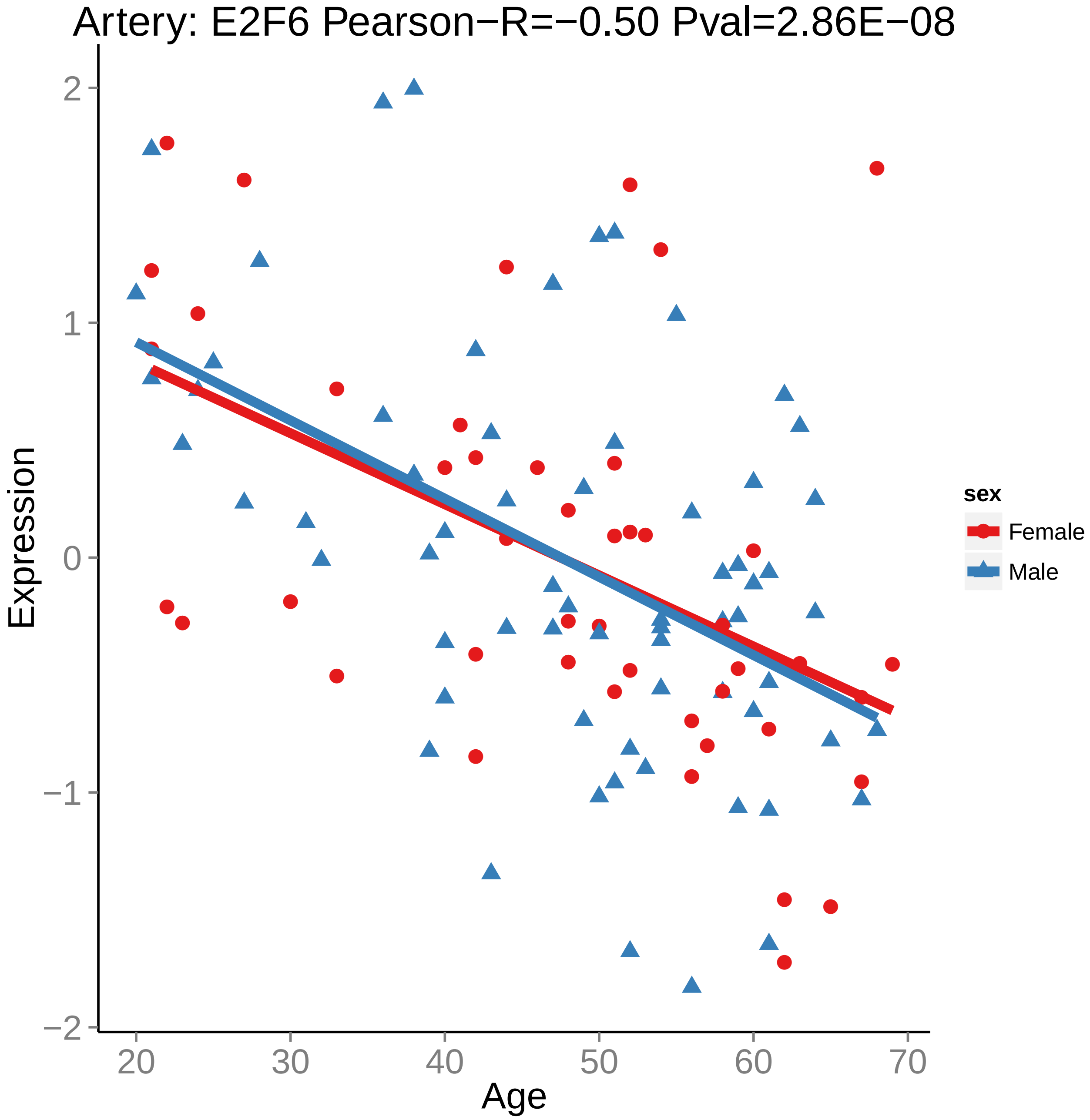

Artery: E2F6 Pearson−R=−0.50 Pval=2.86E−08
Expression
2
1
0
−1
−2
Age
20
30
40
50
60
70
sex
Female
Male

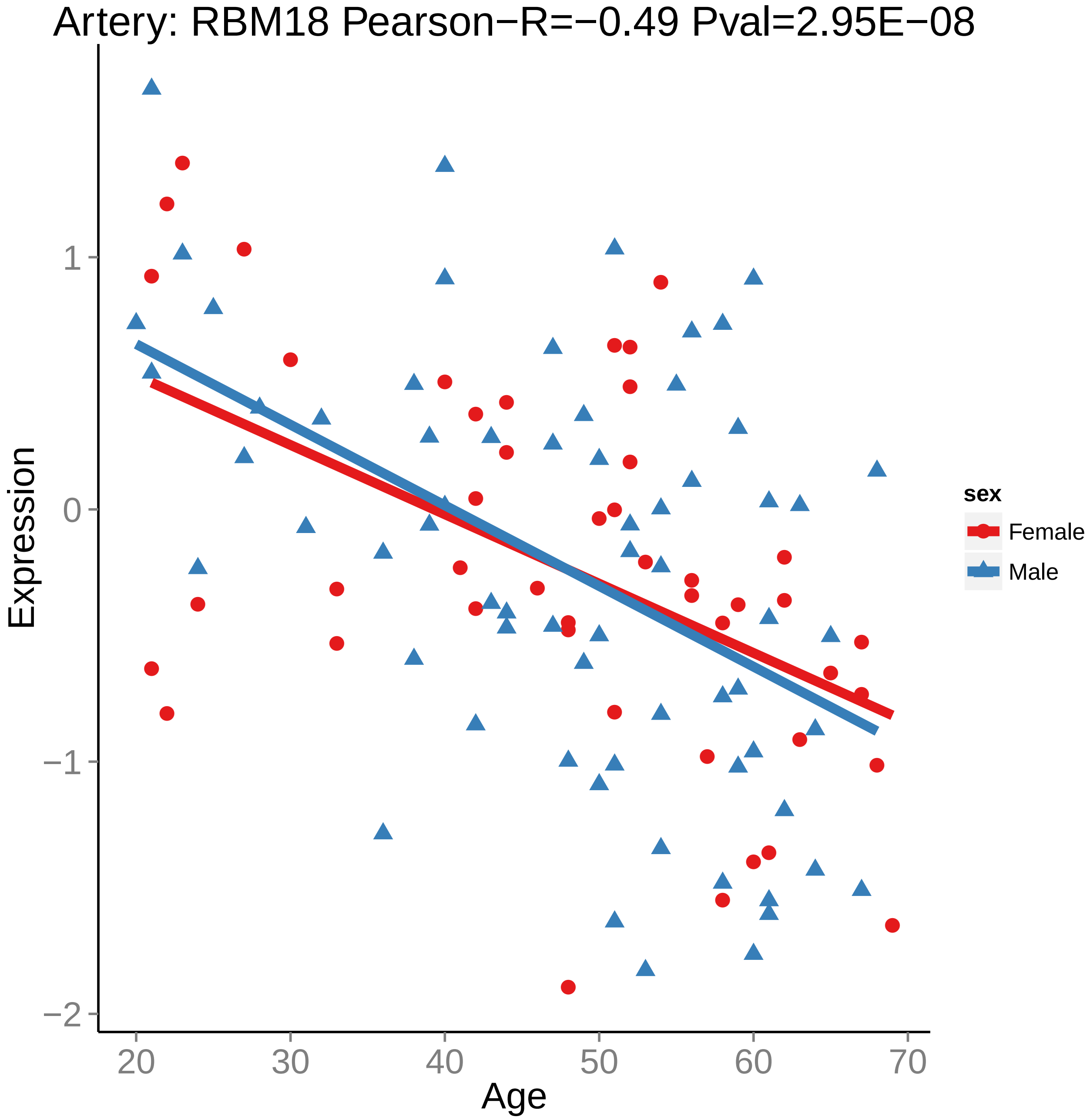
Artery: RBM18 Pearson−R=−0.49 Pval=2.95E−08
Expression
Age
1
0
−1
−2
20
30
40
50
60
70
sex
Female
Male

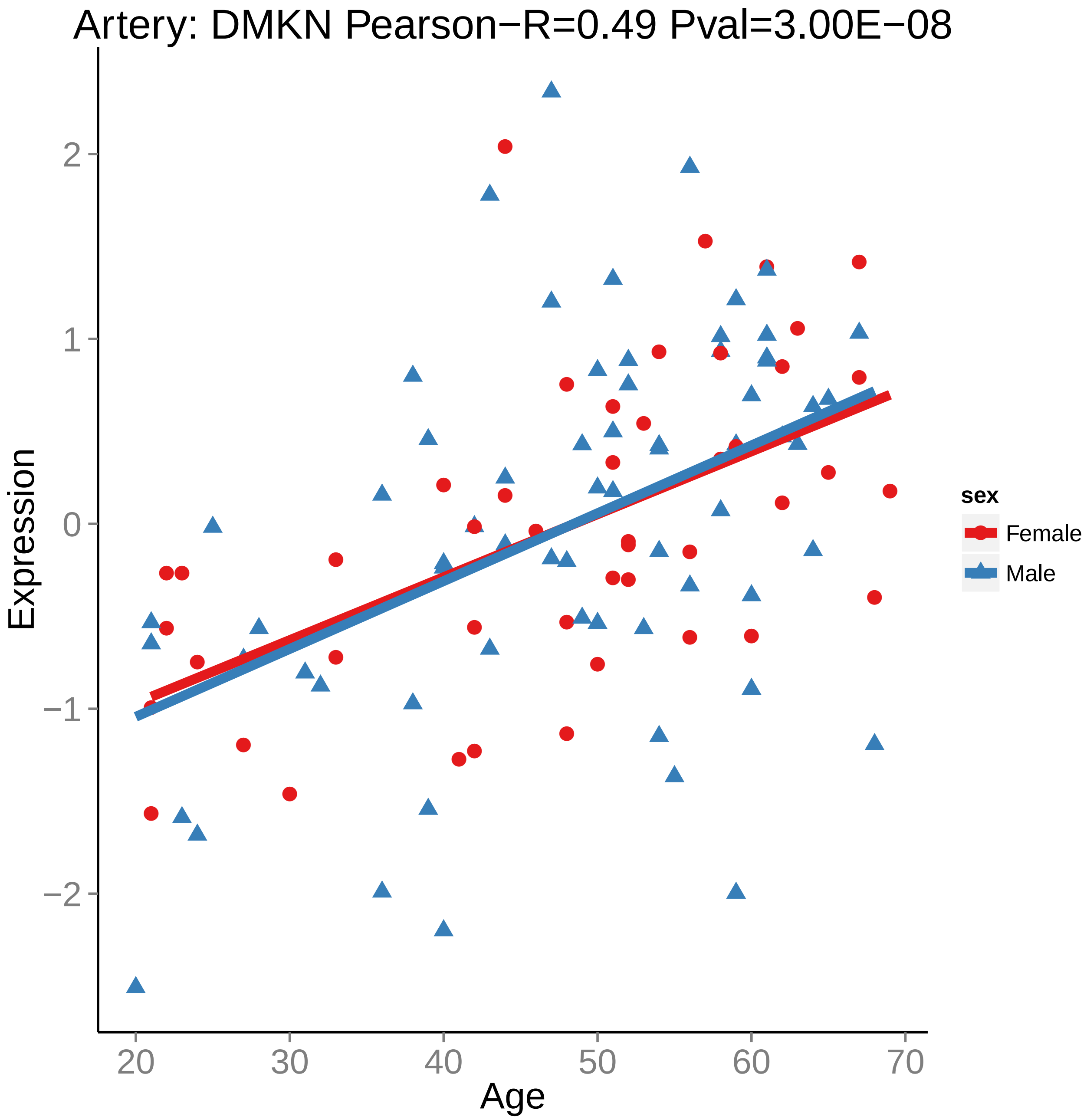

Artery: DMKN Pearson−R=0.49 Pval=3.00E−08
Expression
Age
2
1
0
−1
−2
20
30
40
50
60
70
sex
Female
Male

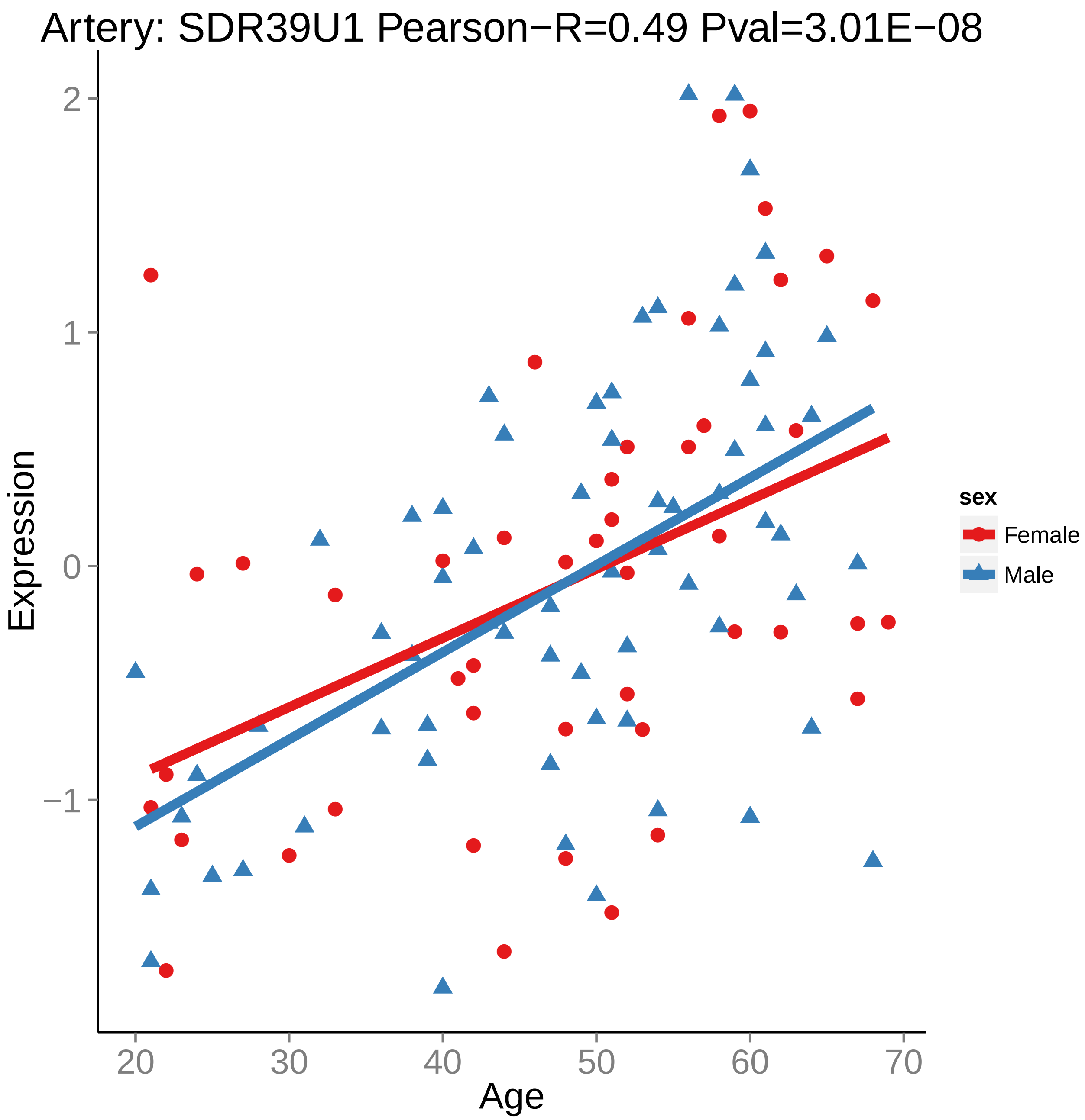

Artery: SDR39U1 Pearson−R=0.49 Pval=3.01E−08
Expression
2
1
0
−1
20
30
40
50
60
70
Age
sex
Female
Male

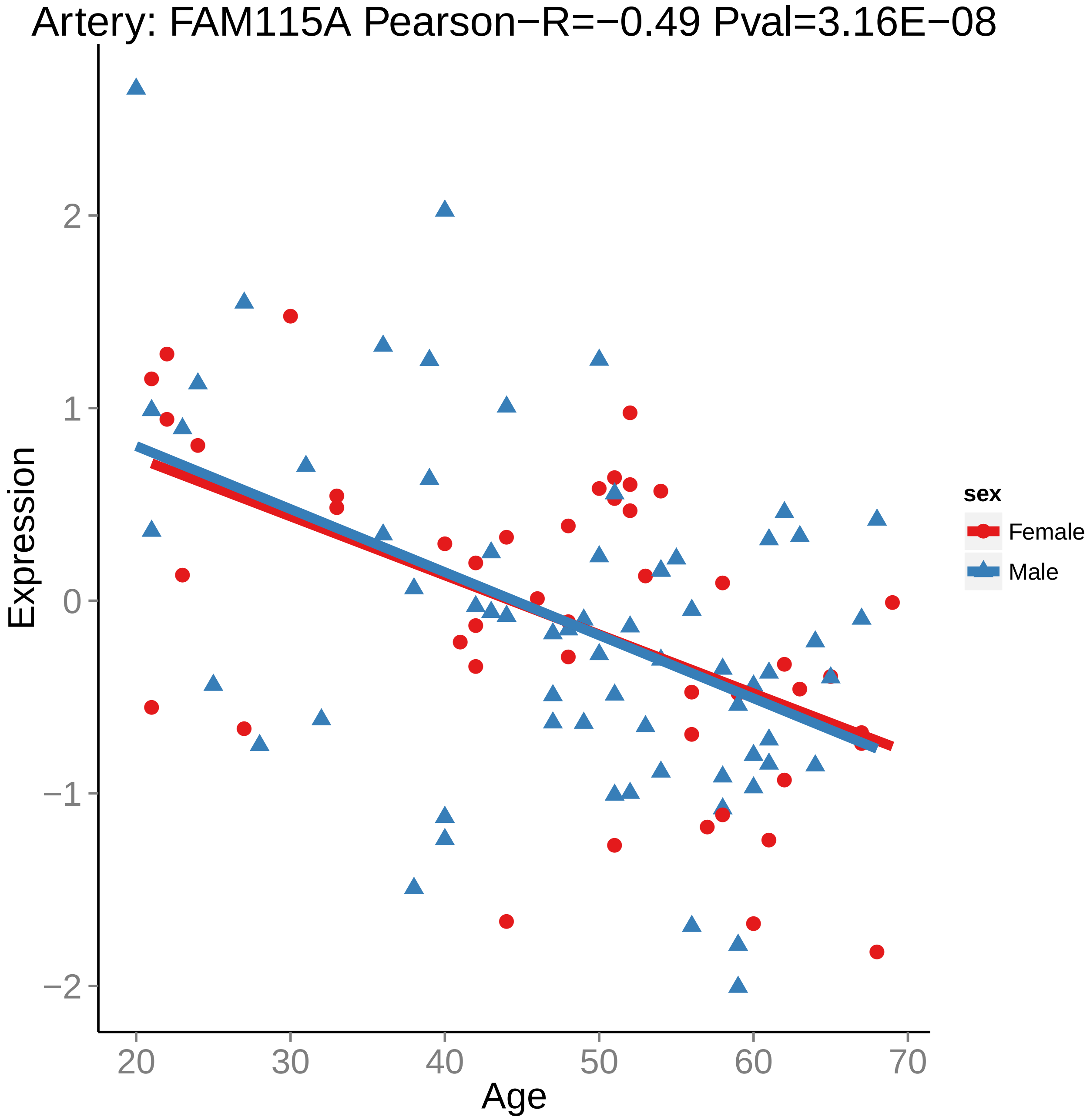

Artery: FAM115A Pearson−R=−0.49 Pval=3.16E−08
Expression
2
1
0
−1
−2
20
30
40
50
60
70
Age
sex
Female
Male

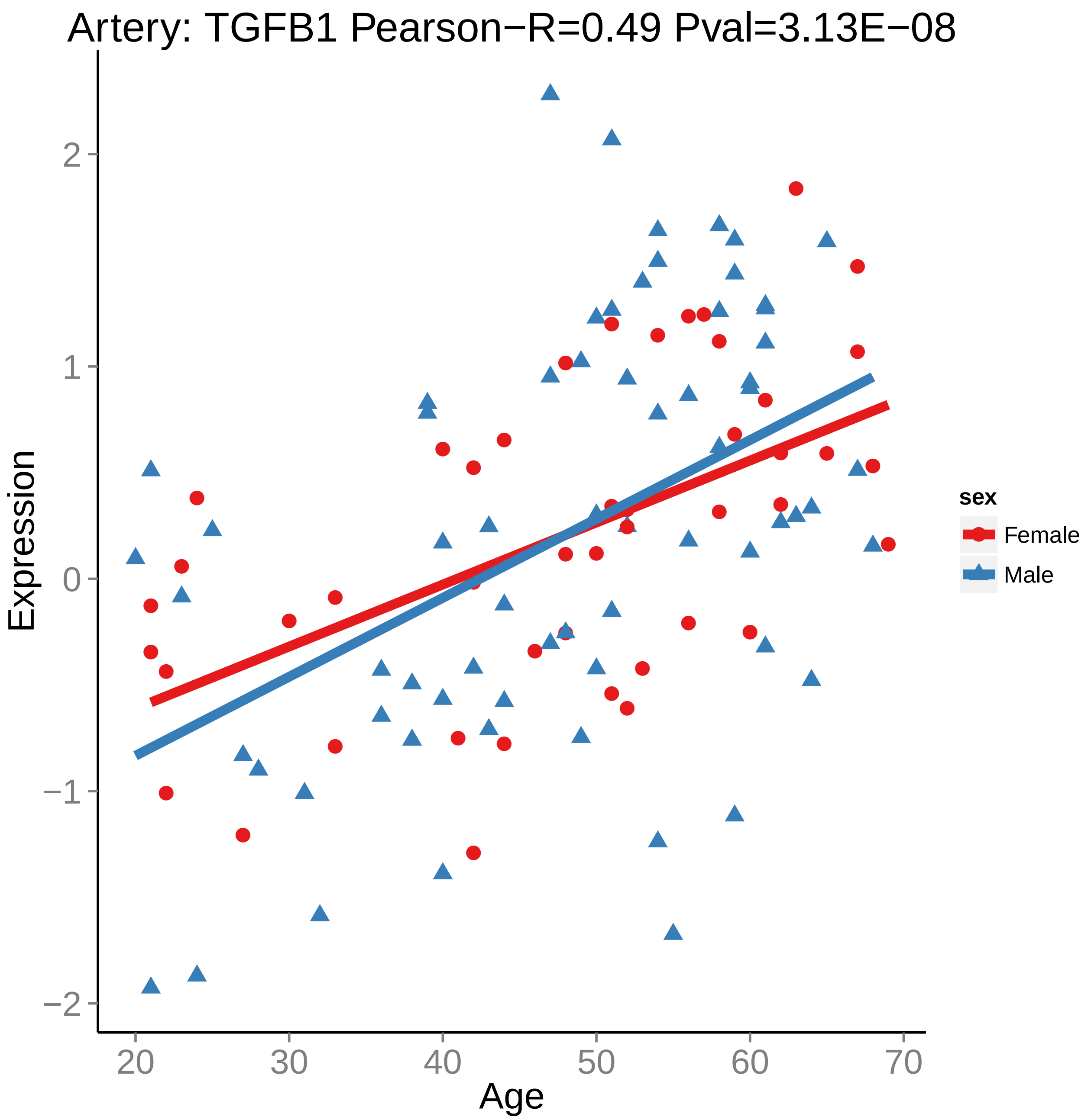
Artery: TGFB1 Pearson−R=0.49 Pval=3.13E−08
Expression
Age
2
1
0
−1
−2
20
30
40
50
60
70
sex
Female
Male

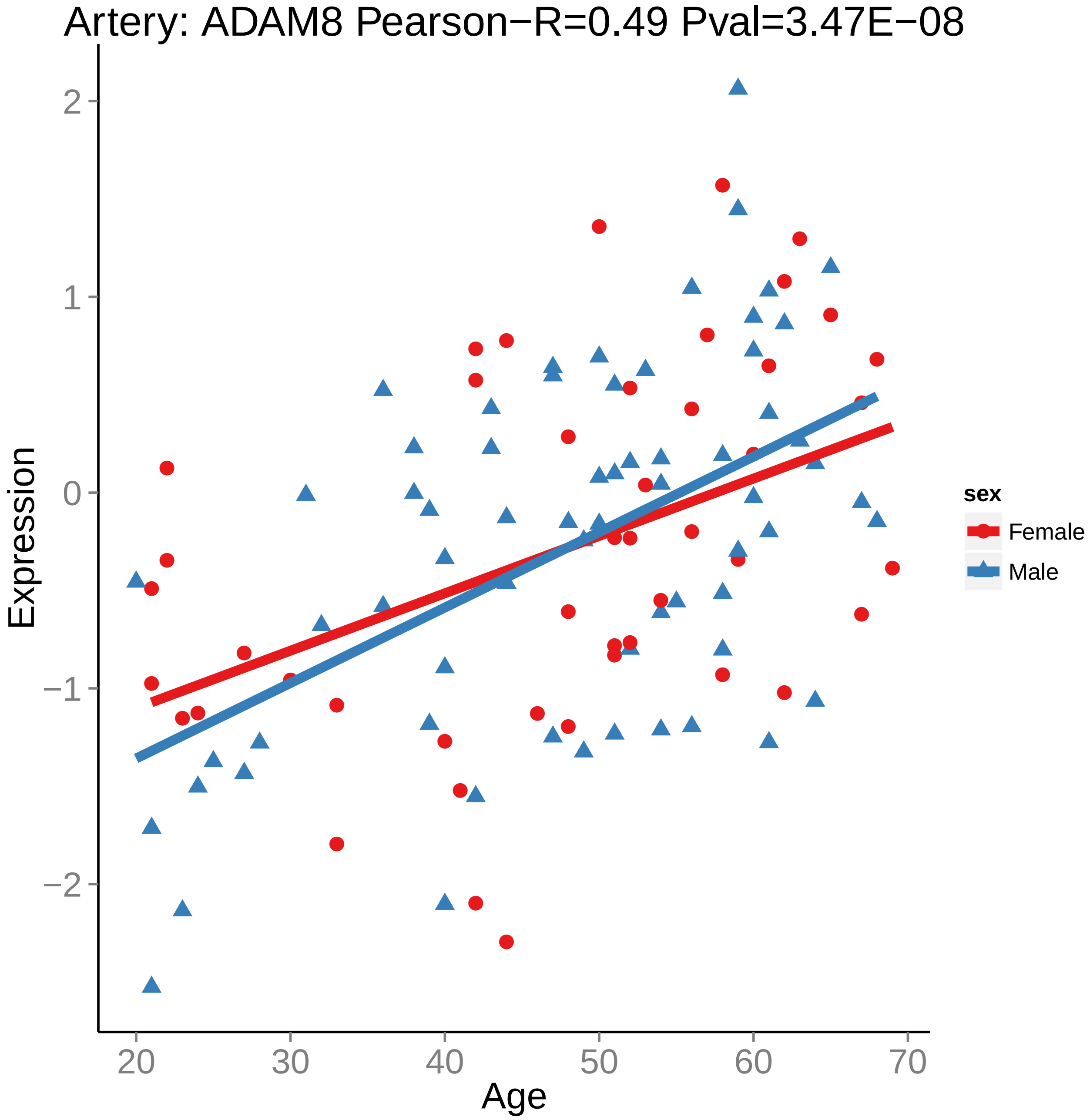

Artery: ADAM8 Pearson−R=0.49 Pval=3.47E−08
Expression
2
1
0
−1
−2
20
30
40
50
60
70
Age
sex
Female
Male

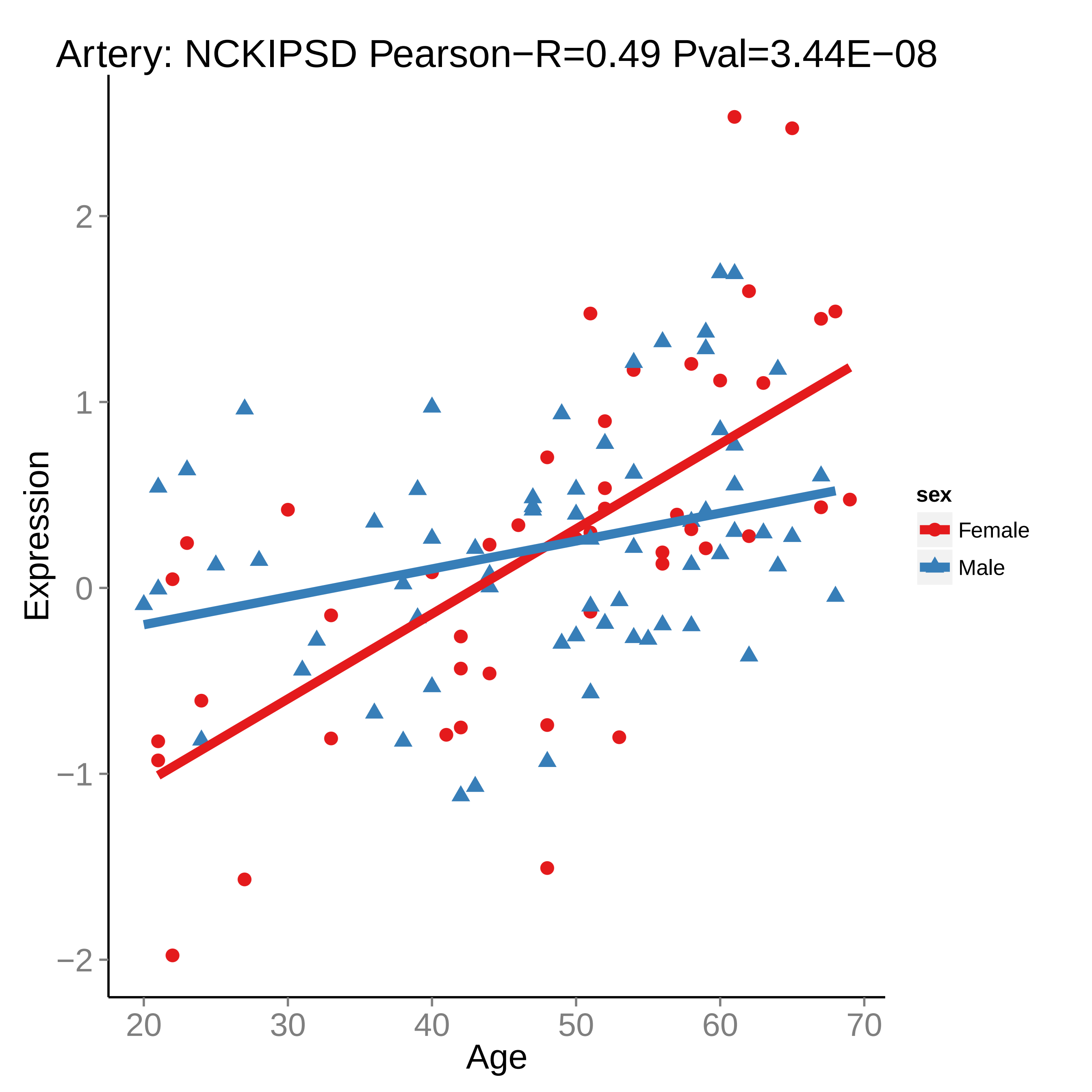

Artery: NCKIPSD Pearson−R=0.49 Pval=3.44E−08
Expression
2
1
0
−1
−2
20
30
40
50
60
70
Age
sex
Female
Male

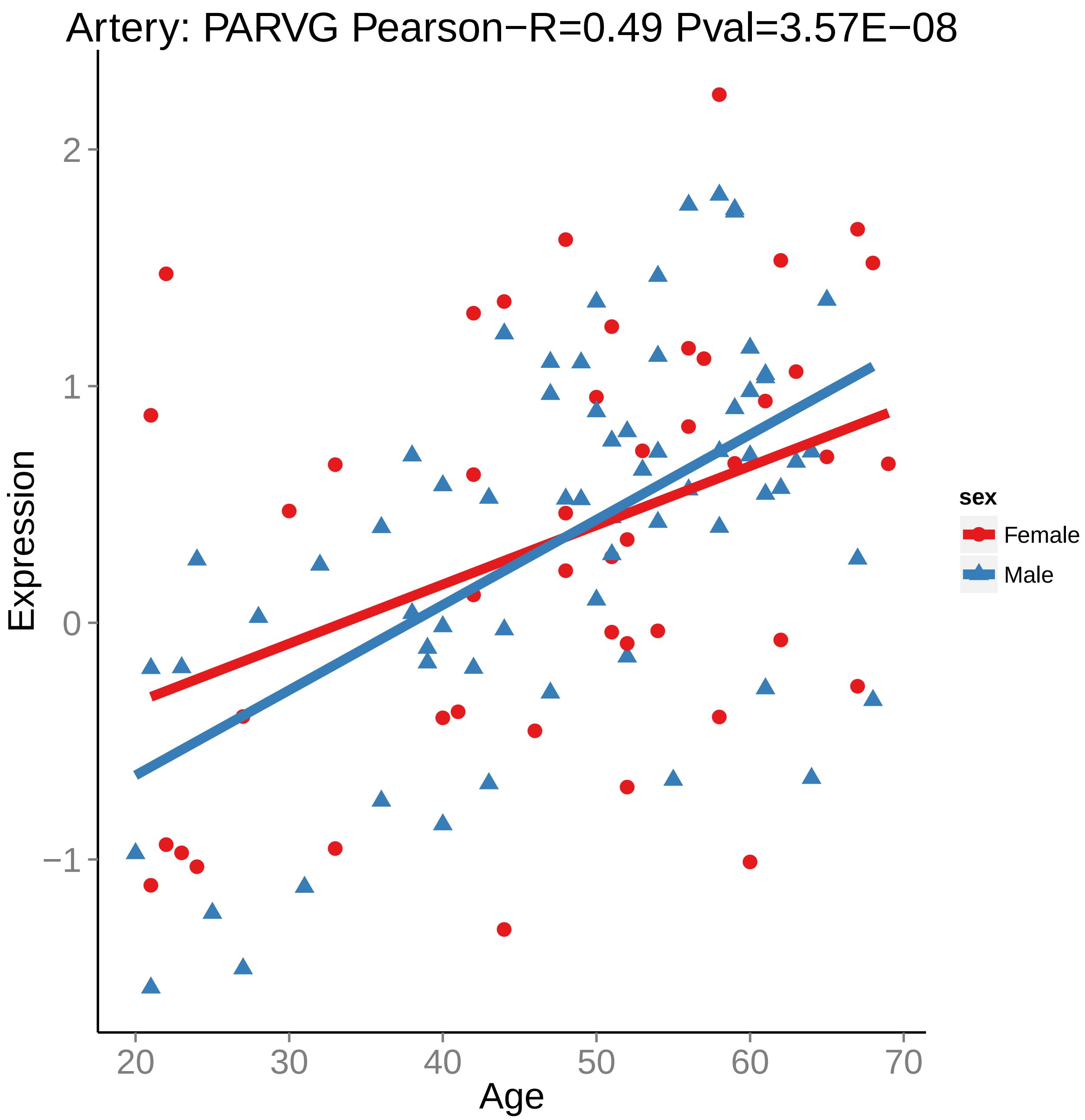

Artery: PARVG Pearson−R=0.49 Pval=3.57E−08
Expression
Age
2
1
0
−1
20
30
40
50
60
70
sex
Female
Male

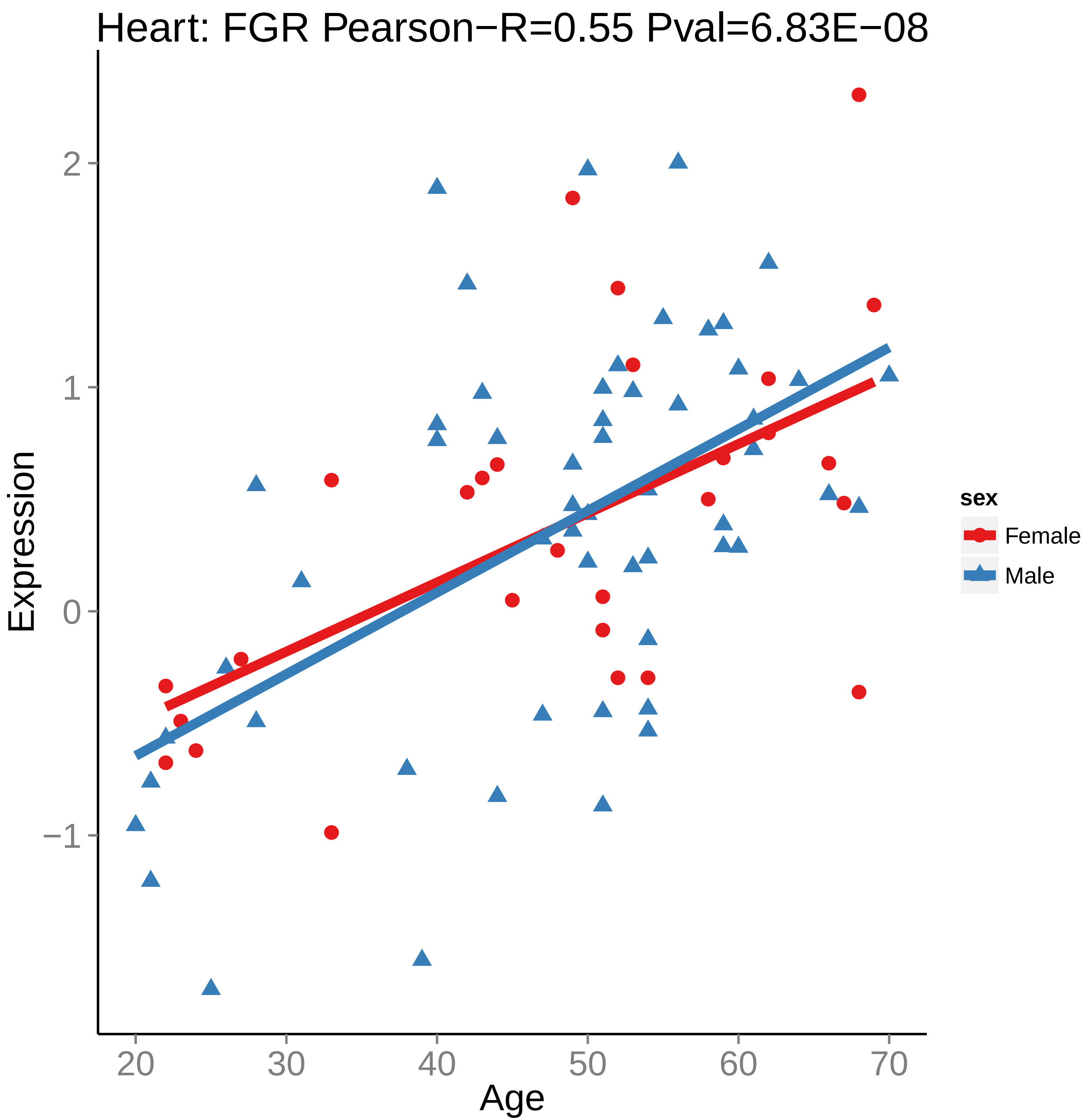

Heart: FGR Pearson−R=0.55 Pval=6.83E−08
Expression
Age
2
1
0
−1
20
30
40
50
60
70
sex
Female
Male

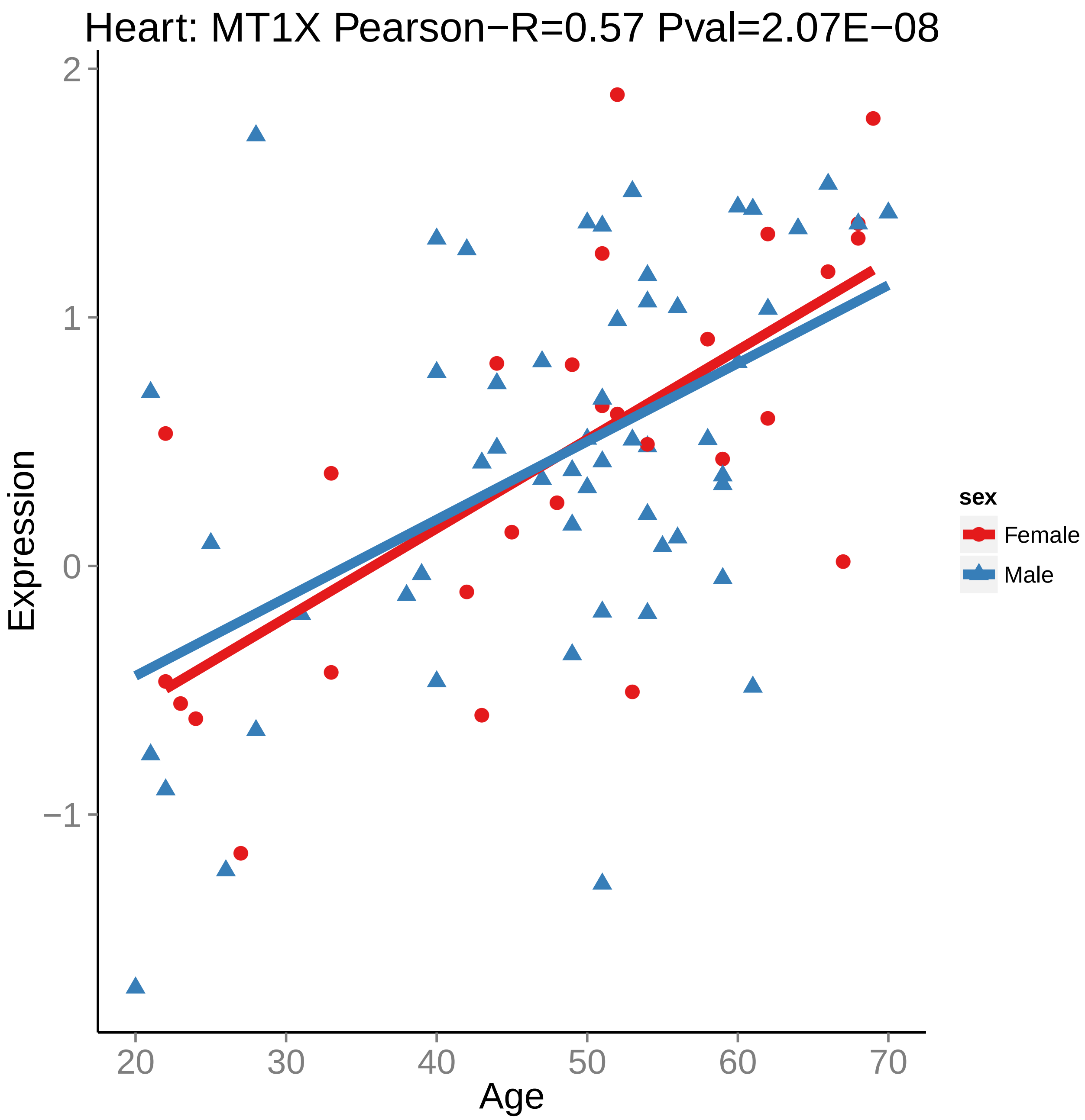
Heart: MT1X Pearson−R=0.57 Pval=2.07E−08
Expression
Age
2
1
0
−1
20
30
40
50
60
70
sex
Female
Male

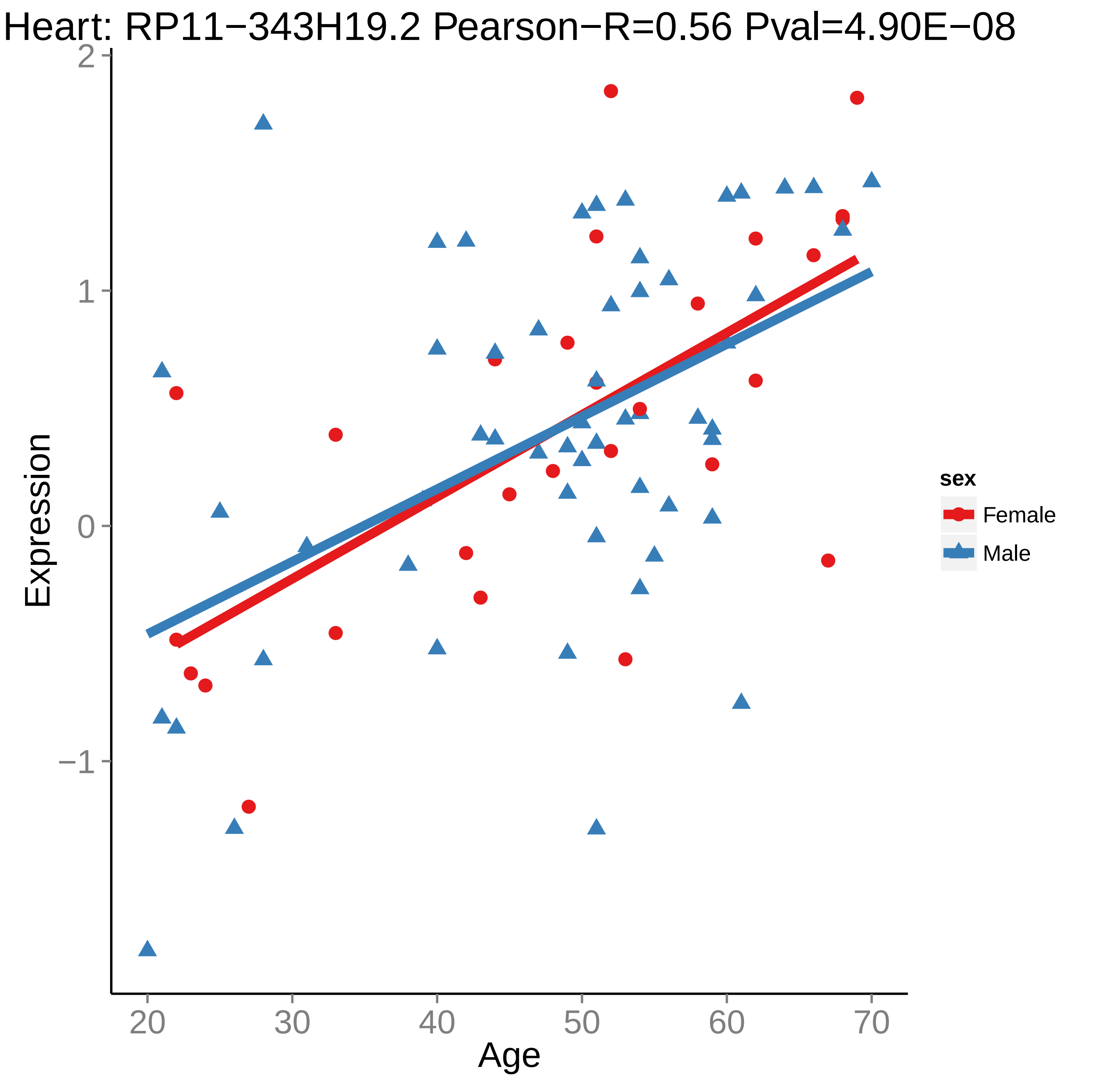

Heart: RP11−343H19.2 Pearson−R=0.56 Pval=4.90E−08
Expression
Age
2
1
0
−1
20
30
40
50
60
70
sex
Female
Male

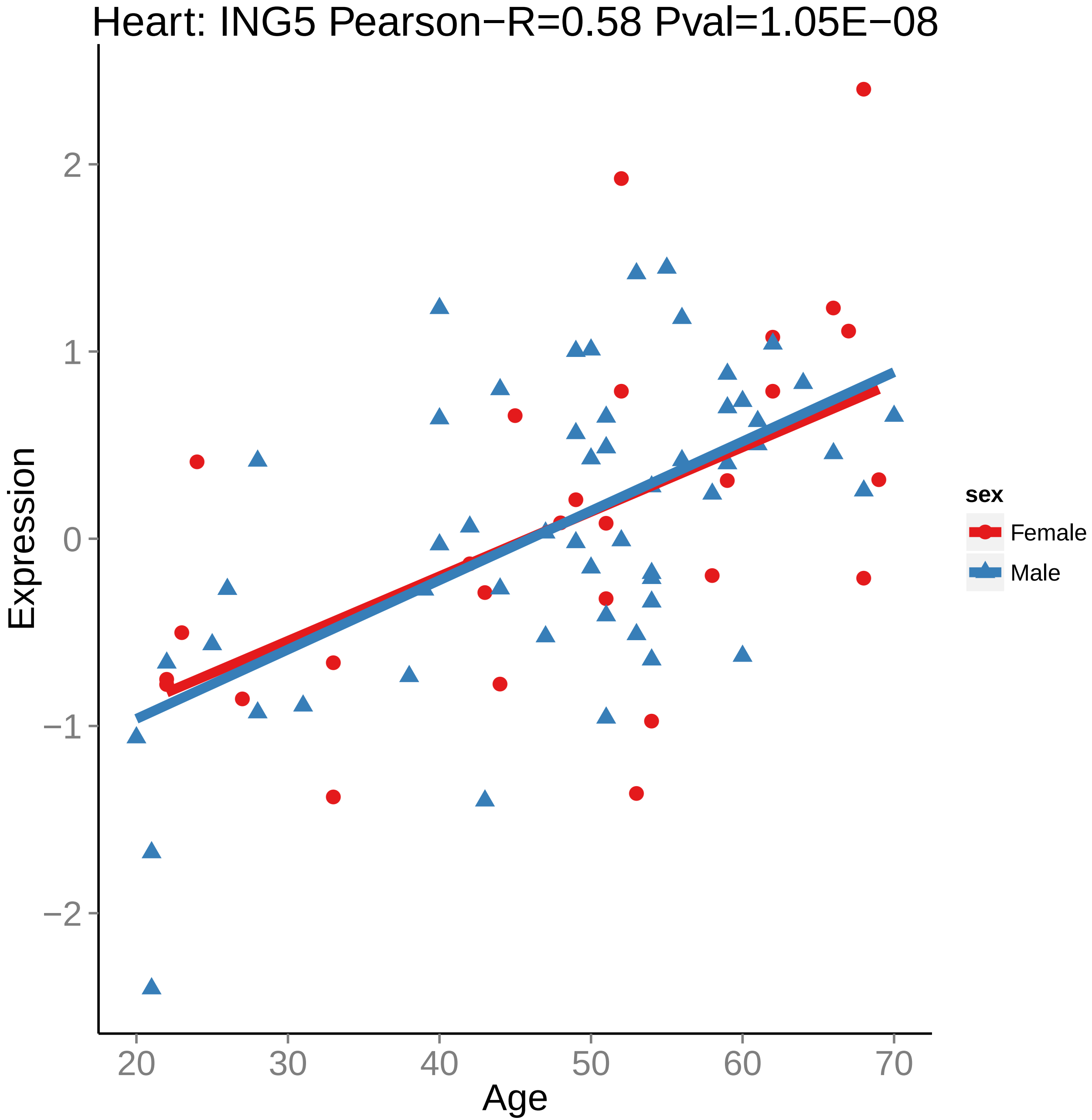

Heart: ING5 Pearson−R=0.58 Pval=1.05E−08
Expression
Age
2
1
0
−1
−2
20
30
40
50
60
70
sex
Female
Male

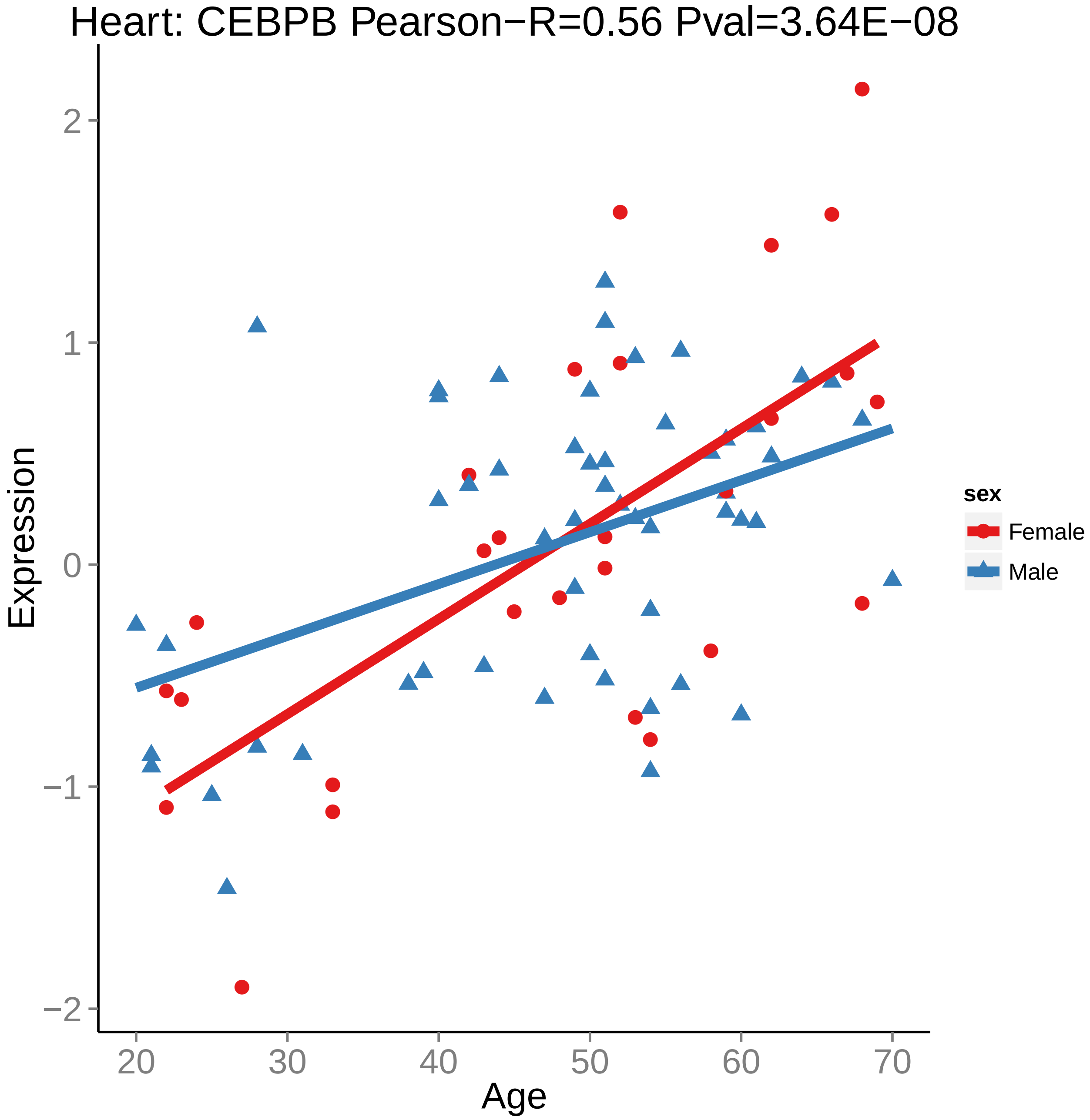
Heart: CEBPB Pearson−R=0.56 Pval=3.64E−08
Expression
Age
2
1
0
−1
−2
20
30
40
50
60
70
sex
Female
Male

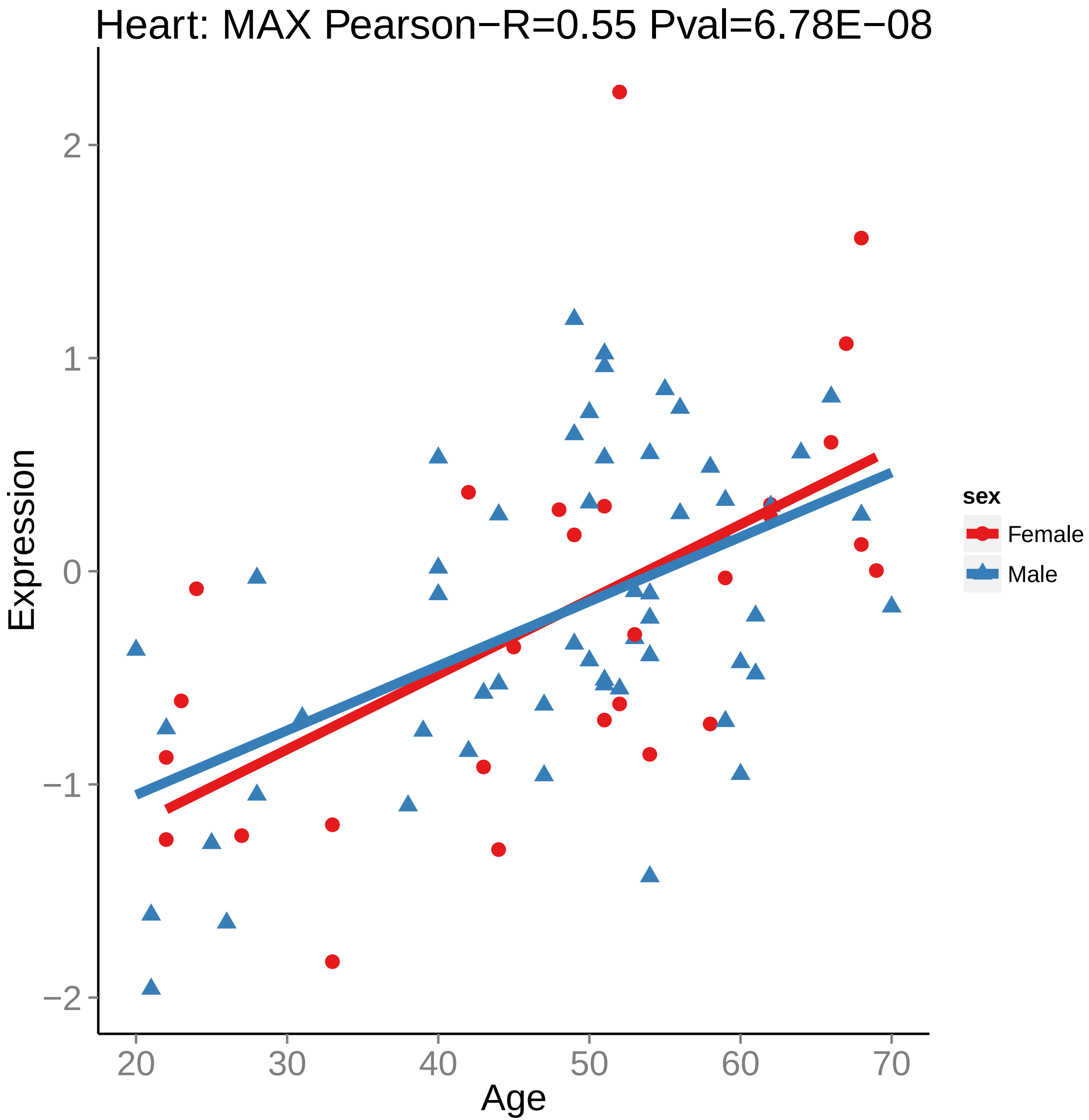

Heart: MAX Pearson−R=0.55 Pval=6.78E−08
Expression
Age
2
1
0
−1
−2
20
30
40
50
60
70
sex
Female
Male

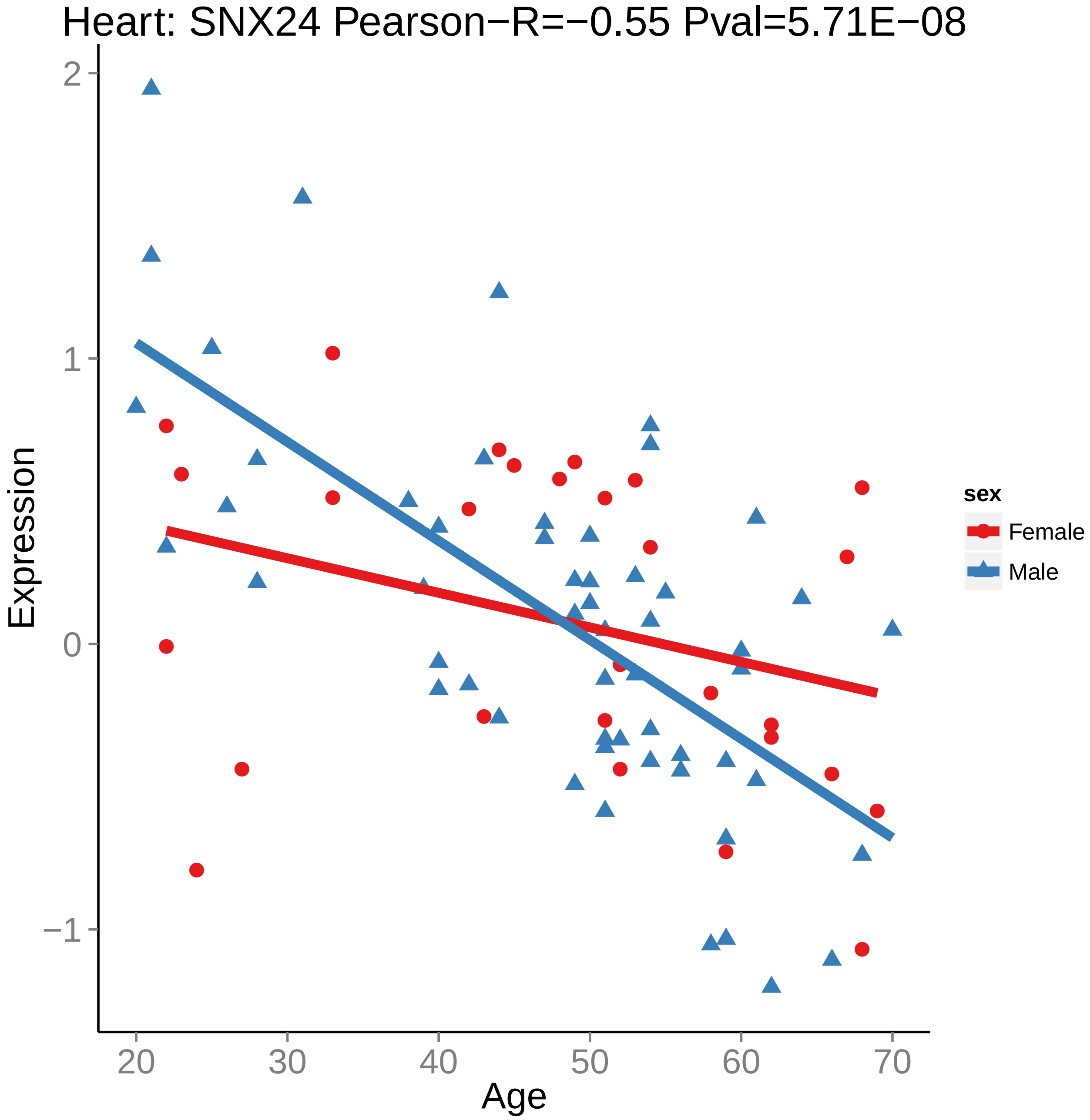

Heart: SNX24 Pearson−R=−0.55 Pval=5.71E−08
Expression
Age
2
1
0
−1
20
30
40
50
60
70
sex
Female
Male

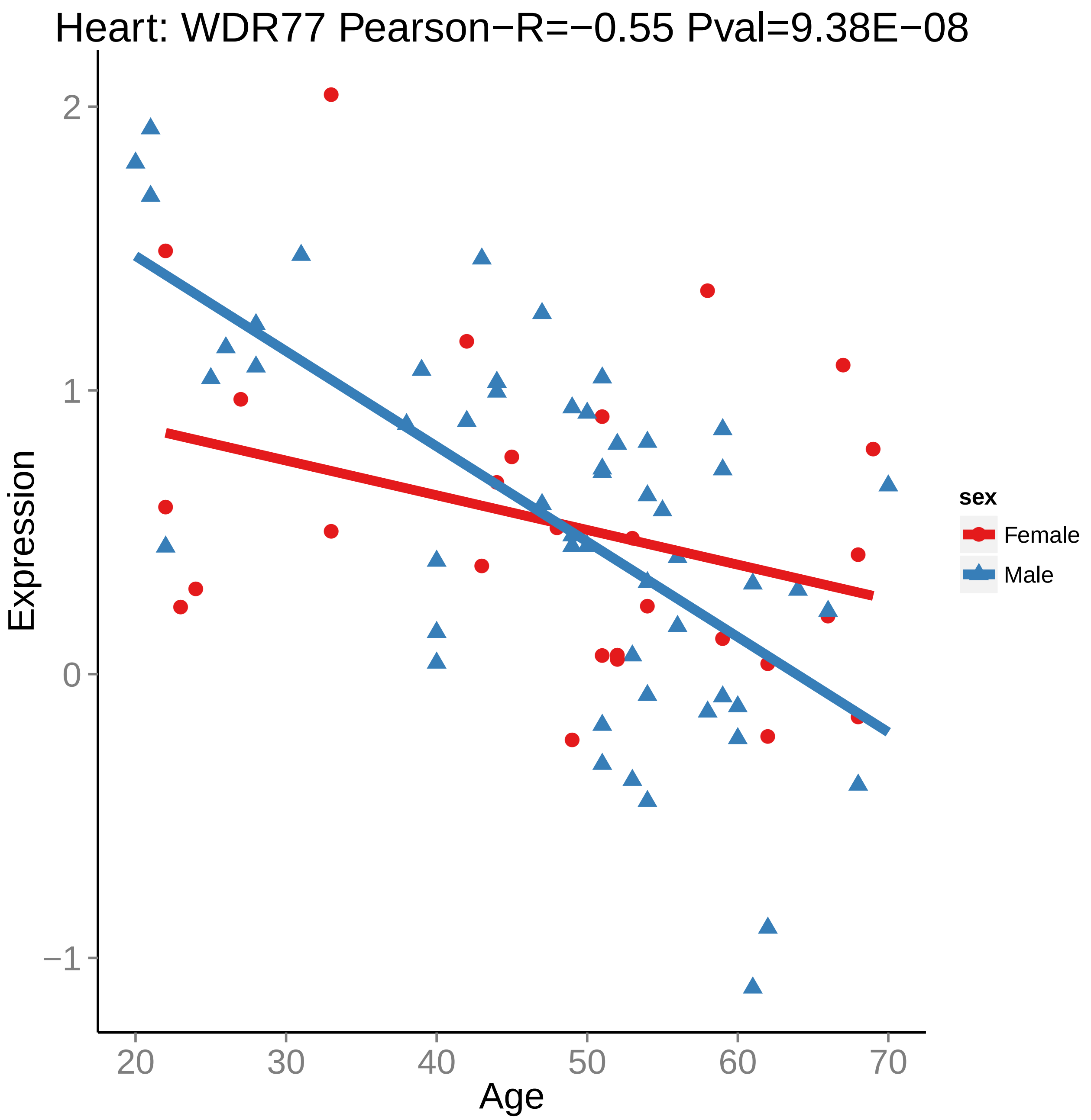

Heart: WDR77 Pearson−R=−0.55 Pval=9.38E−08
Expression
Age
2
1
0
−1
20
30
40
50
60
70
sex
Female
Male

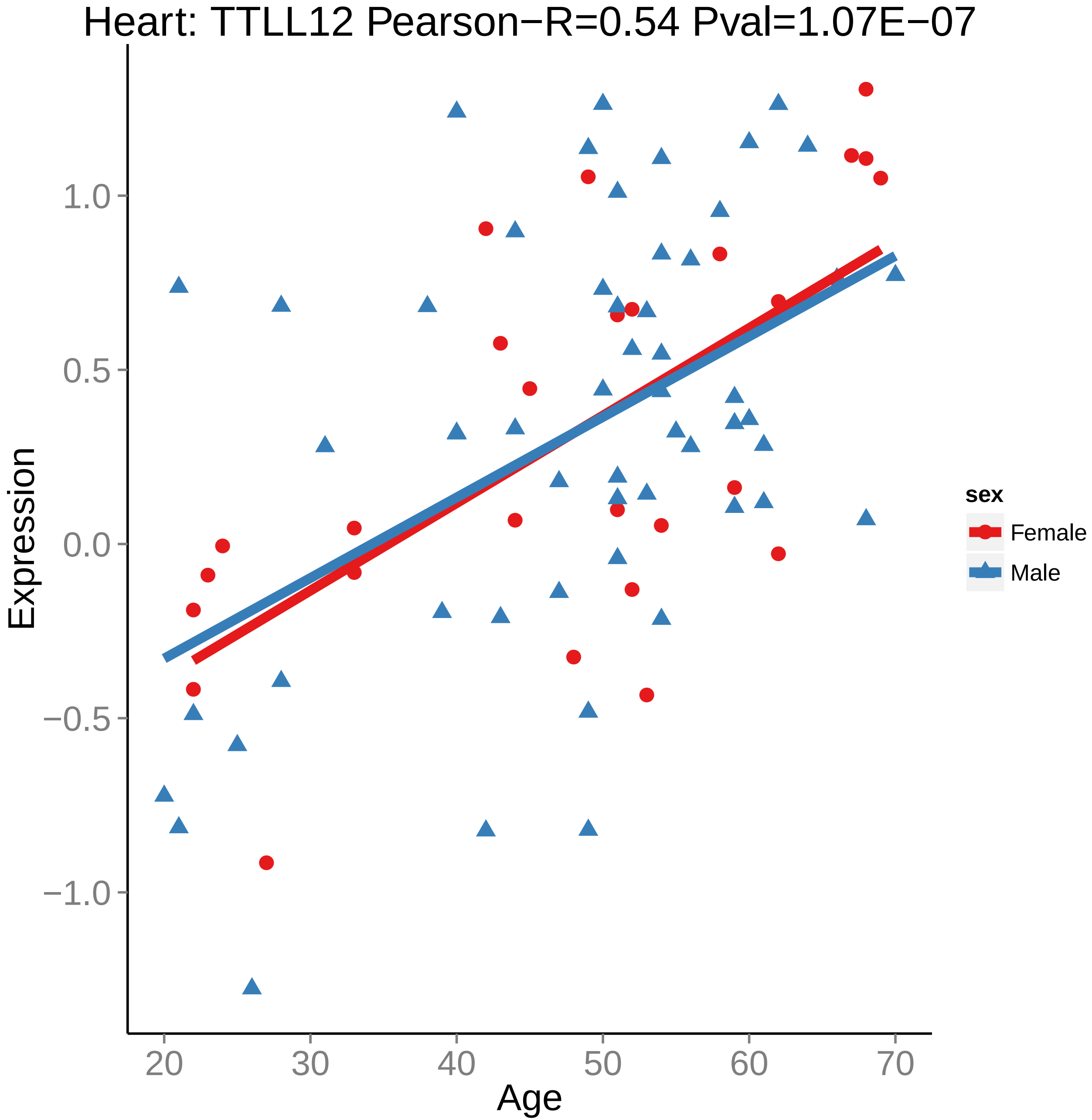

Heart: TTLL12 Pearson−R=0.54 Pval=1.07E−07
Expression
1.0
0.5
0.0
−0.5
−1.0
20
30
40
50
60
70
Age
sex
Female
Male

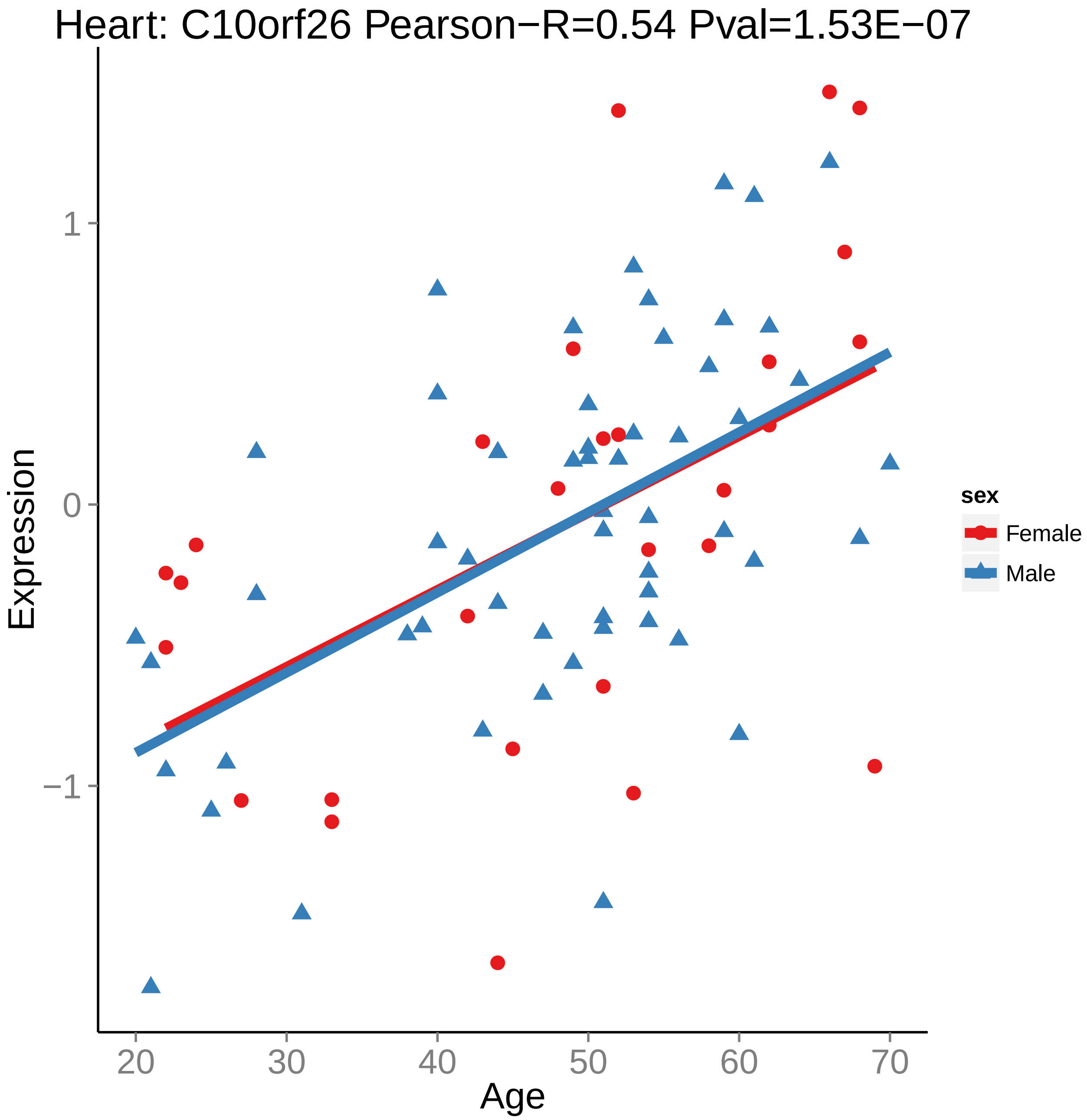

Heart: C10orf26 Pearson−R=0.54 Pval=1.53E−07
Expression
Age
1
0
−1
20
30
40
50
60
70
sex
Female
Male

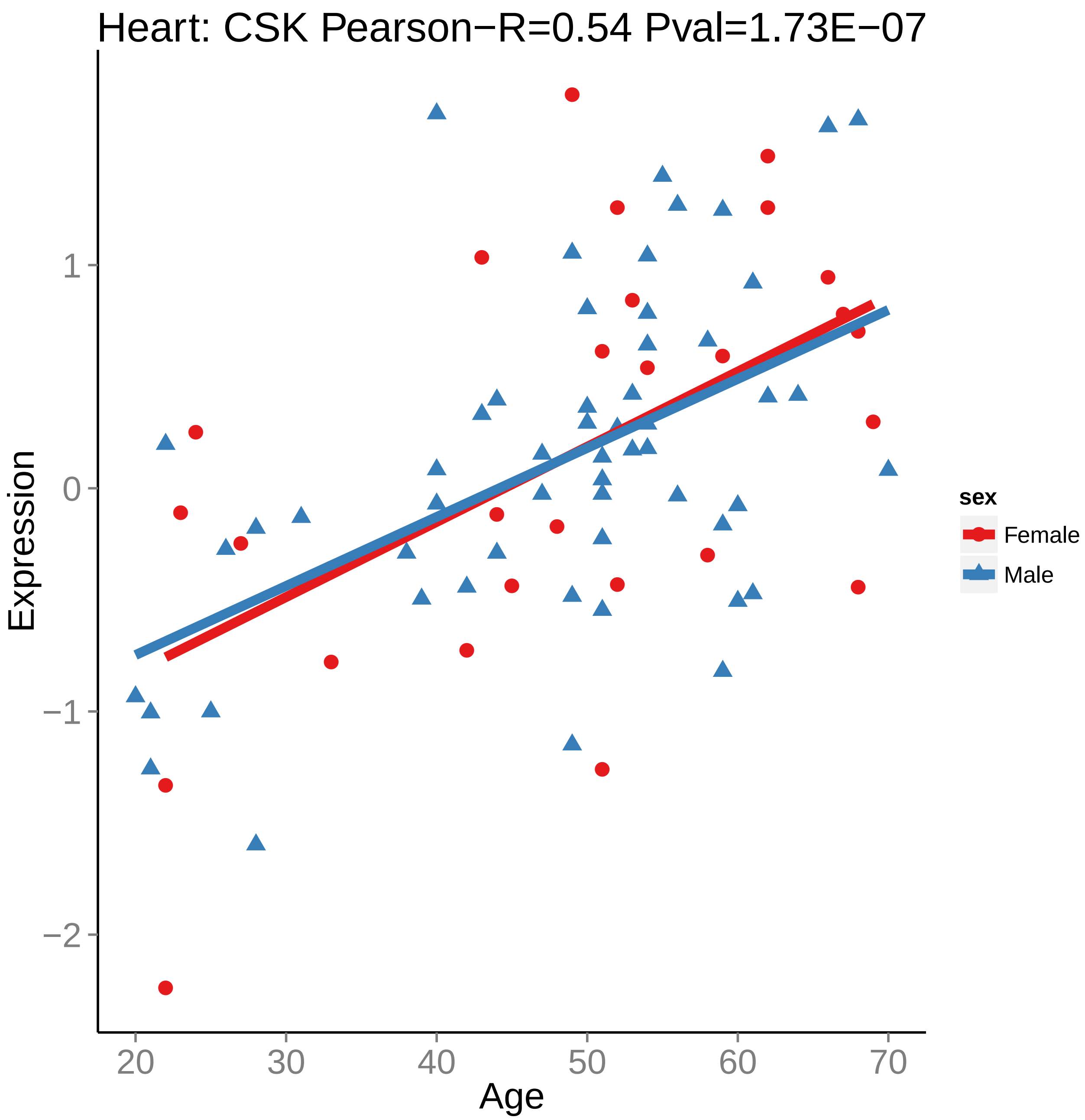

Heart: CSK Pearson−R=0.54 Pval=1.73E−07
Expression
1
0
−1
−2
20
30
40
50
60
70
Age
sex
Female
Male

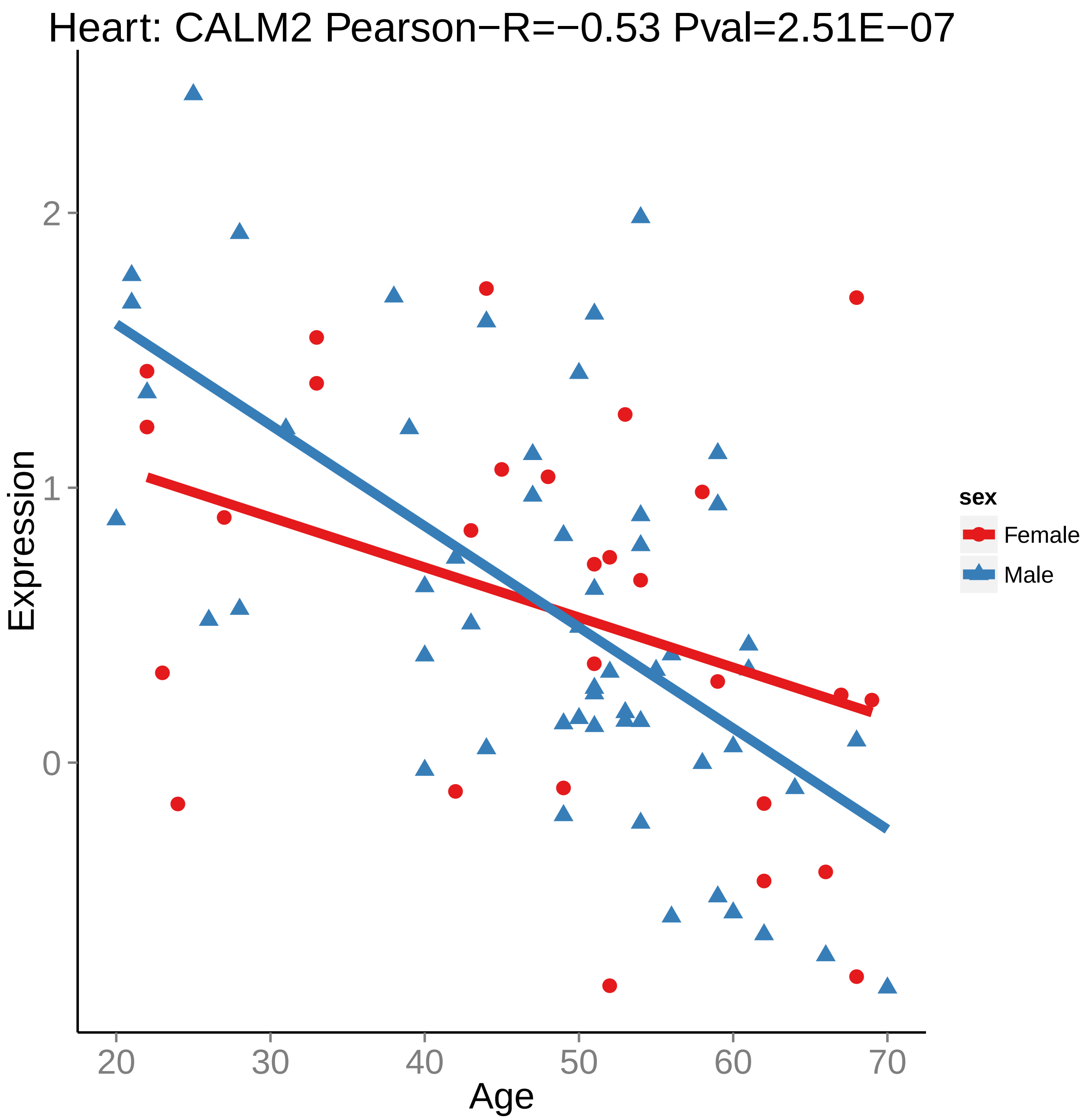

Heart: CALM2 Pearson−R=−0.53 Pval=2.51E−07
Expression
2
1
0
20
30
40
50
60
70
Age
sex
Female
Male

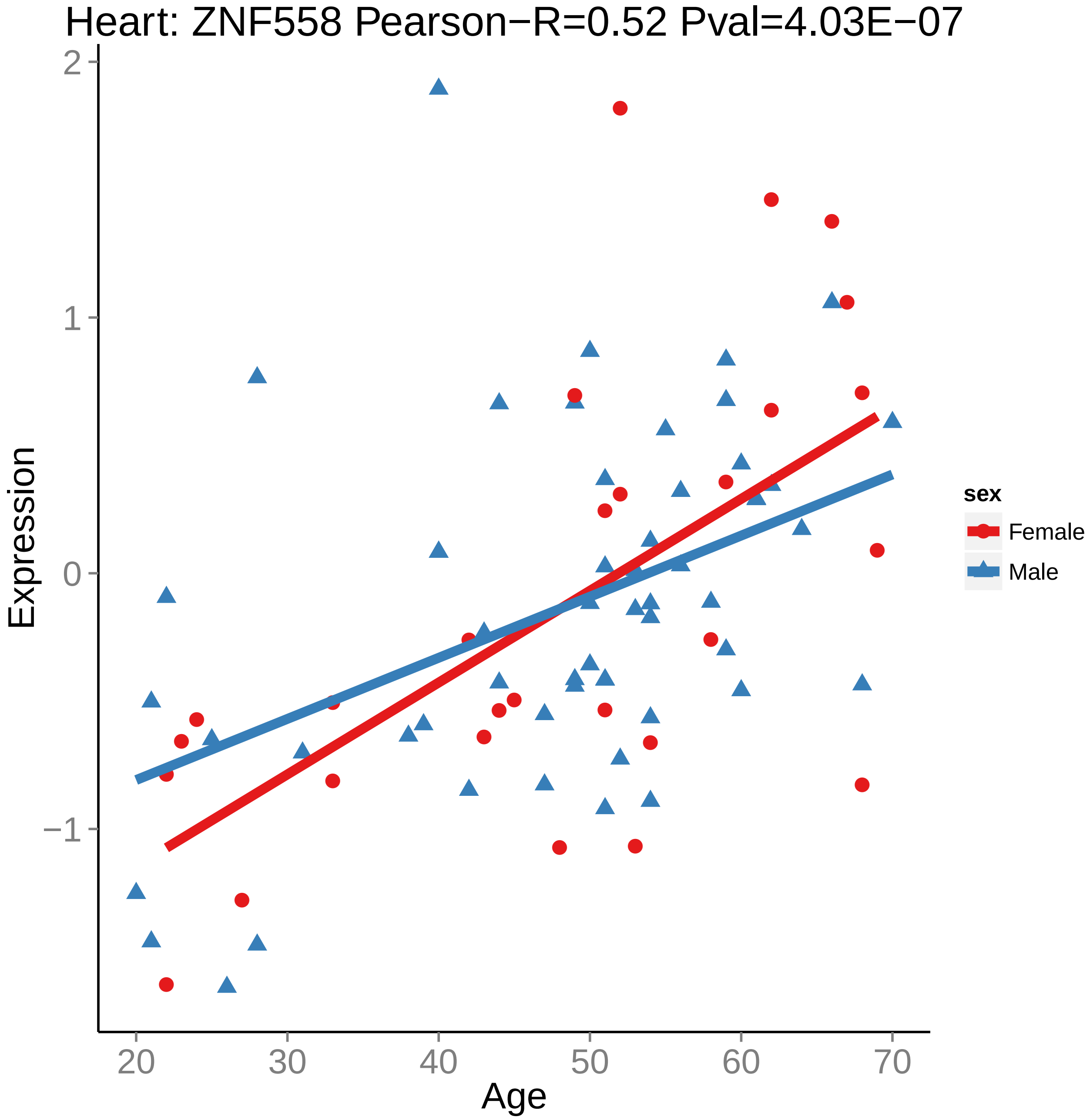

Heart: ZNF558 Pearson−R=0.52 Pval=4.03E−07
Expression
Age
2
1
0
−1
20
30
40
50
60
70
sex
Female
Male

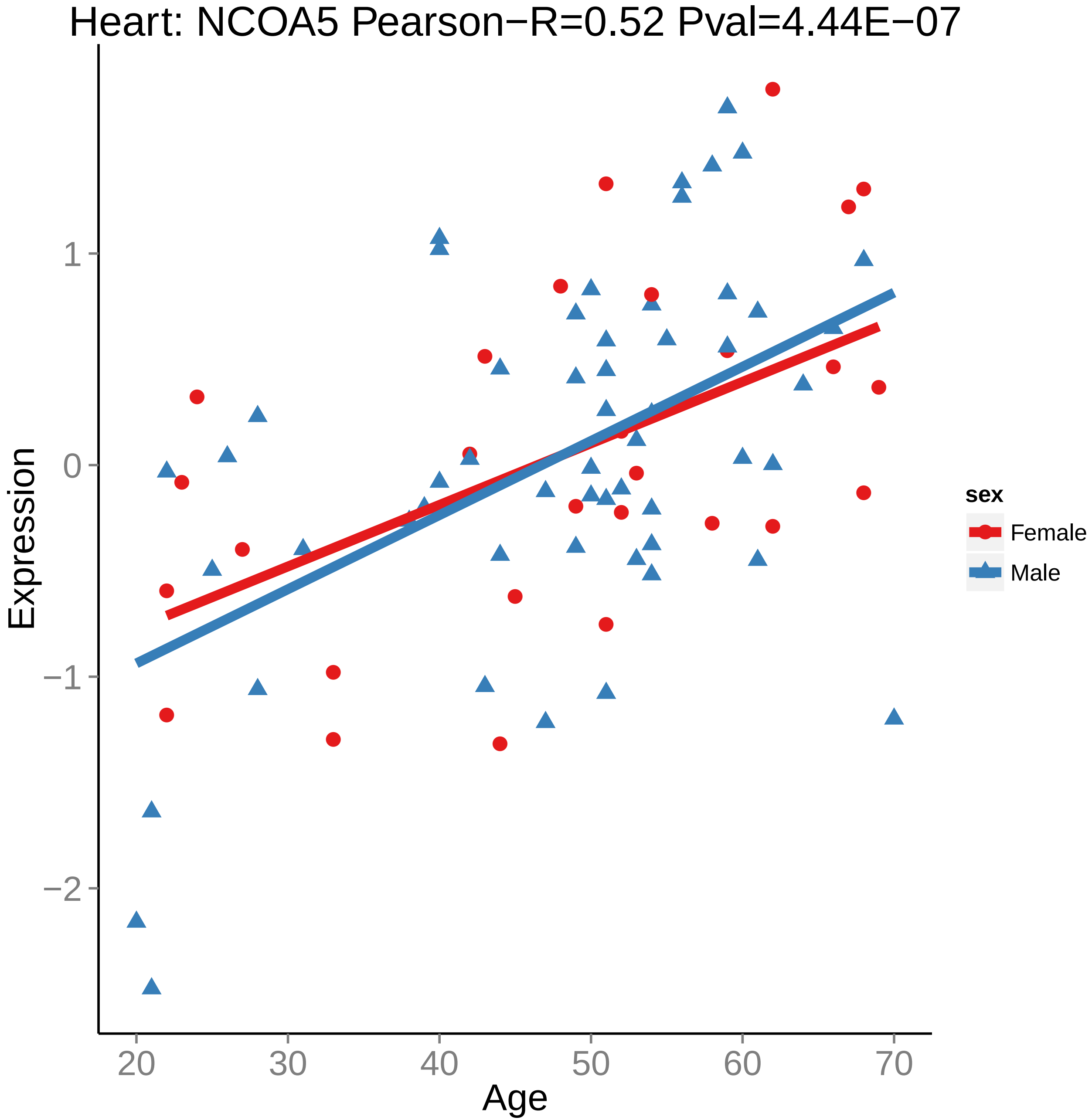

Heart: NCOA5 Pearson−R=0.52 Pval=4.44E−07
Expression
Age
1
0
−1
−2
20
30
40
50
60
70
sex
Female
Male

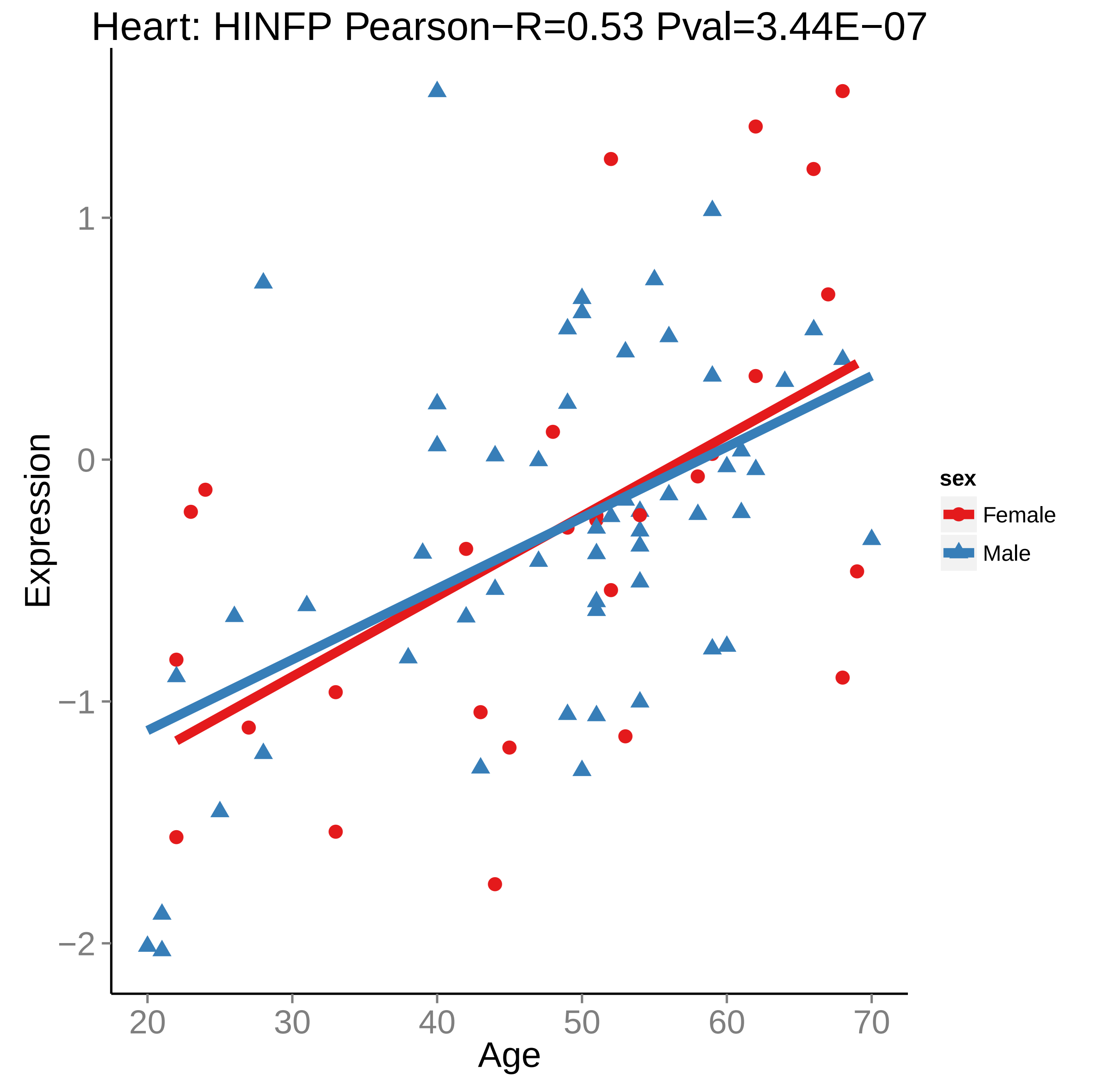

Heart: HINFP Pearson−R=0.53 Pval=3.44E−07
Expression
1
0
−1
−2
20
30
40
50
60
70
Age
sex
Female
Male

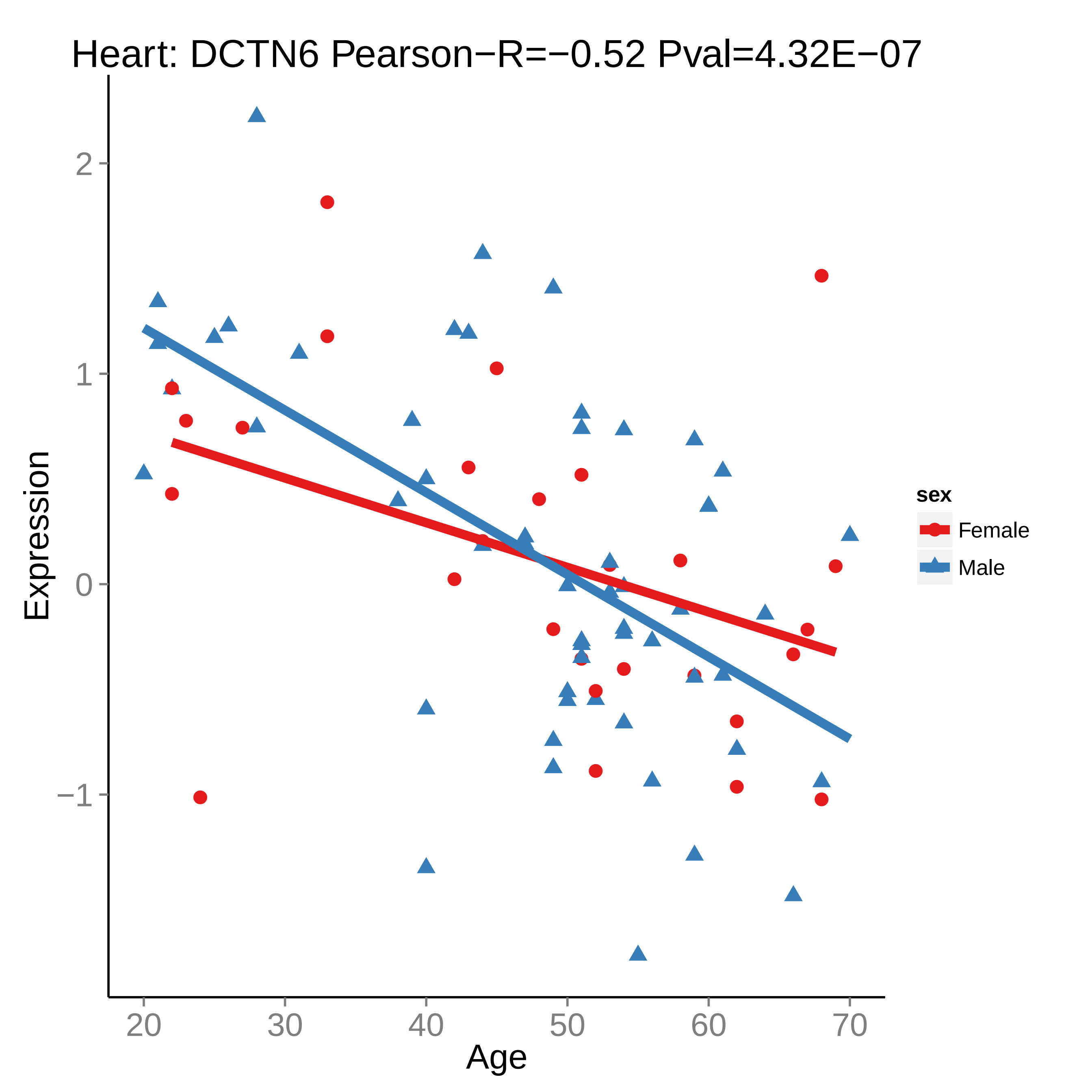
Heart: DCTN6 Pearson−R=−0.52 Pval=4.32E−07
Expression
2
1
0
−1
20
30
40
50
60
70
Age
sex
Female
Male

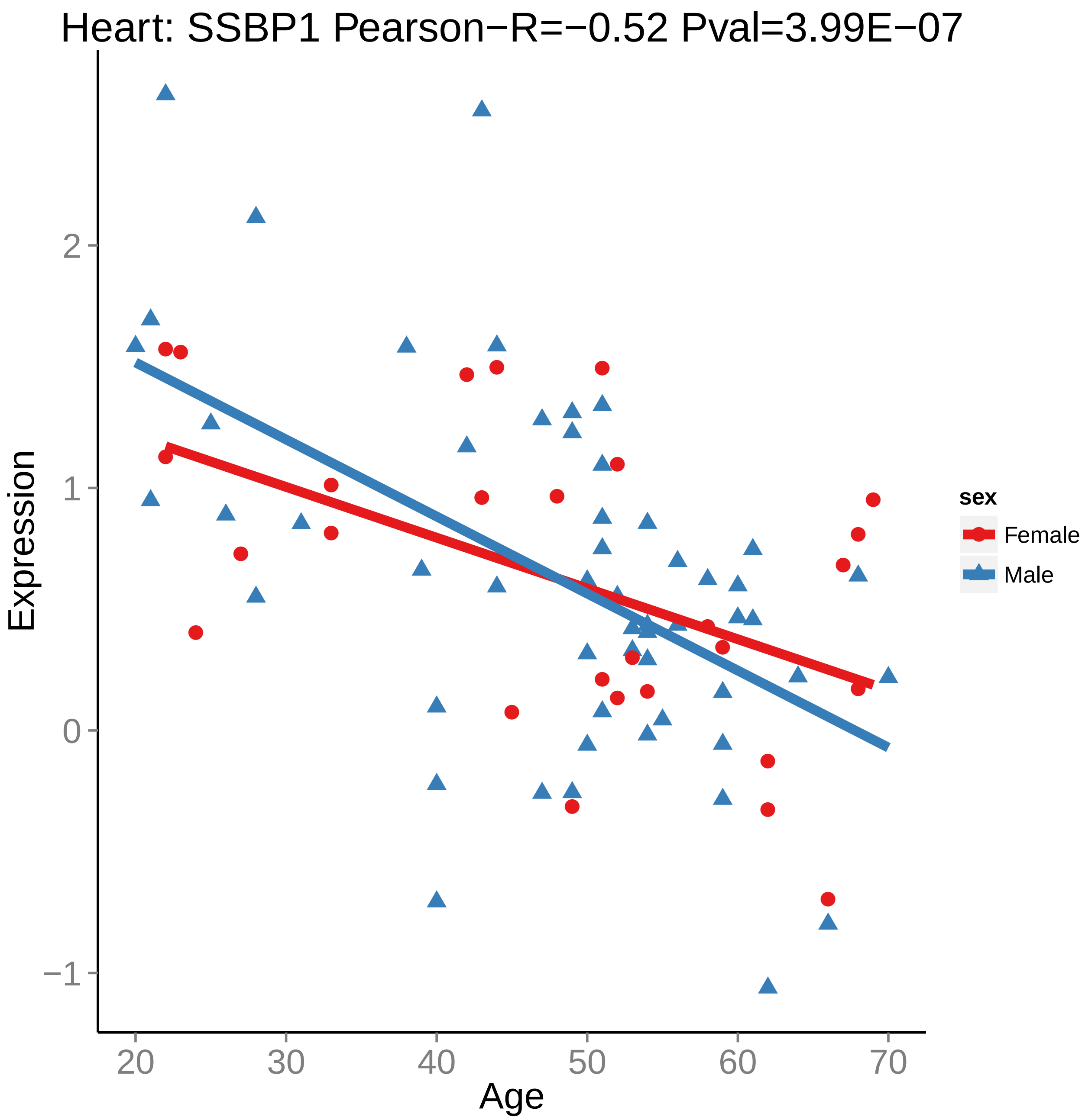

Heart: SSBP1 Pearson−R=−0.52 Pval=3.99E−07
Expression
2
1
0
−1
20
30
40
50
60
70
Age
sex
Female
Male

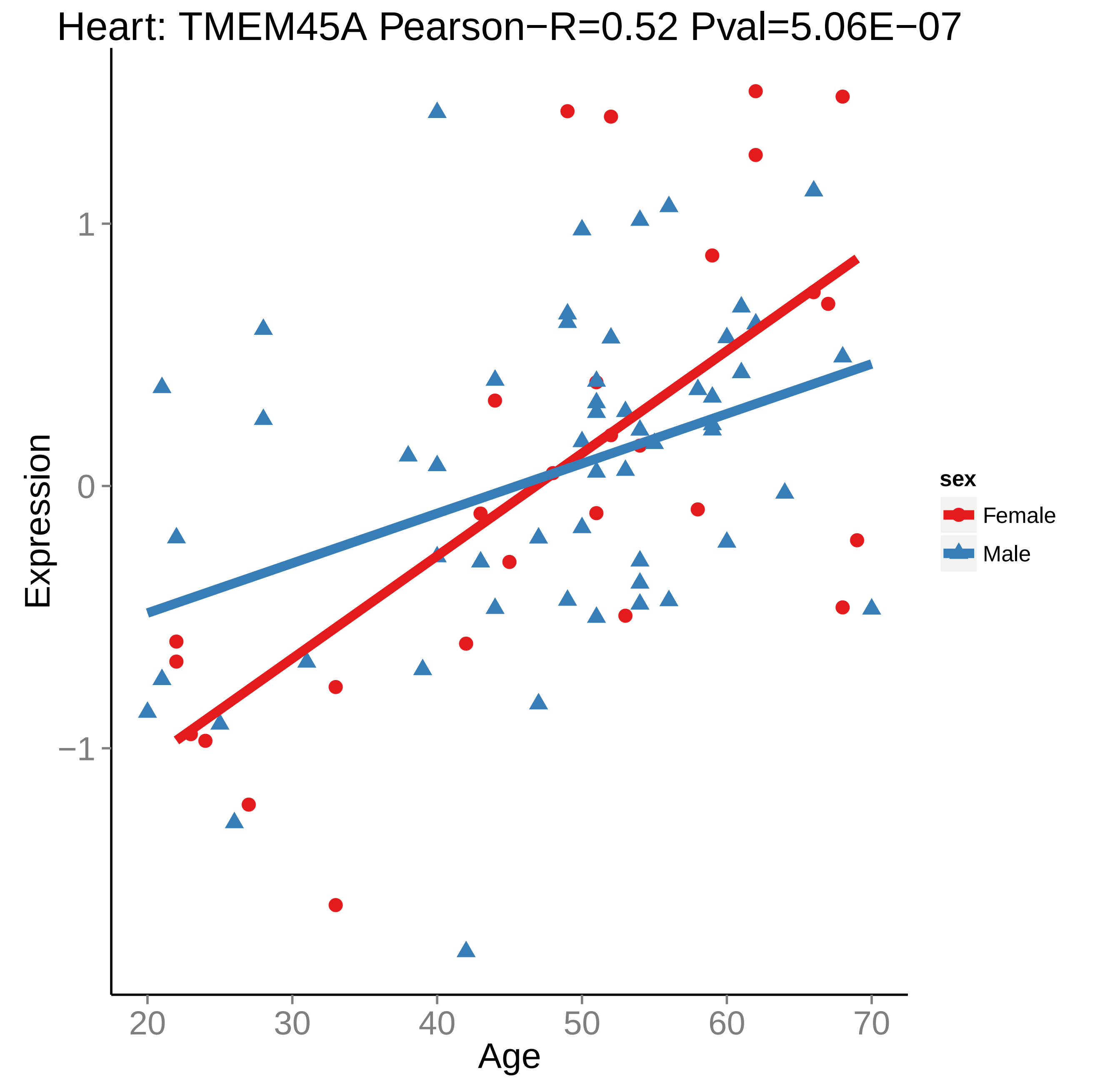

Heart: TMEM45A Pearson−R=0.52 Pval=5.06E−07
Expression
1
0
−1
20
30
40
50
60
70
Age
sex
Female
Male

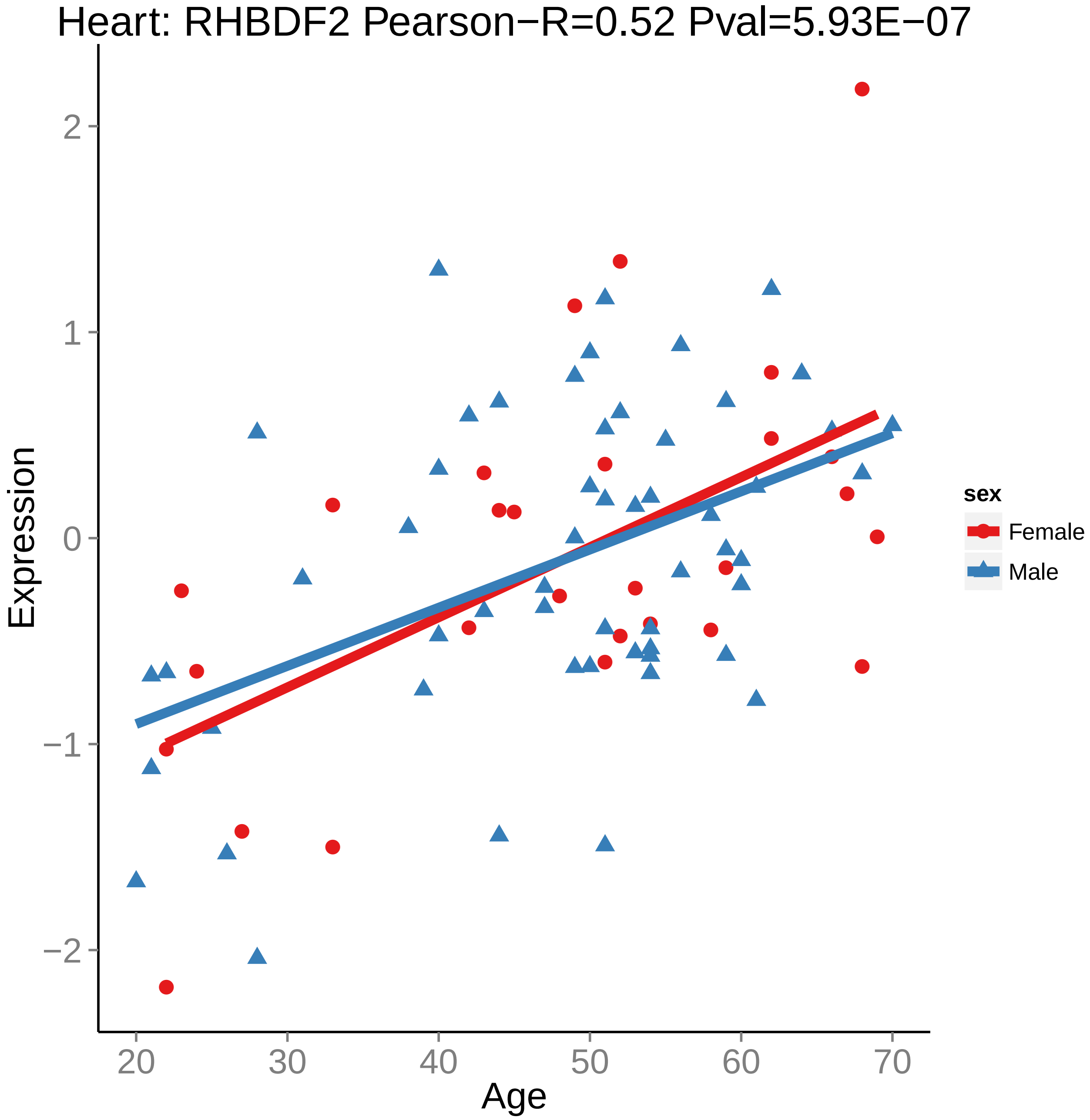
Heart: RHBDF2 Pearson−R=0.52 Pval=5.93E−07
Expression
2
1
0
−1
−2
20
30
40
50
60
70
Age
sex
Female
Male

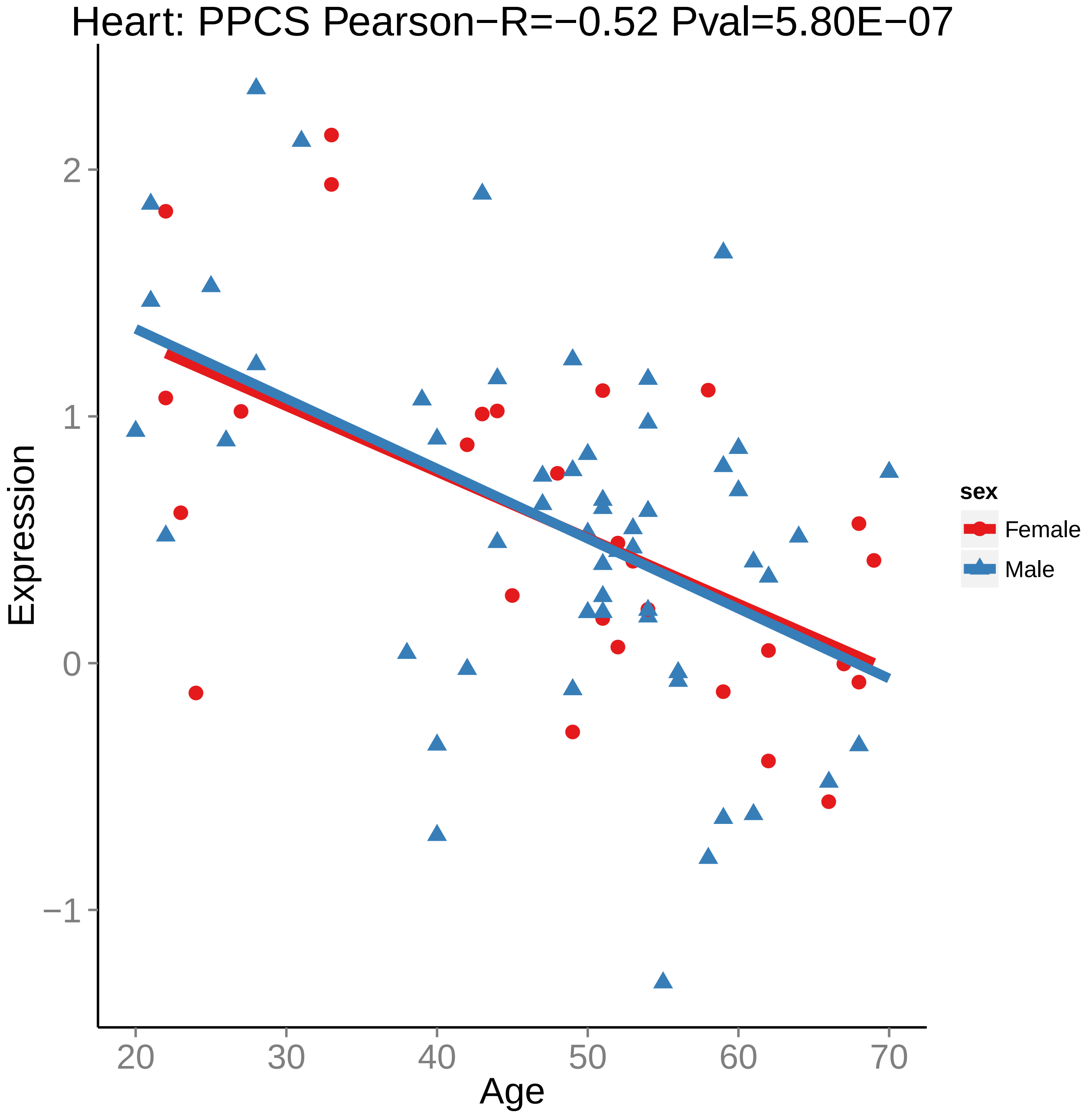

Heart: PPCS Pearson−R=−0.52 Pval=5.80E−07
Expression
2
1
0
−1
20
30
40
50
60
70
Age
sex
Female
Male

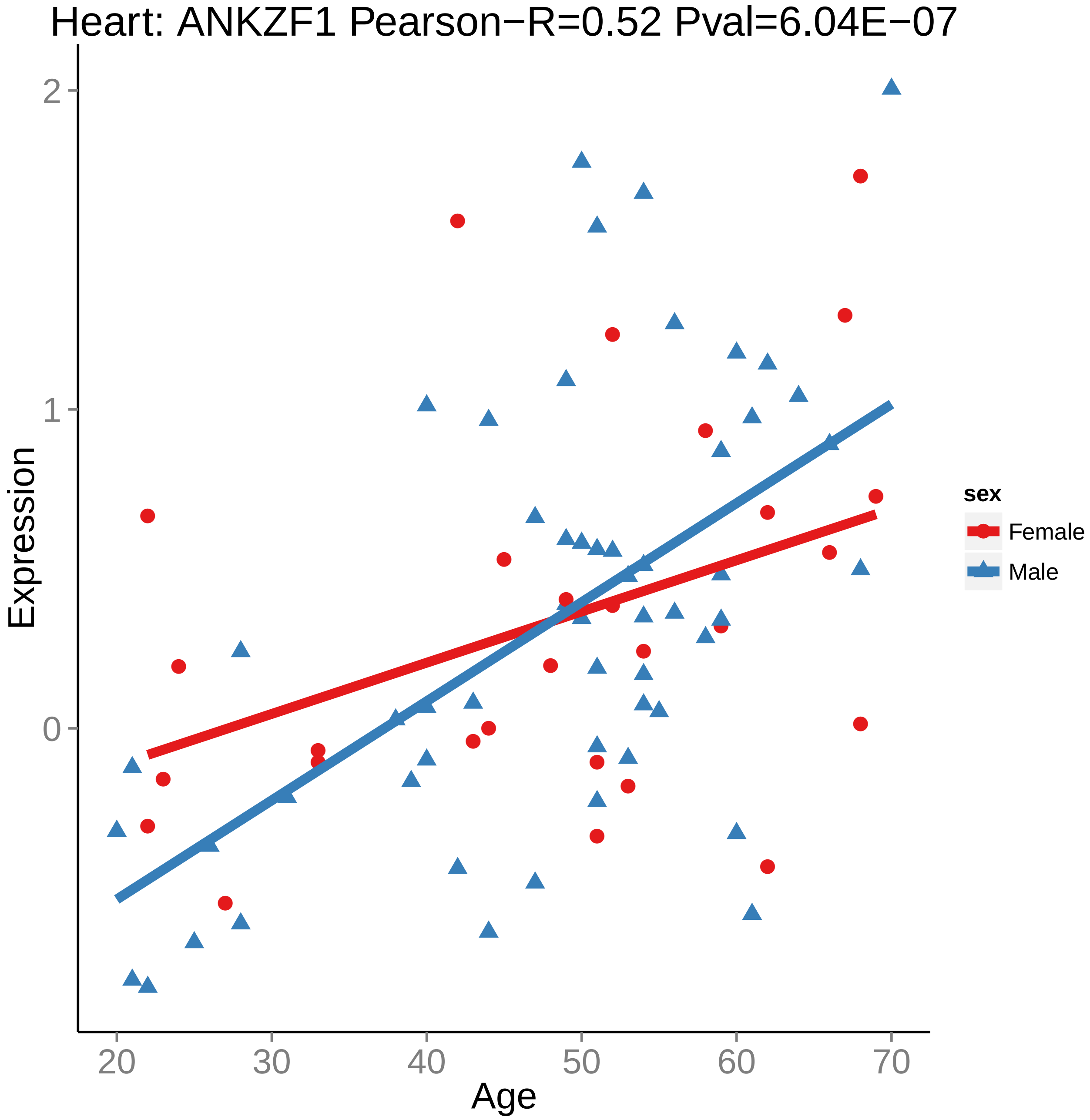

Heart: ANKZF1 Pearson−R=0.52 Pval=6.04E−07
Expression
Age
sex
Female
Male
0
1
2
20
30
40
50
60
70

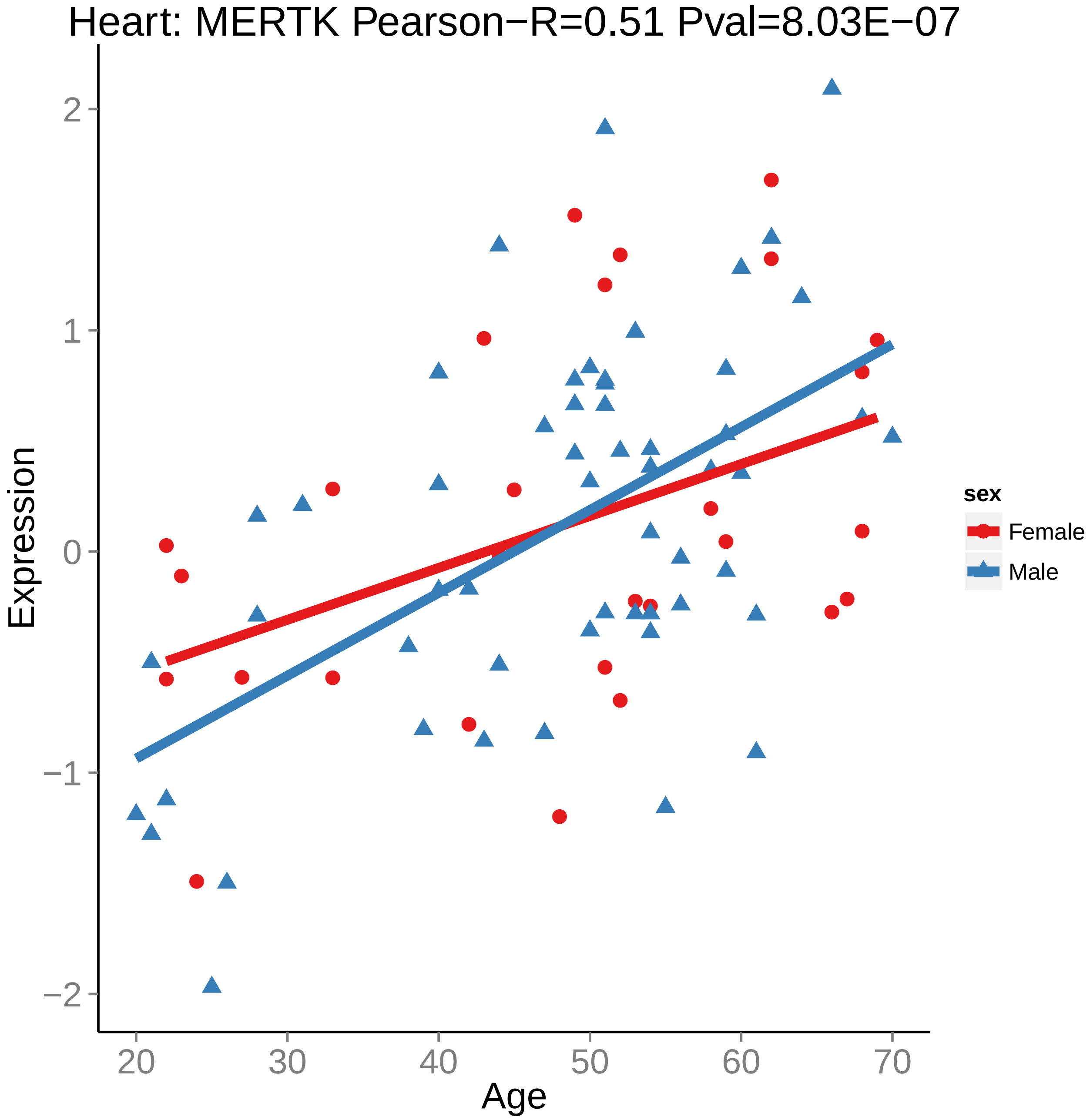

Heart: MERTK Pearson−R=0.51 Pval=8.03E−07
Expression
2
1
0
−1
−2
20
30
40
50
60
70
Age
sex
Female
Male

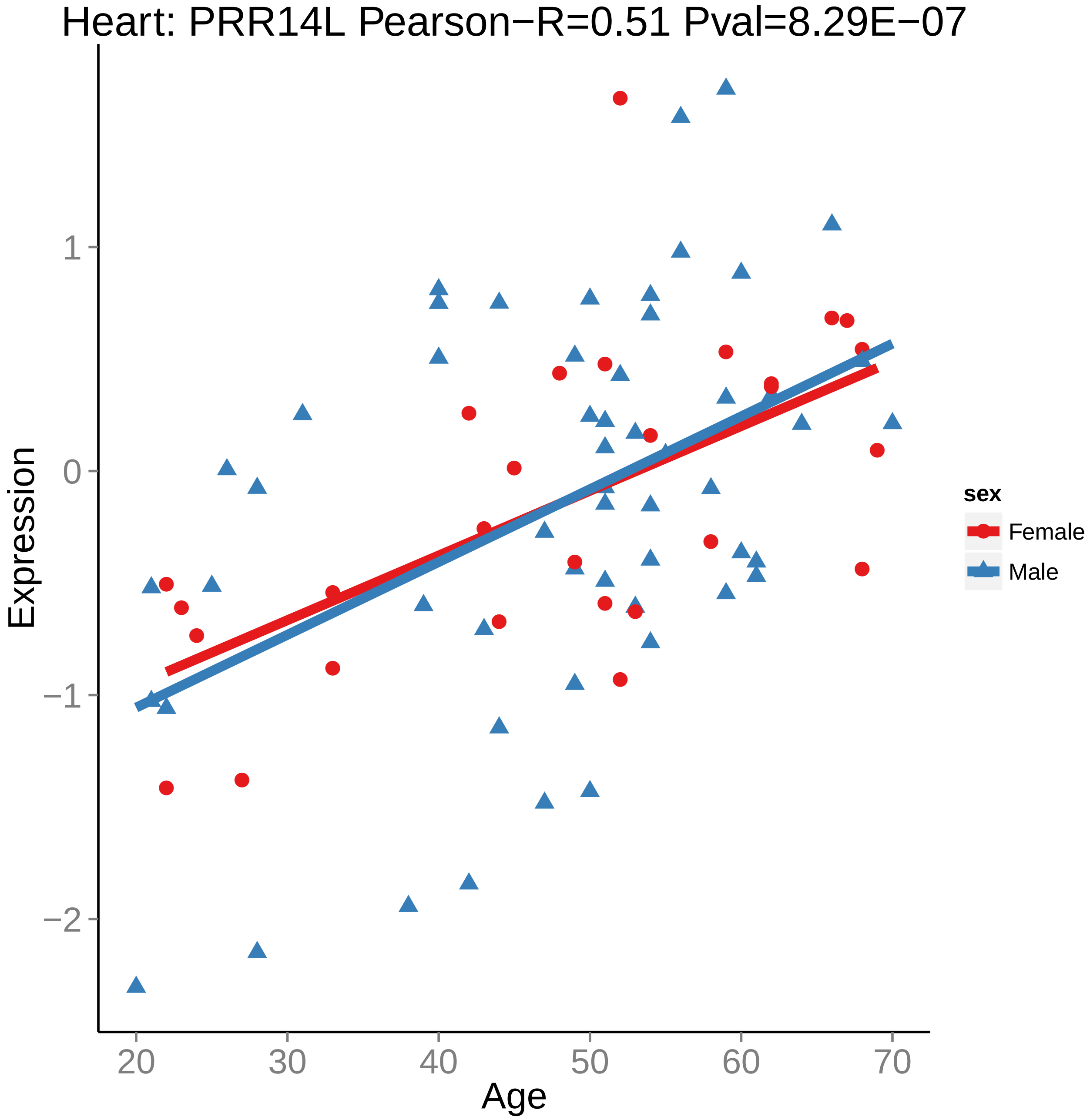

Heart: PRR14L Pearson−R=0.51 Pval=8.29E−07
Expression
Age
1
0
−1
−2
20
30
40
50
60
70
sex
Female
Male

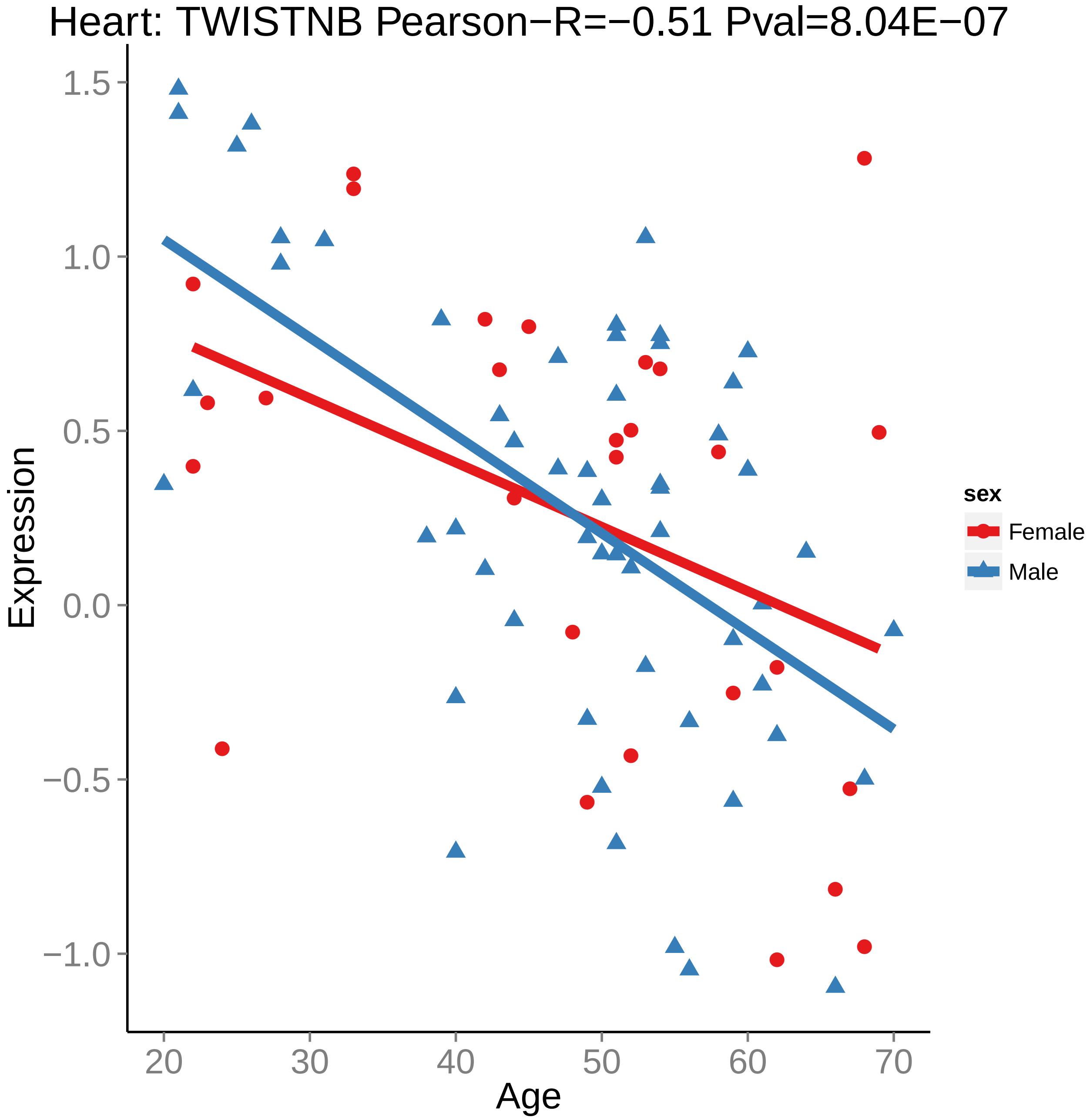
Heart: TWISTNB Pearson−R=−0.51 Pval=8.04E−07
Expression
Age
1.5
1.0
0.5
0.0
−0.5
−1.0
20
30
40
50
60
70
sex
Female
Male

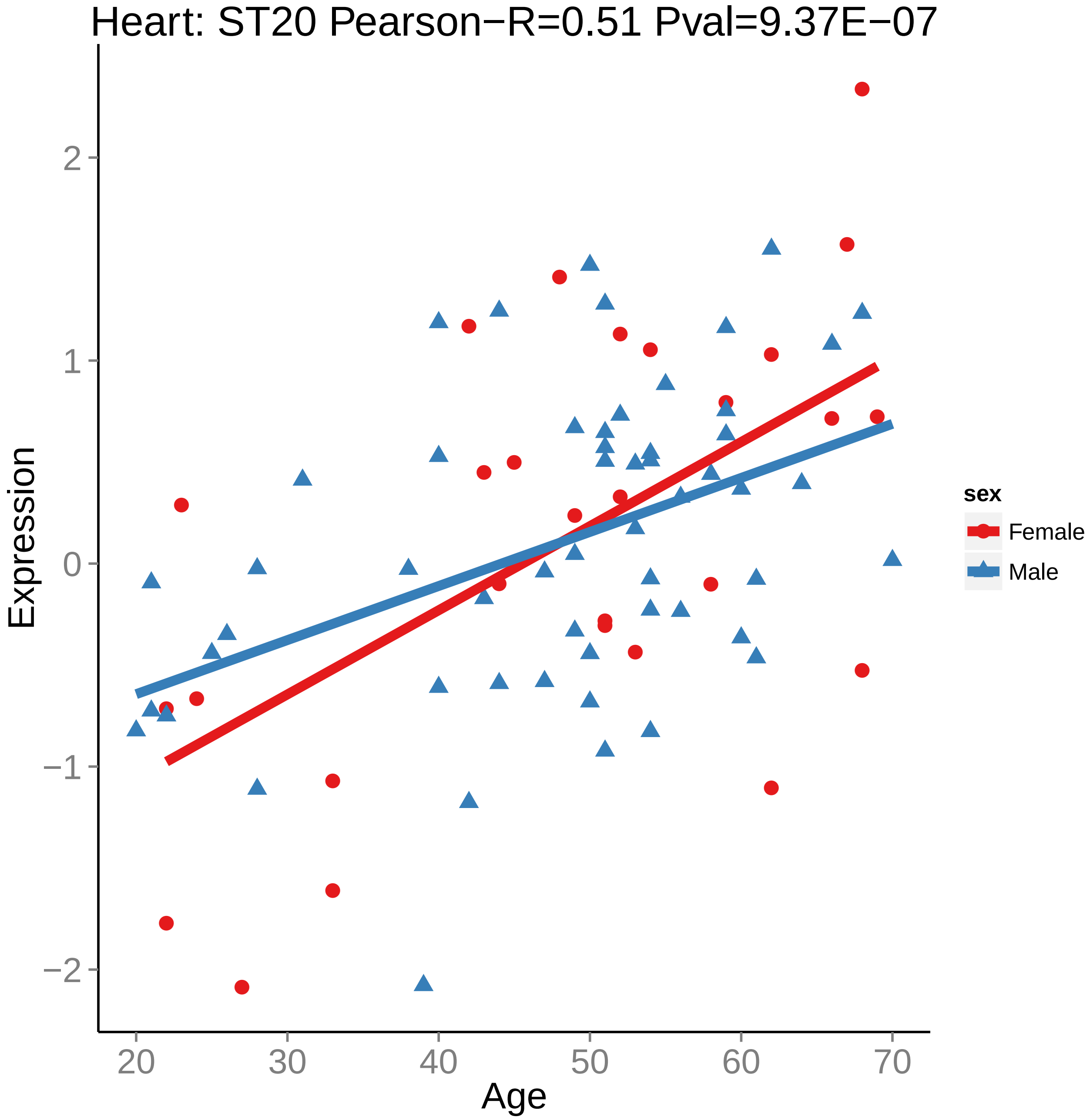

Heart: ST20 Pearson−R=0.51 Pval=9.37E−07
Expression
Age
2
1
0
−1
−2
20
30
40
50
60
70
sex
Female
Male

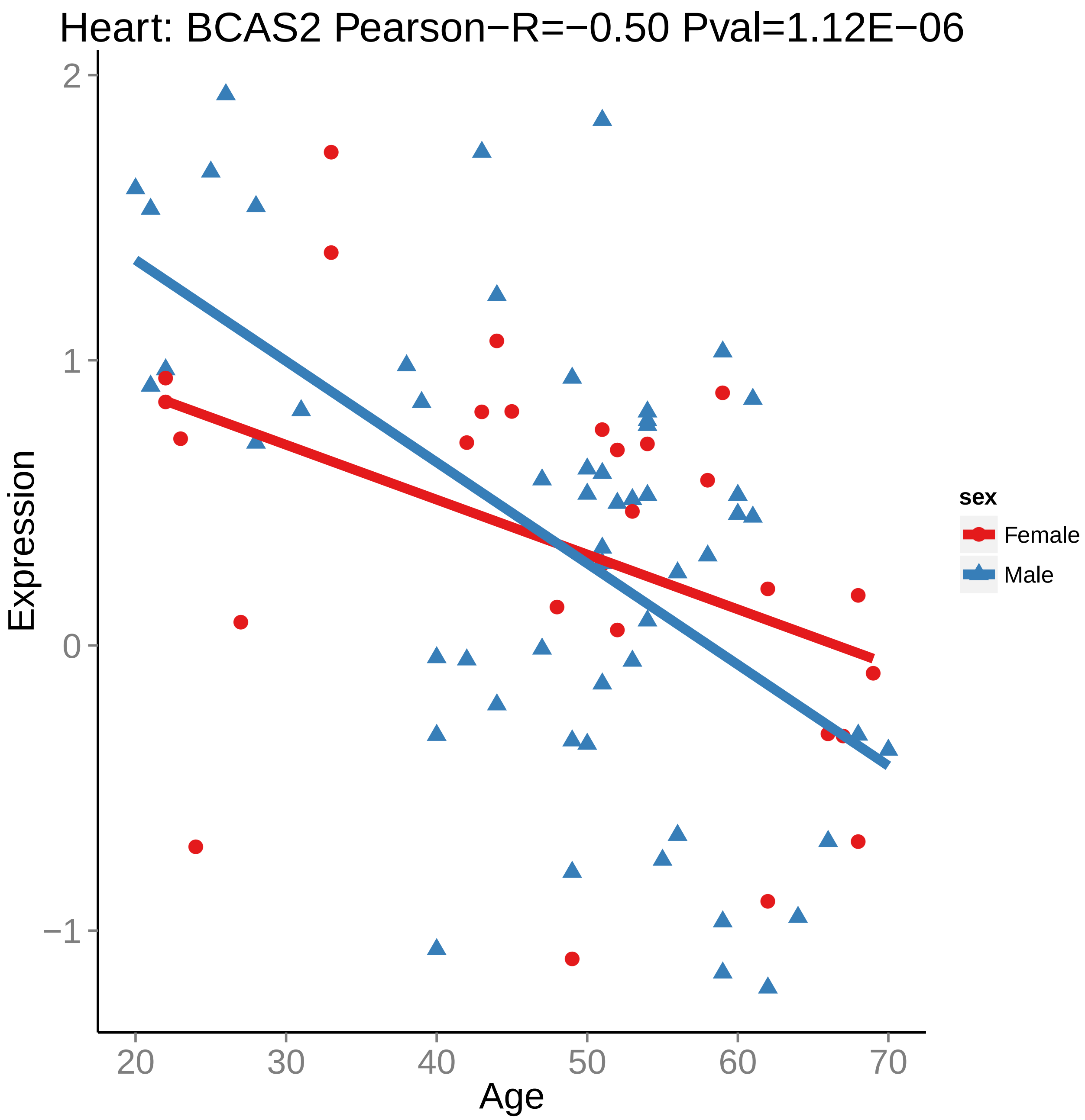

Heart: BCAS2 Pearson−R=−0.50 Pval=1.12E−06
2
1
0
−1
Expression
20
30
40
50
60
70
Age
sex
Female
Male

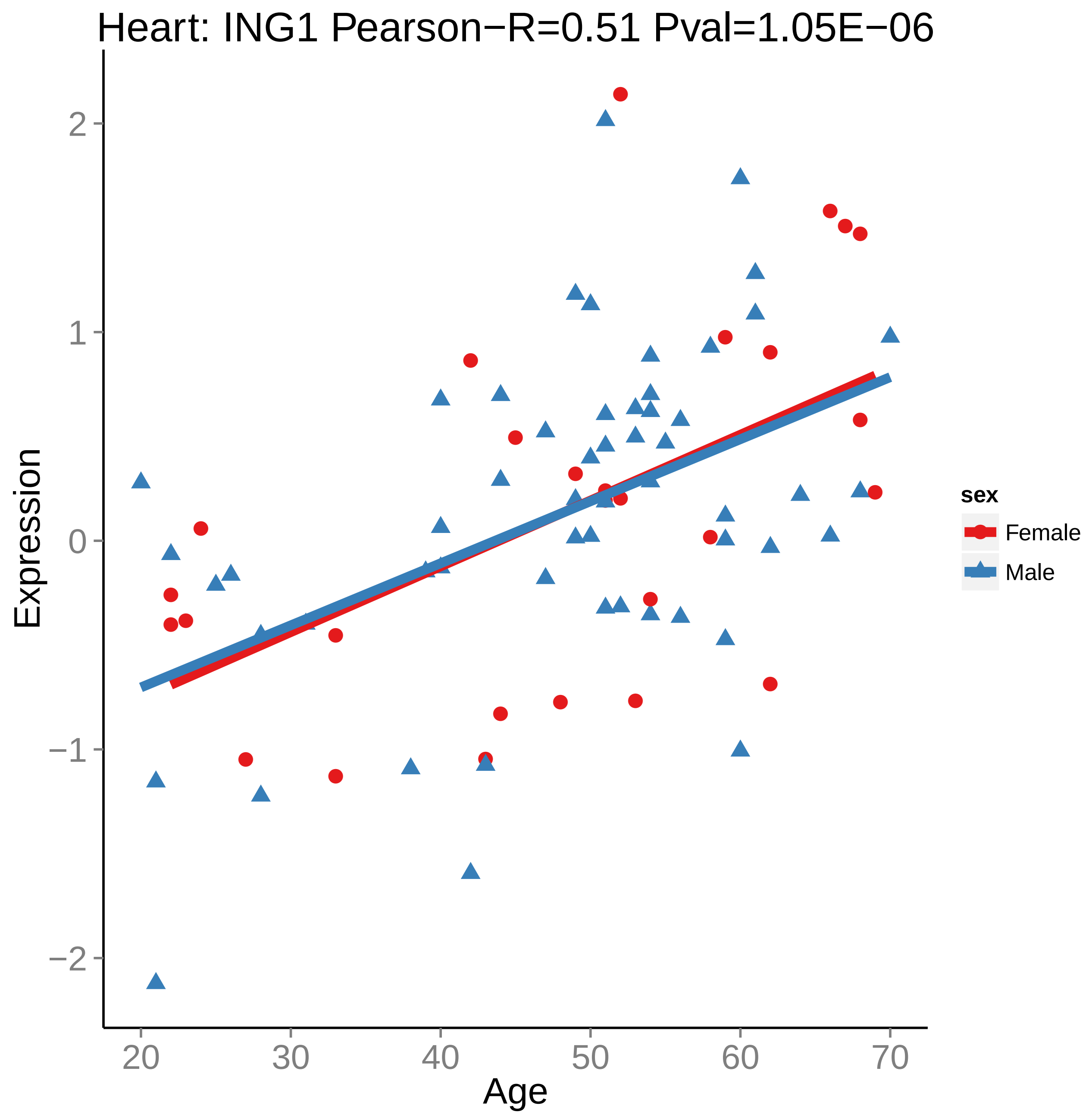

Heart: ING1 Pearson−R=0.51 Pval=1.05E−06
Expression
Age
2
1
0
−1
−2
20
30
40
50
60
70
sex
Female
Male

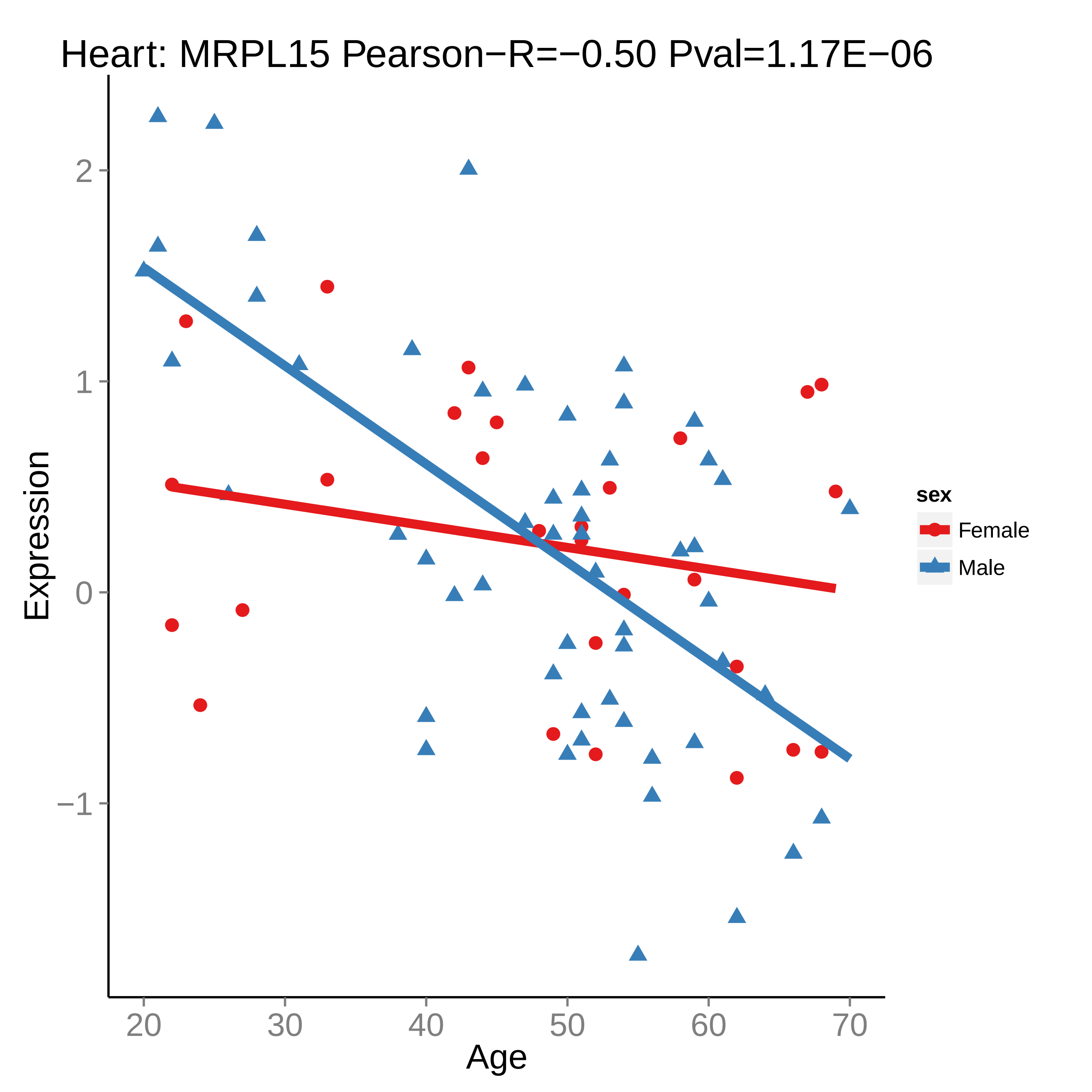
Heart: MRPL15 Pearson−R=−0.50 Pval=1.17E−06
Expression
Age
2
1
0
−1
20
30
40
50
60
70
sex
Female
Male

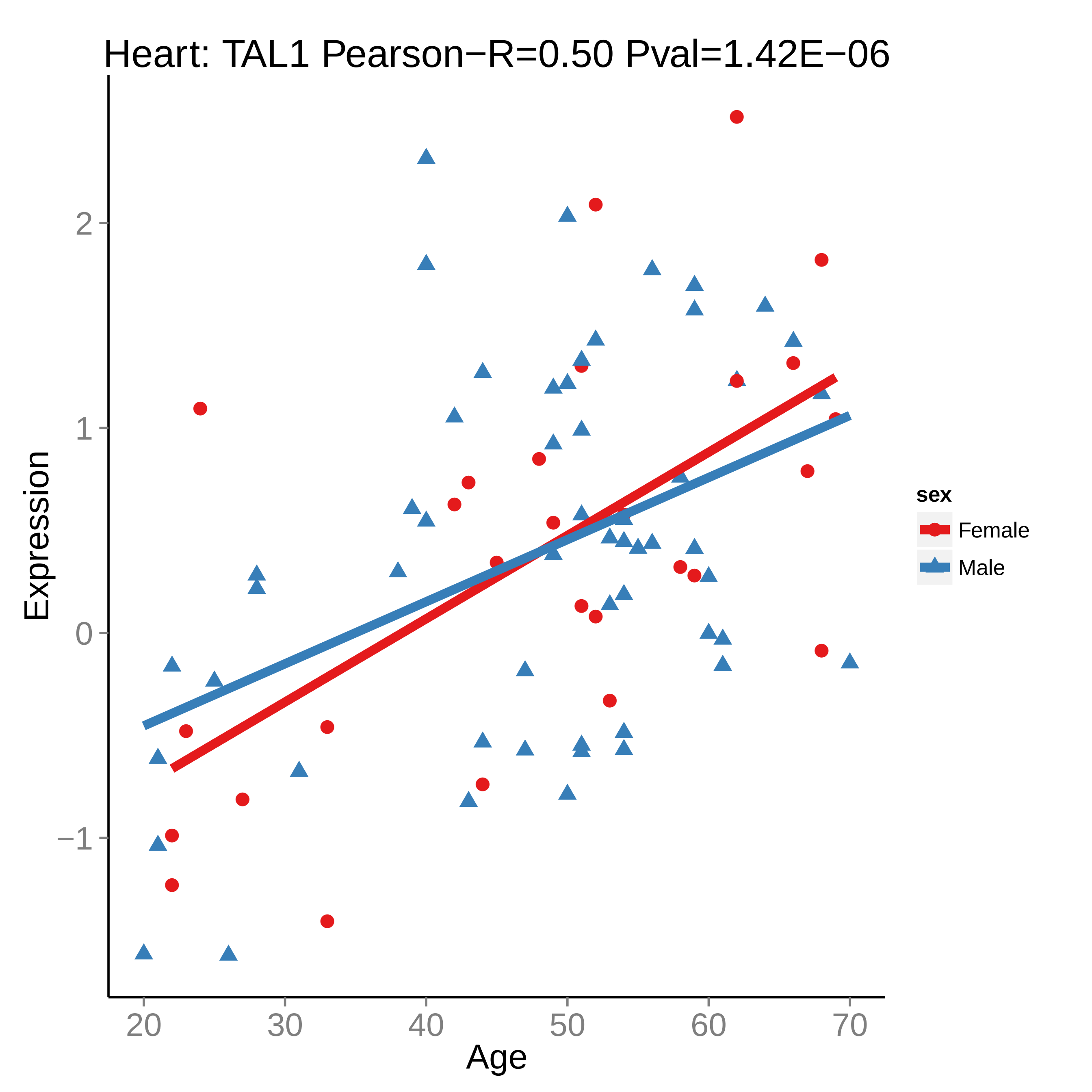

Heart: TAL1 Pearson−R=0.50 Pval=1.42E−06
Expression
Age
sex
Female
Male
2
1
0
−1
20
30
40
50
60
70

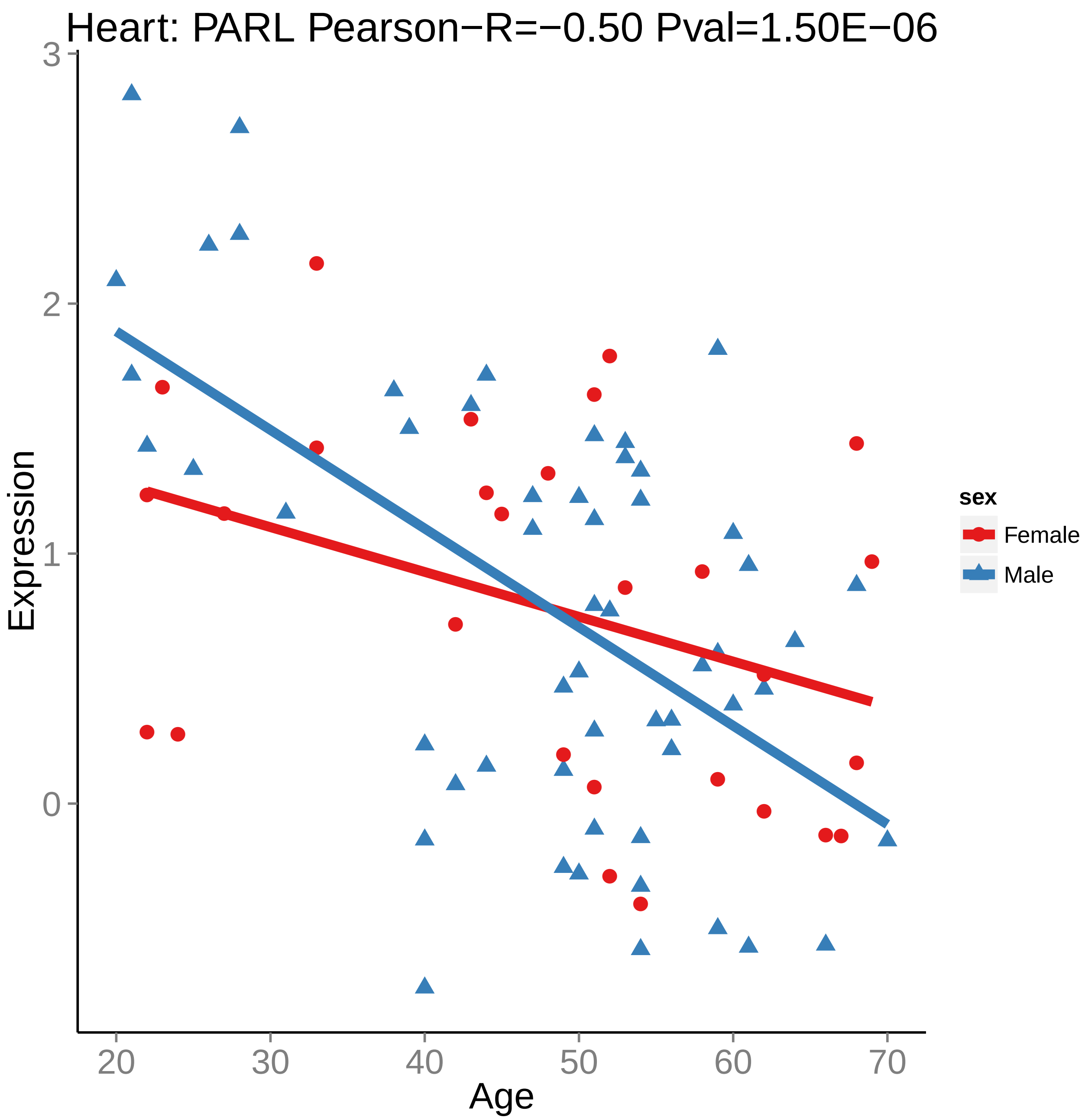

Heart: PARL Pearson−R=−0.50 Pval=1.50E−06
Expression
Age
3
2
1
0
20
30
40
50
60
70
sex
Female
Male

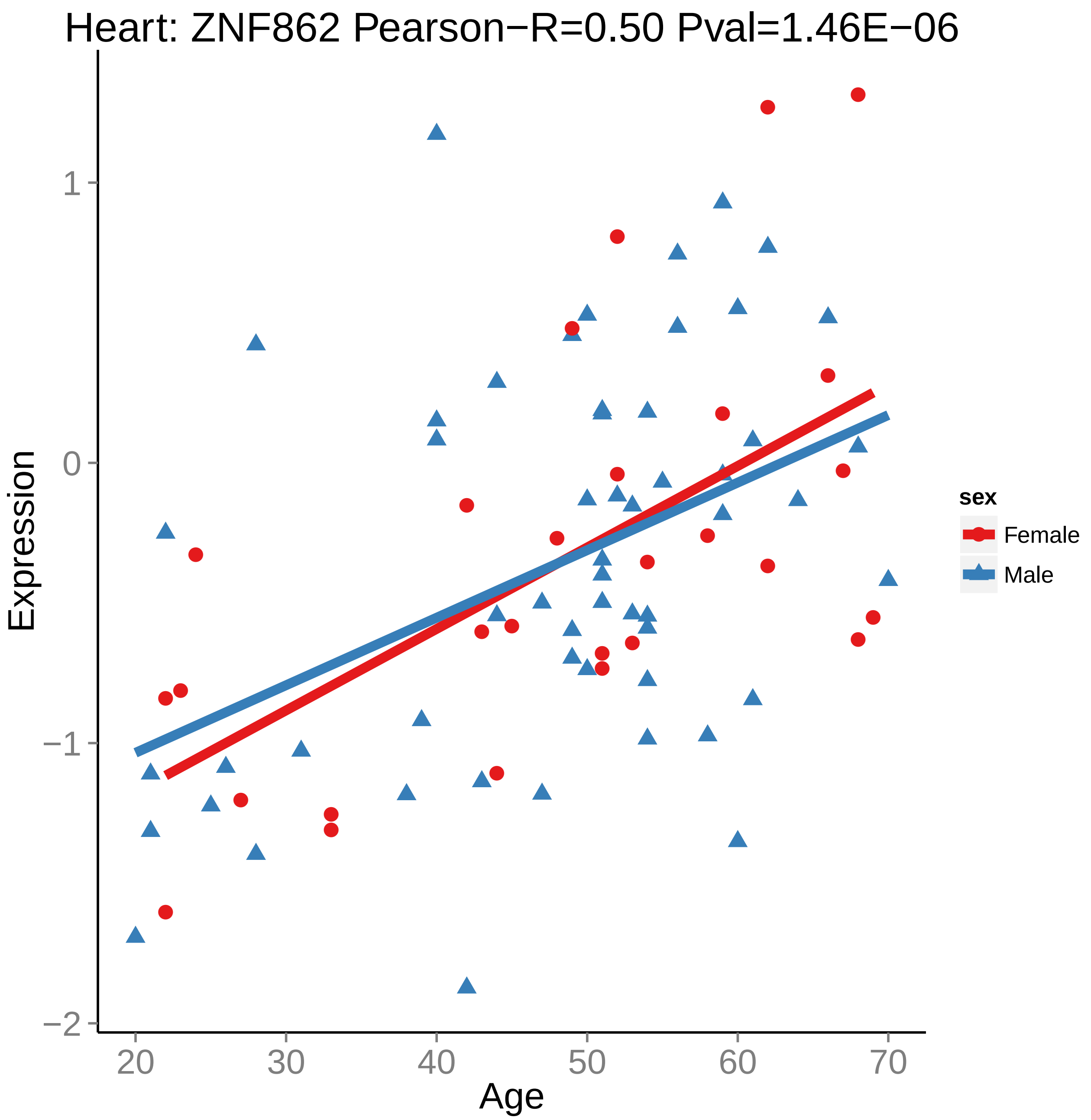
Heart: ZNF862 Pearson−R=0.50 Pval=1.46E−06
Expression
1
0
−1
−2
20
30
40
50
60
70
Age
sex
Female
Male

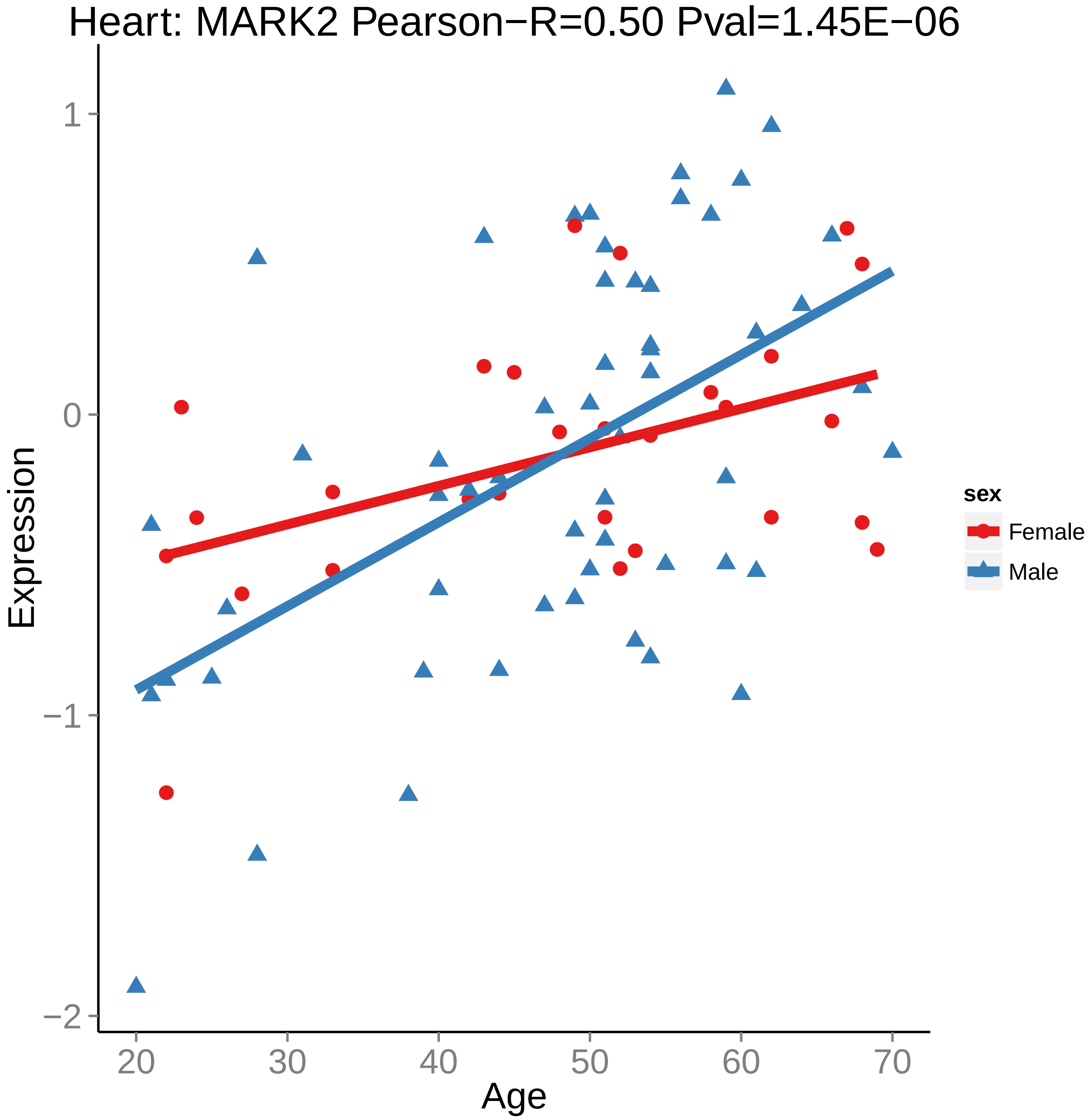

Heart: MARK2 Pearson−R=0.50 Pval=1.45E−06
Expression
Age
1
0
−1
−2
20
30
40
50
60
70
sex
Female
Male

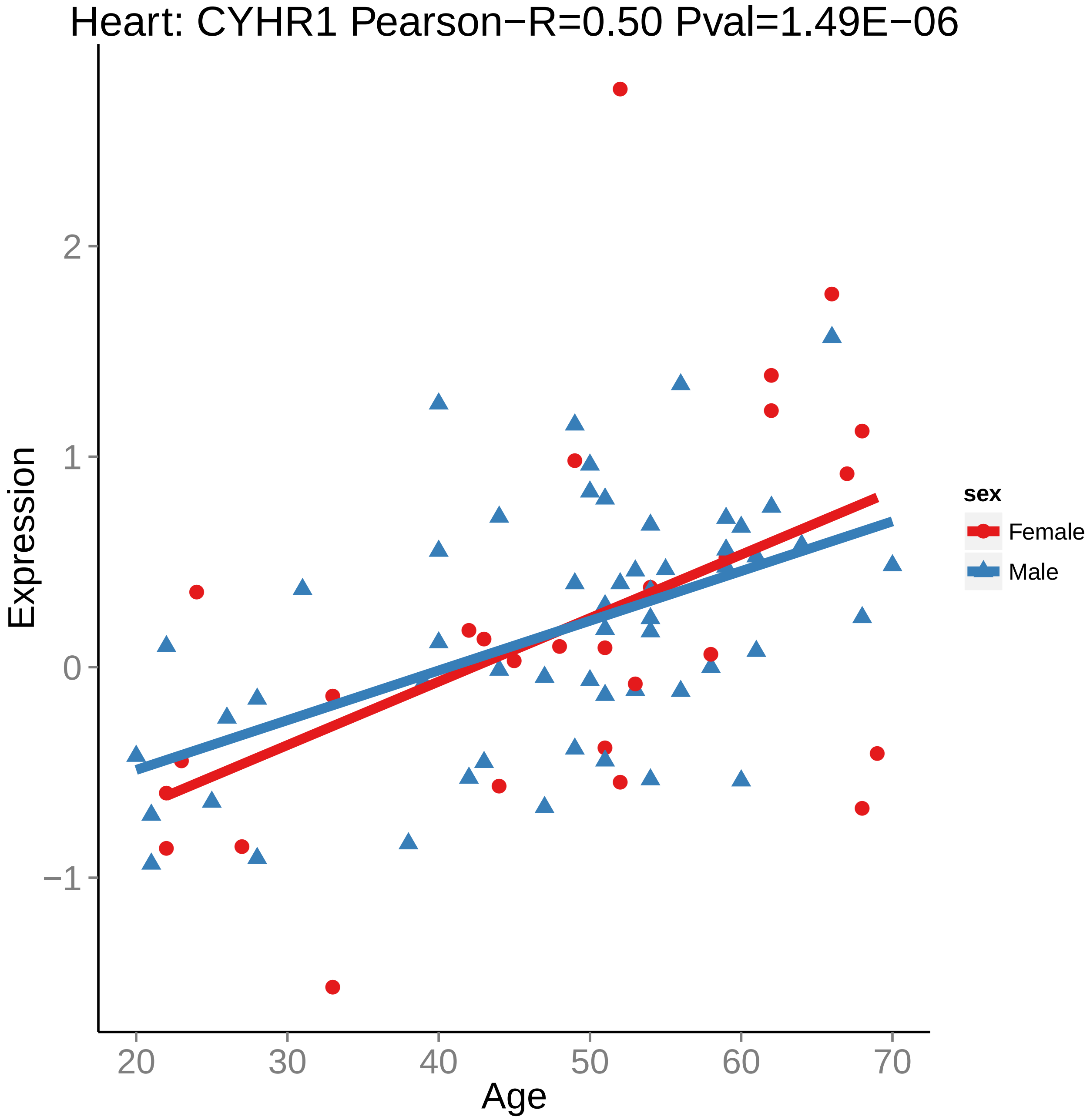
Heart: CYHR1 Pearson−R=0.50 Pval=1.49E−06
Expression
2
1
0
−1
20
30
40
50
60
70
Age
sex
Female
Male

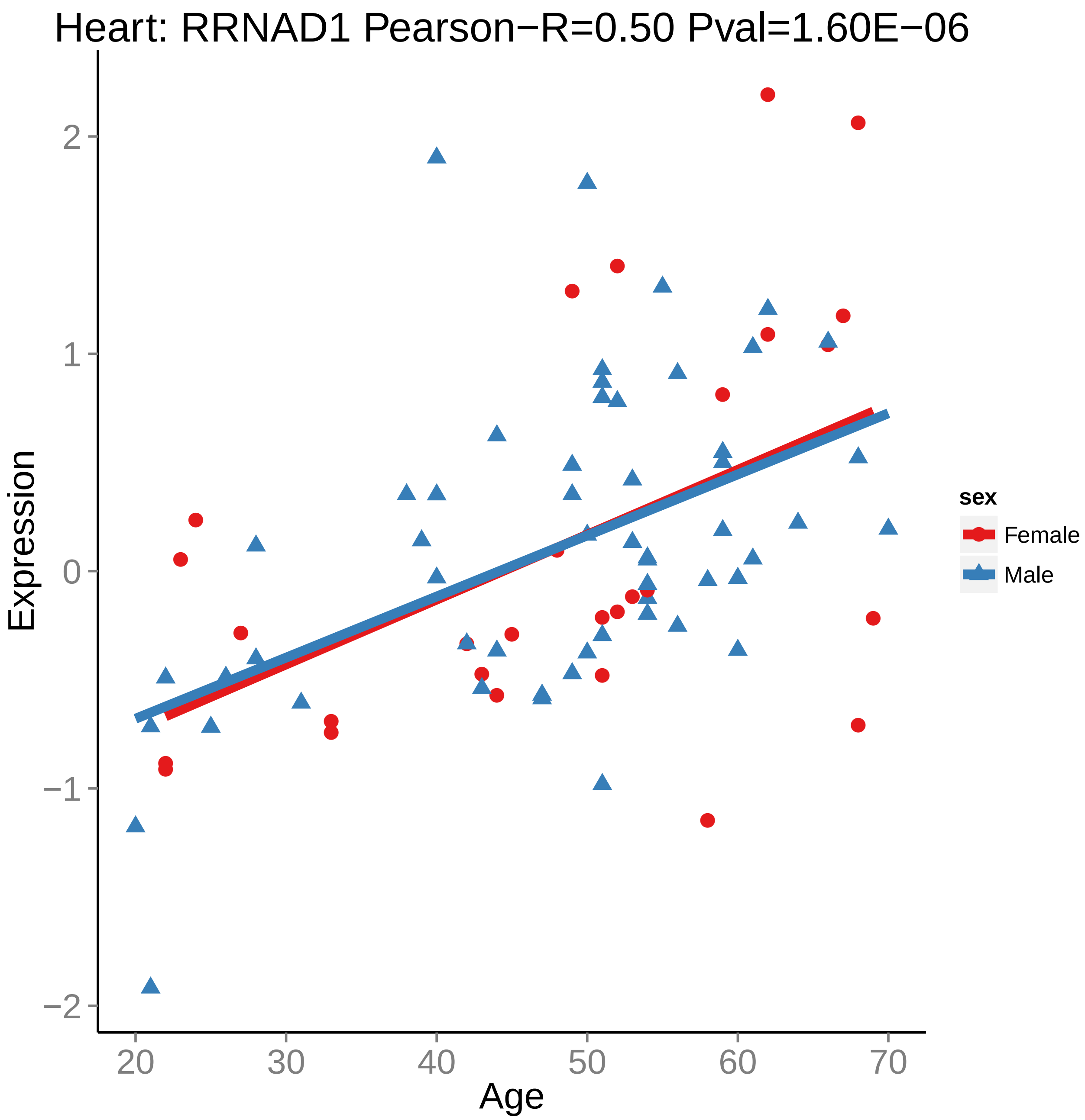

Heart: RRNAD1 Pearson−R=0.50 Pval=1.60E−06
Expression
Age
2
1
0
−1
−2
20
30
40
50
60
70
sex
Female
Male

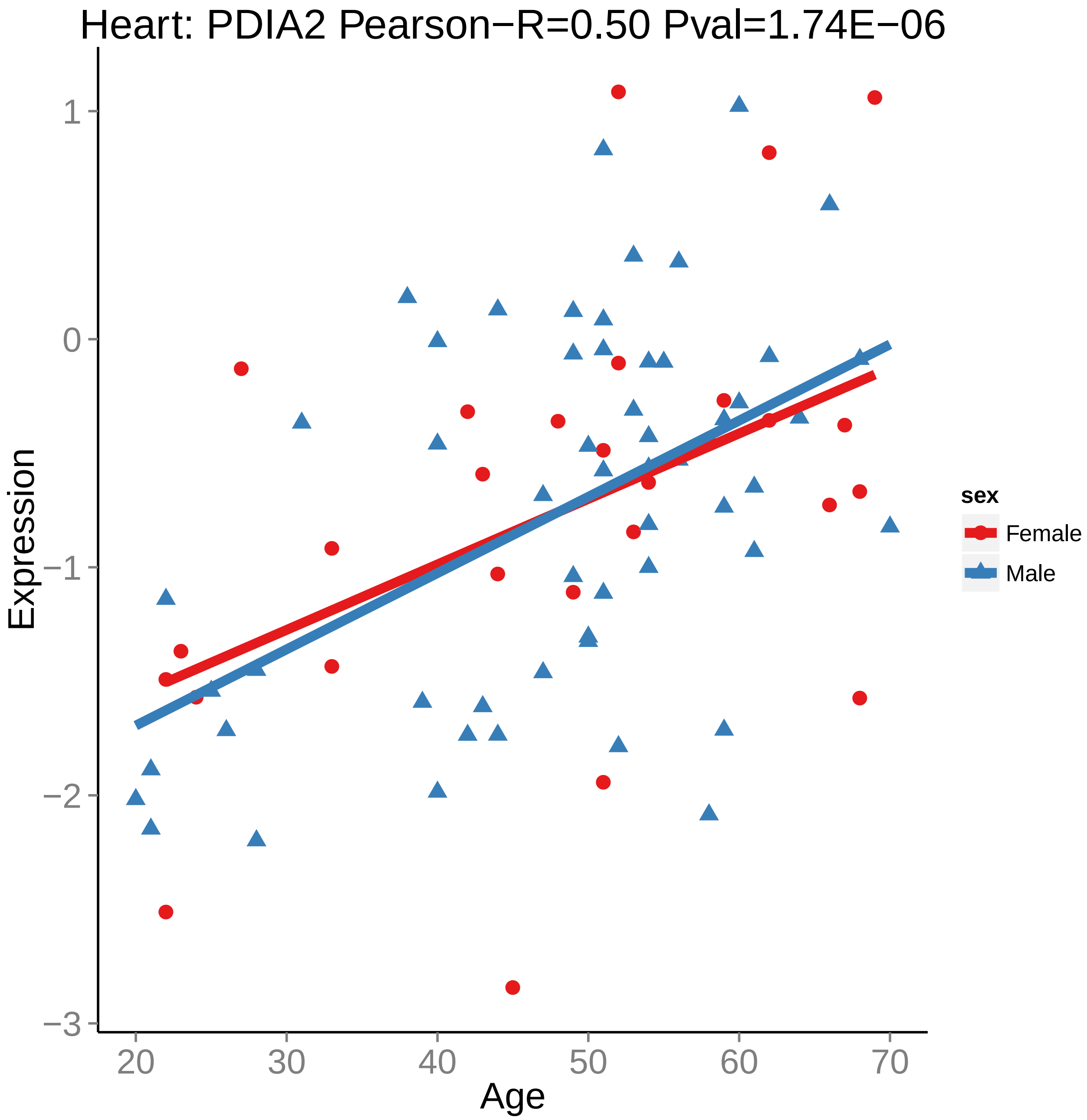
Heart: PDIA2 Pearson−R=0.50 Pval=1.74E−06
Expression
1
0
−1
−2
−3
20
30
40
50
60
70
Age
sex
Female
Male

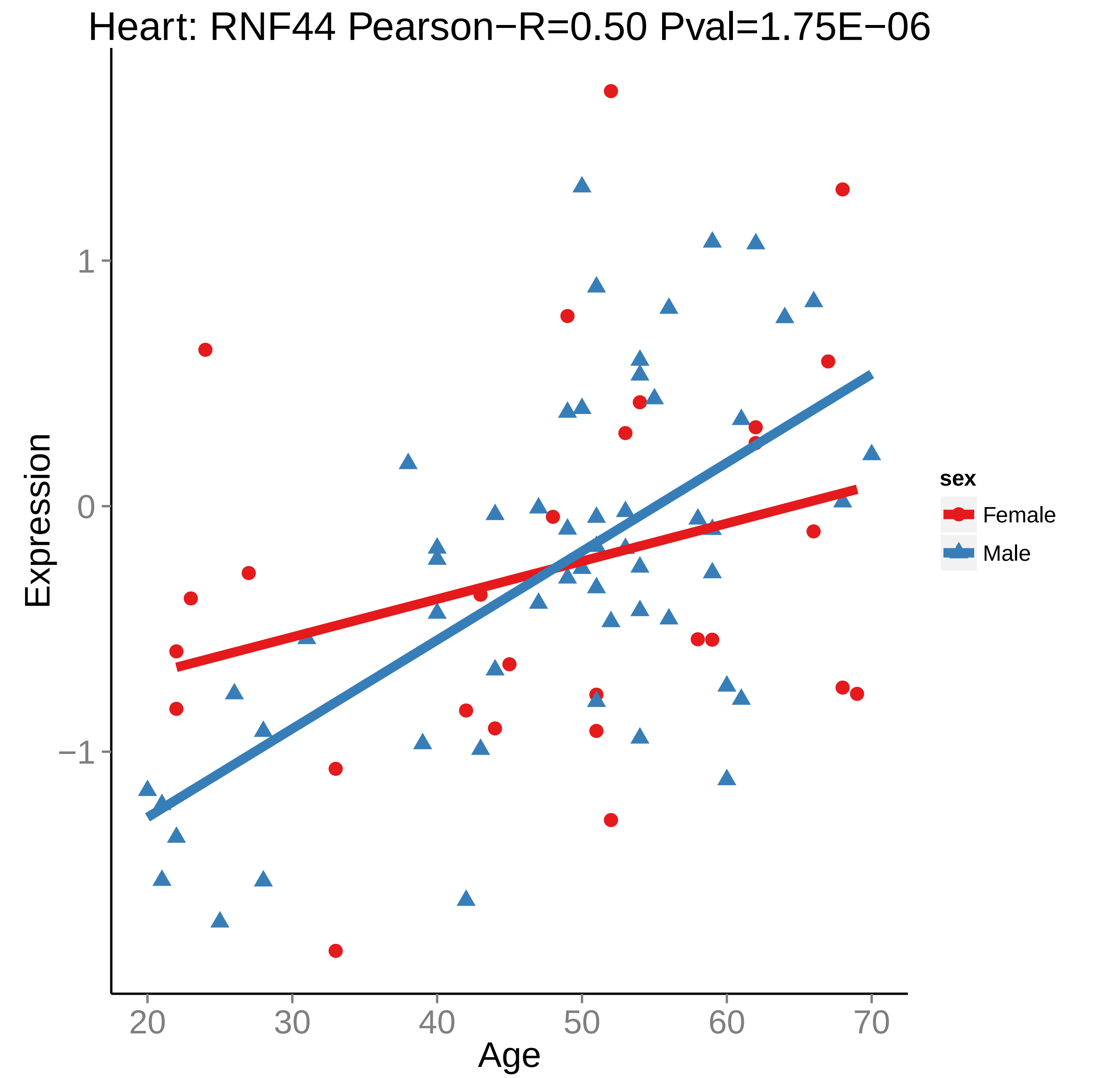

Heart: RNF44 Pearson−R=0.50 Pval=1.75E−06
Expression
1
0
−1
20
30
40
50
60
70
Age
sex
Female
Male

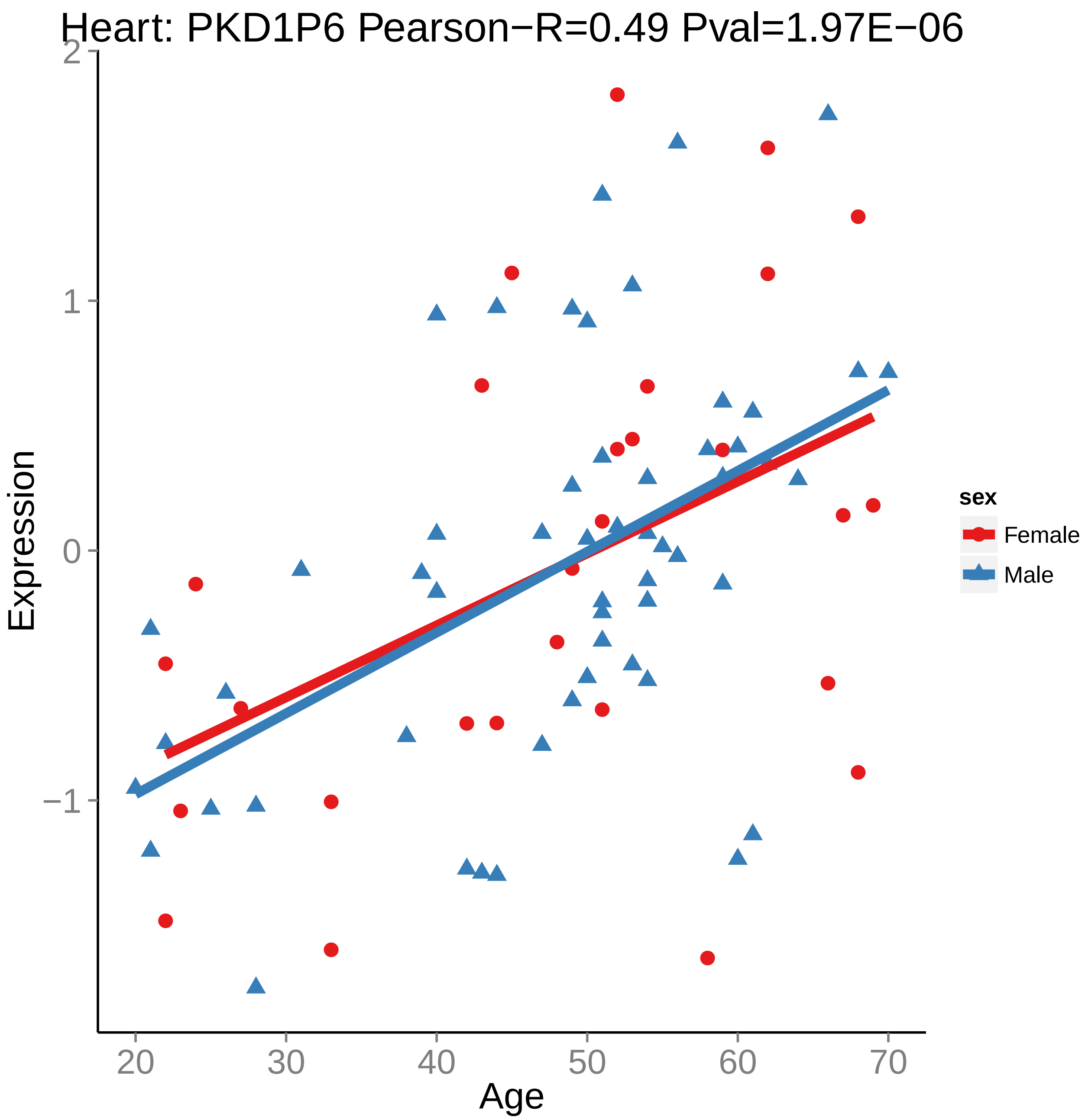

Heart: PKD1P6 Pearson−R=0.49 Pval=1.97E−06
Expression
Age
2
1
0
−1
20
30
40
50
60
70
sex
Female
Male

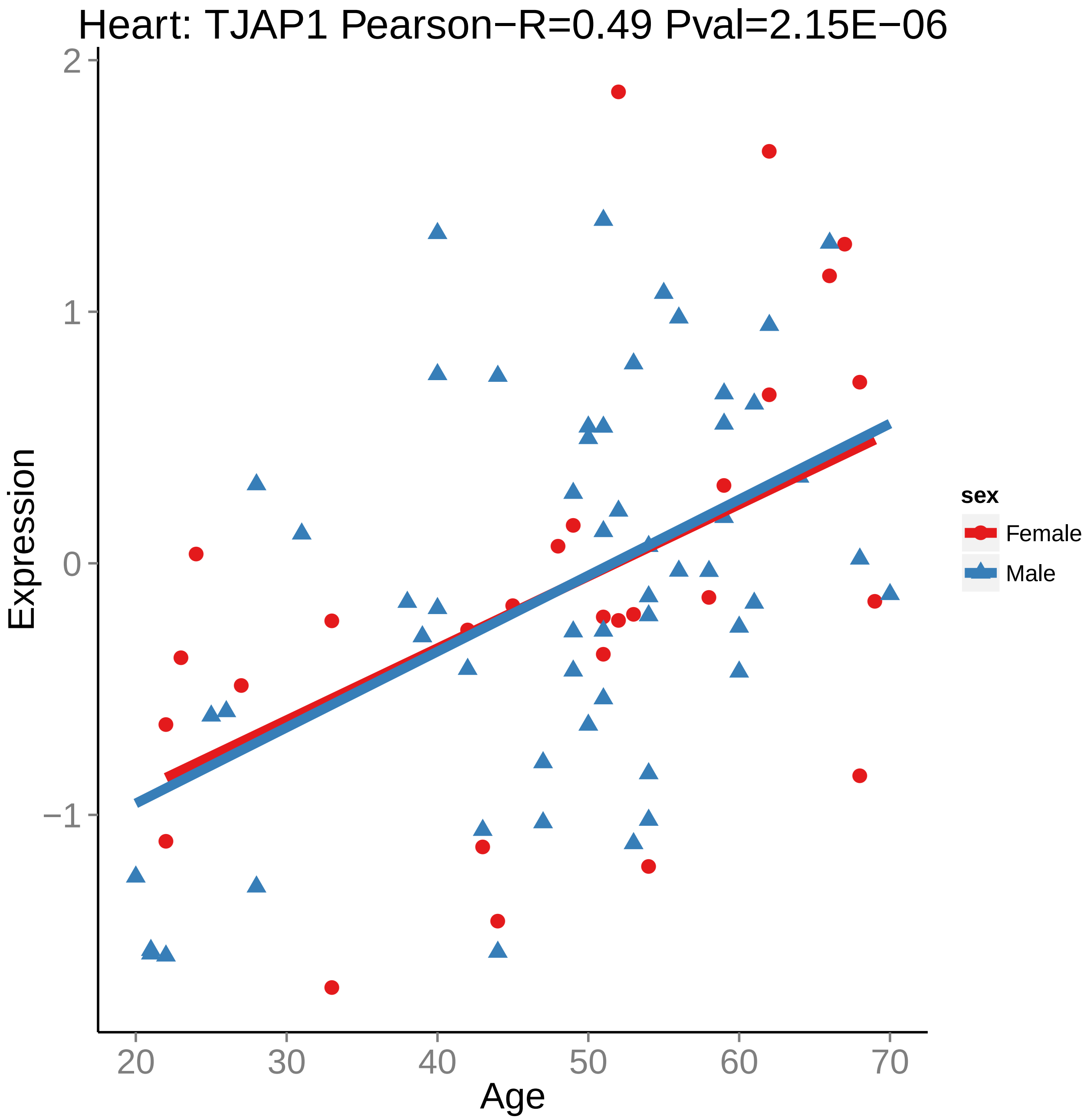

Heart: TJAP1 Pearson−R=0.49 Pval=2.15E−06
Expression
Age
2
1
0
−1
20
30
40
50
60
70
sex
Female
Male

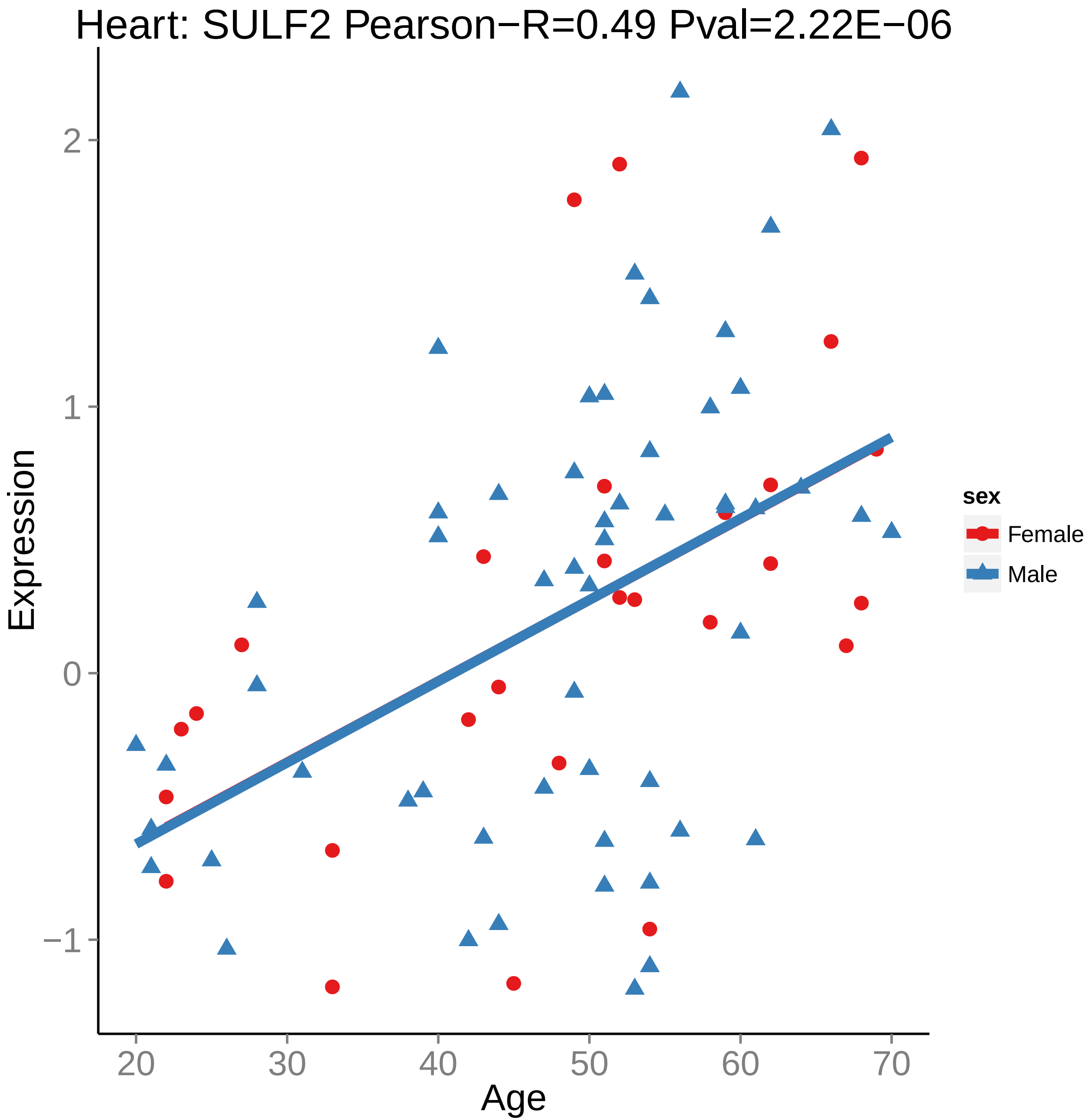

Heart: SULF2 Pearson−R=0.49 Pval=2.22E−06
Expression
Age
2
1
0
−1
20
30
40
50
60
70
sex
Female
Male

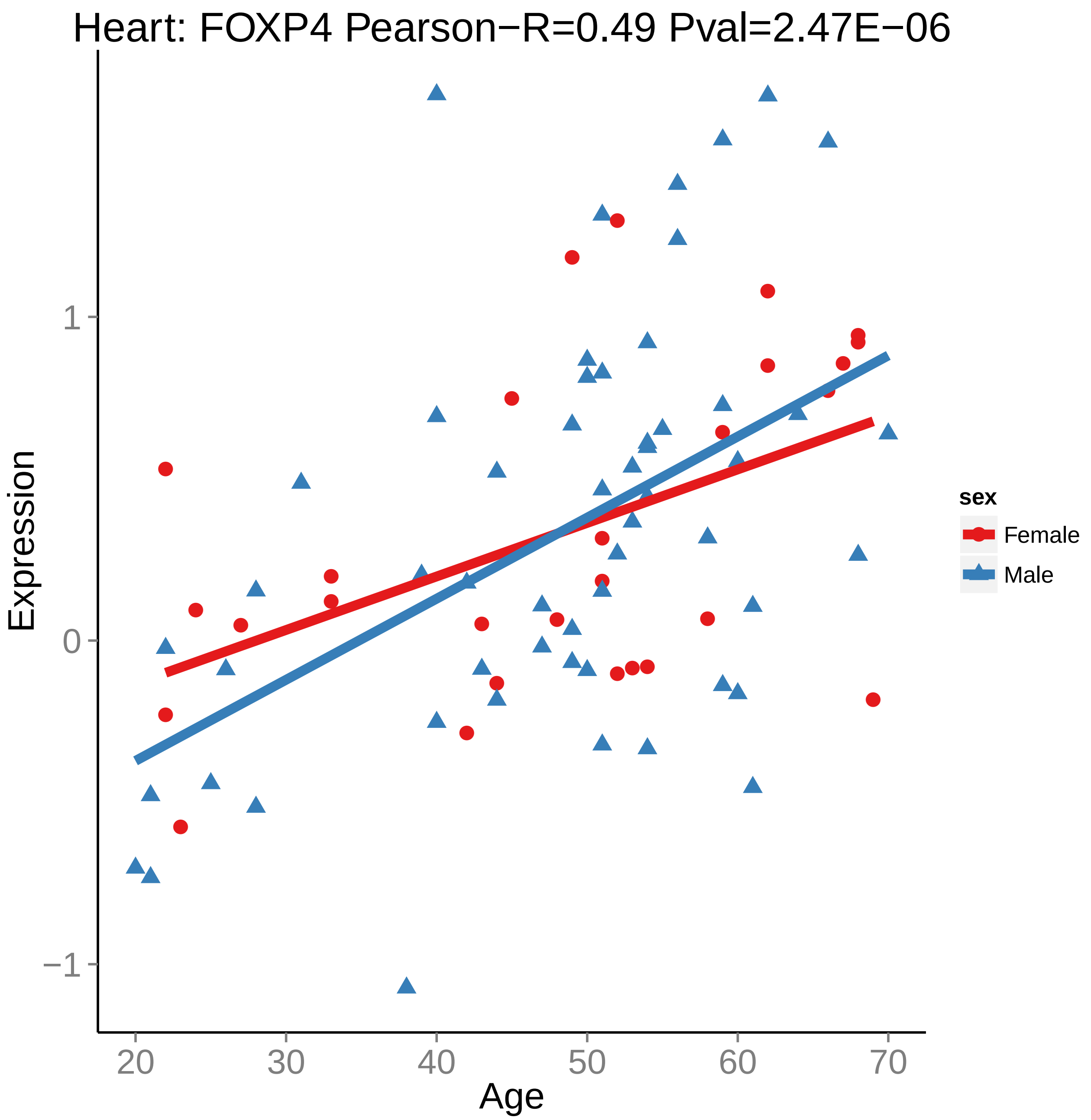
Heart: FOXP4 Pearson−R=0.49 Pval=2.47E−06
Expression
1
0
−1
20
30
40
50
60
70
Age
sex
Female
Male

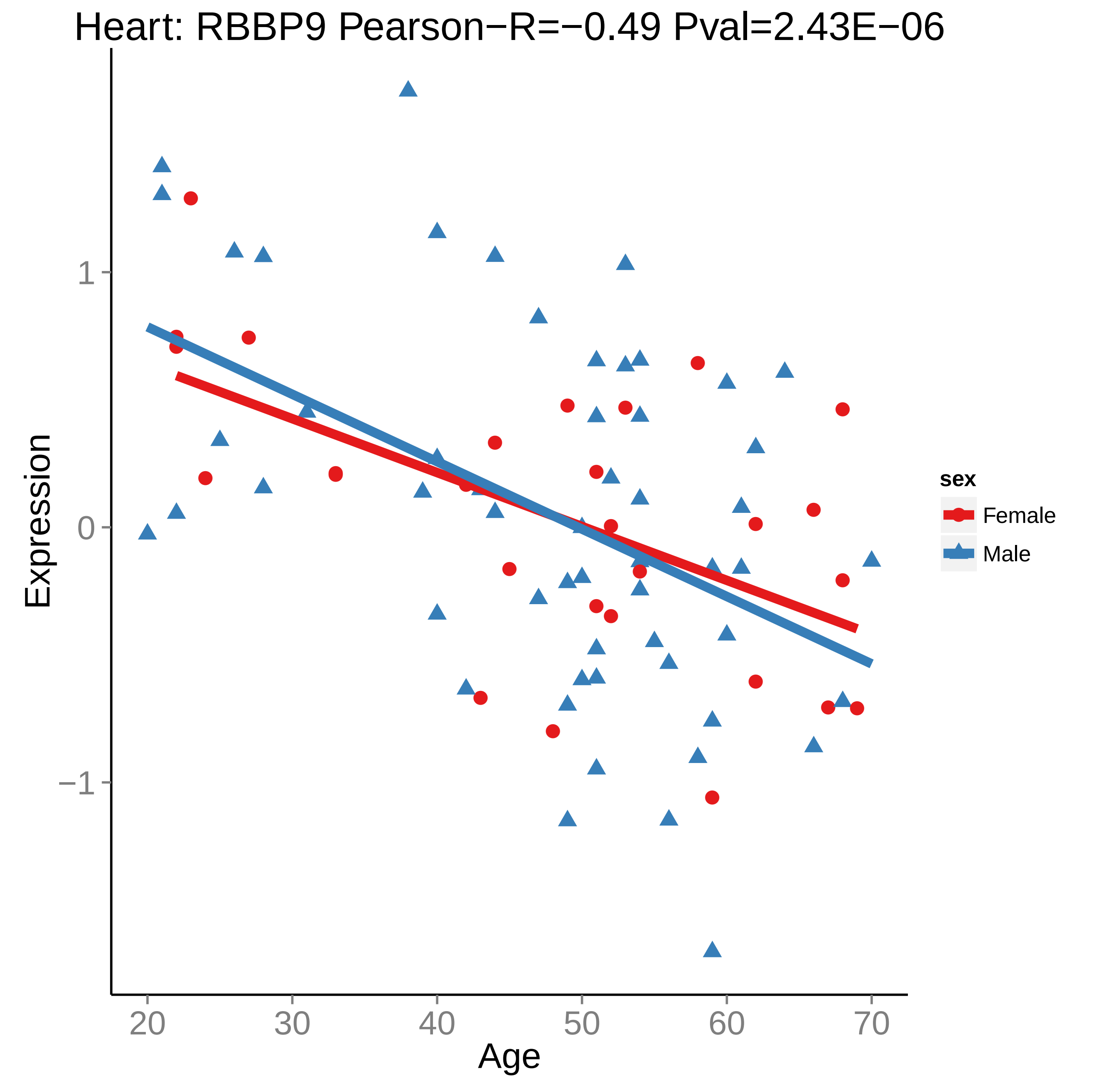
Heart: RBBP9 Pearson−R=−0.49 Pval=2.43E−06
Expression
Age
1
0
−1
20
30
40
50
60
70
sex
Female
Male

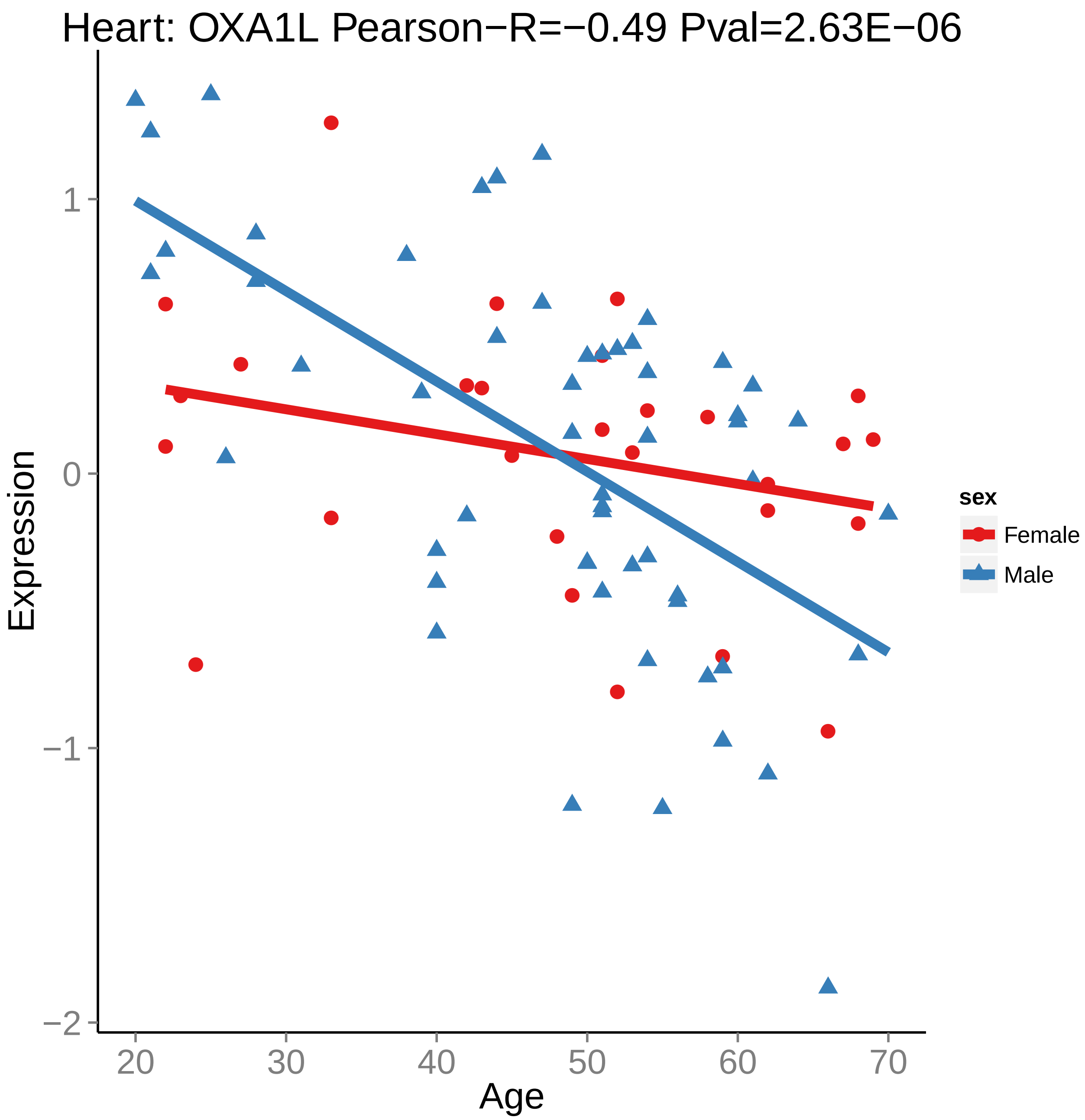

Heart: OXA1L Pearson−R=−0.49 Pval=2.63E−06
Expression
Age
1
0
−1
−2
20
30
40
50
60
70
sex
Female
Male

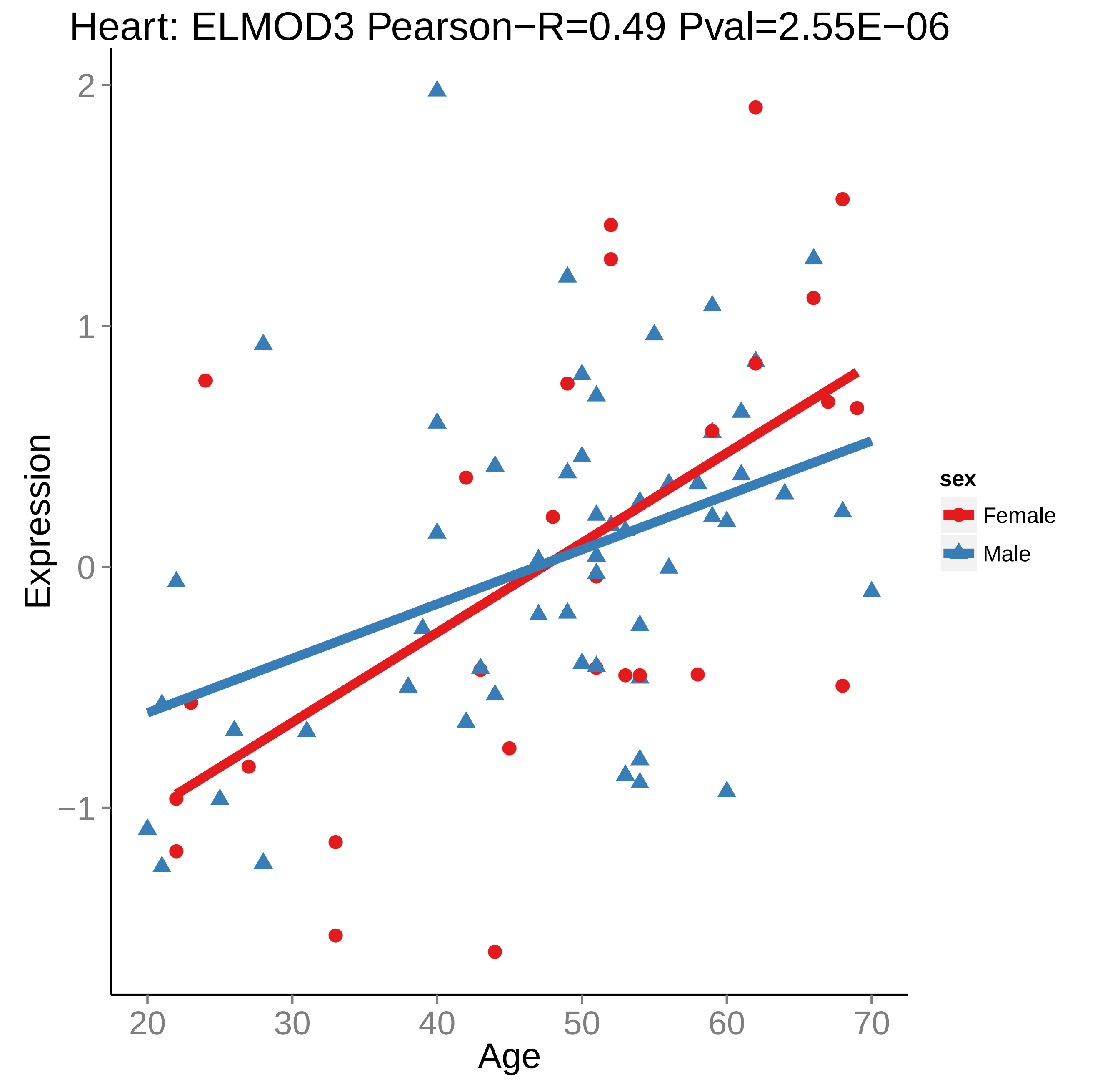

Heart: ELMOD3 Pearson−R=0.49 Pval=2.55E−06
Expression
Age
2
1
0
−1
20
30
40
50
60
70
sex
Female
Male

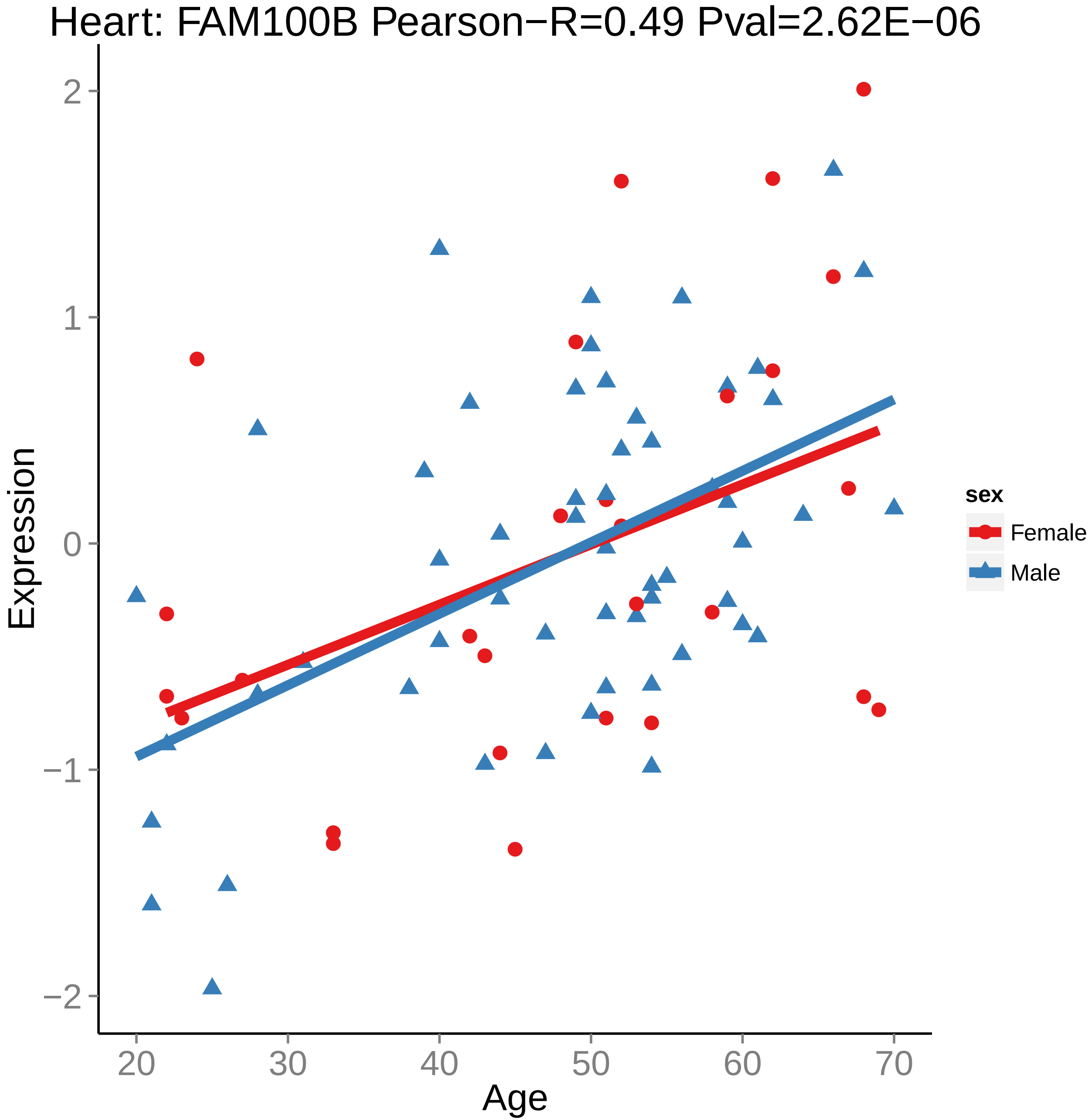
Heart: FAM100B Pearson−R=0.49 Pval=2.62E−06
Expression
Age
2
1
0
−1
−2
20
30
40
50
60
70
sex
Female
Male

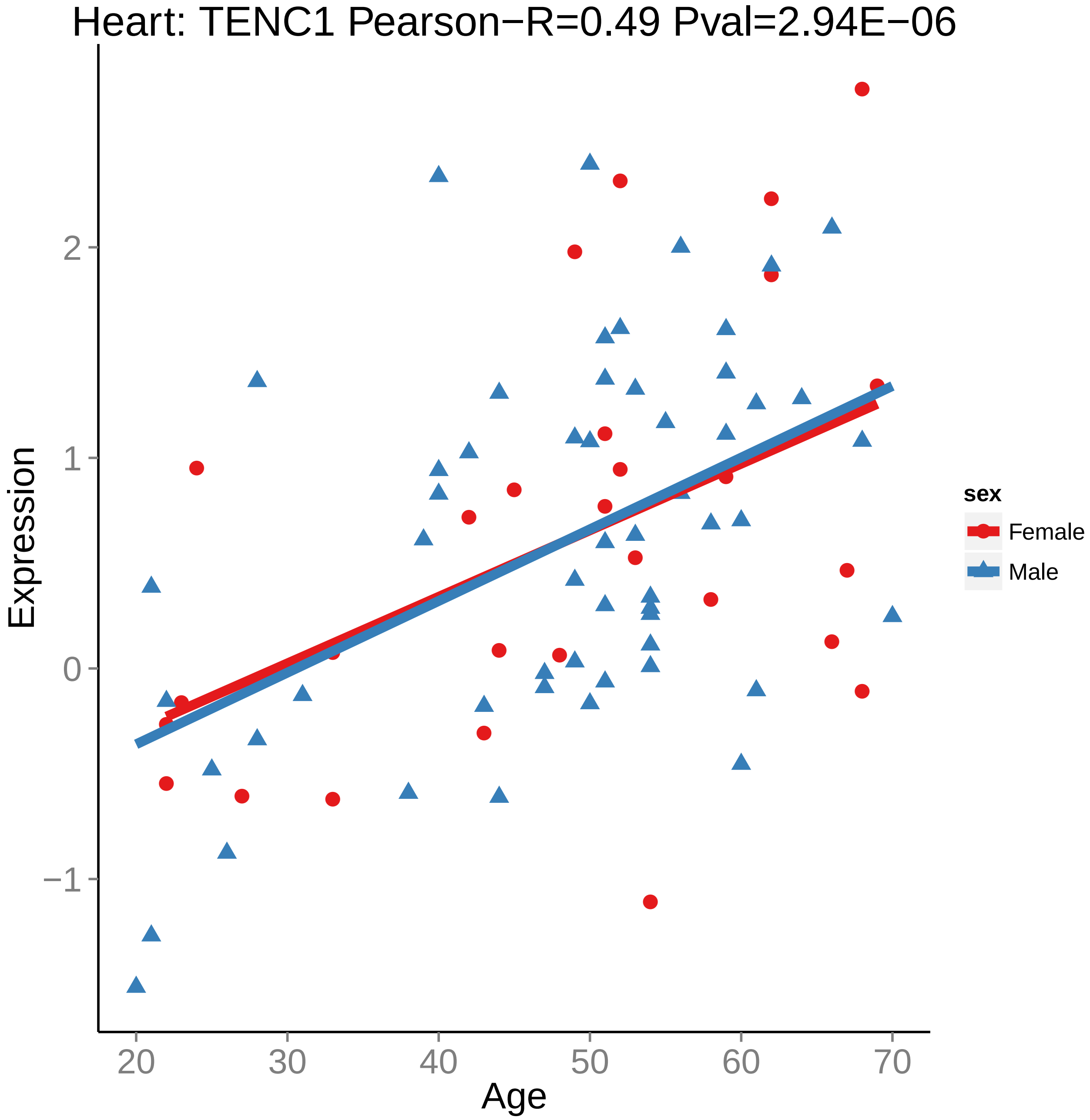

Heart: TENC1 Pearson−R=0.49 Pval=2.94E−06
Expression
2
1
0
−1
20
30
40
50
60
70
Age
sex
Female
Male

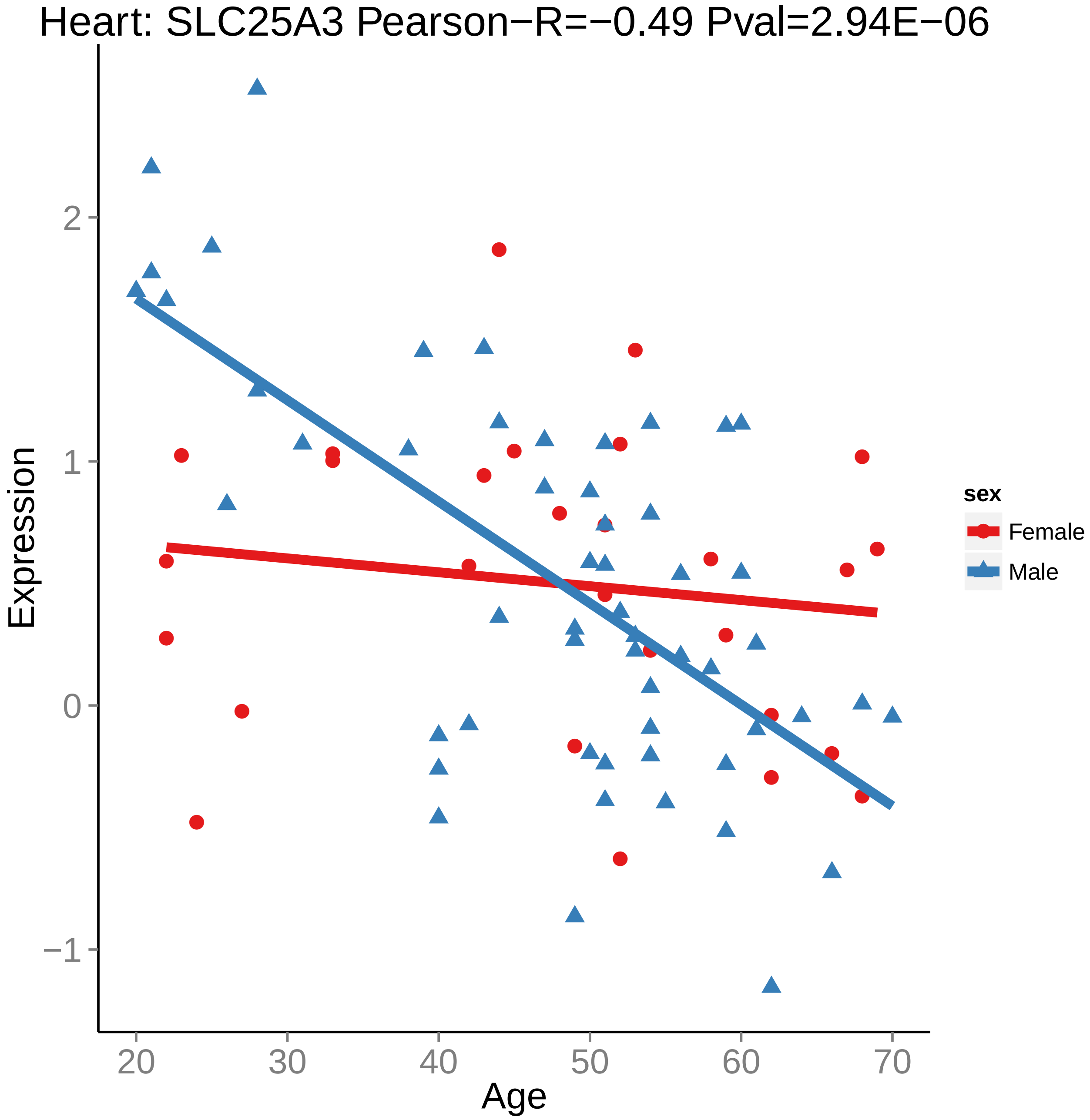
Heart: SLC25A3 Pearson−R=−0.49 Pval=2.94E−06
Expression
Age
2
1
0
−1
20
30
40
50
60
70
sex
Female
Male

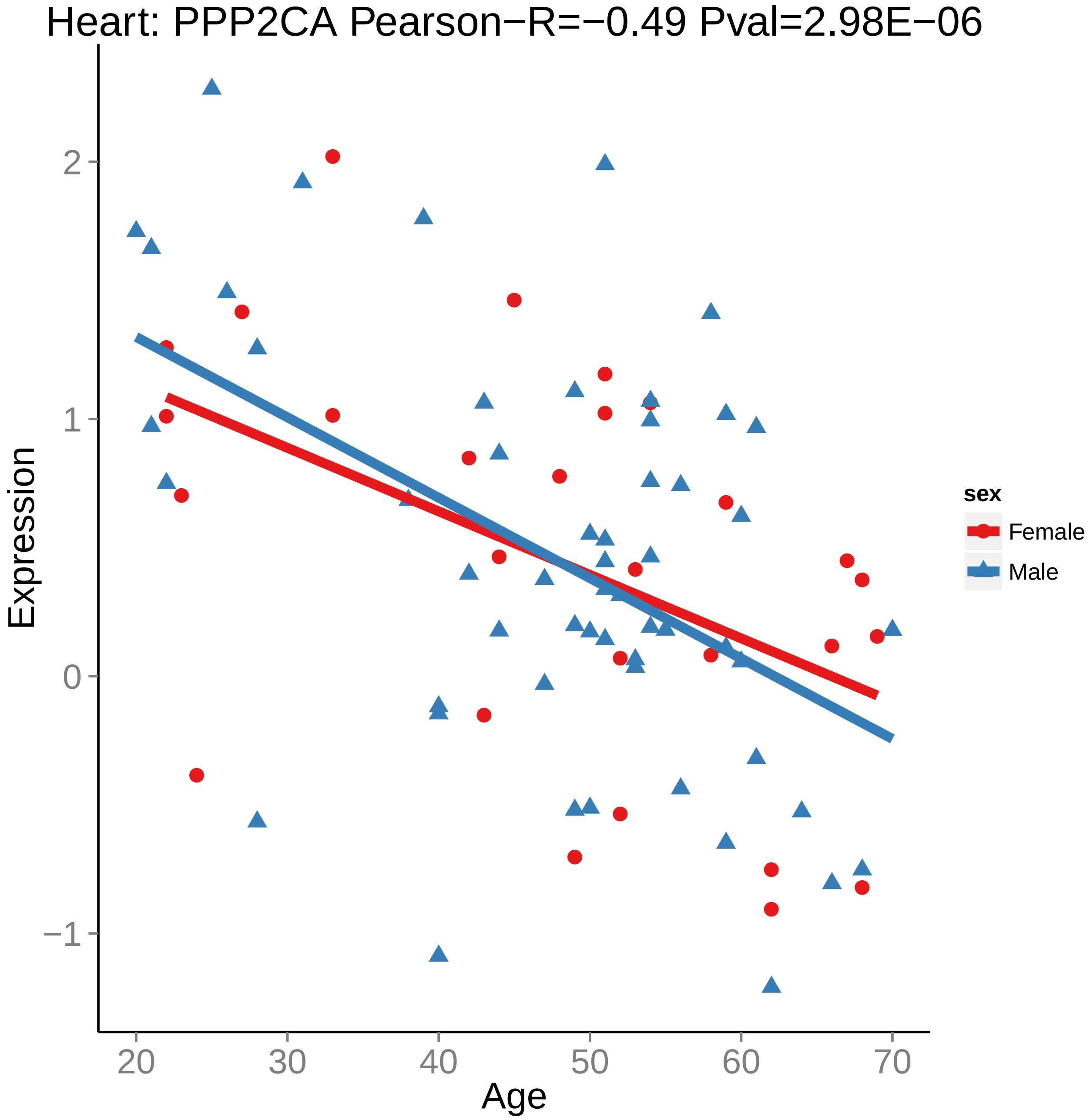

Heart: PPP2CA Pearson−R=−0.49 Pval=2.98E−06
Expression
Age
2
1
0
−1
20
30
40
50
60
70
sex
Female
Male

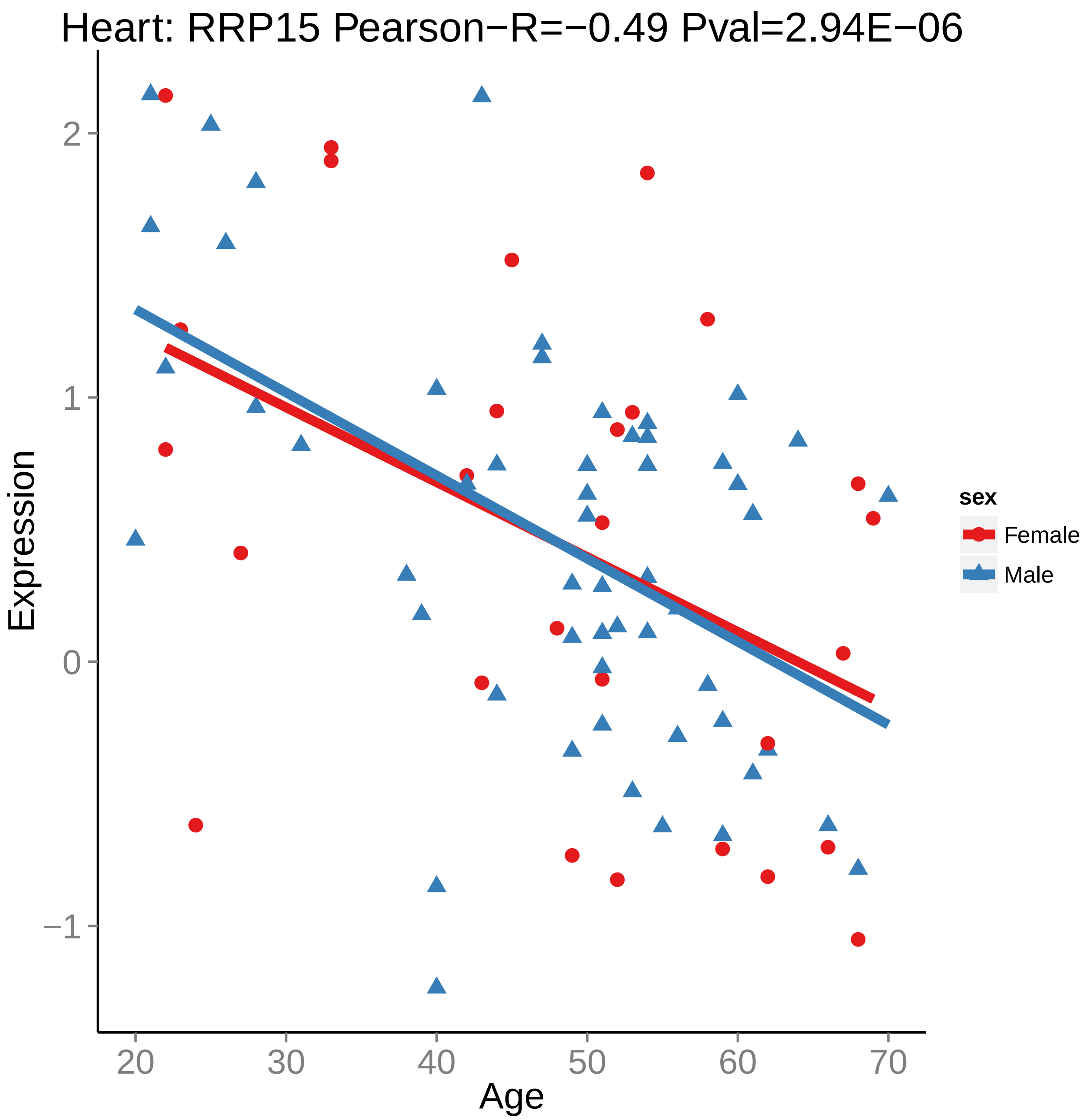

Heart: RRP15 Pearson−R=−0.49 Pval=2.94E−06
Expression
2
1
0
−1
20
30
40
50
60
70
Age
sex
Female
Male

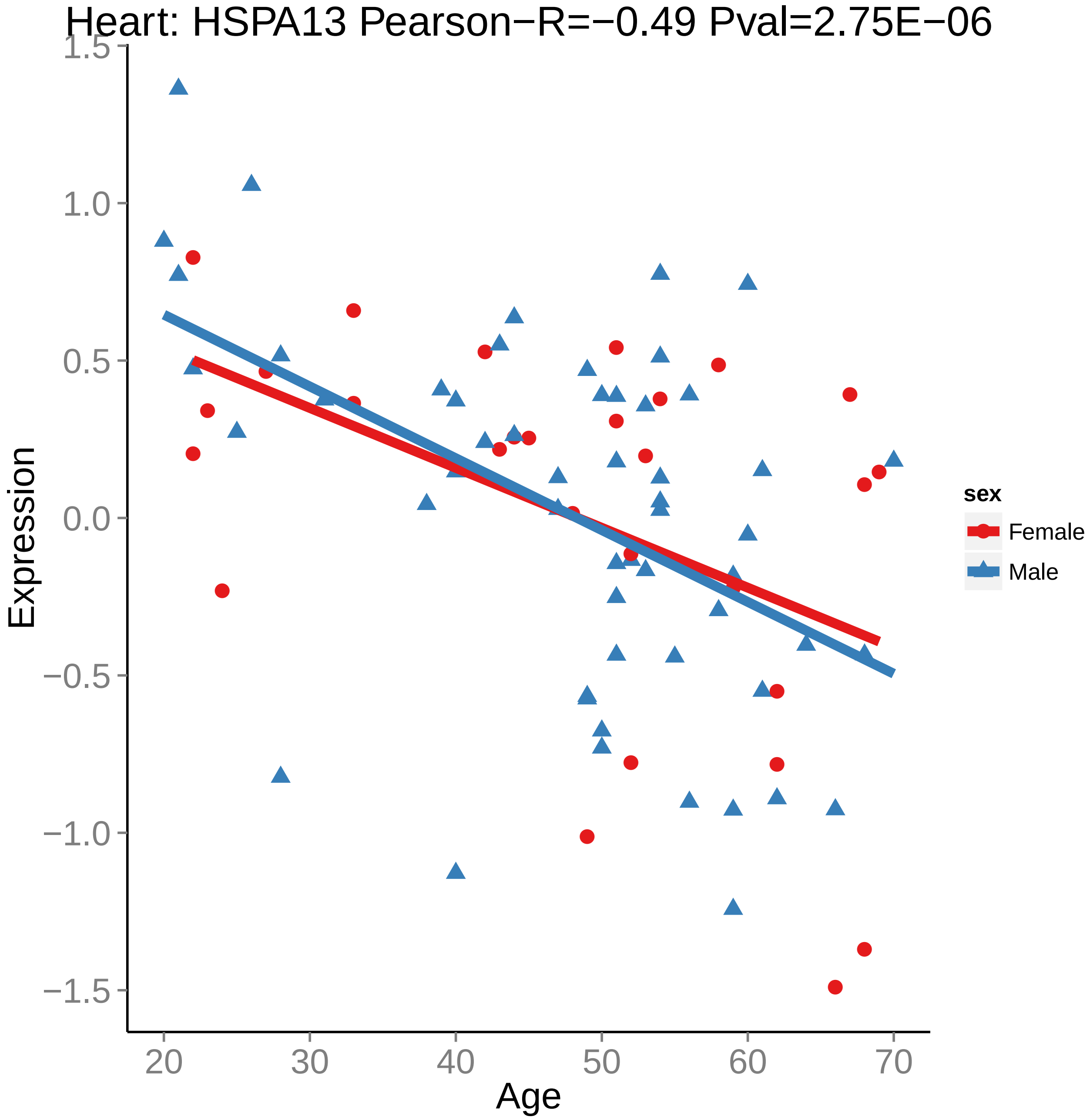
Heart: HSPA13 Pearson−R=−0.49 Pval=2.75E−06
Expression
Age
1.5
1.0
0.5
0.0
−0.5
−1.0
−1.5
20
30
40
50
60
70
sex
Female
Male

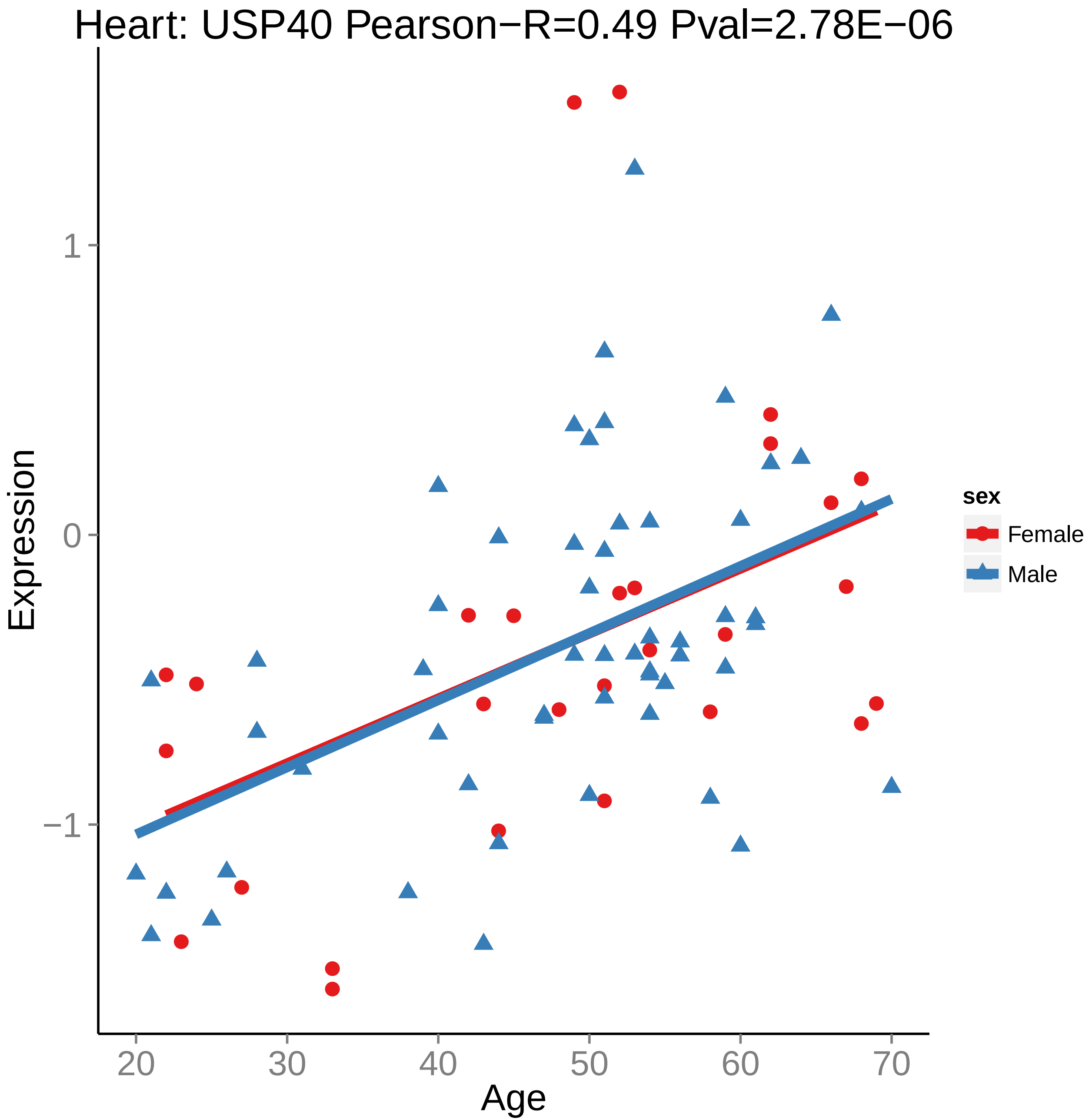
Heart: USP40 Pearson−R=0.49 Pval=2.78E−06
Expression
1
0
−1
20
30
40
50
60
70
Age
sex
Female
Male

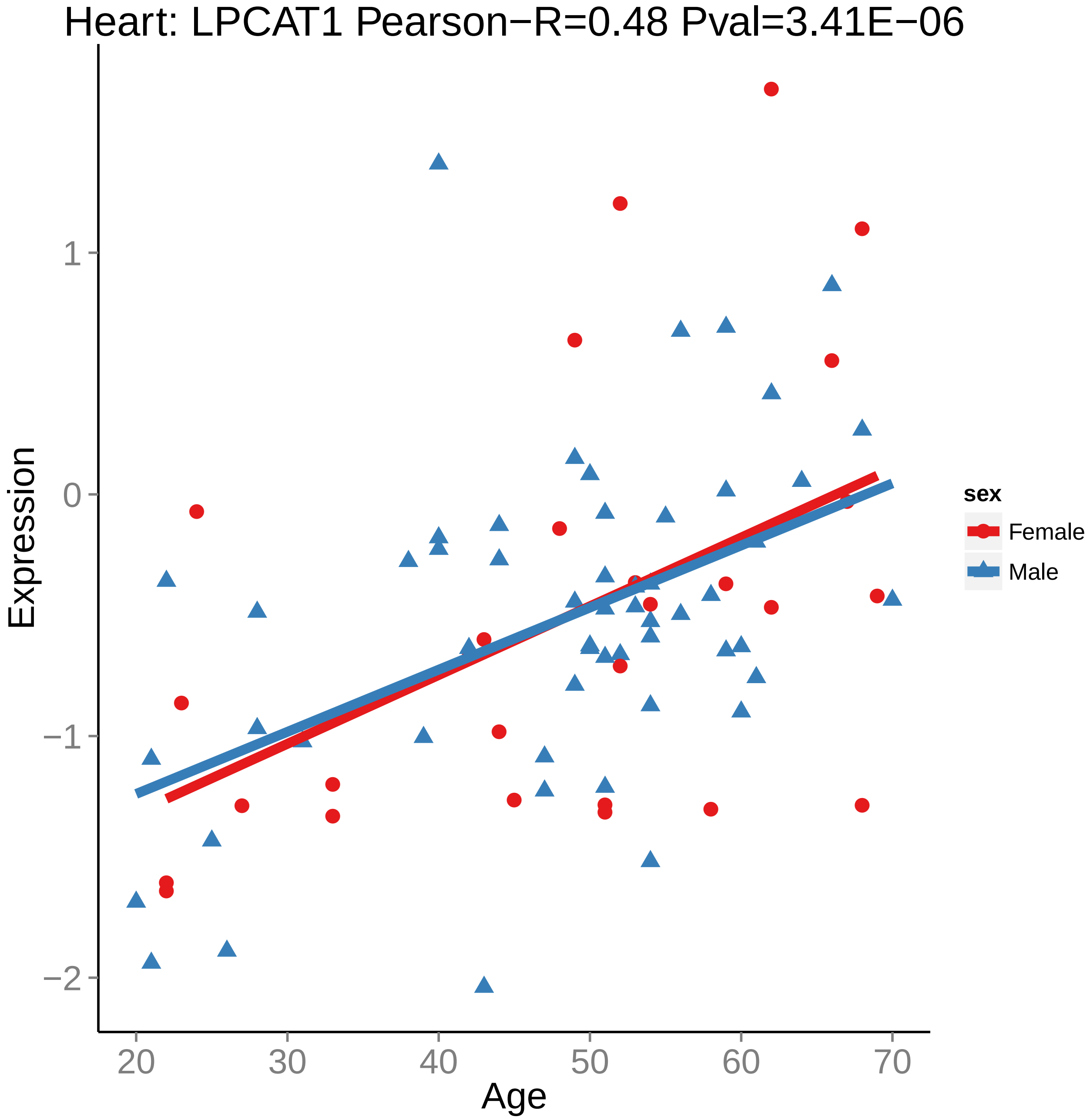

Heart: LPCAT1 Pearson−R=0.48 Pval=3.41E−06
Expression
Age
1
0
−1
−2
20
30
40
50
60
70
sex
Female
Male

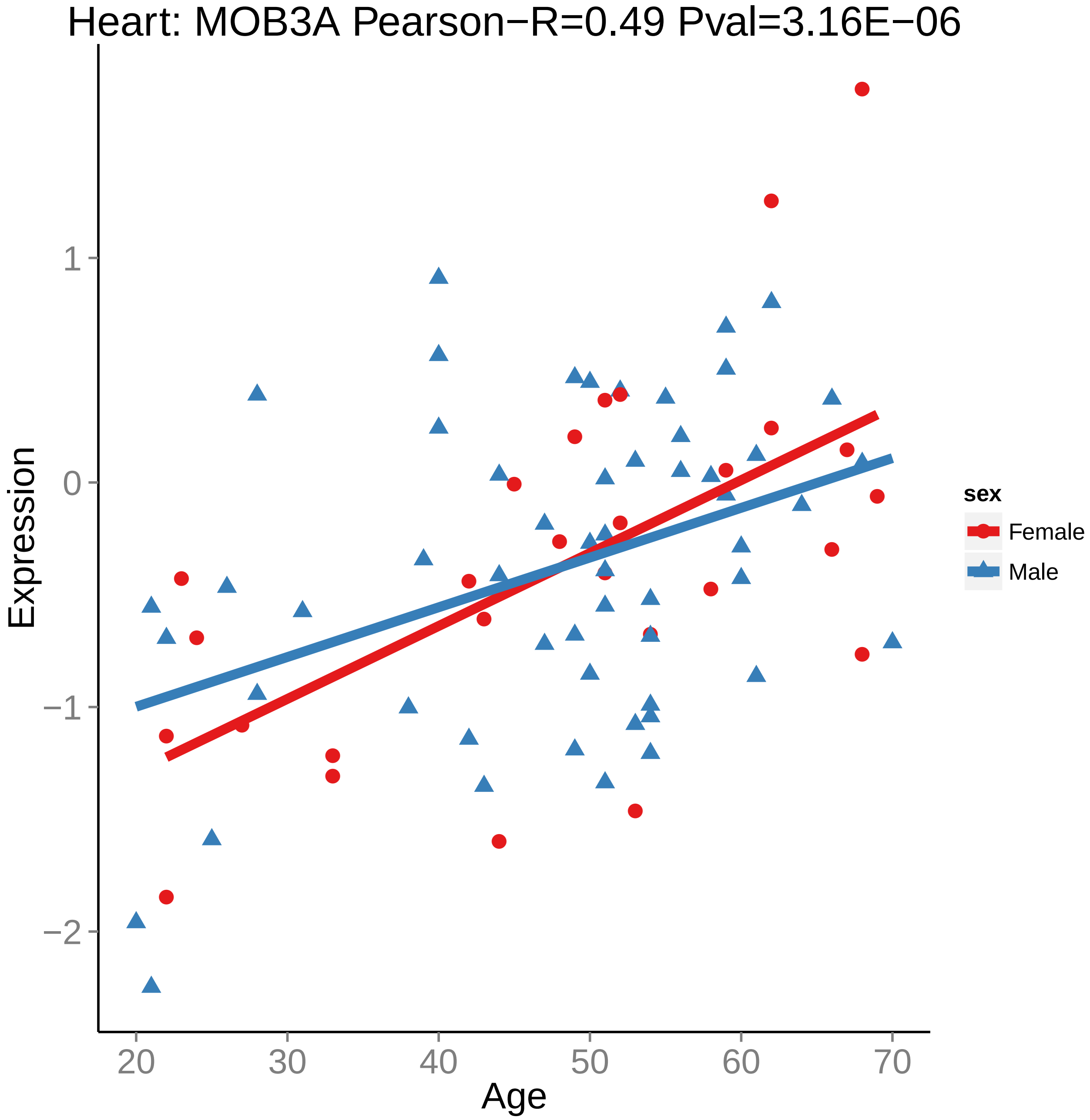

Heart: MOB3A Pearson−R=0.49 Pval=3.16E−06
Expression
1
0
−1
−2
20
30
40
50
60
70
Age
sex
Female
Male

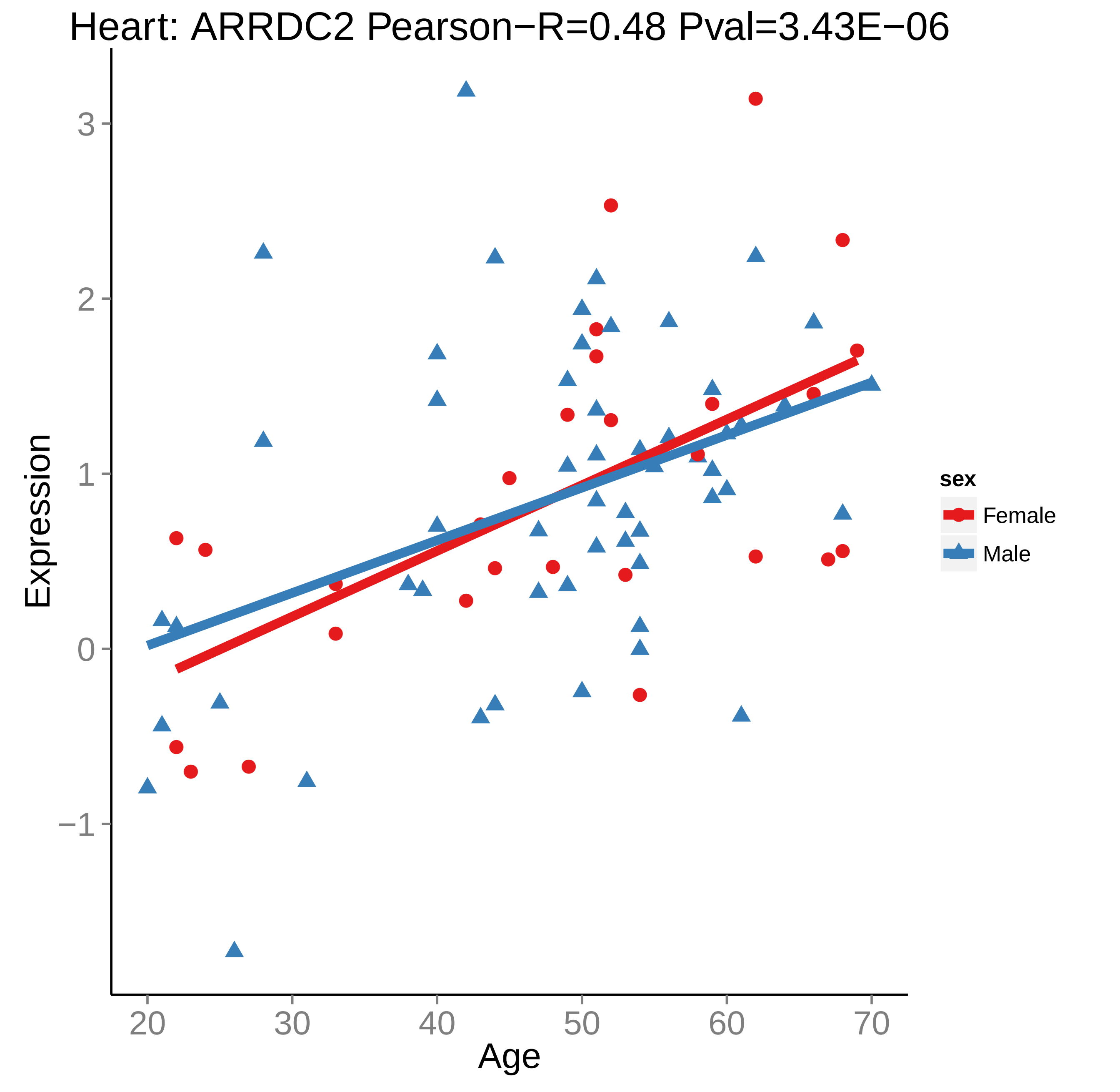

Heart: ARRDC2 Pearson−R=0.48 Pval=3.43E−06
Expression
Age
3
2
1
0
−1
20
30
40
50
60
70
sex
Female
Male

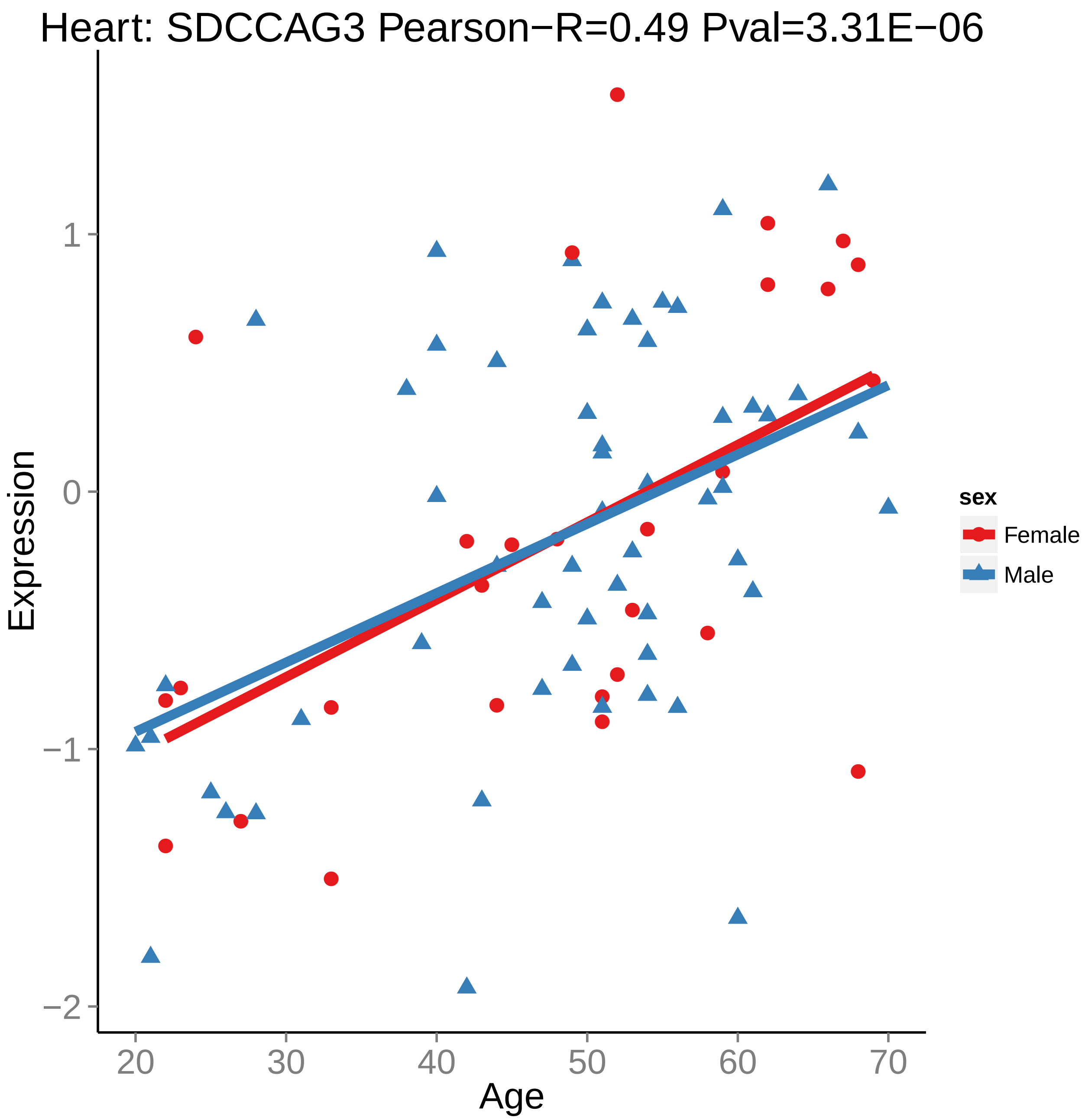

Heart: SDCCAG3 Pearson−R=0.49 Pval=3.31E−06
Expression
Age
1
0
−1
−2
20
30
40
50
60
70
sex
Female
Male

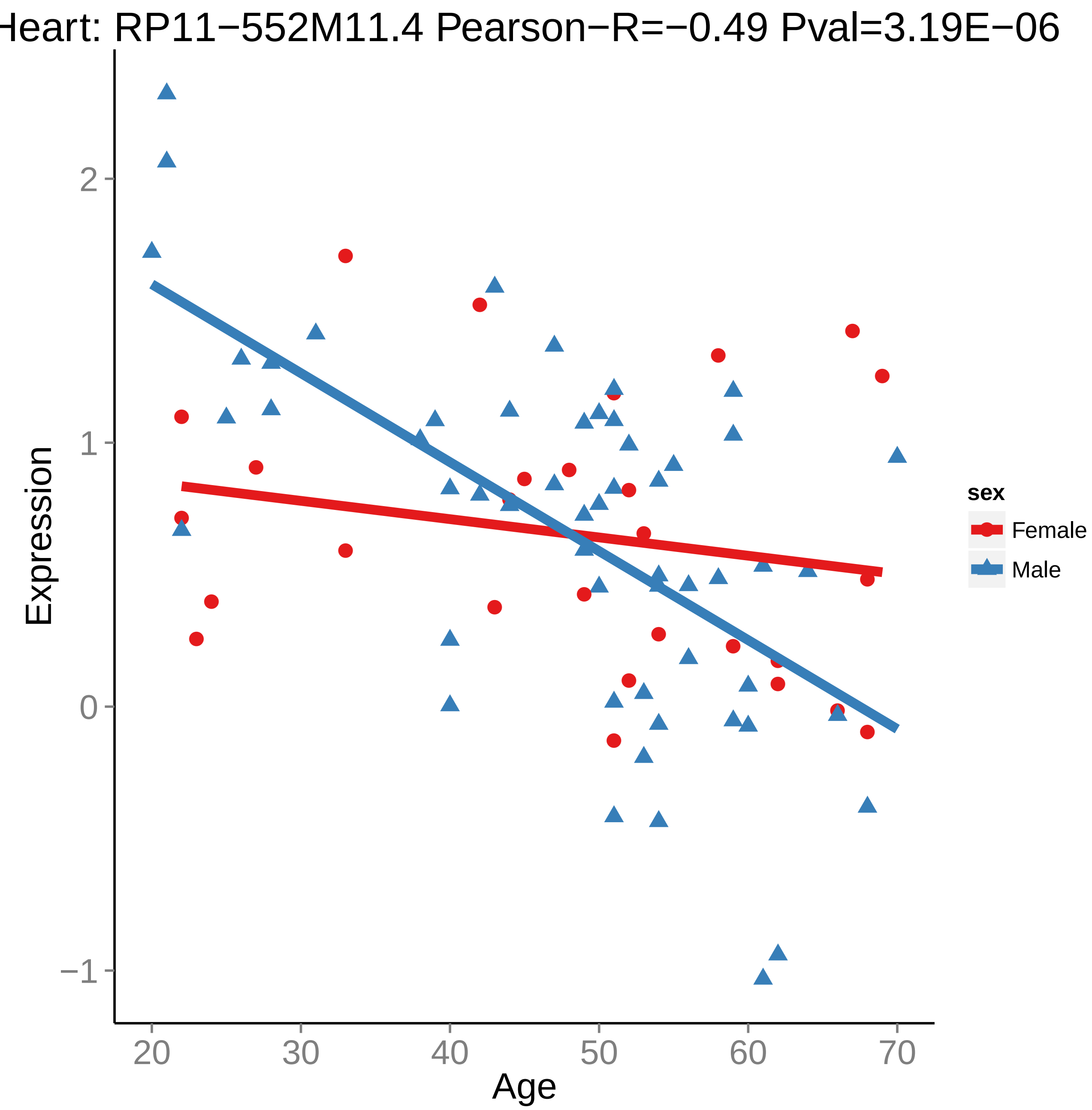

Heart: RP11−552M11.4 Pearson−R=−0.49 Pval=3.19E−06
Expression
2
1
0
−1
Age
20
30
40
50
60
70
sex
Female
Male

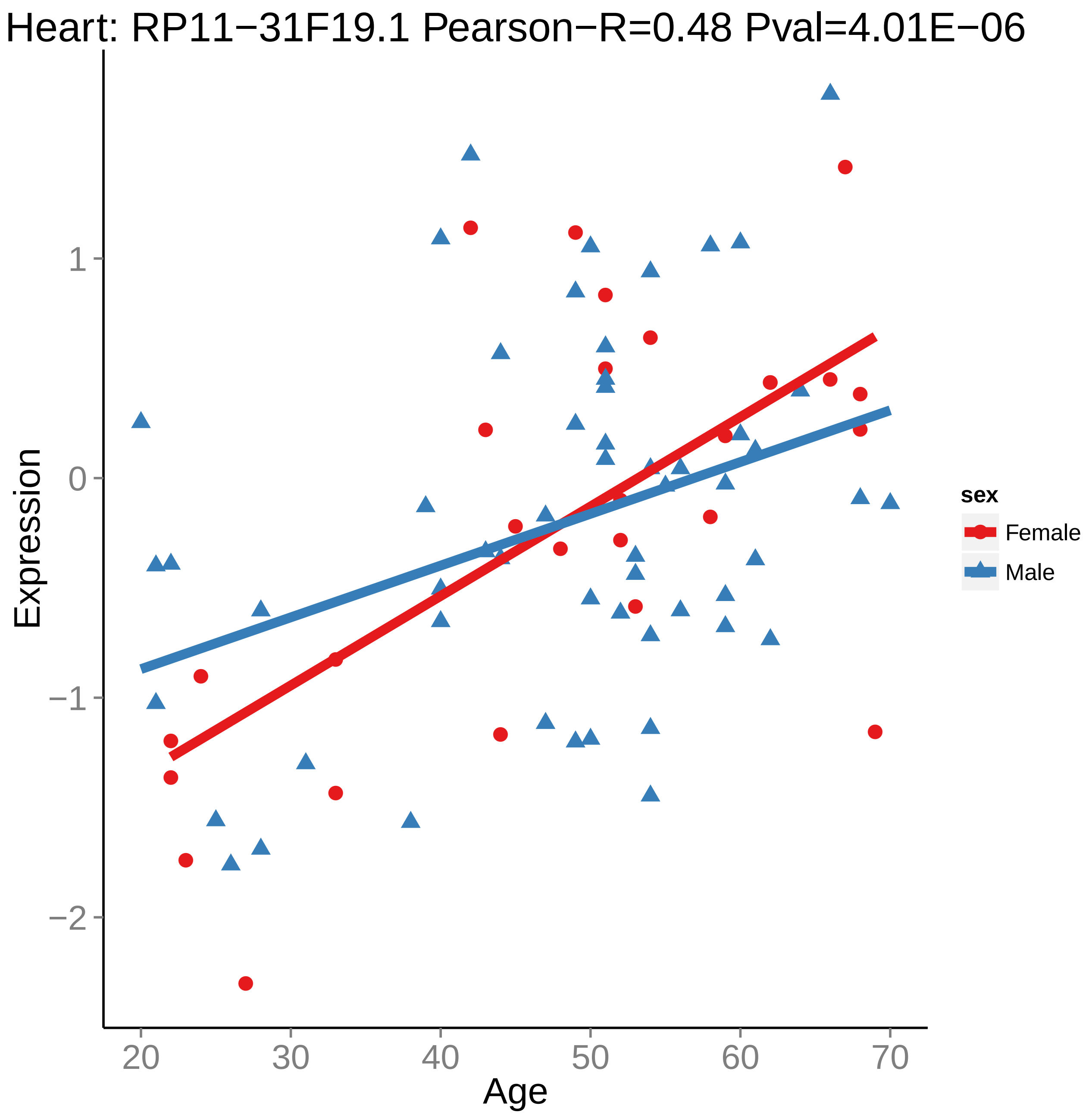
Heart: RP11−31F19.1 Pearson−R=0.48 Pval=4.01E−06
Expression
1
0
−1
−2
20
30
40
50
60
70
Age
sex
Female
Male

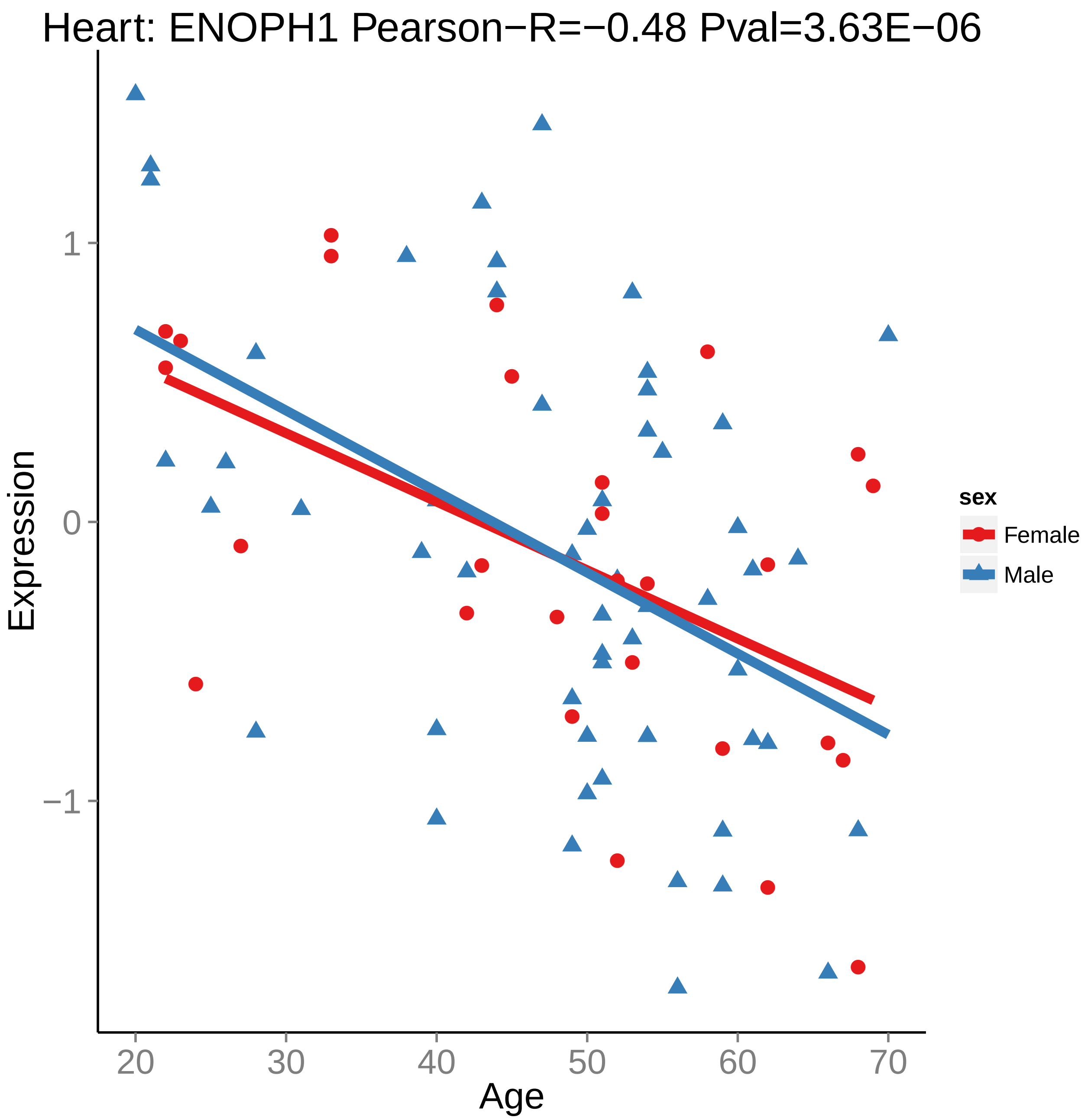
Heart: ENOPH1 Pearson−R=−0.48 Pval=3.63E−06
Expression
1
0
−1
20
30
40
50
60
70
Age
sex
Female
Male

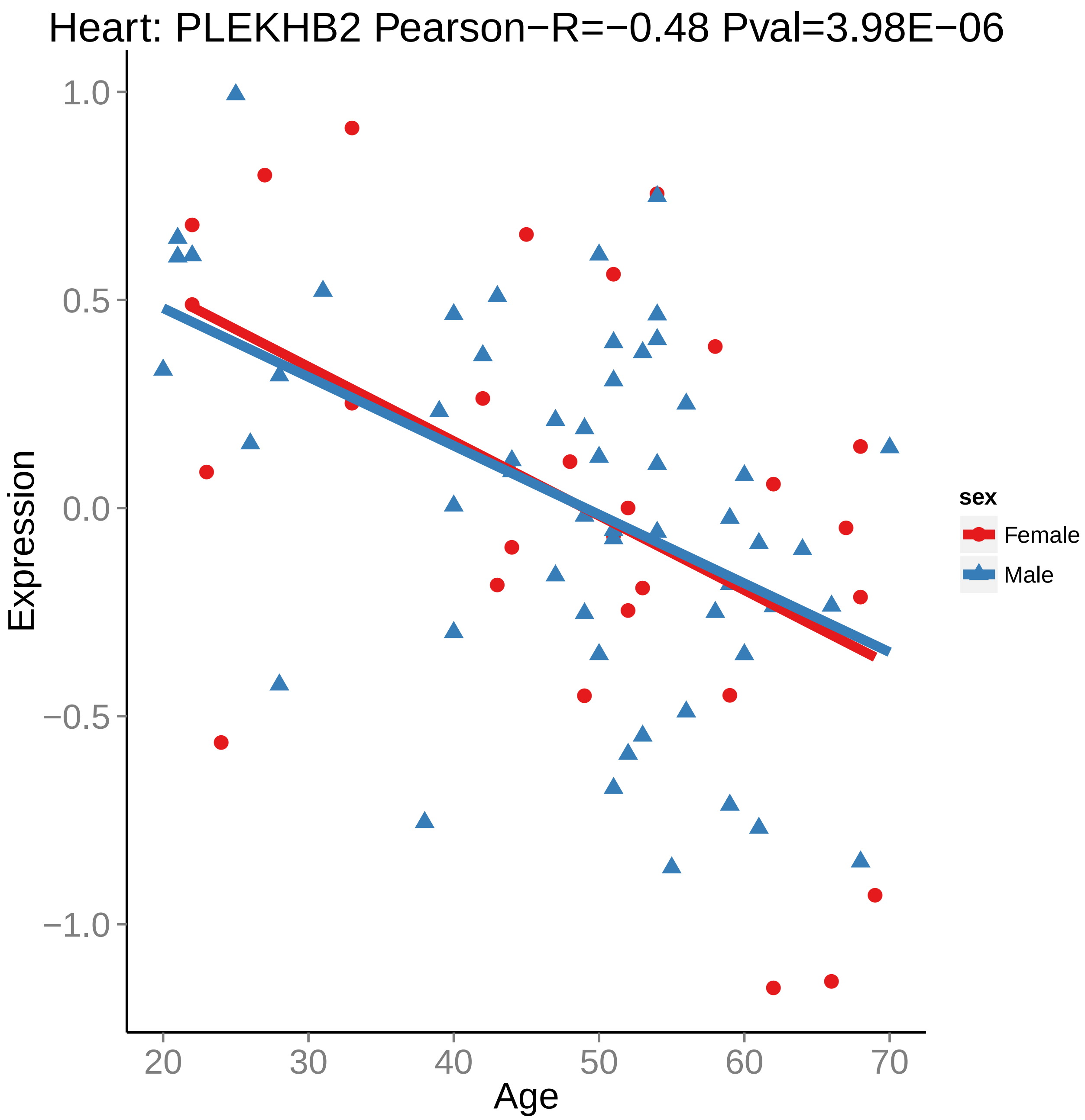

Heart: PLEKHB2 Pearson−R=−0.48 Pval=3.98E−06
Expression
Age
1.0
0.5
0.0
−0.5
−1.0
20
30
40
50
60
70
sex
Female
Male

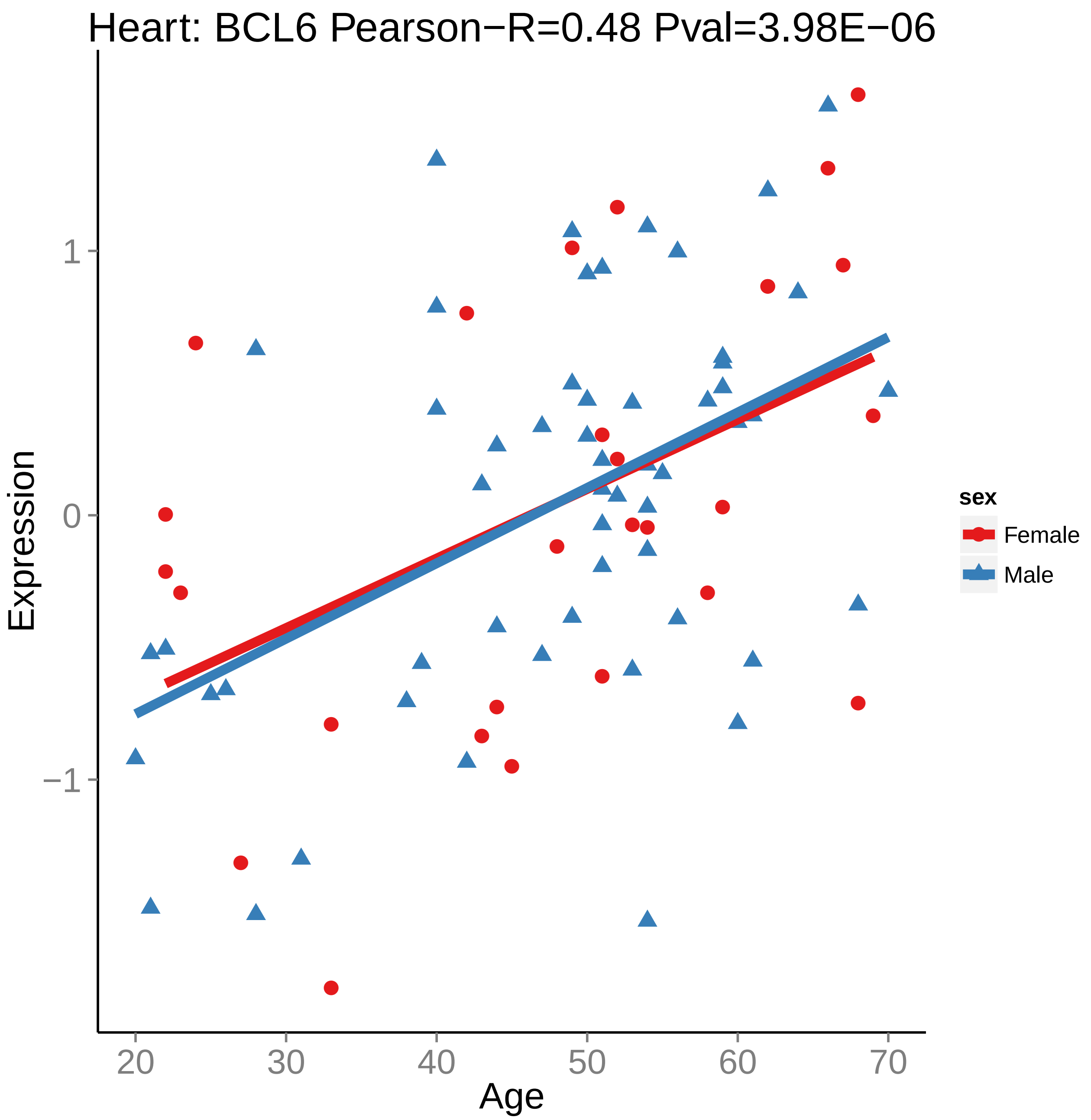
Heart: BCL6 Pearson−R=0.48 Pval=3.98E−06
Expression
1
0
−1
20
30
40
50
60
70
Age
sex
Female
Male

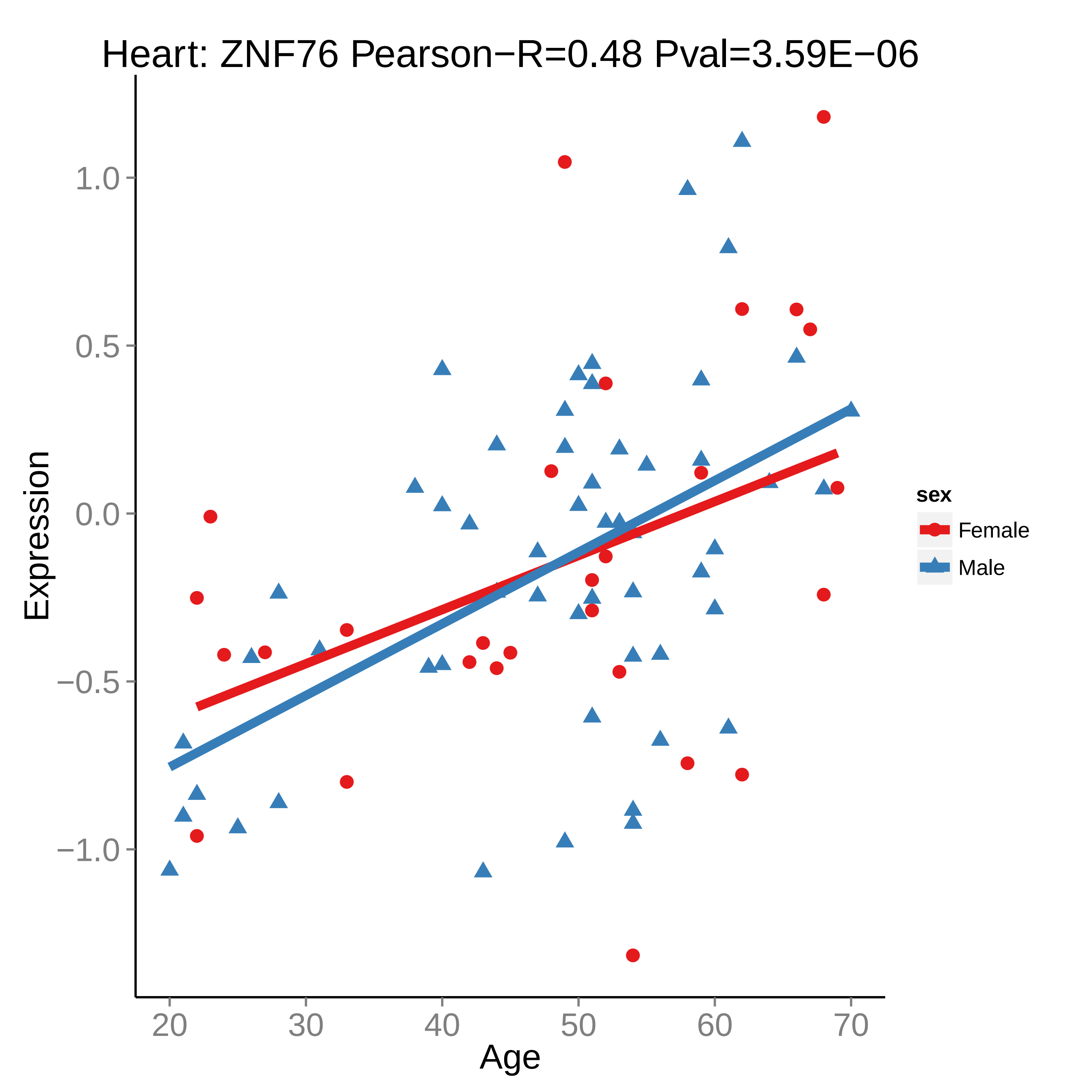
Heart: ZNF76 Pearson−R=0.48 Pval=3.59E−06
Expression
1.0
0.5
0.0
−0.5
−1.0
20
30
40
50
60
70
Age
sex
Female
Male

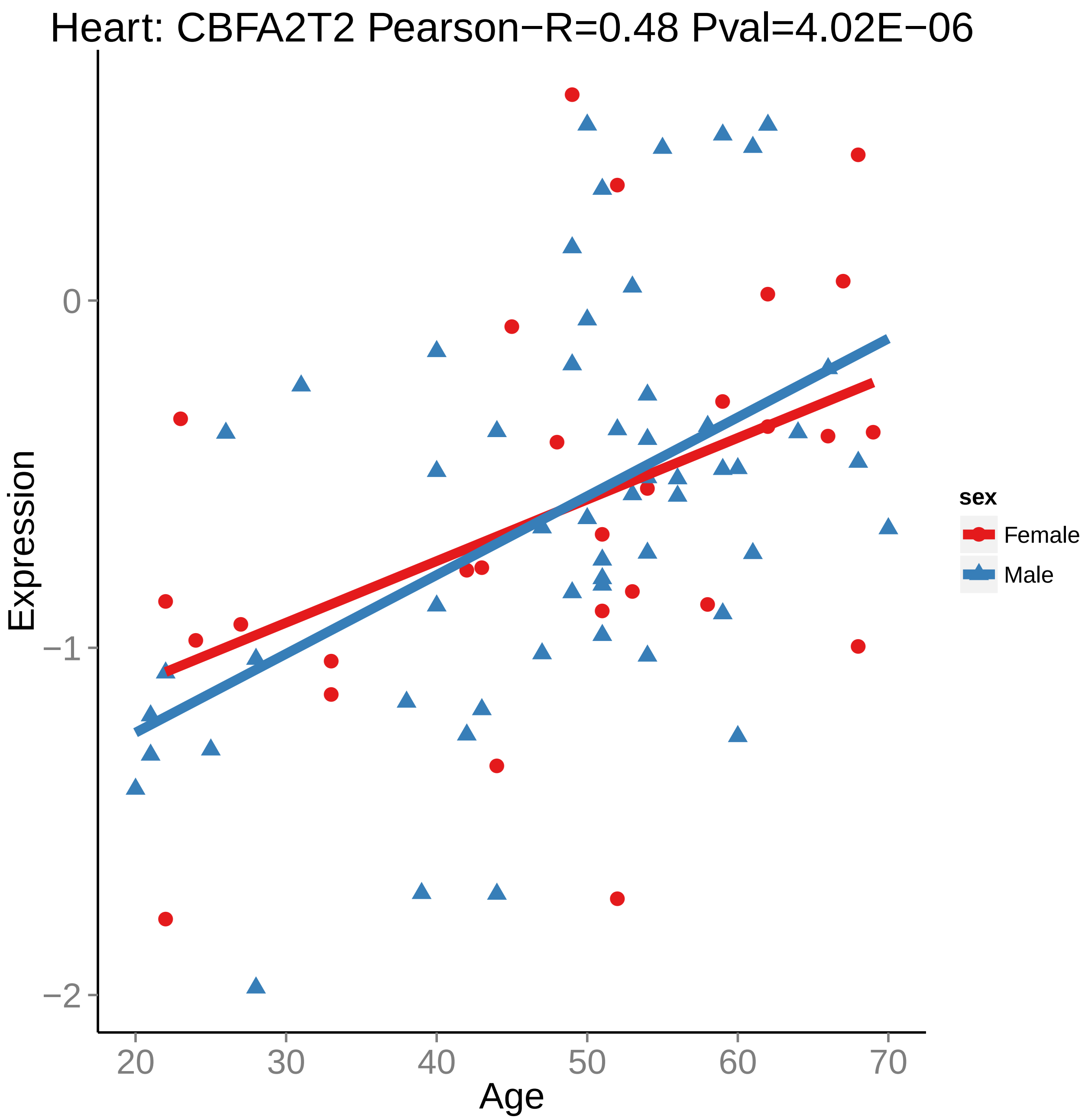

Heart: CBFA2T2 Pearson−R=0.48 Pval=4.02E−06
Expression
0
−1
−2
20
30
40
50
60
70
Age
sex
Female
Male

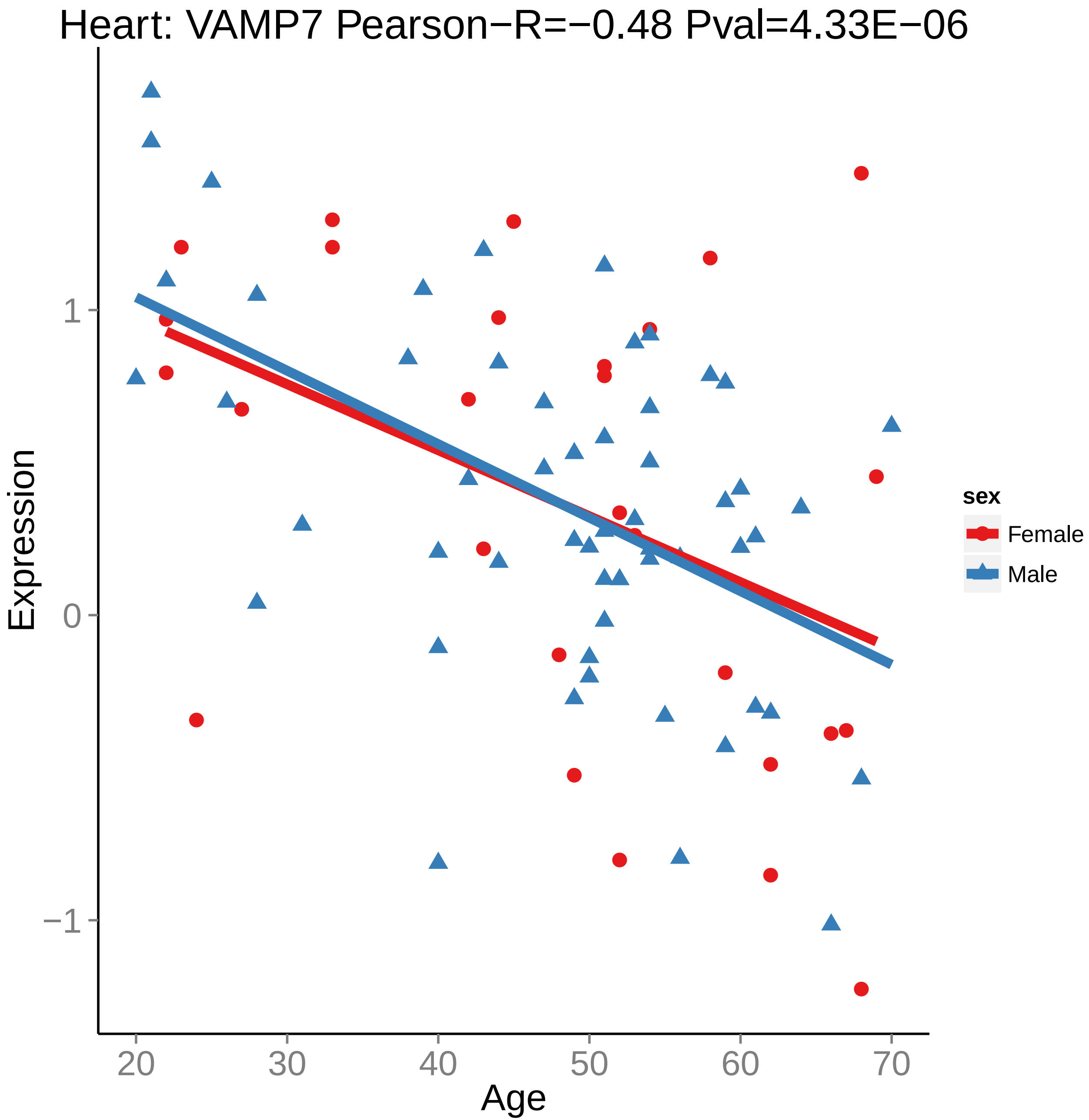

Heart: VAMP7 Pearson−R=−0.48 Pval=4.33E−06
Expression
1
0
−1
20
30
40
50
60
70
Age
sex
Female
Male

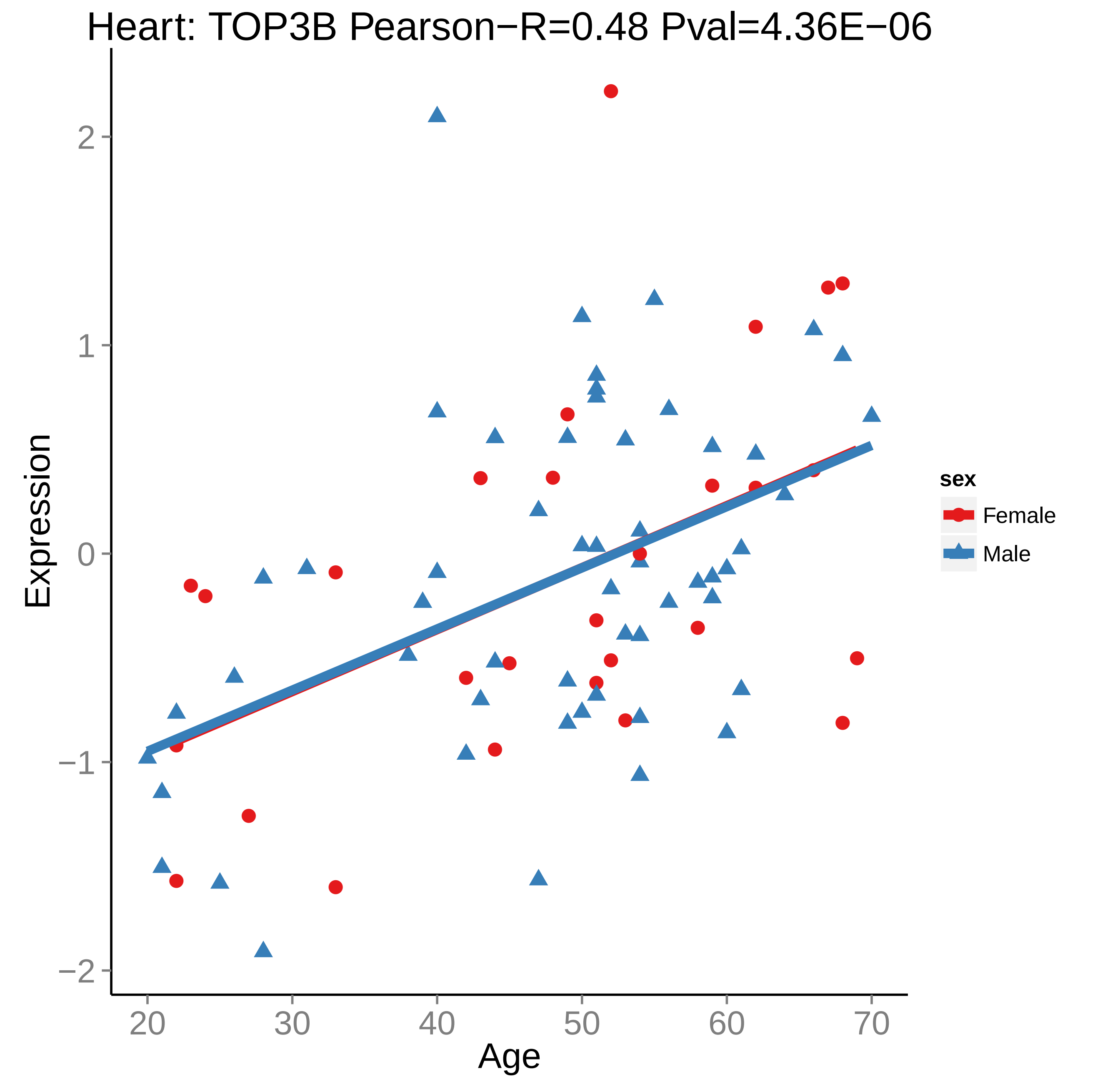

Heart: TOP3B Pearson−R=0.48 Pval=4.36E−06
Expression
2
1
0
−1
−2
20
30
40
50
60
70
Age
sex
Female
Male

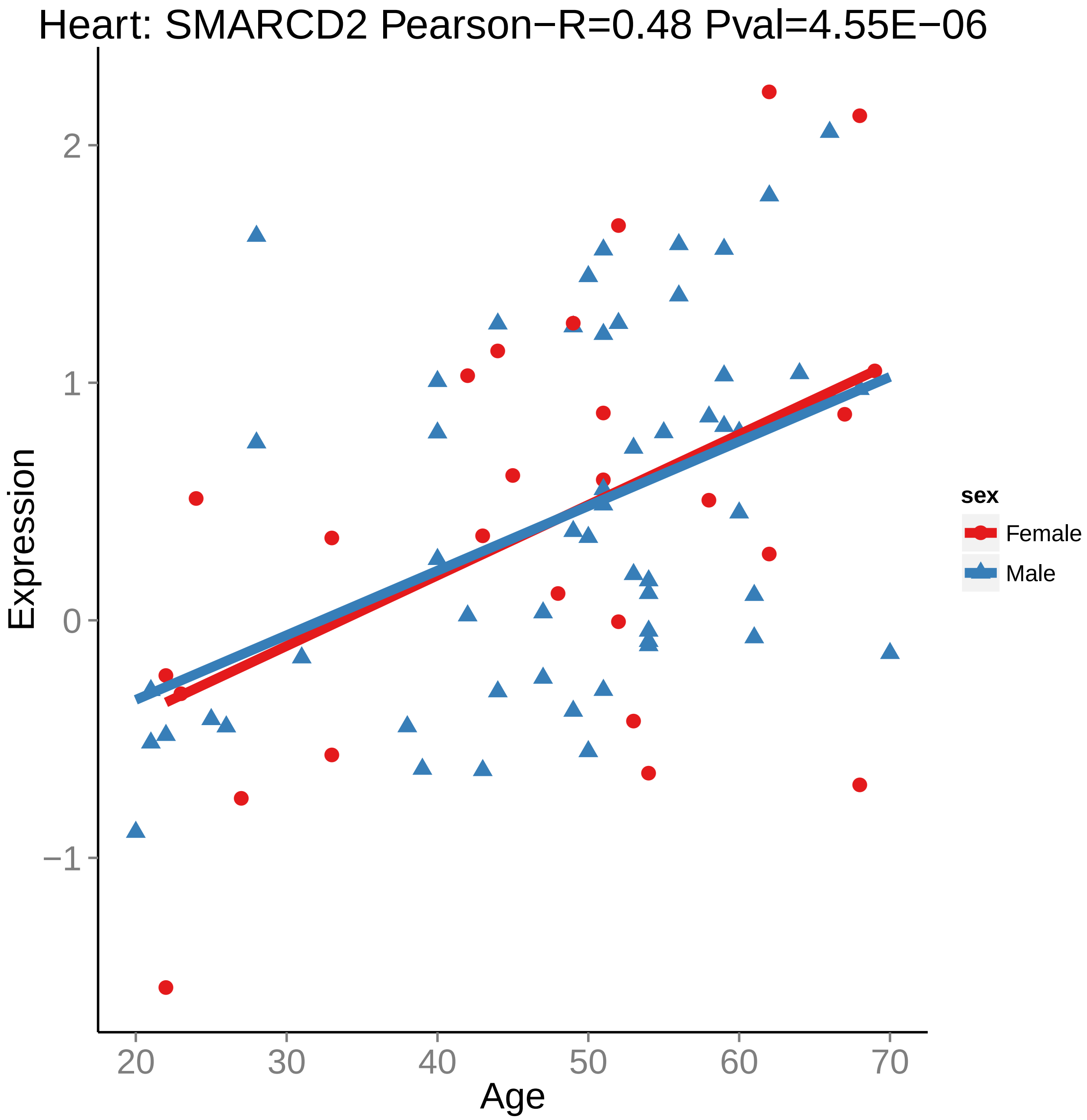

Heart: SMARCD2 Pearson−R=0.48 Pval=4.55E−06
Expression
Age
2
1
0
−1
20
30
40
50
60
70
sex
Female
Male

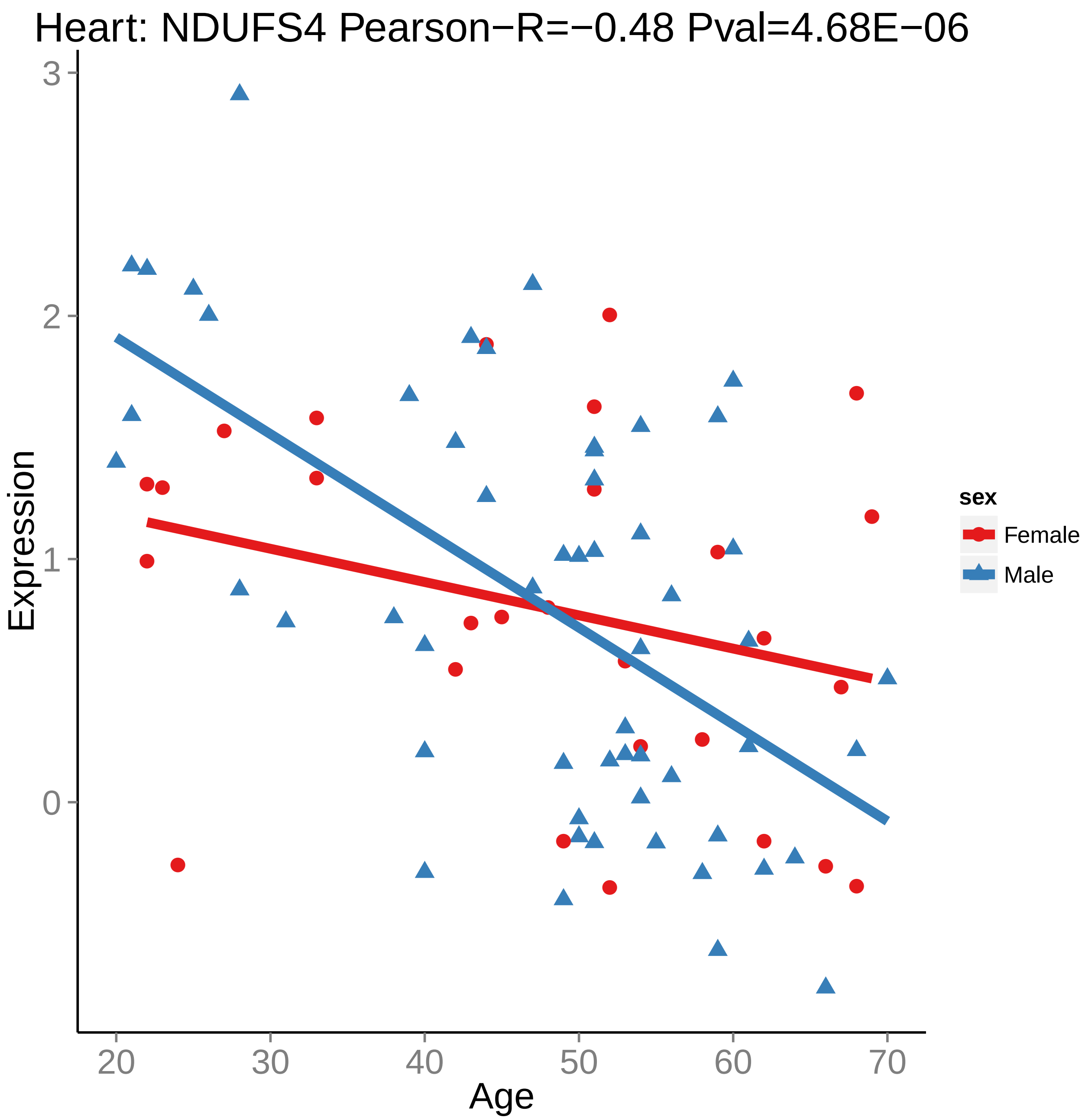

Heart: NDUFS4 Pearson−R=−0.48 Pval=4.68E−06
Expression
Age
0
1
2
3
20
30
40
50
60
70
sex
Female
Male

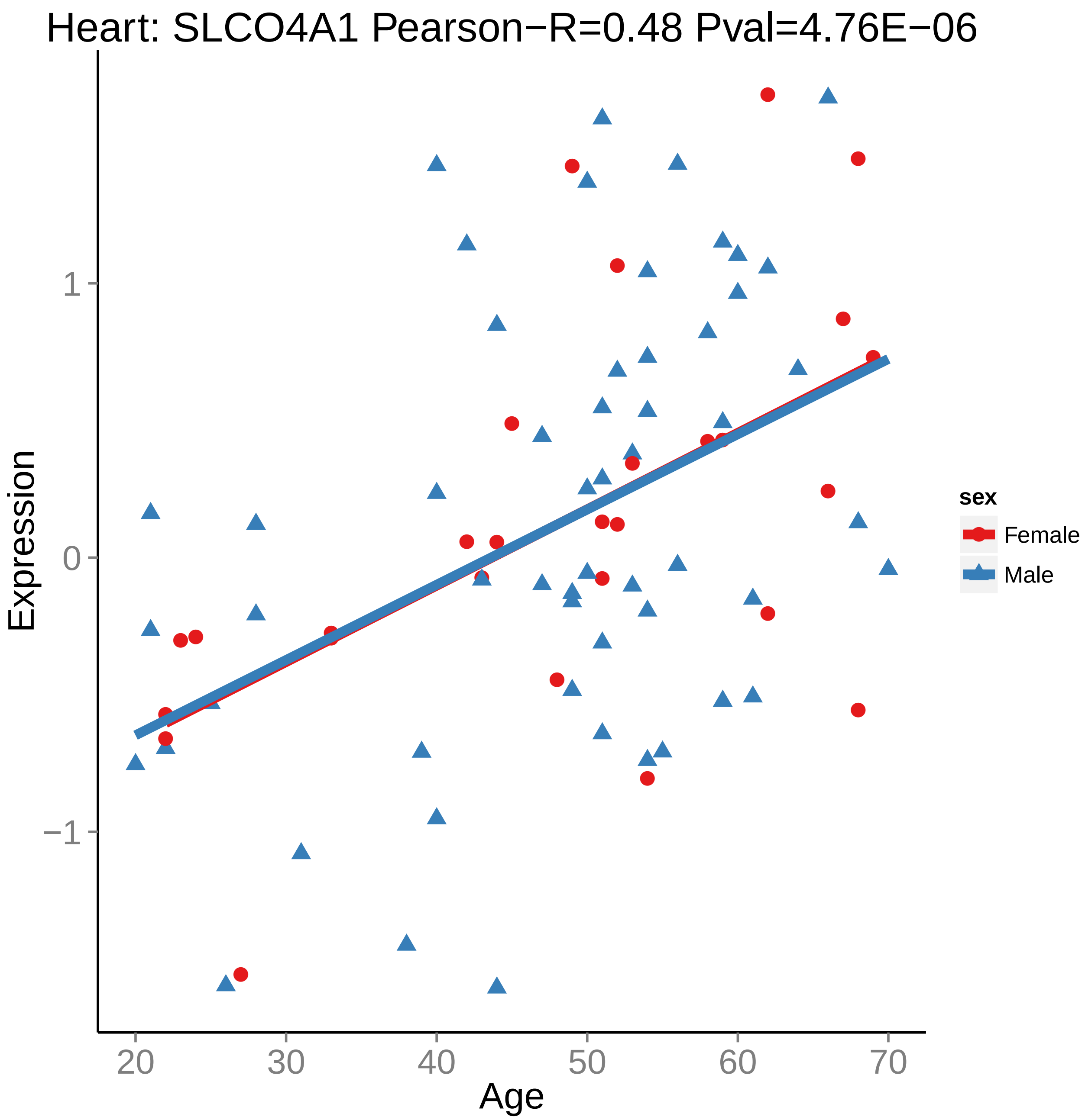

Heart: SLCO4A1 Pearson−R=0.48 Pval=4.76E−06
Expression
1
0
−1
20
30
40
50
60
70
Age
sex
Female
Male

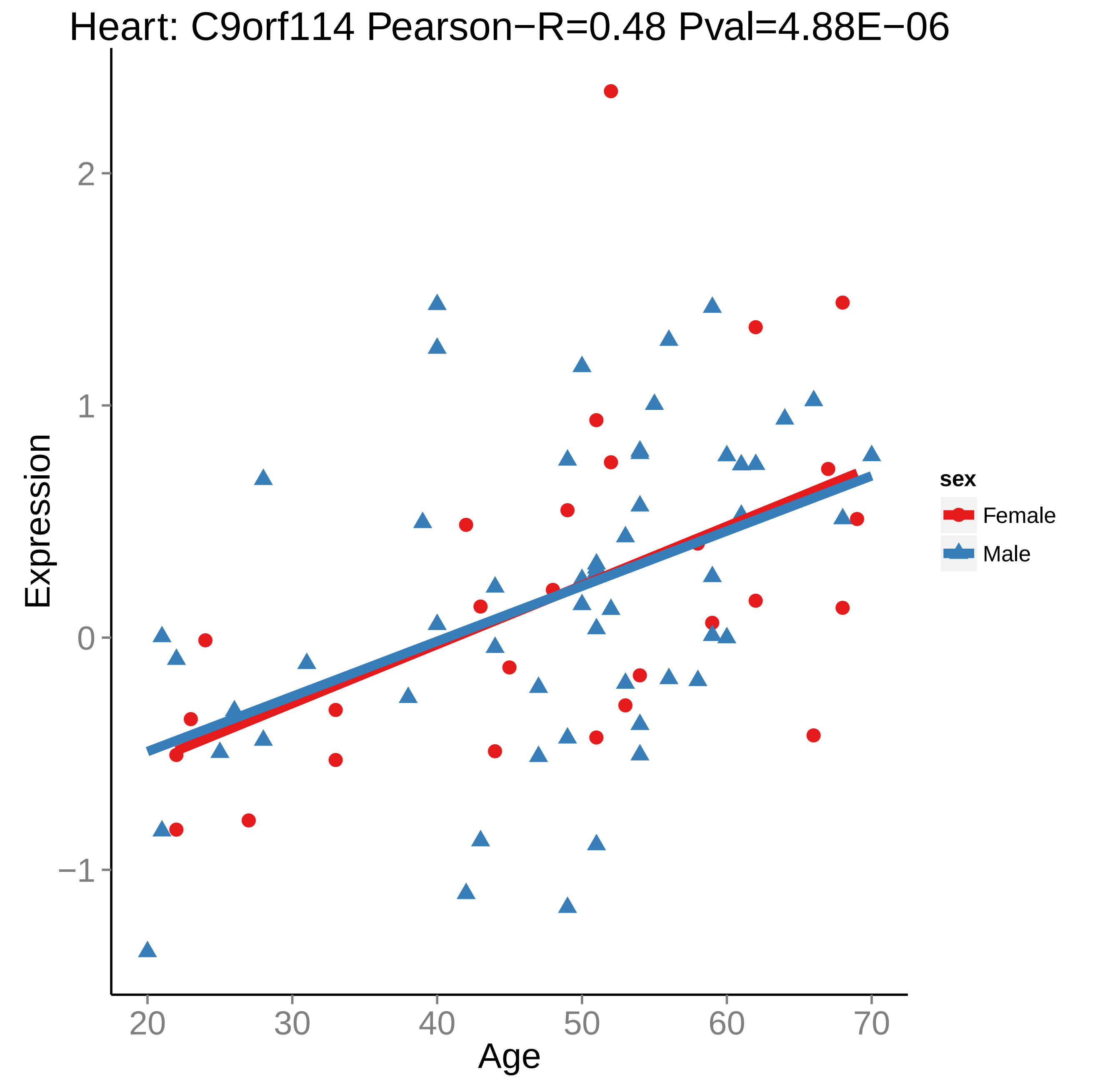

Heart: C9orf114 Pearson−R=0.48 Pval=4.88E−06
Expression
2
1
0
−1
20
30
40
50
60
70
Age
sex
Female
Male

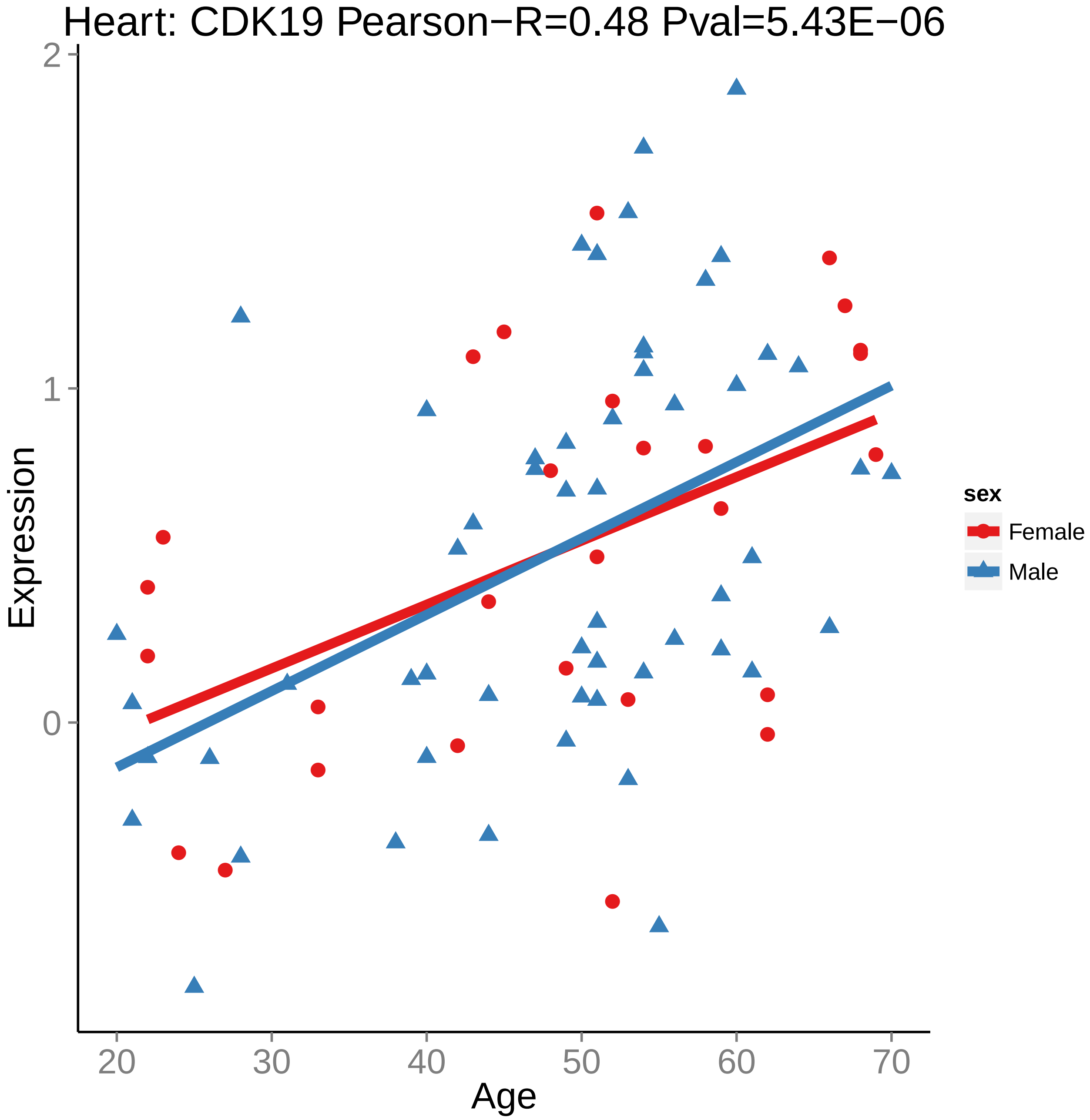

Heart: CDK19 Pearson−R=0.48 Pval=5.43E−06
2
1
0
Expression
20
30
40
50
60
70
Age
sex
Female
Male

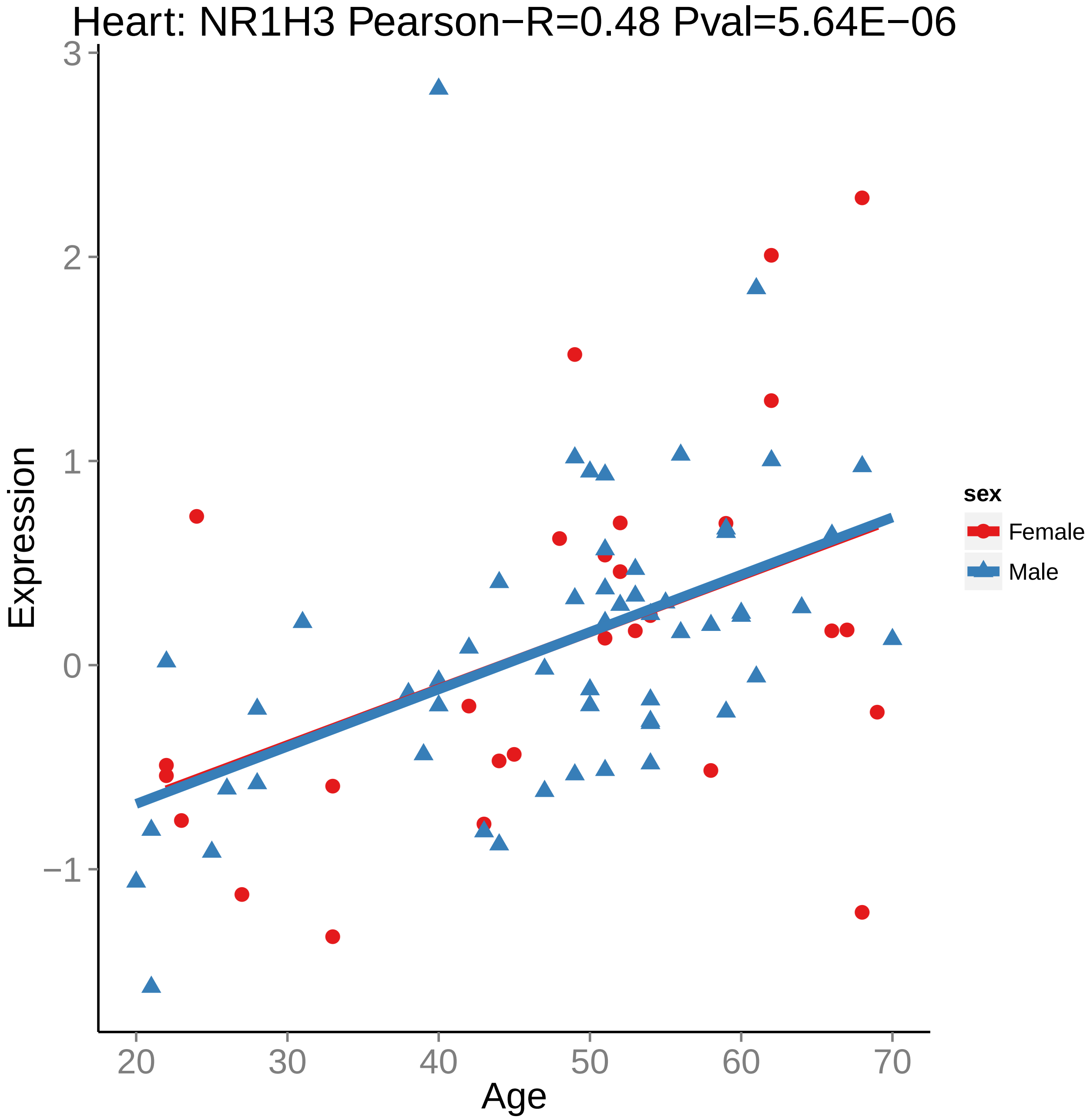

Heart: NR1H3 Pearson−R=0.48 Pval=5.64E−06
Expression
Age
3
2
1
0
−1
20
30
40
50
60
70
sex
Female
Male

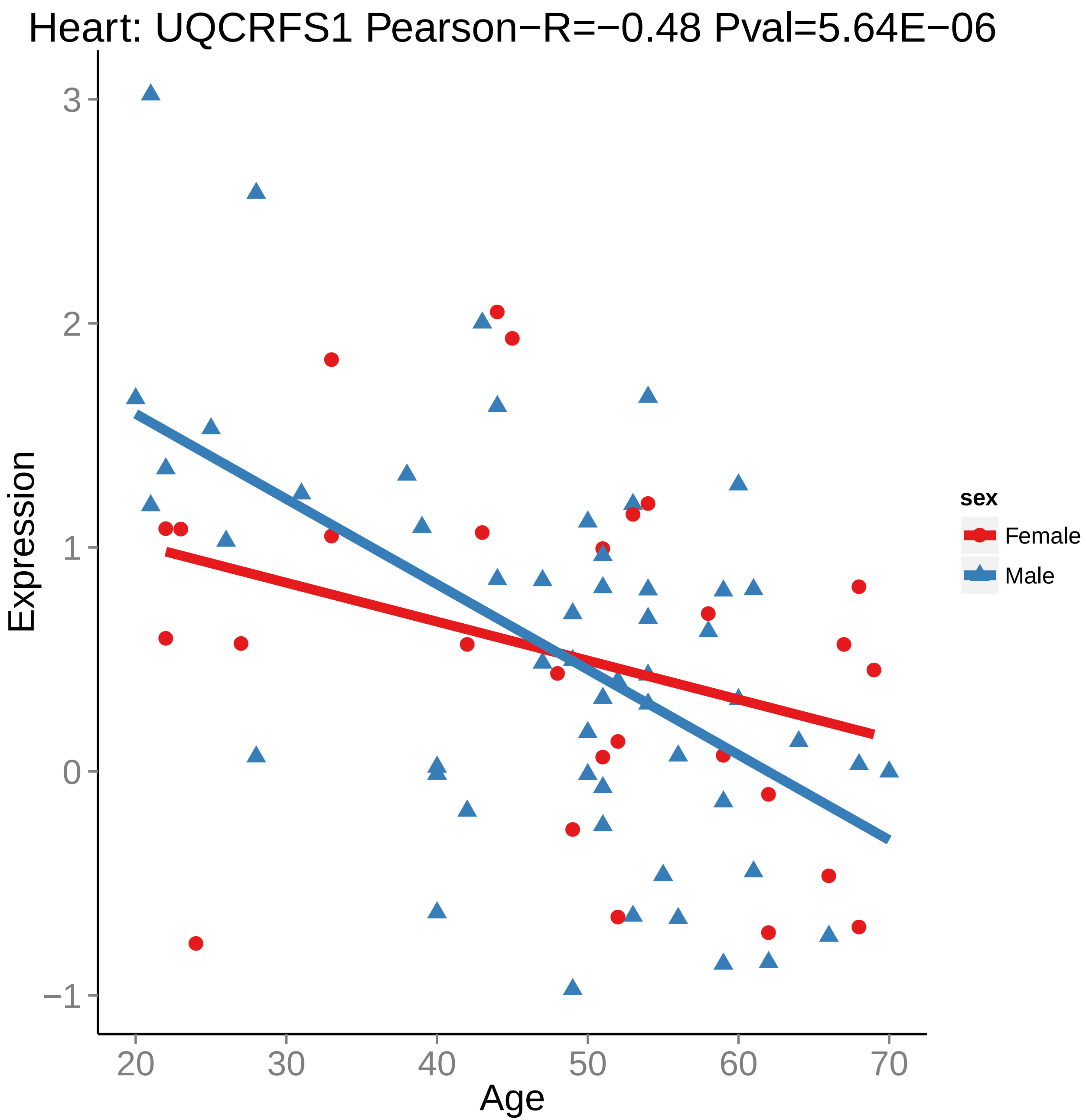

Heart: UQCRFS1 Pearson−R=−0.48 Pval=5.64E−06
Expression
3
2
1
0
−1
20
30
40
50
60
70
Age
sex
Female
Male

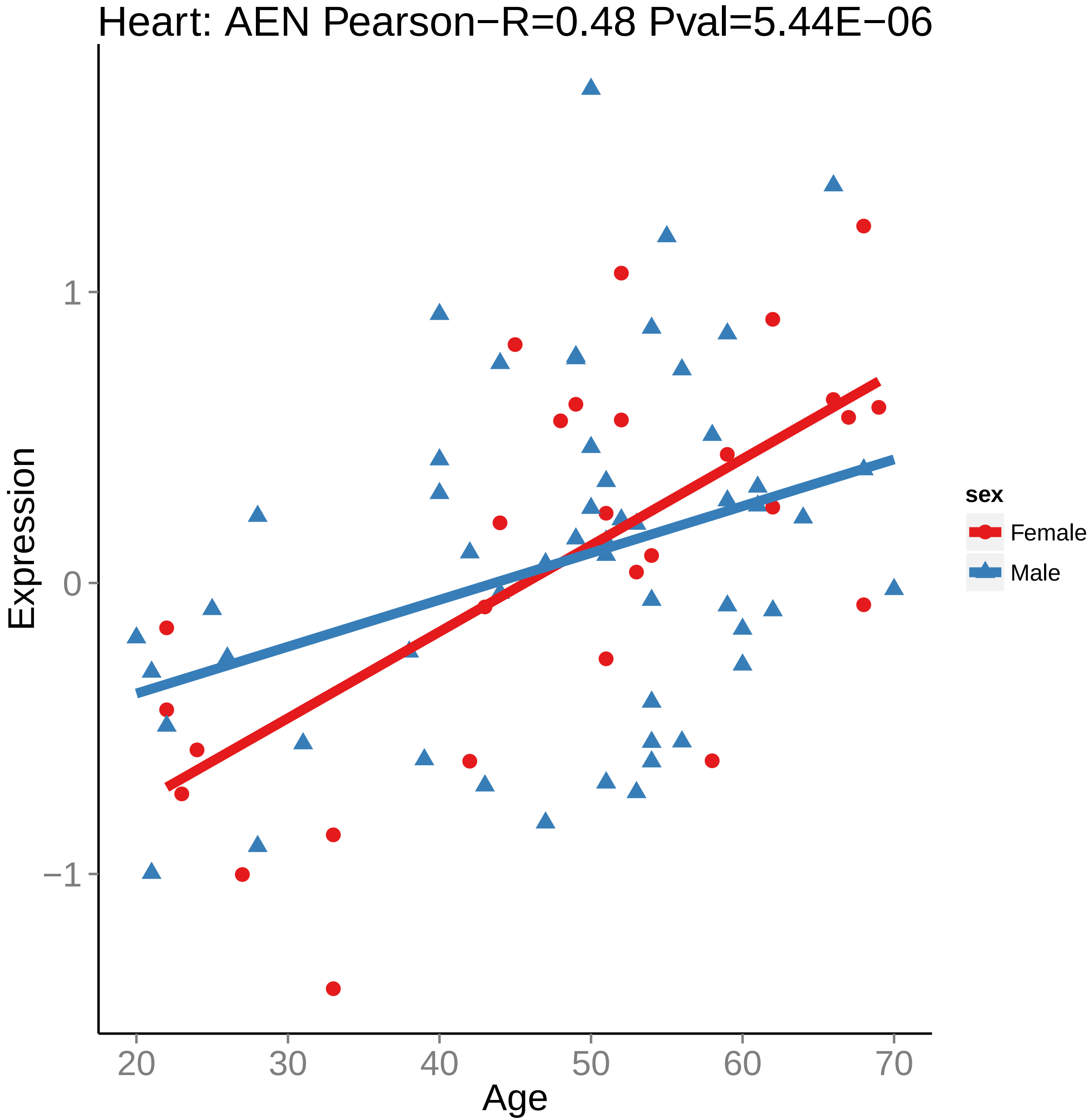
Heart: AEN Pearson−R=0.48 Pval=5.44E−06
Expression
1
0
−1
20
30
40
50
60
70
Age
sex
Female
Male

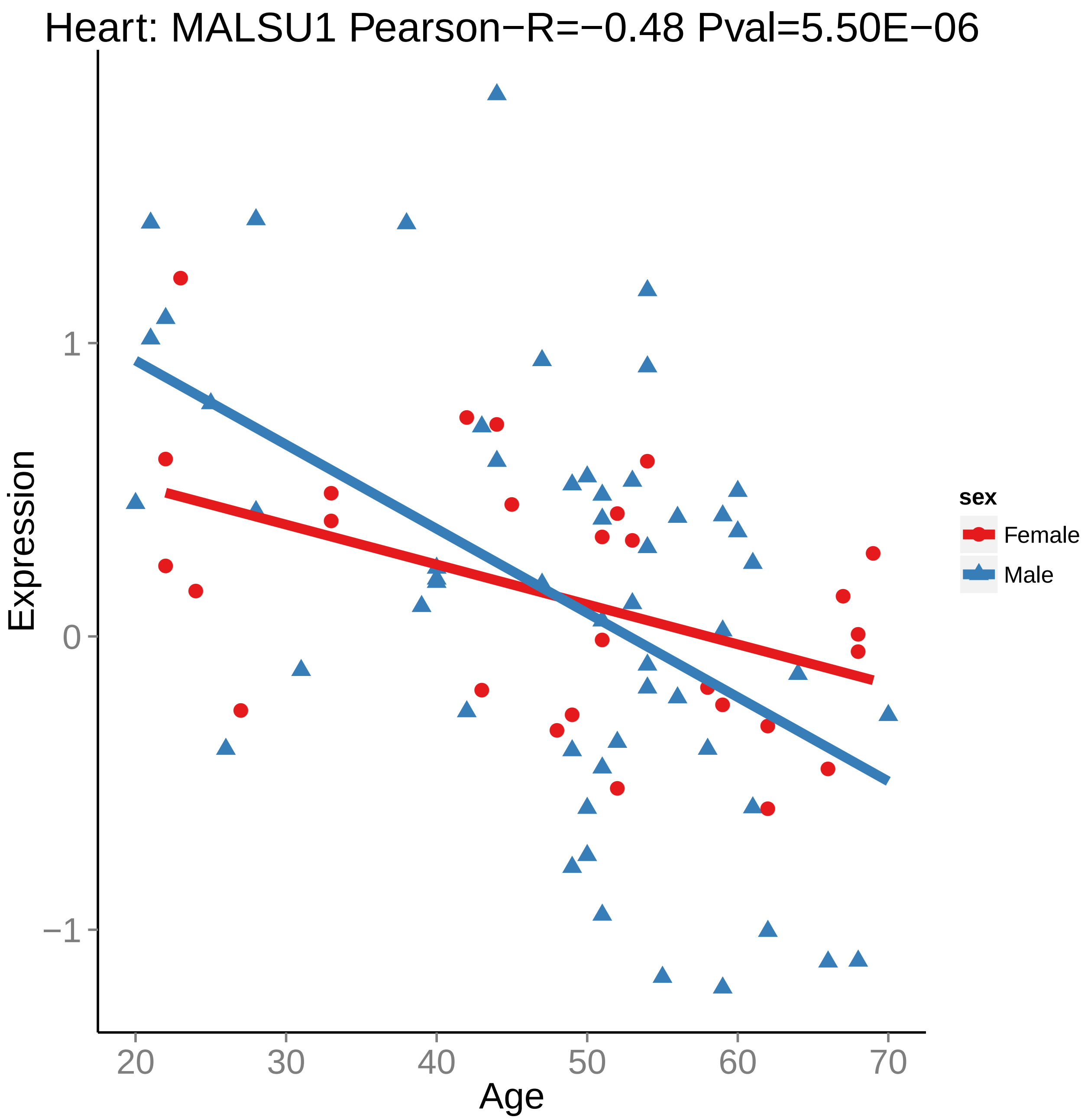
Heart: MALSU1 Pearson−R=−0.48 Pval=5.50E−06
Expression
1
0
−1
20
30
40
50
60
70
Age
sex
Female
Male

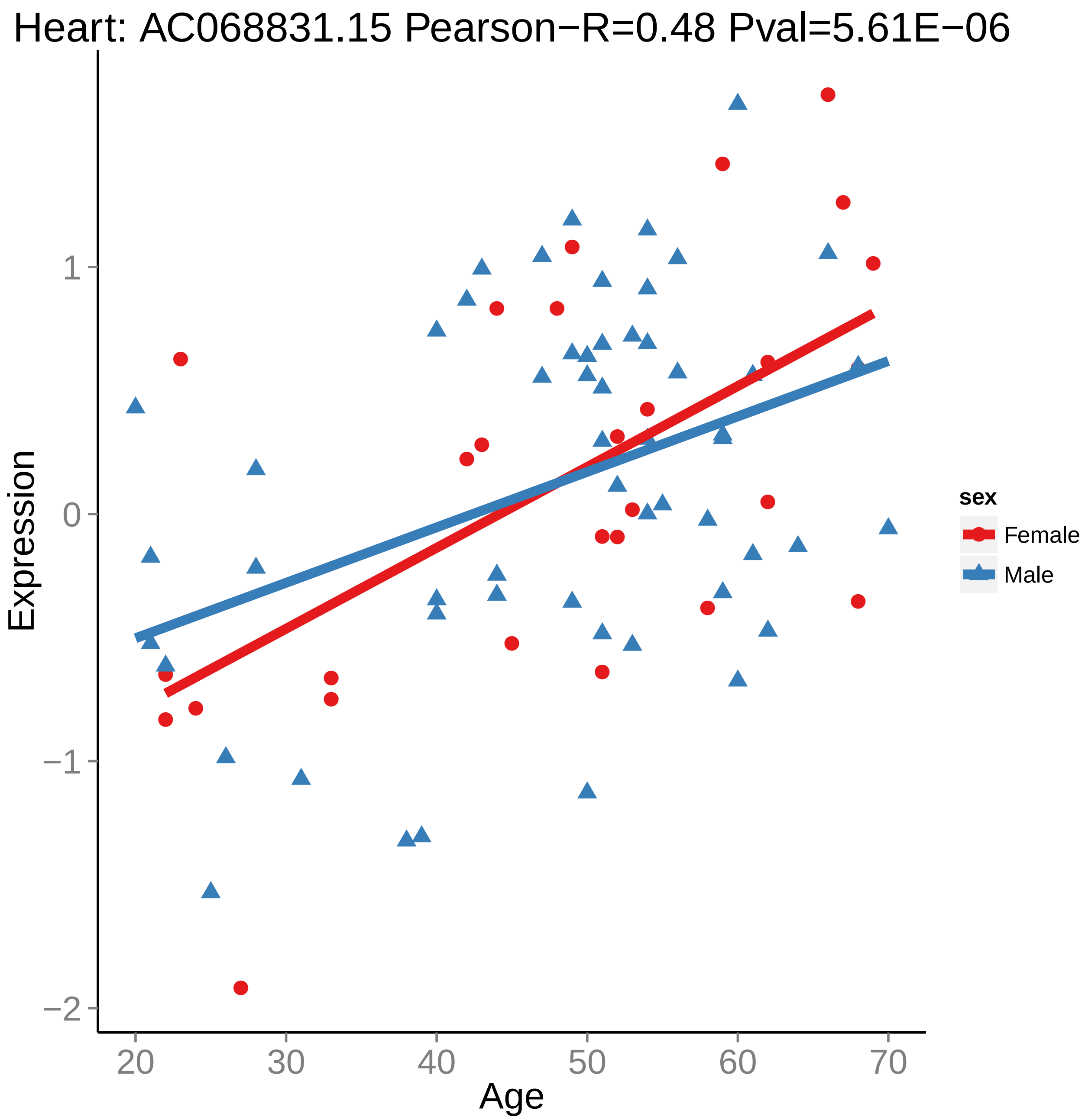

Heart: AC068831.15 Pearson−R=0.48 Pval=5.61E−06
Expression
1
0
−1
−2
20
30
40
50
60
70
Age
sex
Female
Male

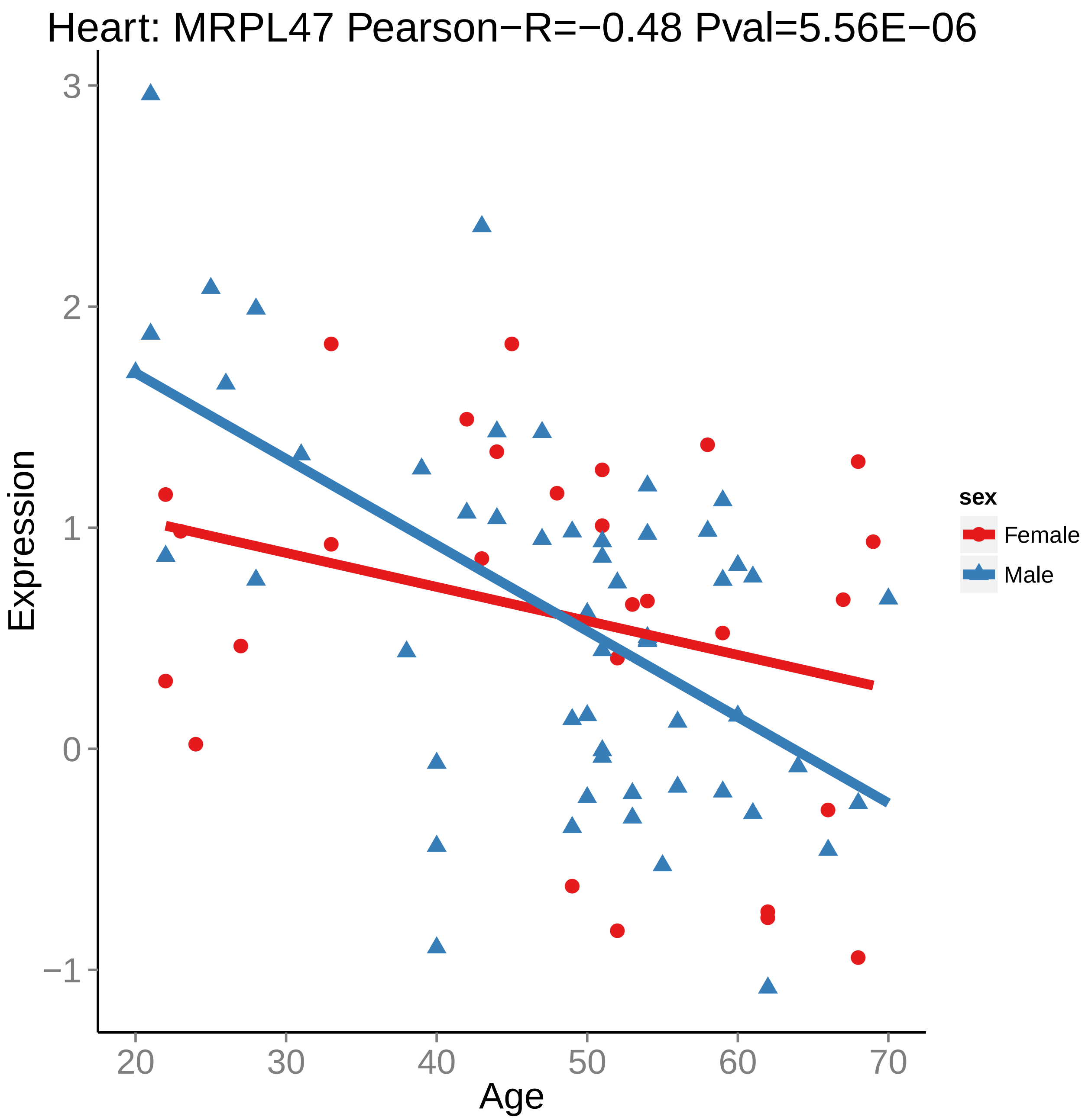

Heart: MRPL47 Pearson−R=−0.48 Pval=5.56E−06
Expression
3
2
1
0
−1
Age
20
30
40
50
60
70
sex
Female
Male

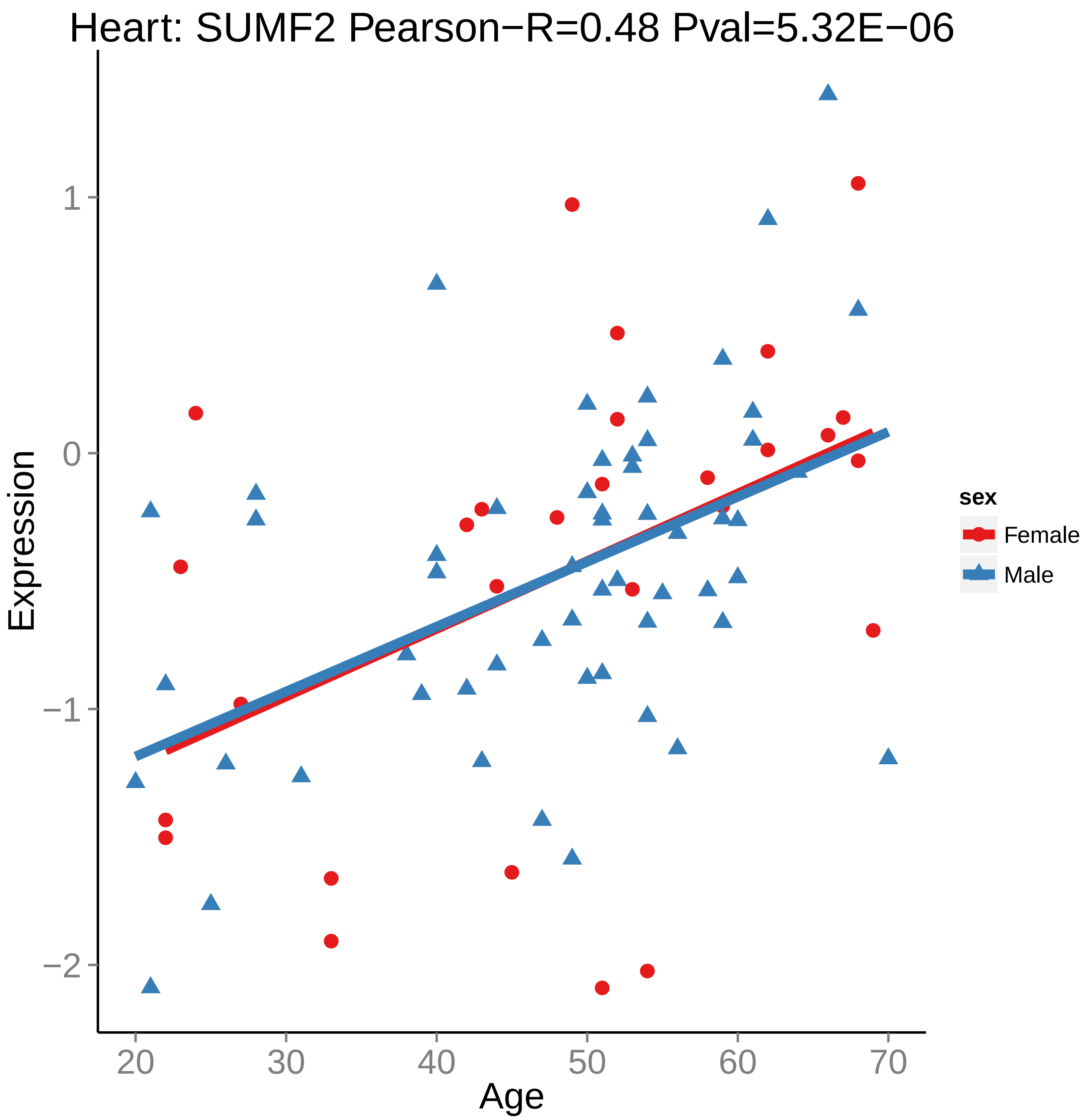

Heart: SUMF2 Pearson−R=0.48 Pval=5.32E−06
Expression
1
0
−1
−2
20
30
40
50
60
70
Age
sex
Female
Male

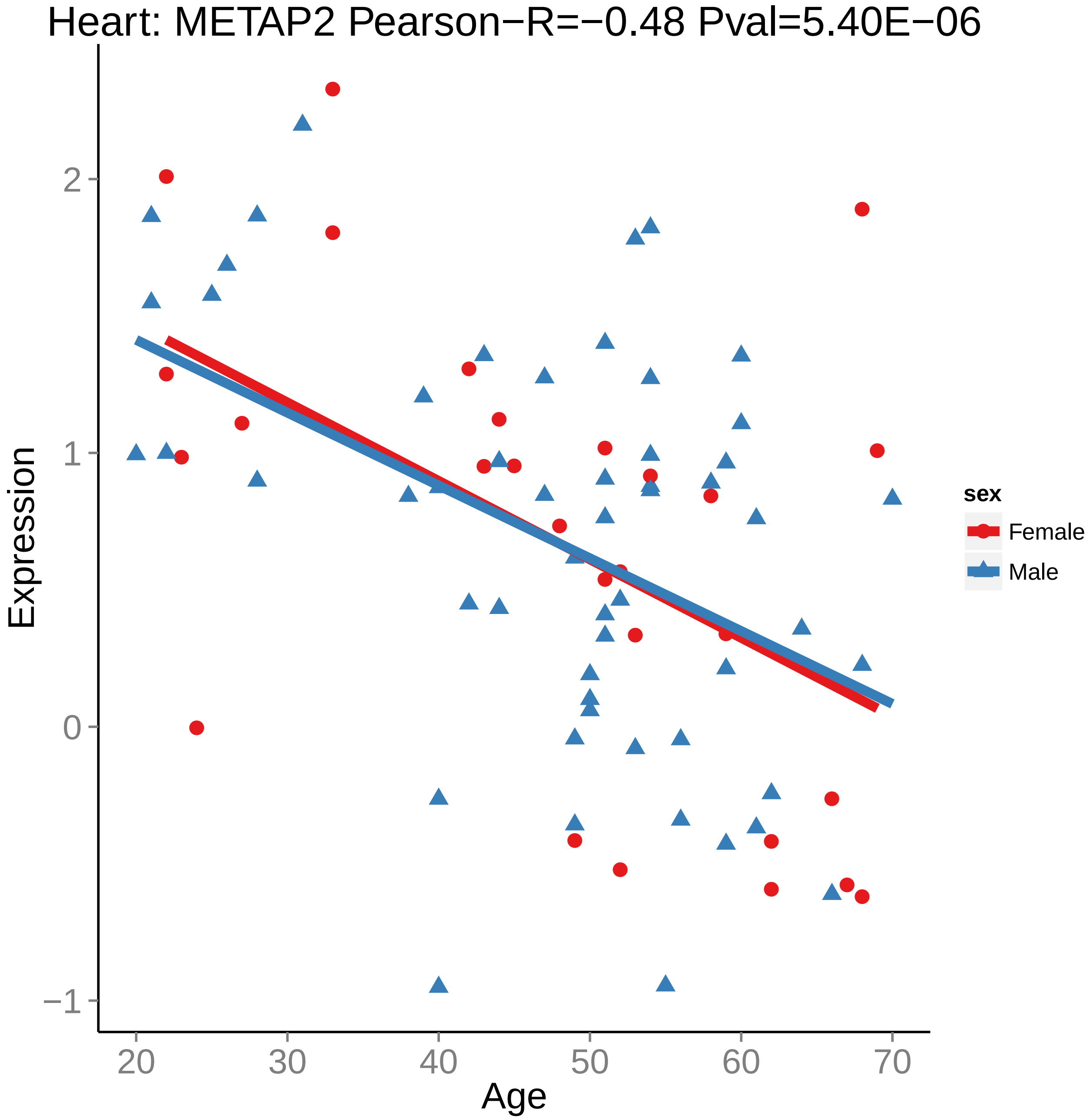
Heart: METAP2 Pearson−R=−0.48 Pval=5.40E−06
Expression
Age
sex
Female
Male
2
1
0
−1
20
30
40
50
60
70

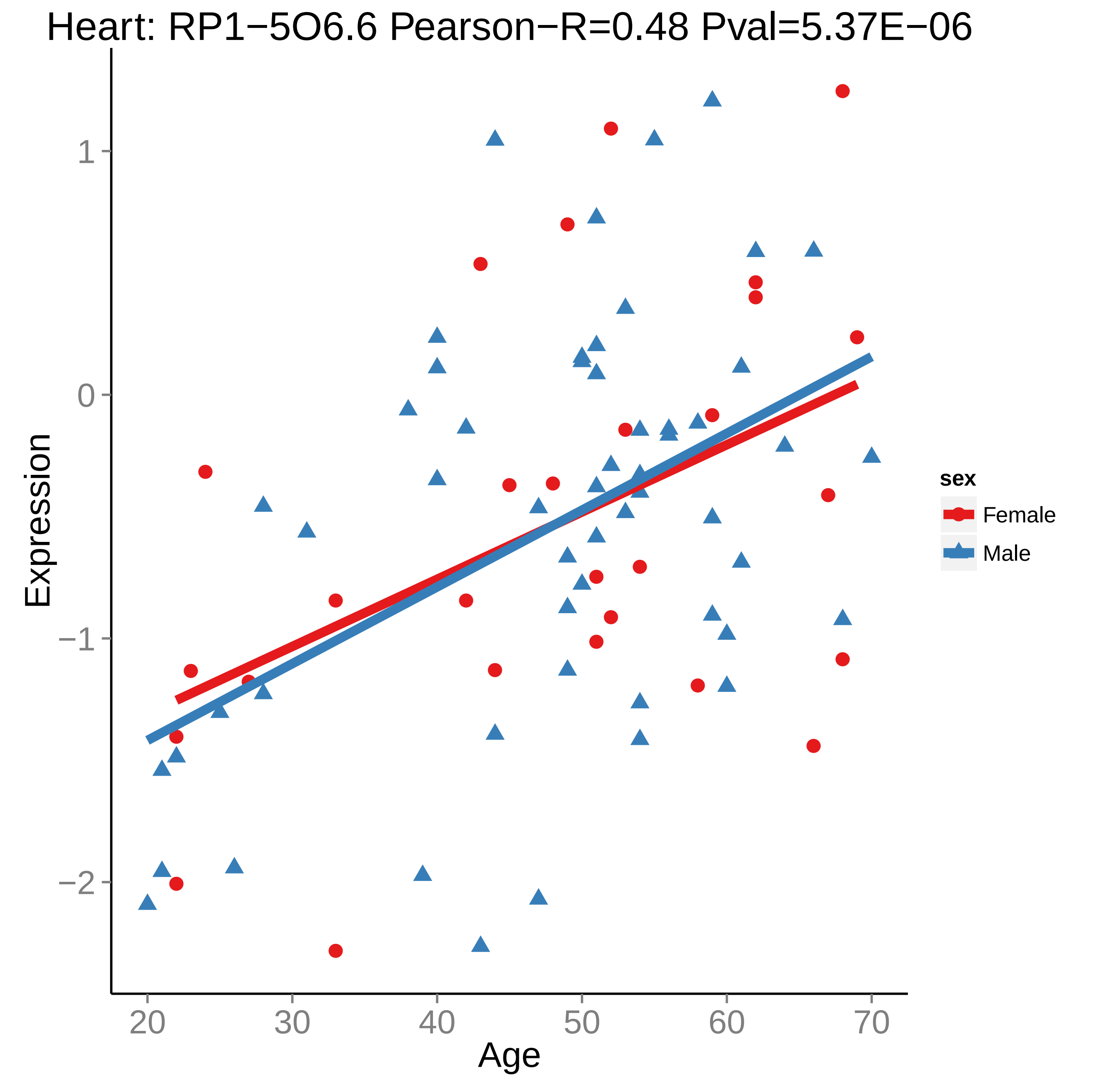

Heart: RP1−5O6.6 Pearson−R=0.48 Pval=5.37E−06
Expression
1
0
−1
−2
20
30
40
50
60
70
Age
sex
Female
Male

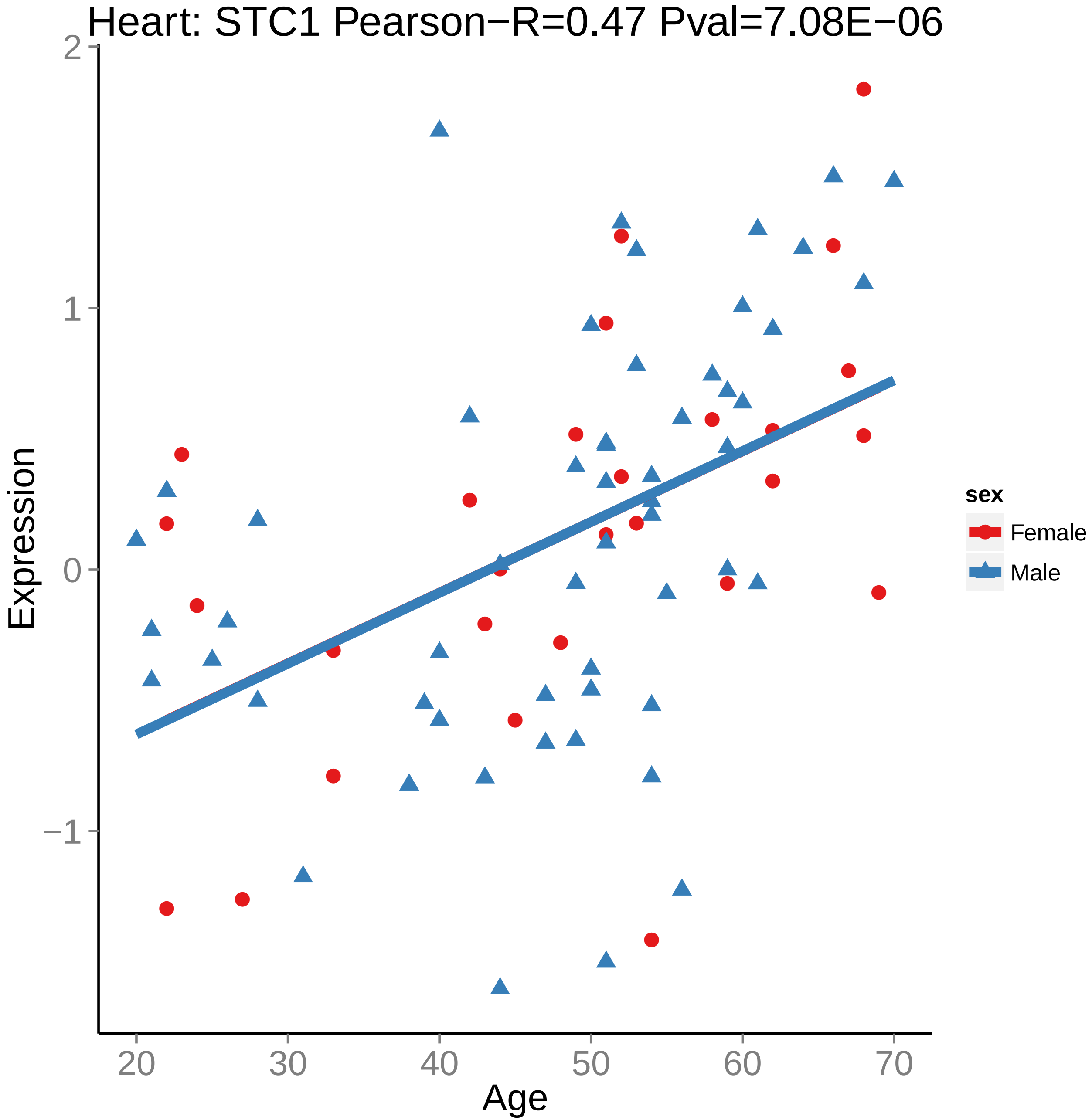
Heart: STC1 Pearson−R=0.47 Pval=7.08E−06
Expression
Age
2
1
0
−1
20
30
40
50
60
70
sex
Female
Male

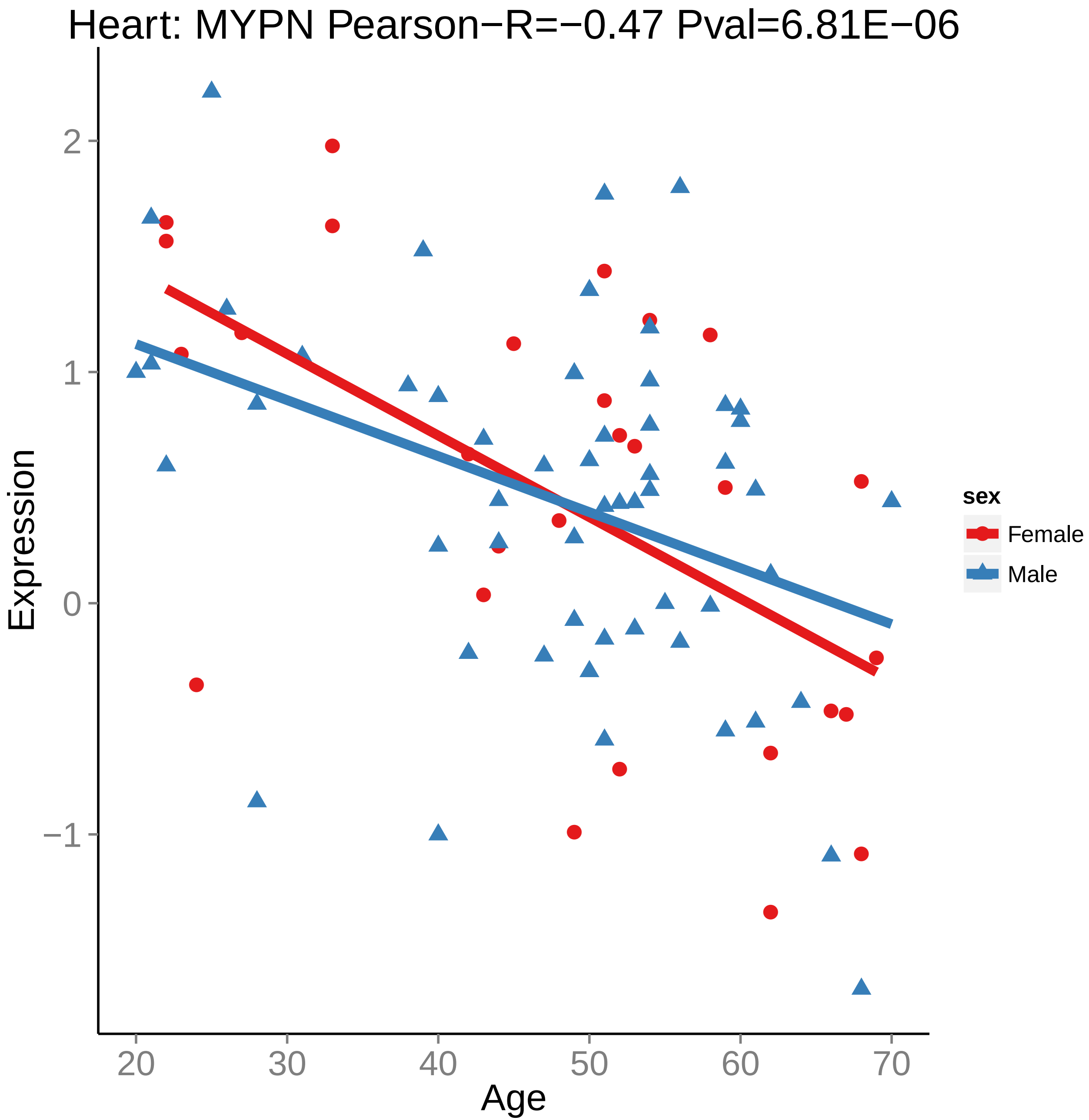

Heart: MYPN Pearson−R=−0.47 Pval=6.81E−06
Expression
2
1
0
−1
20
30
40
50
60
70
Age
sex
Female
Male

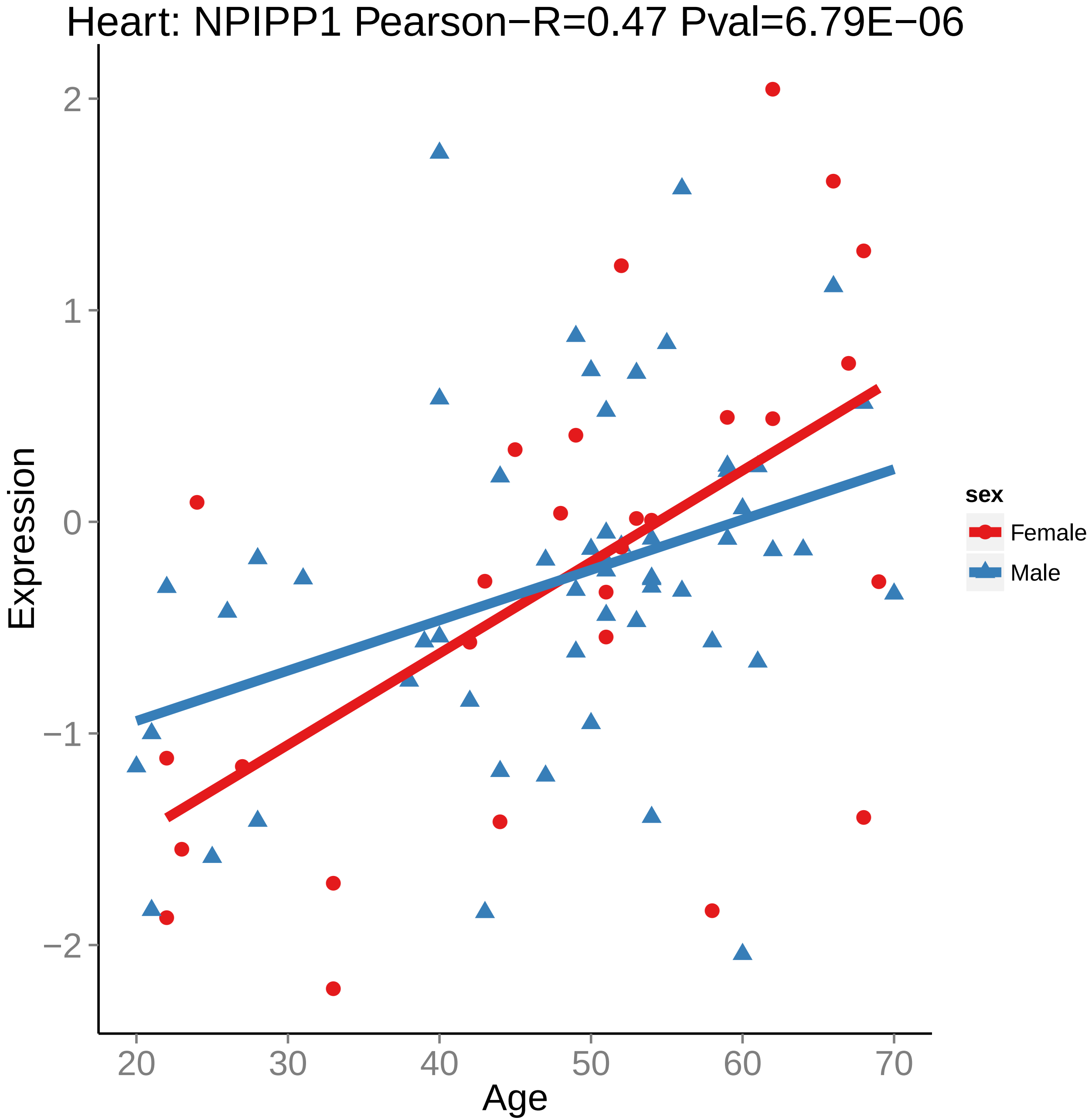
Heart: NPIPP1 Pearson−R=0.47 Pval=6.79E−06
Expression
Age
2
1
0
−1
−2
20
30
40
50
60
70
sex
Female
Male

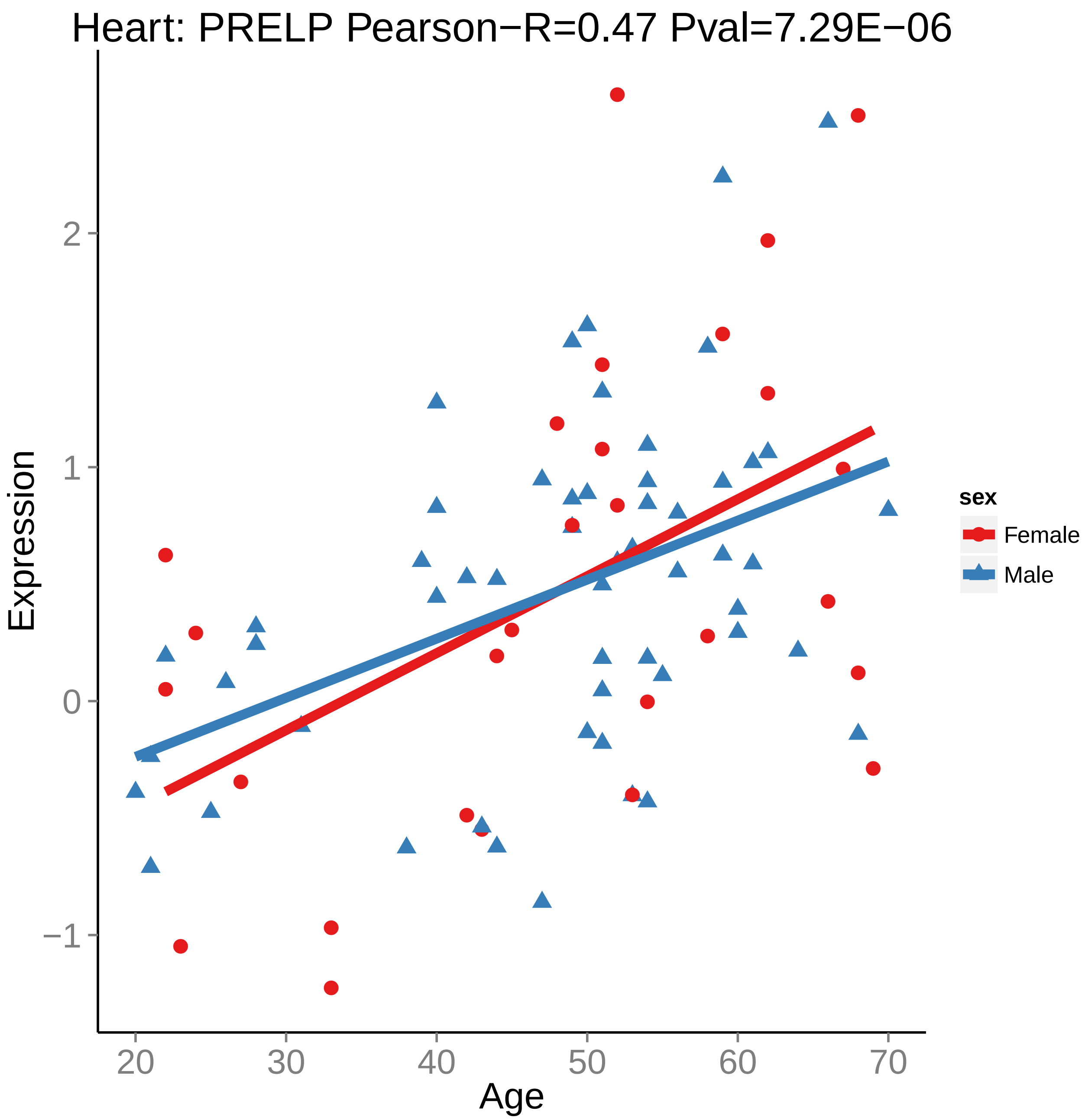
Heart: PRELP Pearson−R=0.47 Pval=7.29E−06
Expression
2
1
0
−1
20
30
40
50
60
70
Age
sex
Female
Male

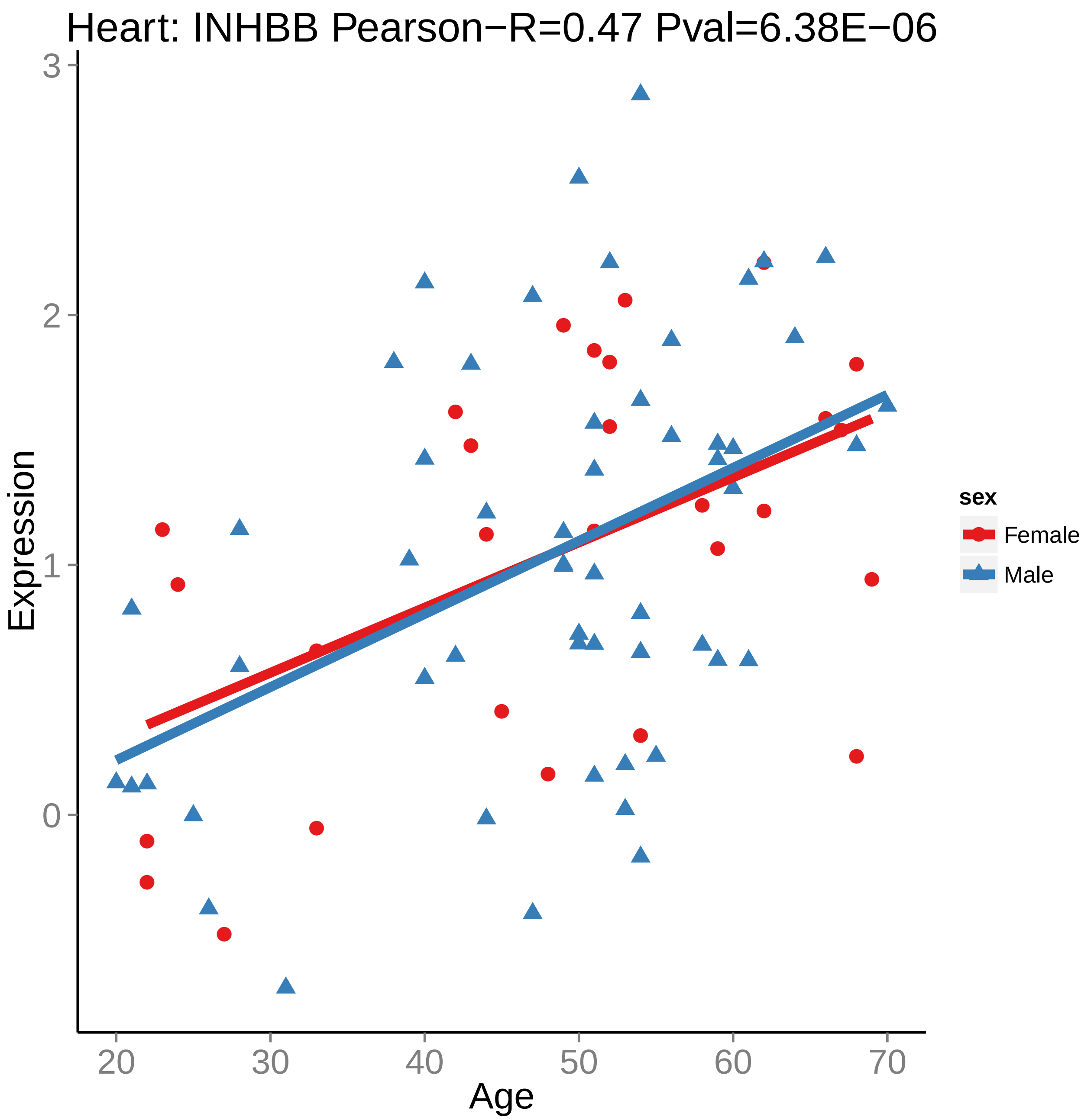

Heart: INHBB Pearson−R=0.47 Pval=6.38E−06
Expression
Age
3
2
1
0
20
30
40
50
60
70
sex
Female
Male

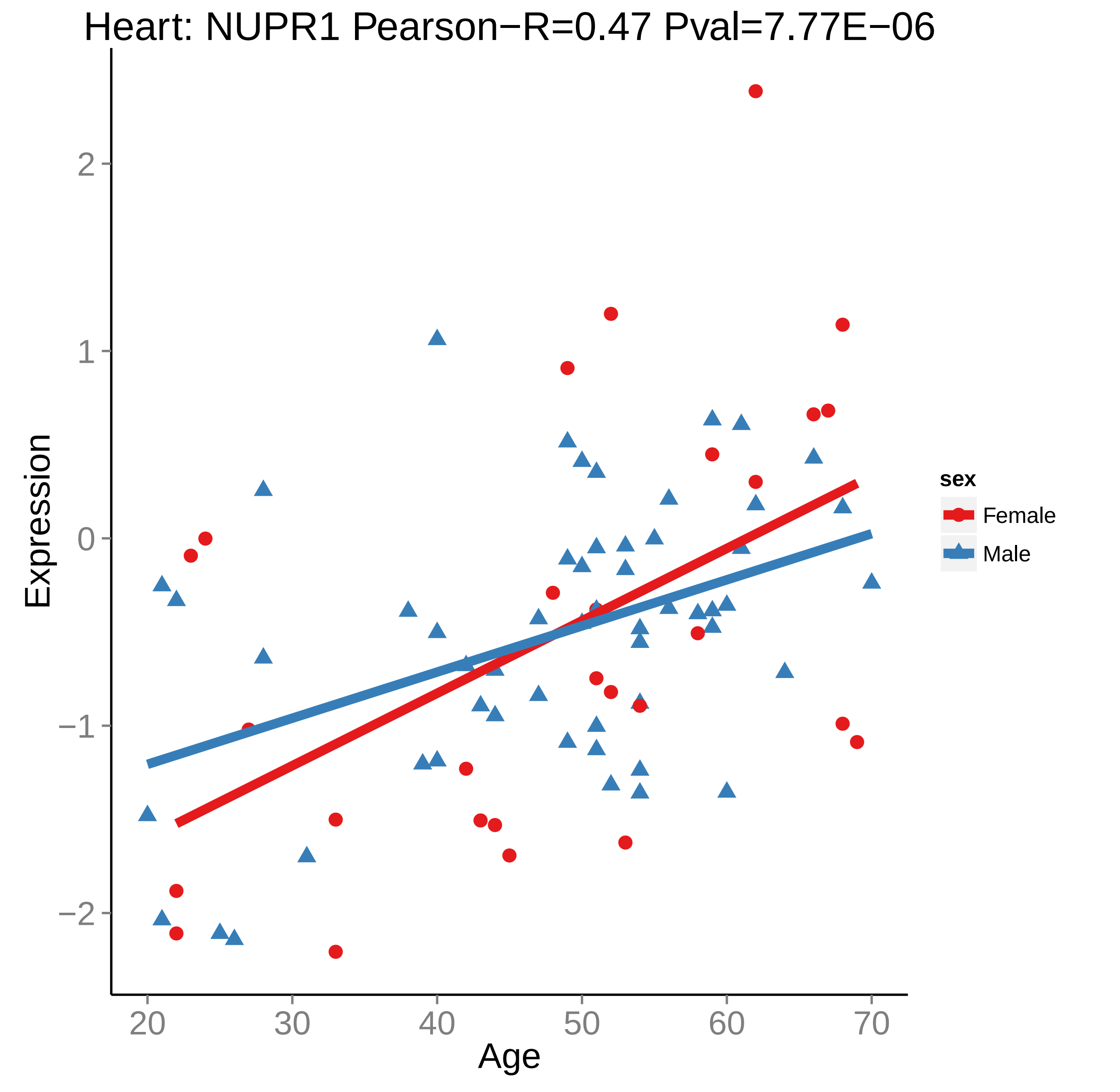

Heart: NUPR1 Pearson−R=0.47 Pval=7.77E−06
Expression
2
1
0
−1
−2
20
30
40
50
60
70
Age
sex
Female
Male

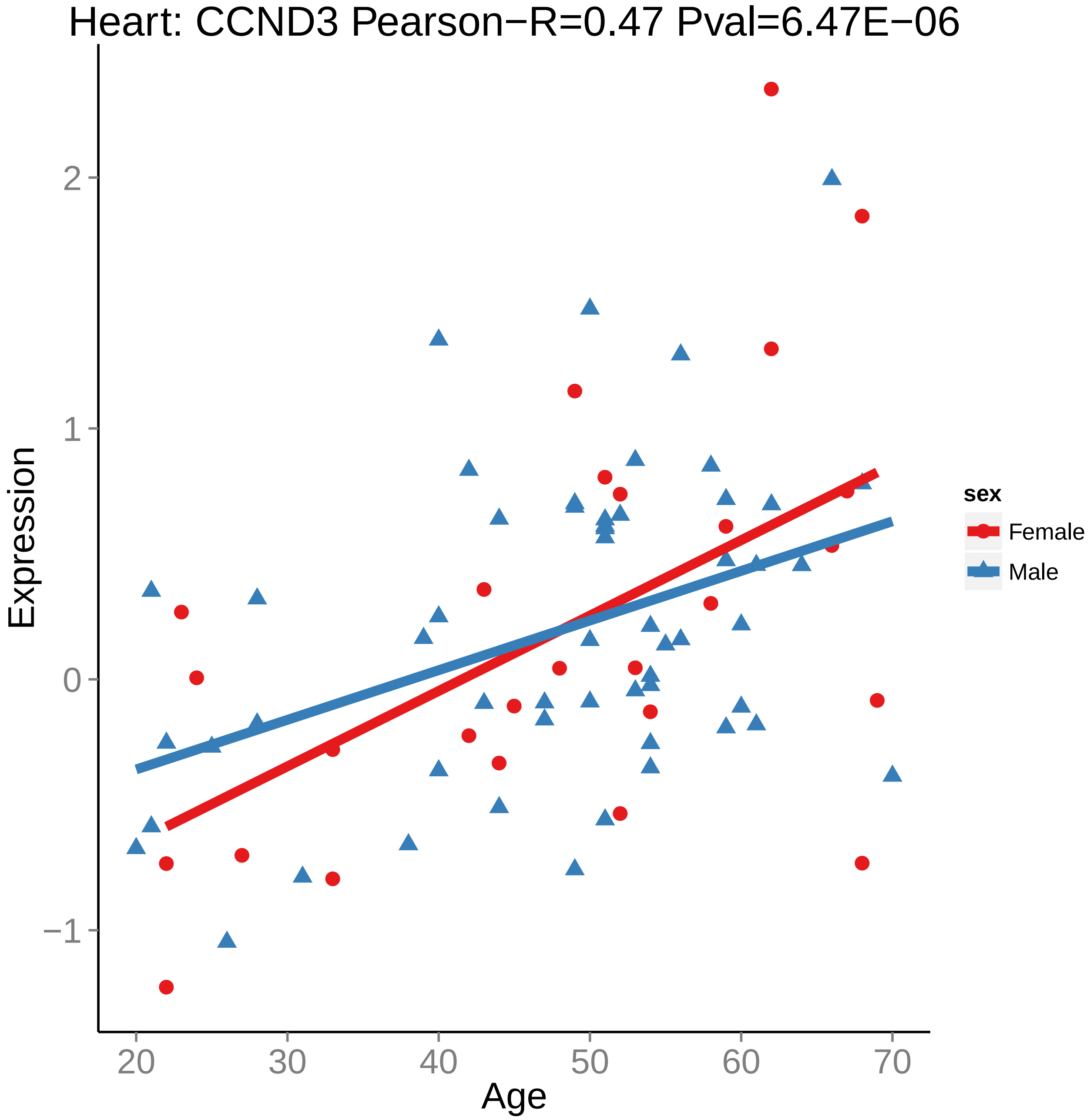

Heart: CCND3 Pearson−R=0.47 Pval=6.47E−06
Expression
2
1
0
−1
20
30
40
50
60
70
Age
sex
Female
Male

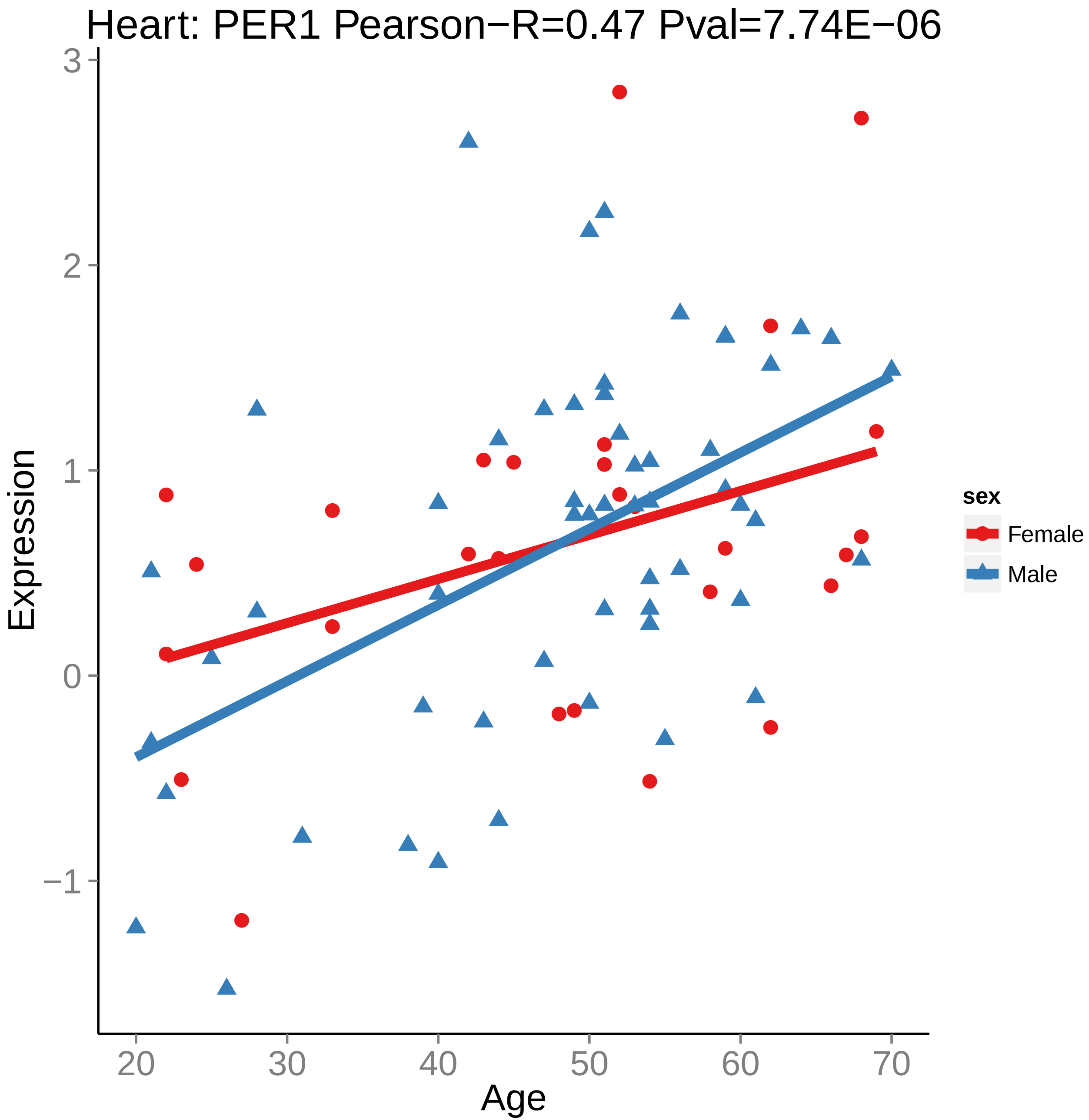
Heart: PER1 Pearson−R=0.47 Pval=7.74E−06
Expression
Age
3
2
1
0
−1
20
30
40
50
60
70
sex
Female
Male

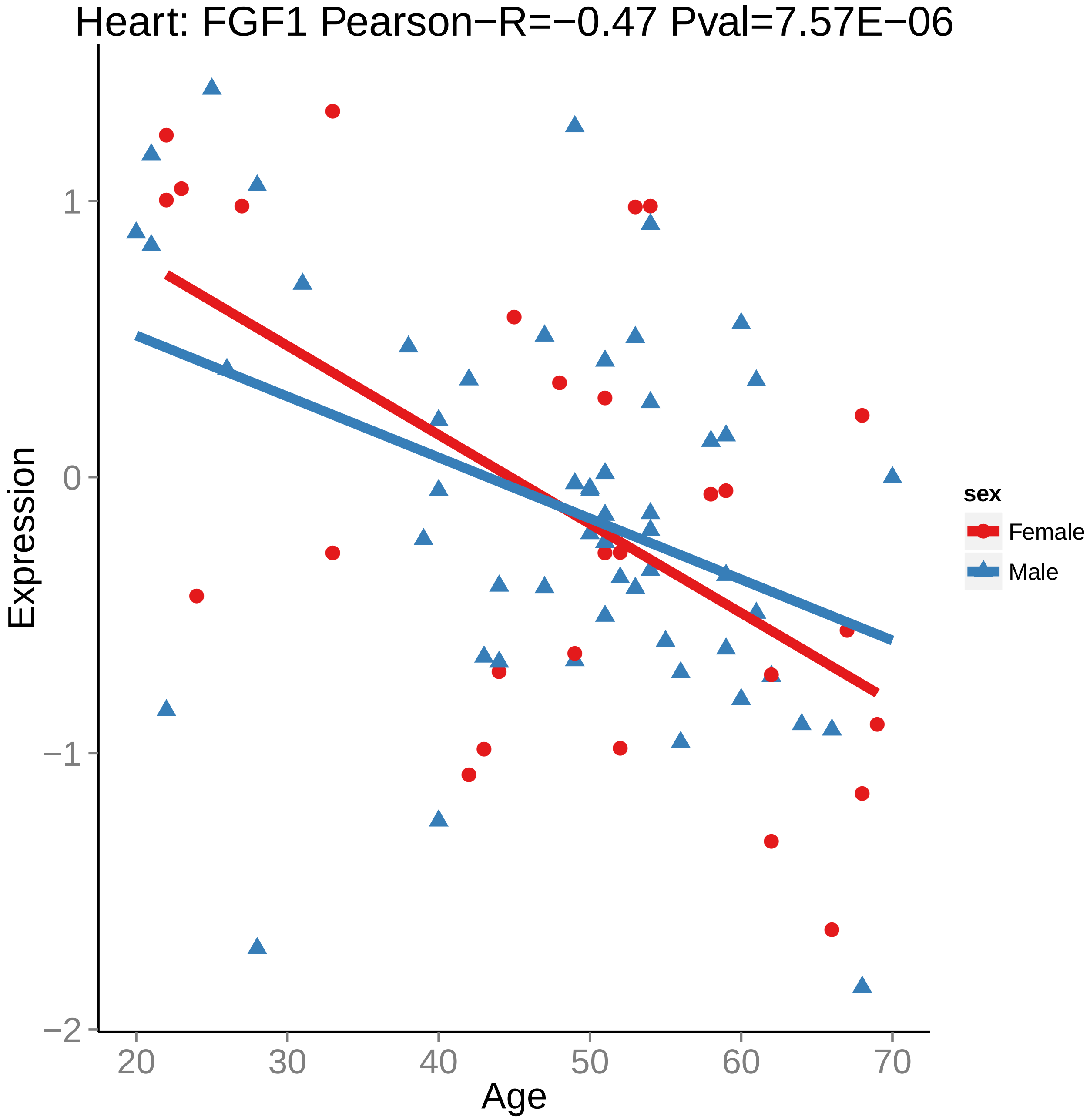

Heart: FGF1 Pearson−R=−0.47 Pval=7.57E−06
Expression
1
0
−1
−2
Age
20
30
40
50
60
70
sex
Female
Male

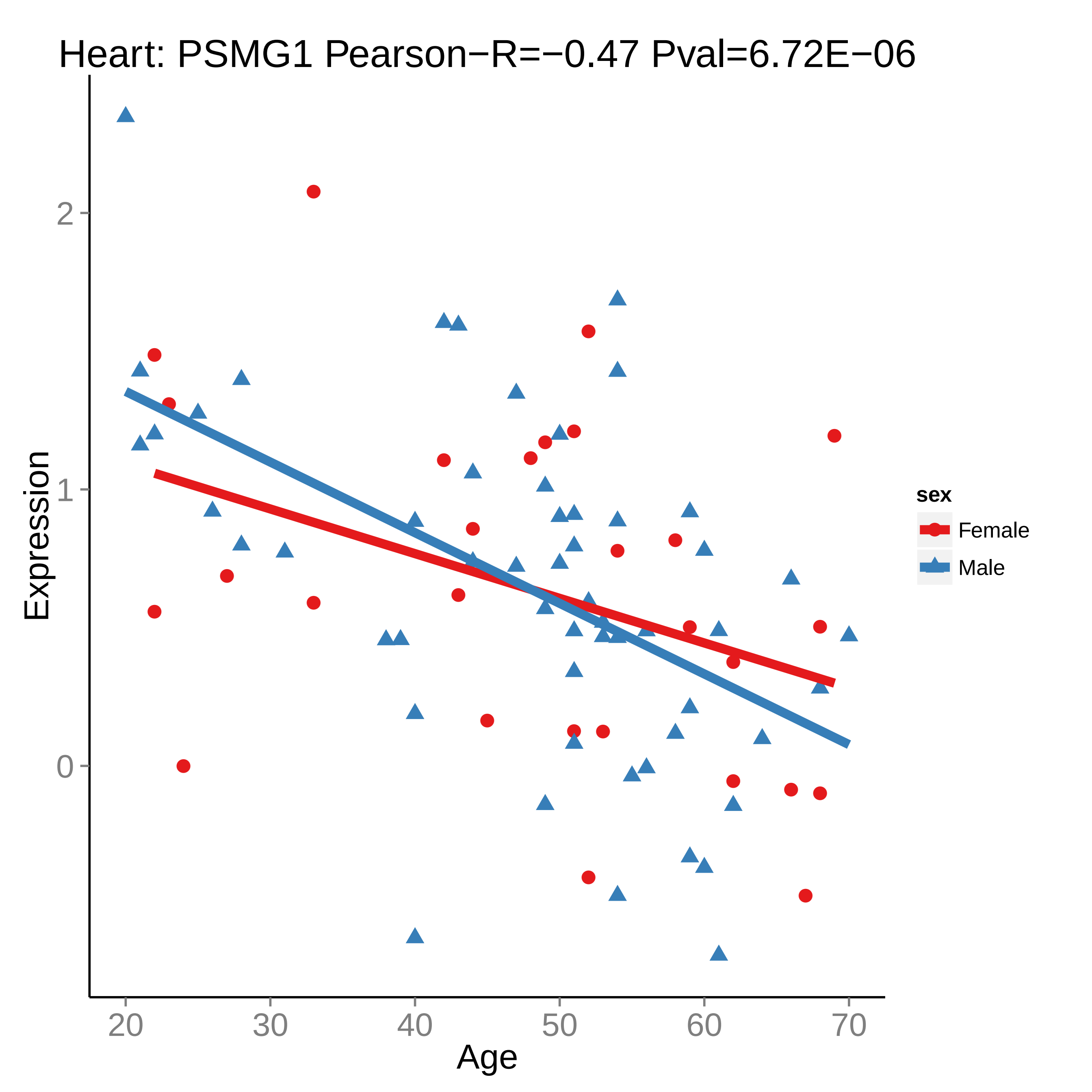
Heart: PSMG1 Pearson−R=−0.47 Pval=6.72E−06
Expression
Age
0
1
2
20
30
40
50
60
70
sex
Female
Male

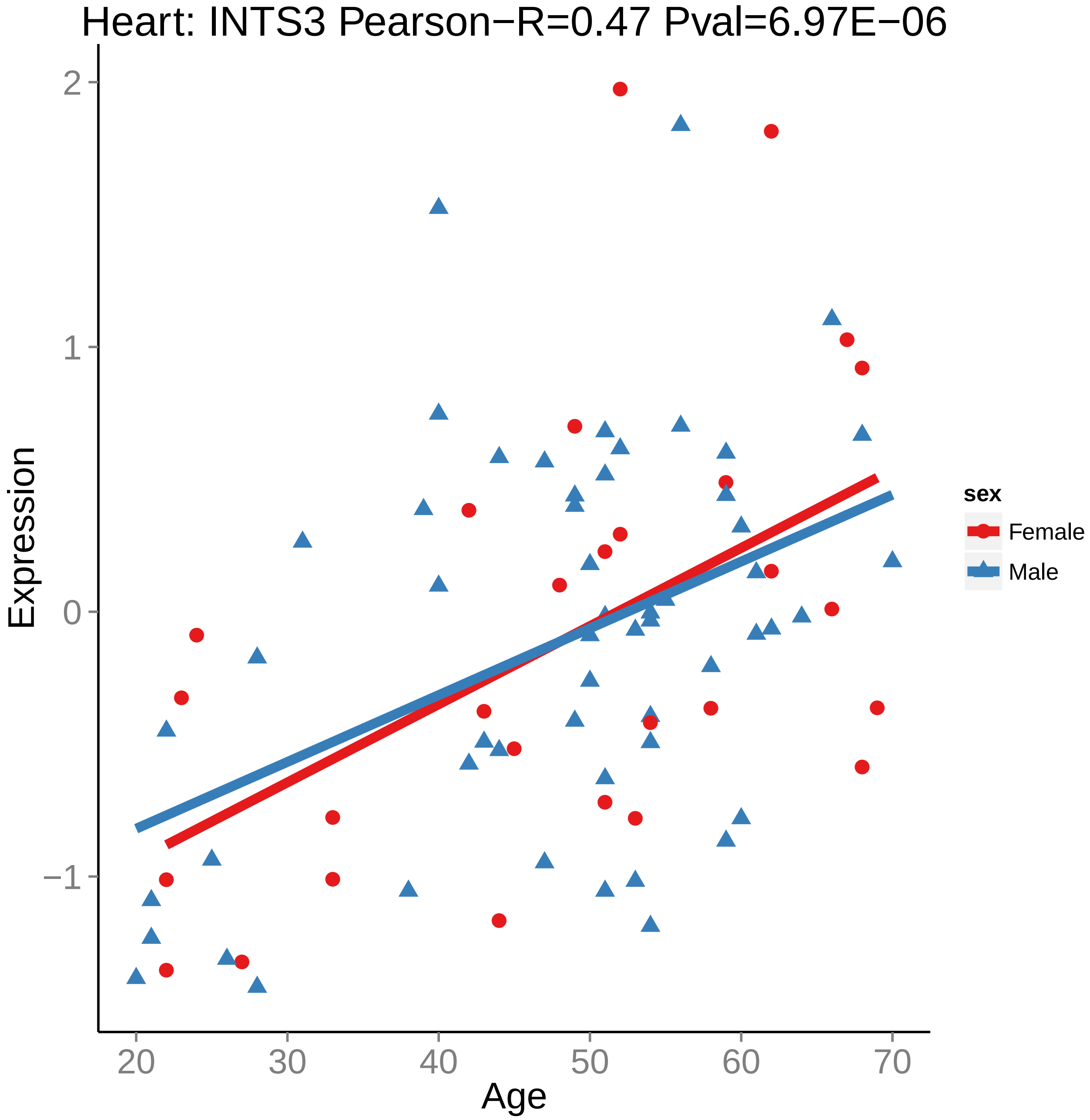

Heart: INTS3 Pearson−R=0.47 Pval=6.97E−06
Expression
Age
2
1
0
−1
20
30
40
50
60
70
sex
Female
Male

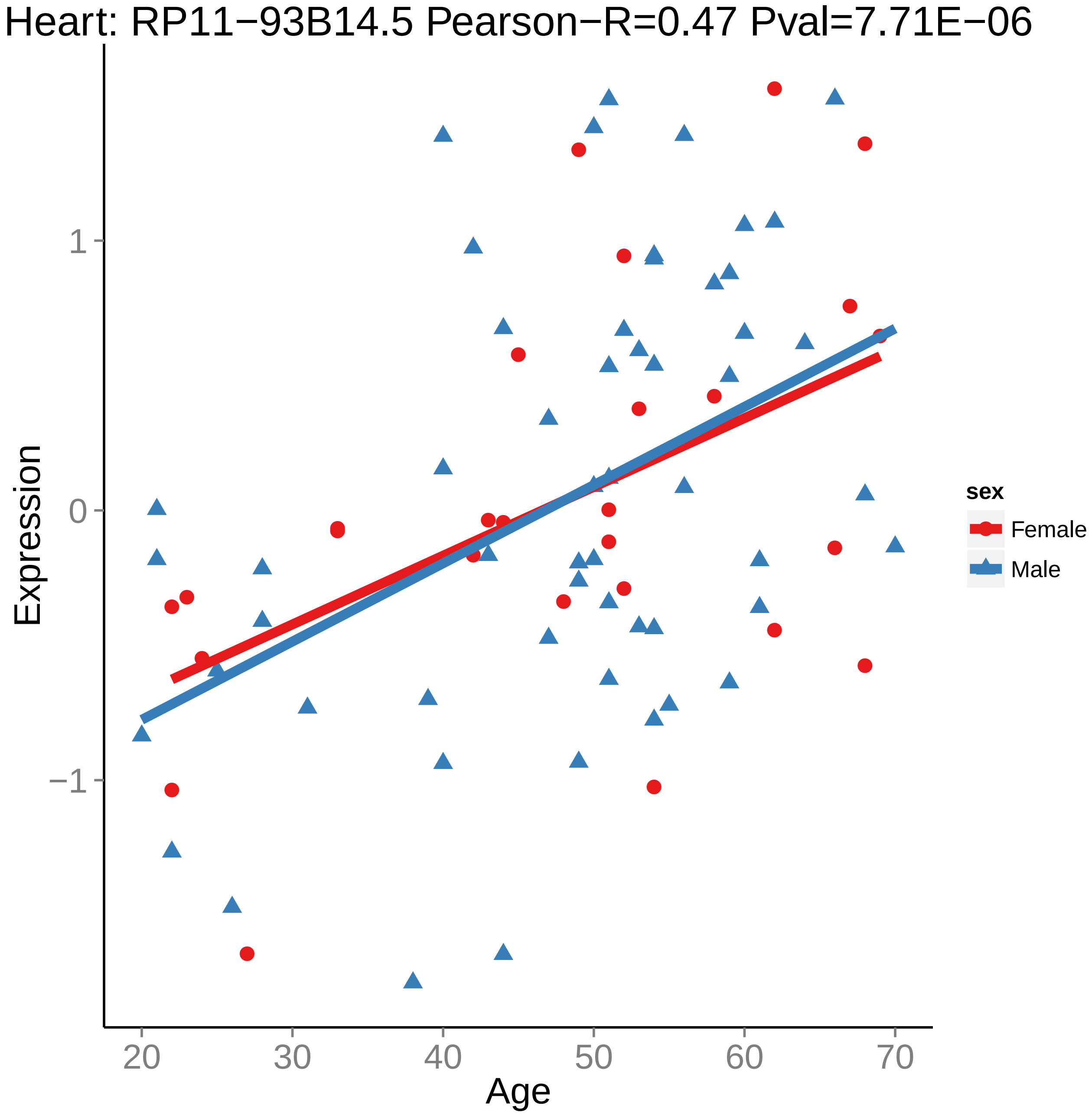

Heart: RP11−93B14.5 Pearson−R=0.47 Pval=7.71E−06
Expression
1
0
−1
20
30
40
50
60
70
Age
sex
Female
Male

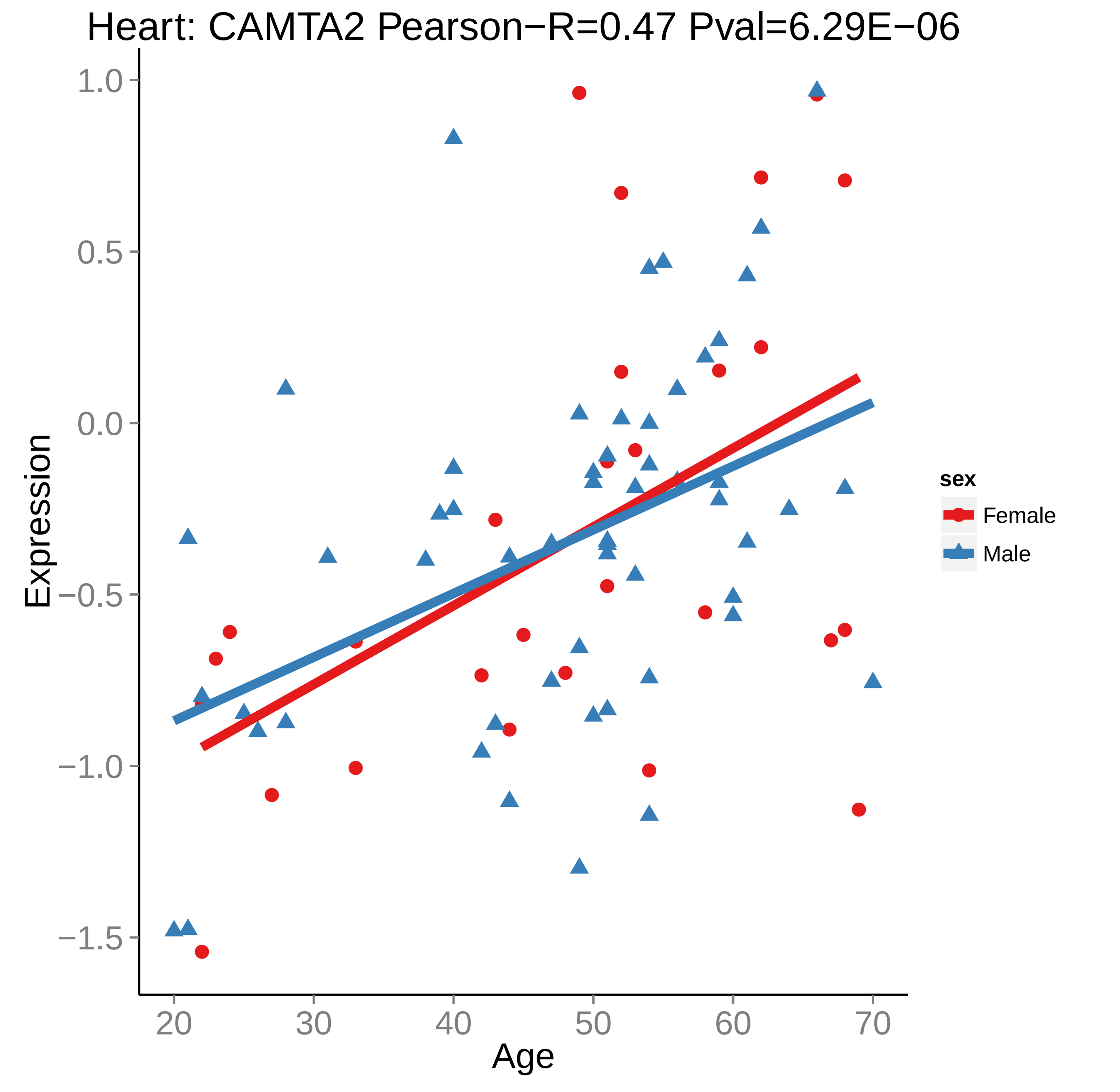

Heart: CAMTA2 Pearson−R=0.47 Pval=6.29E−06
Expression
1.0
0.5
0.0
−0.5
−1.0
−1.5
20
30
40
50
60
70
Age
sex
Female
Male

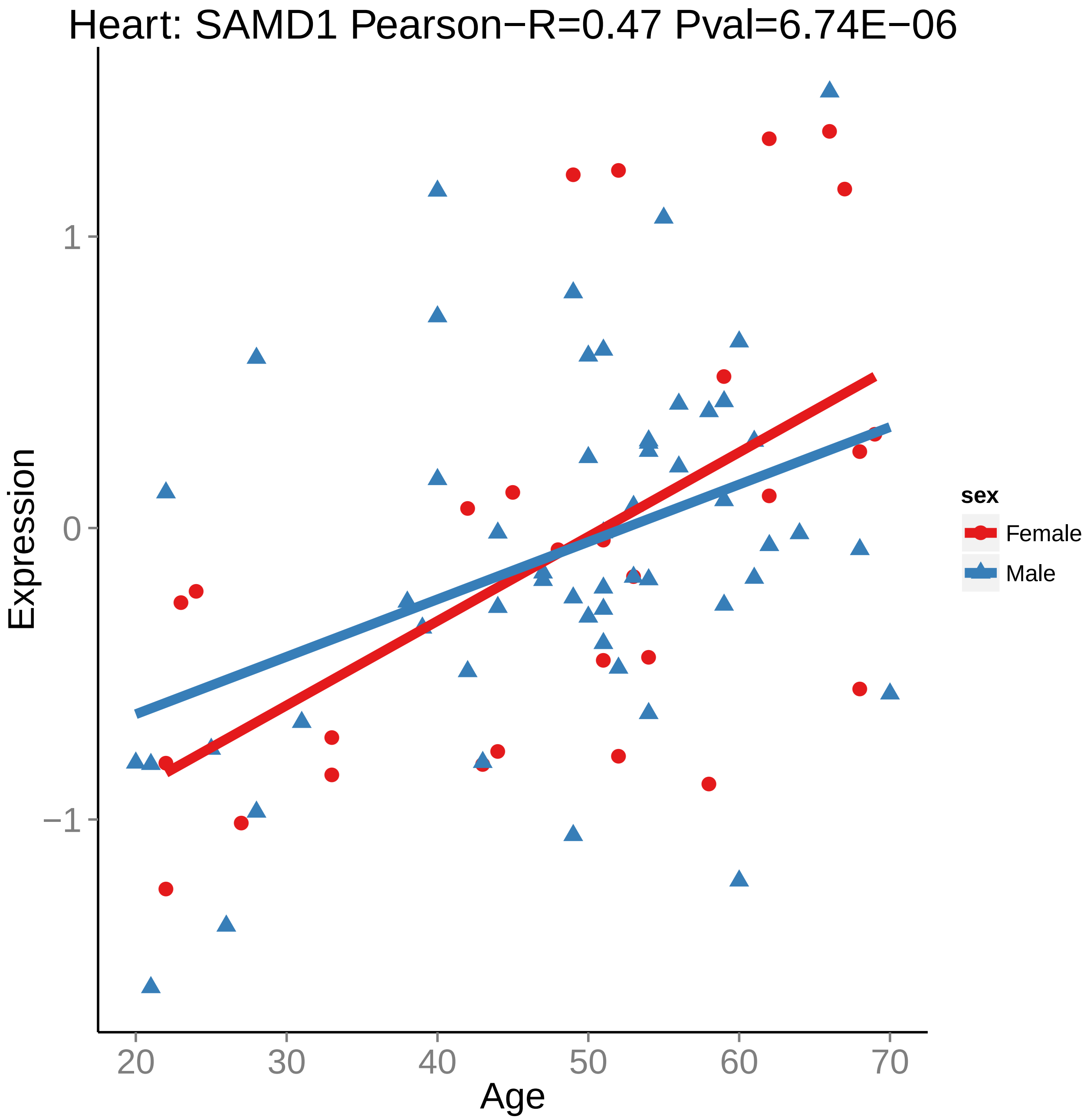

Heart: SAMD1 Pearson−R=0.47 Pval=6.74E−06
Expression
Age
1
0
−1
20
30
40
50
60
70
sex
Female
Male

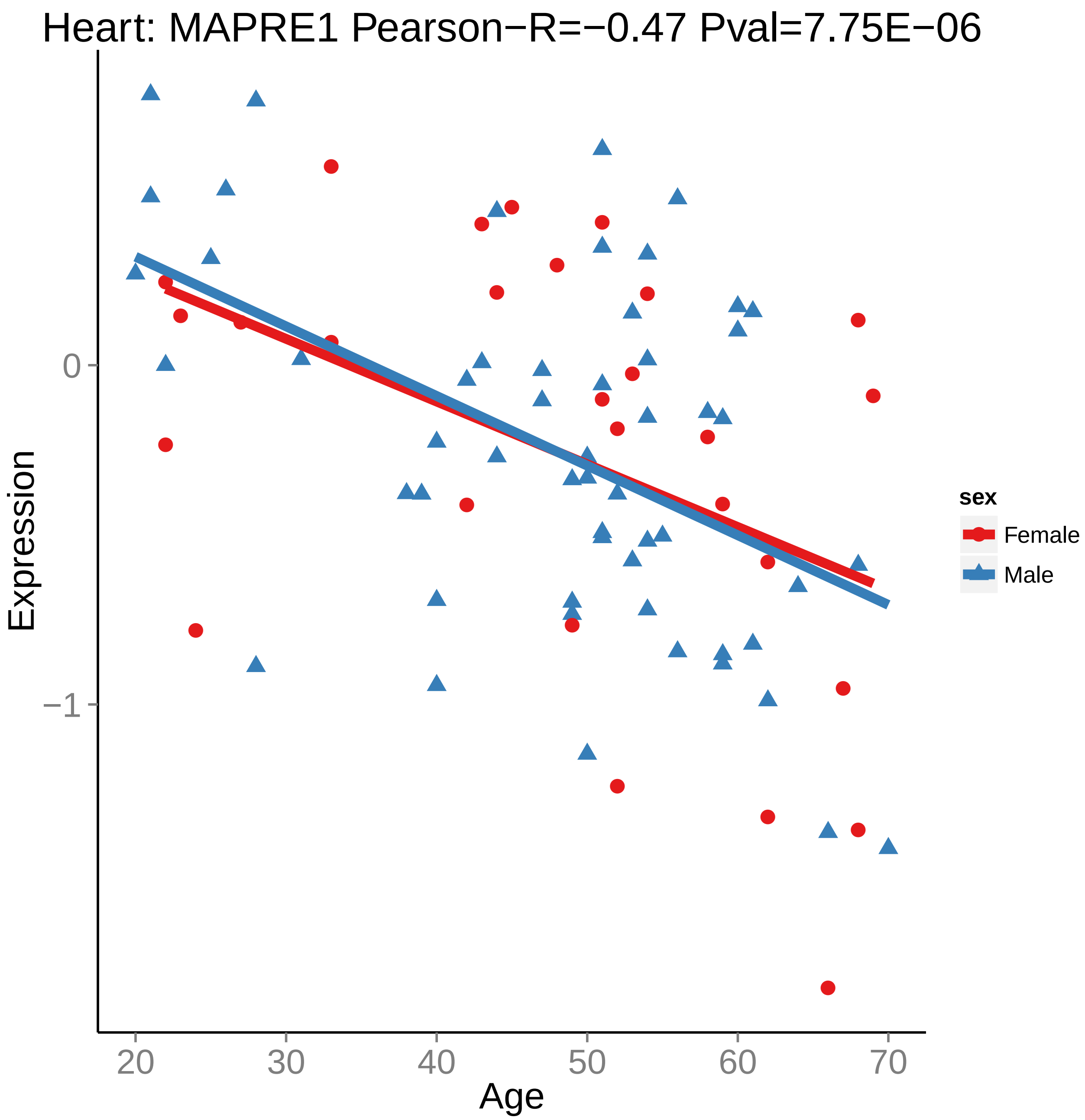

Heart: MAPRE1 Pearson−R=−0.47 Pval=7.75E−06
Expression
0
−1
20
30
40
50
60
70
Age
sex
Female
Male

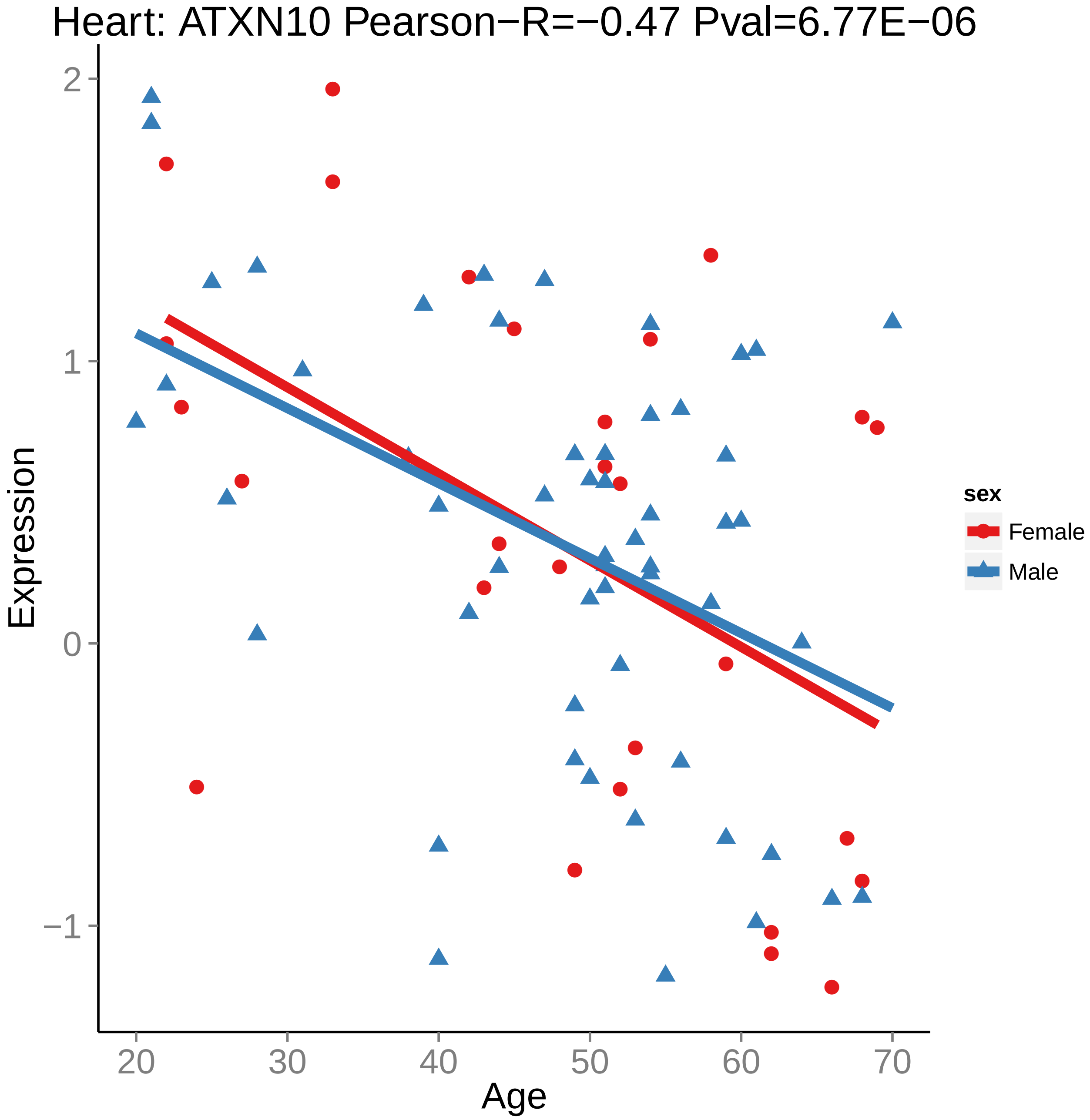
Heart: ATXN10 Pearson−R=−0.47 Pval=6.77E−06
Expression
Age
2
1
0
−1
20
30
40
50
60
70
sex
Female
Male

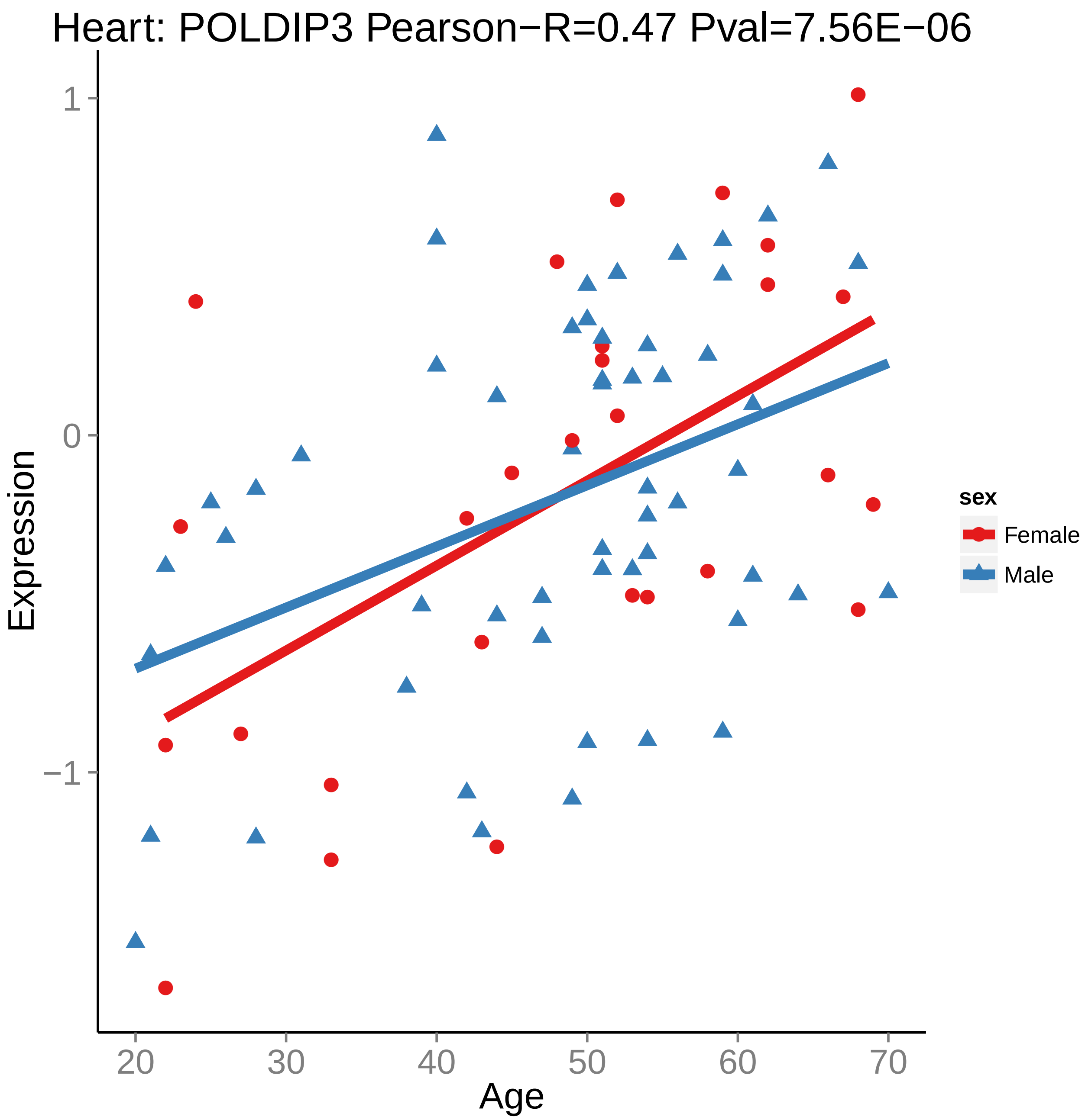
Heart: POLDIP3 Pearson−R=0.47 Pval=7.56E−06
Expression
1
0
−1
Age
20
30
40
50
60
70
sex
Female
Male

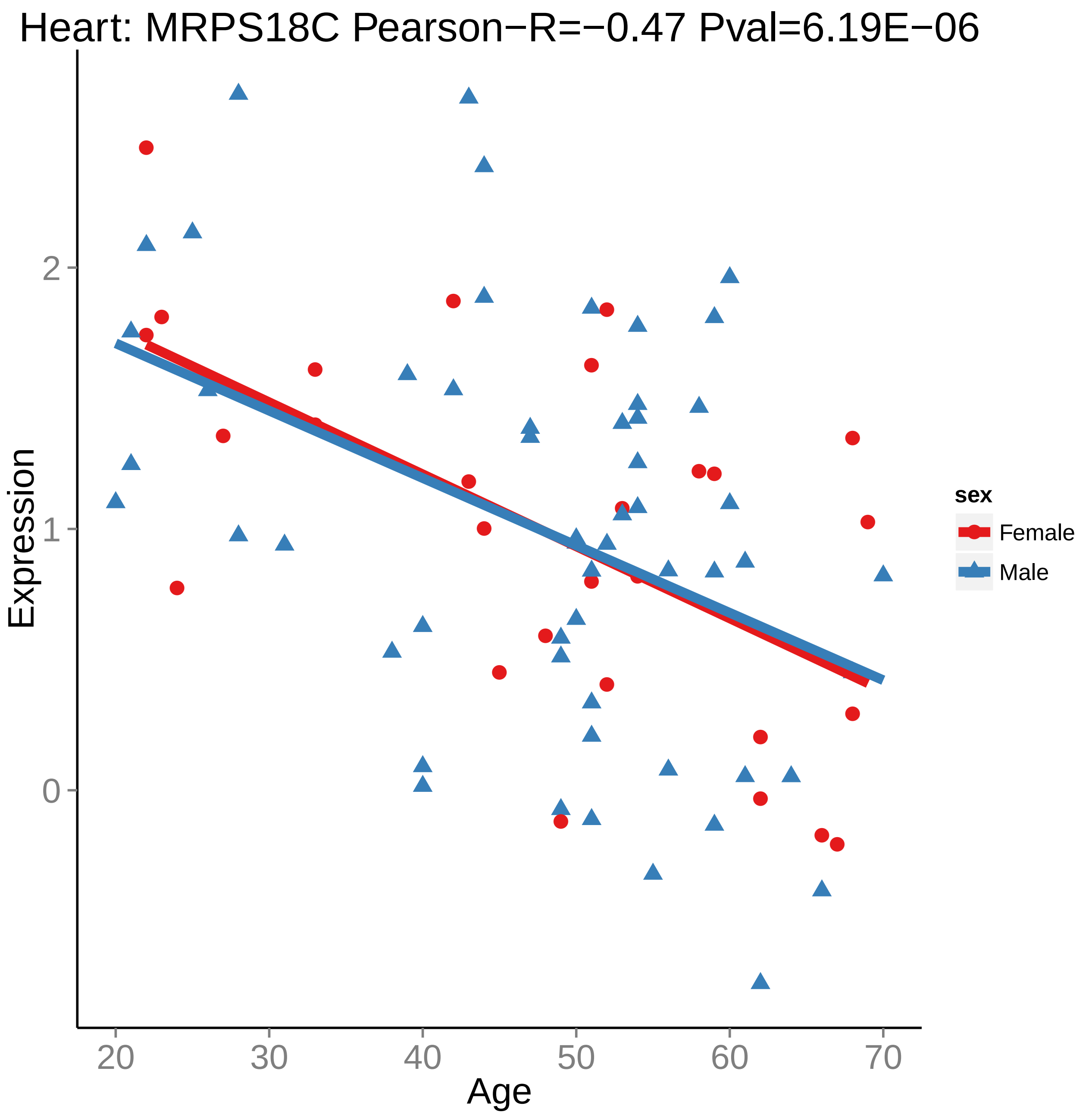
Heart: MRPS18C Pearson−R=−0.47 Pval=6.19E−06
Expression
2
1
0
20
30
40
50
60
70
Age
sex
Female
Male

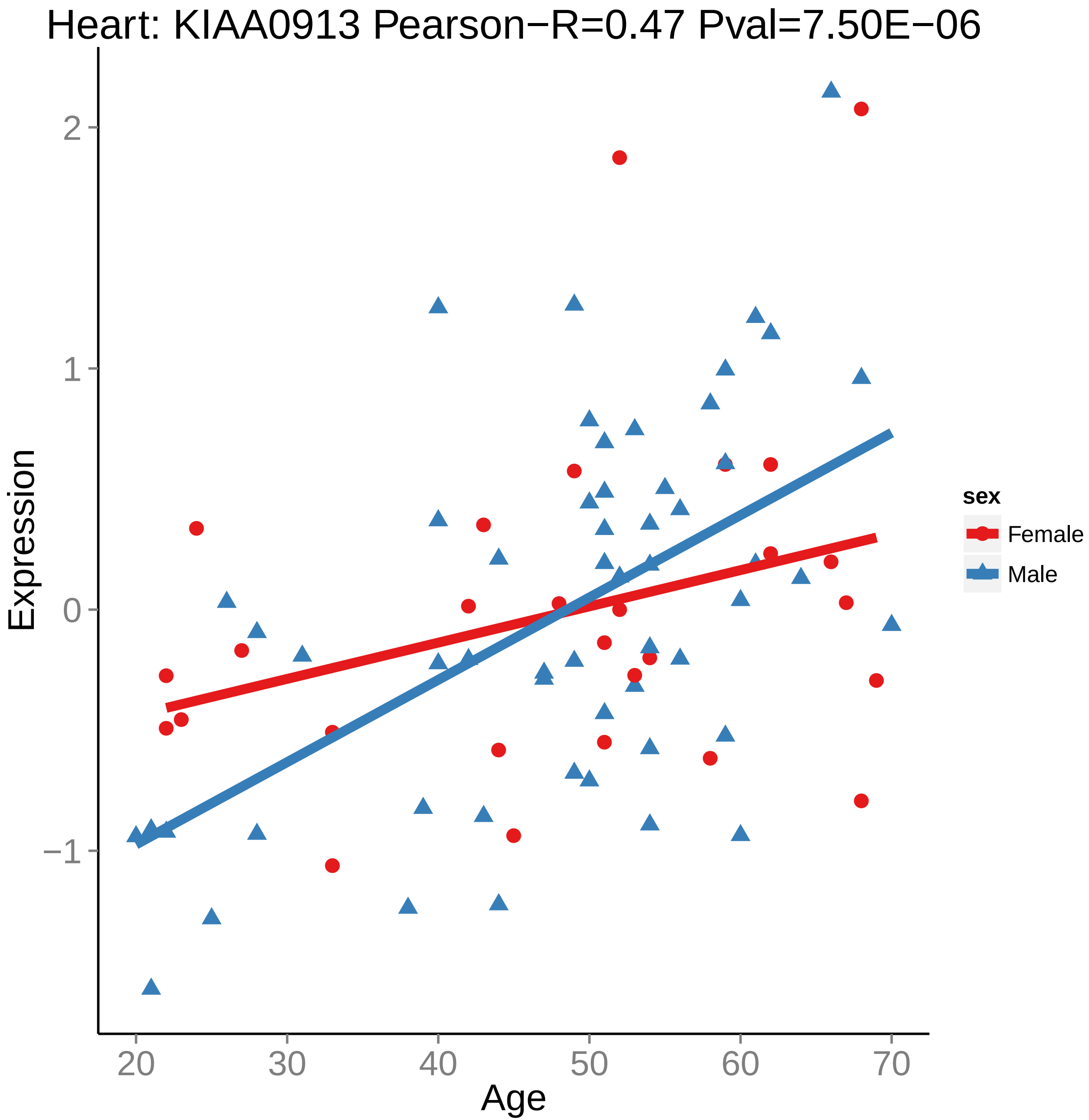
Heart: KIAA0913 Pearson−R=0.47 Pval=7.50E−06
Expression
2
1
0
−1
20
30
40
50
60
70
Age
sex
Female
Male

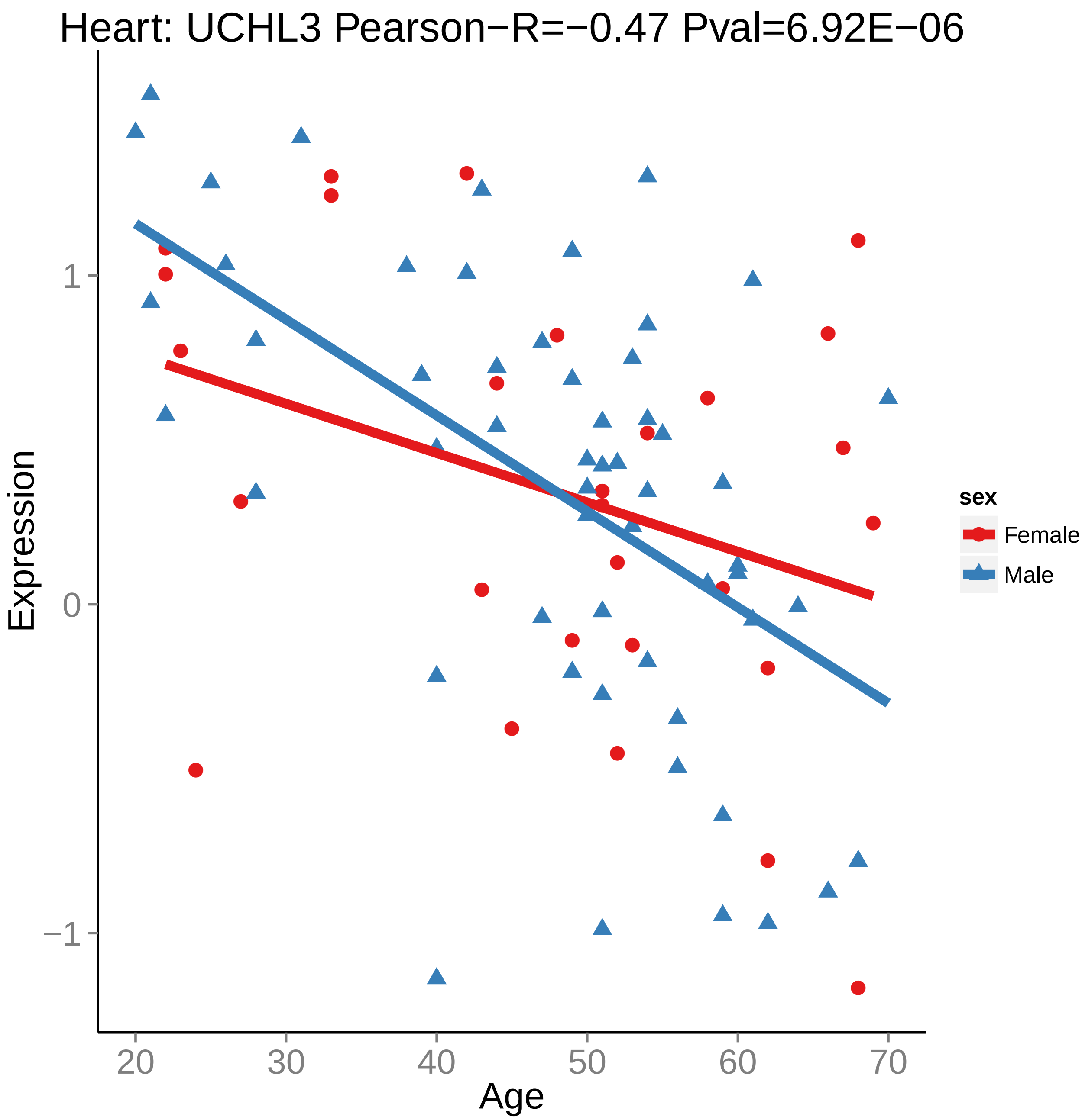

Heart: UCHL3 Pearson−R=−0.47 Pval=6.92E−06
Expression
1
0
−1
20
30
40
50
60
70
Age
sex
Female
Male

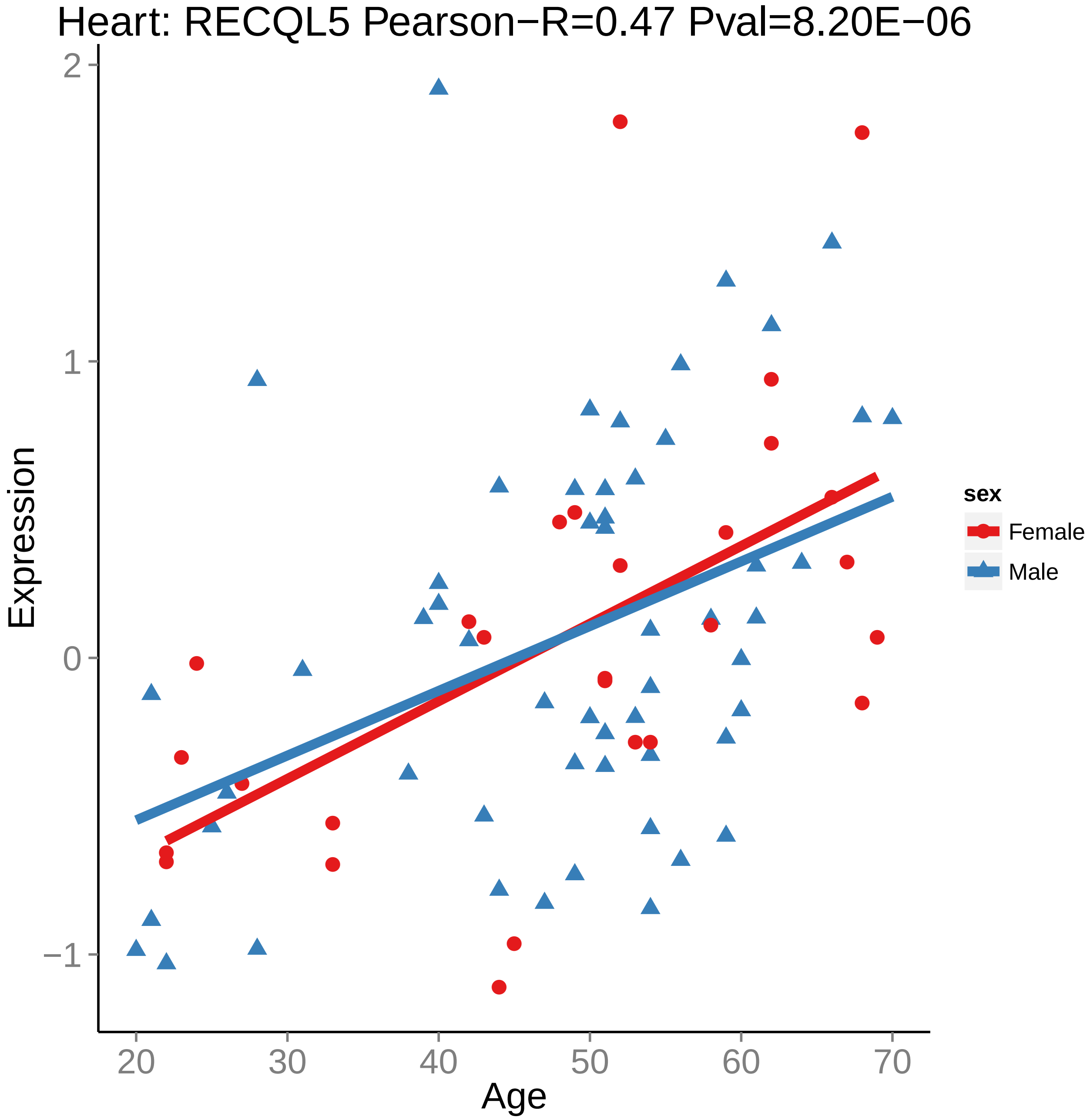

Heart: RECQL5 Pearson−R=0.47 Pval=8.20E−06
Expression
Age
2
1
0
−1
20
30
40
50
60
70
sex
Female
Male

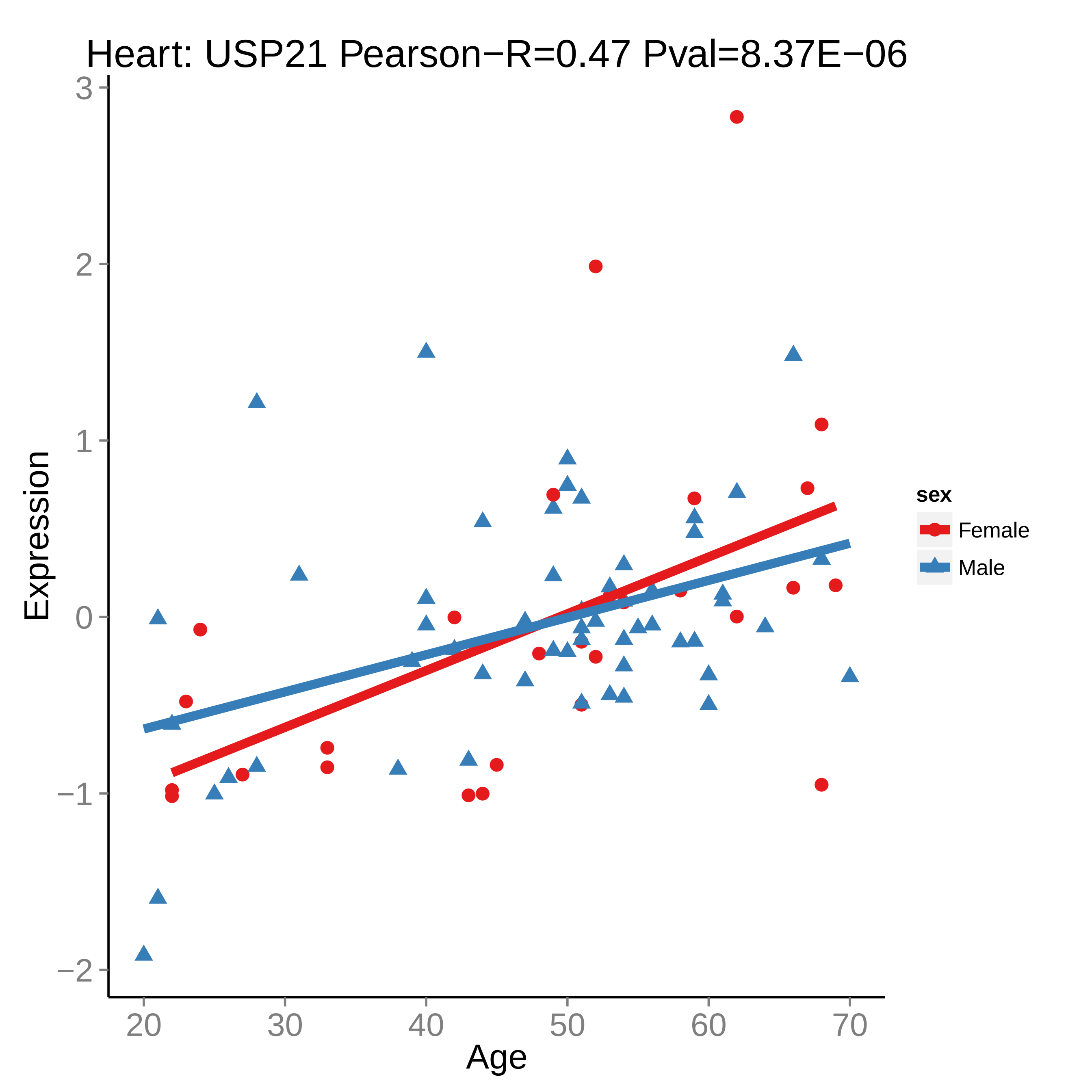
Heart: USP21 Pearson−R=0.47 Pval=8.37E−06
Expression
3
2
1
0
−1
−2
20
30
40
50
60
70
Age
sex
Female
Male

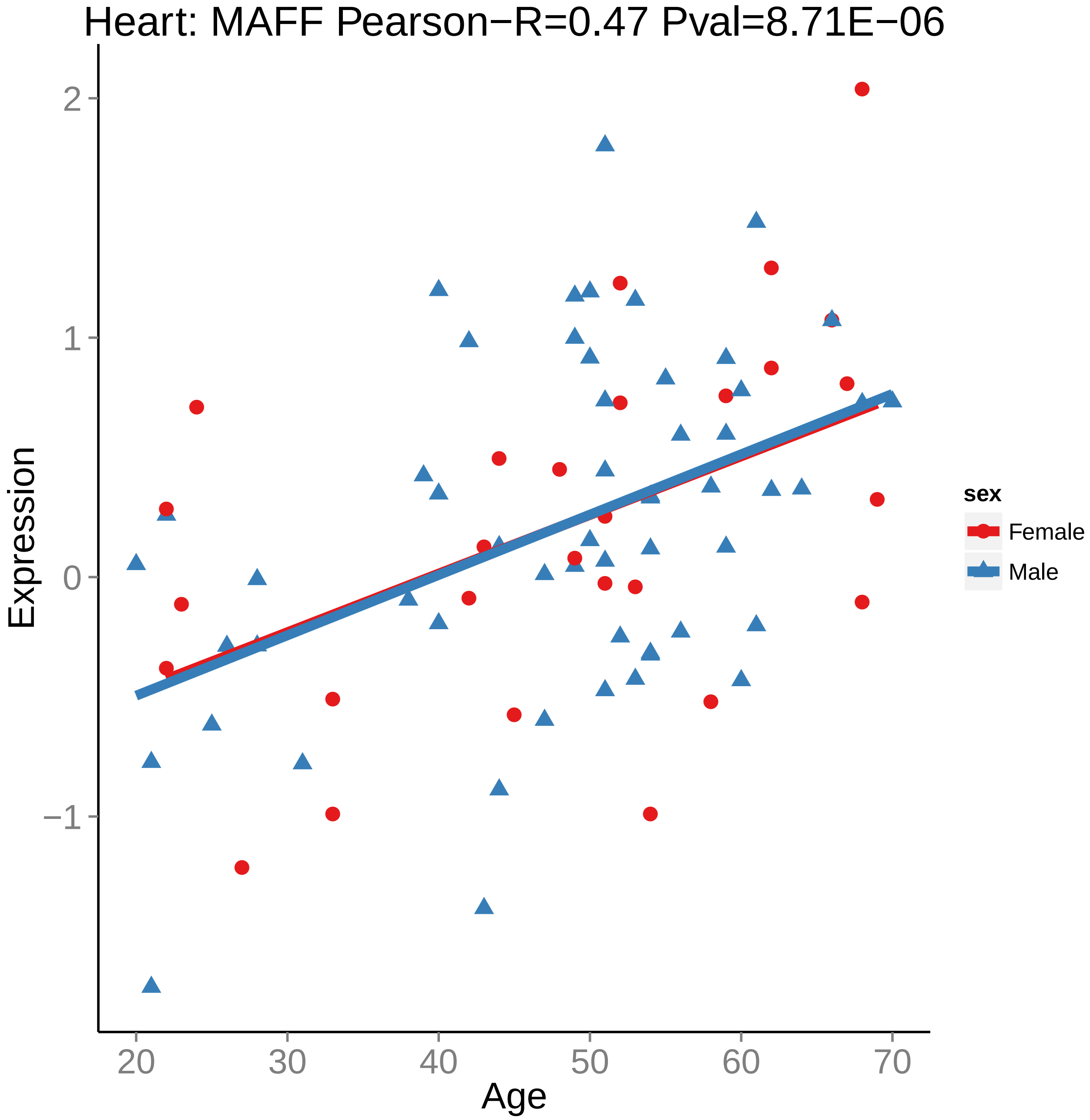
Heart: MAFF Pearson−R=0.47 Pval=8.71E−06
Expression
2
1
0
−1
20
30
40
50
60
70
Age
sex
Female
Male

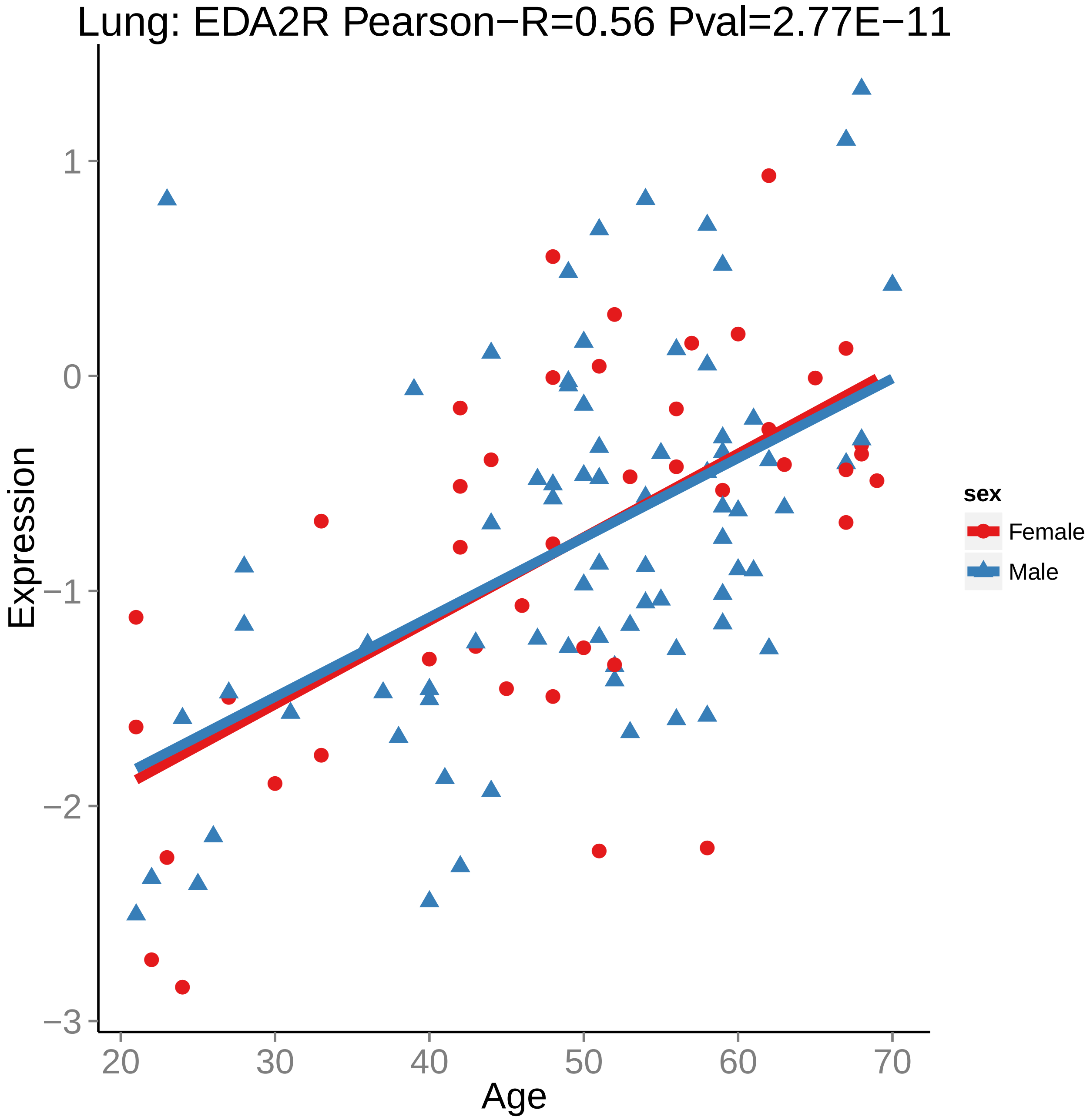

Lung: EDA2R Pearson−R=0.56 Pval=2.77E−11
Expression
Age
1
0
−1
−2
−3
20
30
40
50
60
70
sex
Female
Male

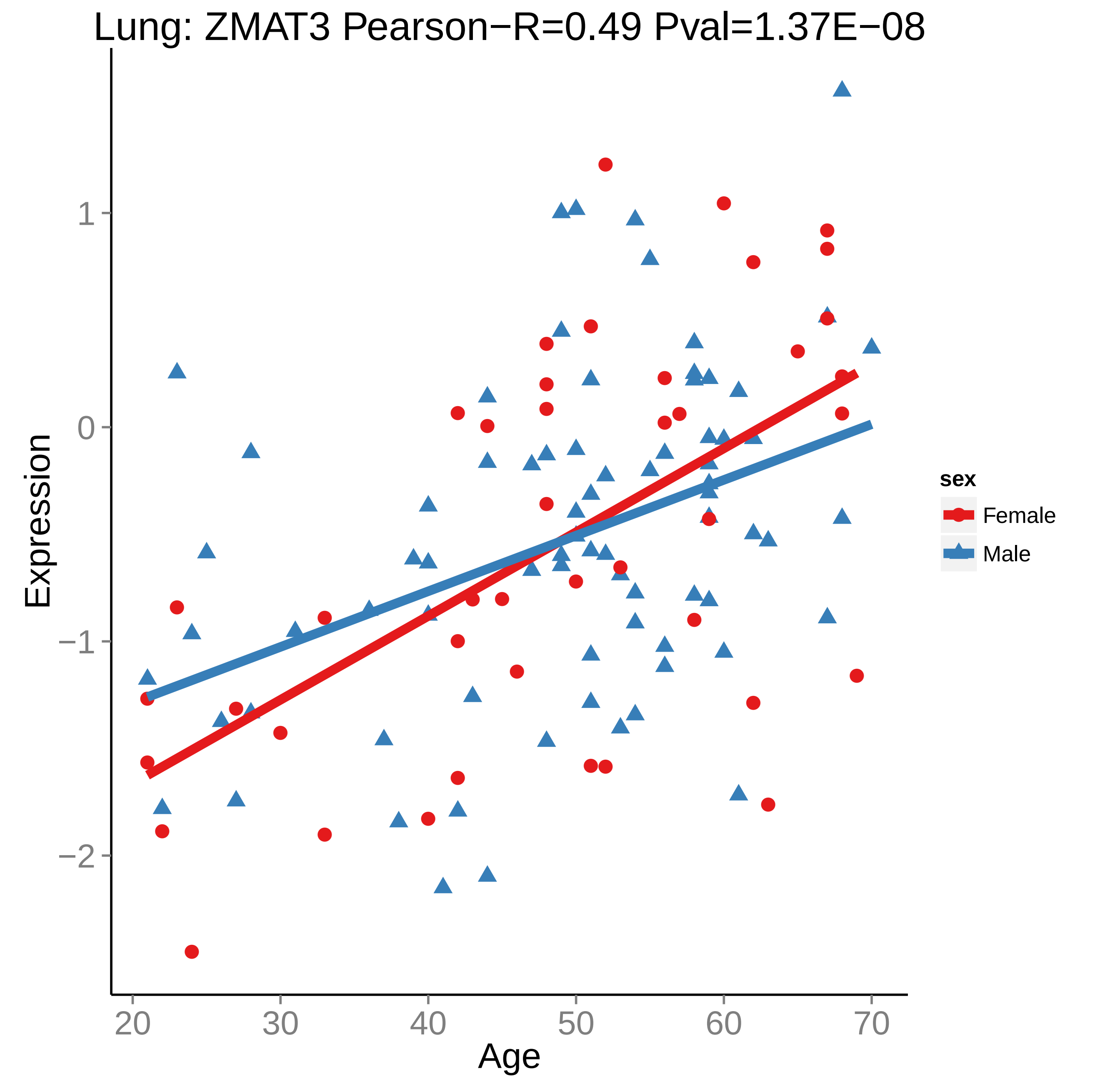

Lung: ZMAT3 Pearson−R=0.49 Pval=1.37E−08
Expression
Age
1
0
−1
−2
20
30
40
50
60
70
sex
Female
Male

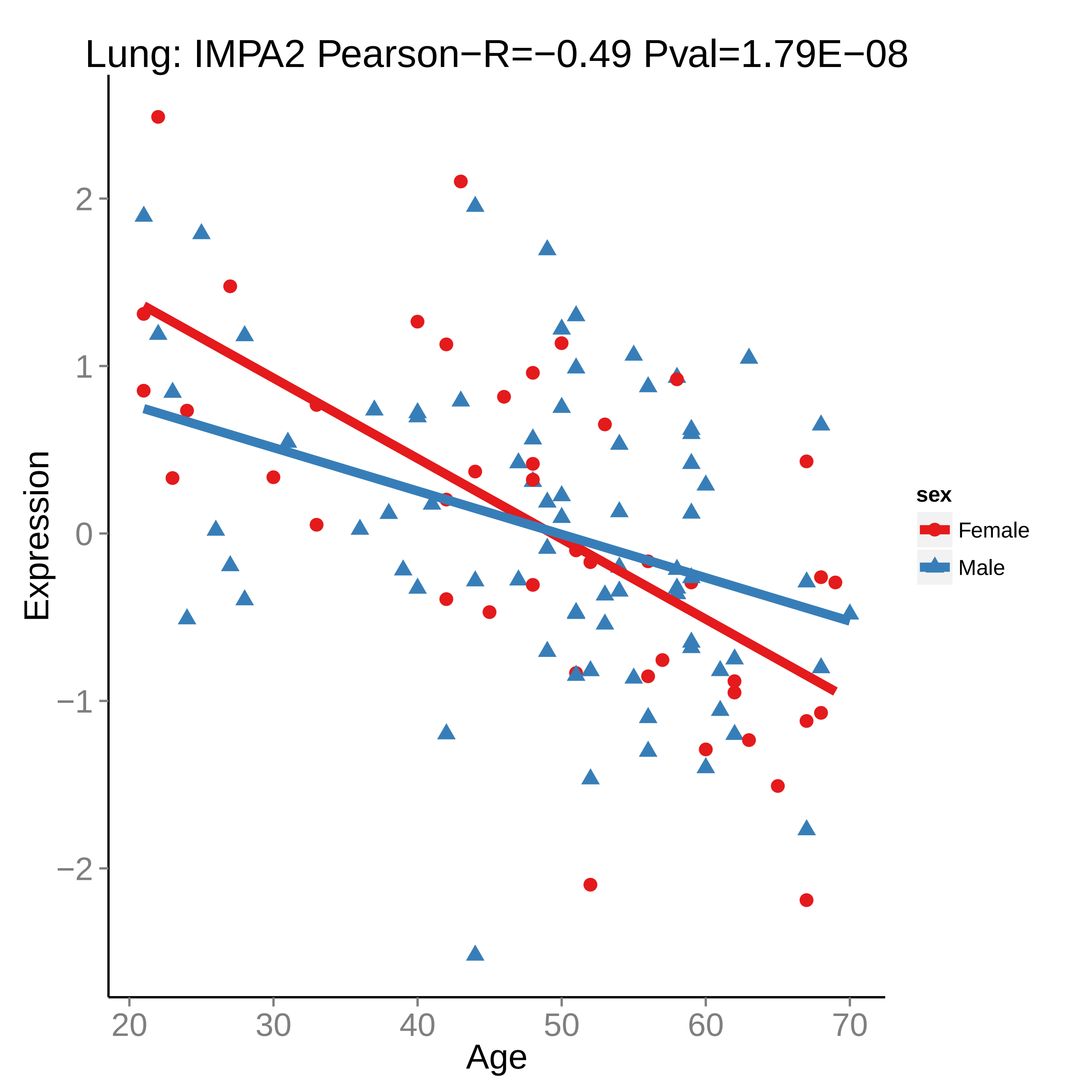

Lung: IMPA2 Pearson−R=−0.49 Pval=1.79E−08
Expression
Age
2
1
0
−1
−2
20
30
40
50
60
70
sex
Female
Male

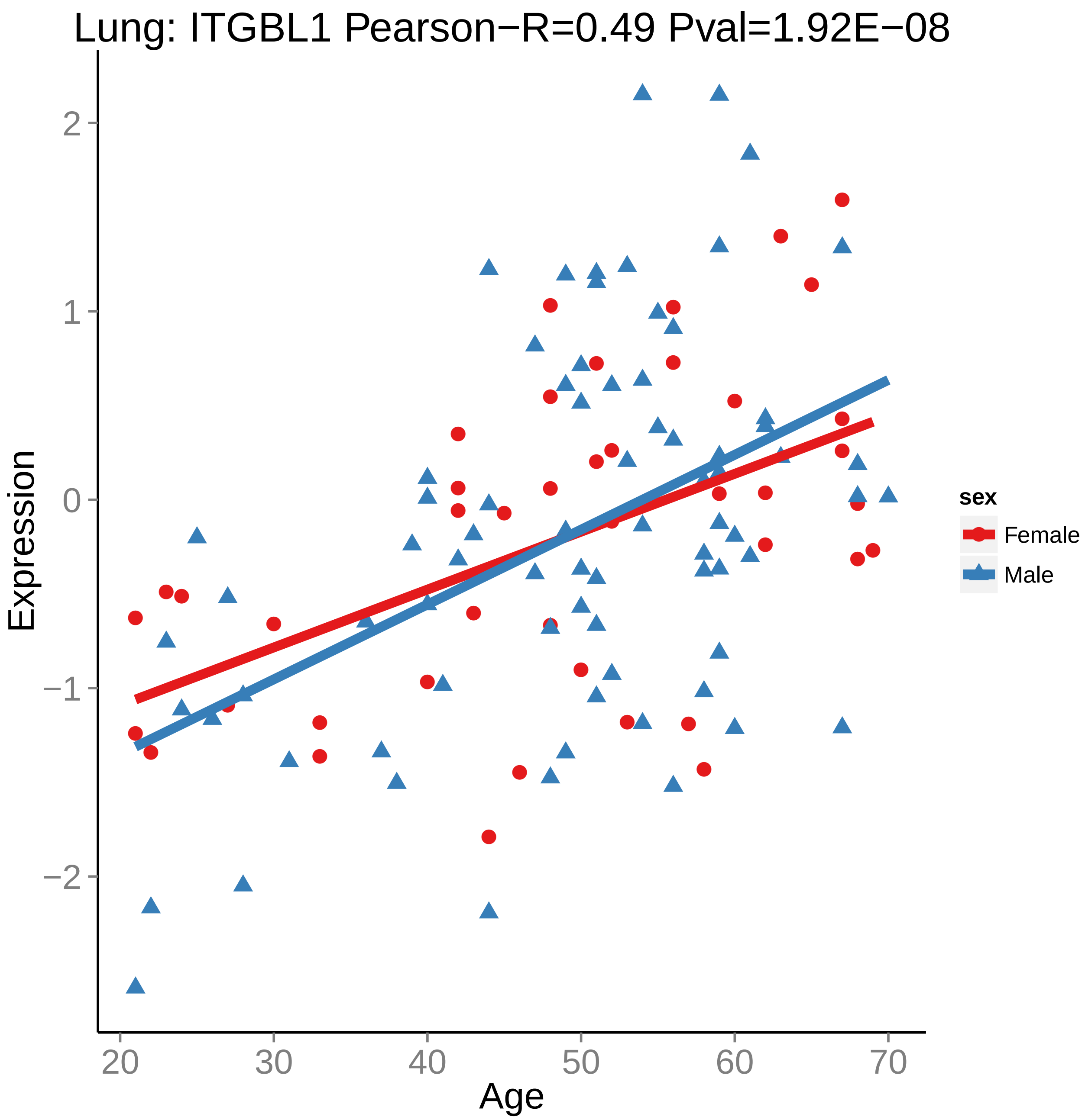

Lung: ITGBL1 Pearson−R=0.49 Pval=1.92E−08
Expression
Age
2
1
0
−1
−2
20
30
40
50
60
70
sex
Female
Male

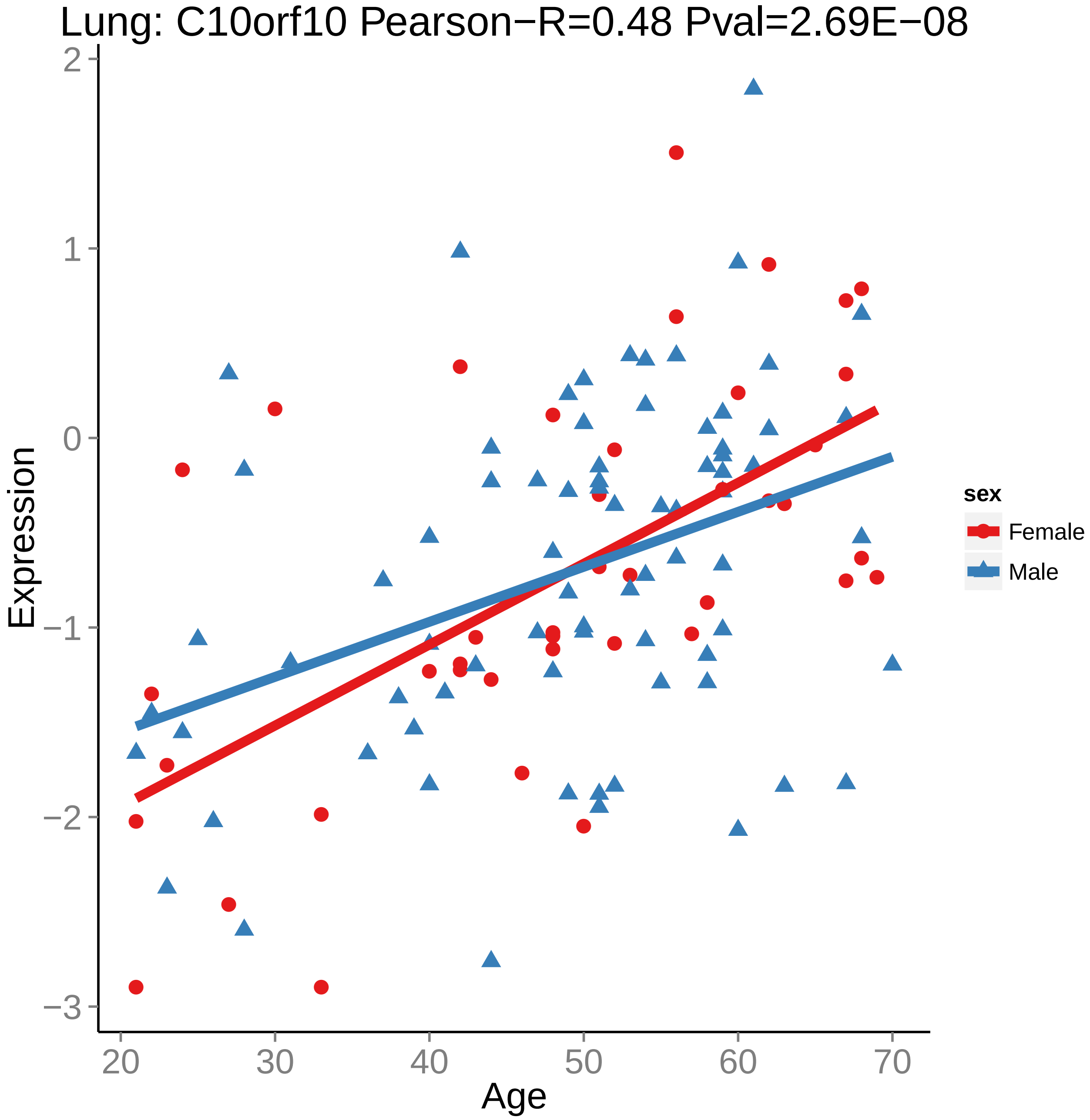

Lung: C10orf10 Pearson−R=0.48 Pval=2.69E−08
Expression
Age
2
1
0
−1
−2
−3
20
30
40
50
60
70
sex
Female
Male

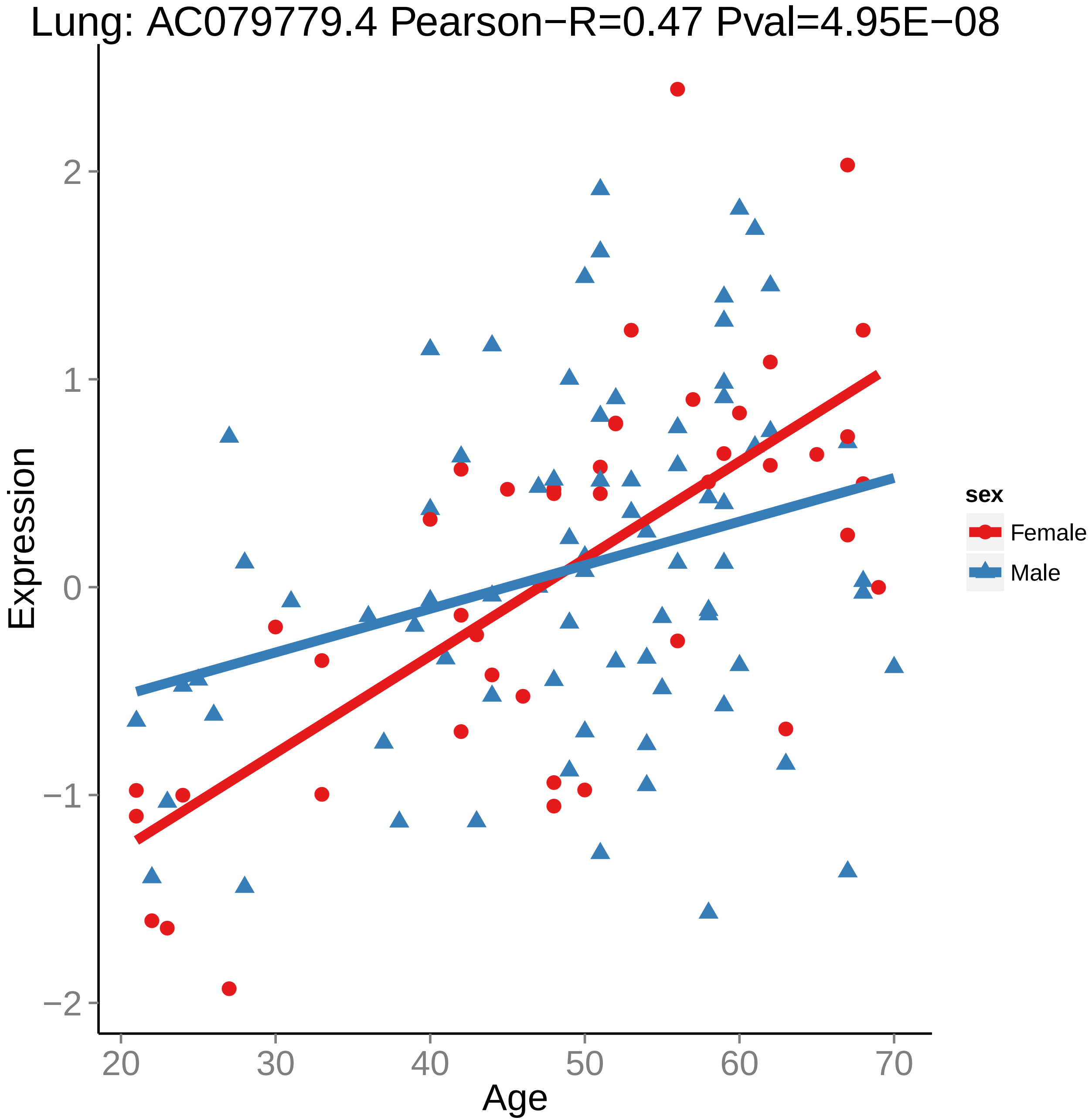

Lung: AC079779.4 Pearson−R=0.47 Pval=4.95E−08
Expression
Age
2
1
0
−1
−2
20
30
40
50
60
70
sex
Female
Male

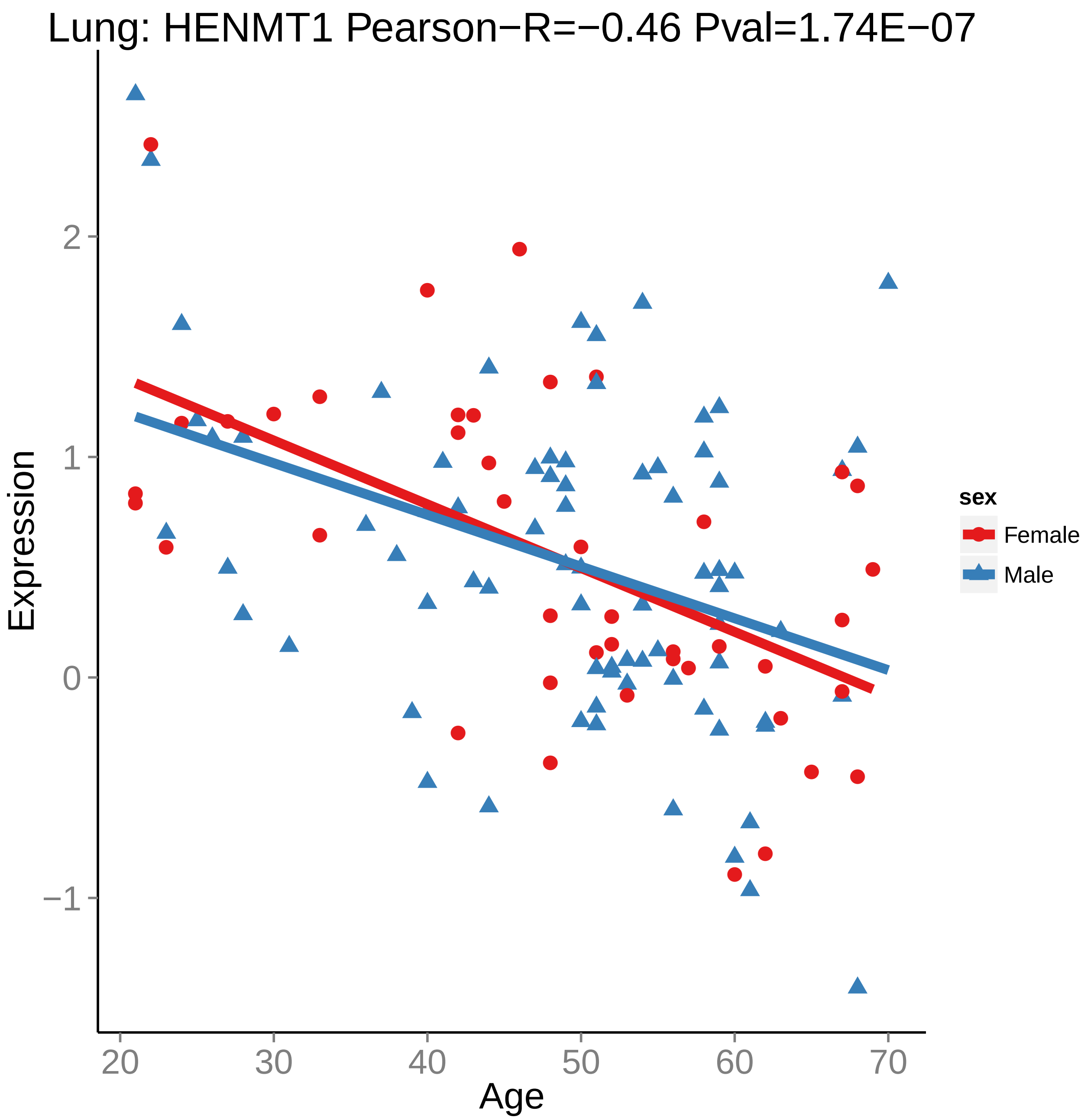

Lung: HENMT1 Pearson−R=−0.46 Pval=1.74E−07
Expression
2
1
0
−1
20
30
40
50
60
70
Age
sex
Female
Male

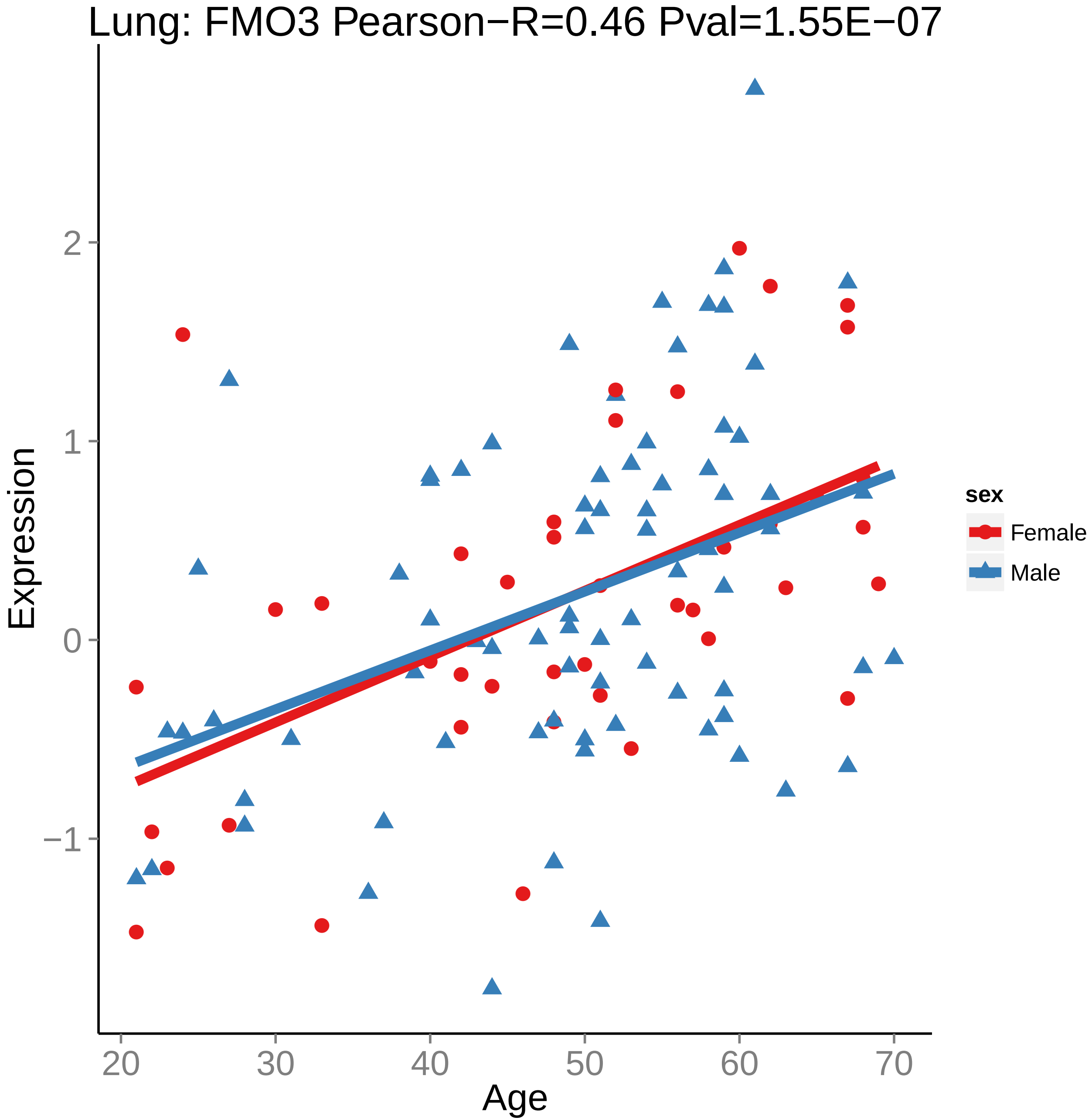

Lung: FMO3 Pearson−R=0.46 Pval=1.55E−07
Expression
2
1
0
−1
20
30
40
50
60
70
Age
sex
Female
Male

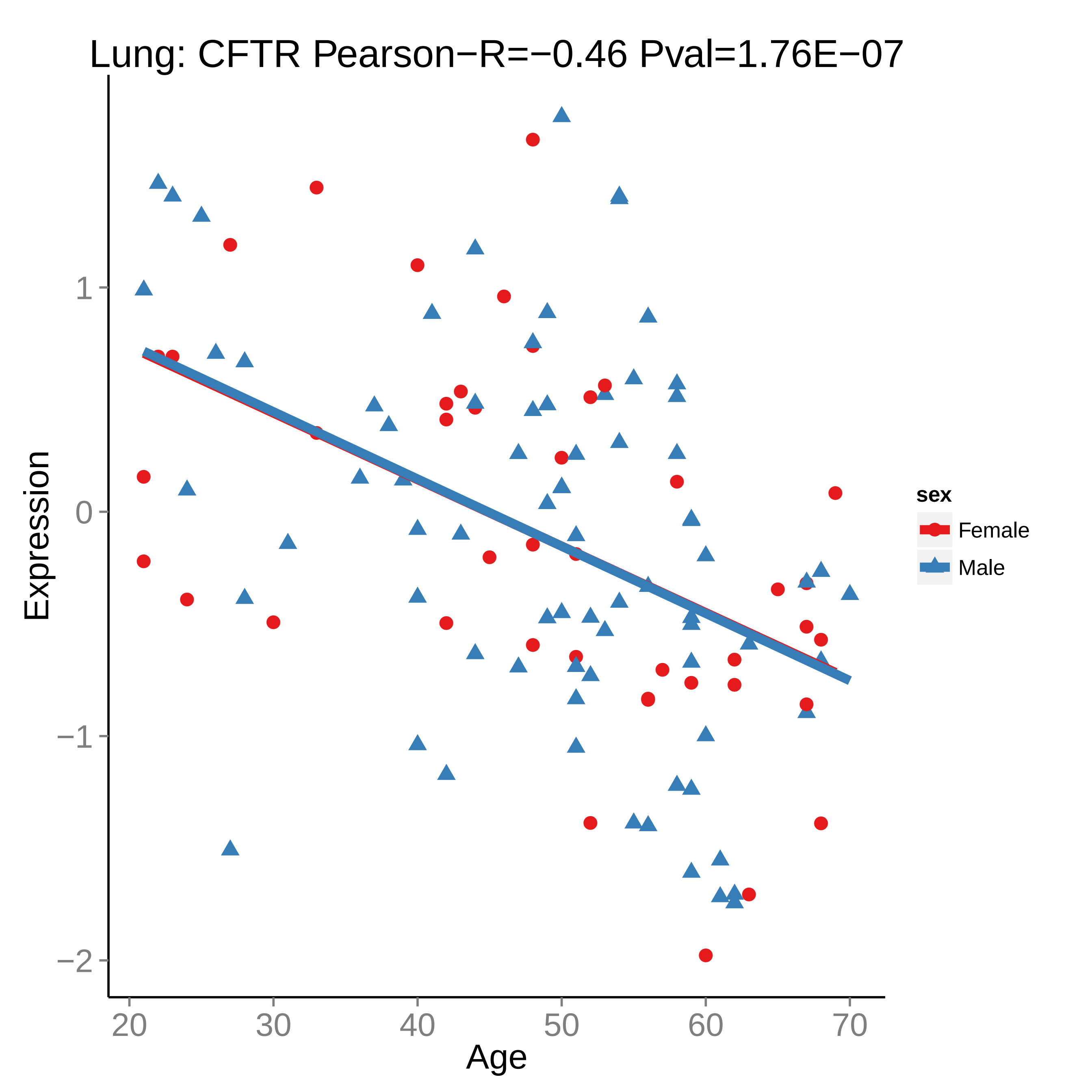
Lung: CFTR Pearson−R=−0.46 Pval=1.76E−07
Expression
1
0
−1
−2
20
30
40
50
60
70
Age
sex
Female
Male

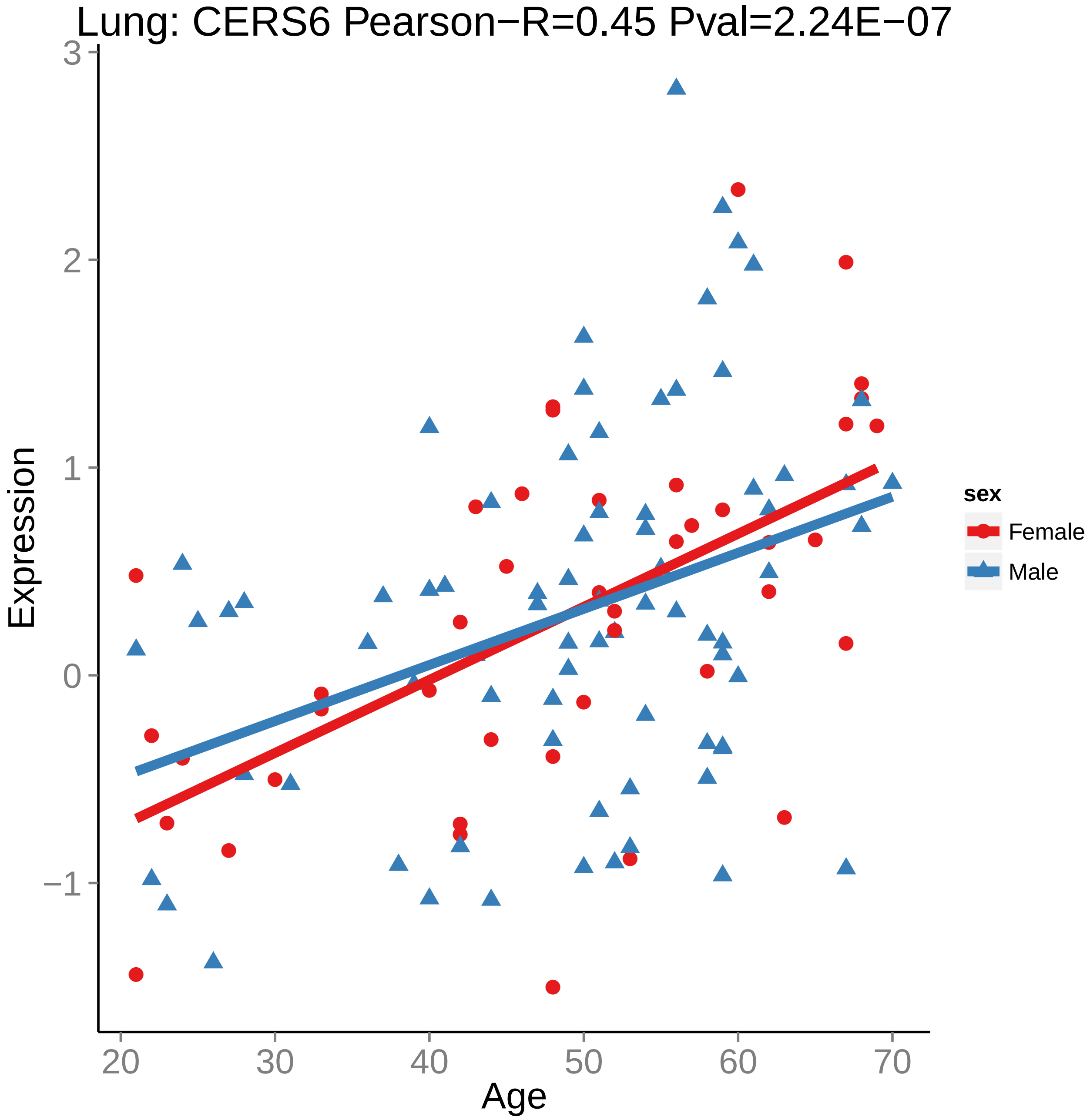

Lung: CERS6 Pearson−R=0.45 Pval=2.24E−07
Expression
Age
3
2
1
0
−1
20
30
40
50
60
70
sex
Female
Male

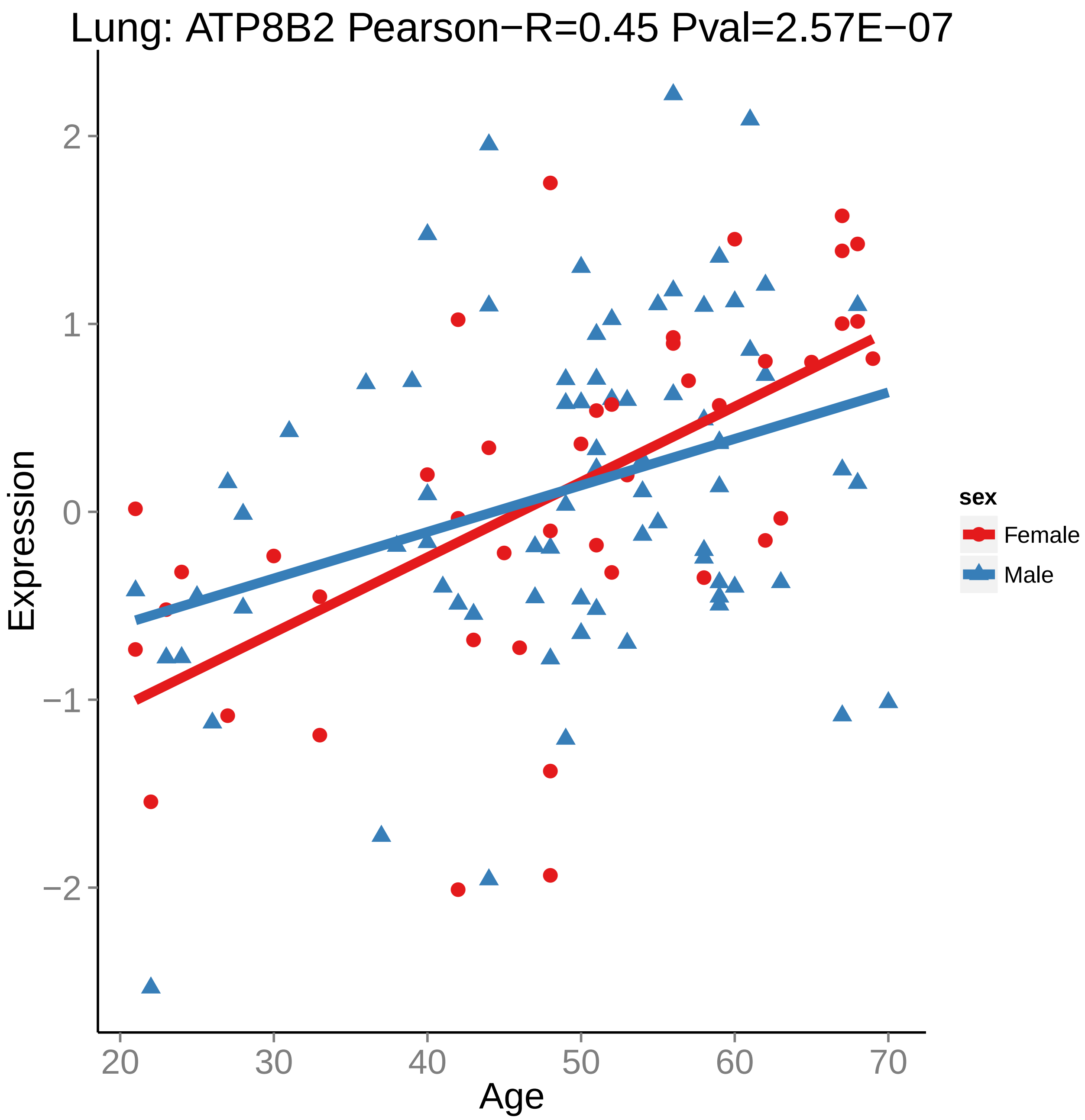

Lung: ATP8B2 Pearson−R=0.45 Pval=2.57E−07
Expression
2
1
0
−1
−2
20
30
40
50
60
70
Age
sex
Female
Male

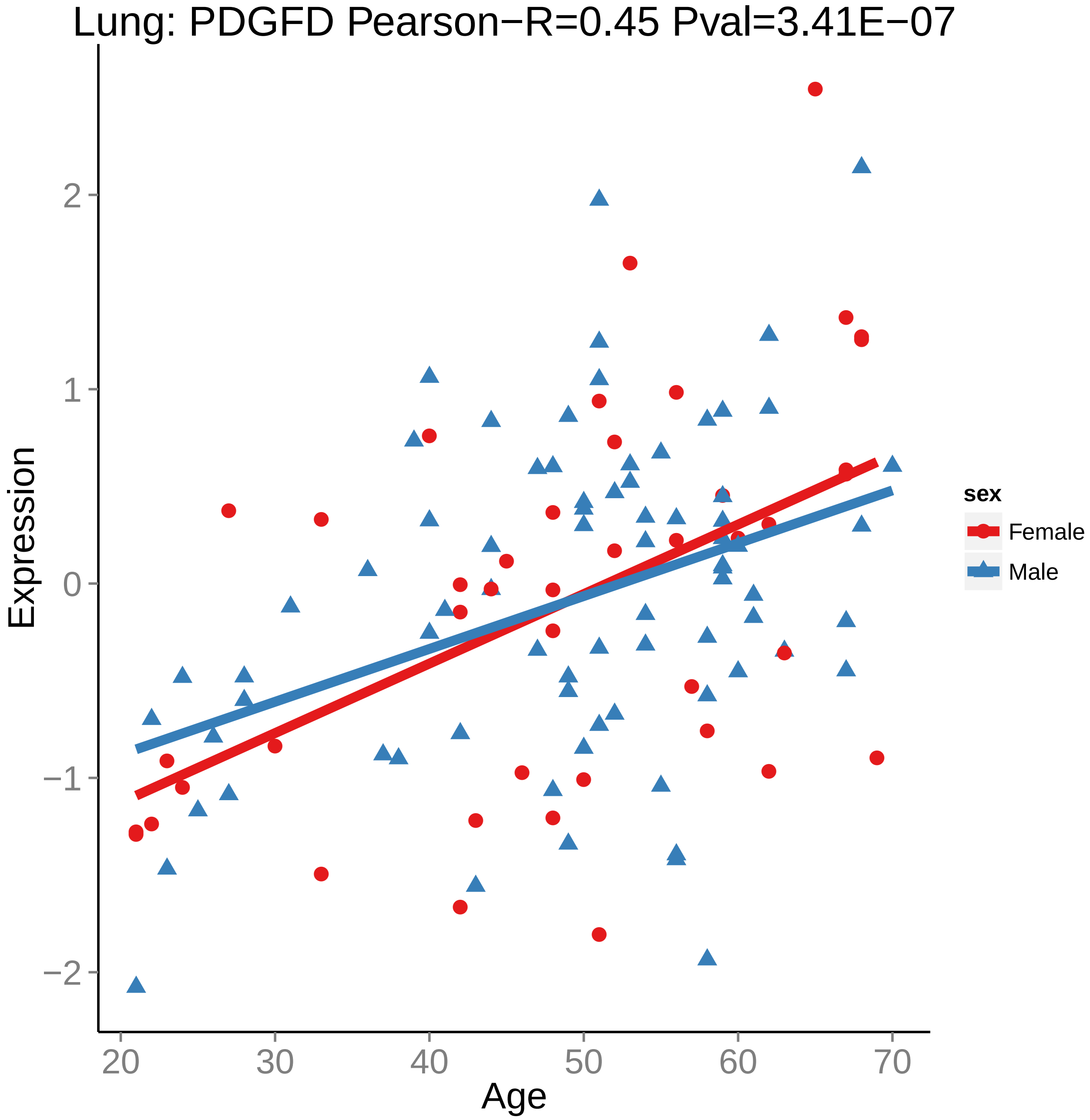

Lung: PDGFD Pearson−R=0.45 Pval=3.41E−07
Expression
2
1
0
−1
−2
20
30
40
50
60
70
Age
sex
Female
Male

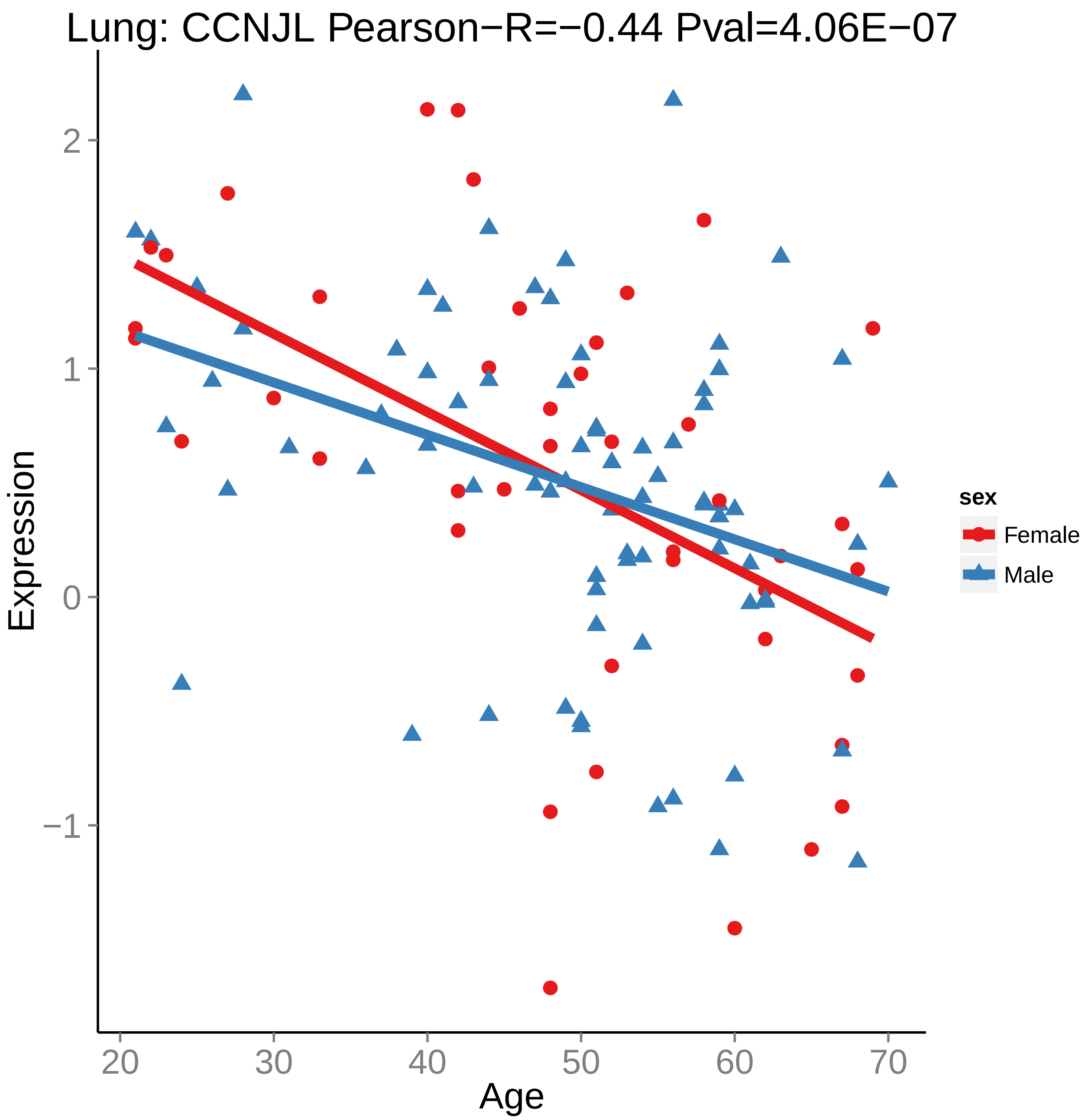

Lung: CCNJL Pearson−R=−0.44 Pval=4.06E−07
Expression
2
1
0
−1
20
30
40
50
60
70
Age
sex
Female
Male

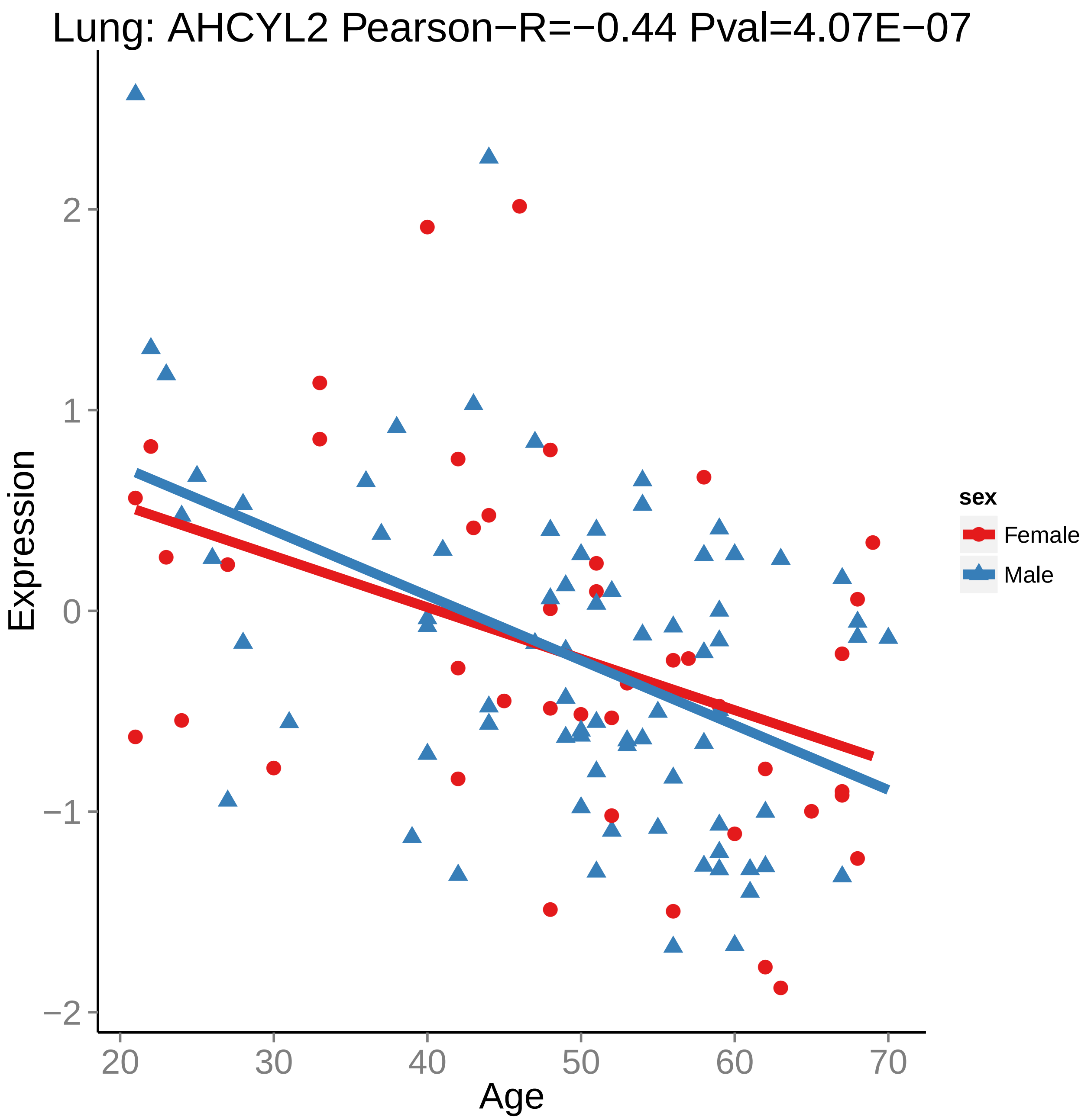

Lung: AHCYL2 Pearson−R=−0.44 Pval=4.07E−07
Expression
2
1
0
−1
−2
20
30
40
50
60
70
Age
sex
Female
Male

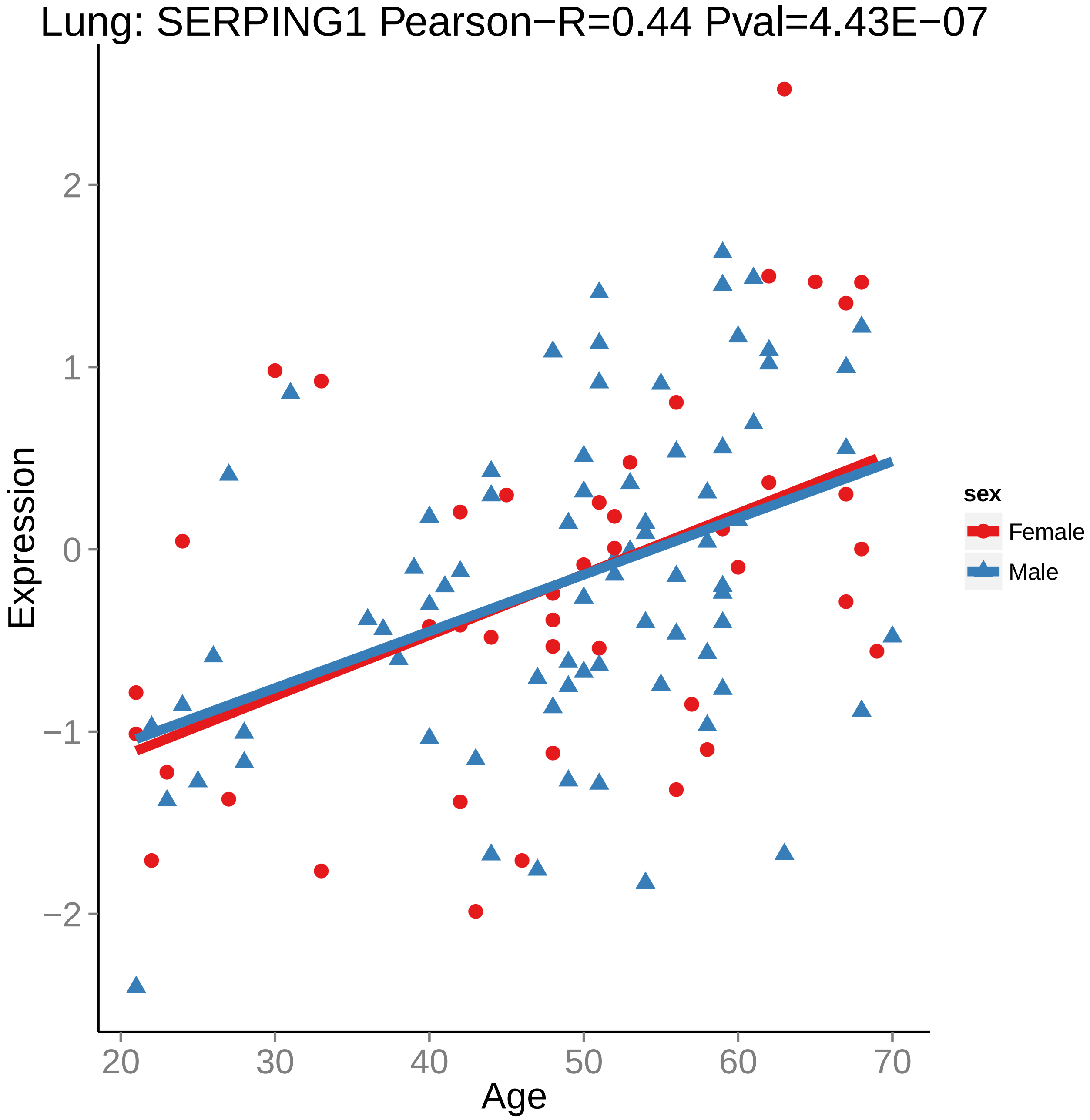
Lung: SERPING1 Pearson−R=0.44 Pval=4.43E−07
Expression
2
1
0
−1
−2
20
30
40
50
60
70
Age
sex
Female
Male

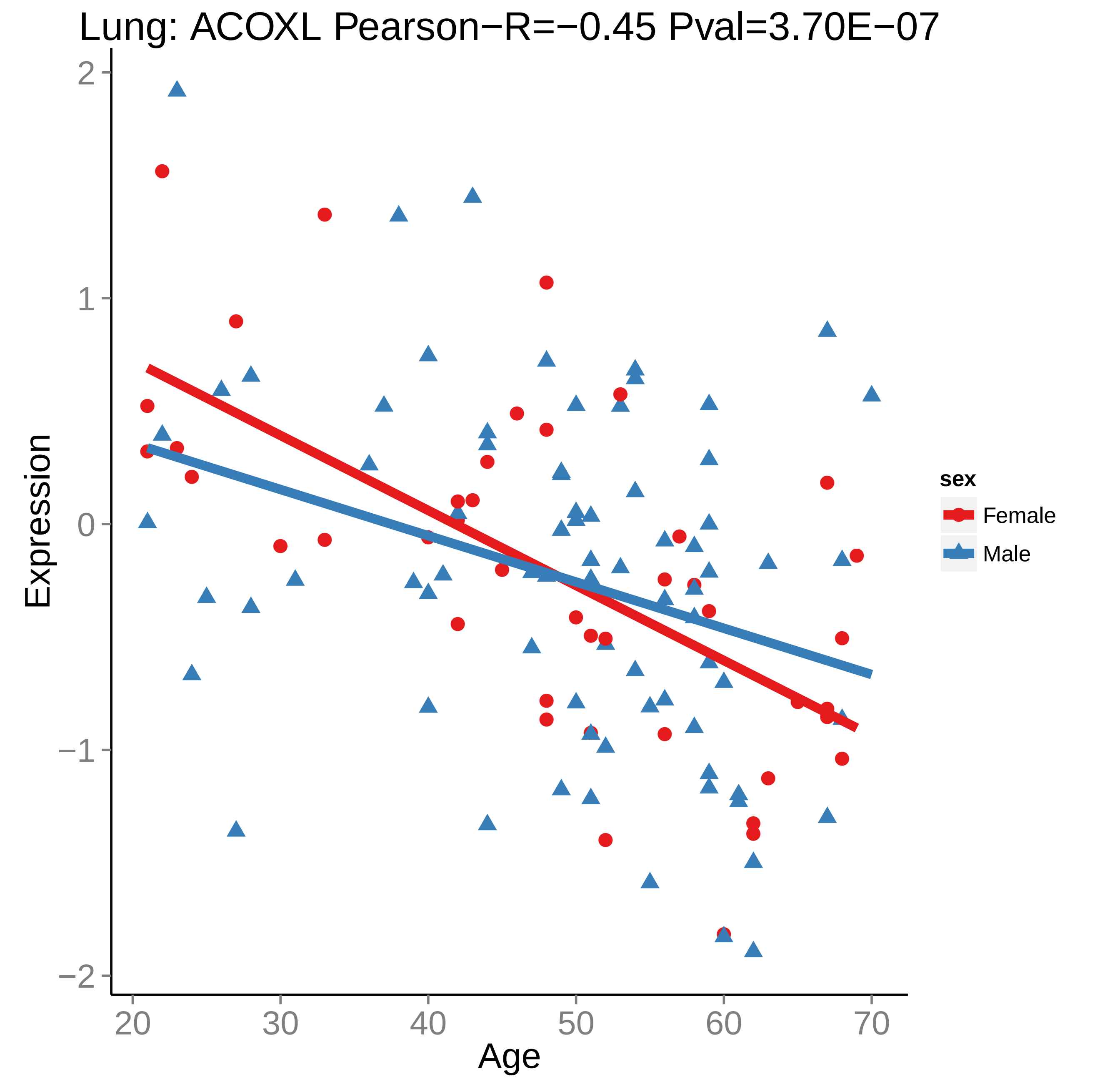

Lung: ACOXL Pearson−R=−0.45 Pval=3.70E−07
Expression
Age
2
1
0
−1
−2
20
30
40
50
60
70
sex
Female
Male

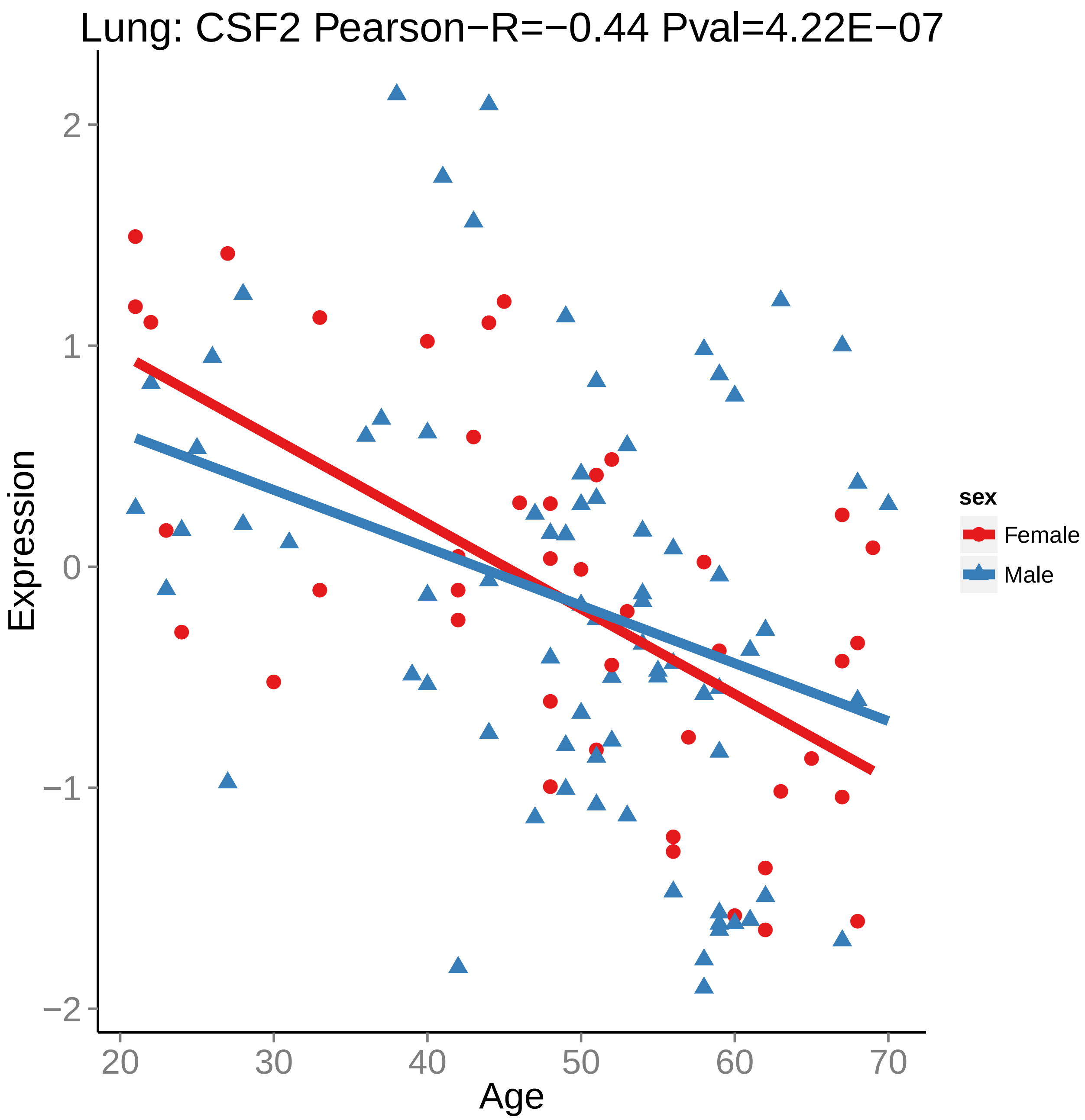

Lung: CSF2 Pearson−R=−0.44 Pval=4.22E−07
Expression
Age
2
1
0
−1
−2
20
30
40
50
60
70
sex
Female
Male

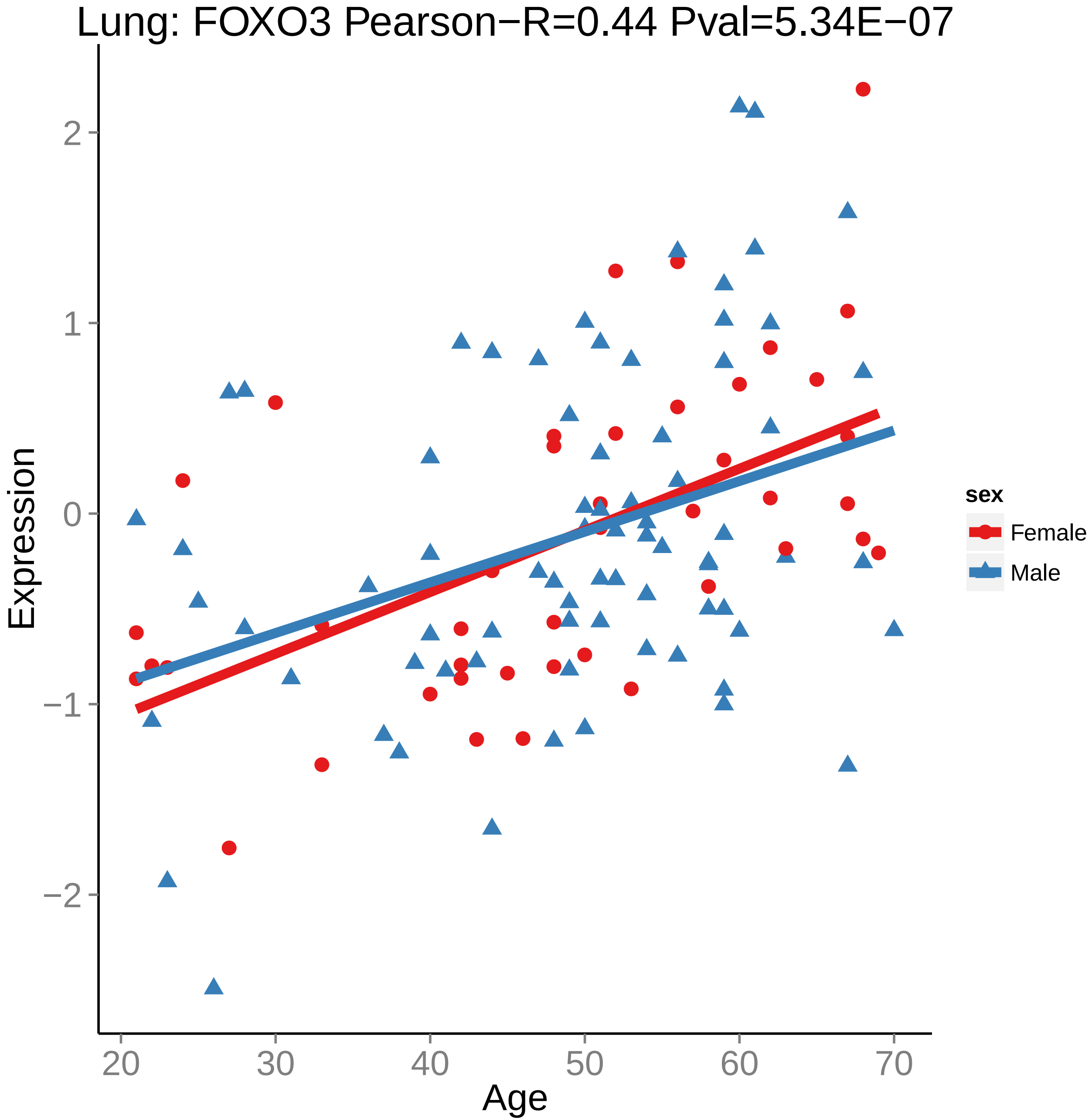

Lung: FOXO3 Pearson−R=0.44 Pval=5.34E−07
Expression
Age
2
1
0
−1
−2
20
30
40
50
60
70
sex
Female
Male

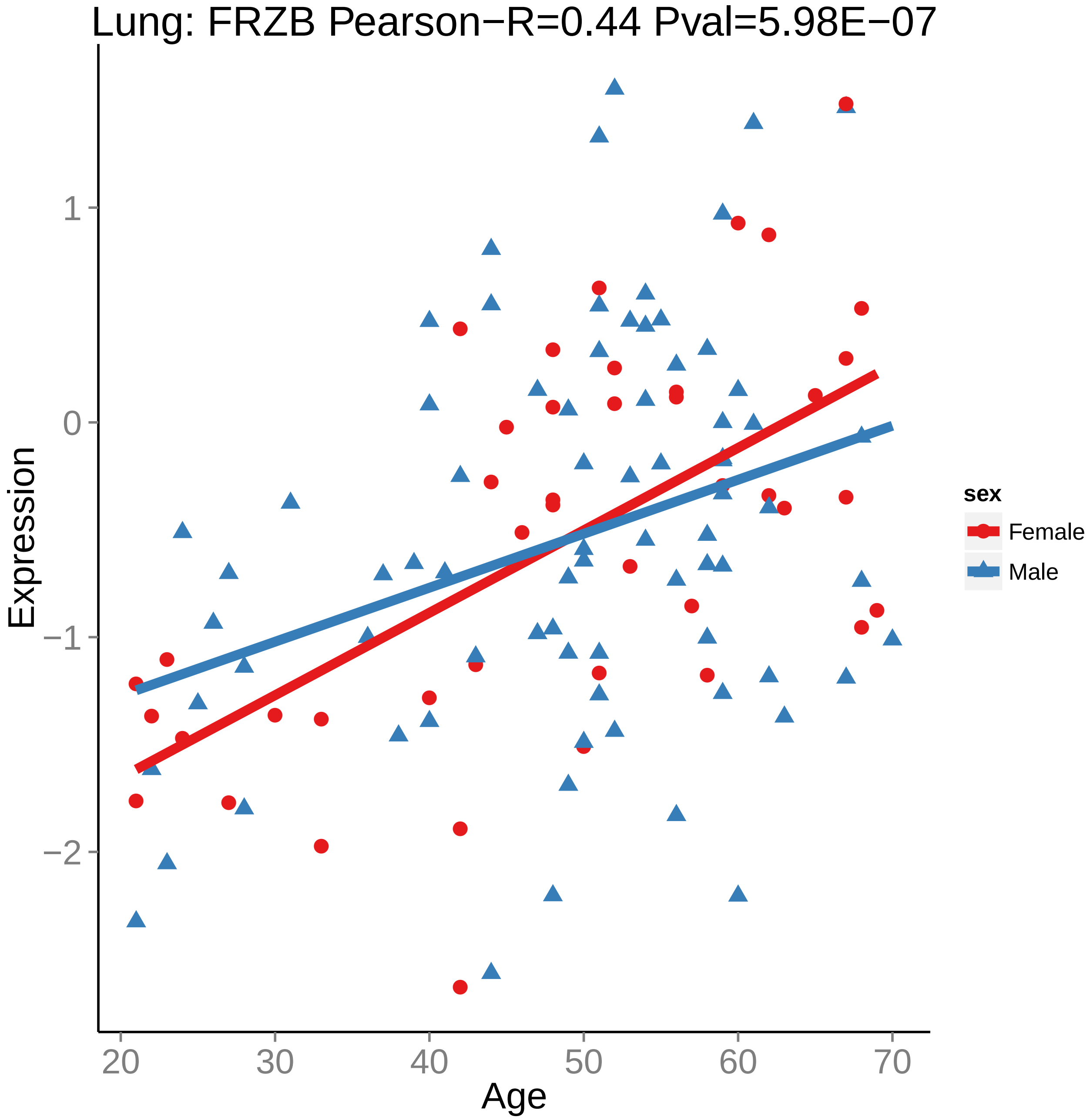
Lung: FRZB Pearson−R=0.44 Pval=5.98E−07
Expression
Age
sex
Female
Male
1
0
−1
−2
20
30
40
50
60
70

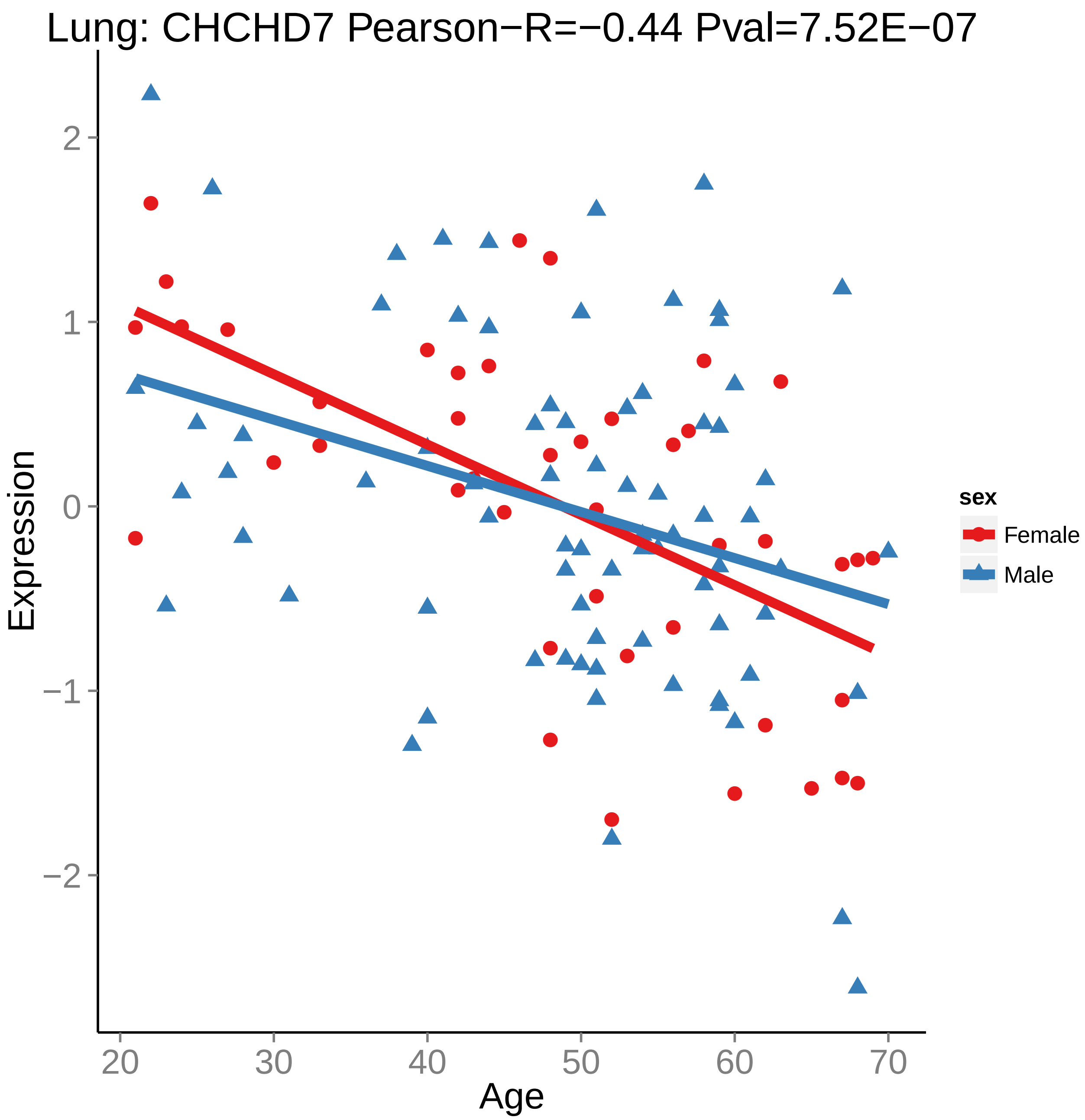

Lung: CHCHD7 Pearson−R=−0.44 Pval=7.52E−07
Expression
2
1
0
−1
−2
20
30
40
50
60
70
Age
sex
Female
Male

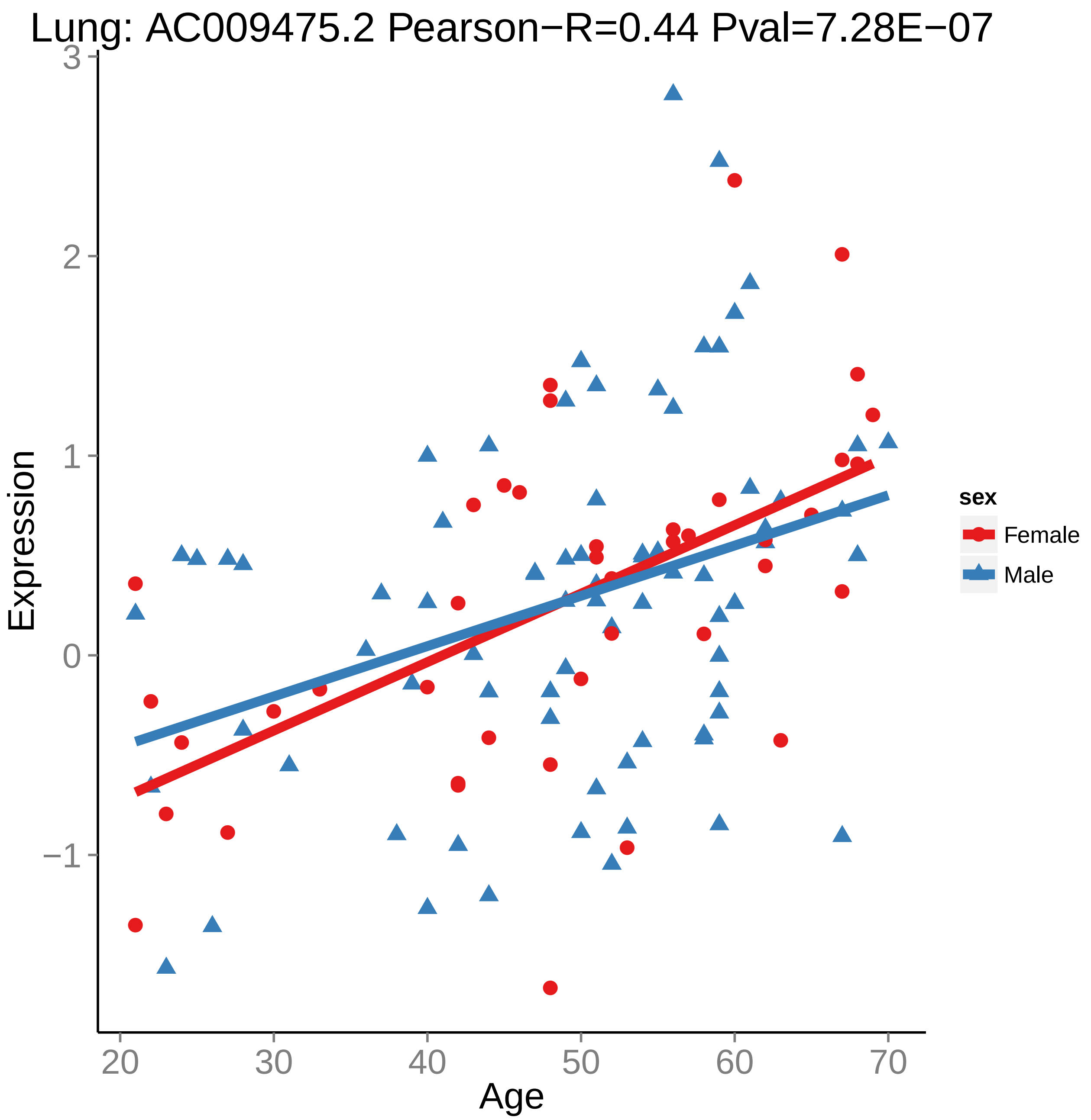

Lung: AC009475.2 Pearson−R=0.44 Pval=7.28E−07
Expression
Age
sex
Female
Male
3
2
1
0
−1
20
30
40
50
60
70

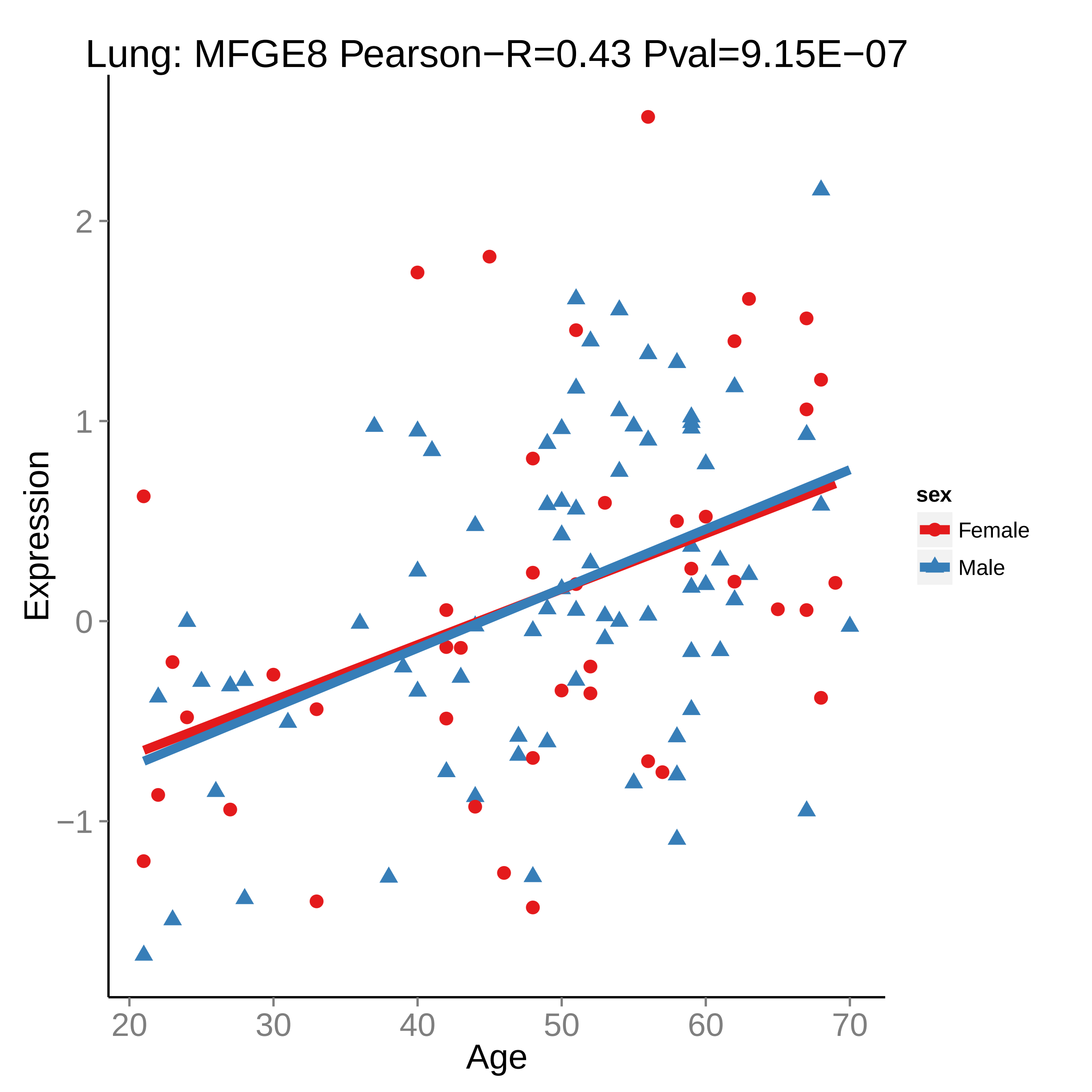

Lung: MFGE8 Pearson−R=0.43 Pval=9.15E−07
Expression
2
1
0
−1
20
30
40
50
60
70
Age
sex
Female
Male

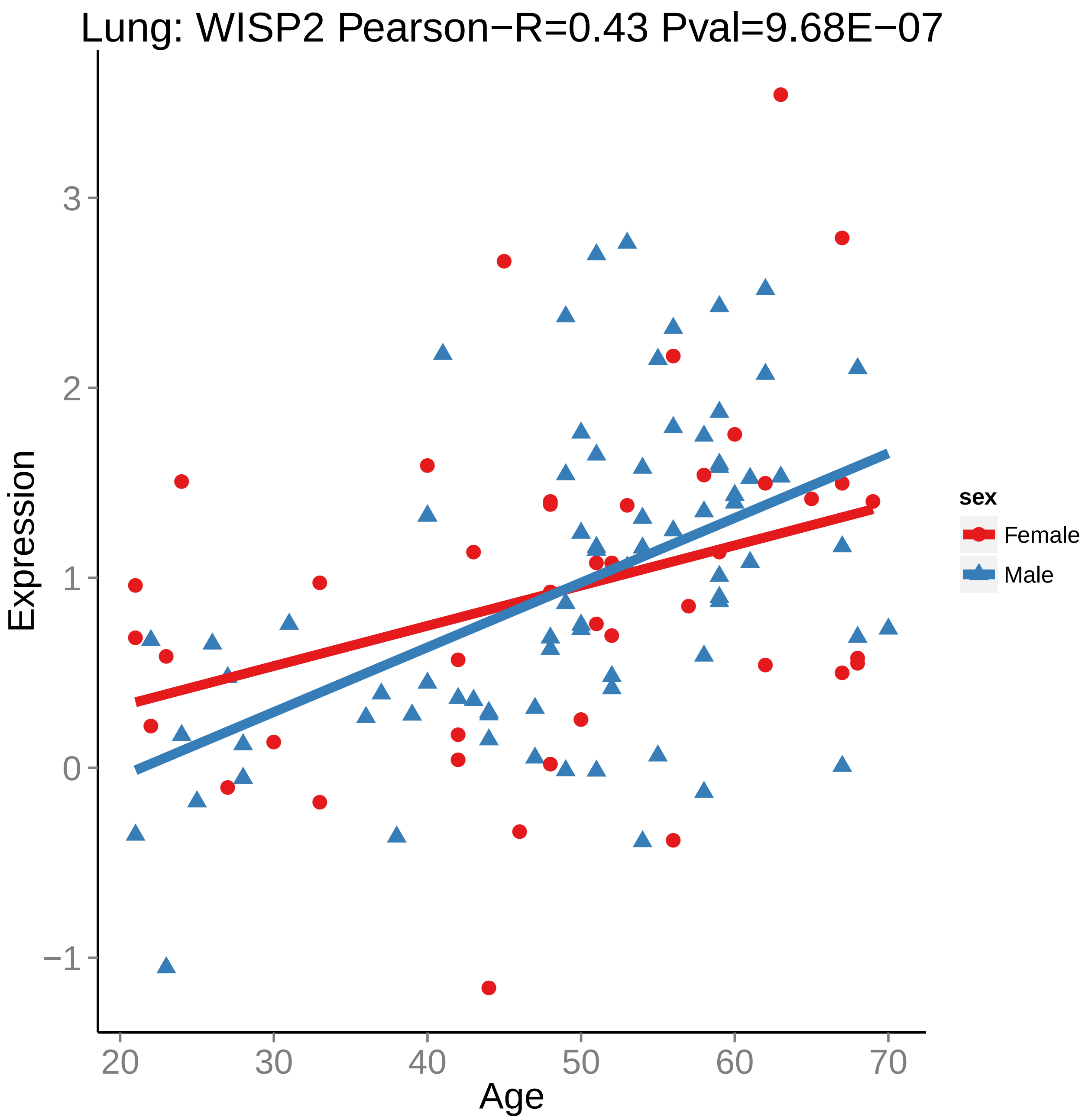

Lung: WISP2 Pearson−R=0.43 Pval=9.68E−07
Expression
Age
3
2
1
0
−1
20
30
40
50
60
70
sex
Female
Male

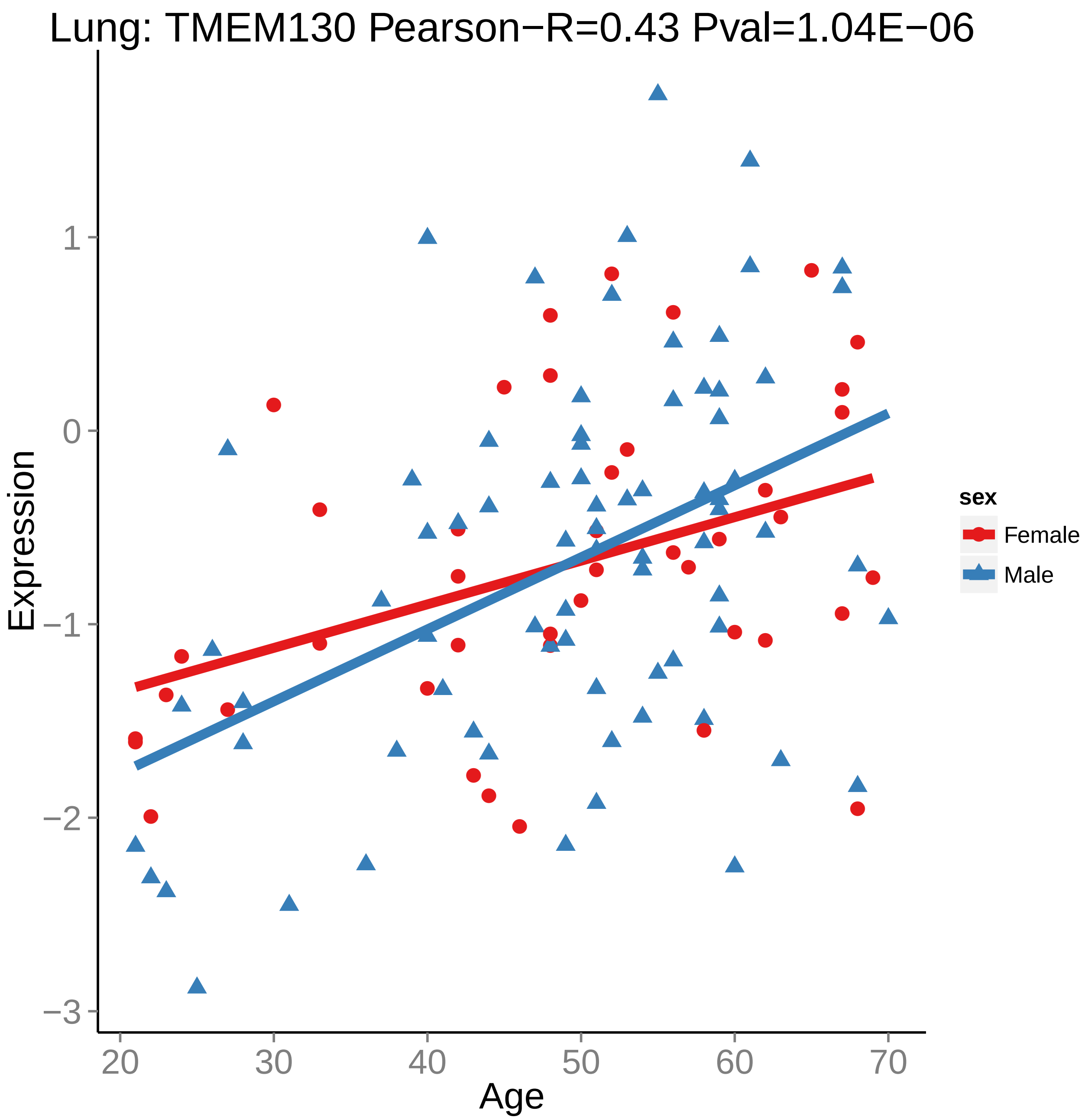

Lung: TMEM130 Pearson−R=0.43 Pval=1.04E−06
Expression
1
0
−1
−2
−3
20
30
40
50
60
70
Age
sex
Female
Male

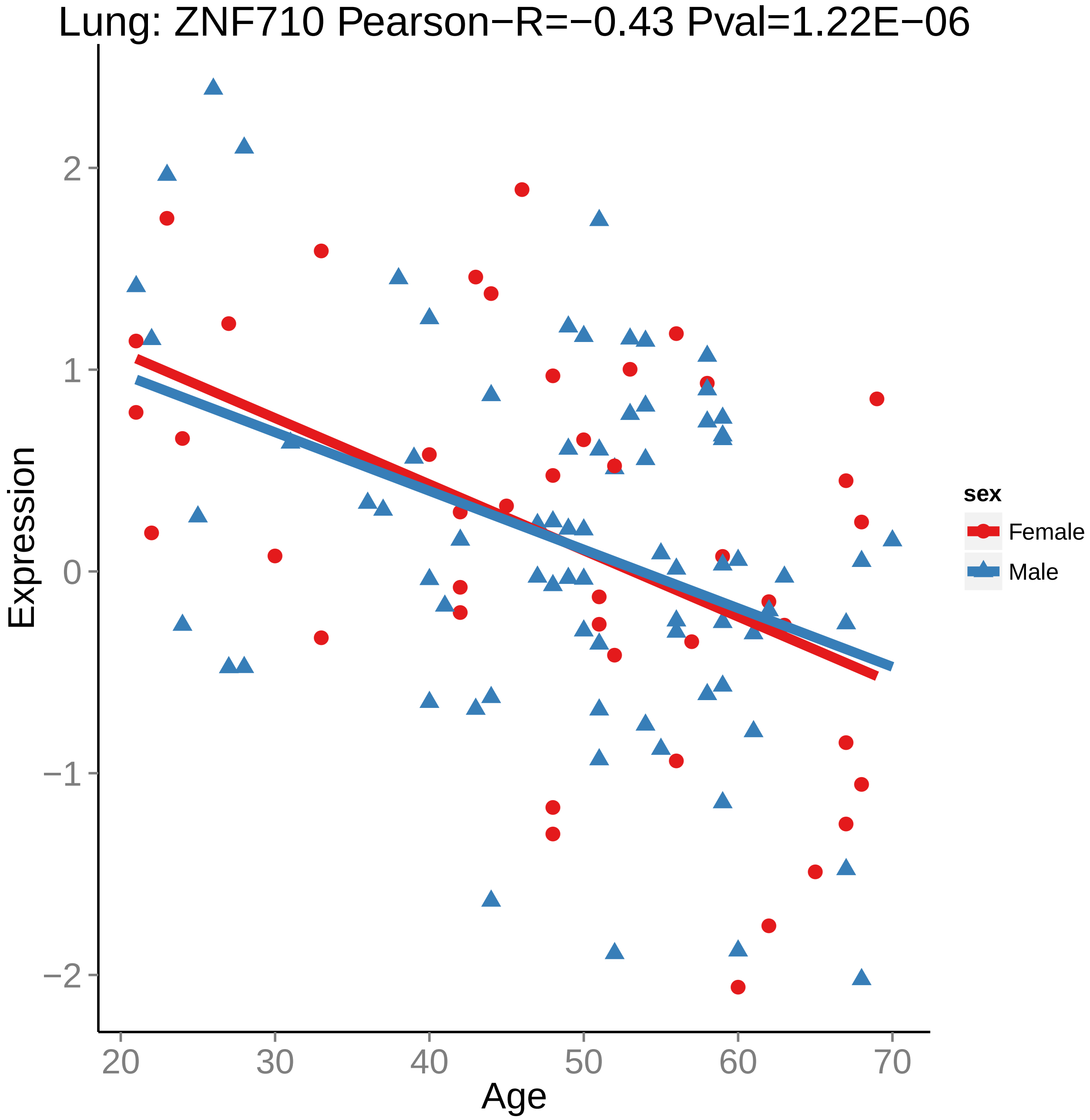
Lung: ZNF710 Pearson−R=−0.43 Pval=1.22E−06
Expression
2
1
0
−1
−2
20
30
40
50
60
70
Age
sex
Female
Male

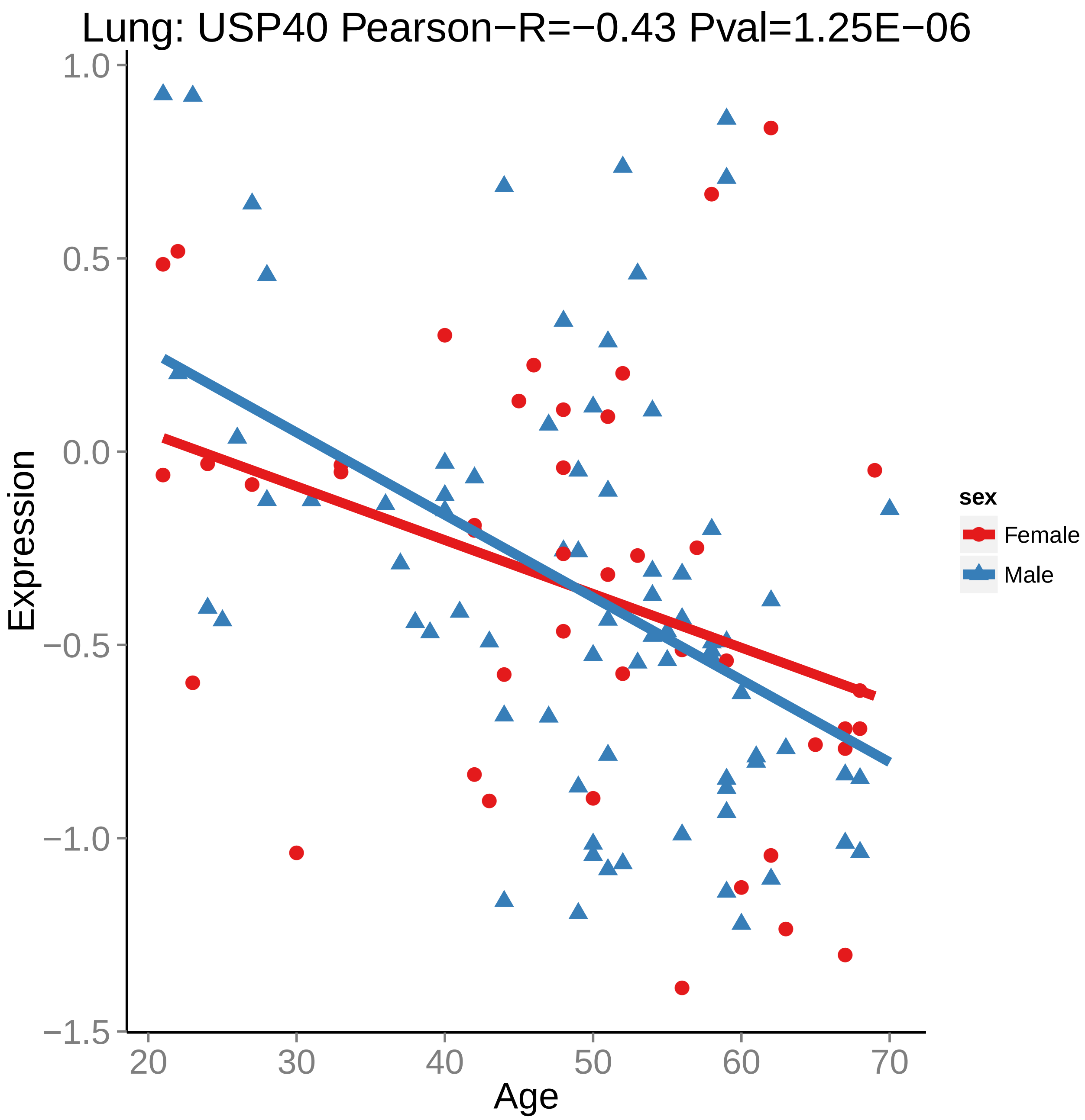

Lung: USP40 Pearson−R=−0.43 Pval=1.25E−06
1.0
0.5
0.0
−0.5
−1.0
−1.5
Expression
20
30
40
50
60
70
Age
sex
Female
Male

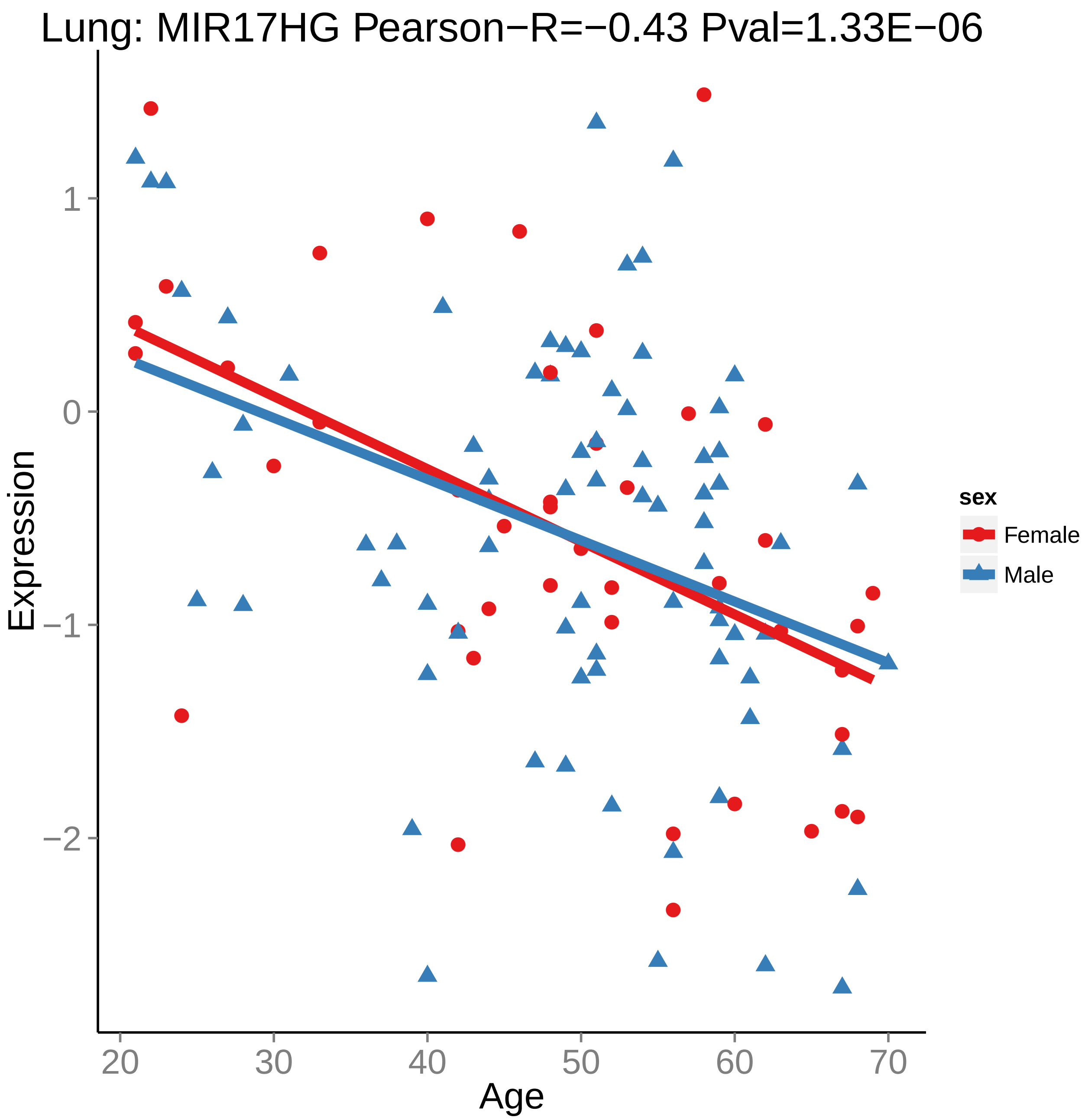

Lung: MIR17HG Pearson−R=−0.43 Pval=1.33E−06
Expression
1
0
−1
−2
20
30
40
50
60
70
Age
sex
Female
Male

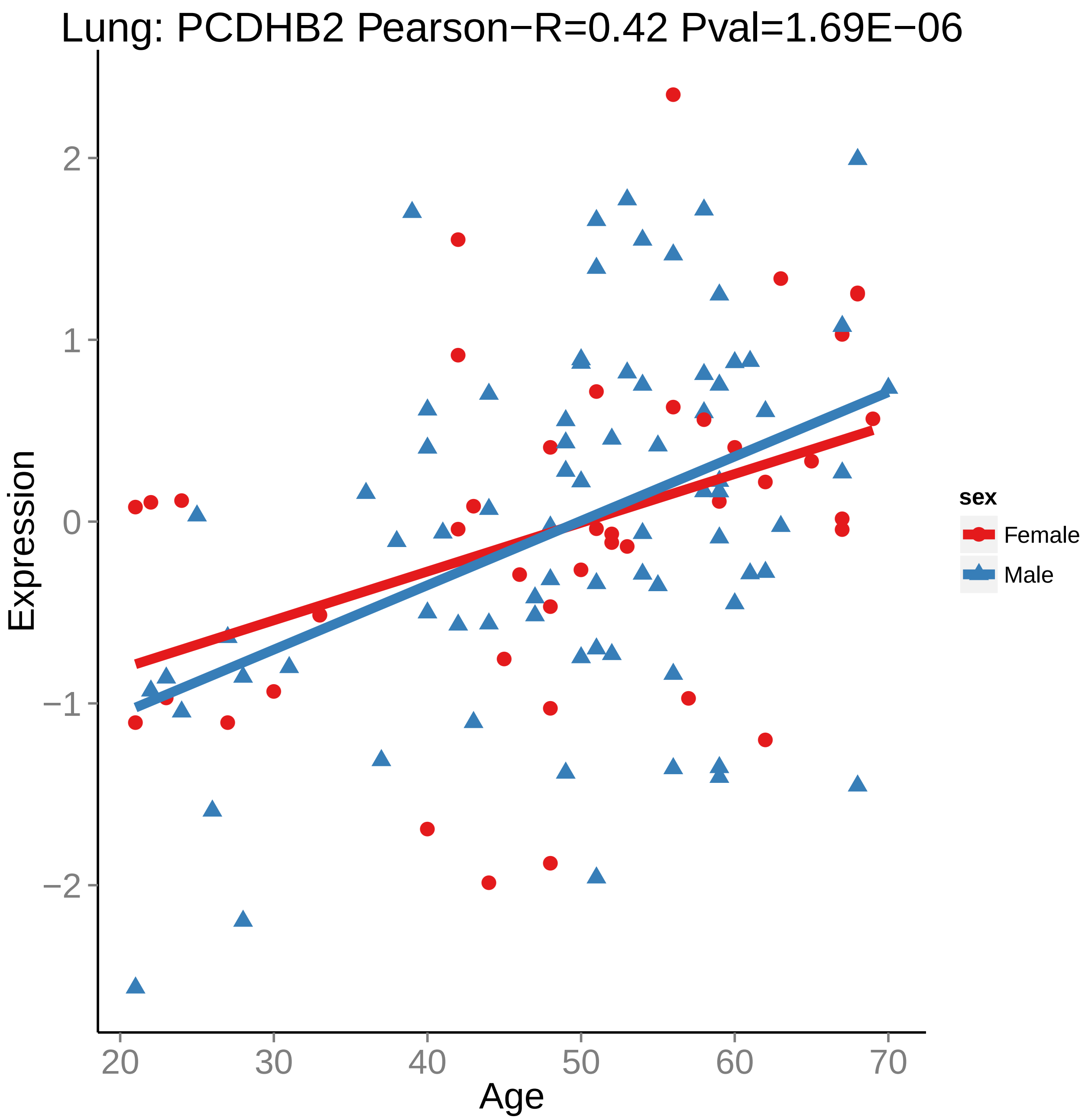

Lung: PCDHB2 Pearson−R=0.42 Pval=1.69E−06
Expression
2
1
0
−1
−2
20
30
40
50
60
70
Age
sex
Female
Male

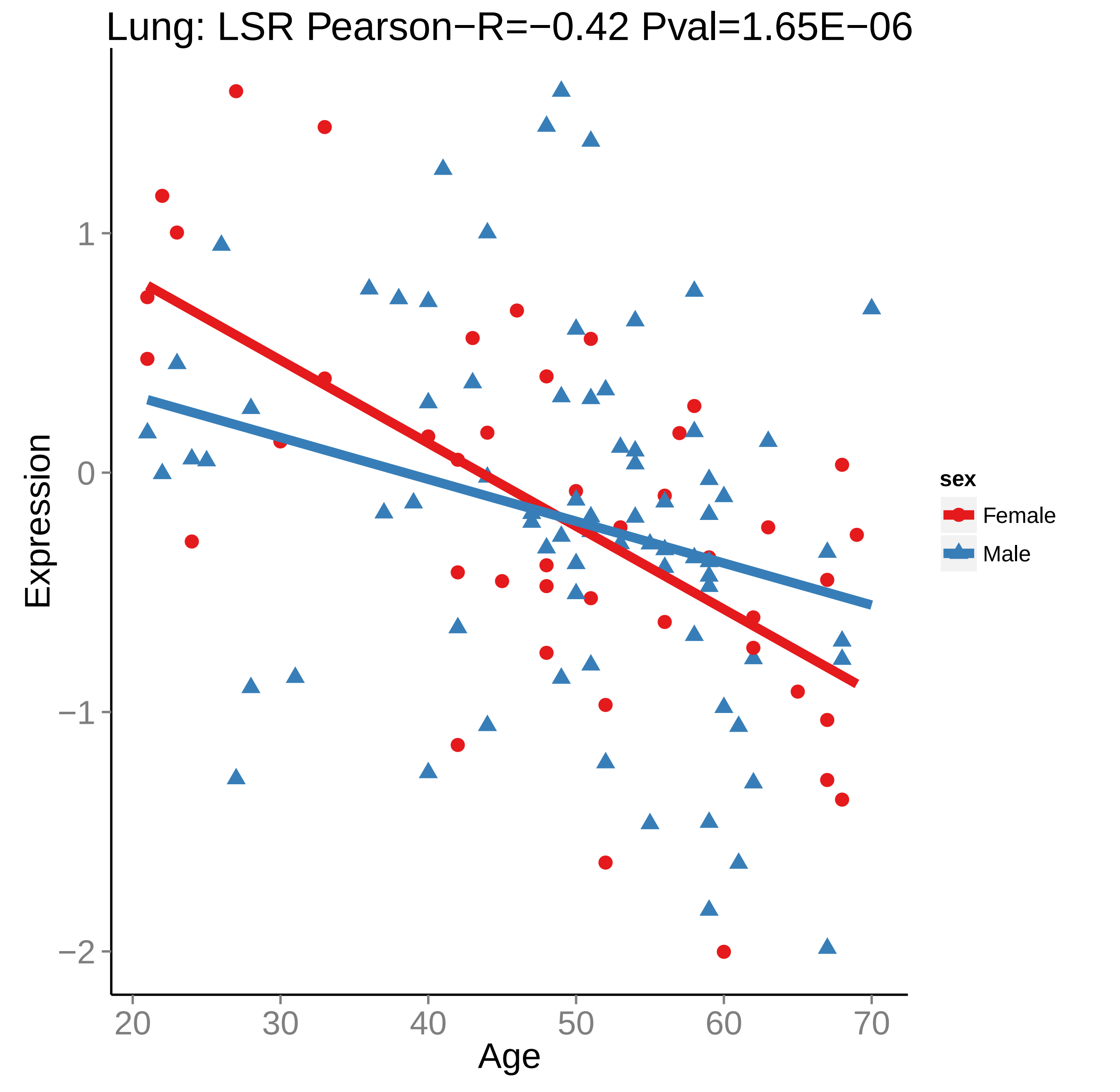

Lung: LSR Pearson−R=−0.42 Pval=1.65E−06
Expression
Age
1
0
−1
−2
20
30
40
50
60
70
sex
Female
Male

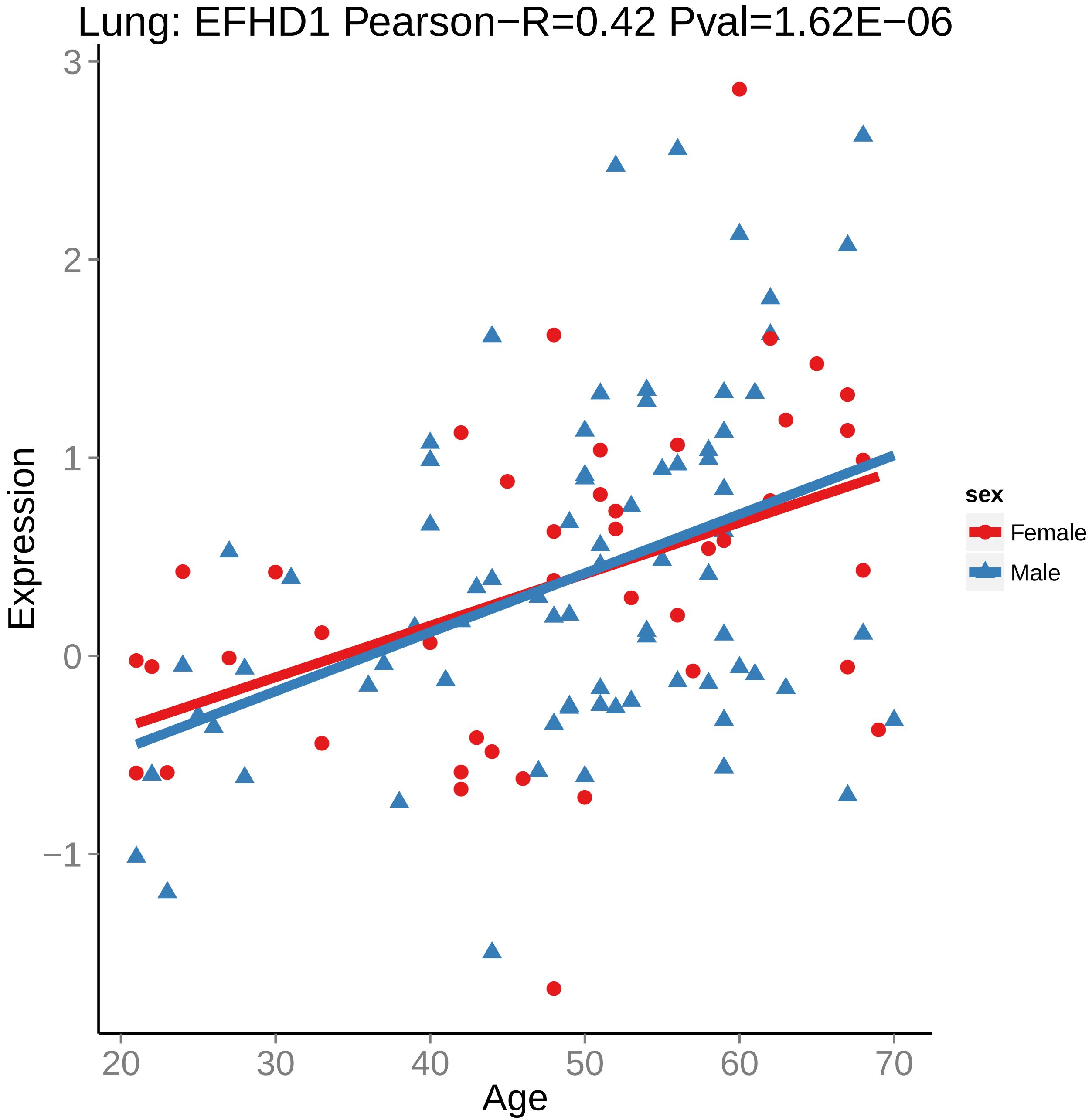

Lung: EFHD1 Pearson−R=0.42 Pval=1.62E−06
Expression
3
2
1
0
−1
20
30
40
50
60
70
Age
sex
Female
Male

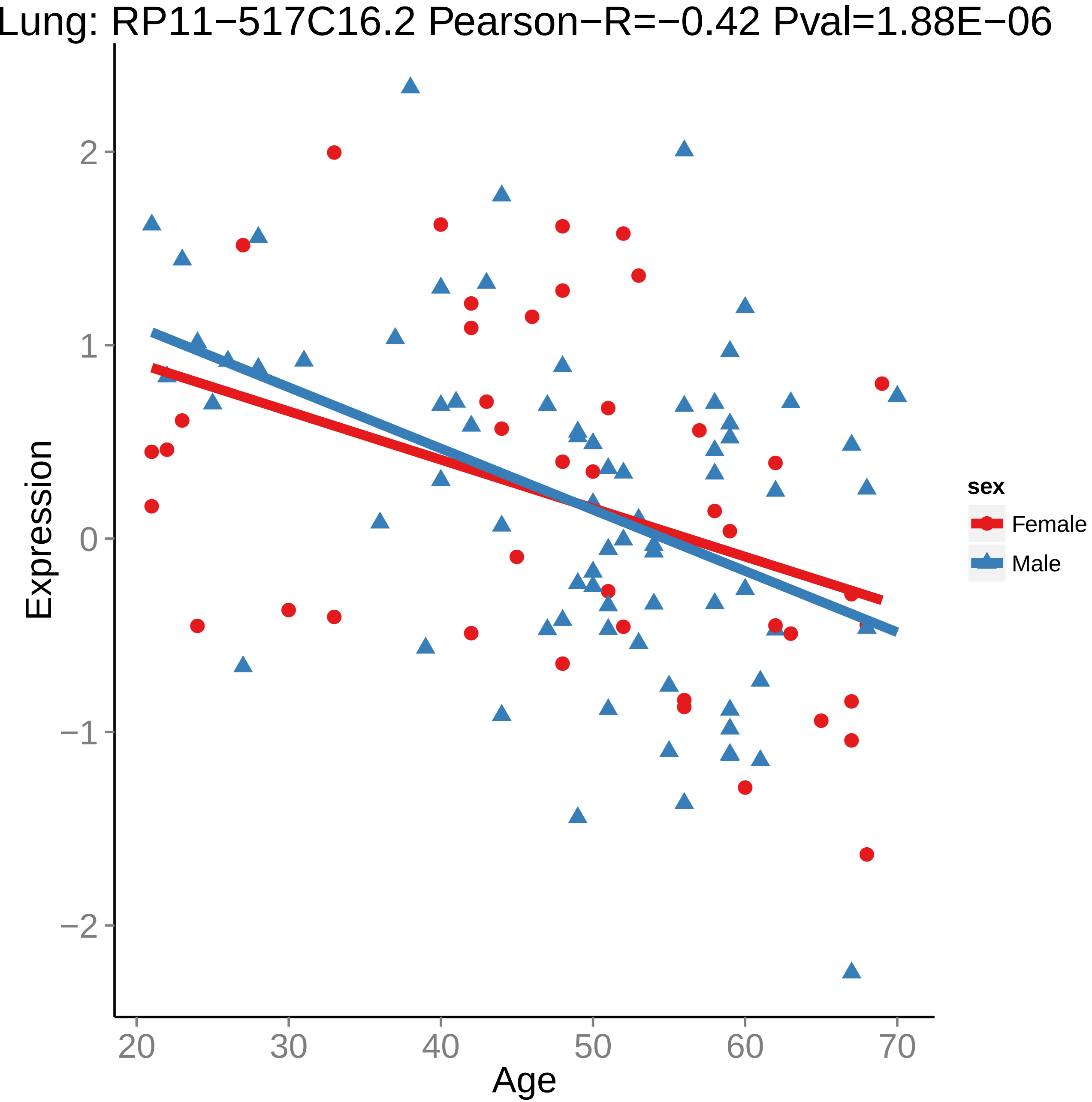

Lung: RP11−517C16.2 Pearson−R=−0.42 Pval=1.88E−06
Expression
Age
2
1
0
−1
−2
20
30
40
50
60
70
sex
Female
Male

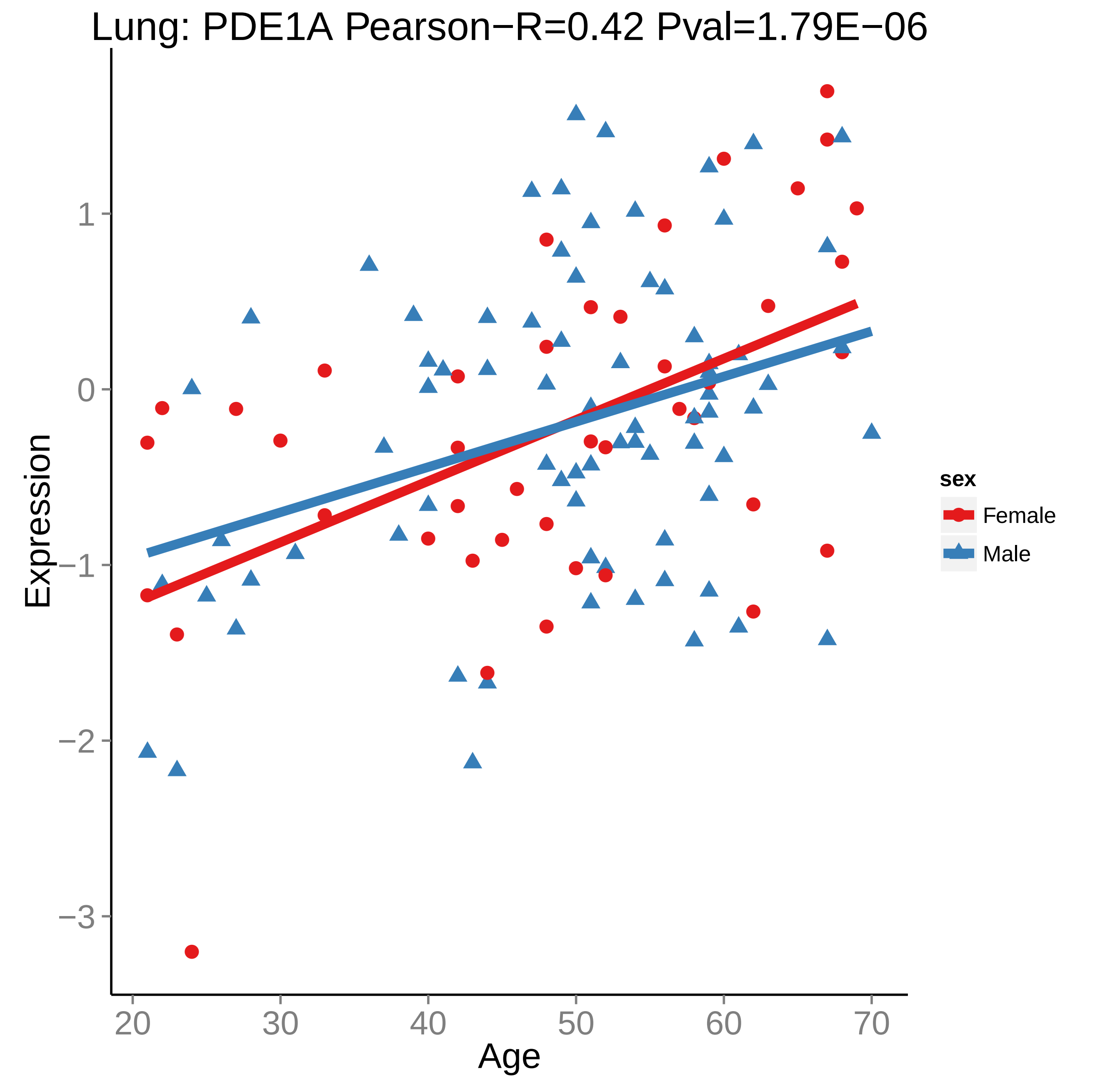

Lung: PDE1A Pearson−R=0.42 Pval=1.79E−06
Expression
1
0
−1
−2
−3
20
30
40
50
60
70
Age
sex
Female
Male

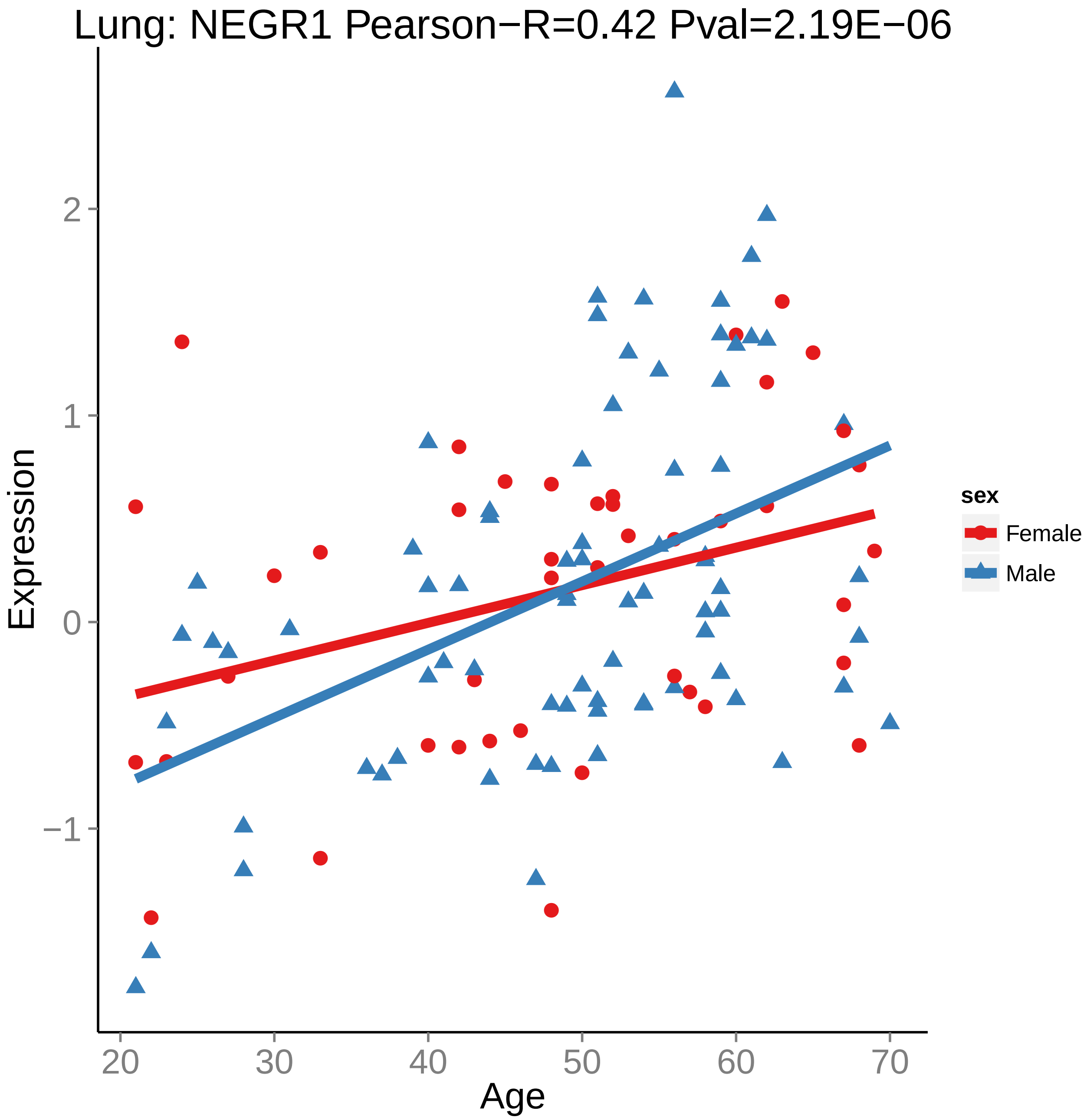

Lung: NEGR1 Pearson−R=0.42 Pval=2.19E−06
Expression
2
1
0
−1
20
30
40
50
60
70
Age
sex
Female
Male

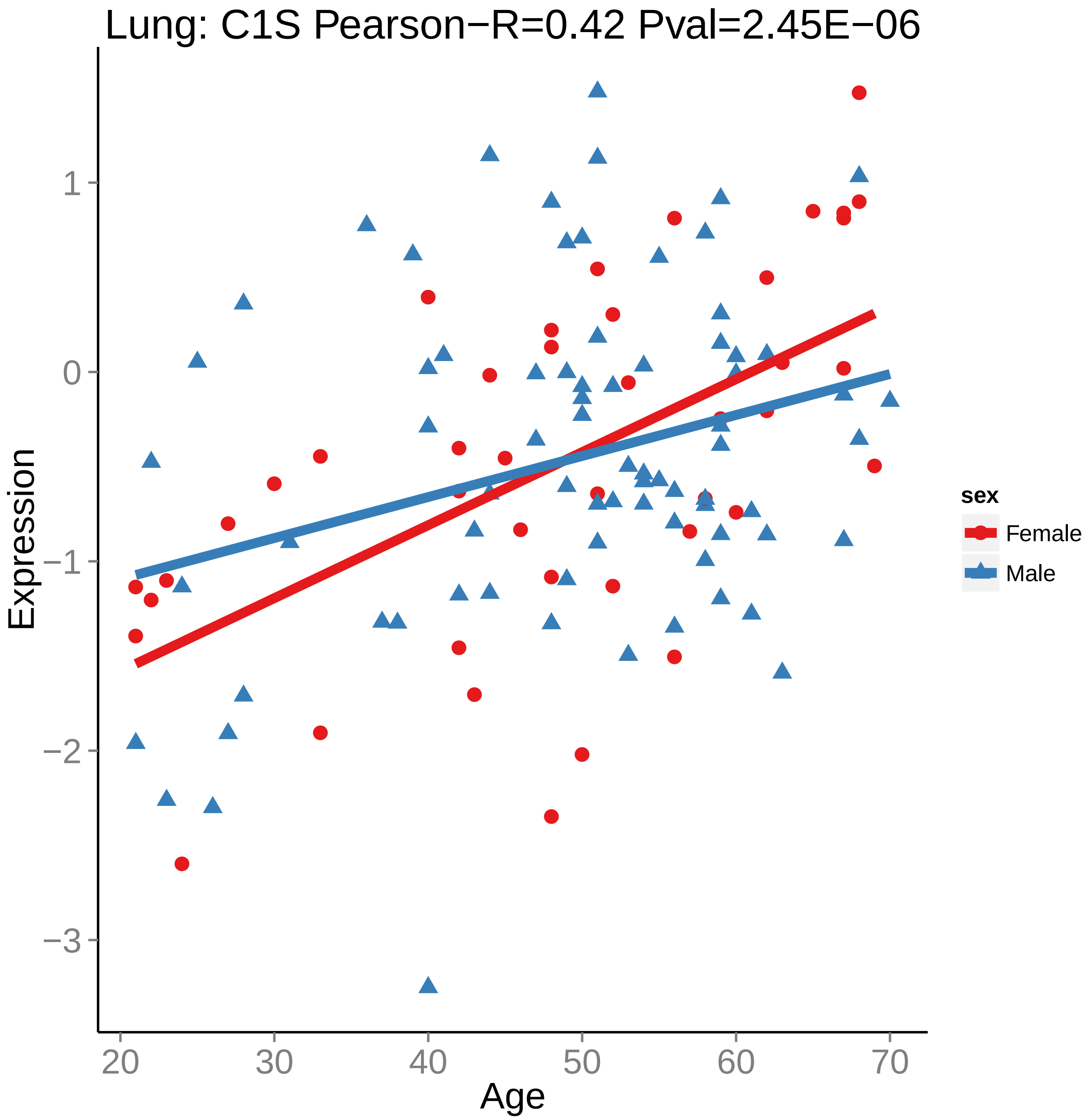

Lung: C1S Pearson−R=0.42 Pval=2.45E−06
Expression
1
0
−1
−2
−3
20
30
40
50
60
70
Age
sex
Female
Male

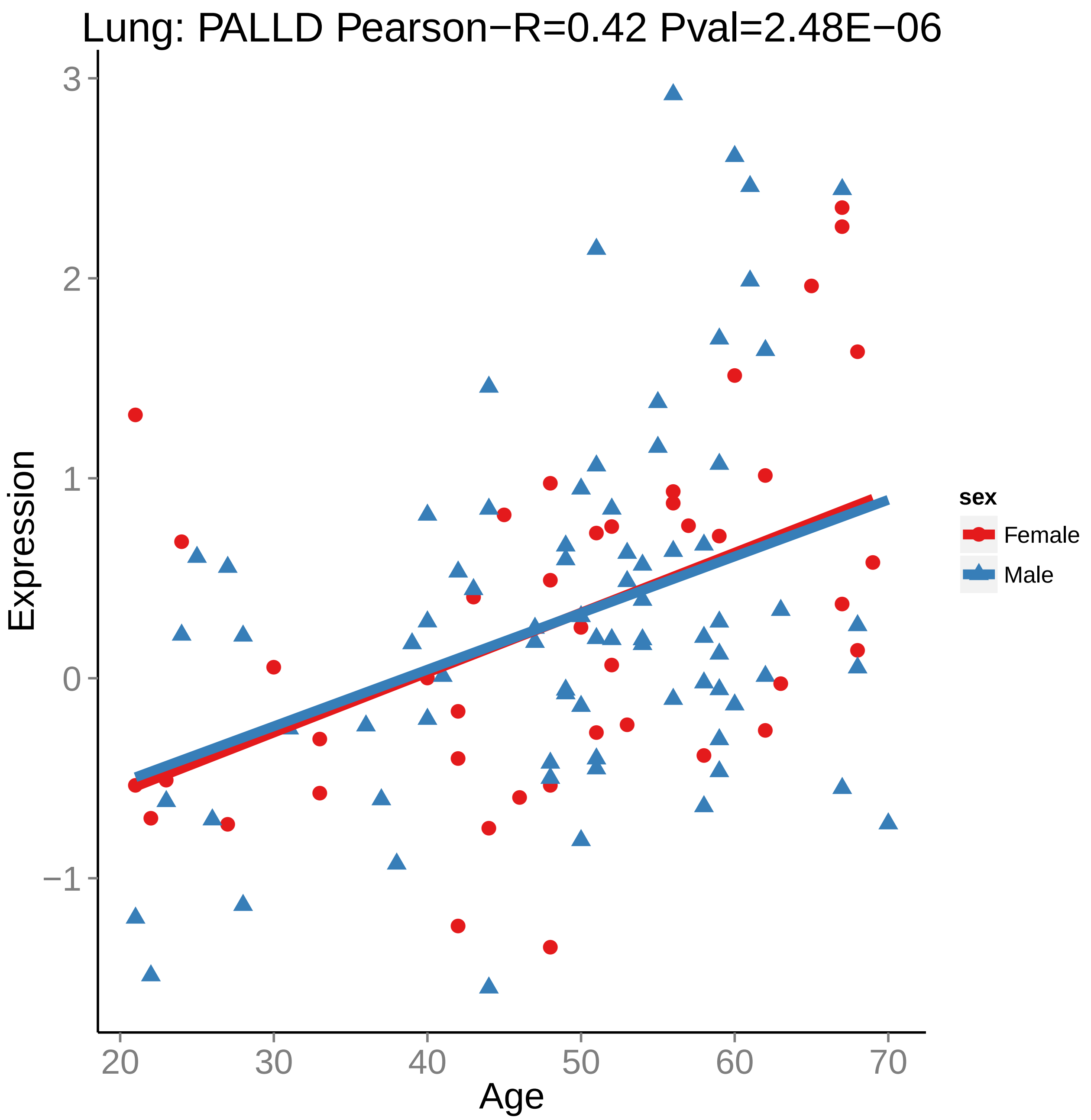

Lung: PALLD Pearson−R=0.42 Pval=2.48E−06
Expression
Age
sex
Female
Male
3
2
1
0
−1
20
30
40
50
60
70

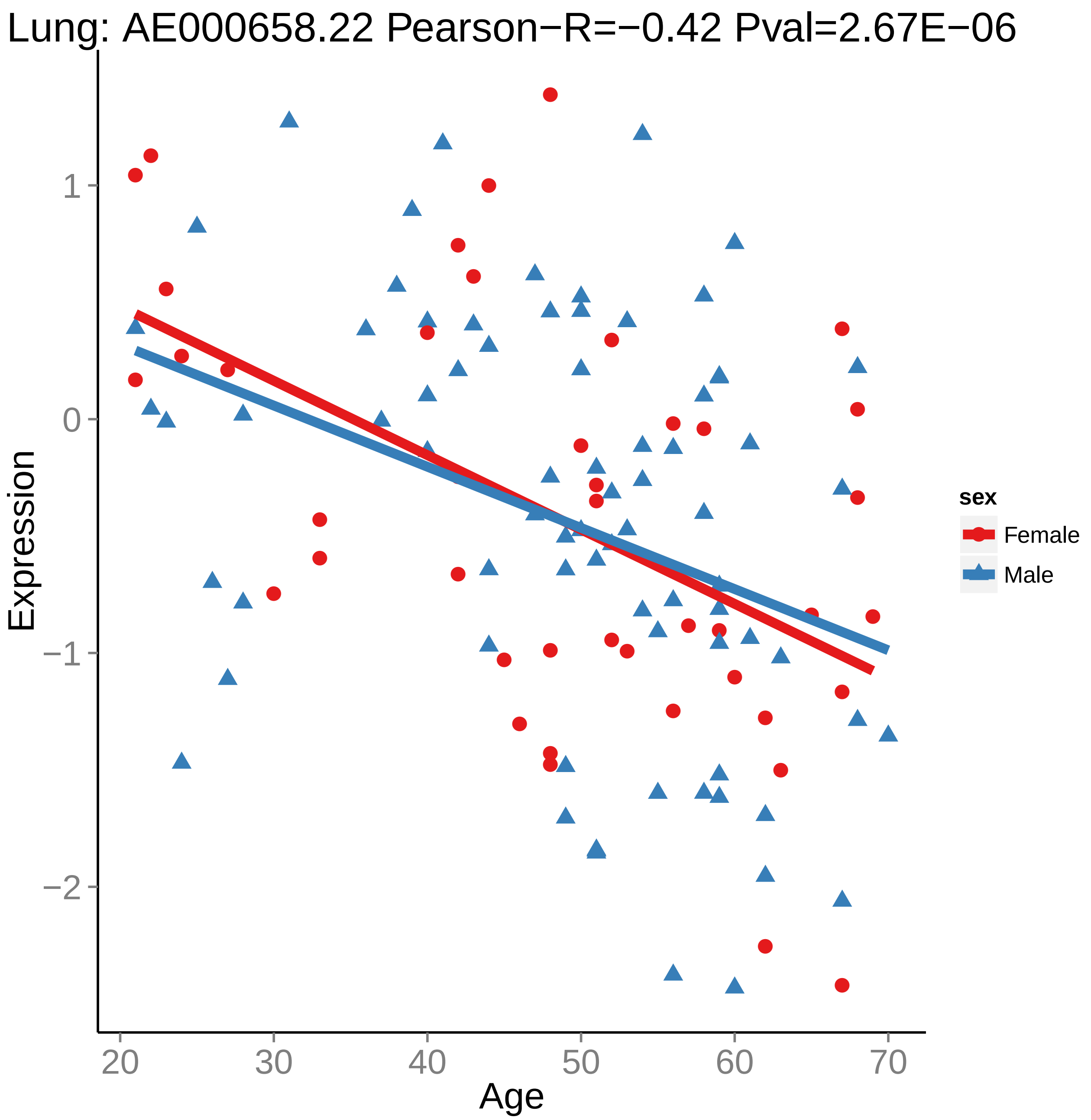

Lung: AE000658.22 Pearson−R=−0.42 Pval=2.67E−06
Expression
Age
1
0
−1
−2
20
30
40
50
60
70
sex
Female
Male

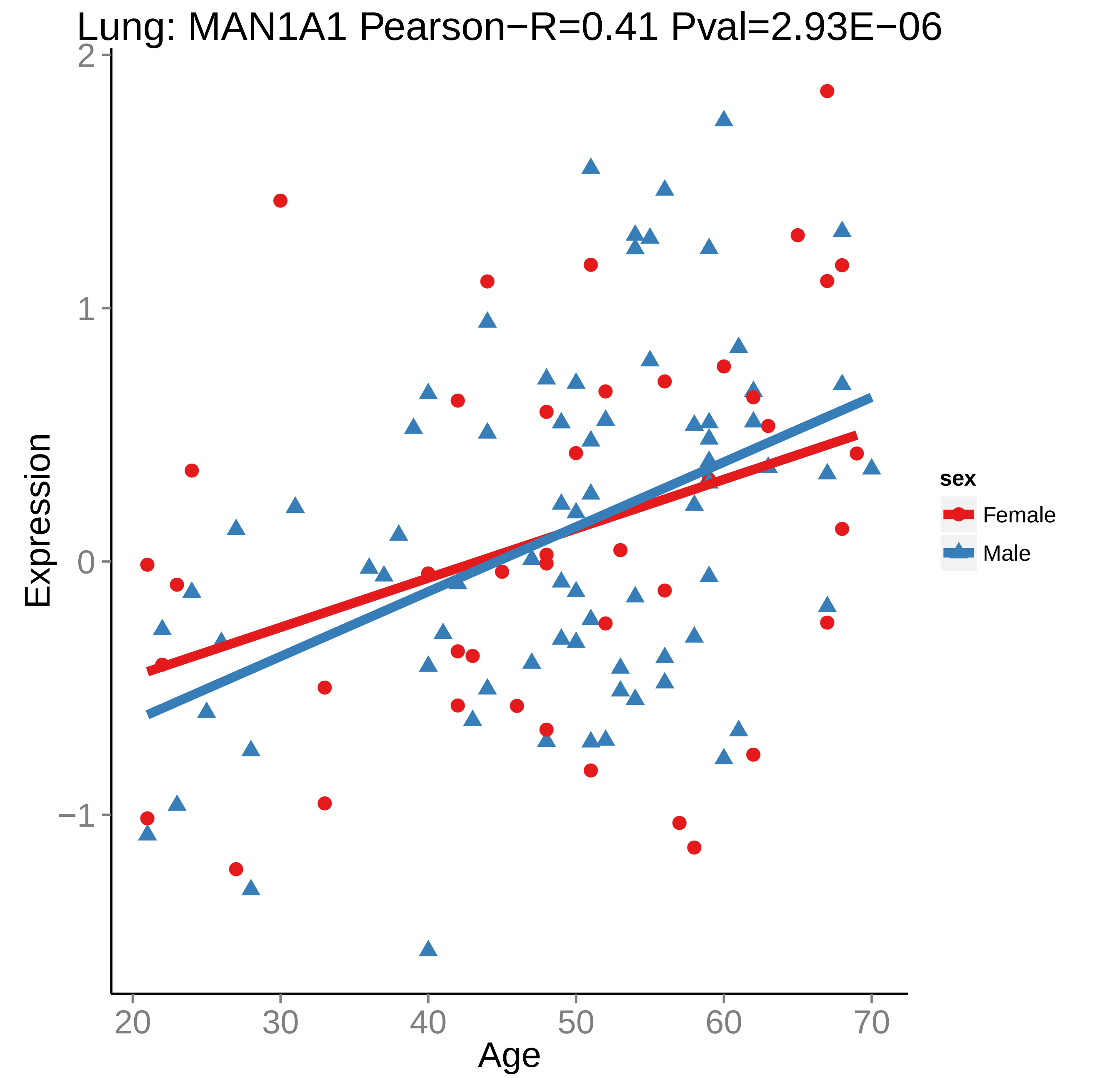

Lung: MAN1A1 Pearson−R=0.41 Pval=2.93E−06
Expression
Age
2
1
0
−1
20
30
40
50
60
70
sex
Female
Male

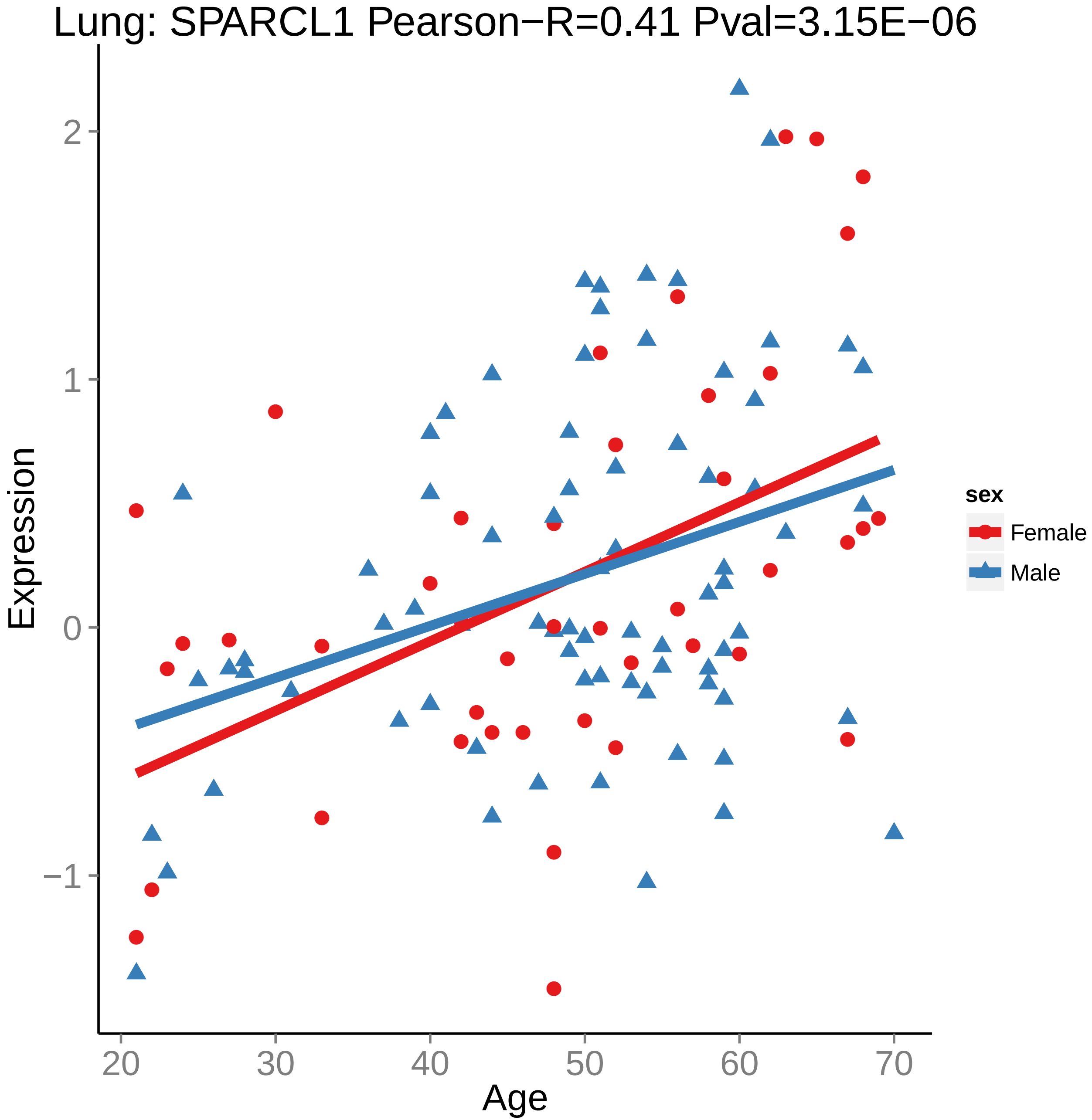

Lung: SPARCL1 Pearson−R=0.41 Pval=3.15E−06
Expression
Age
2
1
0
−1
20
30
40
50
60
70
sex
Female
Male

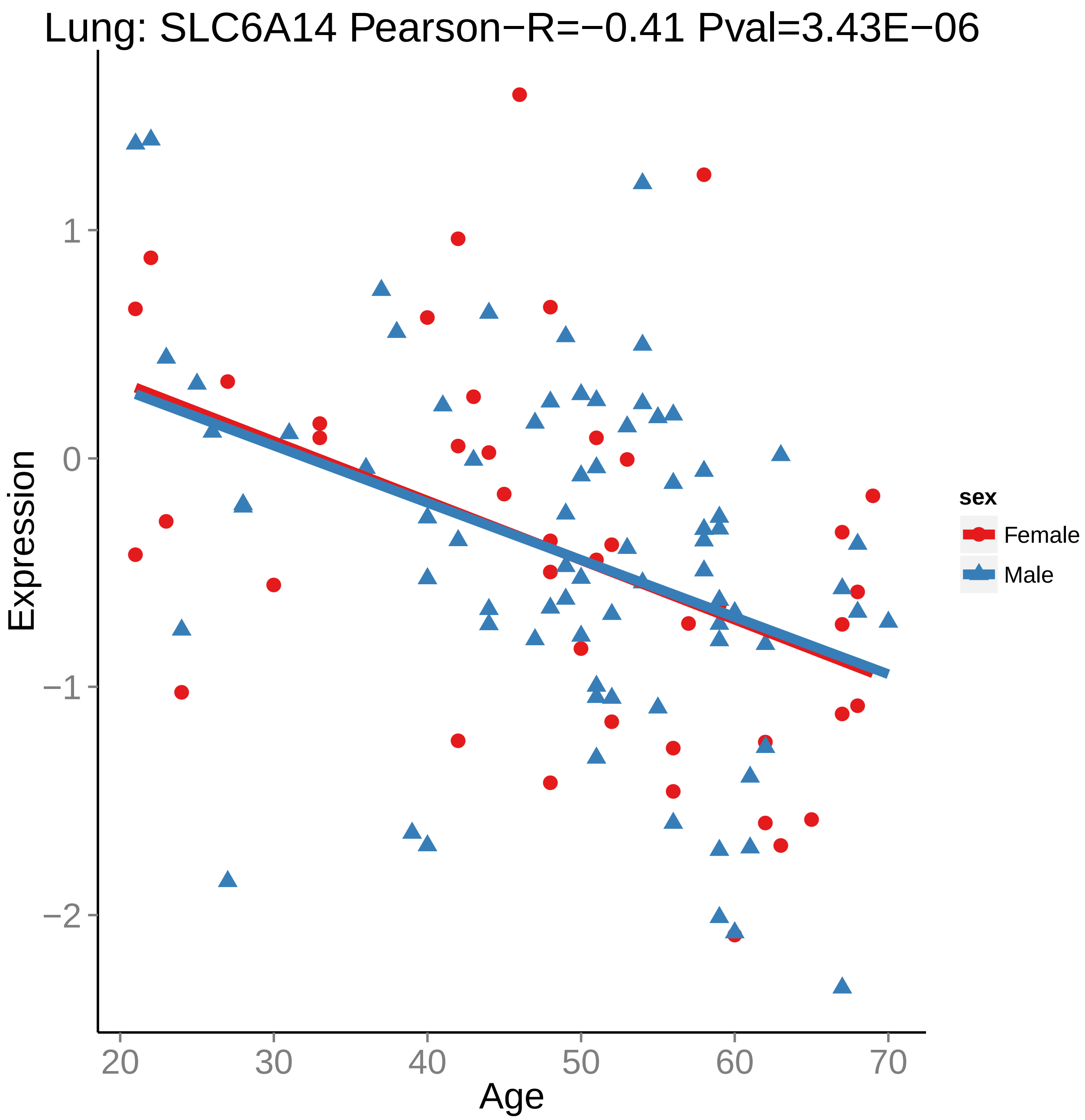

Lung: SLC6A14 Pearson−R=−0.41 Pval=3.43E−06
Expression
Age
1
0
−1
−2
20
30
40
50
60
70
sex
Female
Male

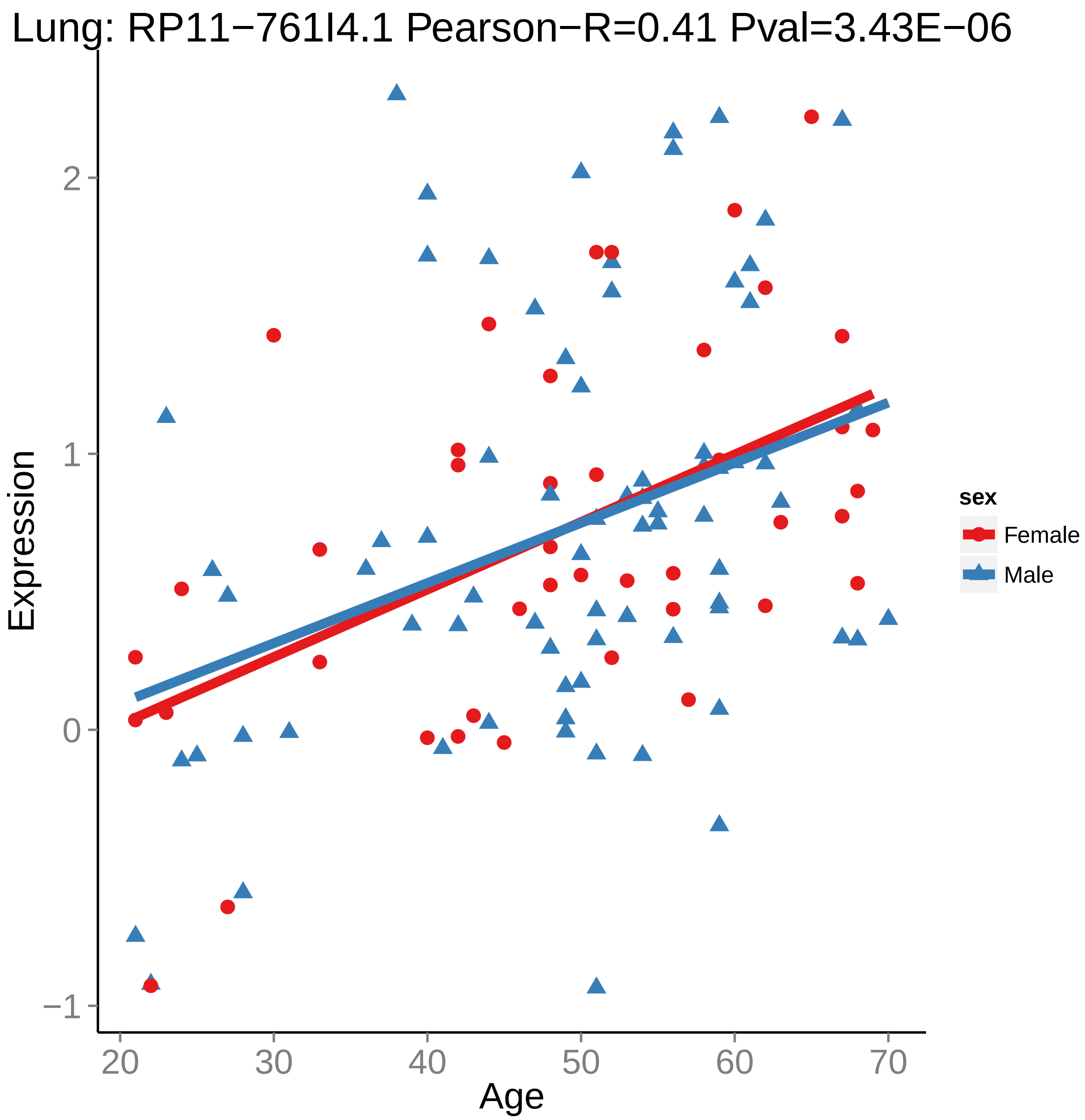

Lung: RP11−761I4.1 Pearson−R=0.41 Pval=3.43E−06
Expression
Age
2
1
0
−1
20
30
40
50
60
70
sex
Female
Male

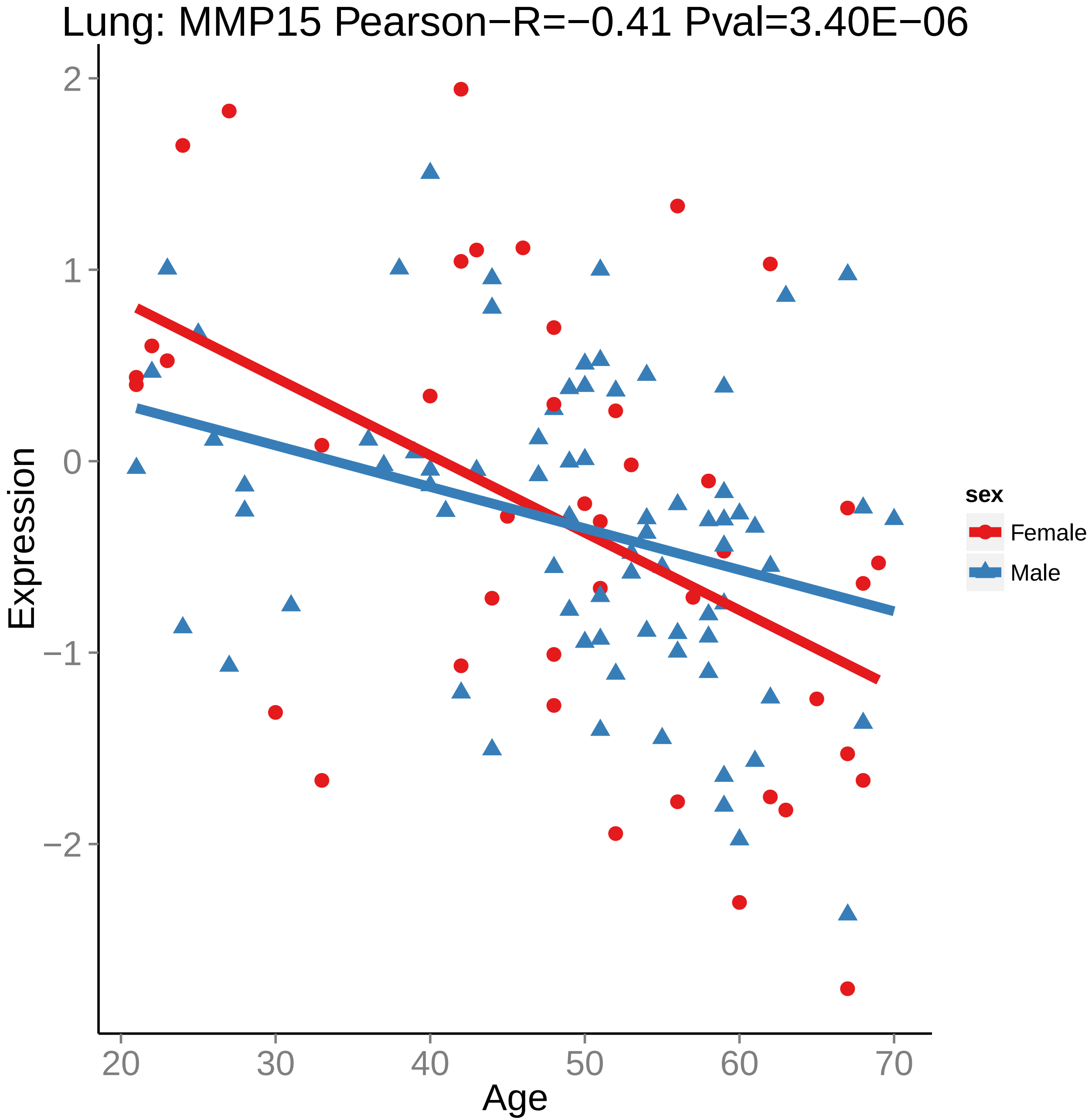

Lung: MMP15 Pearson−R=−0.41 Pval=3.40E−06
Expression
Age
2
1
0
−1
−2
20
30
40
50
60
70
sex
Female
Male

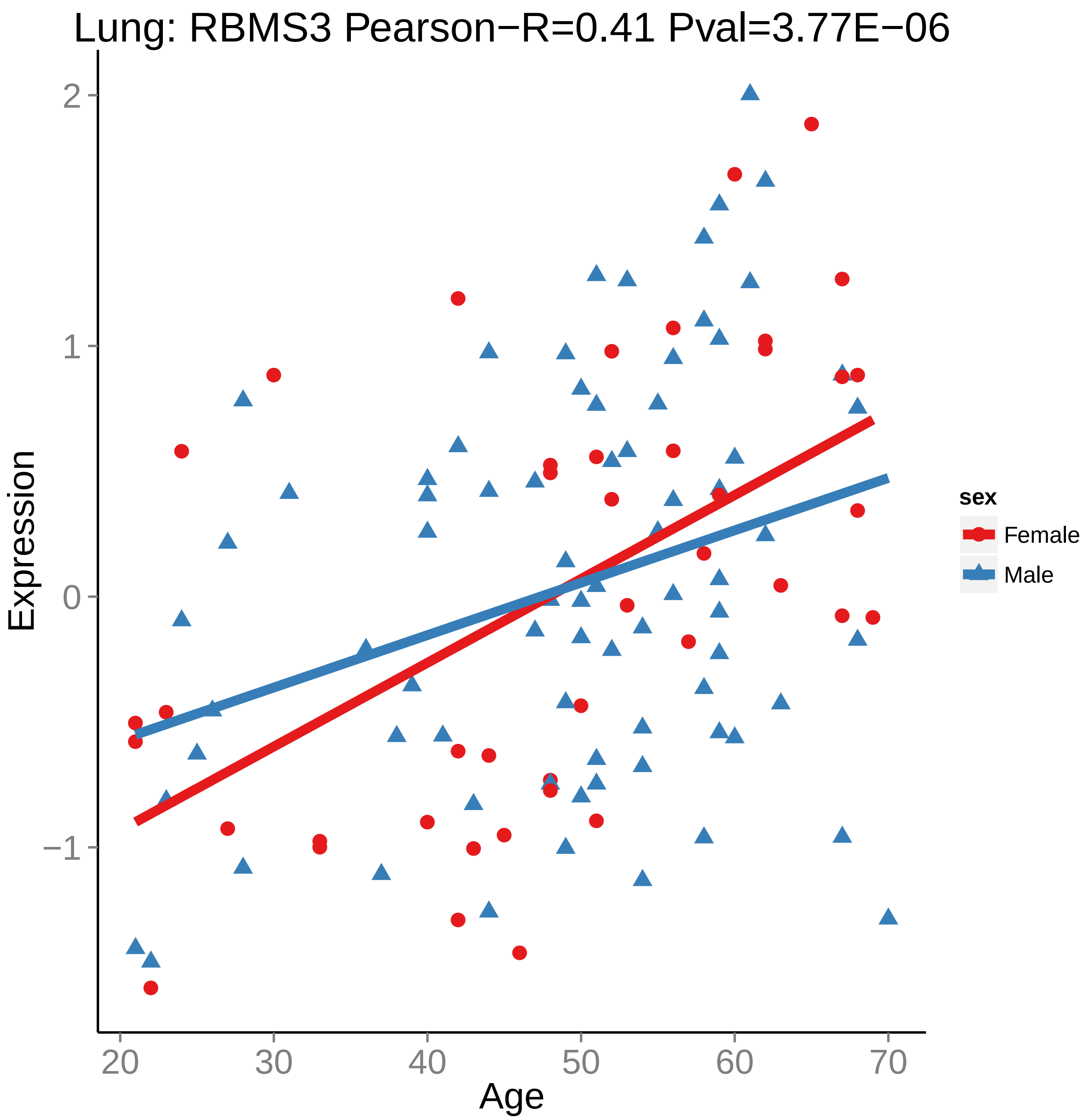

Lung: RBMS3 Pearson−R=0.41 Pval=3.77E−06
Expression
Age
2
1
0
−1
20
30
40
50
60
70
sex
Female
Male

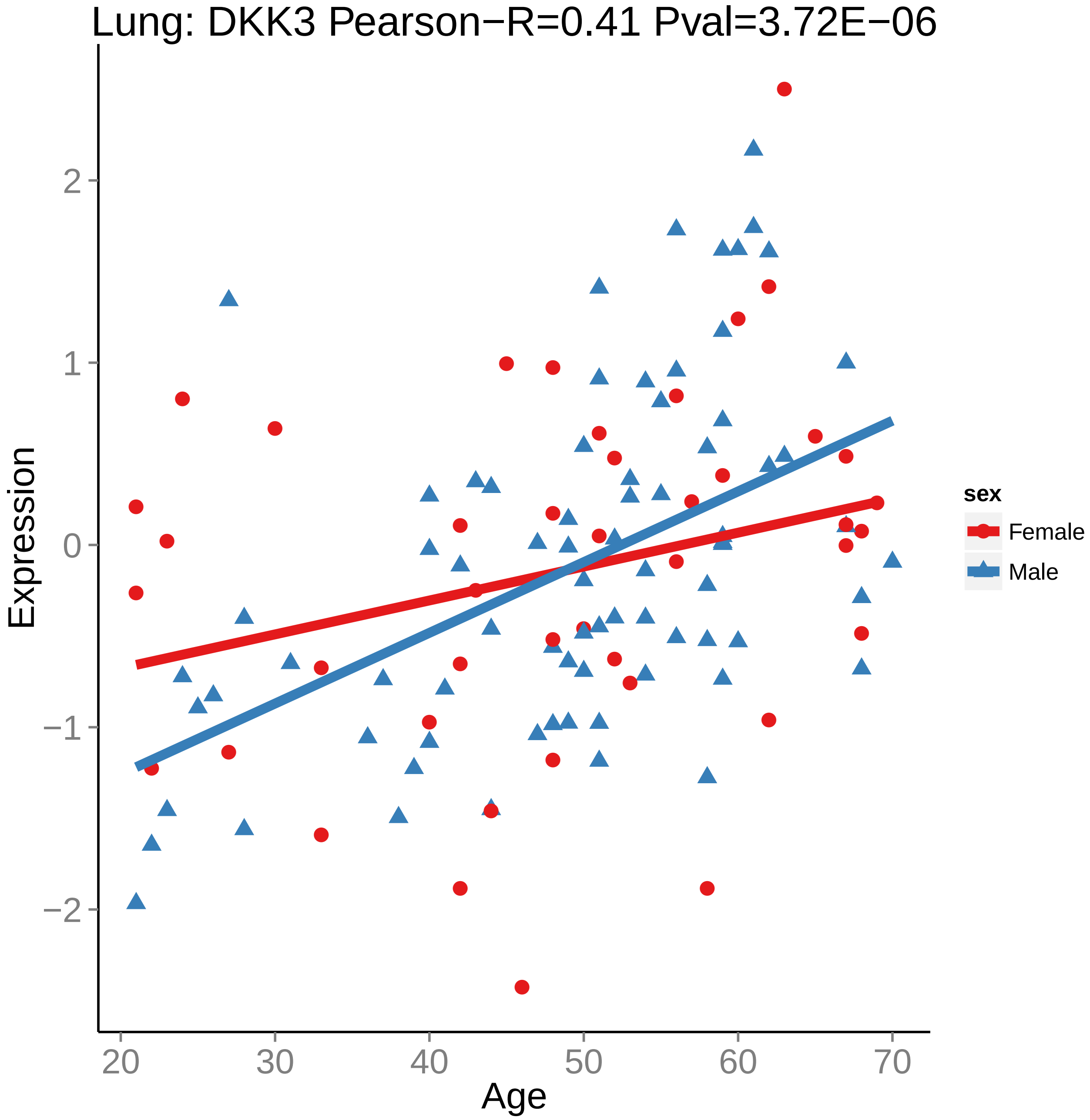

Lung: DKK3 Pearson−R=0.41 Pval=3.72E−06
Expression
Age
2
1
0
−1
−2
20
30
40
50
60
70
sex
Female
Male

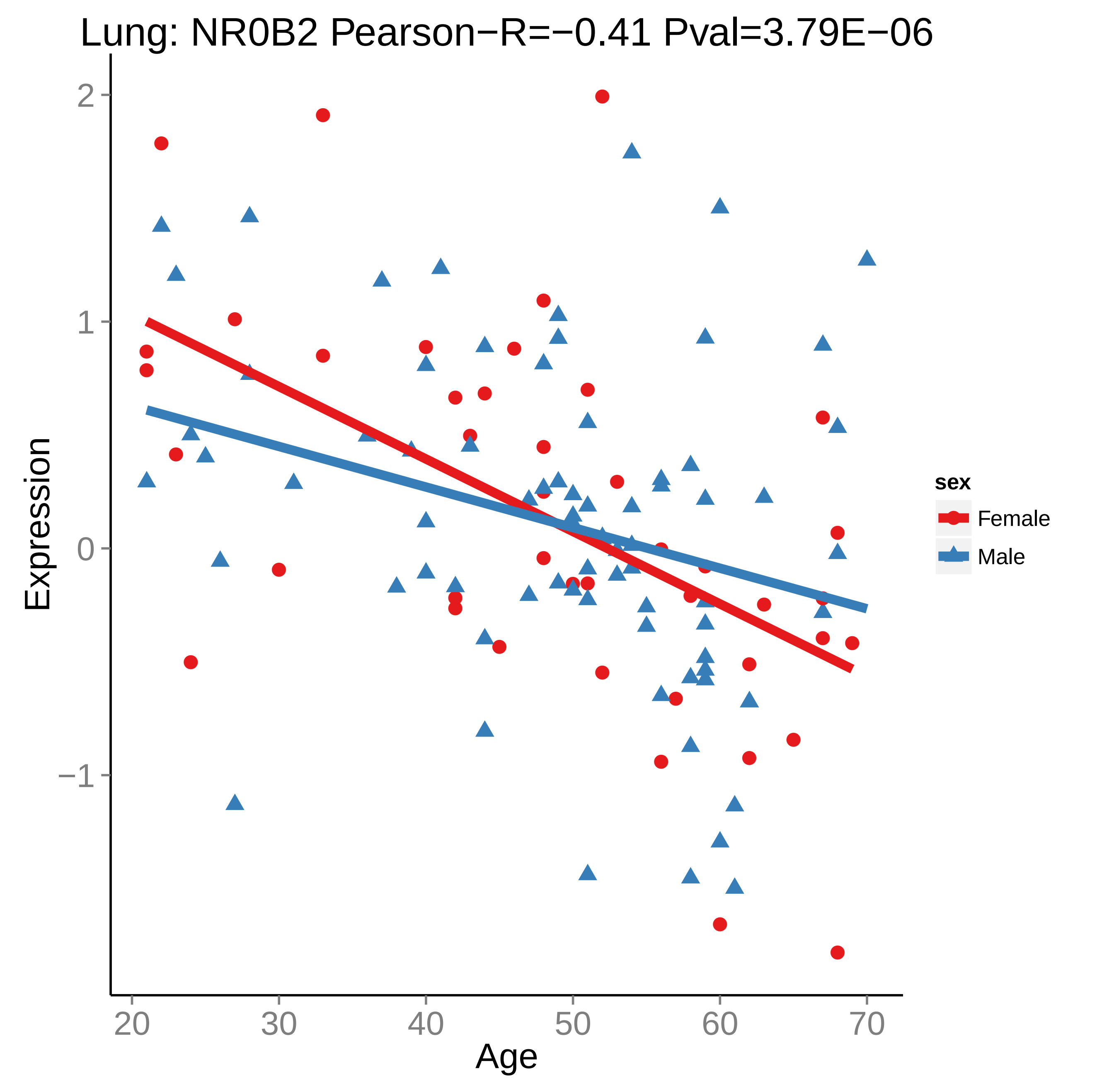

Lung: NR0B2 Pearson−R=−0.41 Pval=3.79E−06
Expression
Age
2
1
0
−1
20
30
40
50
60
70
sex
Female
Male

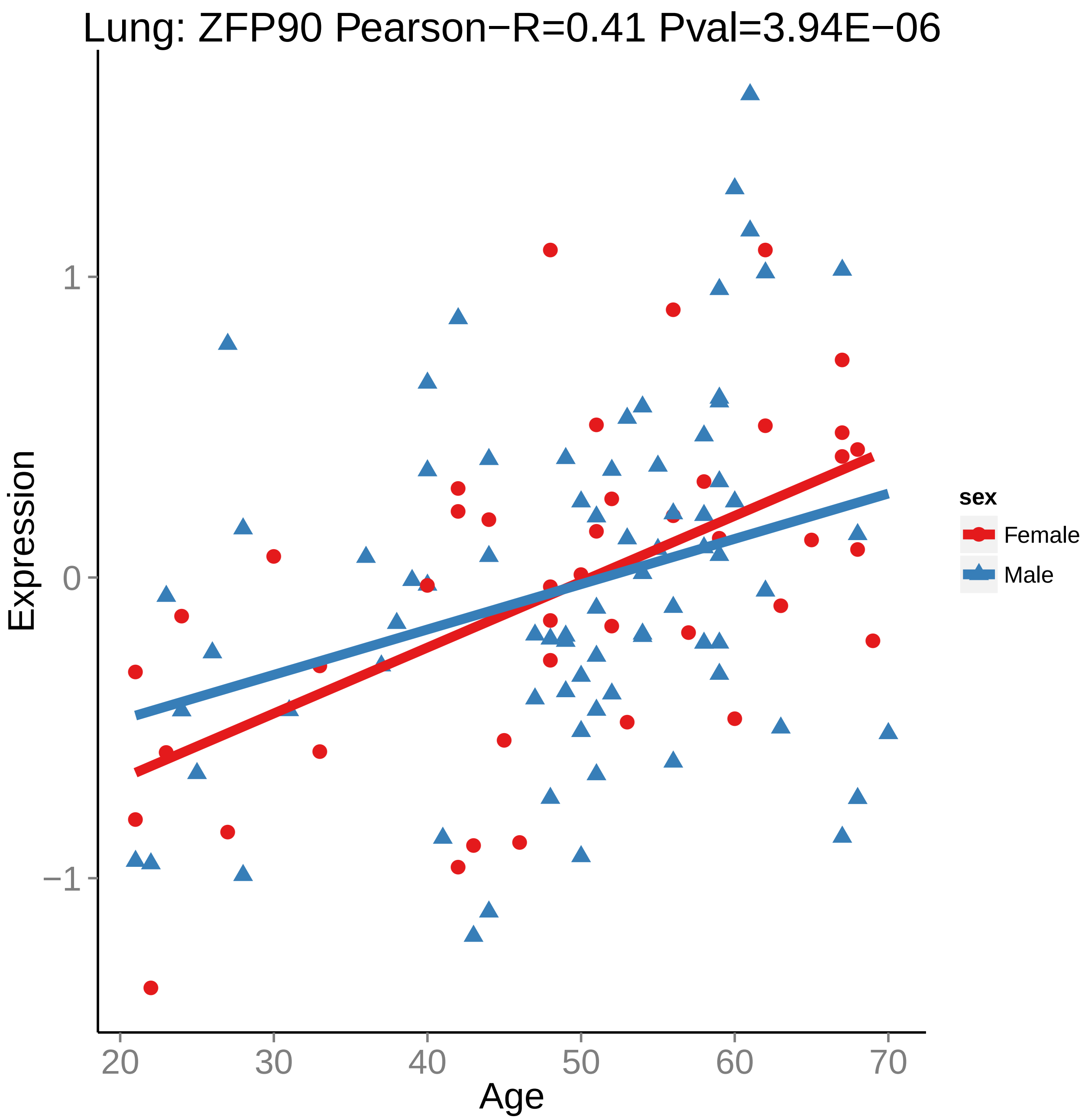

Lung: ZFP90 Pearson−R=0.41 Pval=3.94E−06
Expression
1
0
−1
20
30
40
50
60
70
Age
sex
Female
Male

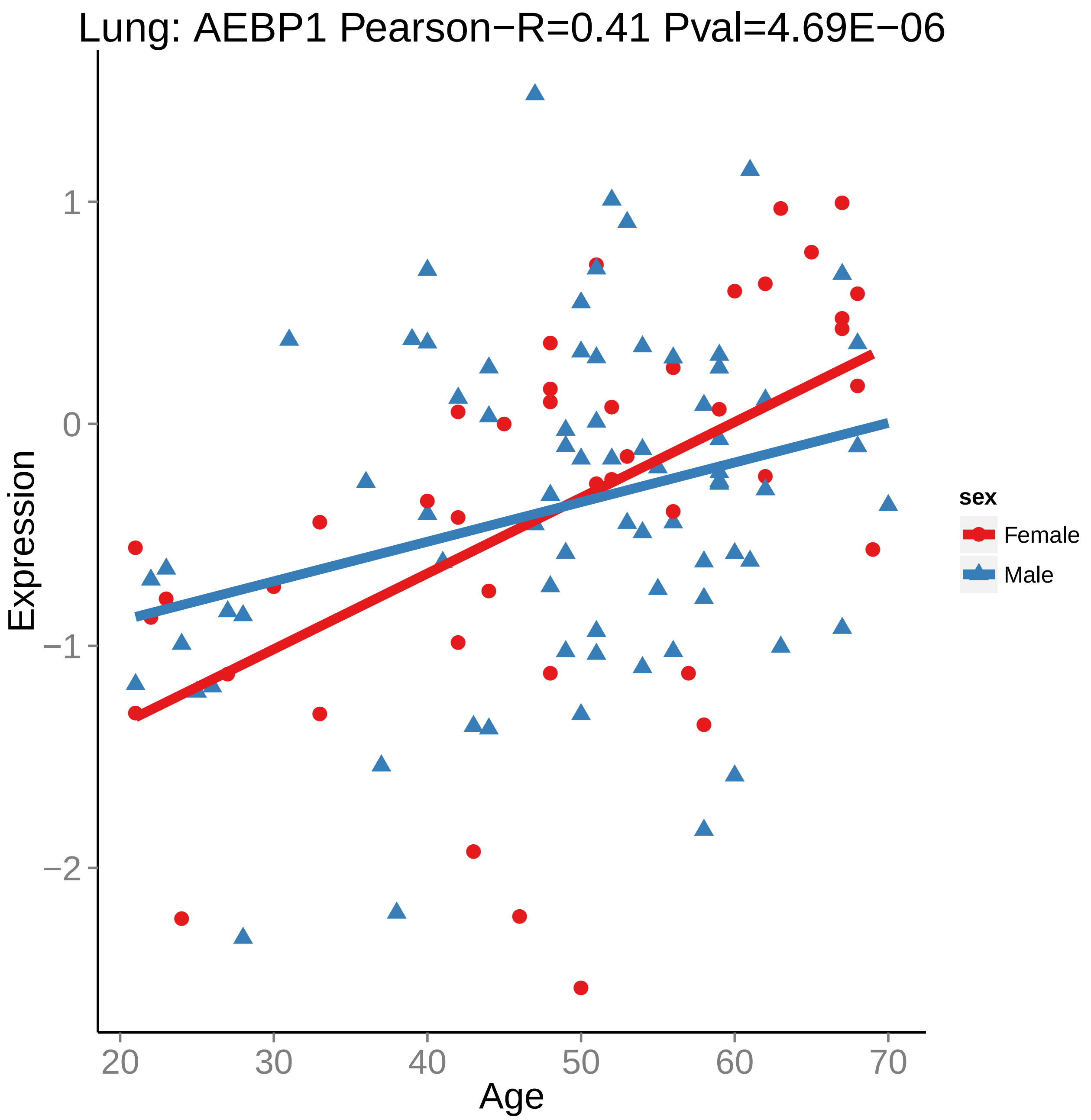
Lung: AEBP1 Pearson−R=0.41 Pval=4.69E−06
Expression
1
0
−1
−2
20
30
40
50
60
70
Age
sex
Female
Male

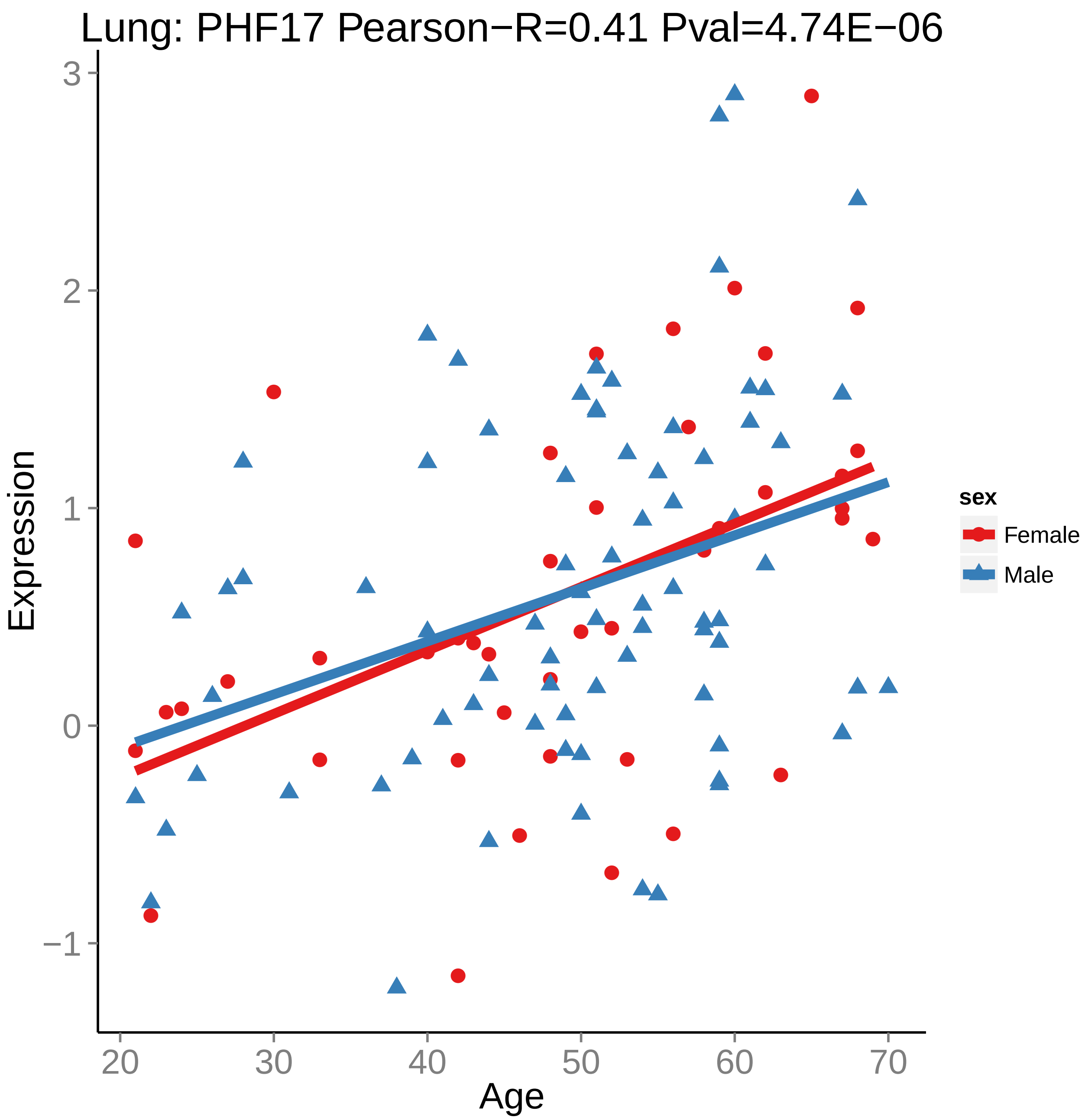
Lung: PHF17 Pearson−R=0.41 Pval=4.74E−06
Expression
Age
3
2
1
0
−1
20
30
40
50
60
70
sex
Female
Male

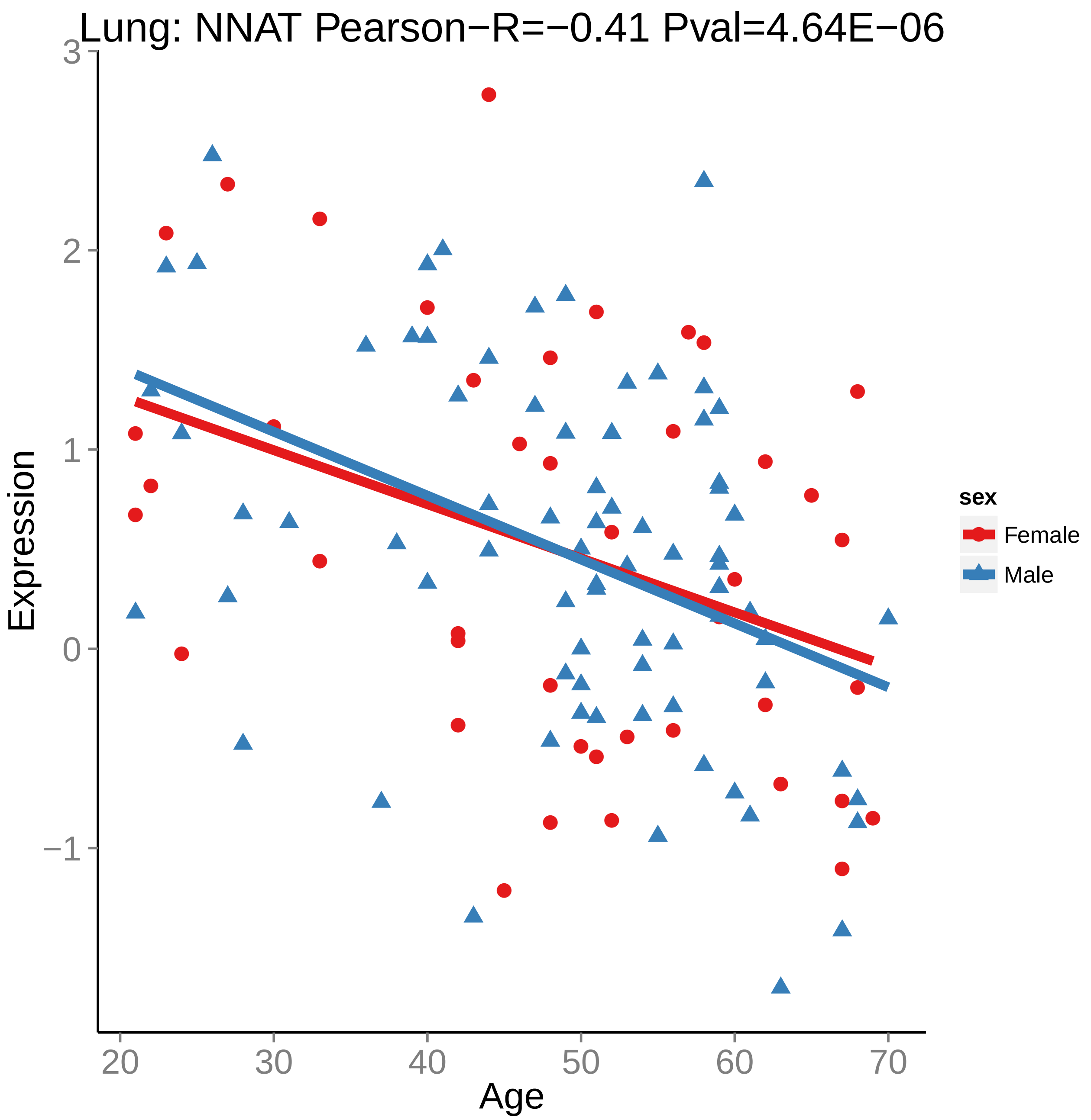

Lung: NNAT Pearson−R=−0.41 Pval=4.64E−06
Expression
Age
3
2
1
0
−1
20
30
40
50
60
70
sex
Female
Male

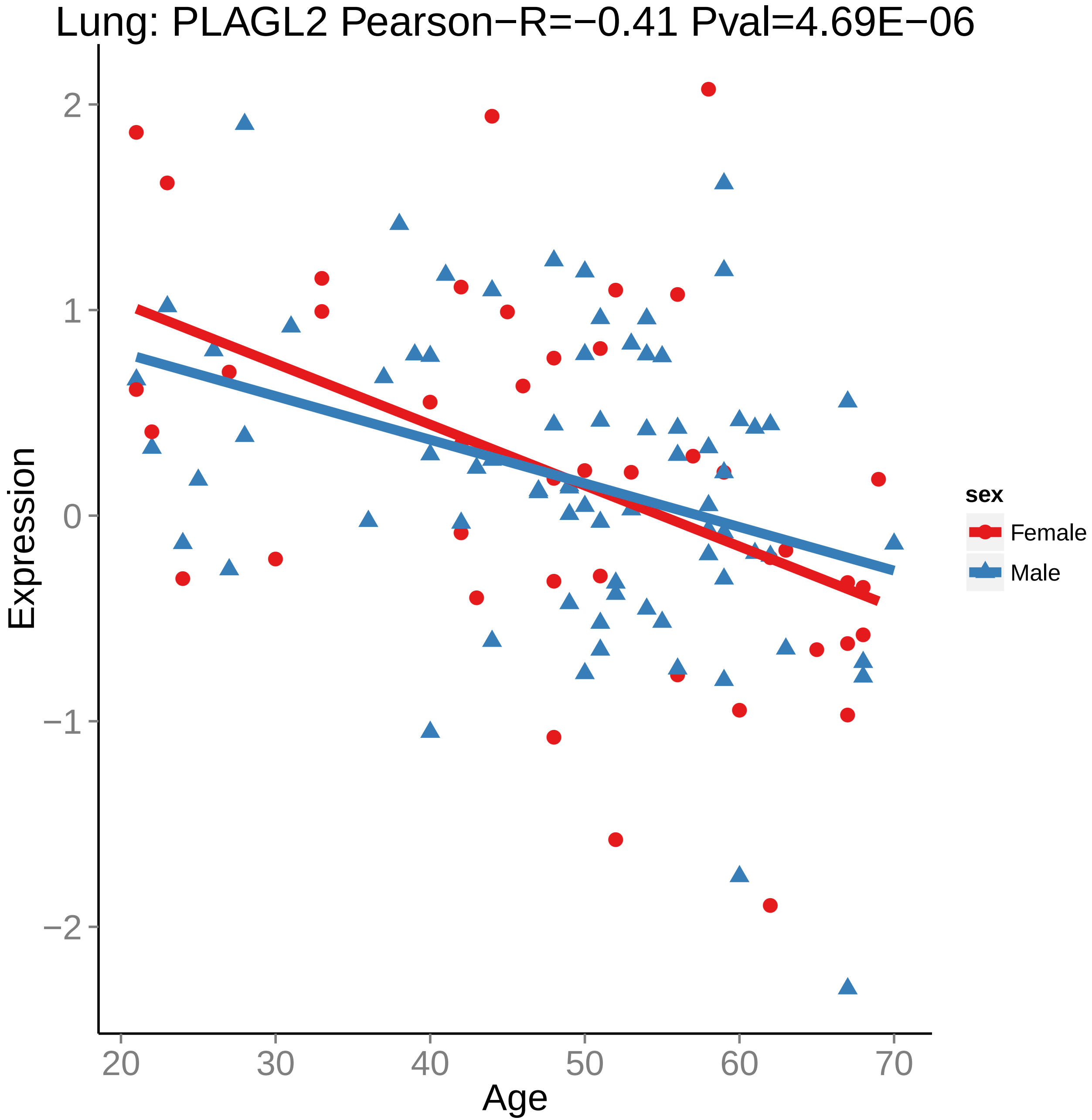

Lung: PLAGL2 Pearson−R=−0.41 Pval=4.69E−06
Expression
Age
2
1
0
−1
−2
20
30
40
50
60
70
sex
Female
Male

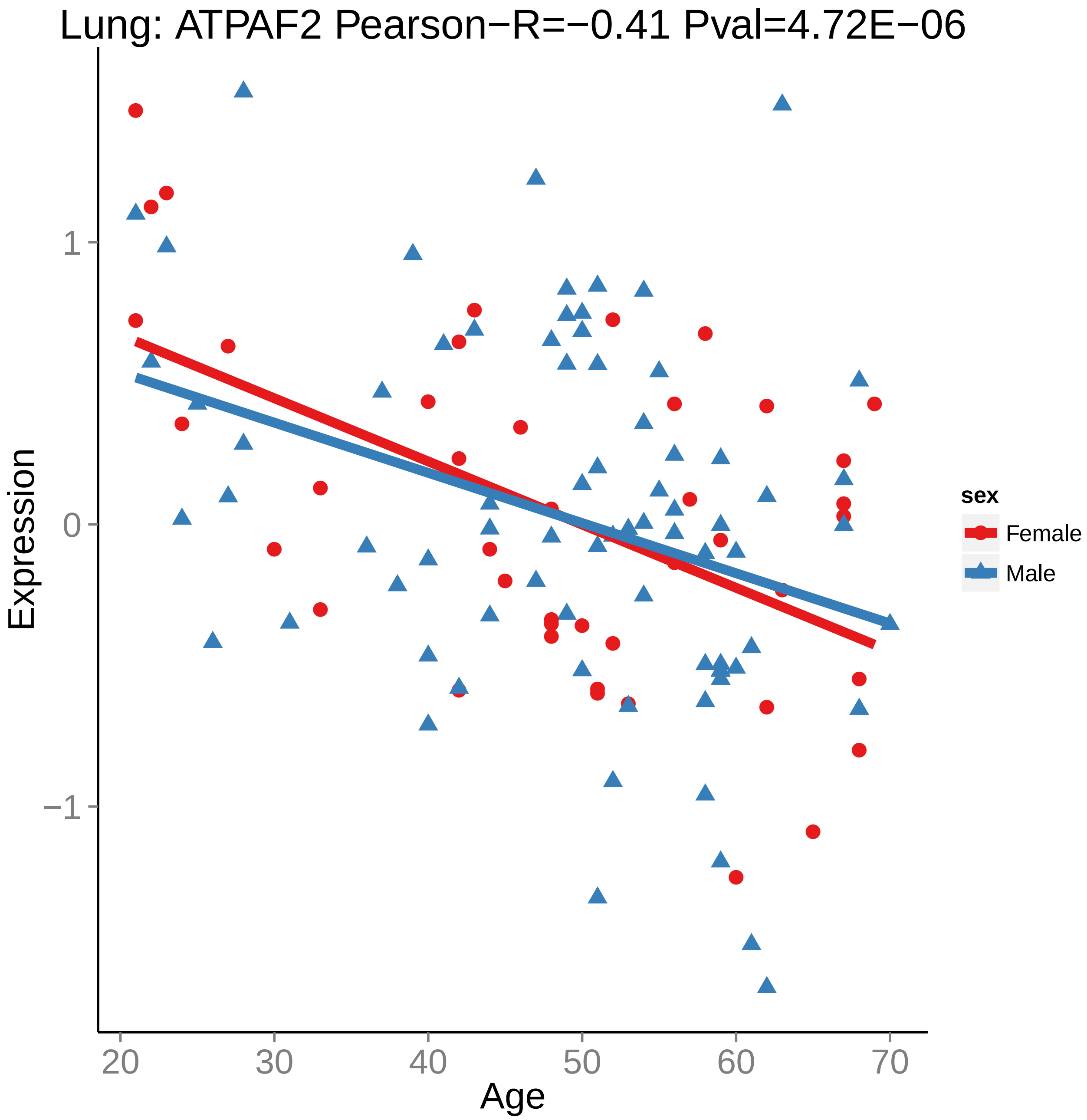

Lung: ATPAF2 Pearson−R=−0.41 Pval=4.72E−06
Expression
1
0
−1
20
30
40
50
60
70
Age
sex
Female
Male

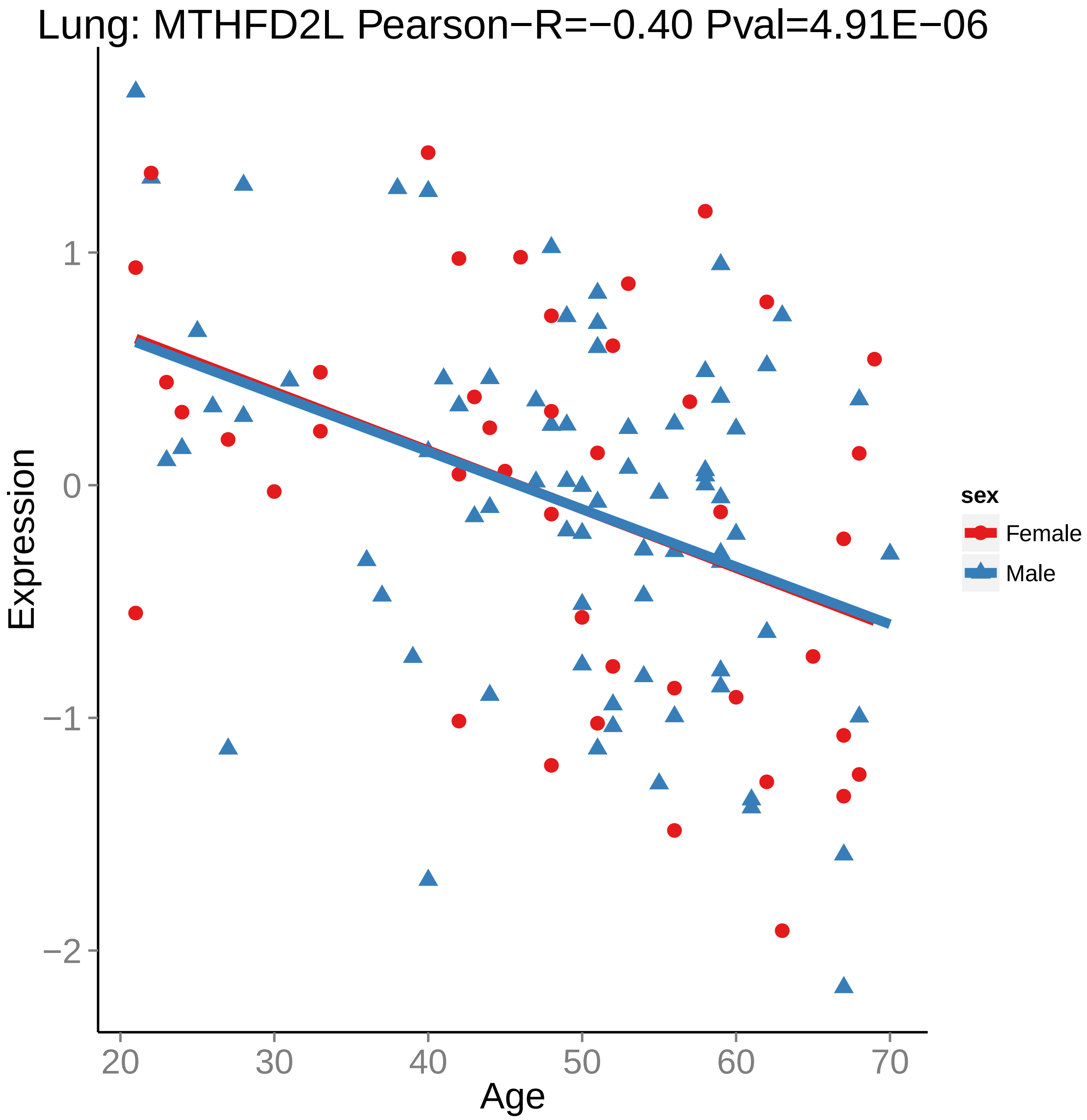

Lung: MTHFD2L Pearson−R=−0.40 Pval=4.91E−06
Expression
1
0
−1
−2
20
30
40
50
60
70
Age
sex
Female
Male

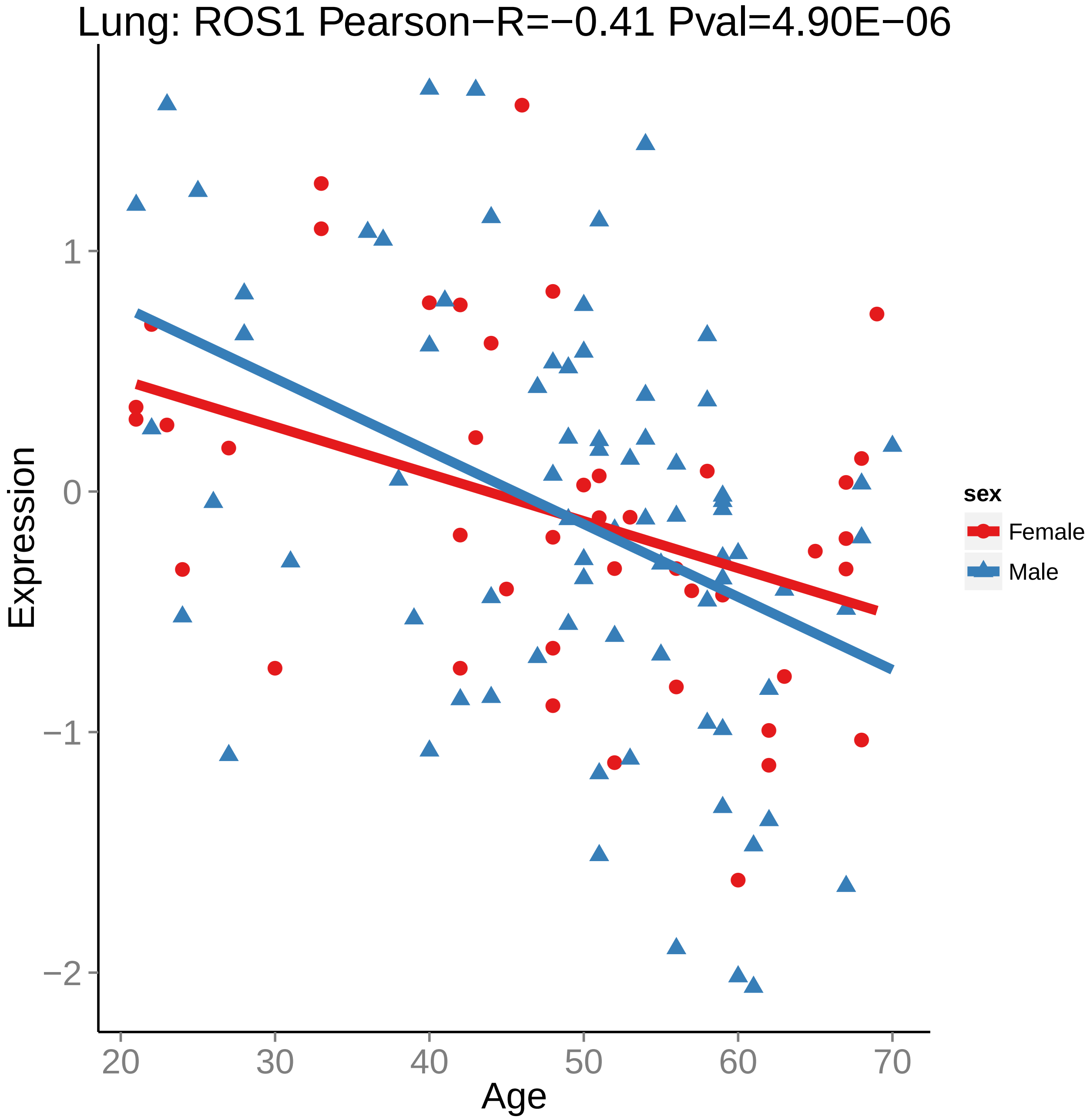

Lung: ROS1 Pearson−R=−0.41 Pval=4.90E−06
Expression
1
0
−1
−2
20
30
40
50
60
70
Age
sex
Female
Male

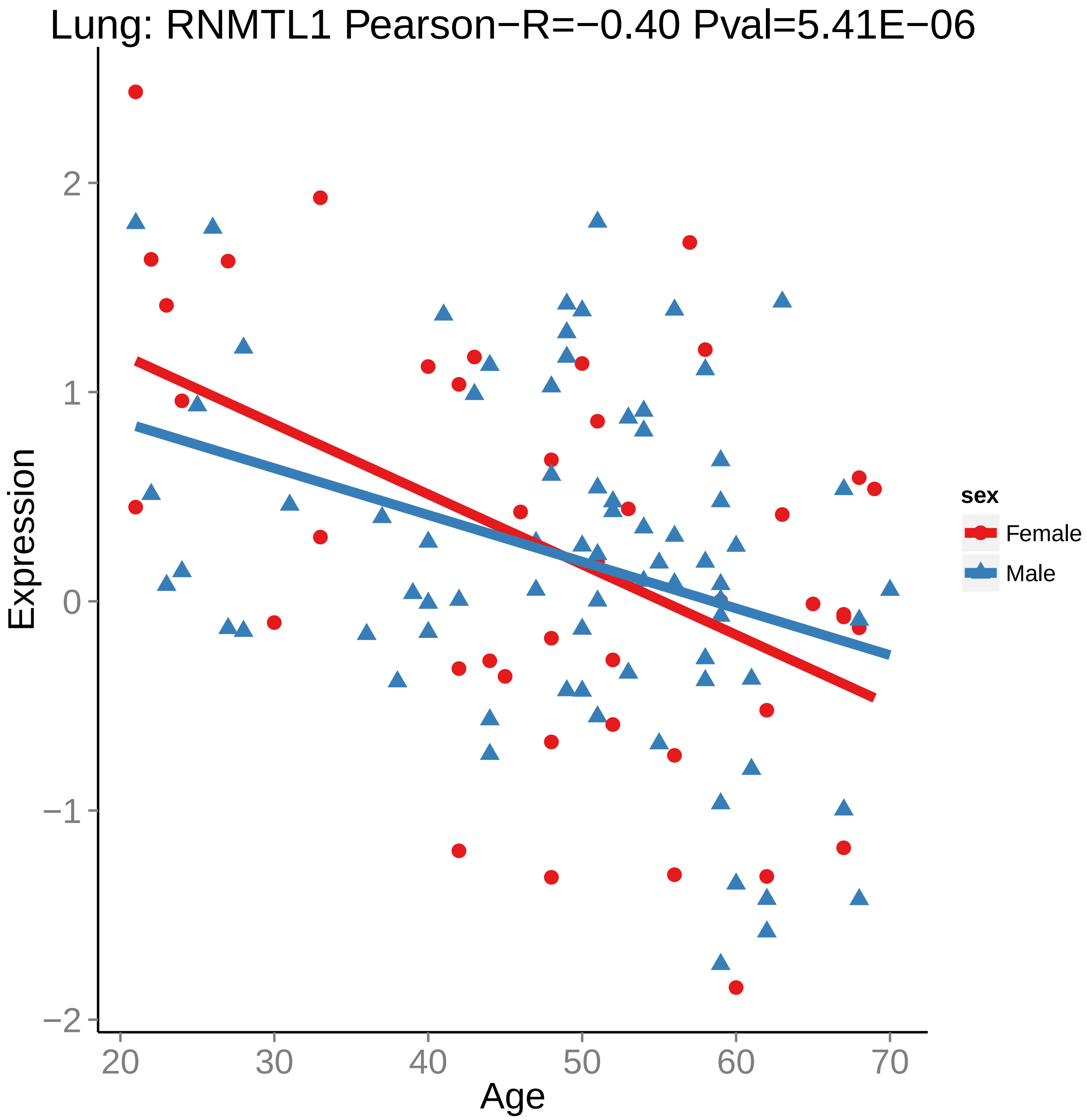

Lung: RNMTL1 Pearson−R=−0.40 Pval=5.41E−06
Expression
Age
sex
Female
Male
2
1
0
−1
−2
20
30
40
50
60
70

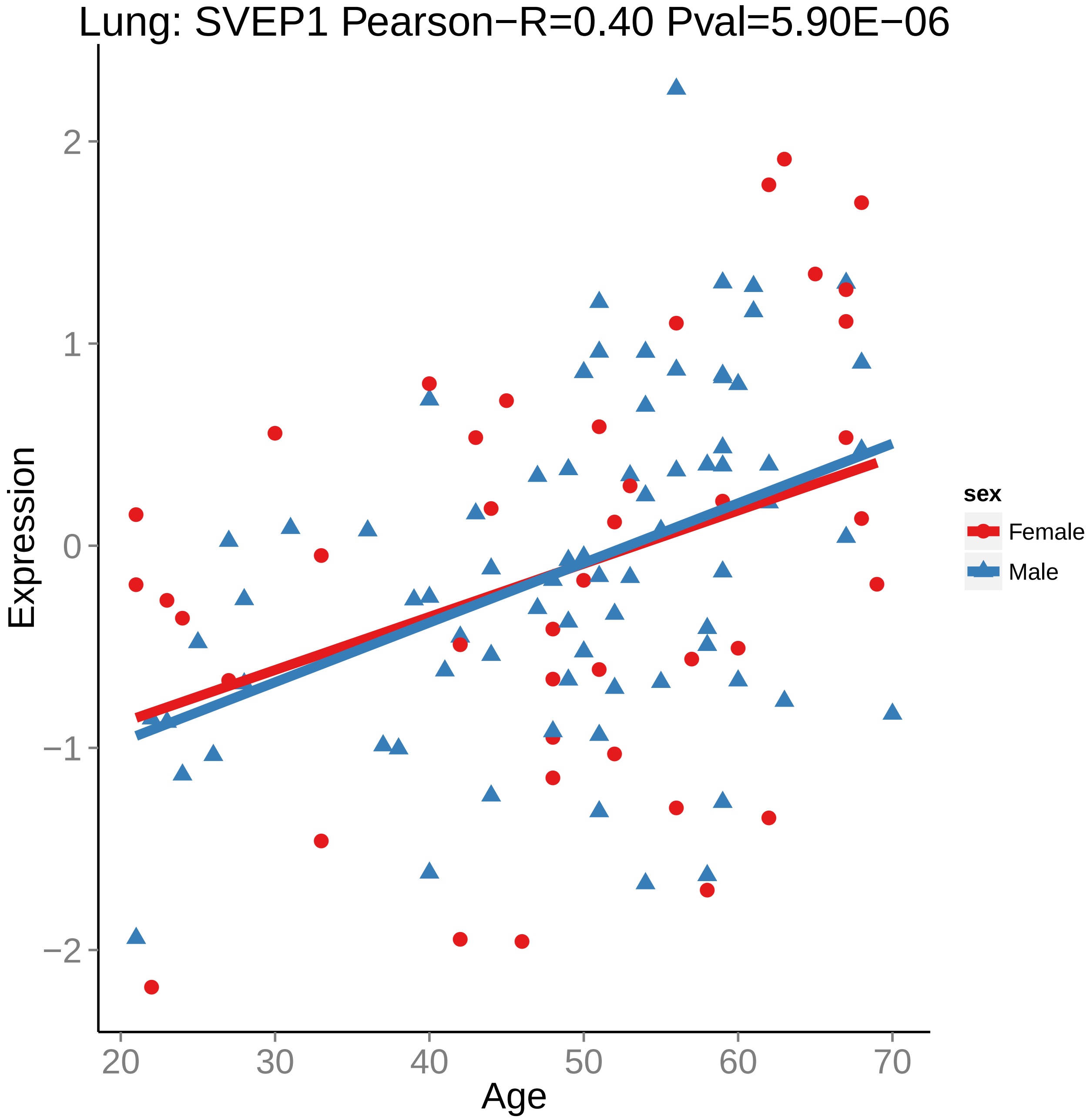

Lung: SVEP1 Pearson−R=0.40 Pval=5.90E−06
Expression
2
1
0
−1
−2
20
30
40
50
60
70
Age
sex
Female
Male

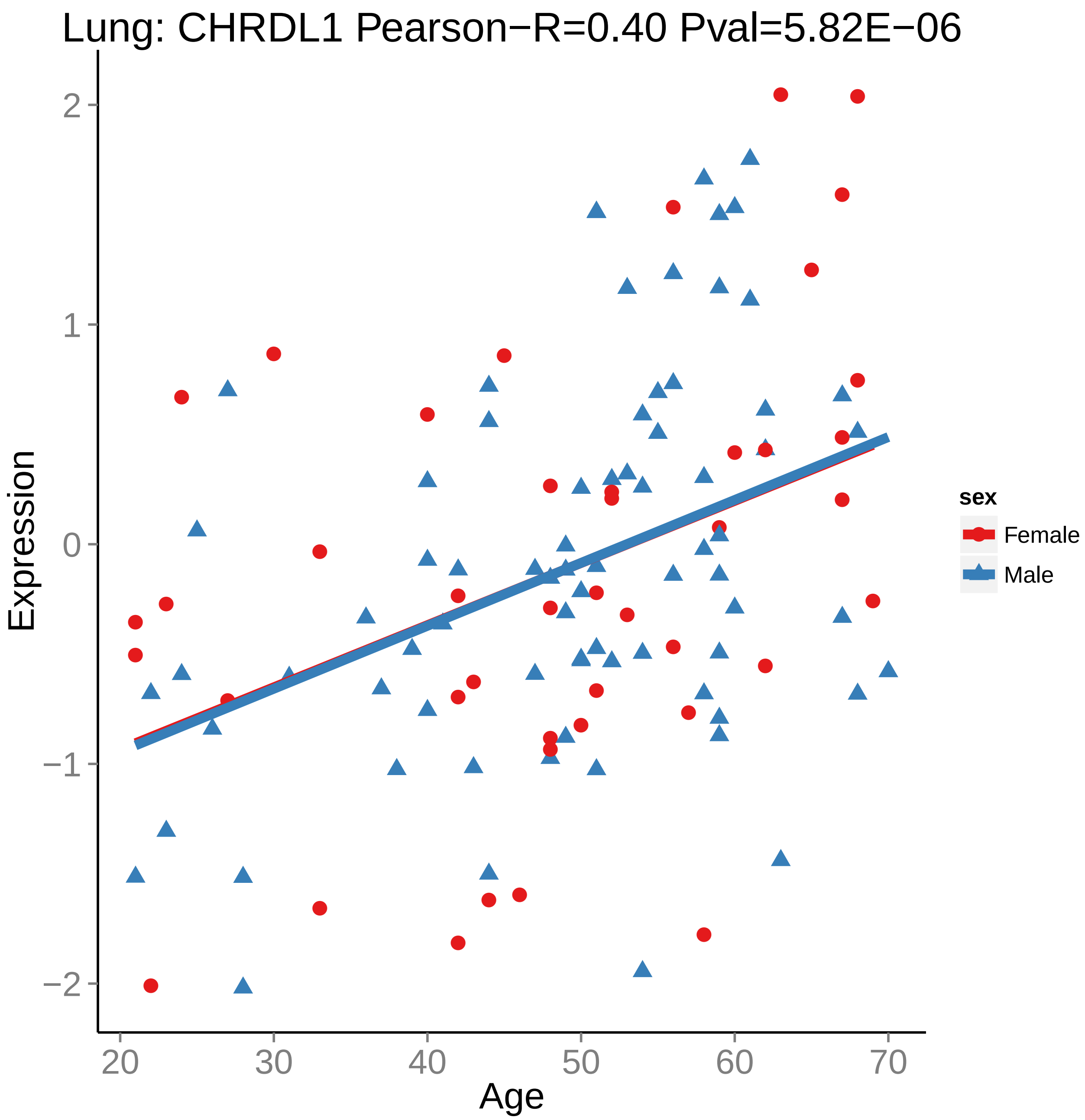

Lung: CHRDL1 Pearson−R=0.40 Pval=5.82E−06
Expression
2
1
0
−1
−2
20
30
40
50
60
70
Age
sex
Female
Male

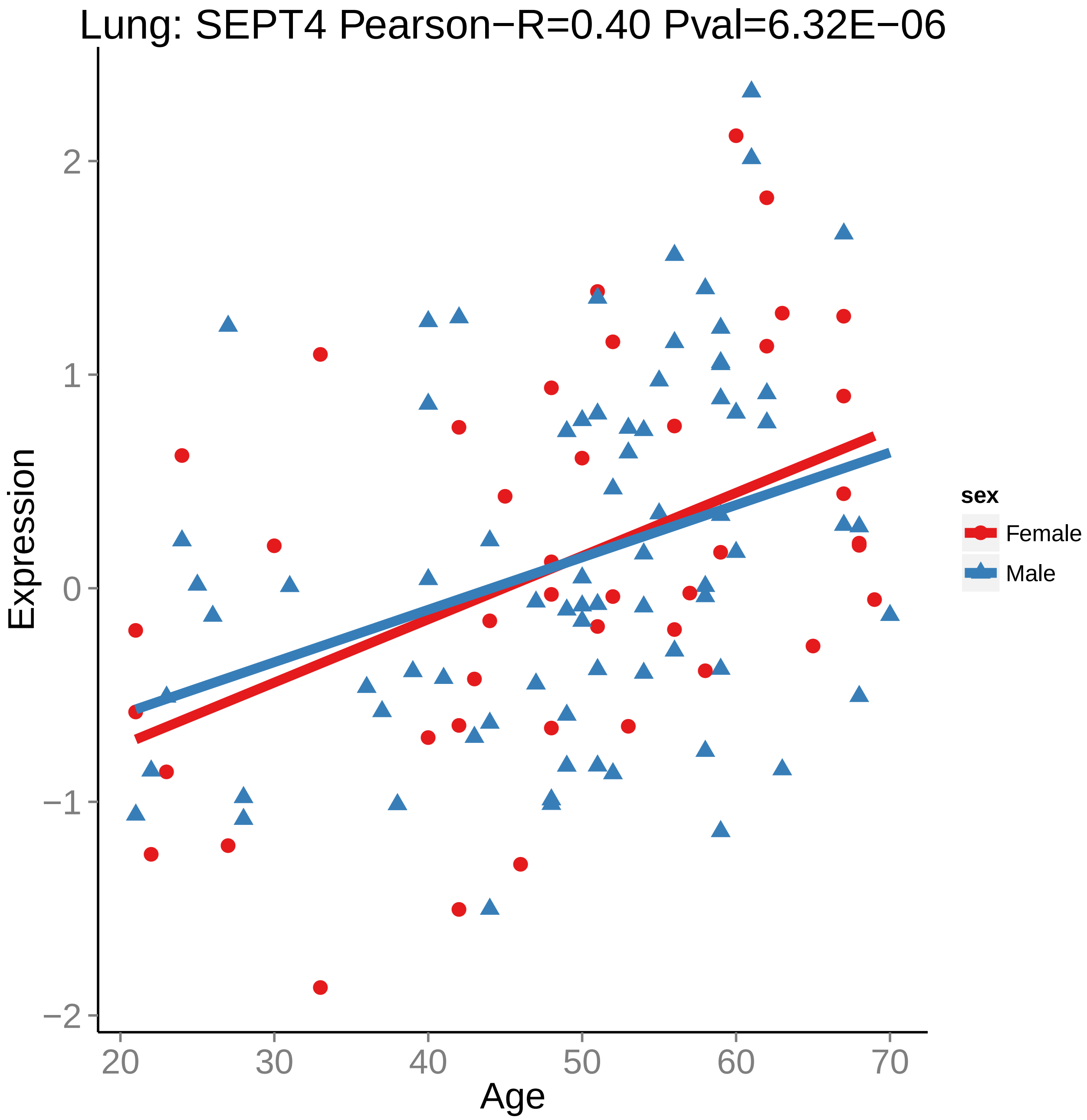

Lung: SEPT4 Pearson−R=0.40 Pval=6.32E−06
Expression
Age
2
1
0
−1
−2
20
30
40
50
60
70
sex
Female
Male

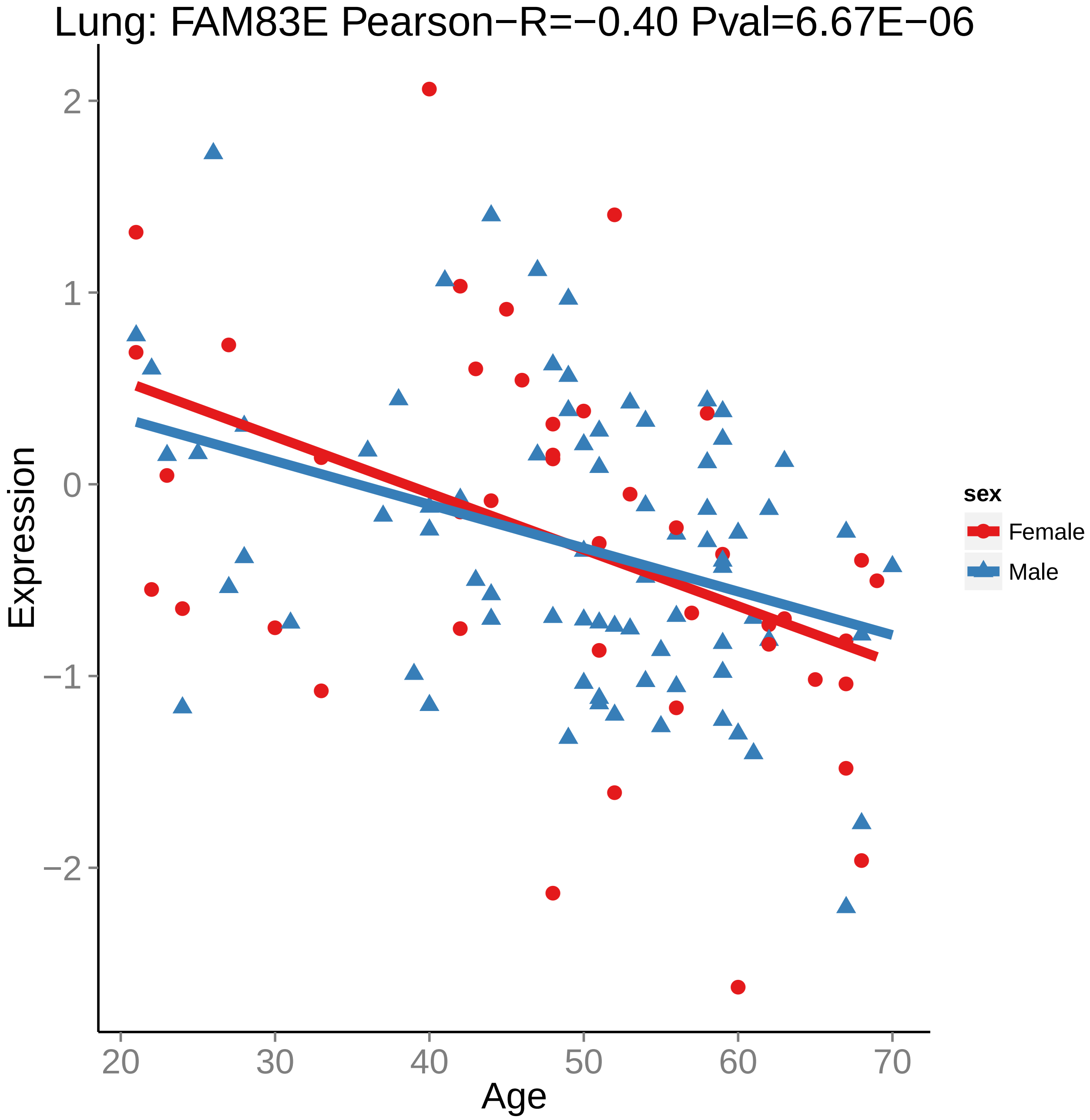
Lung: FAM83E Pearson−R=−0.40 Pval=6.67E−06
Expression
2
1
0
−1
−2
20
30
40
50
60
70
Age
sex
Female
Male

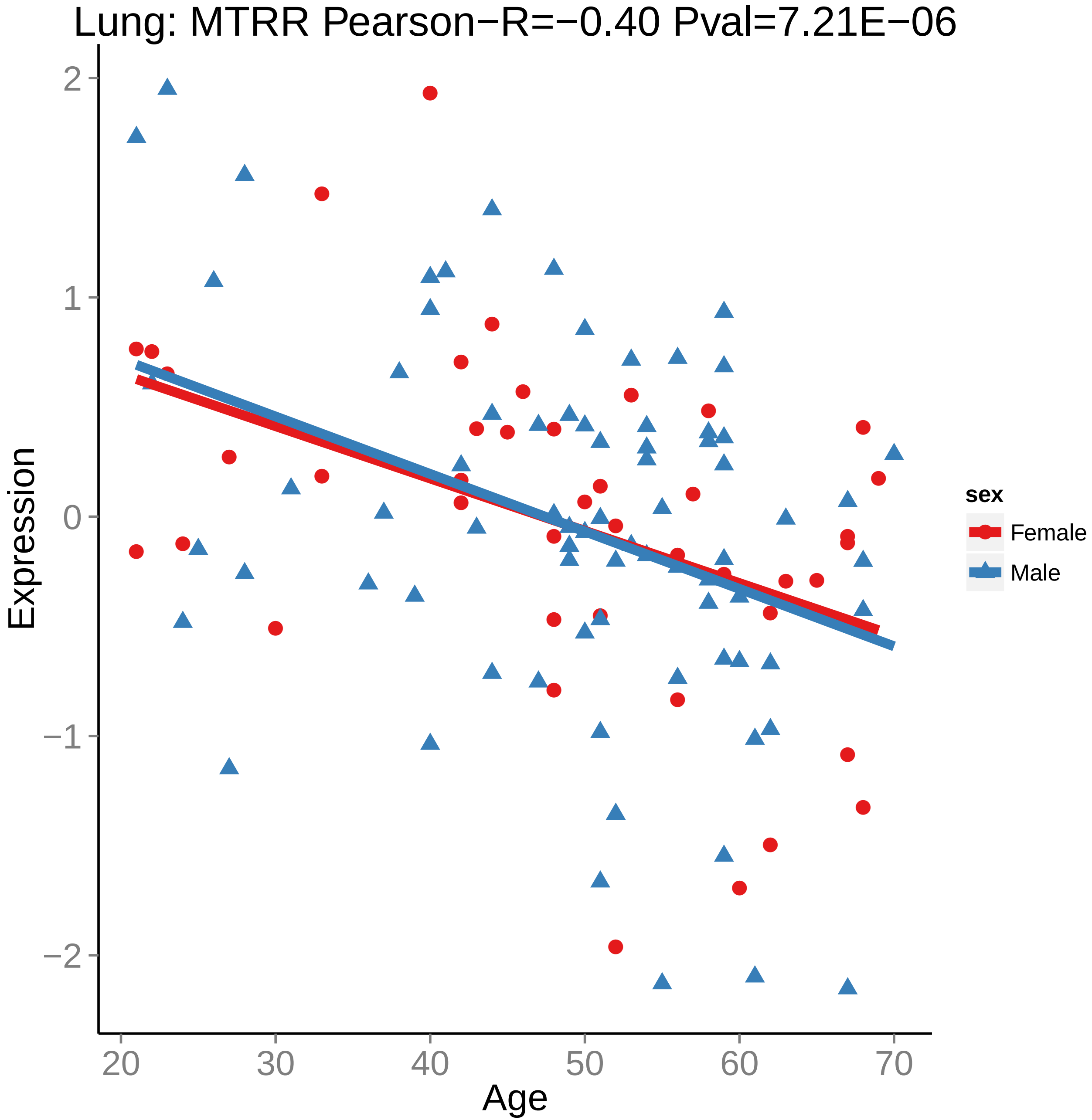

Lung: MTRR Pearson−R=−0.40 Pval=7.21E−06
Expression
2
1
0
−1
−2
Age
20
30
40
50
60
70
sex
Female
Male

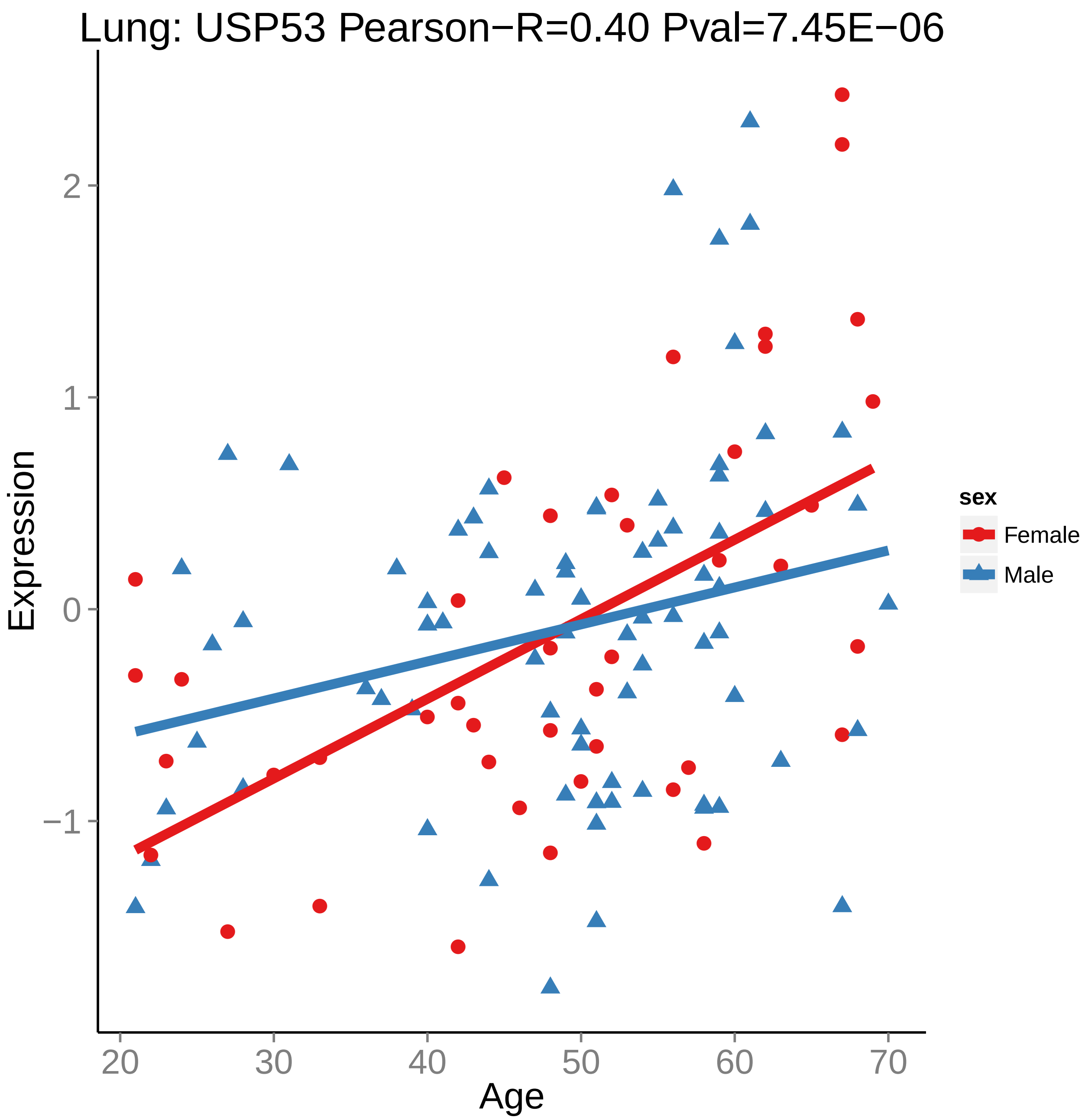
Lung: USP53 Pearson−R=0.40 Pval=7.45E−06
Expression
Age
2
1
0
−1
20
30
40
50
60
70
sex
Female
Male

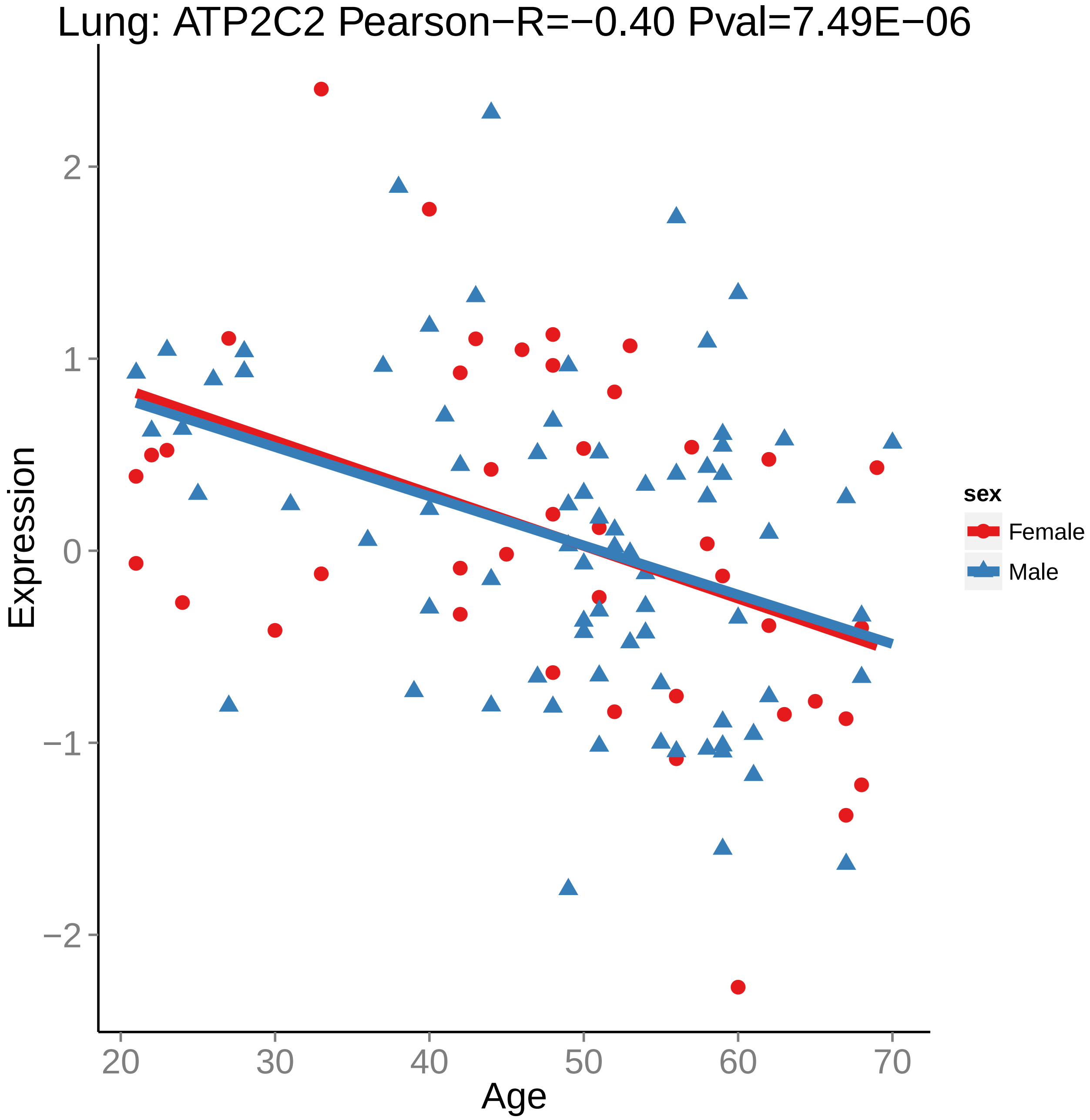

Lung: ATP2C2 Pearson−R=−0.40 Pval=7.49E−06
Expression
2
1
0
−1
−2
20
30
40
50
60
70
Age
sex
Female
Male

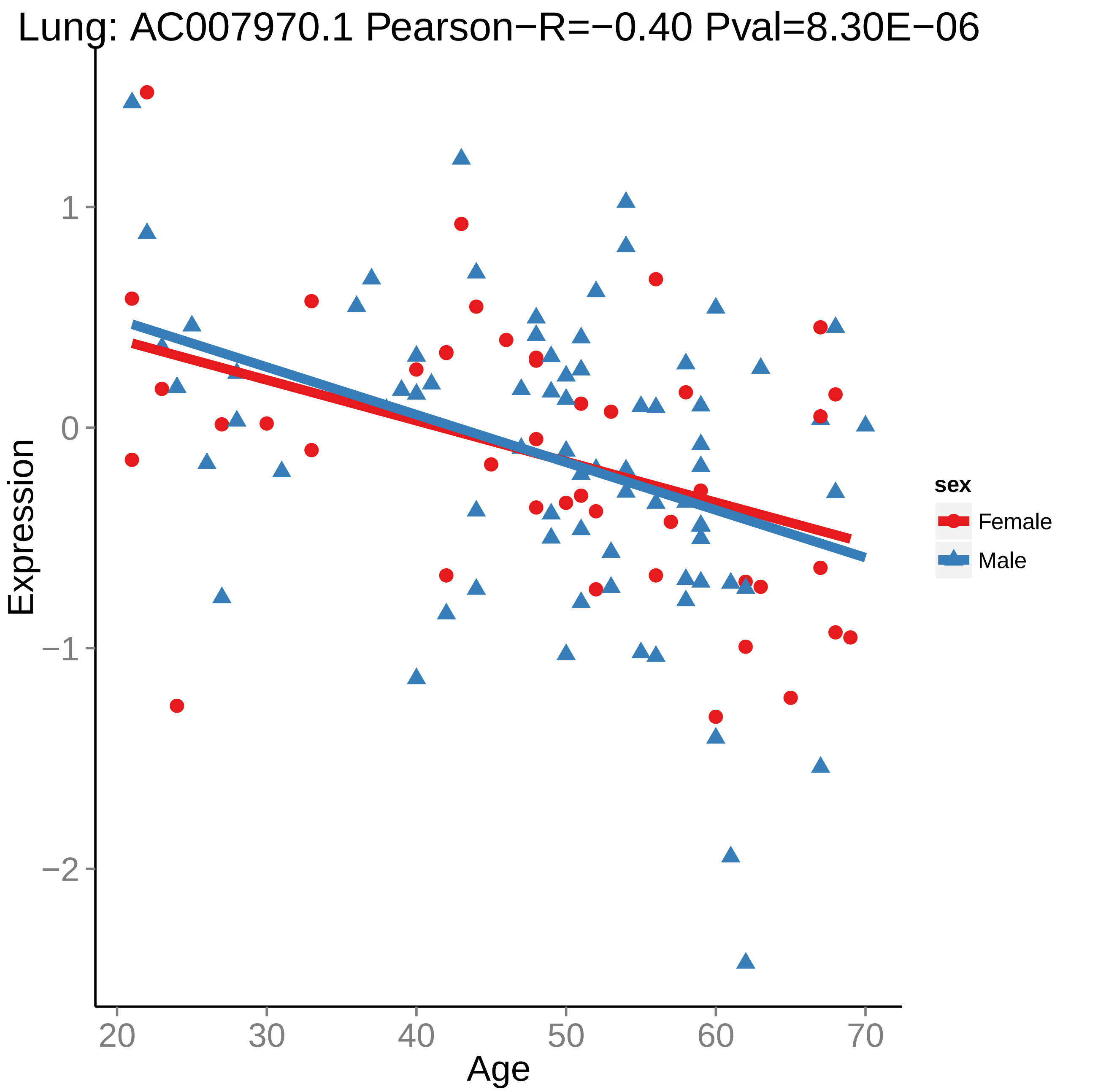

Lung: AC007970.1 Pearson−R=−0.40 Pval=8.30E−06
Expression
1
0
−1
−2
20
30
40
50
60
70
Age
sex
Female
Male

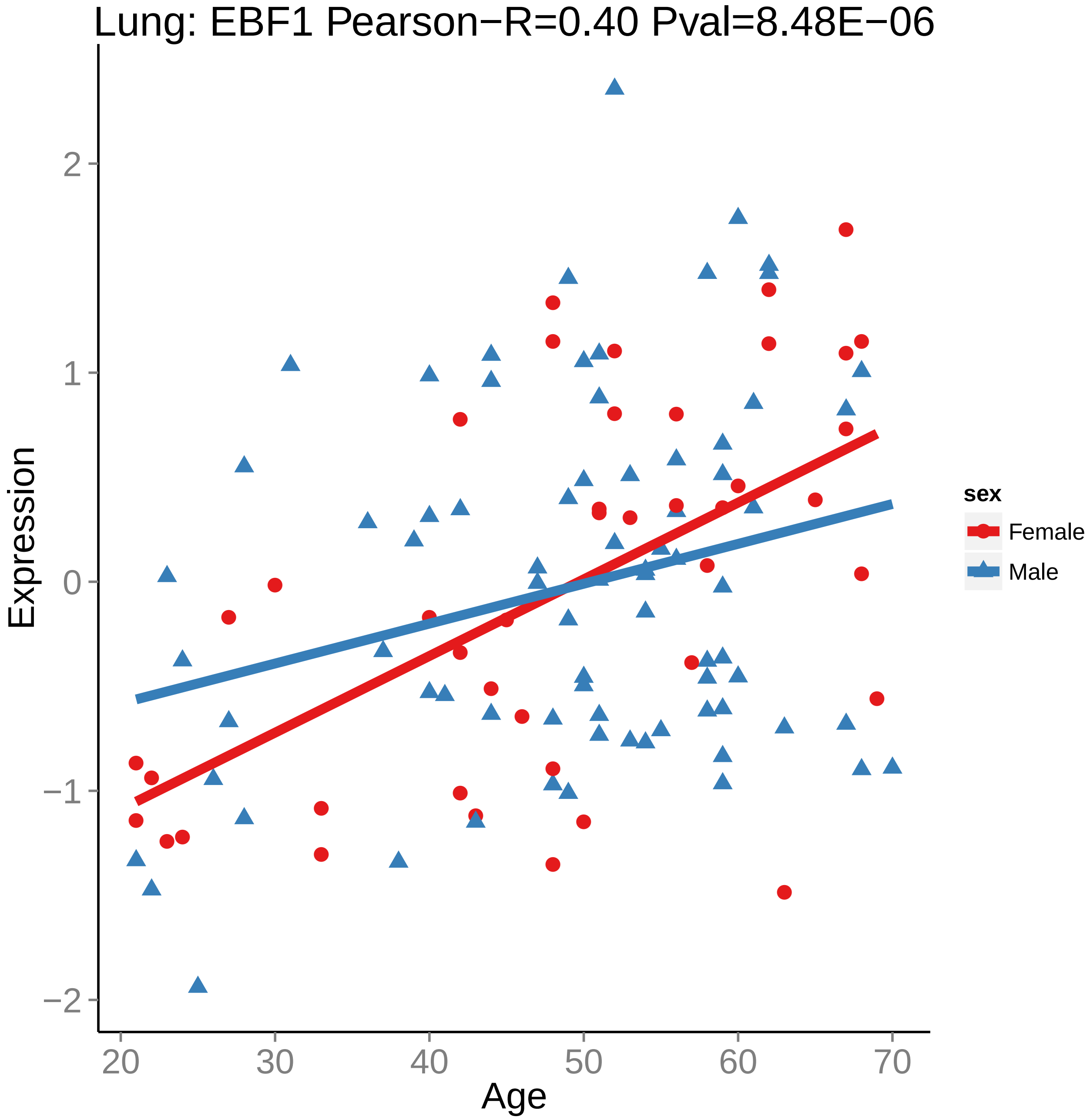

Lung: EBF1 Pearson−R=0.40 Pval=8.48E−06
Expression
2
1
0
−1
−2
20
30
40
50
60
70
Age
sex
Female
Male

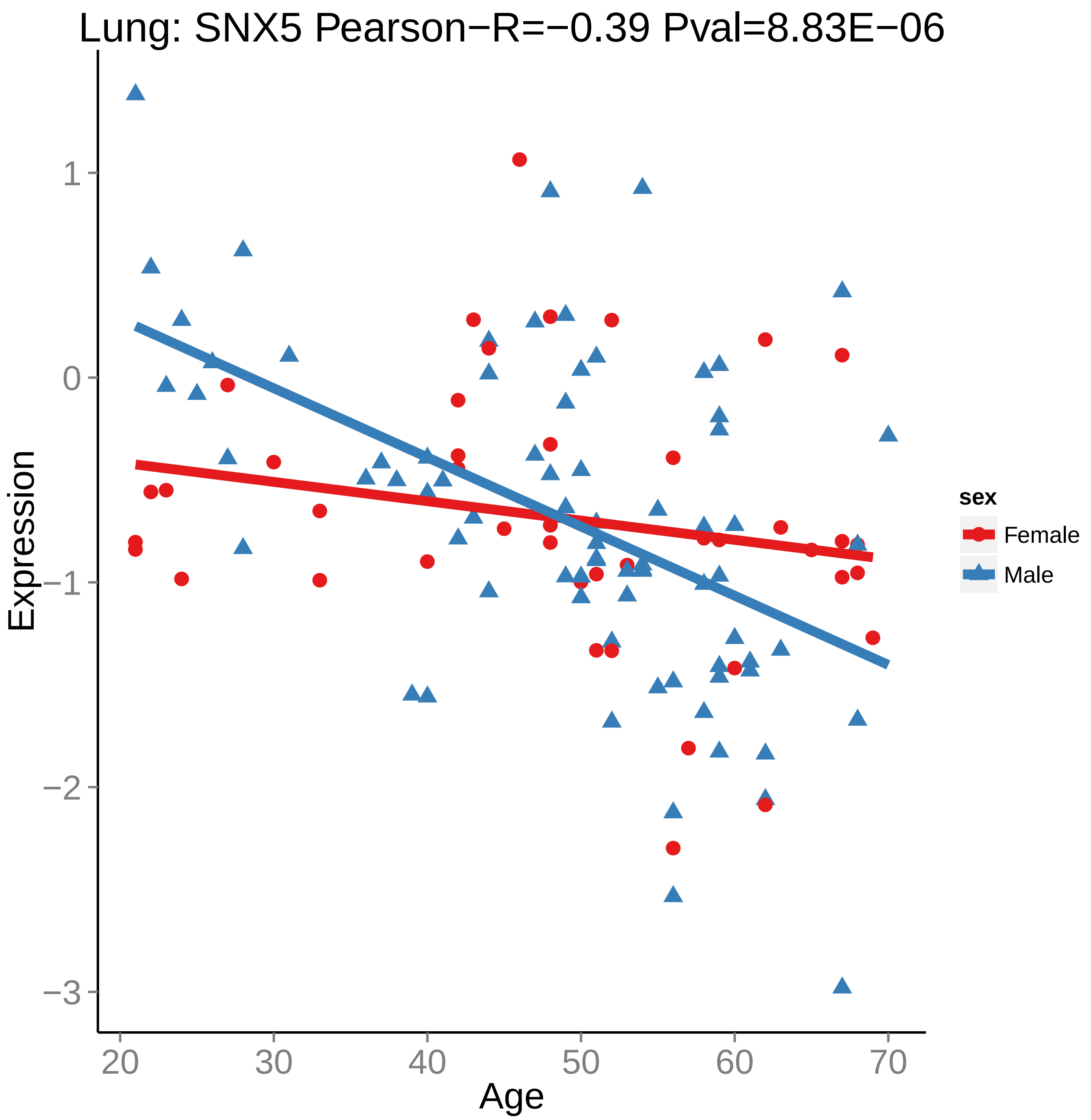

Lung: SNX5 Pearson−R=−0.39 Pval=8.83E−06
Expression
Age
1
0
−1
−2
−3
20
30
40
50
60
70
sex
Female
Male

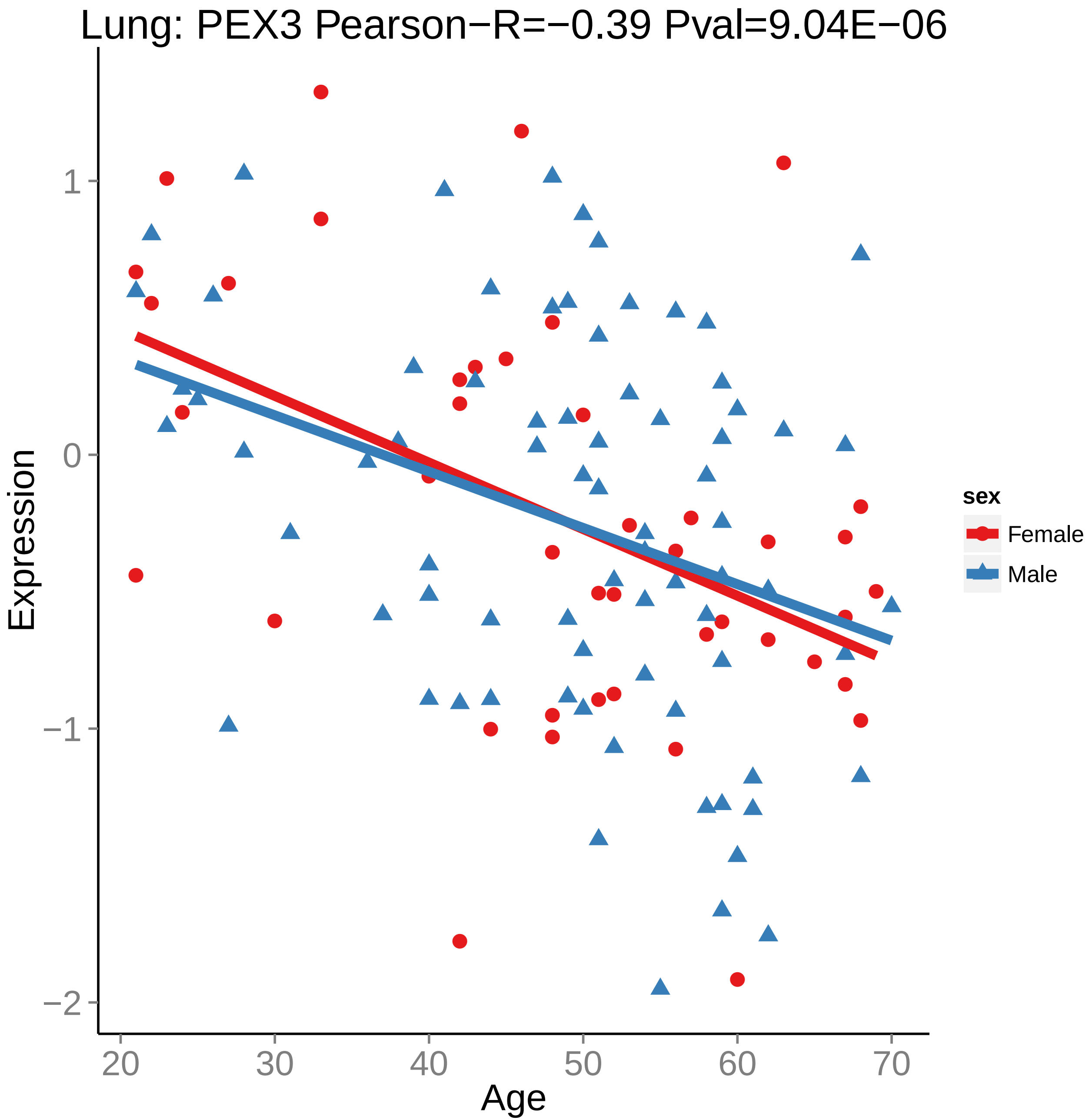

Lung: PEX3 Pearson−R=−0.39 Pval=9.04E−06
Expression
Age
1
0
−1
−2
20
30
40
50
60
70
sex
Female
Male

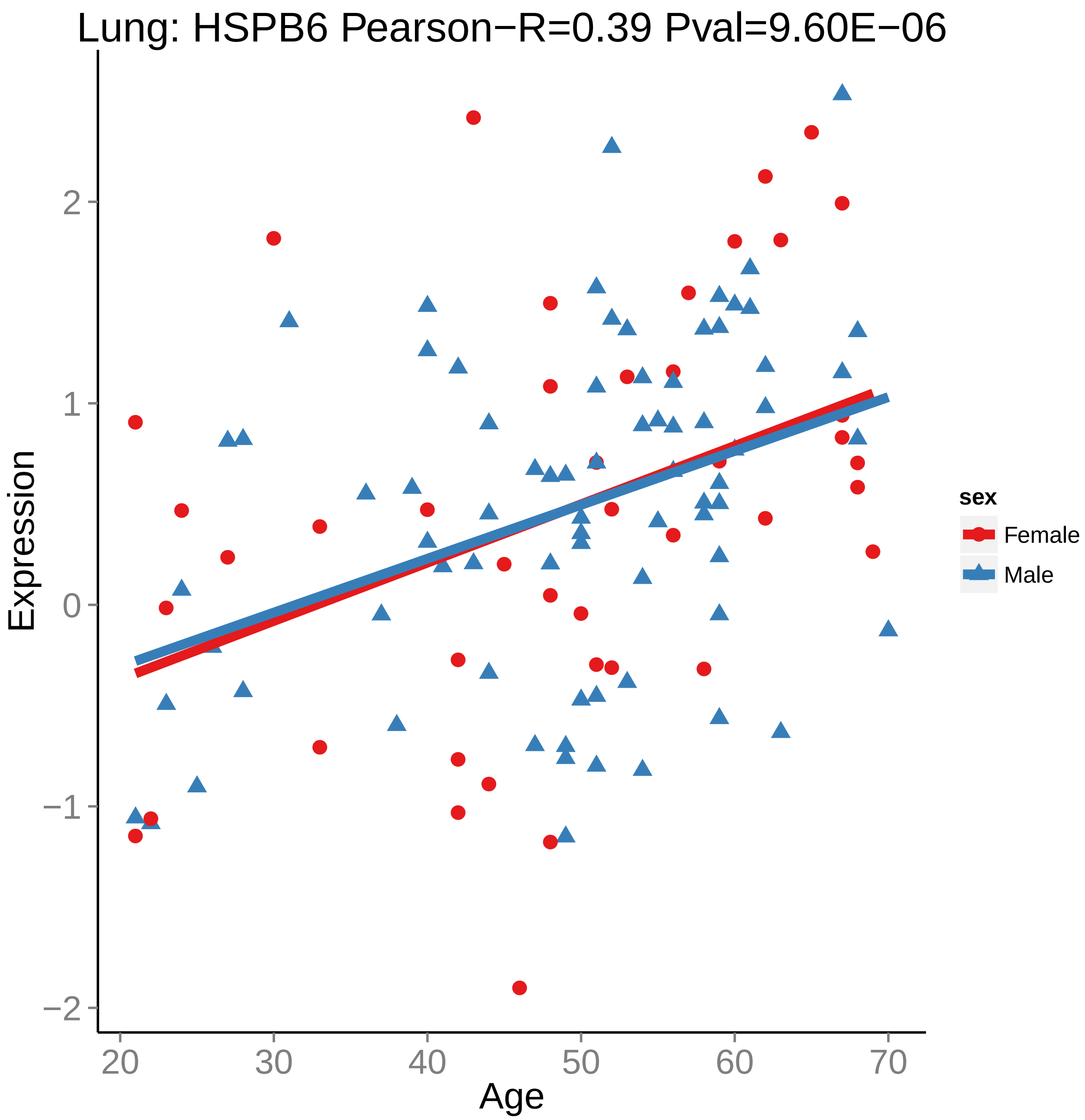

Lung: HSPB6 Pearson−R=0.39 Pval=9.60E−06
Expression
2
1
0
−1
−2
20
30
40
50
60
70
Age
sex
Female
Male

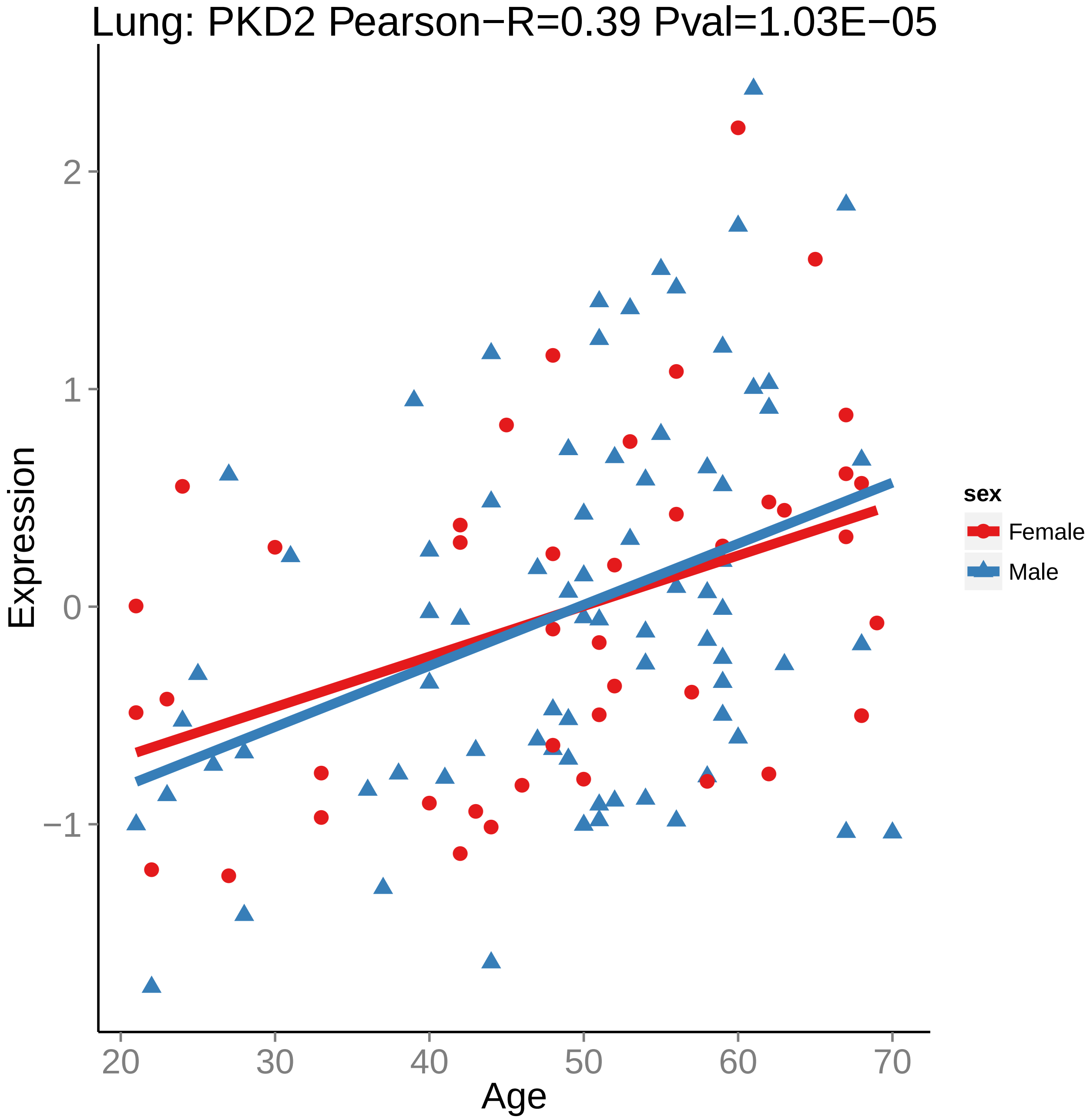

Lung: PKD2 Pearson−R=0.39 Pval=1.03E−05
Expression
2
1
0
−1
20
30
40
50
60
70
Age
sex
Female
Male

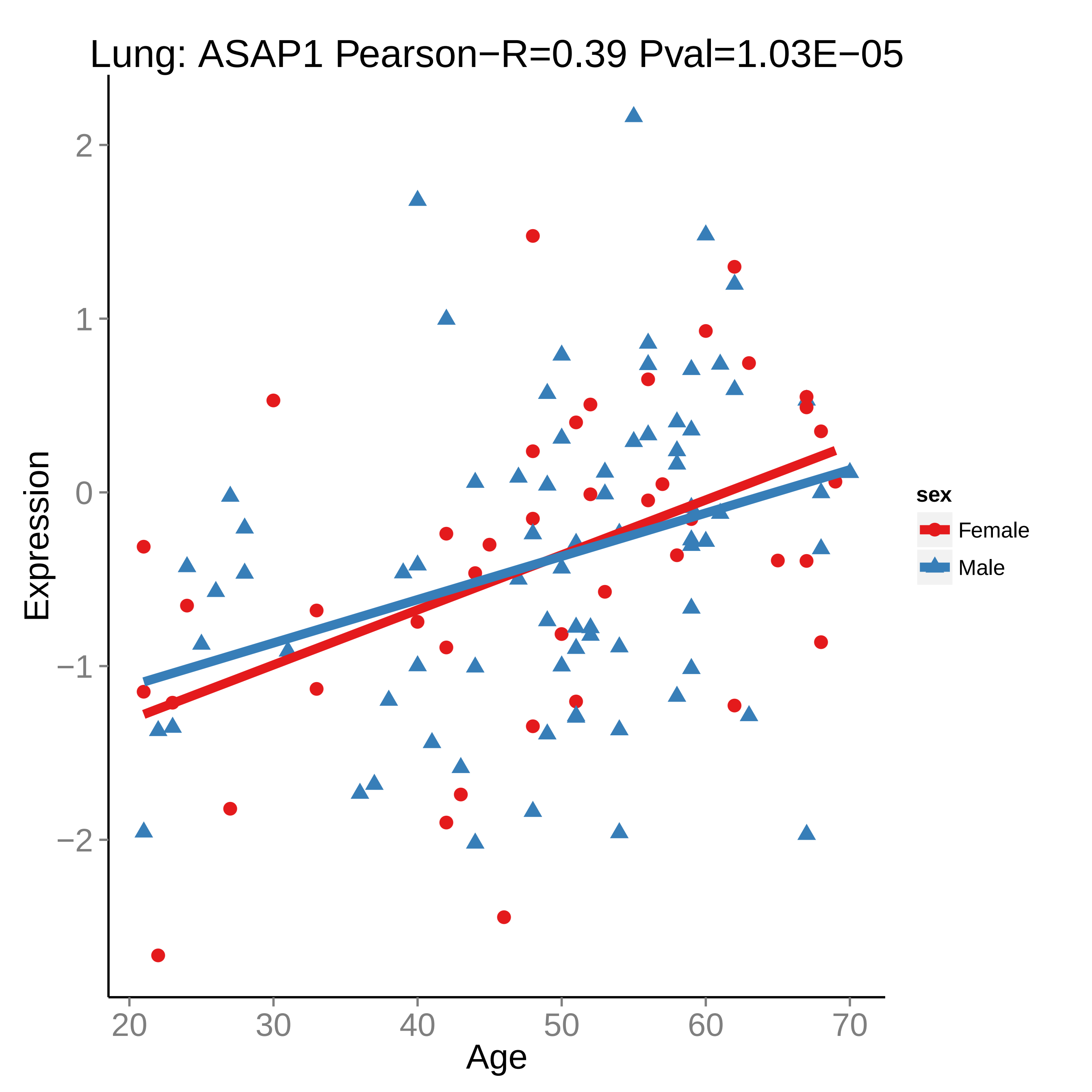

Lung: ASAP1 Pearson−R=0.39 Pval=1.03E−05
Expression
Age
2
1
0
−1
−2
20
30
40
50
60
70
sex
Female
Male

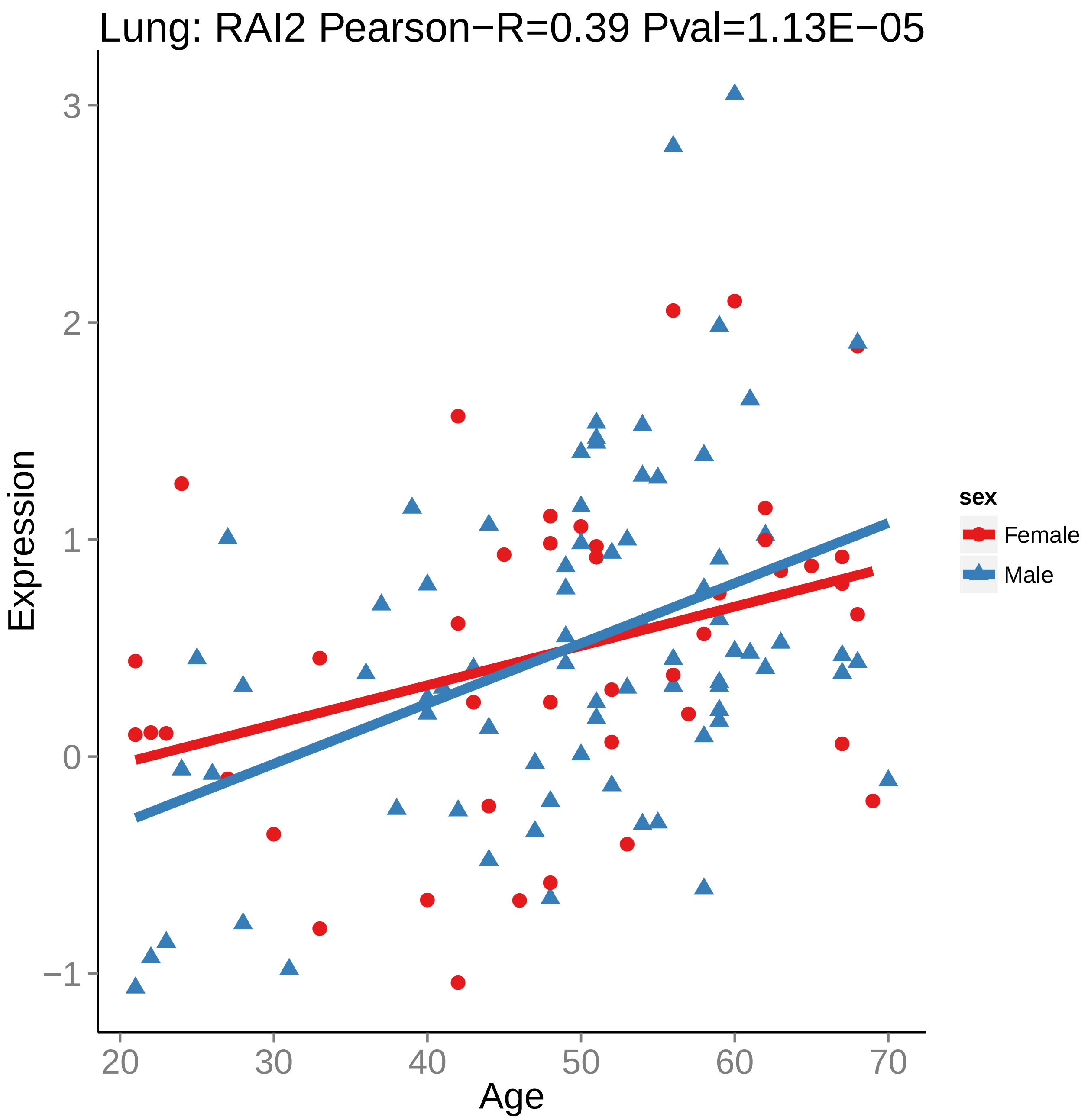

Lung: RAI2 Pearson−R=0.39 Pval=1.13E−05
Expression
3
2
1
0
−1
20
30
40
50
60
70
Age
sex
Female
Male

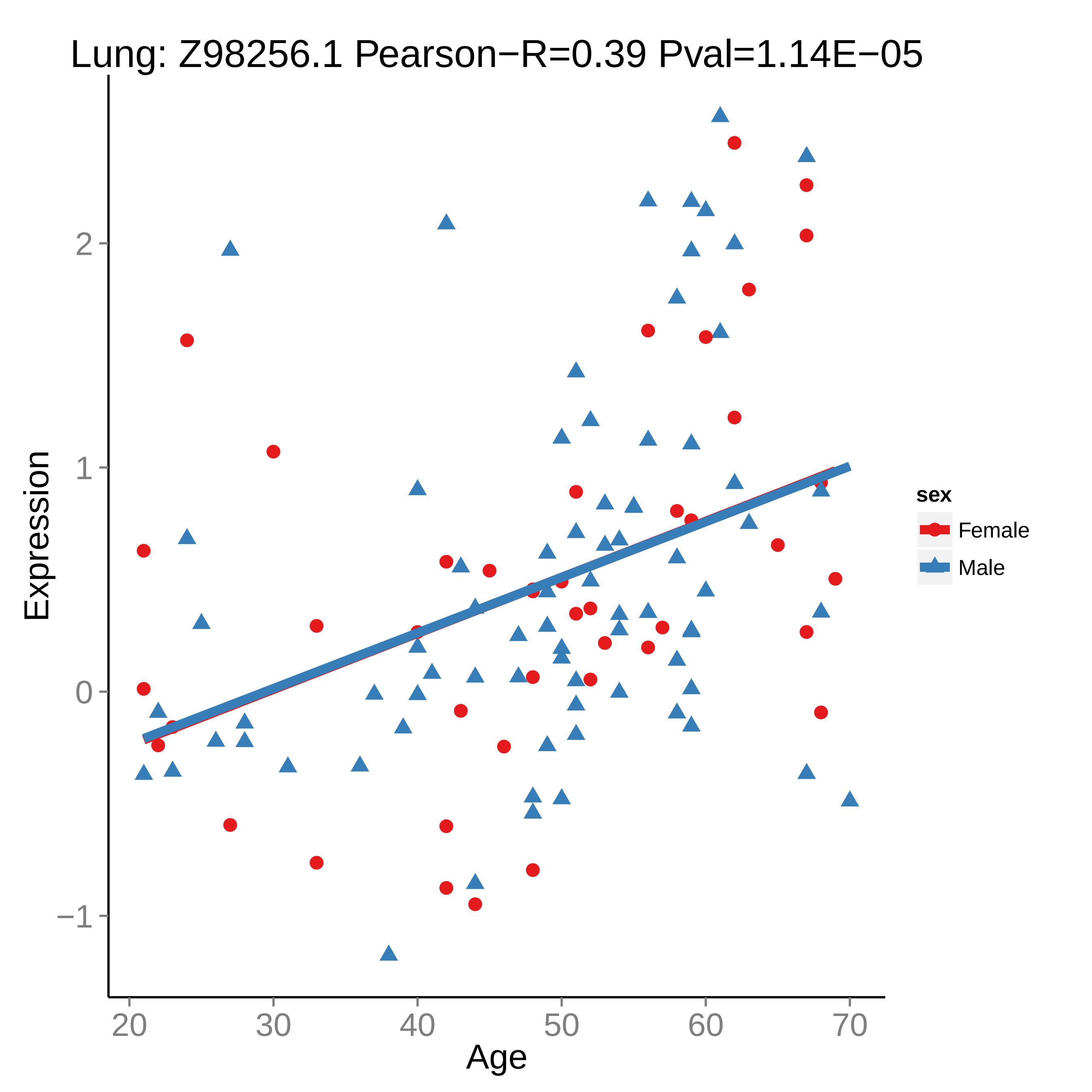
Lung: Z98256.1 Pearson−R=0.39 Pval=1.14E−05
Expression
Age
2
1
0
−1
20
30
40
50
60
70
sex
Female
Male

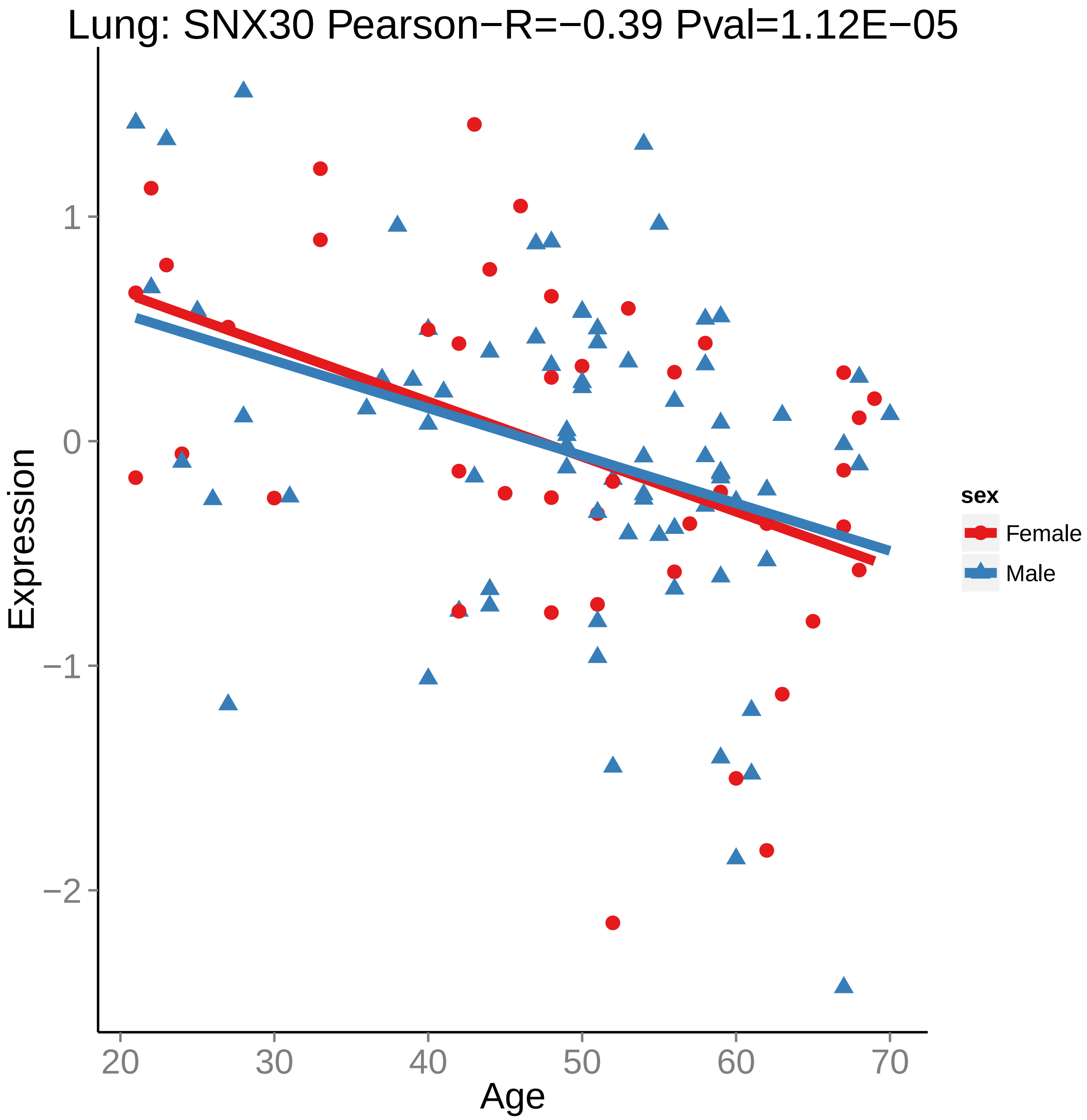

Lung: SNX30 Pearson−R=−0.39 Pval=1.12E−05
Expression
1
0
−1
−2
Age
20
30
40
50
60
70
sex
Female
Male

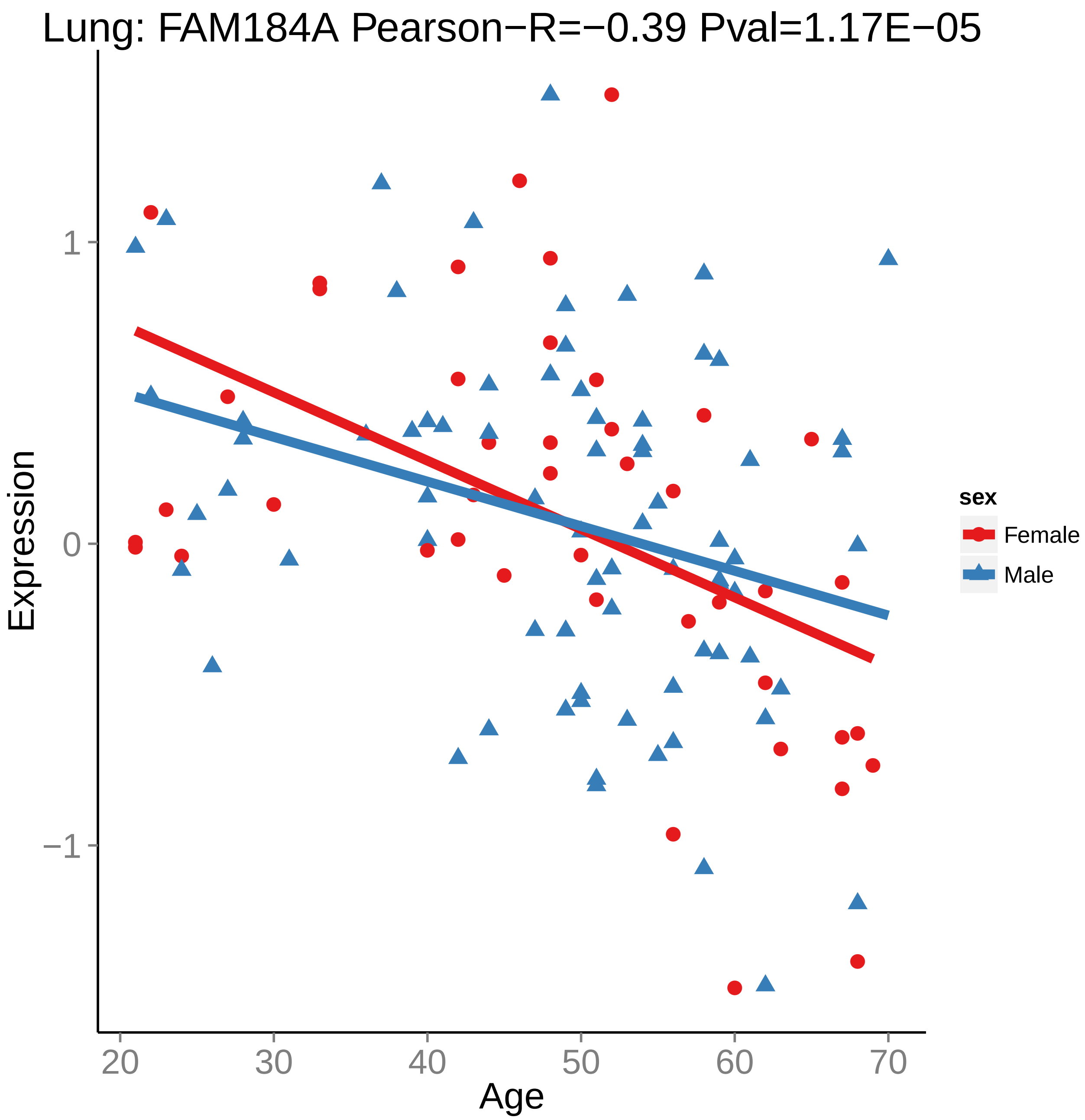
Lung: FAM184A Pearson−R=−0.39 Pval=1.17E−05
Expression
1
0
−1
20
30
40
50
60
70
Age
sex
Female
Male

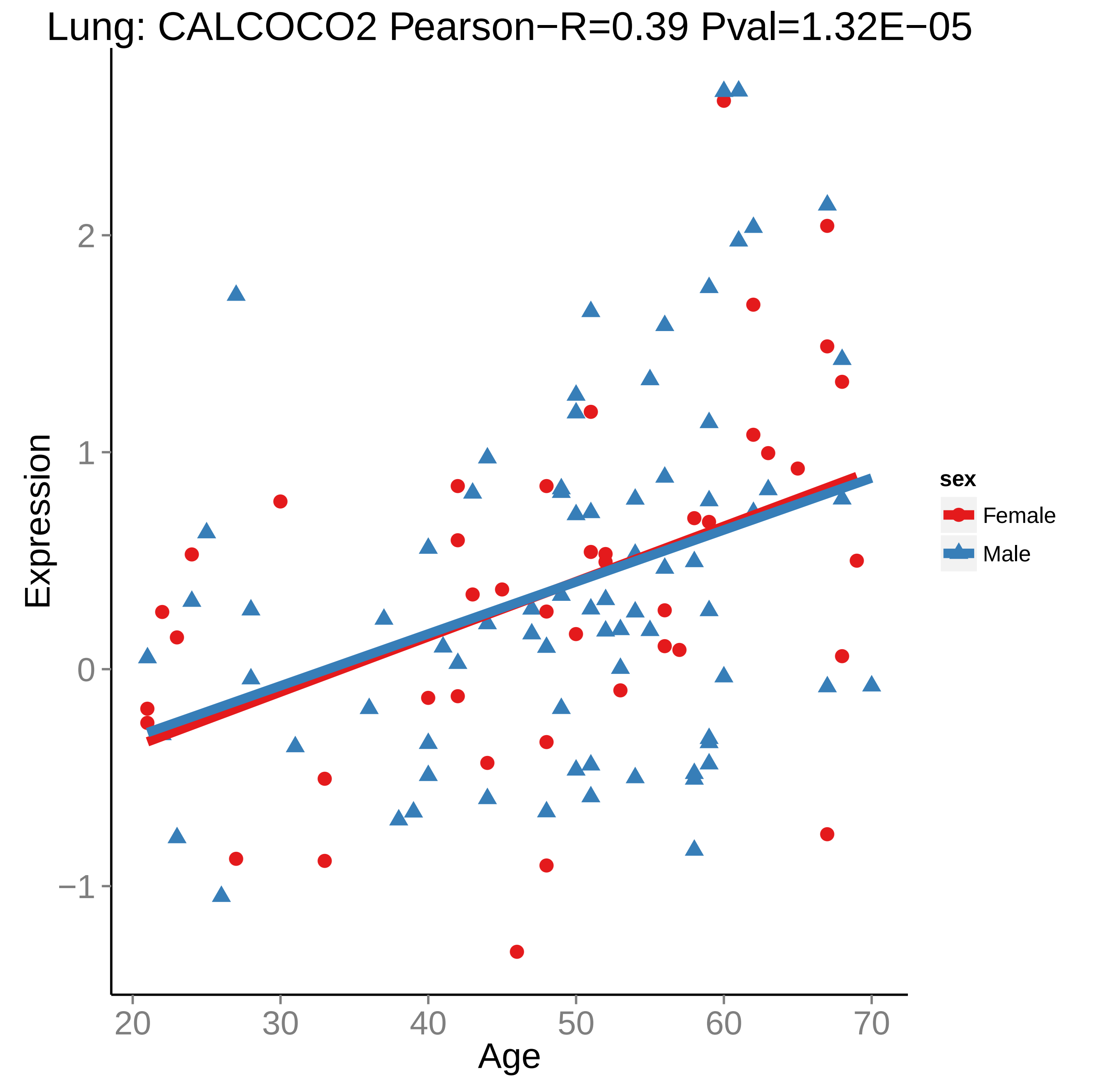

Lung: CALCOCO2 Pearson−R=0.39 Pval=1.32E−05
Expression
Age
2
1
0
−1
20
30
40
50
60
70
sex
Female
Male

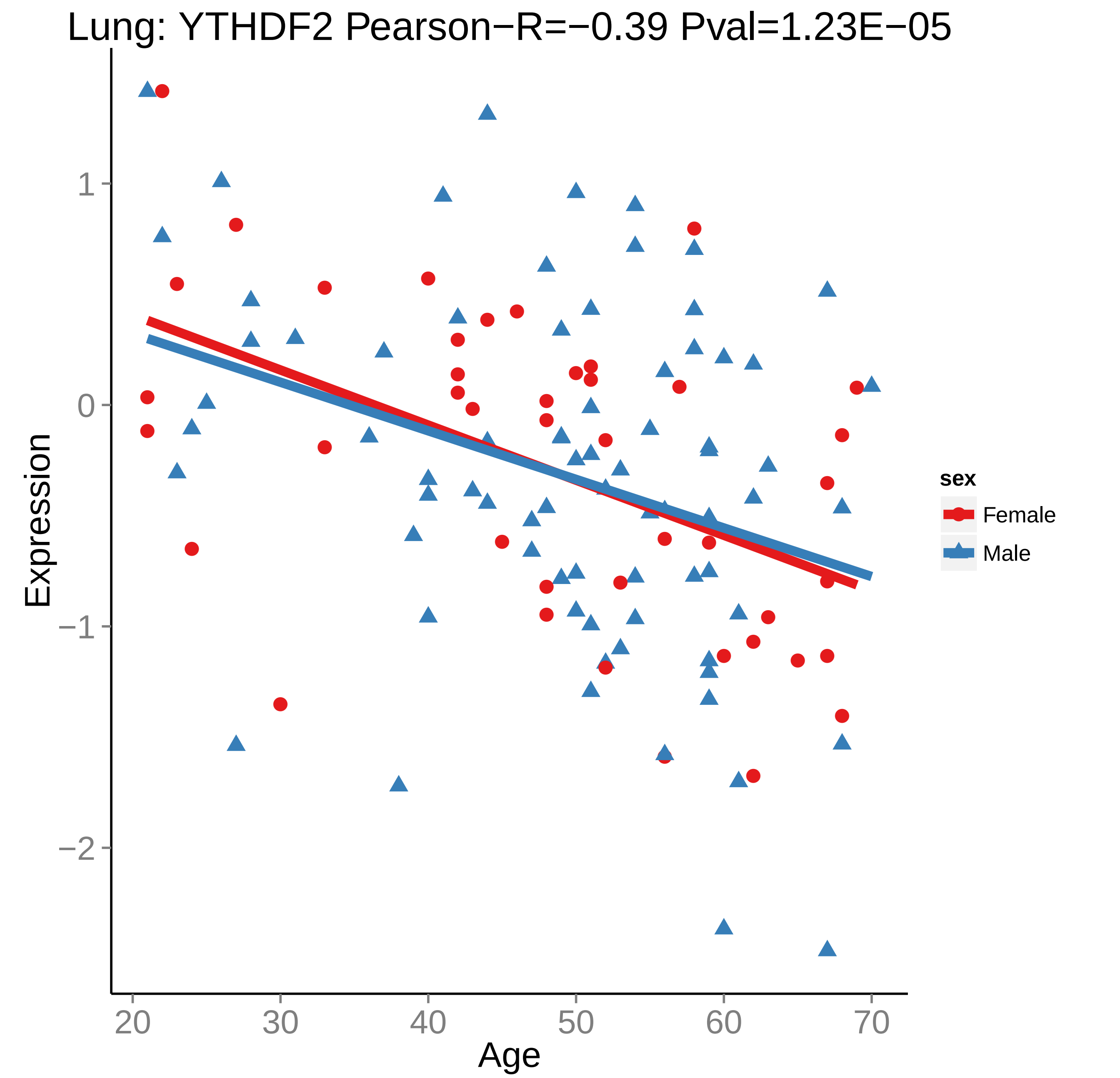

Lung: YTHDF2 Pearson−R=−0.39 Pval=1.23E−05
Expression
Age
1
0
−1
−2
20
30
40
50
60
70
sex
Female
Male

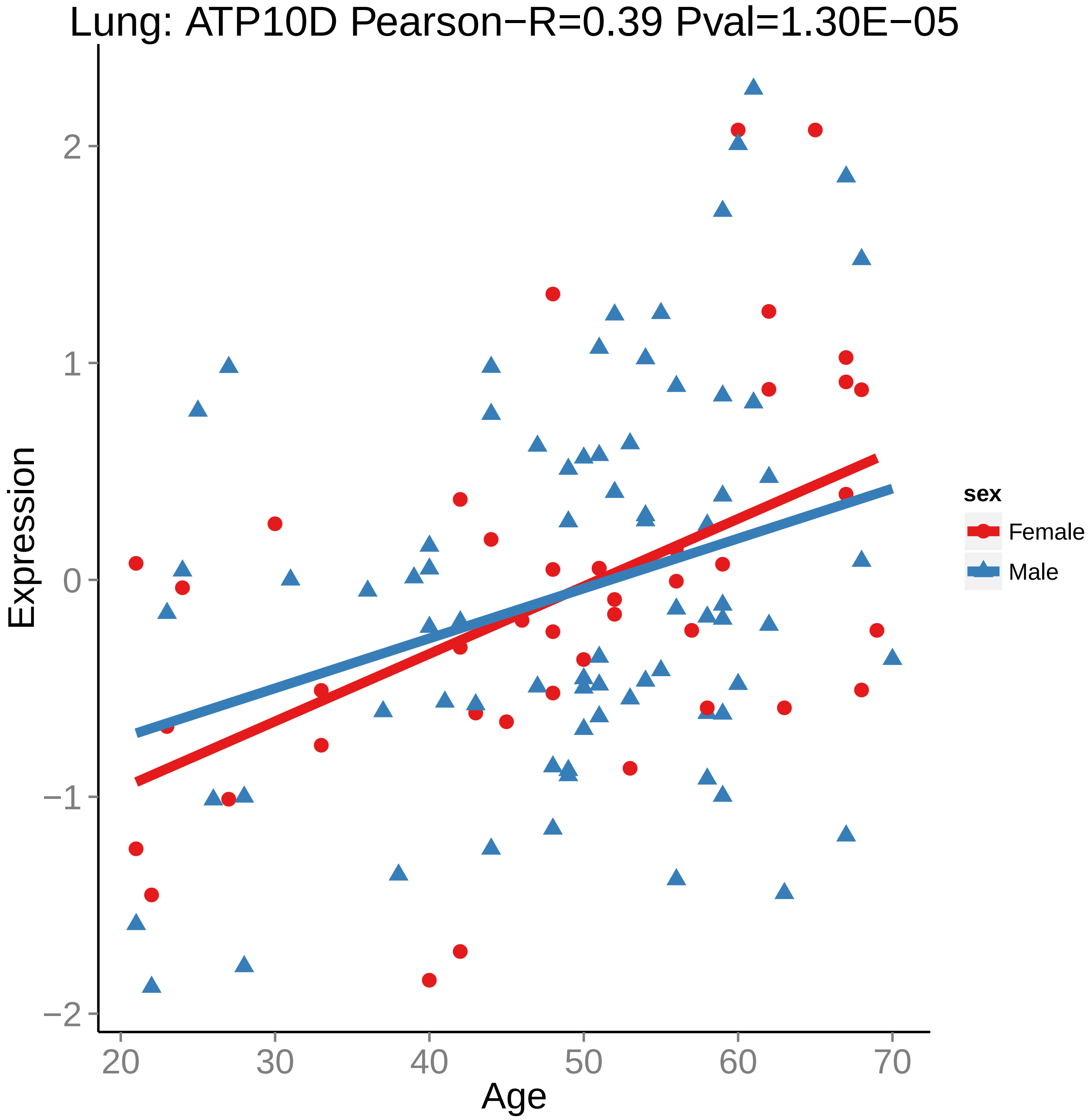

Lung: ATP10D Pearson−R=0.39 Pval=1.30E−05
Expression
Age
2
1
0
−1
−2
20
30
40
50
60
70
sex
Female
Male

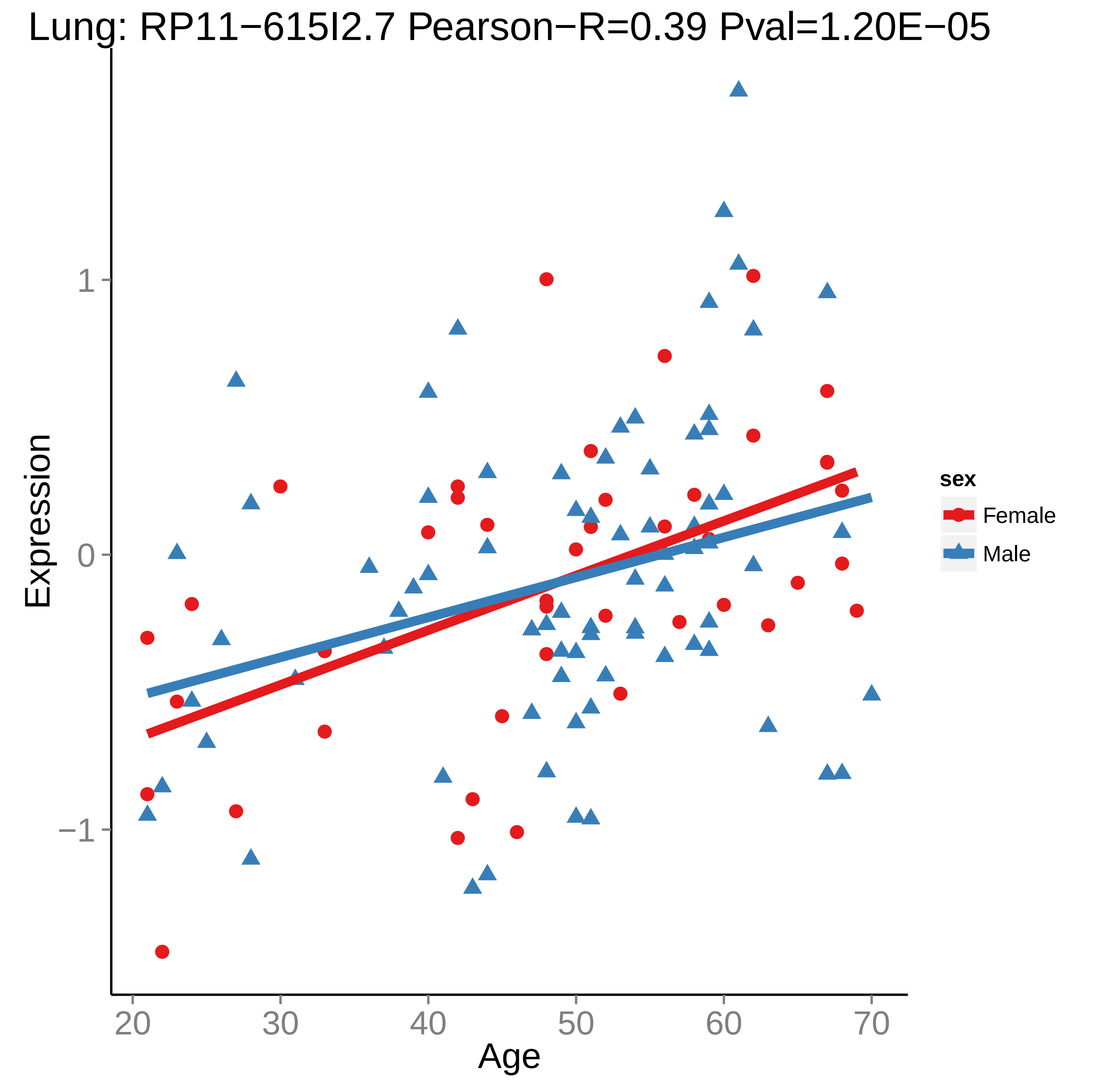

Lung: RP11−615I2.7 Pearson−R=0.39 Pval=1.20E−05
Expression
1
0
−1
20
30
40
50
60
70
Age
sex
Female
Male

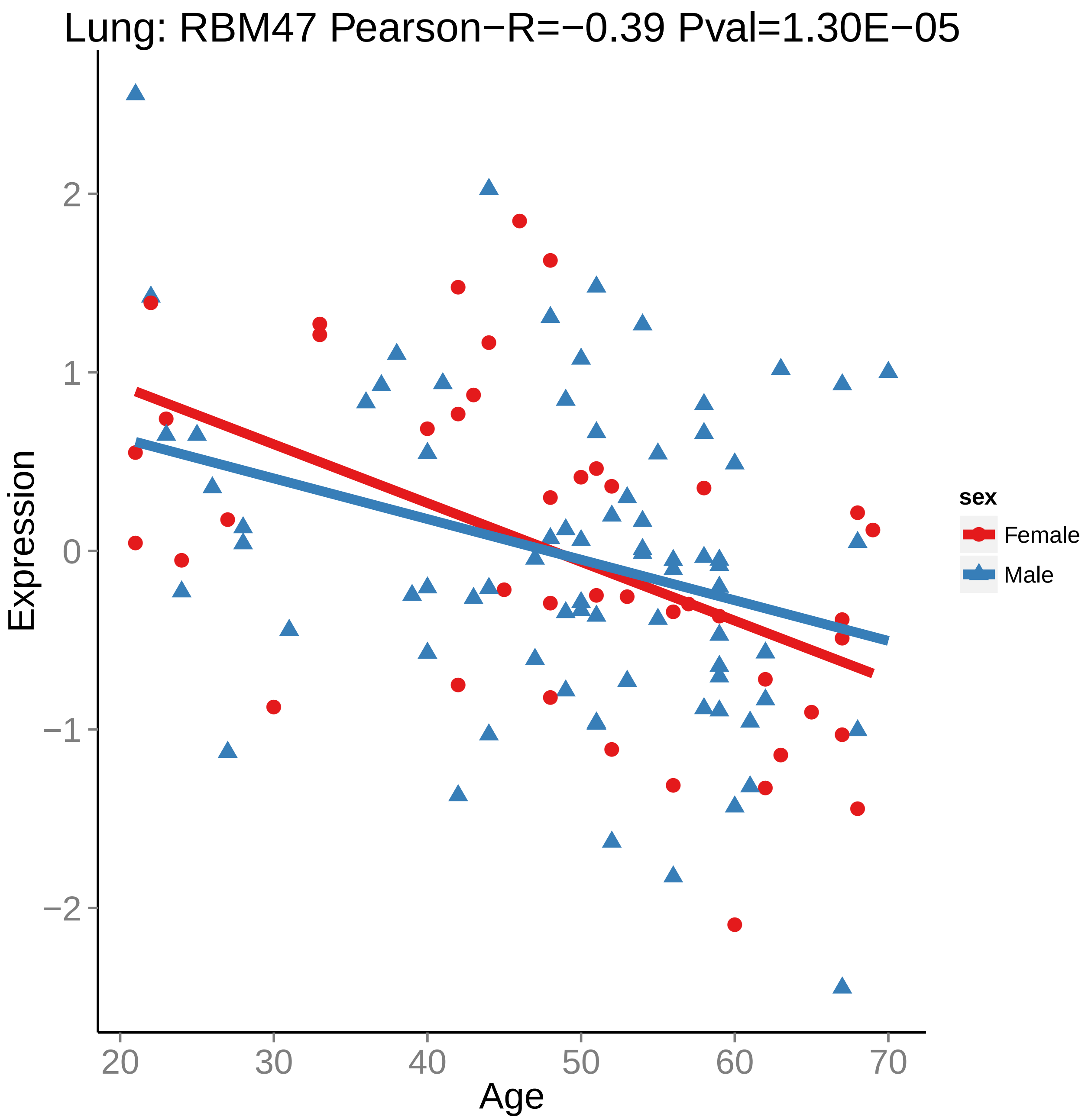

Lung: RBM47 Pearson−R=−0.39 Pval=1.30E−05
Expression
2
1
0
−1
−2
Age
20
30
40
50
60
70
sex
Female
Male

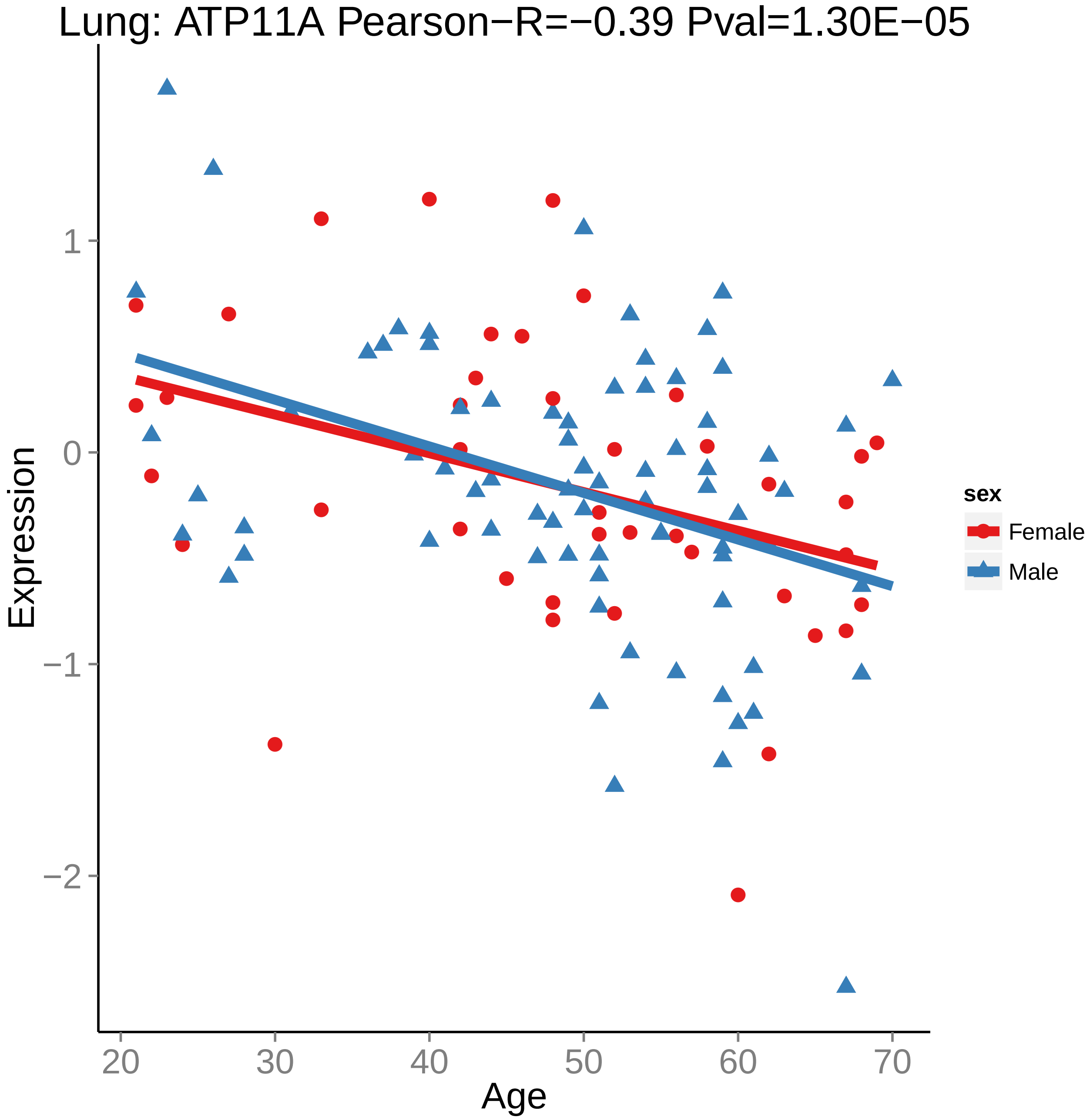

Lung: ATP11A Pearson−R=−0.39 Pval=1.30E−05
Expression
1
0
−1
−2
20
30
40
50
60
70
Age
sex
Female
Male

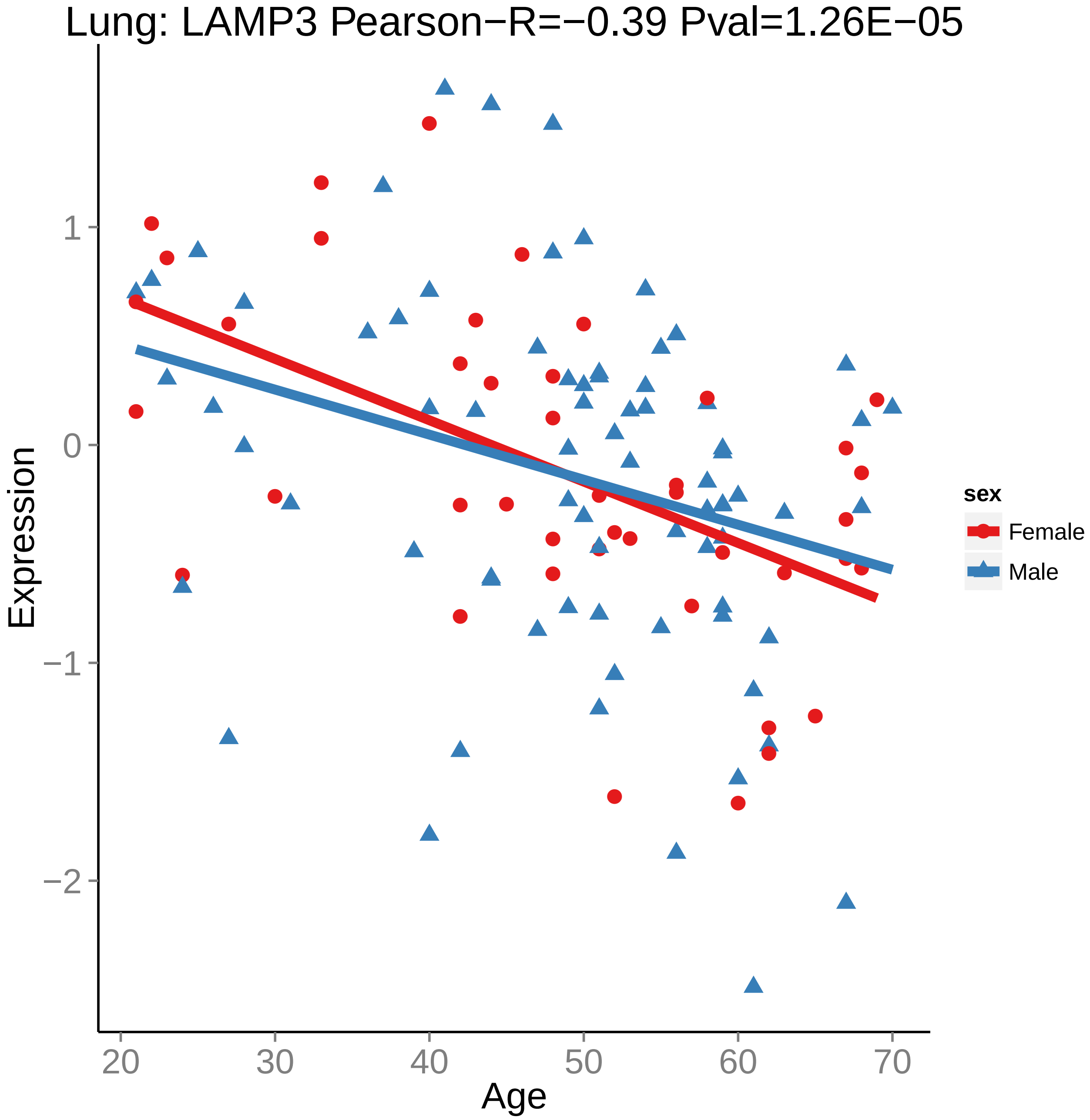

Lung: LAMP3 Pearson−R=−0.39 Pval=1.26E−05
Expression
1
0
−1
−2
20
30
40
50
60
70
Age
sex
Female
Male

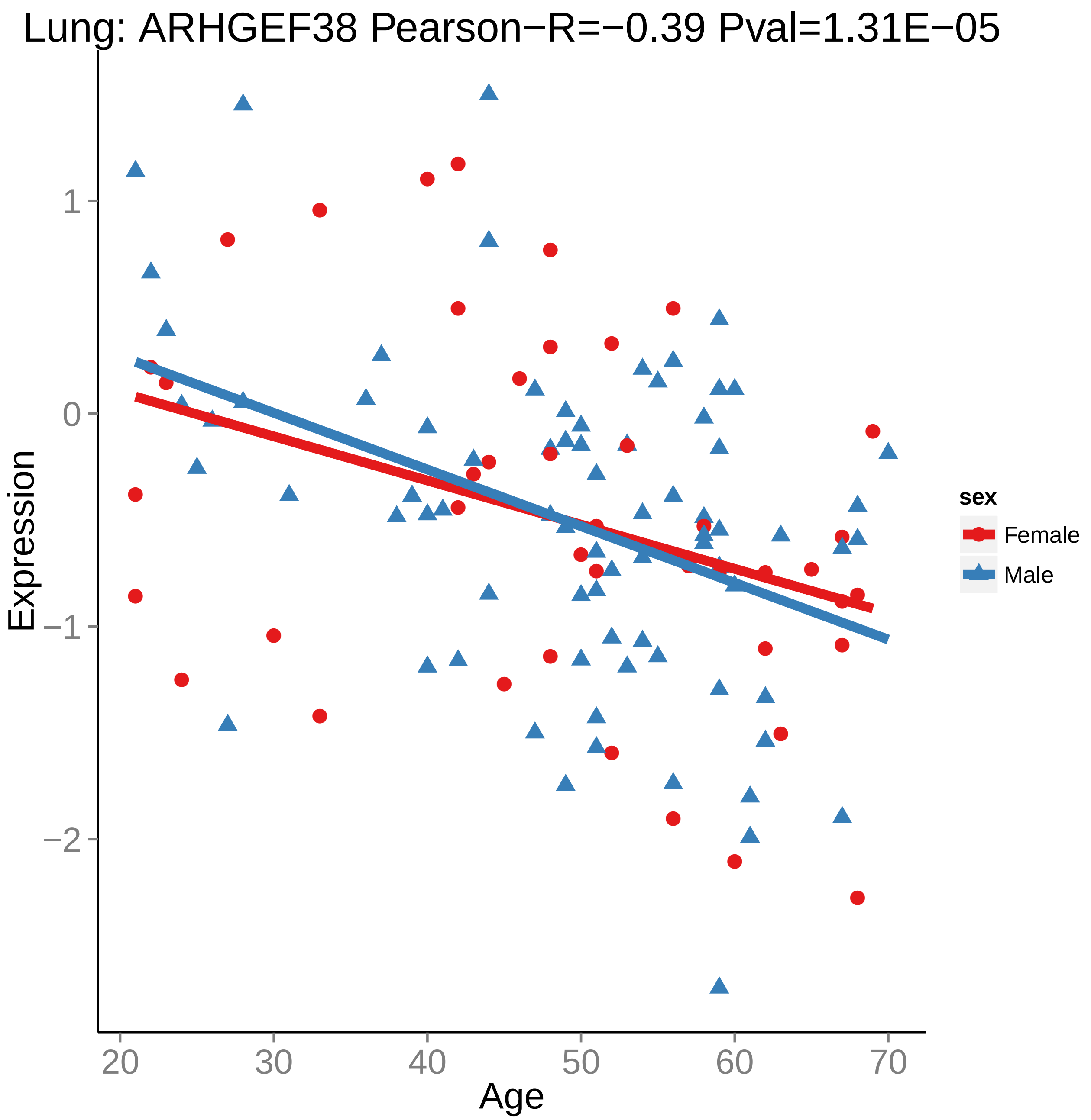

Lung: ARHGEF38 Pearson−R=−0.39 Pval=1.31E−05
Expression
1
0
−1
−2
20
30
40
50
60
70
Age
sex
Female
Male

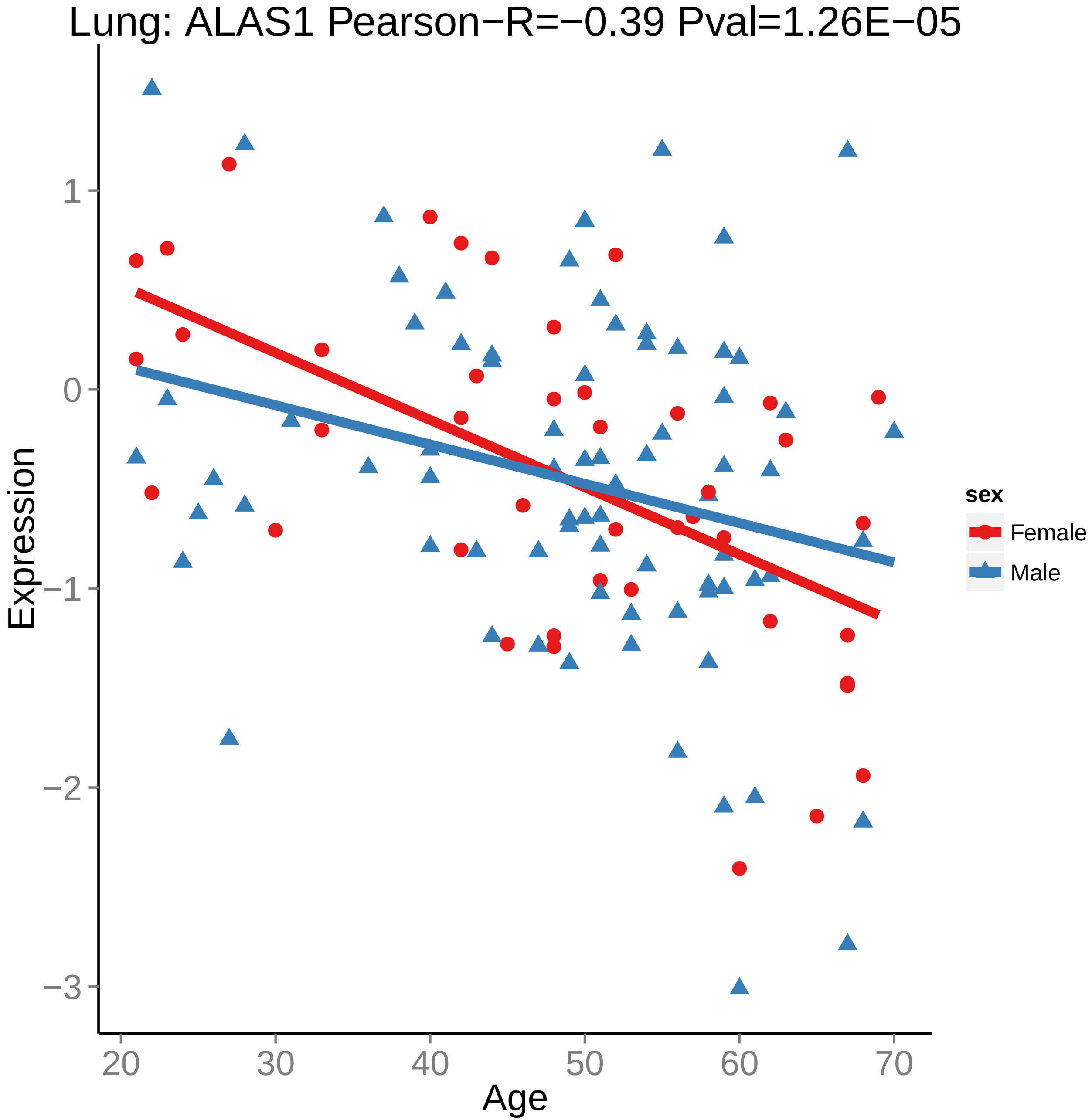
Lung: ALAS1 Pearson−R=−0.39 Pval=1.26E−05
Expression
Age
1
0
−1
−2
−3
20
30
40
50
60
70
sex
Female
Male

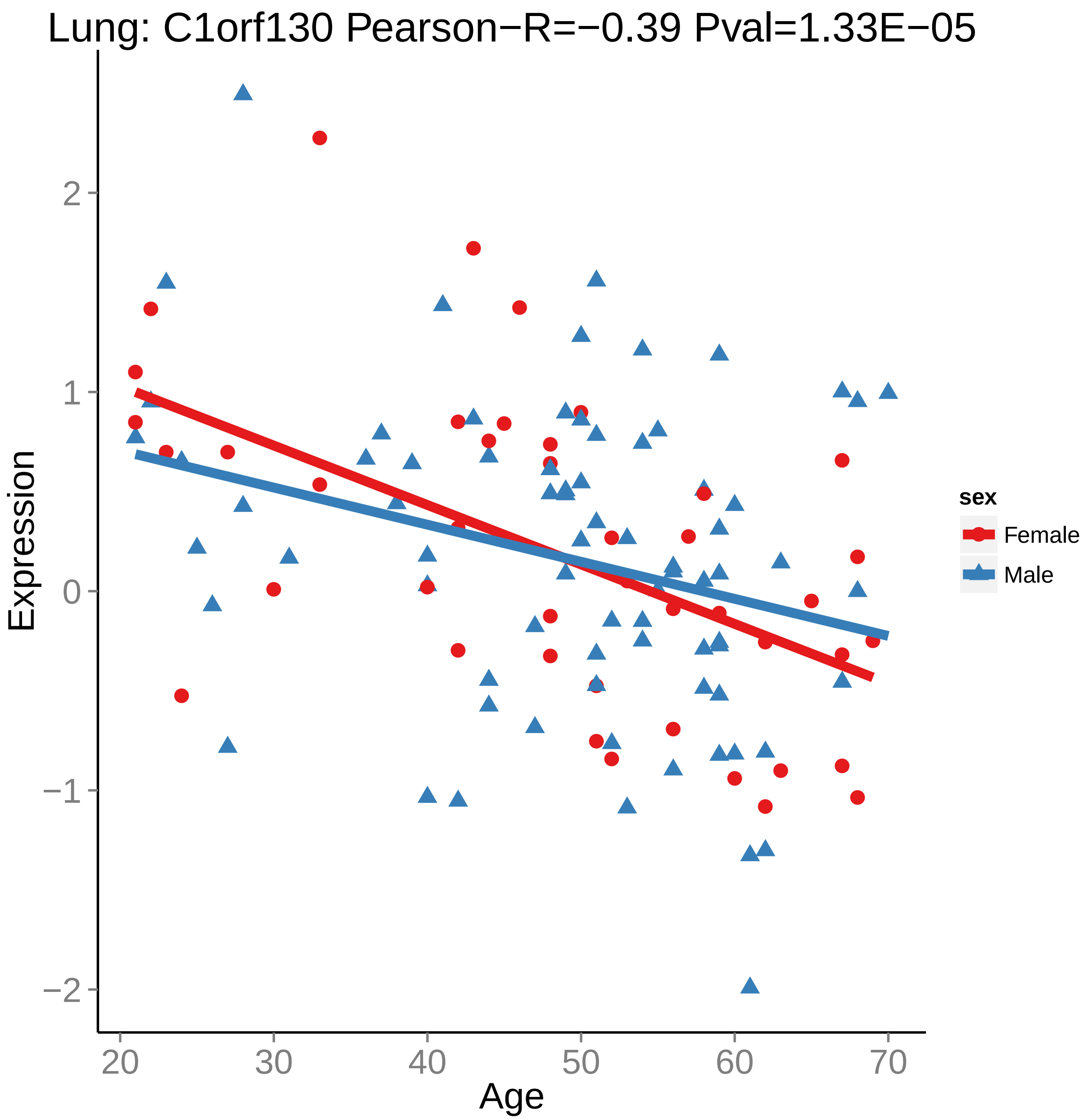
Lung: C1orf130 Pearson−R=−0.39 Pval=1.33E−05
Expression
2
1
0
−1
−2
20
30
40
50
60
70
Age
sex
Female
Male

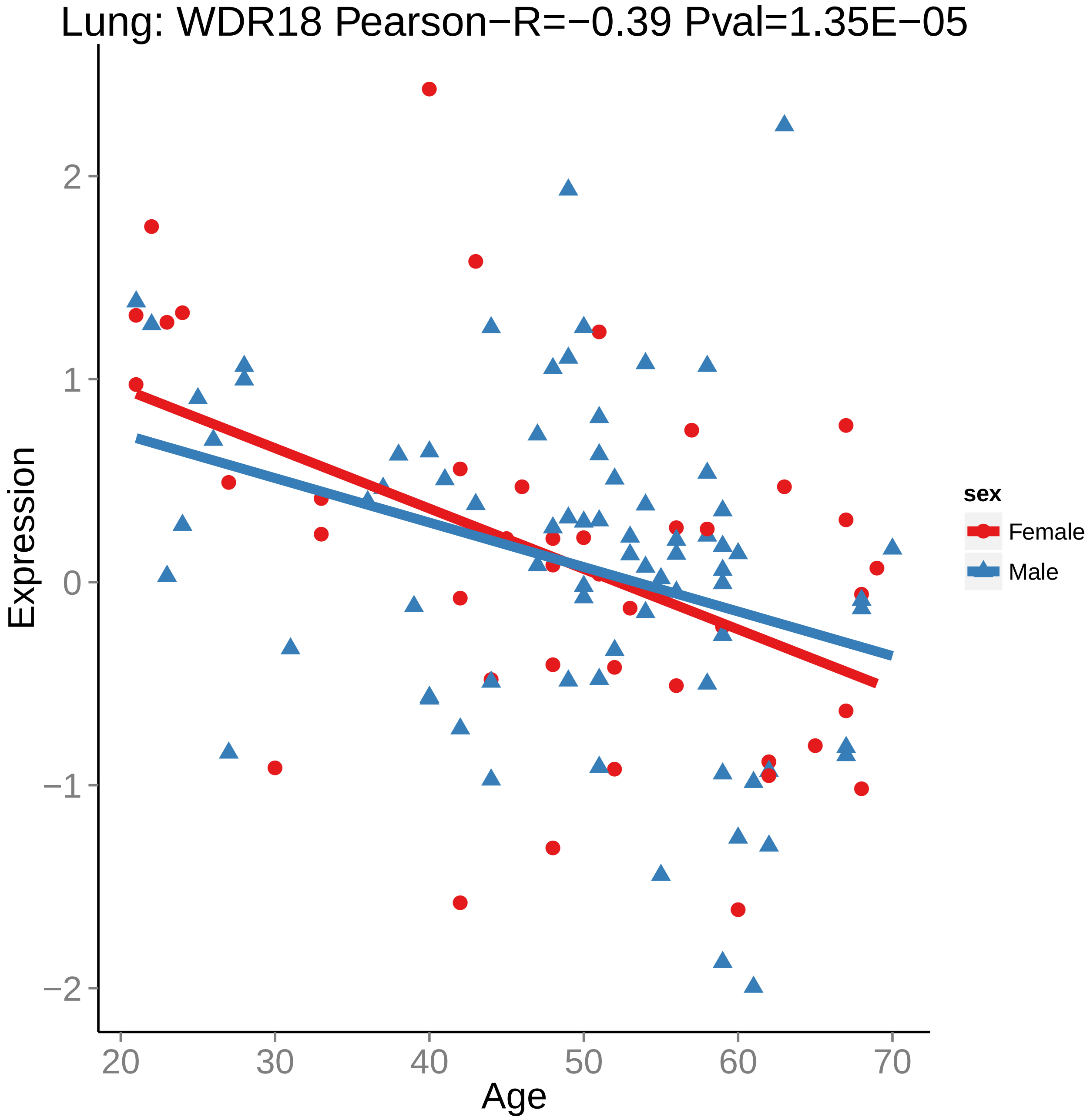
Lung: WDR18 Pearson−R=−0.39 Pval=1.35E−05
Expression
2
1
0
−1
−2
20
30
40
50
60
70
Age
sex
Female
Male

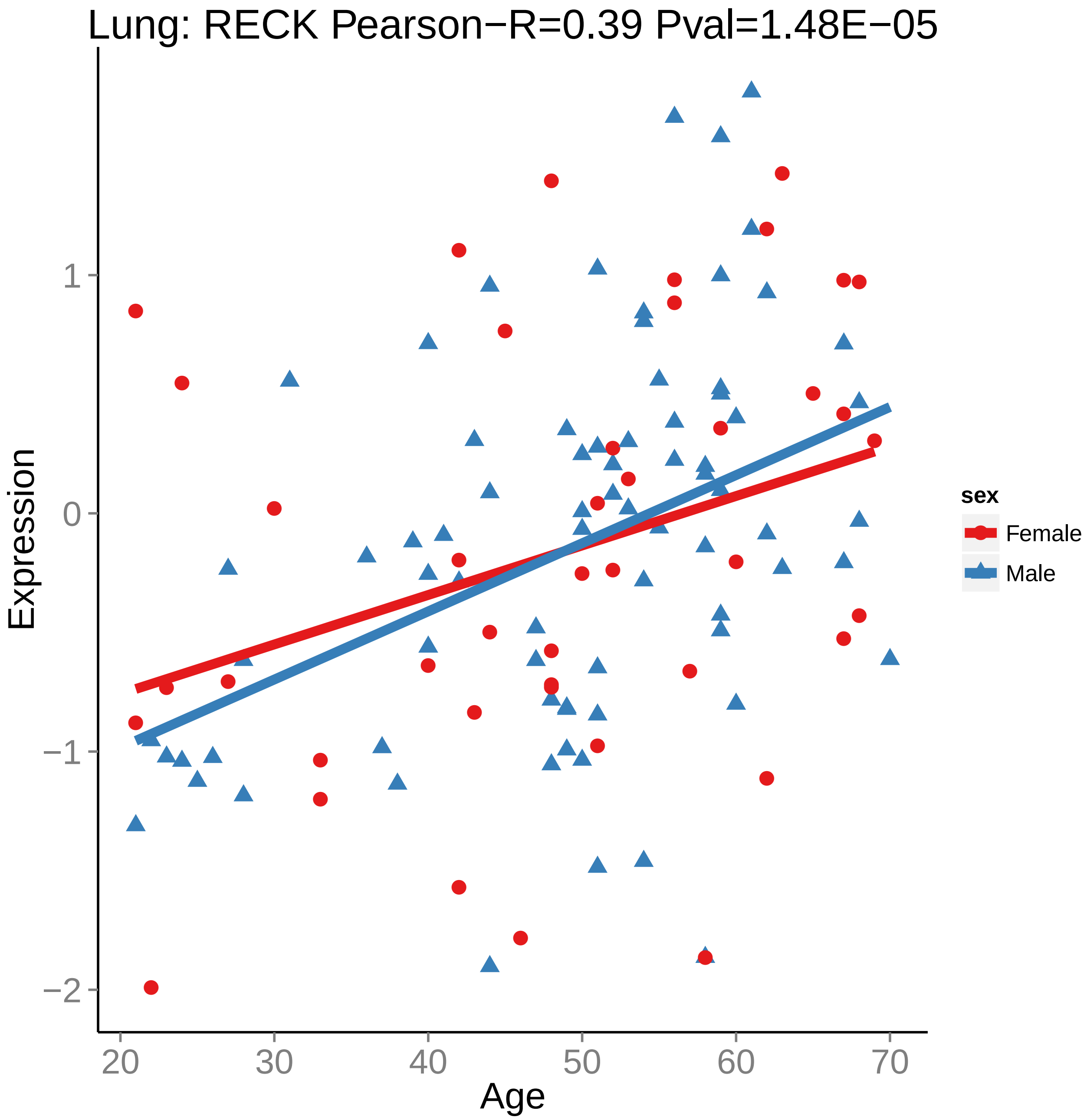

Lung: RECK Pearson−R=0.39 Pval=1.48E−05
Expression
1
0
−1
−2
20
30
40
50
60
70
Age
sex
Female
Male

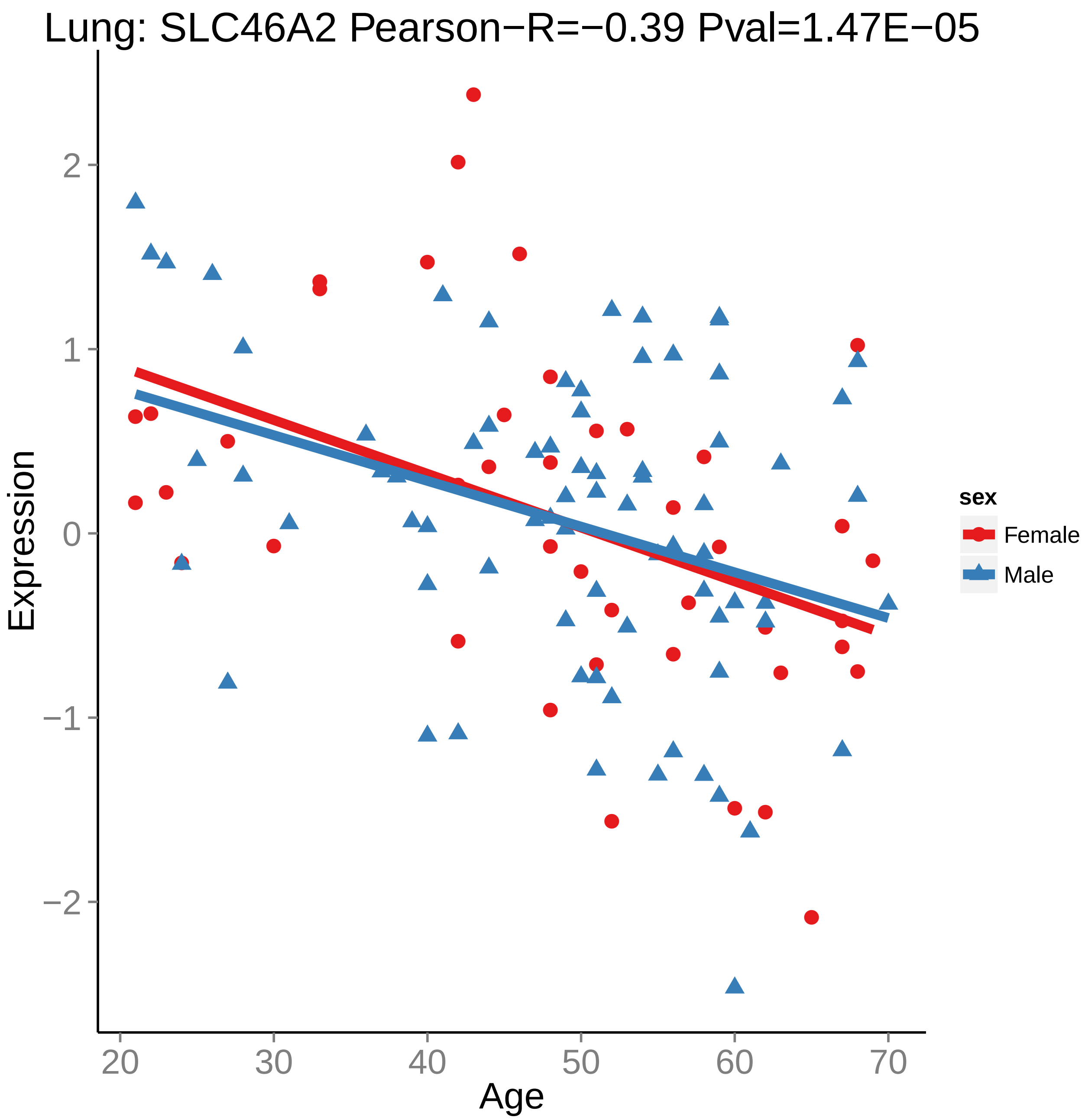

Lung: SLC46A2 Pearson−R=−0.39 Pval=1.47E−05
Expression
Age
2
1
0
−1
−2
20
30
40
50
60
70
sex
Female
Male

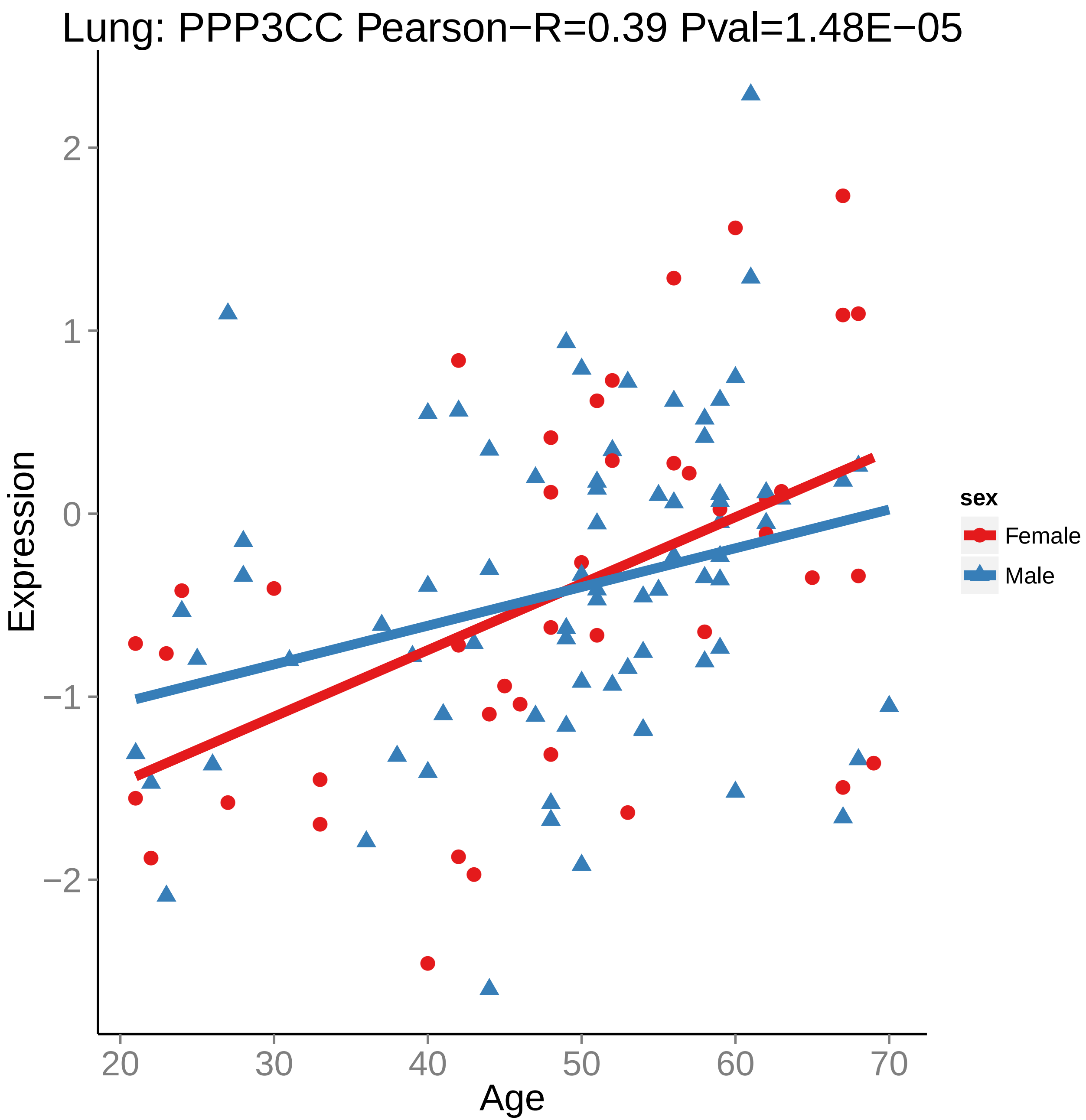

Lung: PPP3CC Pearson−R=0.39 Pval=1.48E−05
Expression
Age
2
1
0
−1
−2
20
30
40
50
60
70
sex
Female
Male

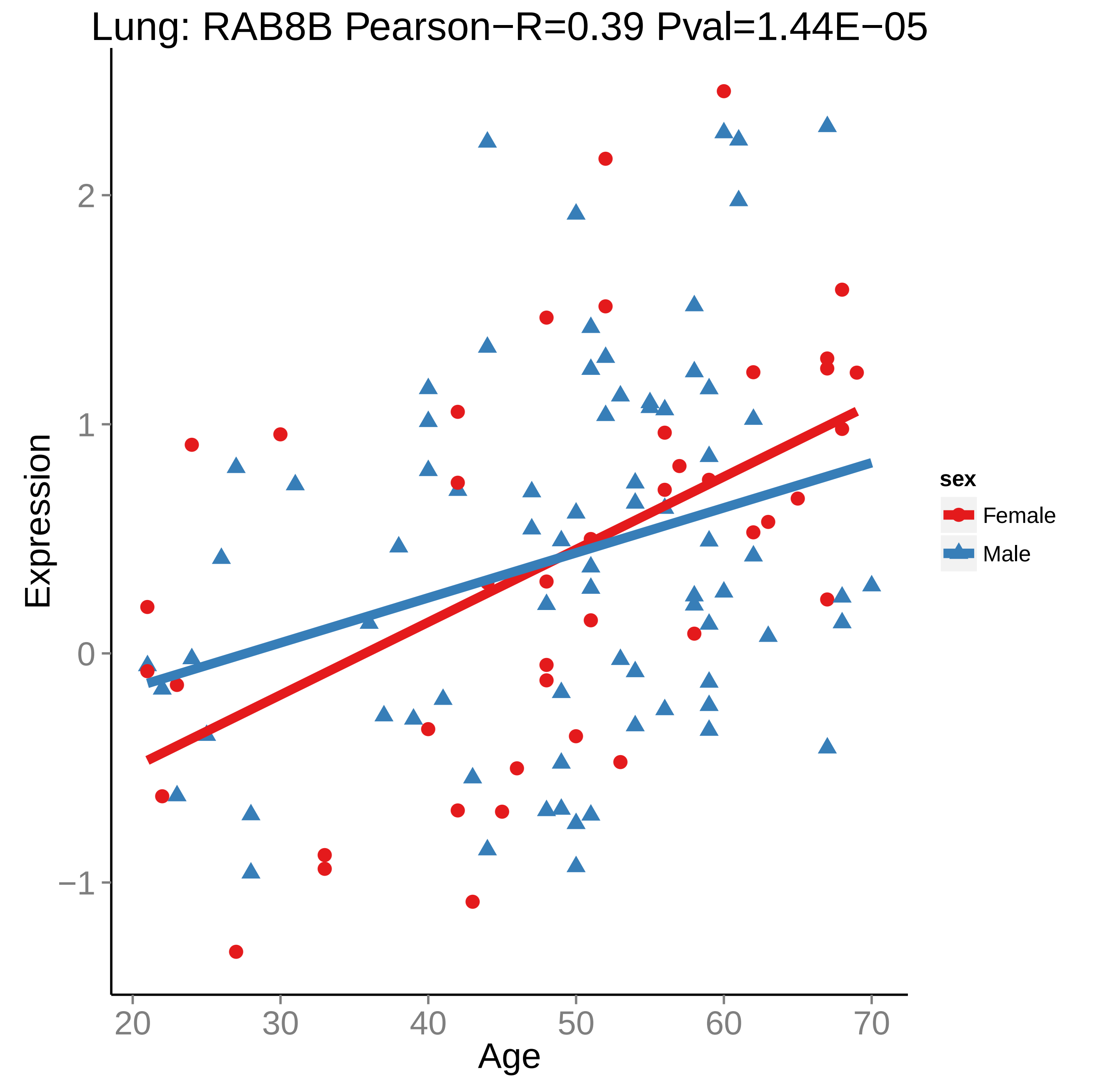

Lung: RAB8B Pearson−R=0.39 Pval=1.44E−05
Expression
2
1
0
−1
Age
20
30
40
50
60
70
sex
Female
Male

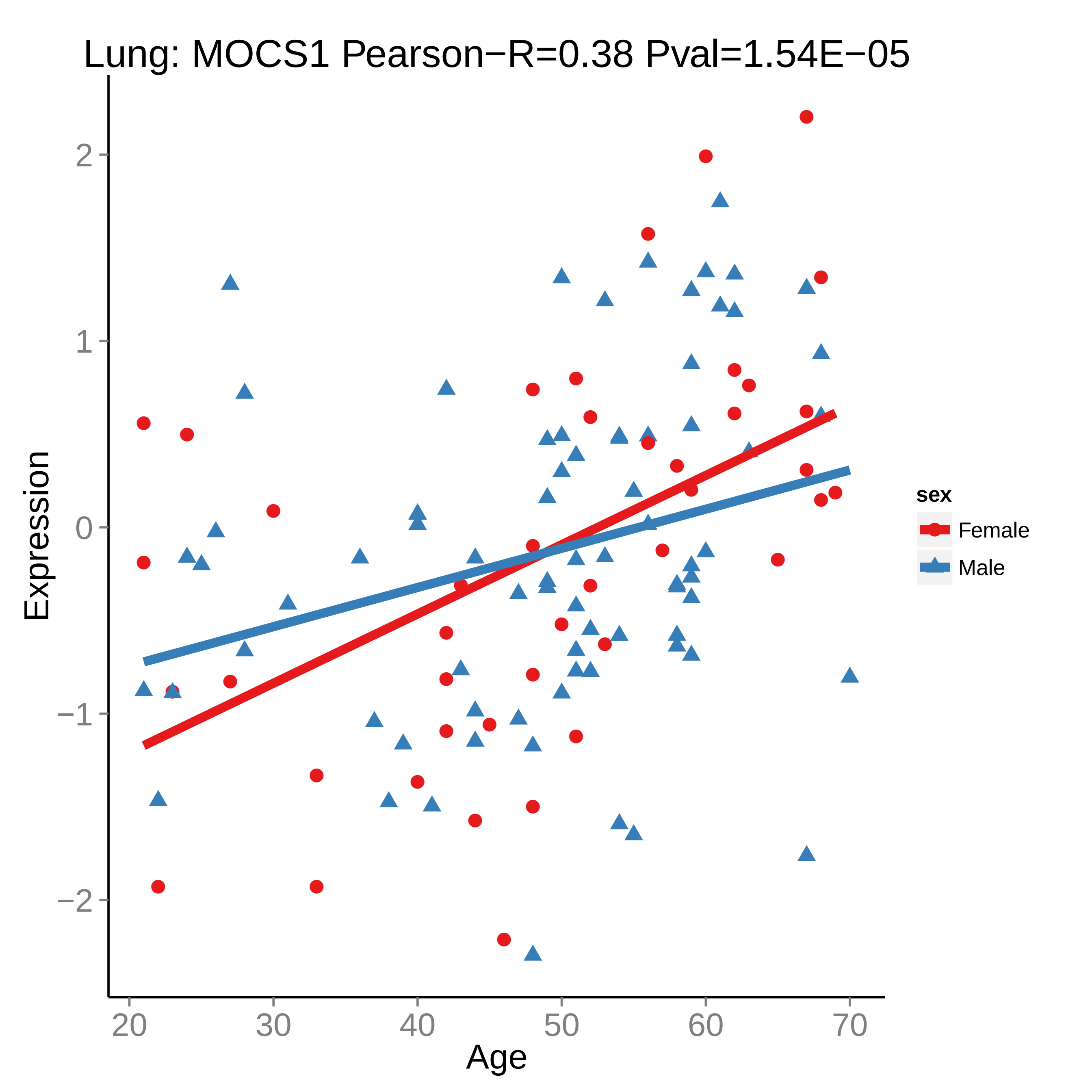

Lung: MOCS1 Pearson−R=0.38 Pval=1.54E−05
Expression
2
1
0
−1
−2
20
30
40
50
60
70
Age
sex
Female
Male

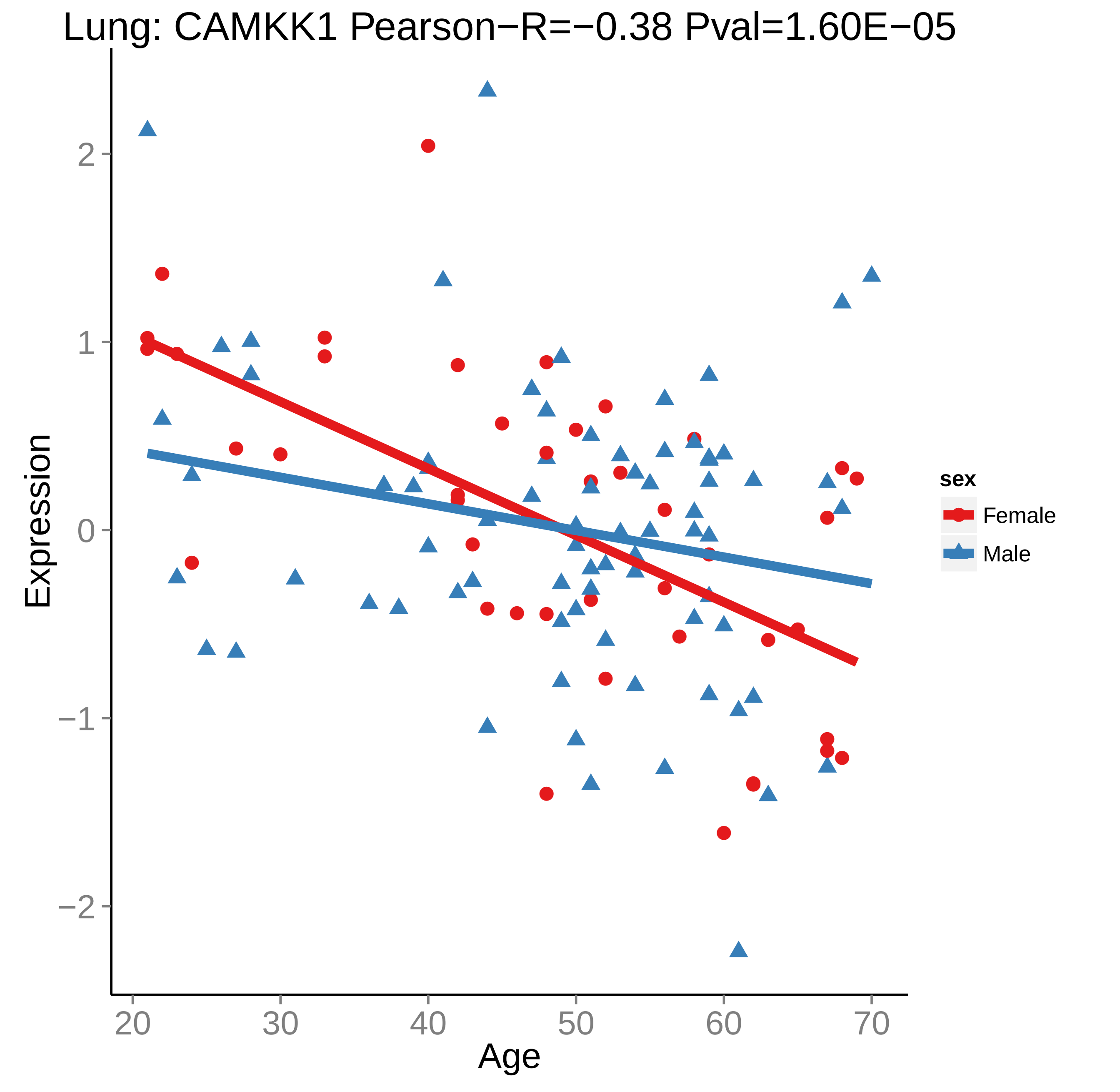

Lung: CAMKK1 Pearson−R=−0.38 Pval=1.60E−05
Expression
Age
2
1
0
−1
−2
20
30
40
50
60
70
sex
Female
Male

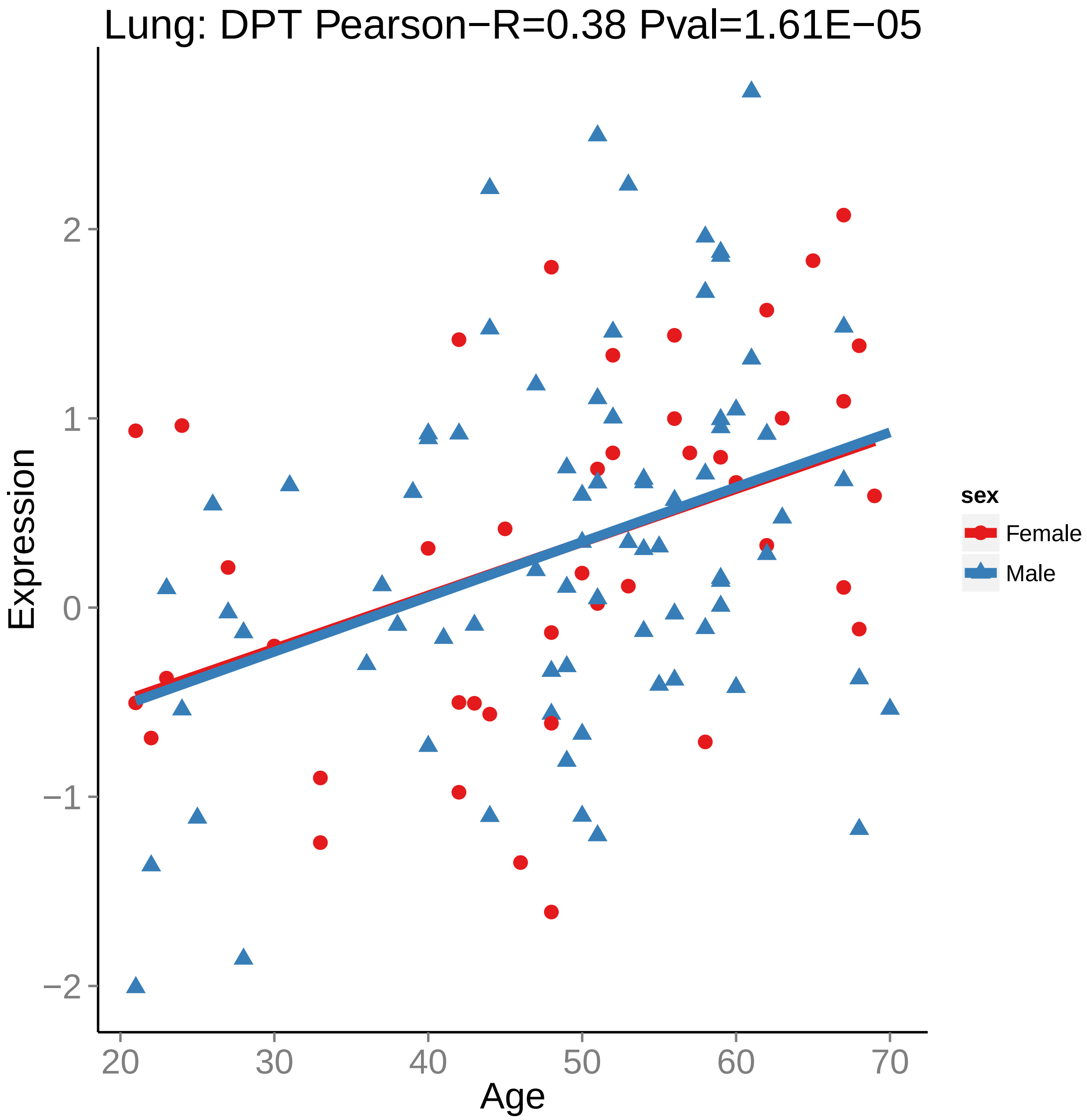
Lung: DPT Pearson−R=0.38 Pval=1.61E−05
Expression
Age
2
1
0
−1
−2
20
30
40
50
60
70
sex
Female
Male

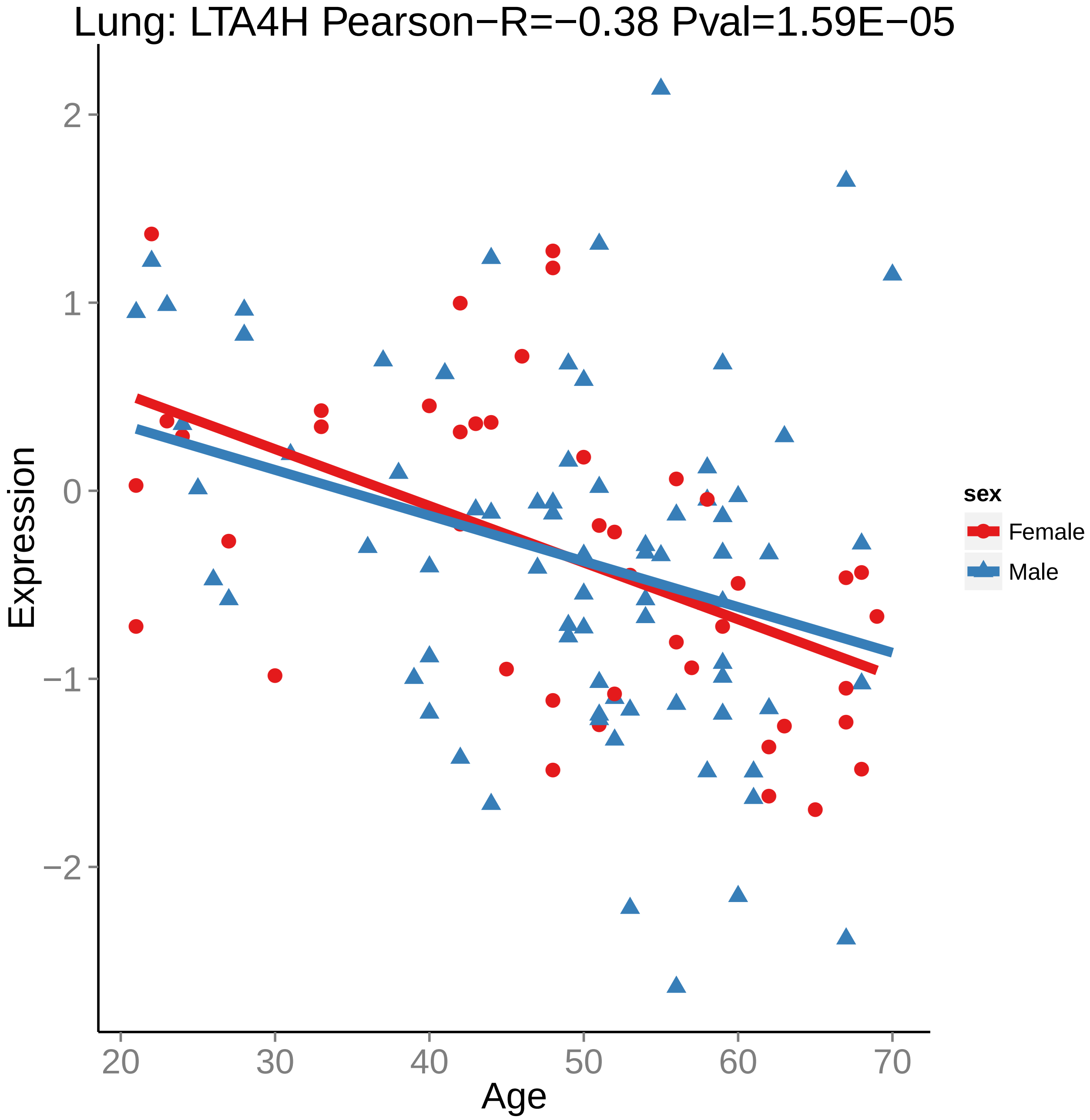

Lung: LTA4H Pearson−R=−0.38 Pval=1.59E−05
Expression
2
1
0
−1
−2
20
30
40
50
60
70
Age
sex
Female
Male

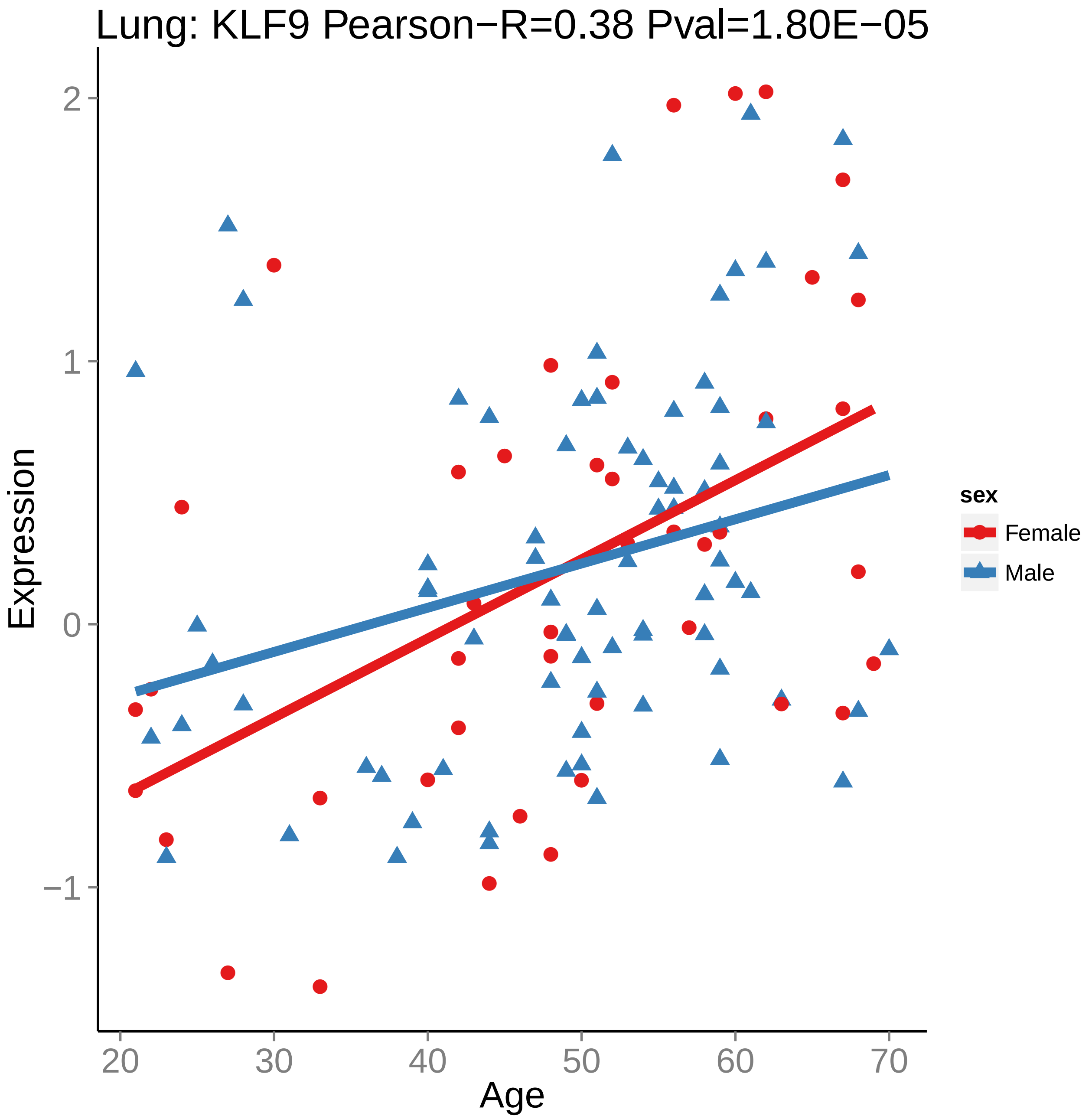

Lung: KLF9 Pearson−R=0.38 Pval=1.80E−05
Expression
2
1
0
−1
Age
20
30
40
50
60
70
sex
Female
Male

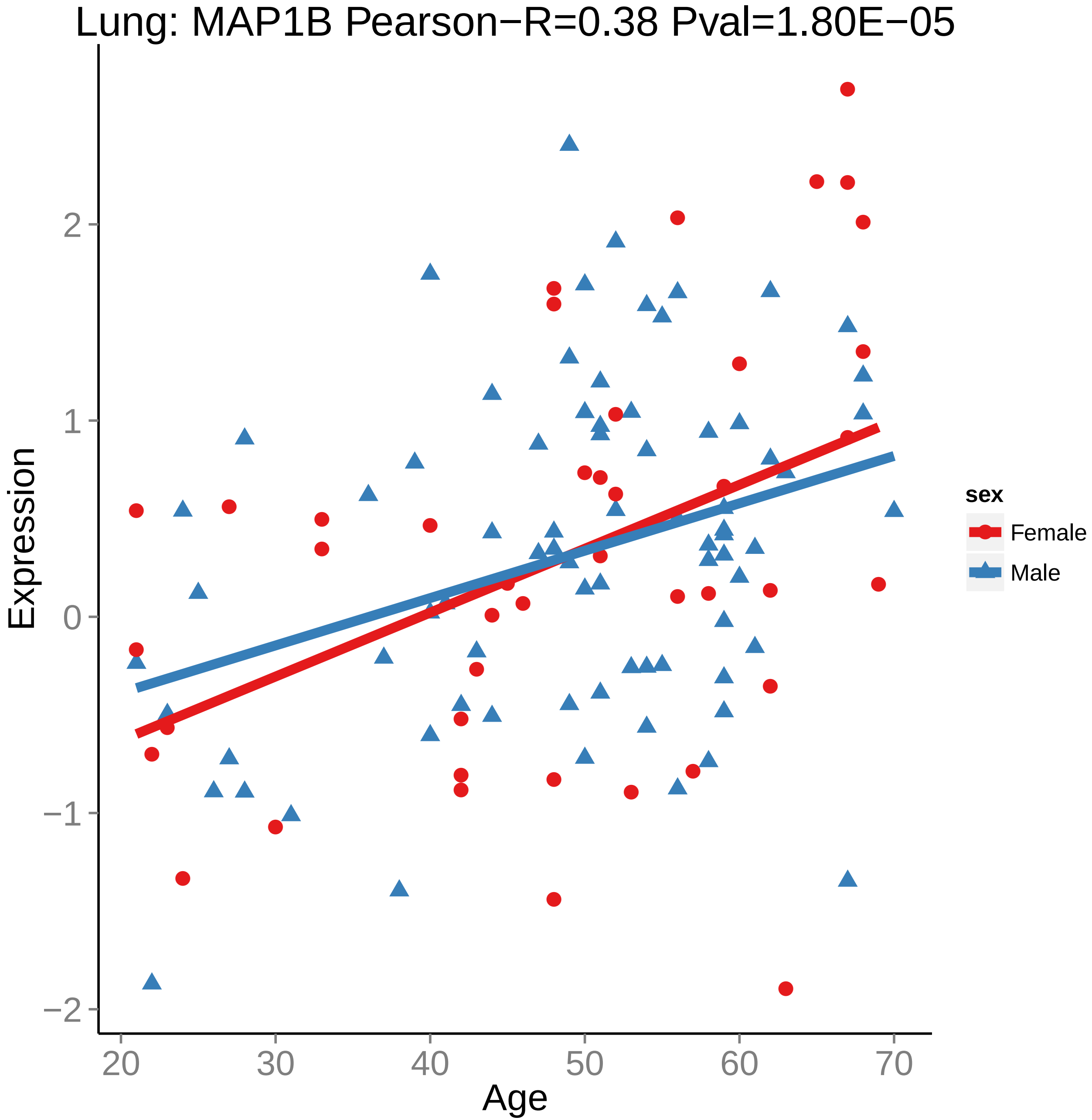

Lung: MAP1B Pearson−R=0.38 Pval=1.80E−05
Expression
2
1
0
−1
−2
20
30
40
50
60
70
Age
sex
Female
Male

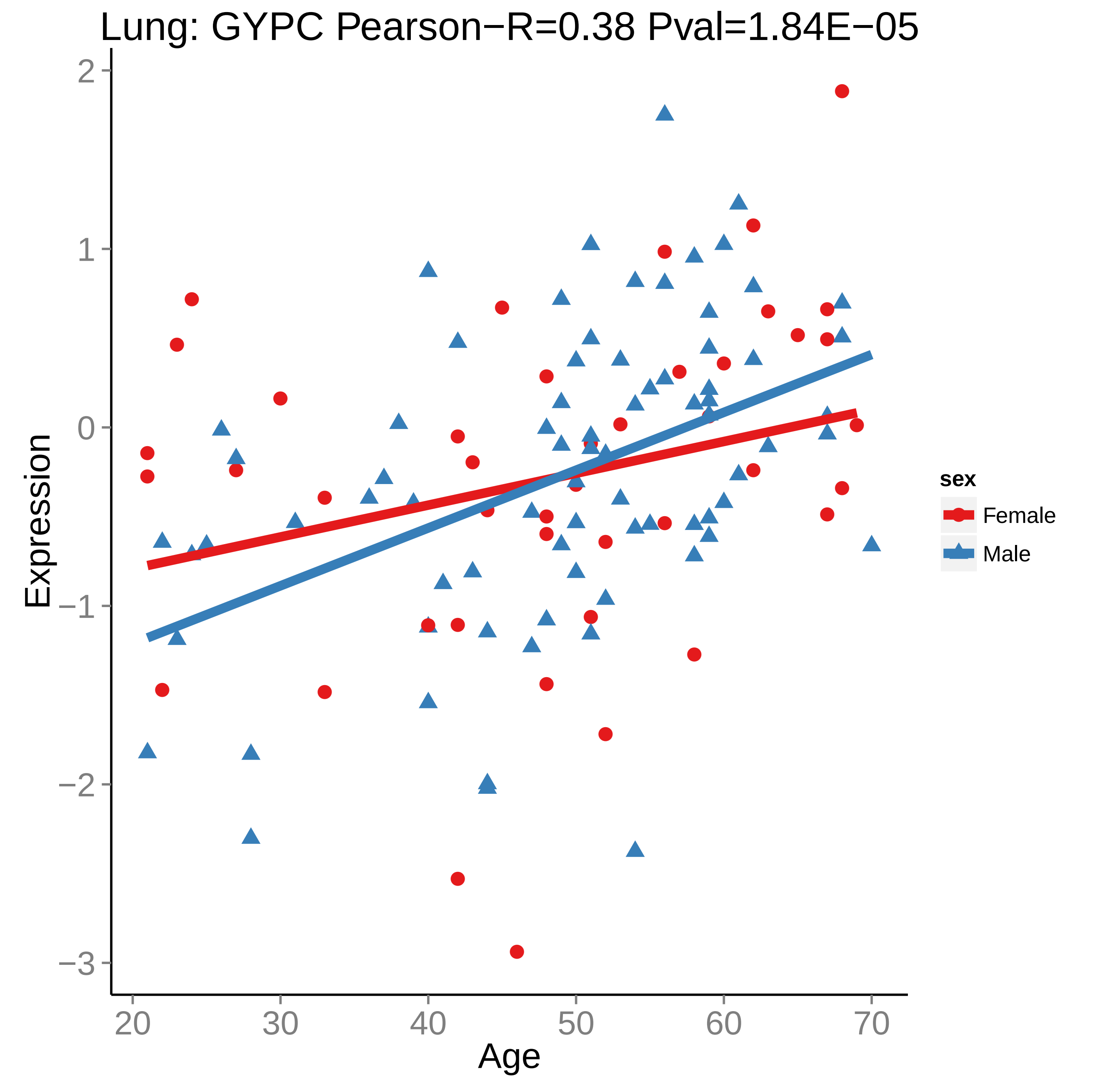

Lung: GYPC Pearson−R=0.38 Pval=1.84E−05
Expression
Age
sex
Female
Male
2
1
0
−1
−2
−3
20
30
40
50
60
70

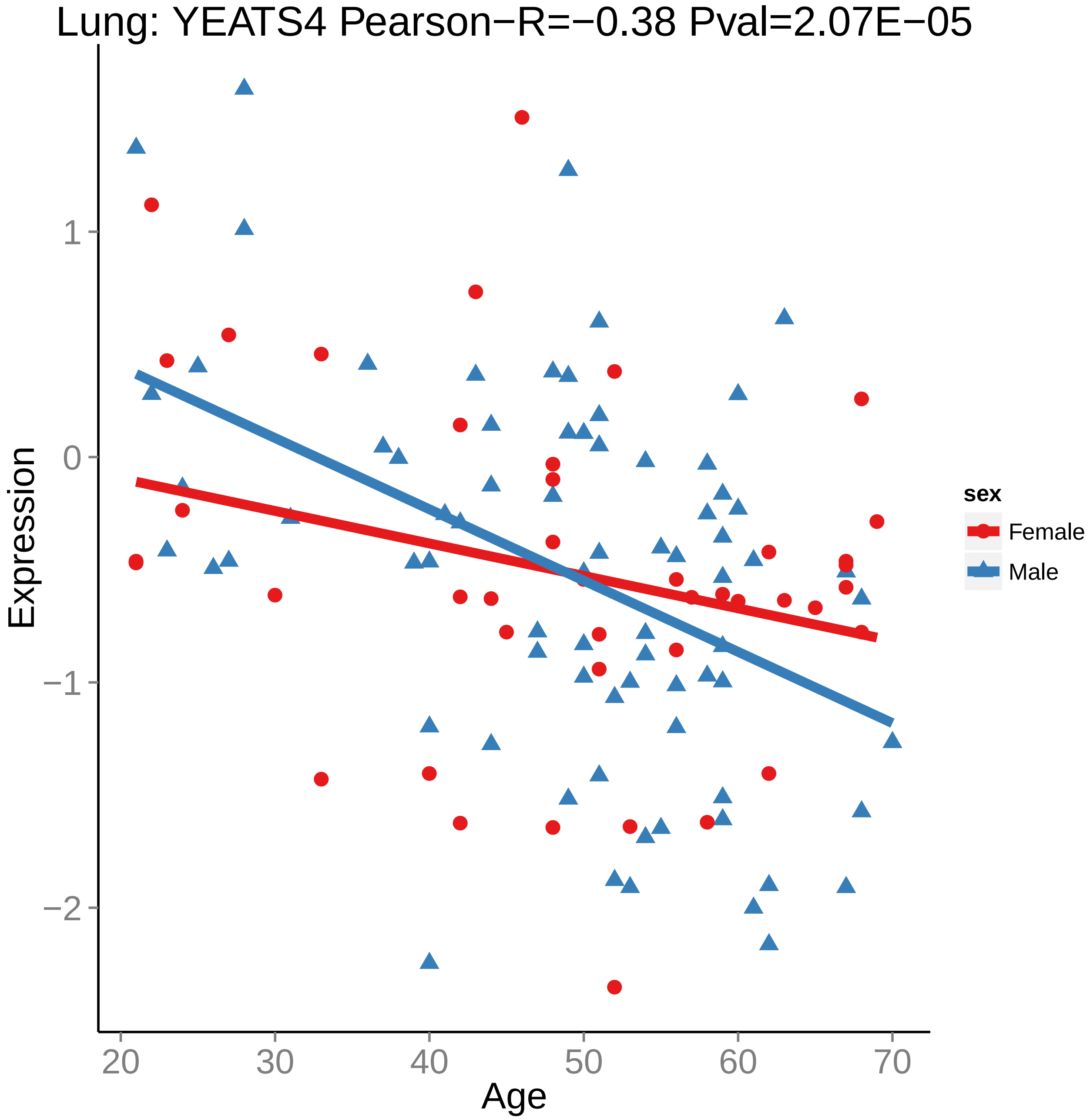

Lung: YEATS4 Pearson−R=−0.38 Pval=2.07E−05
Expression
1
0
−1
−2
Age
20
30
40
50
60
70
sex
Female
Male

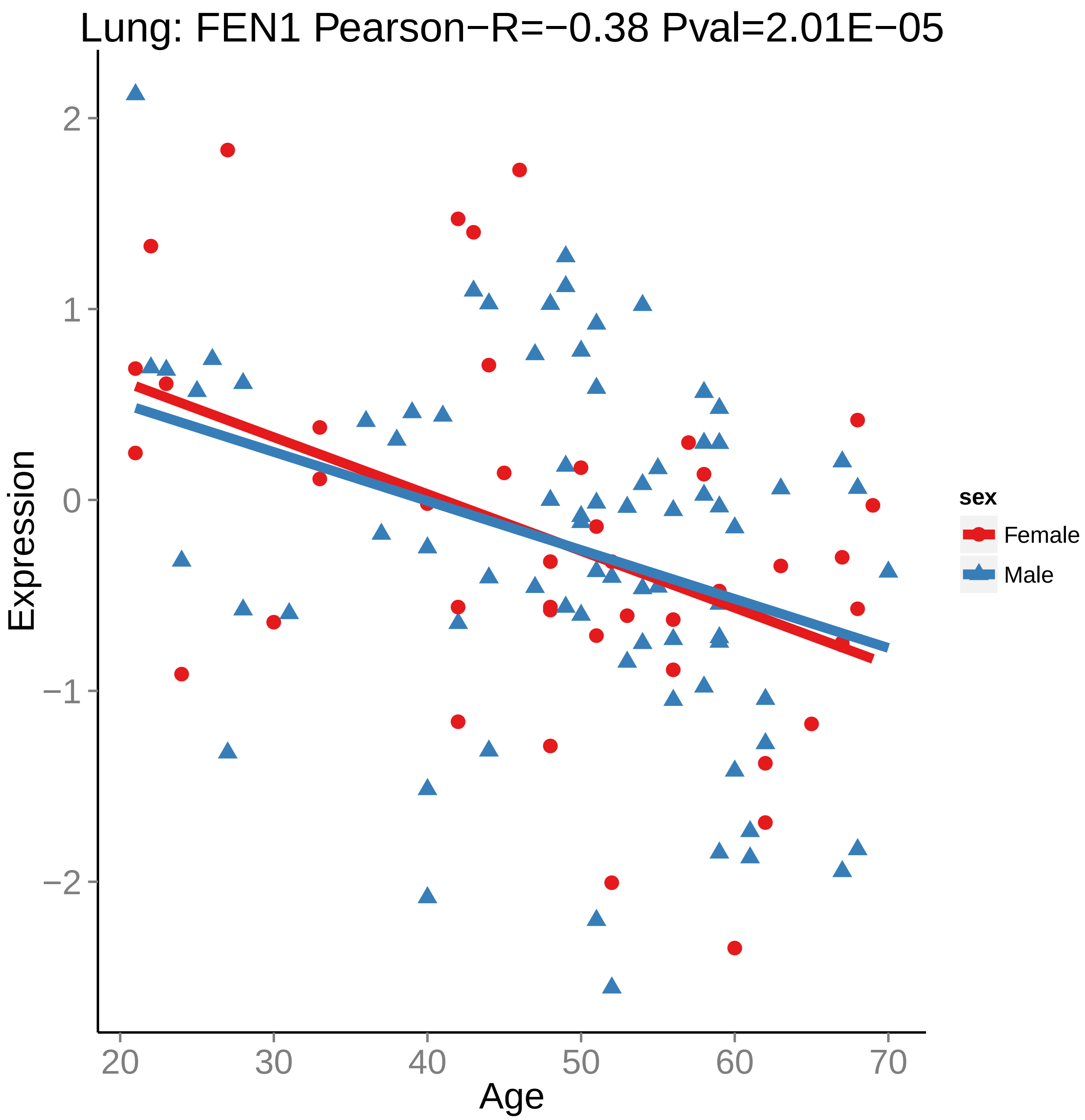
Lung: FEN1 Pearson−R=−0.38 Pval=2.01E−05
Expression
2
1
0
−1
−2
20
30
40
50
60
70
Age
sex
Female
Male

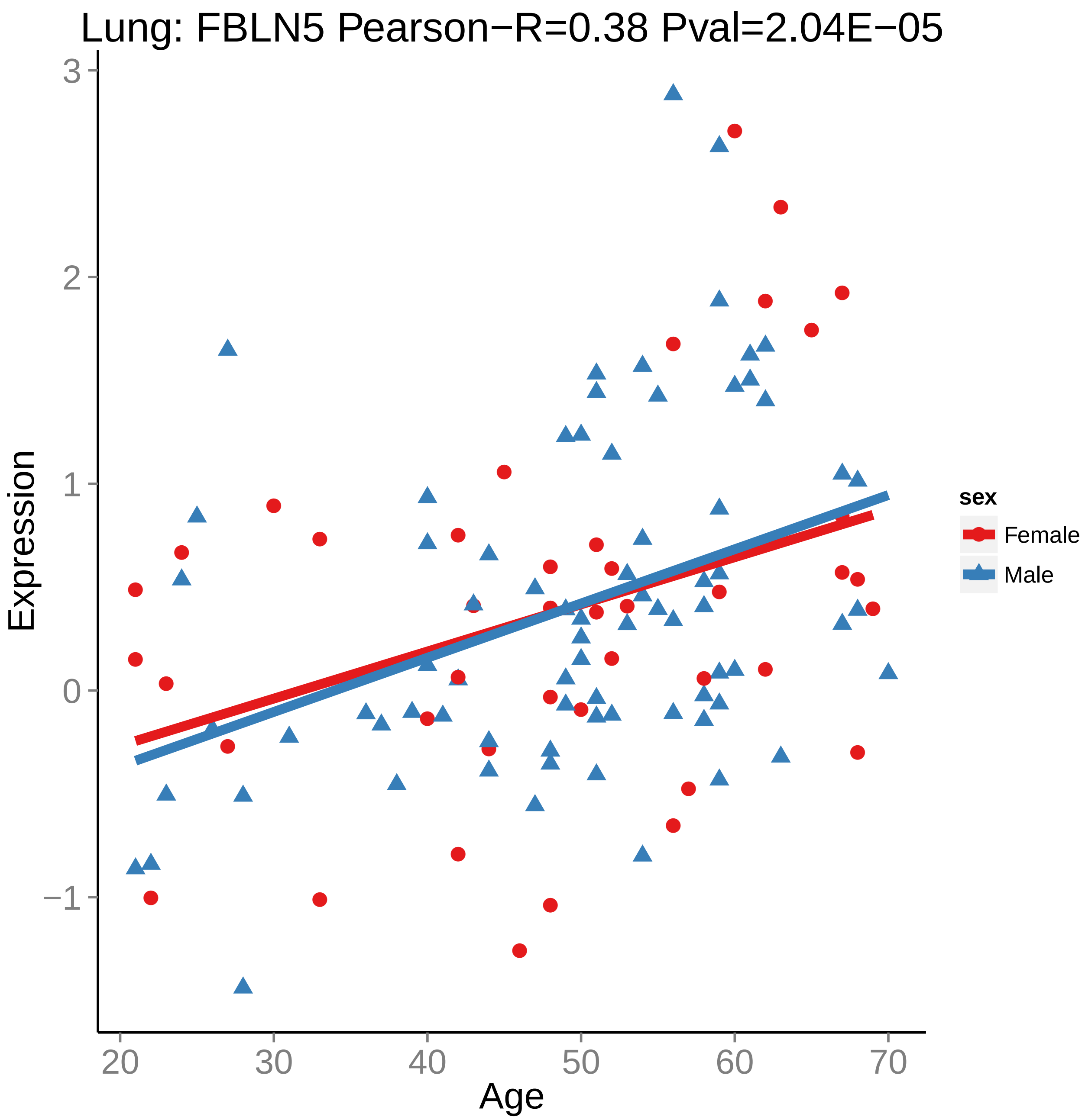

Lung: FBLN5 Pearson−R=0.38 Pval=2.04E−05
Expression
Age
3
2
1
0
−1
20
30
40
50
60
70
sex
Female
Male

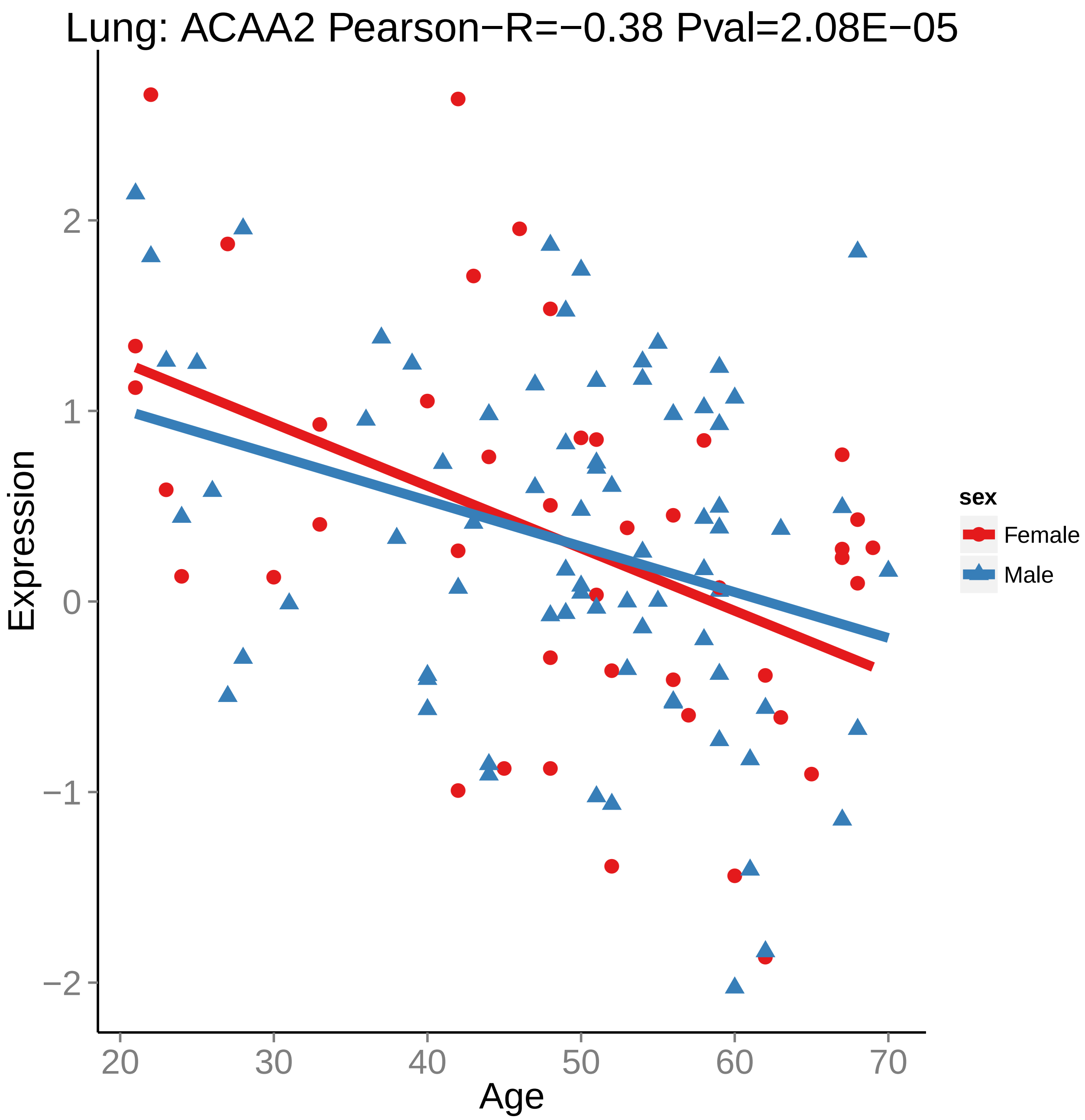
Lung: ACAA2 Pearson−R=−0.38 Pval=2.08E−05
Expression
Age
2
1
0
−1
−2
20
30
40
50
60
70
sex
Female
Male

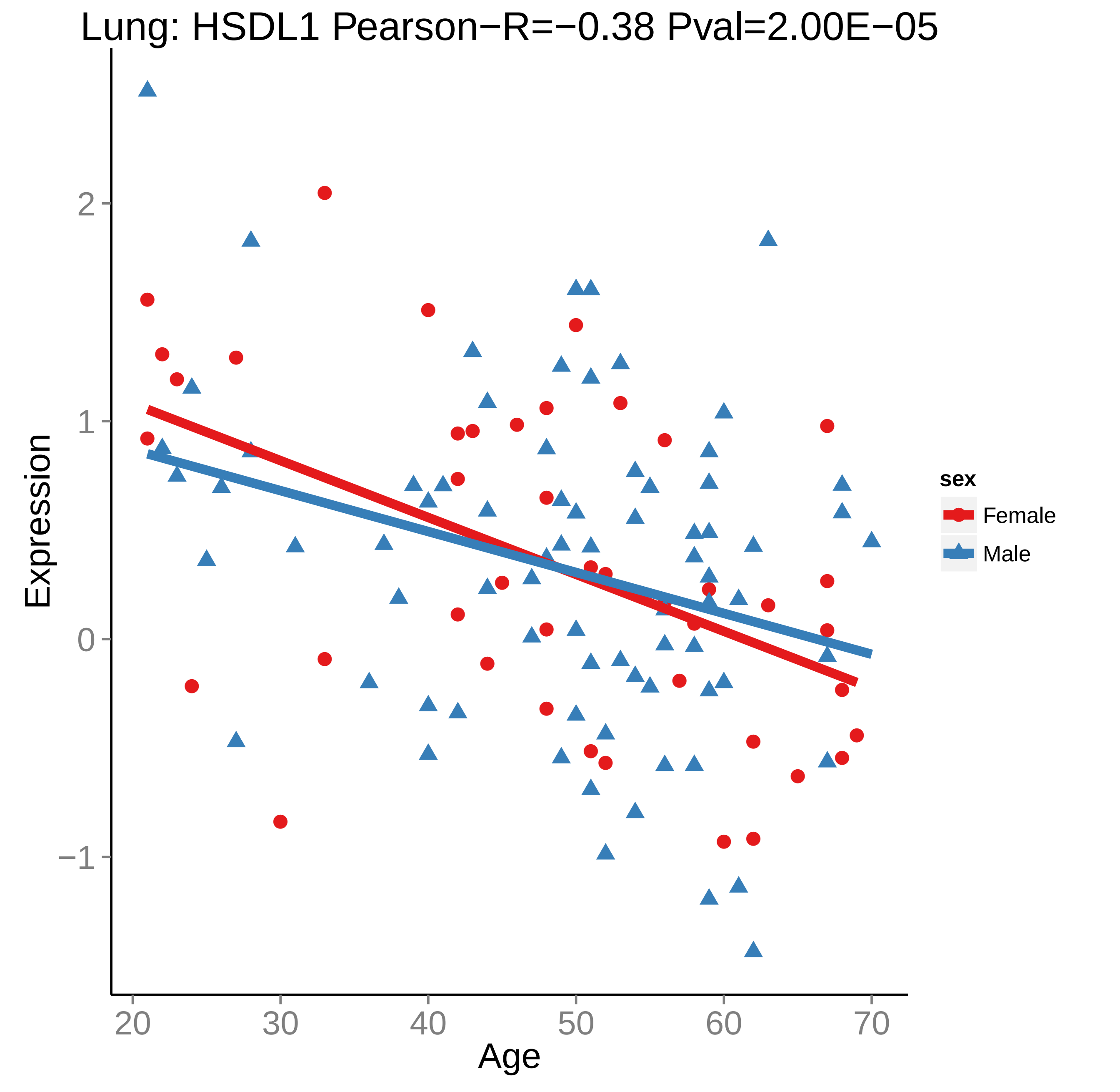

Lung: HSDL1 Pearson−R=−0.38 Pval=2.00E−05
Expression
Age
2
1
0
−1
20
30
40
50
60
70
sex
Female
Male

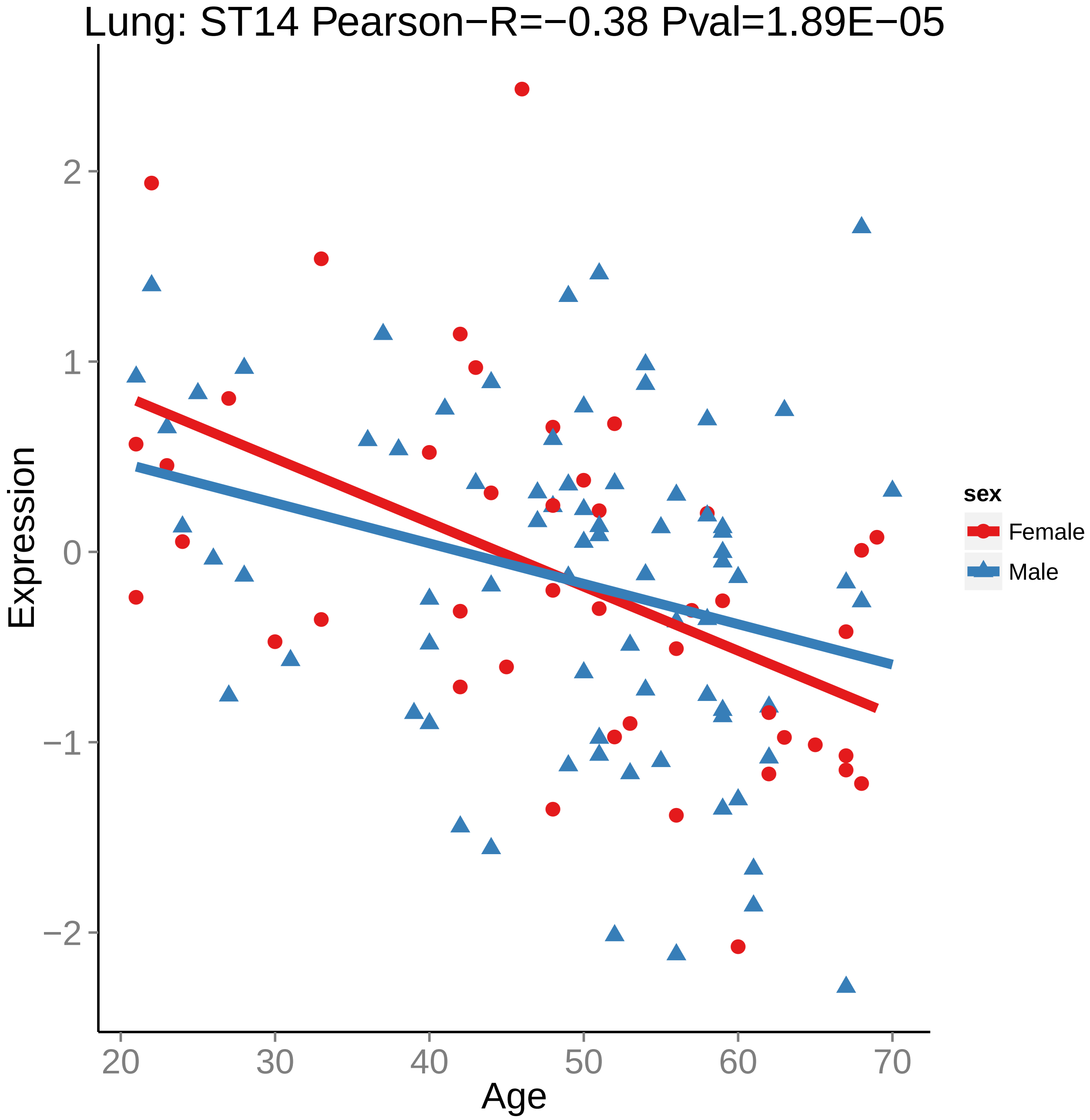

Lung: ST14 Pearson−R=−0.38 Pval=1.89E−05
Expression
2
1
0
−1
−2
20
30
40
50
60
70
Age
sex
Female
Male

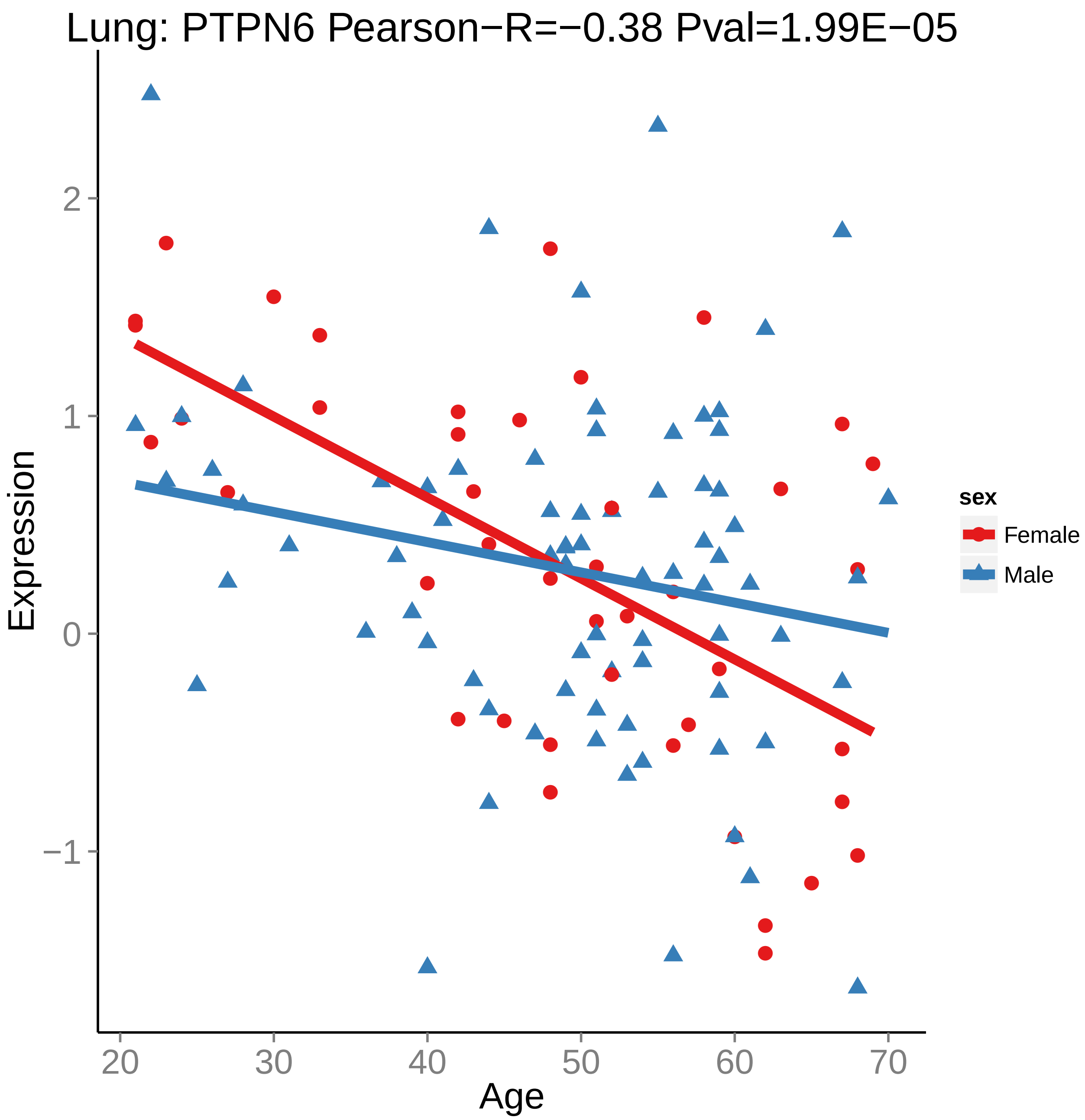
Lung: PTPN6 Pearson−R=−0.38 Pval=1.99E−05
Expression
2
1
0
−1
20
30
40
50
60
70
Age
sex
Female
Male

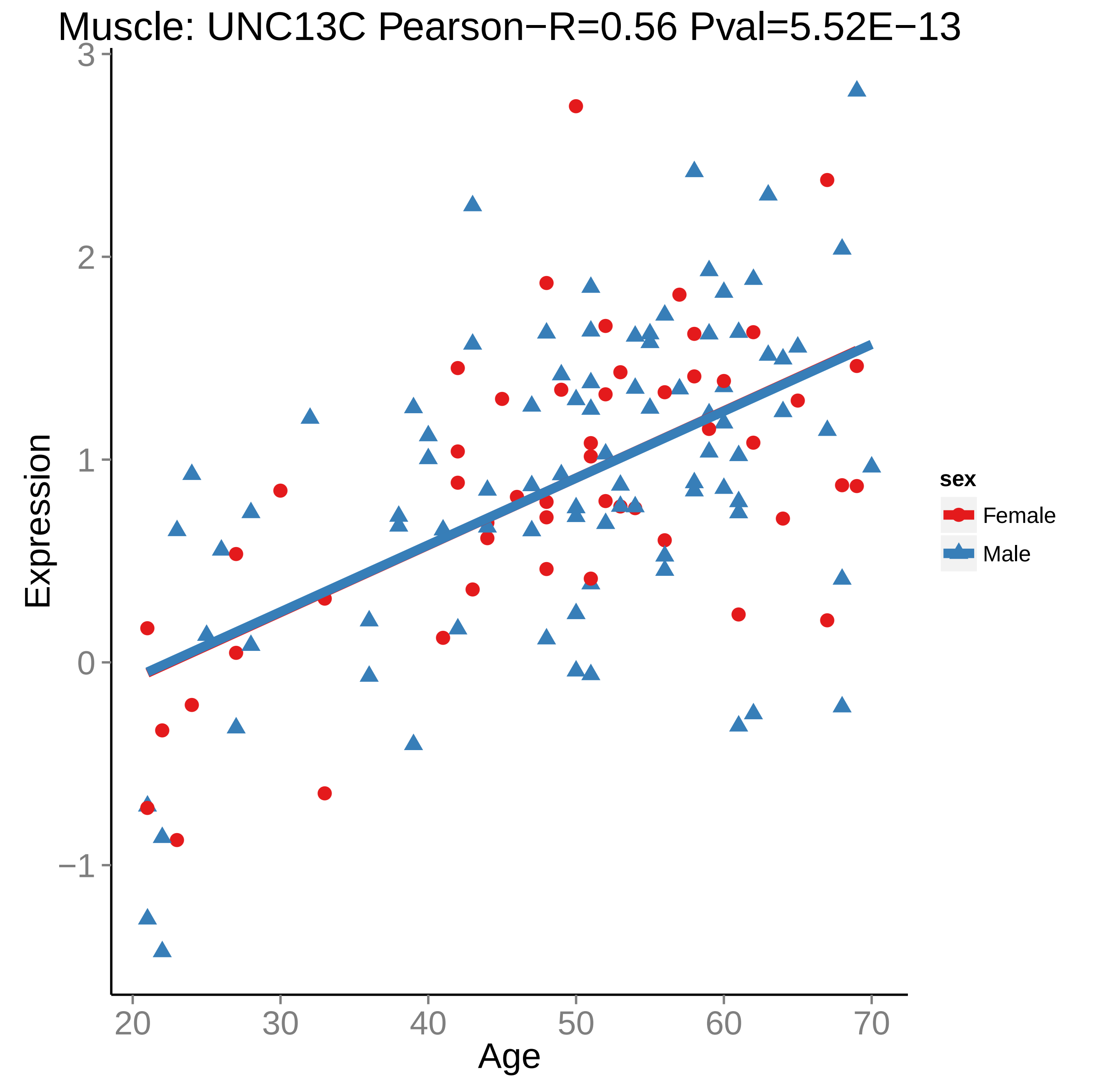

Muscle: UNC13C Pearson−R=0.56 Pval=5.52E−13
Expression
3
2
1
0
−1
Age
20
30
40
50
60
70
sex
Female
Male

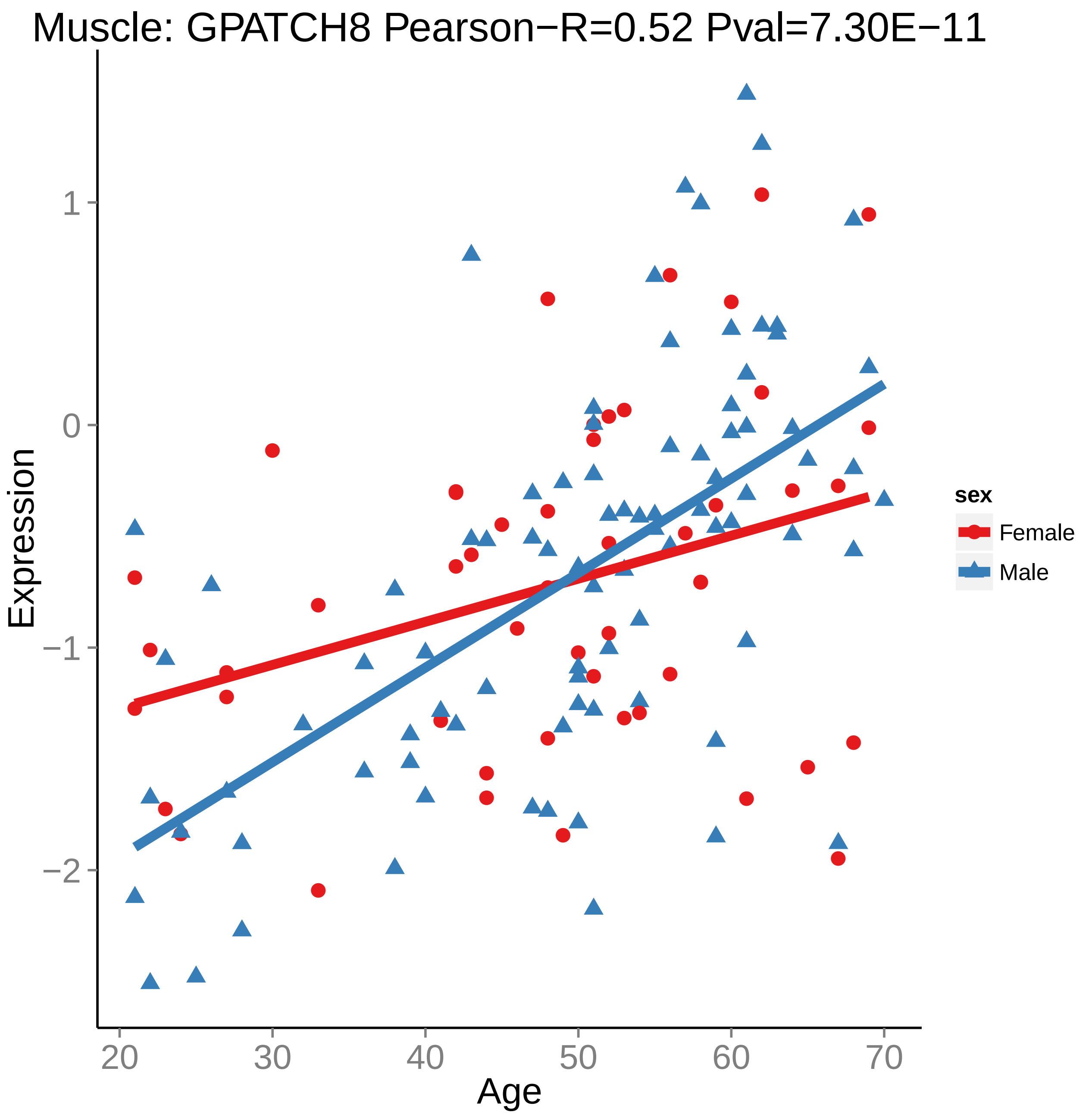

Muscle: GPATCH8 Pearson−R=0.52 Pval=7.30E−11
Expression
1
0
−1
−2
20
30
40
50
60
70
Age
sex
Female
Male

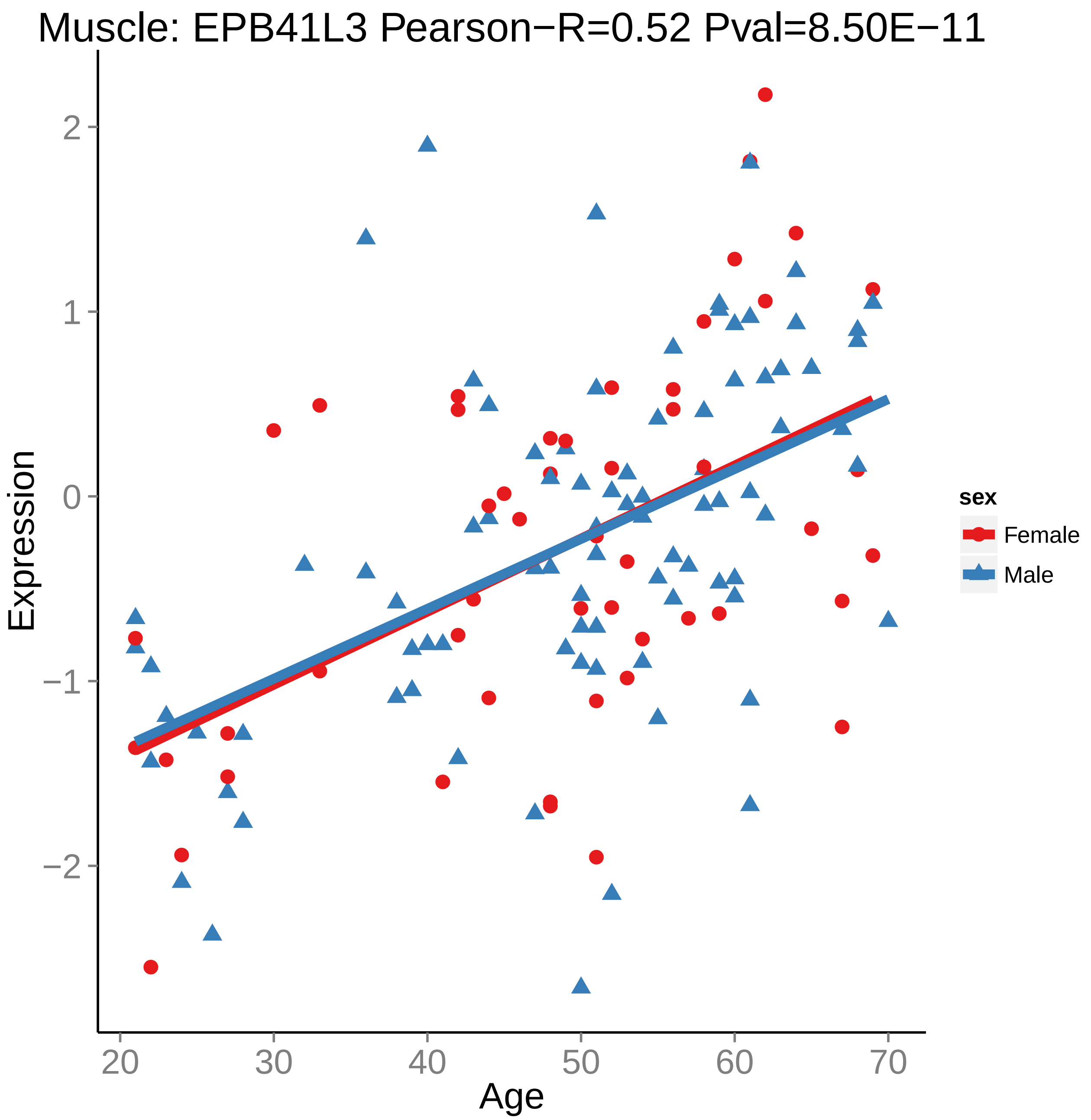
Muscle: EPB41L3 Pearson−R=0.52 Pval=8.50E−11
Expression
Age
2
1
0
−1
−2
20
30
40
50
60
70
sex
Female
Male

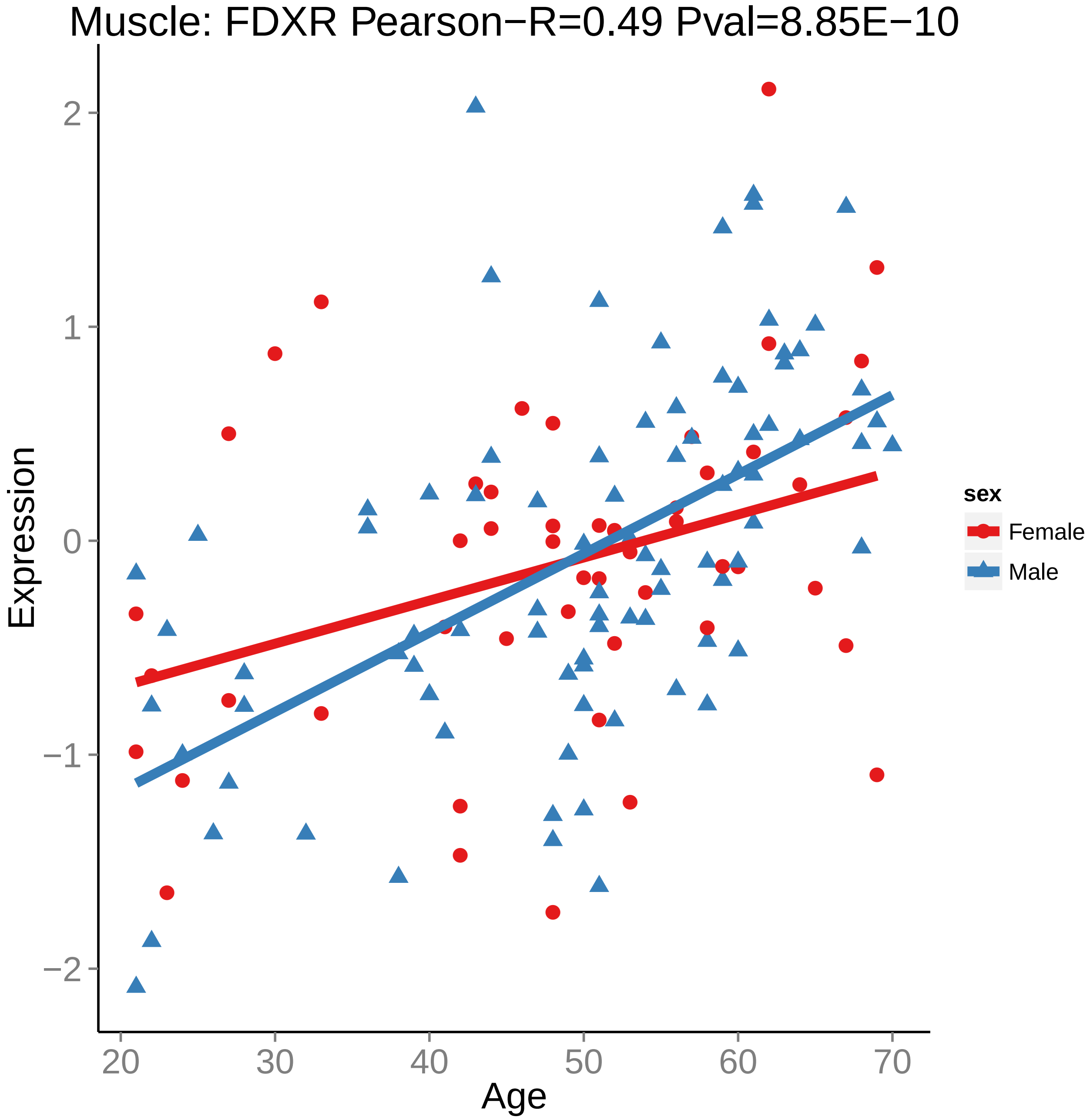

Muscle: FDXR Pearson−R=0.49 Pval=8.85E−10
Expression
2
1
0
−1
−2
20
30
40
50
60
70
Age
sex
Female
Male

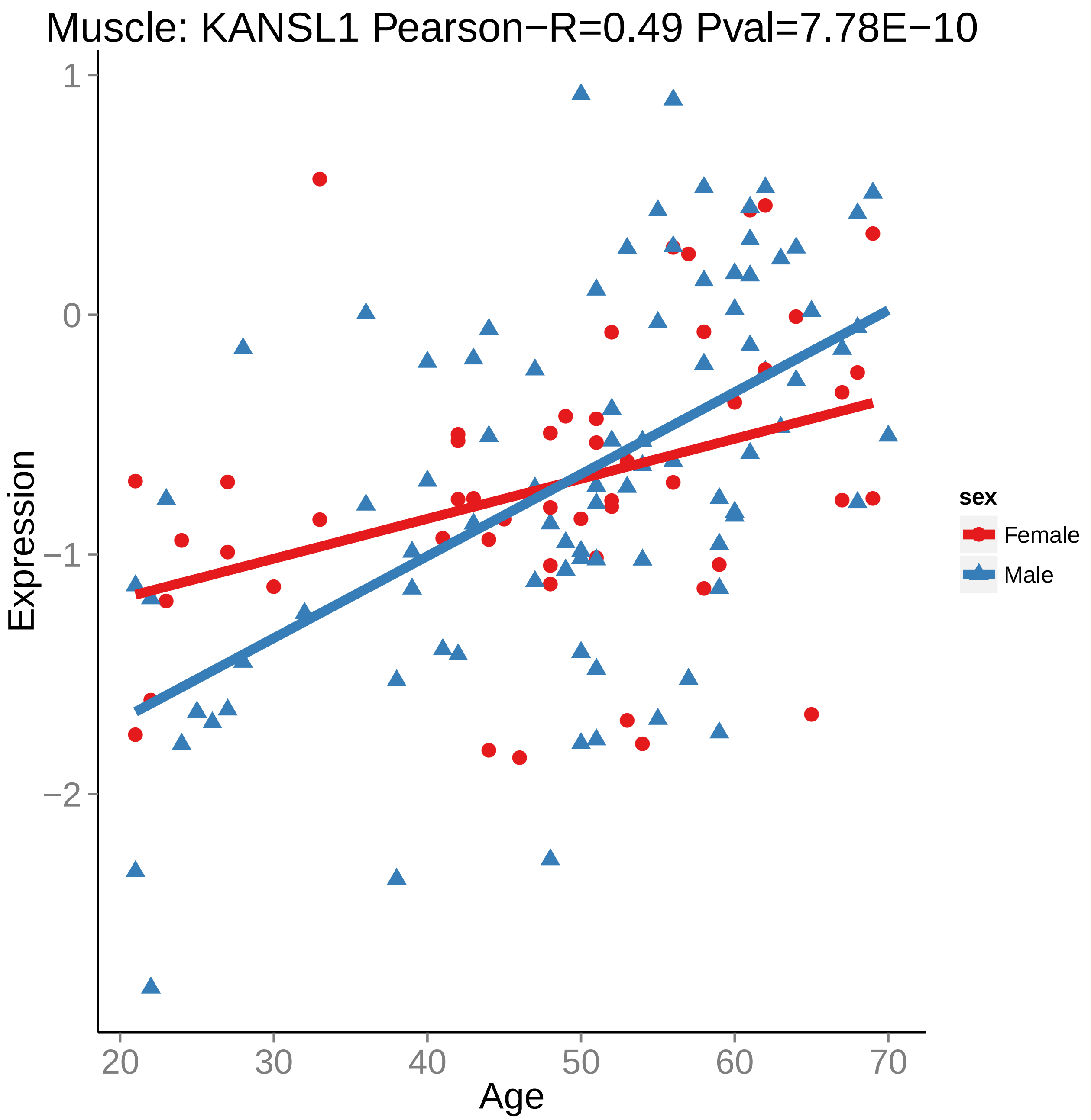

Muscle: KANSL1 Pearson−R=0.49 Pval=7.78E−10
1
0
−1
−2
Expression
20
30
40
50
60
70
Age
sex
Female
Male

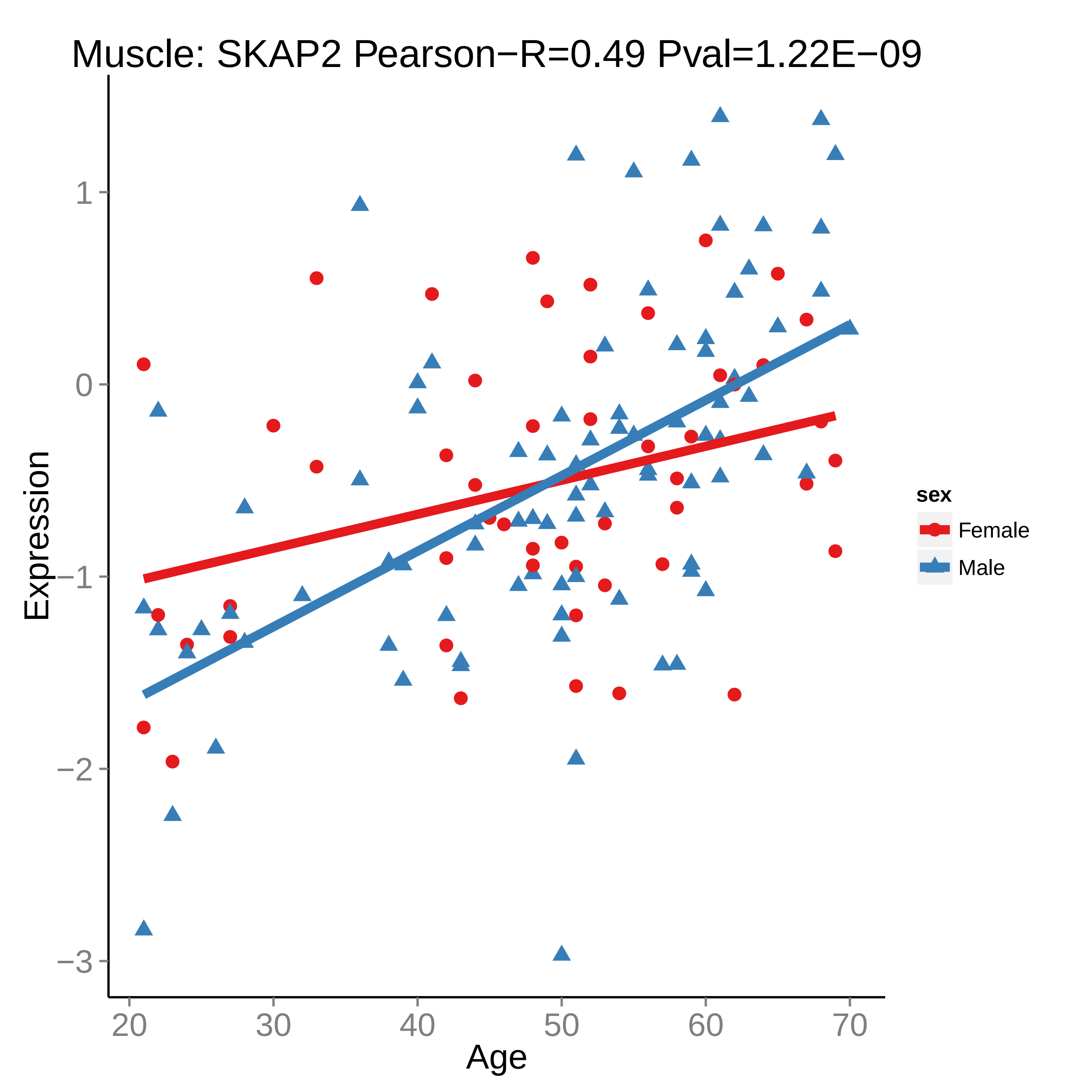

Muscle: SKAP2 Pearson−R=0.49 Pval=1.22E−09
Expression
Age
1
0
−1
−2
−3
20
30
40
50
60
70
sex
Female
Male

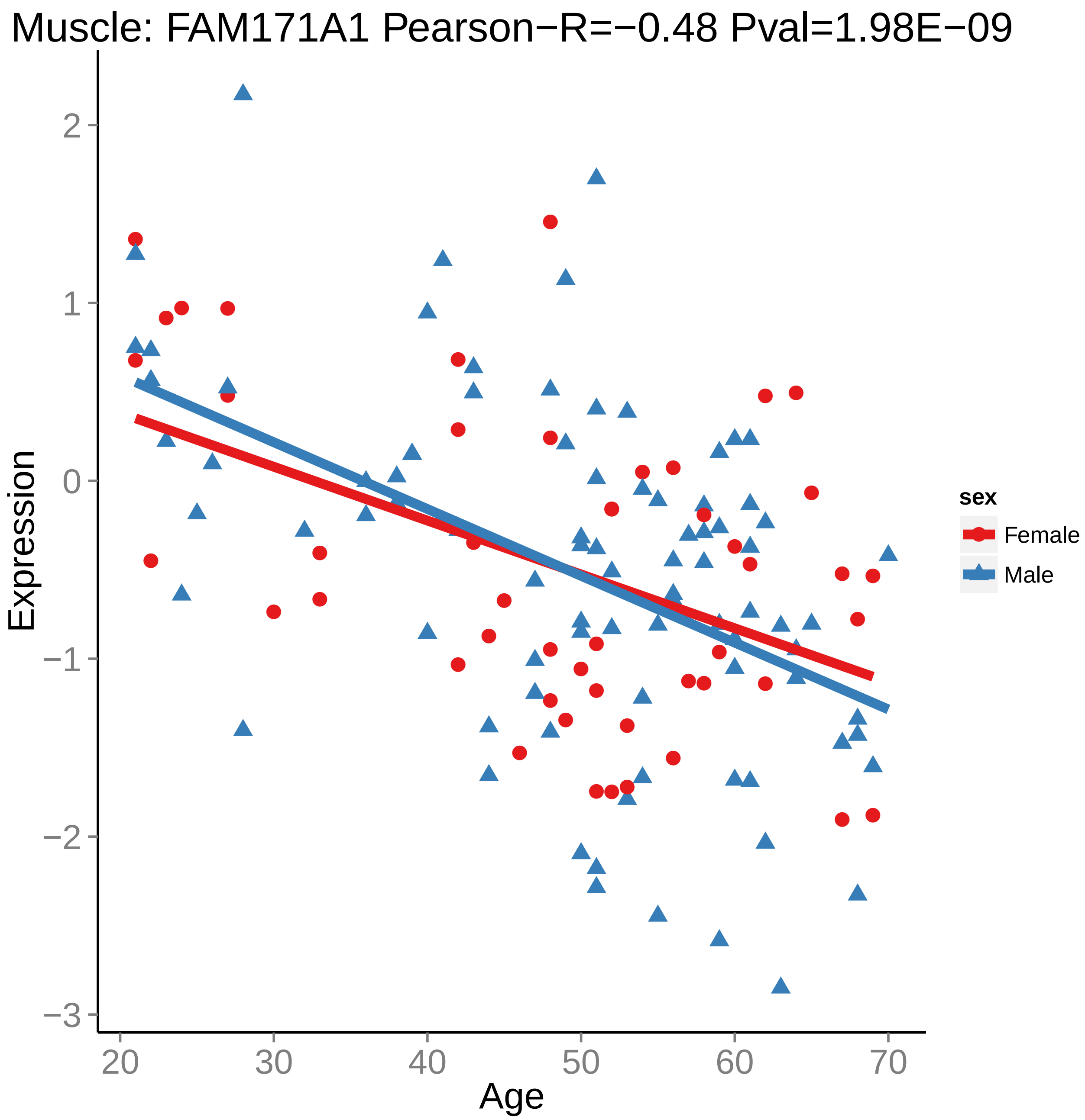

Muscle: FAM171A1 Pearson−R=−0.48 Pval=1.98E−09
Expression
Age
2
1
0
−1
−2
−3
20
30
40
50
60
70
sex
Female
Male

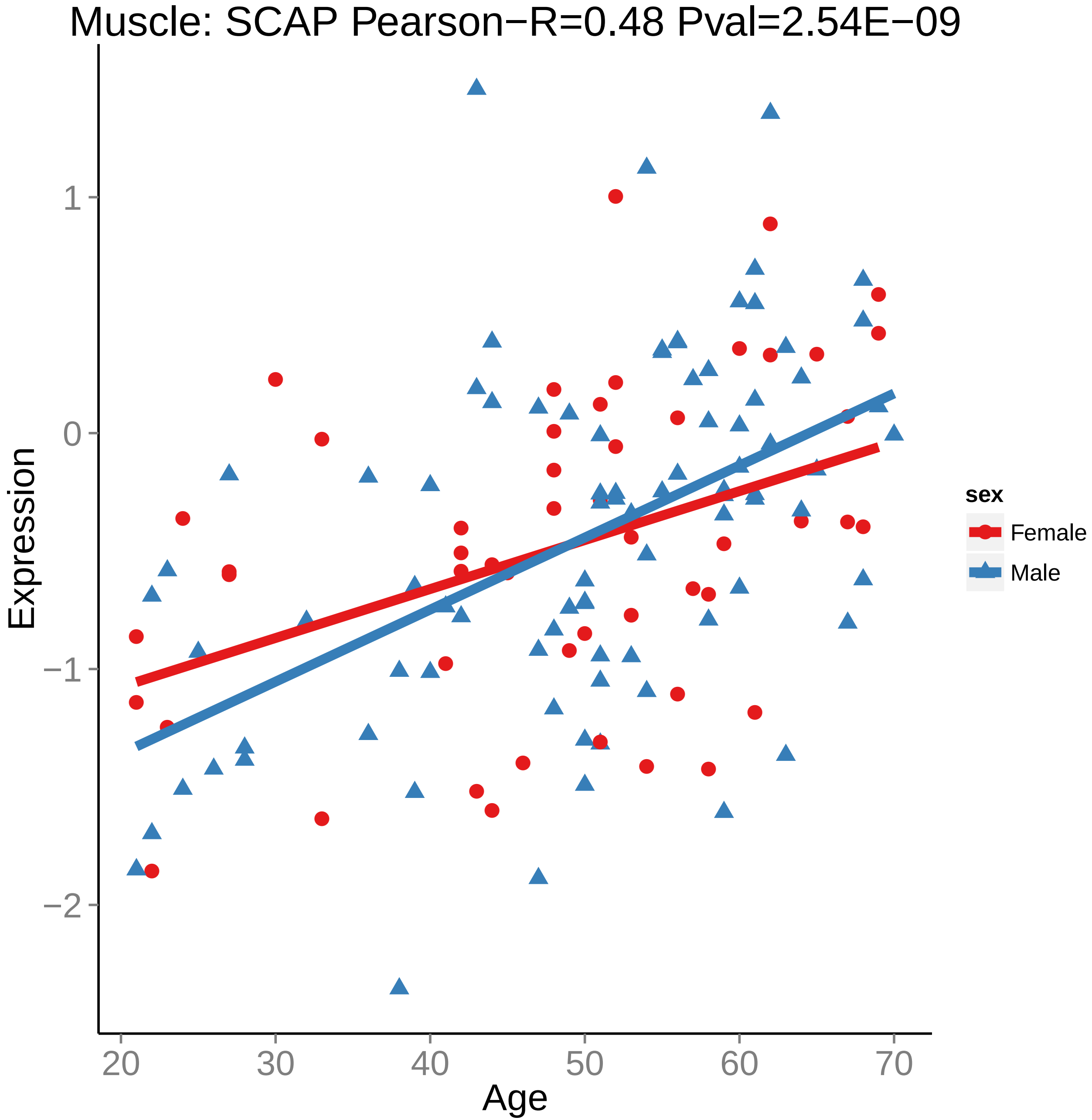

Muscle: SCAP Pearson−R=0.48 Pval=2.54E−09
Expression
Age
sex
Female
Male
1
0
−1
−2
20
30
40
50
60
70

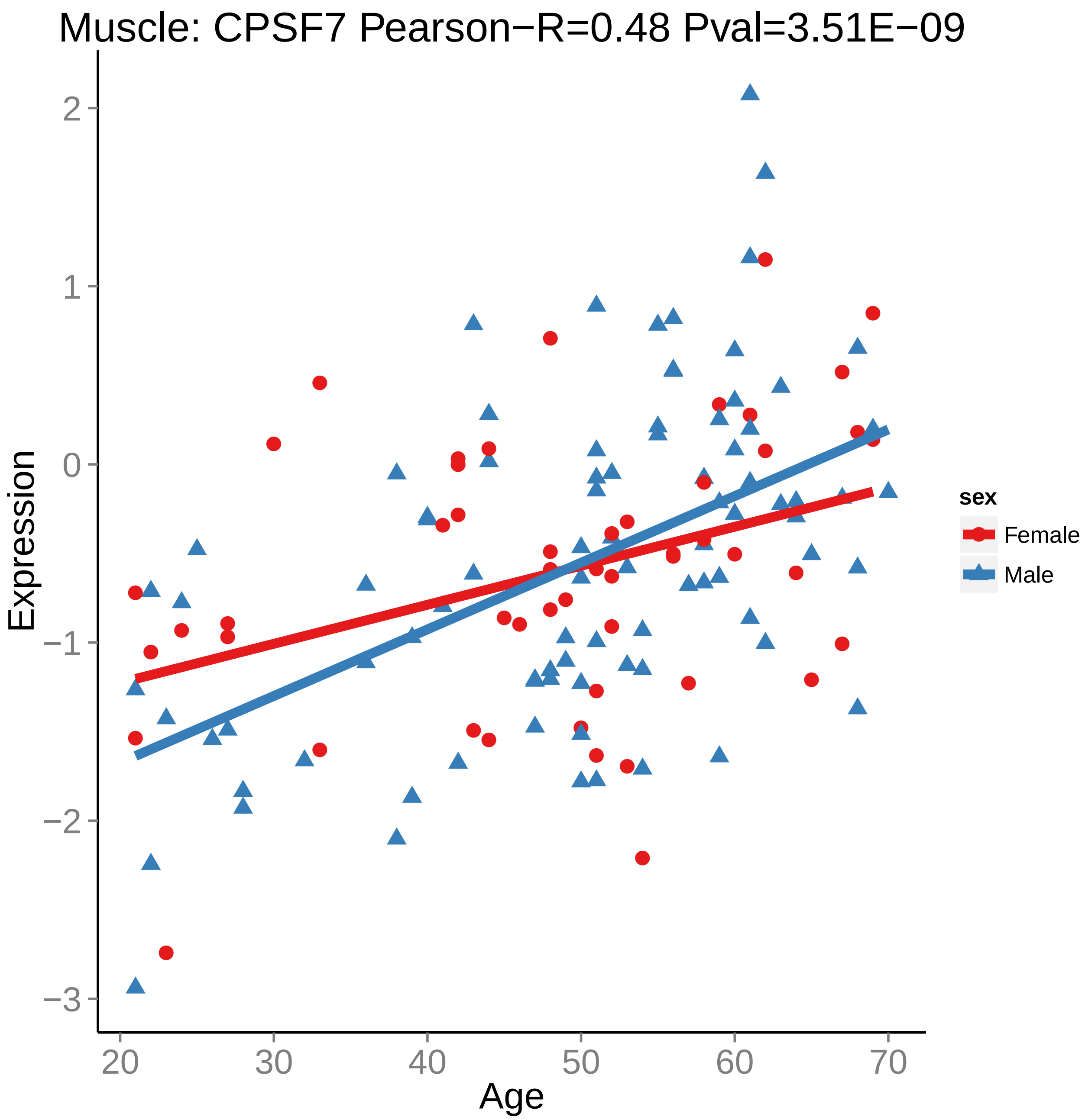

Muscle: CPSF7 Pearson−R=0.48 Pval=3.51E−09
Expression
Age
2
1
0
−1
−2
−3
20
30
40
50
60
70
sex
Female
Male

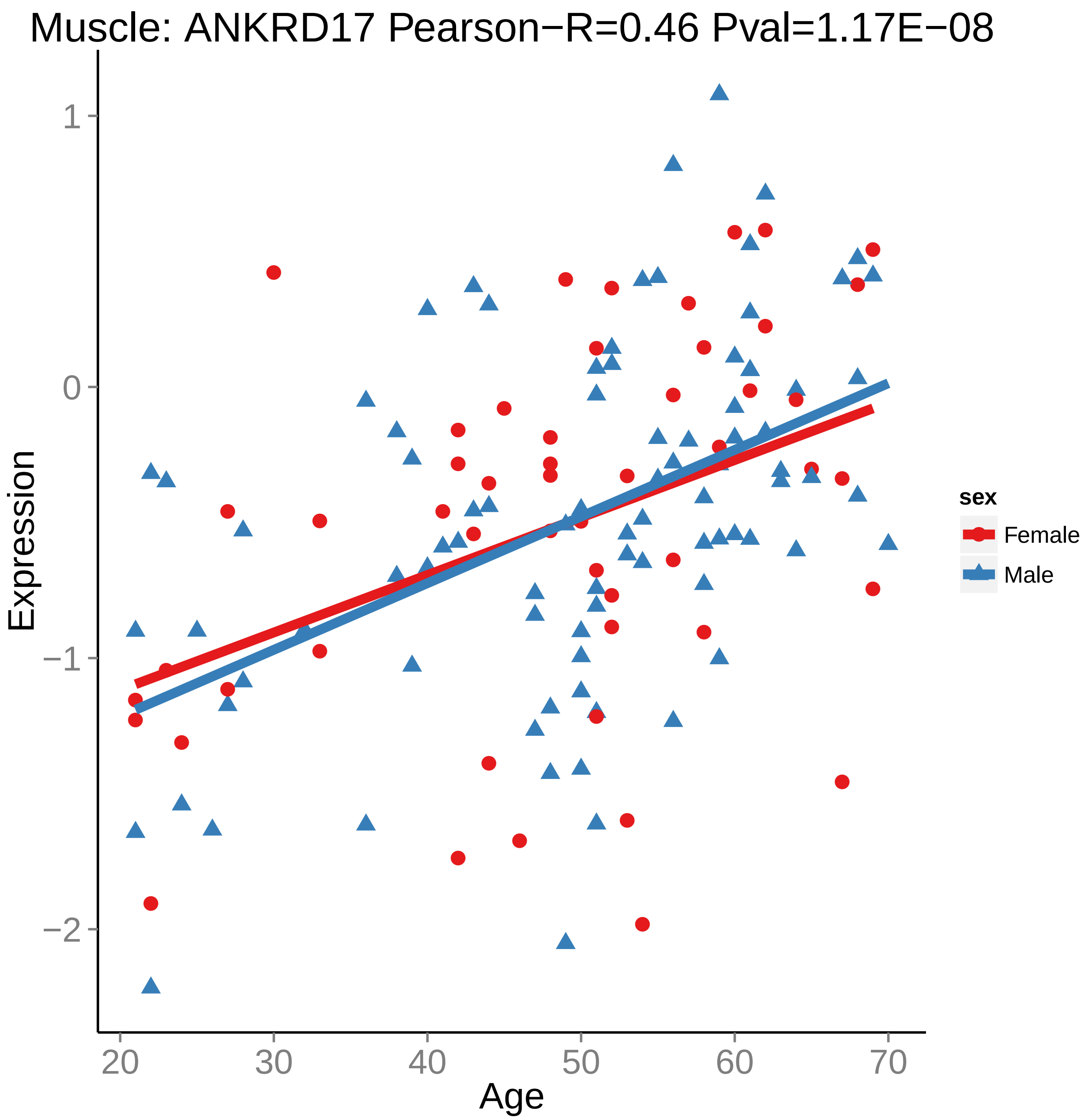
Muscle: ANKRD17 Pearson−R=0.46 Pval=1.17E−08
Expression
Age
1
0
−1
−2
20
30
40
50
60
70
sex
Female
Male

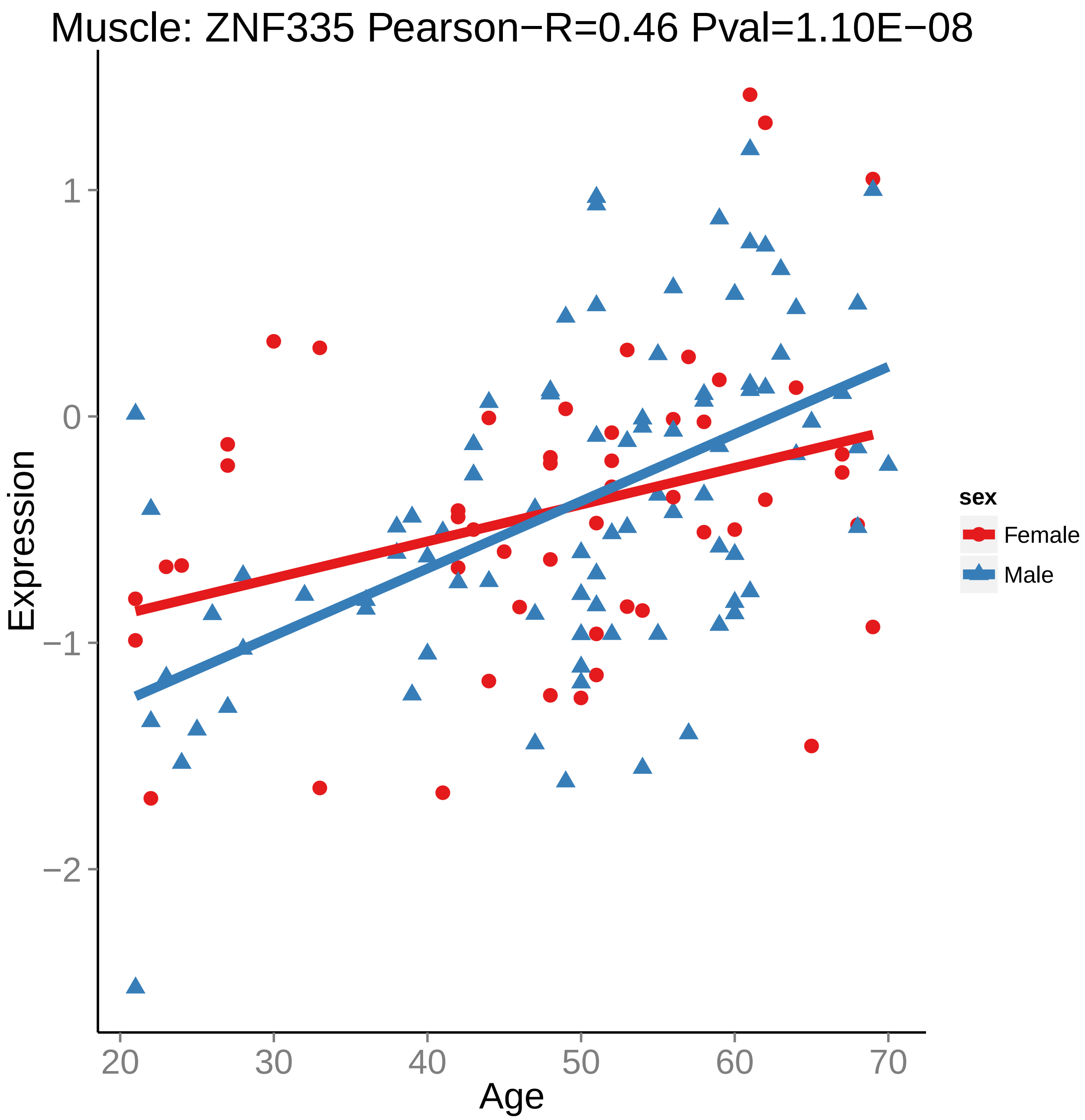

Muscle: ZNF335 Pearson−R=0.46 Pval=1.10E−08
Expression
1
0
−1
−2
20
30
40
50
60
70
Age
sex
Female
Male

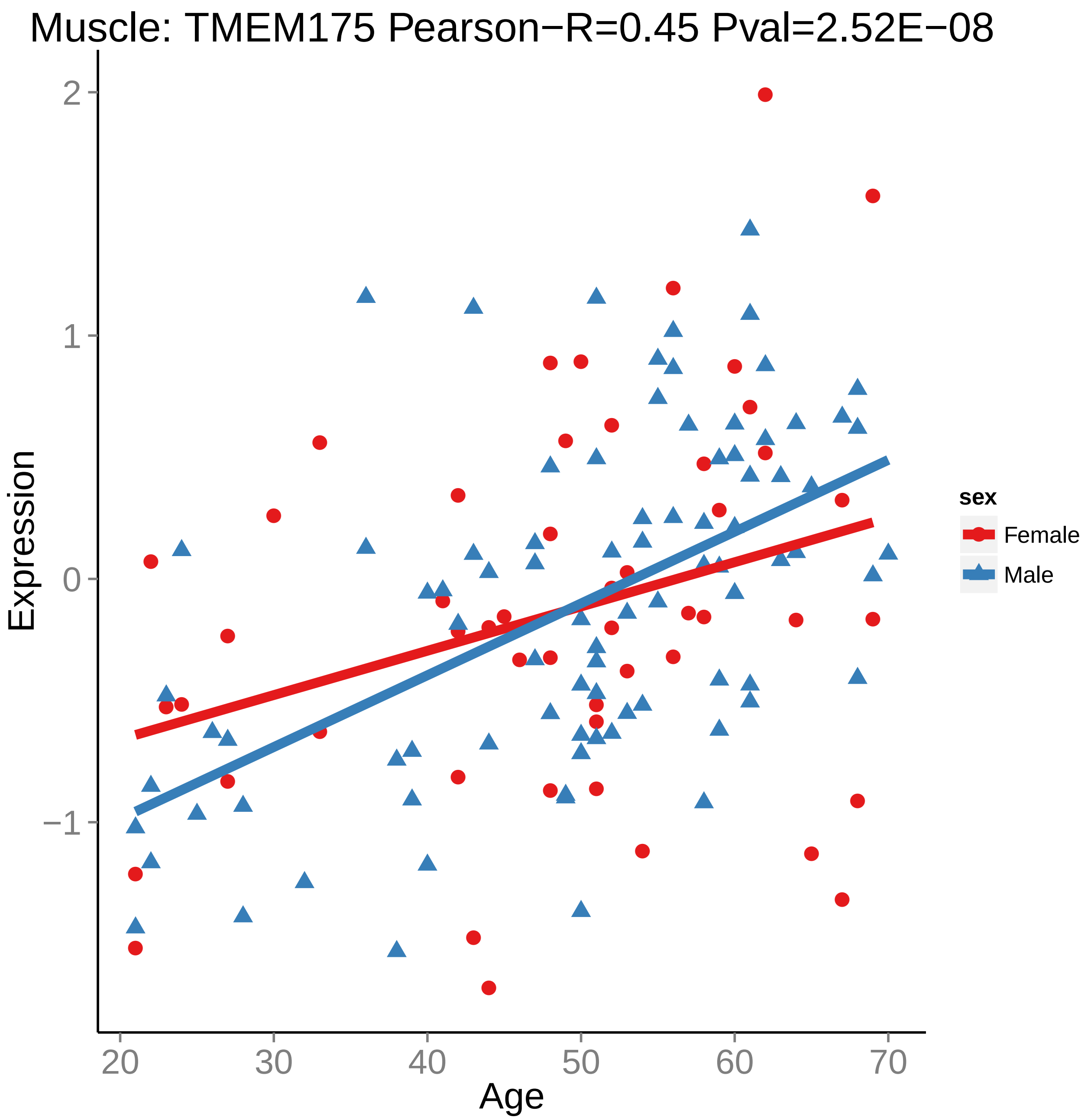
Muscle: TMEM175 Pearson−R=0.45 Pval=2.52E−08
Expression
2
1
0
−1
Age
20
30
40
50
60
70
sex
Female
Male

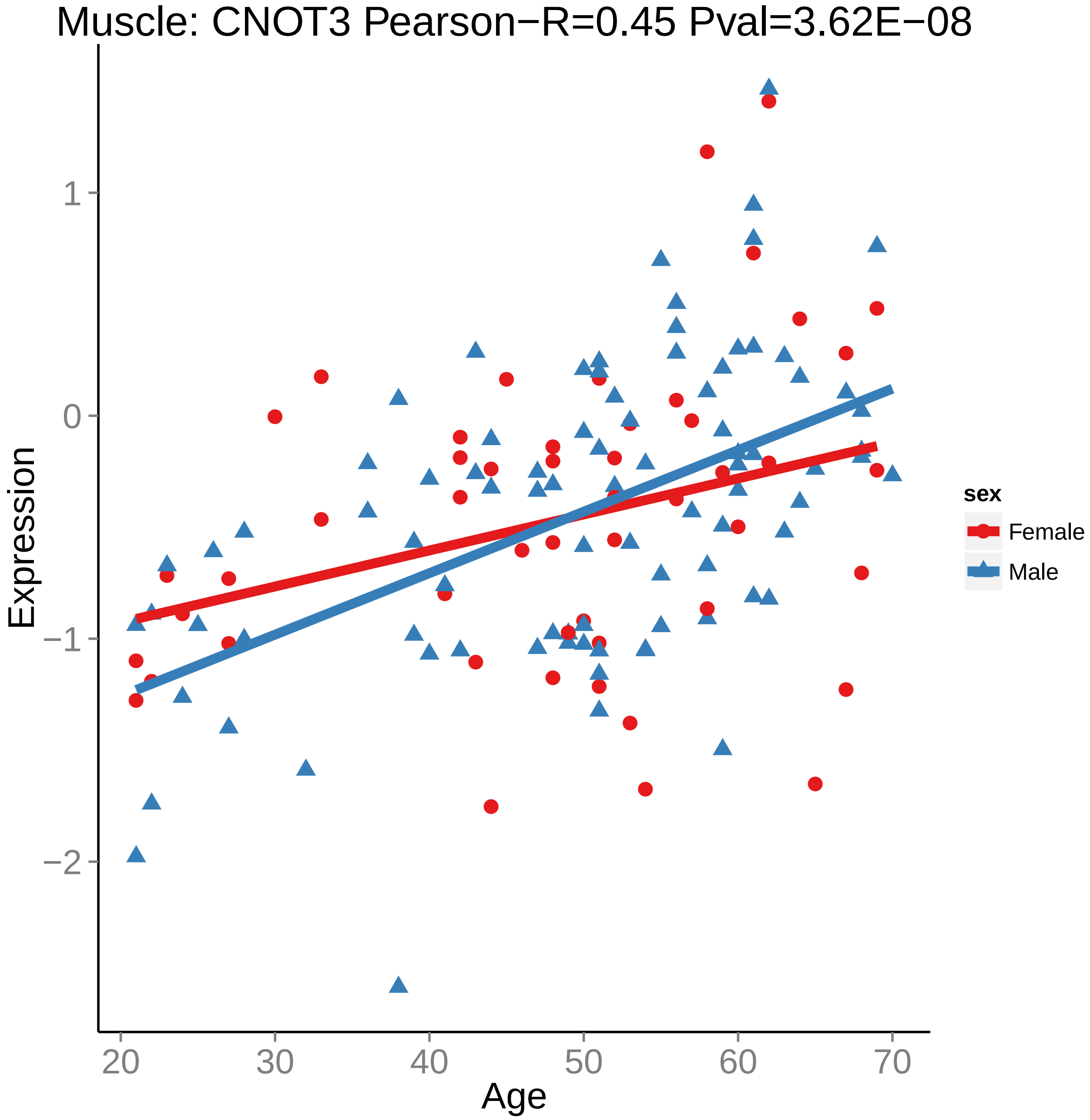

Muscle: CNOT3 Pearson−R=0.45 Pval=3.62E−08
Expression
1
0
−1
−2
20
30
40
50
60
70
Age
sex
Female
Male

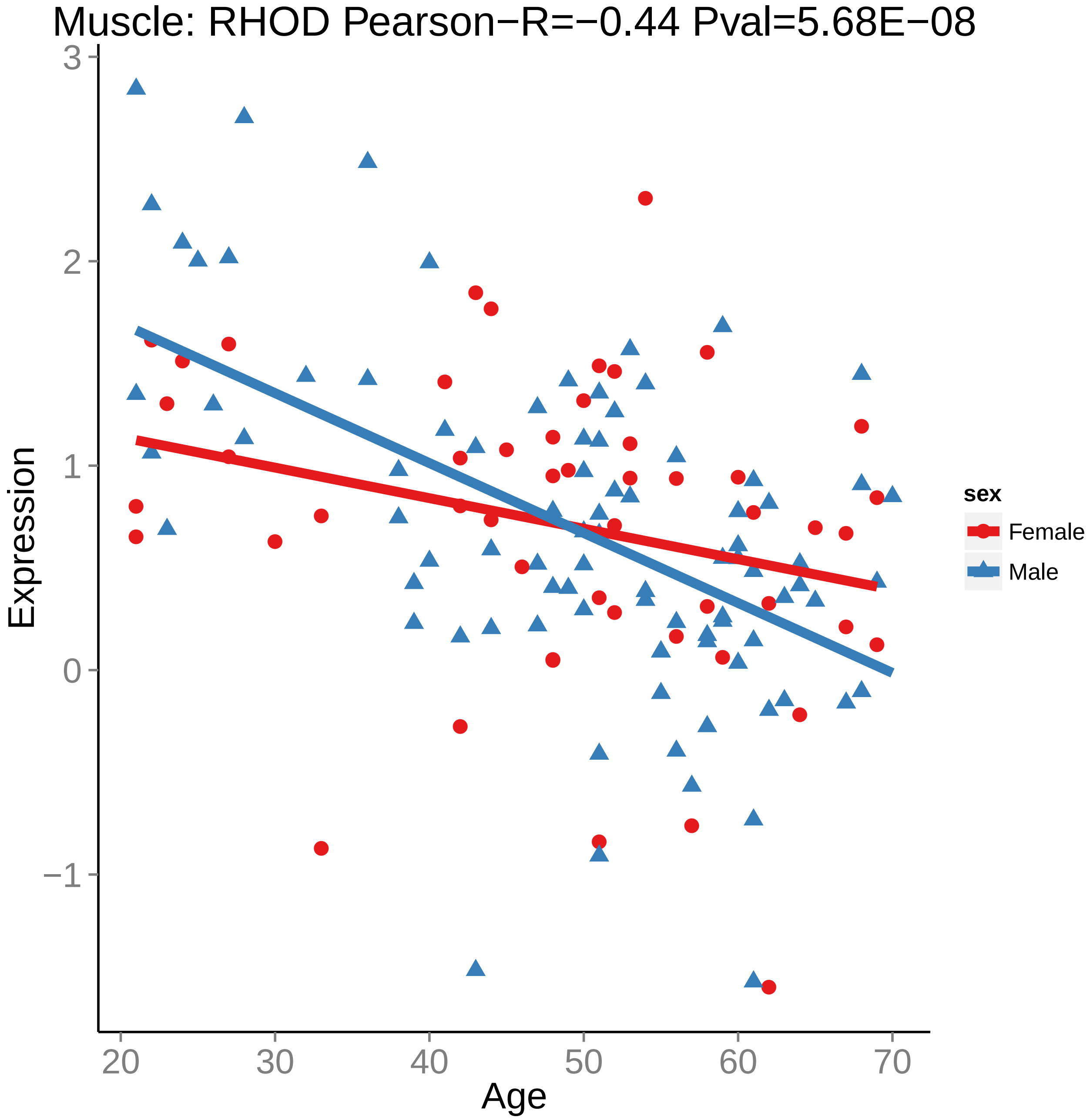

Muscle: RHOD Pearson−R=−0.44 Pval=5.68E−08
Expression
3
2
1
0
−1
Age
20
30
40
50
60
70
sex
Female
Male

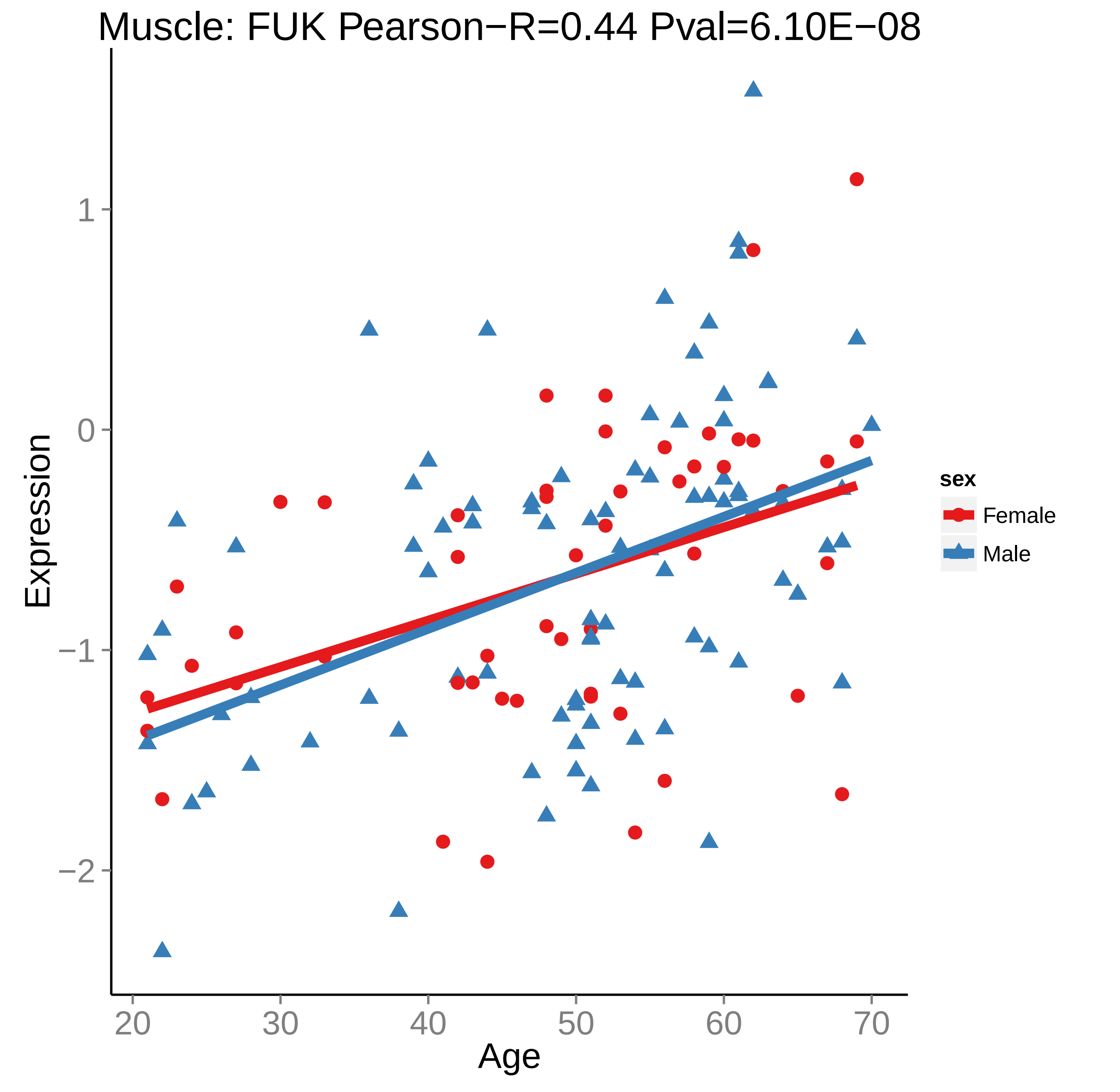
Muscle: FUK Pearson−R=0.44 Pval=6.10E−08
Expression
1
0
−1
−2
20
30
40
50
60
70
Age
sex
Female
Male

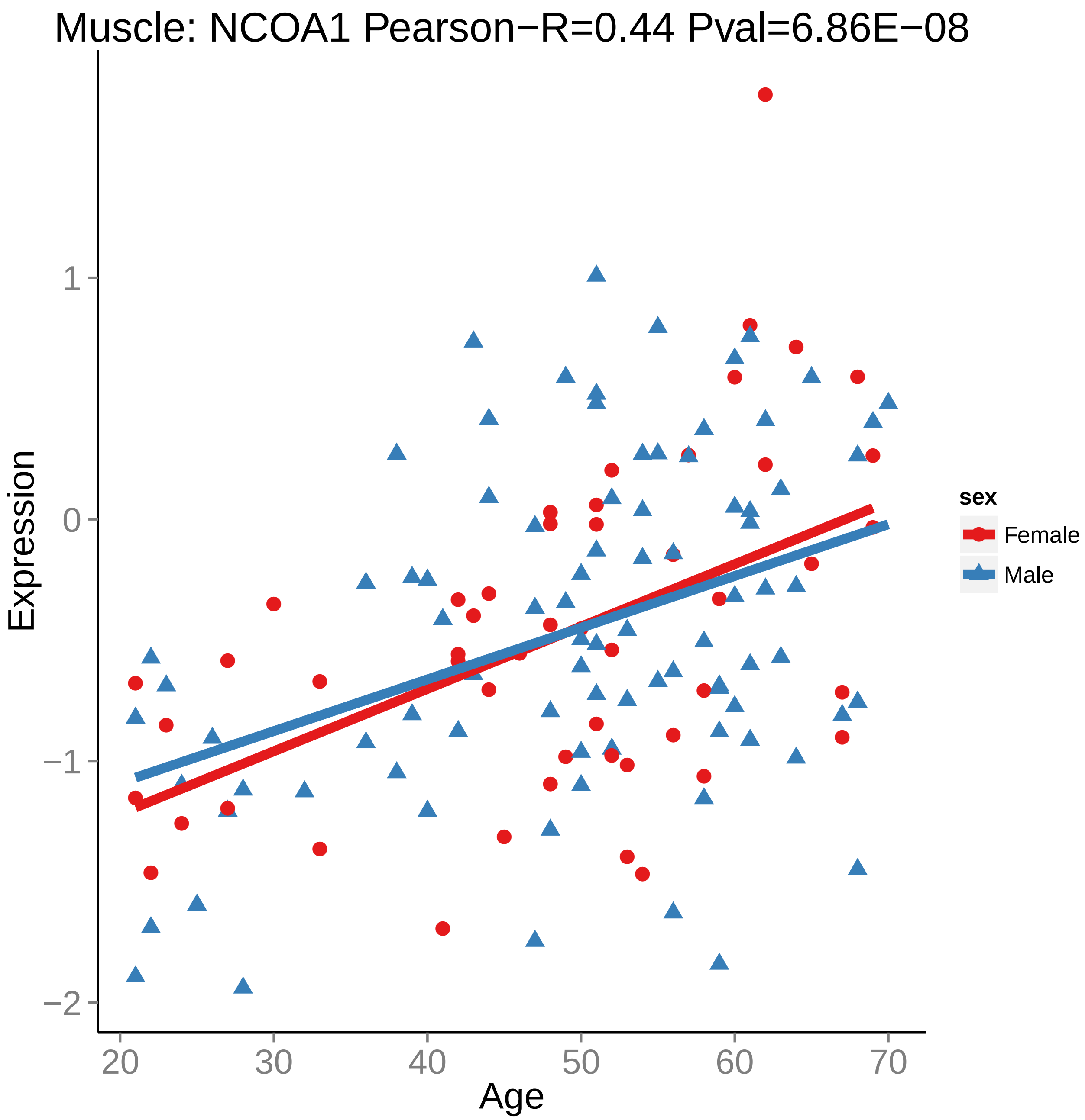

Muscle: NCOA1 Pearson−R=0.44 Pval=6.86E−08
Expression
1
0
−1
−2
20
30
40
50
60
70
Age
sex
Female
Male

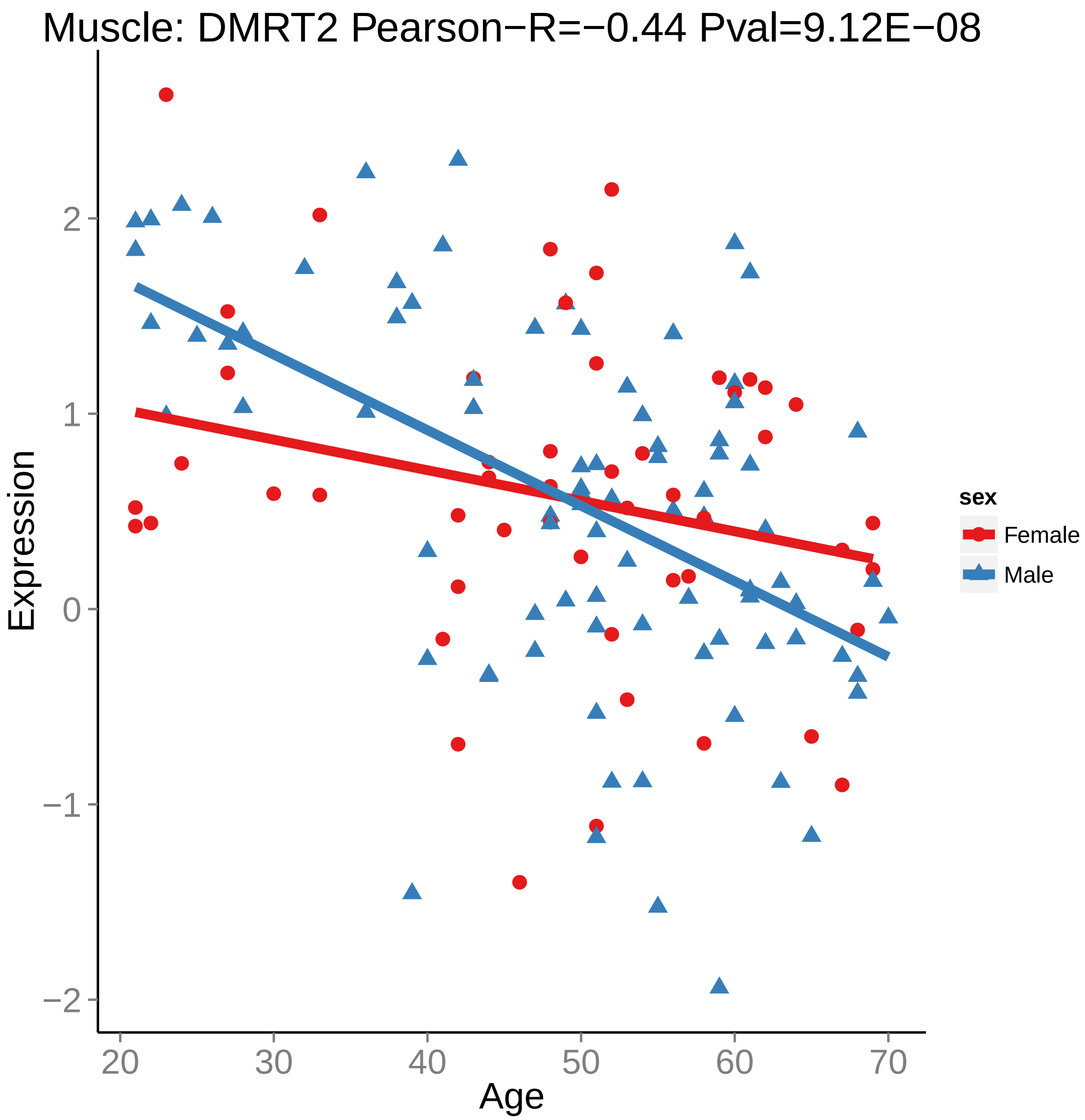

Muscle: DMRT2 Pearson−R=−0.44 Pval=9.12E−08
Expression
Age
sex
Female
Male
2
1
0
−1
−2
20
30
40
50
60
70

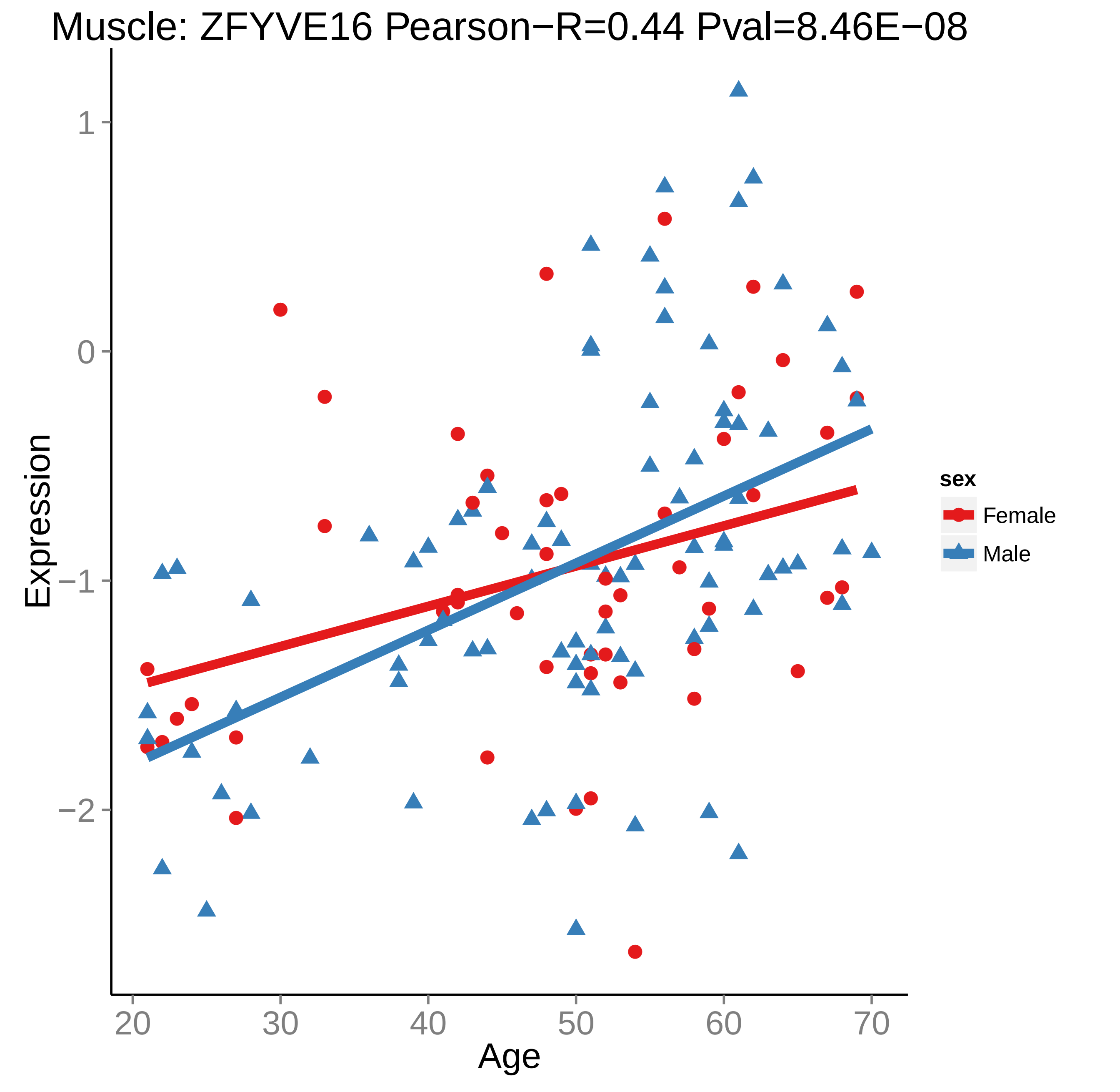

Muscle: ZFYVE16 Pearson−R=0.44 Pval=8.46E−08
Expression
1
0
−1
−2
20
30
40
50
60
70
Age
sex
Female
Male

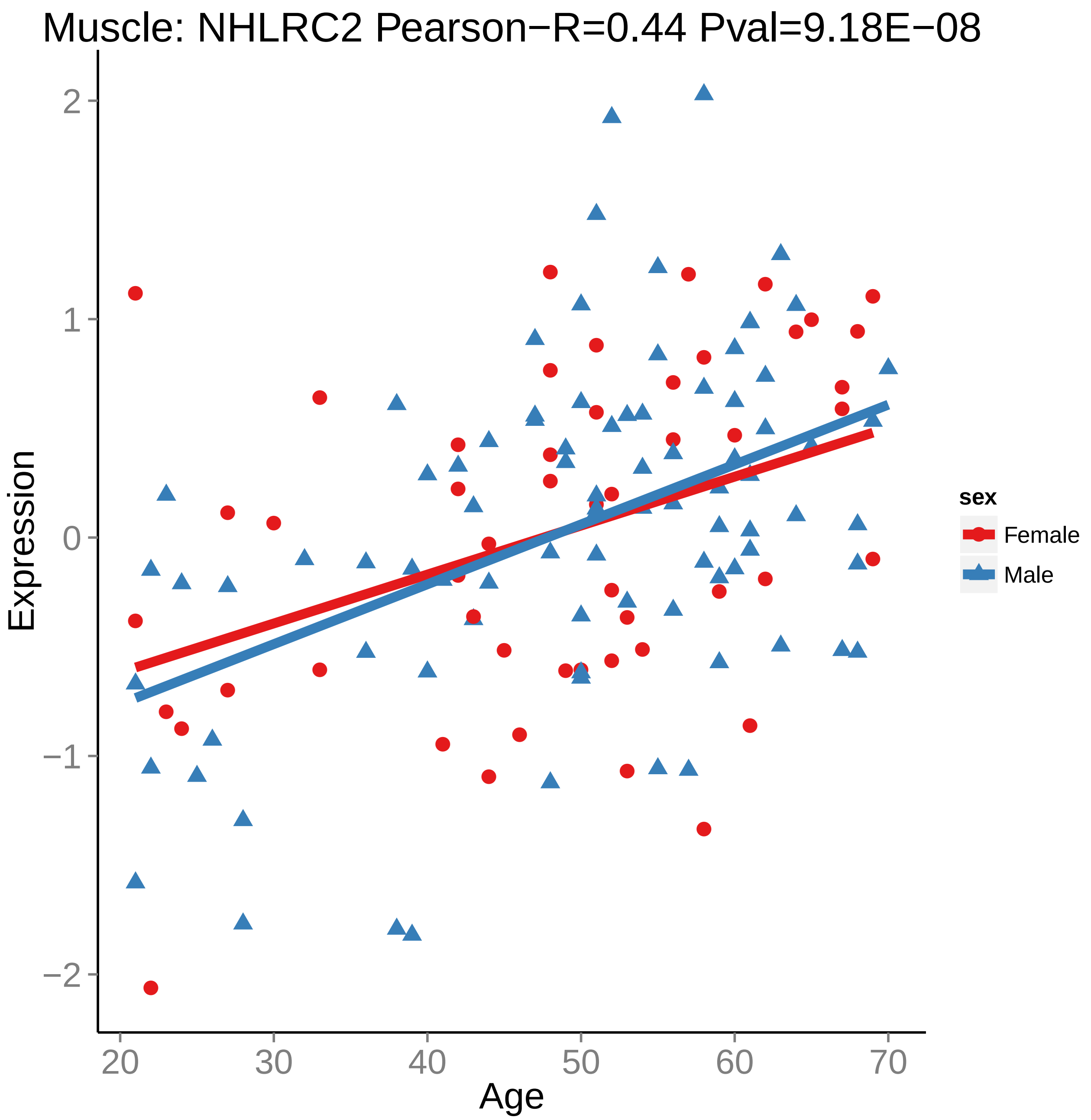
Muscle: NHLRC2 Pearson−R=0.44 Pval=9.18E−08
Expression
Age
2
1
0
−1
−2
20
30
40
50
60
70
sex
Female
Male

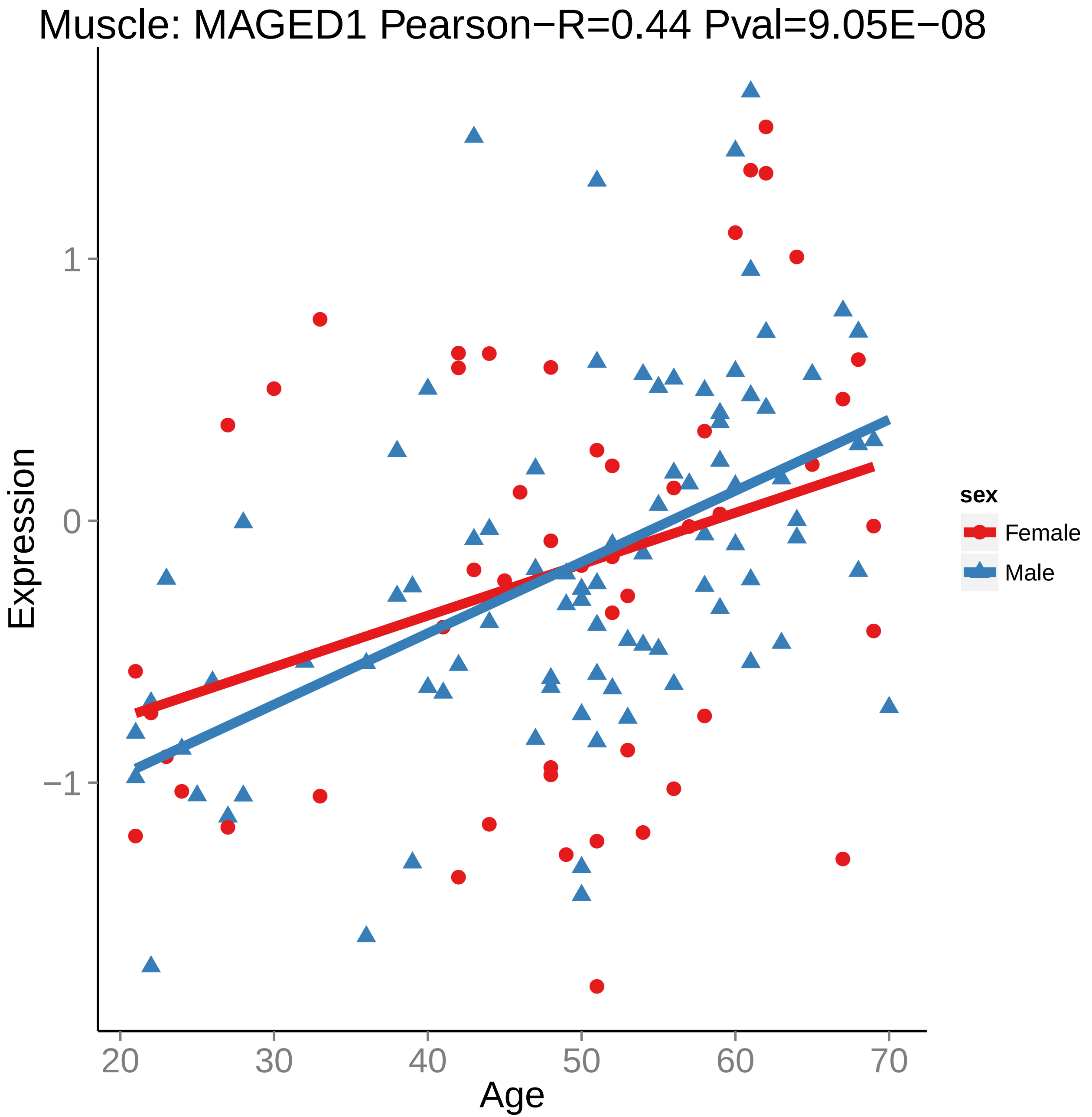

Muscle: MAGED1 Pearson−R=0.44 Pval=9.05E−08
Expression
1
0
−1
20
30
40
50
60
70
Age
sex
Female
Male

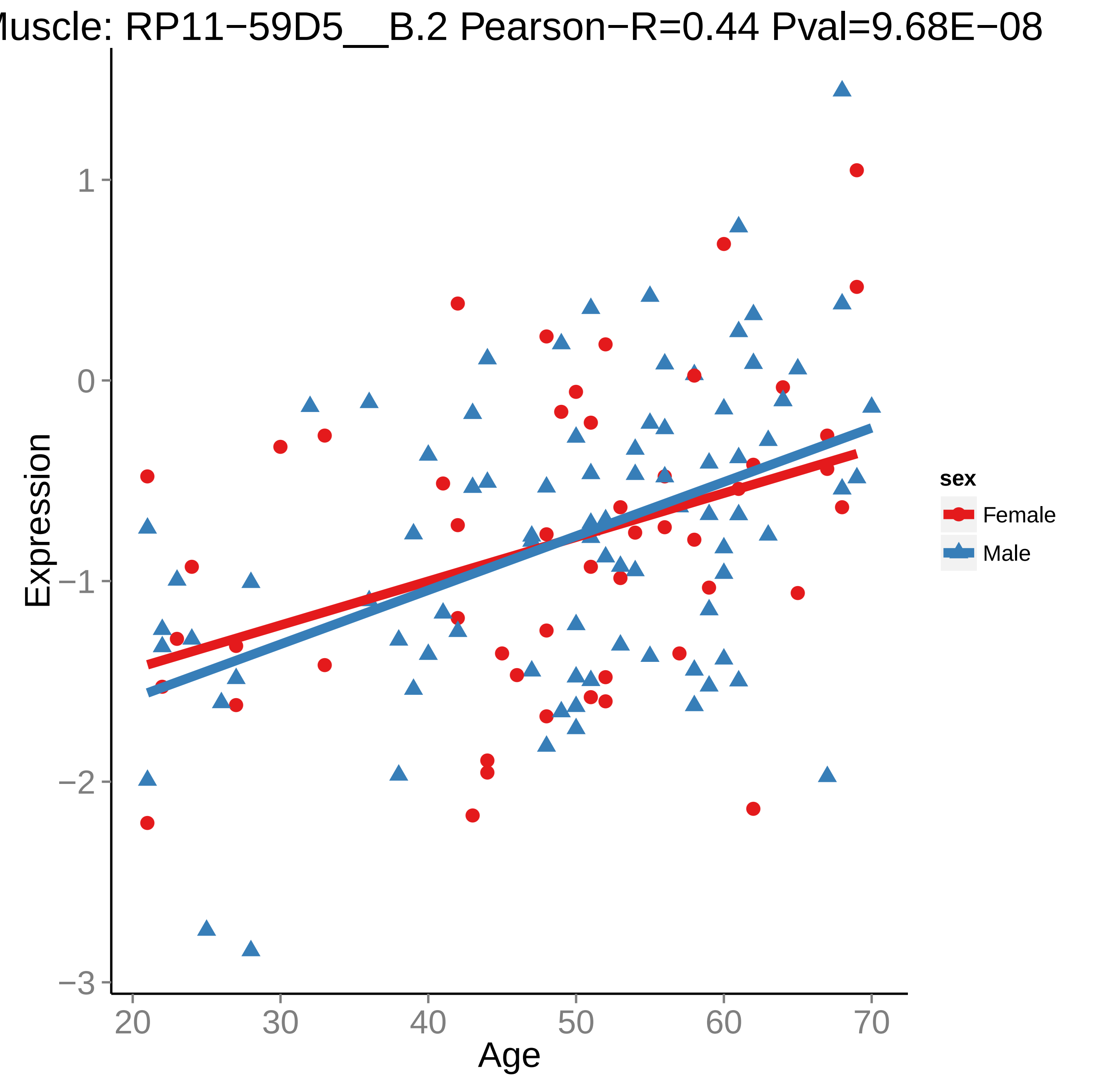

Muscle: RP11−59D5__B.2 Pearson−R=0.44 Pval=9.68E−08
Expression
1
0
−1
−2
−3
20
30
40
50
60
70
Age
sex
Female
Male

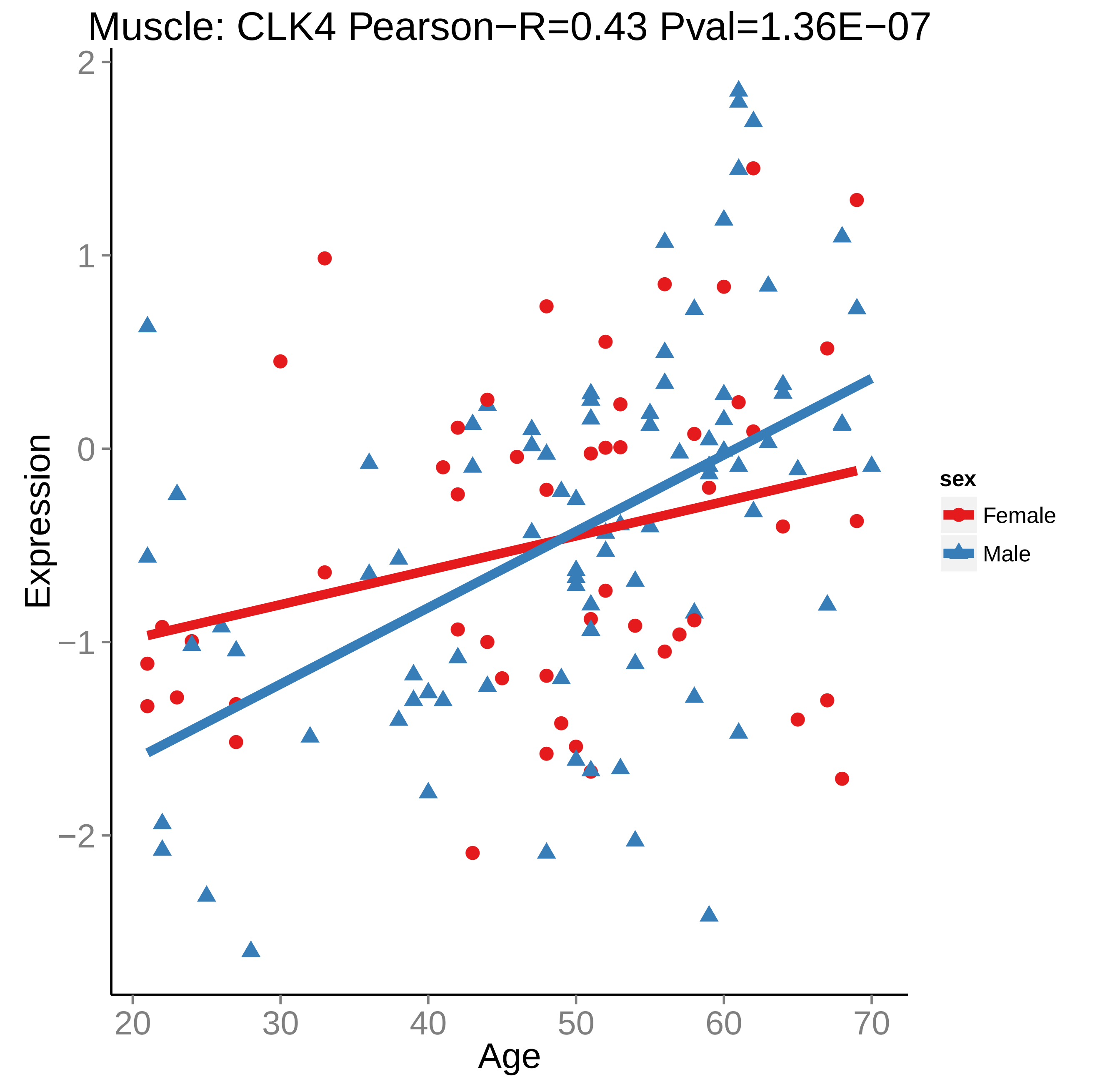

Muscle: CLK4 Pearson−R=0.43 Pval=1.36E−07
Expression
Age
2
1
0
−1
−2
20
30
40
50
60
70
sex
Female
Male

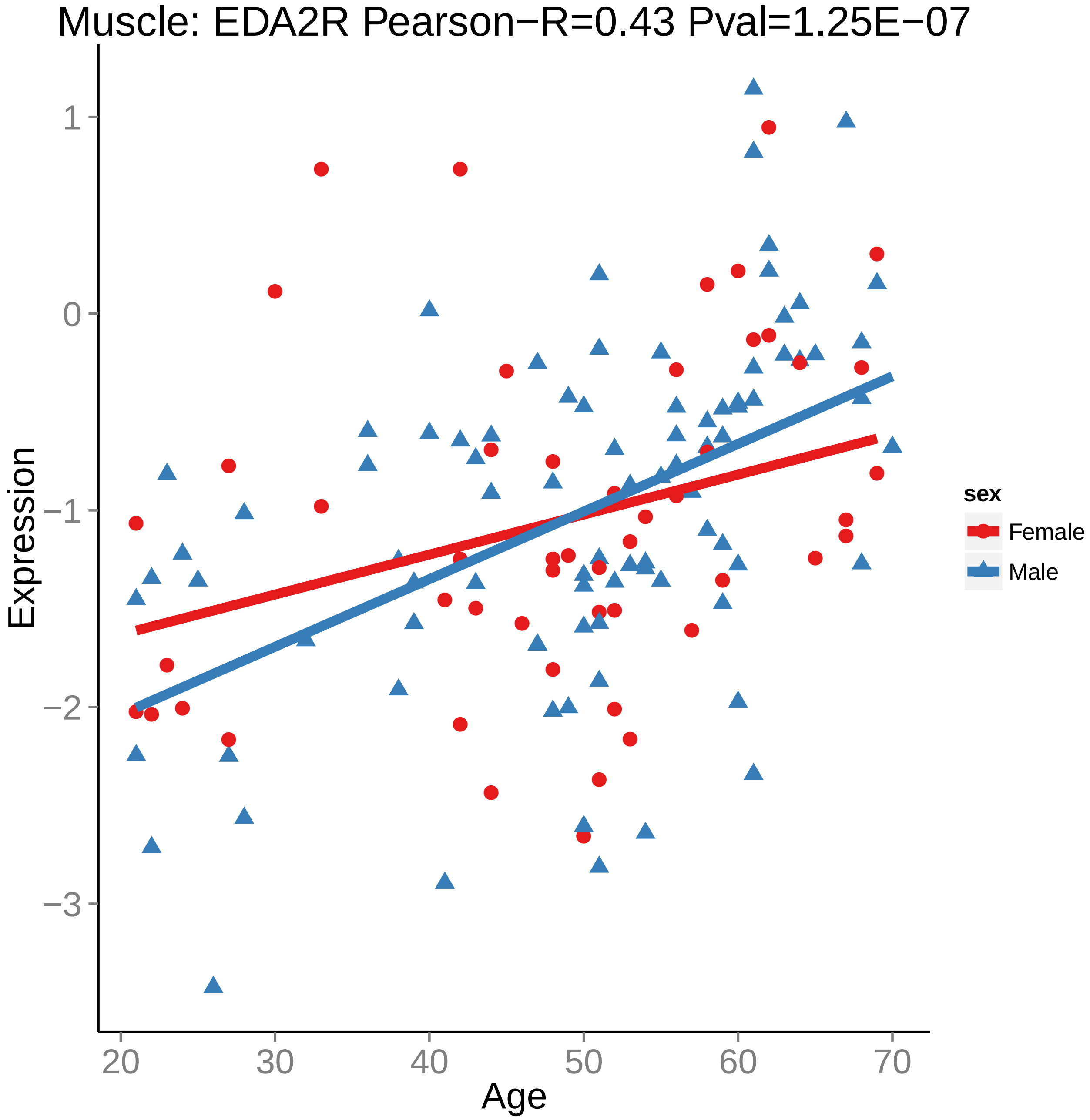

Muscle: EDA2R Pearson−R=0.43 Pval=1.25E−07
Expression
1
0
−1
−2
−3
20
30
40
50
60
70
Age
sex
Female
Male

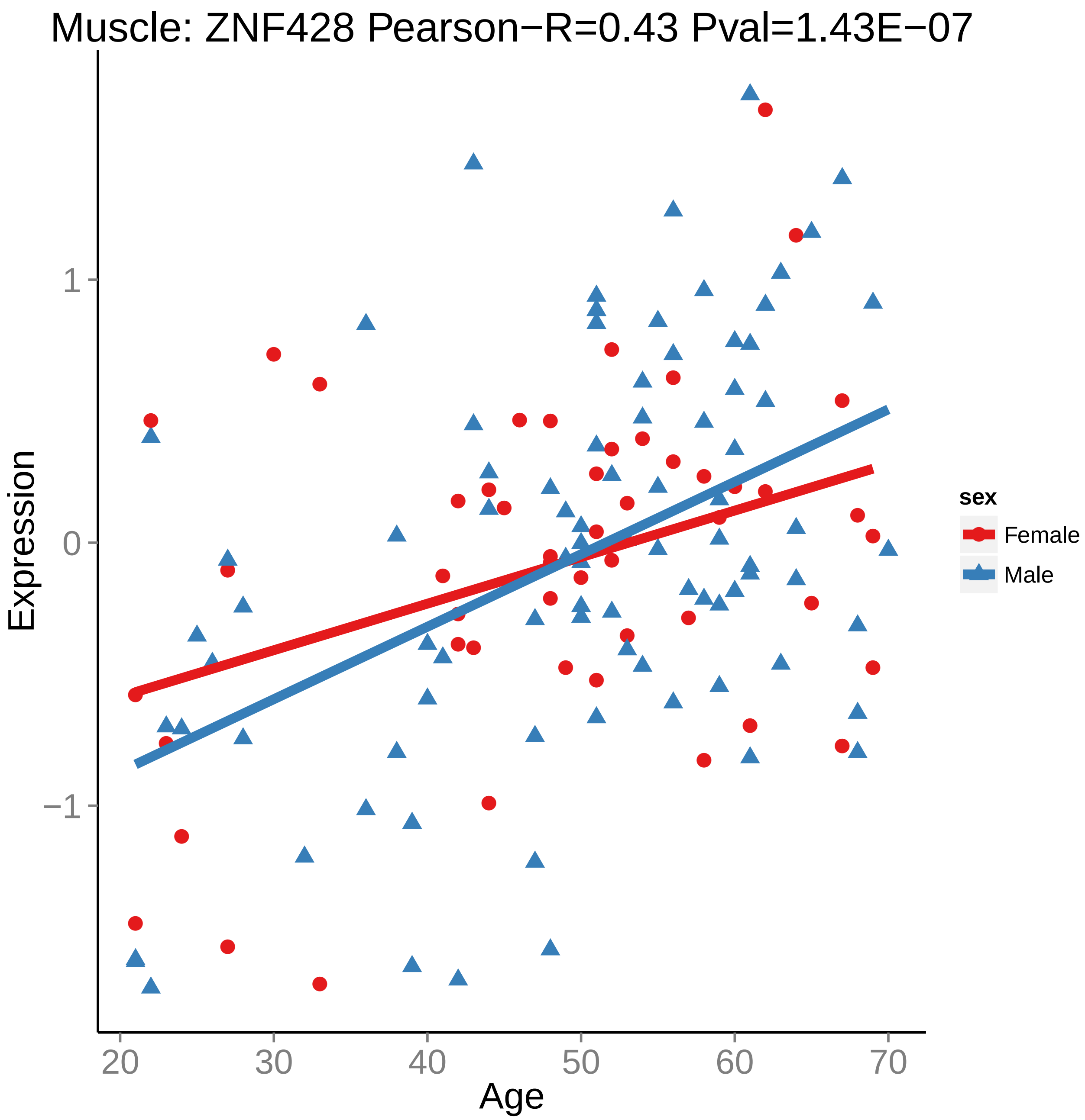
Muscle: ZNF428 Pearson−R=0.43 Pval=1.43E−07
Expression
Age
1
0
−1
20
30
40
50
60
70
sex
Female
Male

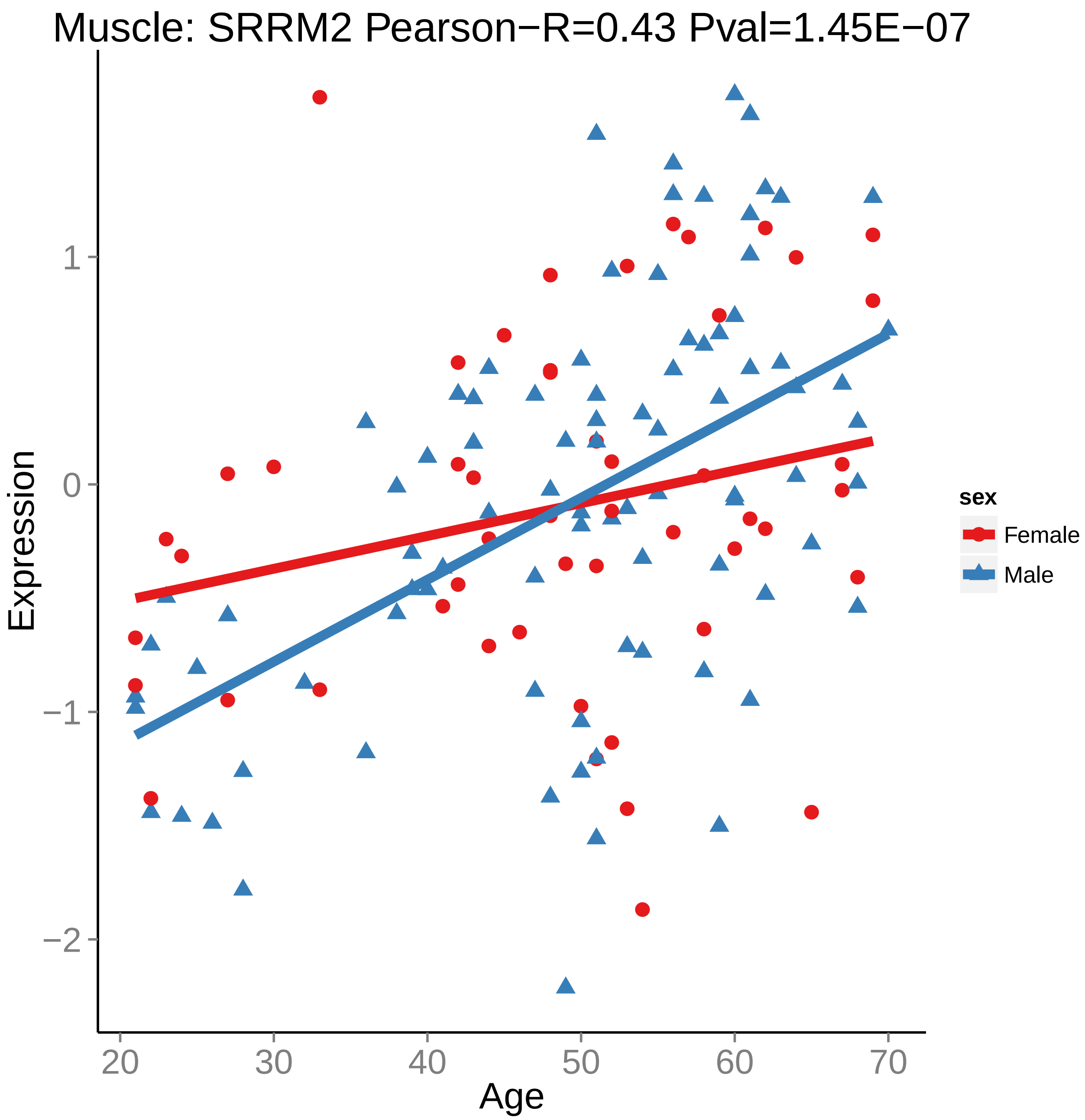

Muscle: SRRM2 Pearson−R=0.43 Pval=1.45E−07
Expression
1
0
−1
−2
20
30
40
50
60
70
Age
sex
Female
Male

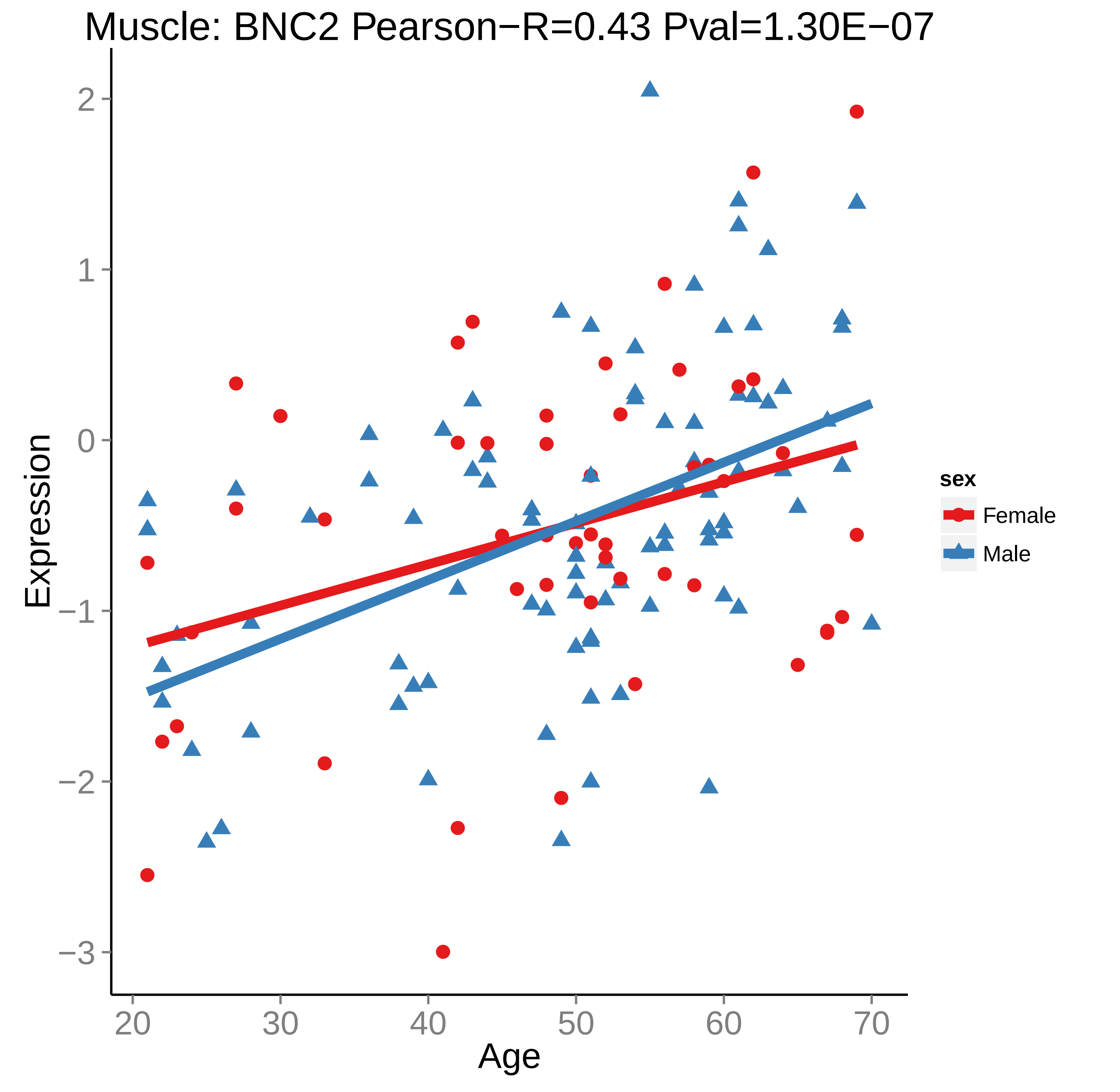
Muscle: BNC2 Pearson−R=0.43 Pval=1.30E−07
Expression
Age
2
1
0
−1
−2
−3
20
30
40
50
60
70
sex
Female
Male

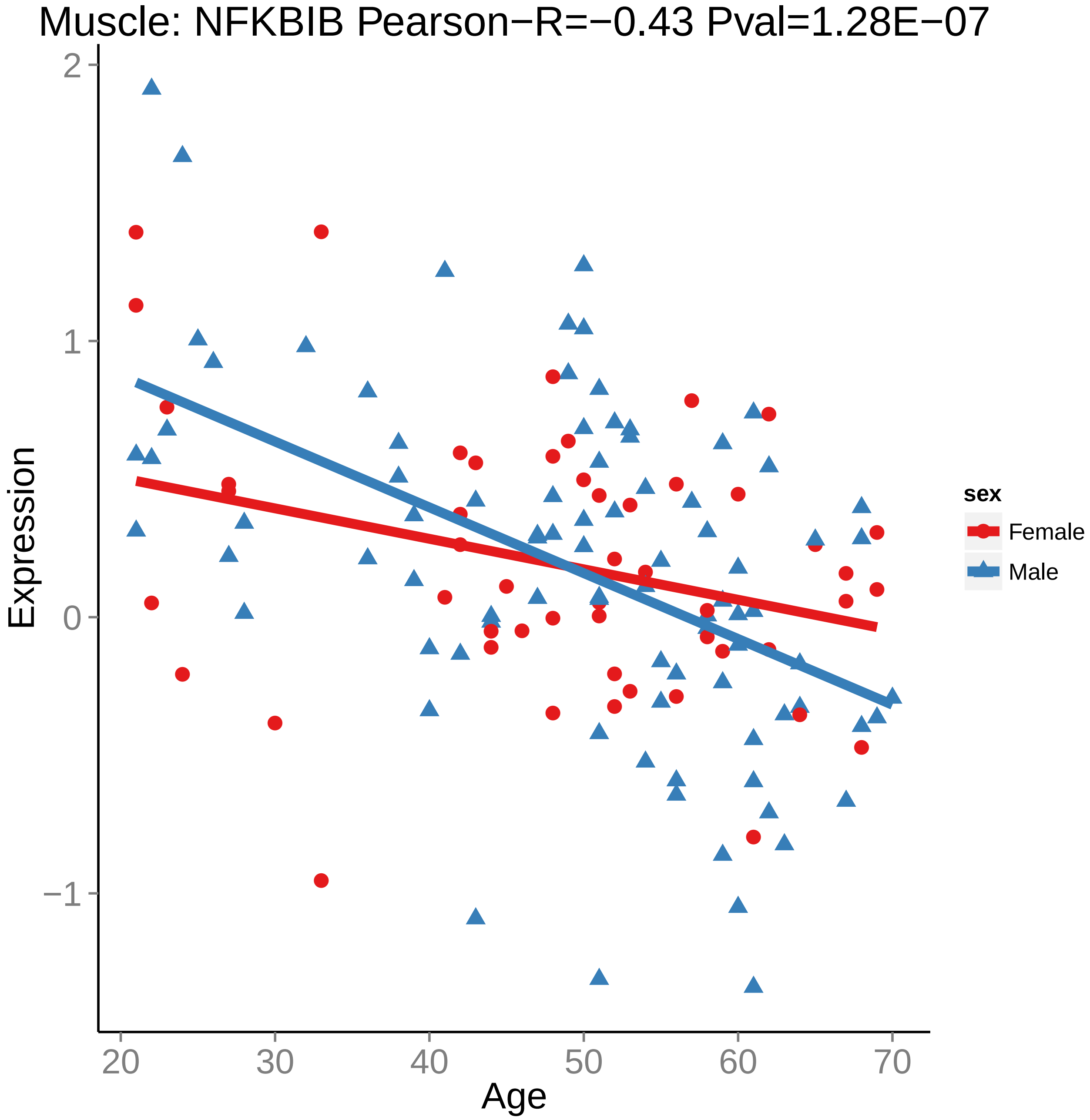

Muscle: NFKBIB Pearson−R=−0.43 Pval=1.28E−07
Expression
Age
2
1
0
−1
20
30
40
50
60
70
sex
Female
Male

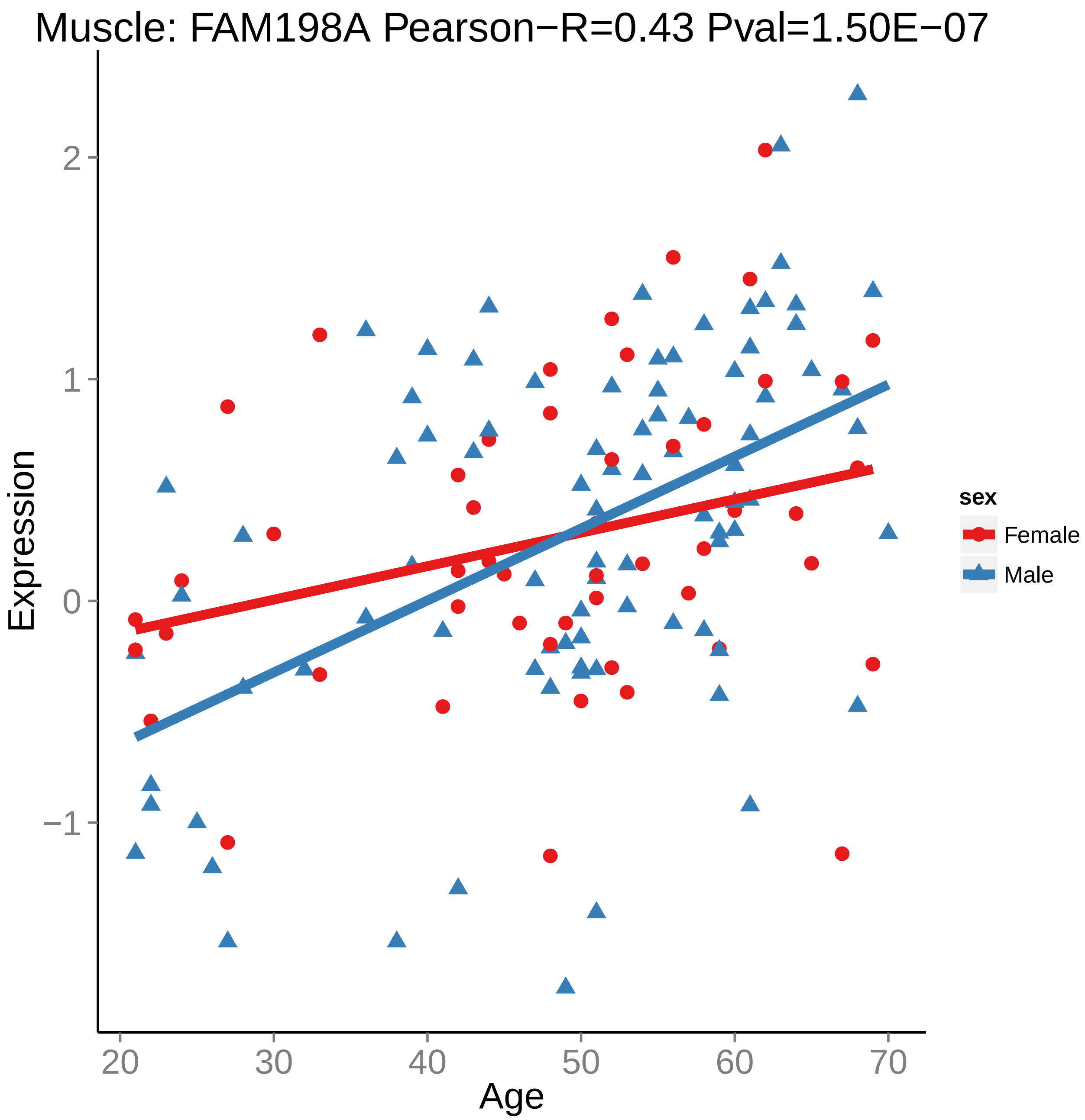

Muscle: FAM198A Pearson−R=0.43 Pval=1.50E−07
Expression
Age
2
1
0
−1
20
30
40
50
60
70
sex
Female
Male

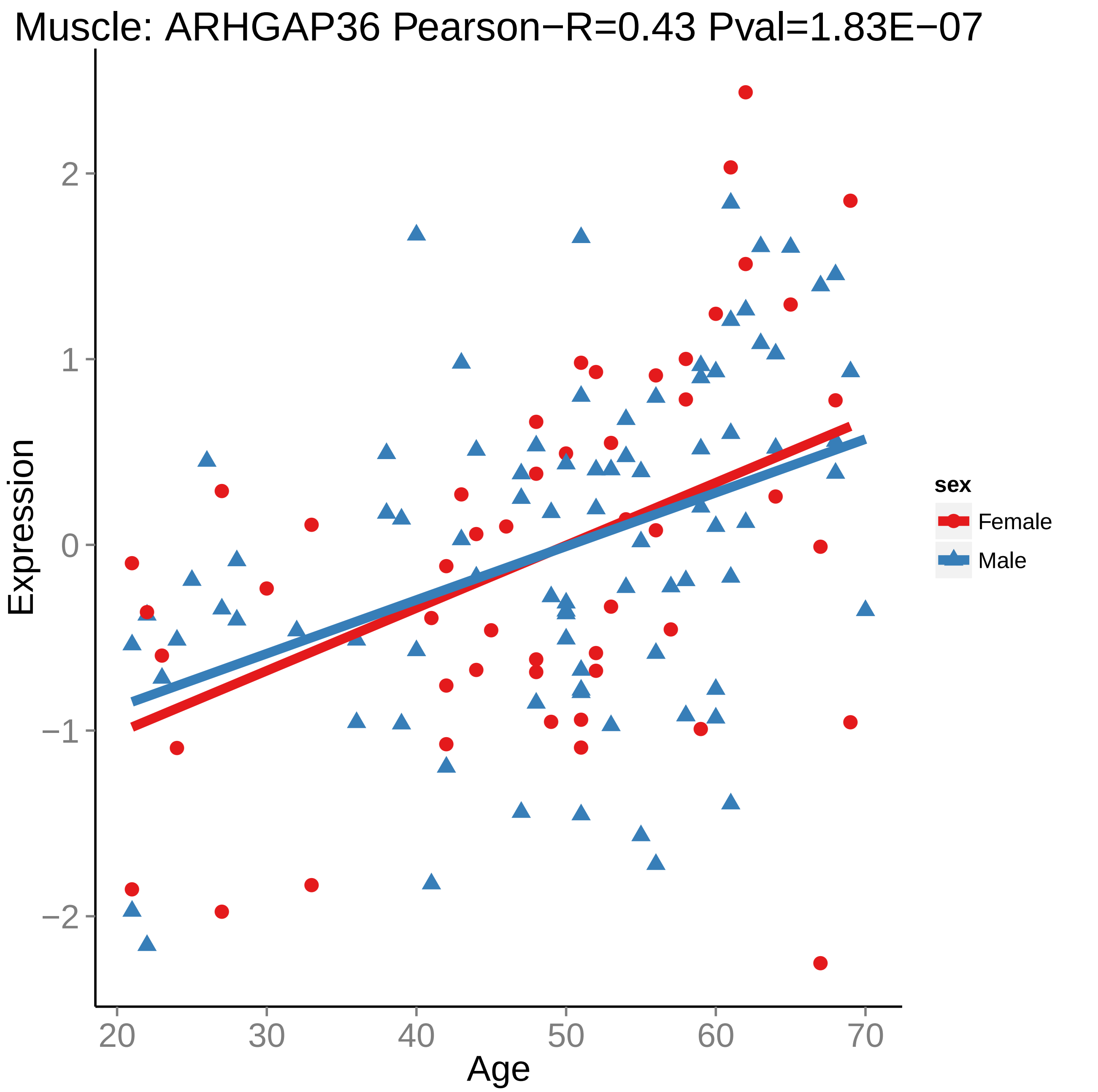

Muscle: ARHGAP36 Pearson−R=0.43 Pval=1.83E−07
Expression
Age
2
1
0
−1
−2
20
30
40
50
60
70
sex
Female
Male

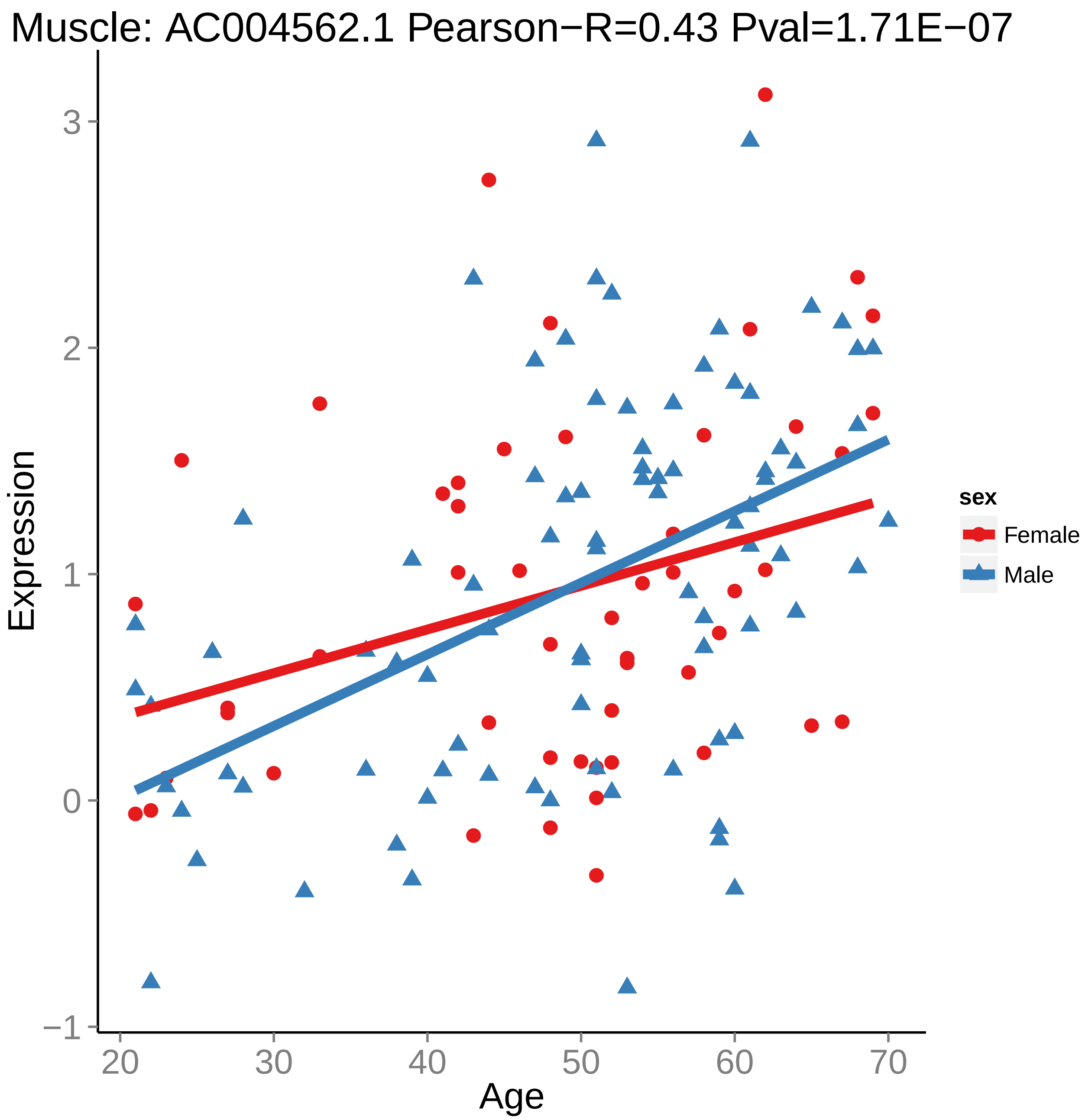

Muscle: AC004562.1 Pearson−R=0.43 Pval=1.71E−07
Expression
Age
3
2
1
0
−1
20
30
40
50
60
70
sex
Female
Male

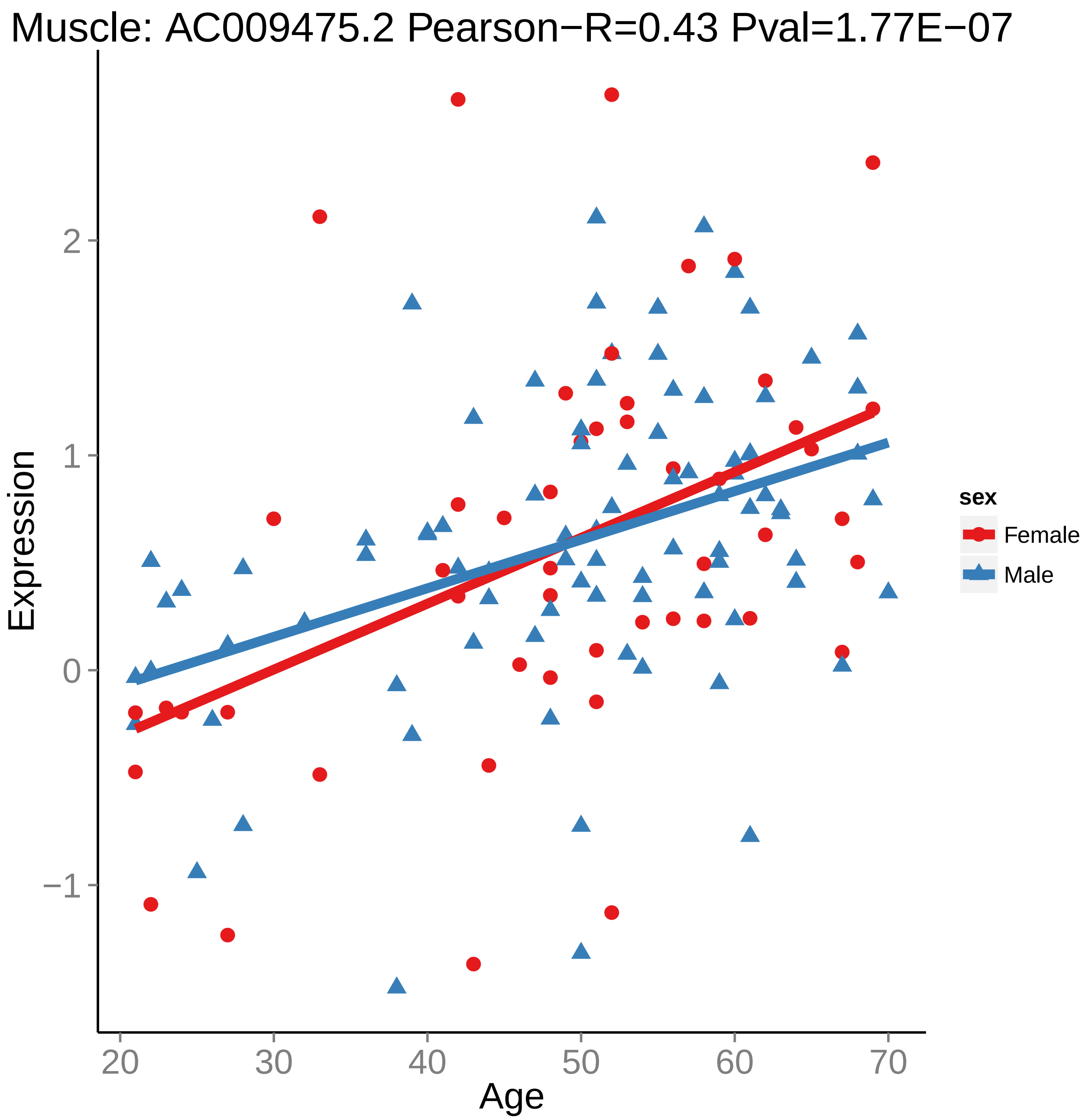
Muscle: AC009475.2 Pearson−R=0.43 Pval=1.77E−07
Expression
Age
2
1
0
−1
20
30
40
50
60
70
sex
Female
Male

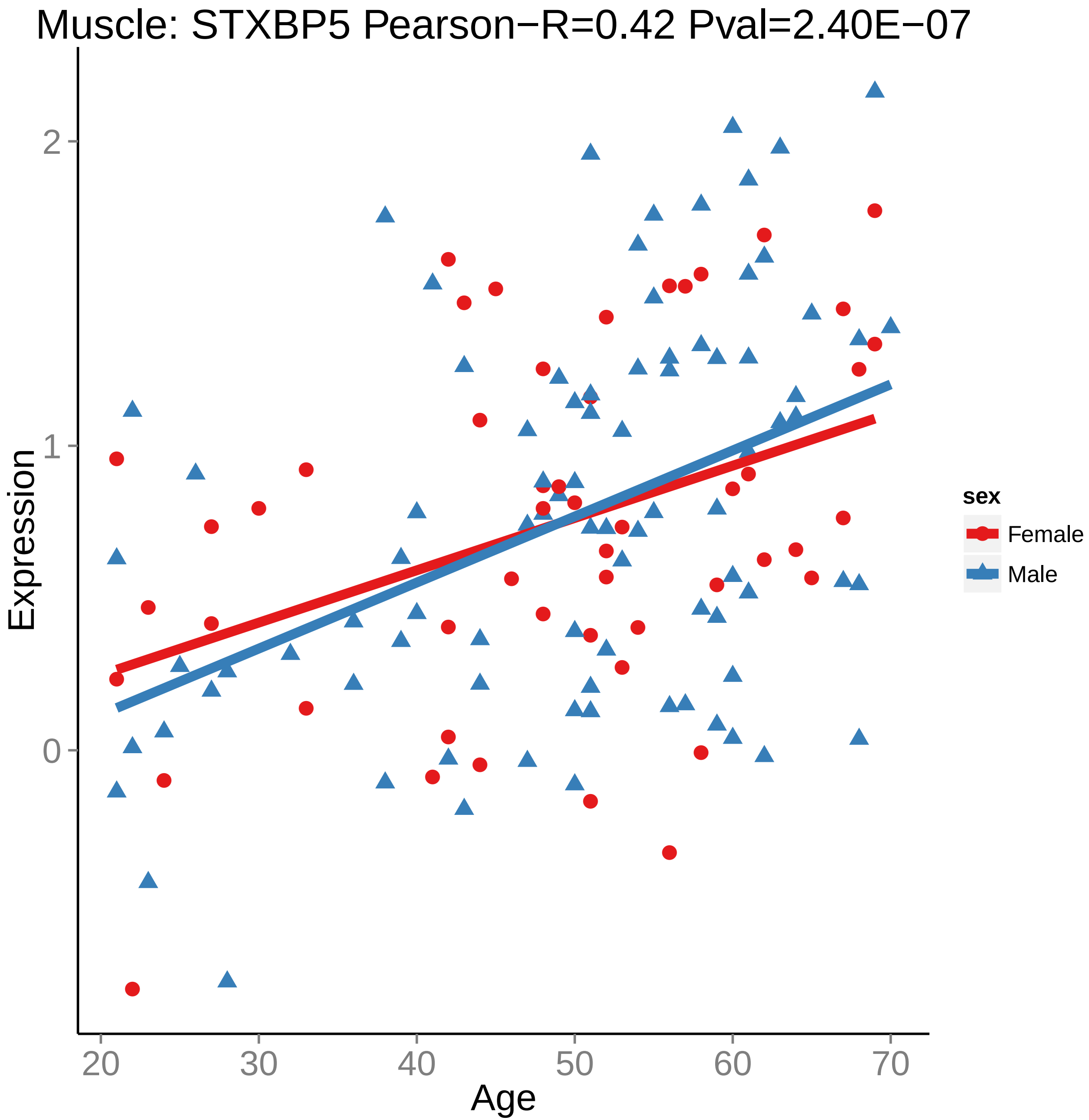
Muscle: STXBP5 Pearson−R=0.42 Pval=2.40E−07
Expression
Age
0
1
2
20
30
40
50
60
70
sex
Female
Male

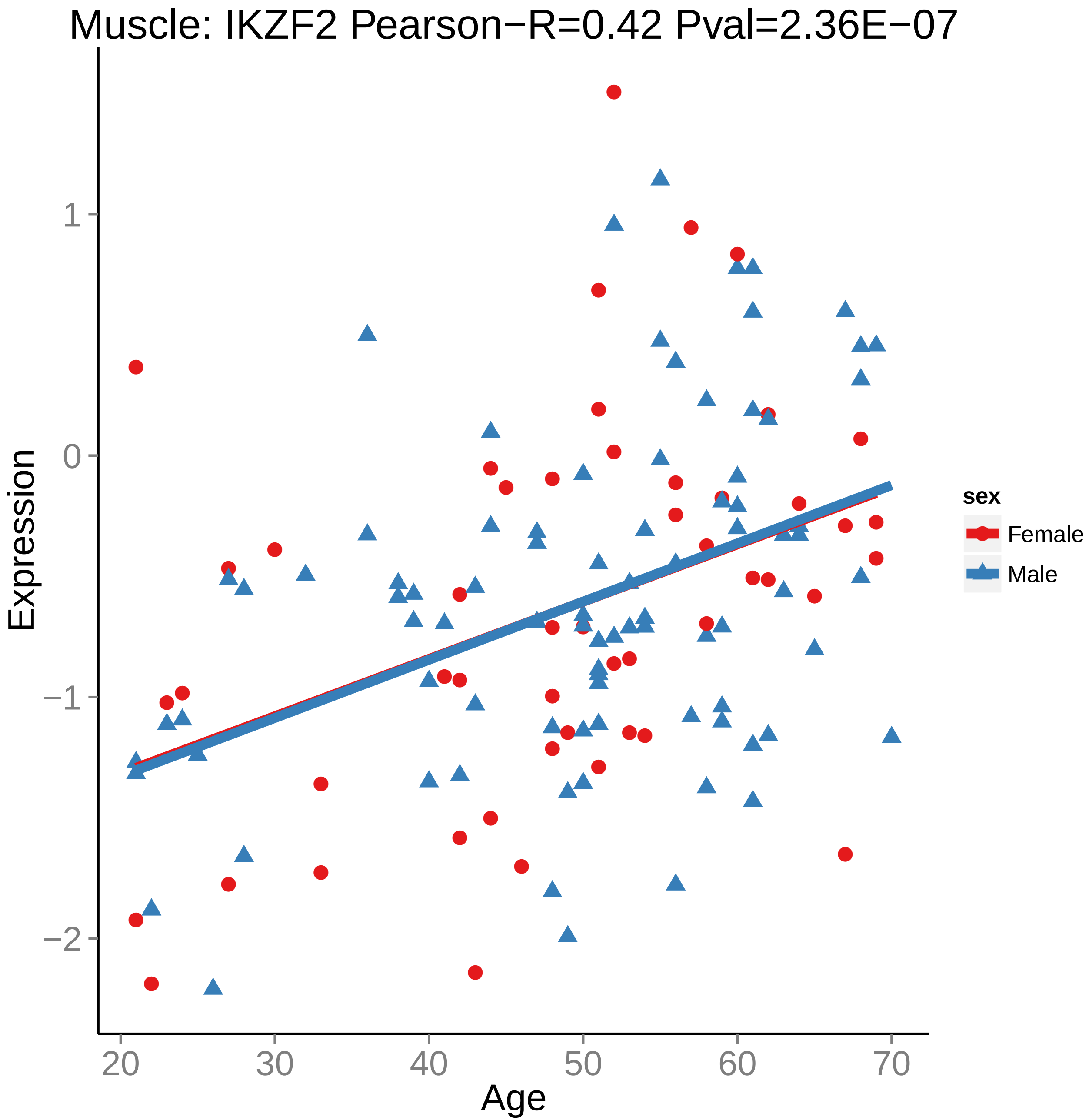
Muscle: IKZF2 Pearson−R=0.42 Pval=2.36E−07
Expression
Age
1
0
−1
−2
20
30
40
50
60
70
sex
Female
Male

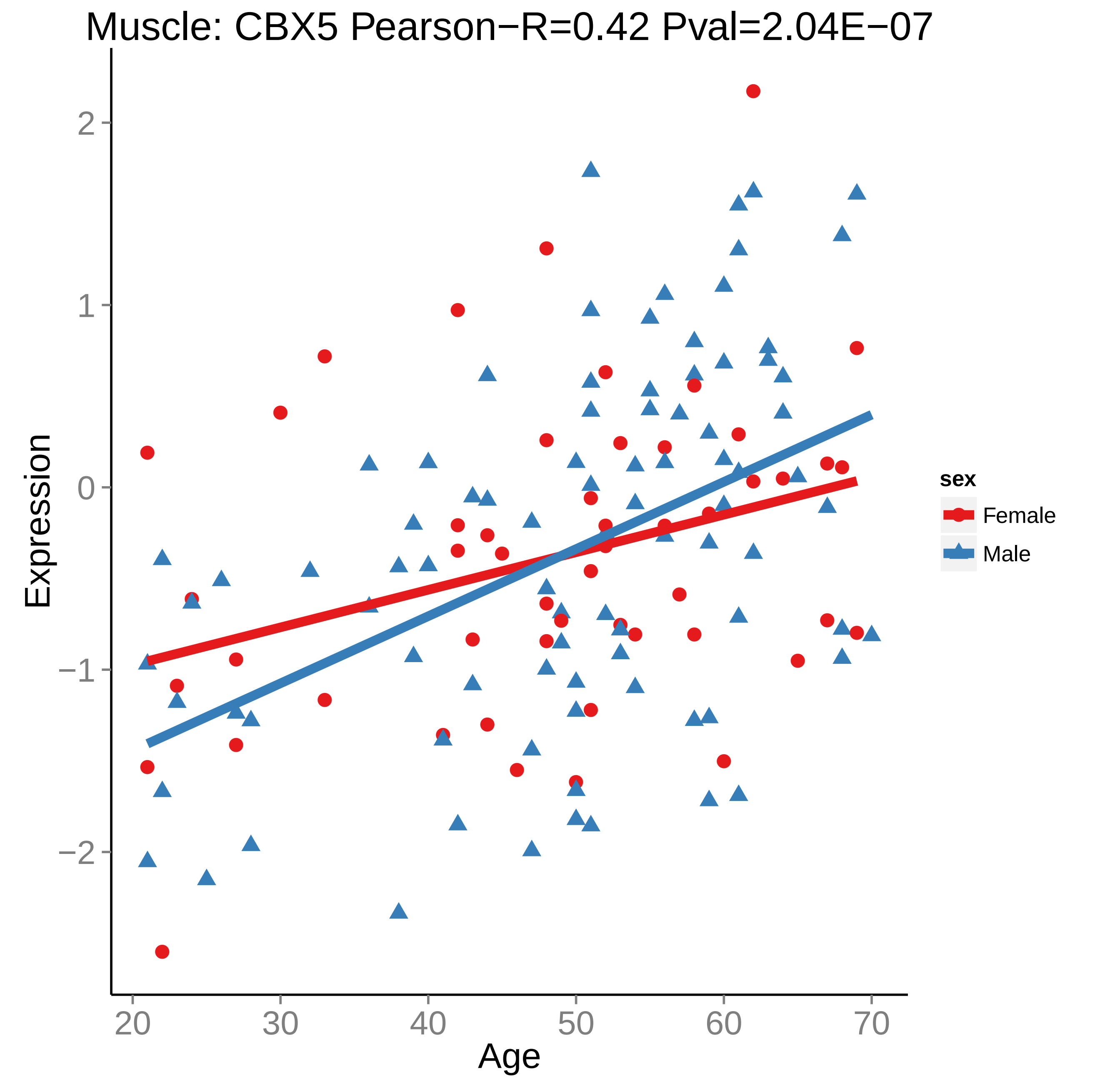

Muscle: CBX5 Pearson−R=0.42 Pval=2.04E−07
Expression
Age
sex
Female
Male

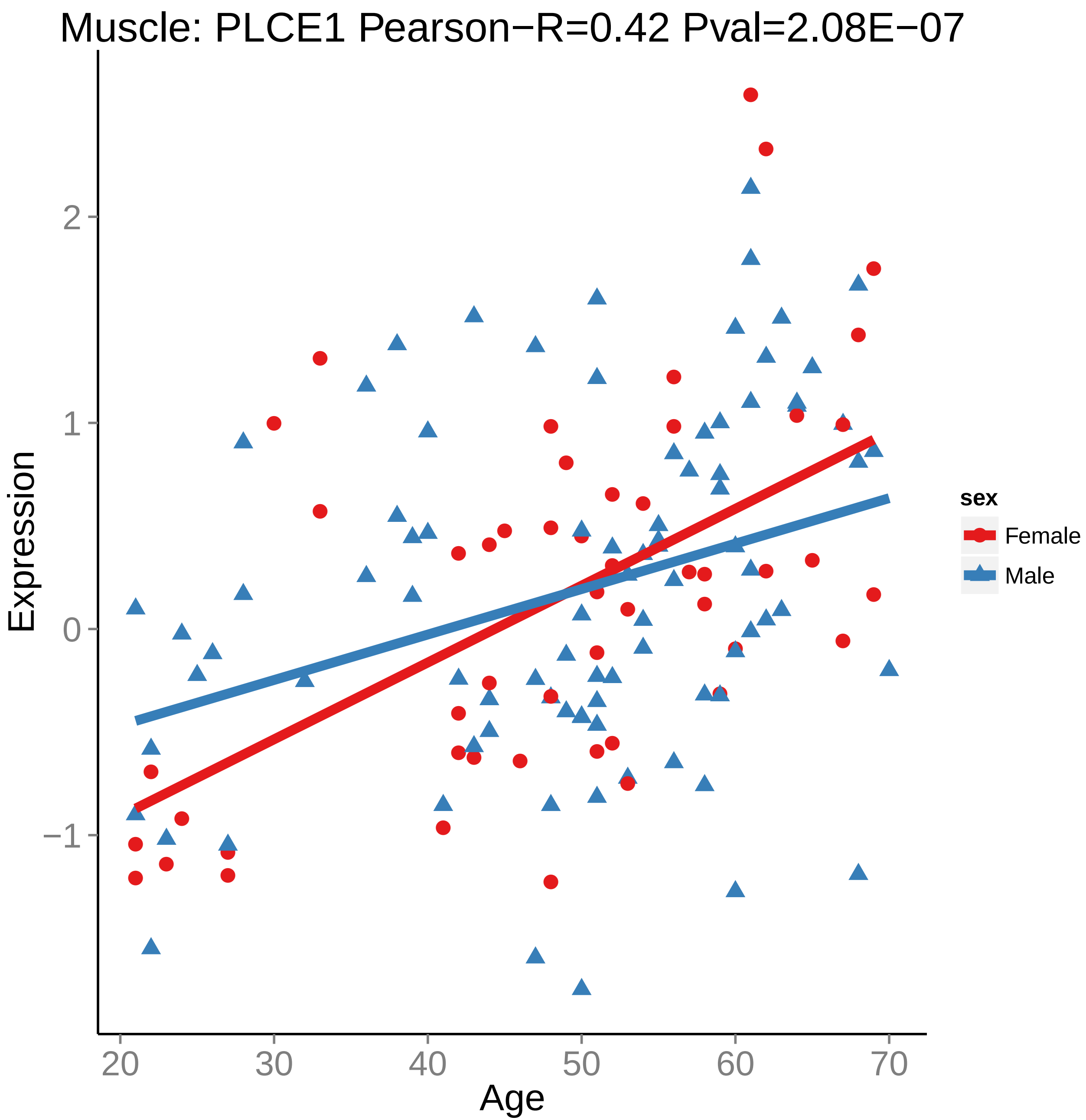
Muscle: PLCE1 Pearson−R=0.42 Pval=2.08E−07
Expression
Age
−1
0
1
2
20
30
40
50
60
70
sex
Female
Male

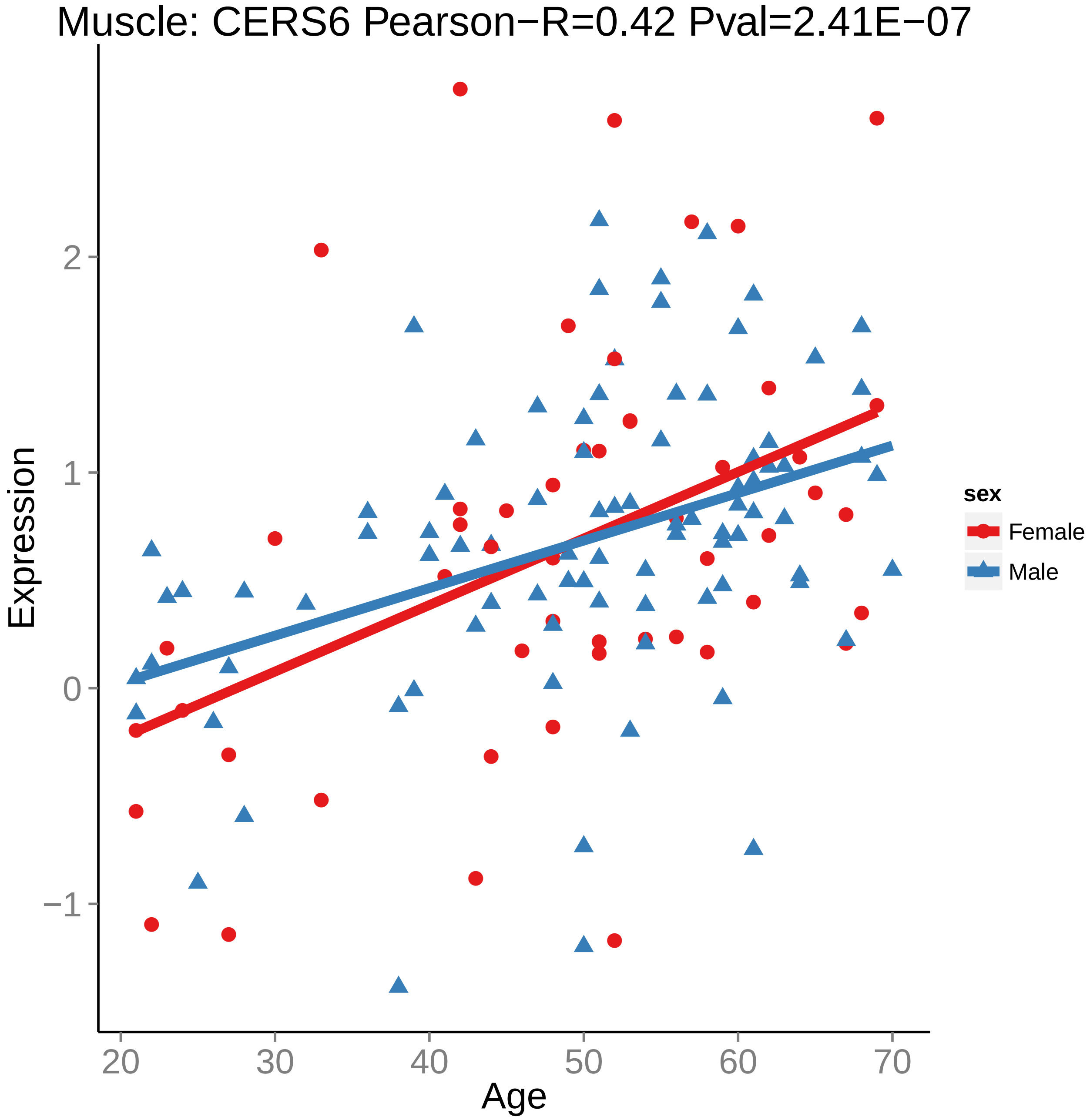

Muscle: CERS6 Pearson−R=0.42 Pval=2.41E−07
Expression
Age
2
1
0
−1
20
30
40
50
60
70
sex
Female
Male

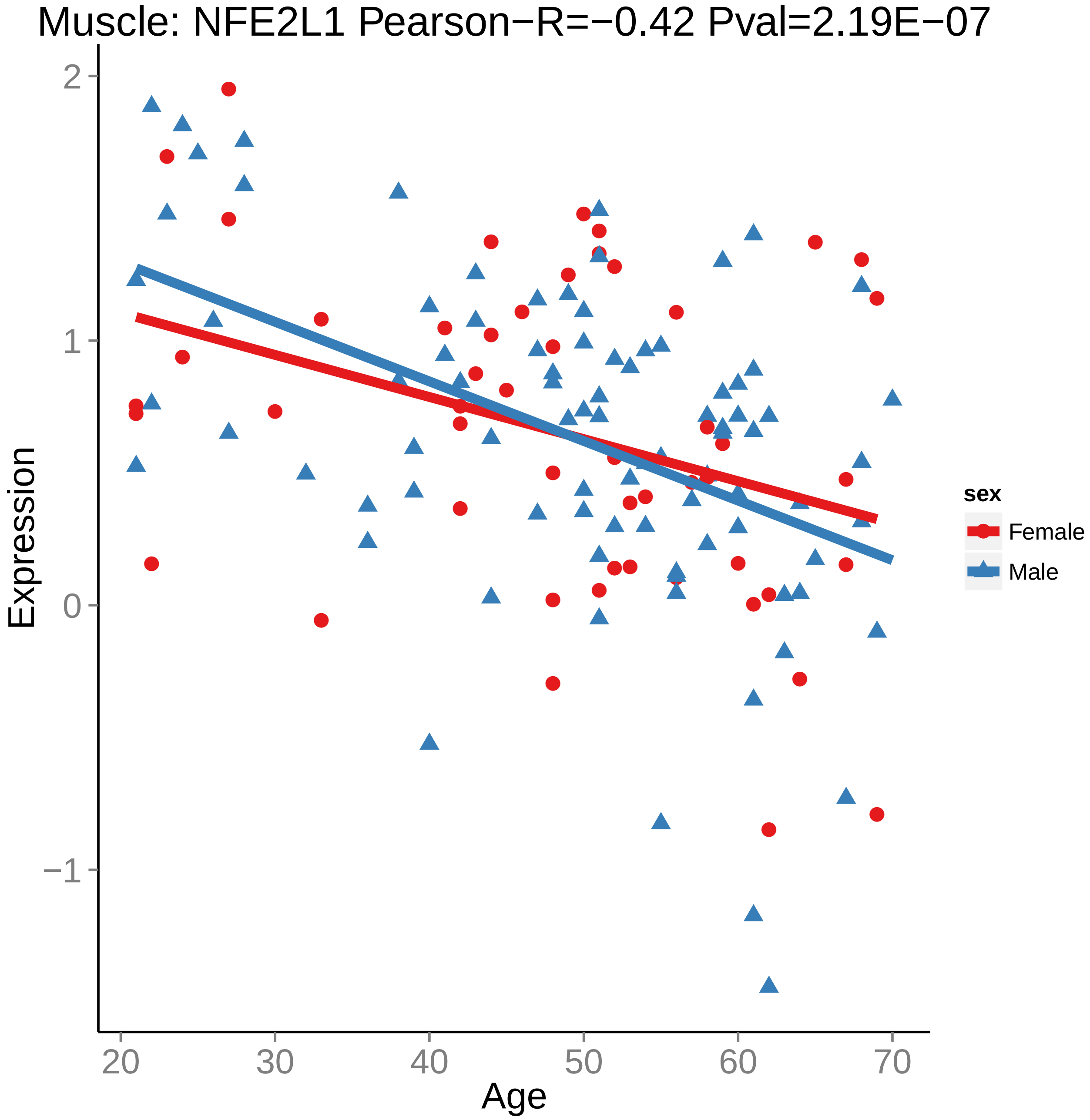
Muscle: NFE2L1 Pearson−R=−0.42 Pval=2.19E−07
Expression
2
1
0
−1
20
30
40
50
60
70
Age
sex
Female
Male

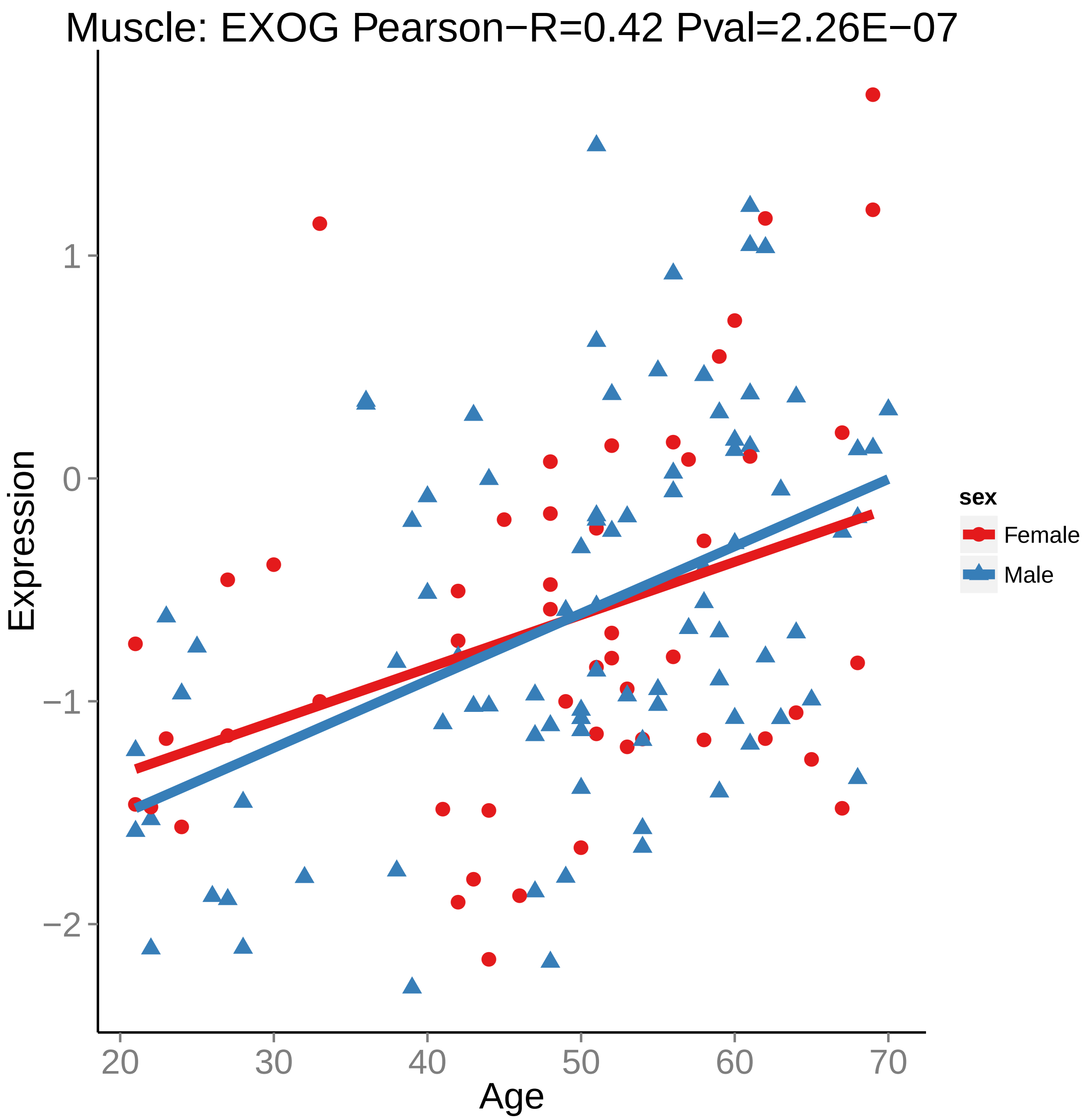

Muscle: EXOG Pearson−R=0.42 Pval=2.26E−07
Expression
1
0
−1
−2
20
30
40
50
60
70
Age
sex
Female
Male

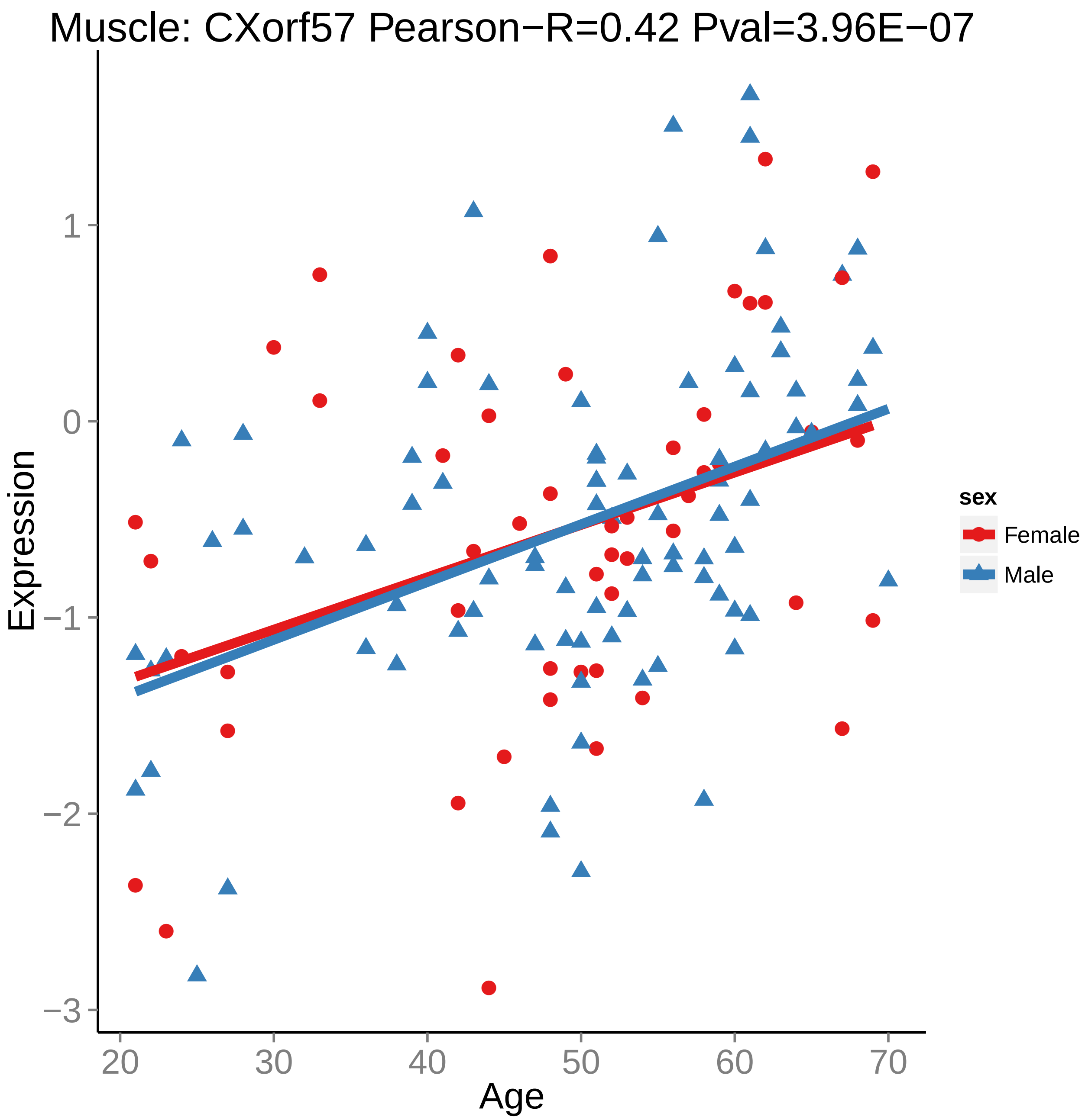

Muscle: CXorf57 Pearson−R=0.42 Pval=3.96E−07
Expression
1
0
−1
−2
−3
20
30
40
50
60
70
Age
sex
Female
Male

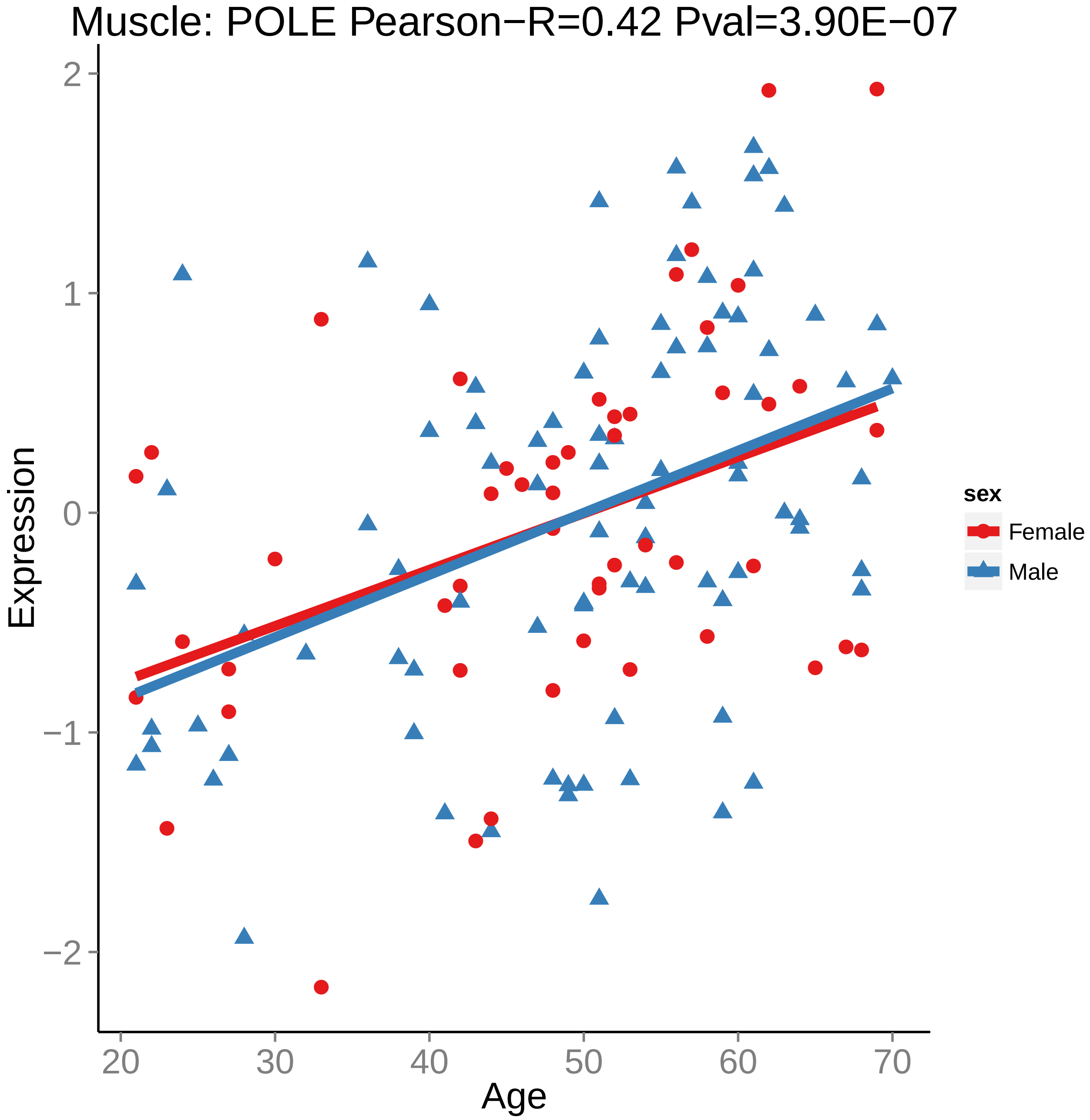

Muscle: POLE Pearson−R=0.42 Pval=3.90E−07
Expression
Age
2
1
0
−1
−2
20
30
40
50
60
70
sex
Female
Male

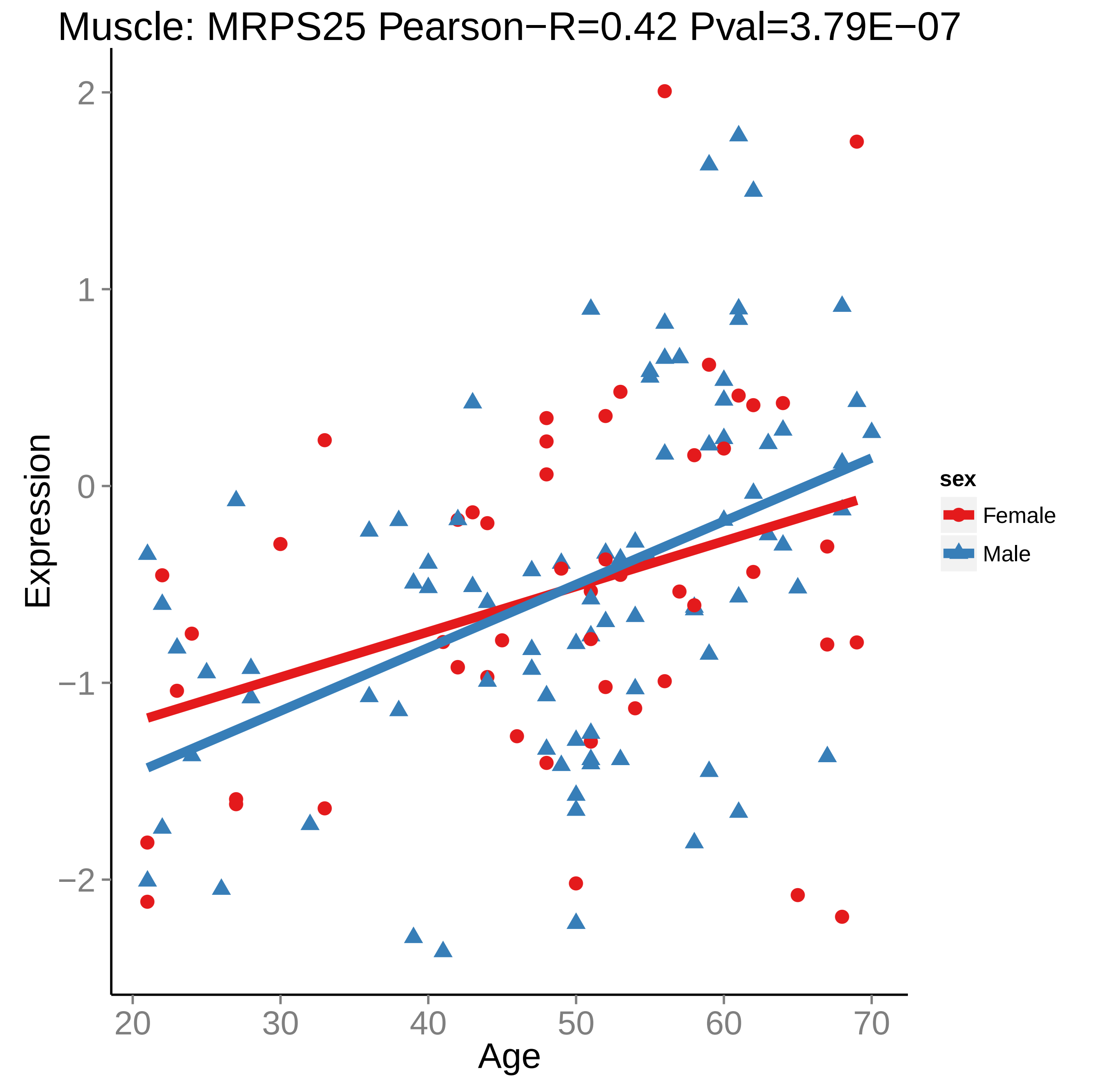

Muscle: MRPS25 Pearson−R=0.42 Pval=3.79E−07
Expression
Age
sex
Female
Male
2
1
0
−1
−2
20
30
40
50
60
70

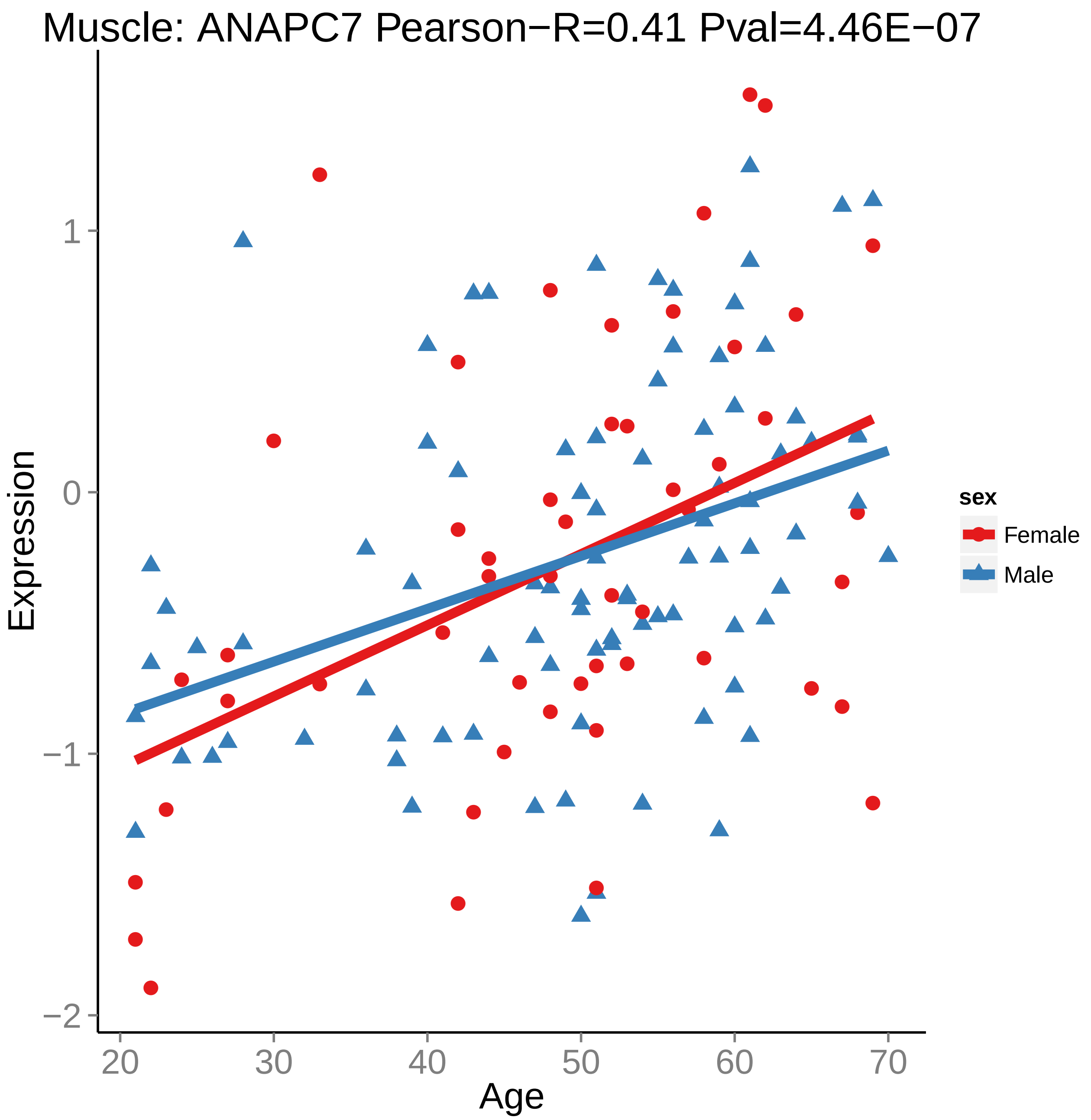
Muscle: ANAPC7 Pearson−R=0.41 Pval=4.46E−07
Expression
1
0
−1
−2
20
30
40
50
60
70
Age
sex
Female
Male

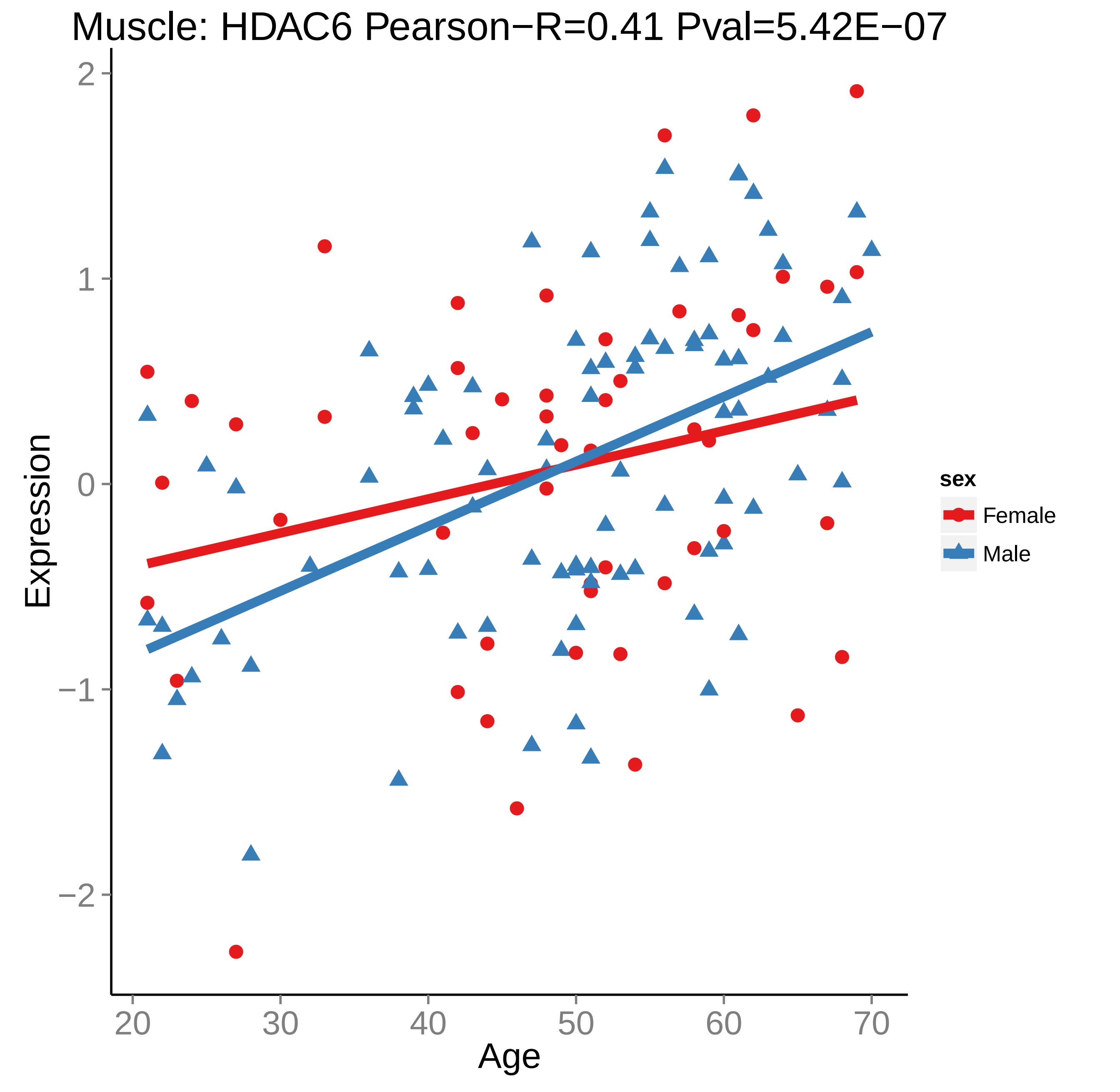

Muscle: HDAC6 Pearson−R=0.41 Pval=5.42E−07
Expression
Age
2
1
0
−1
−2
20
30
40
50
60
70
sex
Female
Male

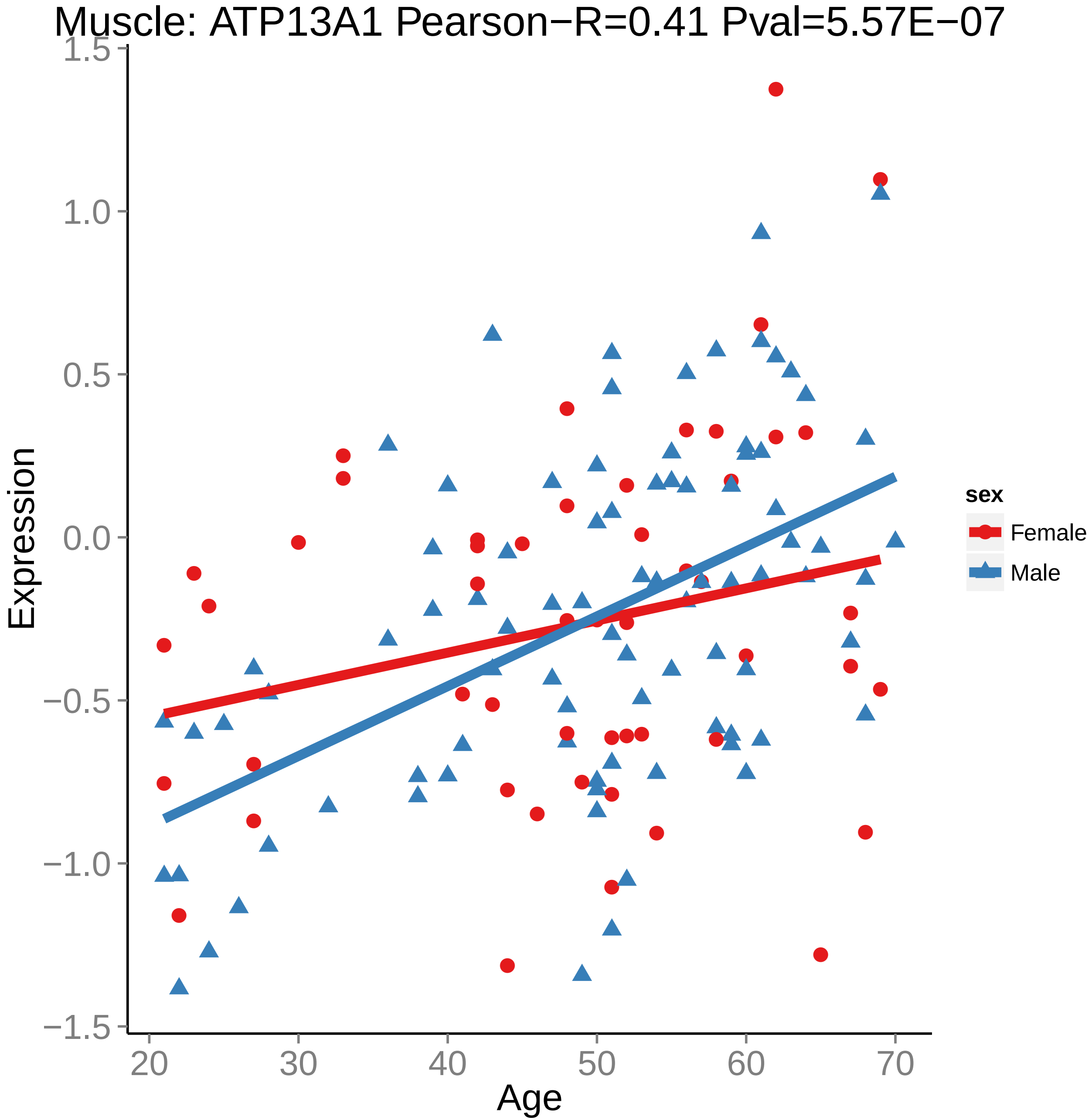

Muscle: ATP13A1 Pearson−R=0.41 Pval=5.57E−07
Expression
Age
1.5
1.0
0.5
0.0
−0.5
−1.0
−1.5
20
30
40
50
60
70
sex
Female
Male

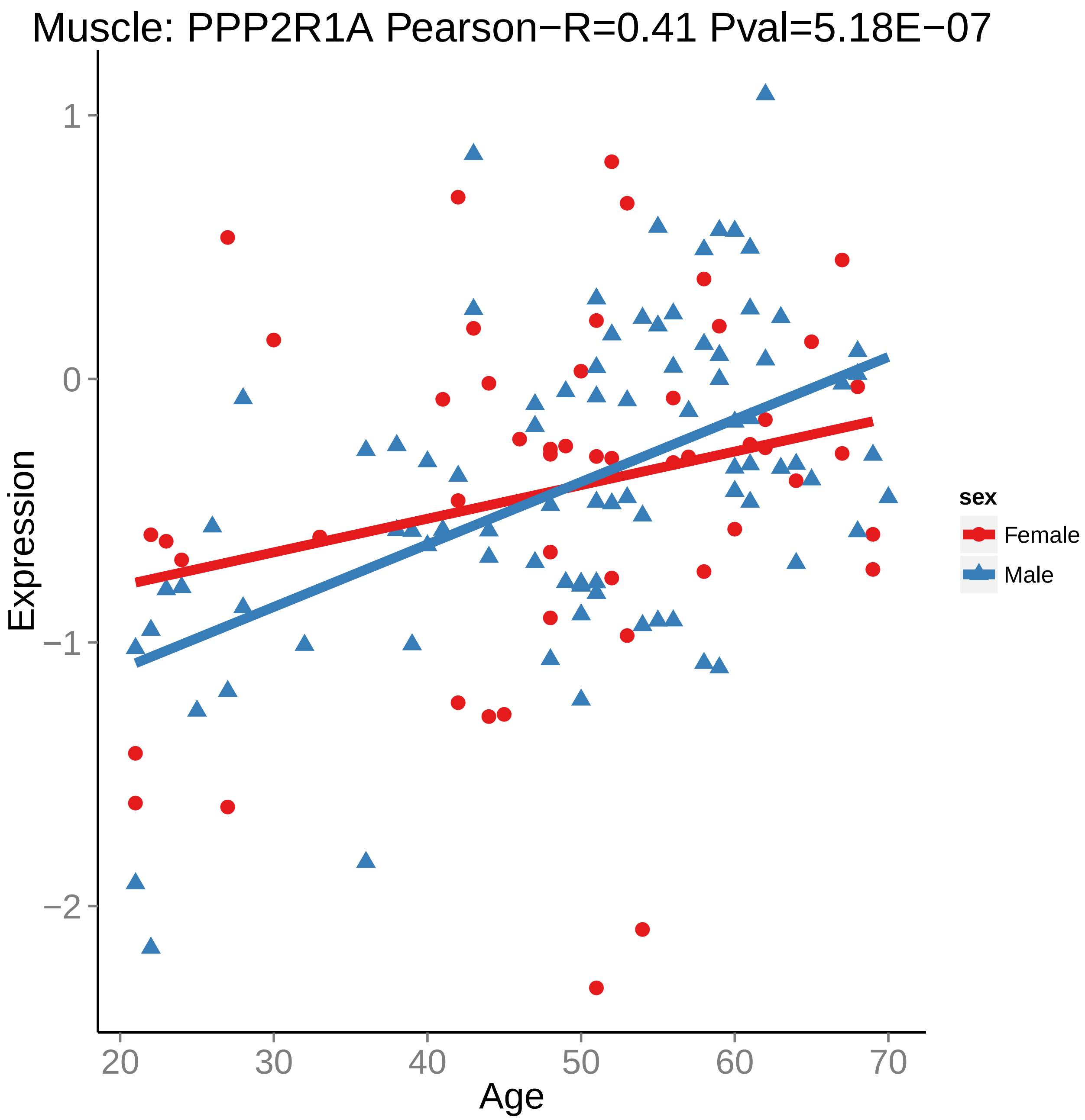

Muscle: PPP2R1A Pearson−R=0.41 Pval=5.18E−07
Expression
Age
1
0
−1
−2
20
30
40
50
60
70
sex
Female
Male

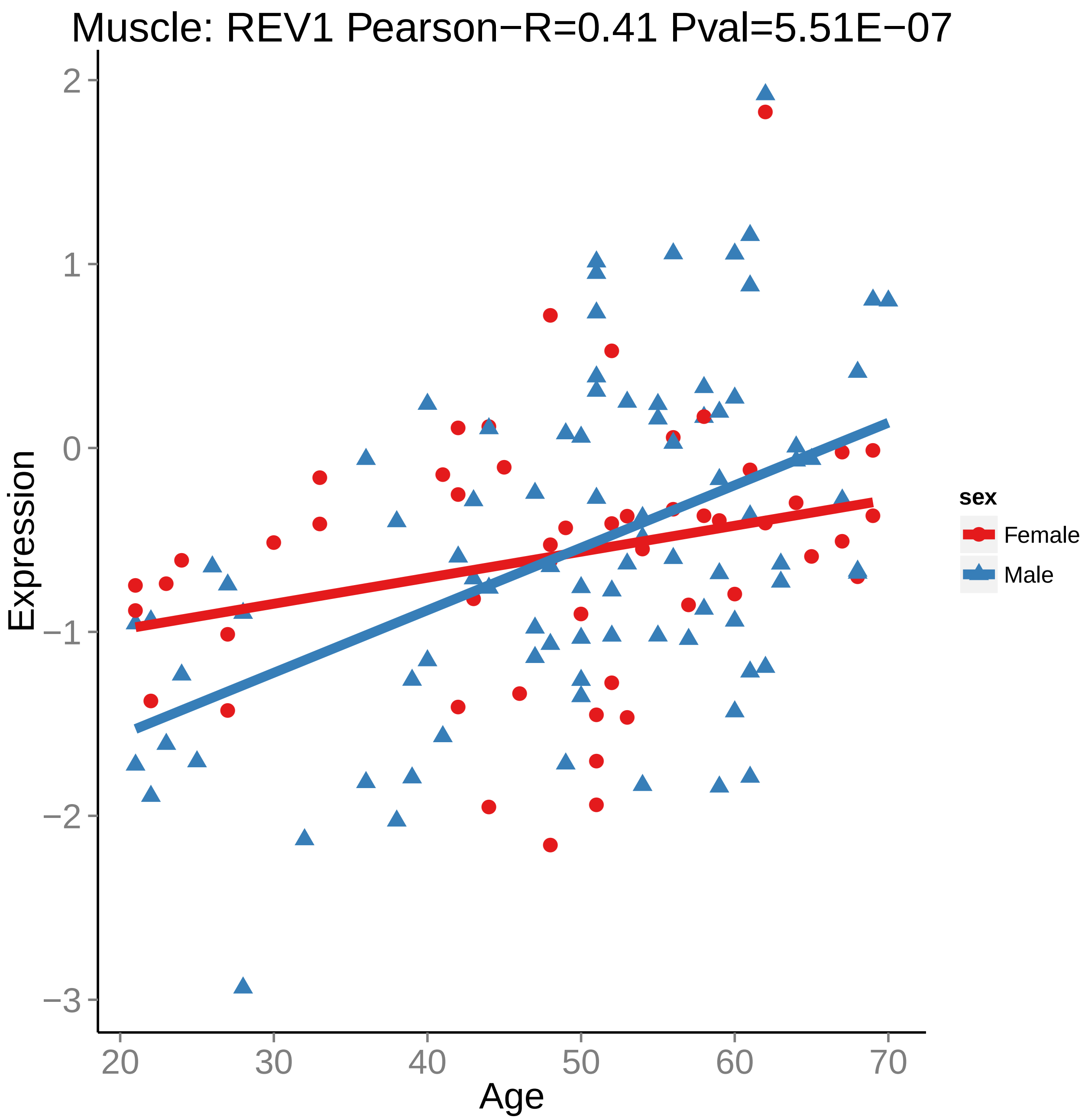

Muscle: REV1 Pearson−R=0.41 Pval=5.51E−07
Expression
2
1
0
−1
−2
−3
20
30
40
50
60
70
Age
sex
Female
Male

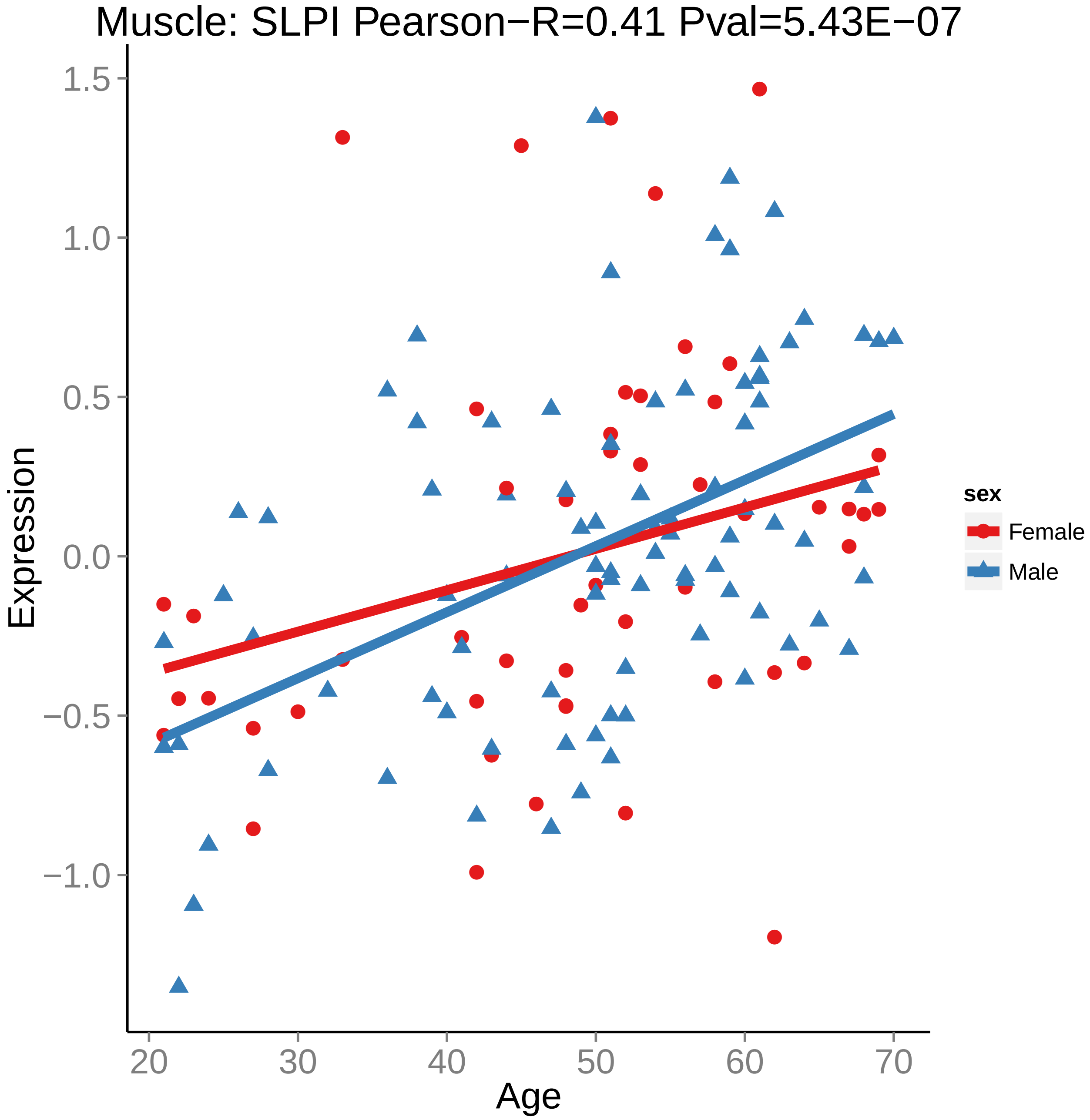

Muscle: SLPI Pearson−R=0.41 Pval=5.43E−07
1.5
1.0
0.5
0.0
−0.5
−1.0
Expression
20
30
40
50
60
70
Age
sex
Female
Male

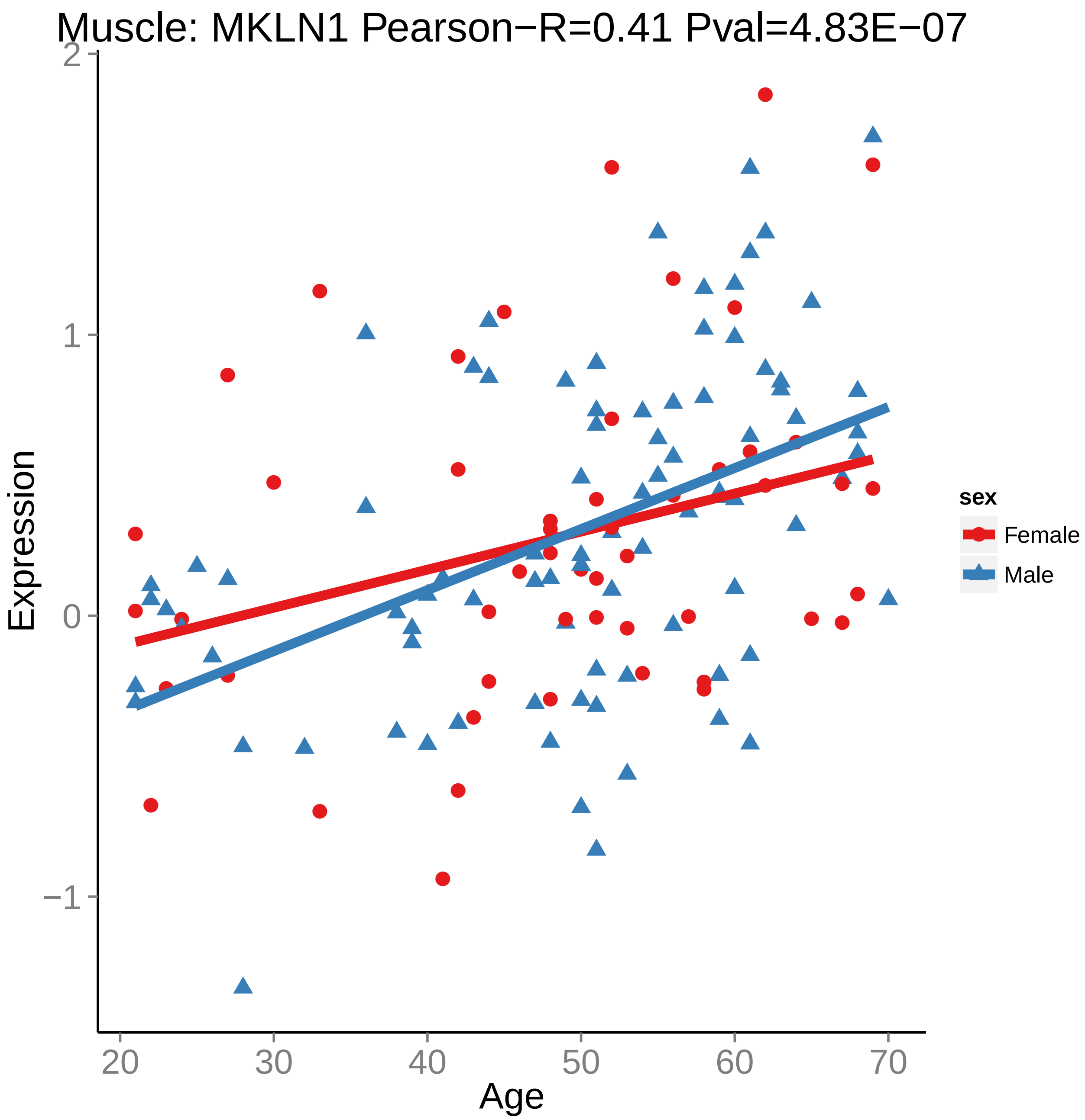
Muscle: MKLN1 Pearson−R=0.41 Pval=4.83E−07
Expression
Age
2
1
0
−1
20
30
40
50
60
70
sex
Female
Male

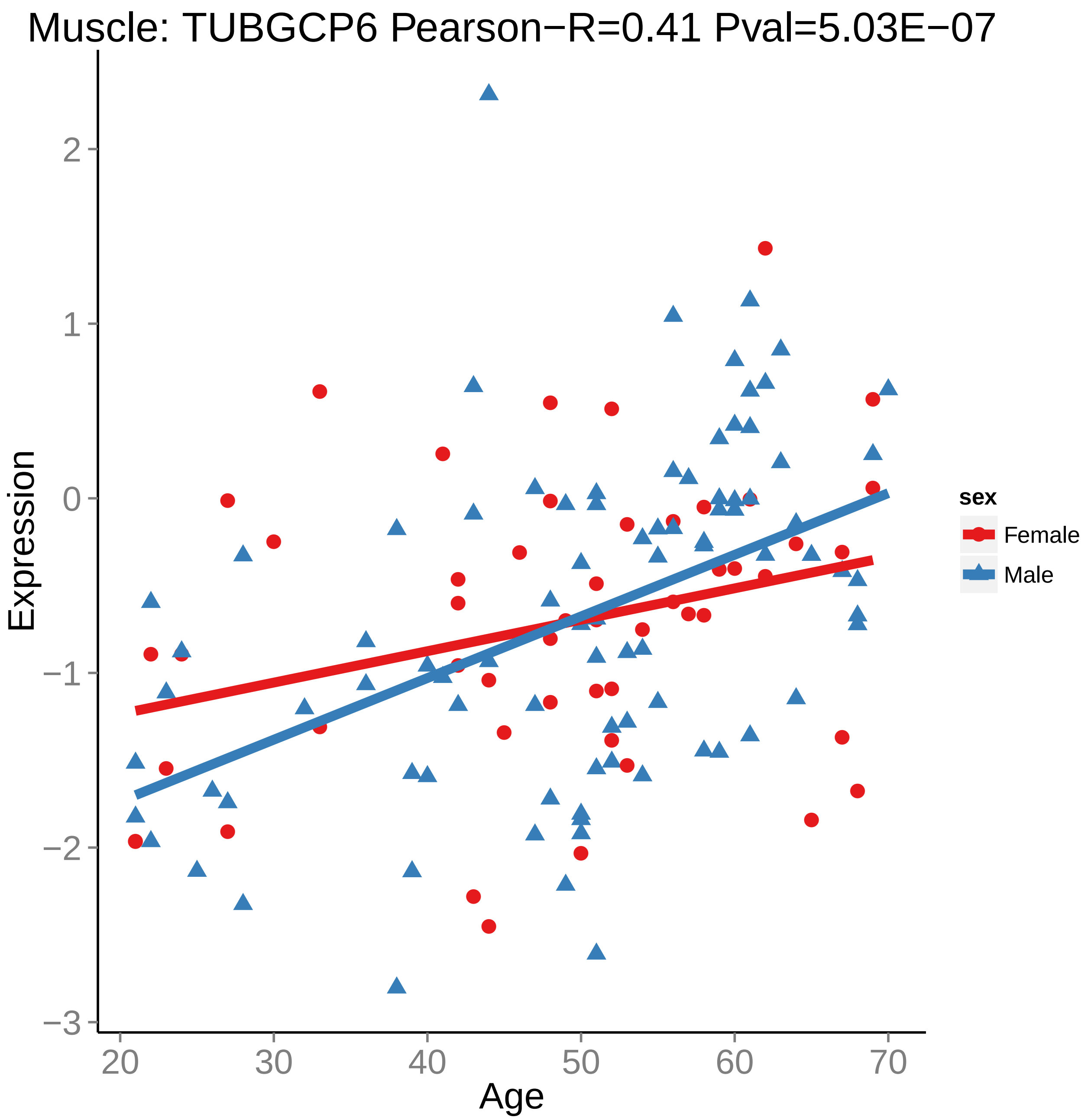

Muscle: TUBGCP6 Pearson−R=0.41 Pval=5.03E−07
Expression
Age
sex
Female
Male
2
1
0
−1
−2
−3
20
30
40
50
60
70

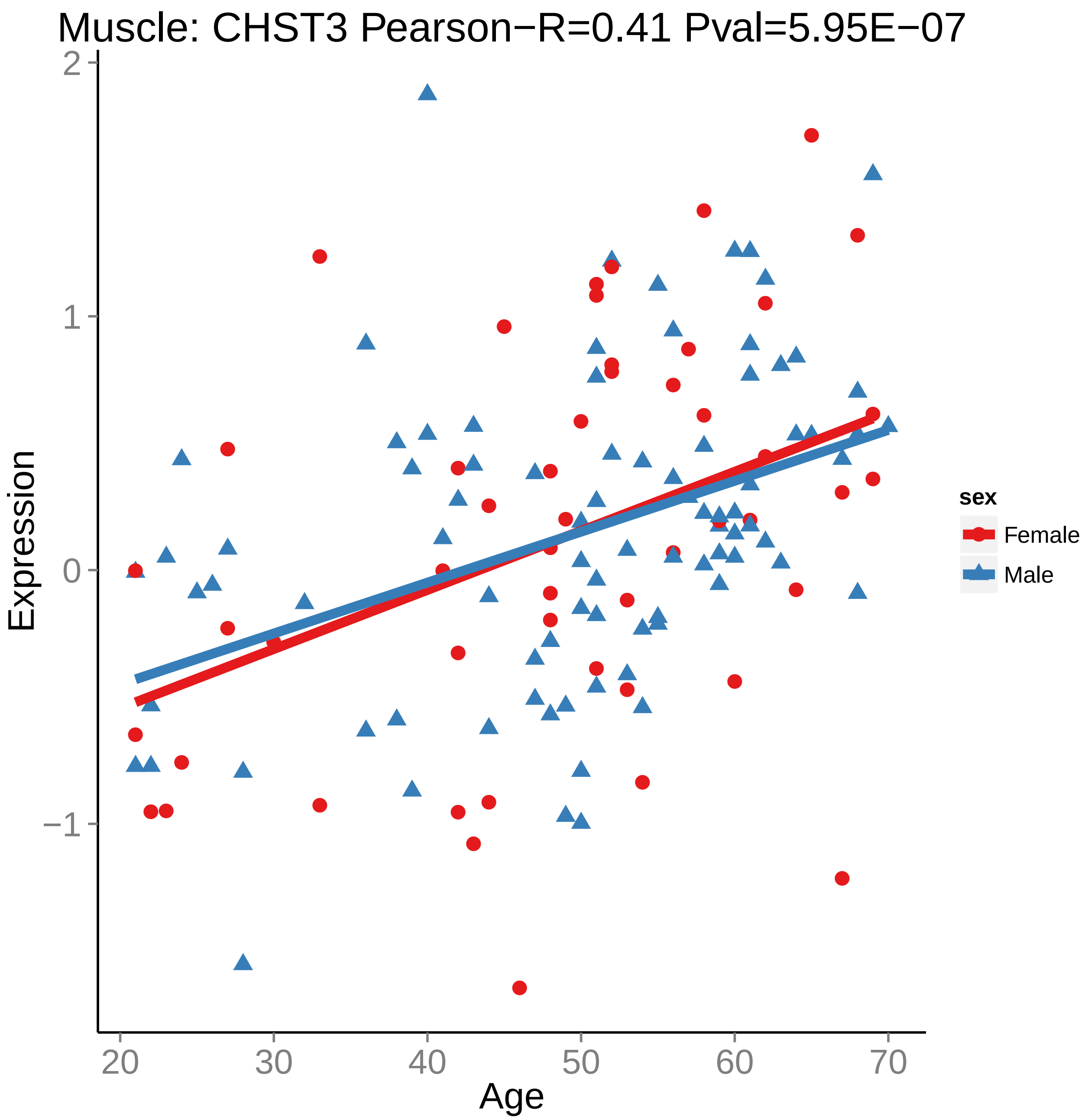

Muscle: CHST3 Pearson−R=0.41 Pval=5.95E−07
Expression
Age
2
1
0
−1
20
30
40
50
60
70
sex
Female
Male

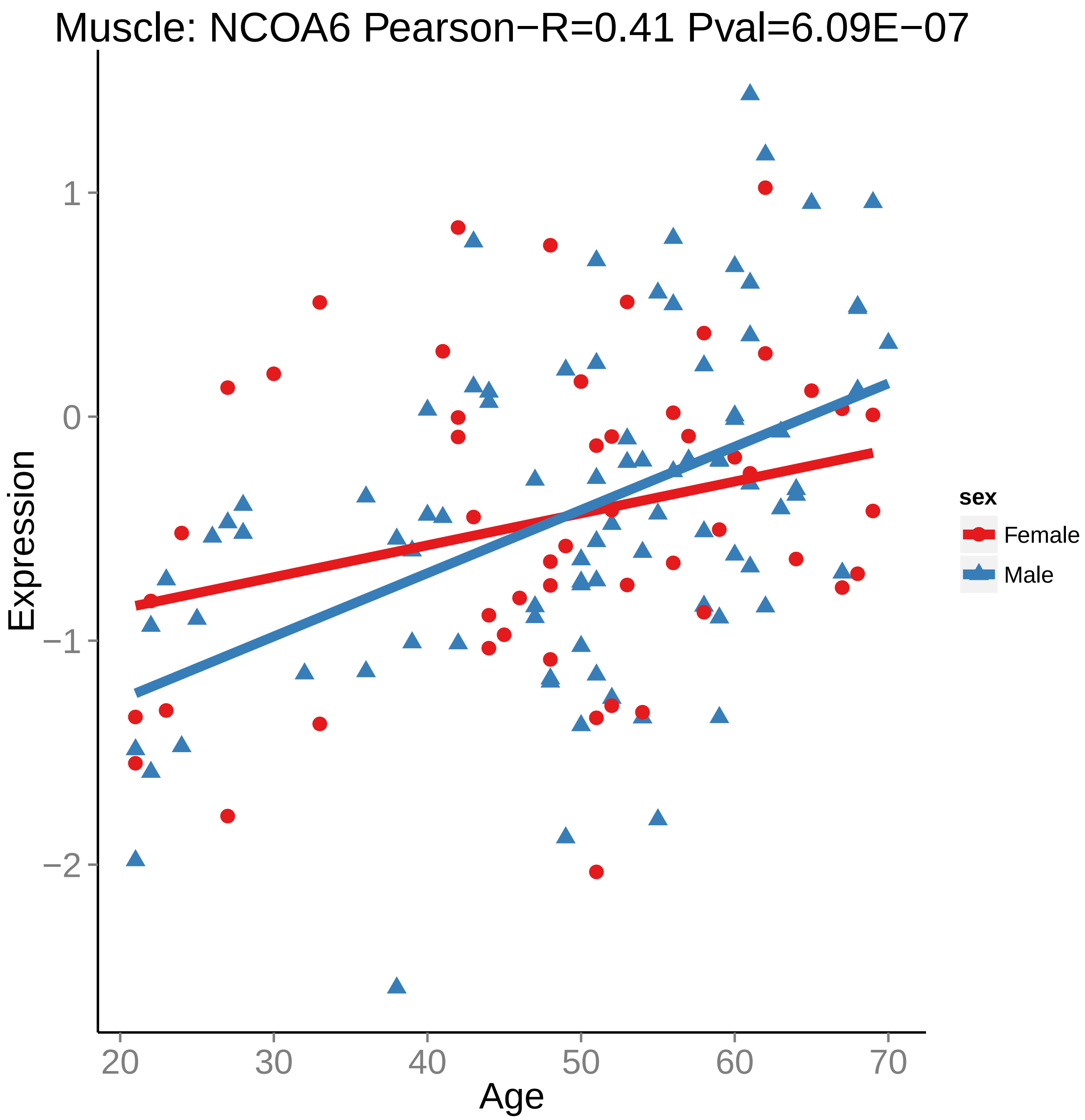

Muscle: NCOA6 Pearson−R=0.41 Pval=6.09E−07
Expression
1
0
−1
−2
20
30
40
50
60
70
Age
sex
Female
Male

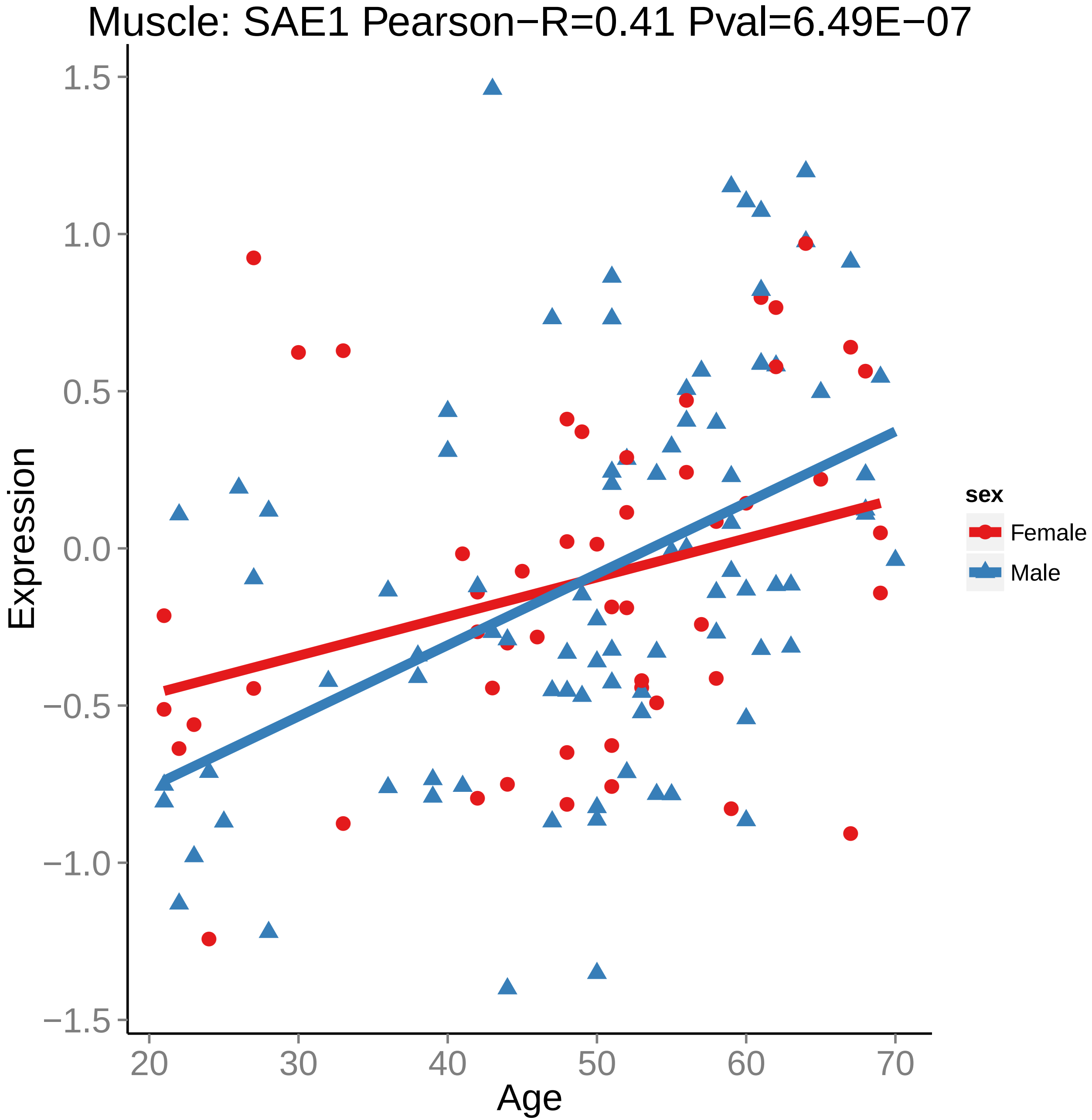

Muscle: SAE1 Pearson−R=0.41 Pval=6.49E−07
Expression
1.5
1.0
0.5
0.0
−0.5
−1.0
−1.5
20
30
40
50
60
70
Age
sex
Female
Male

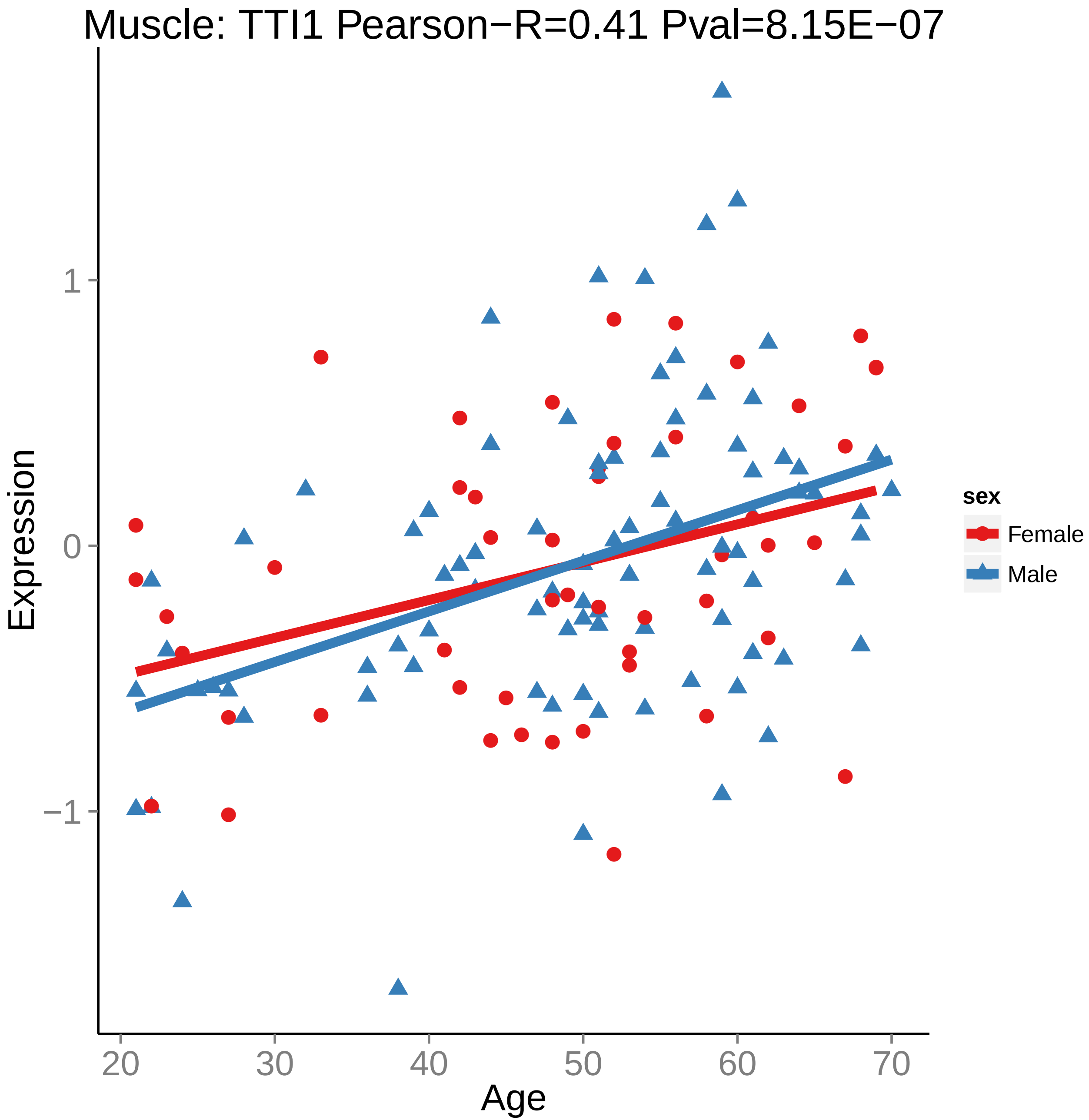
Muscle: TTI1 Pearson−R=0.41 Pval=8.15E−07
Expression
Age
1
0
−1
20
30
40
50
60
70
sex
Female
Male

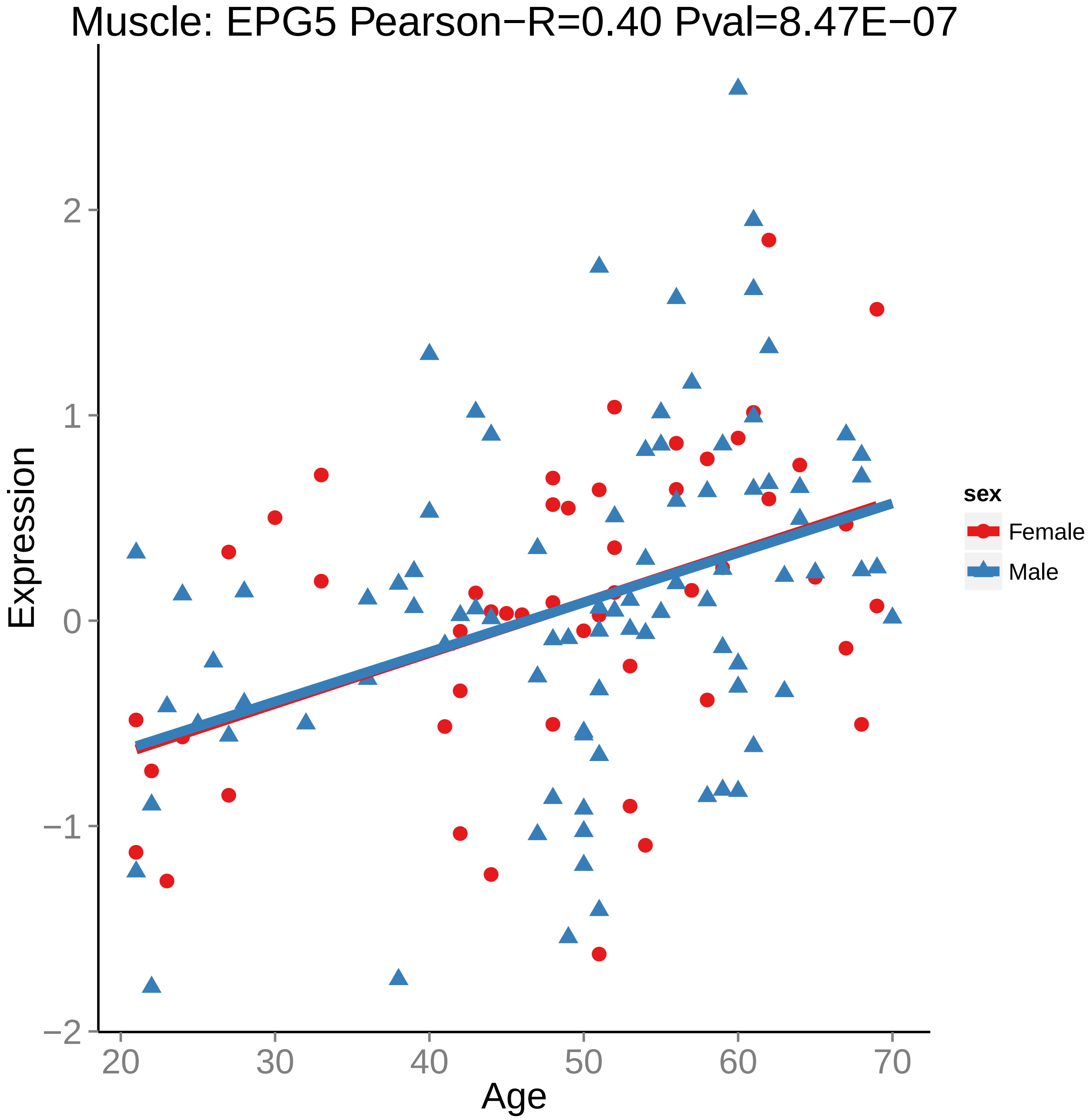

Muscle: EPG5 Pearson−R=0.40 Pval=8.47E−07
Expression
Age
sex
Female
Male
2
1
0
−1
−2
20
30
40
50
60
70

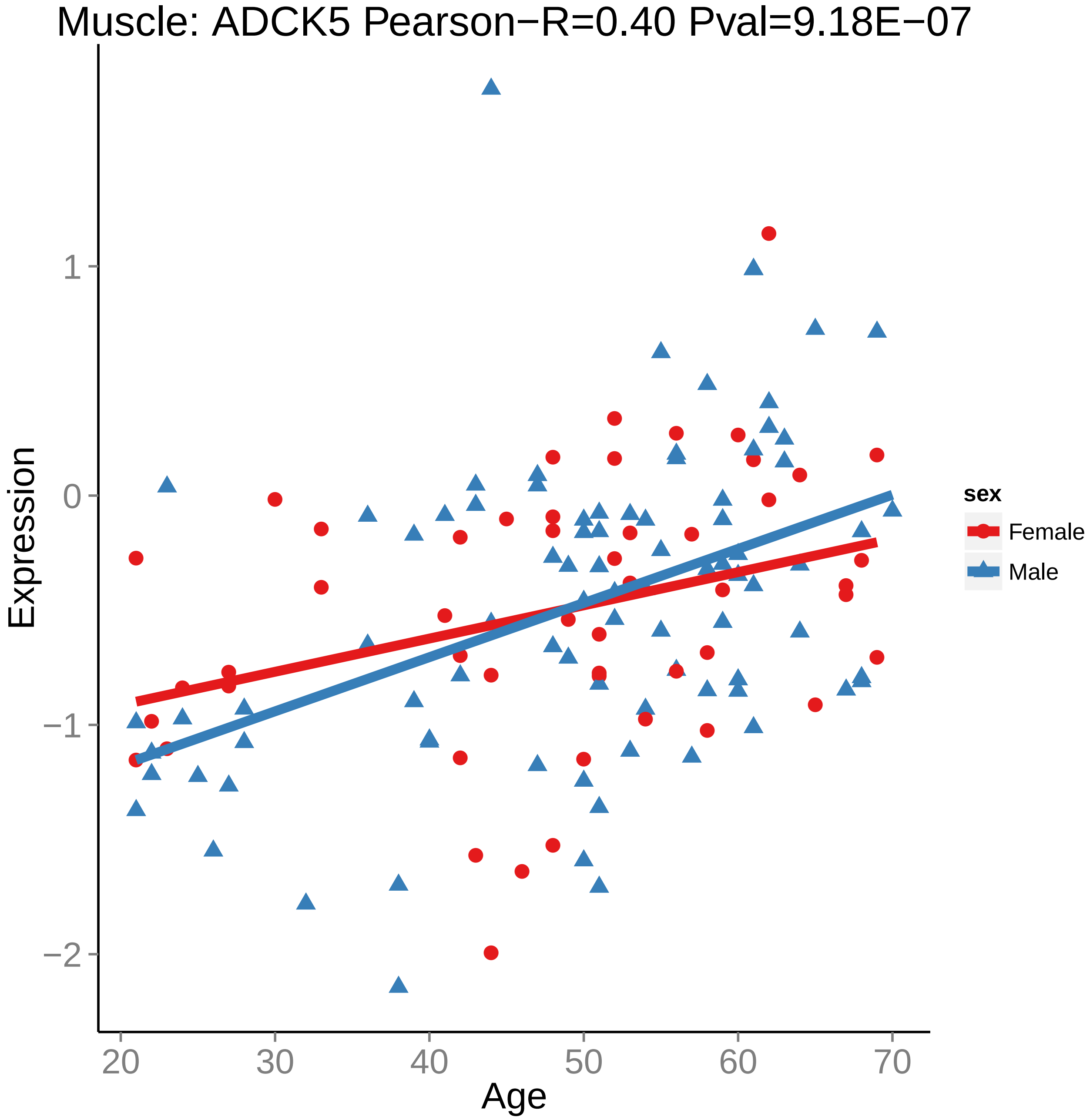

Muscle: ADCK5 Pearson−R=0.40 Pval=9.18E−07
Expression
Age
1
0
−1
−2
20
30
40
50
60
70
sex
Female
Male

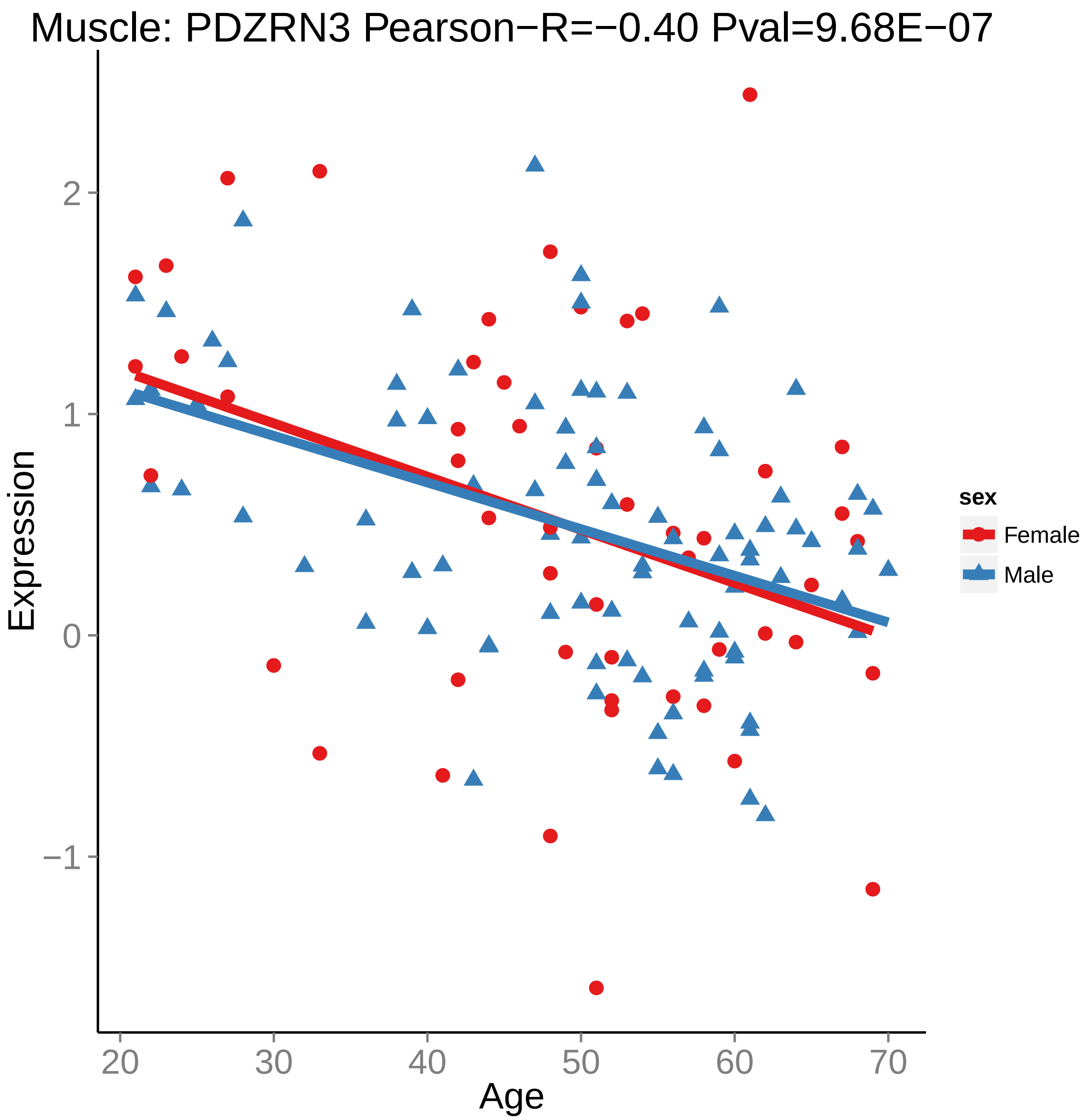
Muscle: PDZRN3 Pearson−R=−0.40 Pval=9.68E−07
Expression
Age
2
1
0
−1
20
30
40
50
60
70
sex
Female
Male

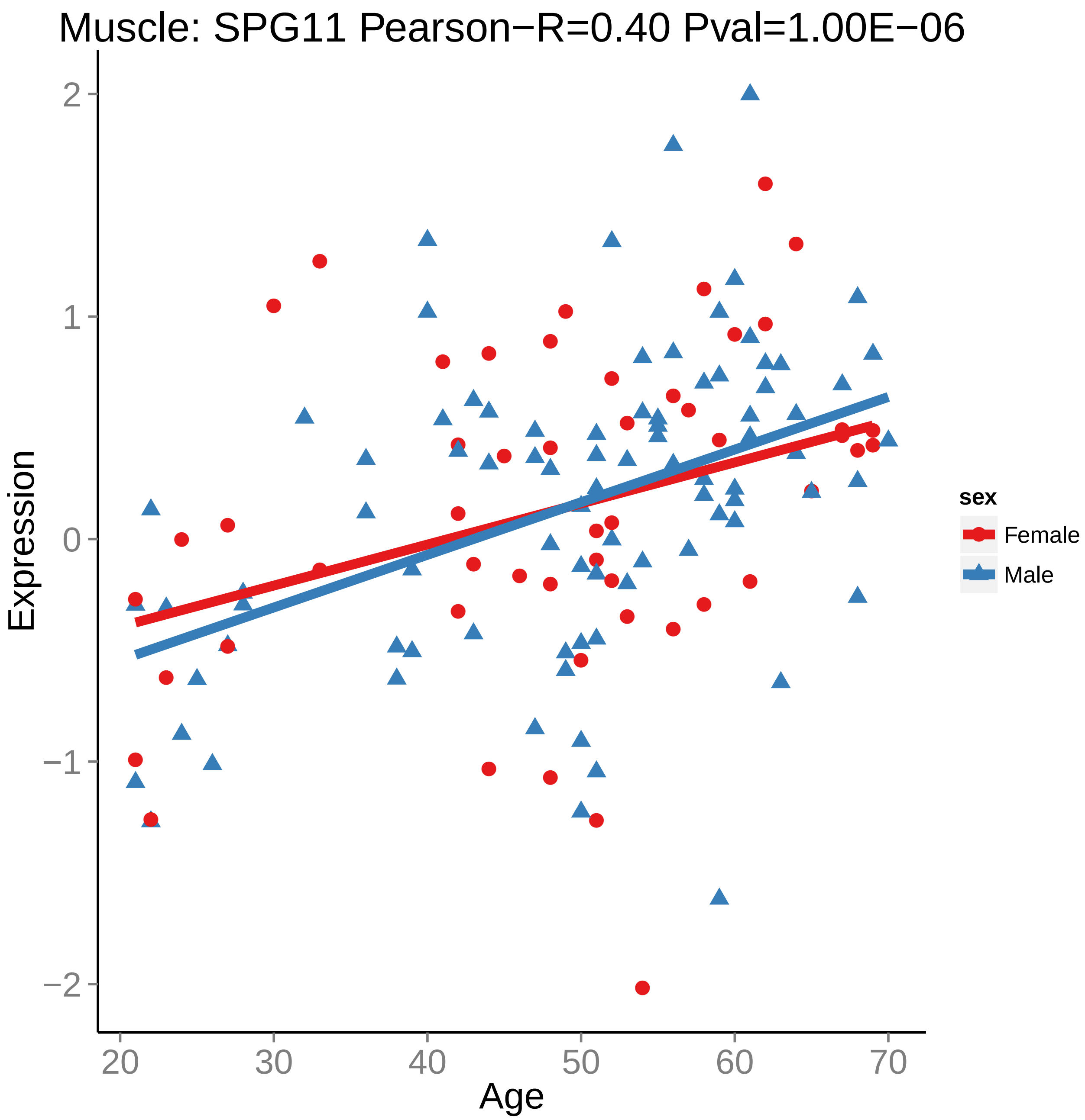

Muscle: SPG11 Pearson−R=0.40 Pval=1.00E−06
Expression
2
1
0
−1
−2
20
30
40
50
60
70
Age
sex
Female
Male

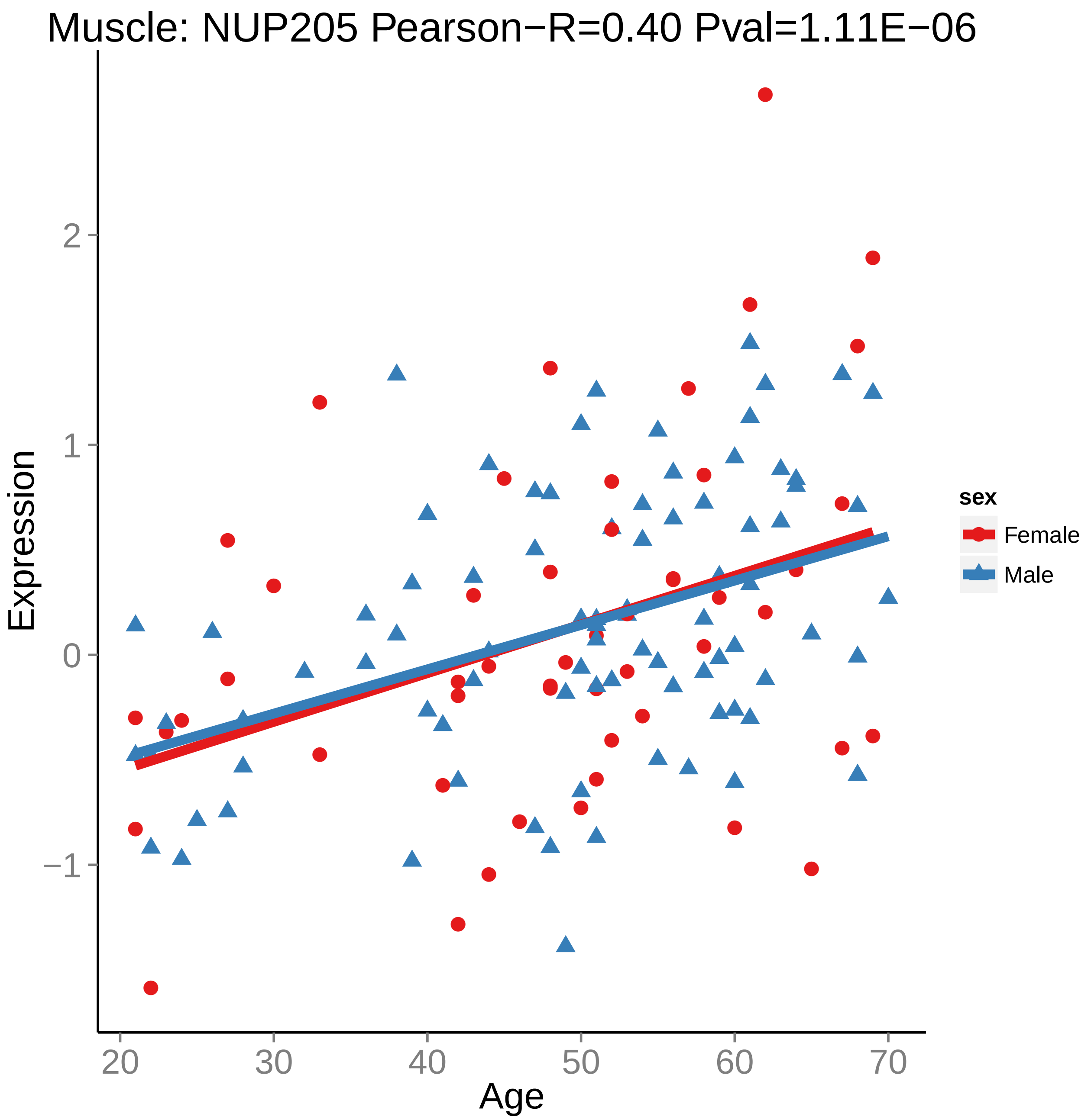

Muscle: NUP205 Pearson−R=0.40 Pval=1.11E−06
Expression
2
1
0
−1
20
30
40
50
60
70
Age
sex
Female
Male

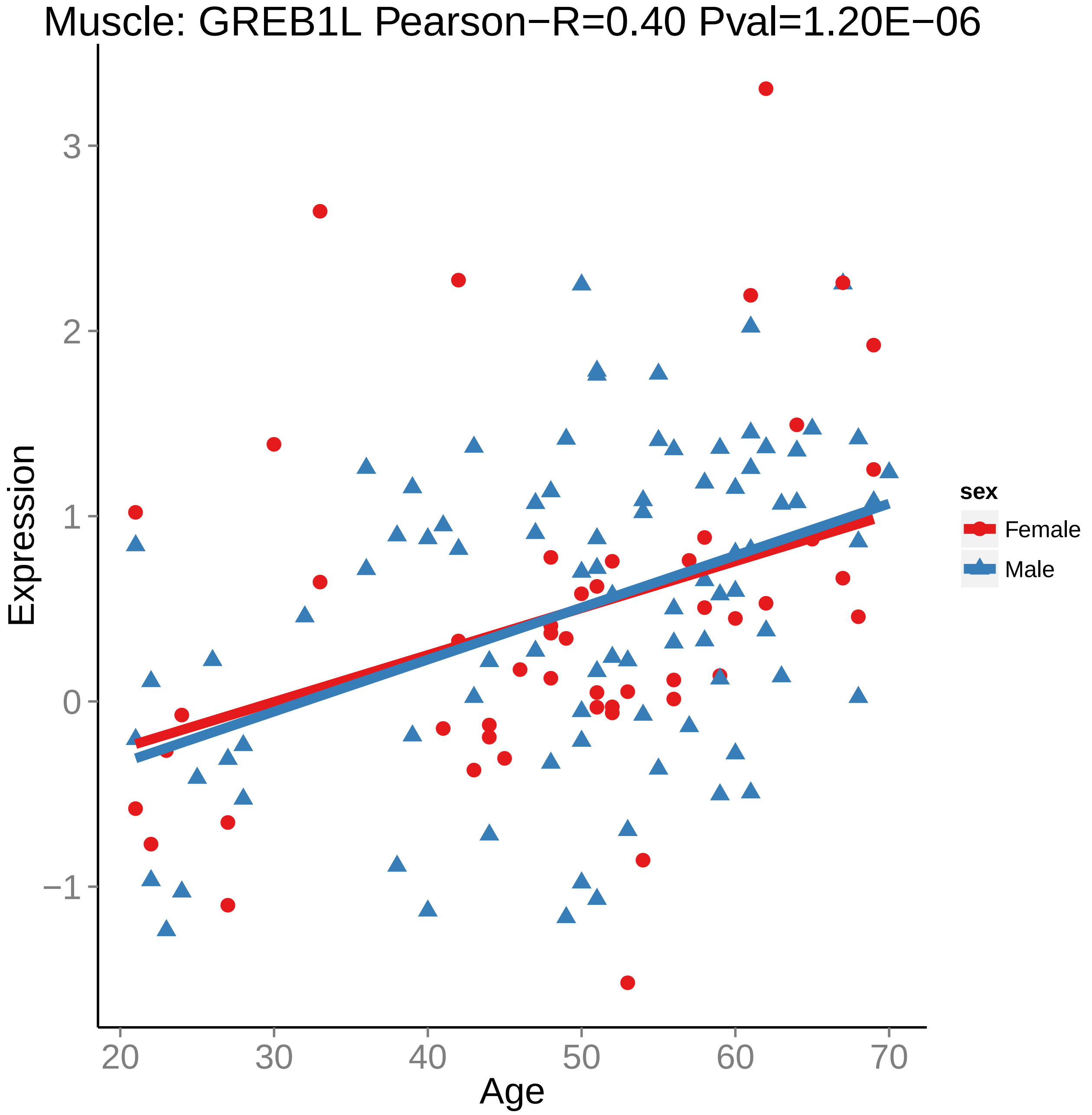

Muscle: GREB1L Pearson−R=0.40 Pval=1.20E−06
Expression
3
2
1
0
−1
20
30
40
50
60
70
Age
sex
Female
Male

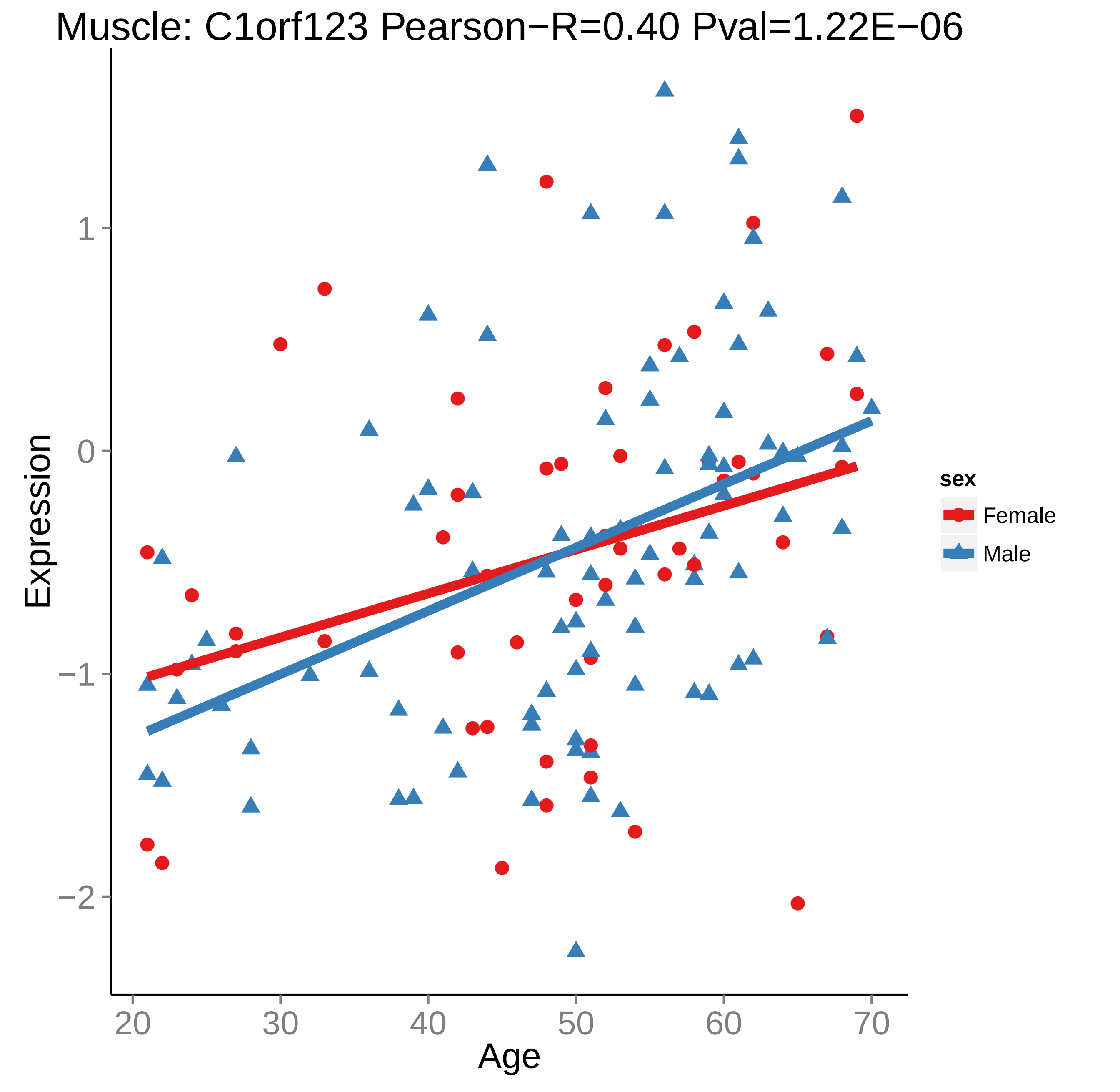

Muscle: C1orf123 Pearson−R=0.40 Pval=1.22E−06
Expression
1
0
−1
−2
20
30
40
50
60
70
Age
sex
Female
Male

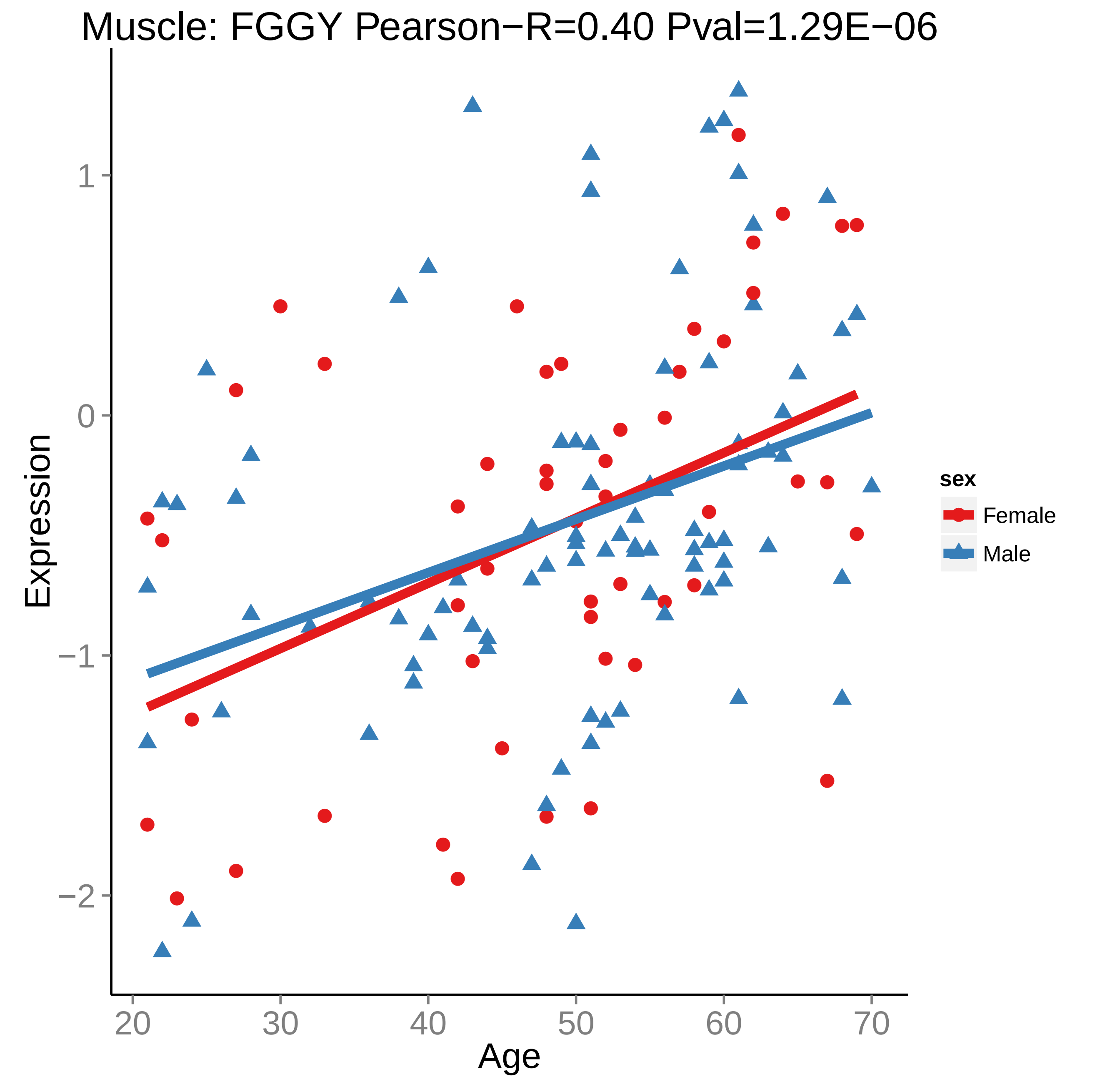

Muscle: FGGY Pearson−R=0.40 Pval=1.29E−06
Expression
1
0
−1
−2
20
30
40
50
60
70
Age
sex
Female
Male

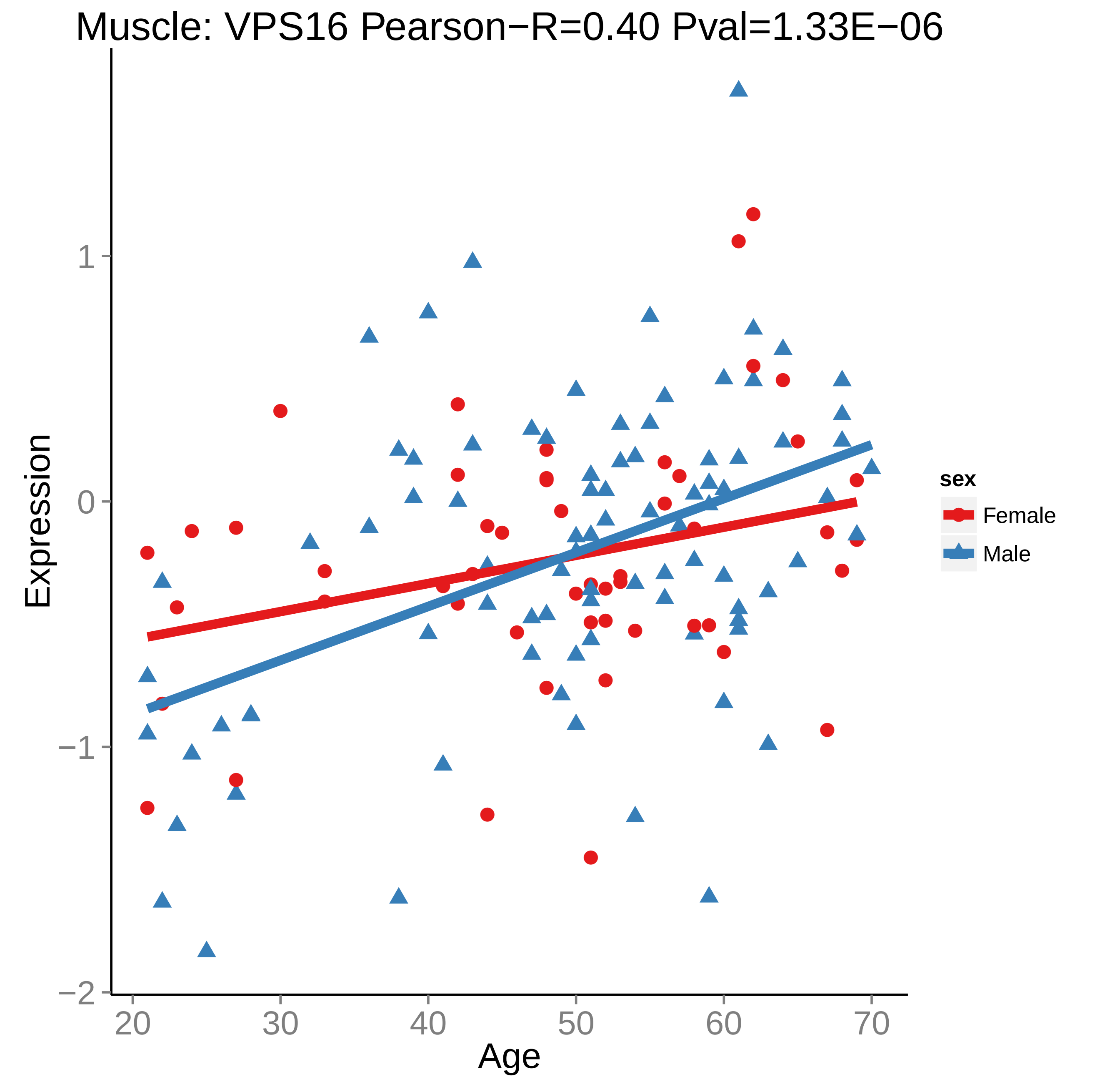

Muscle: VPS16 Pearson−R=0.40 Pval=1.33E−06
Expression
Age
1
0
−1
−2
20
30
40
50
60
70
sex
Female
Male

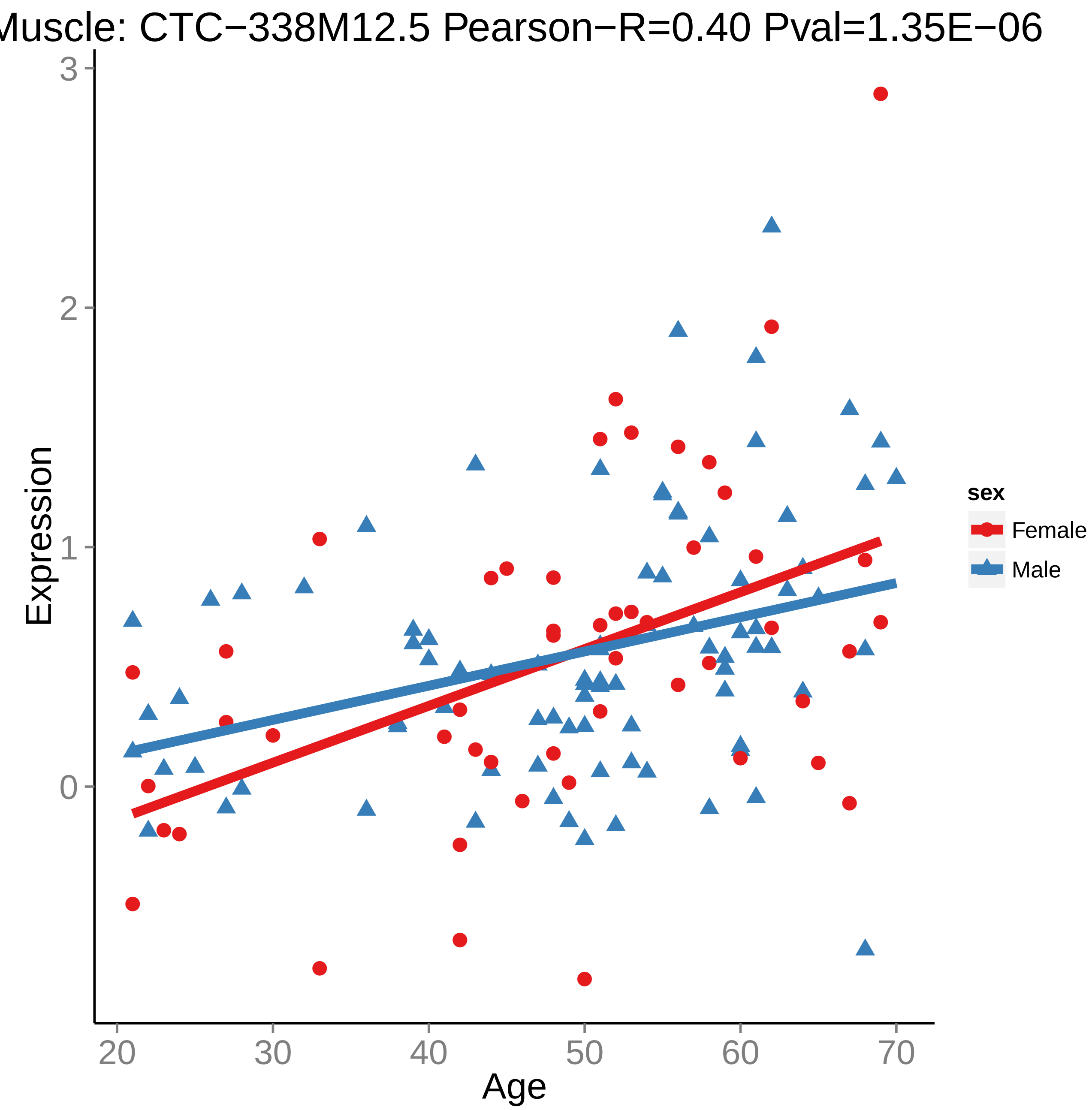

Muscle: CTC−338M12.5 Pearson−R=0.40 Pval=1.35E−06
Expression
3
2
1
0
20
30
40
50
60
70
Age
sex
Female
Male

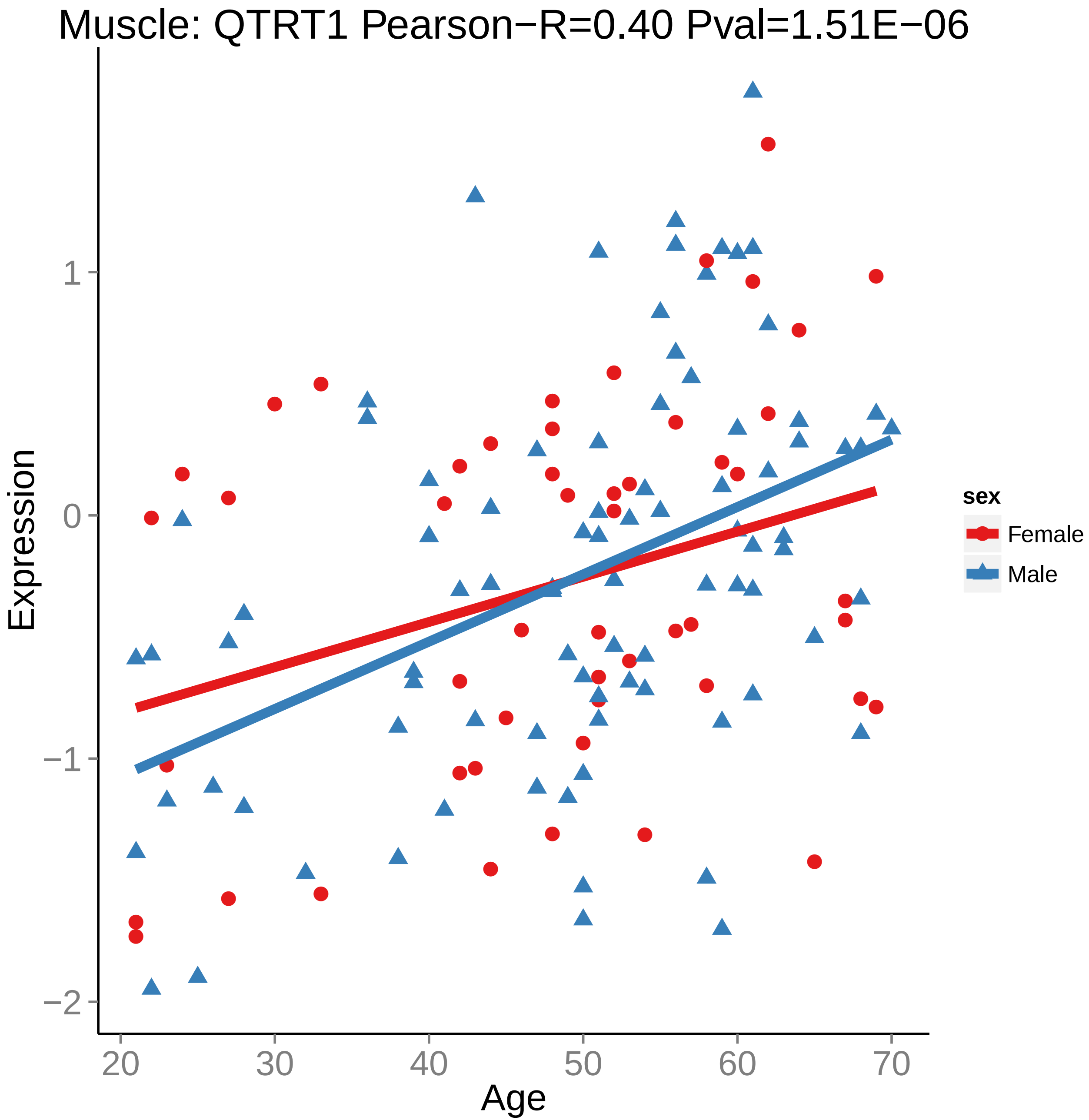

Muscle: QTRT1 Pearson−R=0.40 Pval=1.51E−06
Expression
Age
1
0
−1
−2
20
30
40
50
60
70
sex
Female
Male

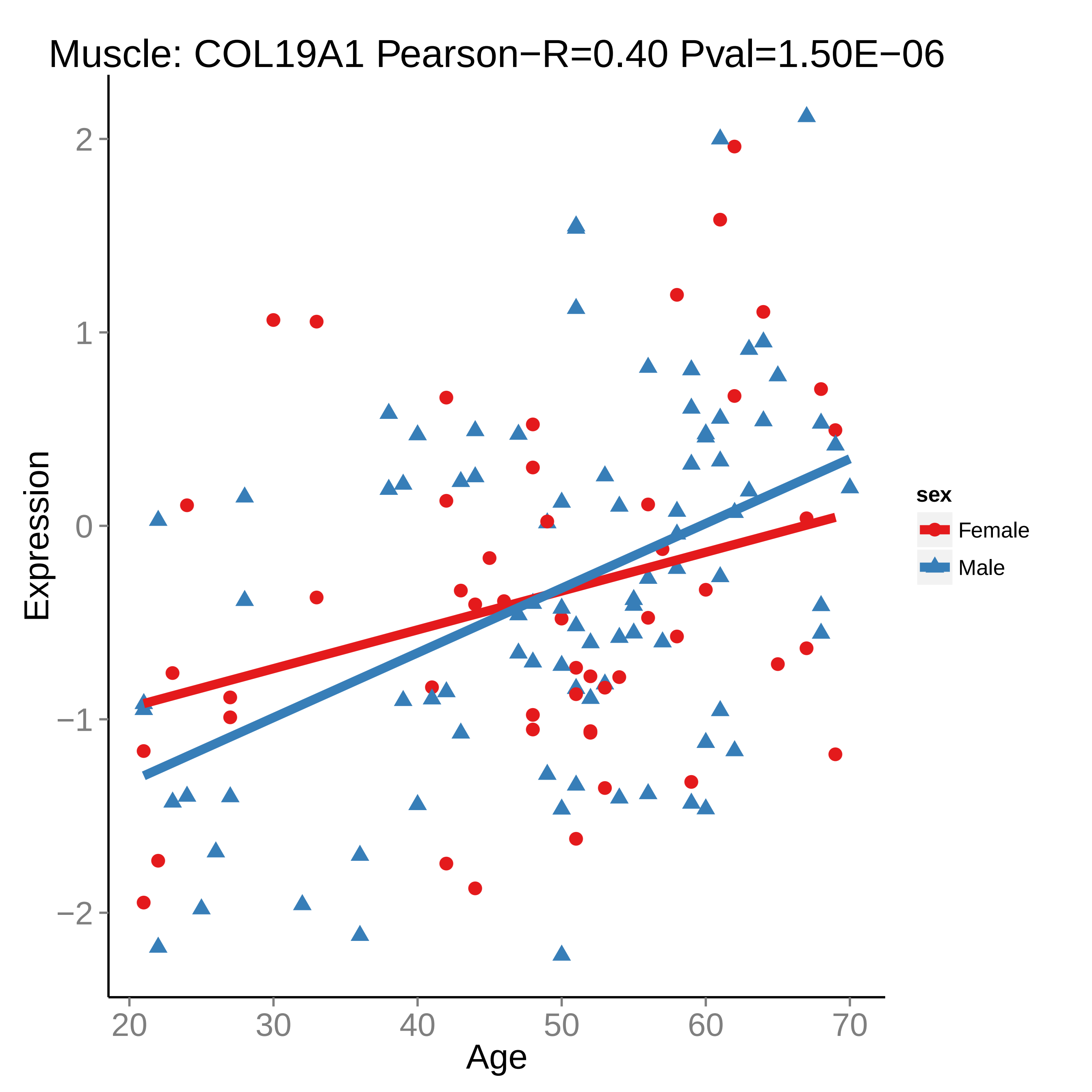

Muscle: COL19A1 Pearson−R=0.40 Pval=1.50E−06
Expression
Age
2
1
0
−1
−2
20
30
40
50
60
70
sex
Female
Male

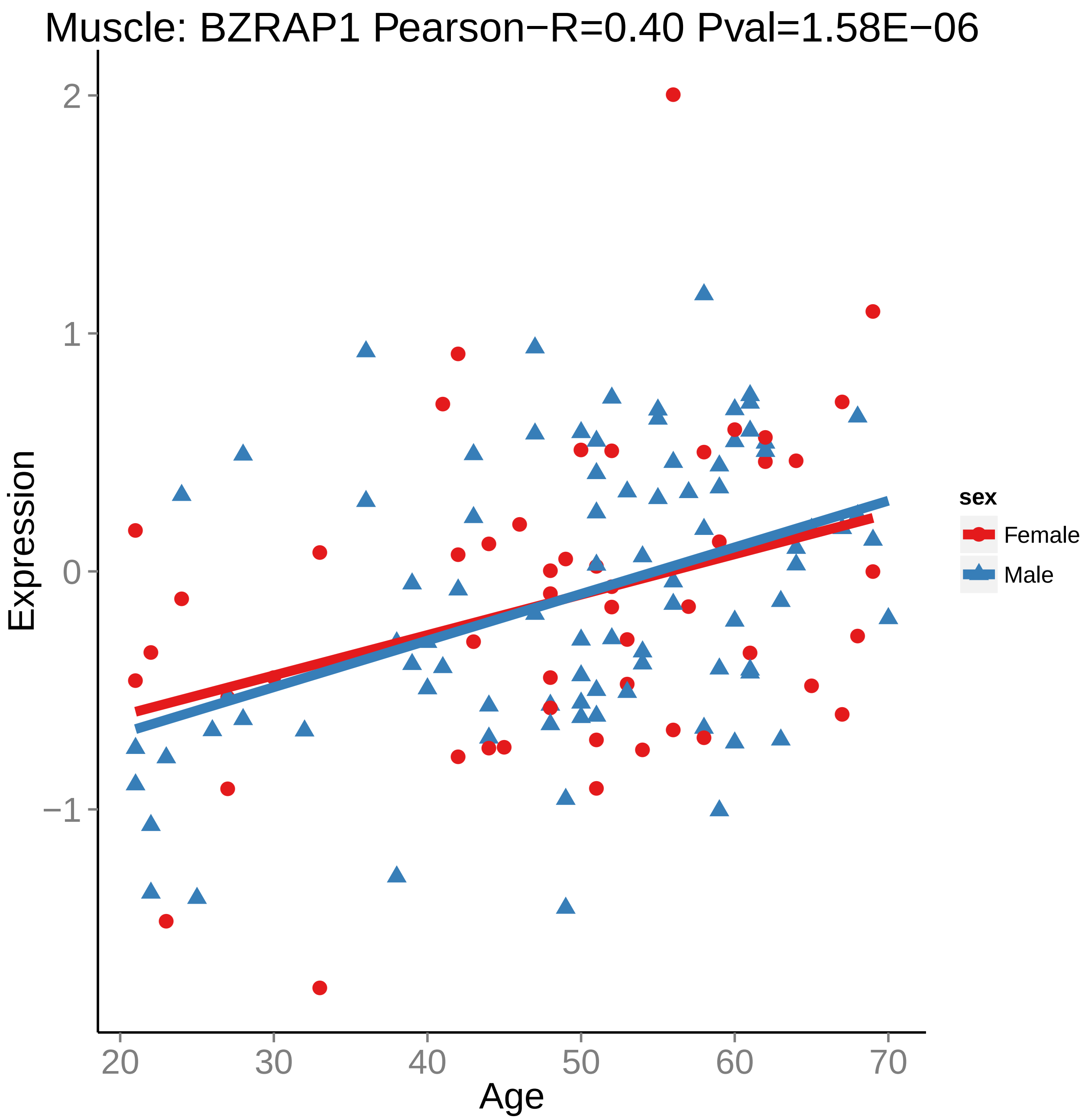

Muscle: BZRAP1 Pearson−R=0.40 Pval=1.58E−06
Expression
Age
2
1
0
−1
20
30
40
50
60
70
sex
Female
Male

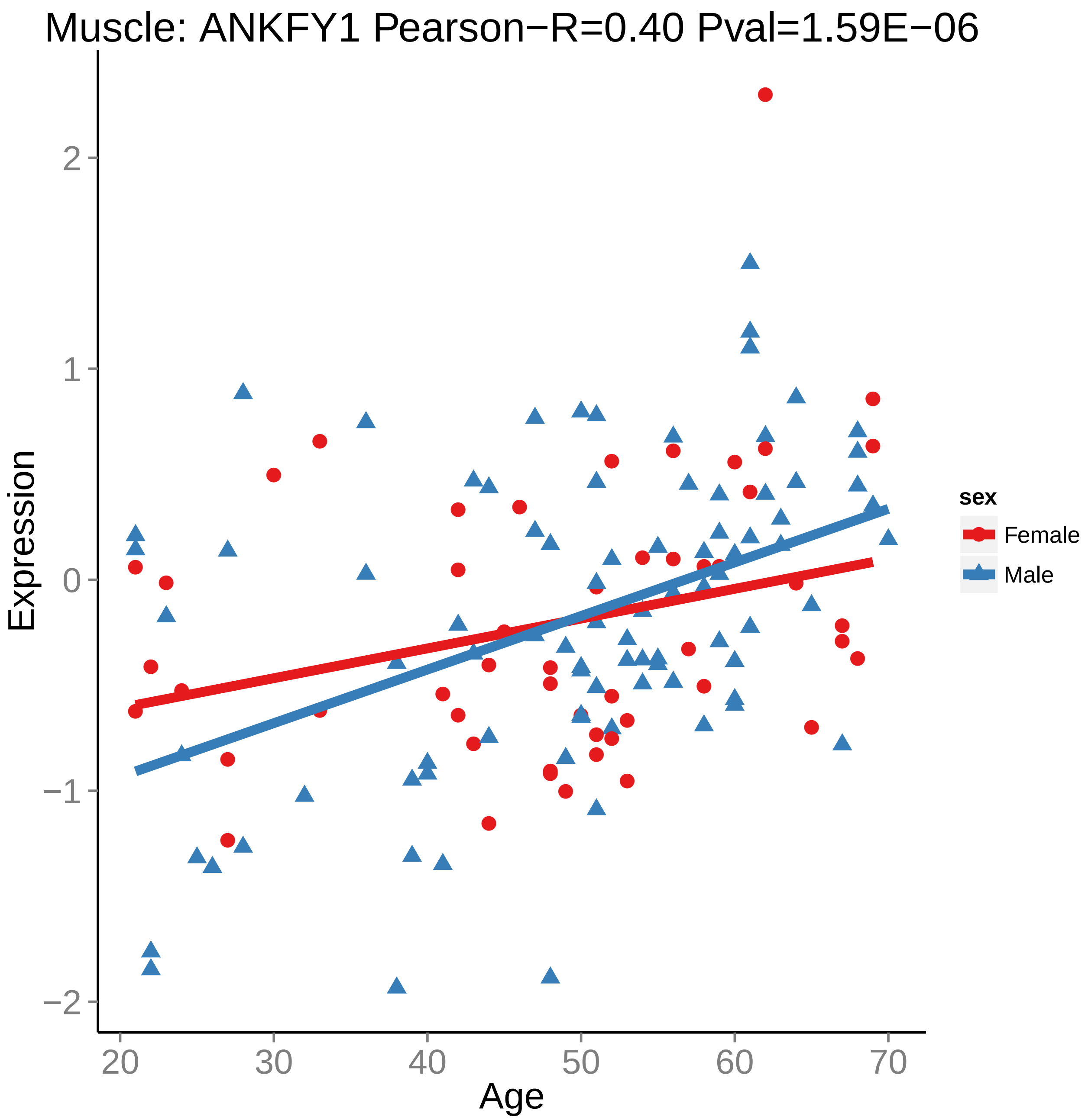

Muscle: ANKFY1 Pearson−R=0.40 Pval=1.59E−06
Expression
2
1
0
−1
−2
20
30
40
50
60
70
Age
sex
Female
Male

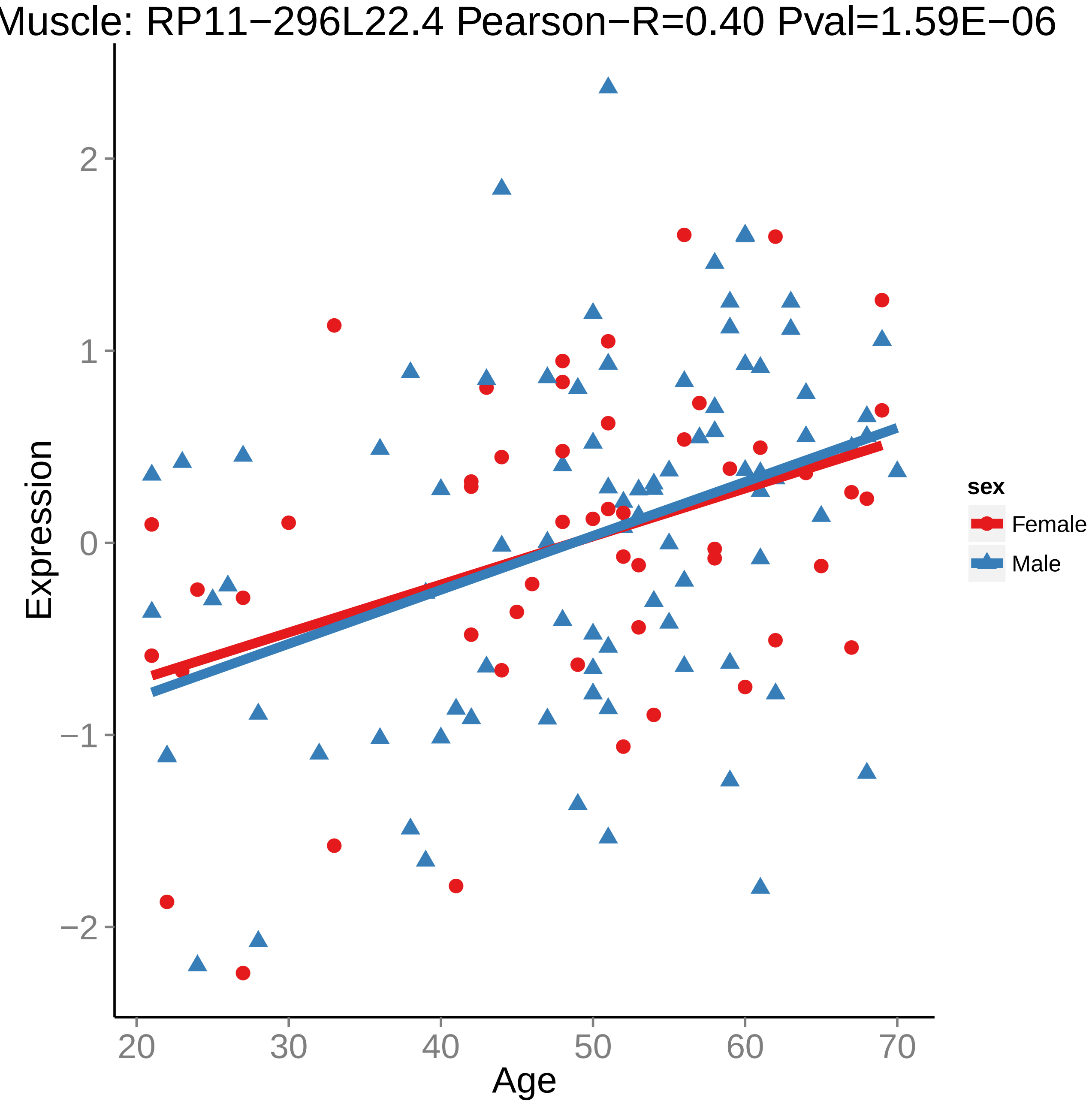

Muscle: RP11−296L22.4 Pearson−R=0.40 Pval=1.59E−06
Expression
2
1
0
−1
−2
20
30
40
50
60
70
Age
sex
Female
Male

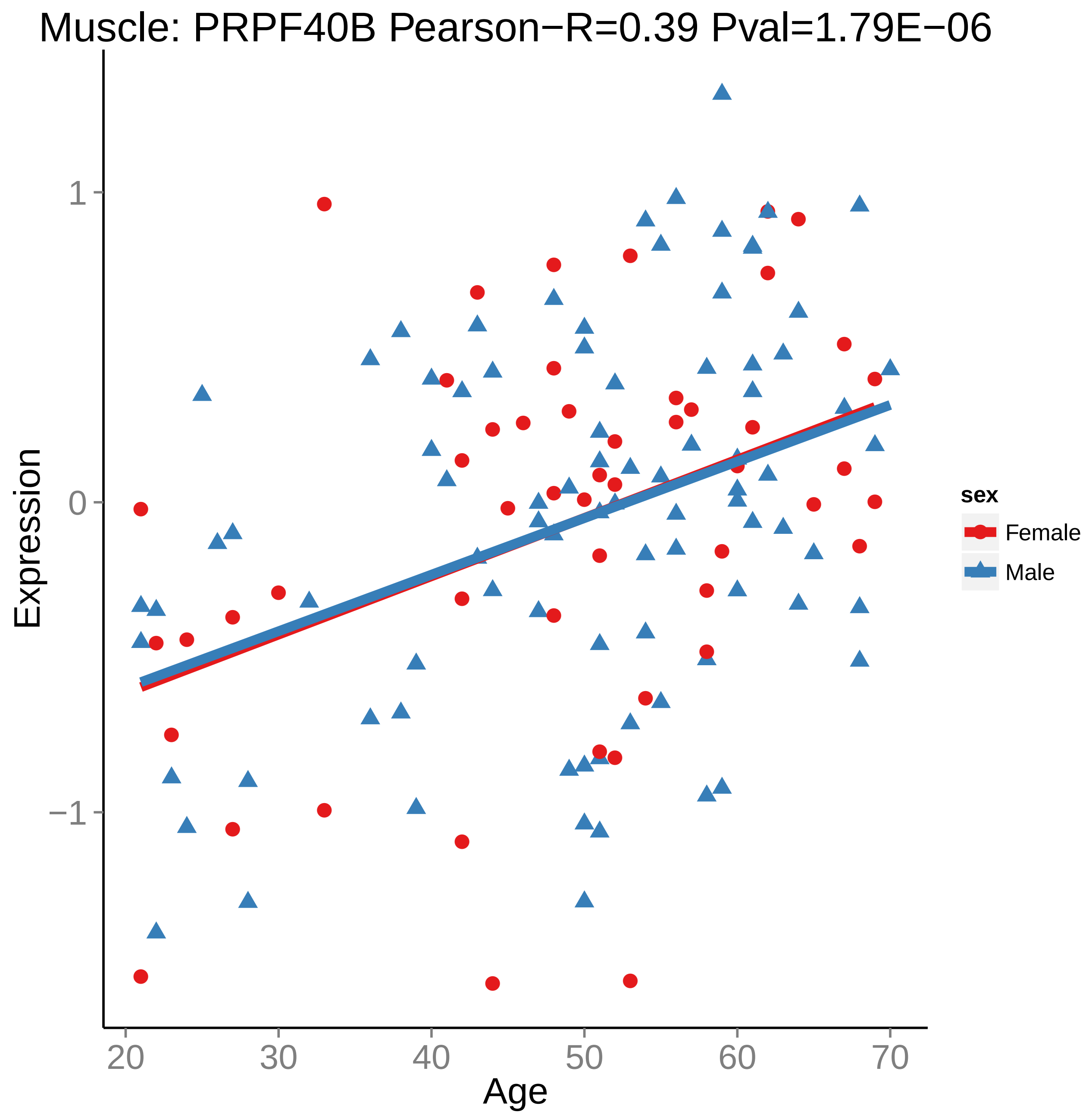
Muscle: PRPF40B Pearson−R=0.39 Pval=1.79E−06
Expression
1
0
−1
20
30
40
50
60
70
Age
sex
Female
Male

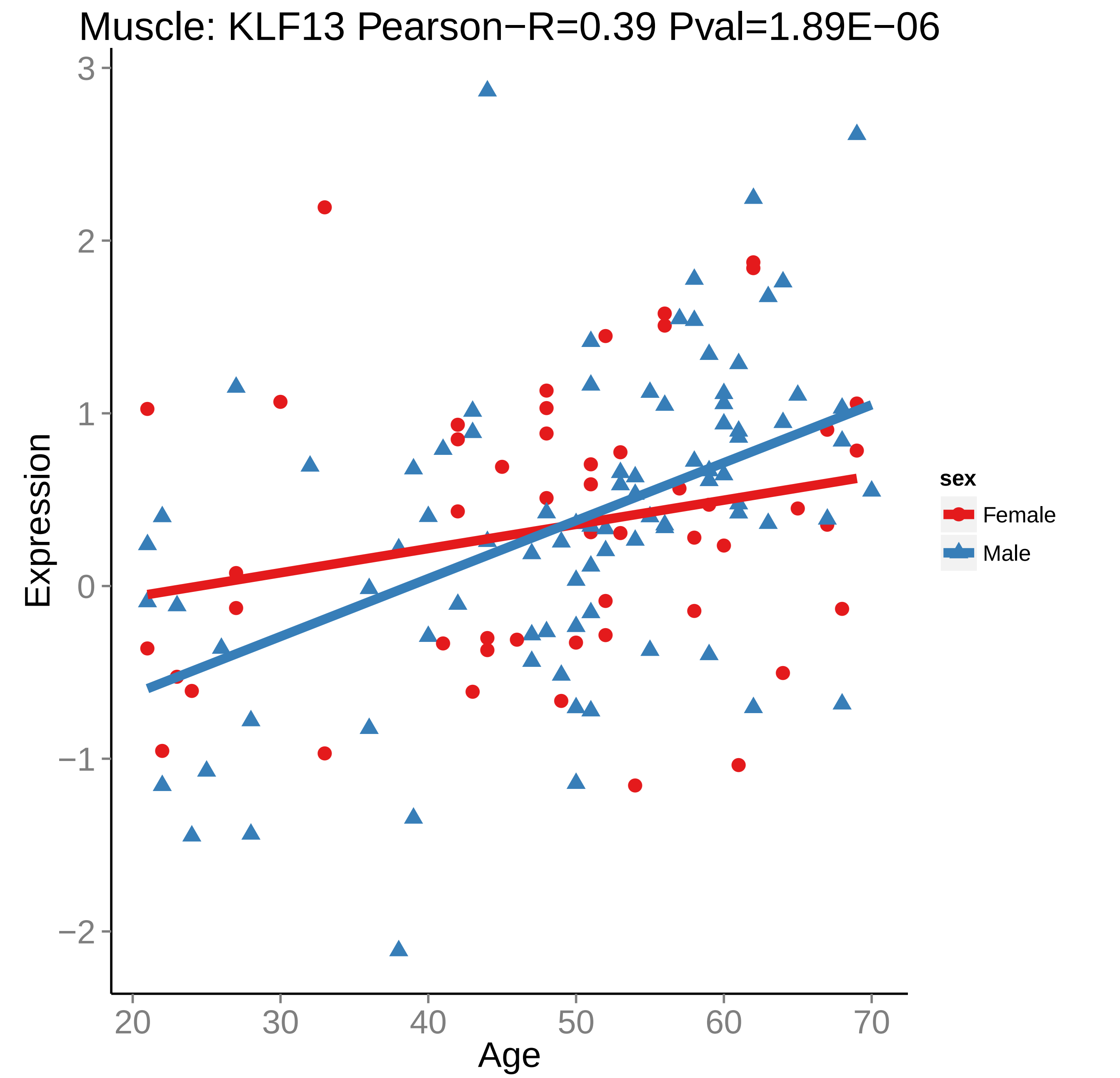

Muscle: KLF13 Pearson−R=0.39 Pval=1.89E−06
Expression
Age
3
2
1
0
−1
−2
20
30
40
50
60
70
sex
Female
Male

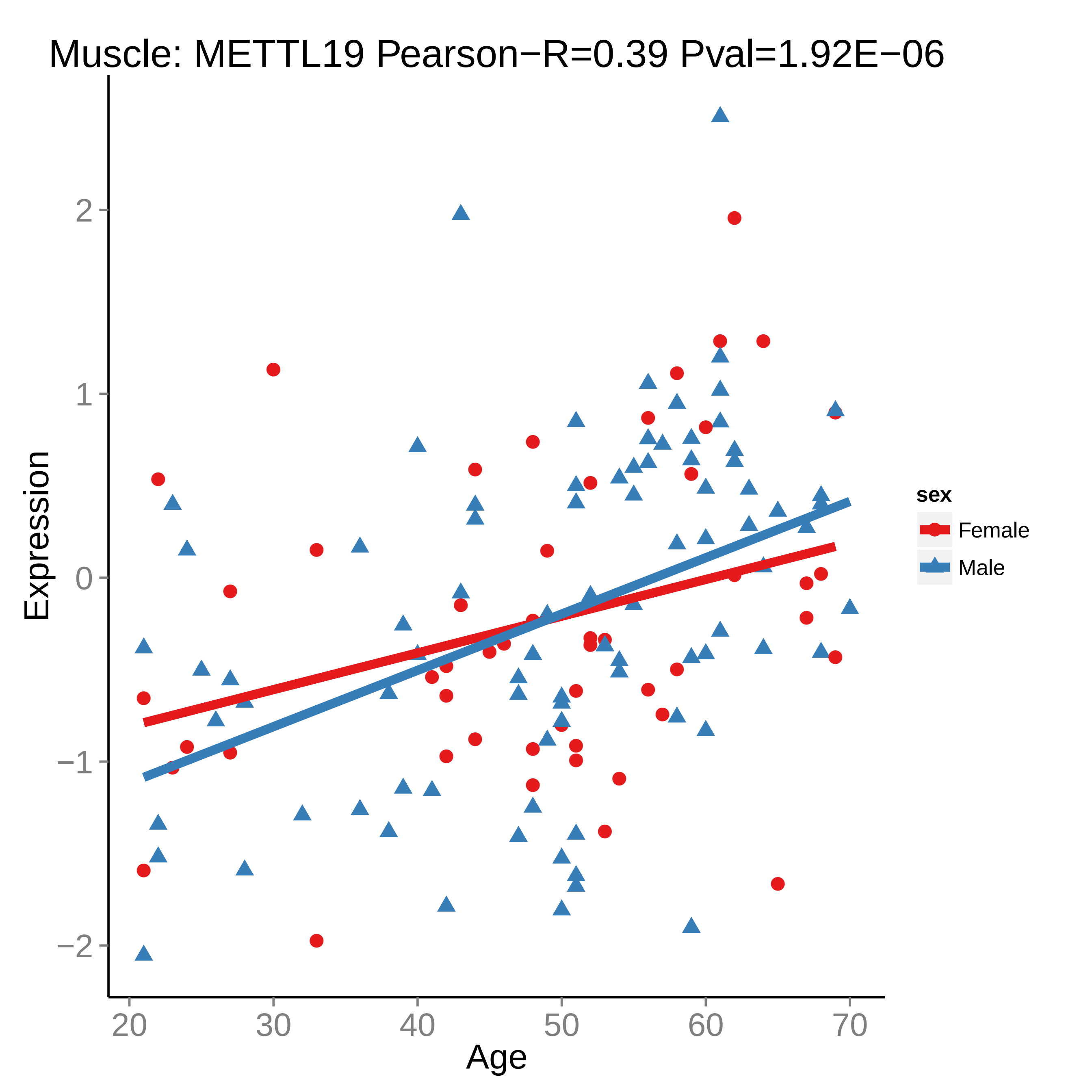
Muscle: METTL19 Pearson−R=0.39 Pval=1.92E−06
Expression
2
1
0
−1
−2
20
30
40
50
60
70
Age
sex
Female
Male

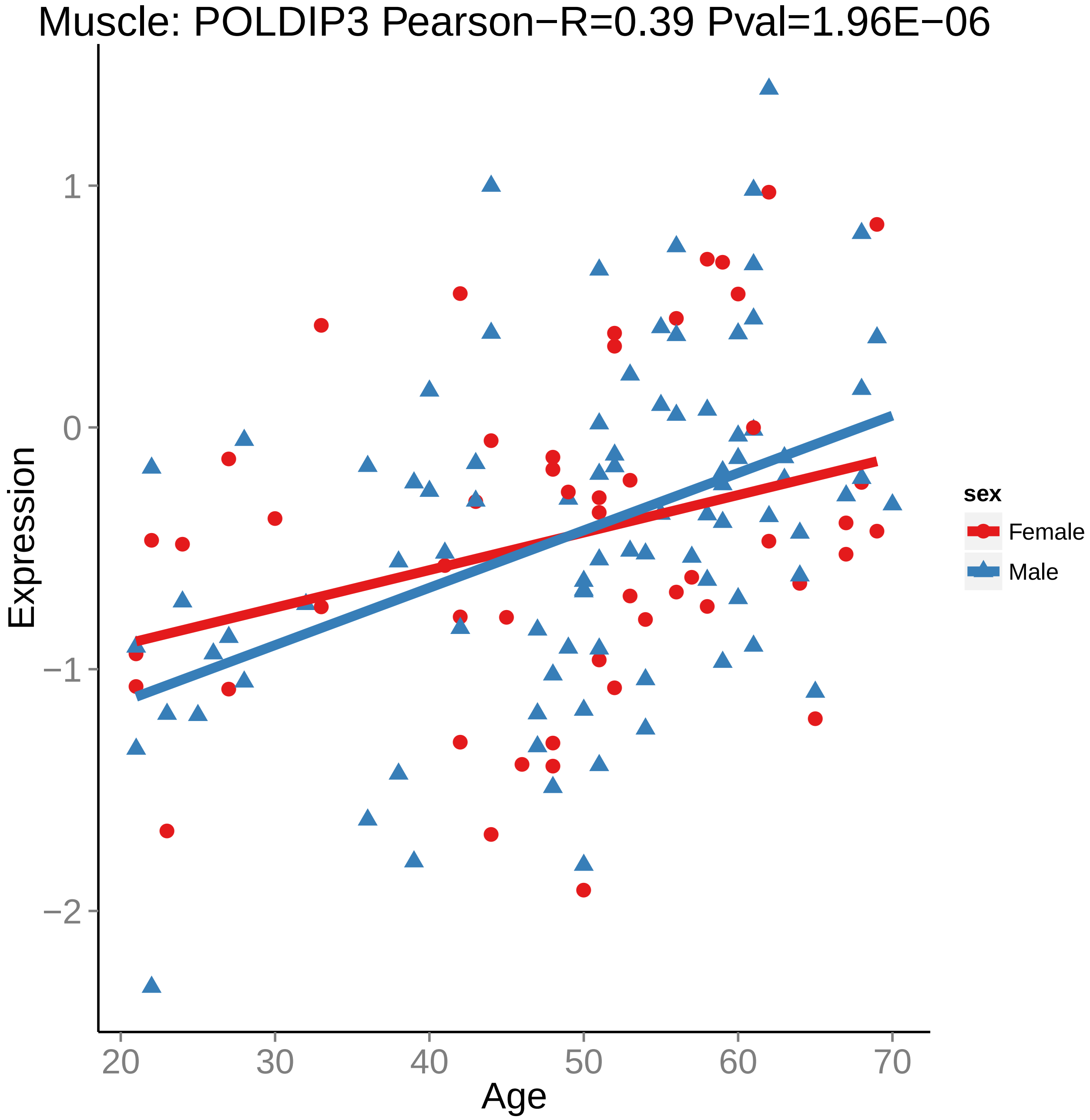

Muscle: POLDIP3 Pearson−R=0.39 Pval=1.96E−06
Expression
1
0
−1
−2
20
30
40
50
60
70
Age
sex
Female
Male

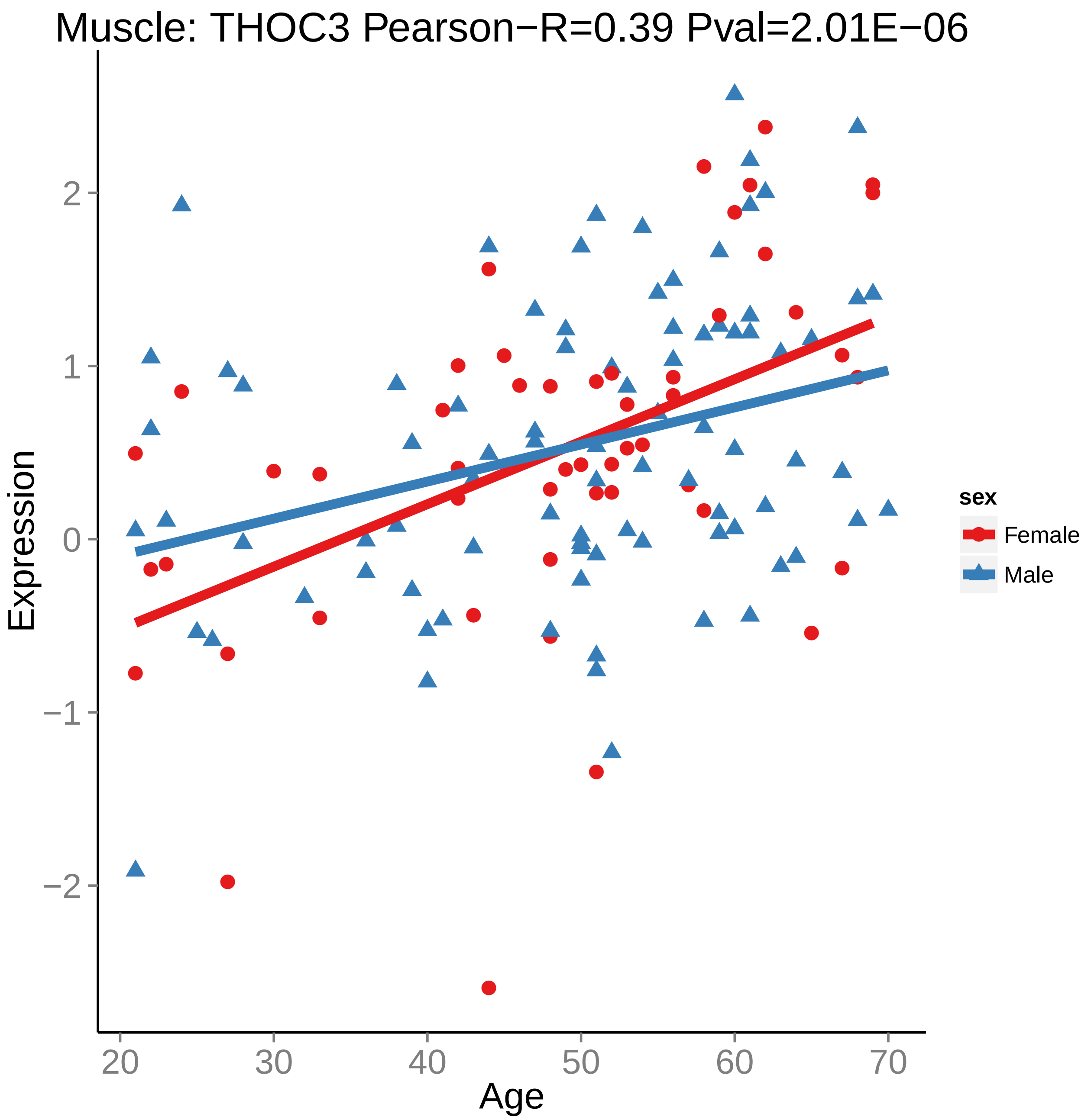
Muscle: THOC3 Pearson−R=0.39 Pval=2.01E−06
Expression
2
1
0
−1
−2
20
30
40
50
60
70
Age
sex
Female
Male

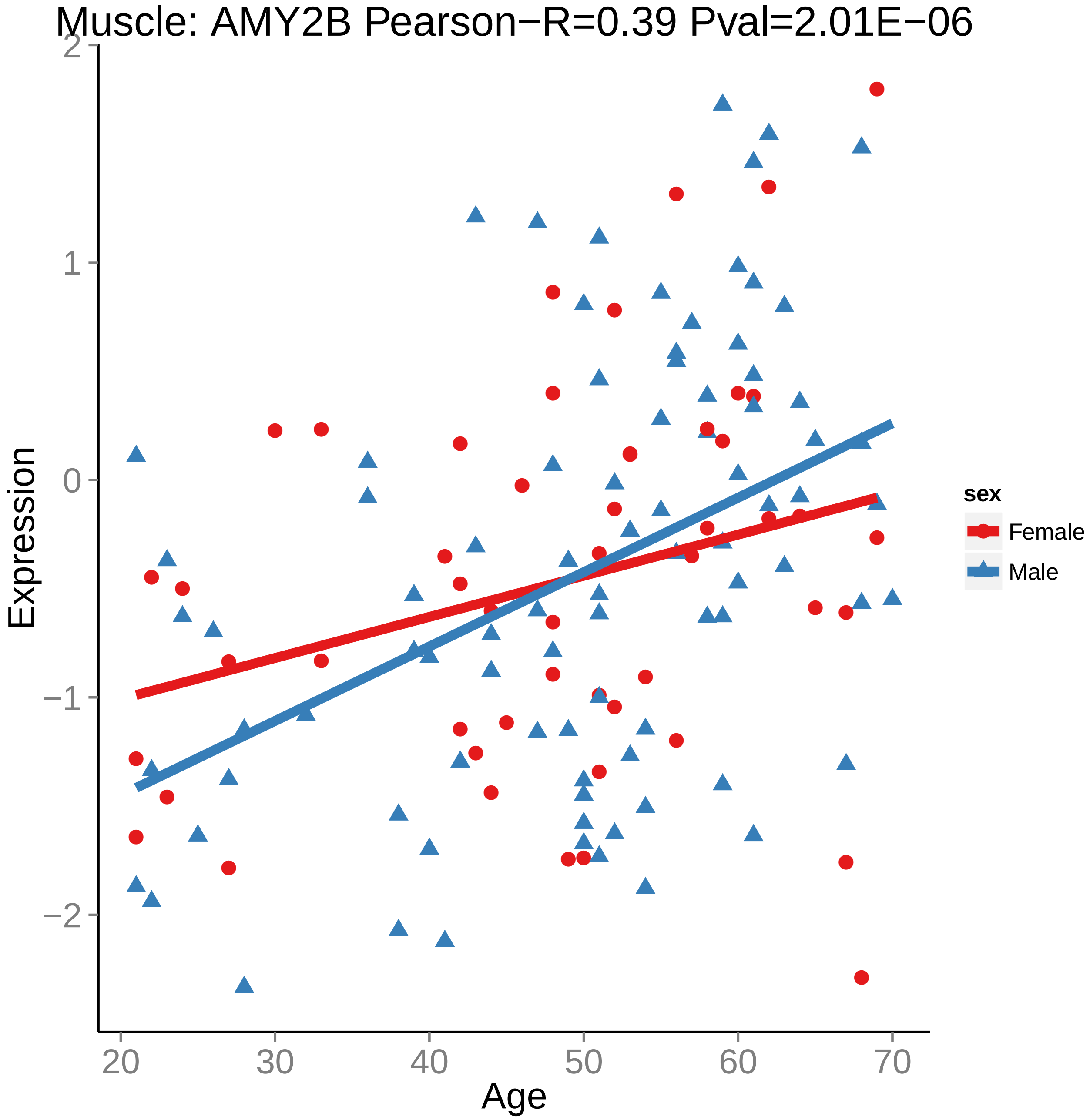

Muscle: AMY2B Pearson−R=0.39 Pval=2.01E−06
Expression
2
1
0
−1
−2
20
30
40
50
60
70
Age
sex
Female
Male

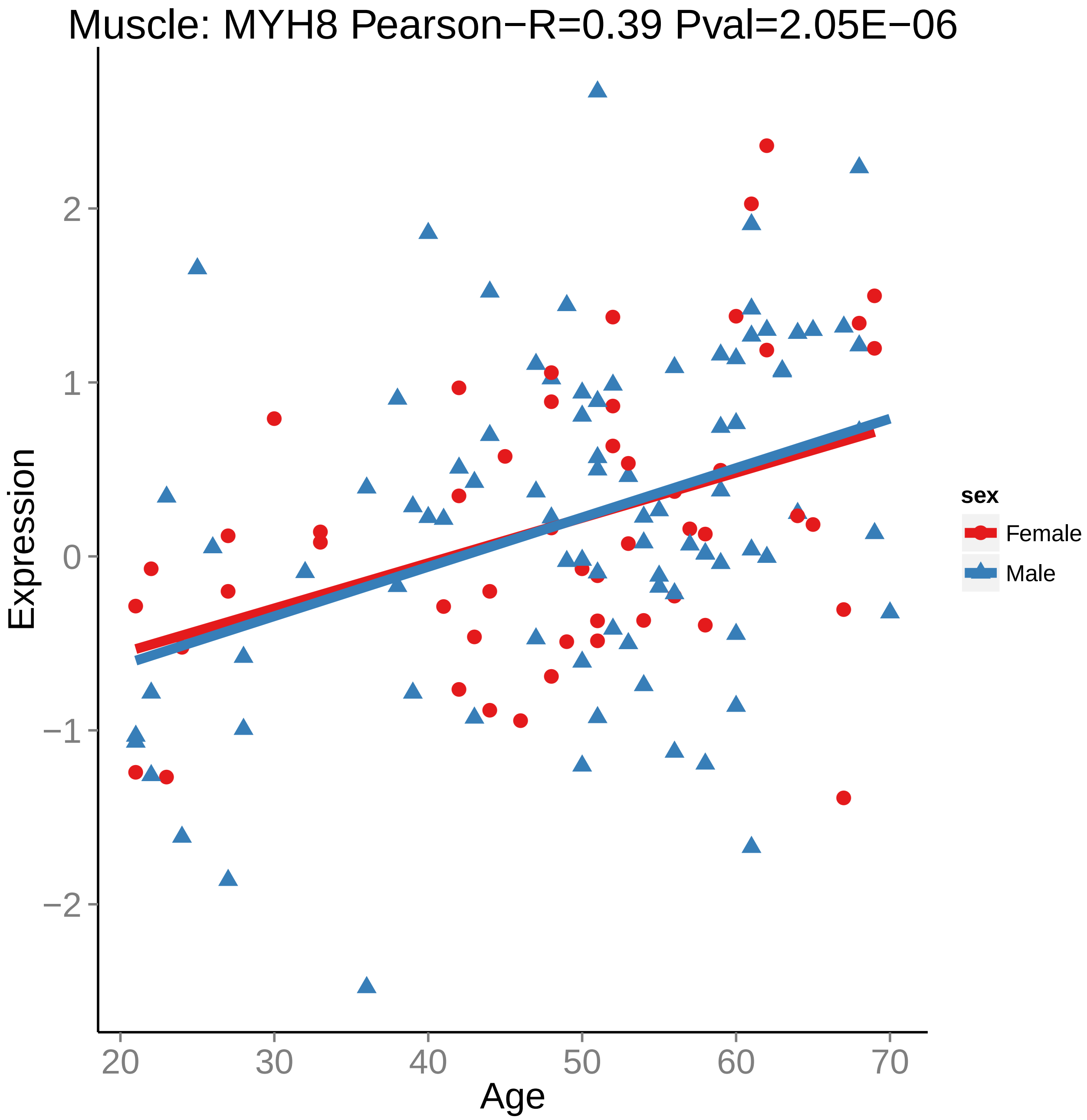

Muscle: MYH8 Pearson−R=0.39 Pval=2.05E−06
Expression
Age
2
1
0
−1
−2
20
30
40
50
60
70
sex
Female
Male

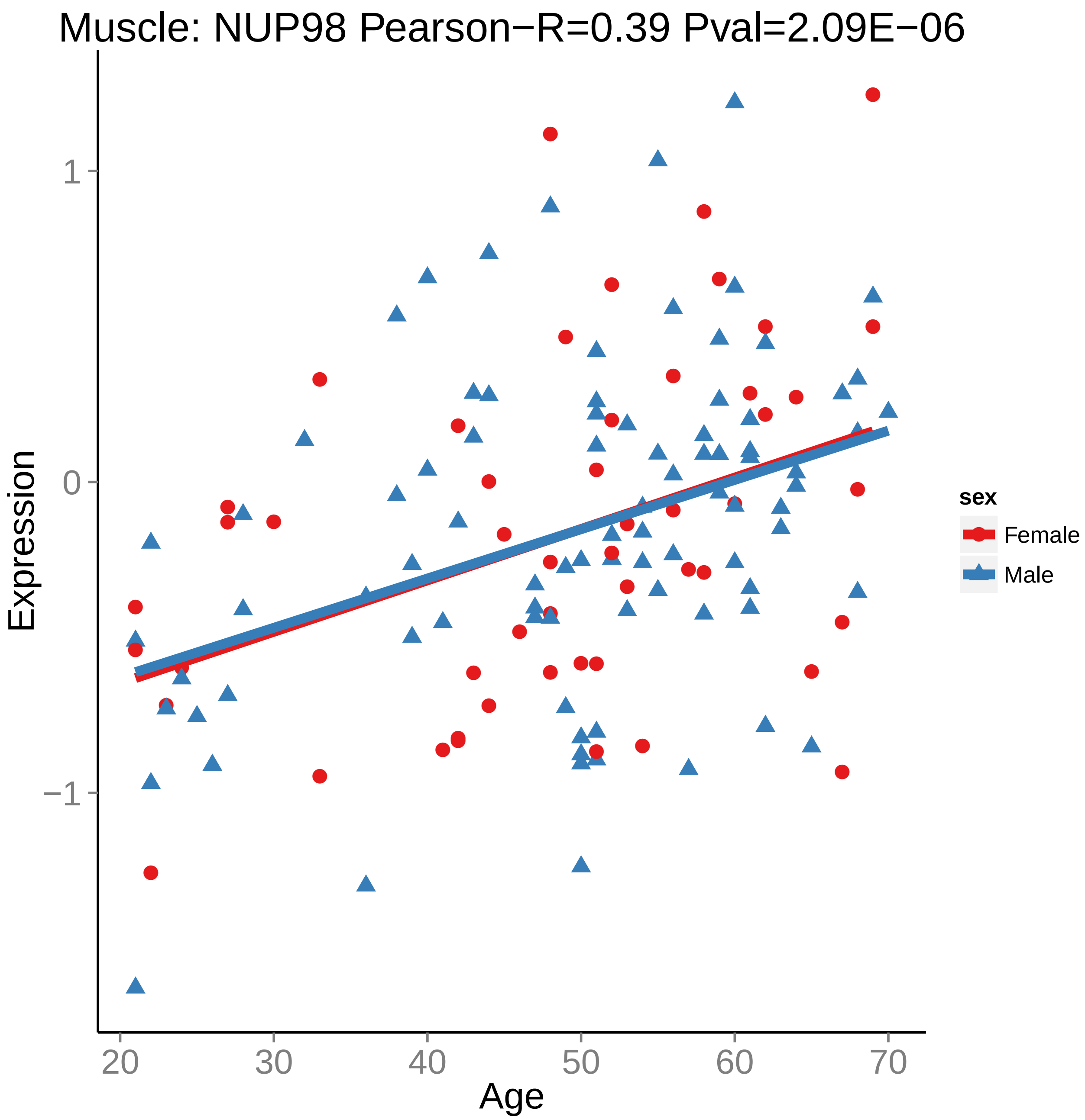
Muscle: NUP98 Pearson−R=0.39 Pval=2.09E−06
Expression
1
0
−1
20
30
40
50
60
70
Age
sex
Female
Male

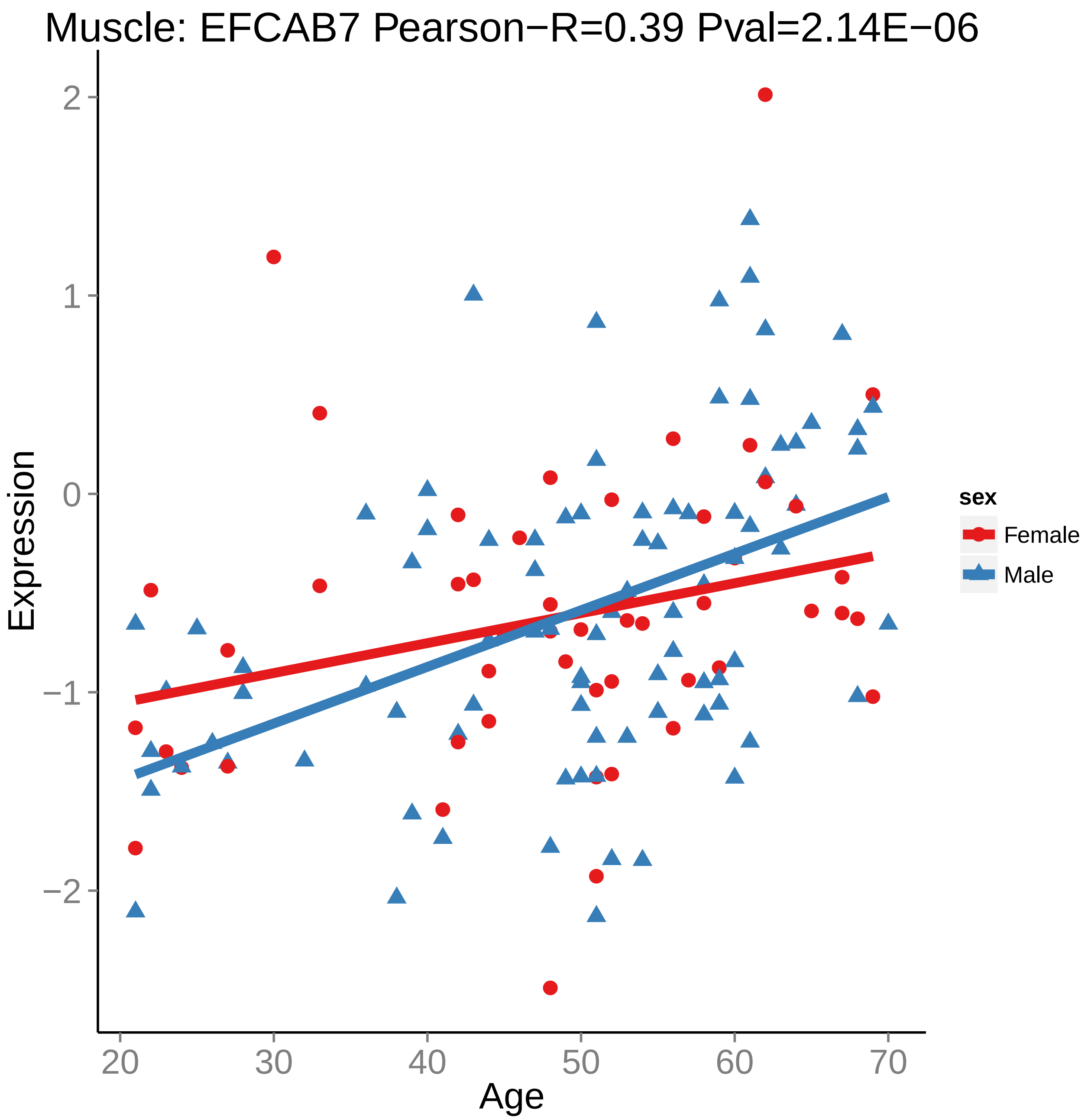

Muscle: EFCAB7 Pearson−R=0.39 Pval=2.14E−06
Expression
2
1
0
−1
−2
20
30
40
50
60
70
Age
sex
Female
Male

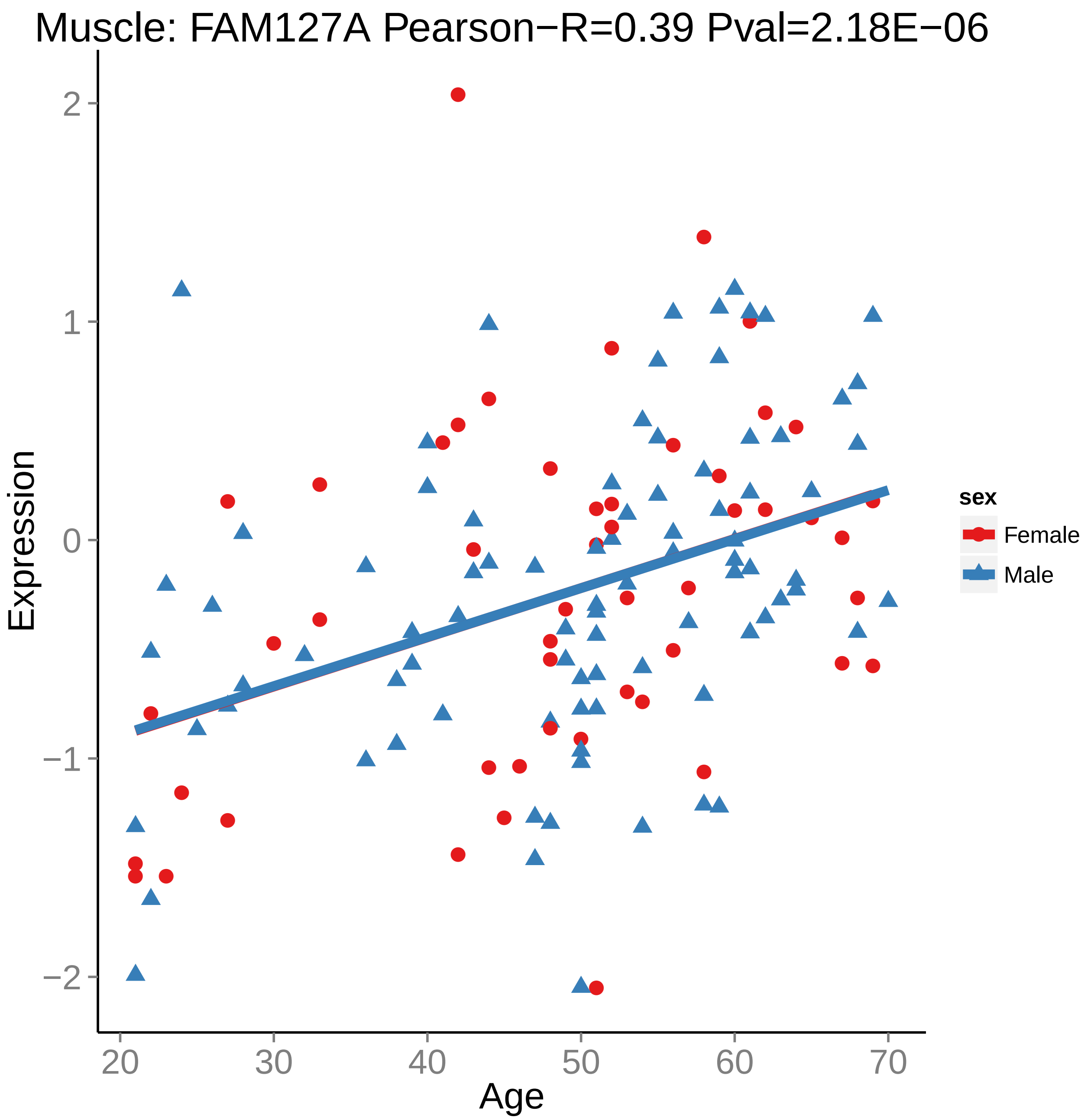

Muscle: FAM127A Pearson−R=0.39 Pval=2.18E−06
Expression
Age
2
1
0
−1
−2
20
30
40
50
60
70
sex
Female
Male

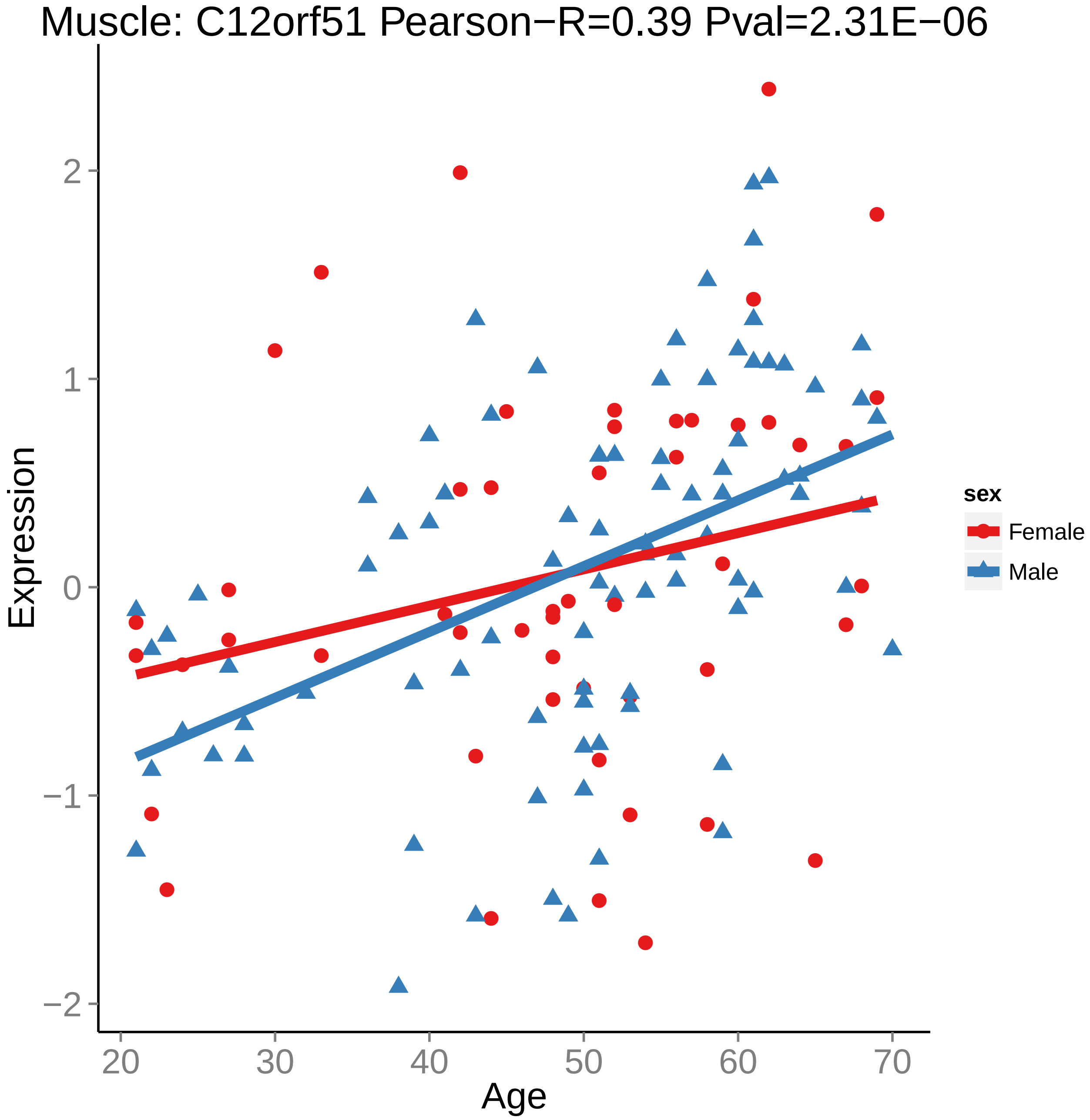

Muscle: C12orf51 Pearson−R=0.39 Pval=2.31E−06
Expression
Age
2
1
0
−1
−2
20
30
40
50
60
70
sex
Female
Male

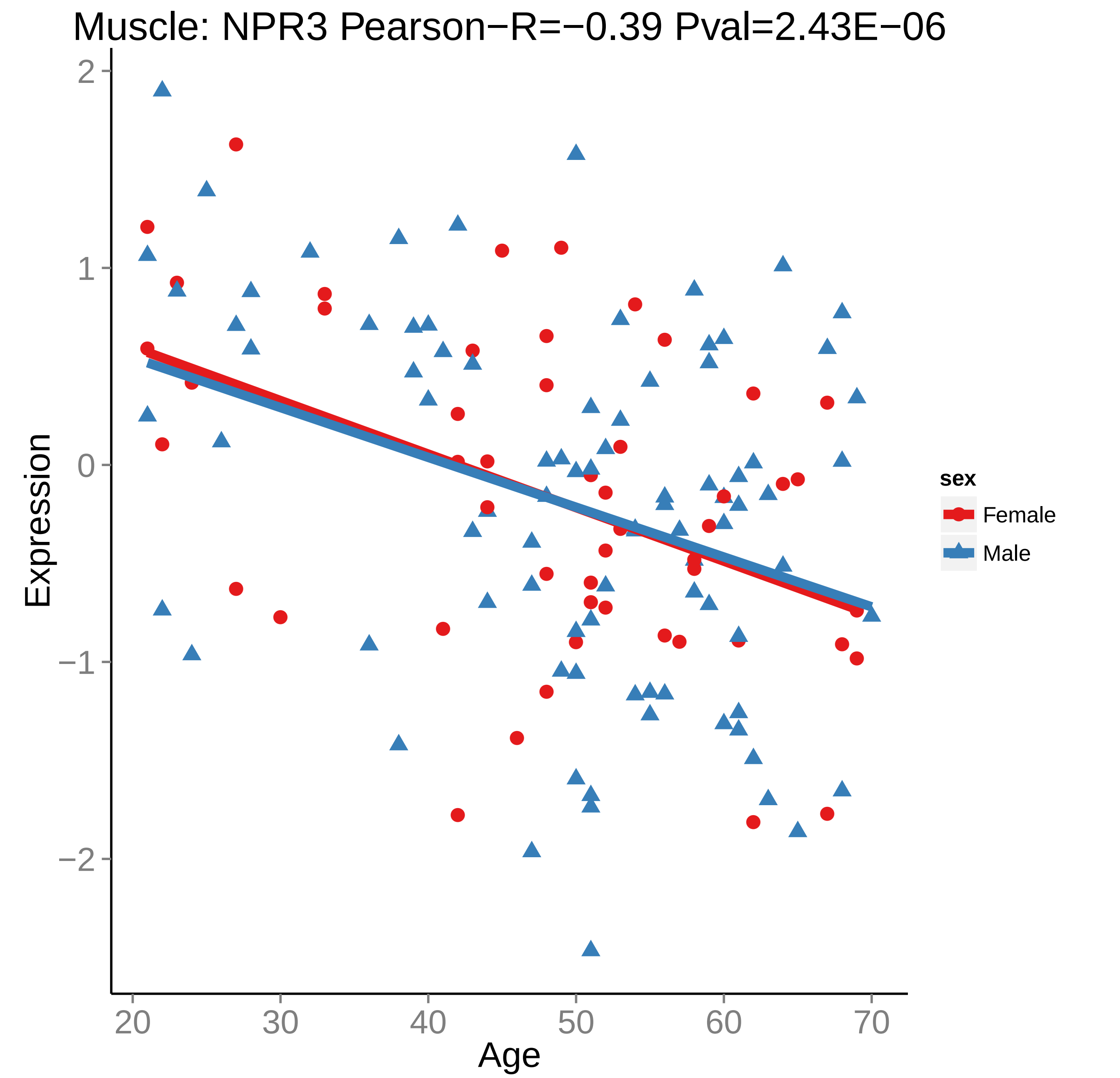

Muscle: NPR3 Pearson−R=−0.39 Pval=2.43E−06
Expression
2
1
0
−1
−2
Age
20
30
40
50
60
70
sex
Female
Male

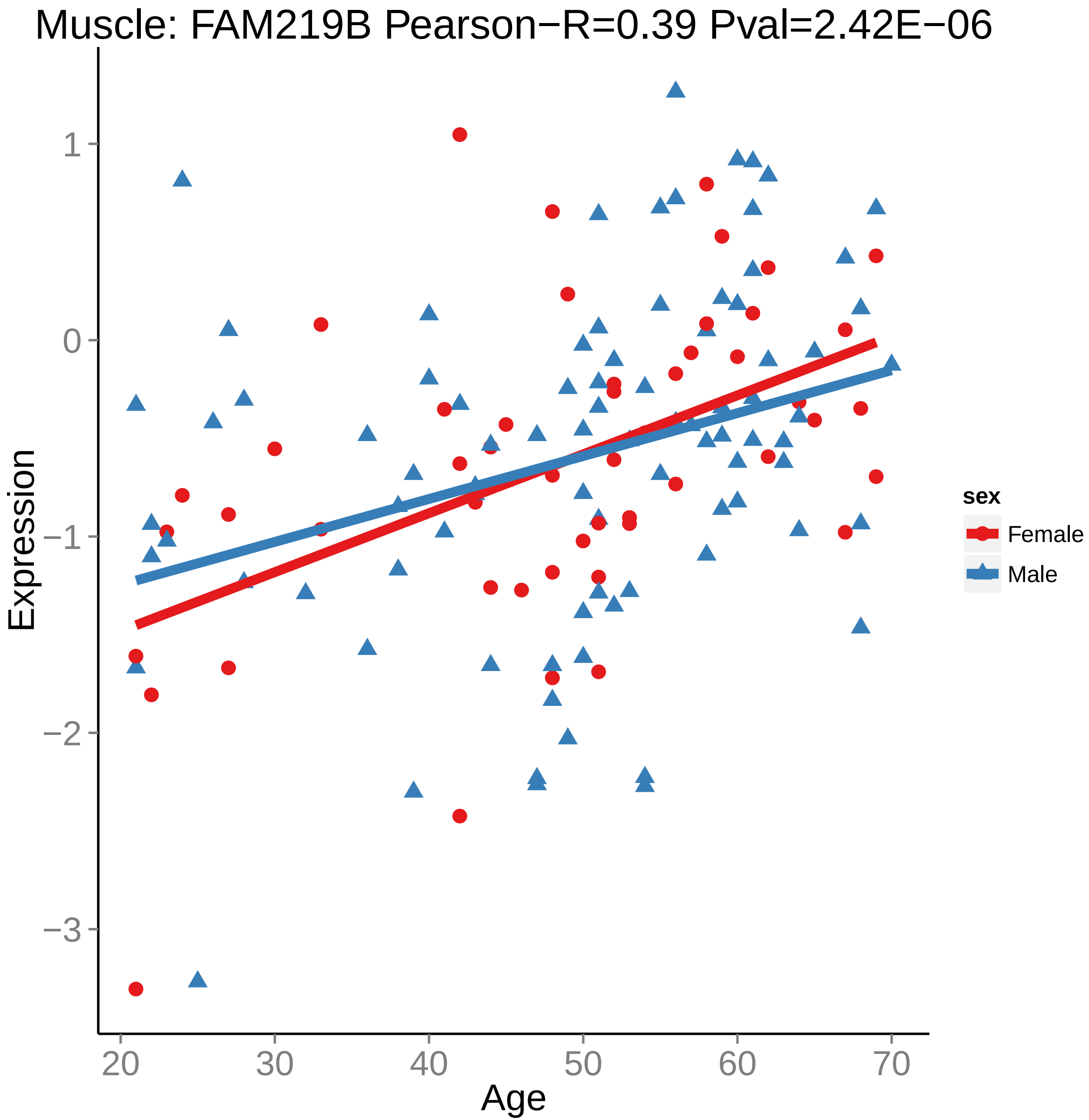

Muscle: FAM219B Pearson−R=0.39 Pval=2.42E−06
Expression
Age
sex
Female
Male
1
0
−1
−2
−3
20
30
40
50
60
70

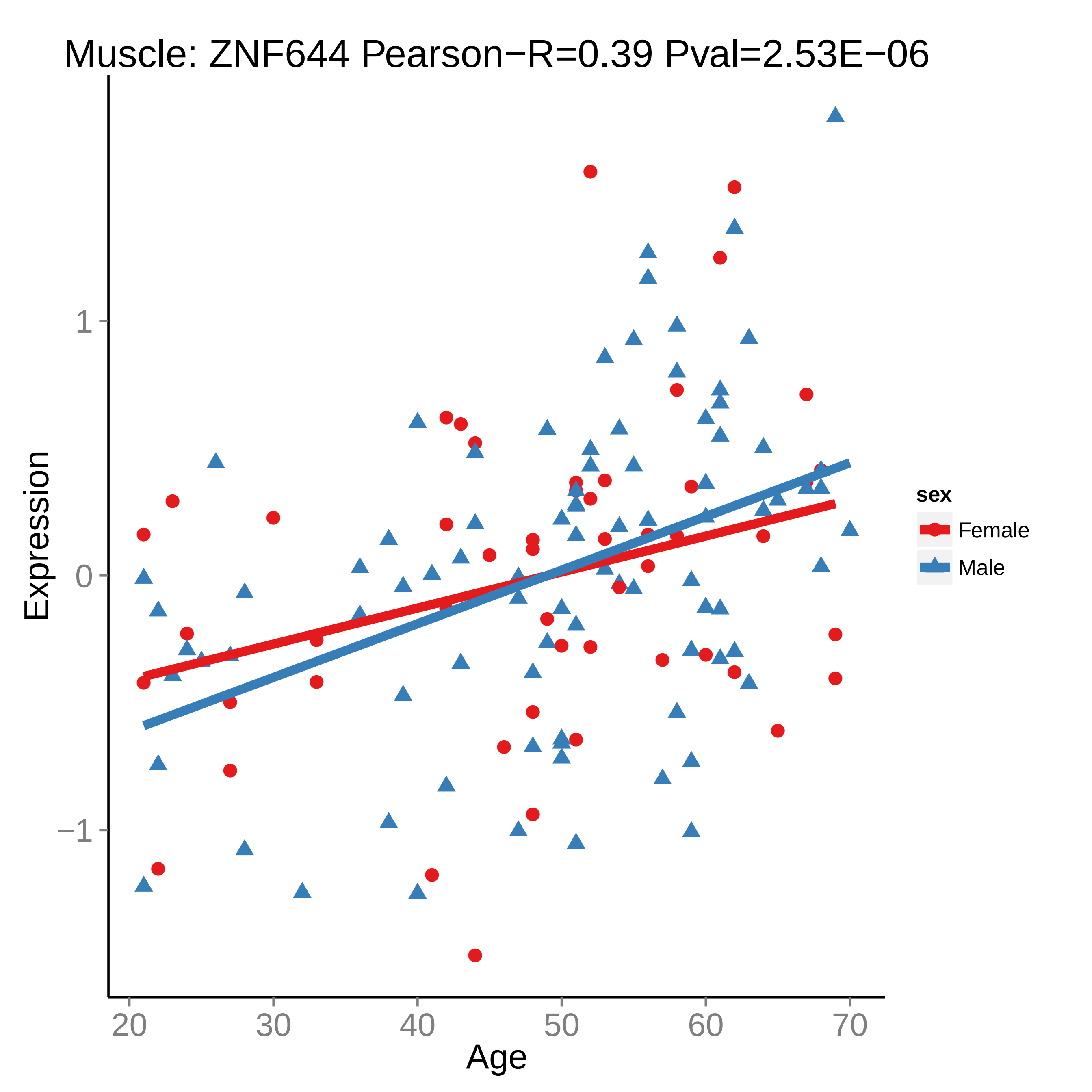

Muscle: ZNF644 Pearson−R=0.39 Pval=2.53E−06
Expression
1
0
−1
20
30
40
50
60
70
Age
sex
Female
Male

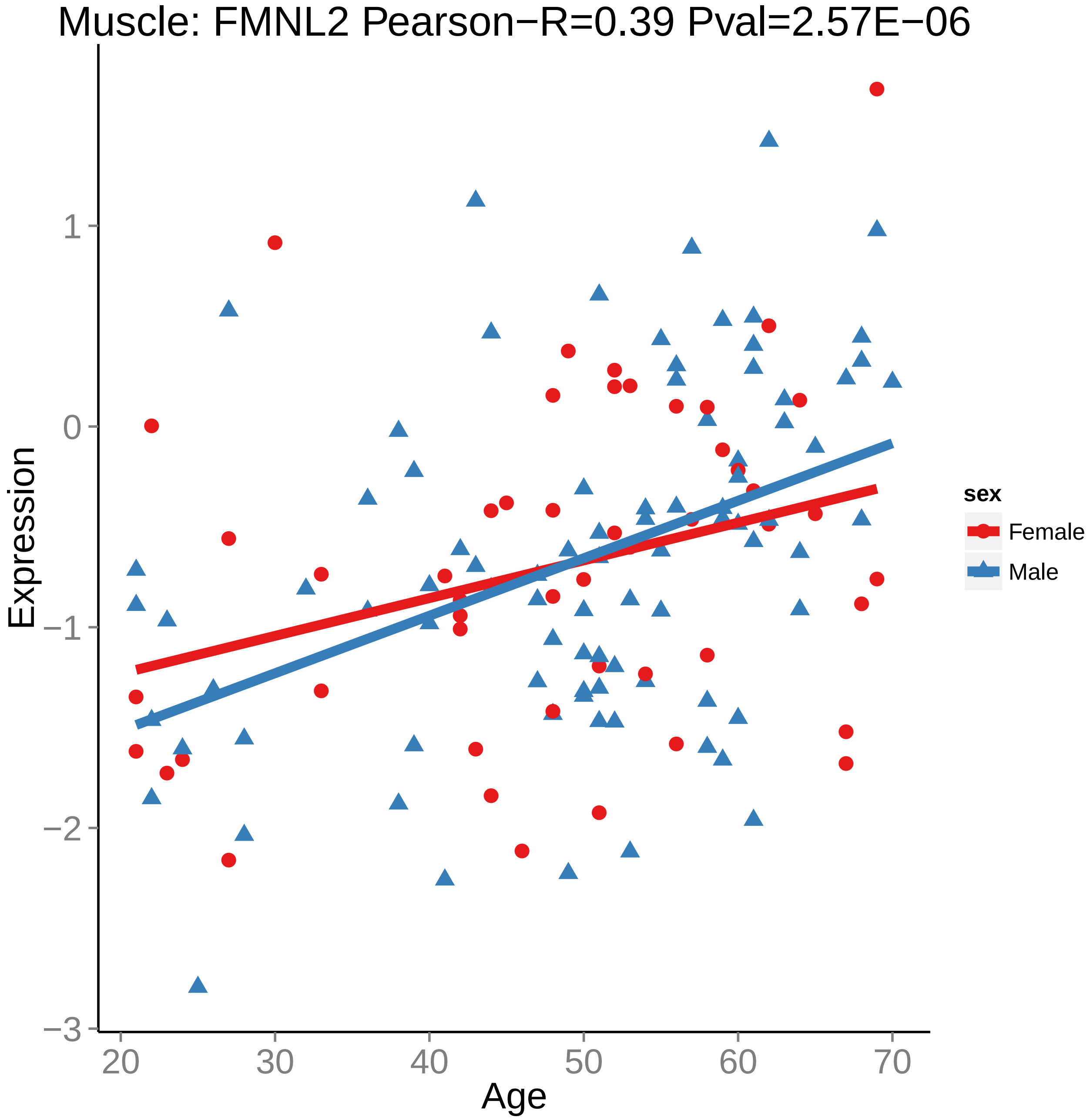

Muscle: FMNL2 Pearson−R=0.39 Pval=2.57E−06
Expression
Age
1
0
−1
−2
−3
20
30
40
50
60
70
sex
Female
Male

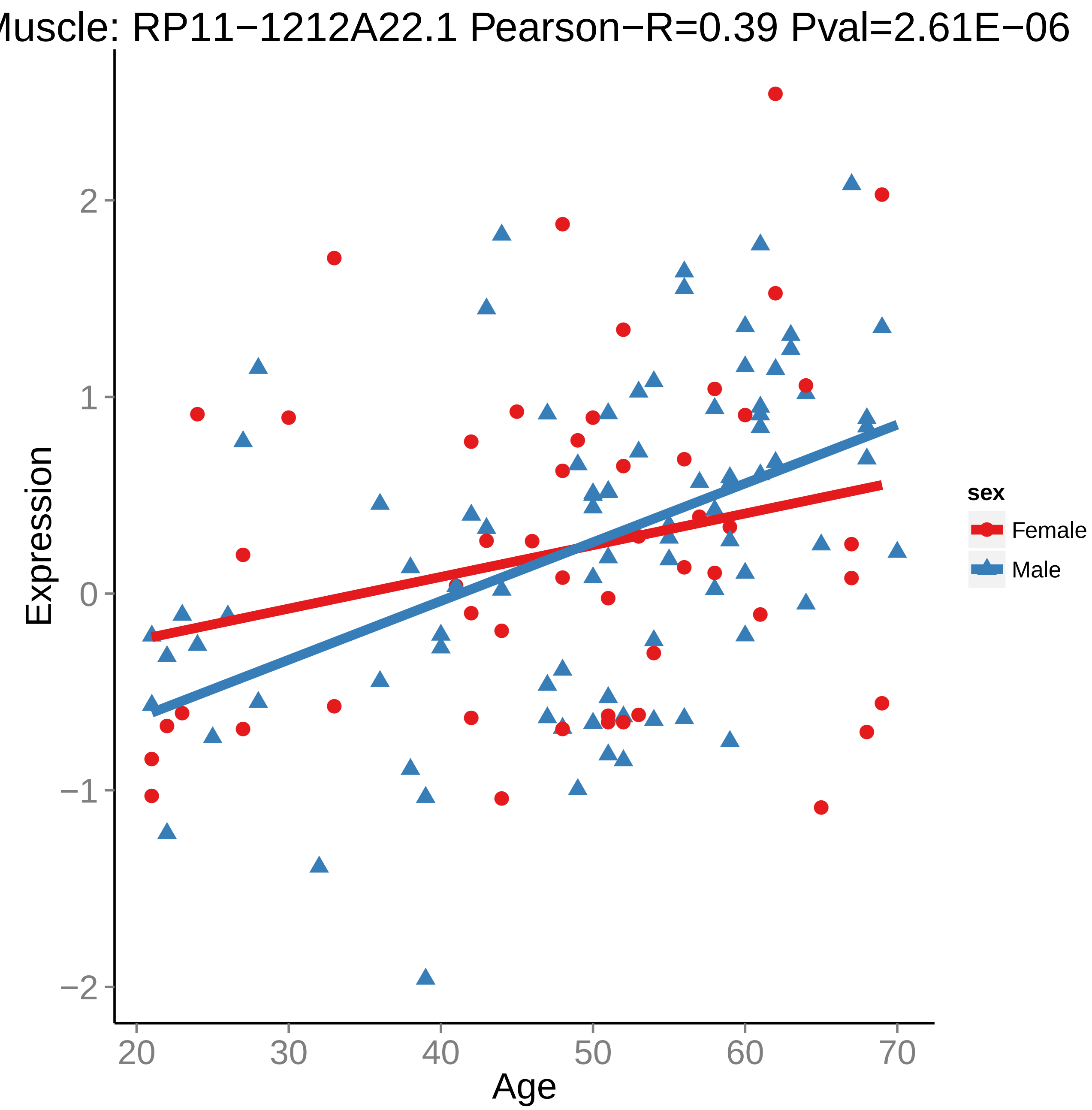

Muscle: RP11−1212A22.1 Pearson−R=0.39 Pval=2.61E−06
Expression
Age
2
1
0
−1
−2
20
30
40
50
60
70
sex
Female
Male

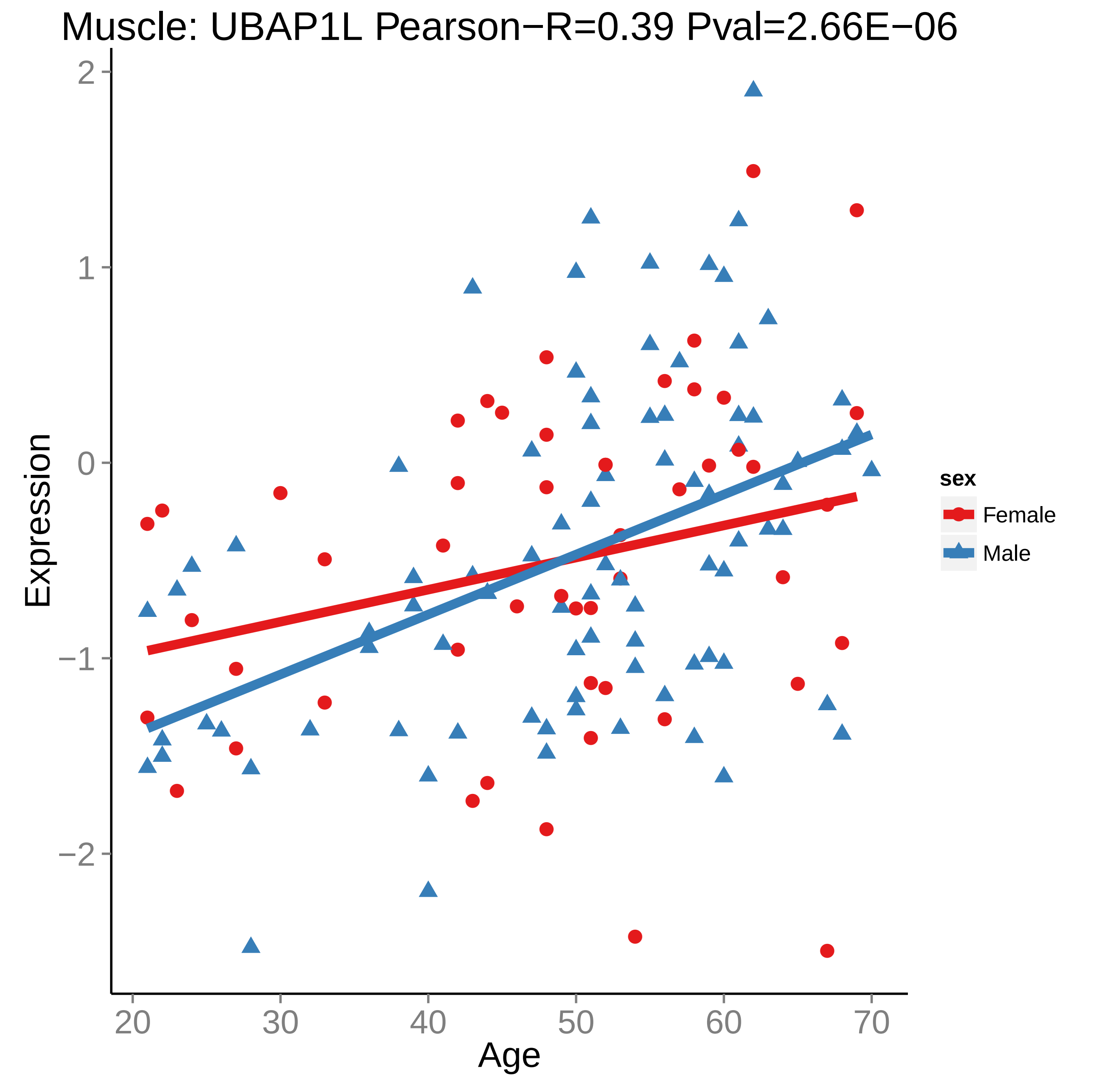

Muscle: UBAP1L Pearson−R=0.39 Pval=2.66E−06
Expression
Age
2
1
0
−1
−2
20
30
40
50
60
70
sex
Female
Male

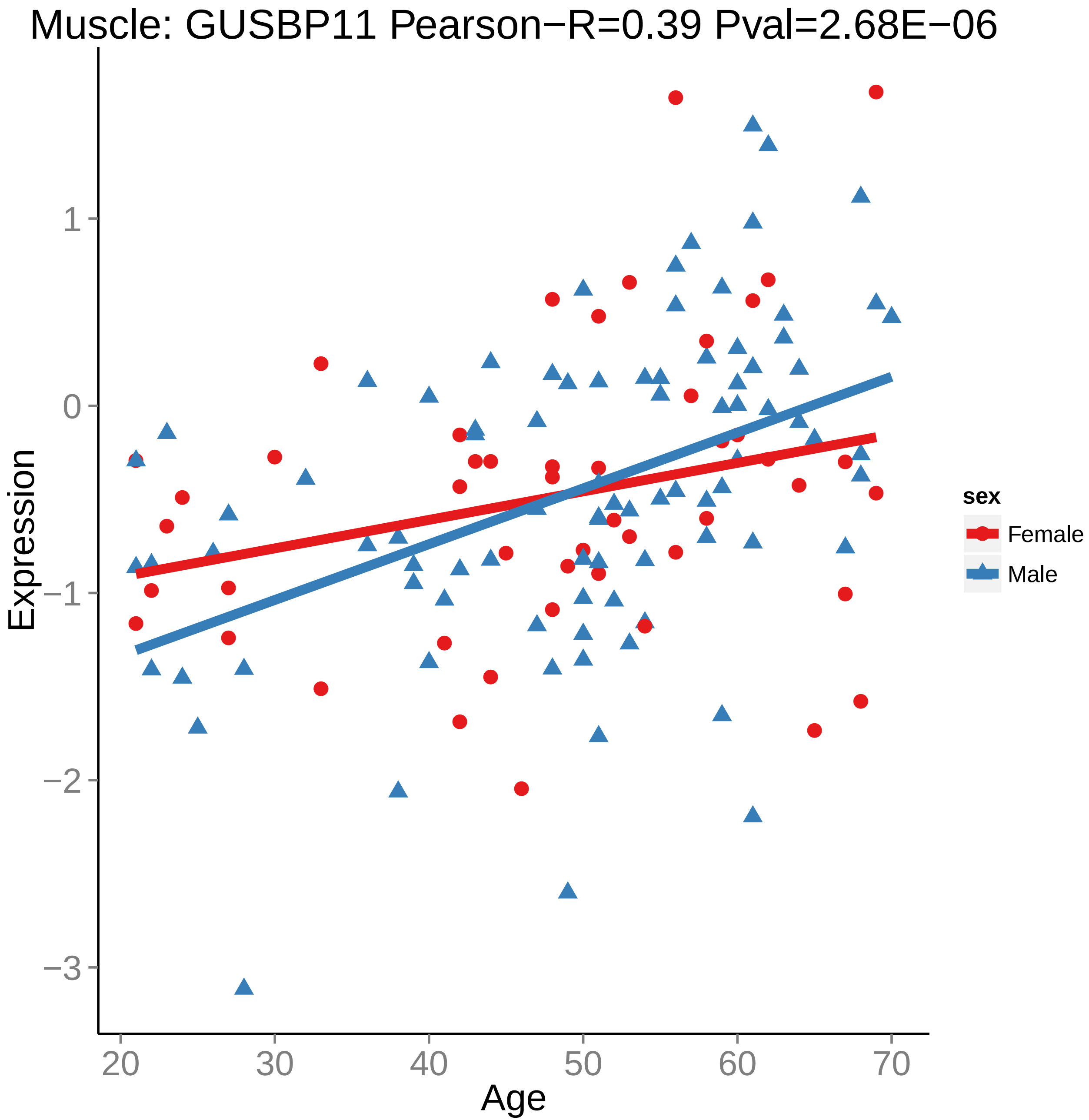

Muscle: GUSBP11 Pearson−R=0.39 Pval=2.68E−06
Expression
Age
1
0
−1
−2
−3
20
30
40
50
60
70
sex
Female
Male

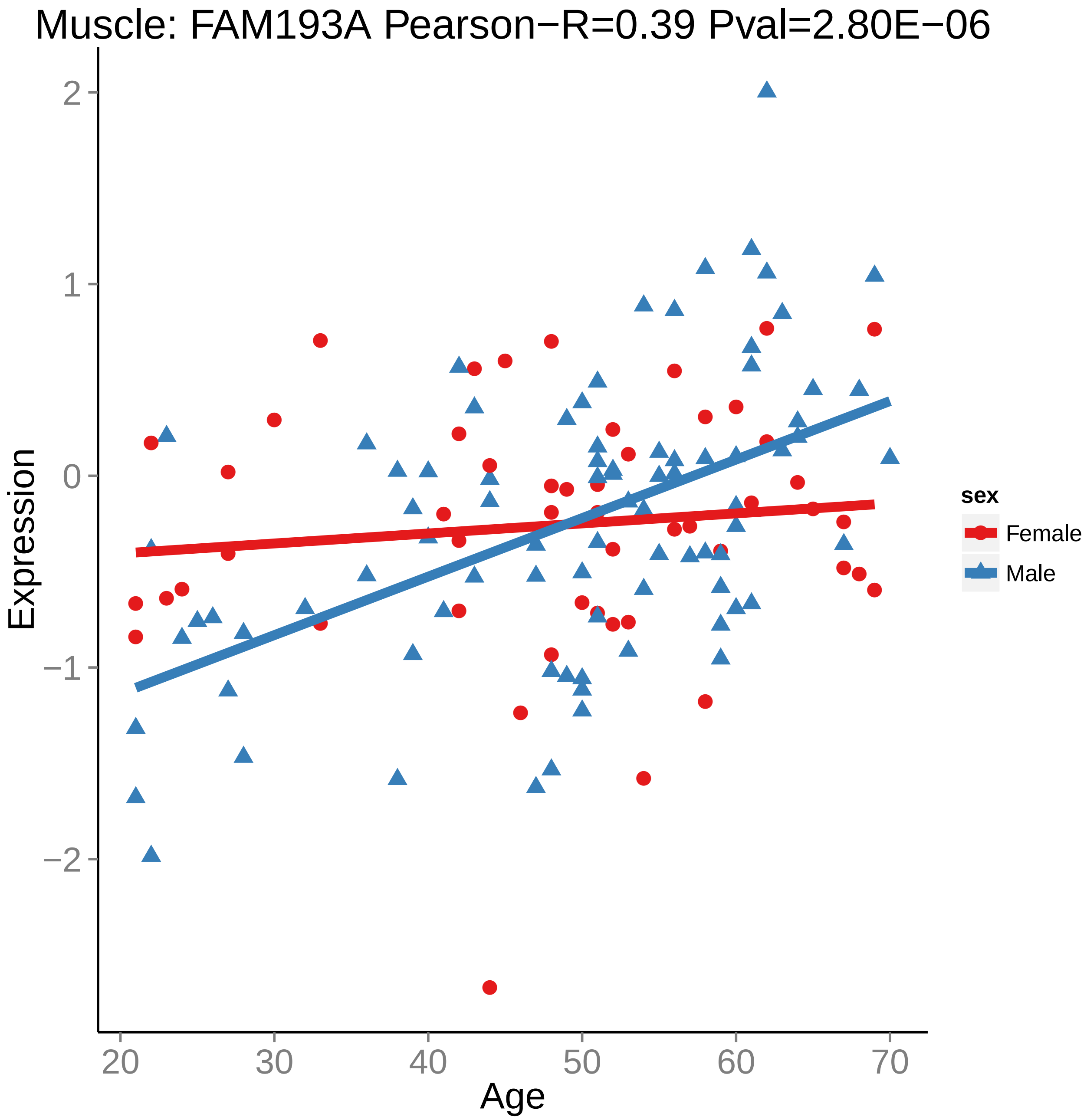

Muscle: FAM193A Pearson−R=0.39 Pval=2.80E−06
Expression
Age
2
1
0
−1
−2
20
30
40
50
60
70
sex
Female
Male

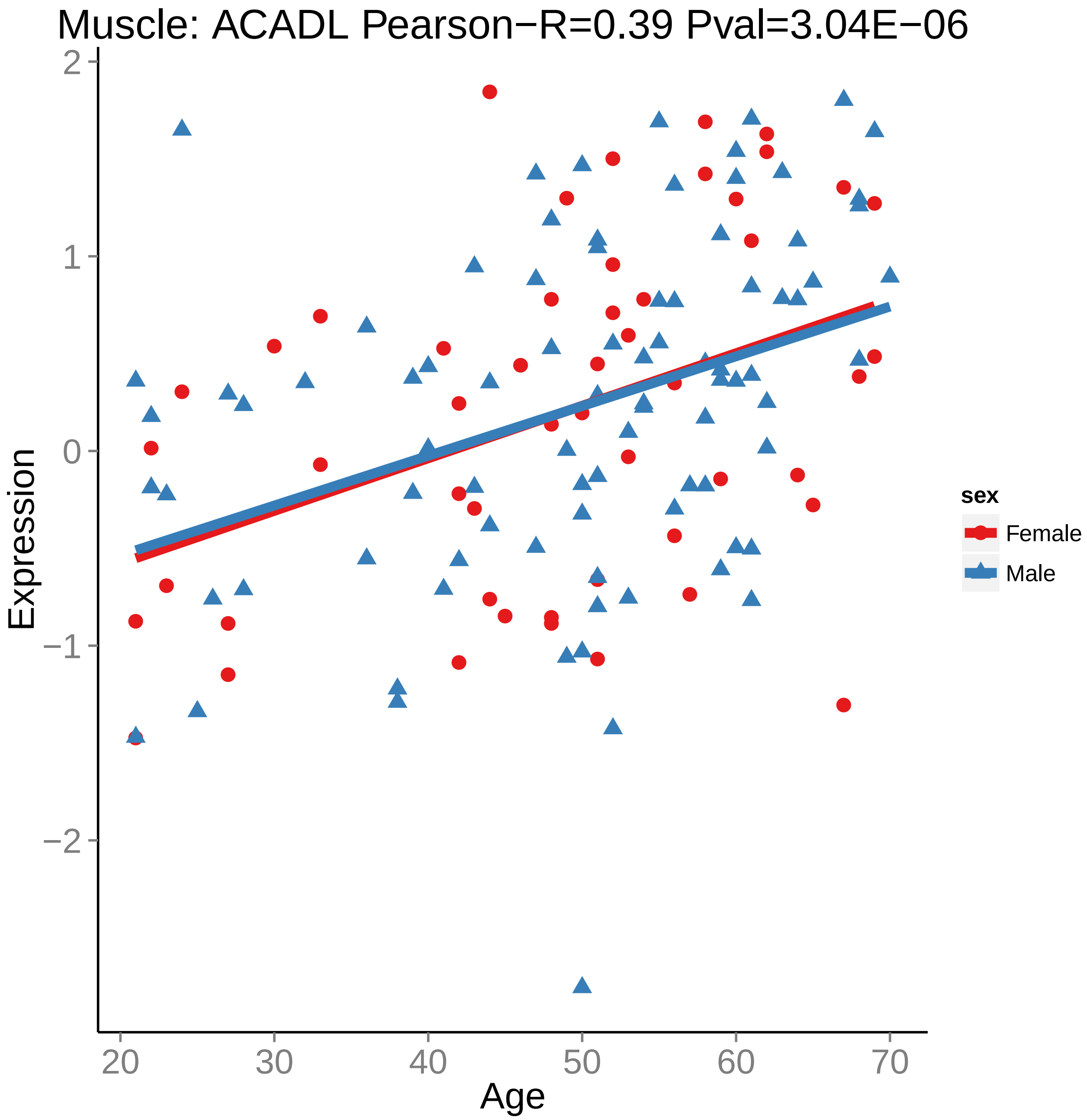

Muscle: ACADL Pearson−R=0.39 Pval=3.04E−06
Expression
Age
2
1
0
−1
−2
20
30
40
50
60
70
sex
Female
Male

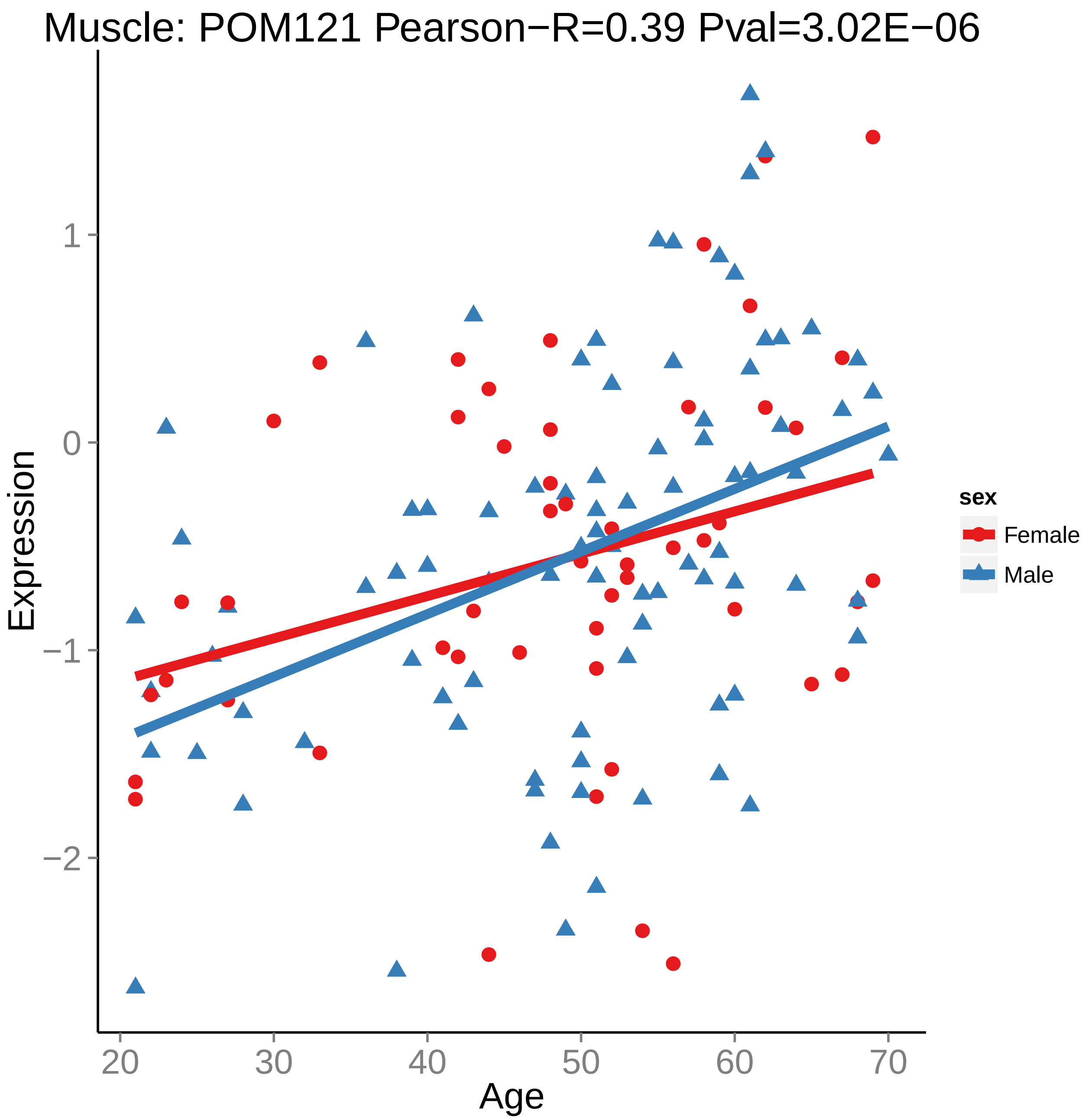

Muscle: POM121 Pearson−R=0.39 Pval=3.02E−06
Expression
1
0
−1
−2
20
30
40
50
60
70
Age
sex
Female
Male

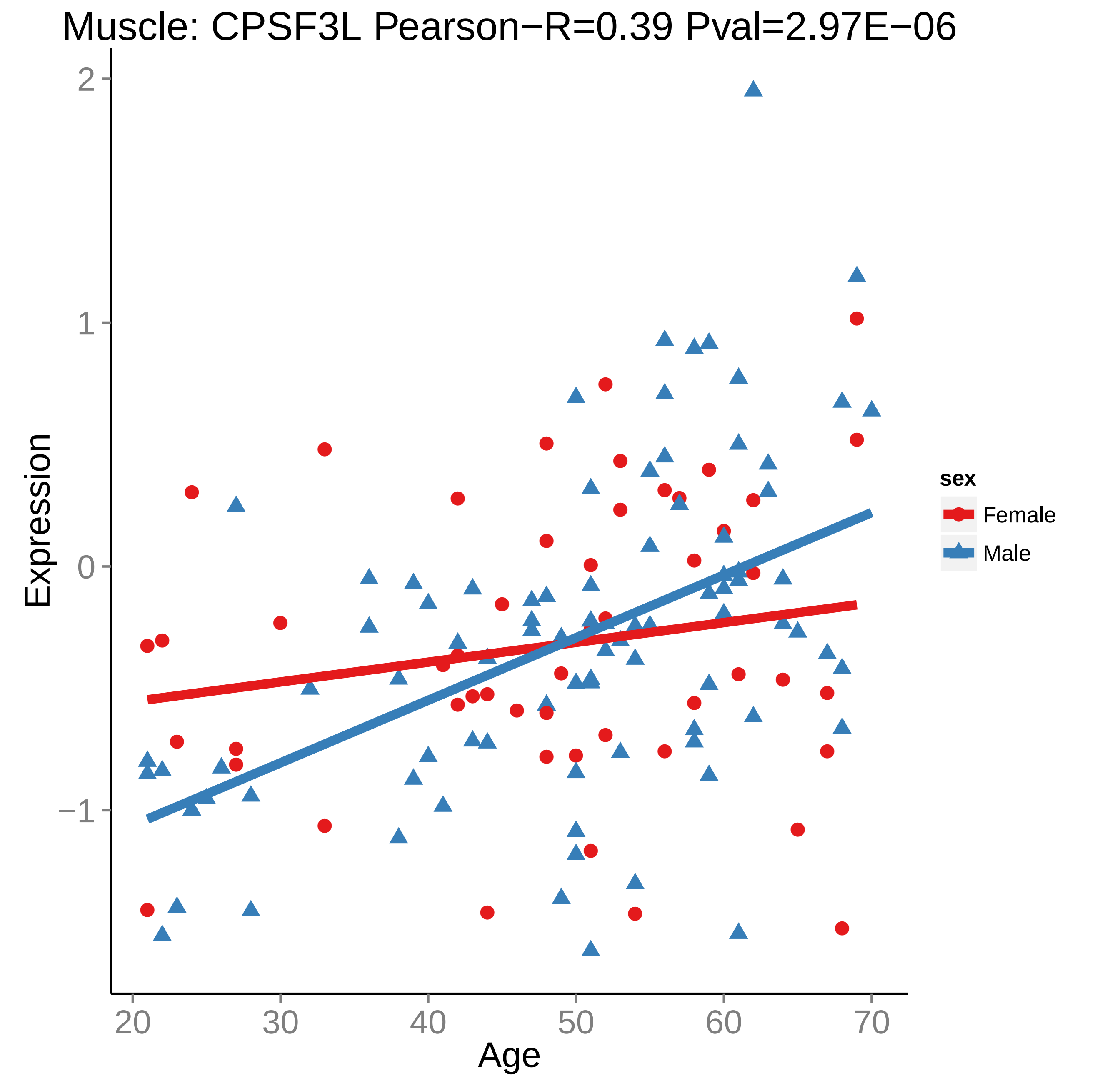

Muscle: CPSF3L Pearson−R=0.39 Pval=2.97E−06
Expression
Age
2
1
0
−1
20
30
40
50
60
70
sex
Female
Male

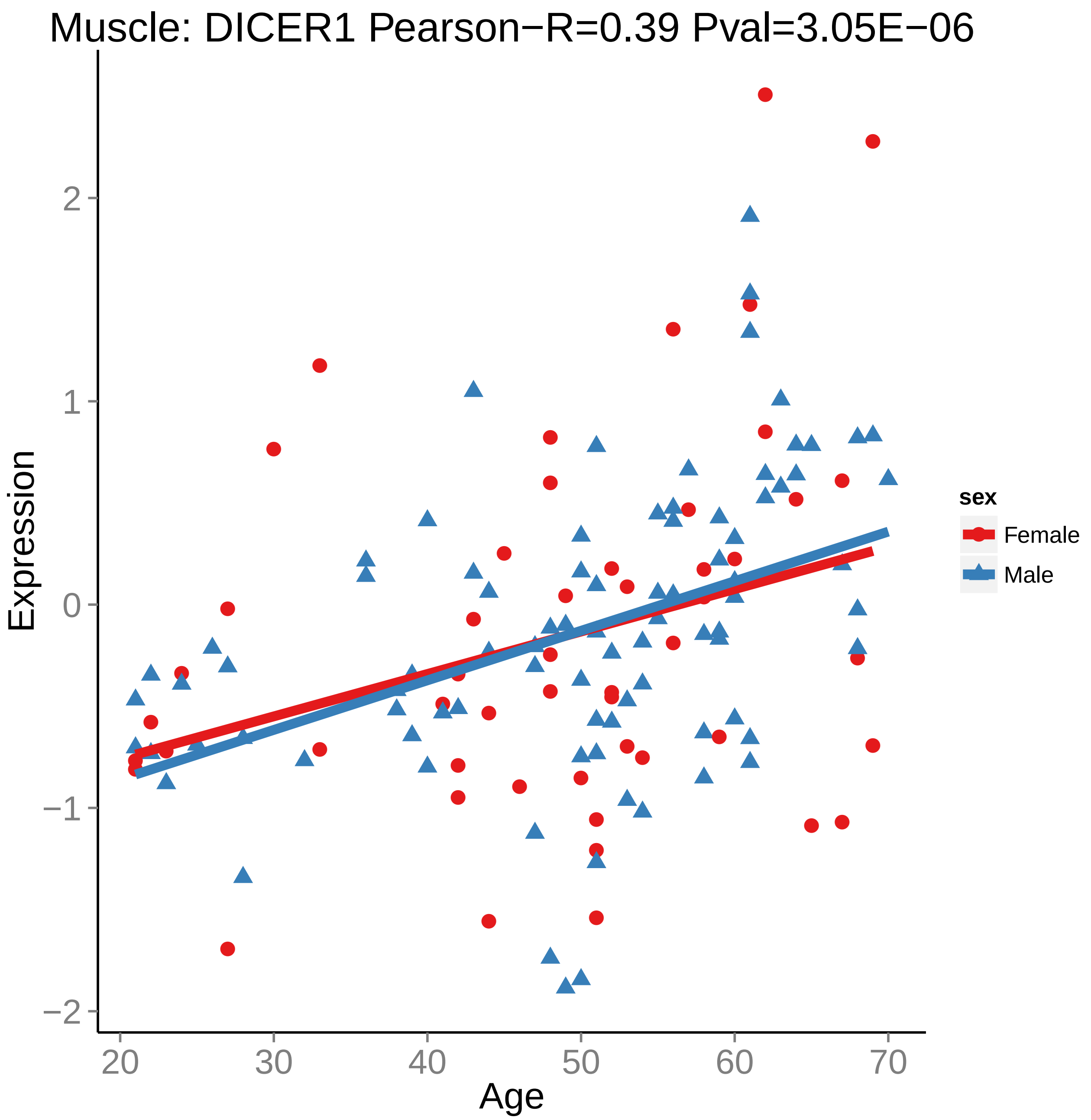

Muscle: DICER1 Pearson−R=0.39 Pval=3.05E−06
Expression
Age
2
1
0
−1
−2
20
30
40
50
60
70
sex
Female
Male

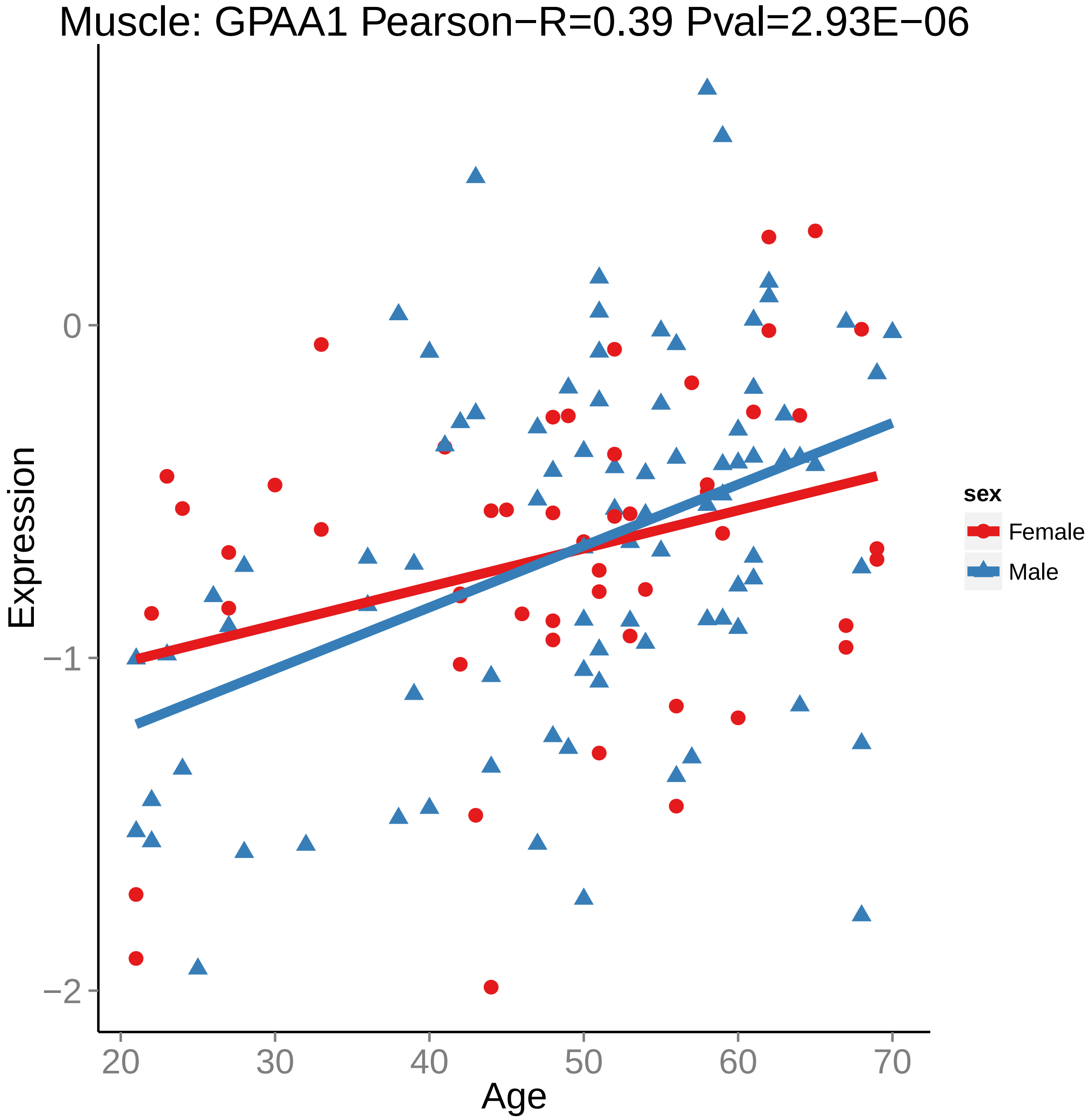

Muscle: GPAA1 Pearson−R=0.39 Pval=2.93E−06
Expression
0
−1
−2
20
30
40
50
60
70
Age
sex
Female
Male

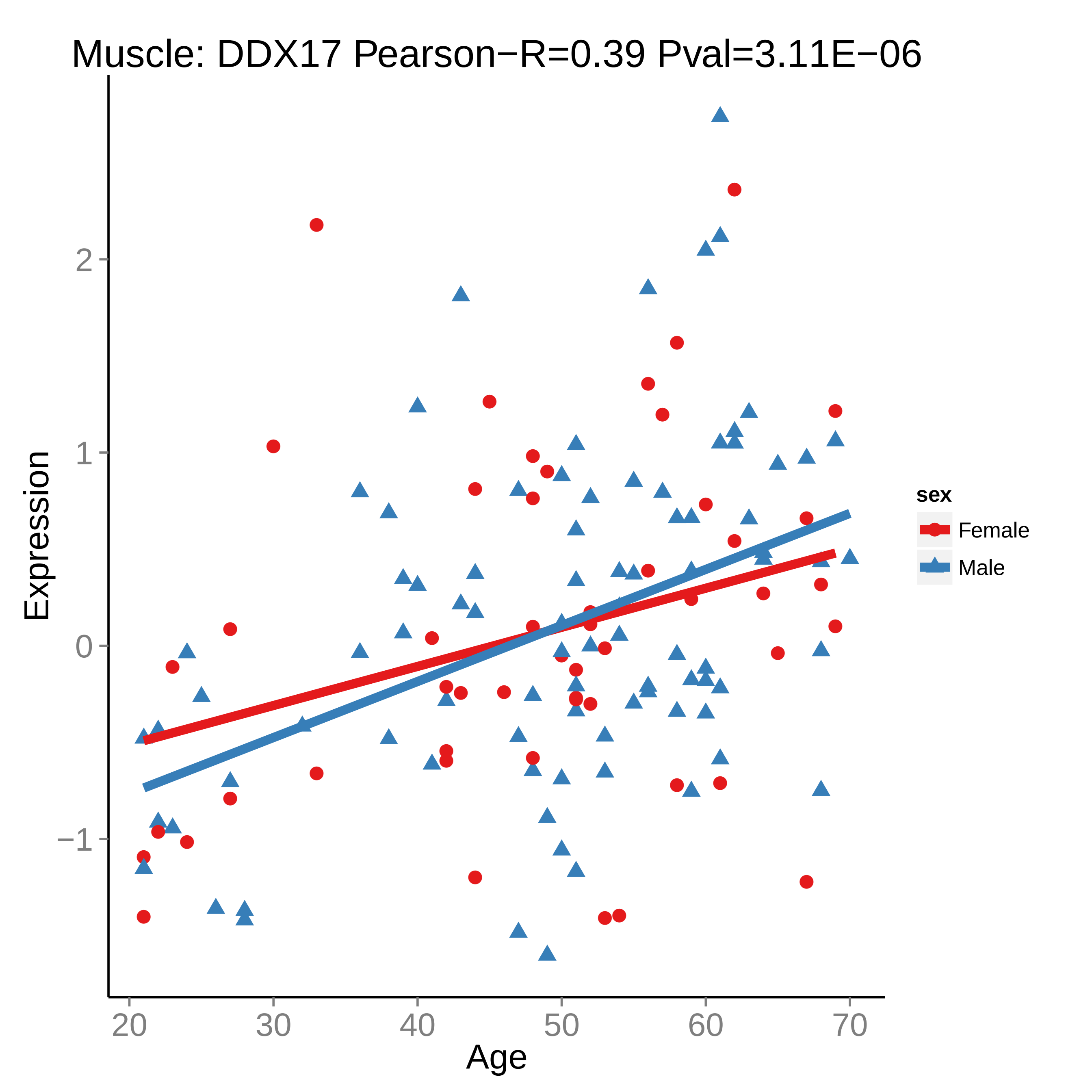

Muscle: DDX17 Pearson−R=0.39 Pval=3.11E−06
Expression
2
1
0
−1
20
30
40
50
60
70
Age
sex
Female
Male

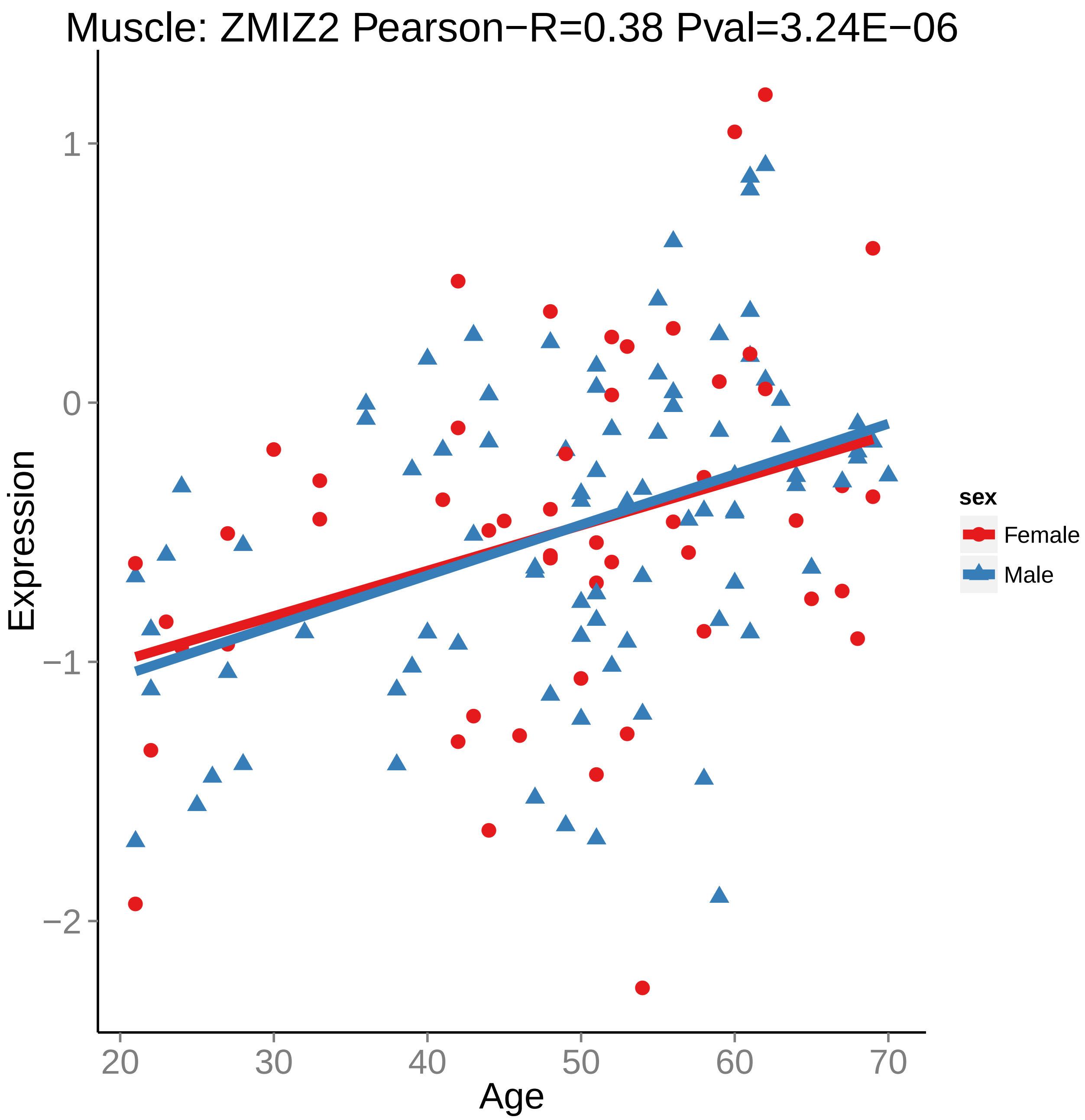

Muscle: ZMIZ2 Pearson−R=0.38 Pval=3.24E−06
Expression
1
0
−1
−2
20
30
40
50
60
70
Age
sex
Female
Male

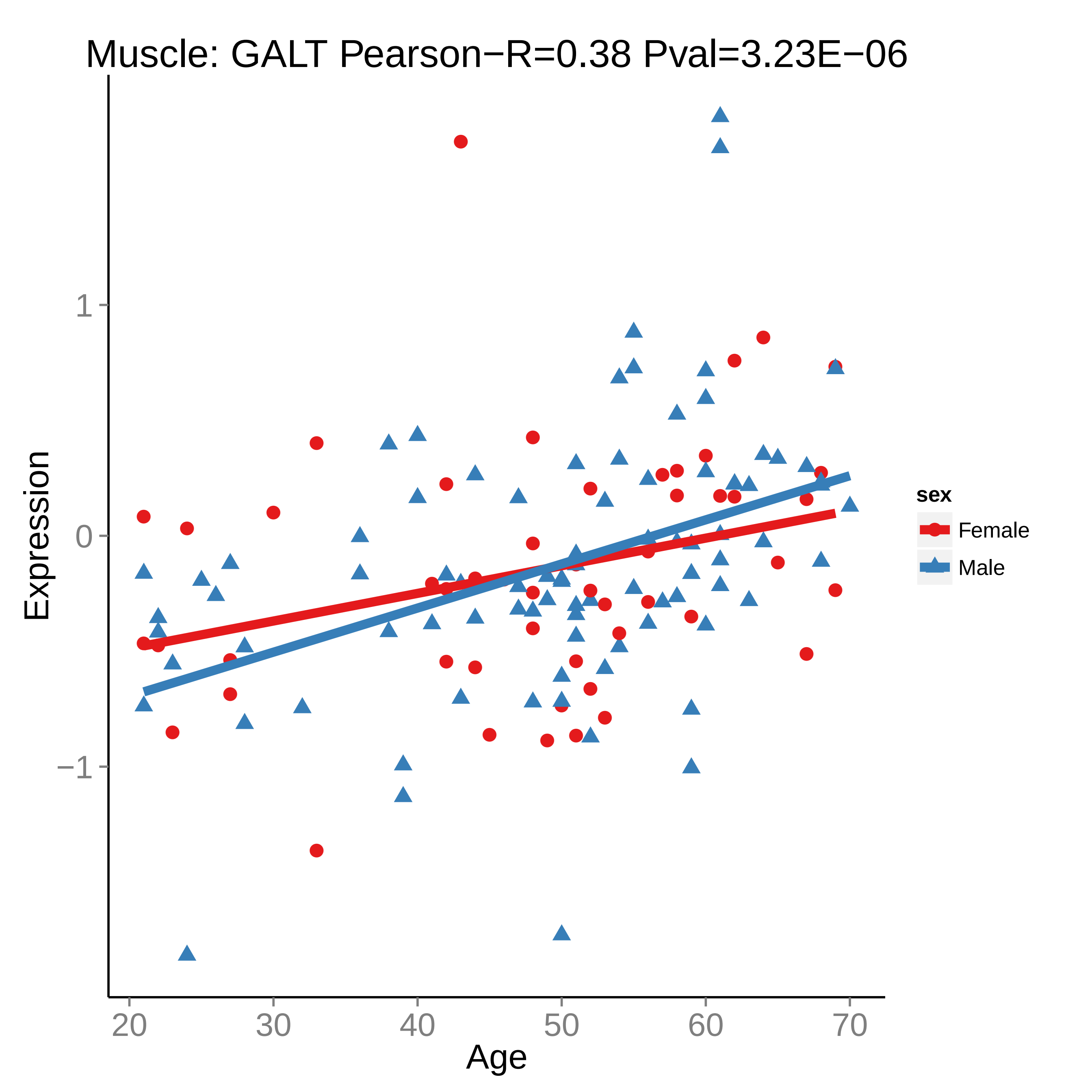
Muscle: GALT Pearson−R=0.38 Pval=3.23E−06
Expression
1
0
−1
20
30
40
50
60
70
Age
sex
Female
Male

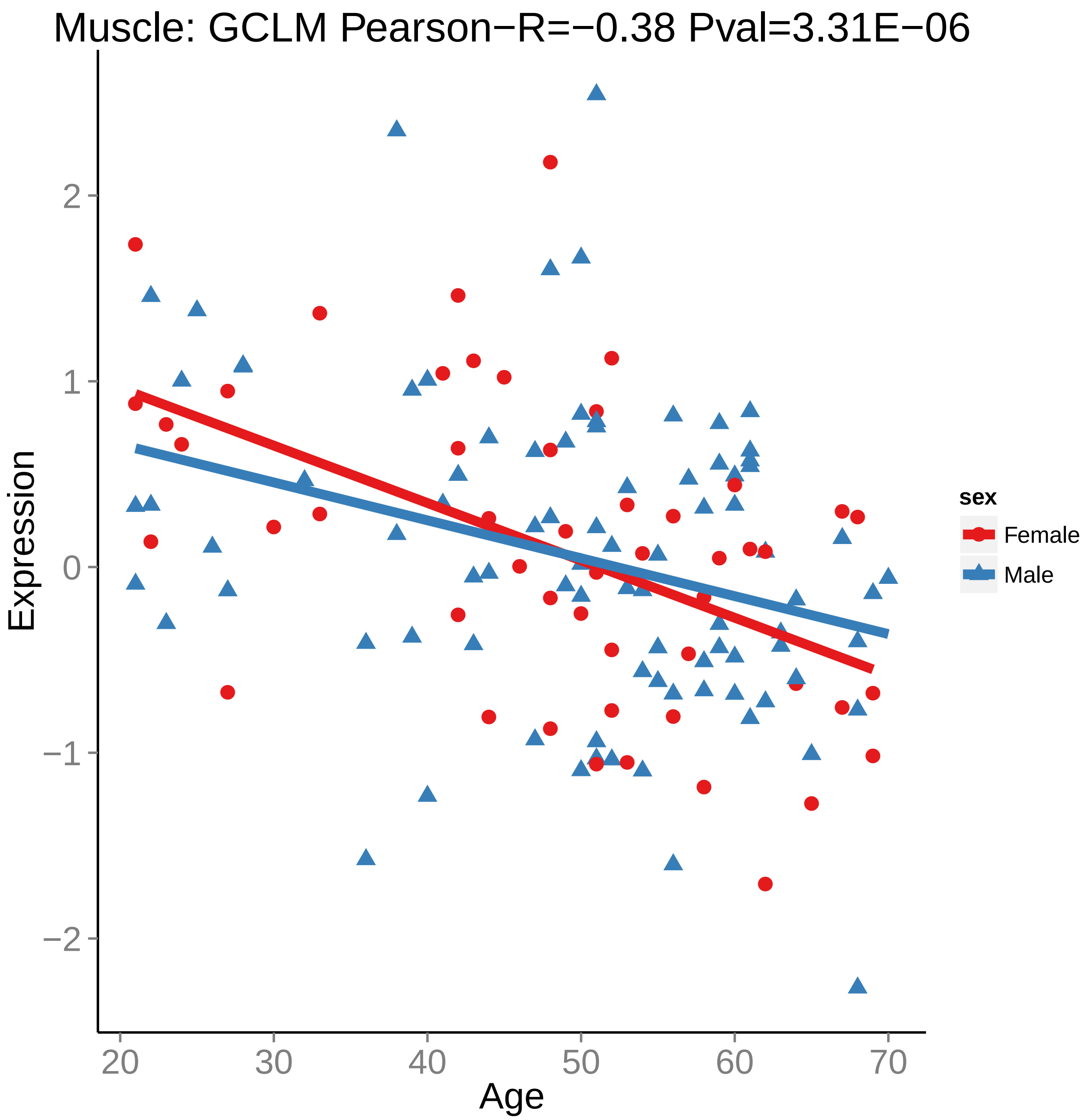

Muscle: GCLM Pearson−R=−0.38 Pval=3.31E−06
Expression
Age
2
1
0
−1
−2
20
30
40
50
60
70
sex
Female
Male

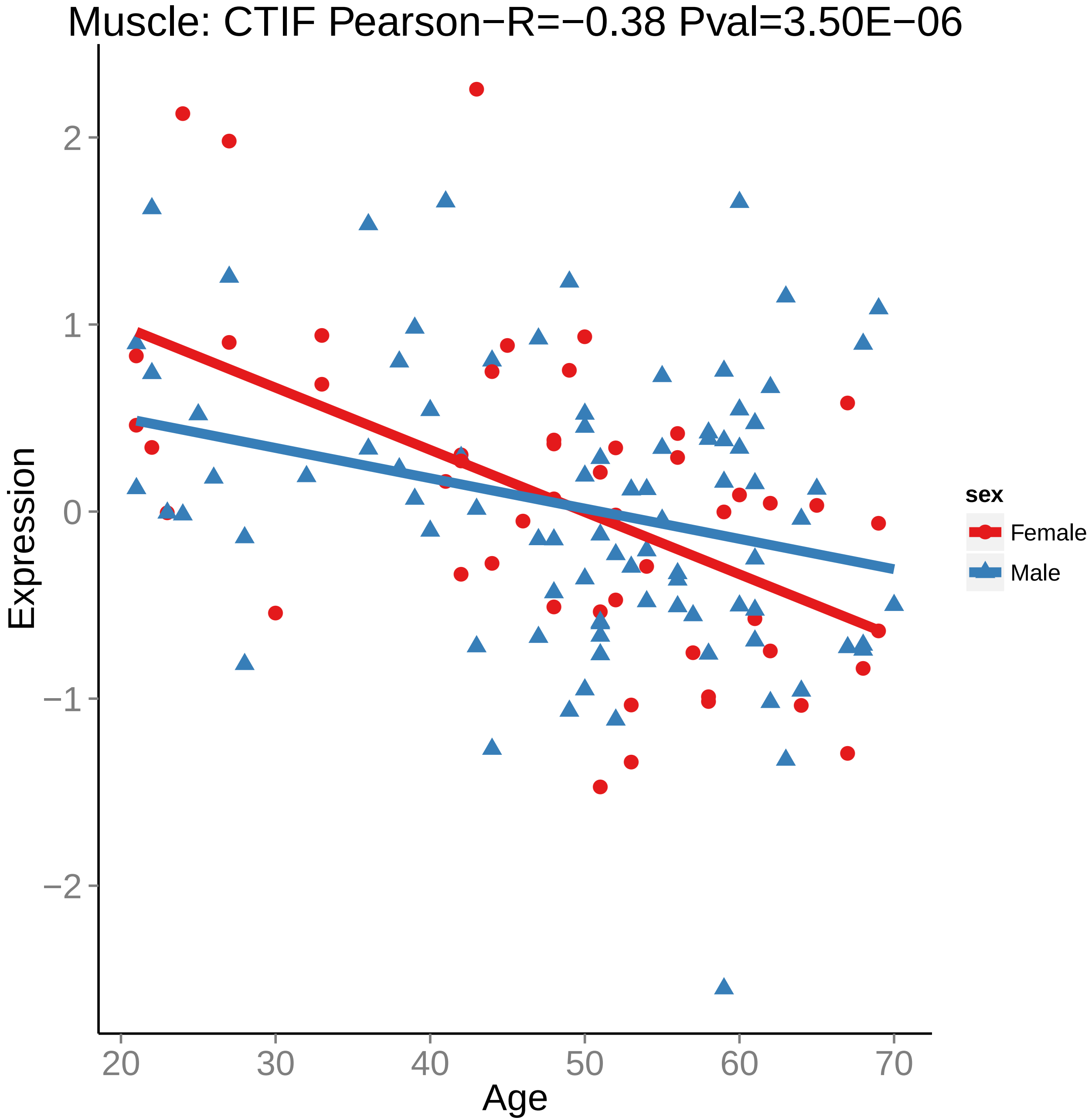

Muscle: CTIF Pearson−R=−0.38 Pval=3.50E−06
Expression
Age
2
1
0
−1
−2
20
30
40
50
60
70
sex
Female
Male

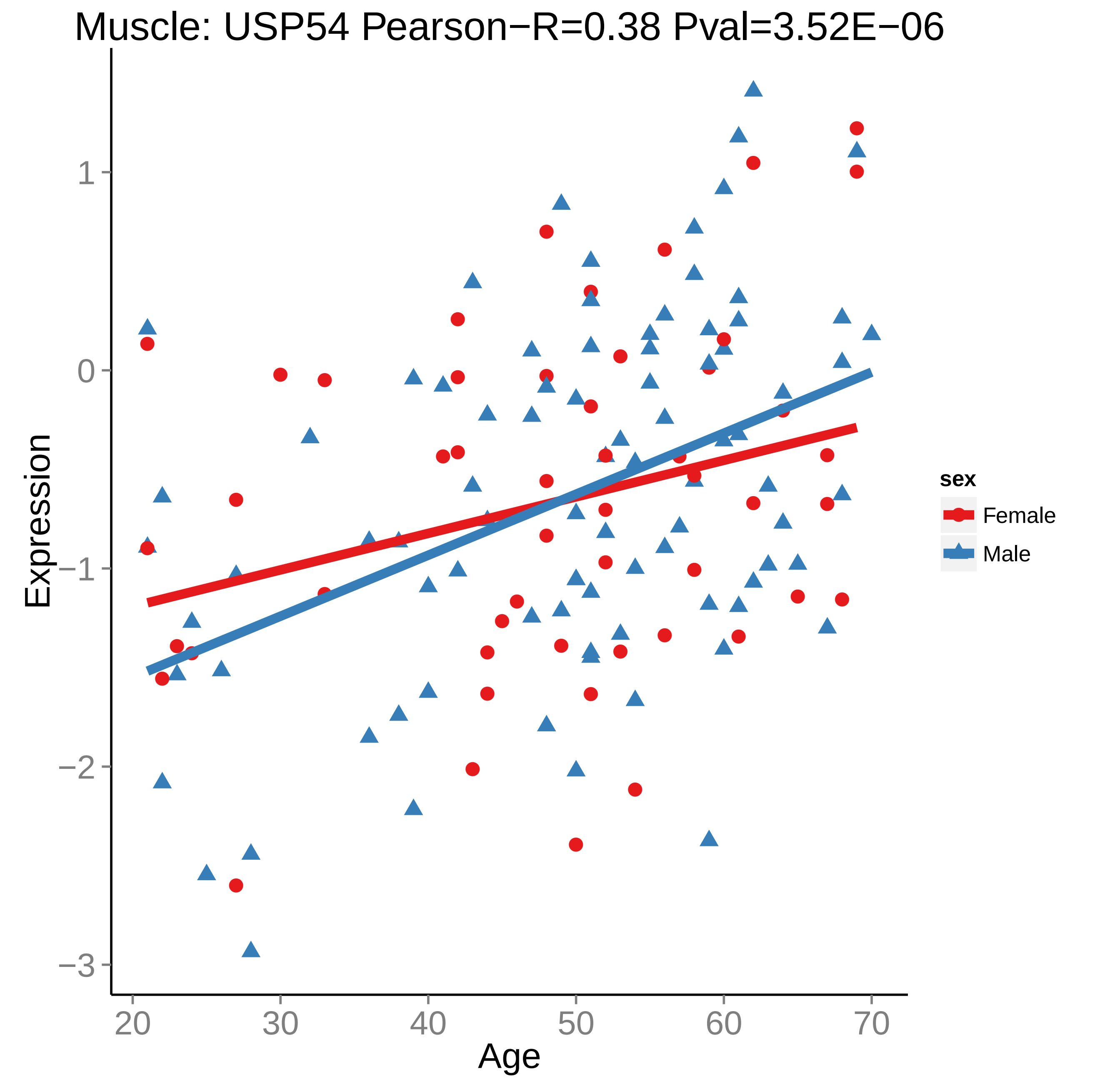
Muscle: USP54 Pearson−R=0.38 Pval=3.52E−06
Expression
Age
1
0
−1
−2
−3
20
30
40
50
60
70
sex
Female
Male

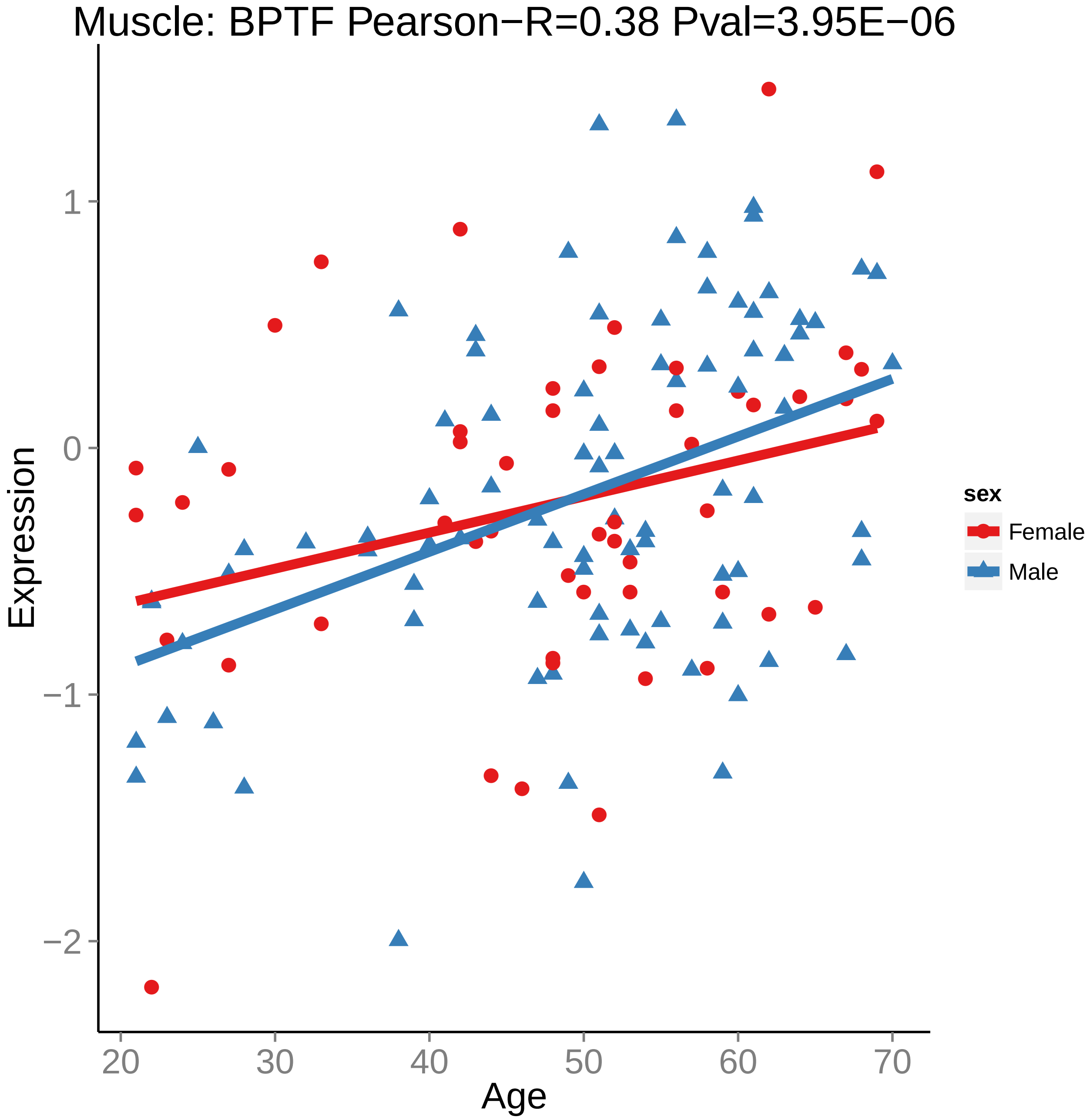
Muscle: BPTF Pearson−R=0.38 Pval=3.95E−06
Expression
Age
1
0
−1
−2
20
30
40
50
60
70
sex
Female
Male

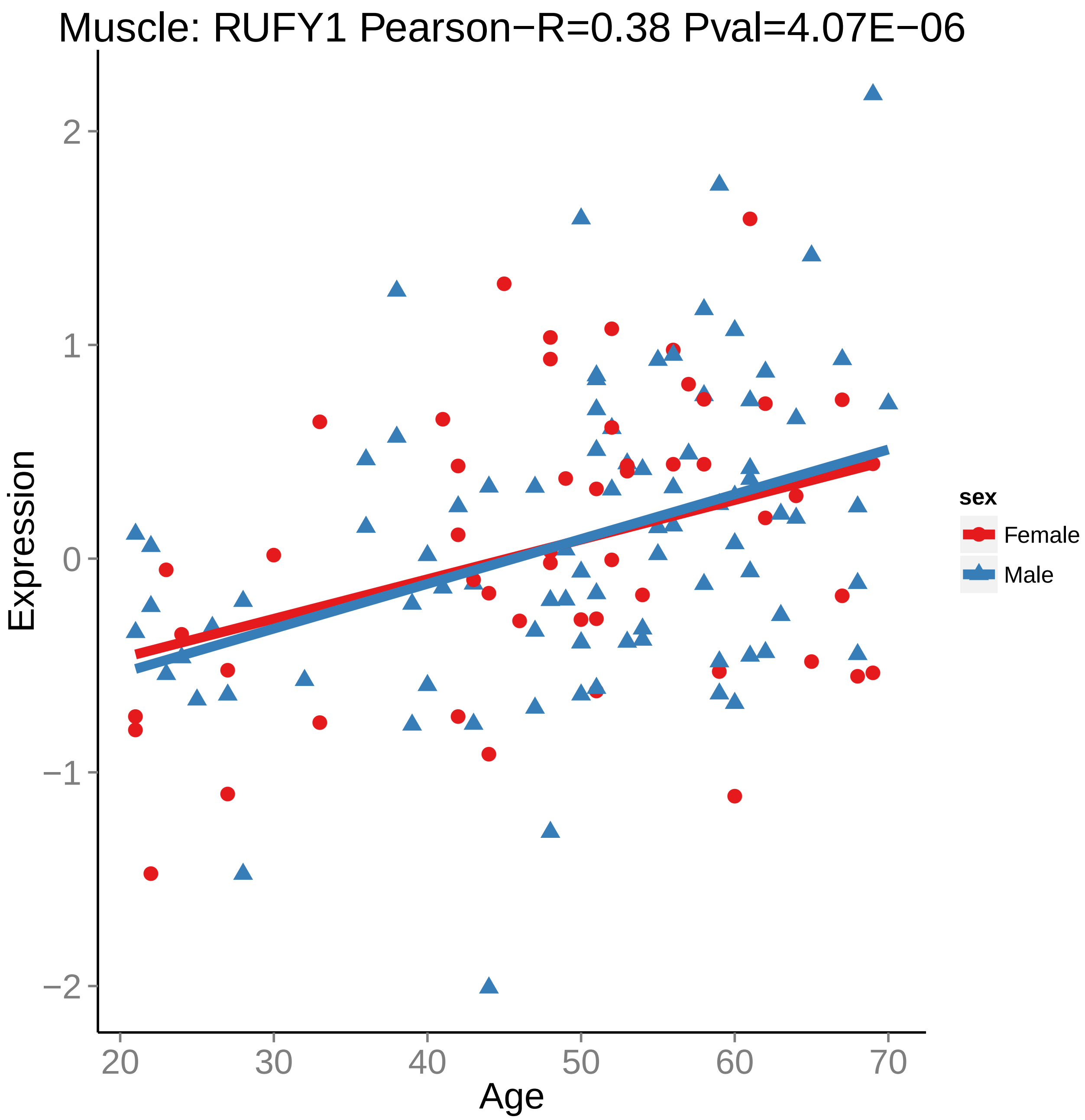

Muscle: RUFY1 Pearson−R=0.38 Pval=4.07E−06
Expression
Age
2
1
0
−1
−2
20
30
40
50
60
70
sex
Female
Male

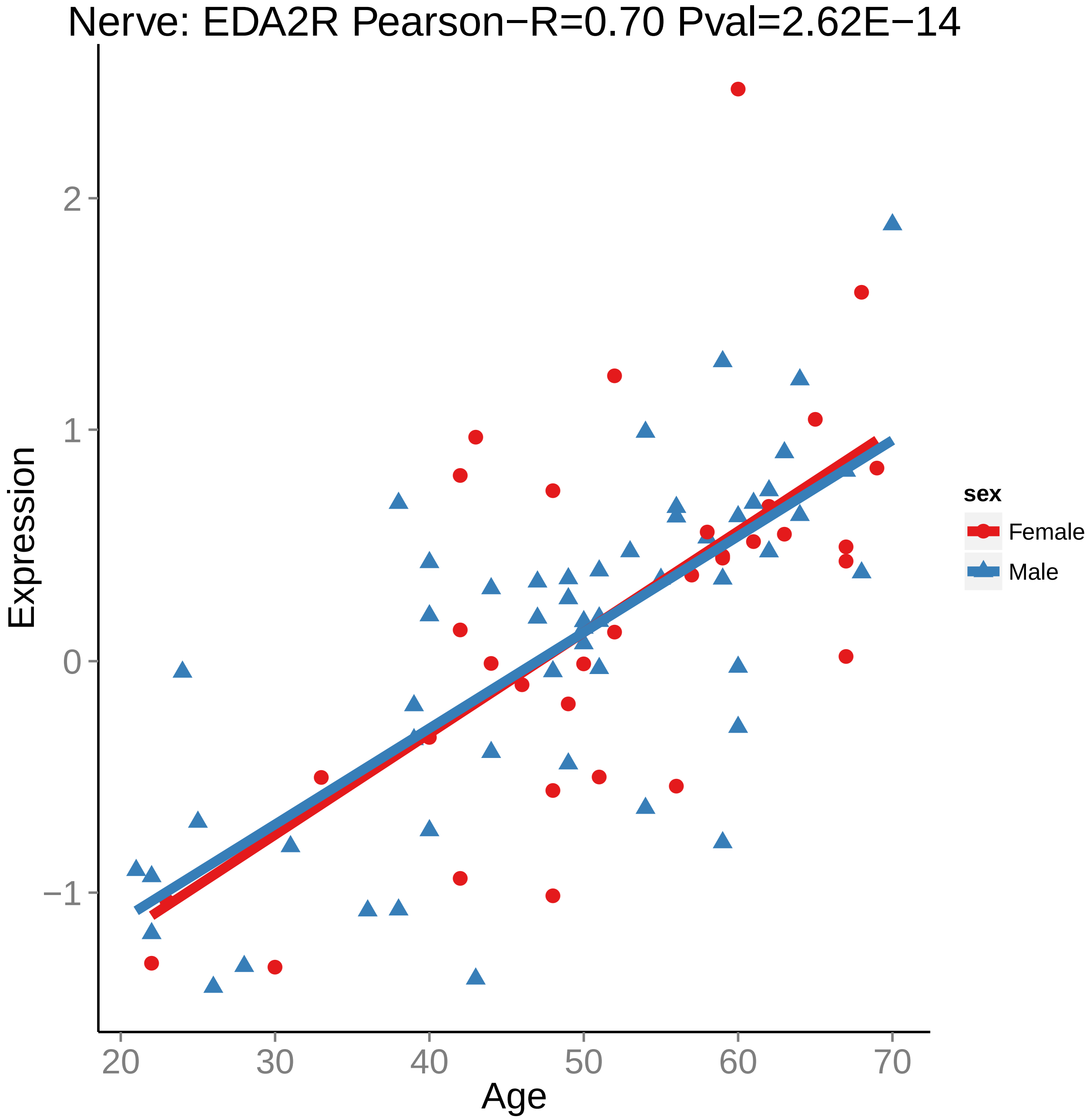
Nerve: EDA2R Pearson−R=0.70 Pval=2.62E−14
Expression
2
1
0
−1
20
30
40
50
60
70
Age
sex
Female
Male

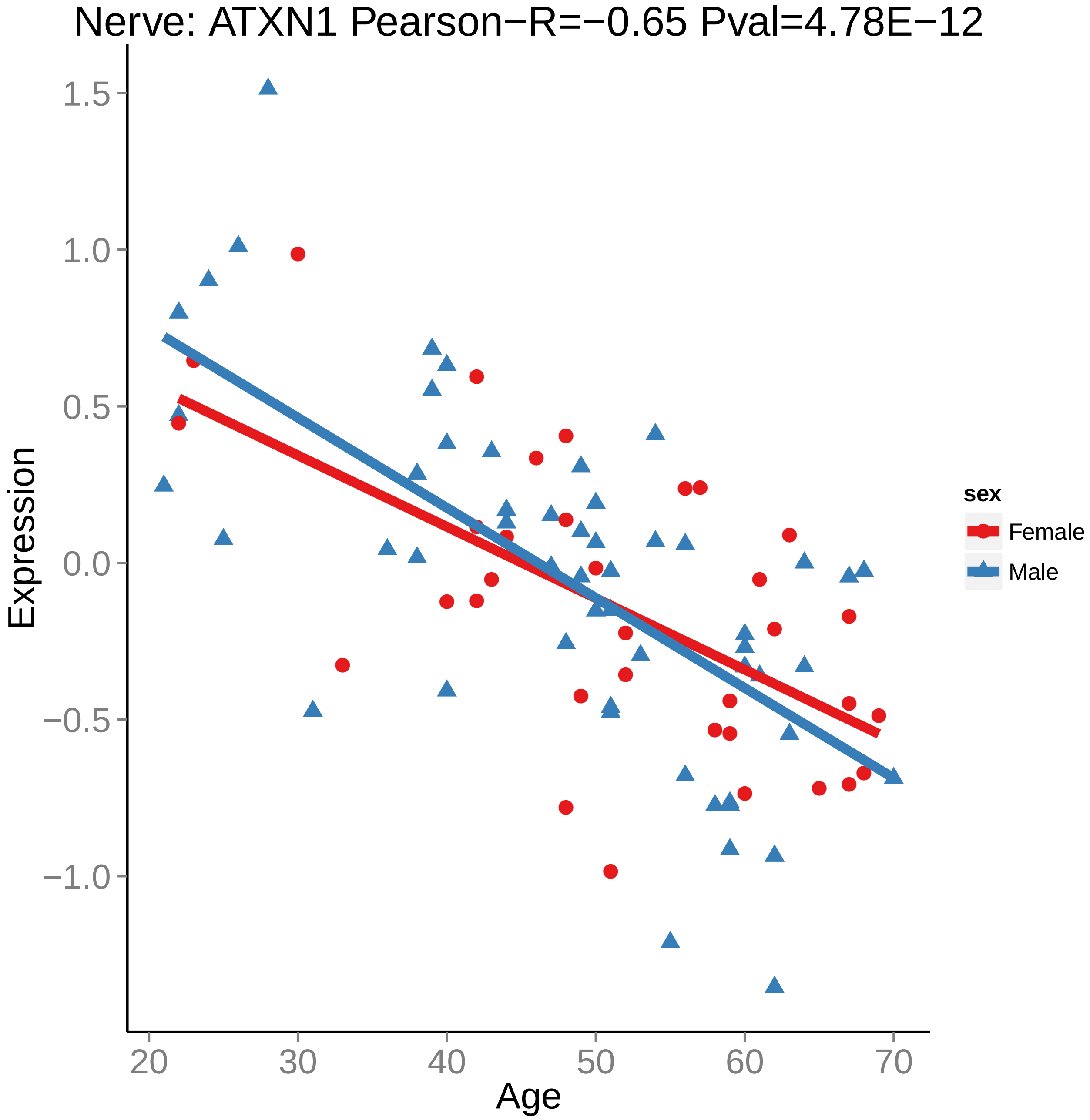

Nerve: ATXN1 Pearson−R=−0.65 Pval=4.78E−12
Expression
Age
1.5
1.0
0.5
0.0
−0.5
−1.0
20
30
40
50
60
70
sex
Female
Male

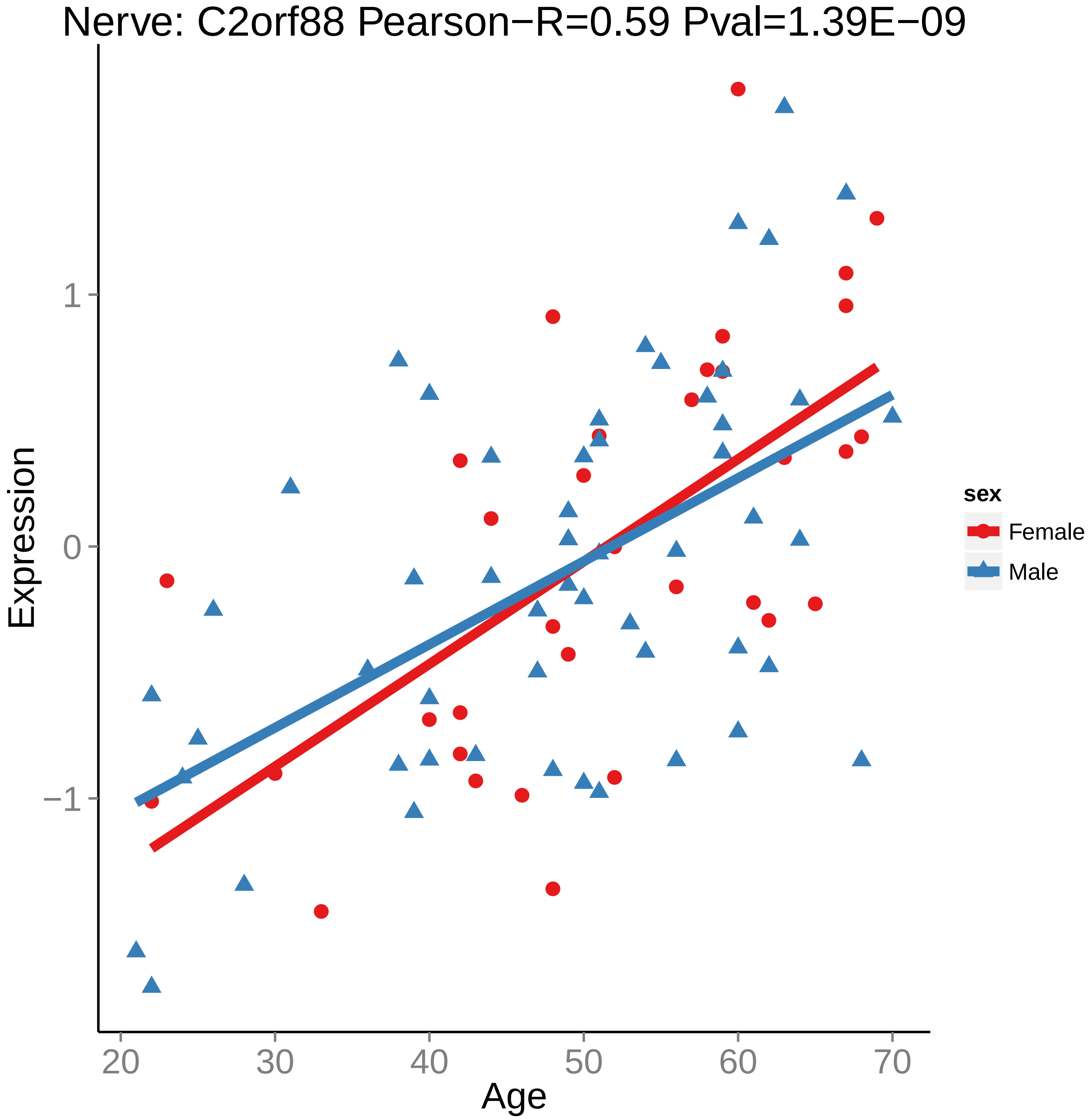

Nerve: C2orf88 Pearson−R=0.59 Pval=1.39E−09
Expression
1
0
−1
20
30
40
50
60
70
Age
sex
Female
Male

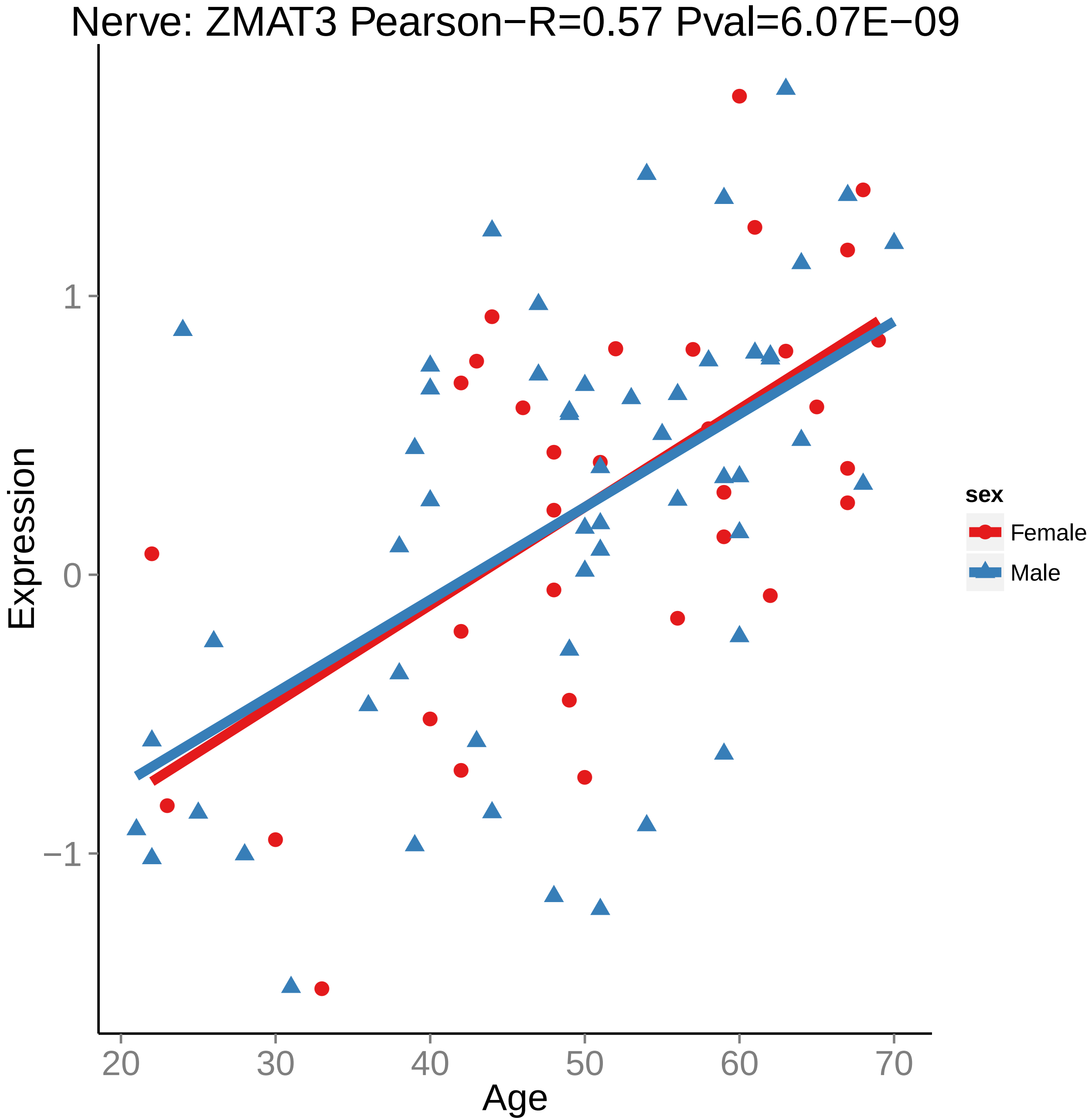
Nerve: ZMAT3 Pearson−R=0.57 Pval=6.07E−09
Expression
1
0
−1
20
30
40
50
60
70
Age
sex
Female
Male

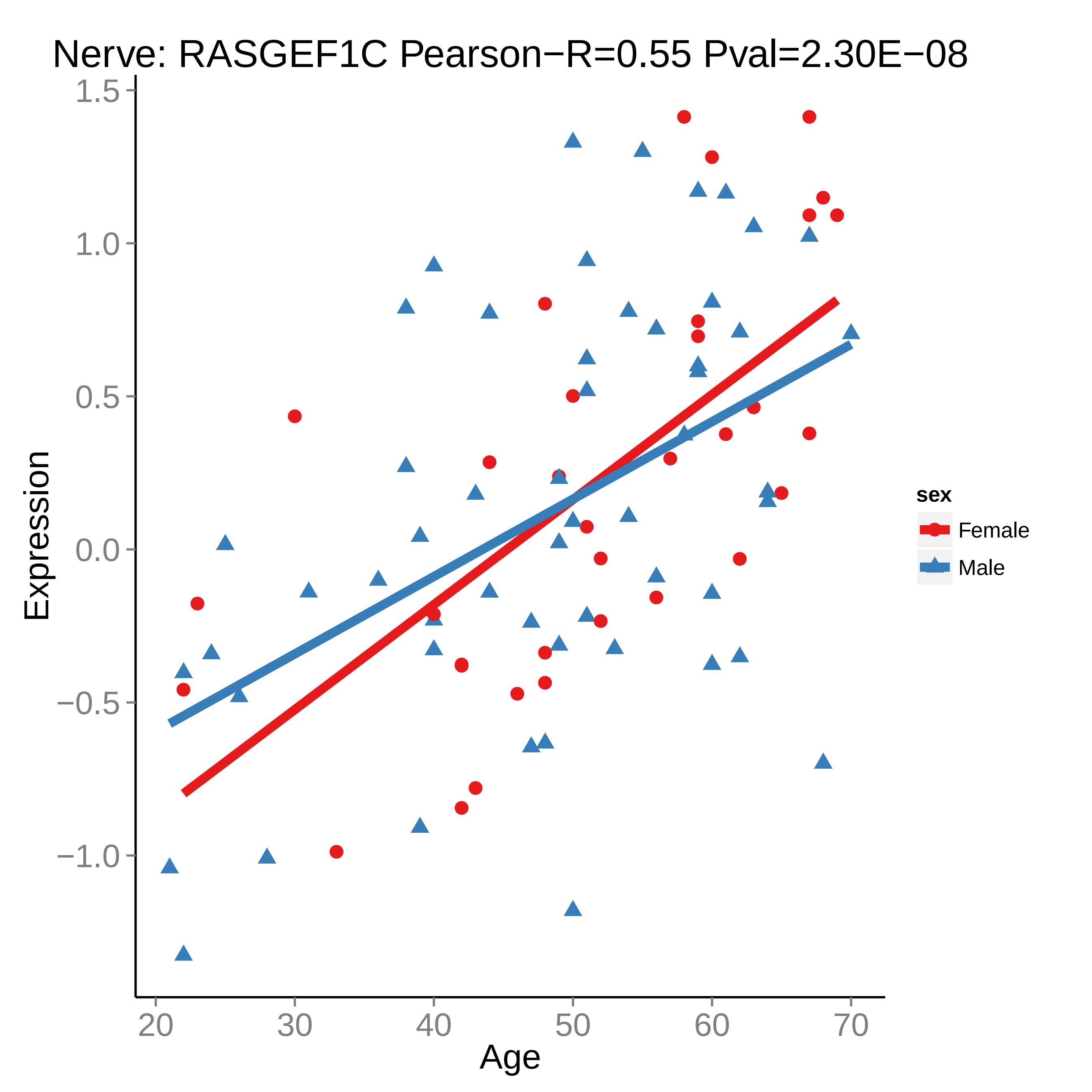

Nerve: RASGEF1C Pearson−R=0.55 Pval=2.30E−08
Expression
Age
1.5
1.0
0.5
0.0
−0.5
−1.0
20
30
40
50
60
70
sex
Female
Male

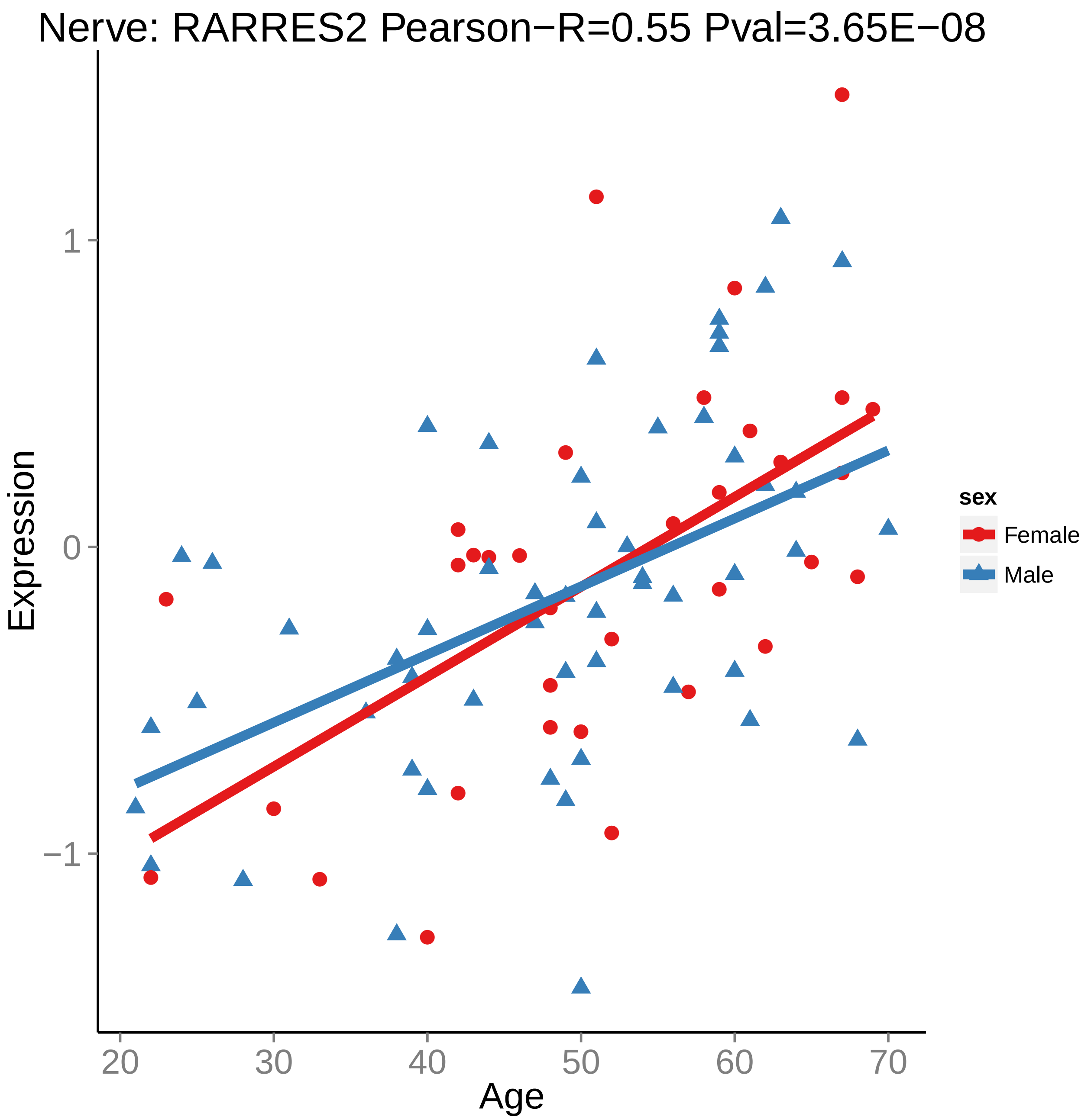

Nerve: RARRES2 Pearson−R=0.55 Pval=3.65E−08
Expression
1
0
−1
20
30
40
50
60
70
Age
sex
Female
Male

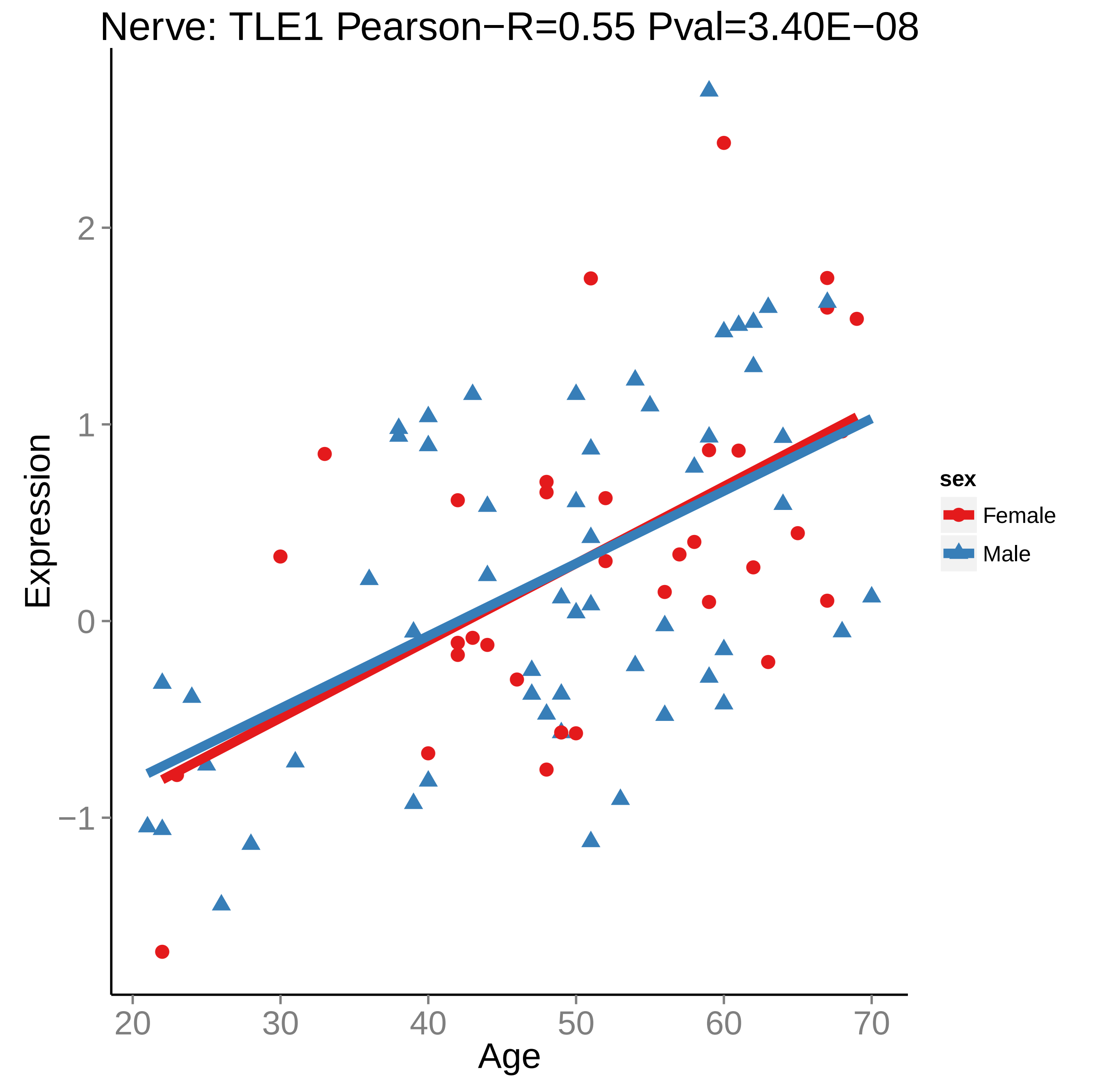

Nerve: TLE1 Pearson−R=0.55 Pval=3.40E−08
Expression
2
1
0
−1
20
30
40
50
60
70
Age
sex
Female
Male

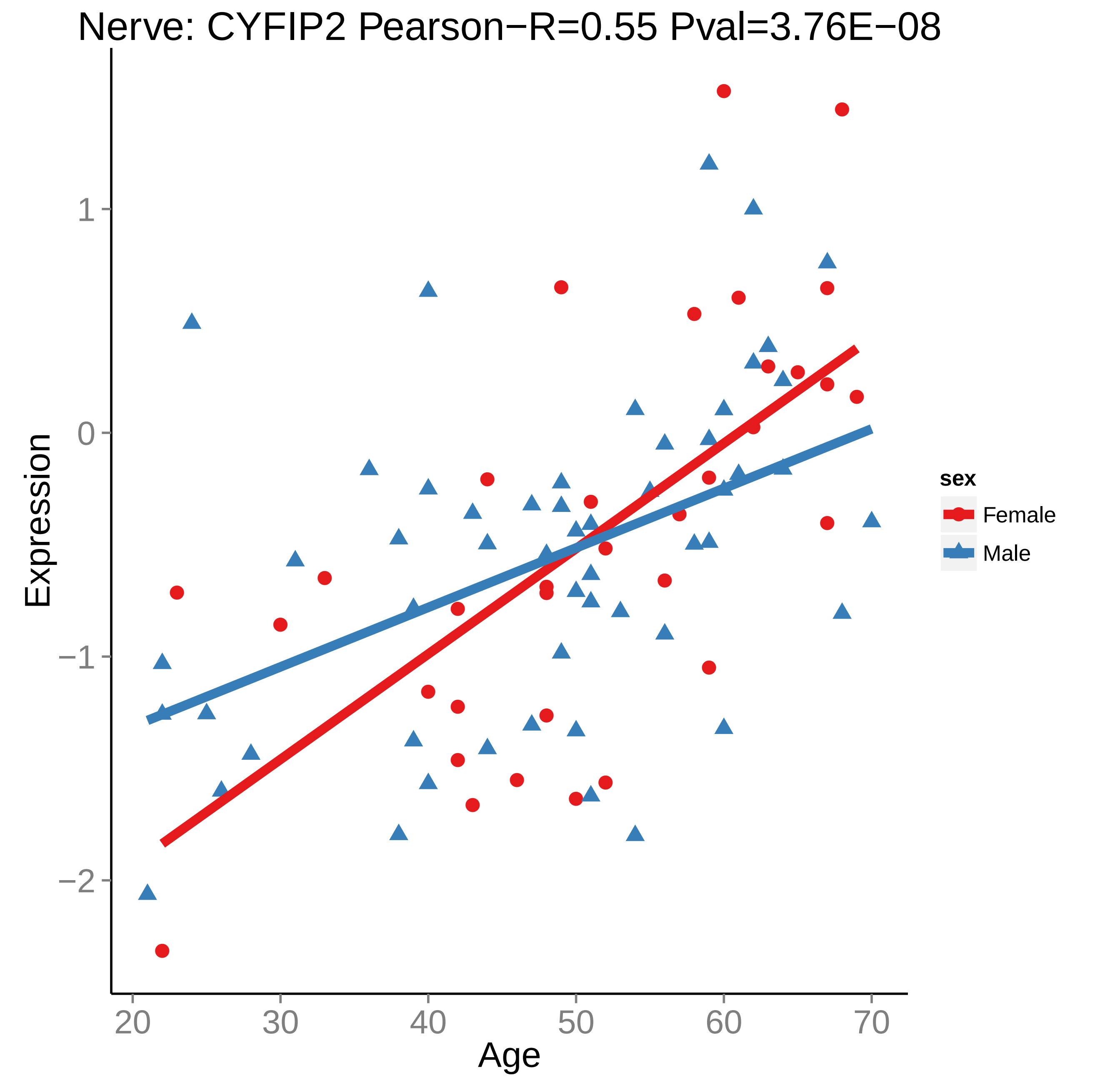

Nerve: CYFIP2 Pearson−R=0.55 Pval=3.76E−08
Expression
1
0
−1
−2
20
30
40
50
60
70
Age
sex
Female
Male

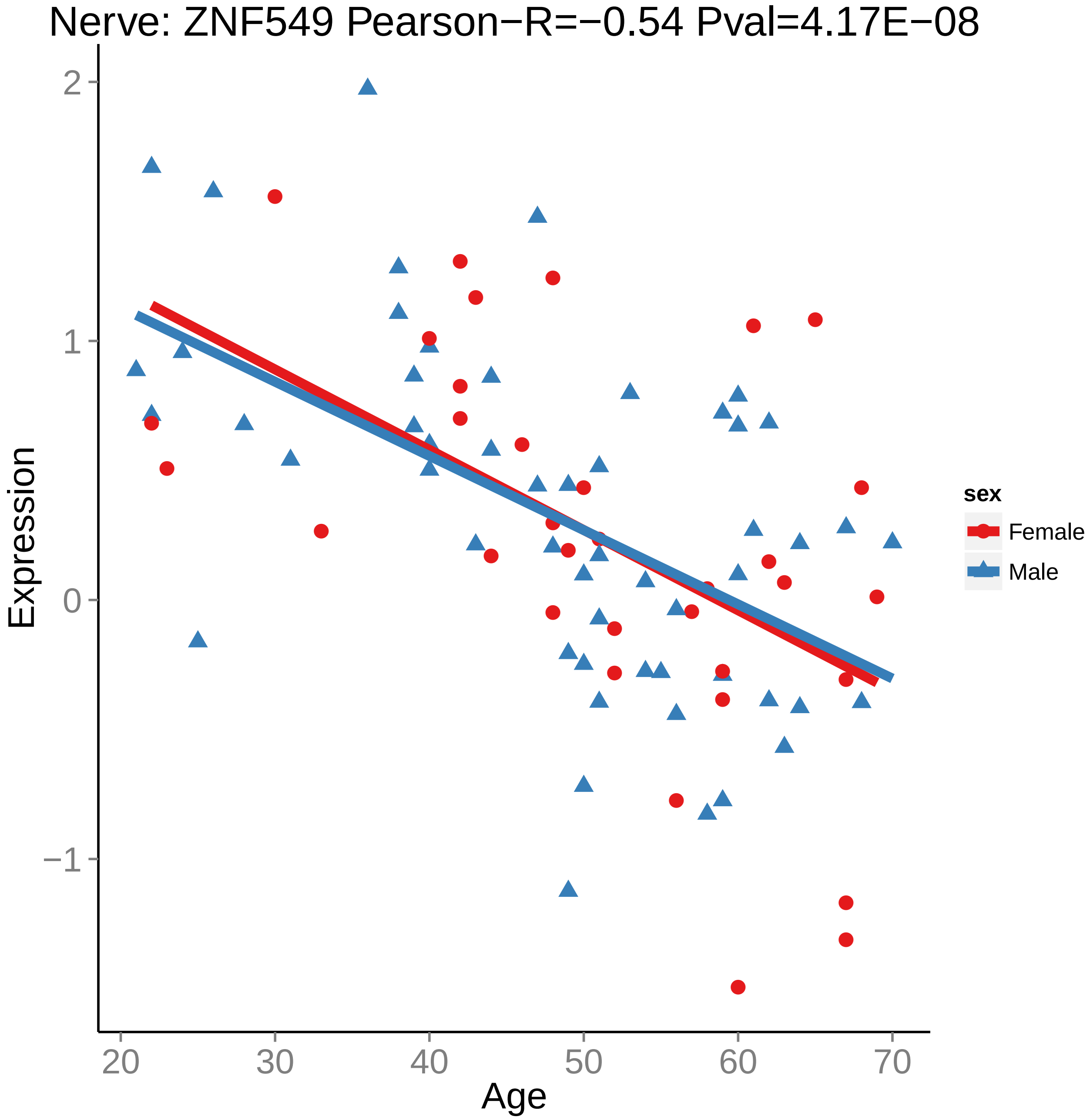

Nerve: ZNF549 Pearson−R=−0.54 Pval=4.17E−08
Expression
Age
2
1
0
−1
20
30
40
50
60
70
sex
Female
Male

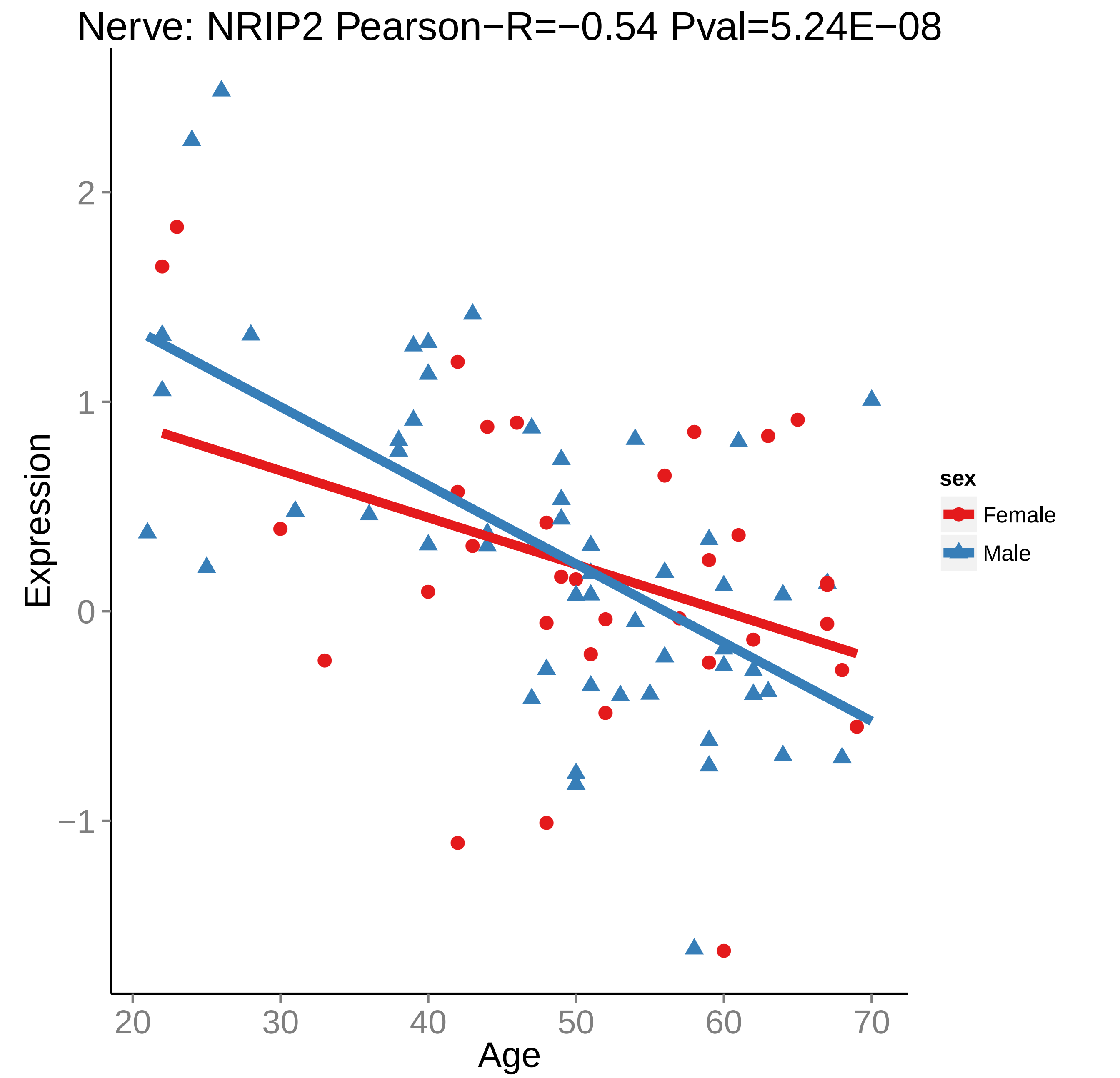

Nerve: NRIP2 Pearson−R=−0.54 Pval=5.24E−08
Expression
2
1
0
−1
20
30
40
50
60
70
Age
sex
Female
Male

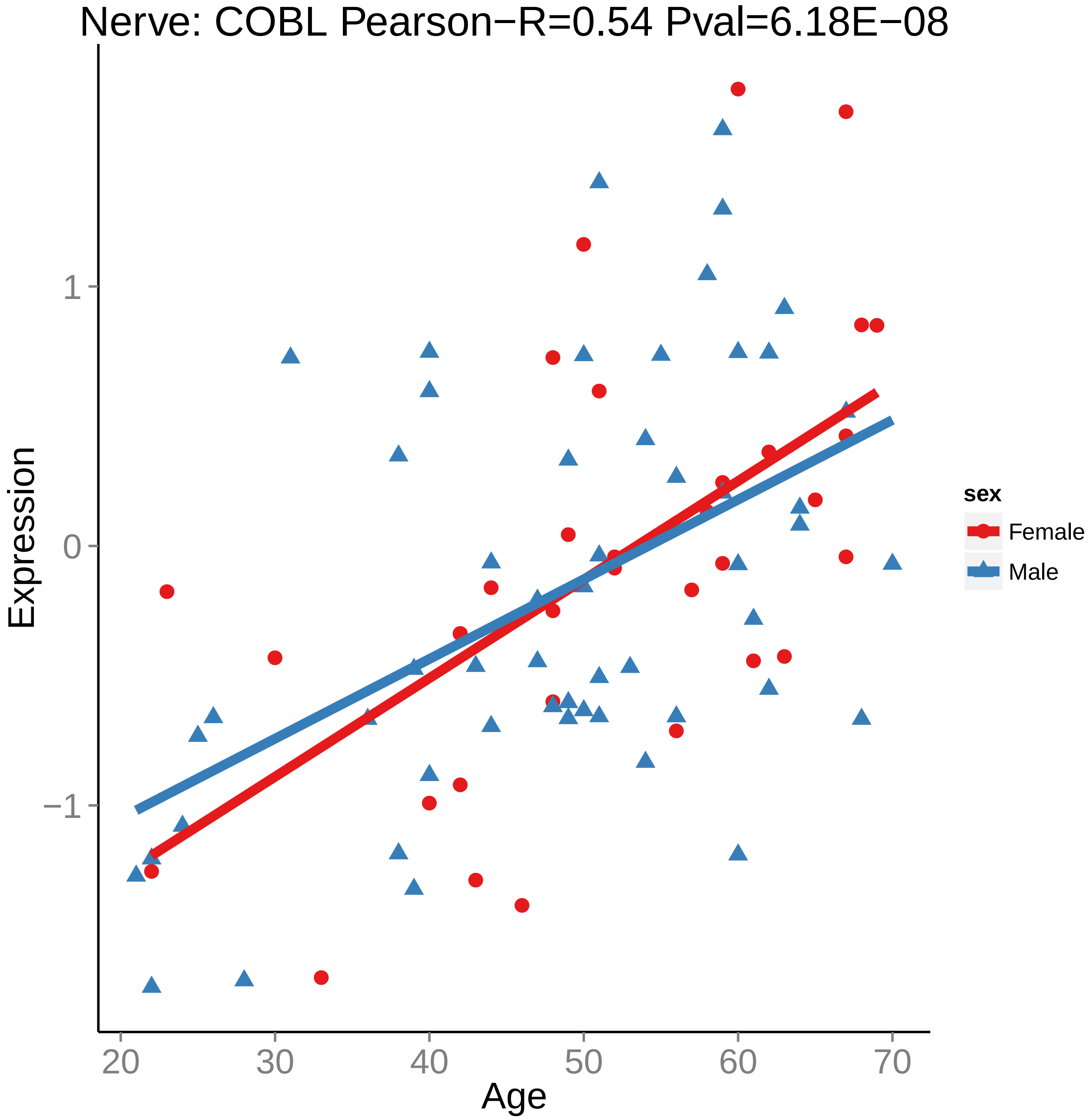

Nerve: COBL Pearson−R=0.54 Pval=6.18E−08
Expression
1
0
−1
20
30
40
50
60
70
Age
sex
Female
Male

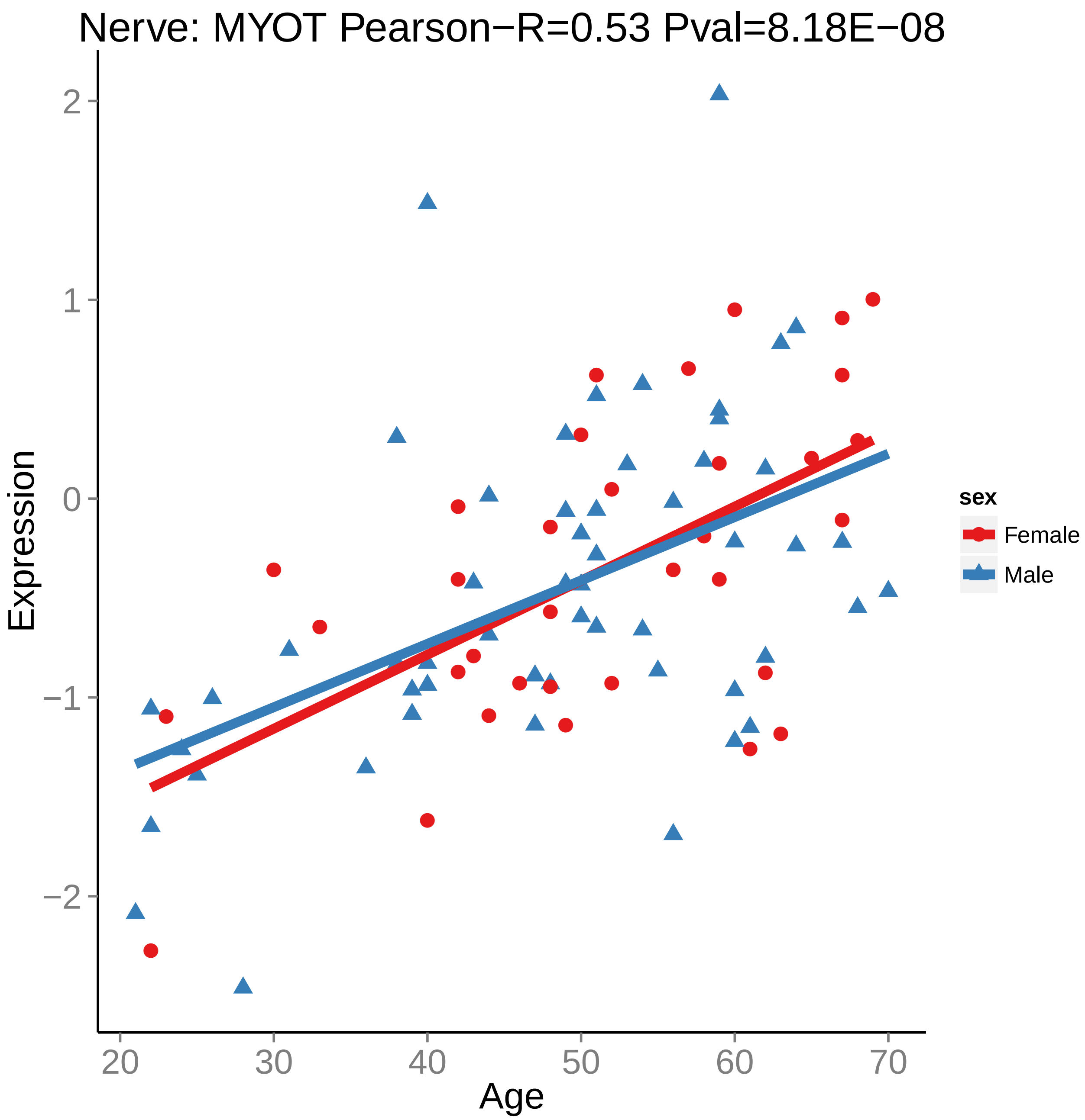

Nerve: MYOT Pearson−R=0.53 Pval=8.18E−08
Expression
Age
2
1
0
−1
−2
20
30
40
50
60
70
sex
Female
Male

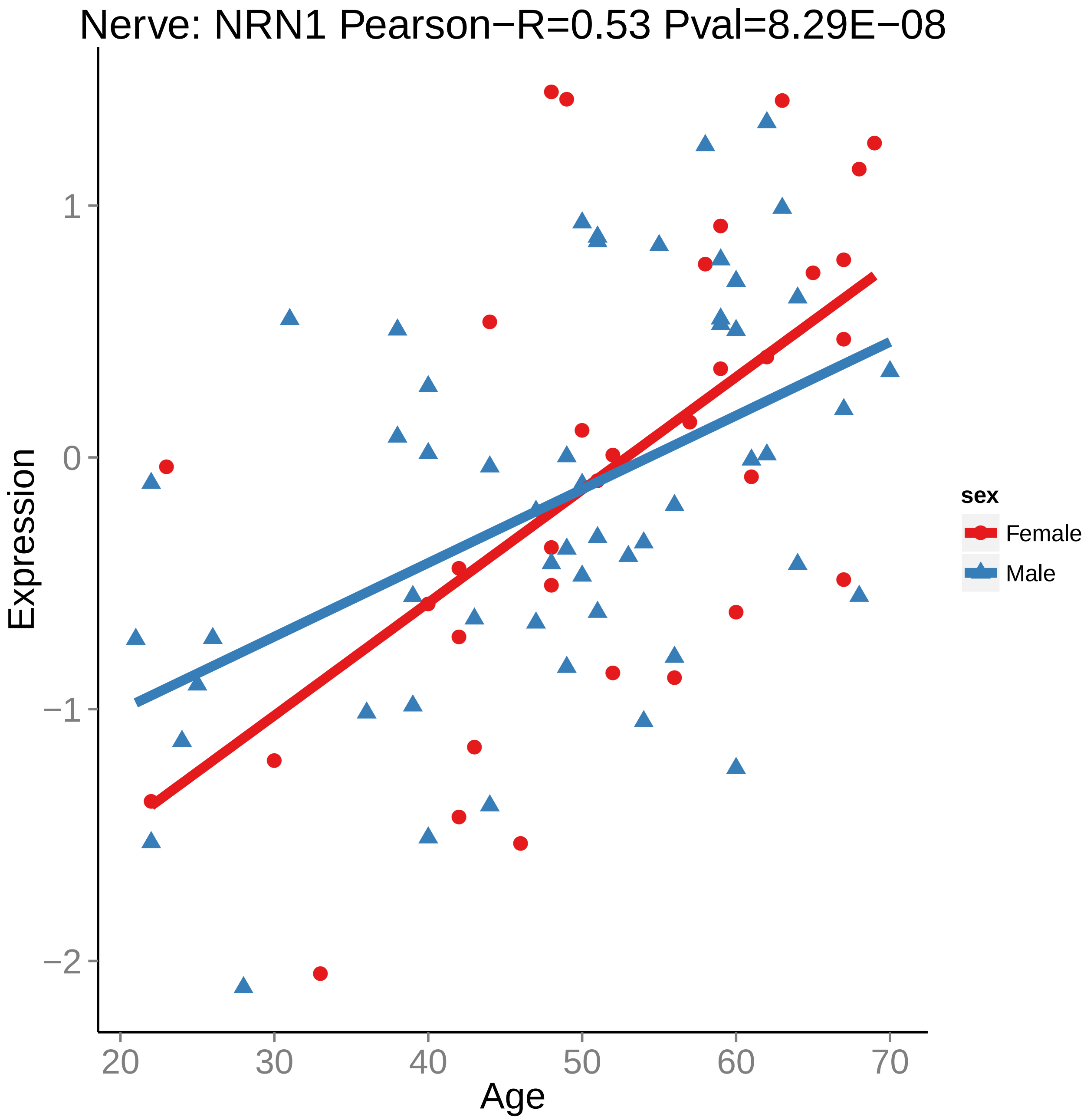

Nerve: NRN1 Pearson−R=0.53 Pval=8.29E−08
Expression
1
0
−1
−2
20
30
40
50
60
70
Age
sex
Female
Male

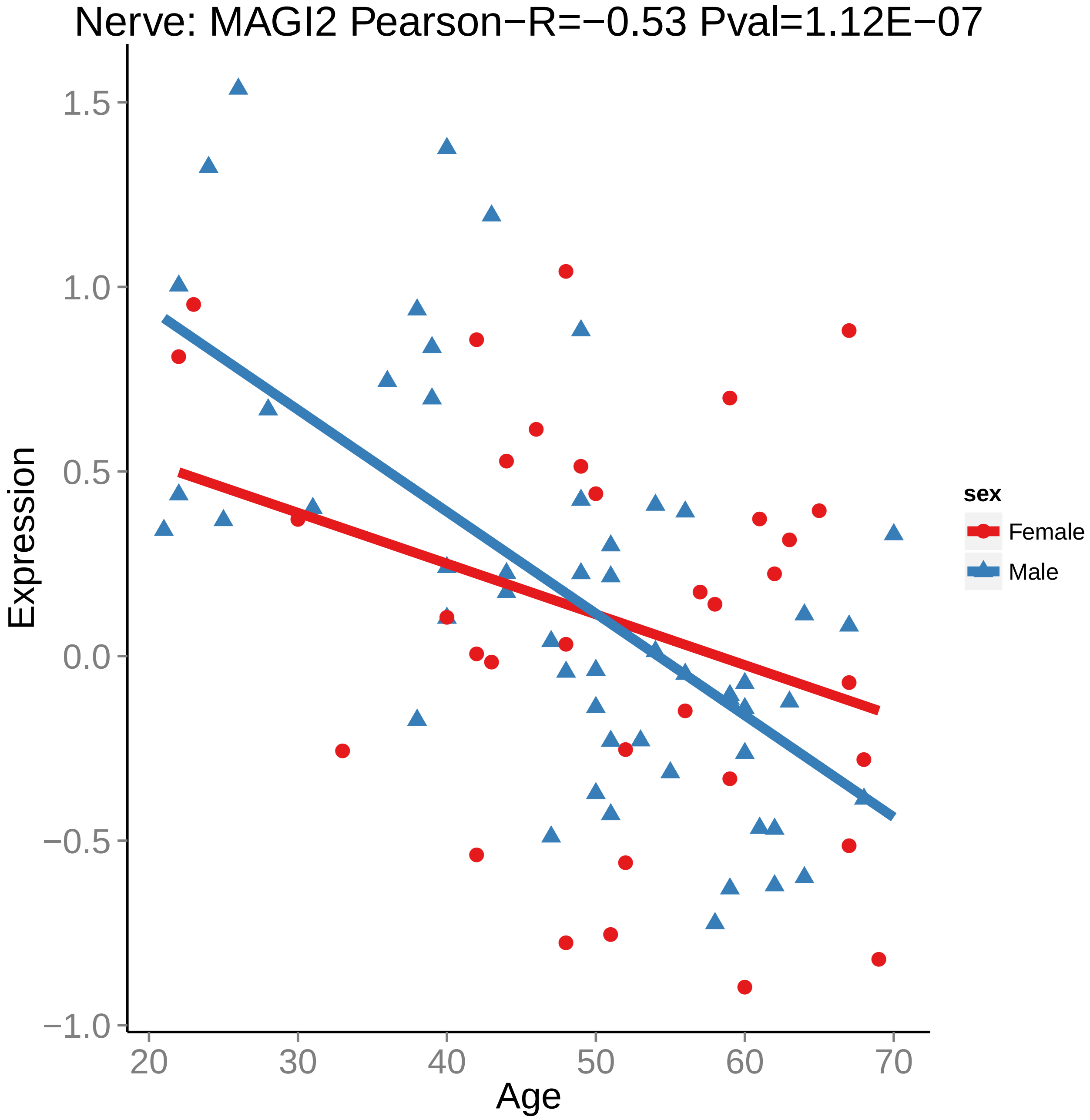

Nerve: MAGI2 Pearson−R=−0.53 Pval=1.12E−07
Expression
1.5
1.0
0.5
0.0
−0.5
−1.0
20
30
40
50
60
70
Age
sex
Female
Male

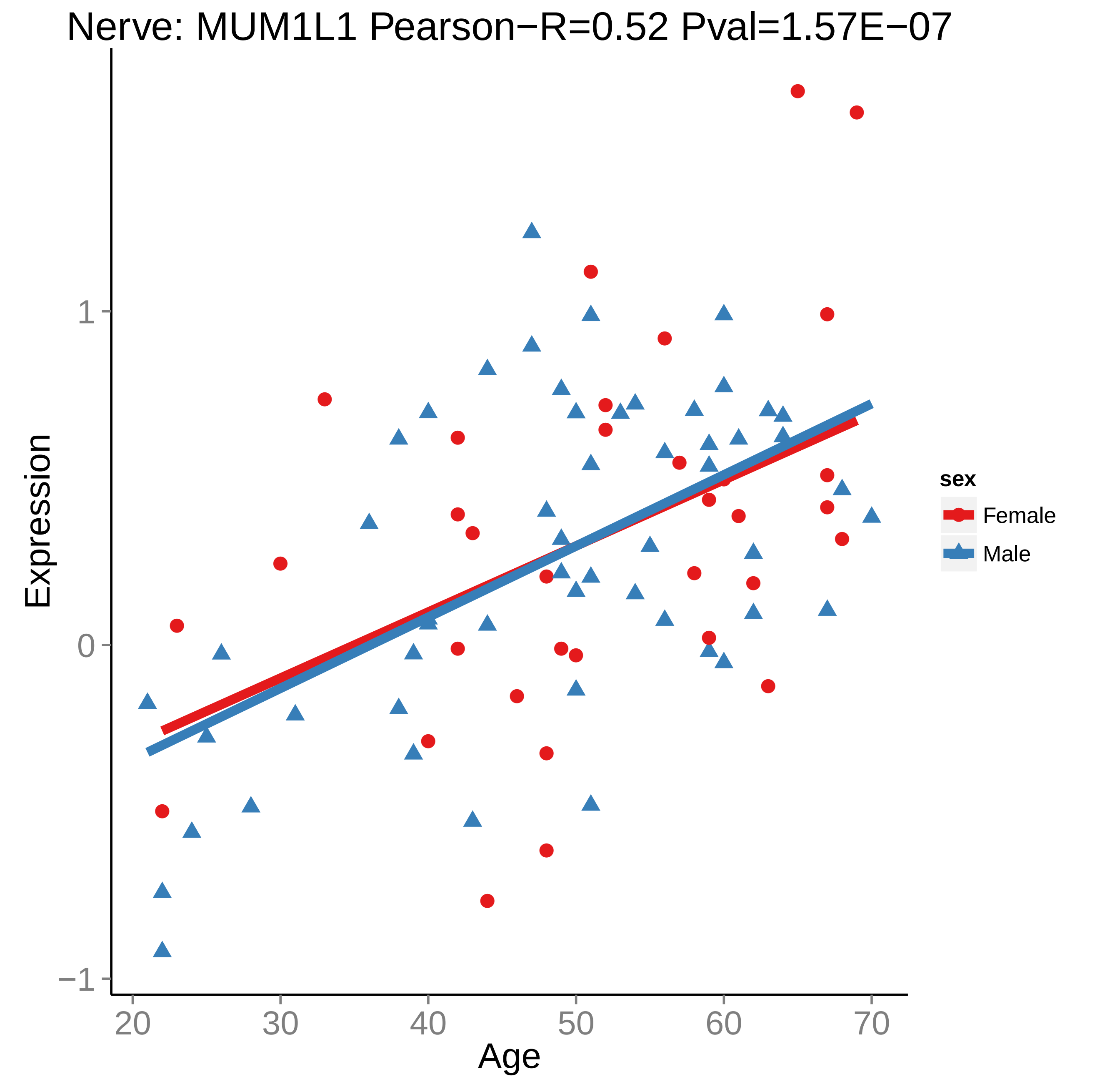

Nerve: MUM1L1 Pearson−R=0.52 Pval=1.57E−07
Expression
1
0
−1
20
30
40
50
60
70
Age
sex
Female
Male

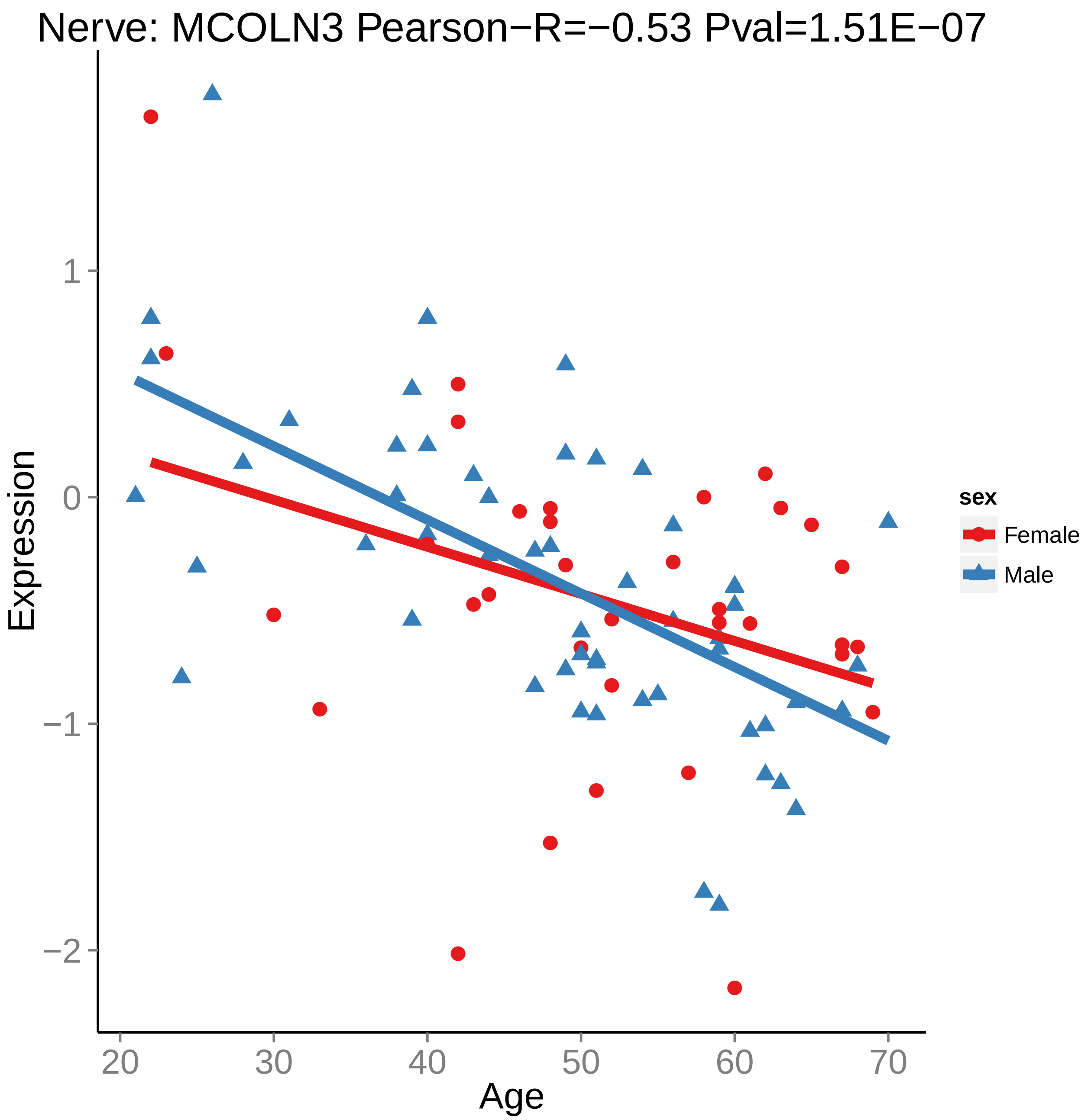

Nerve: MCOLN3 Pearson−R=−0.53 Pval=1.51E−07
Expression
1
0
−1
−2
20
30
40
50
60
70
Age
sex
Female
Male

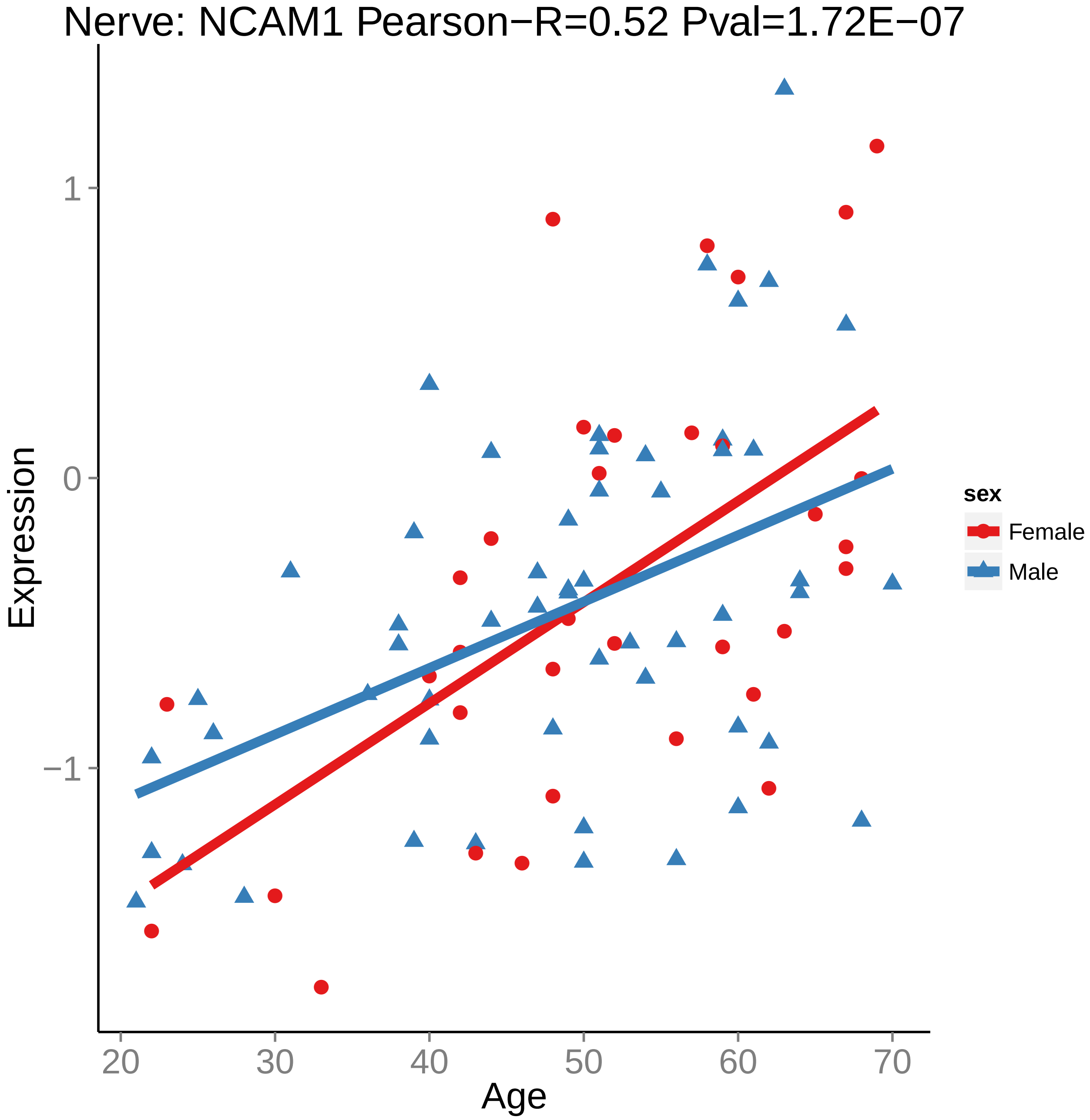

Nerve: NCAM1 Pearson−R=0.52 Pval=1.72E−07
Expression
1
0
−1
20
30
40
50
60
70
Age
sex
Female
Male

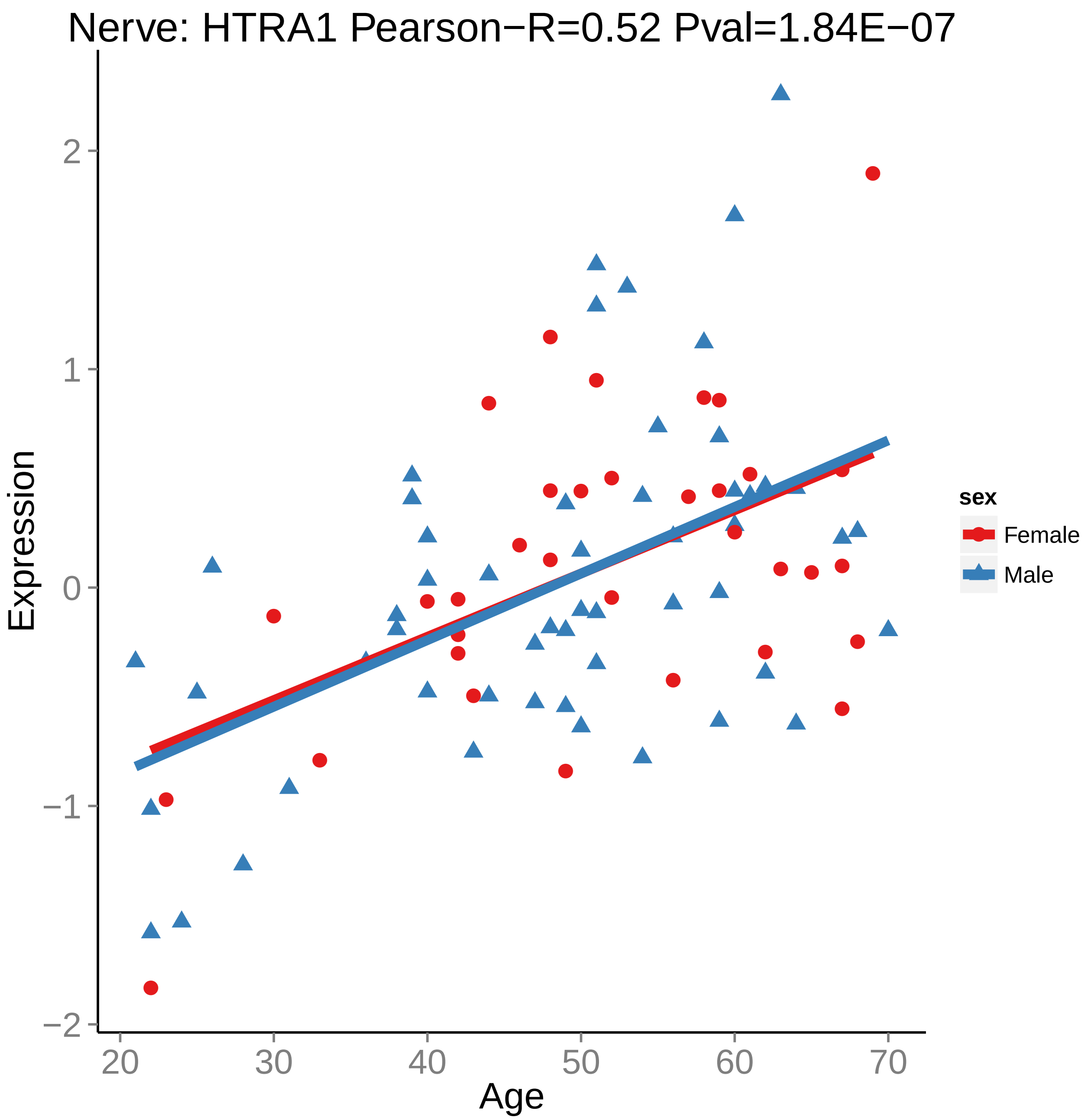
Nerve: HTRA1 Pearson−R=0.52 Pval=1.84E−07
Expression
2
1
0
−1
−2
20
30
40
50
60
70
Age
sex
Female
Male

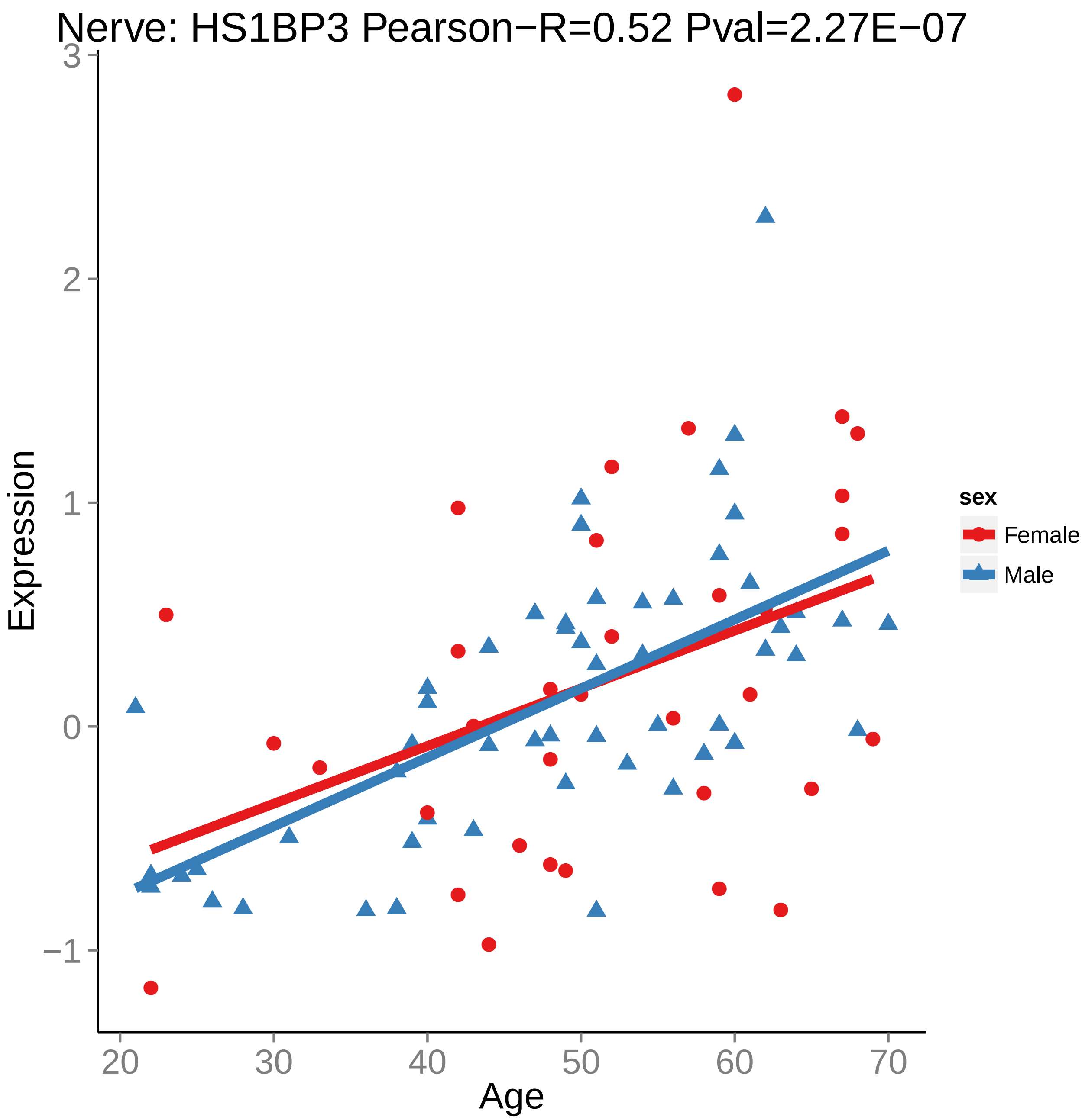
Nerve: HS1BP3 Pearson−R=0.52 Pval=2.27E−07
Expression
Age
3
2
1
0
−1
20
30
40
50
60
70
sex
Female
Male

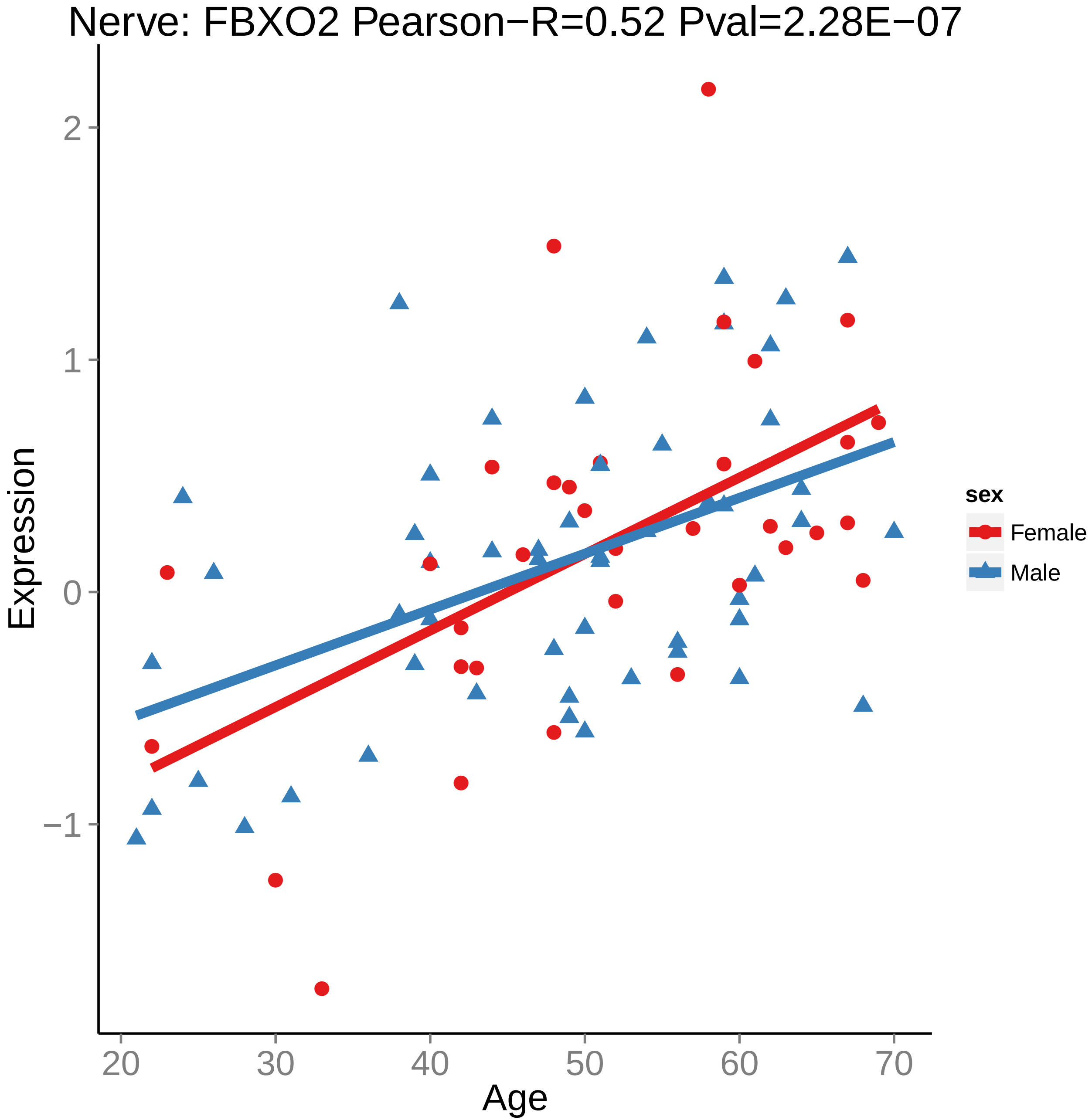

Nerve: FBXO2 Pearson−R=0.52 Pval=2.28E−07
Expression
Age
2
1
0
−1
20
30
40
50
60
70
sex
Female
Male

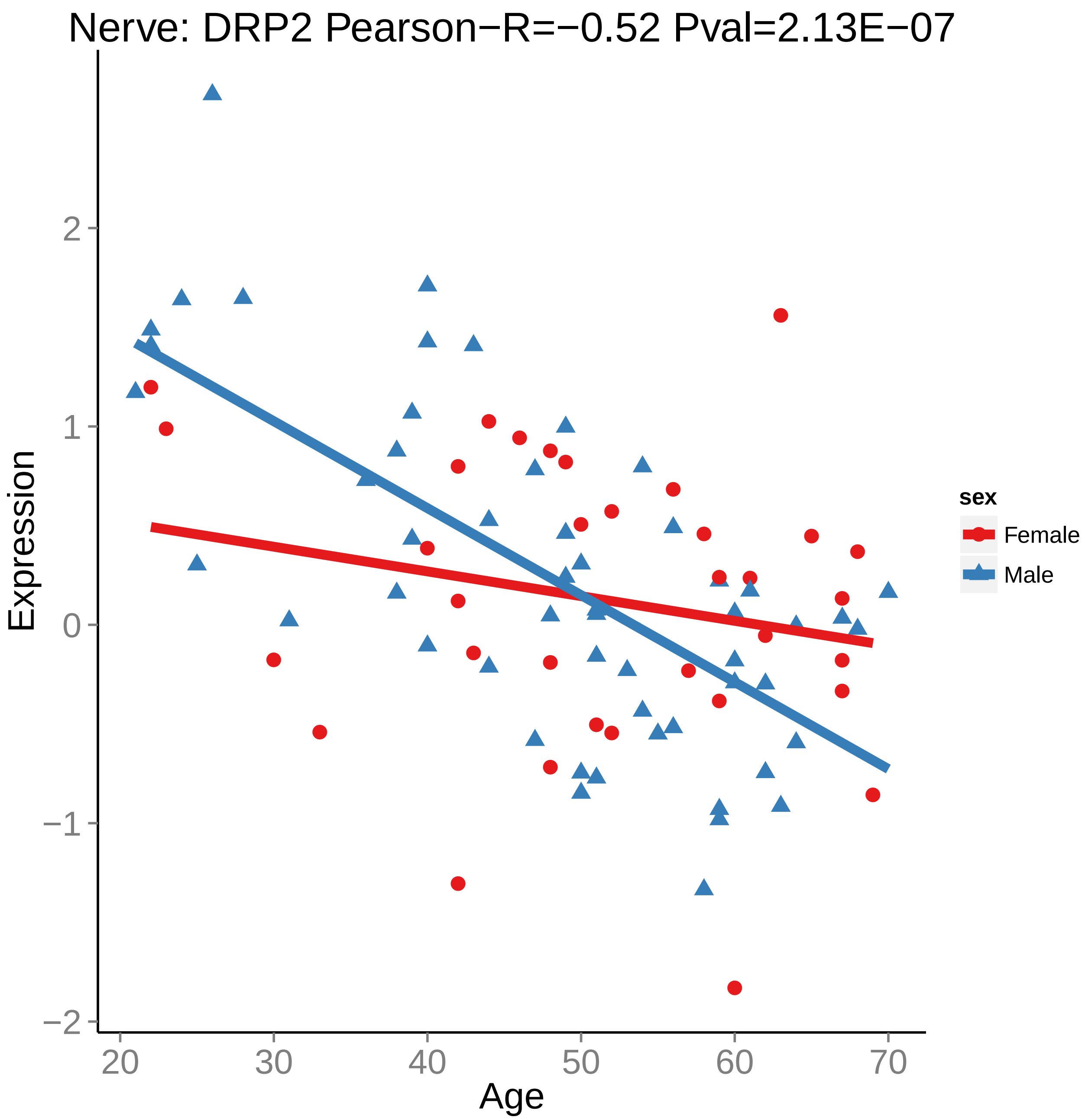

Nerve: DRP2 Pearson−R=−0.52 Pval=2.13E−07
Expression
2
1
0
−1
−2
20
30
40
50
60
70
Age
sex
Female
Male

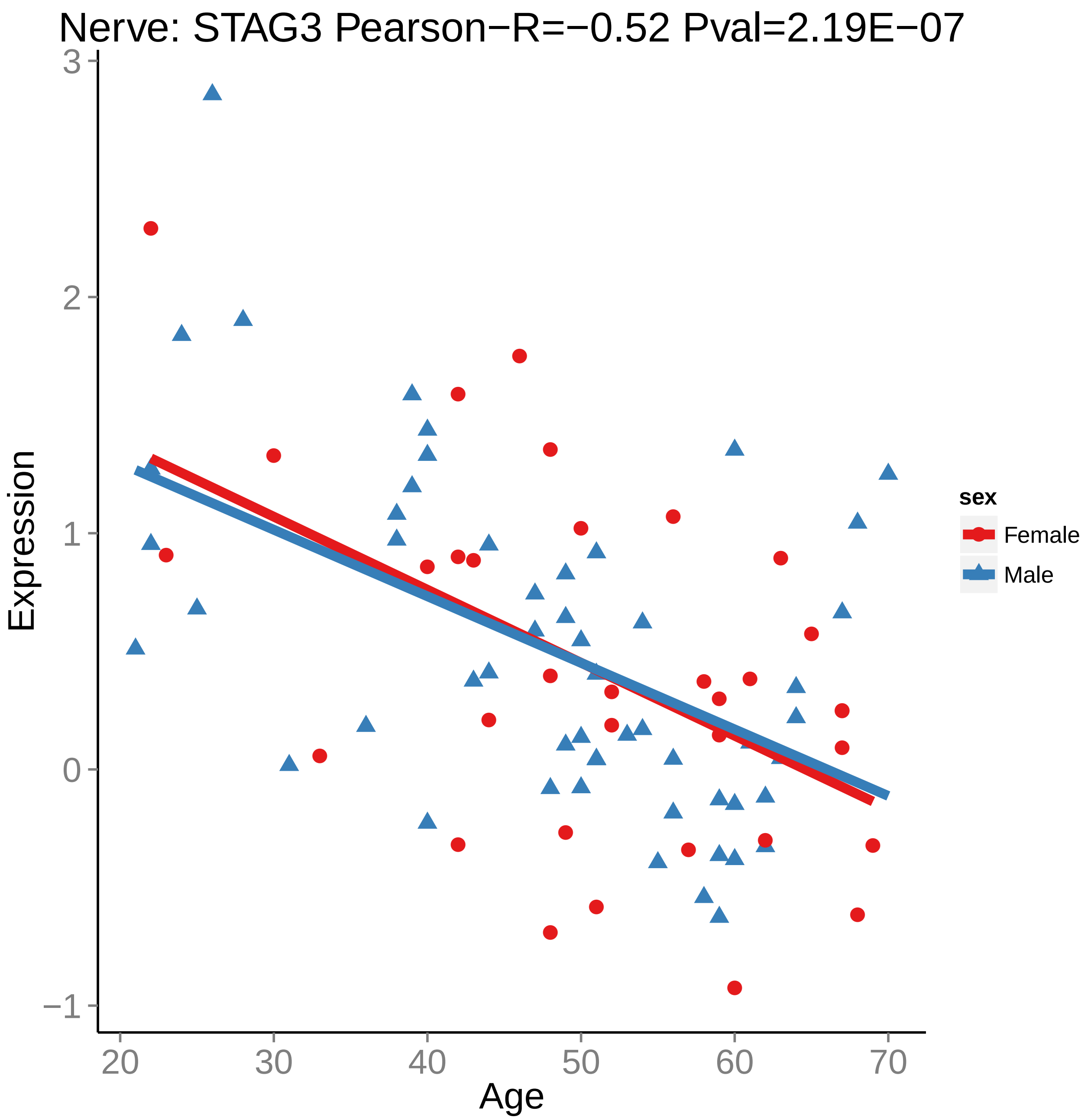

Nerve: STAG3 Pearson−R=−0.52 Pval=2.19E−07
3
2
1
0
−1
Expression
20
30
40
50
60
70
Age
sex
Female
Male

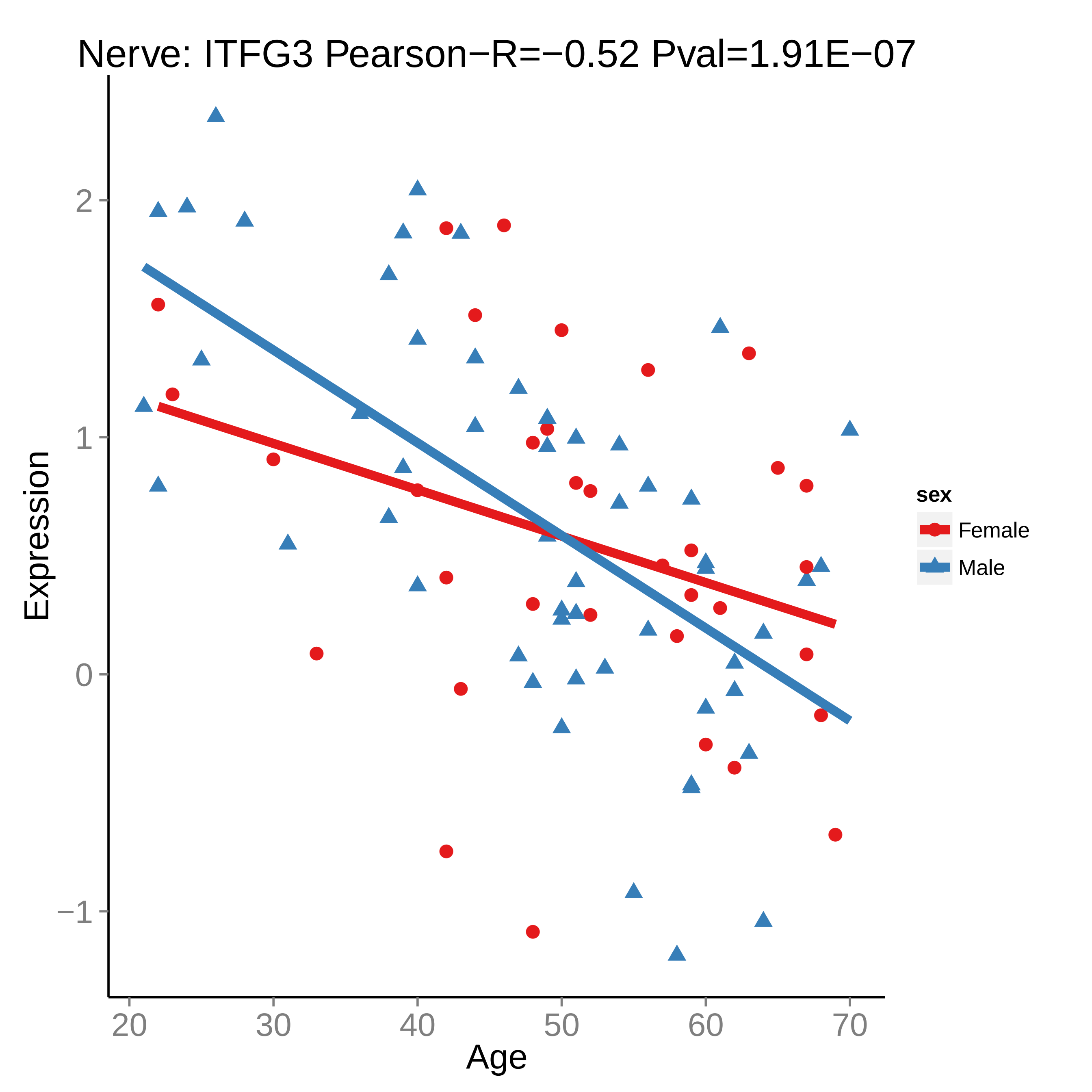

Nerve: ITFG3 Pearson−R=−0.52 Pval=1.91E−07
Expression
Age
2
1
0
−1
20
30
40
50
60
70
sex
Female
Male

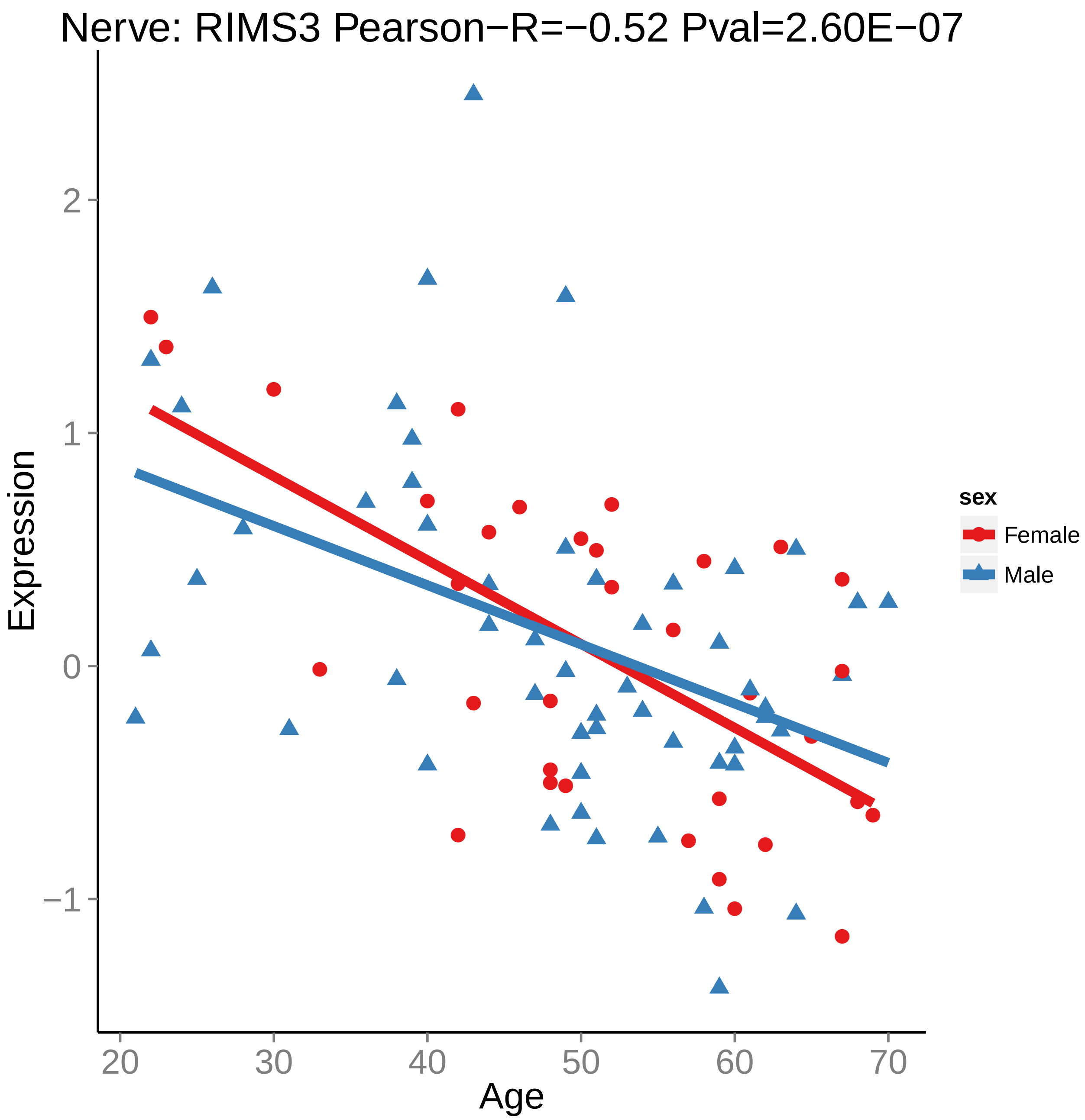
Nerve: RIMS3 Pearson−R=−0.52 Pval=2.60E−07
Expression
Age
2
1
0
−1
20
30
40
50
60
70
sex
Female
Male

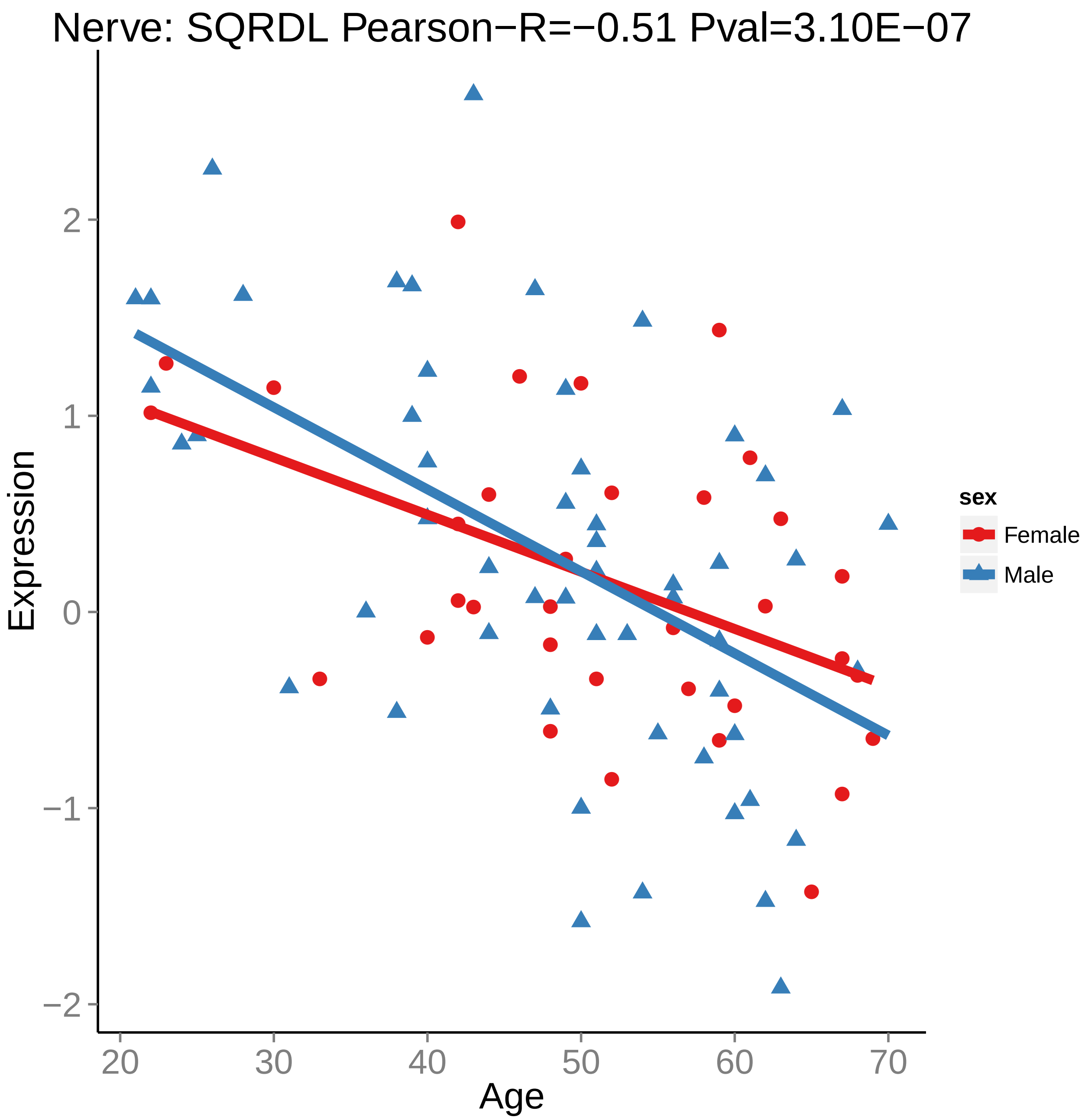
Nerve: SQRDL Pearson−R=−0.51 Pval=3.10E−07
Expression
2
1
0
−1
−2
20
30
40
50
60
70
Age
sex
Female
Male

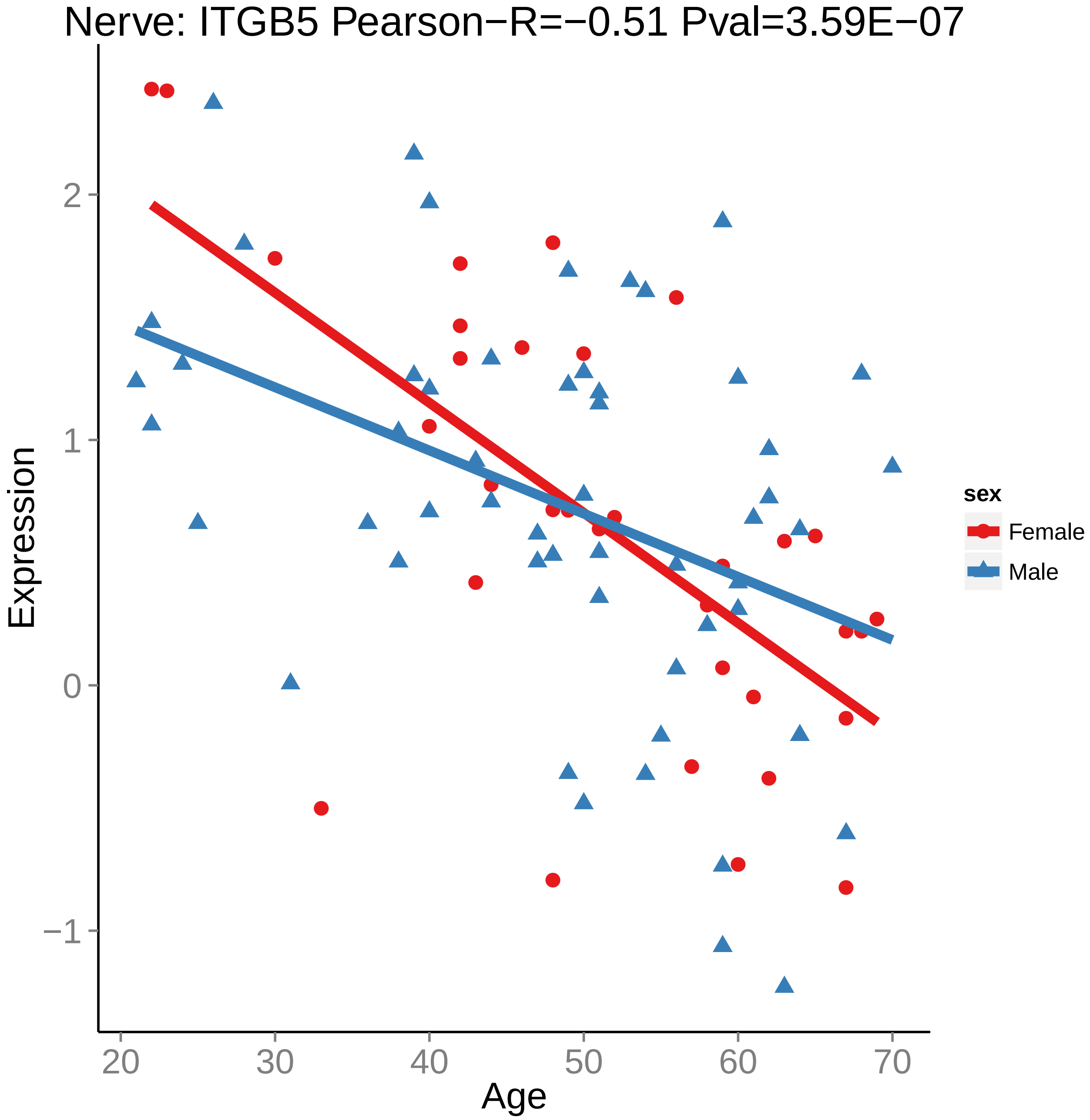

Nerve: ITGB5 Pearson−R=−0.51 Pval=3.59E−07
Expression
2
1
0
−1
20
30
40
50
60
70
Age
sex
Female
Male

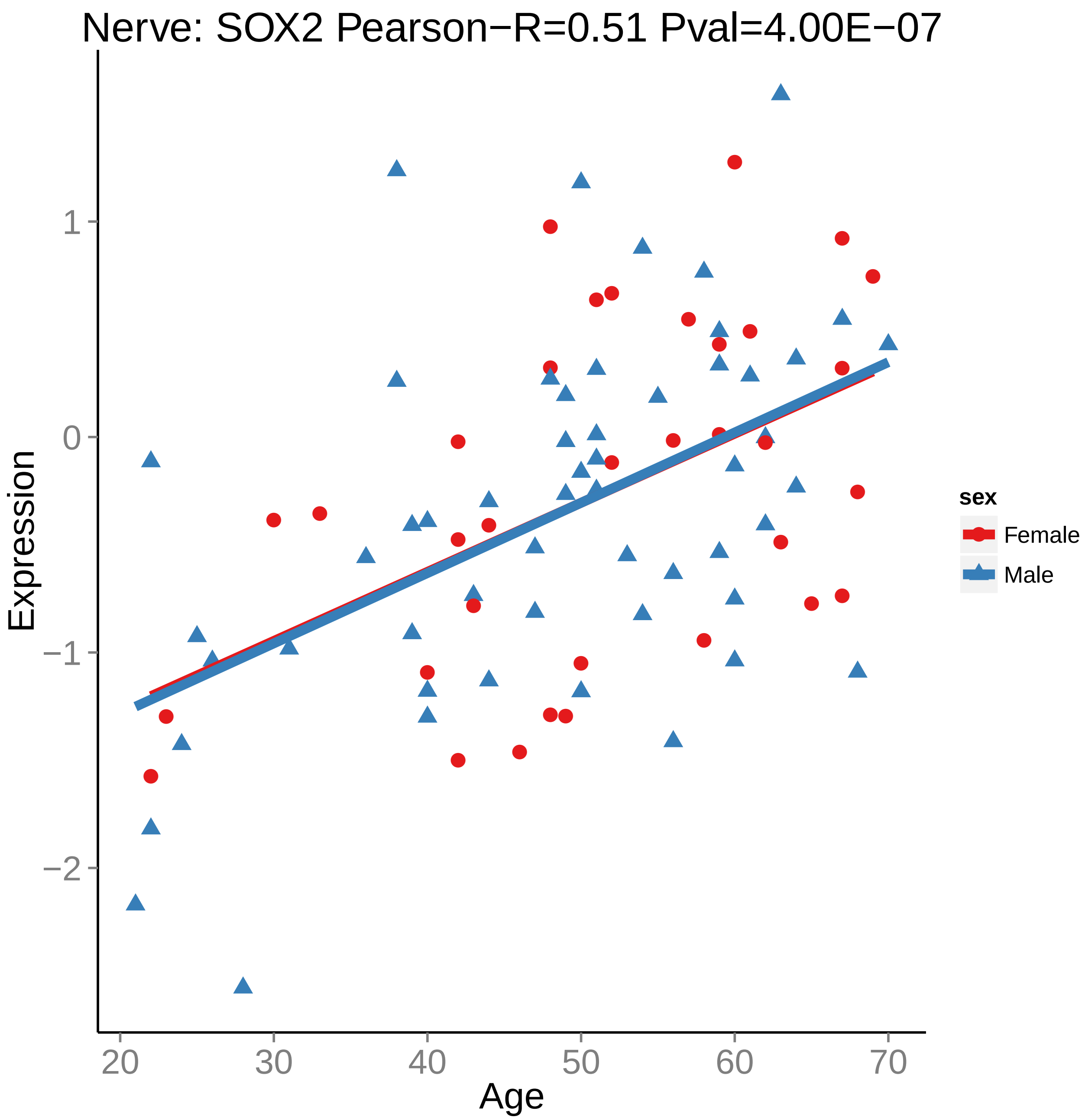

Nerve: SOX2 Pearson−R=0.51 Pval=4.00E−07
Expression
1
0
−1
−2
20
30
40
50
60
70
Age
sex
Female
Male

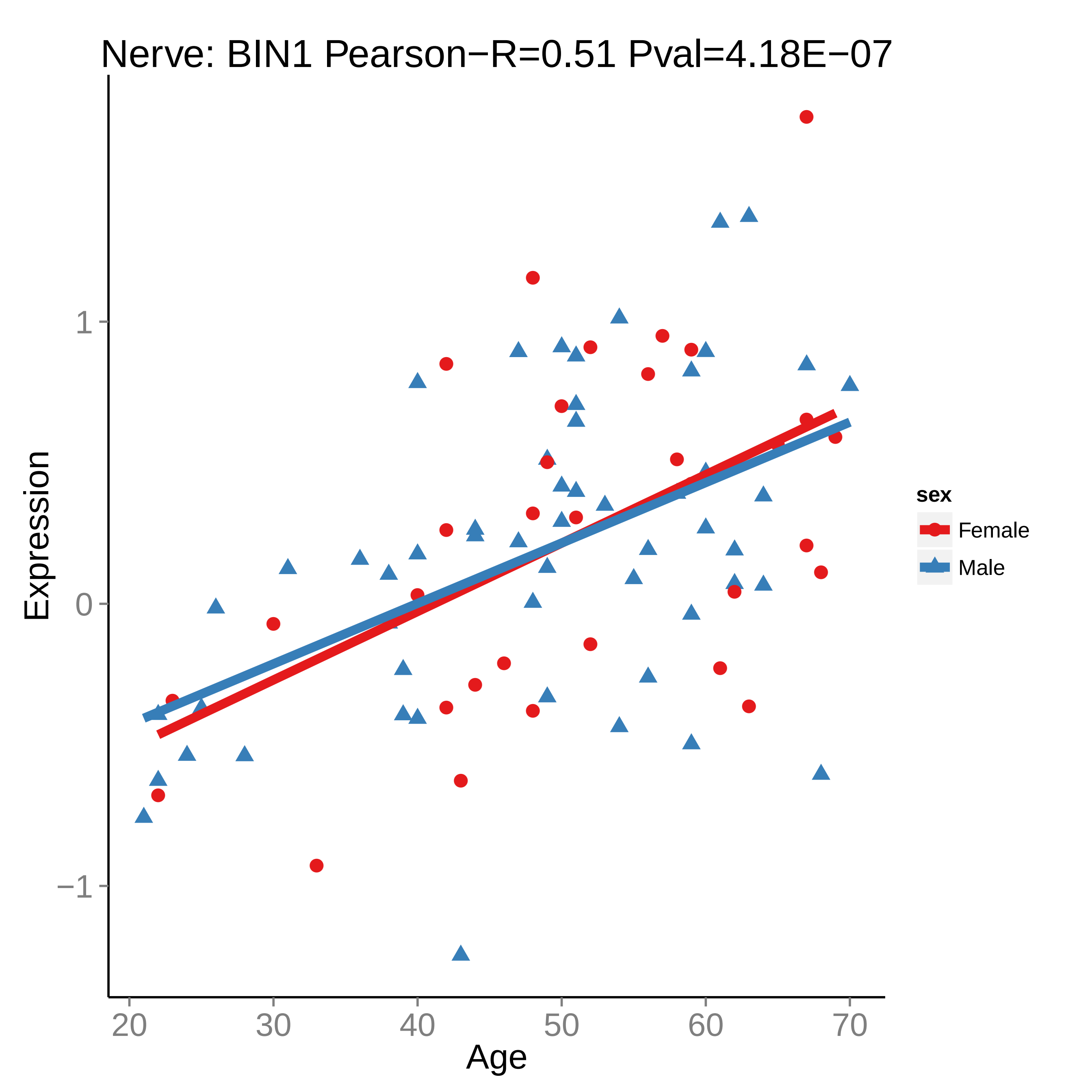
Nerve: BIN1 Pearson−R=0.51 Pval=4.18E−07
Expression
1
0
−1
20
30
40
50
60
70
Age
sex
Female
Male

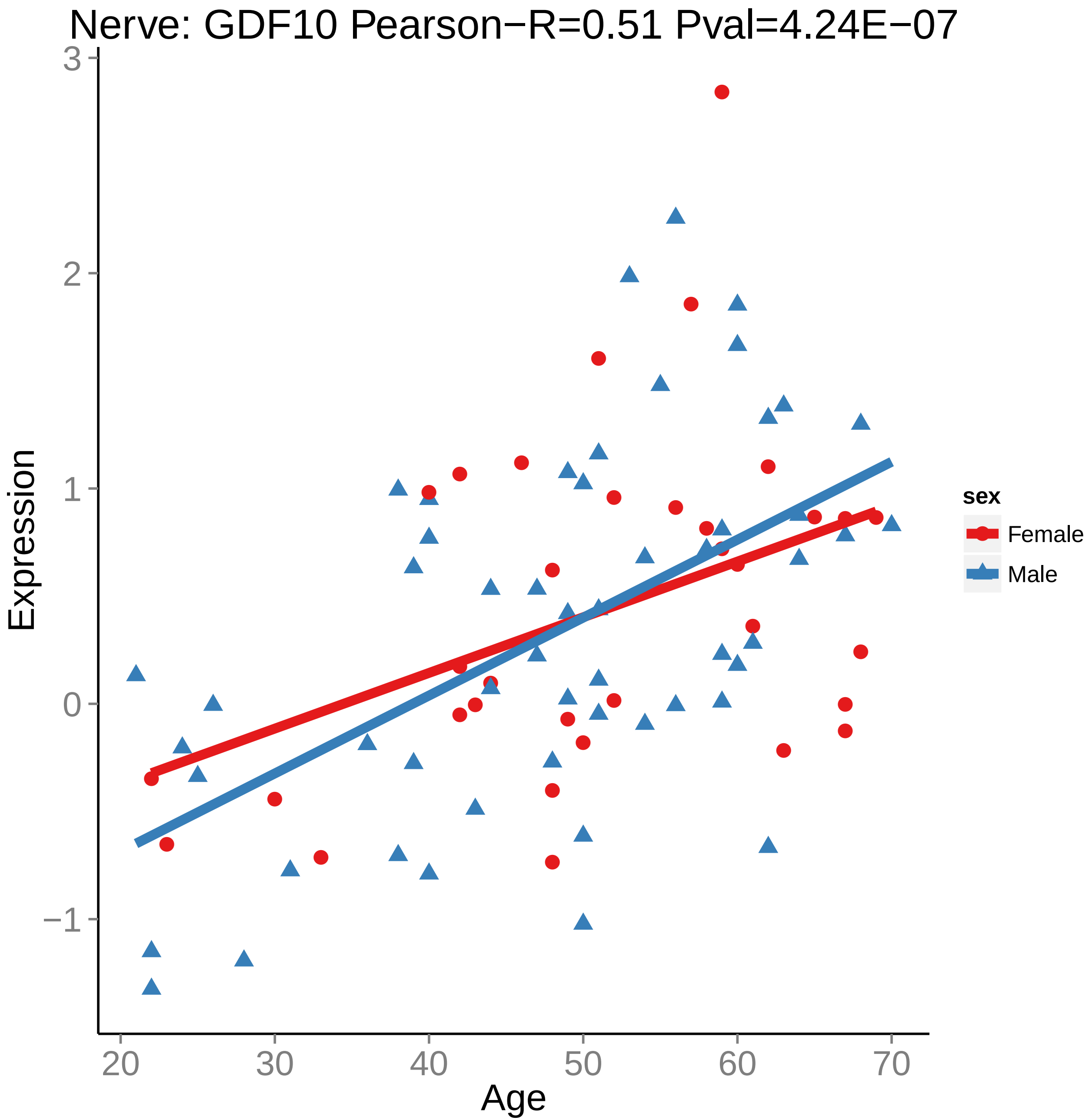

Nerve: GDF10 Pearson−R=0.51 Pval=4.24E−07
Expression
Age
3
2
1
0
−1
20
30
40
50
60
70
sex
Female
Male

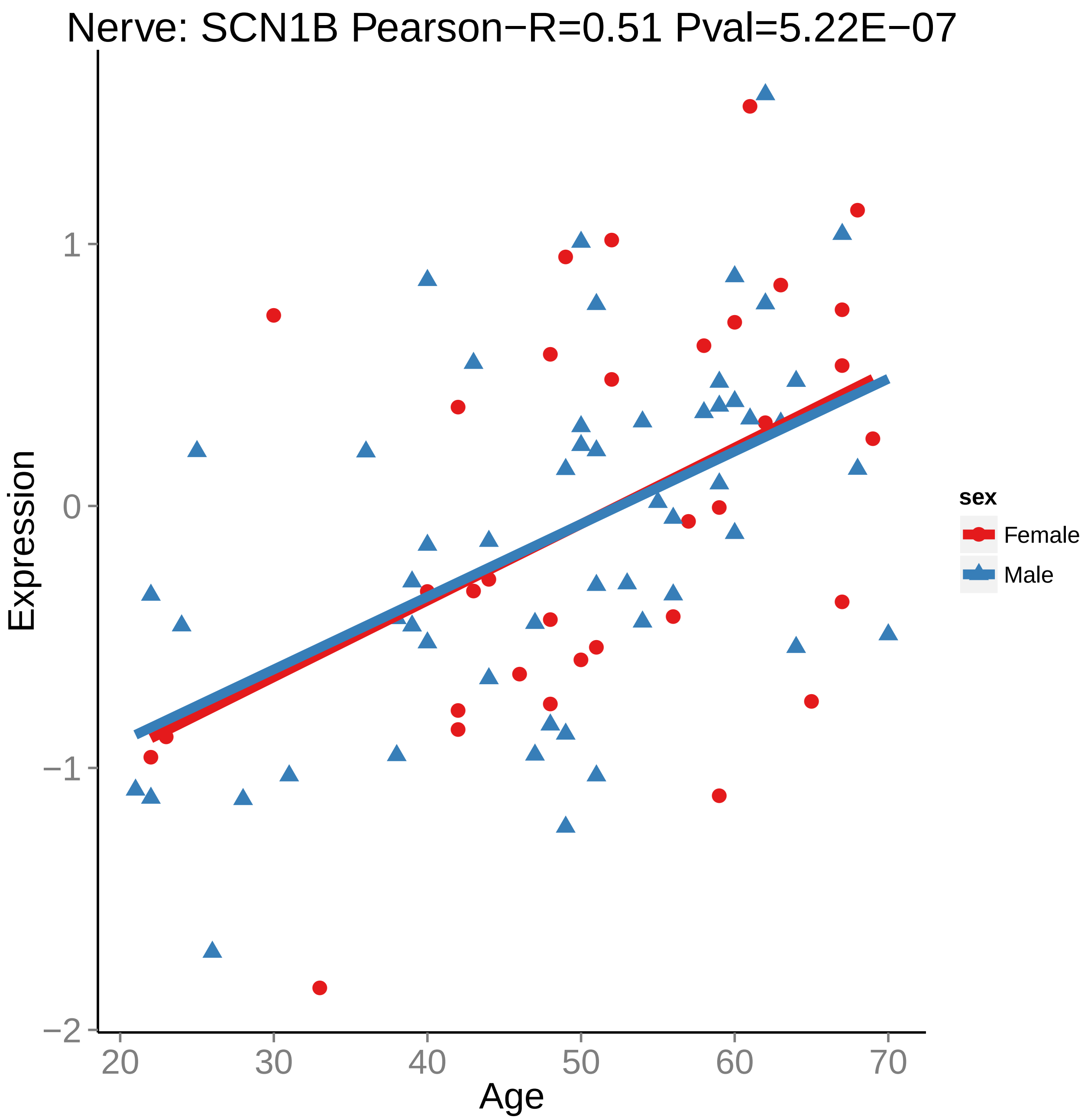
Nerve: SCN1B Pearson−R=0.51 Pval=5.22E−07
Expression
Age
1
0
−1
−2
20
30
40
50
60
70
sex
Female
Male

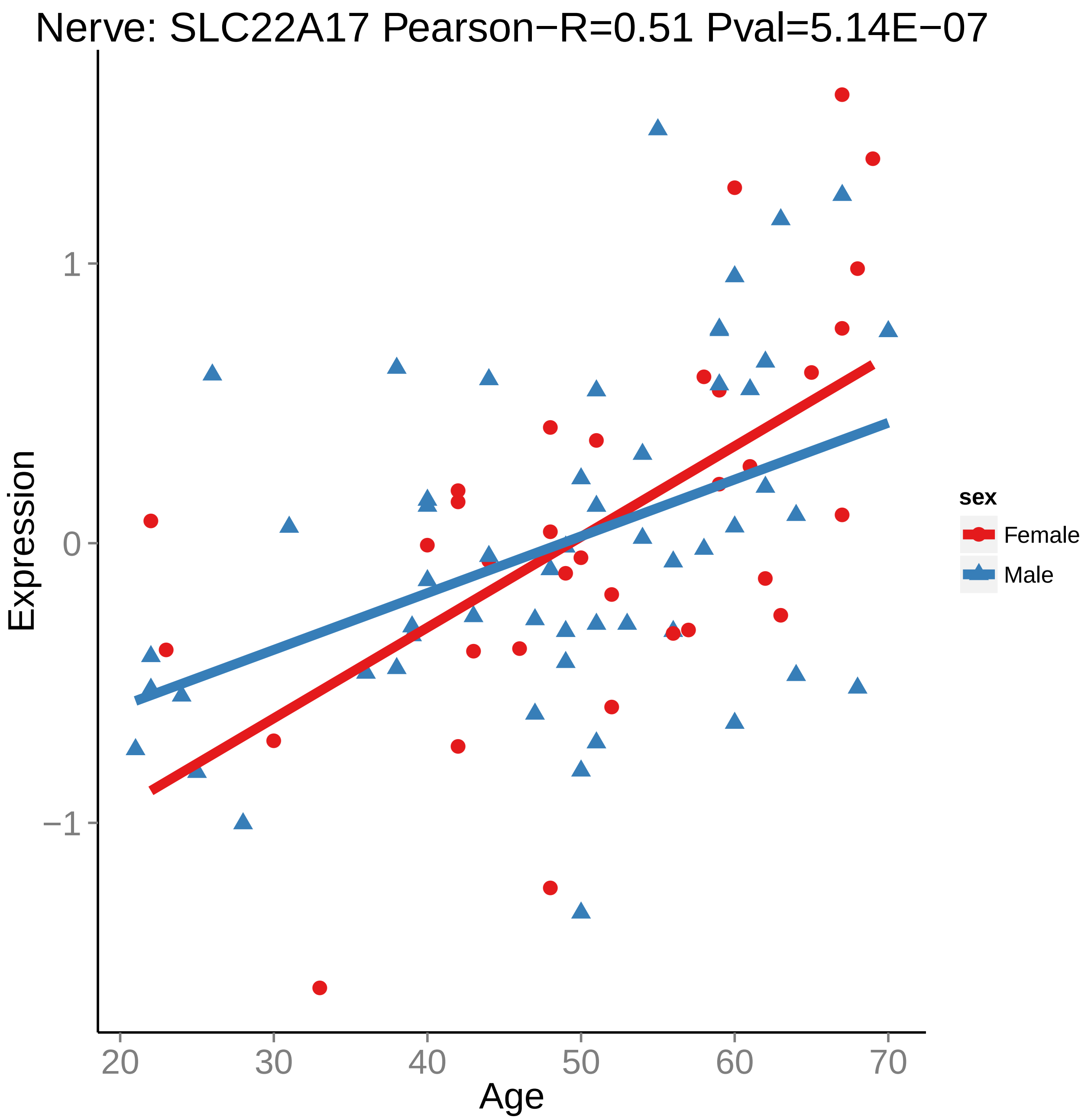

Nerve: SLC22A17 Pearson−R=0.51 Pval=5.14E−07
Expression
1
0
−1
20
30
40
50
60
70
Age
sex
Female
Male

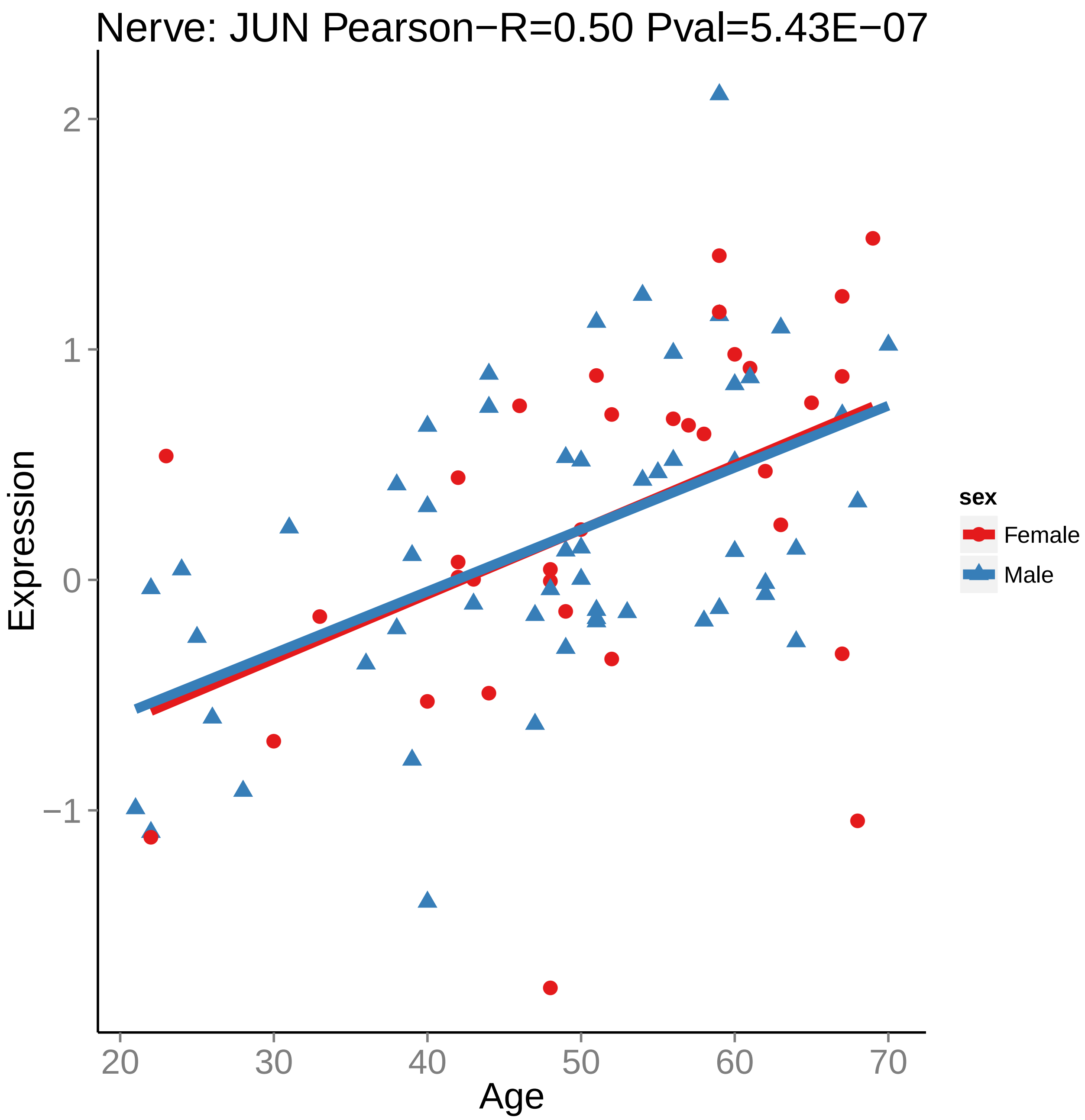
Nerve: JUN Pearson−R=0.50 Pval=5.43E−07
Expression
2
1
0
−1
Age
20
30
40
50
60
70
sex
Female
Male

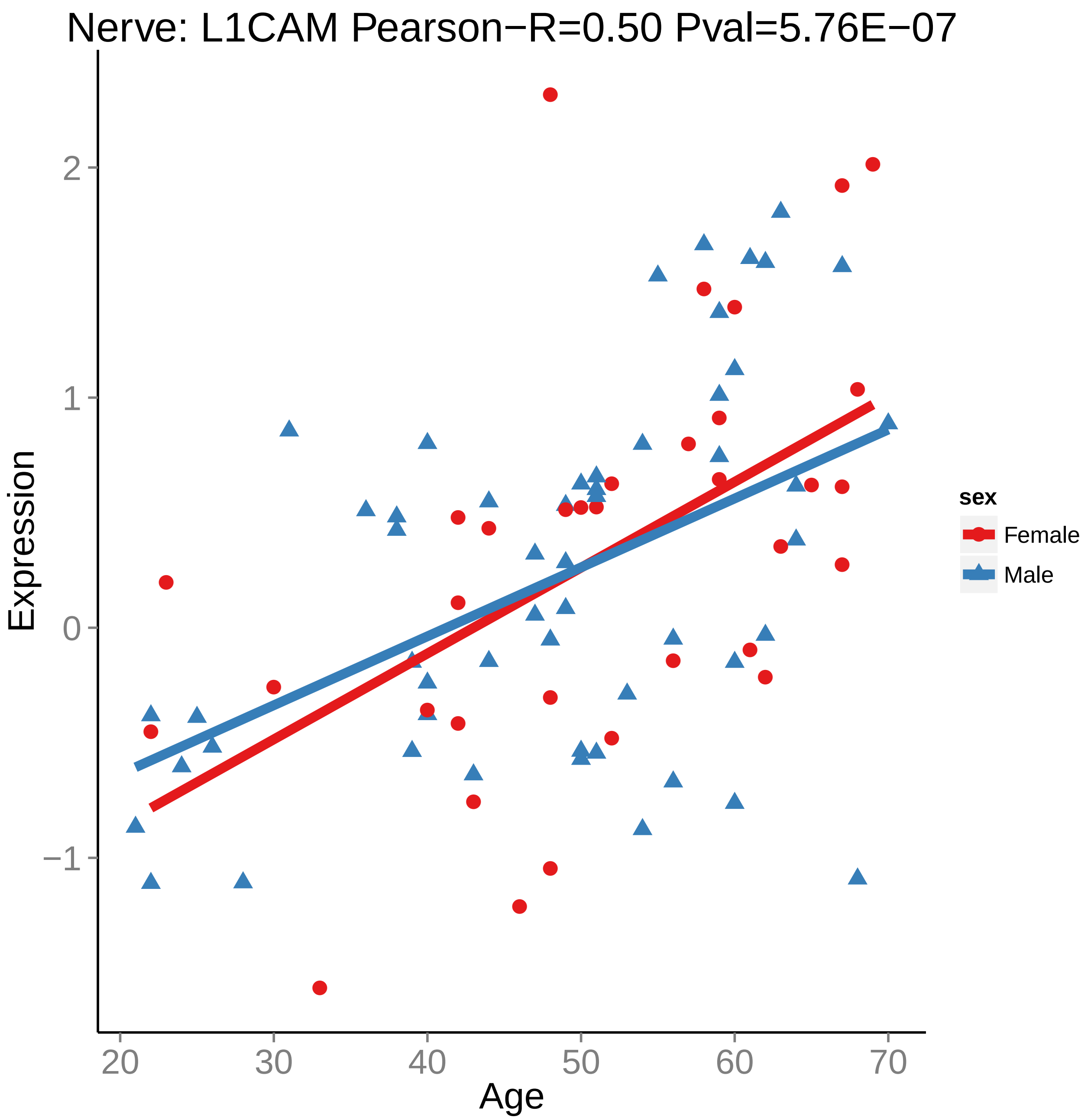

Nerve: L1CAM Pearson−R=0.50 Pval=5.76E−07
Expression
Age
2
1
0
−1
20
30
40
50
60
70
sex
Female
Male

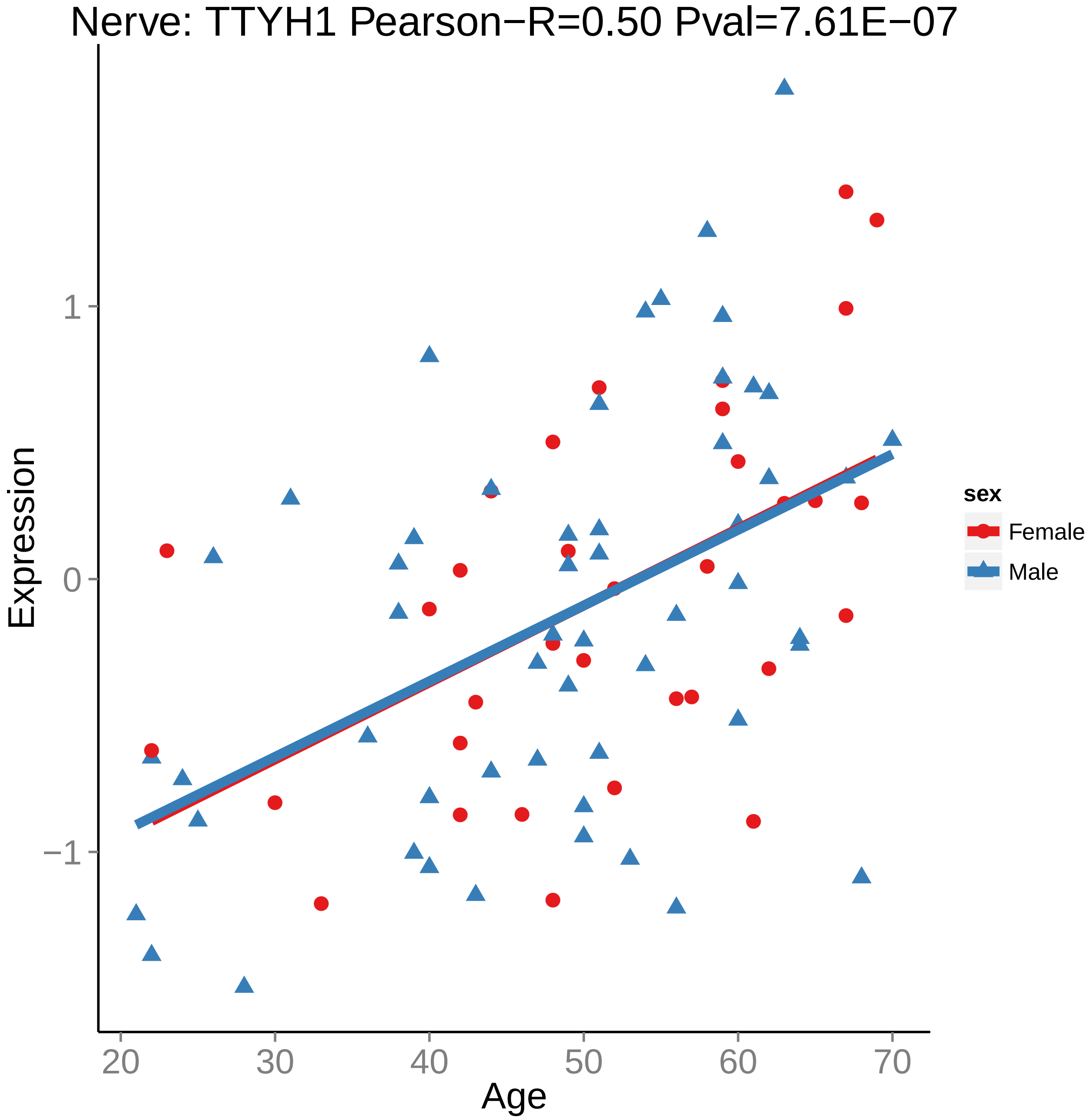

Nerve: TTYH1 Pearson−R=0.50 Pval=7.61E−07
Expression
1
0
−1
20
30
40
50
60
70
Age
sex
Female
Male

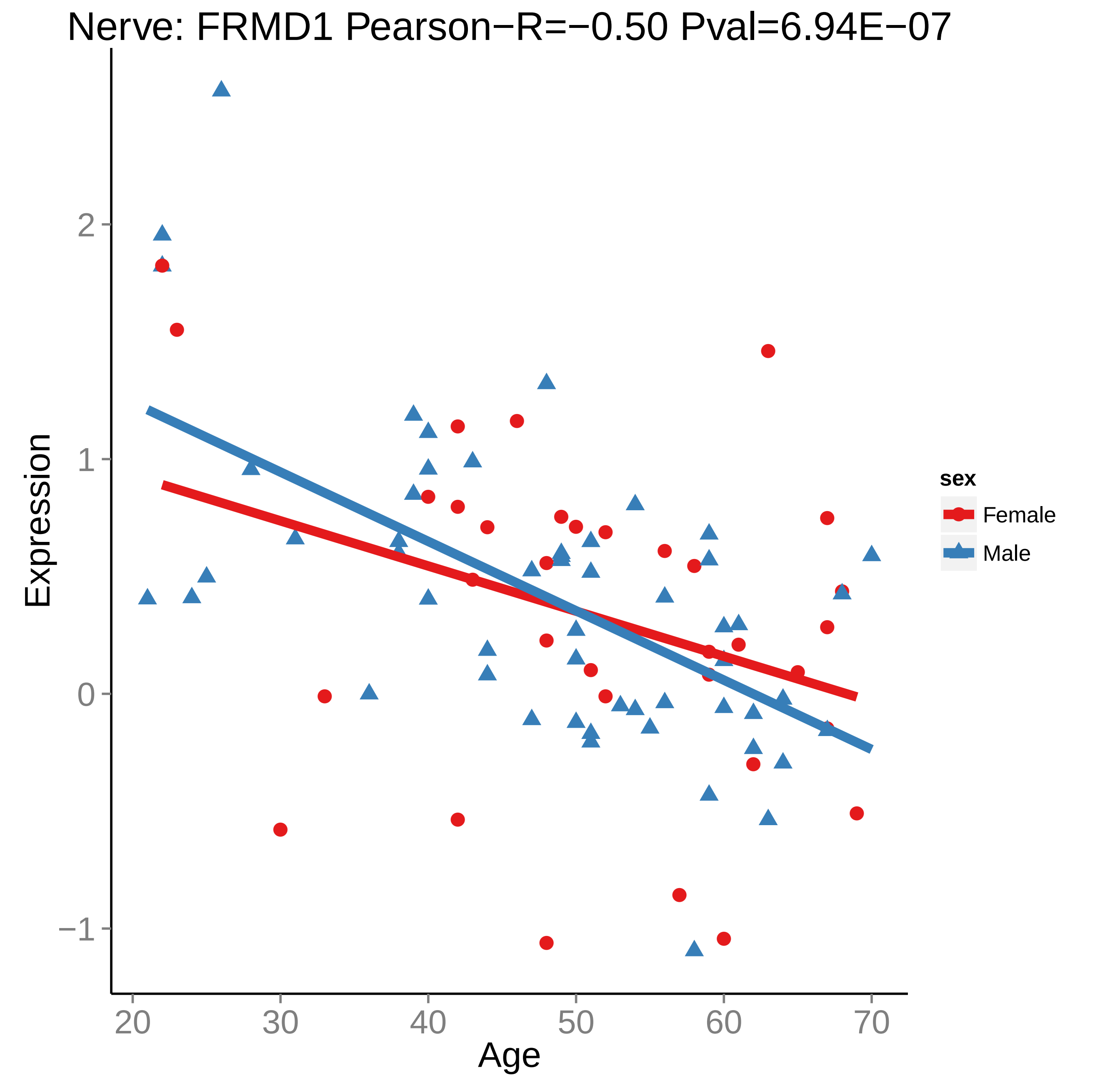

Nerve: FRMD1 Pearson−R=−0.50 Pval=6.94E−07
Expression
2
1
0
−1
Age
20
30
40
50
60
70
sex
Female
Male

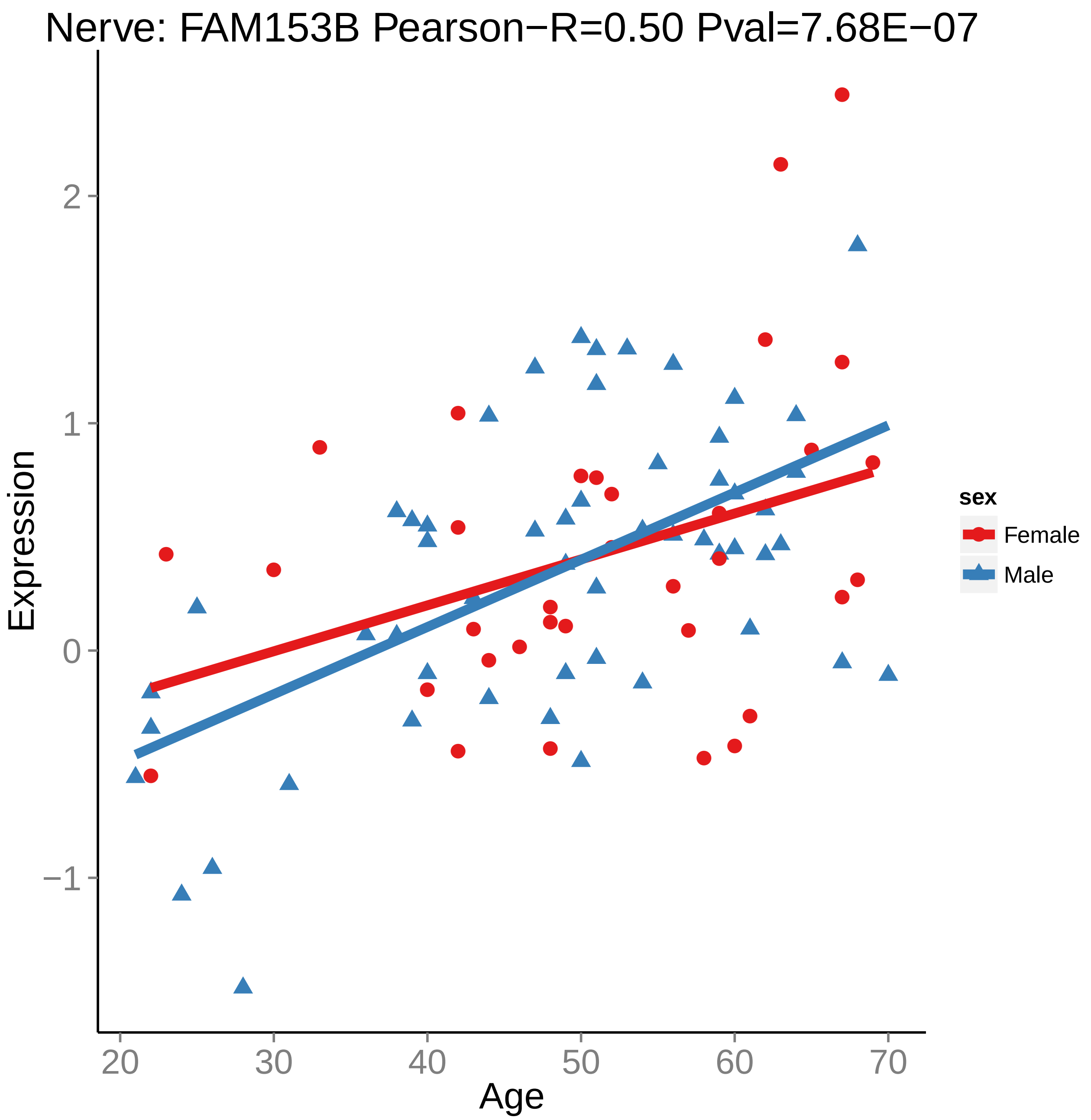

Nerve: FAM153B Pearson−R=0.50 Pval=7.68E−07
Expression
2
1
0
−1
20
30
40
50
60
70
Age
sex
Female
Male

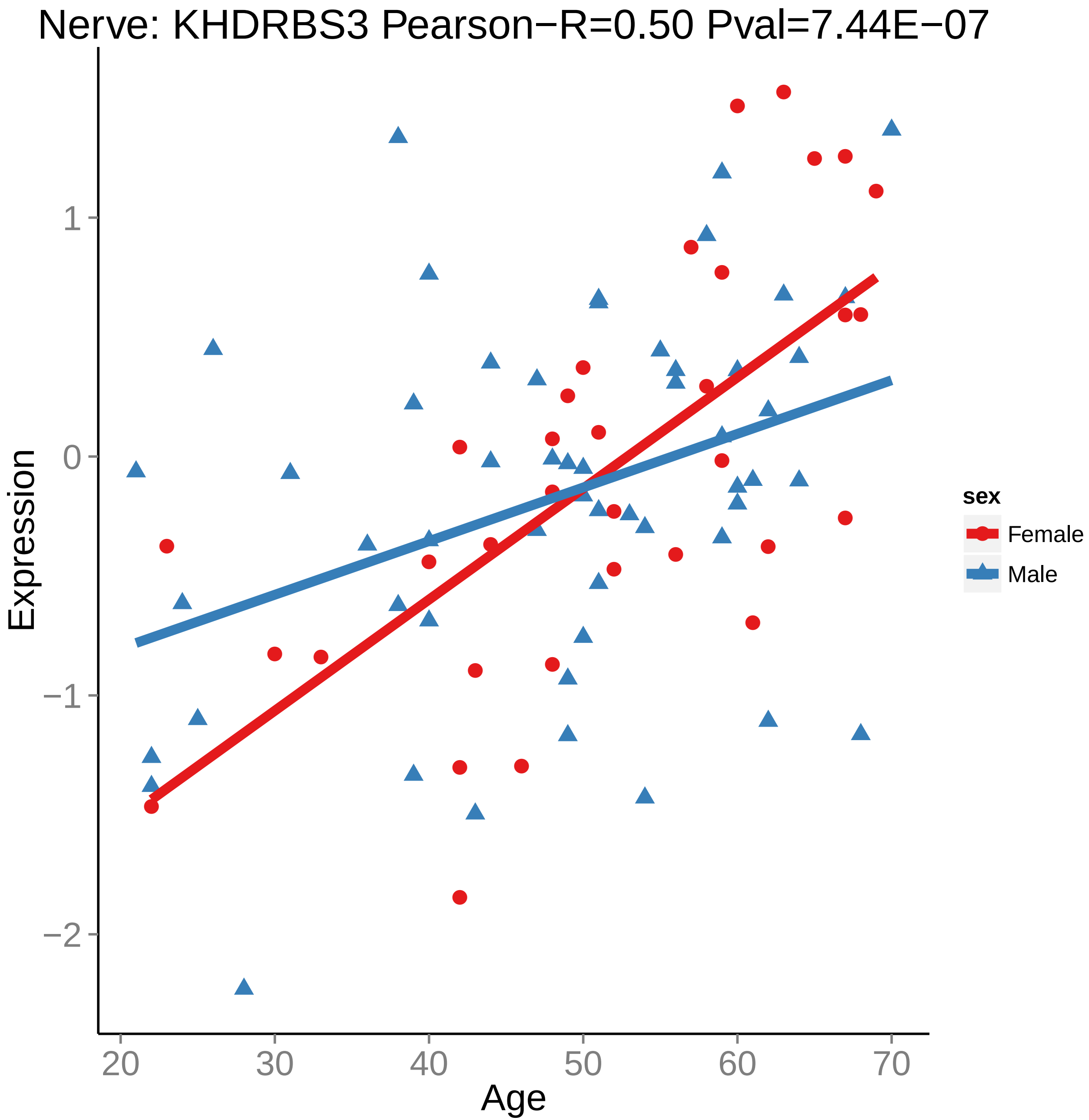
Nerve: KHDRBS3 Pearson−R=0.50 Pval=7.44E−07
Expression
1
0
−1
−2
20
30
40
50
60
70
Age
sex
Female
Male

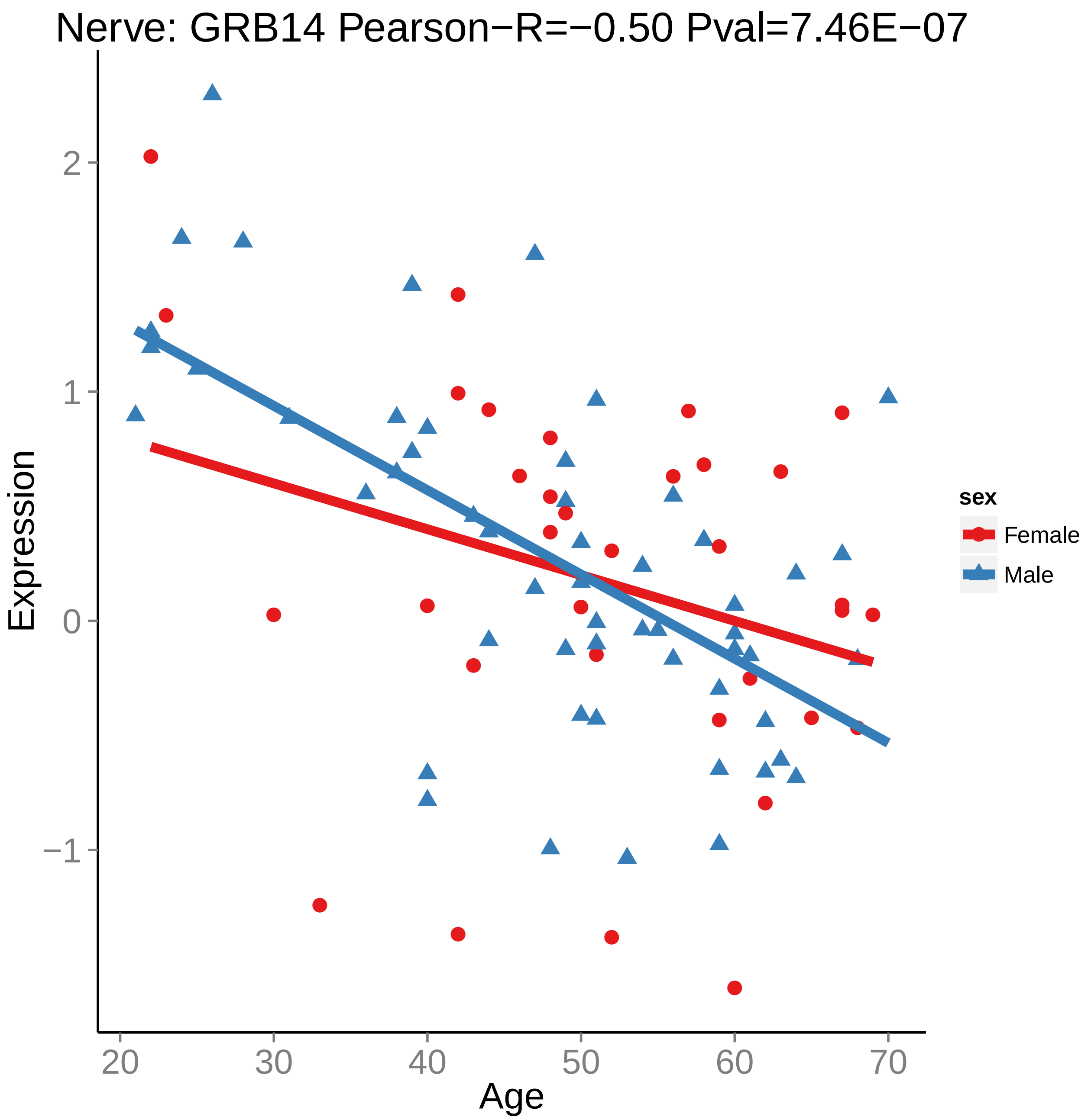

Nerve: GRB14 Pearson−R=−0.50 Pval=7.46E−07
Expression
2
1
0
−1
20
30
40
50
60
70
Age
sex
Female
Male

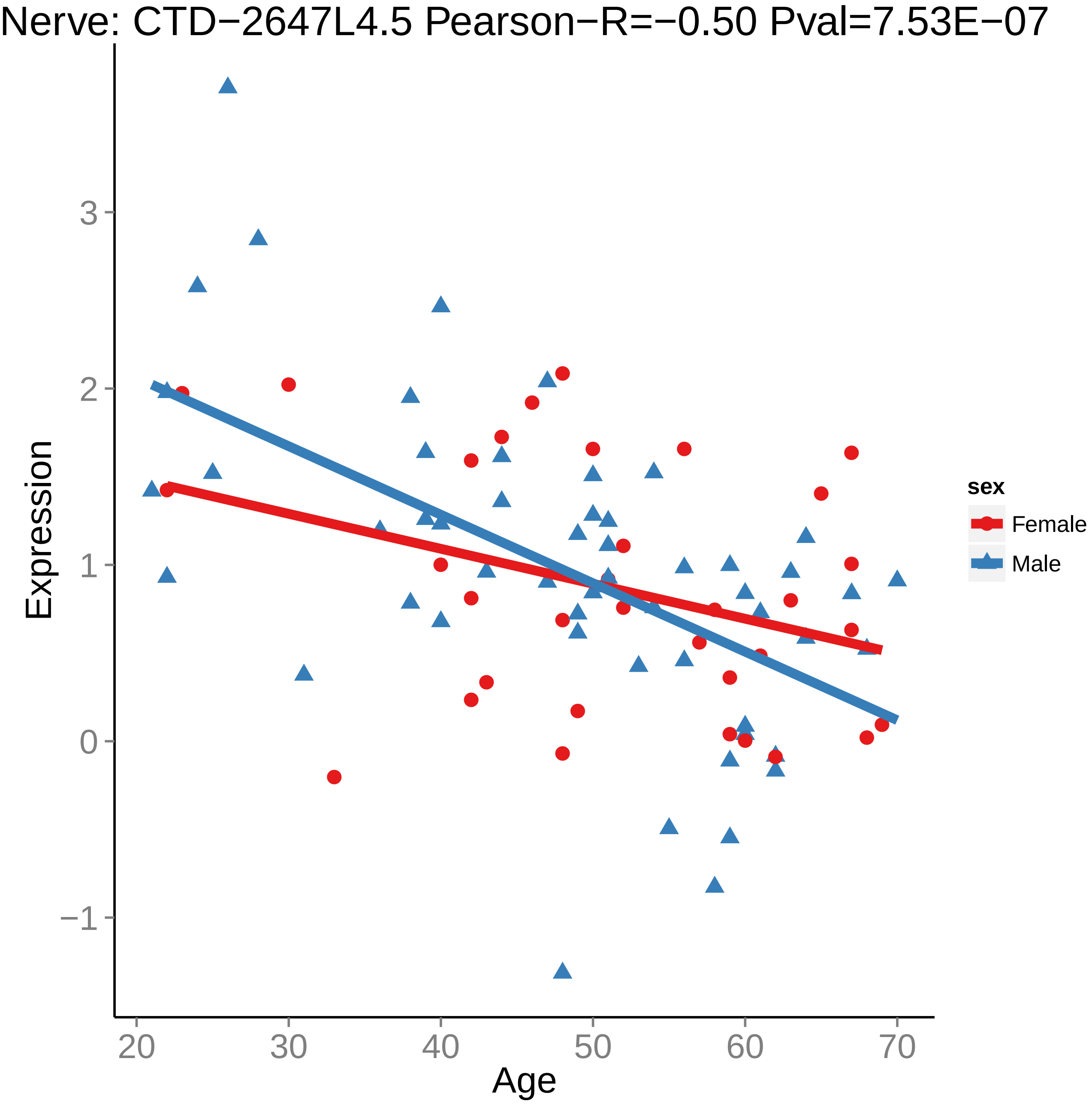

Nerve: CTD−2647L4.5 Pearson−R=−0.50 Pval=7.53E−07
Expression
Age
3
2
1
0
−1
20
30
40
50
60
70
sex
Female
Male

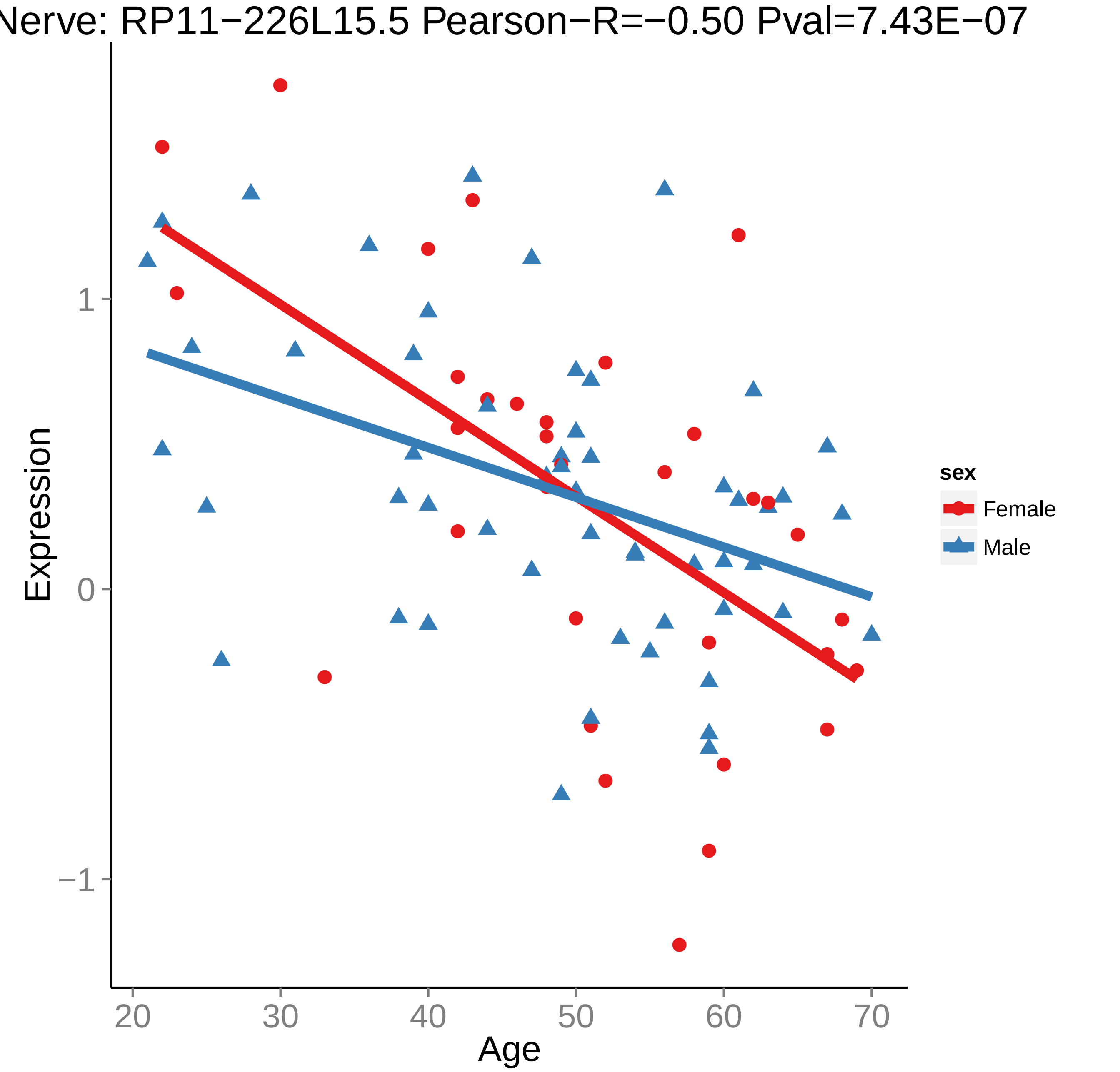

Nerve: RP11−226L15.5 Pearson−R=−0.50 Pval=7.43E−07
Expression
1
0
−1
20
30
40
50
60
70
Age
sex
Female
Male

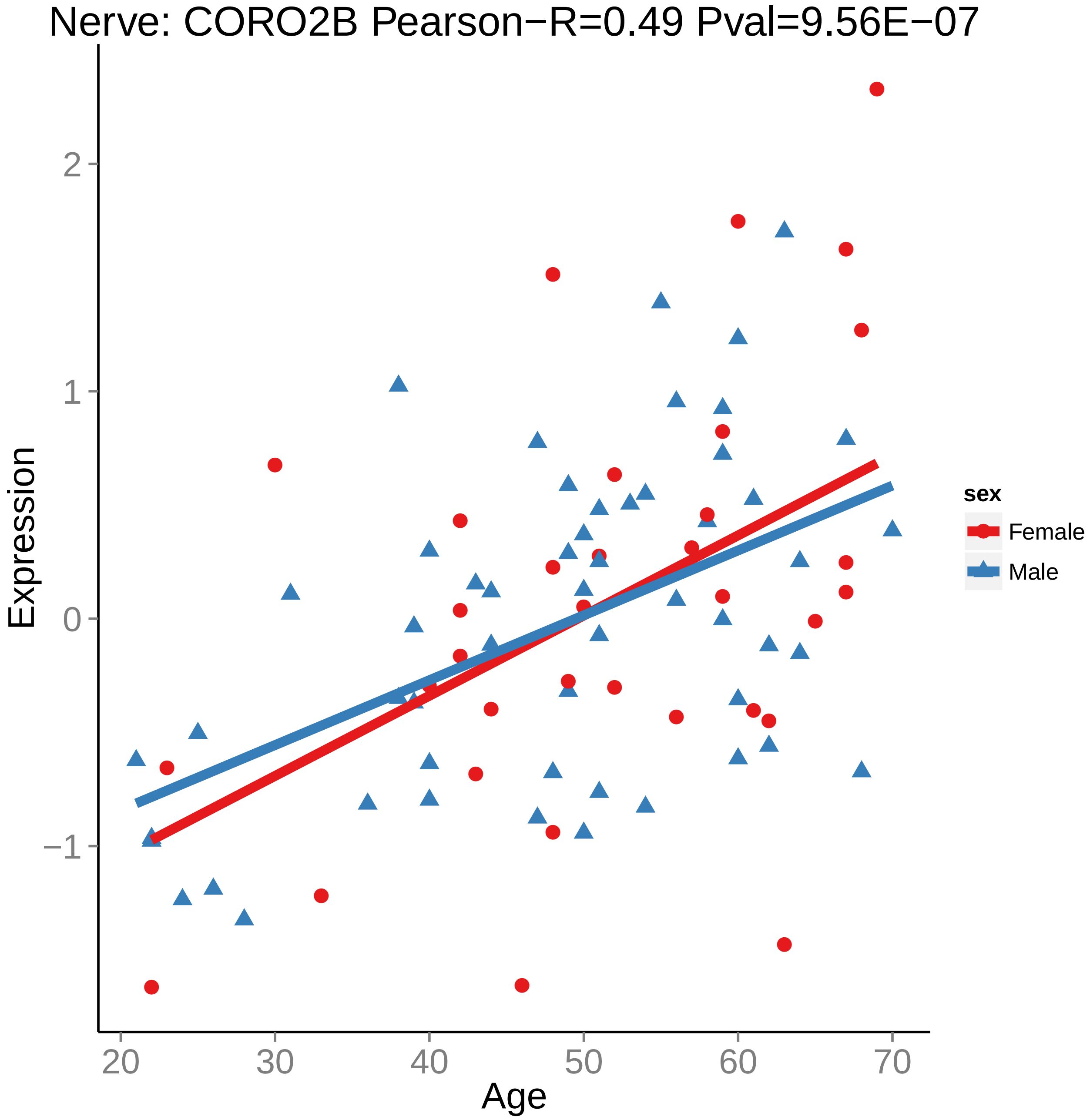
Nerve: CORO2B Pearson−R=0.49 Pval=9.56E−07
Expression
2
1
0
−1
20
30
40
50
60
70
Age
sex
Female
Male

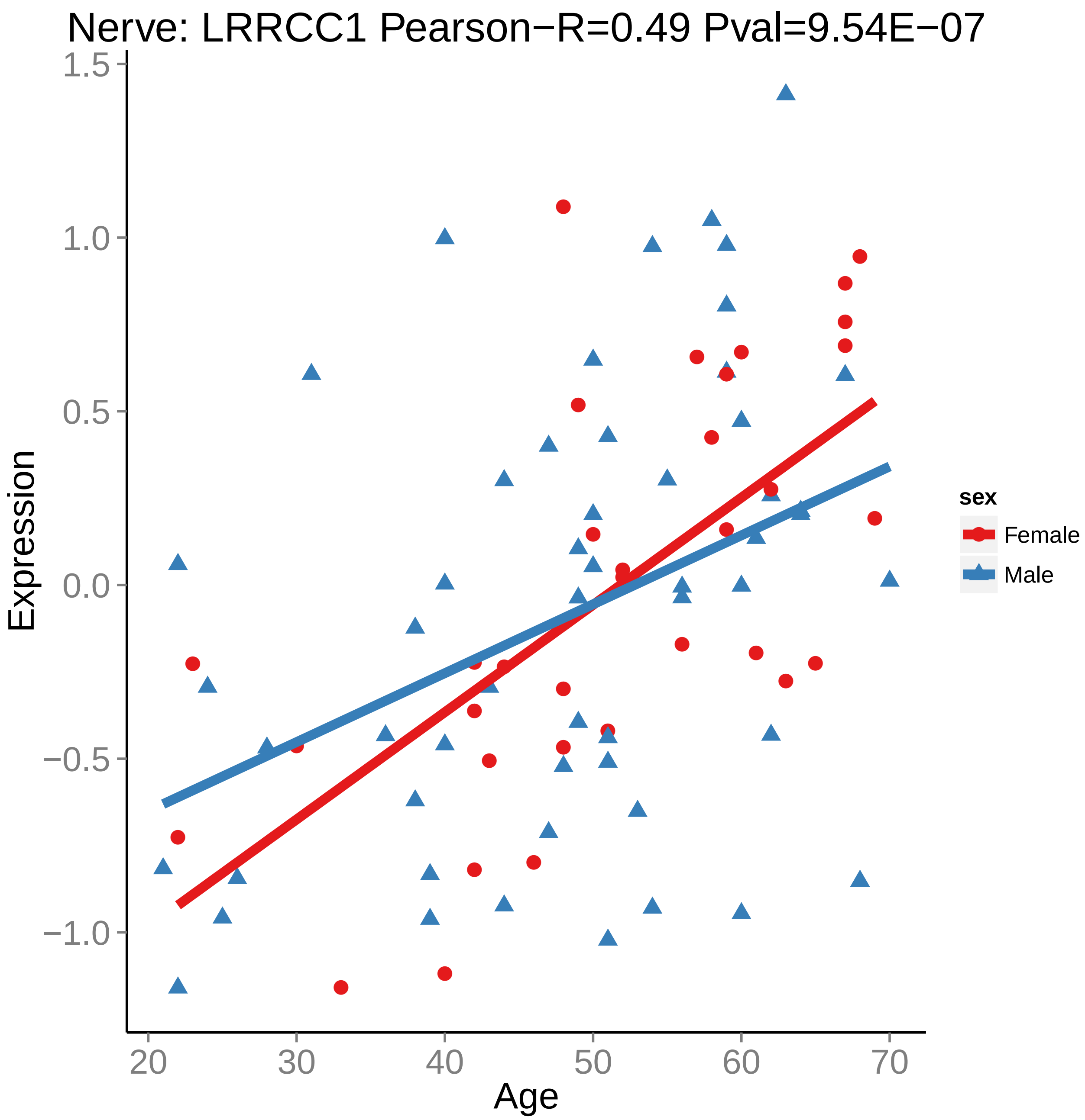

Nerve: LRRCC1 Pearson−R=0.49 Pval=9.54E−07
Expression
Age
1.5
1.0
0.5
0.0
−0.5
−1.0
20
30
40
50
60
70
sex
Female
Male

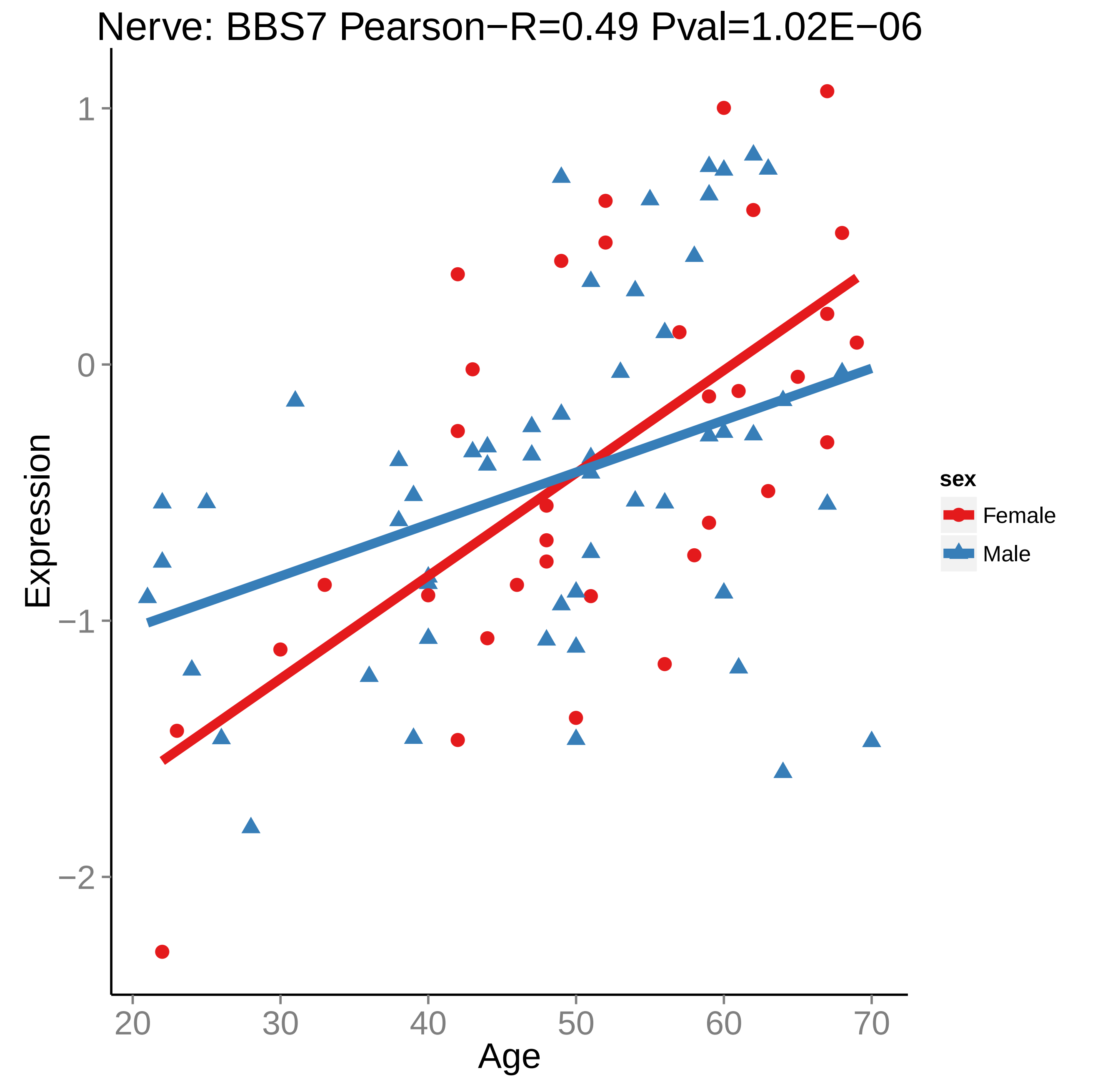

Nerve: BBS7 Pearson−R=0.49 Pval=1.02E−06
Expression
1
0
−1
−2
20
30
40
50
60
70
Age
sex
Female
Male

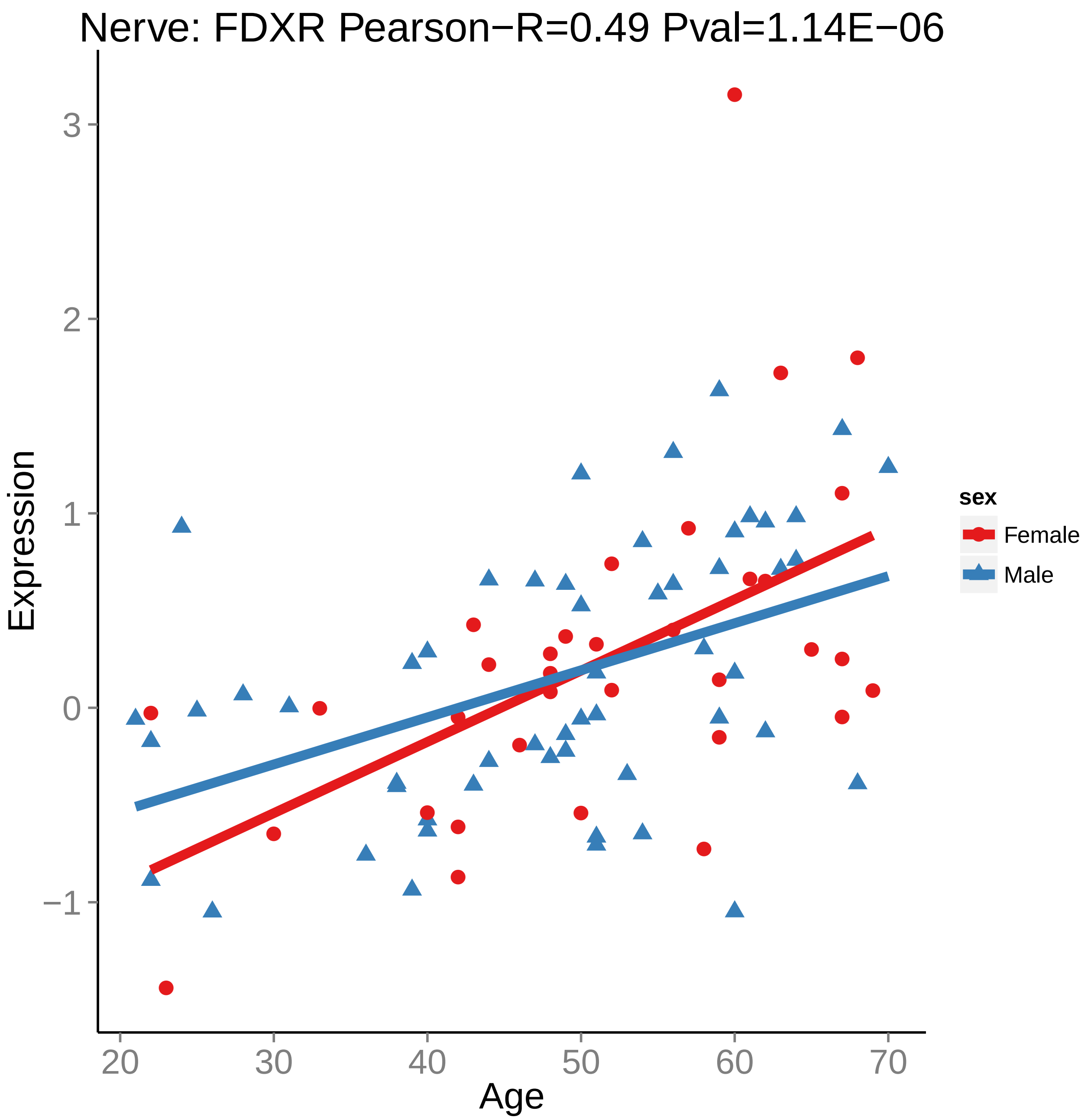
Nerve: FDXR Pearson−R=0.49 Pval=1.14E−06
Expression
3
2
1
0
−1
20
30
40
50
60
70
Age
sex
Female
Male

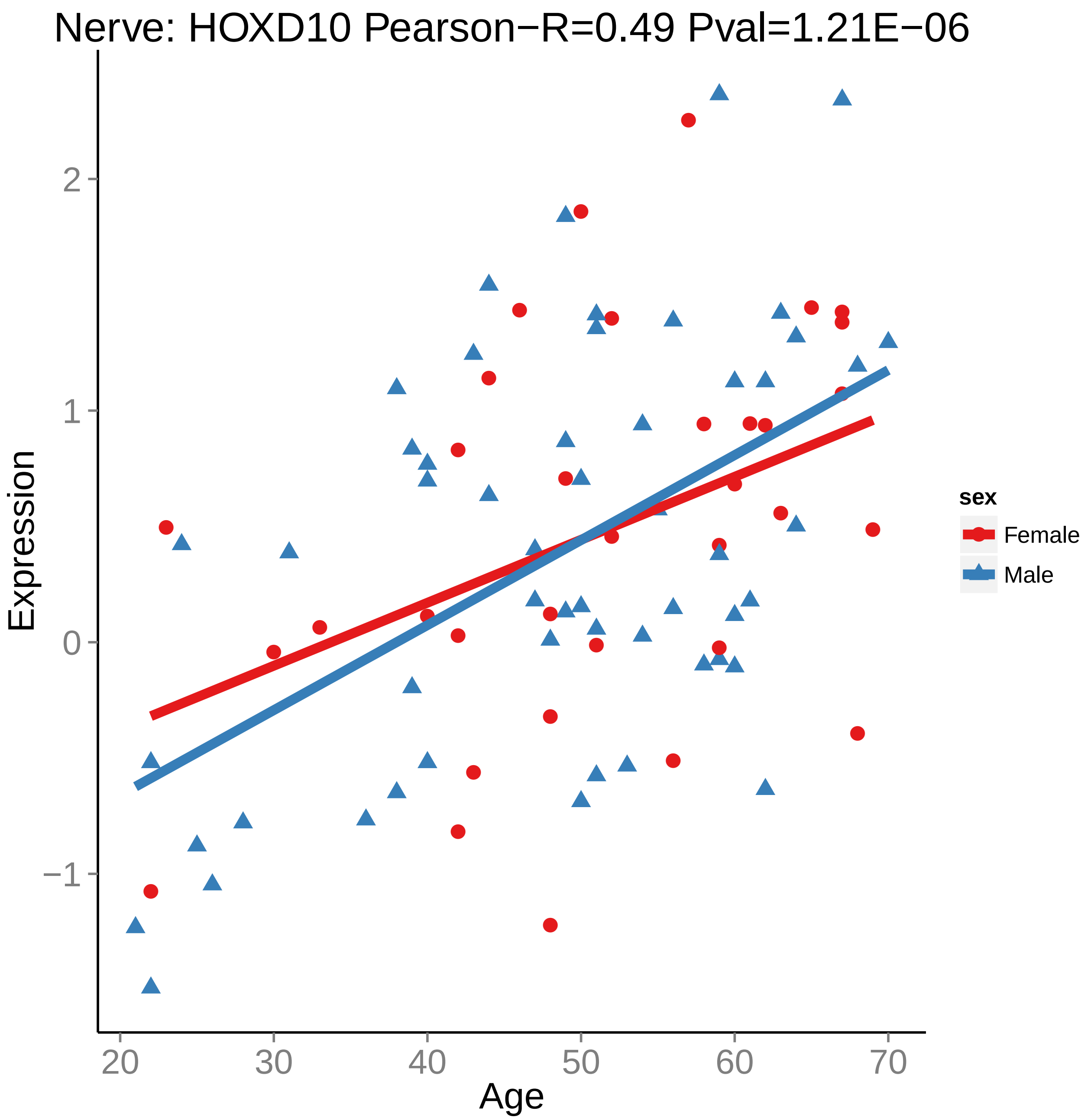
Nerve: HOXD10 Pearson−R=0.49 Pval=1.21E−06
Expression
Age
2
1
0
−1
20
30
40
50
60
70
sex
Female
Male

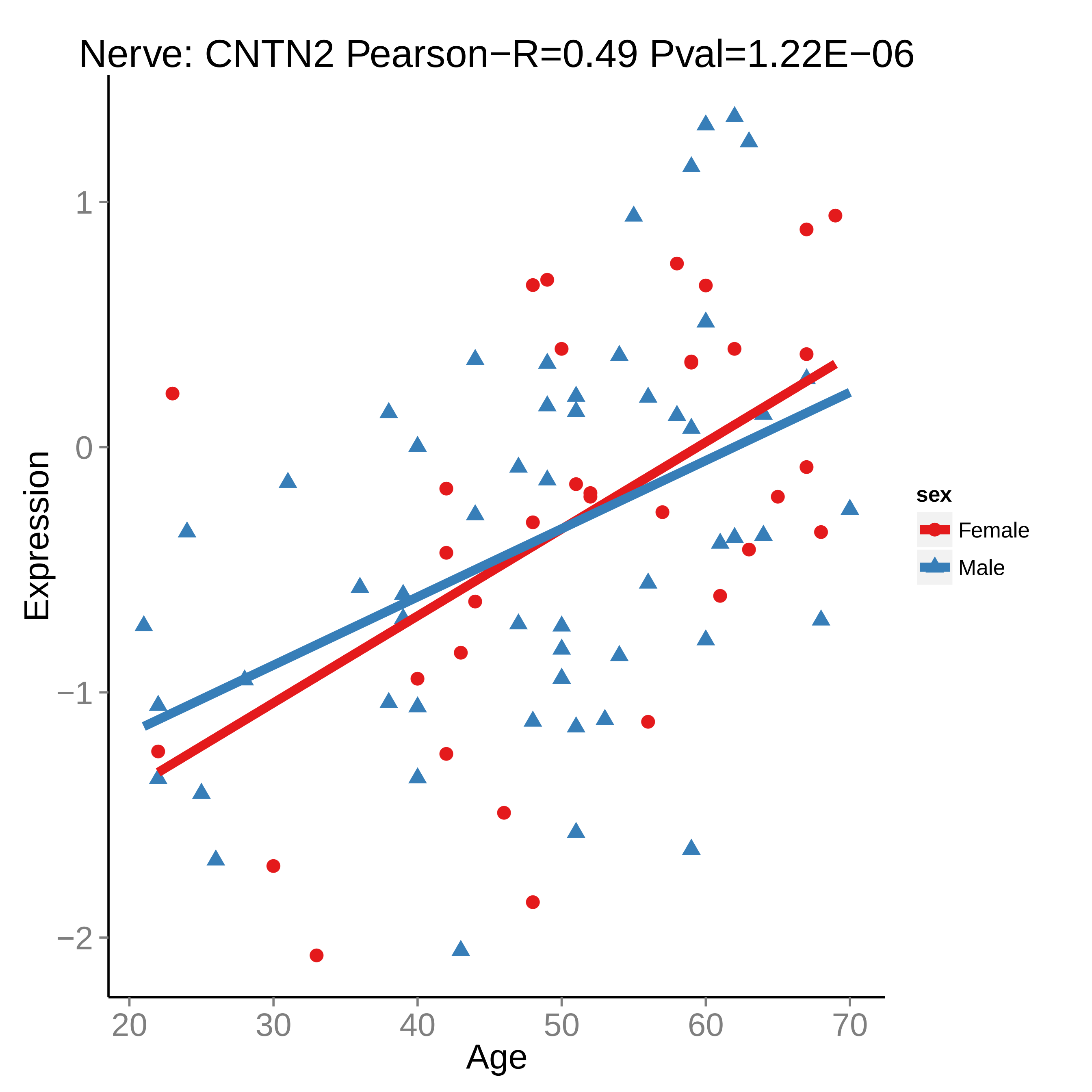

Nerve: CNTN2 Pearson−R=0.49 Pval=1.22E−06
Expression
Age
1
0
−1
−2
20
30
40
50
60
70
sex
Female
Male

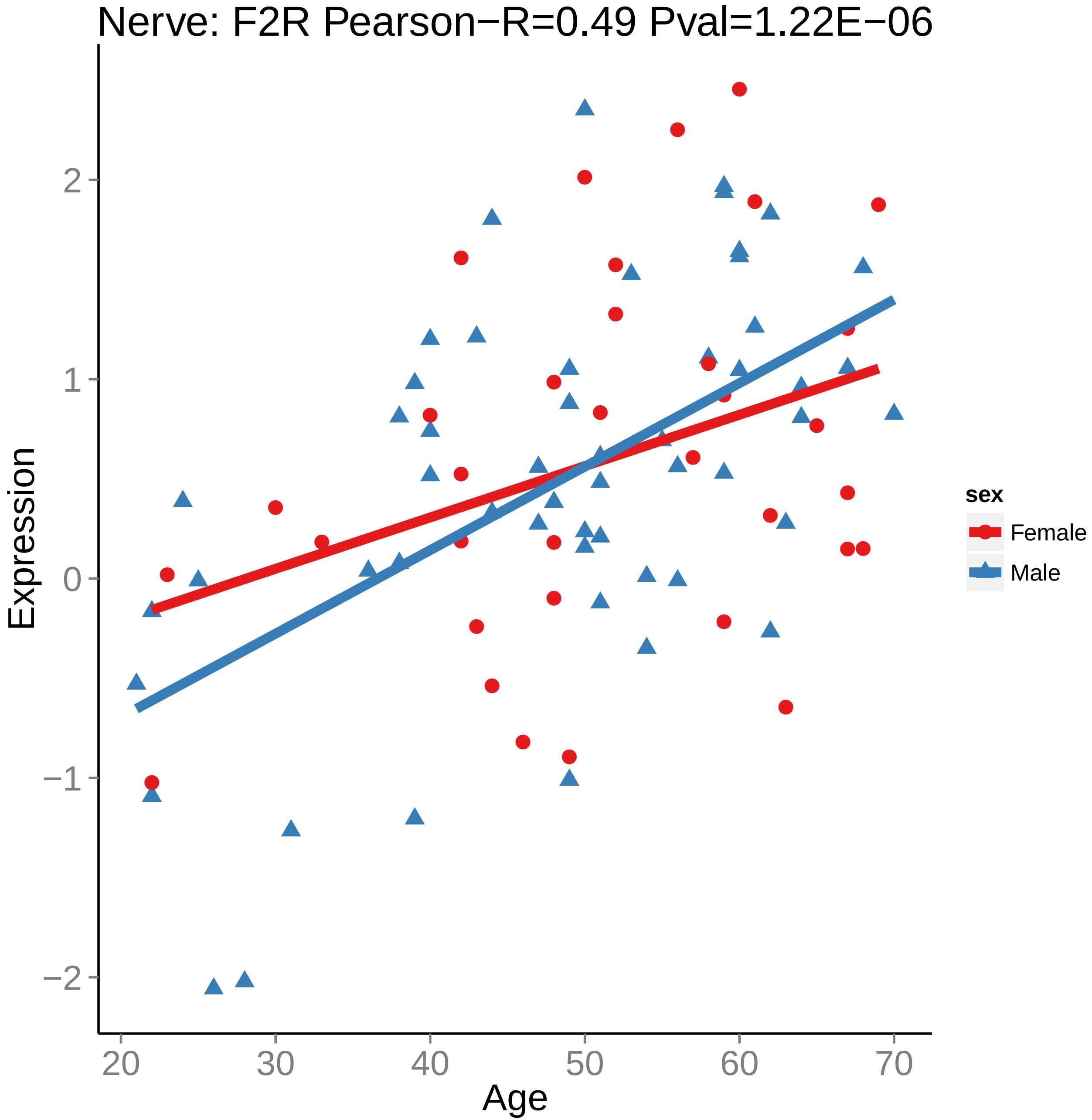
Nerve: F2R Pearson−R=0.49 Pval=1.22E−06
Expression
Age
2
1
0
−1
−2
20
30
40
50
60
70
sex
Female
Male

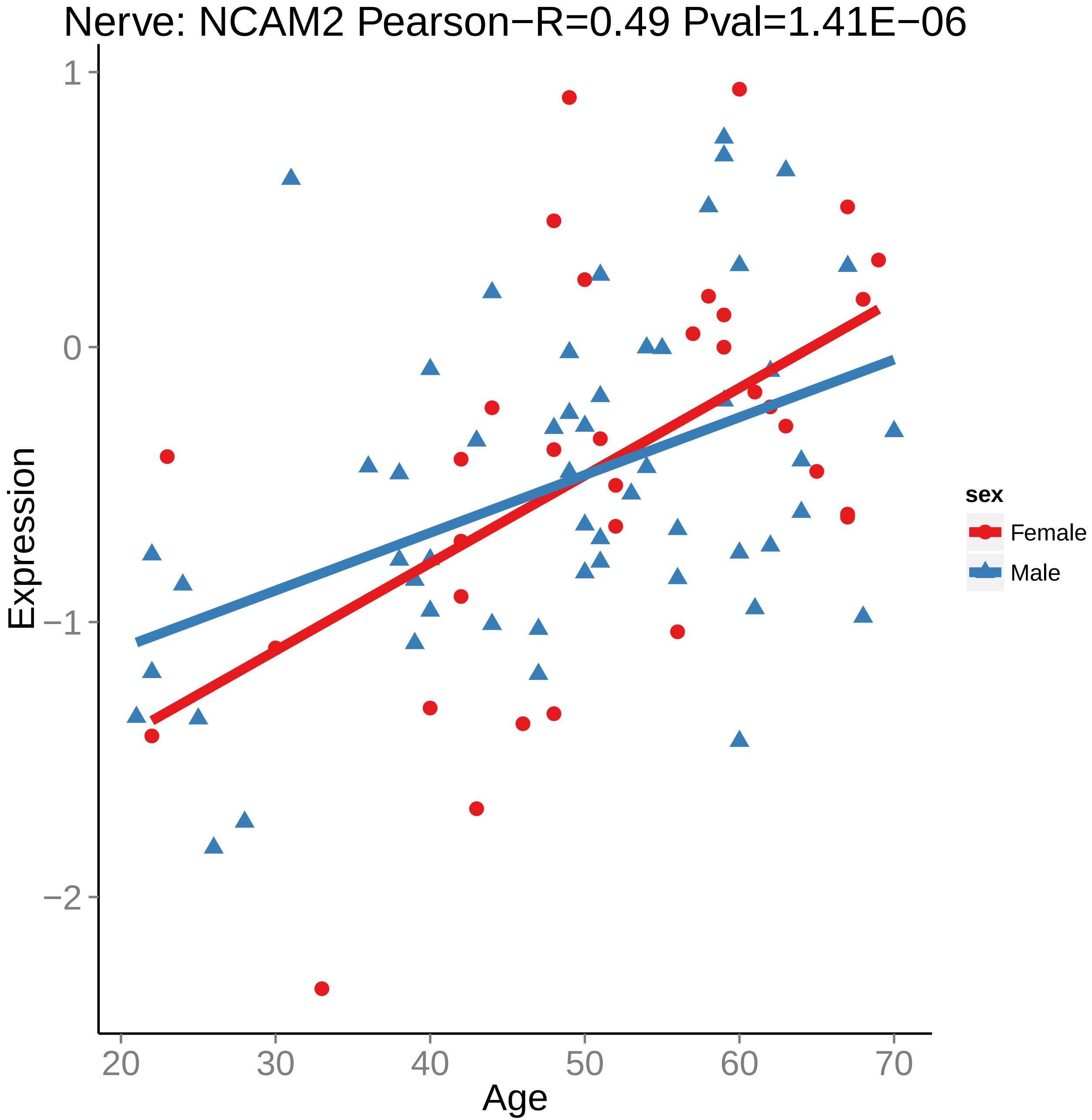

Nerve: NCAM2 Pearson−R=0.49 Pval=1.41E−06
Expression
Age
1
0
−1
−2
20
30
40
50
60
70
sex
Female
Male

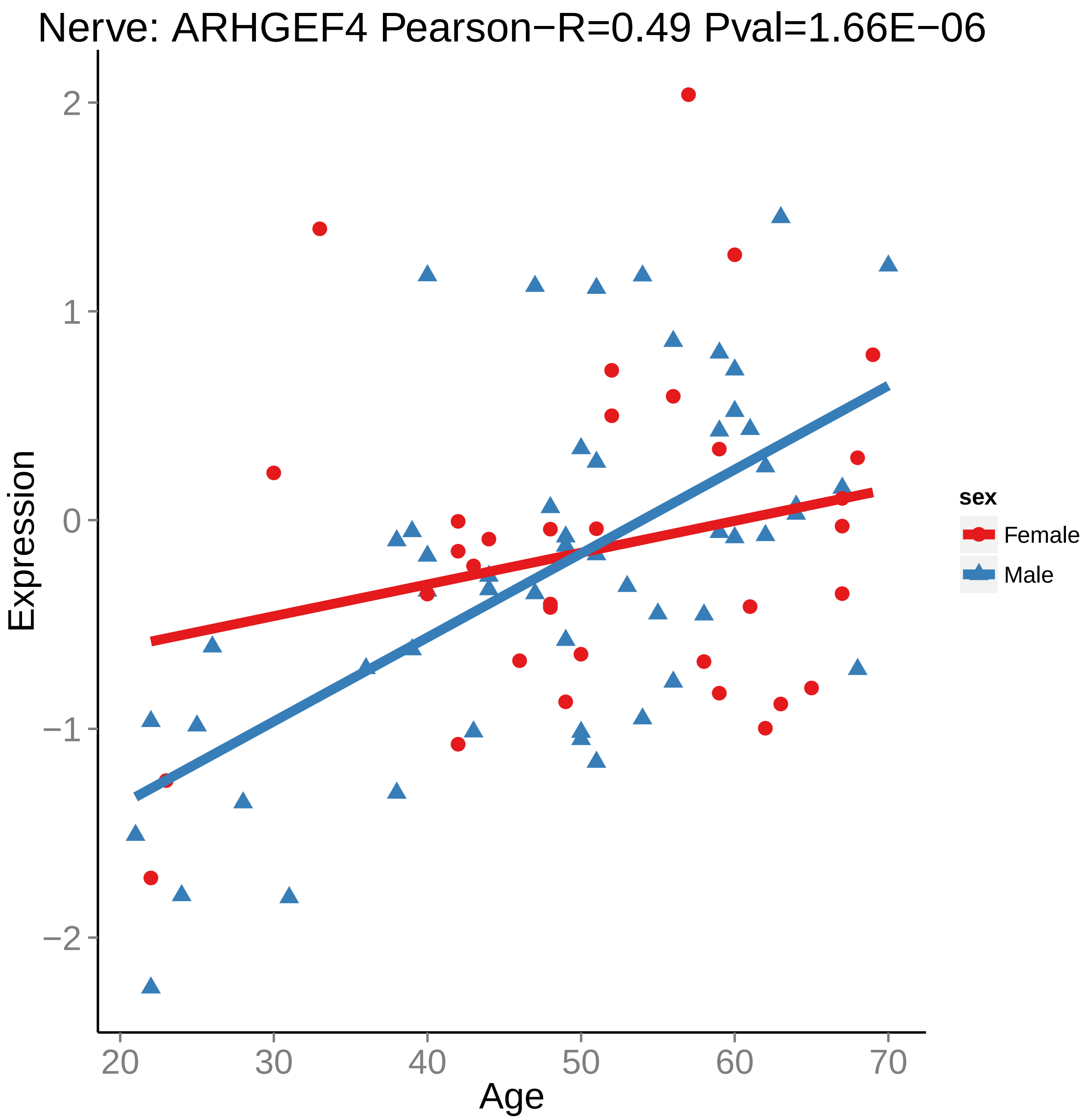
Nerve: ARHGEF4 Pearson−R=0.49 Pval=1.66E−06
Expression
Age
sex
Female
Male
2
1
0
−1
−2
20
30
40
50
60
70

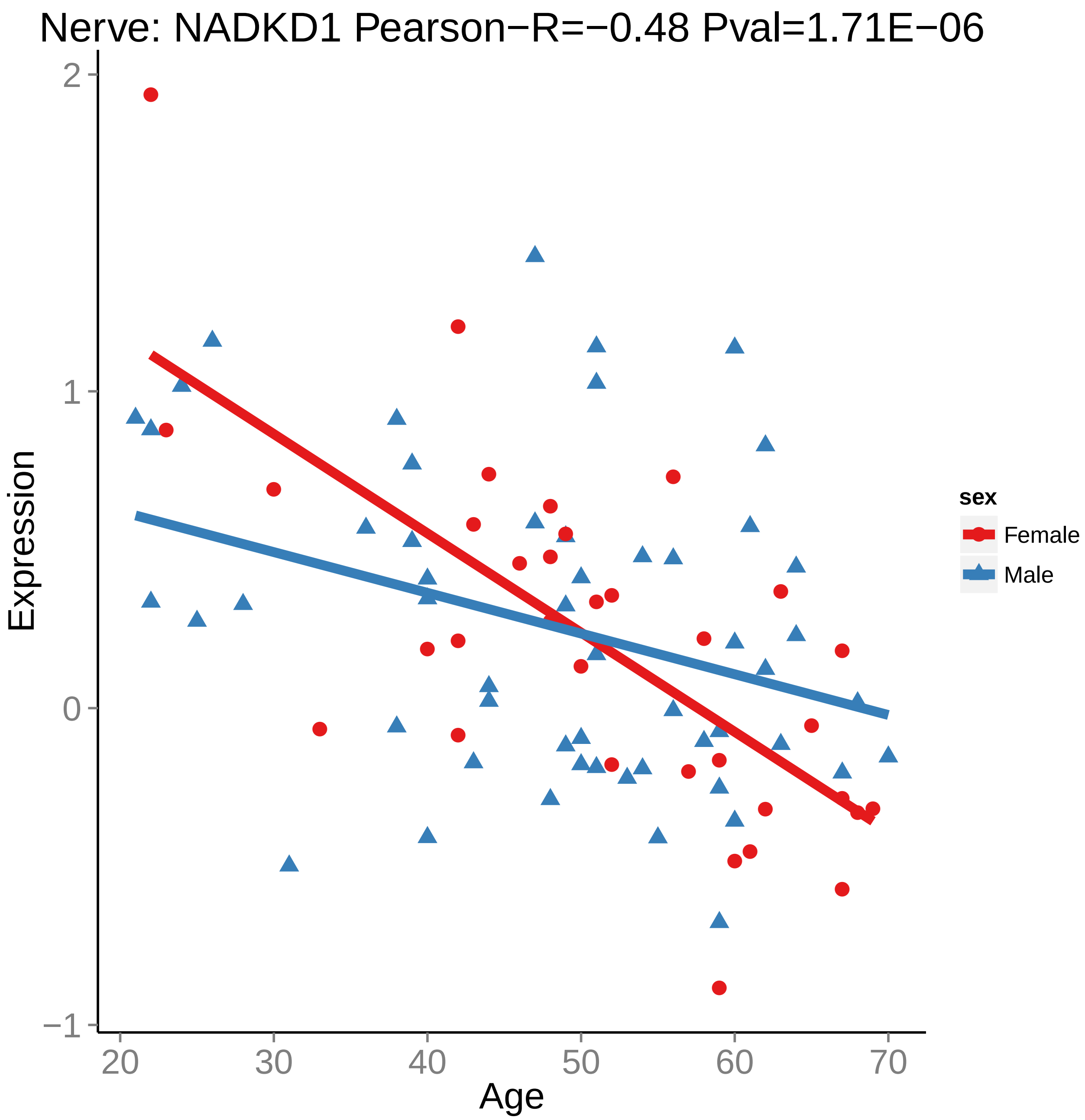

Nerve: NADKD1 Pearson−R=−0.48 Pval=1.71E−06
Expression
Age
2
1
0
−1
20
30
40
50
60
70
sex
Female
Male

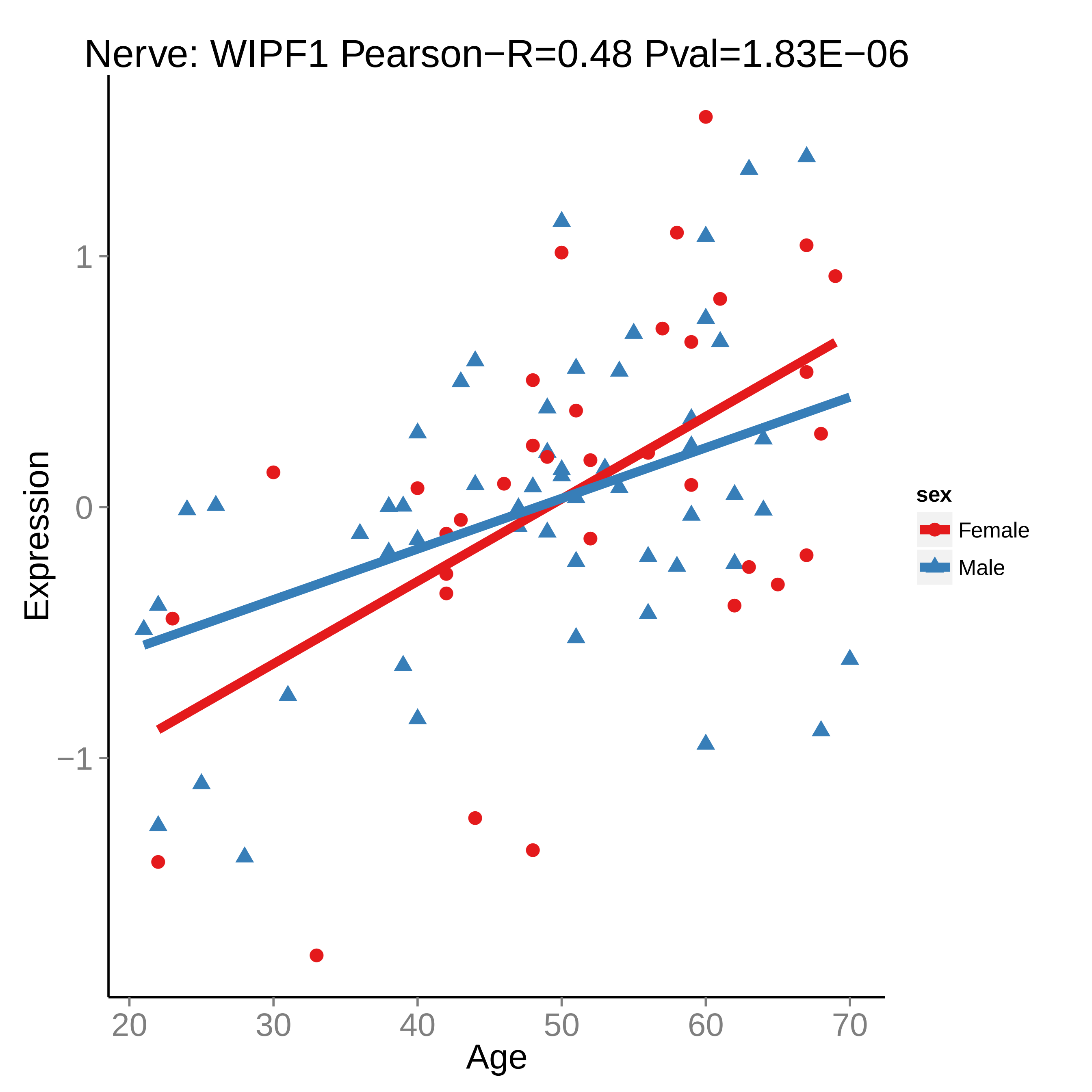

Nerve: WIPF1 Pearson−R=0.48 Pval=1.83E−06
Expression
1
0
−1
20
30
40
50
60
70
Age
sex
Female
Male

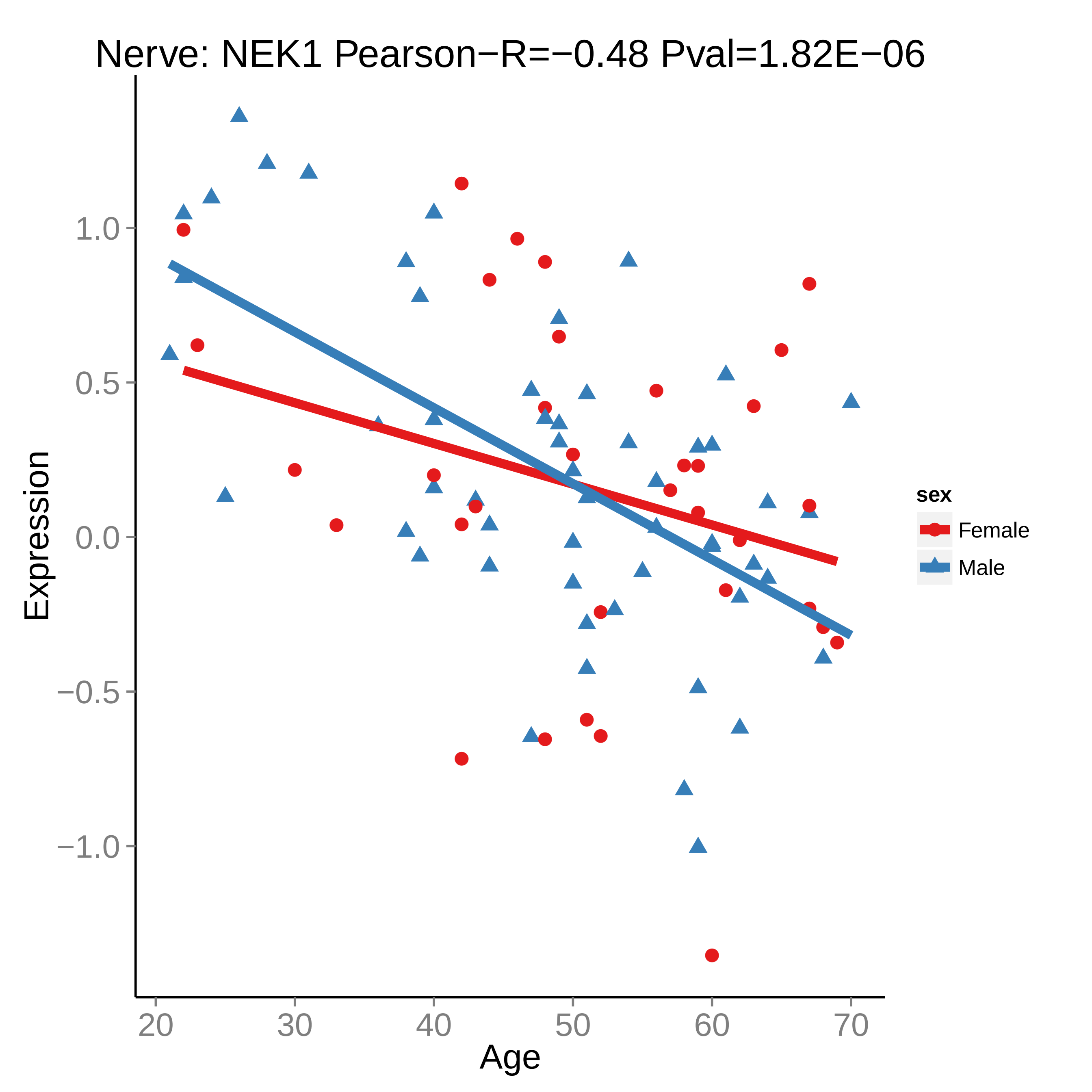
Nerve: NEK1 Pearson−R=−0.48 Pval=1.82E−06
Expression
1.0
0.5
0.0
−0.5
−1.0
20
30
40
50
60
70
Age
sex
Female
Male

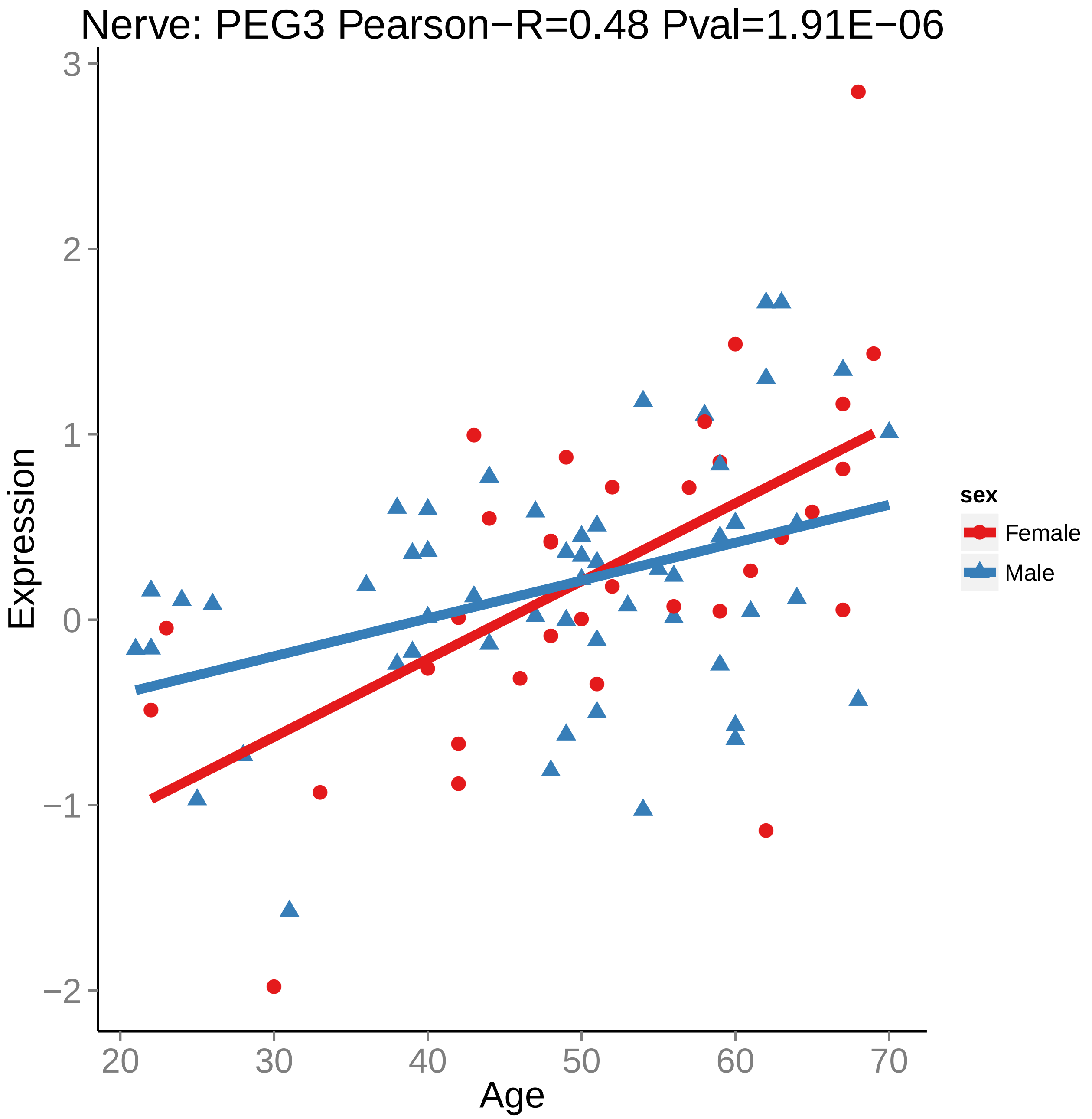
Nerve: PEG3 Pearson−R=0.48 Pval=1.91E−06
Expression
3
2
1
0
−1
−2
20
30
40
50
60
70
Age
sex
Female
Male

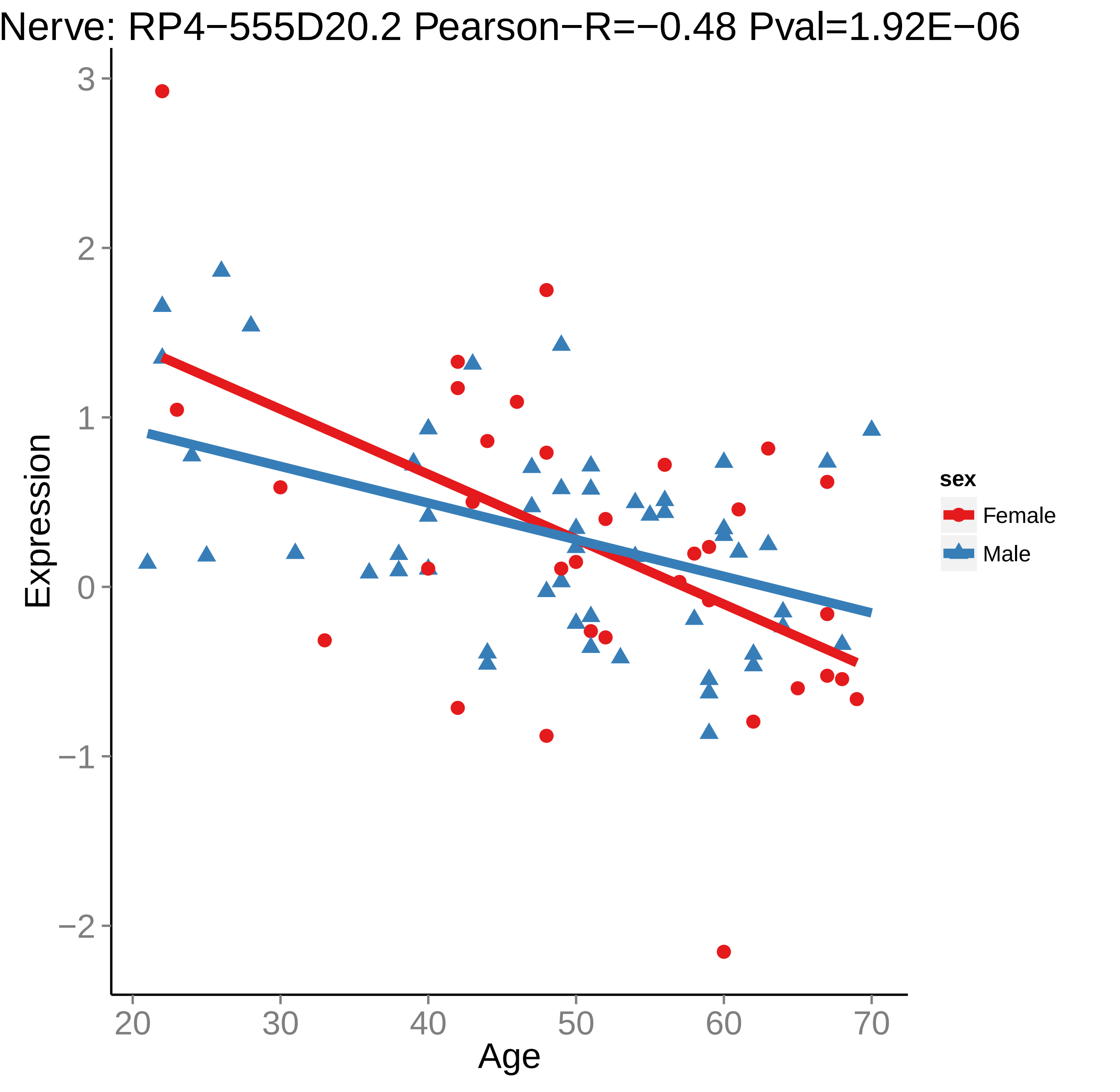
Nerve: RP4−555D20.2 Pearson−R=−0.48 Pval=1.92E−06
Expression
3
2
1
0
−1
−2
20
30
40
50
60
70
Age
sex
Female
Male

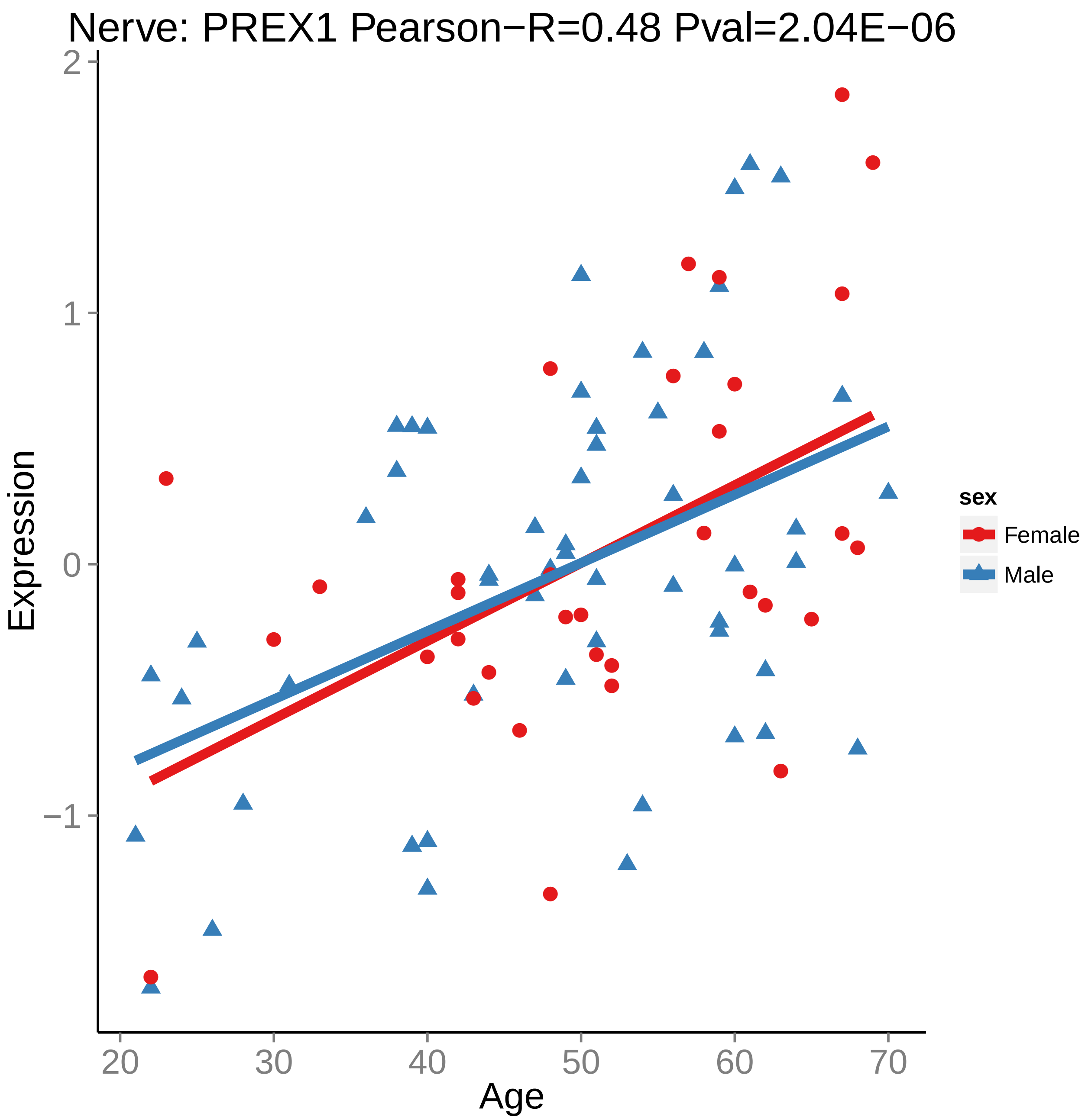

Nerve: PREX1 Pearson−R=0.48 Pval=2.04E−06
Expression
Age
2
1
0
−1
20
30
40
50
60
70
sex
Female
Male

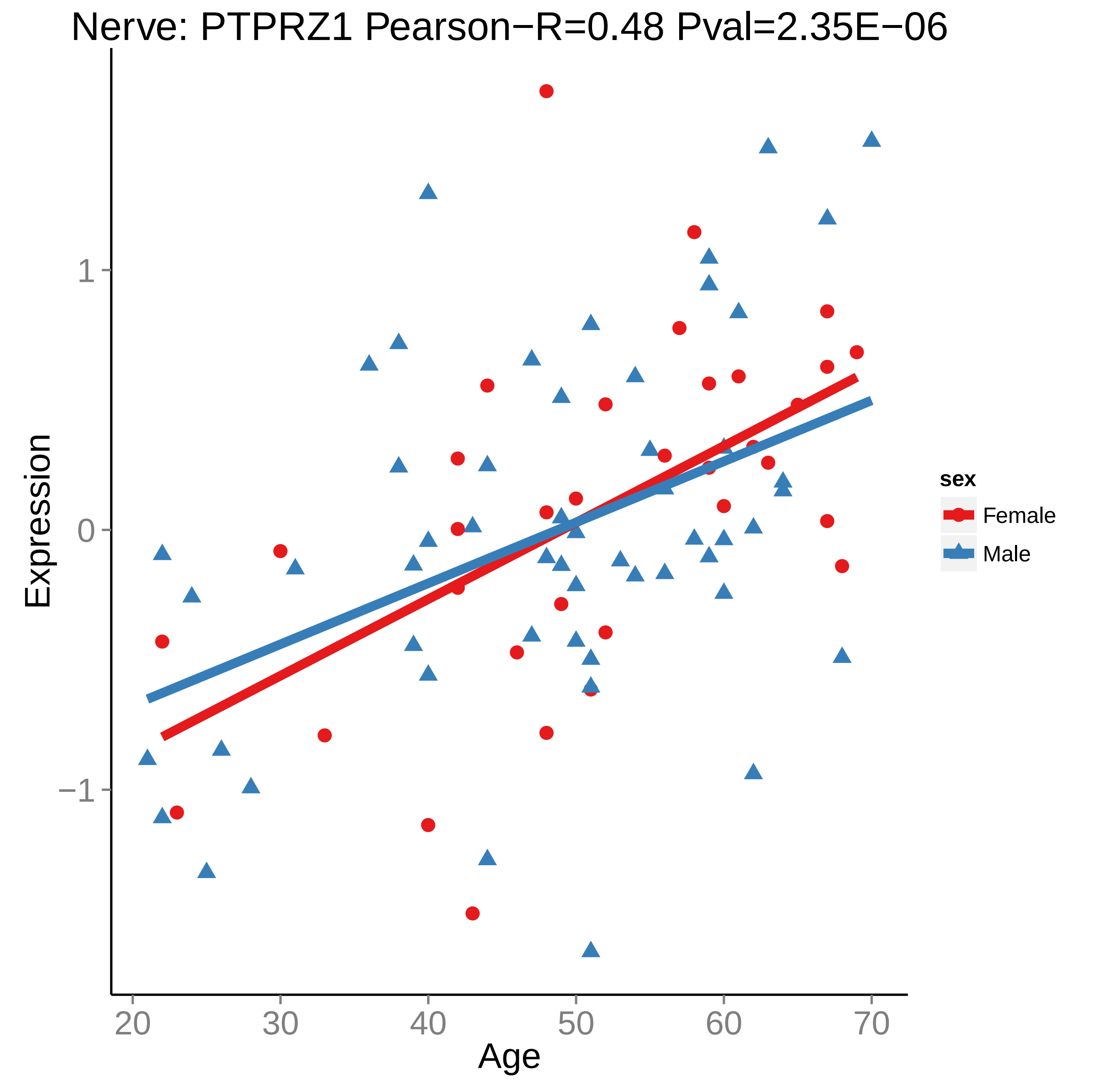

Nerve: PTPRZ1 Pearson−R=0.48 Pval=2.35E−06
Expression
1
0
−1
20
30
40
50
60
70
Age
sex
Female
Male

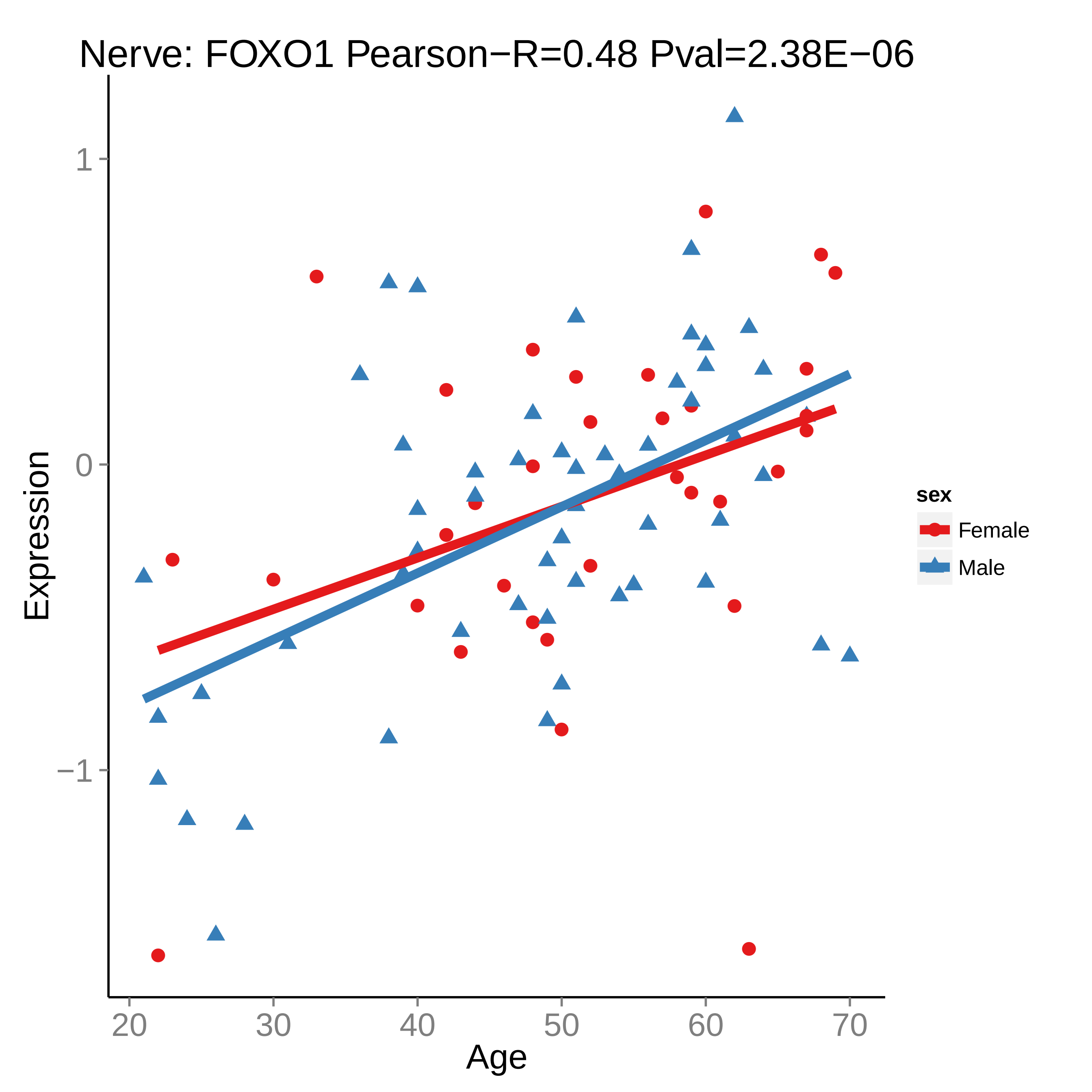
Nerve: FOXO1 Pearson−R=0.48 Pval=2.38E−06
Expression
1
0
−1
20
30
40
50
60
70
Age
sex
Female
Male

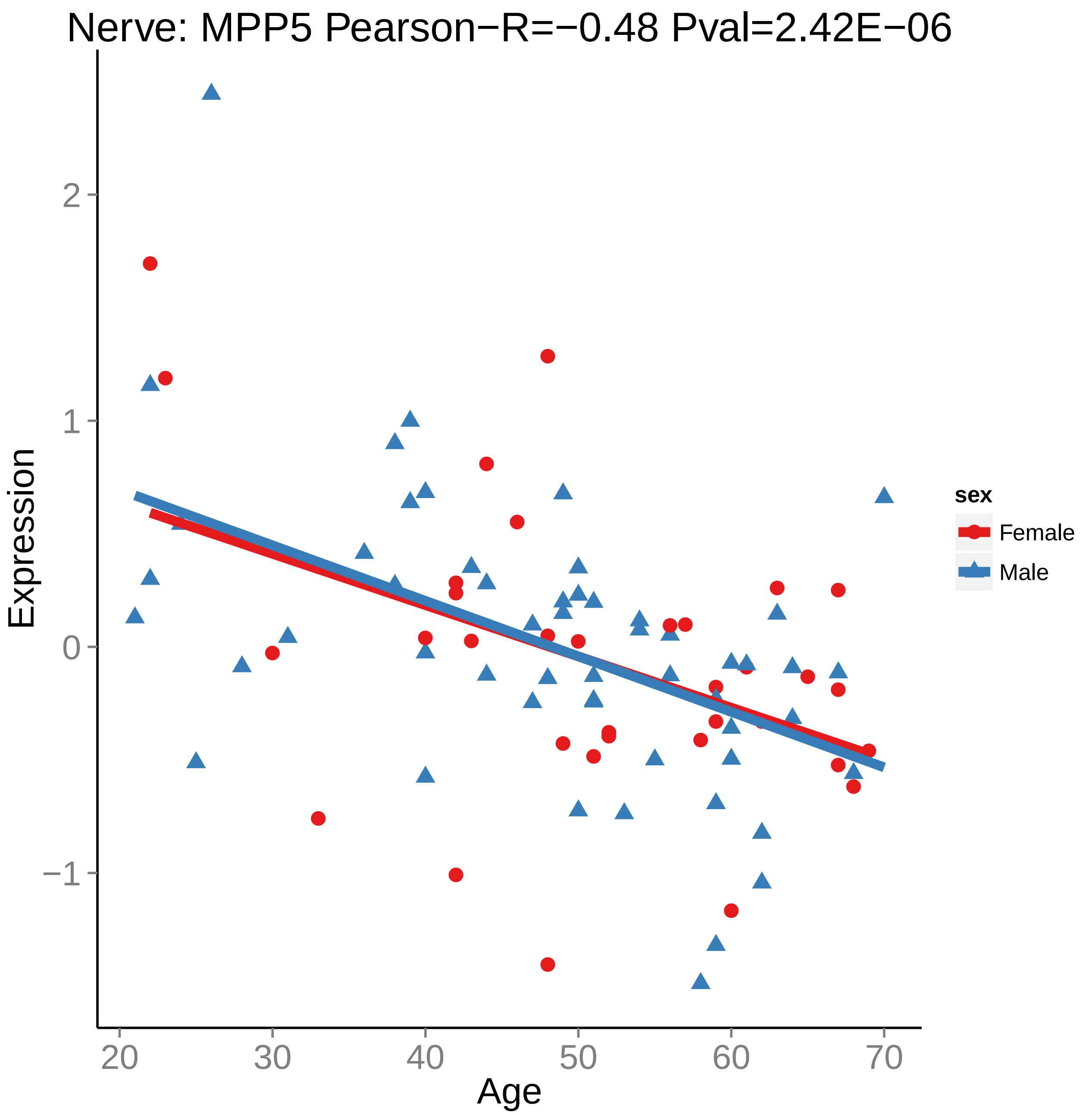
Nerve: MPP5 Pearson−R=−0.48 Pval=2.42E−06
Expression
Age
2
1
0
−1
20
30
40
50
60
70
sex
Female
Male

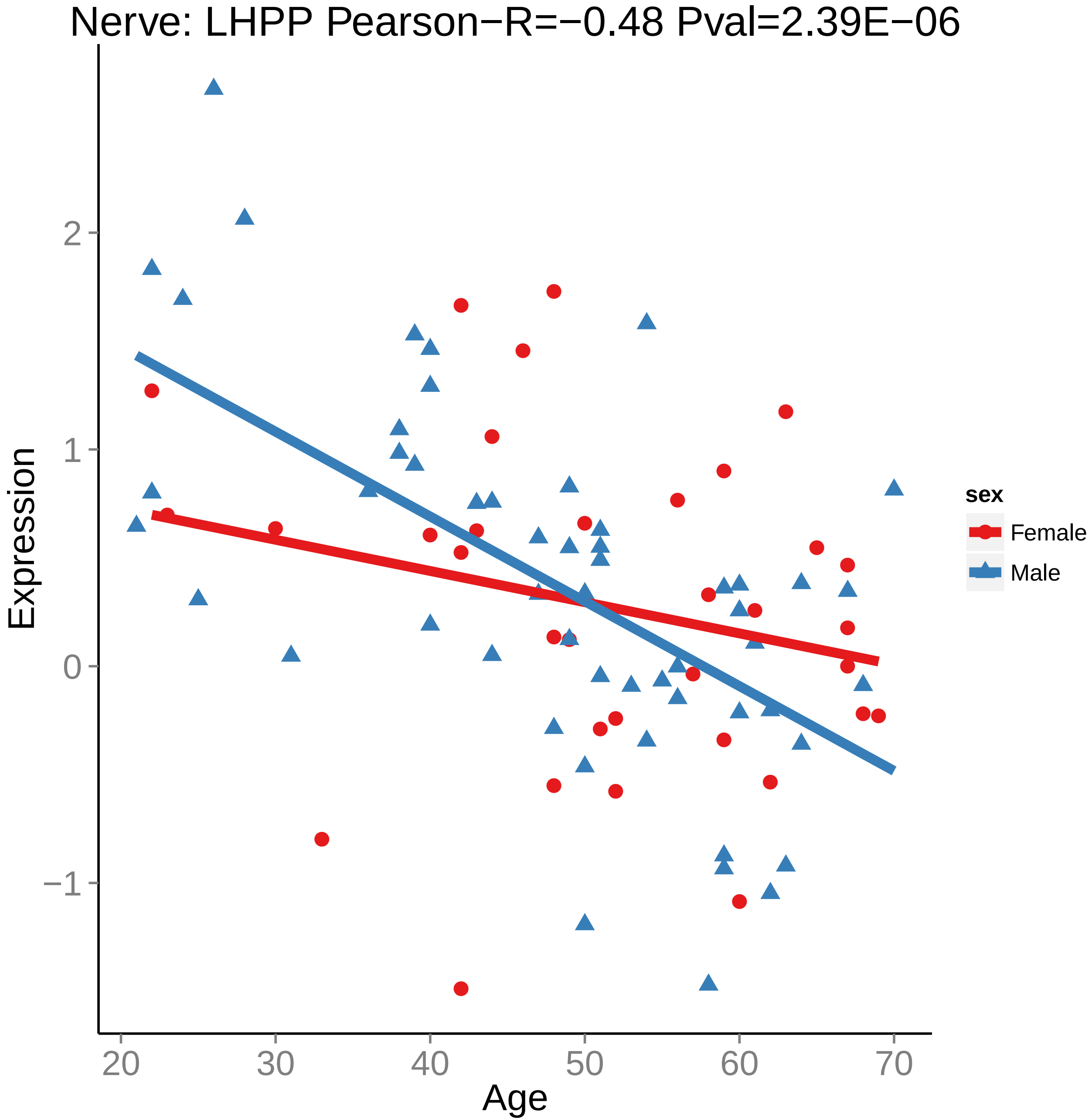

Nerve: LHPP Pearson−R=−0.48 Pval=2.39E−06
Expression
Age
sex
Female
Male
2
1
0
−1
20
30
40
50
60
70

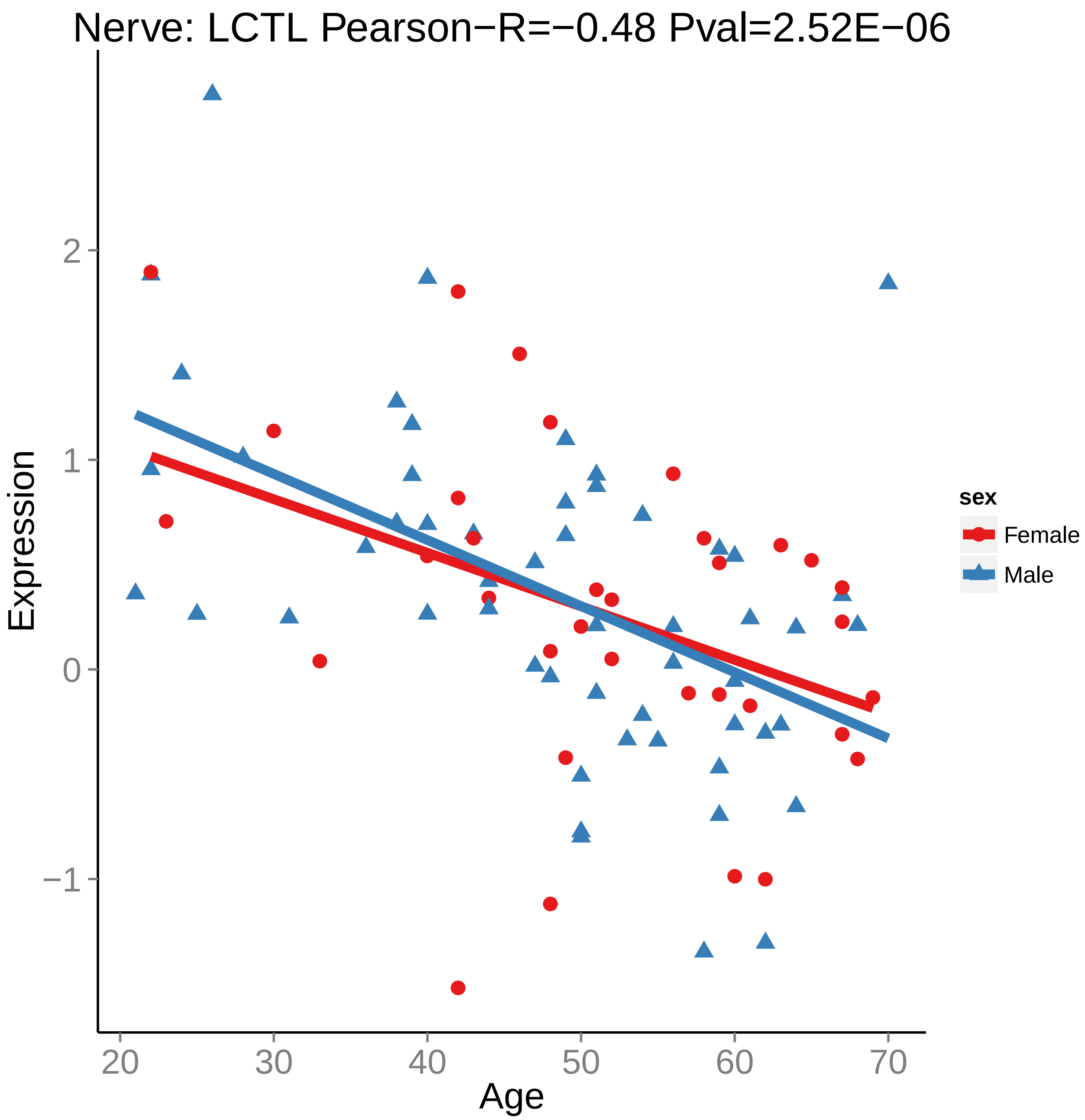
Nerve: LCTL Pearson−R=−0.48 Pval=2.52E−06
Expression
Age
2
1
0
−1
20
30
40
50
60
70
sex
Female
Male

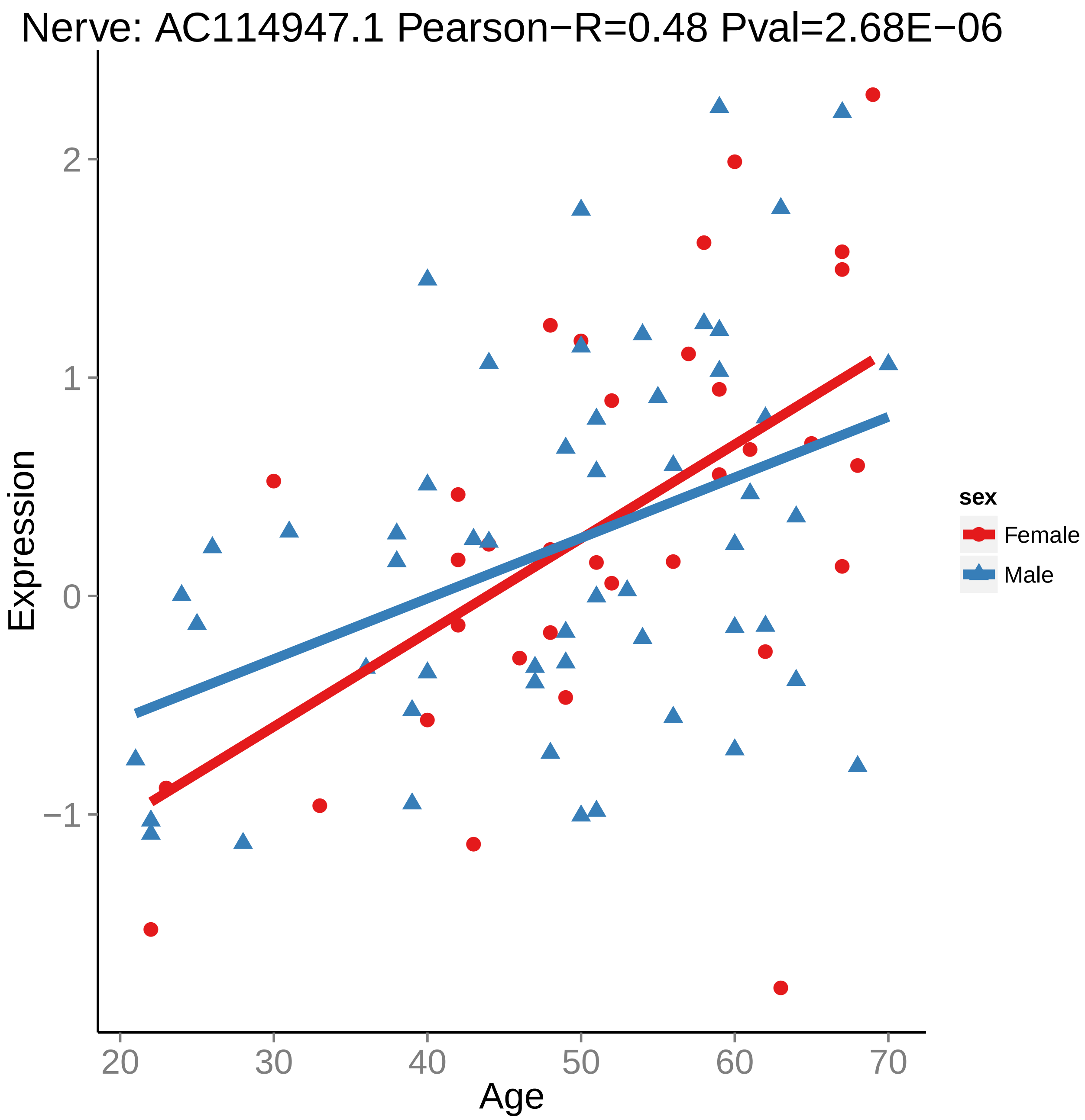

Nerve: AC114947.1 Pearson−R=0.48 Pval=2.68E−06
Expression
2
1
0
−1
20
30
40
50
60
70
Age
sex
Female
Male

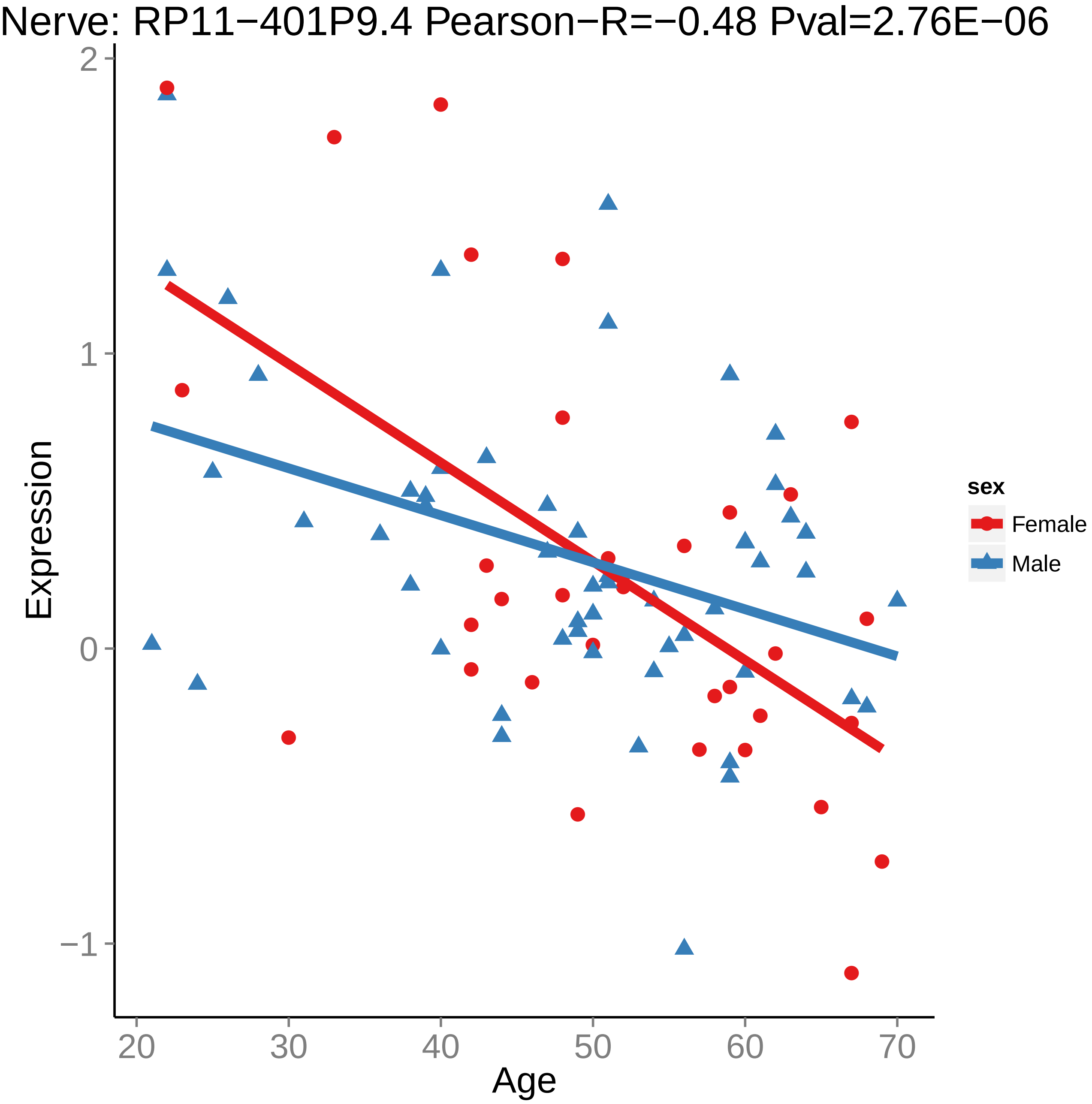

Nerve: RP11−401P9.4 Pearson−R=−0.48 Pval=2.76E−06
Expression
Age
2
1
0
−1
20
30
40
50
60
70
sex
Female
Male

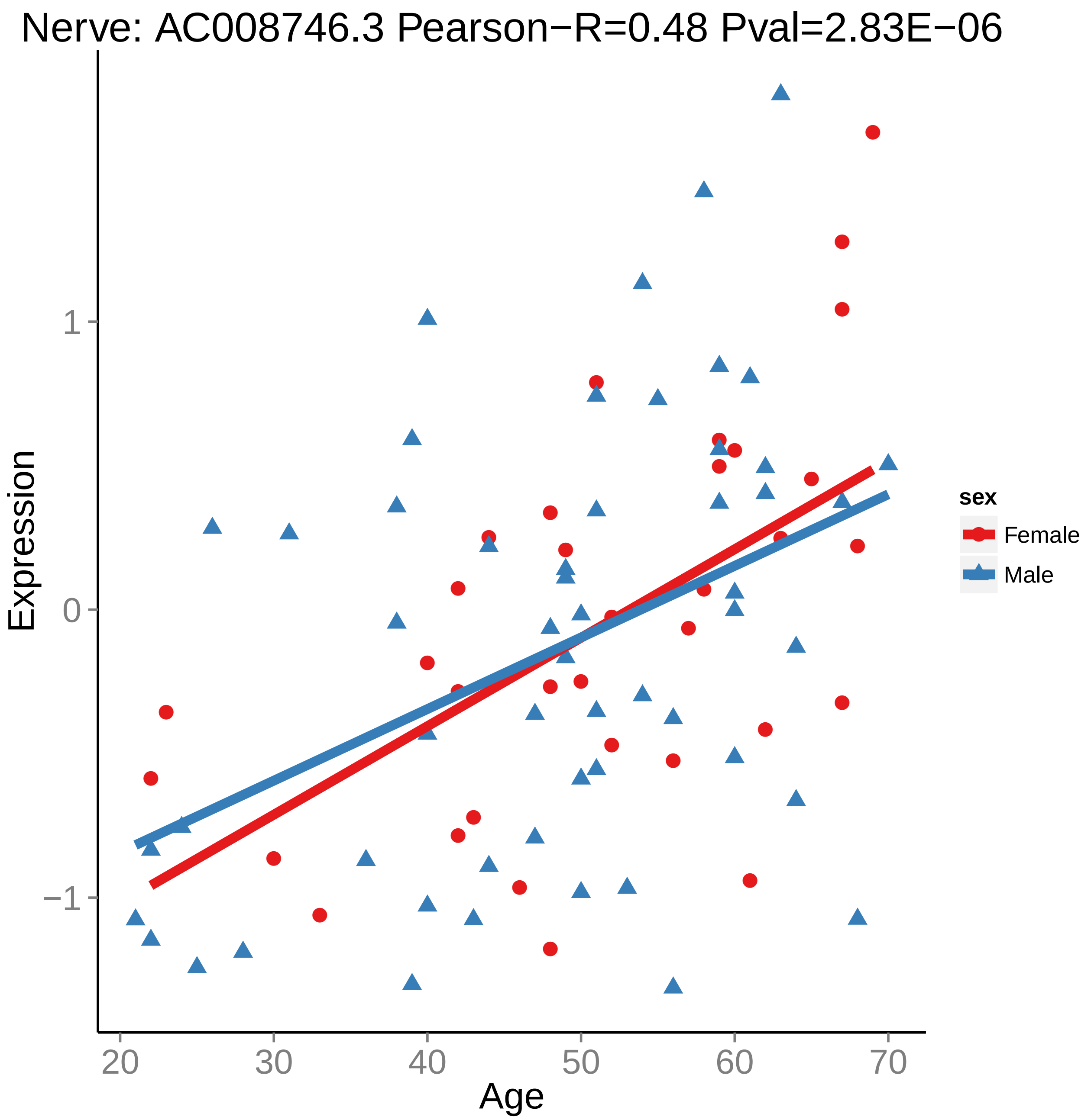

Nerve: AC008746.3 Pearson−R=0.48 Pval=2.83E−06
Expression
1
0
−1
20
30
40
50
60
70
Age
sex
Female
Male

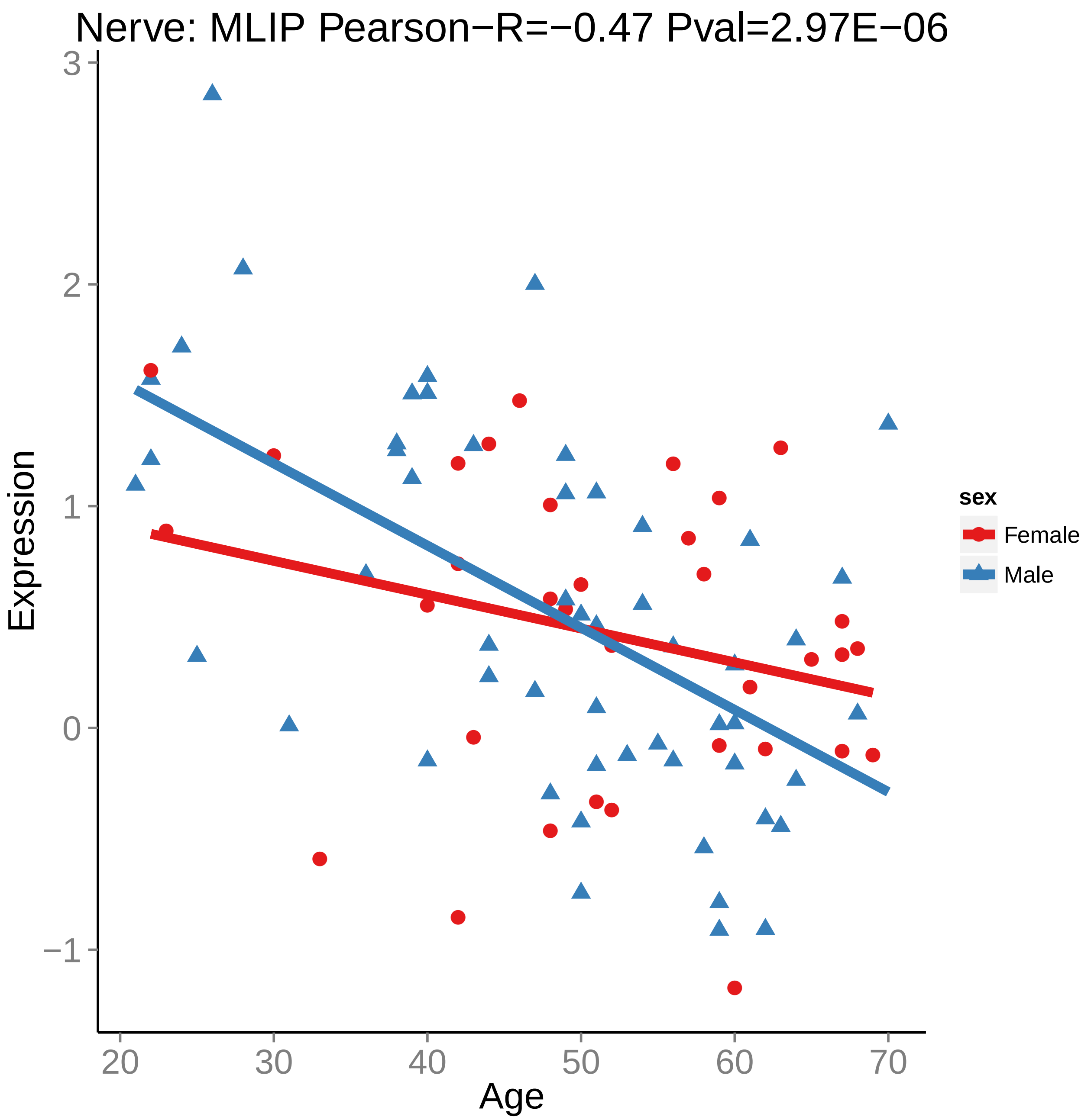

Nerve: MLIP Pearson−R=−0.47 Pval=2.97E−06
Expression
Age
sex
Female
Male
3
2
1
0
−1
20
30
40
50
60
70

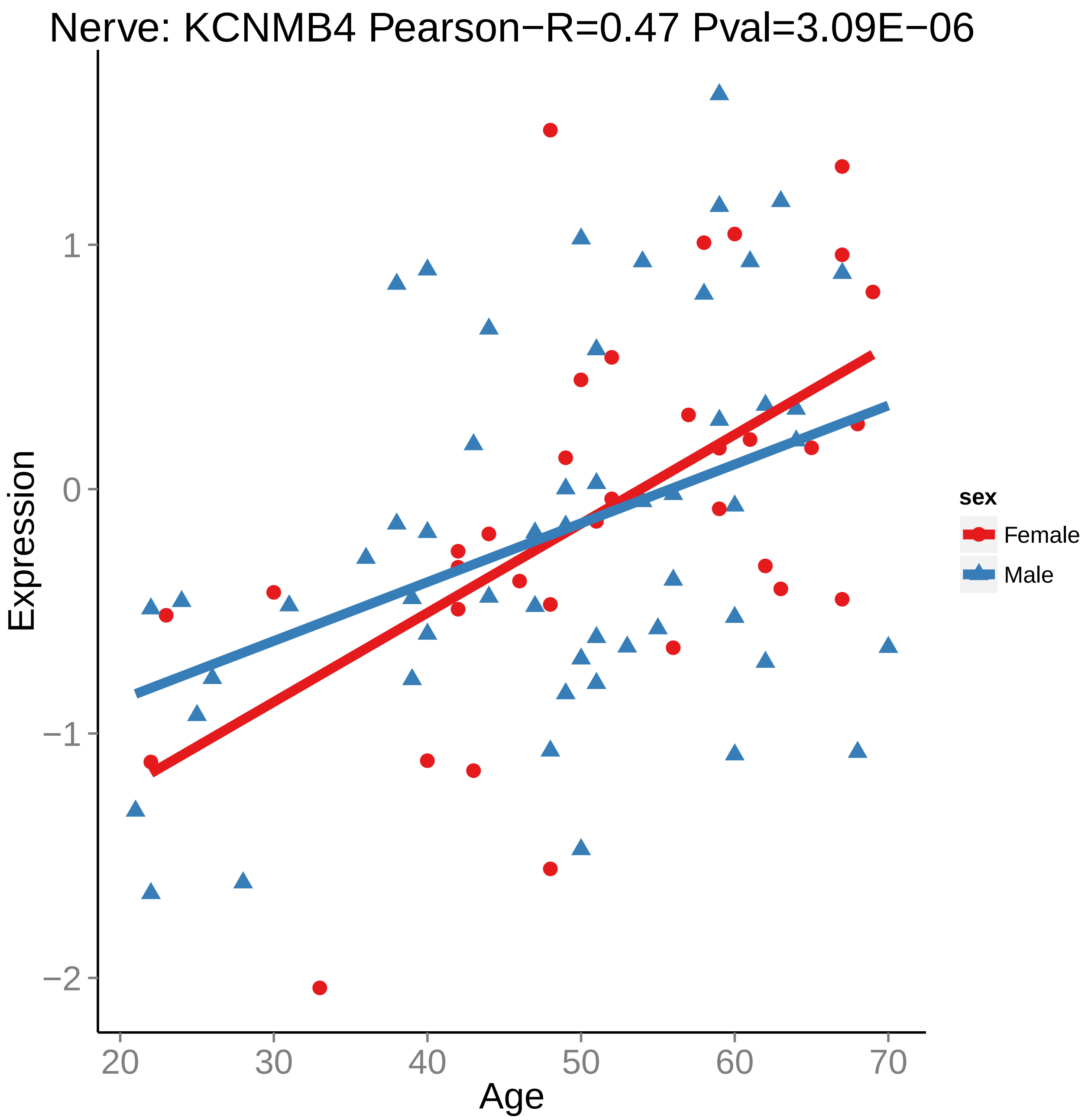

Nerve: KCNMB4 Pearson−R=0.47 Pval=3.09E−06
Expression
1
0
−1
−2
20
30
40
50
60
70
Age
sex
Female
Male

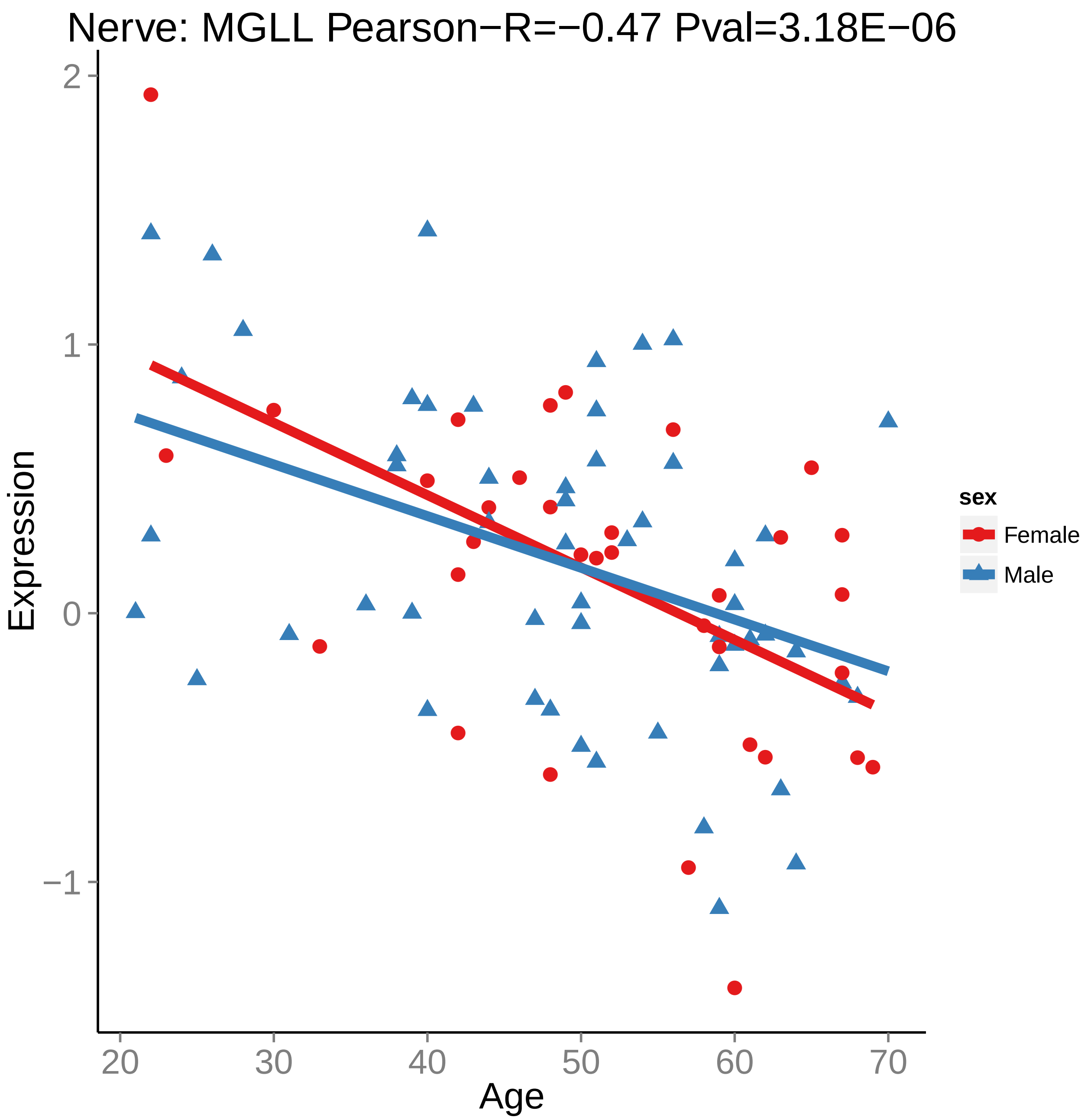

Nerve: MGLL Pearson−R=−0.47 Pval=3.18E−06
2
1
0
−1
Expression
20
30
40
50
60
70
Age
sex
Female
Male

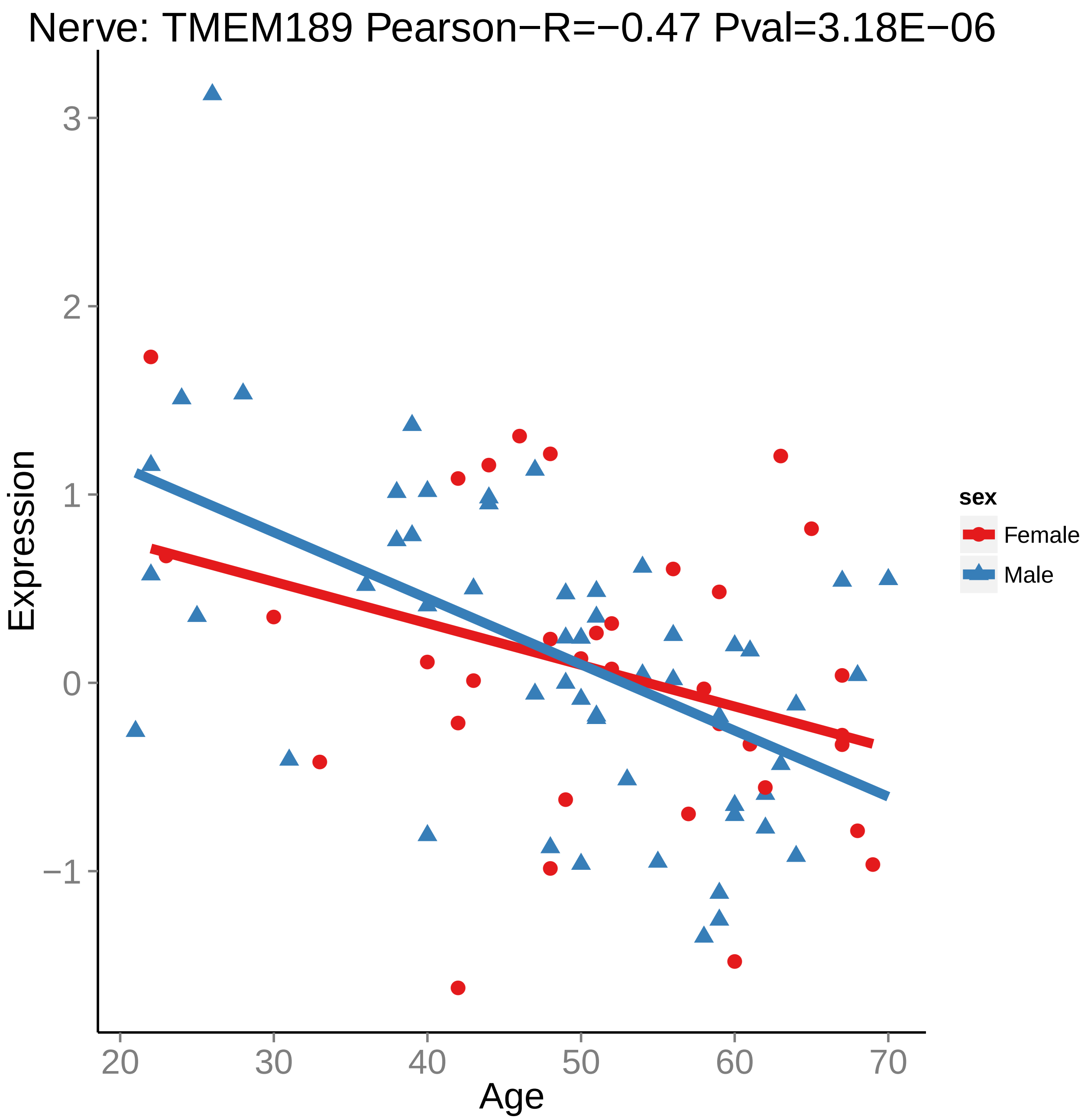

Nerve: TMEM189 Pearson−R=−0.47 Pval=3.18E−06
3
2
1
0
−1
Expression
20
30
40
50
60
70
Age
sex
Female
Male

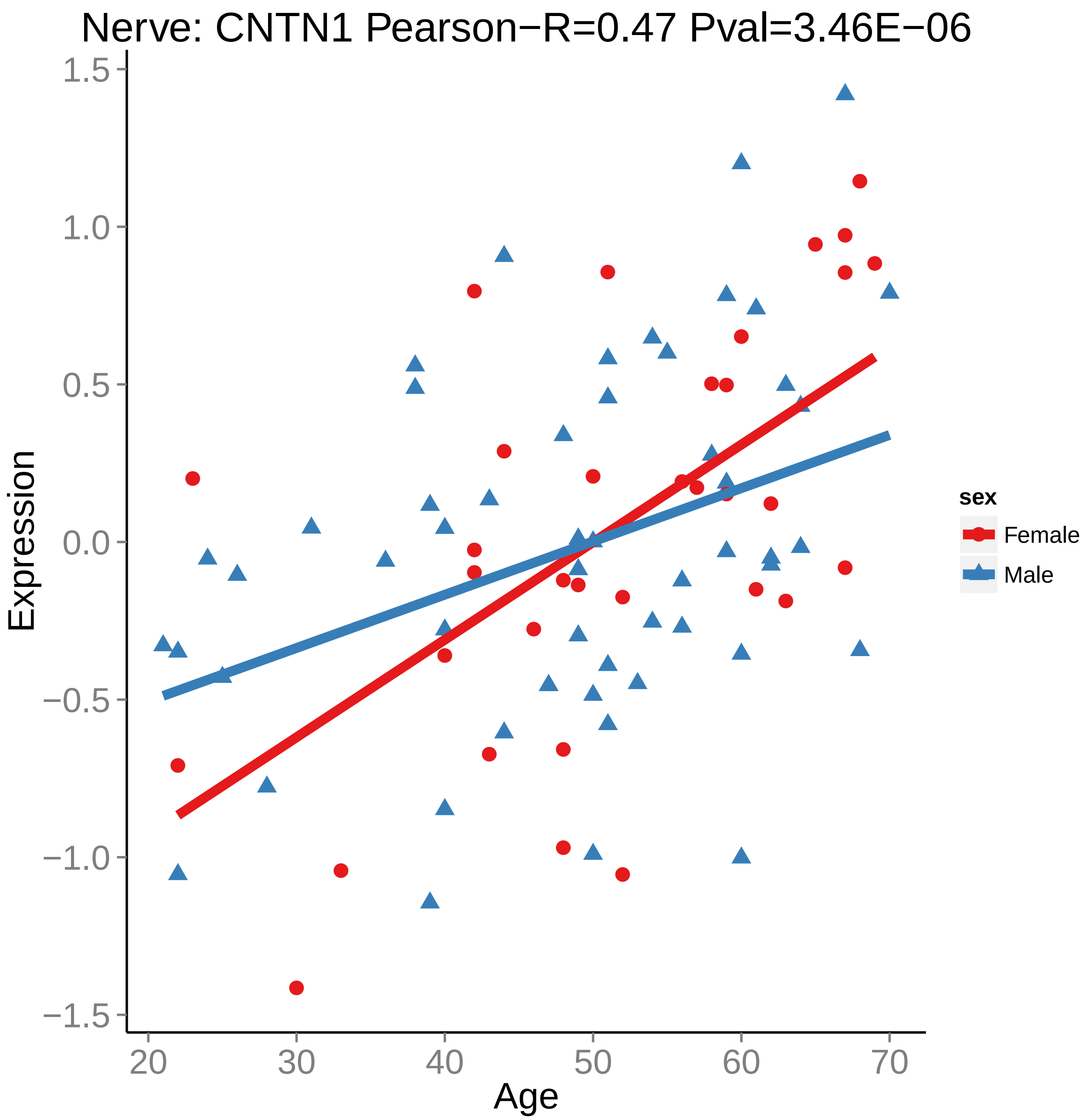

Nerve: CNTN1 Pearson−R=0.47 Pval=3.46E−06
Expression
1.5
1.0
0.5
0.0
−0.5
−1.0
−1.5
20
30
40
50
60
70
Age
sex
Female
Male

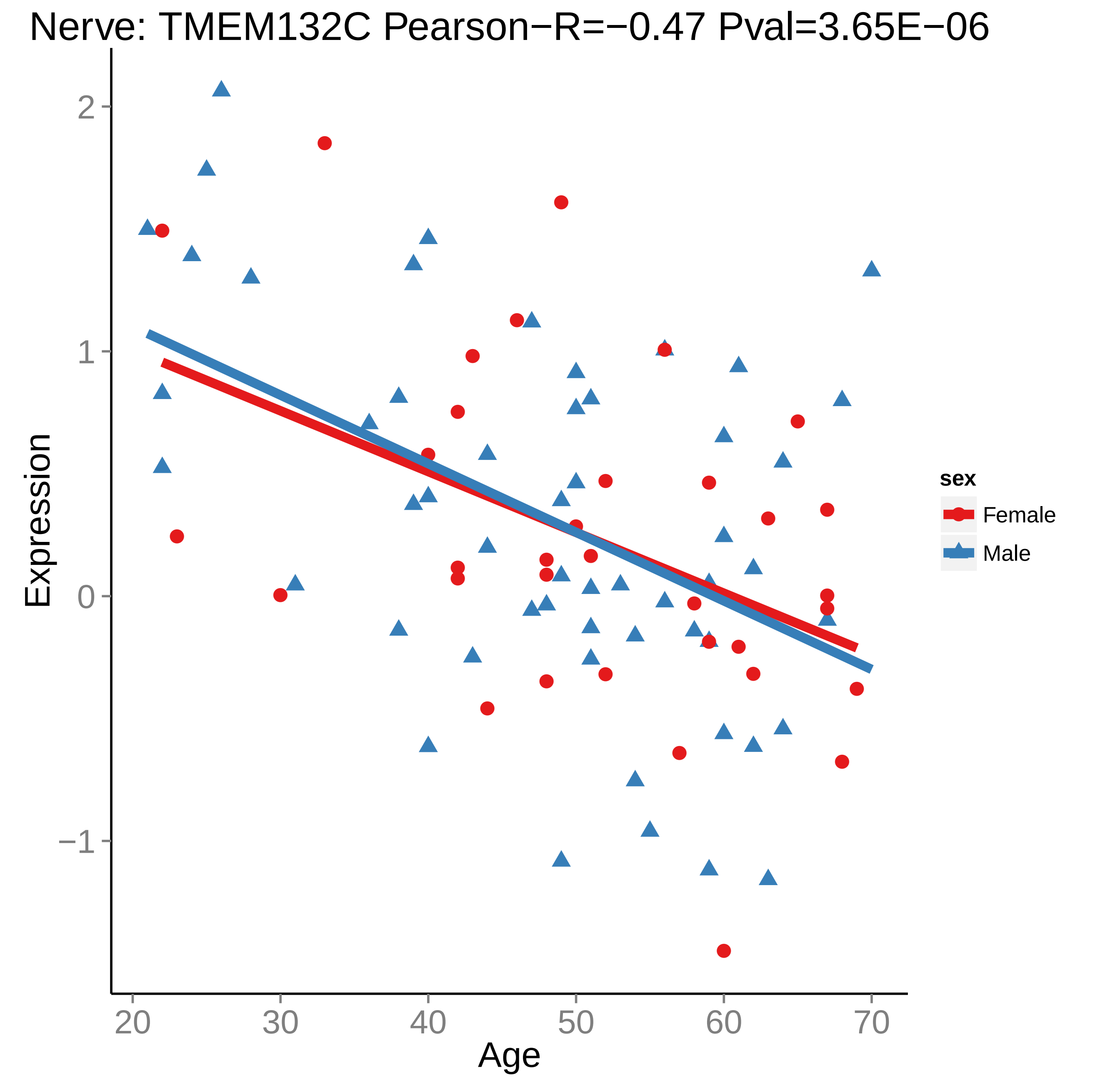

Nerve: TMEM132C Pearson−R=−0.47 Pval=3.65E−06
Expression
2
1
0
−1
Age
20
30
40
50
60
70
sex
Female
Male

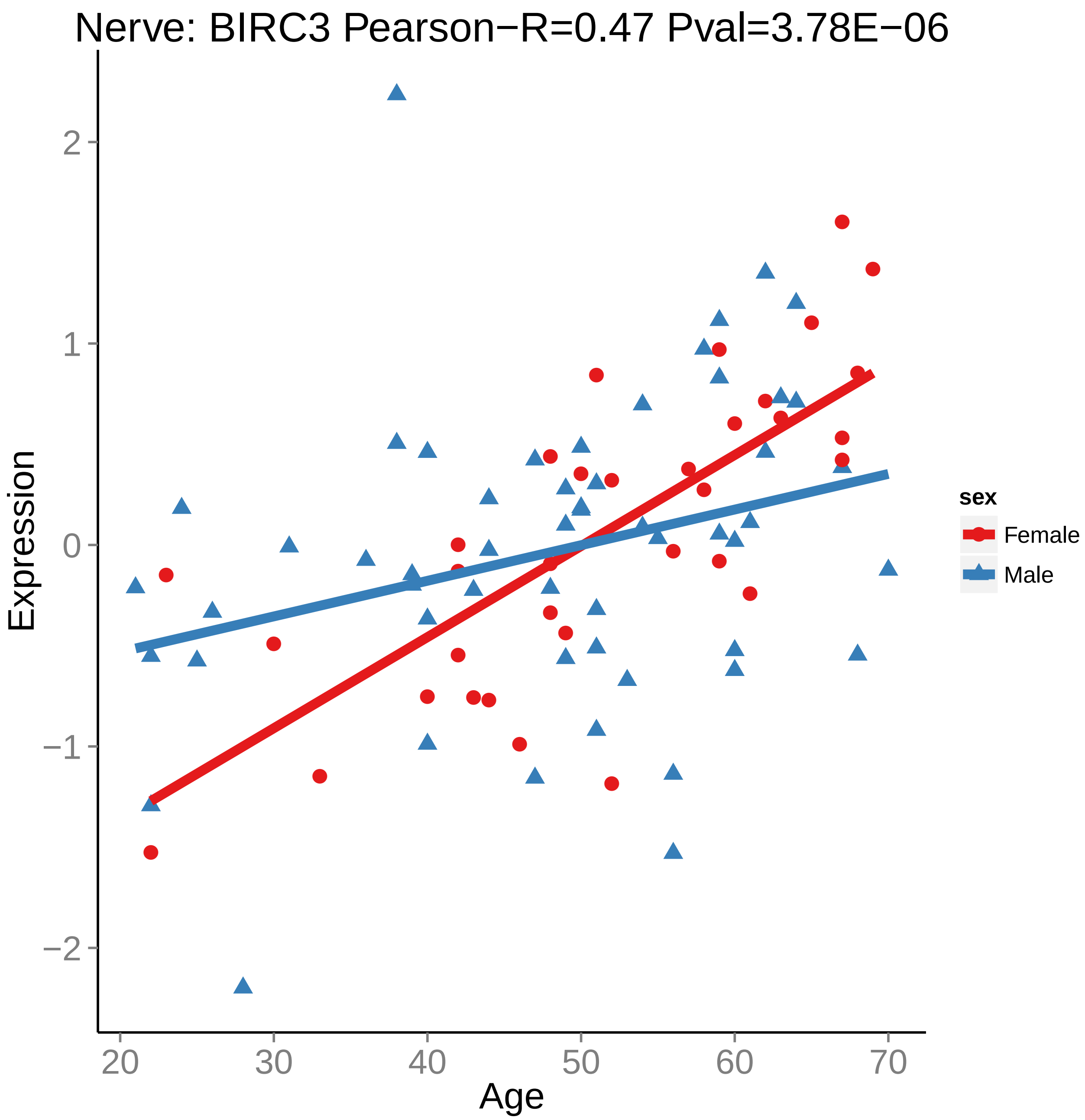

Nerve: BIRC3 Pearson−R=0.47 Pval=3.78E−06
Expression
2
1
0
−1
−2
20
30
40
50
60
70
Age
sex
Female
Male

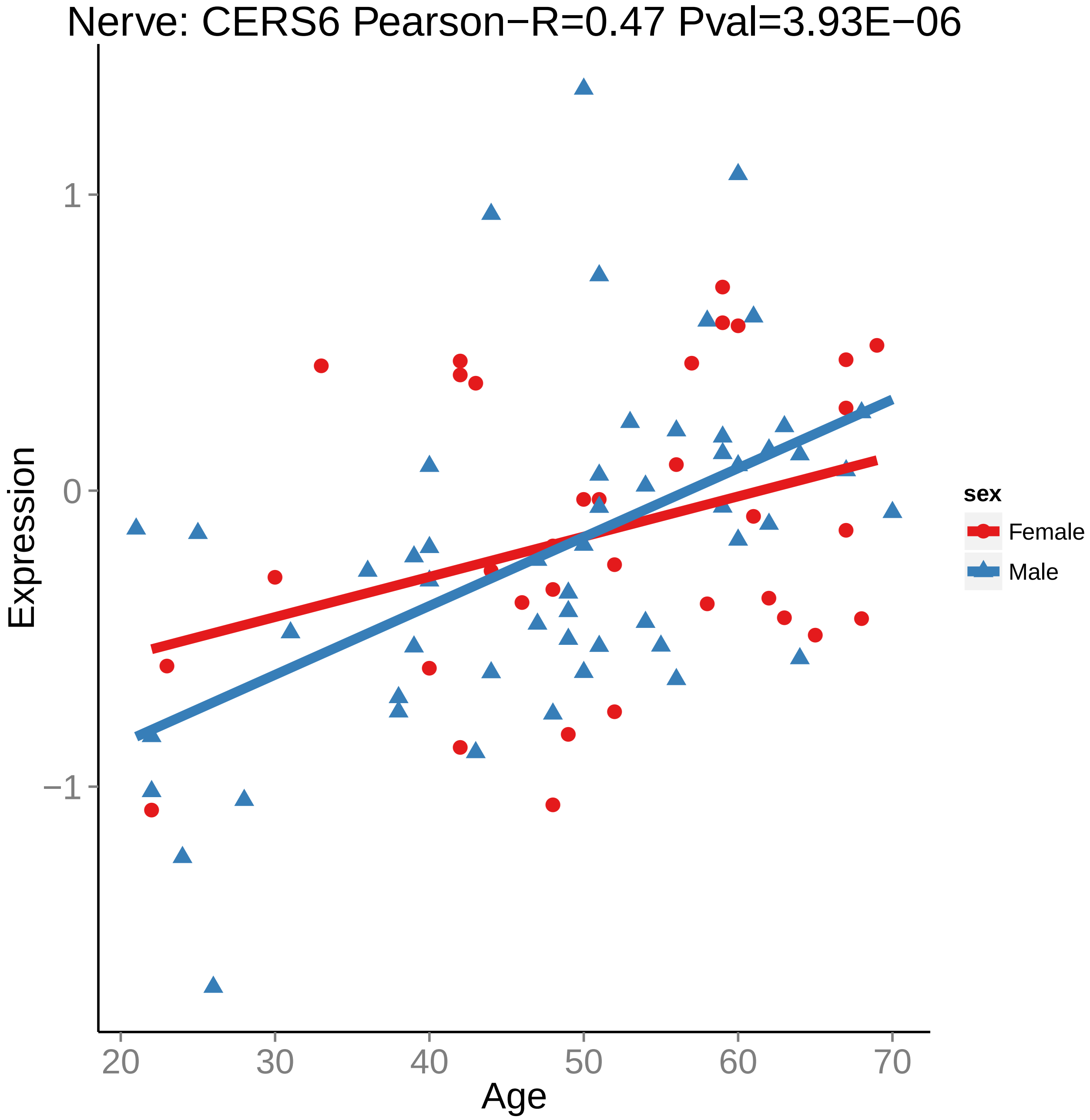

Nerve: CERS6 Pearson−R=0.47 Pval=3.93E−06
Expression
1
0
−1
20
30
40
50
60
70
Age
sex
Female
Male

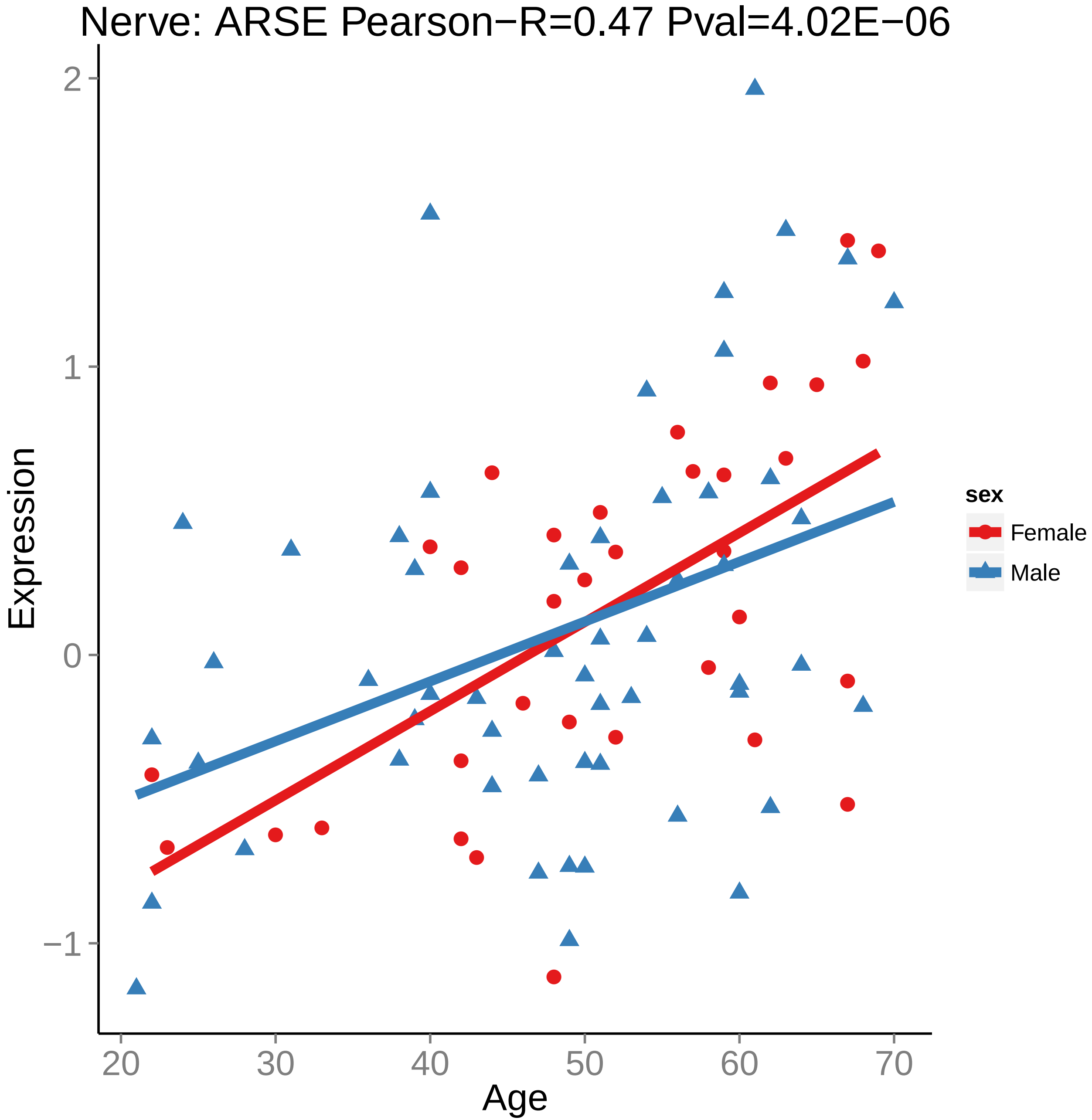

Nerve: ARSE Pearson−R=0.47 Pval=4.02E−06
Expression
Age
2
1
0
−1
20
30
40
50
60
70
sex
Female
Male

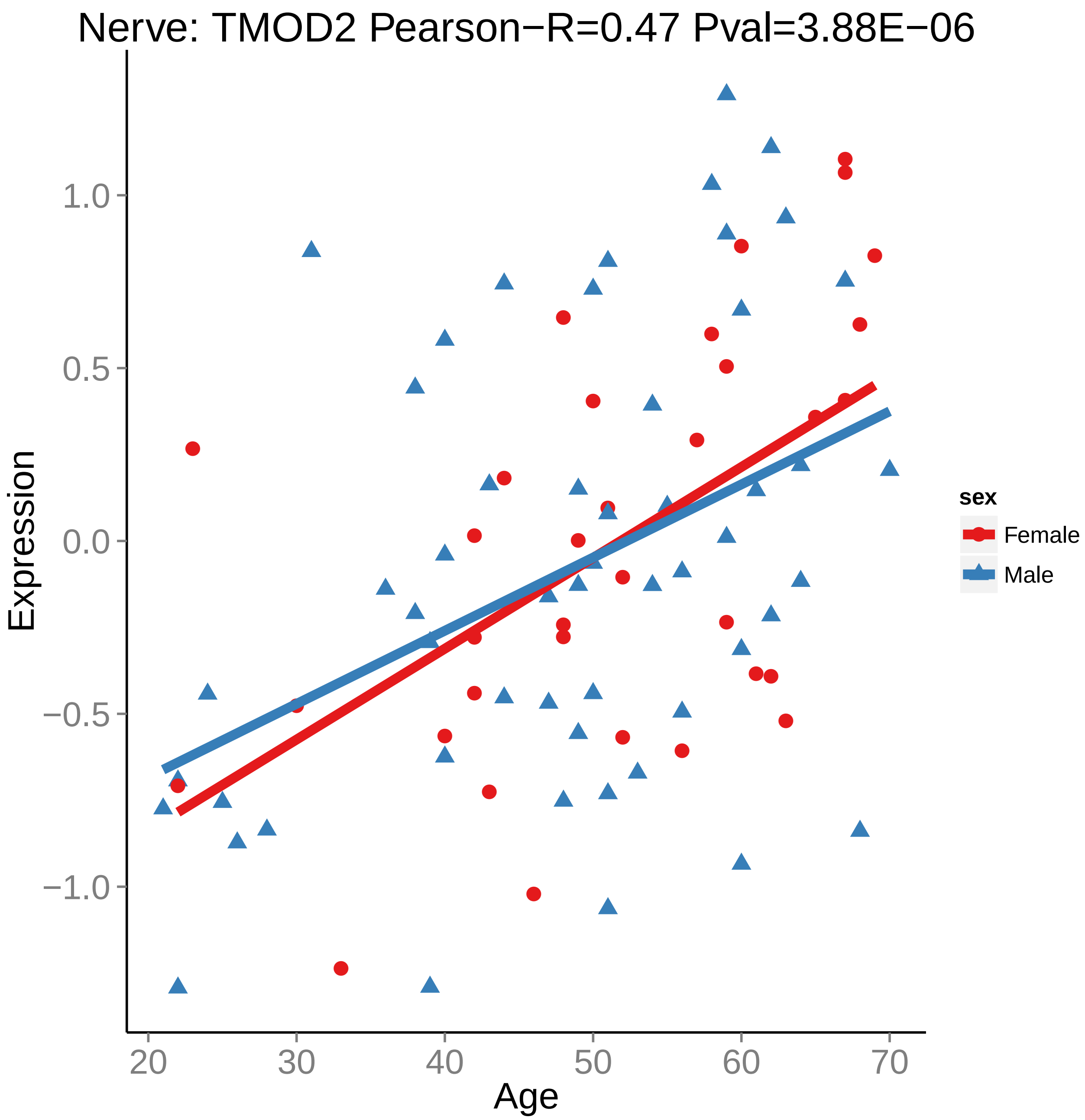
Nerve: TMOD2 Pearson−R=0.47 Pval=3.88E−06
Expression
1.0
0.5
0.0
−0.5
−1.0
20
30
40
50
60
70
Age
sex
Female
Male

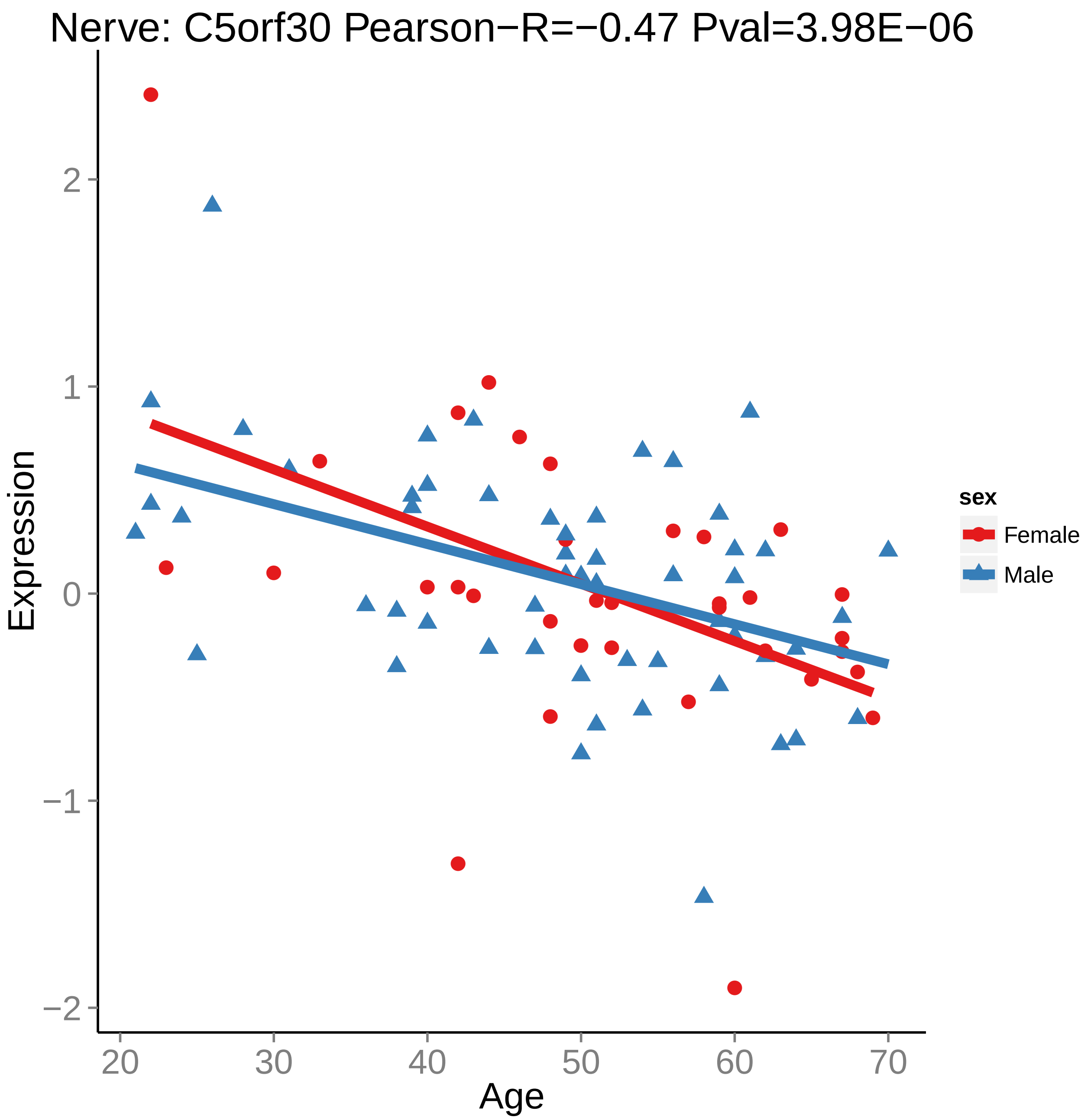
Nerve: C5orf30 Pearson−R=−0.47 Pval=3.98E−06
Expression
Age
sex
Female
Male
2
1
0
−1
−2
20
30
40
50
60
70

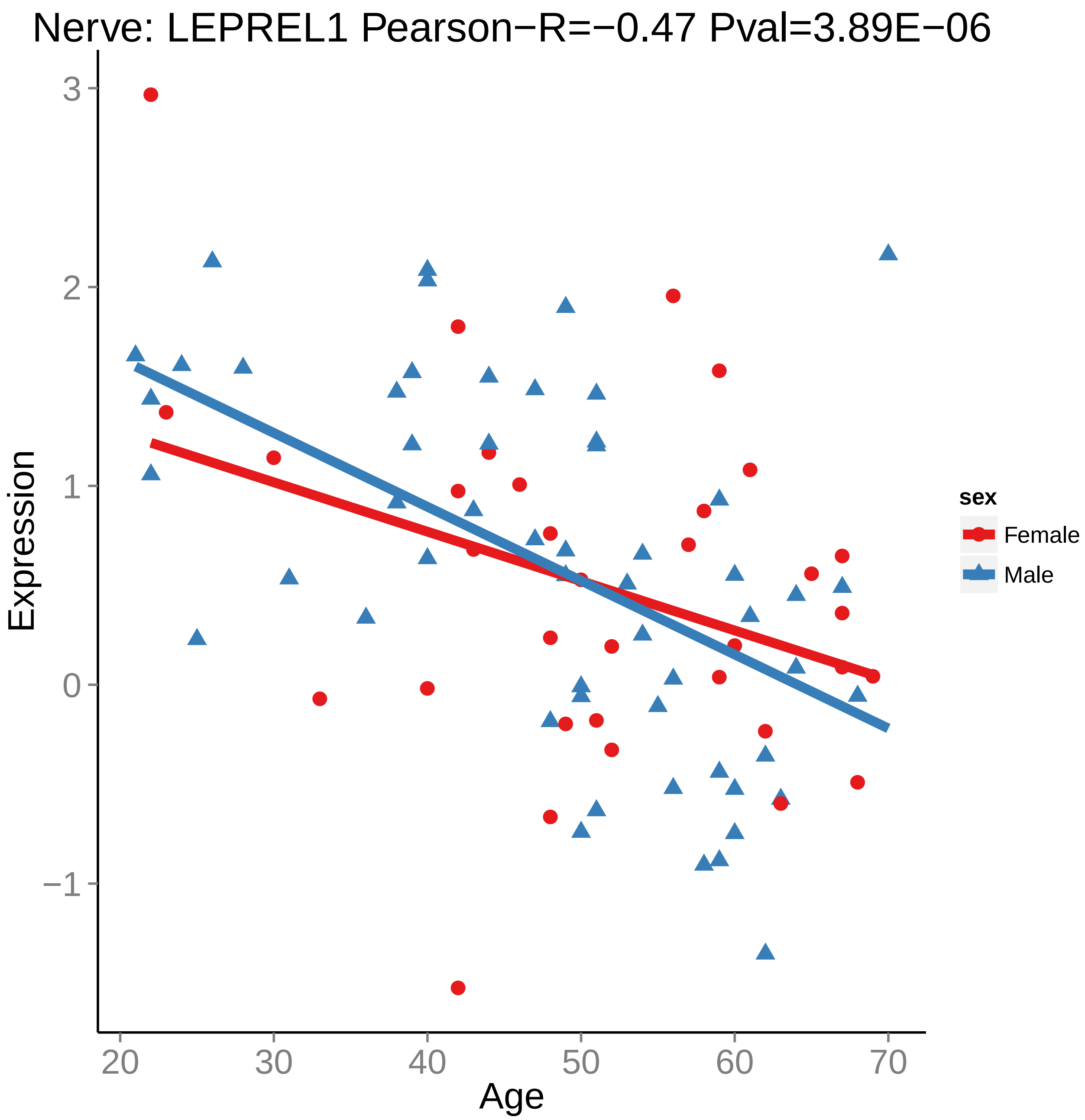

Nerve: LEPREL1 Pearson−R=−0.47 Pval=3.89E−06
Expression
Age
sex
Female
Male
3
2
1
0
−1
20
30
40
50
60
70

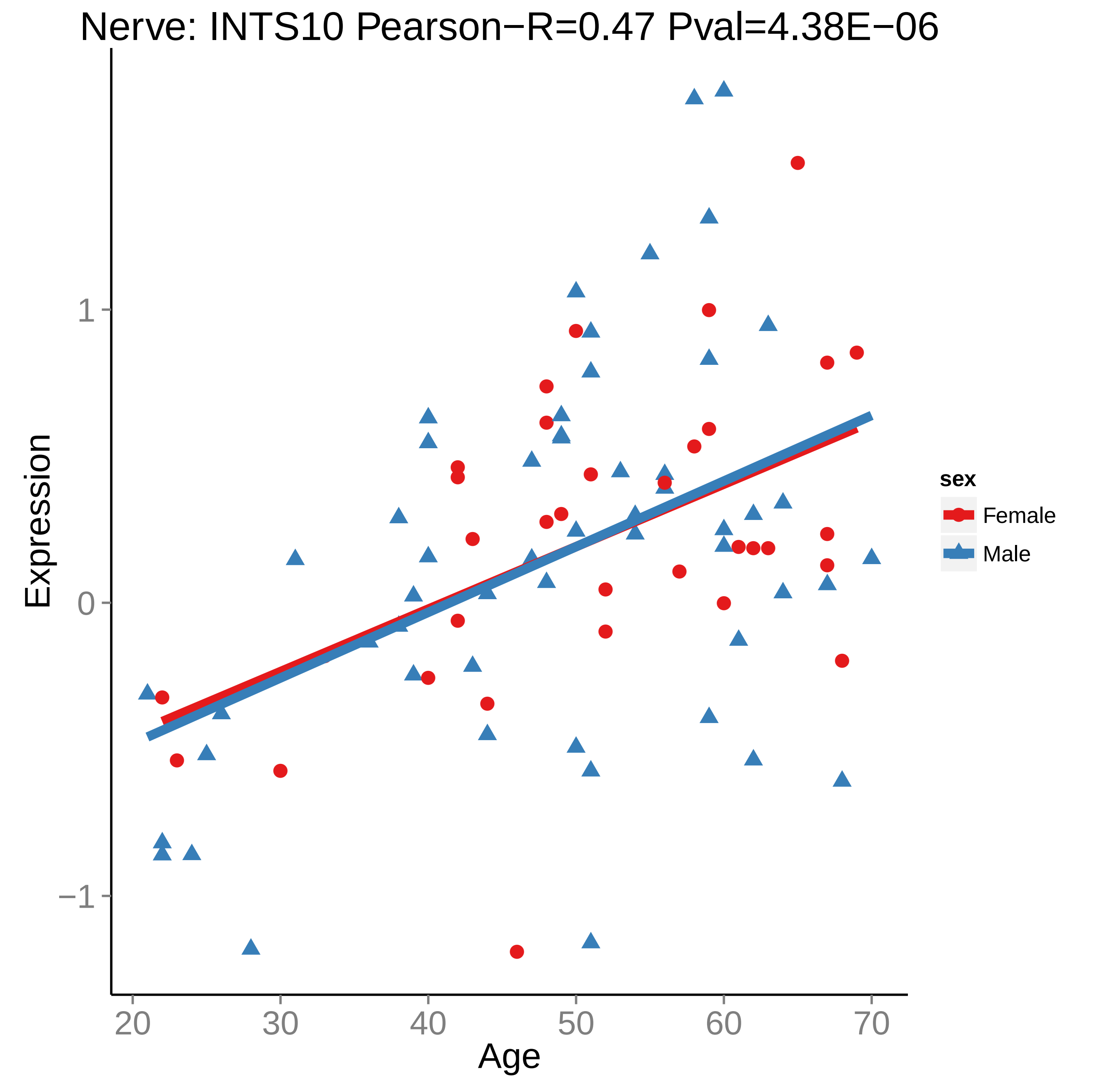

Nerve: INTS10 Pearson−R=0.47 Pval=4.38E−06
Expression
1
0
−1
20
30
40
50
60
70
Age
sex
Female
Male

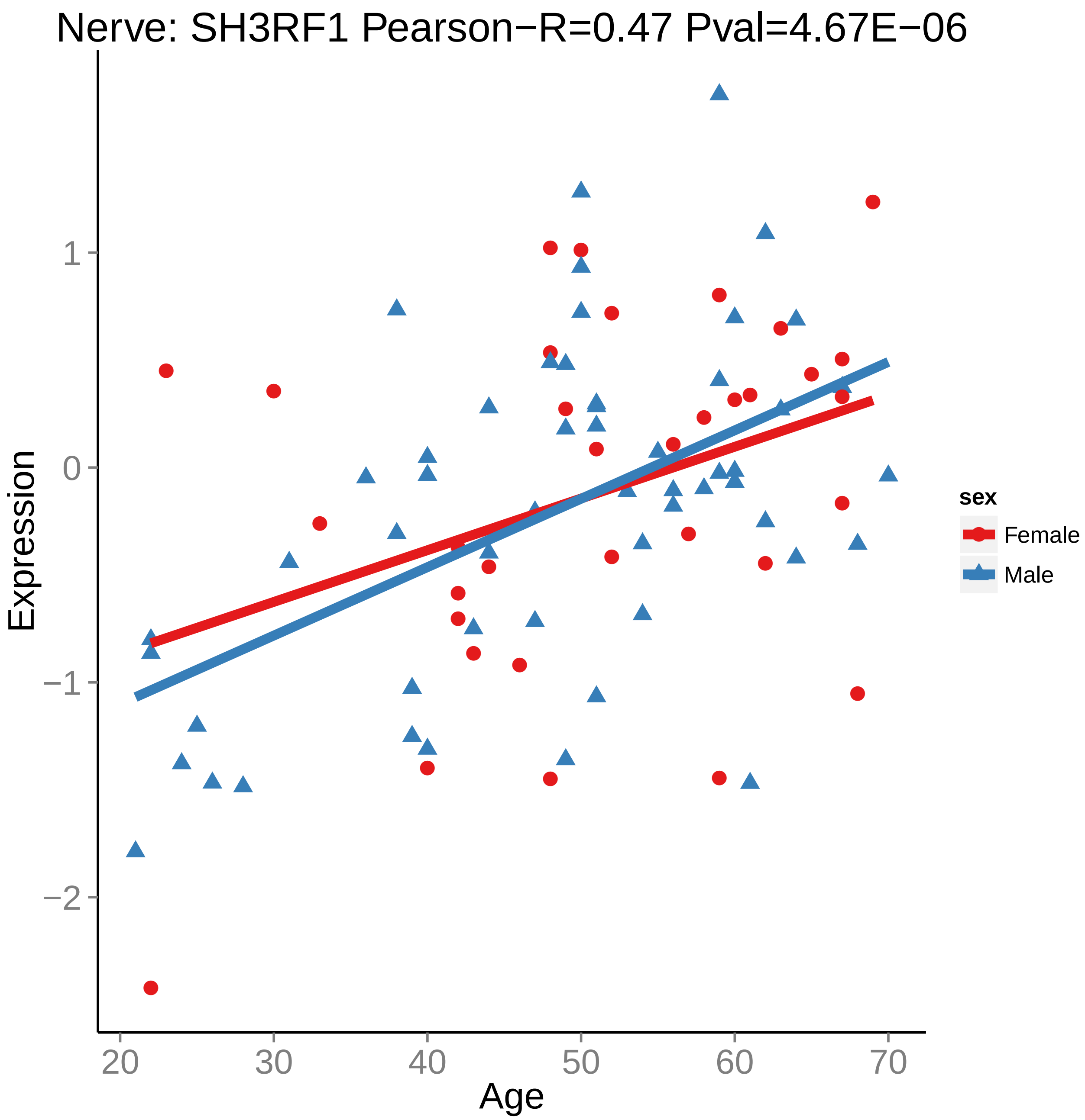

Nerve: SH3RF1 Pearson−R=0.47 Pval=4.67E−06
Expression
Age
1
0
−1
−2
20
30
40
50
60
70
sex
Female
Male

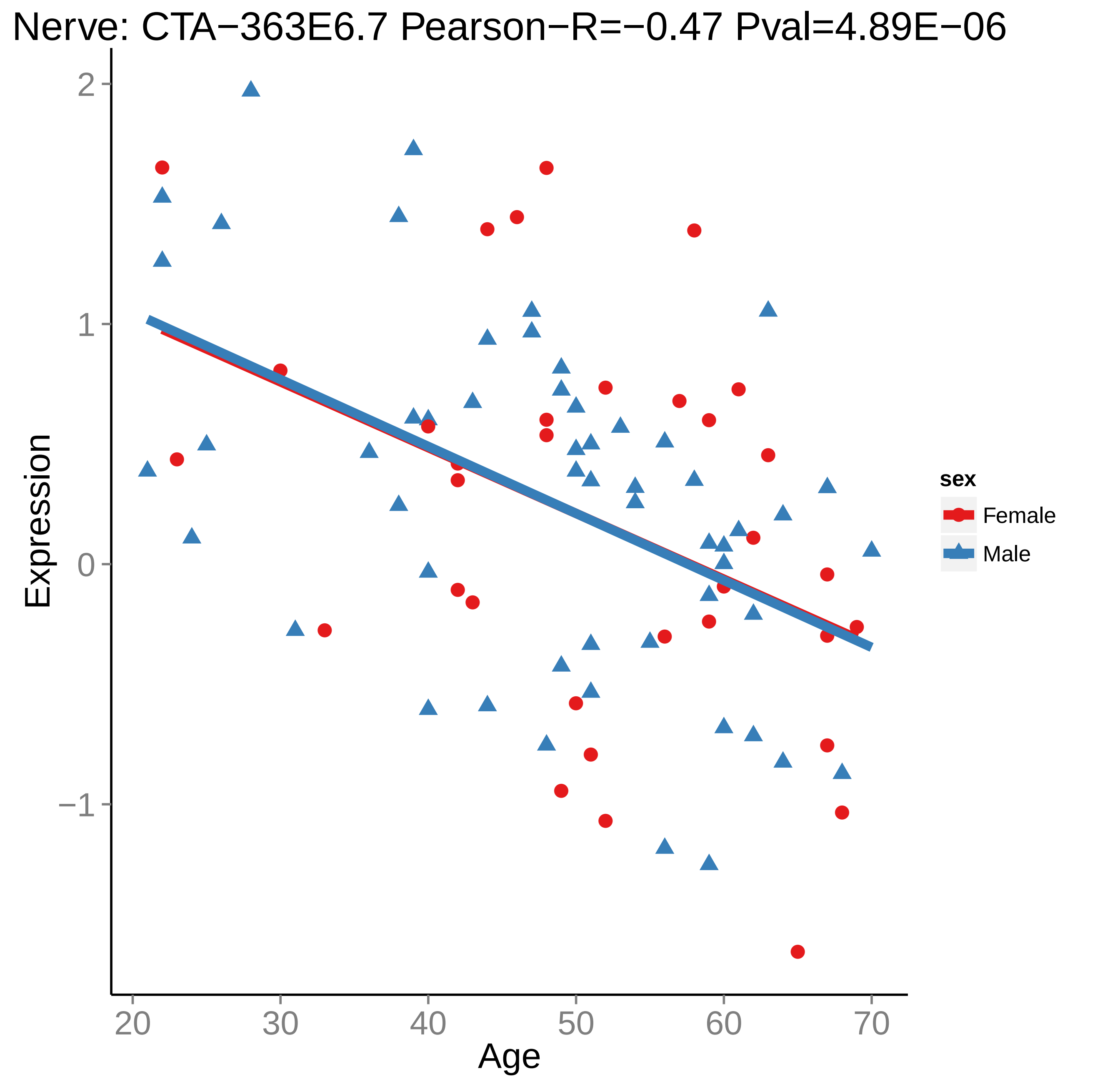
Nerve: CTA−363E6.7 Pearson−R=−0.47 Pval=4.89E−06
Expression
Age
2
1
0
−1
20
30
40
50
60
70
sex
Female
Male

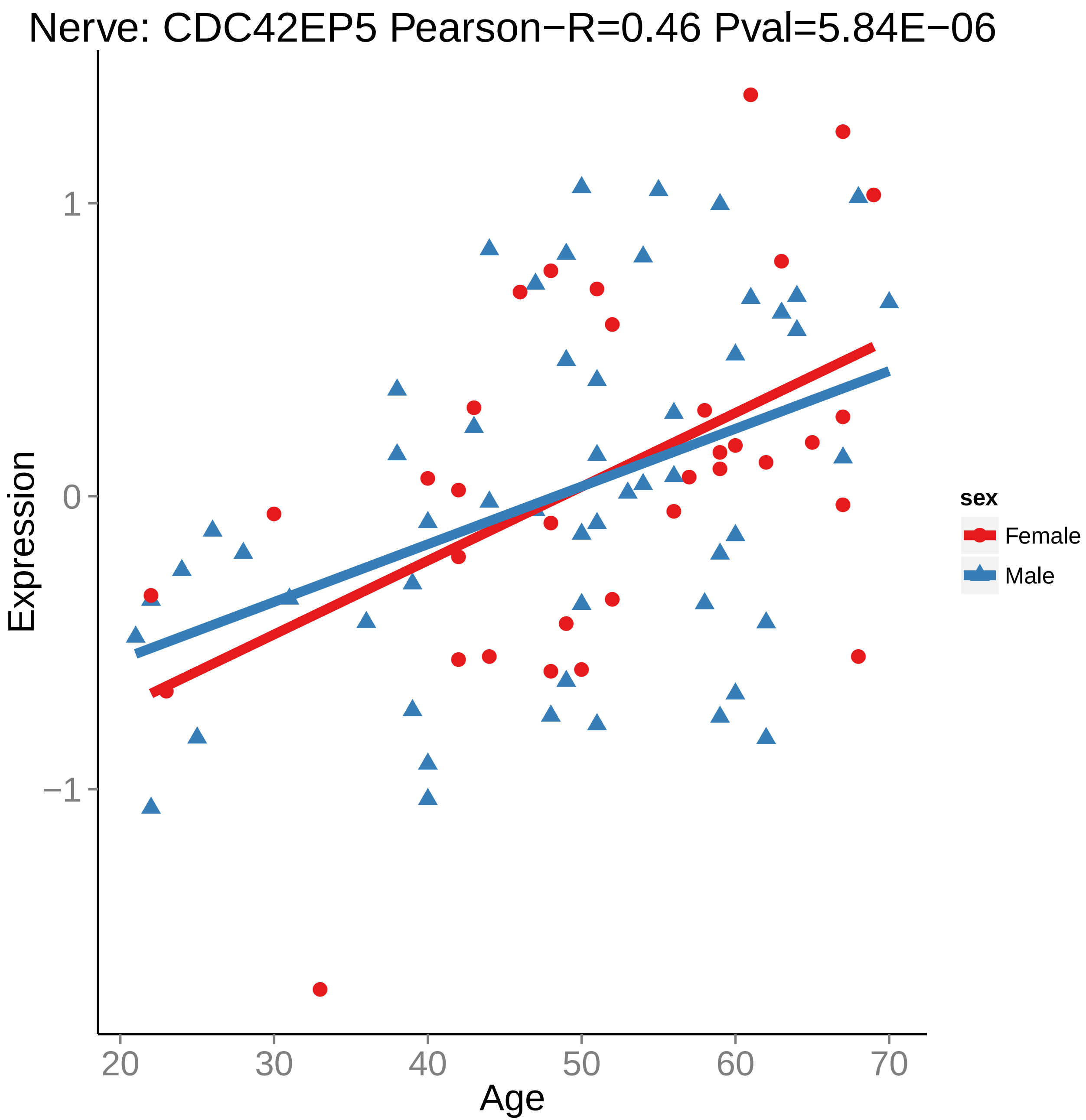

Nerve: CDC42EP5 Pearson−R=0.46 Pval=5.84E−06
Expression
1
0
−1
20
30
40
50
60
70
Age
sex
Female
Male

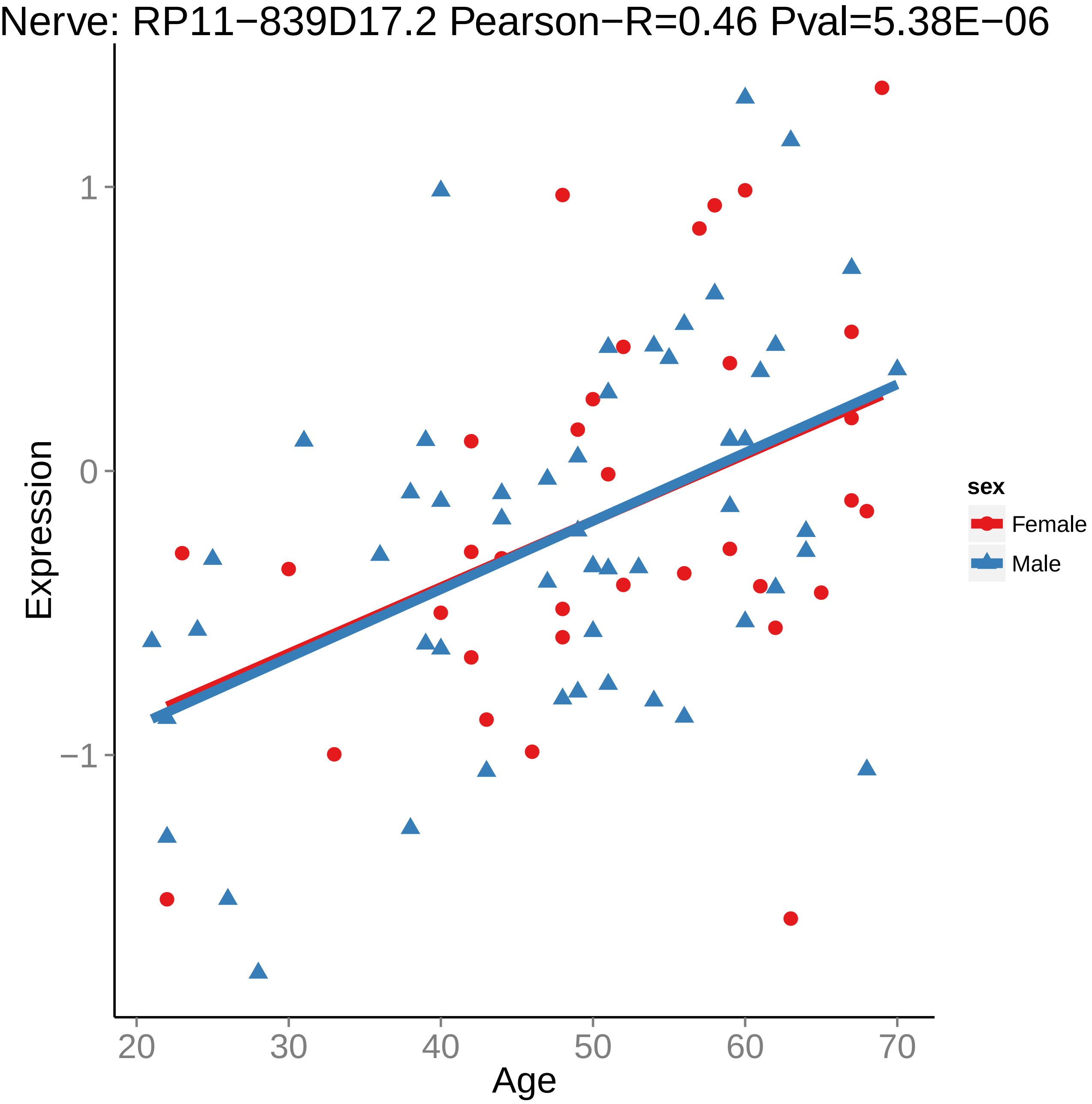

Nerve: RP11−839D17.2 Pearson−R=0.46 Pval=5.38E−06
Expression
1
0
−1
20
30
40
50
60
70
Age
sex
Female
Male

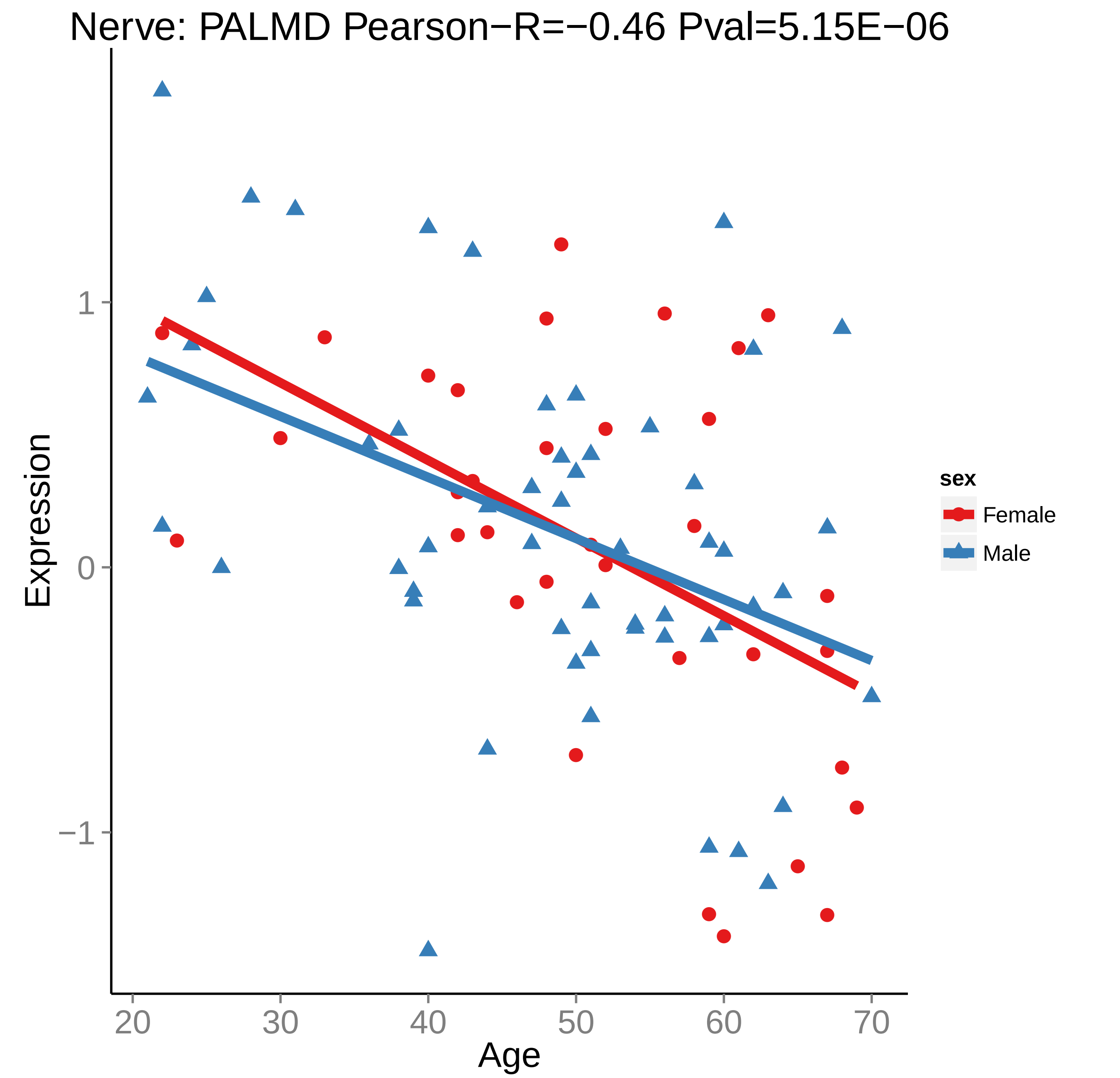

Nerve: PALMD Pearson−R=−0.46 Pval=5.15E−06
Expression
1
0
−1
20
30
40
50
60
70
Age
sex
Female
Male

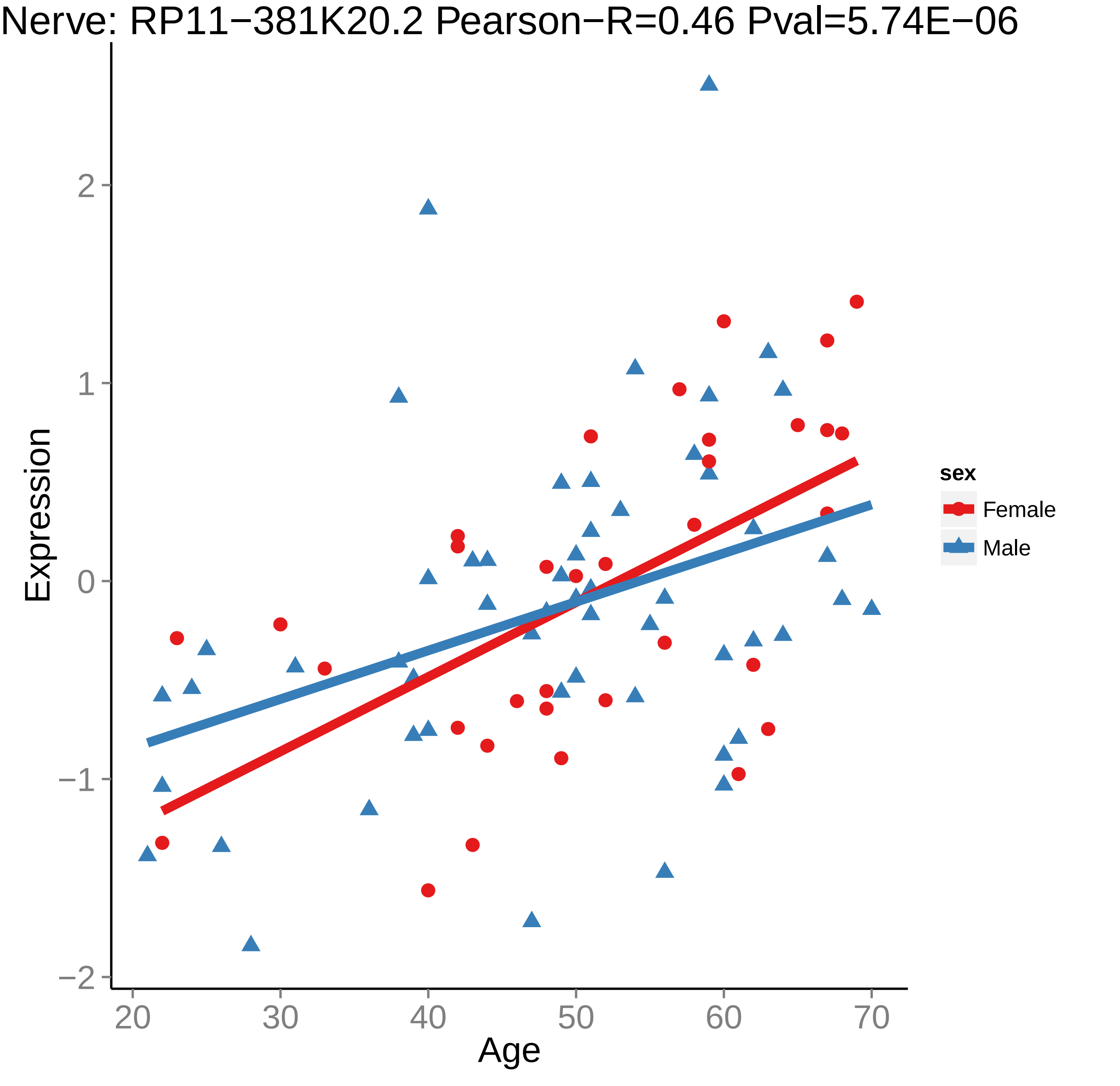

Nerve: RP11−381K20.2 Pearson−R=0.46 Pval=5.74E−06
Expression
2
1
0
−1
−2
20
30
40
50
60
70
Age
sex
Female
Male

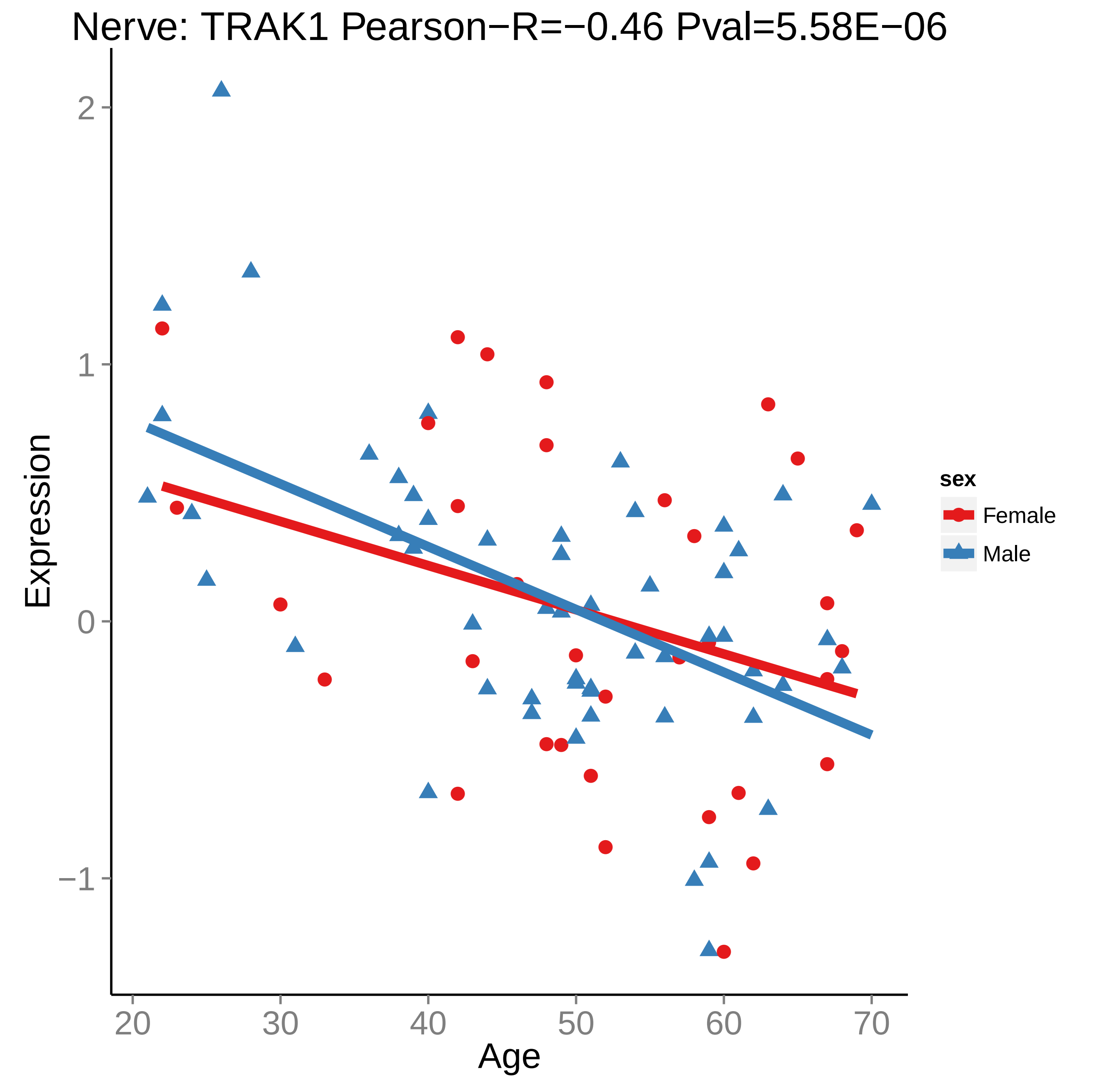
Nerve: TRAK1 Pearson−R=−0.46 Pval=5.58E−06
Expression
Age
2
1
0
−1
20
30
40
50
60
70
sex
Female
Male

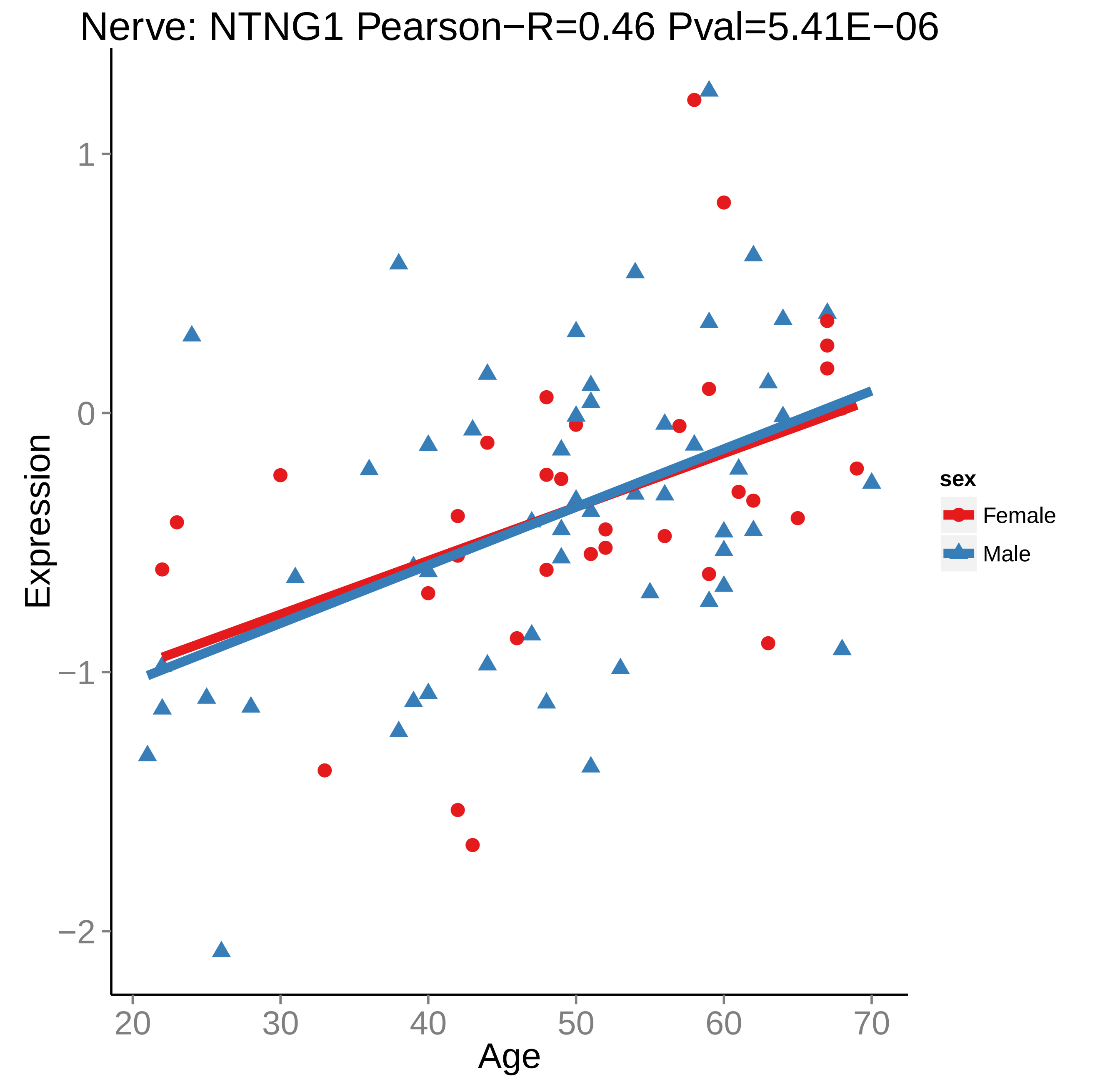

Nerve: NTNG1 Pearson−R=0.46 Pval=5.41E−06
Expression
Age
1
0
−1
−2
20
30
40
50
60
70
sex
Female
Male

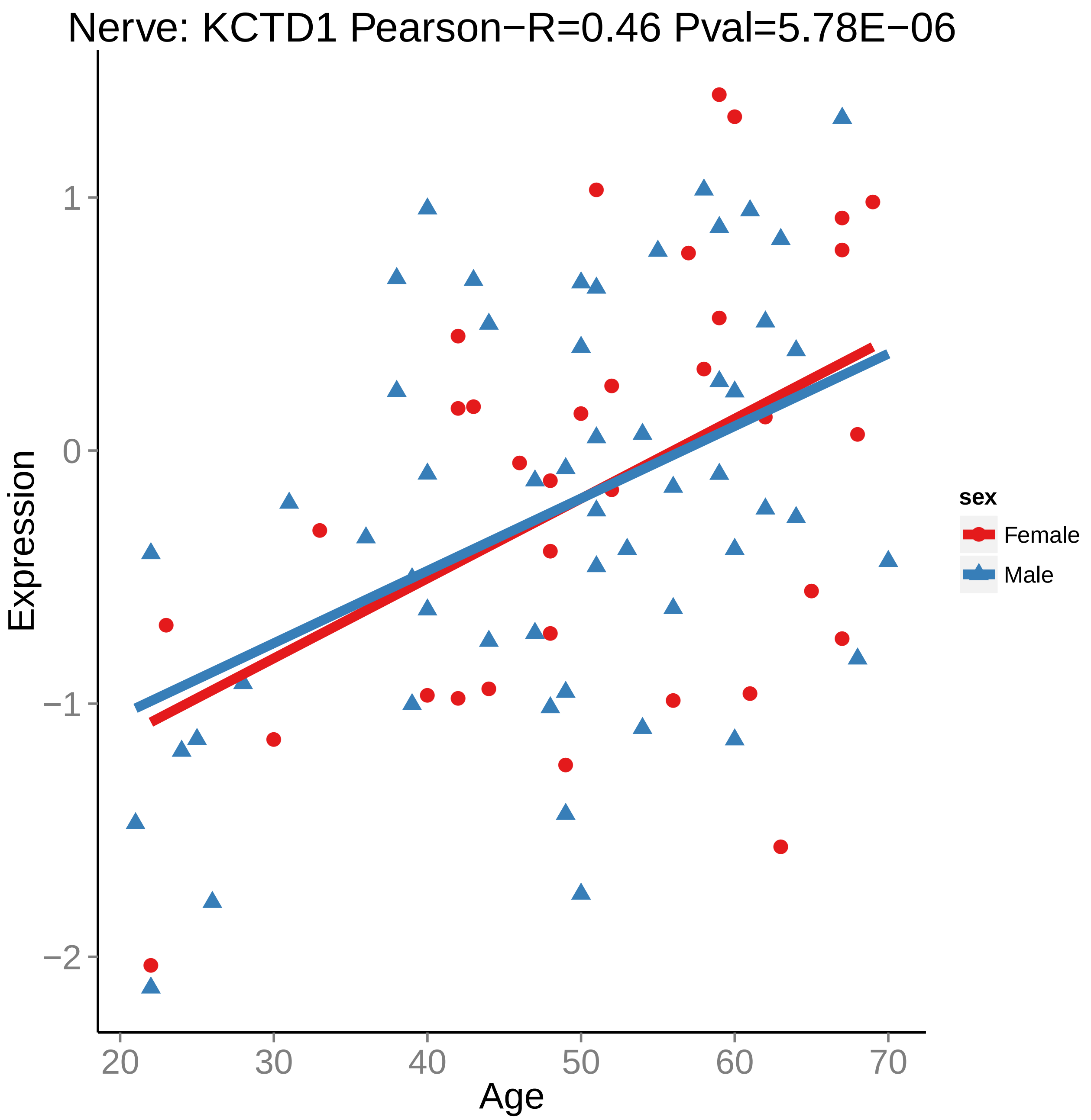

Nerve: KCTD1 Pearson−R=0.46 Pval=5.78E−06
Expression
1
0
−1
−2
20
30
40
50
60
70
Age
sex
Female
Male

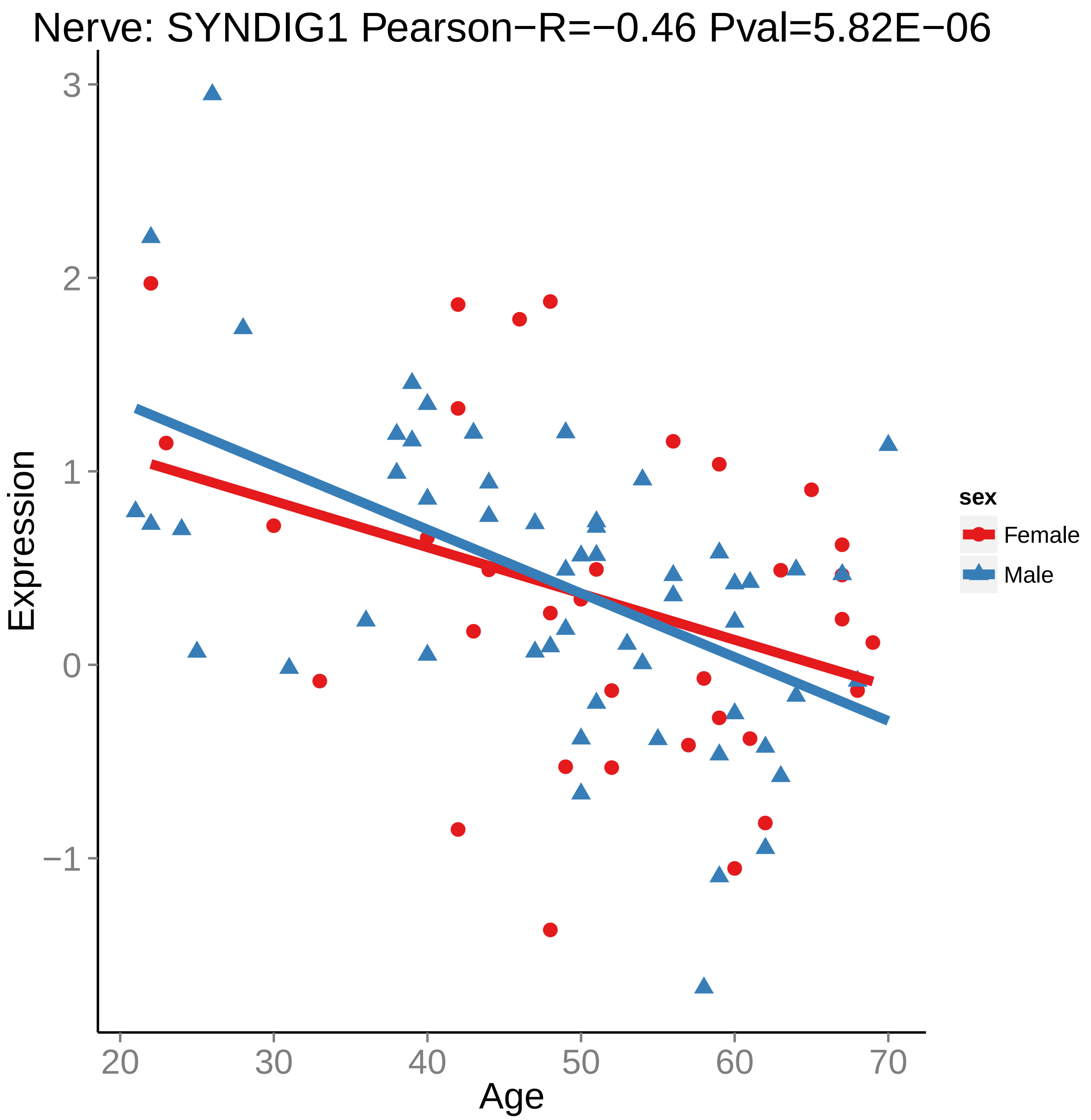

Nerve: SYNDIG1 Pearson−R=−0.46 Pval=5.82E−06
Expression
Age
3
2
1
0
−1
20
30
40
50
60
70
sex
Female
Male

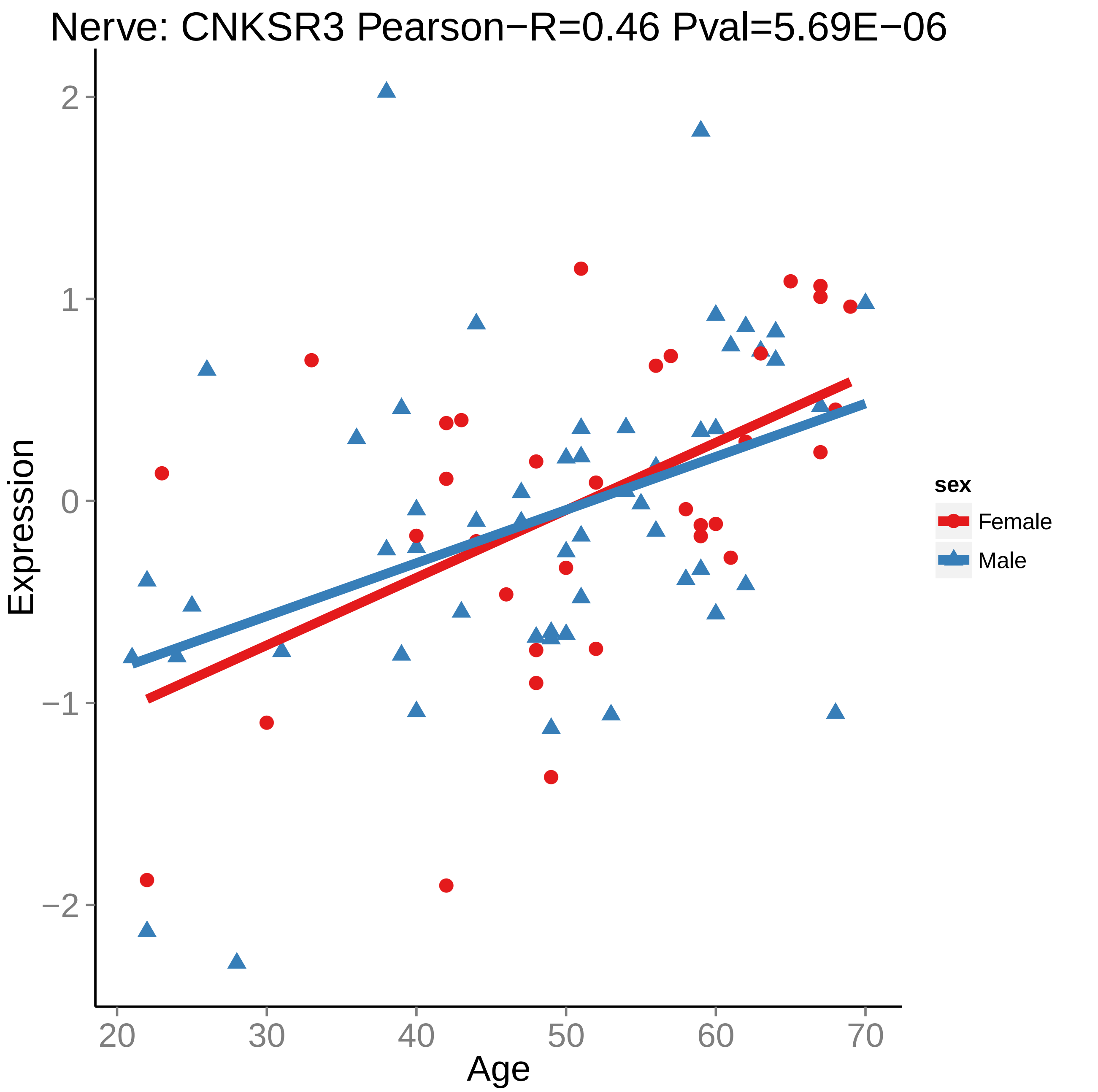

Nerve: CNKSR3 Pearson−R=0.46 Pval=5.69E−06
Expression
2
1
0
−1
−2
20
30
40
50
60
70
Age
sex
Female
Male

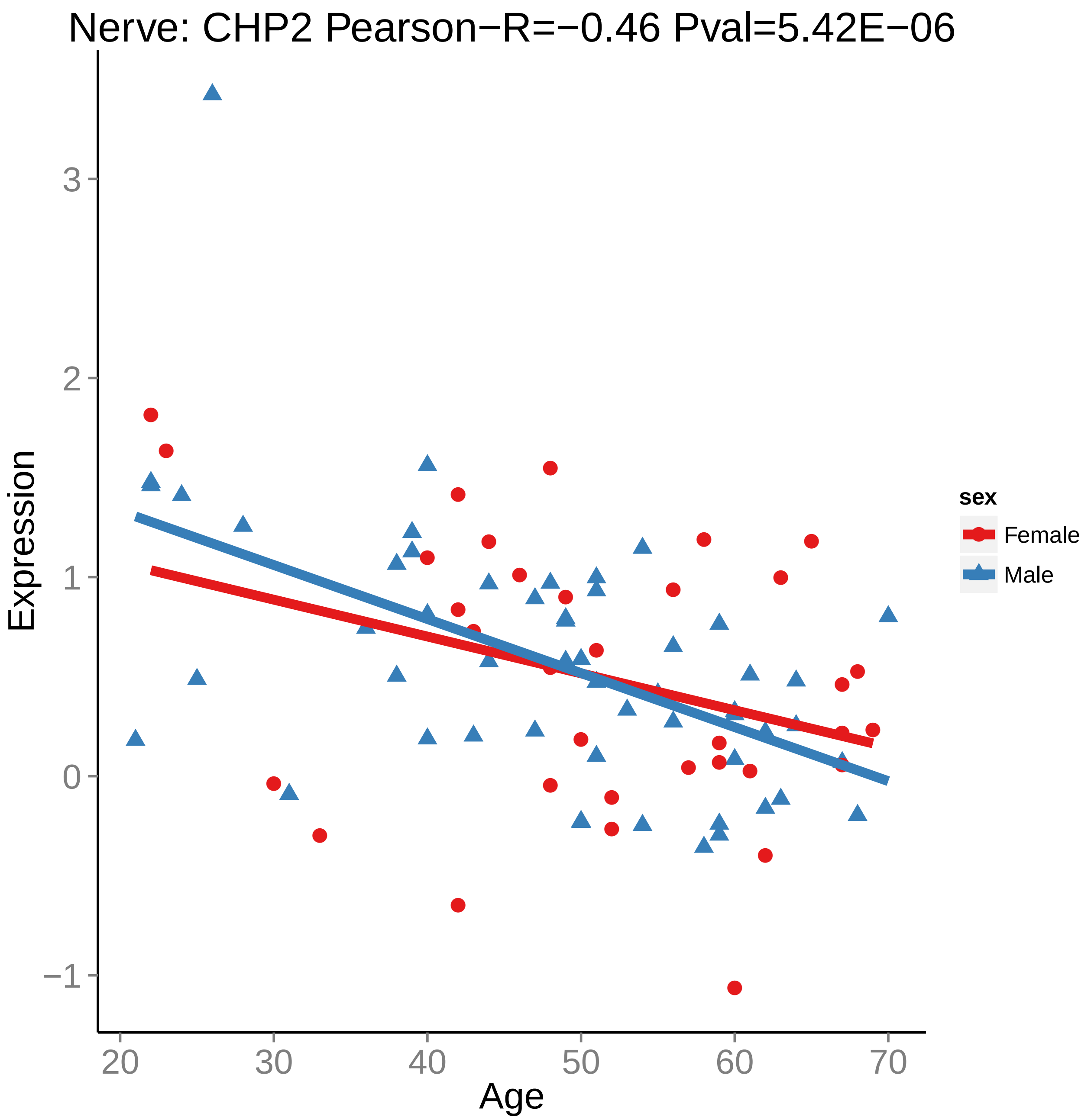
Nerve: CHP2 Pearson−R=−0.46 Pval=5.42E−06
Expression
3
2
1
0
−1
20
30
40
50
60
70
Age
sex
Female
Male

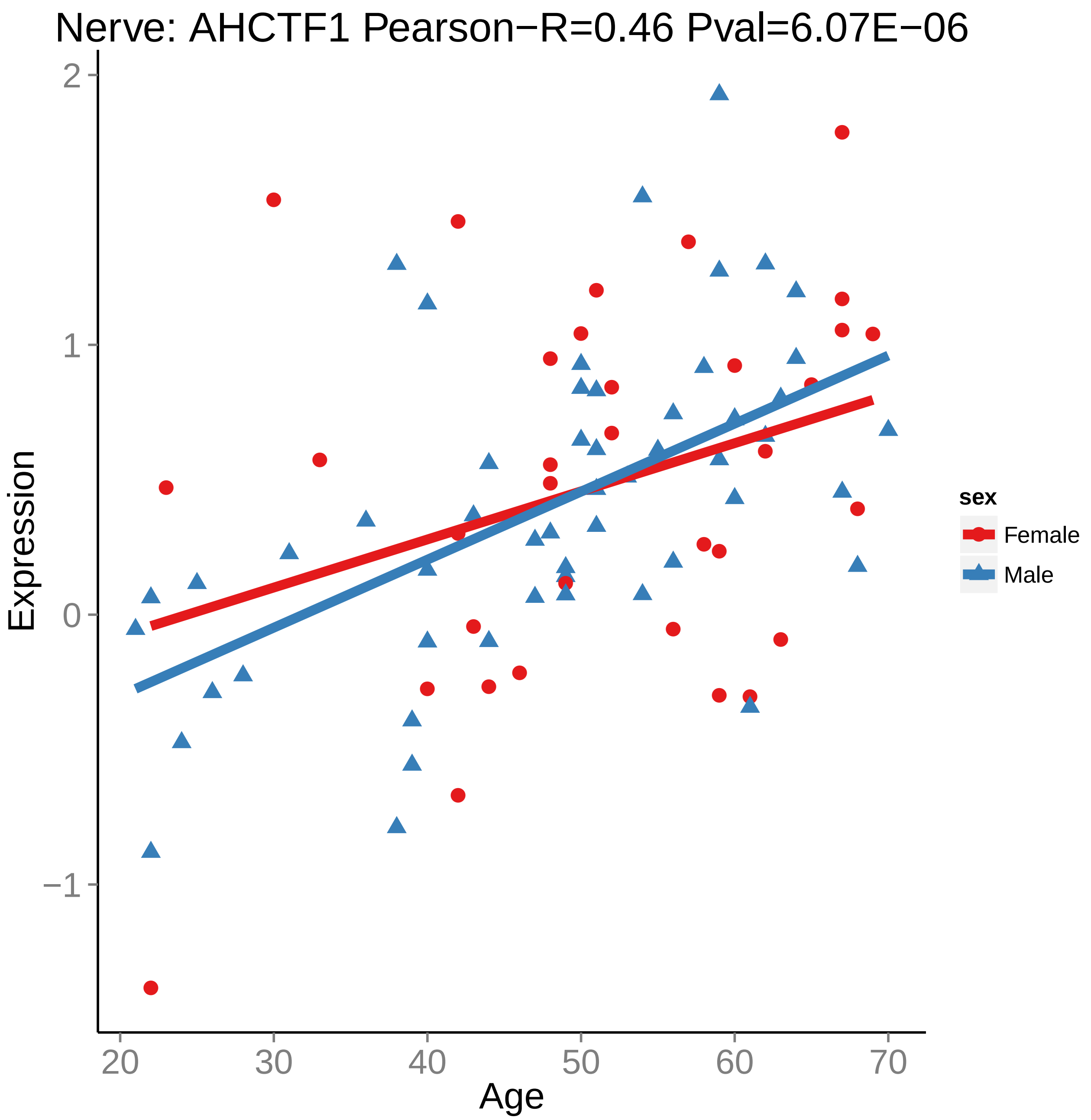

Nerve: AHCTF1 Pearson−R=0.46 Pval=6.07E−06
Expression
Age
2
1
0
−1
20
30
40
50
60
70
sex
Female
Male

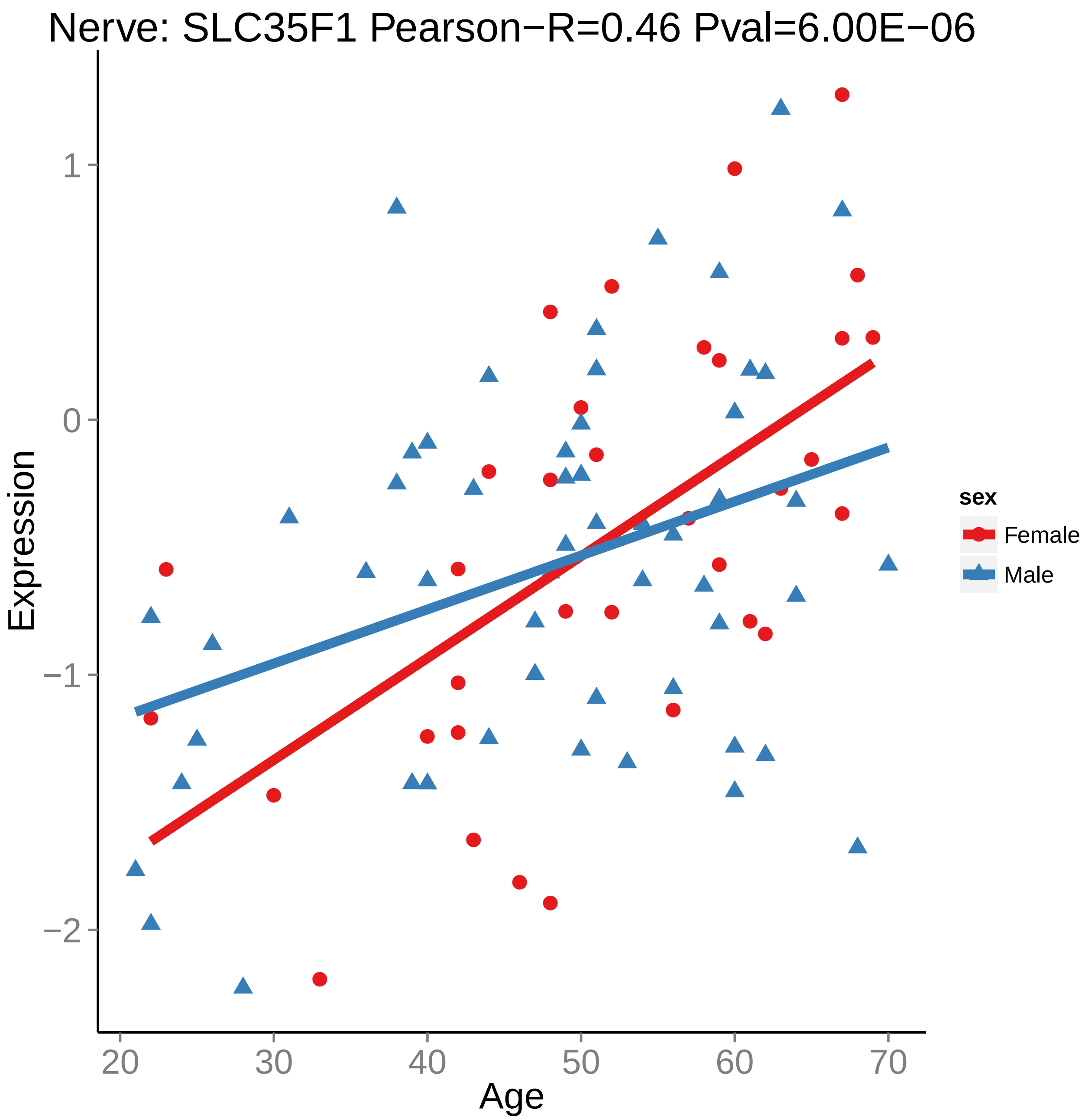

Nerve: SLC35F1 Pearson−R=0.46 Pval=6.00E−06
Expression
Age
1
0
−1
−2
20
30
40
50
60
70
sex
Female
Male

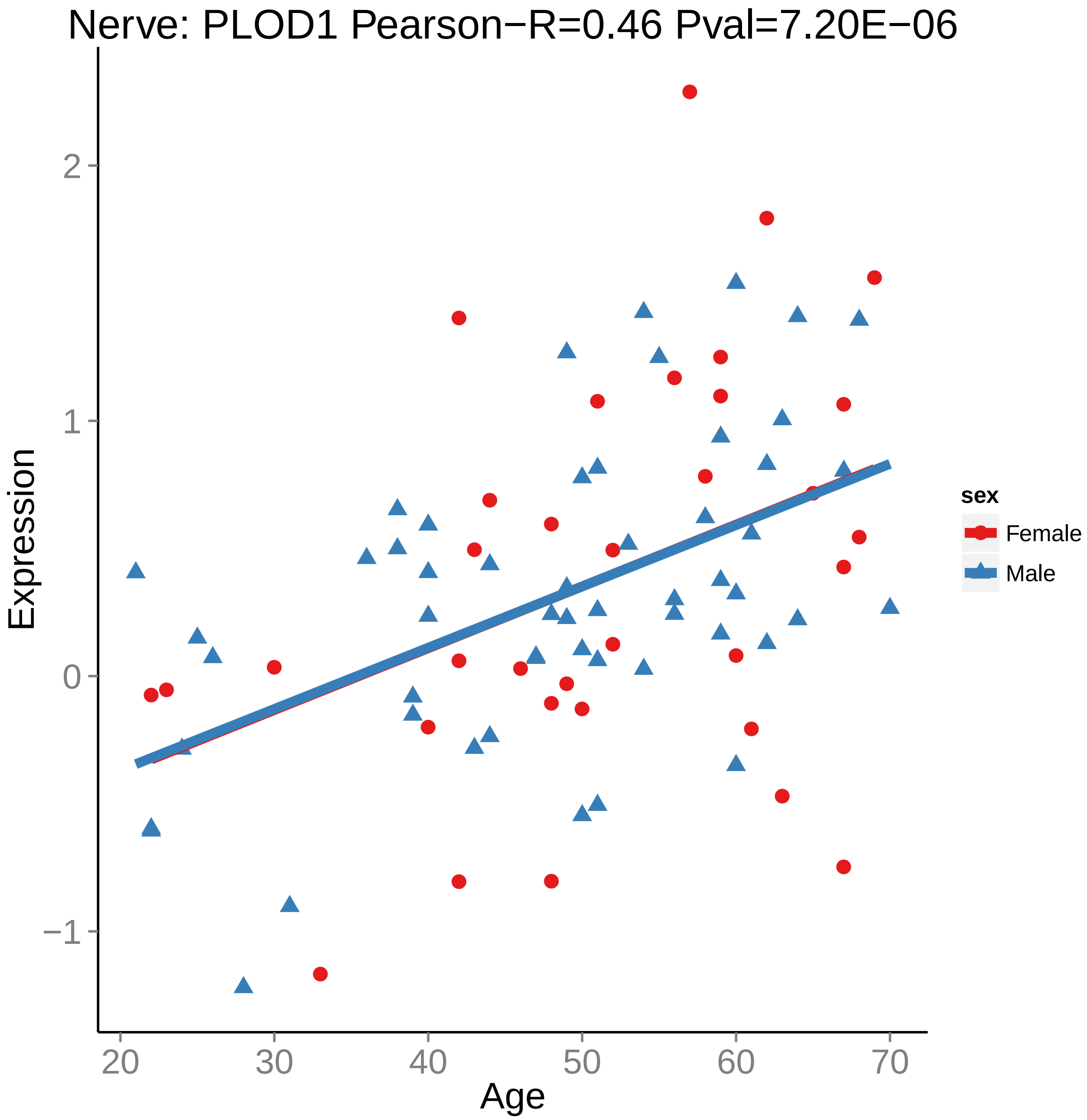
Nerve: PLOD1 Pearson−R=0.46 Pval=7.20E−06
Expression
2
1
0
−1
20
30
40
50
60
70
Age
sex
Female
Male

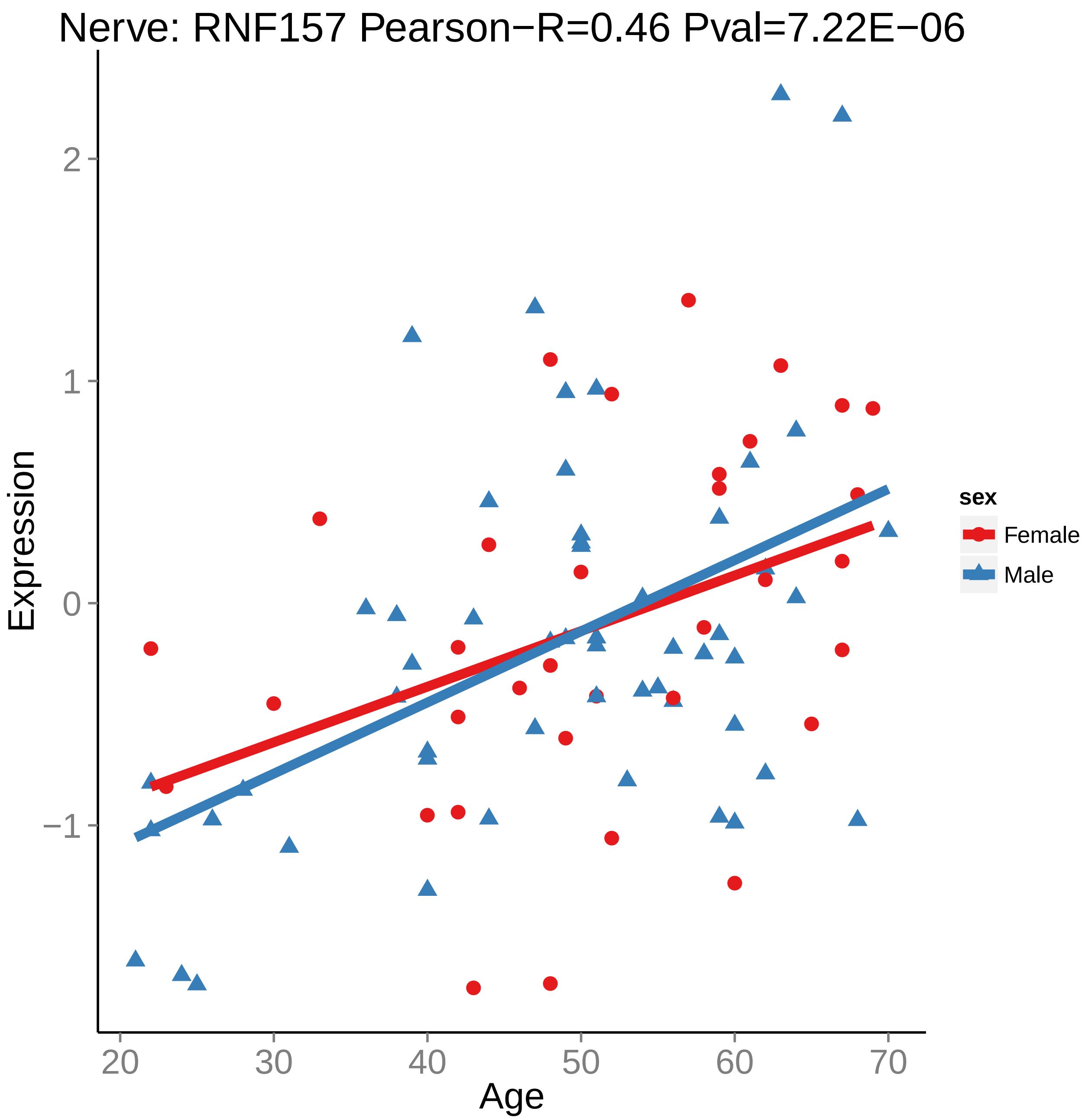

Nerve: RNF157 Pearson−R=0.46 Pval=7.22E−06
Expression
2
1
0
−1
20
30
40
50
60
70
Age
sex
Female
Male

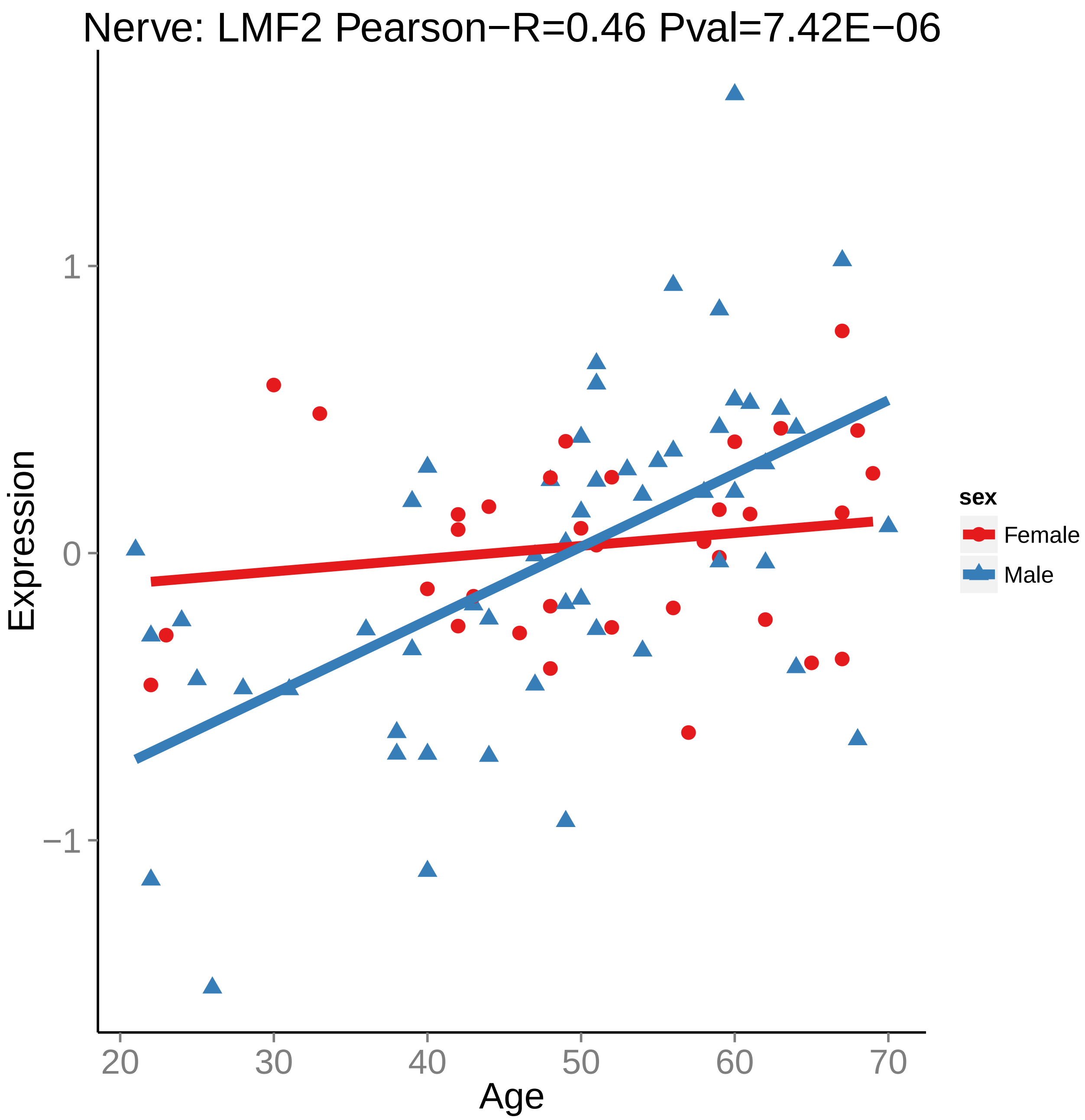

Nerve: LMF2 Pearson−R=0.46 Pval=7.42E−06
Expression
1
0
−1
20
30
40
50
60
70
Age
sex
Female
Male

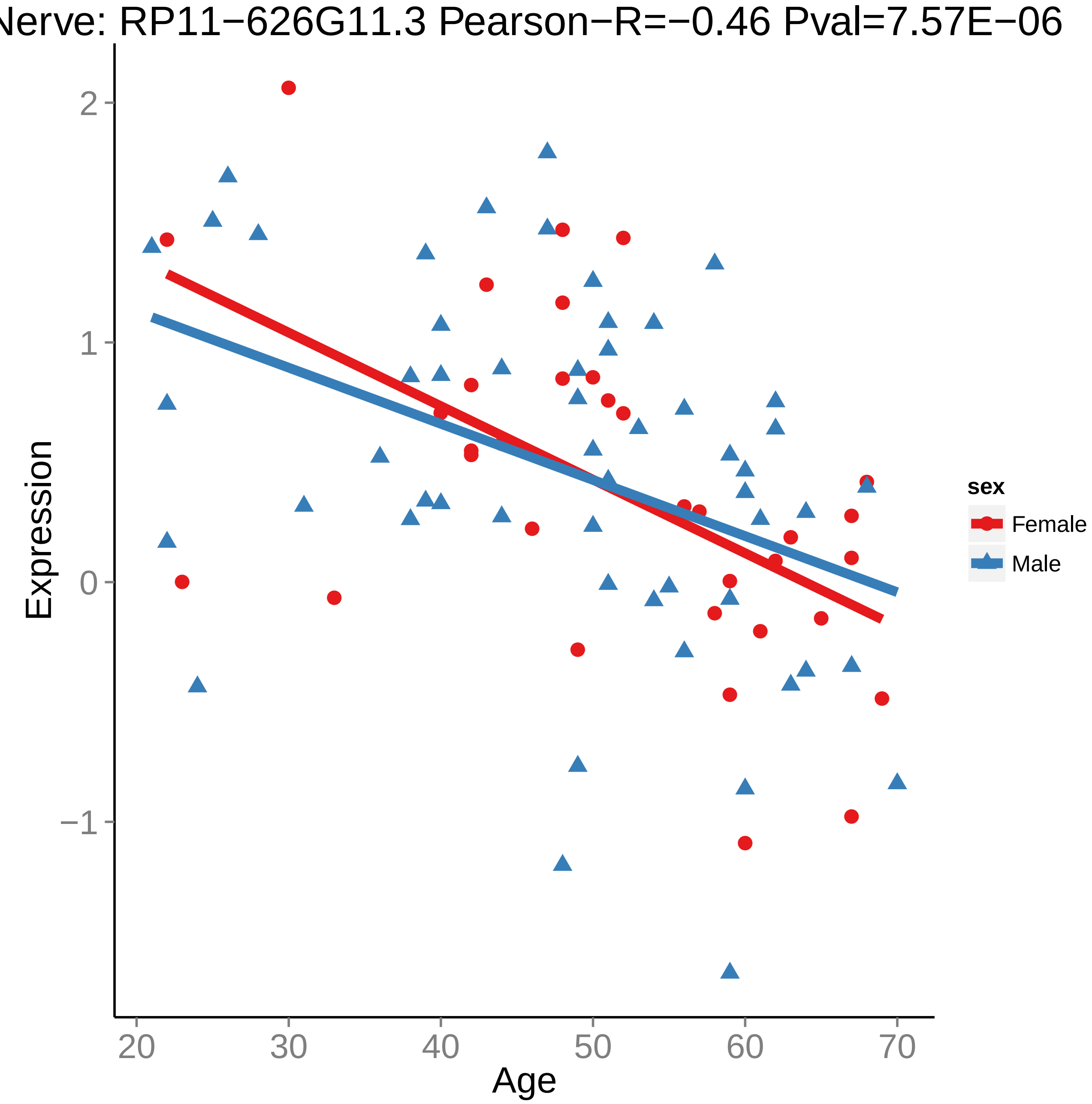
Nerve: RP11−626G11.3 Pearson−R=−0.46 Pval=7.57E−06
Expression
Age
2
1
0
−1
20
30
40
50
60
70
sex
Female
Male

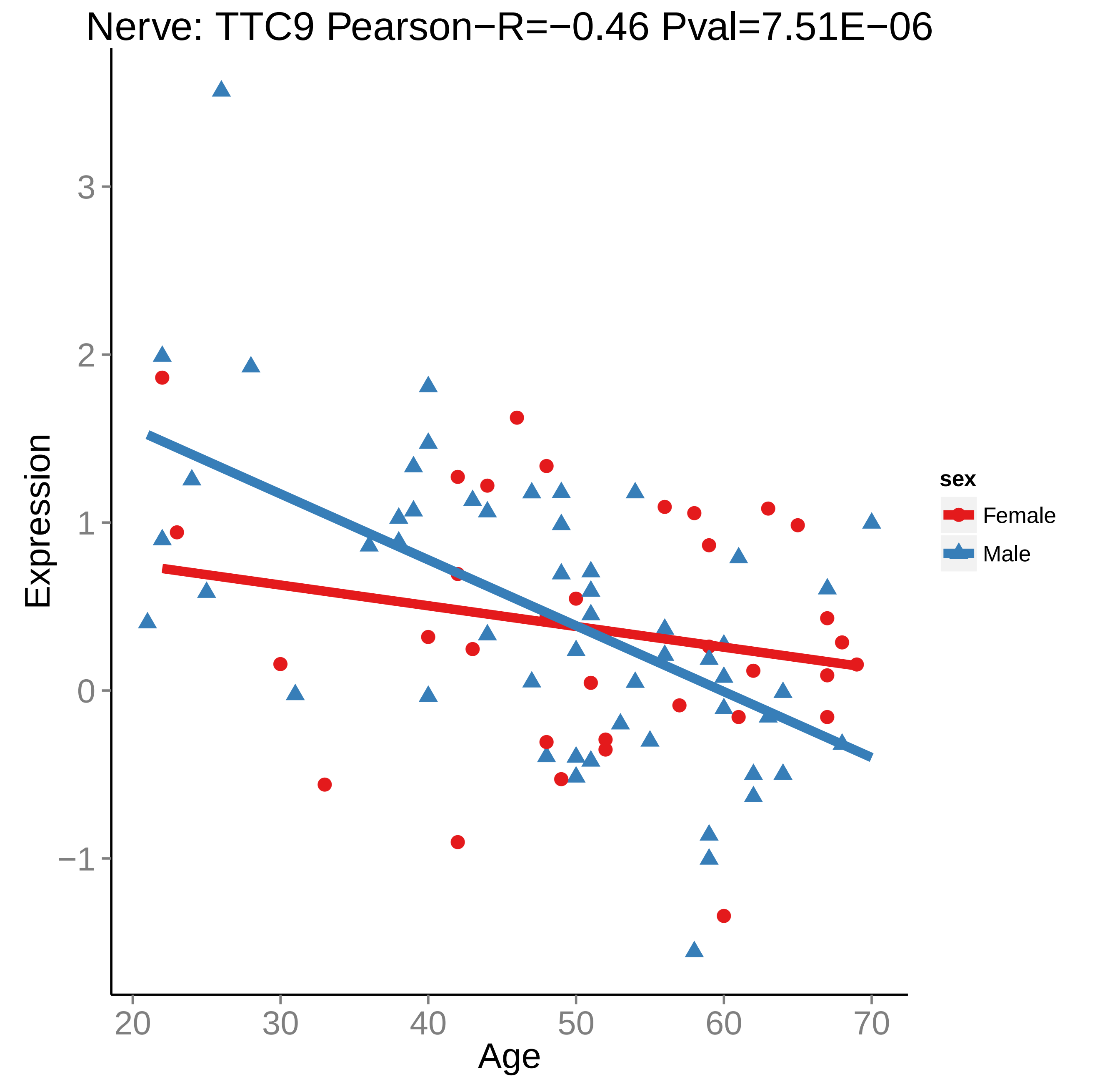

Nerve: TTC9 Pearson−R=−0.46 Pval=7.51E−06
Expression
Age
sex
Female
Male
3
2
1
0
−1
20
30
40
50
60
70

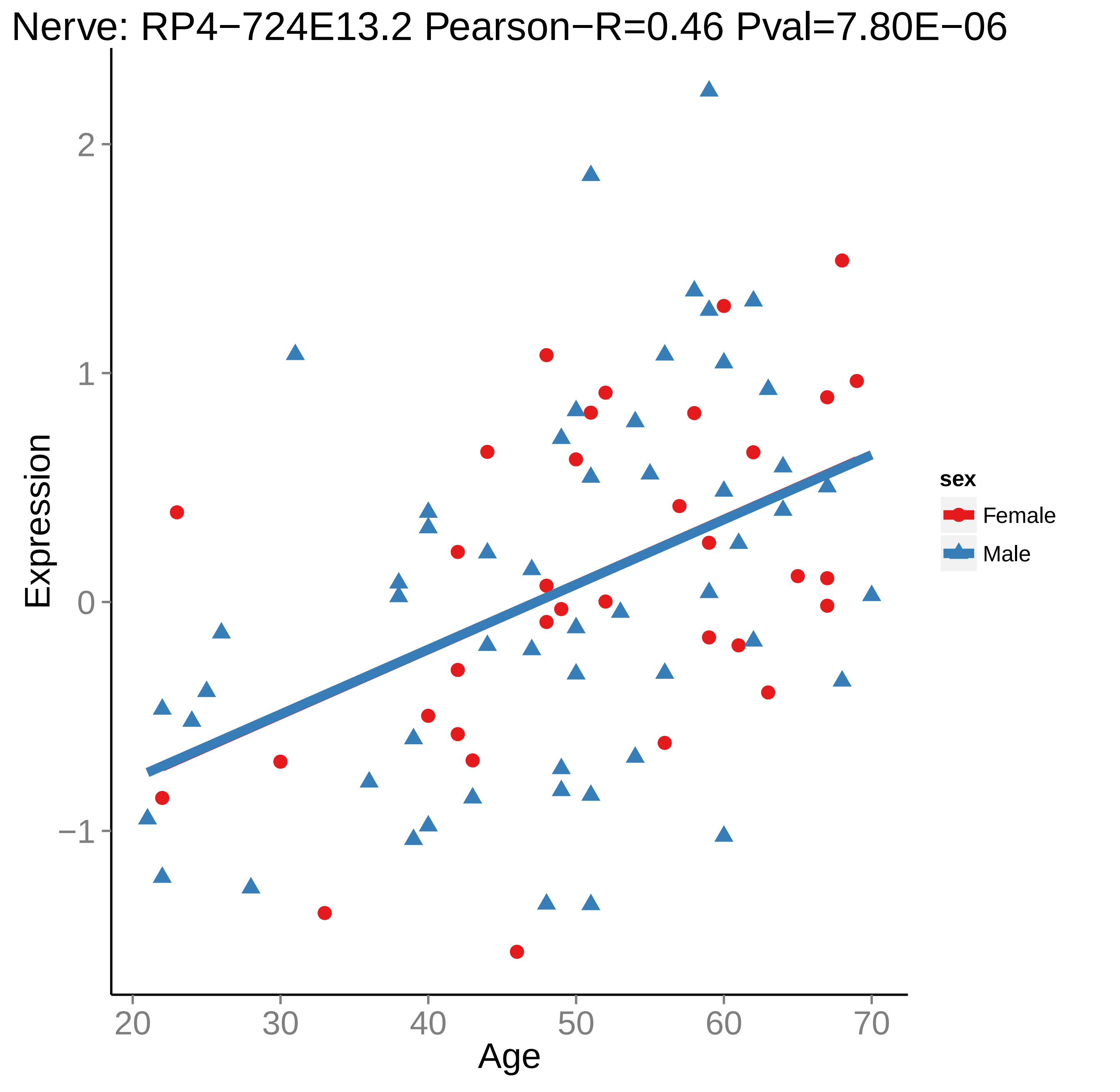

Nerve: RP4−724E13.2 Pearson−R=0.46 Pval=7.80E−06
Expression
Age
sex
Female
Male

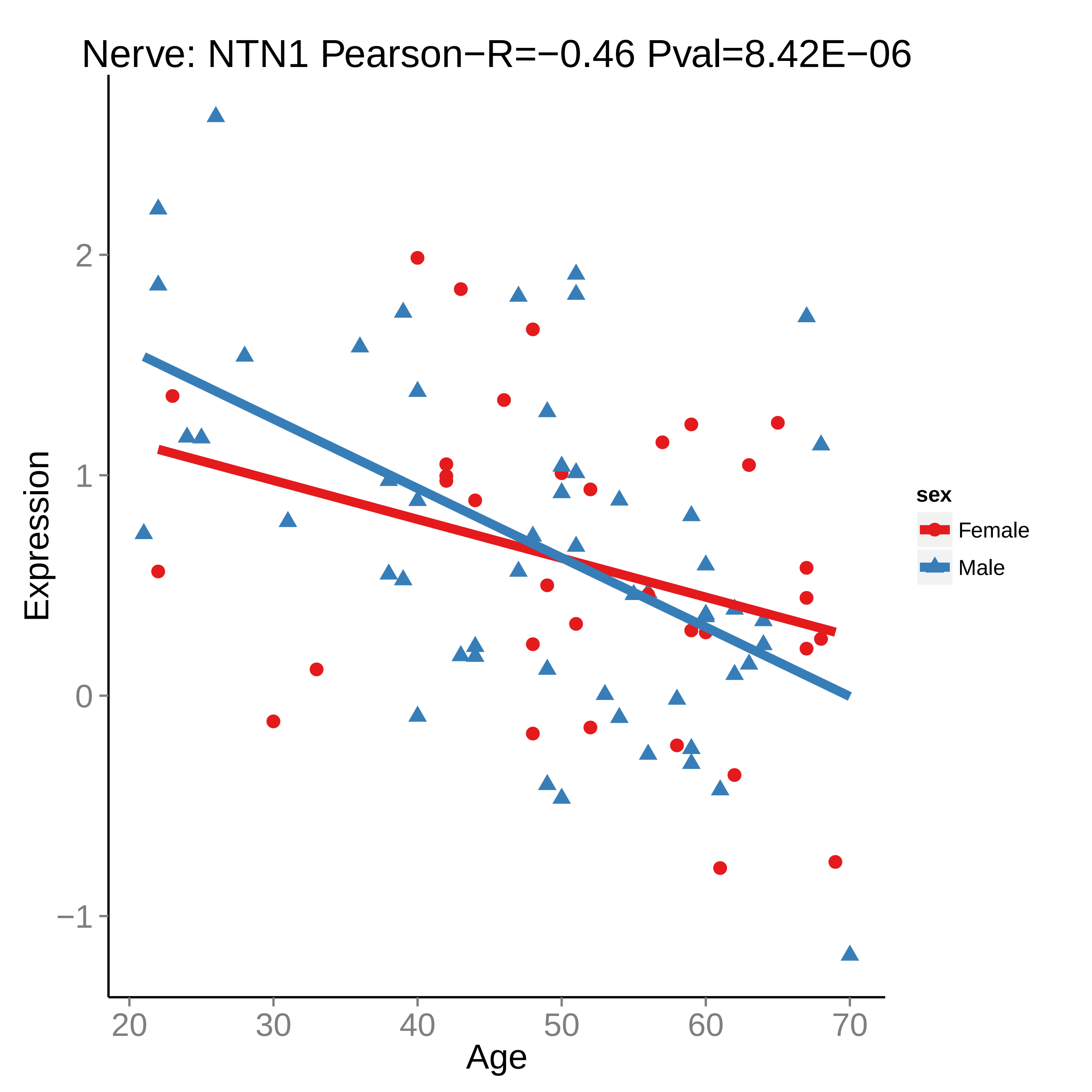

Nerve: NTN1 Pearson−R=−0.46 Pval=8.42E−06
Expression
2
1
0
−1
20
30
40
50
60
70
Age
sex
Female
Male

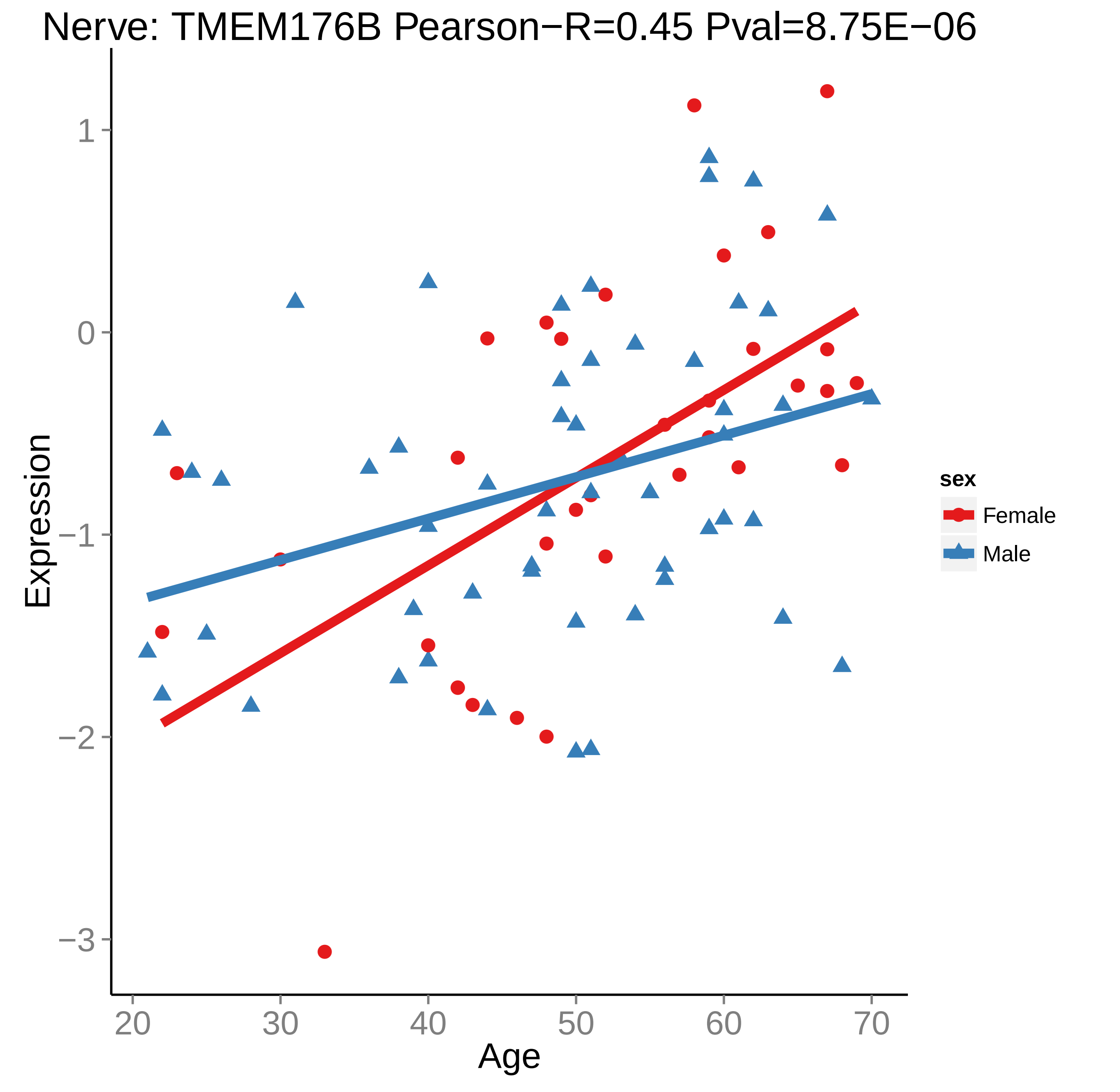

Nerve: TMEM176B Pearson−R=0.45 Pval=8.75E−06
Expression
1
0
−1
−2
−3
20
30
40
50
60
70
Age
sex
Female
Male

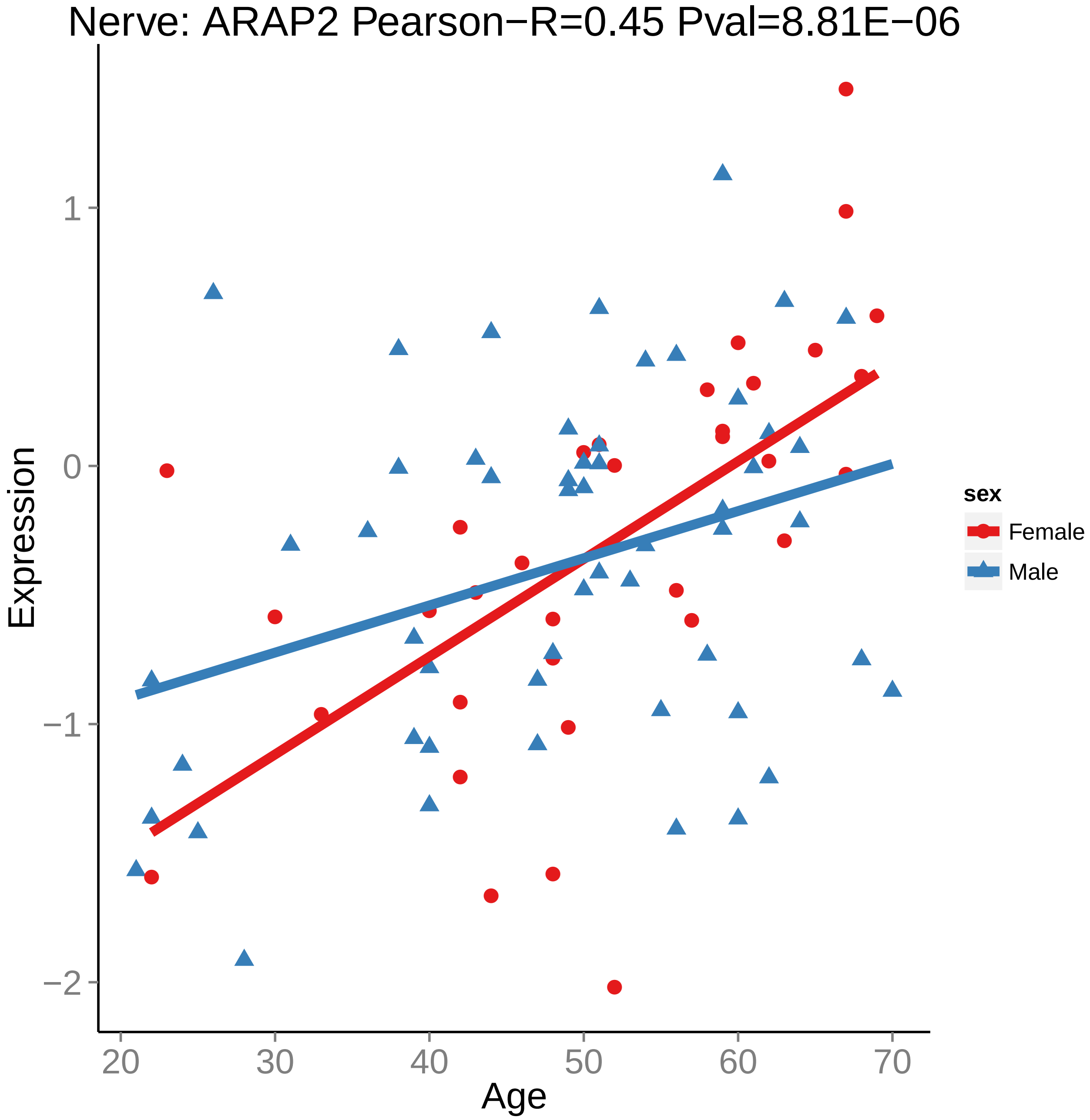

Nerve: ARAP2 Pearson−R=0.45 Pval=8.81E−06
Expression
Age
1
0
−1
−2
20
30
40
50
60
70
sex
Female
Male

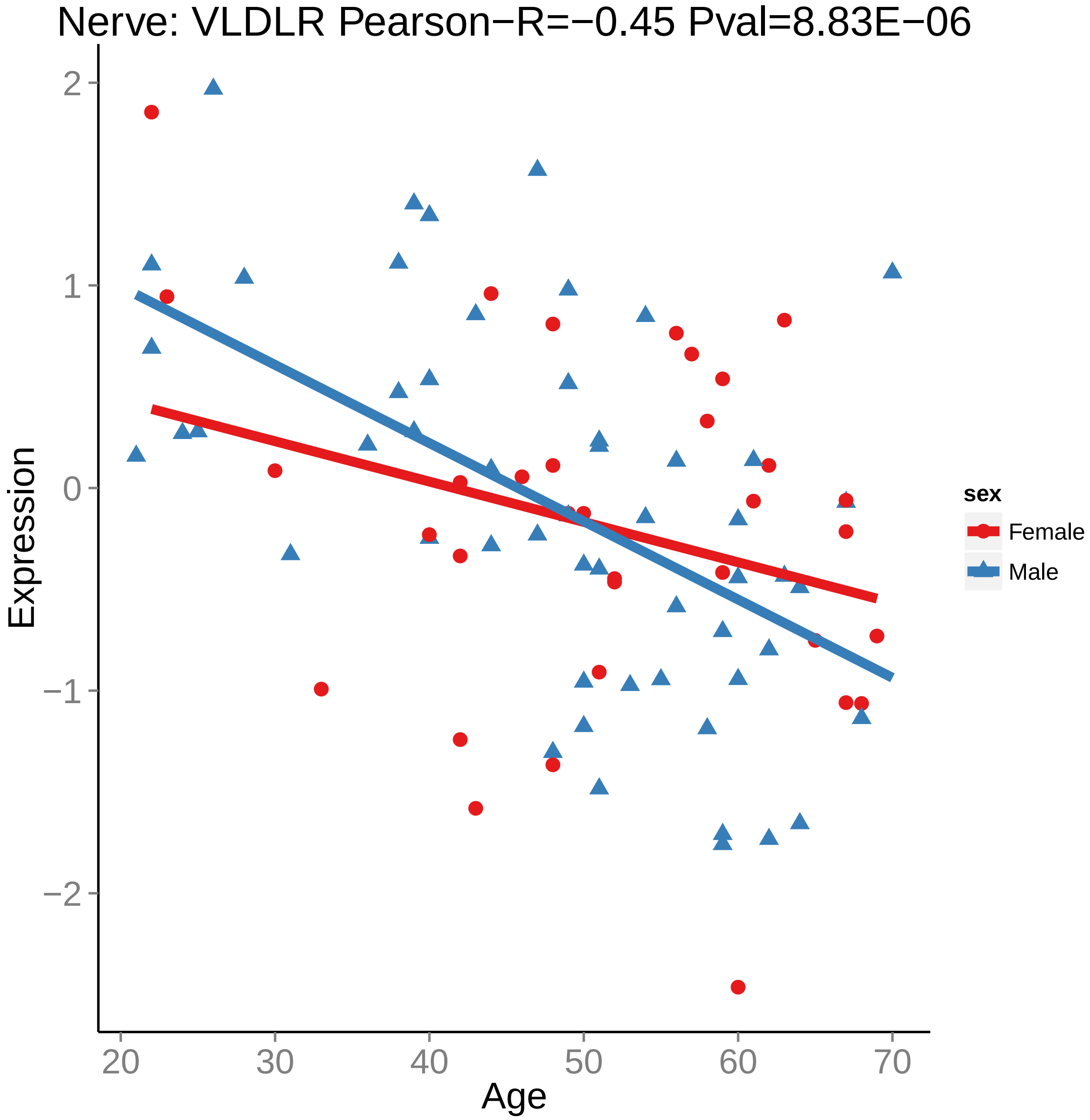

Nerve: VLDLR Pearson−R=−0.45 Pval=8.83E−06
Expression
Age
2
1
0
−1
−2
20
30
40
50
60
70
sex
Female
Male

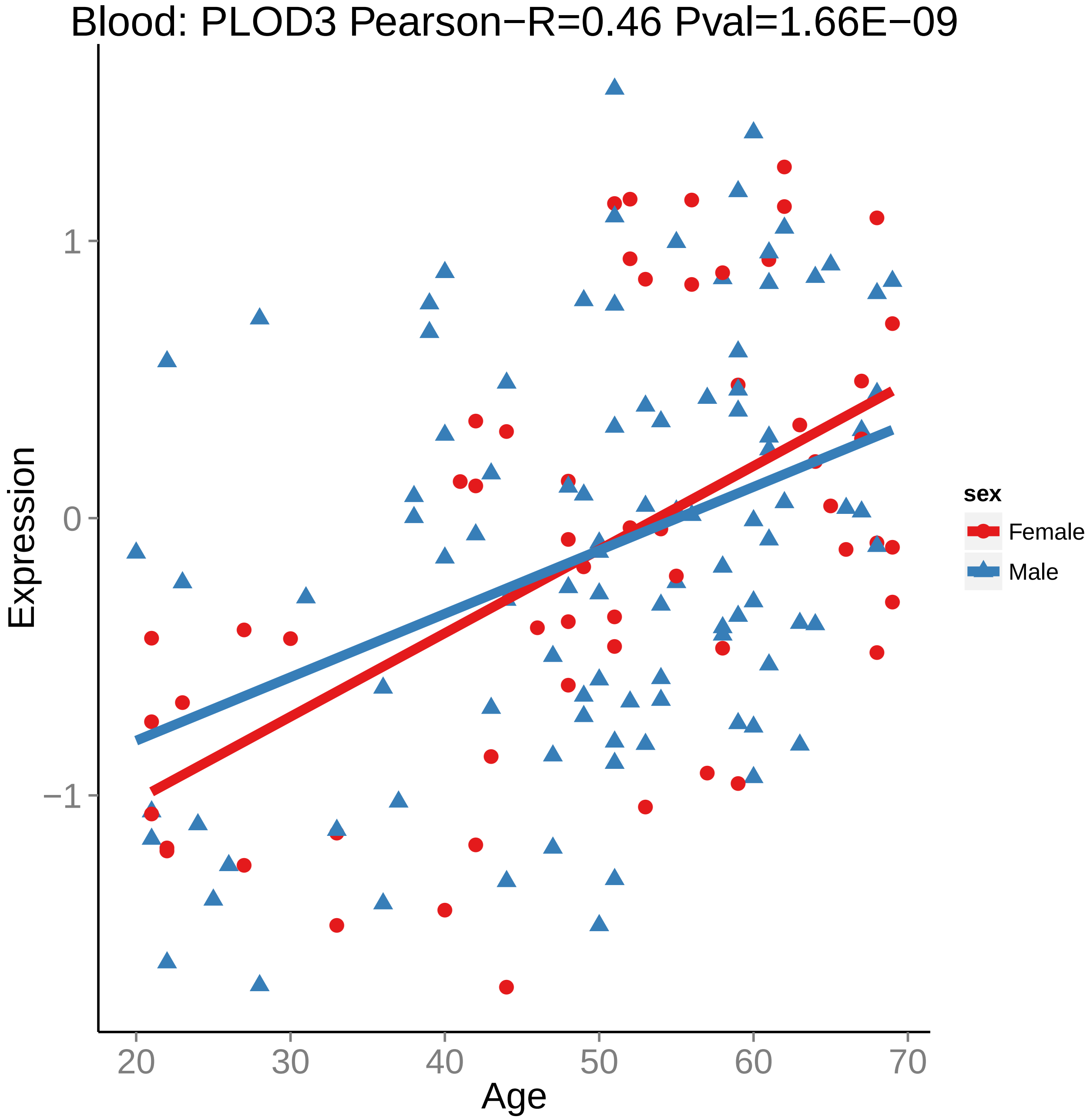

Blood: PLOD3 Pearson−R=0.46 Pval=1.66E−09
Expression
1
0
−1
20
30
40
50
60
70
Age
sex
Female
Male

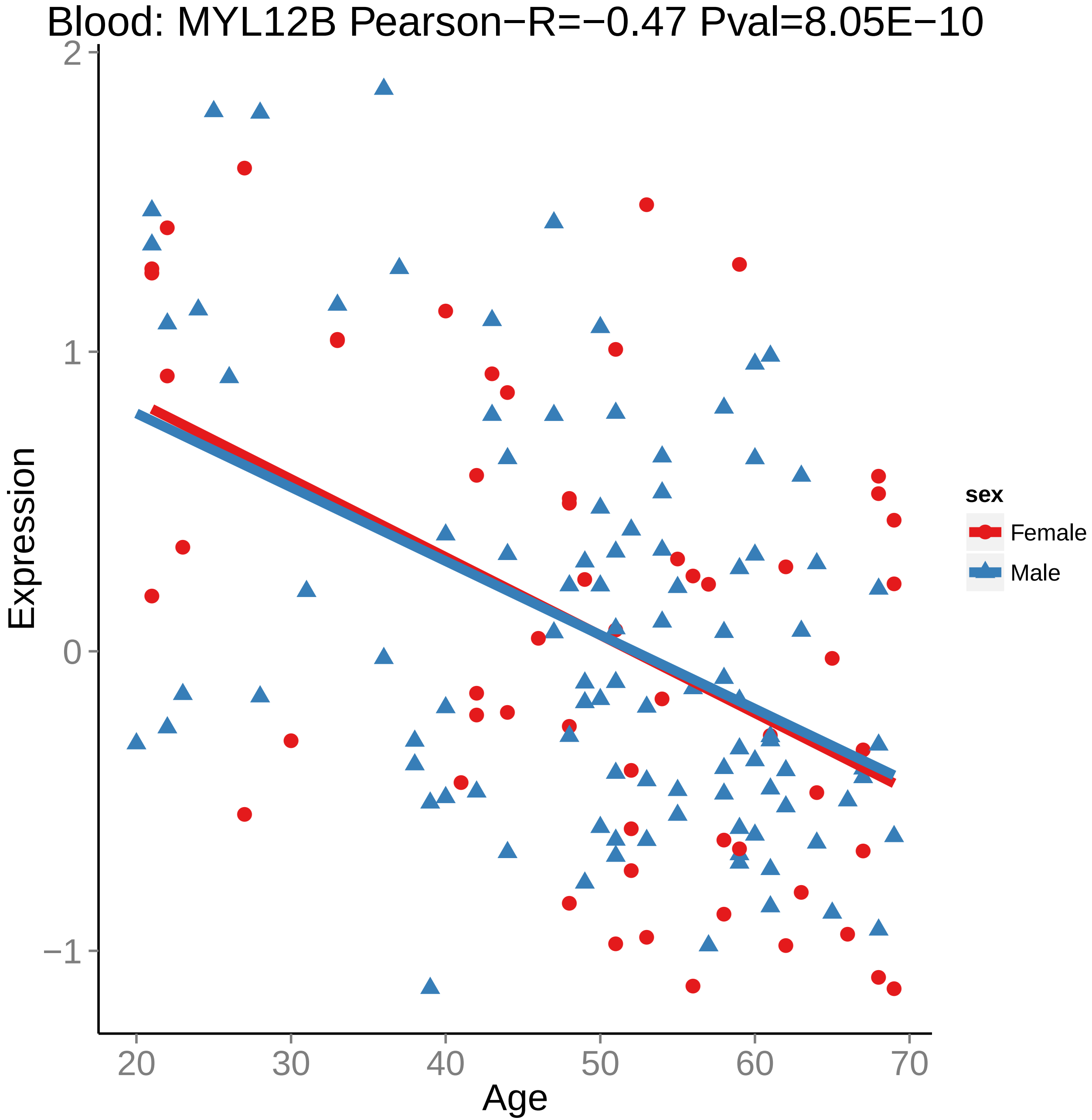

Blood: MYL12B Pearson−R=−0.47 Pval=8.05E−10
Expression
Age
2
1
0
−1
20
30
40
50
60
70
sex
Female
Male

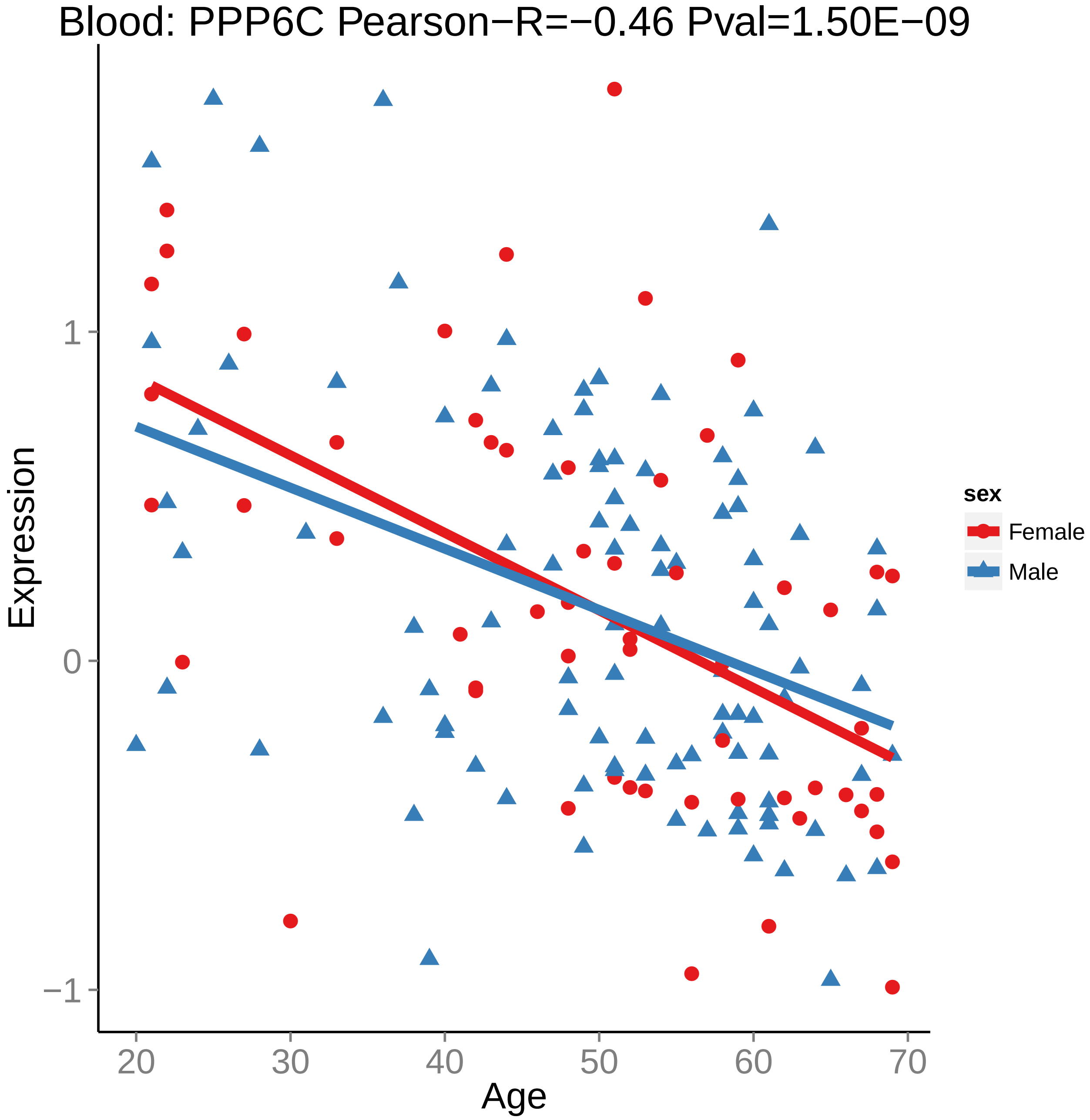

Blood: PPP6C Pearson−R=−0.46 Pval=1.50E−09
Expression
Age
1
0
−1
20
30
40
50
60
70
sex
Female
Male

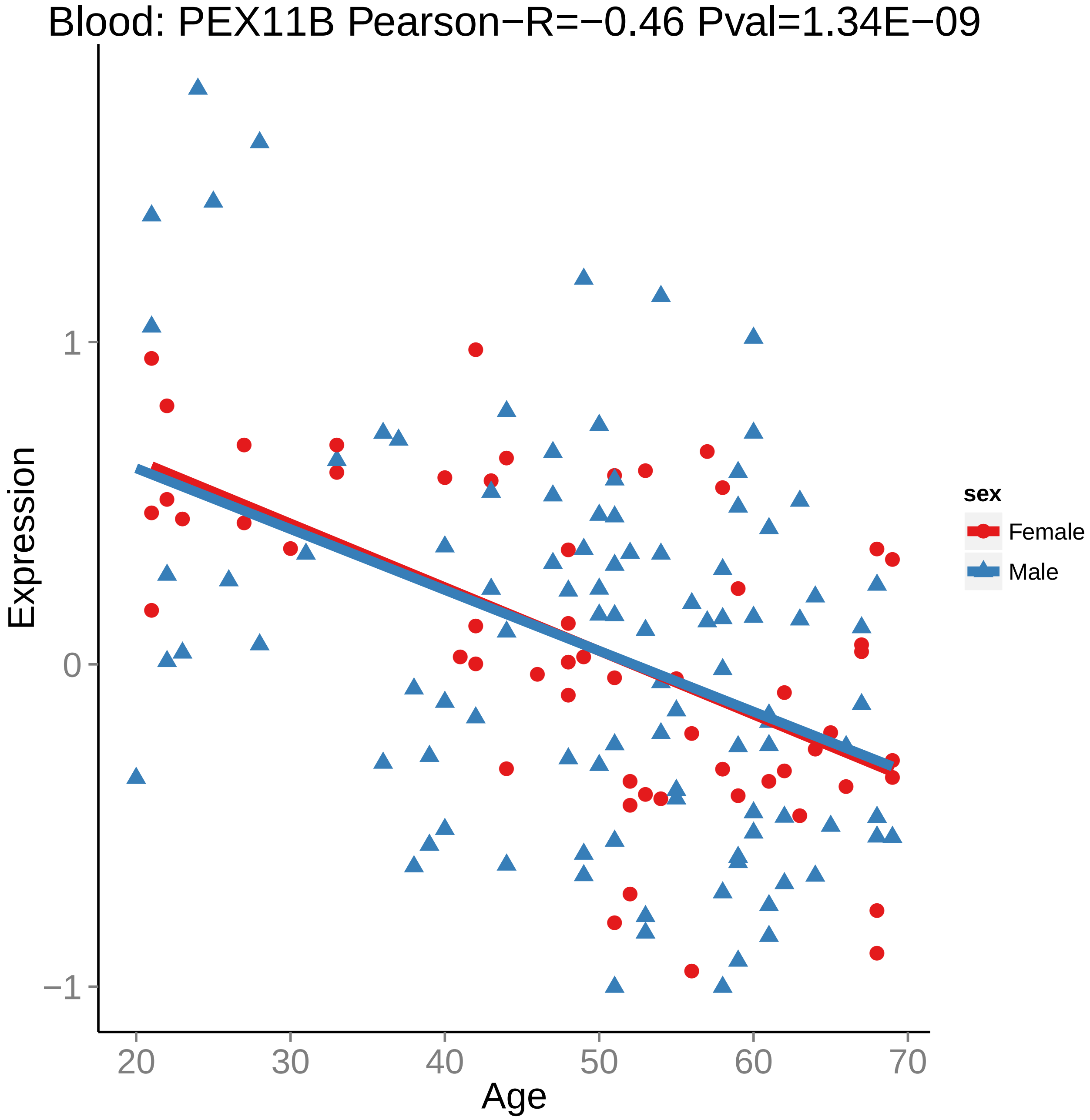

Blood: PEX11B Pearson−R=−0.46 Pval=1.34E−09
Expression
Age
1
0
−1
20
30
40
50
60
70
sex
Female
Male

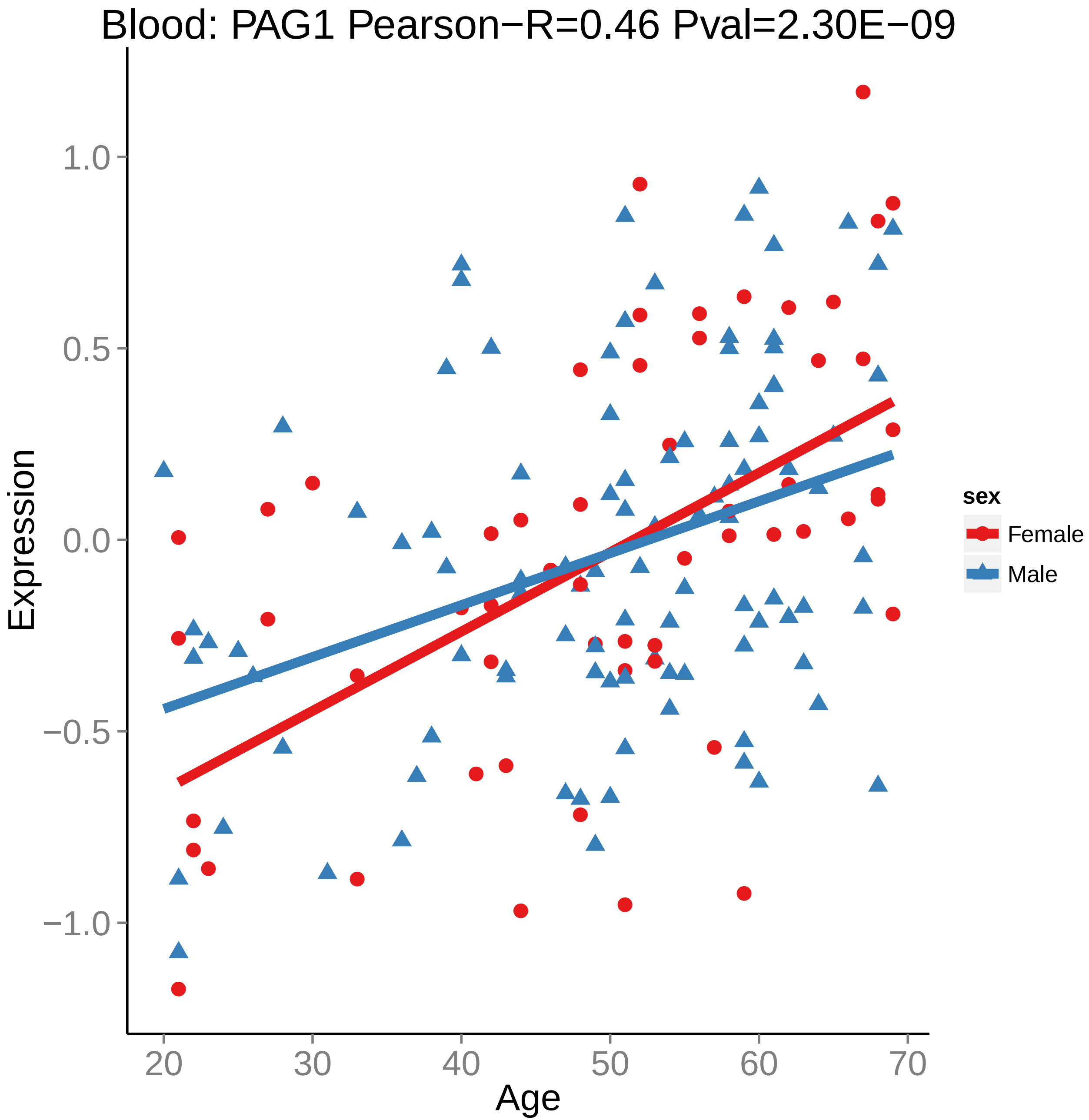

Blood: PAG1 Pearson−R=0.46 Pval=2.30E−09
Expression
1.0
0.5
0.0
−0.5
−1.0
20
30
40
50
60
70
Age
sex
Female
Male

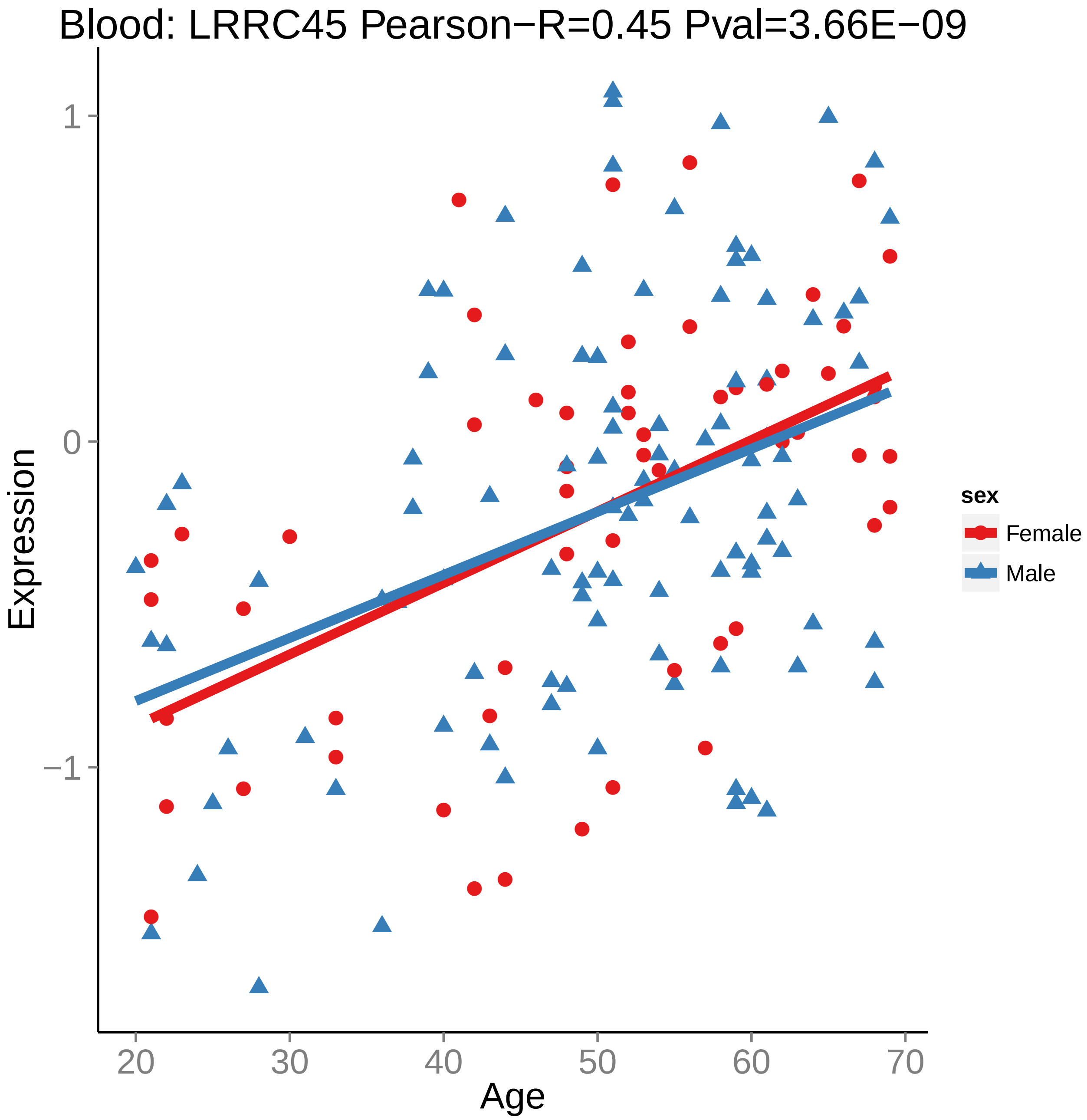

Blood: LRRC45 Pearson−R=0.45 Pval=3.66E−09
Expression
1
0
−1
20
30
40
50
60
70
Age
sex
Female
Male

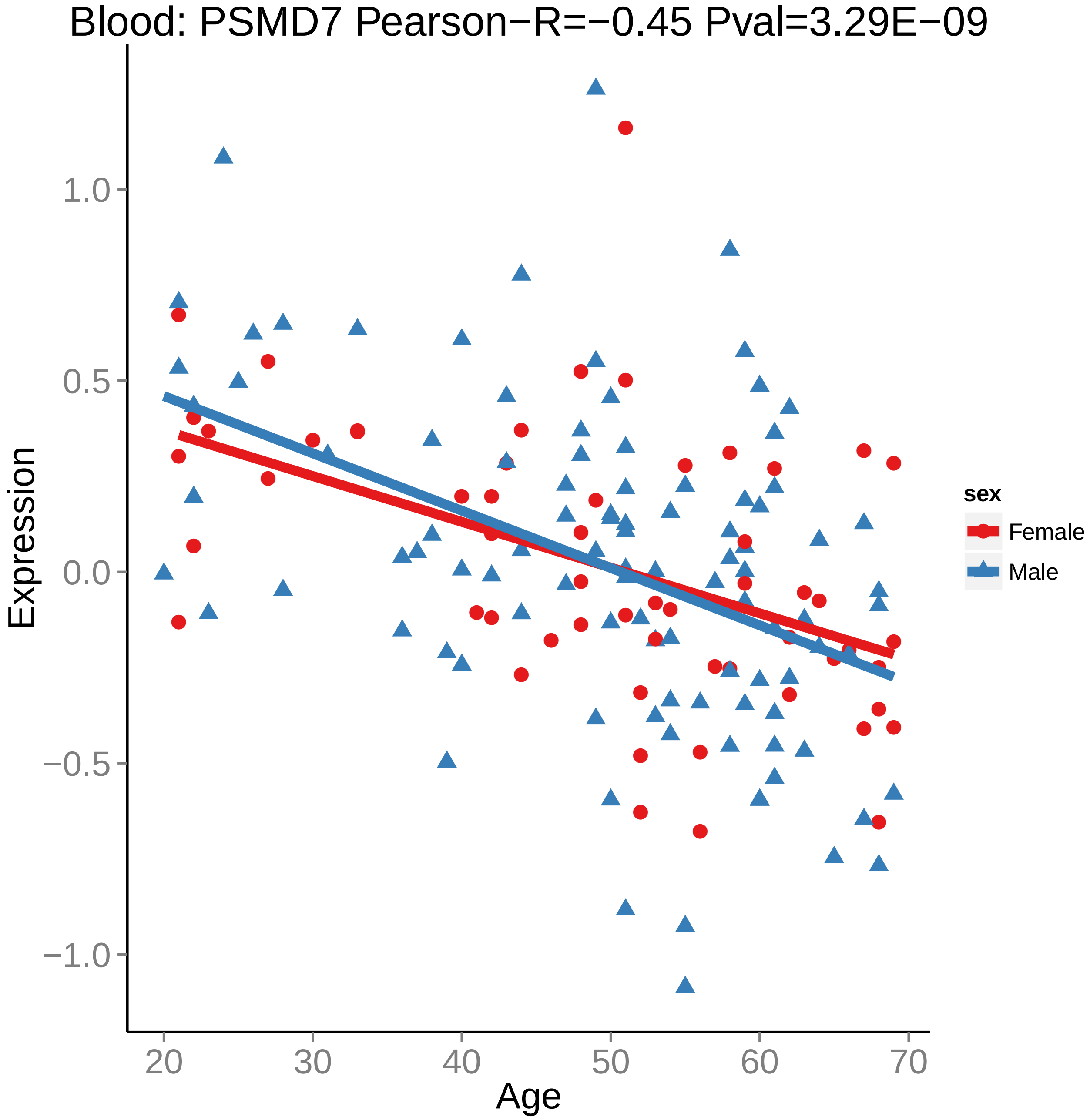

Blood: PSMD7 Pearson−R=−0.45 Pval=3.29E−09
Expression
Age
1.0
0.5
0.0
−0.5
−1.0
20
30
40
50
60
70
sex
Female
Male

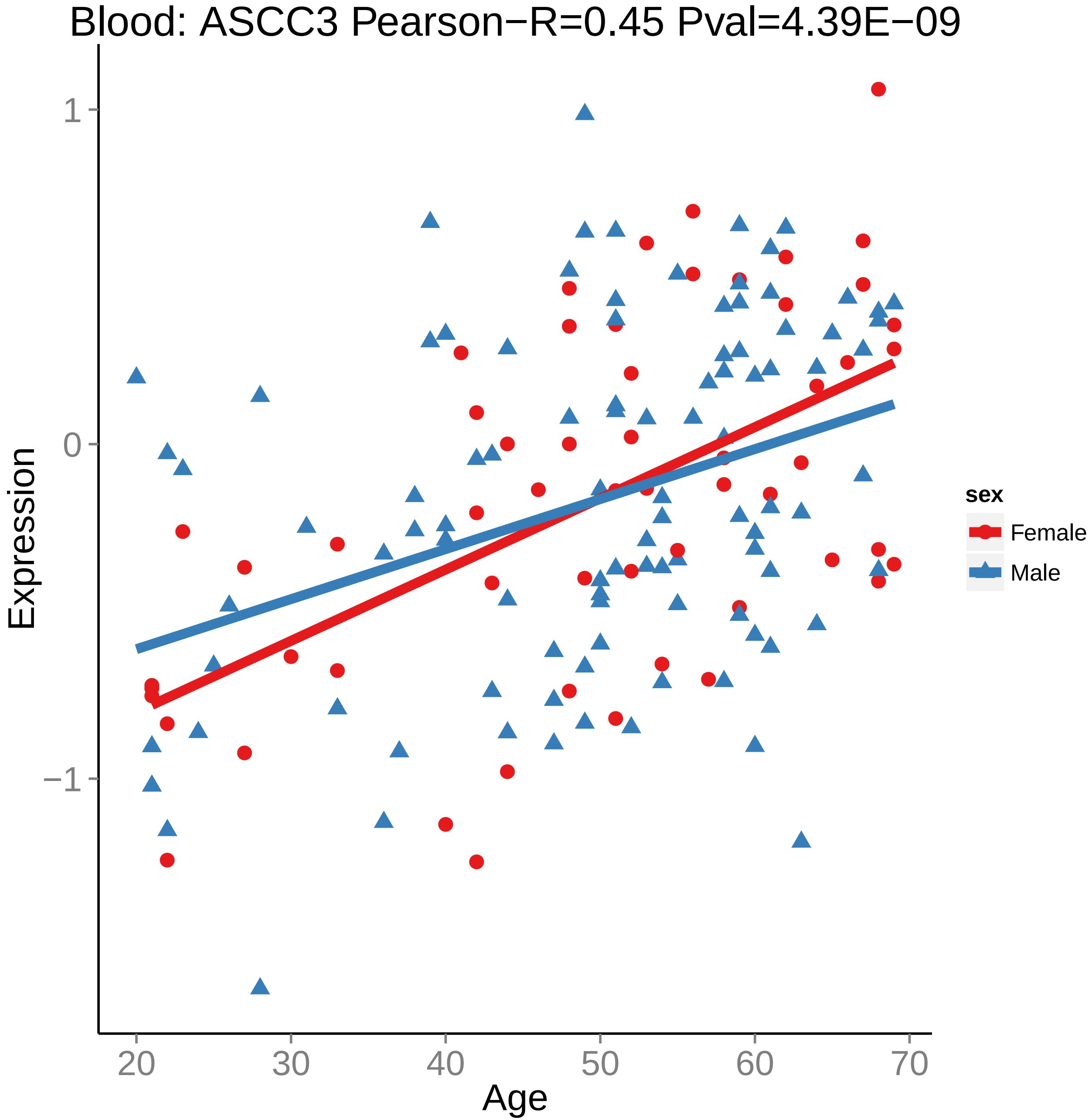

Blood: ASCC3 Pearson−R=0.45 Pval=4.39E−09
Expression
1
0
−1
20
30
40
50
60
70
Age
sex
Female
Male

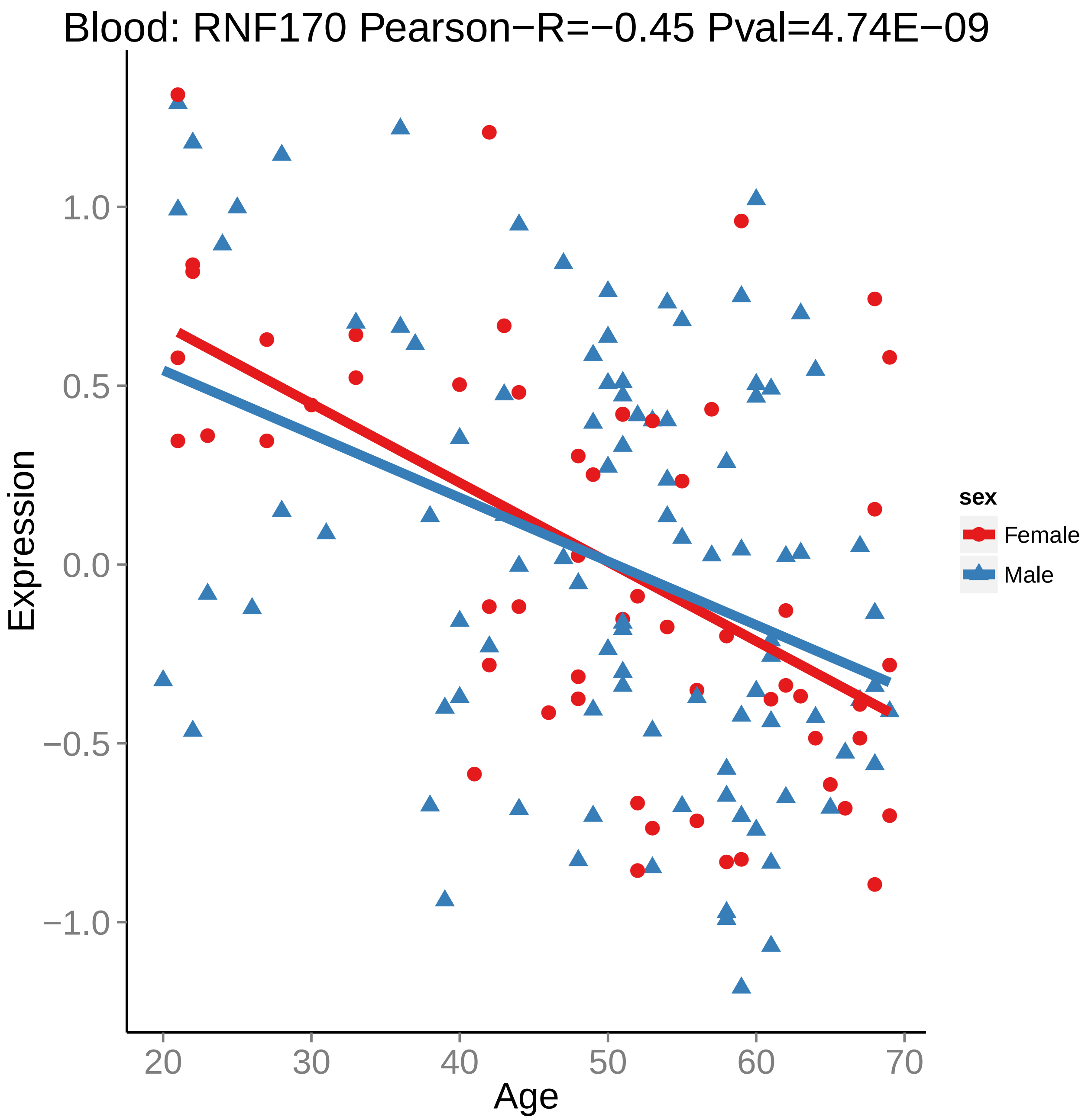

Blood: RNF170 Pearson−R=−0.45 Pval=4.74E−09
Expression
1.0
0.5
0.0
−0.5
−1.0
20
30
40
50
60
70
Age
sex
Female
Male

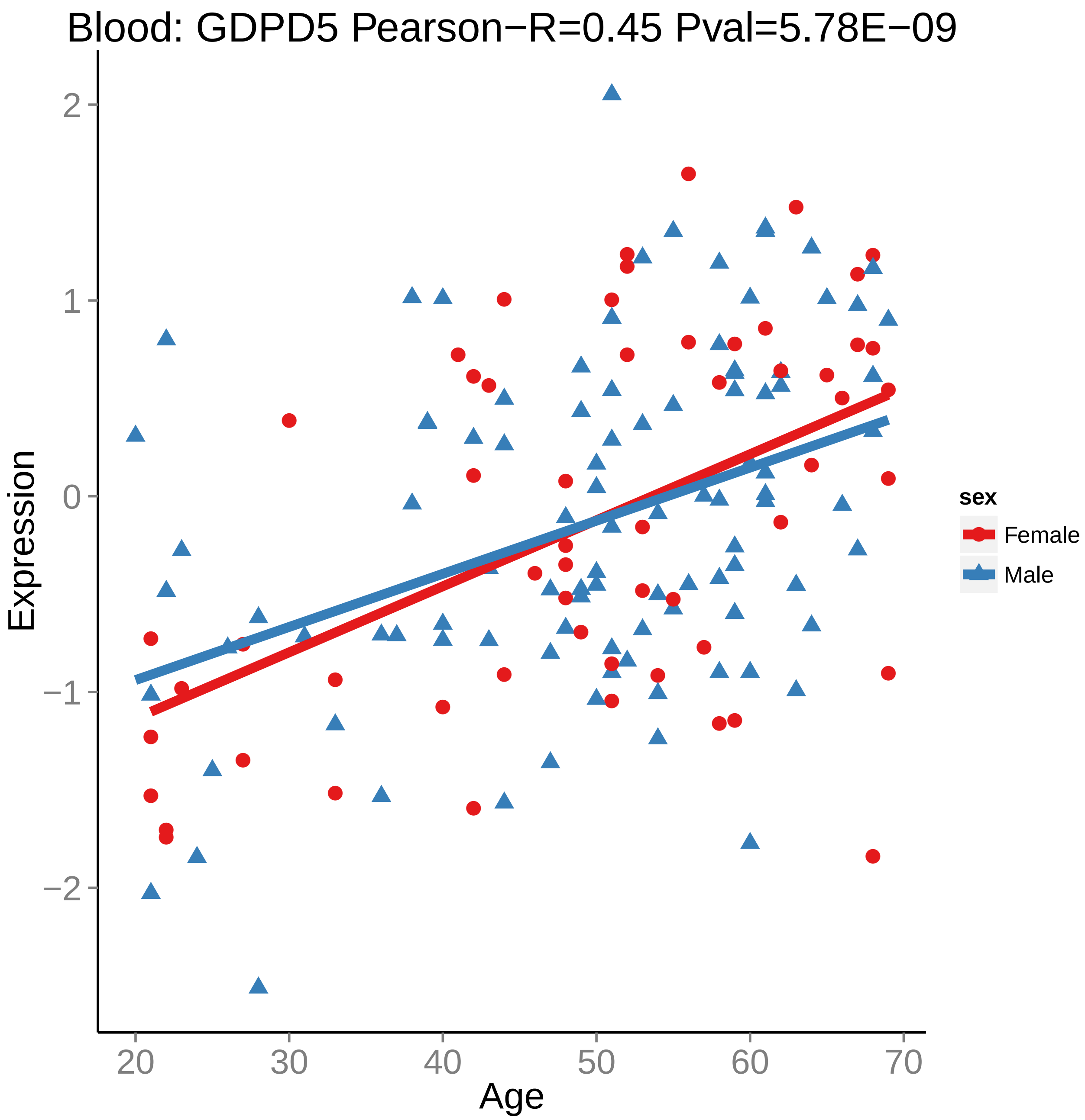

Blood: GDPD5 Pearson−R=0.45 Pval=5.78E−09
Expression
Age
2
1
0
−1
−2
20
30
40
50
60
70
sex
Female
Male

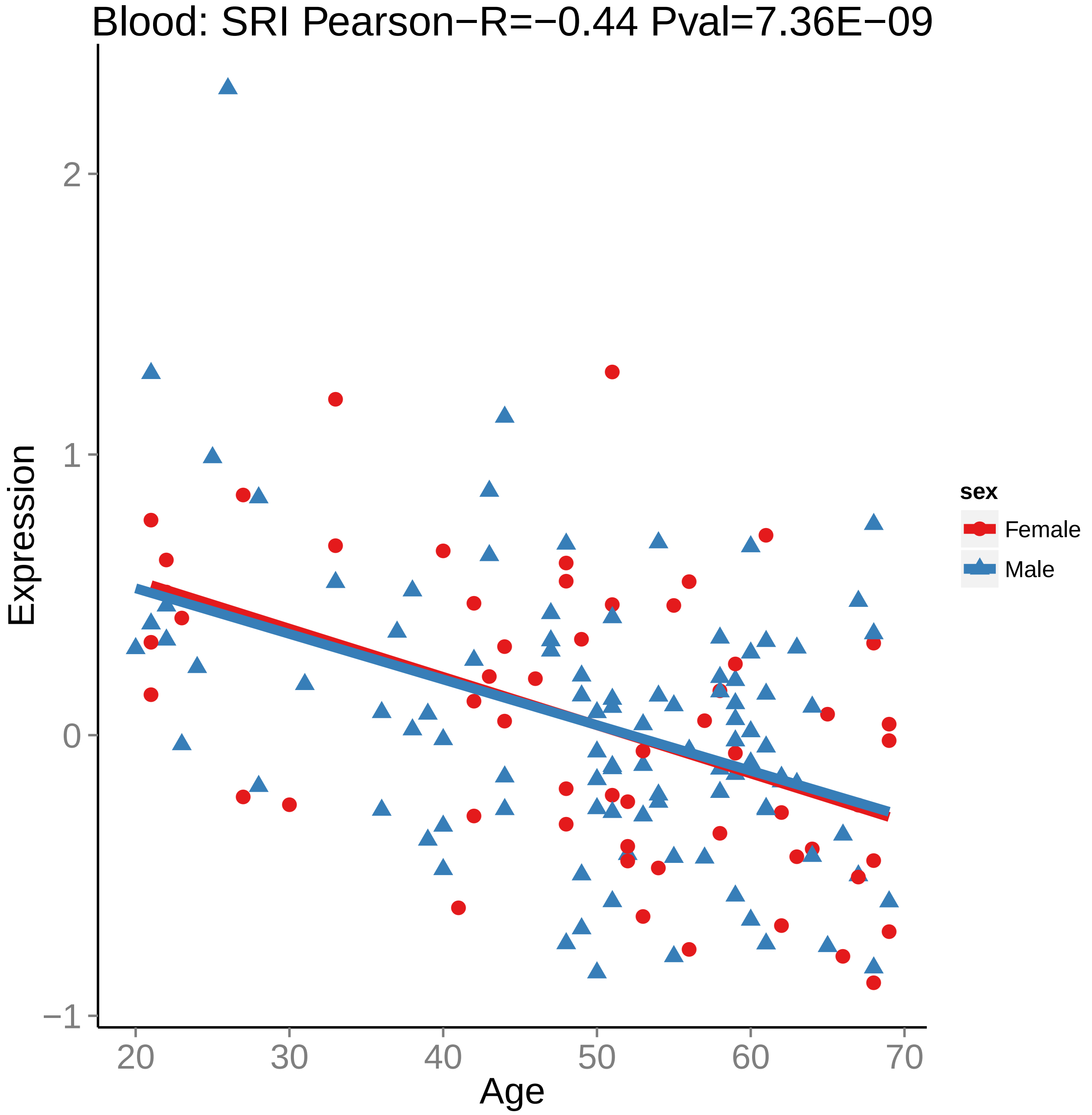

Blood: SRI Pearson−R=−0.44 Pval=7.36E−09
Expression
Age
2
1
0
−1
20
30
40
50
60
70
sex
Female
Male

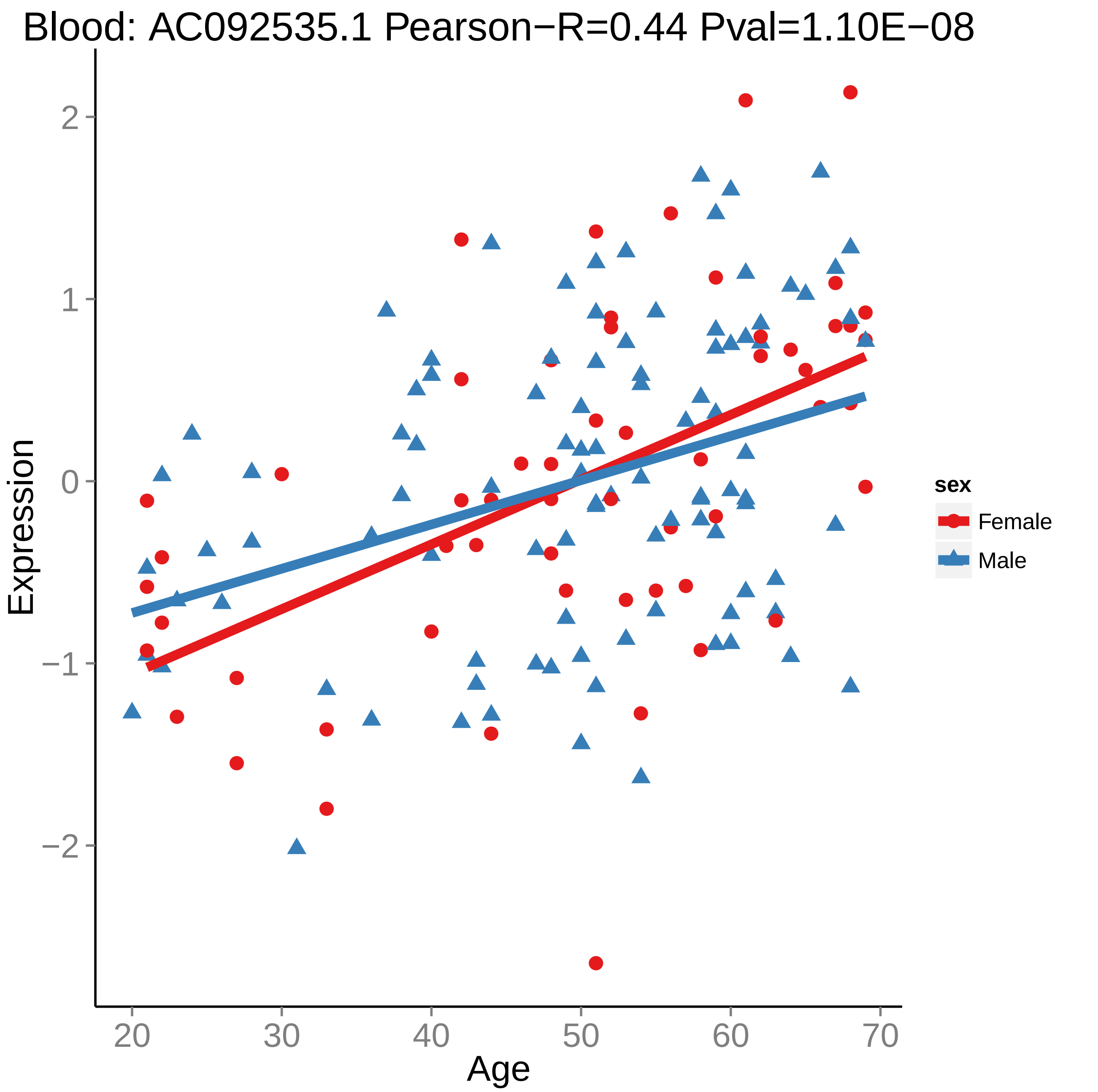
Blood: AC092535.1 Pearson−R=0.44 Pval=1.10E−08
Expression
2
1
0
−1
−2
20
30
40
50
60
70
Age
sex
Female
Male

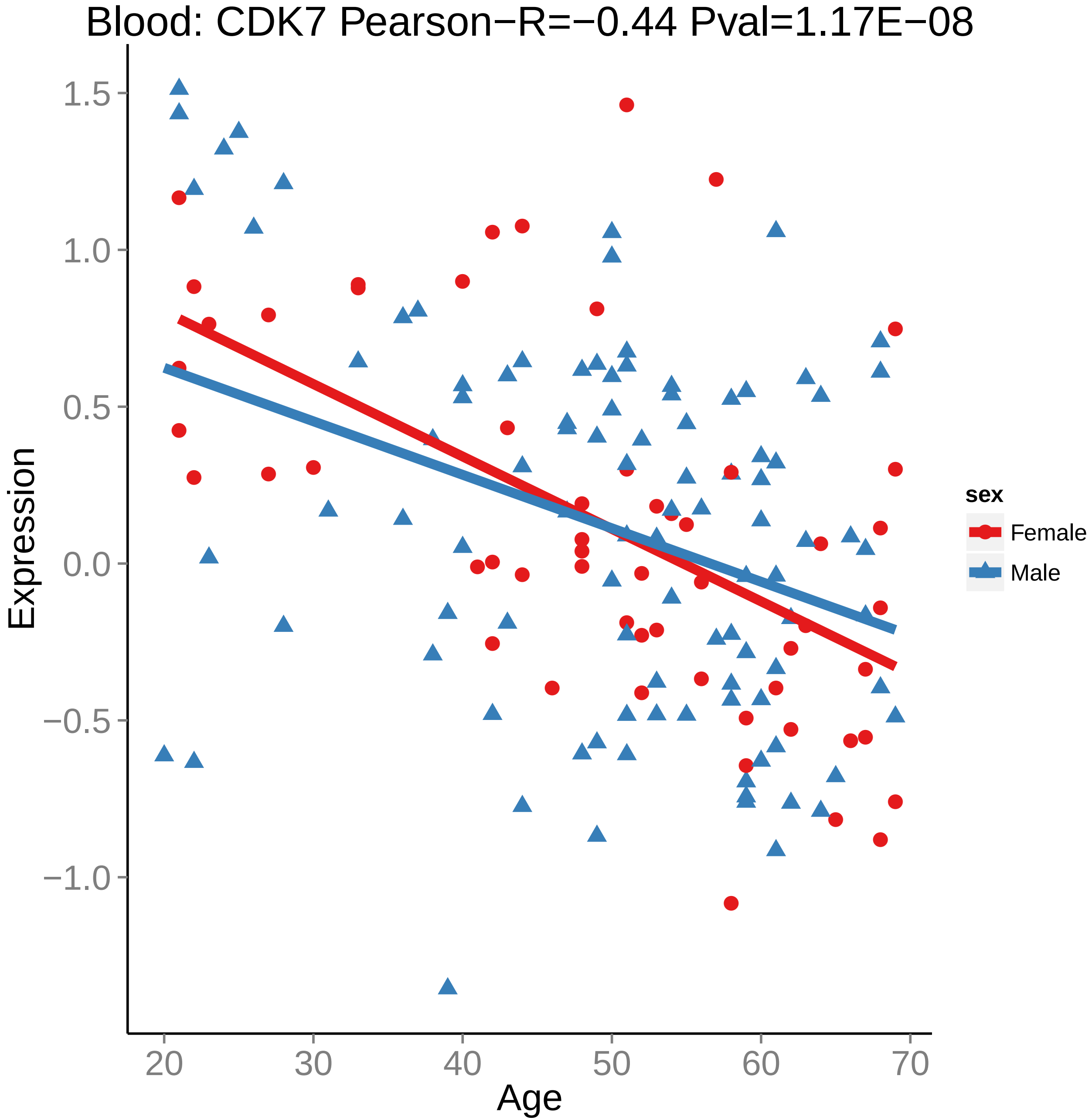

Blood: CDK7 Pearson−R=−0.44 Pval=1.17E−08
Expression
1.5
1.0
0.5
0.0
−0.5
−1.0
20
30
40
50
60
70
Age
sex
Female
Male

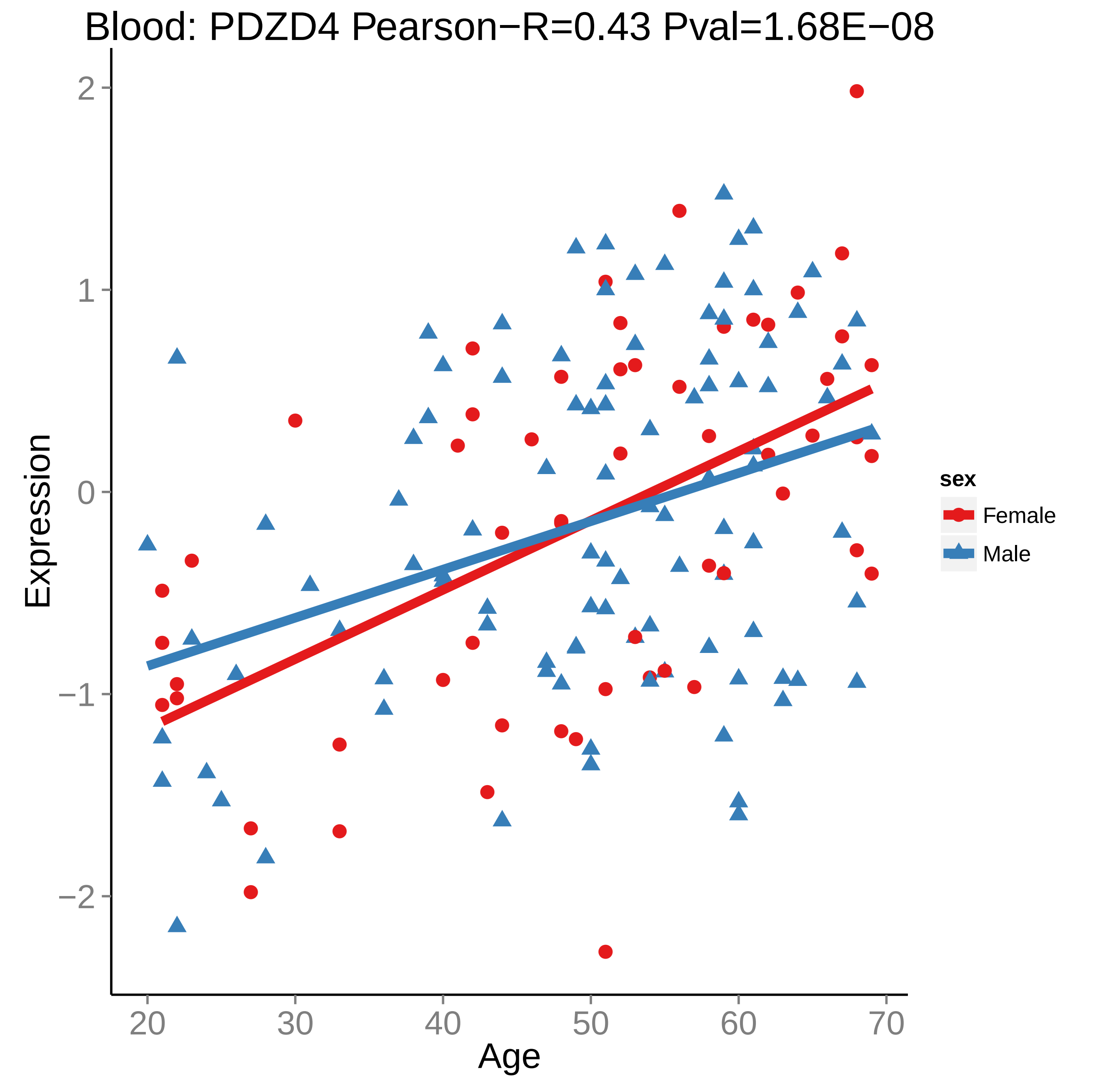

Blood: PDZD4 Pearson−R=0.43 Pval=1.68E−08
Expression
Age
sex
Female
Male

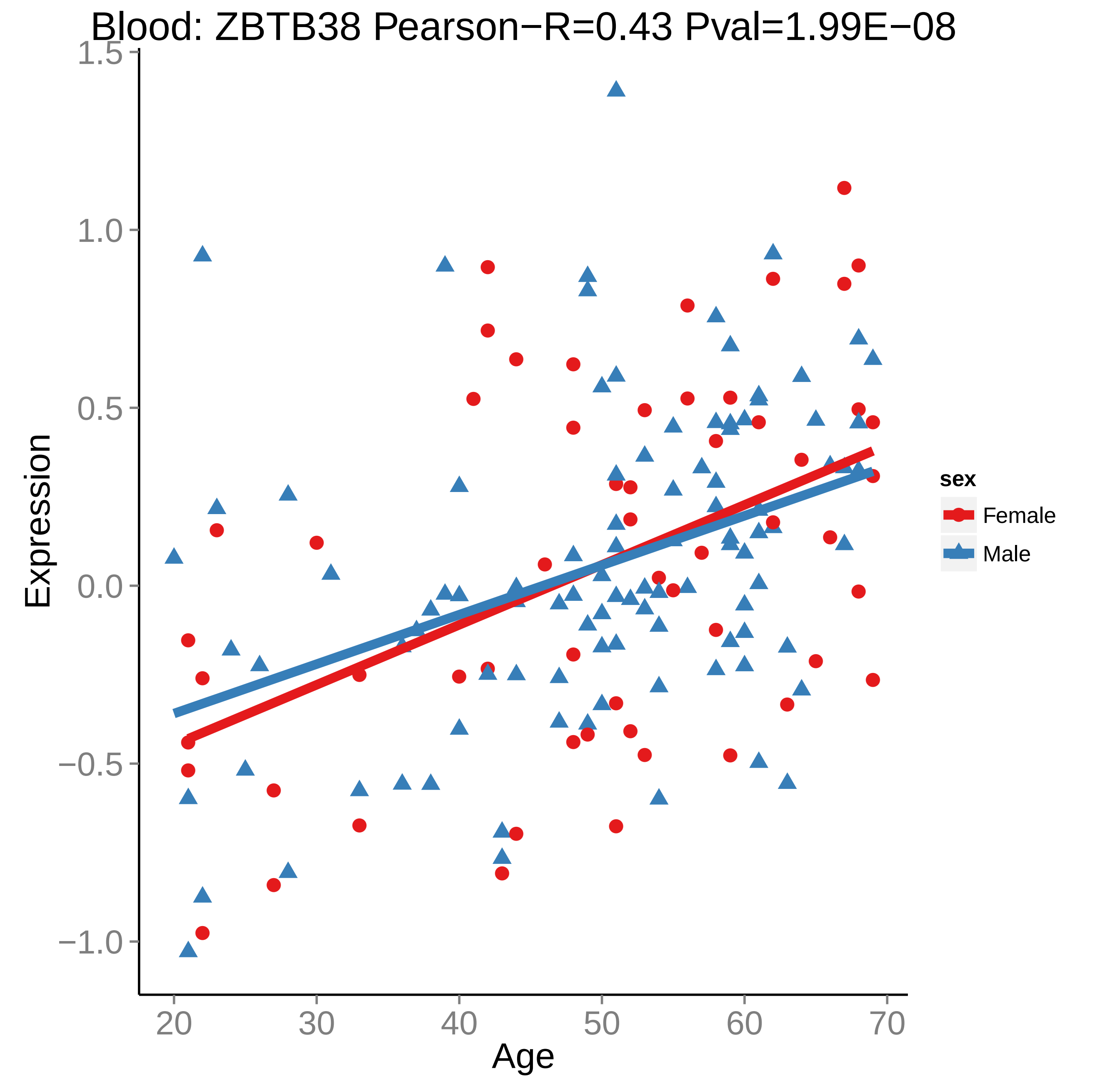

Blood: ZBTB38 Pearson−R=0.43 Pval=1.99E−08
Expression
Age
1.5
1.0
0.5
0.0
−0.5
−1.0
20
30
40
50
60
70
sex
Female
Male

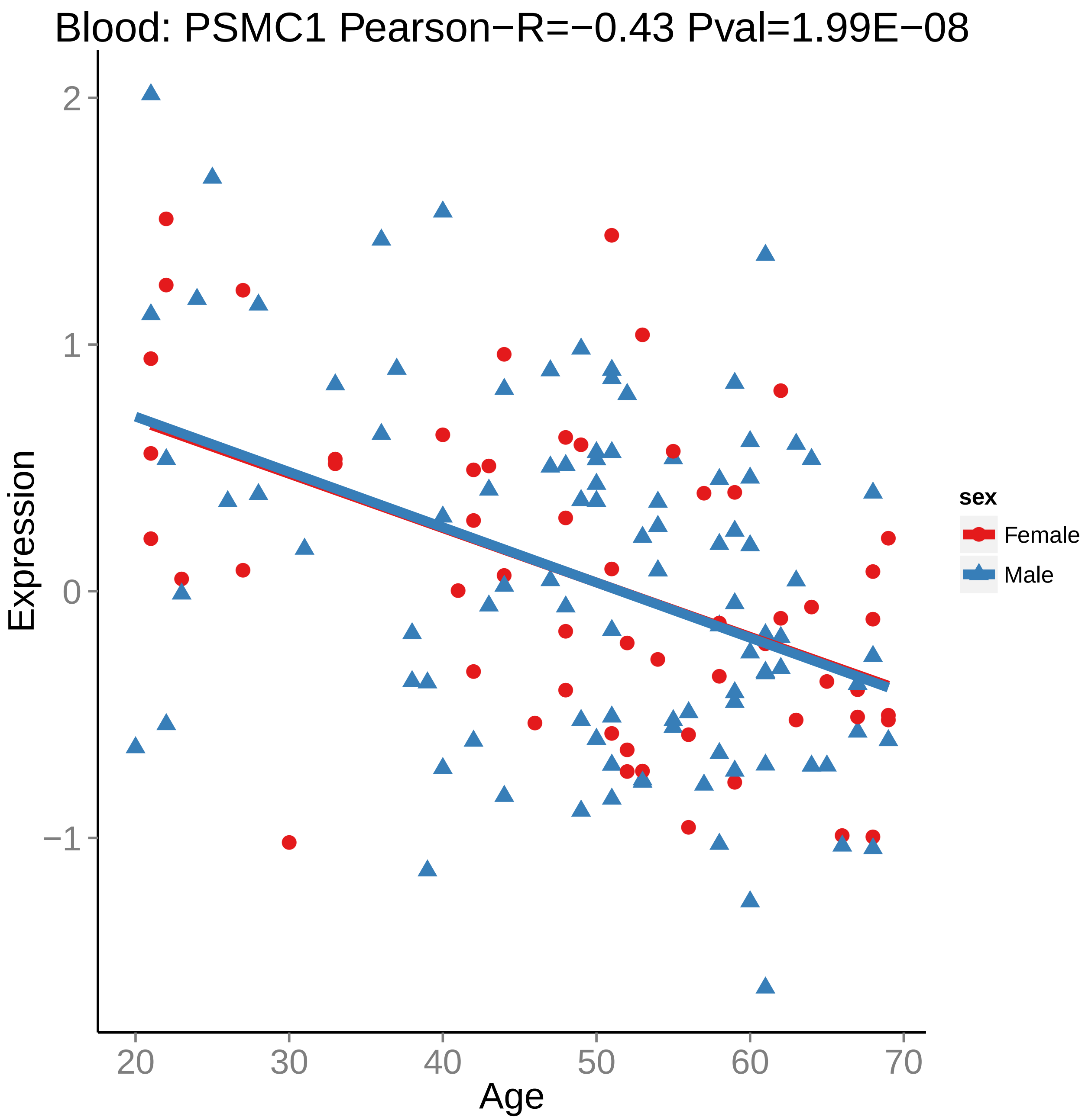
Blood: PSMC1 Pearson−R=−0.43 Pval=1.99E−08
Expression
Age
2
1
0
−1
20
30
40
50
60
70
sex
Female
Male

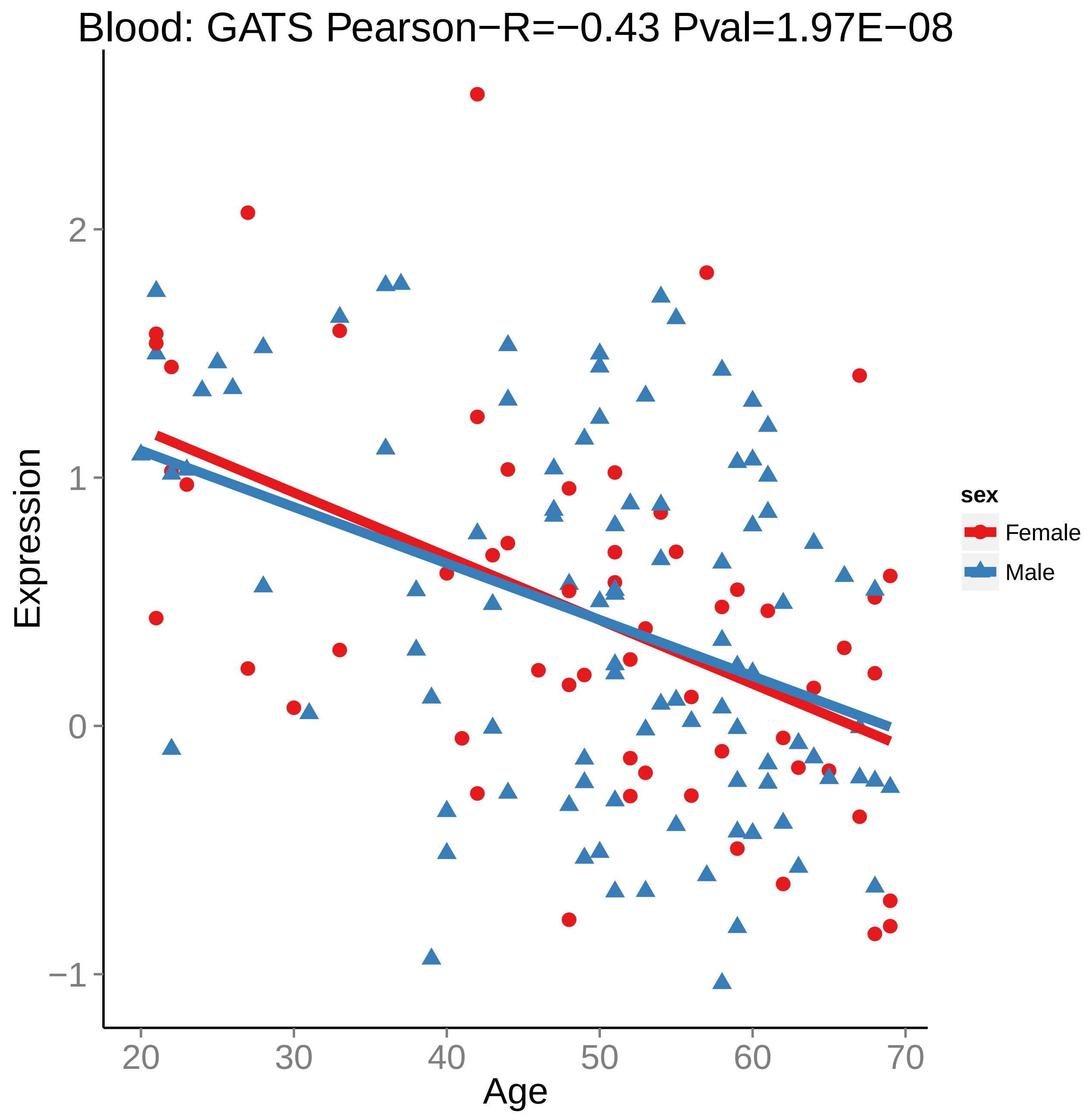
Blood: GATS Pearson−R=−0.43 Pval=1.97E−08
Expression
Age
2
1
0
−1
20
30
40
50
60
70
sex
Female
Male

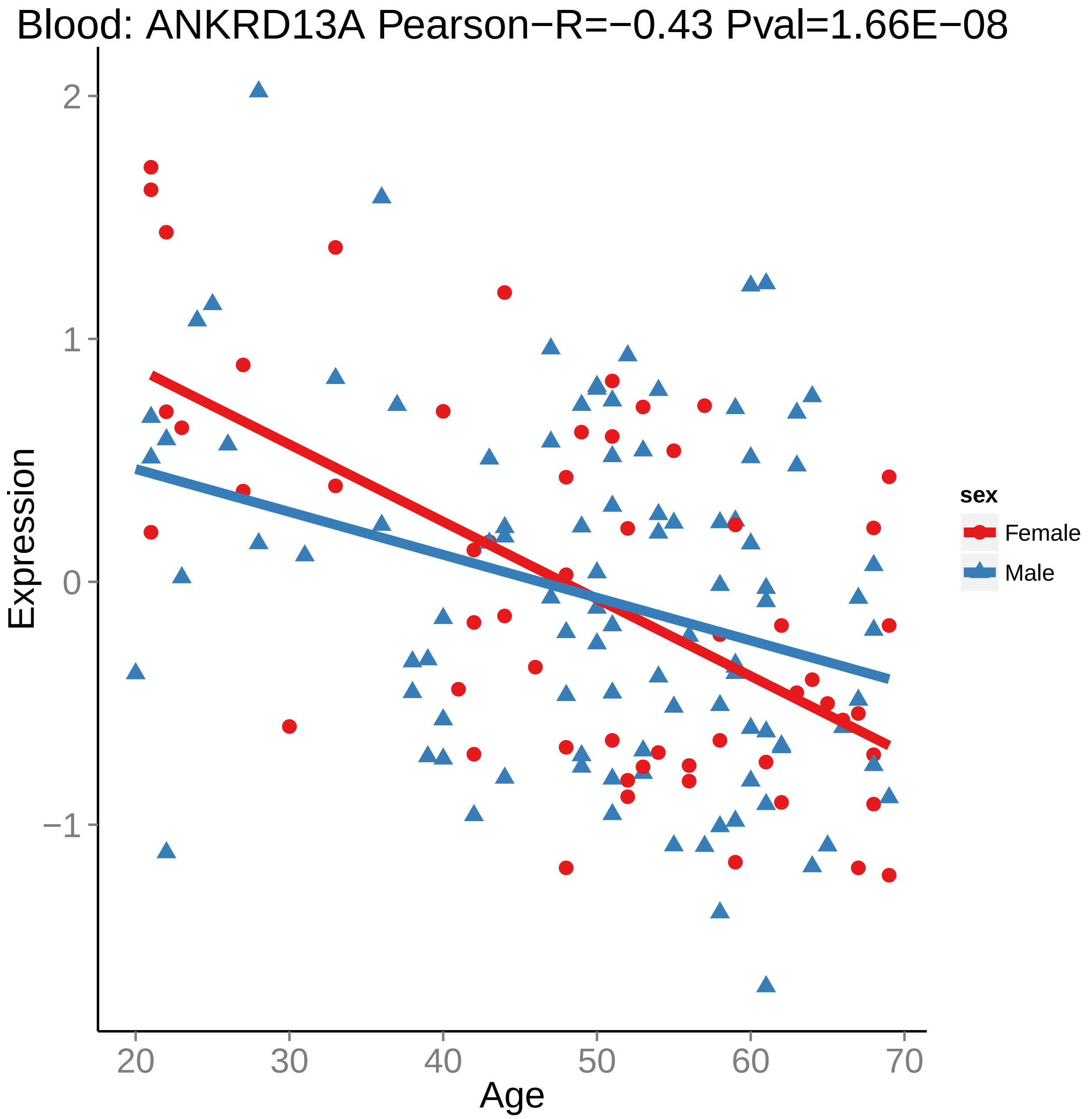

Blood: ANKRD13A Pearson−R=−0.43 Pval=1.66E−08
Expression
Age
2
1
0
−1
20
30
40
50
60
70
sex
Female
Male

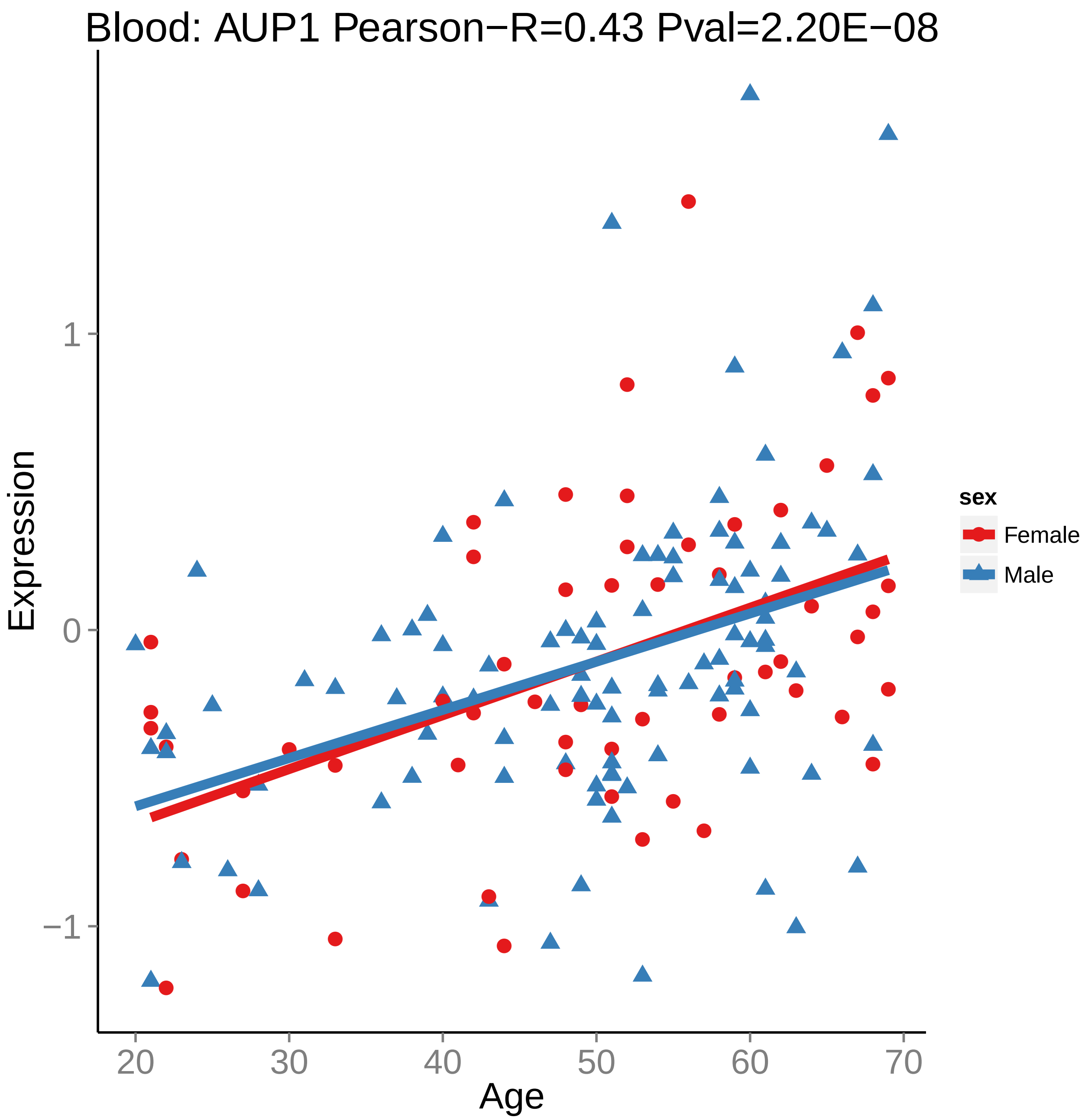
Blood: AUP1 Pearson−R=0.43 Pval=2.20E−08
Expression
1
0
−1
20
30
40
50
60
70
Age
sex
Female
Male

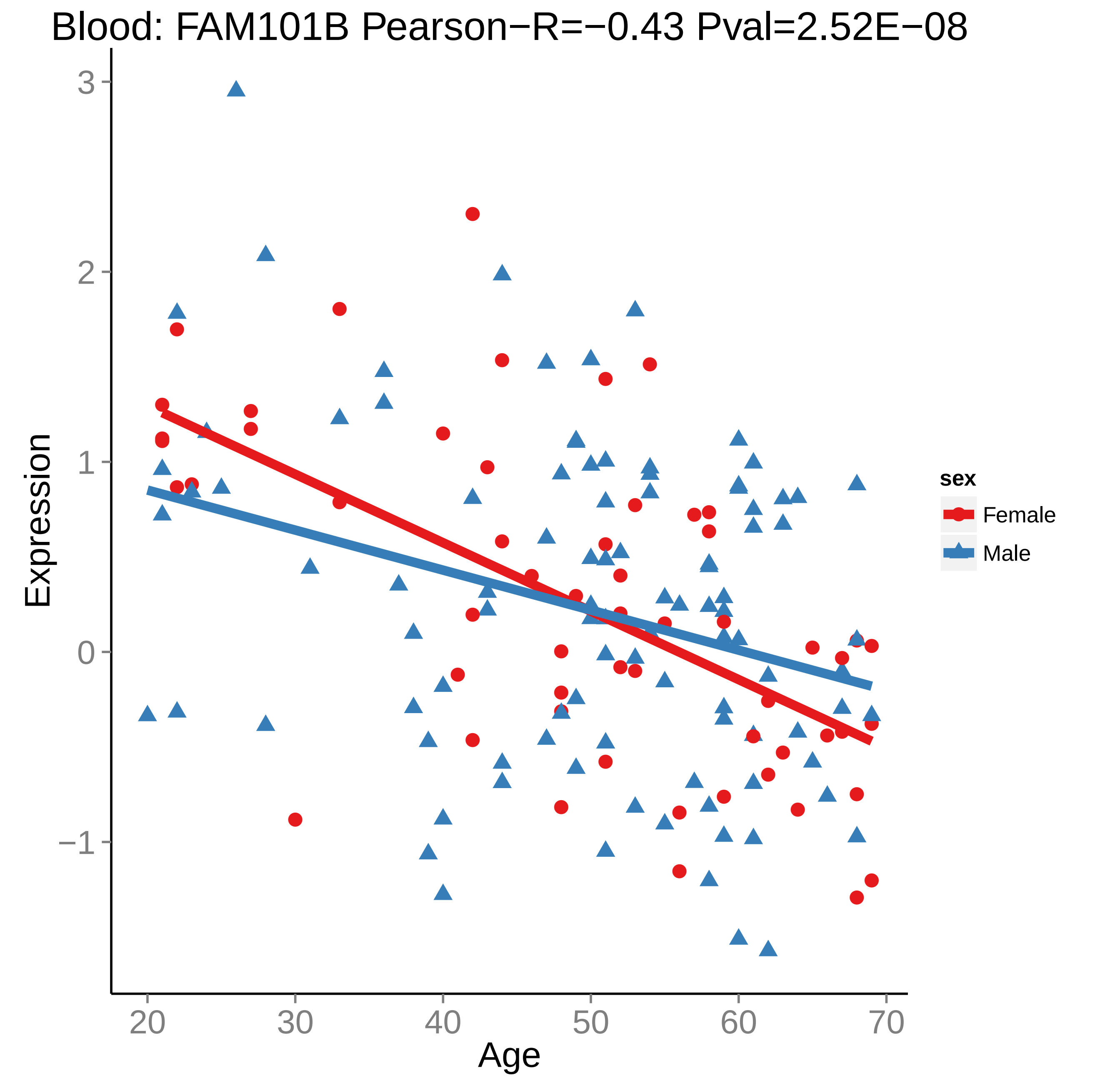

Blood: FAM101B Pearson−R=−0.43 Pval=2.52E−08
Expression
Age
3
2
1
0
−1
20
30
40
50
60
70
sex
Female
Male

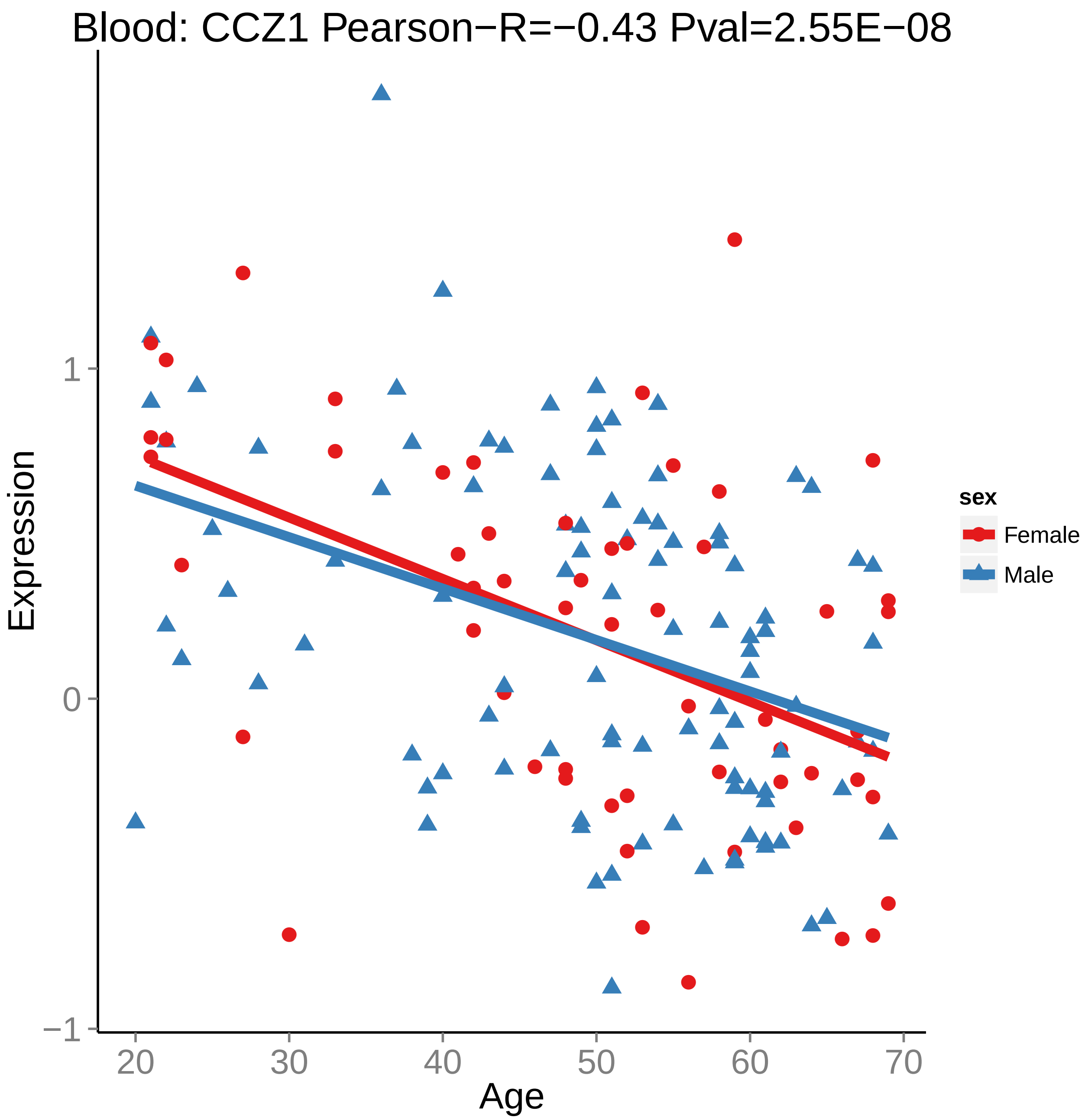
Blood: CCZ1 Pearson−R=−0.43 Pval=2.55E−08
Expression
1
0
−1
20
30
40
50
60
70
Age
sex
Female
Male

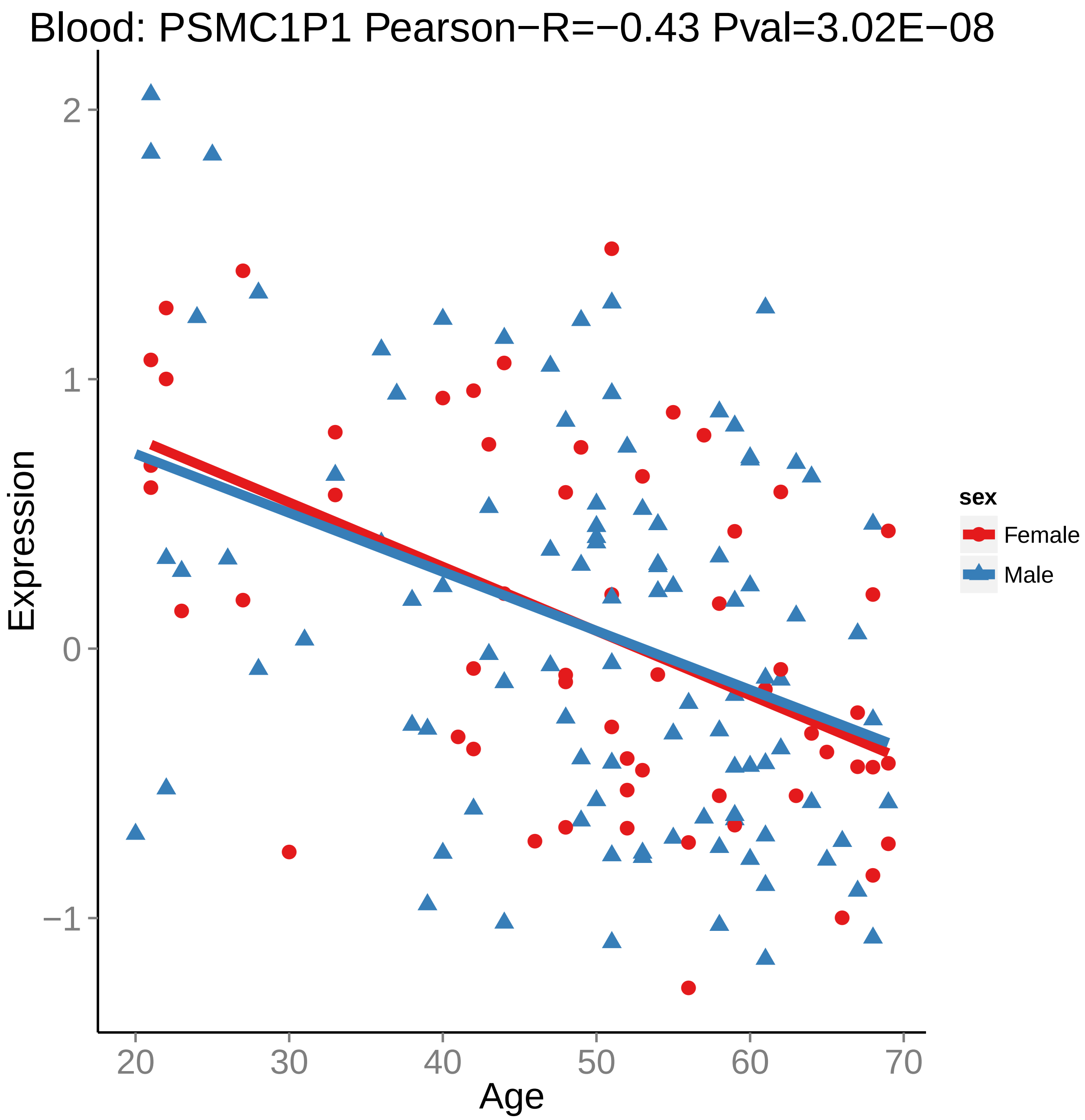
Blood: PSMC1P1 Pearson−R=−0.43 Pval=3.02E−08
Expression
Age
2
1
0
−1
20
30
40
50
60
70
sex
Female
Male

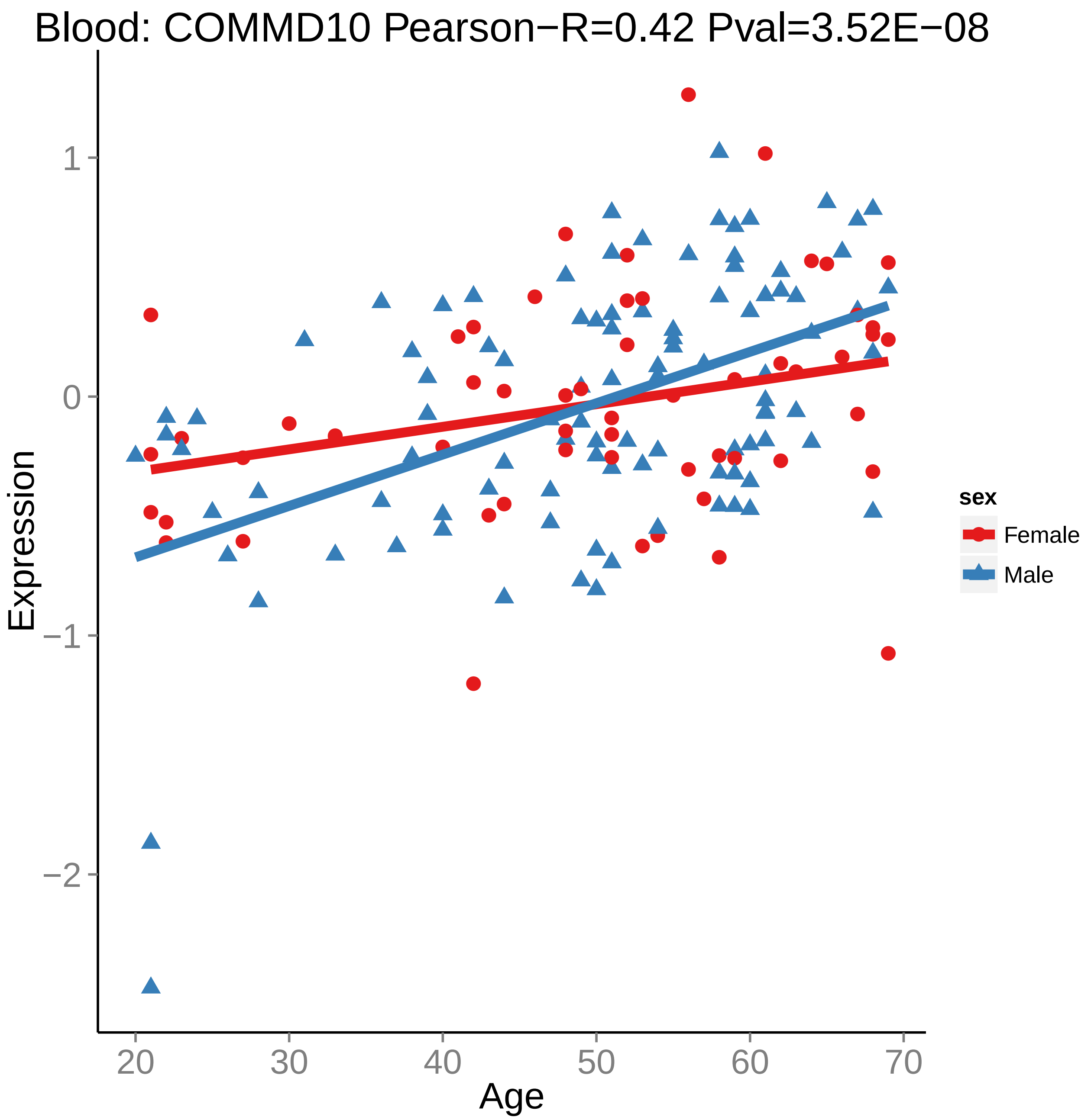

Blood: COMMD10 Pearson−R=0.42 Pval=3.52E−08
Expression
1
0
−1
−2
20
30
40
50
60
70
Age
sex
Female
Male

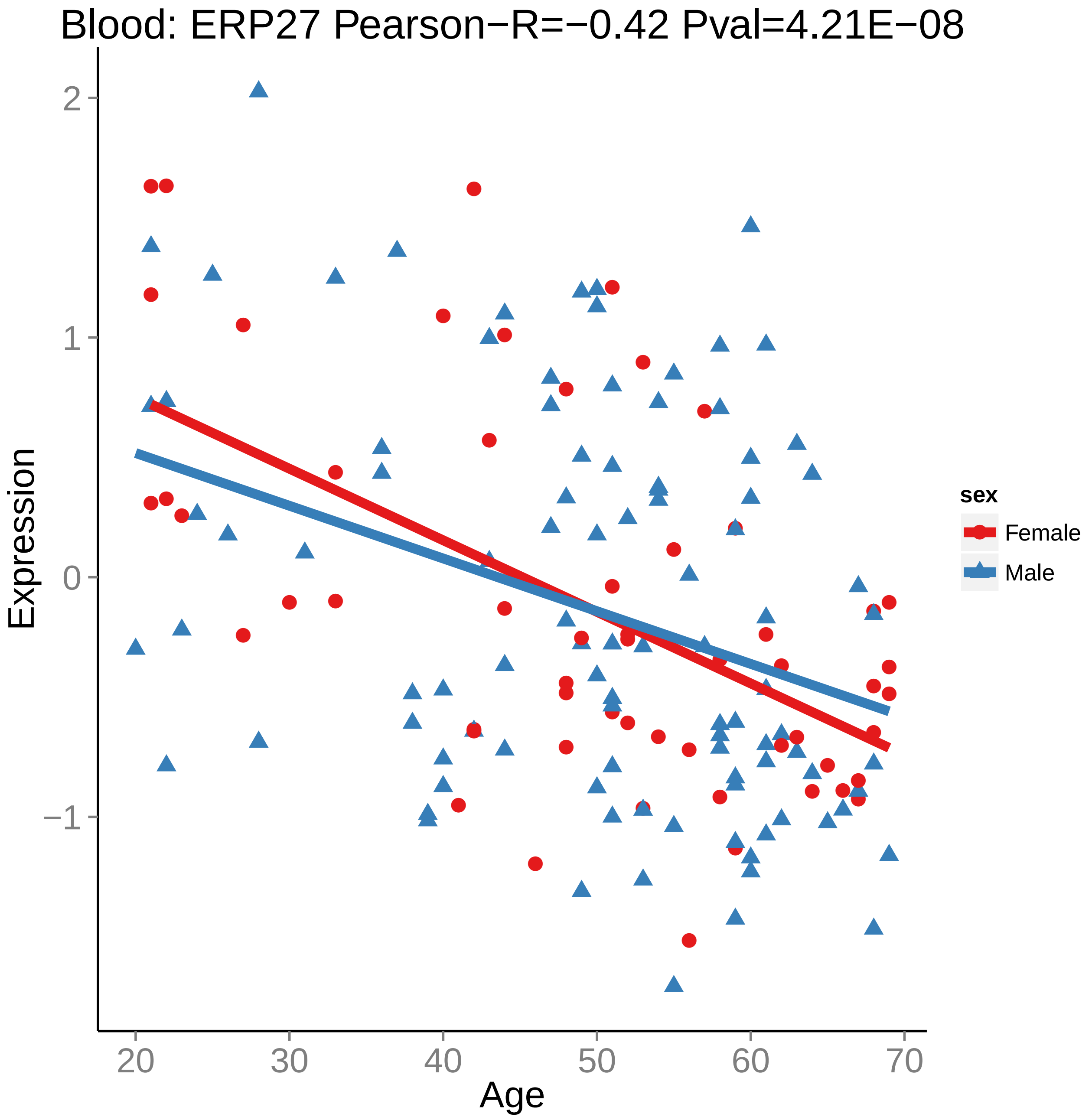
Blood: ERP27 Pearson−R=−0.42 Pval=4.21E−08
Expression
2
1
0
−1
20
30
40
50
60
70
Age
sex
Female
Male

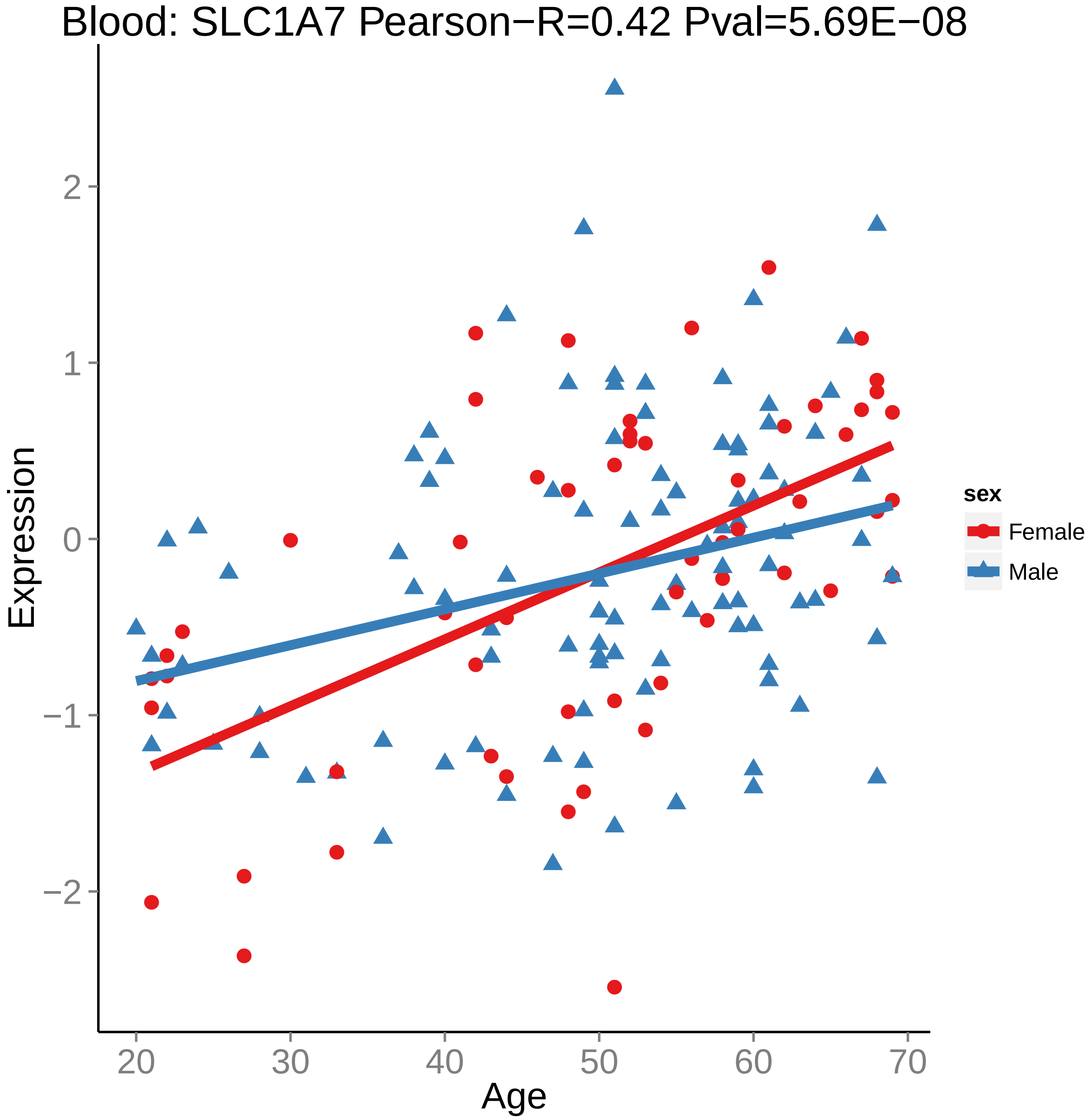

Blood: SLC1A7 Pearson−R=0.42 Pval=5.69E−08
Expression
2
1
0
−1
−2
20
30
40
50
60
70
Age
sex
Female
Male

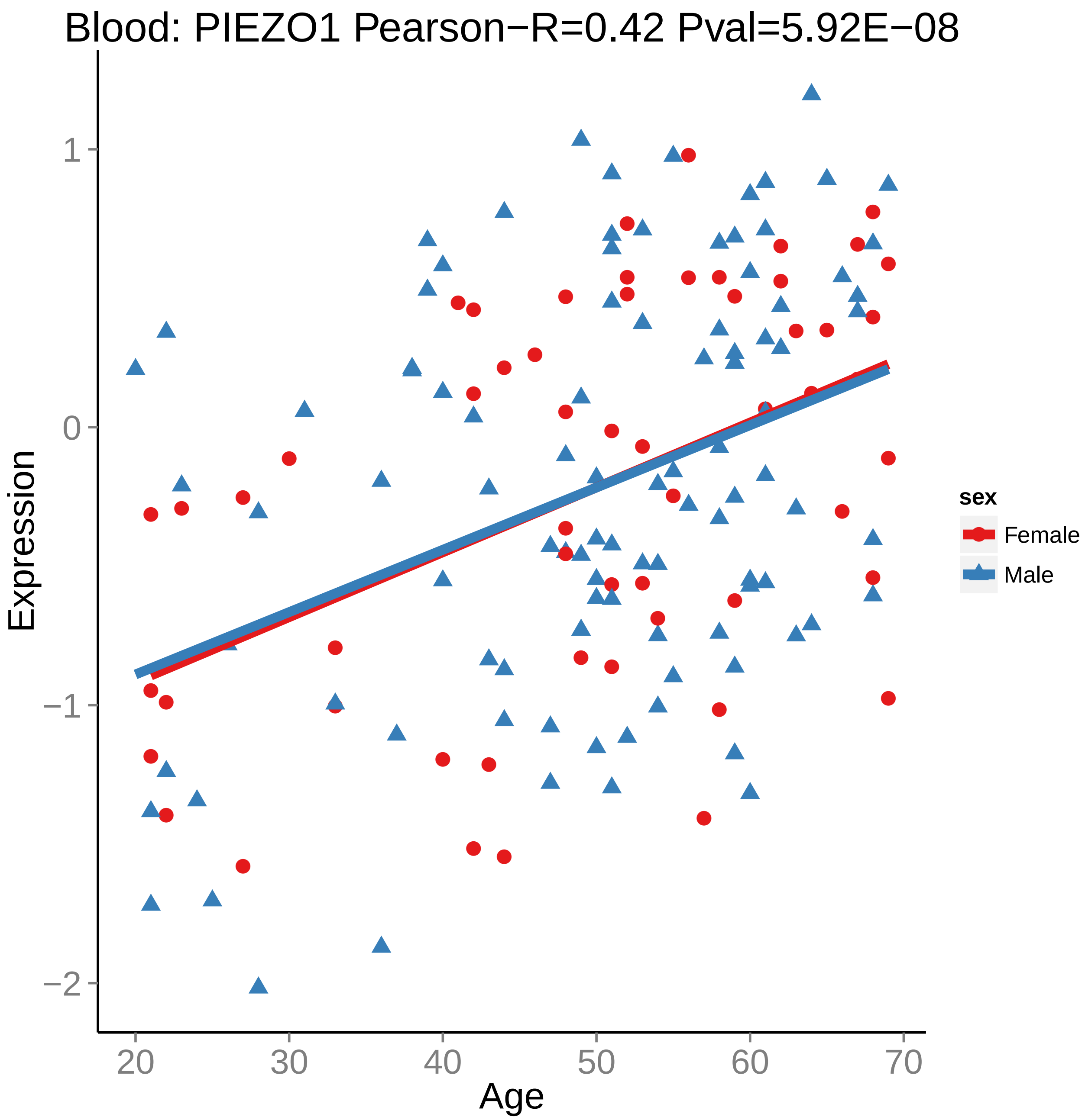

Blood: PIEZO1 Pearson−R=0.42 Pval=5.92E−08
Expression
1
0
−1
−2
Age
20
30
40
50
60
70
sex
Female
Male

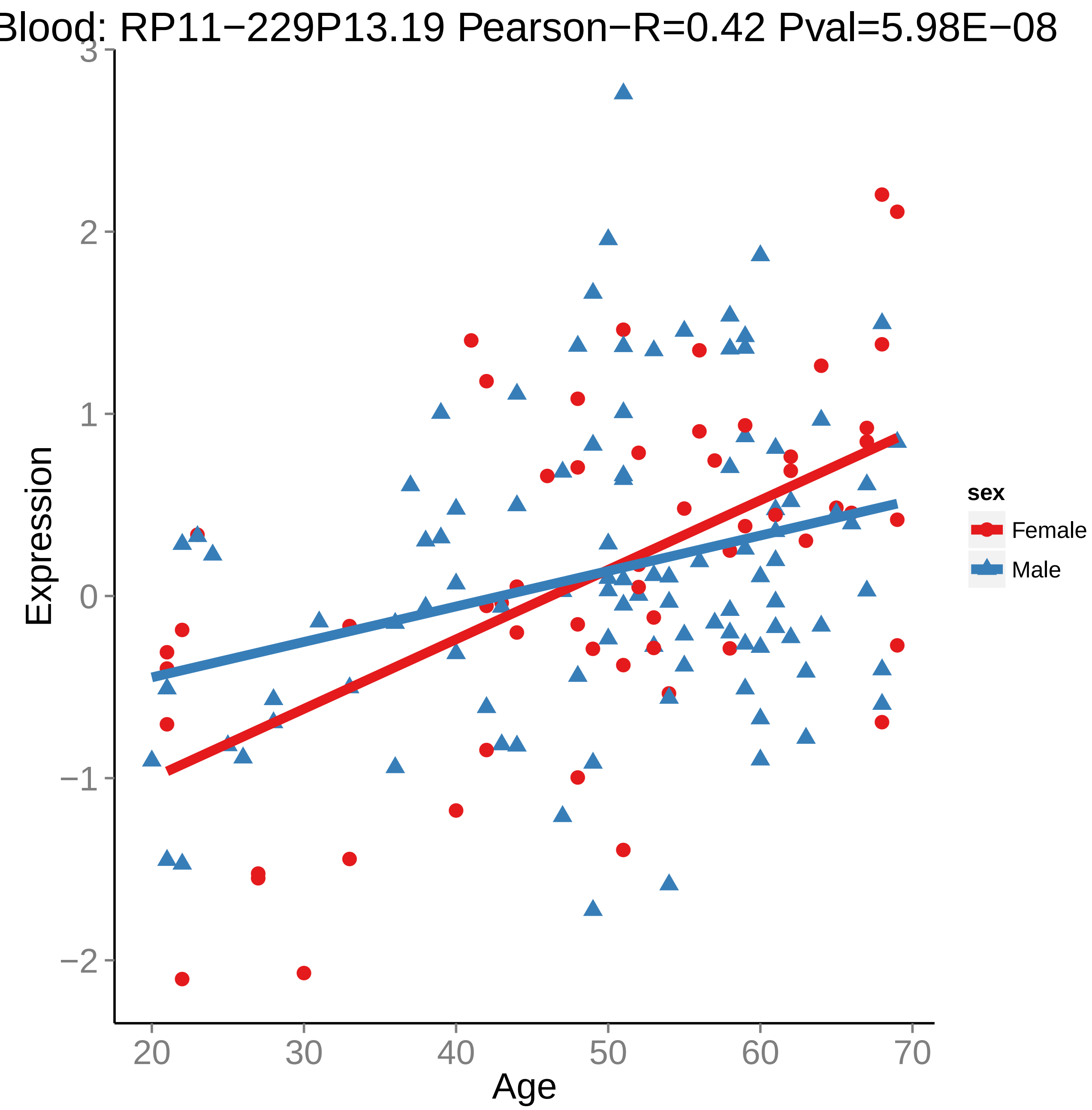

Blood: RP11−229P13.19 Pearson−R=0.42 Pval=5.98E−08
Expression
Age
3
2
1
0
−1
−2
20
30
40
50
60
70
sex
Female
Male

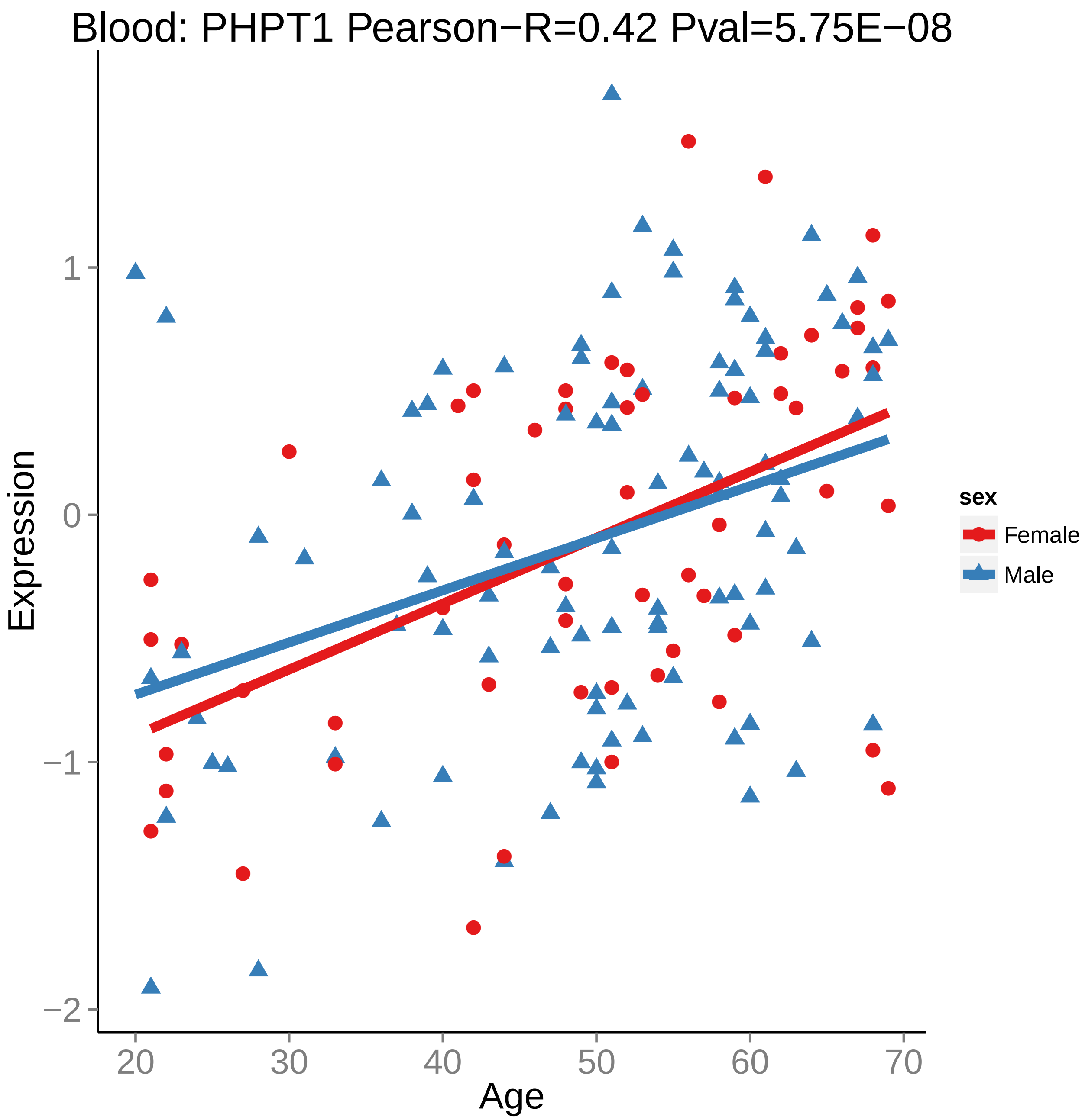

Blood: PHPT1 Pearson−R=0.42 Pval=5.75E−08
Expression
1
0
−1
−2
20
30
40
50
60
70
Age
sex
Female
Male

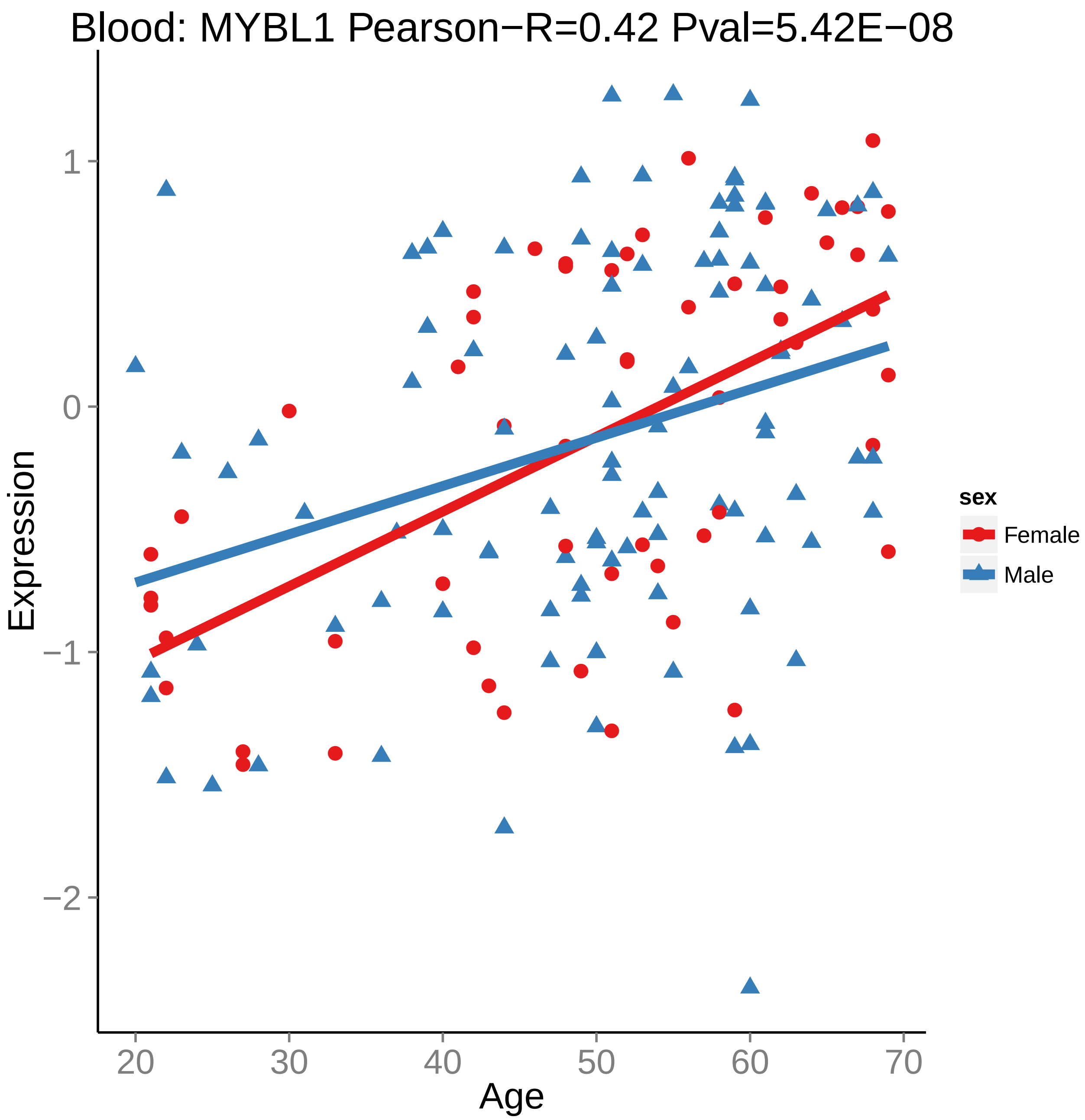

Blood: MYBL1 Pearson−R=0.42 Pval=5.42E−08
Expression
Age
sex
Female
Male

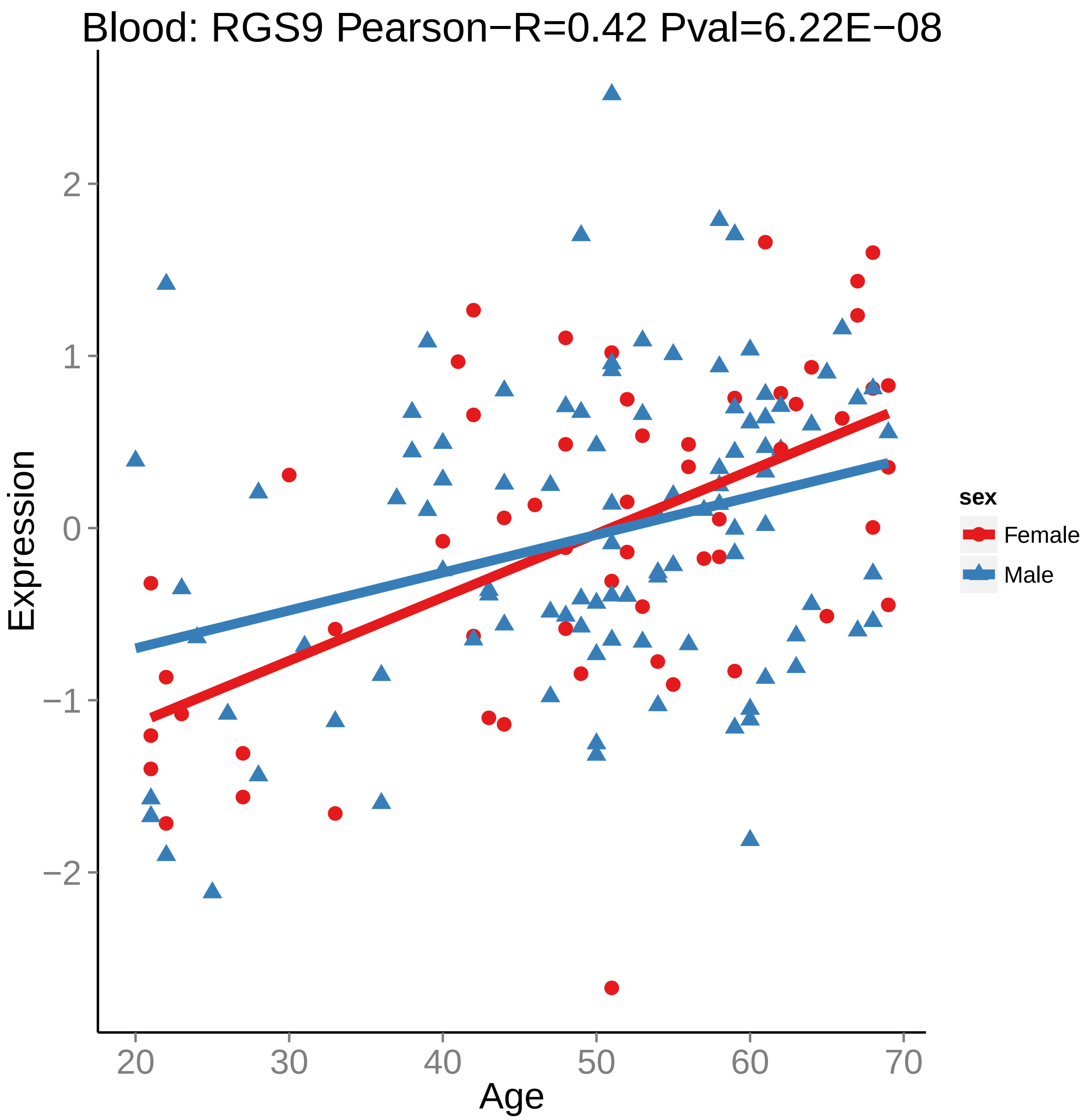

Blood: RGS9 Pearson−R=0.42 Pval=6.22E−08
Expression
Age
2
1
0
−1
−2
20
30
40
50
60
70
sex
Female
Male

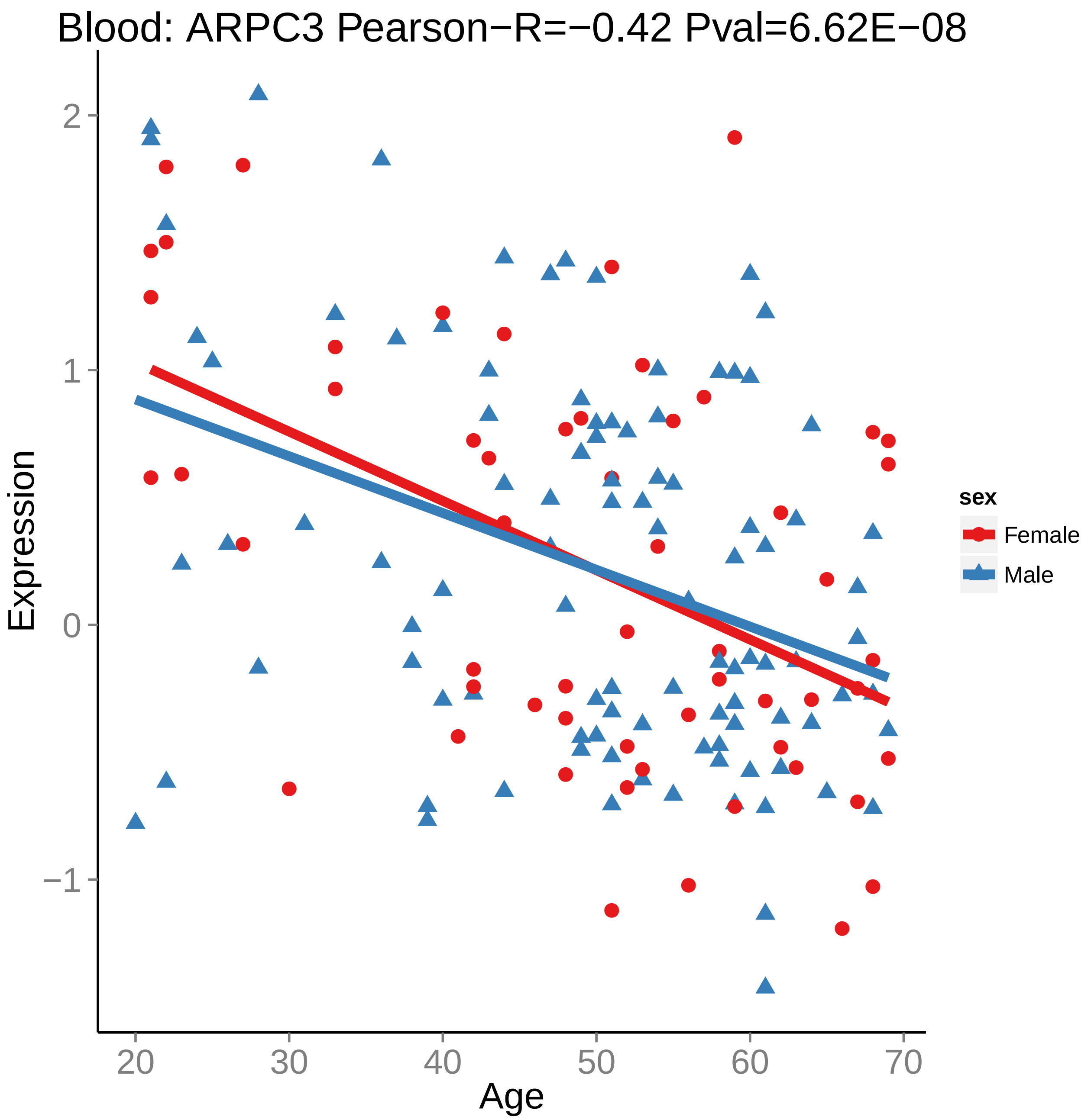
Blood: ARPC3 Pearson−R=−0.42 Pval=6.62E−08
Expression
Age
2
1
0
−1
20
30
40
50
60
70
sex
Female
Male

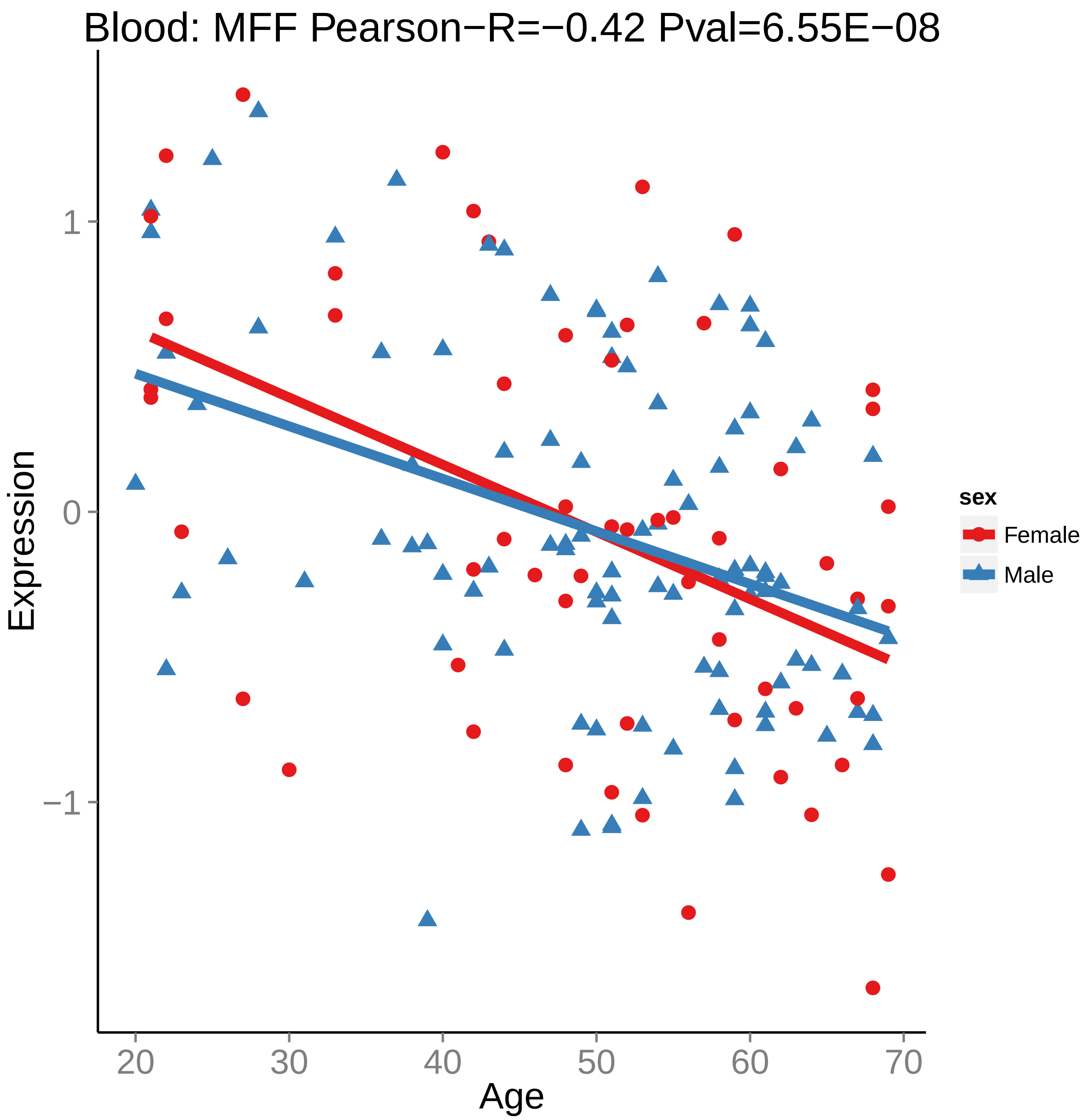

Blood: MFF Pearson−R=−0.42 Pval=6.55E−08
Expression
1
0
−1
20
30
40
50
60
70
Age
sex
Female
Male

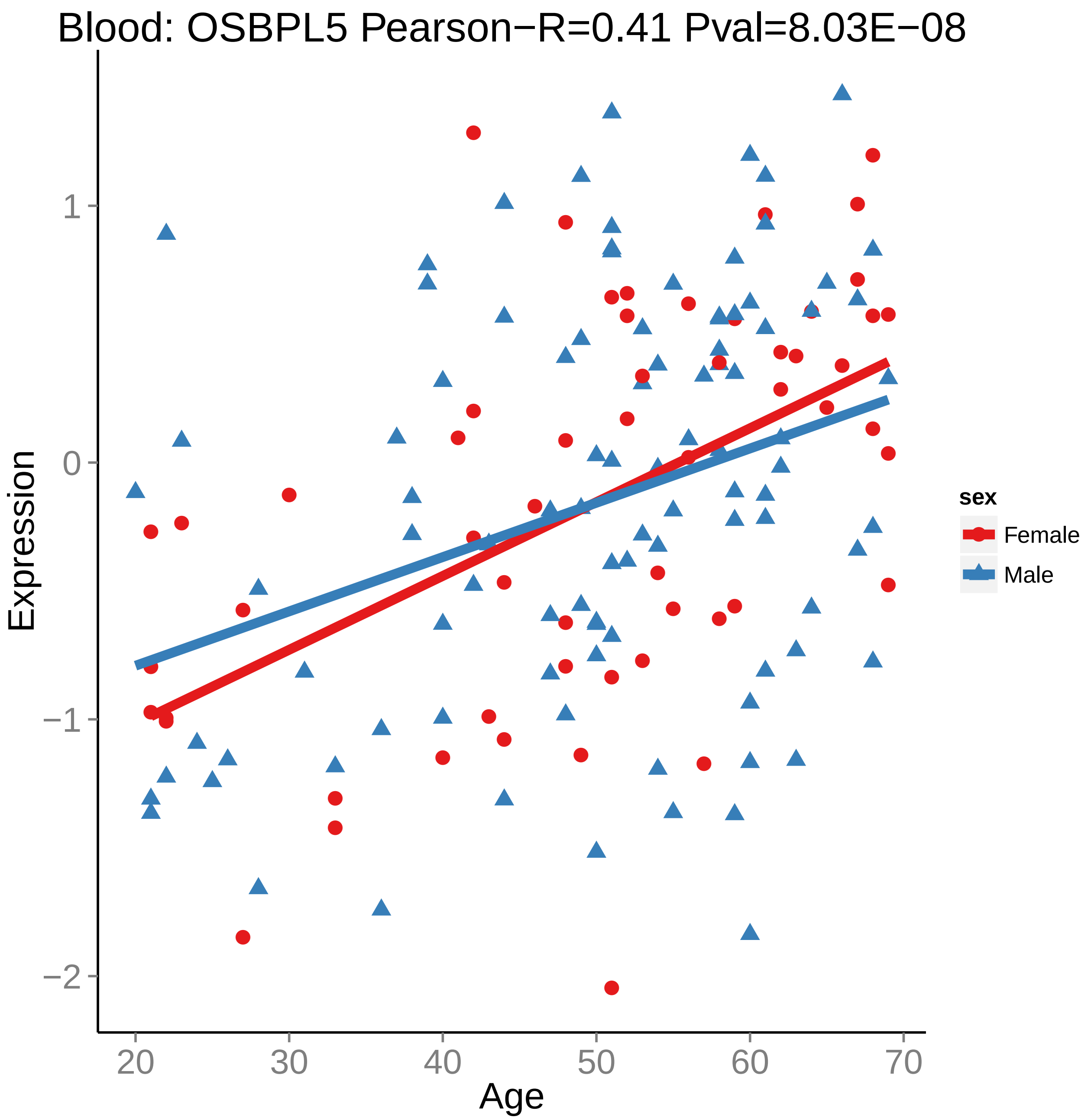
Blood: OSBPL5 Pearson−R=0.41 Pval=8.03E−08
Expression
1
0
−1
−2
20
30
40
50
60
70
Age
sex
Female
Male

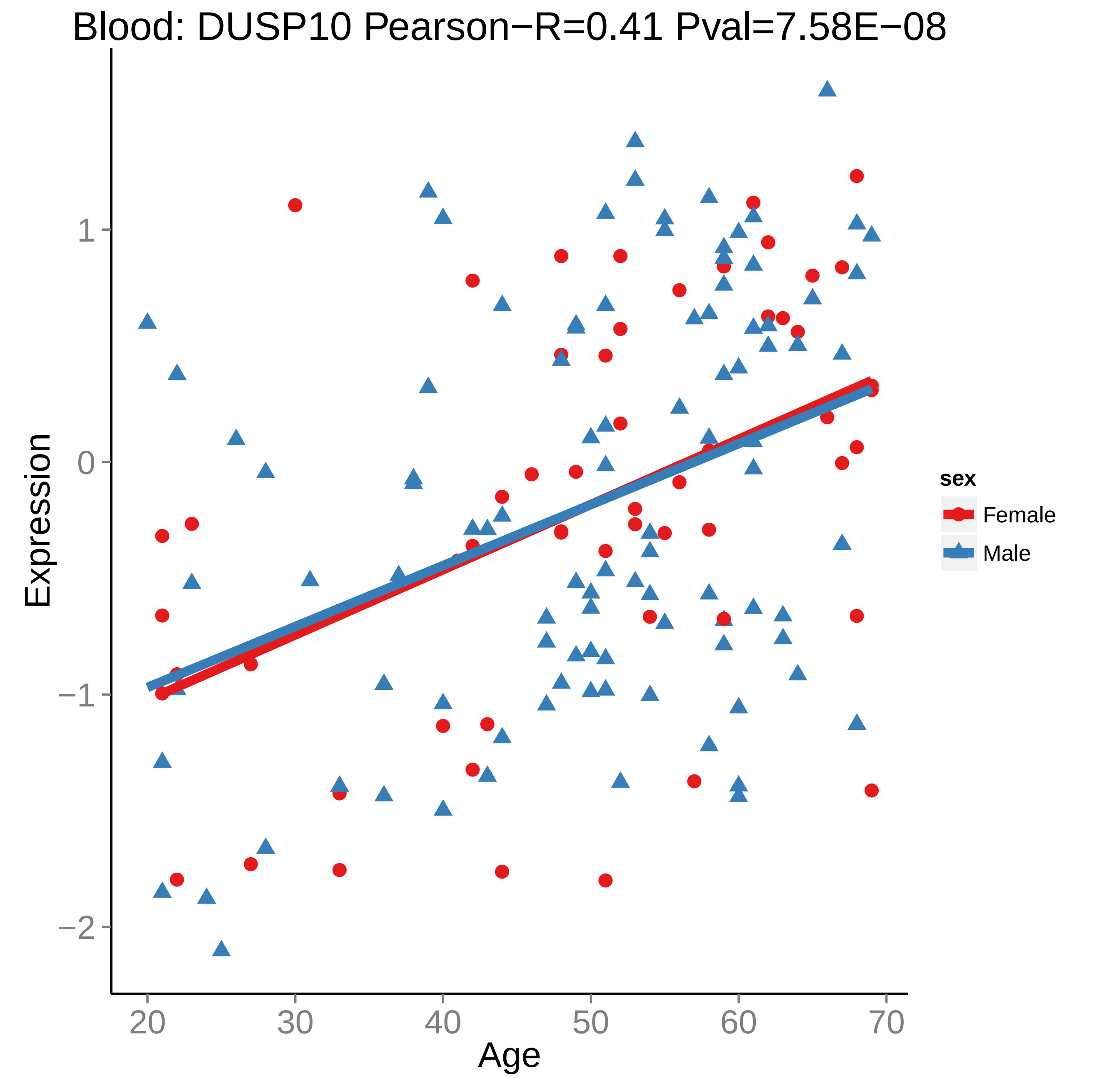

Blood: DUSP10 Pearson−R=0.41 Pval=7.58E−08
Expression
Age
sex
Female
Male
1
0
−1
−2
20
30
40
50
60
70

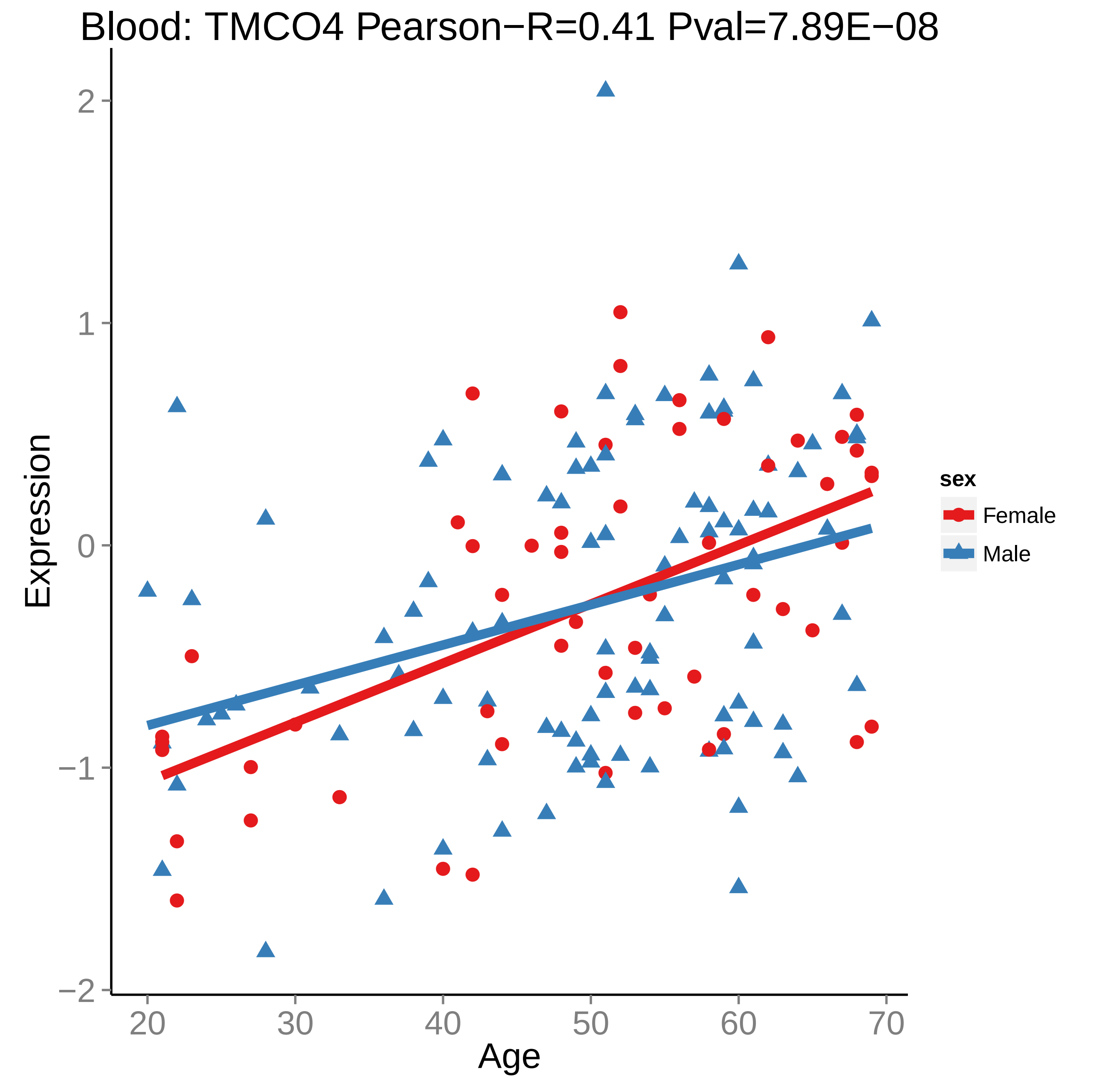
Blood: TMCO4 Pearson−R=0.41 Pval=7.89E−08
Expression
Age
2
1
0
−1
−2
20
30
40
50
60
70
sex
Female
Male

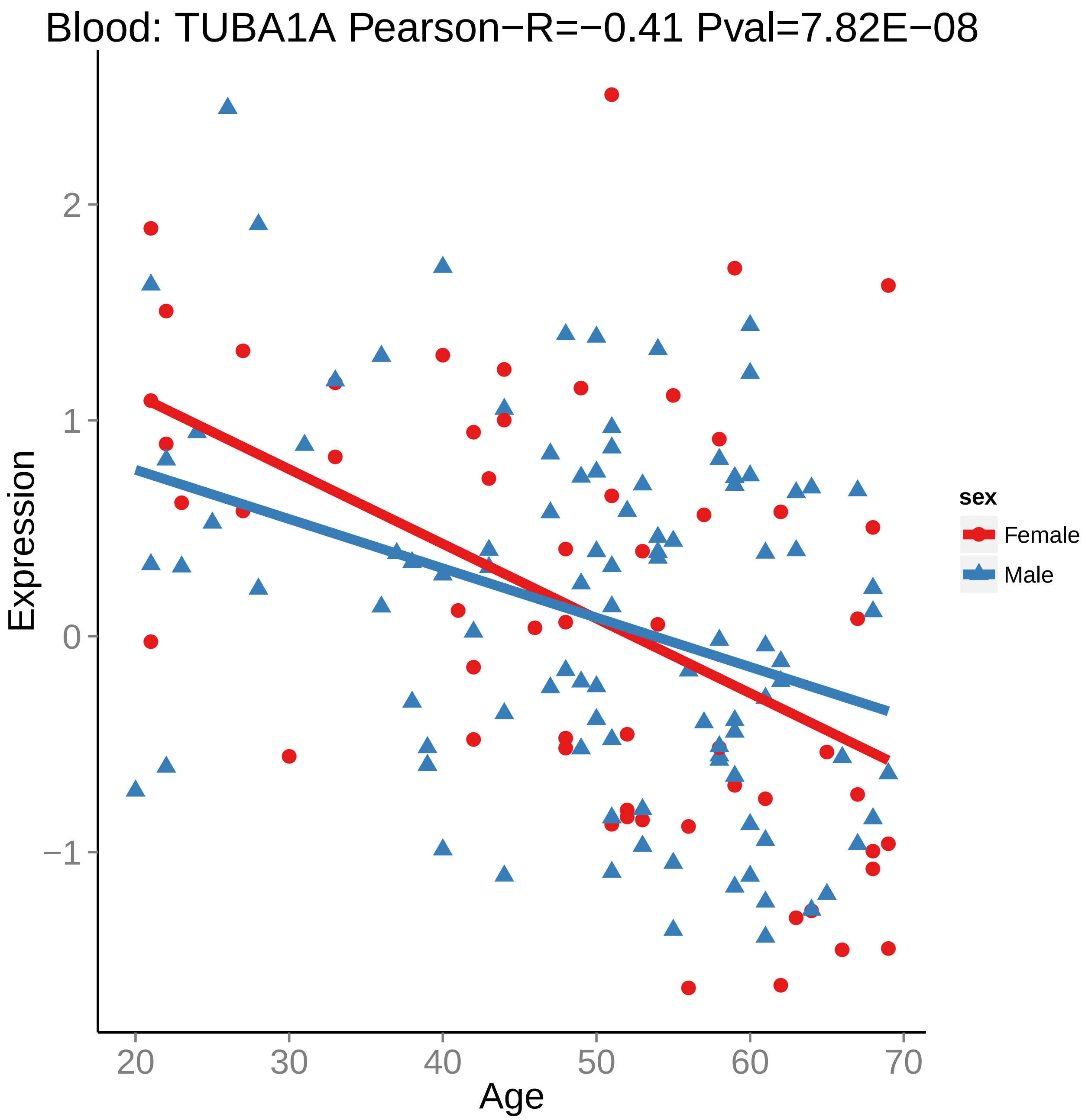

Blood: TUBA1A Pearson−R=−0.41 Pval=7.82E−08
Expression
Age
sex
Female
Male

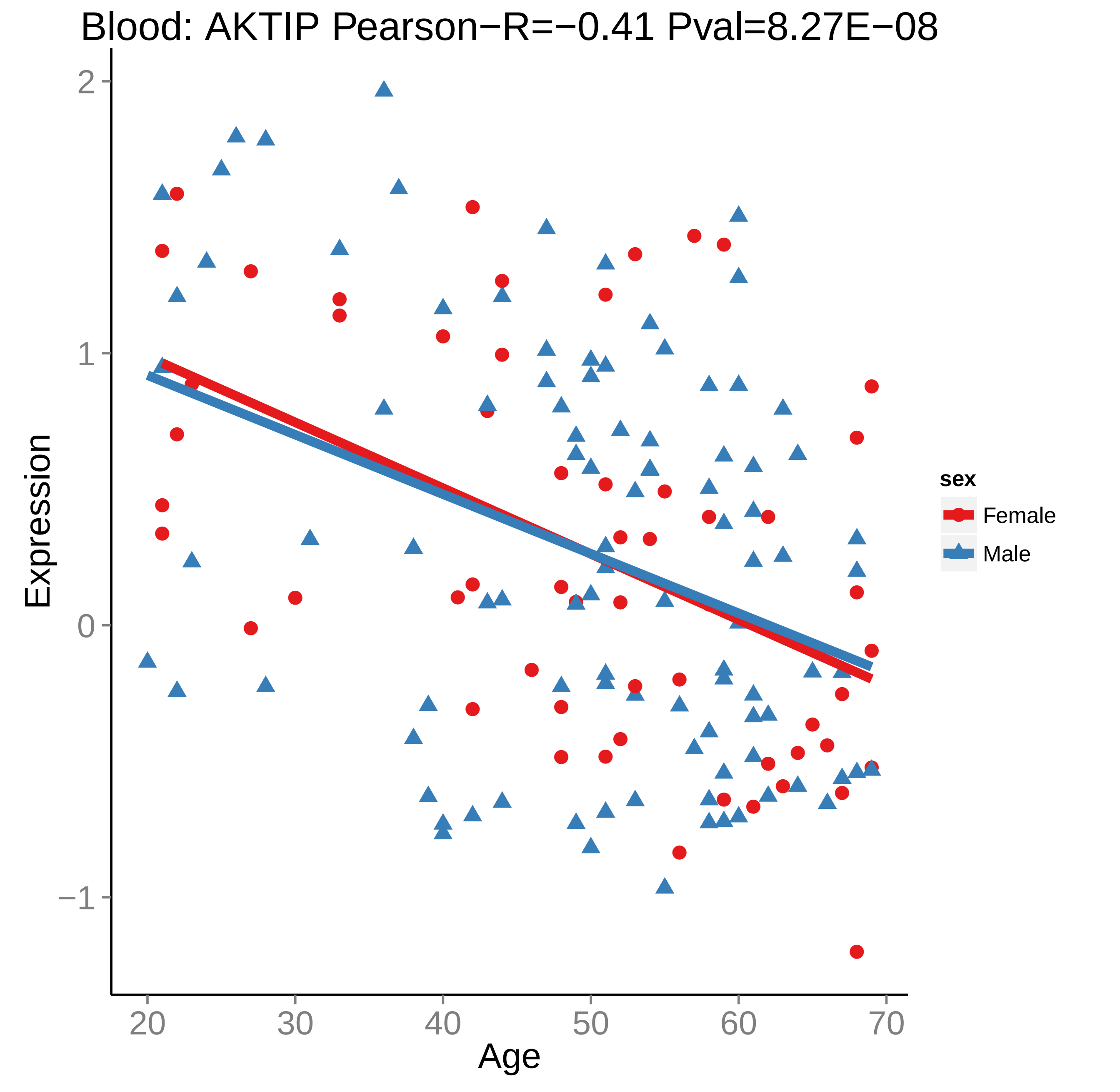

Blood: AKTIP Pearson−R=−0.41 Pval=8.27E−08
Expression
Age
2
1
0
−1
20
30
40
50
60
70
sex
Female
Male

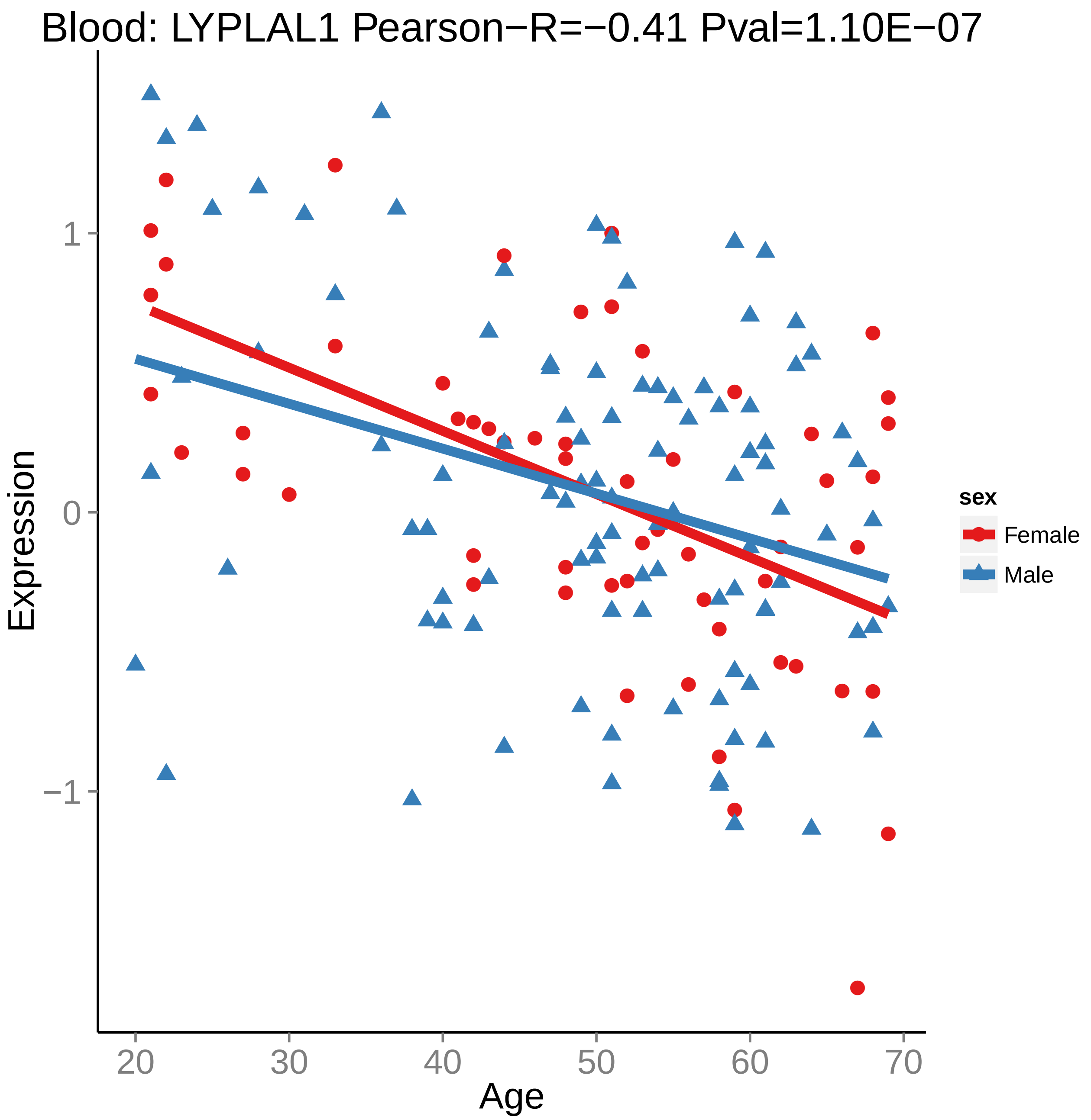

Blood: LYPLAL1 Pearson−R=−0.41 Pval=1.10E−07
Expression
1
0
−1
20
30
40
50
60
70
Age
sex
Female
Male

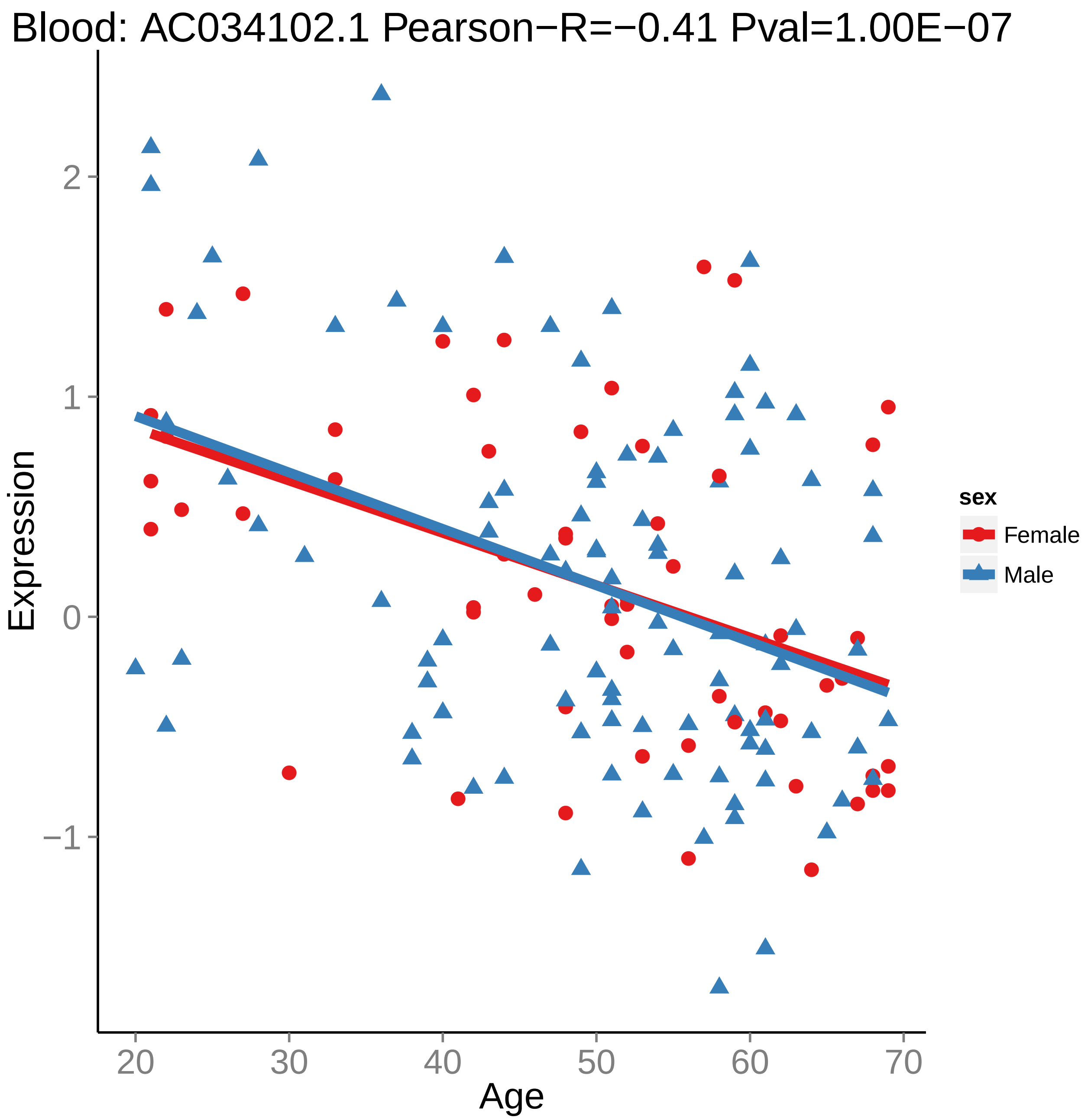
Blood: AC034102.1 Pearson−R=−0.41 Pval=1.00E−07
Expression
2
1
0
−1
20
30
40
50
60
70
Age
sex
Female
Male

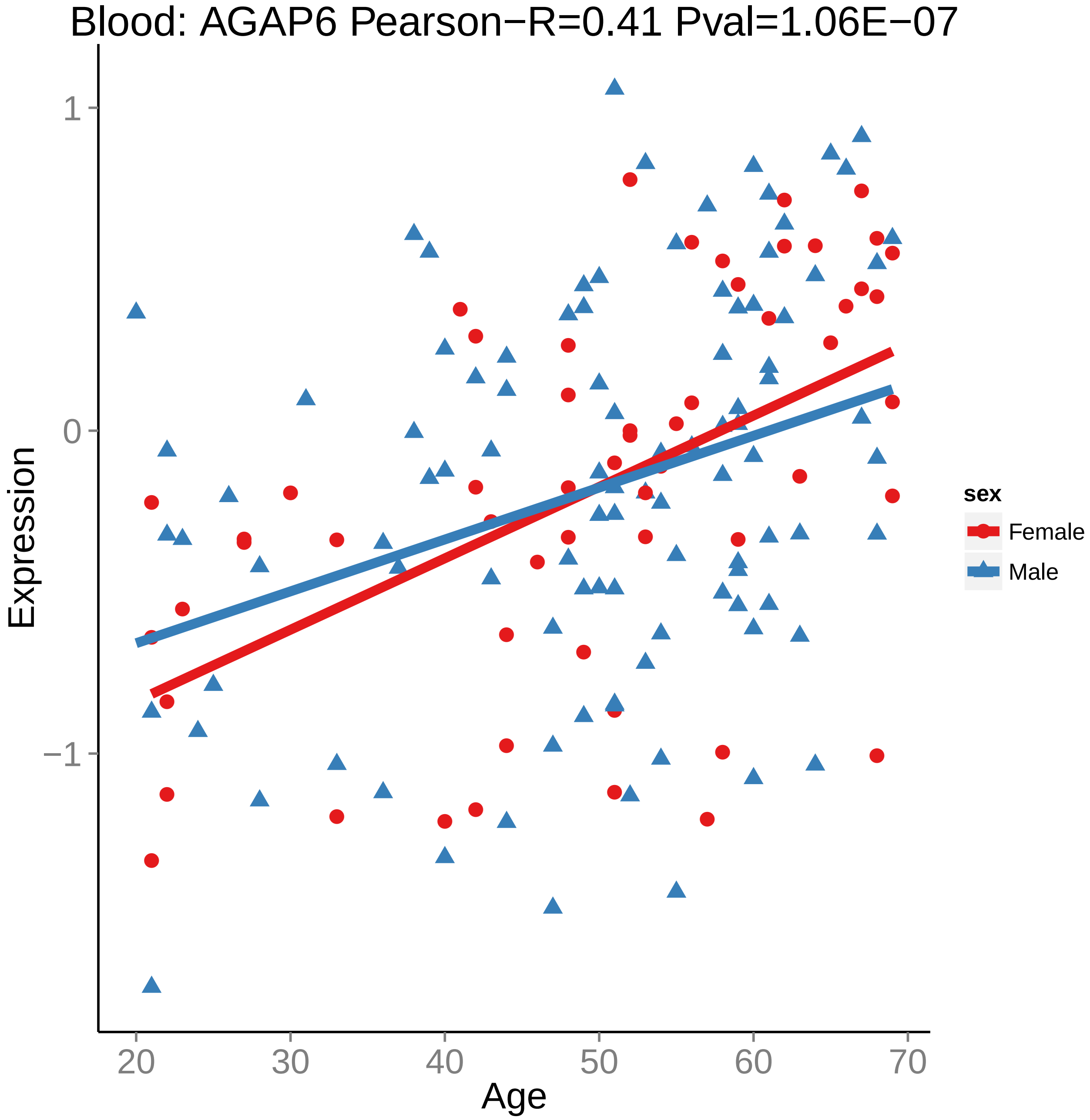

Blood: AGAP6 Pearson−R=0.41 Pval=1.06E−07
Expression
1
0
−1
20
30
40
50
60
70
Age
sex
Female
Male

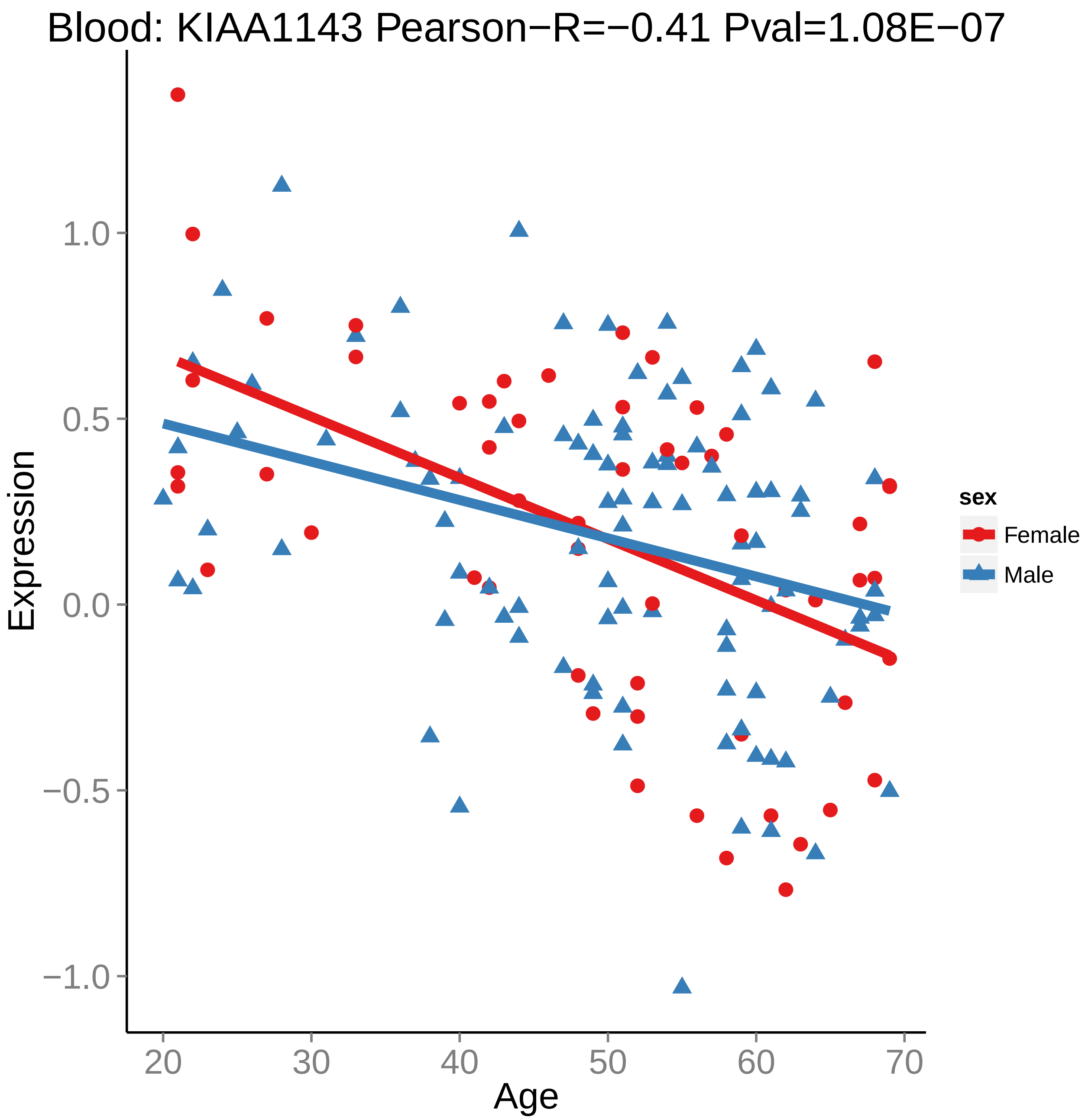

Blood: KIAA1143 Pearson−R=−0.41 Pval=1.08E−07
Expression
Age
1.0
0.5
0.0
−0.5
−1.0
20
30
40
50
60
70
sex
Female
Male

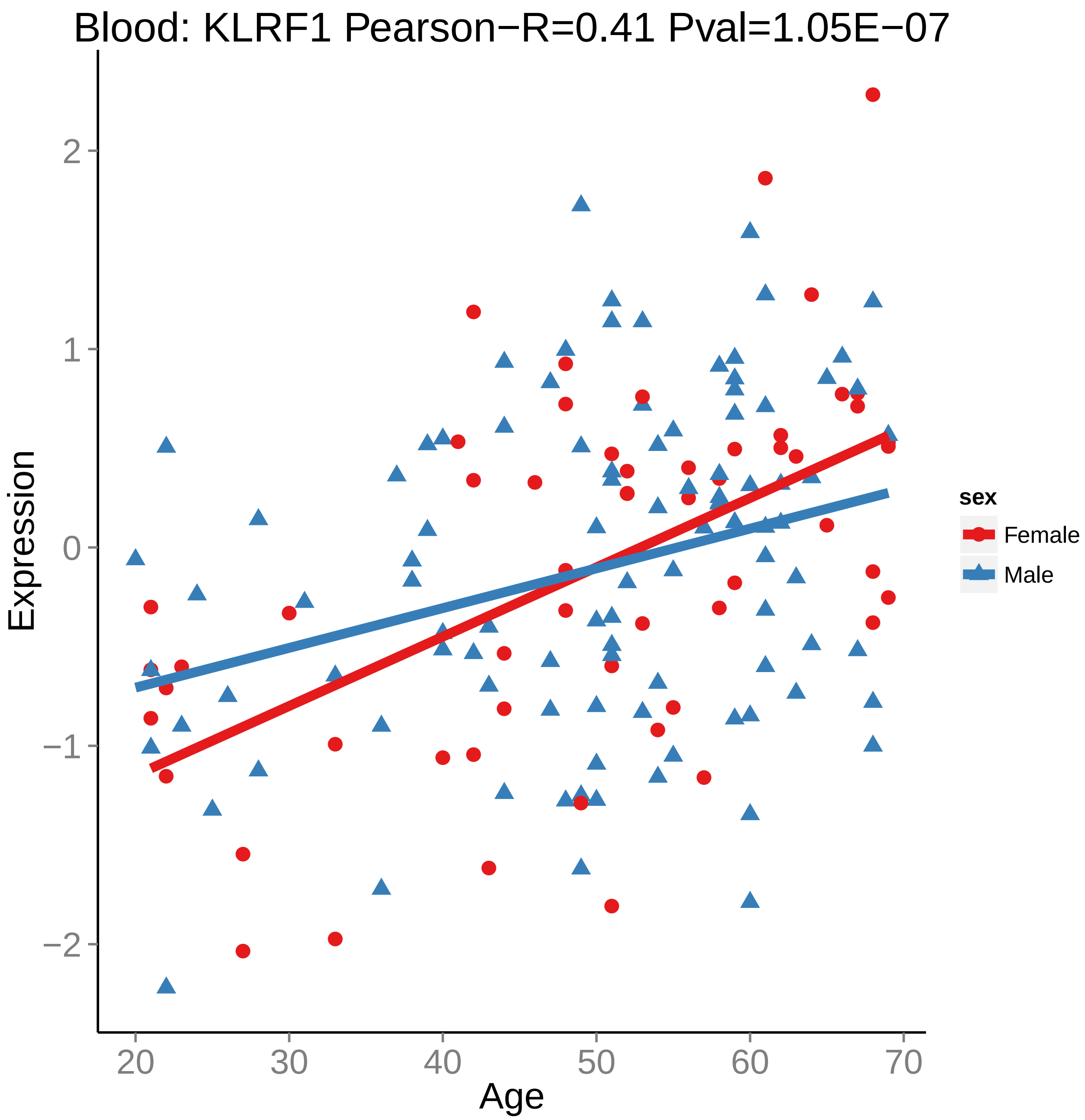

Blood: KLRF1 Pearson−R=0.41 Pval=1.05E−07
Expression
2
1
0
−1
−2
20
30
40
50
60
70
Age
sex
Female
Male

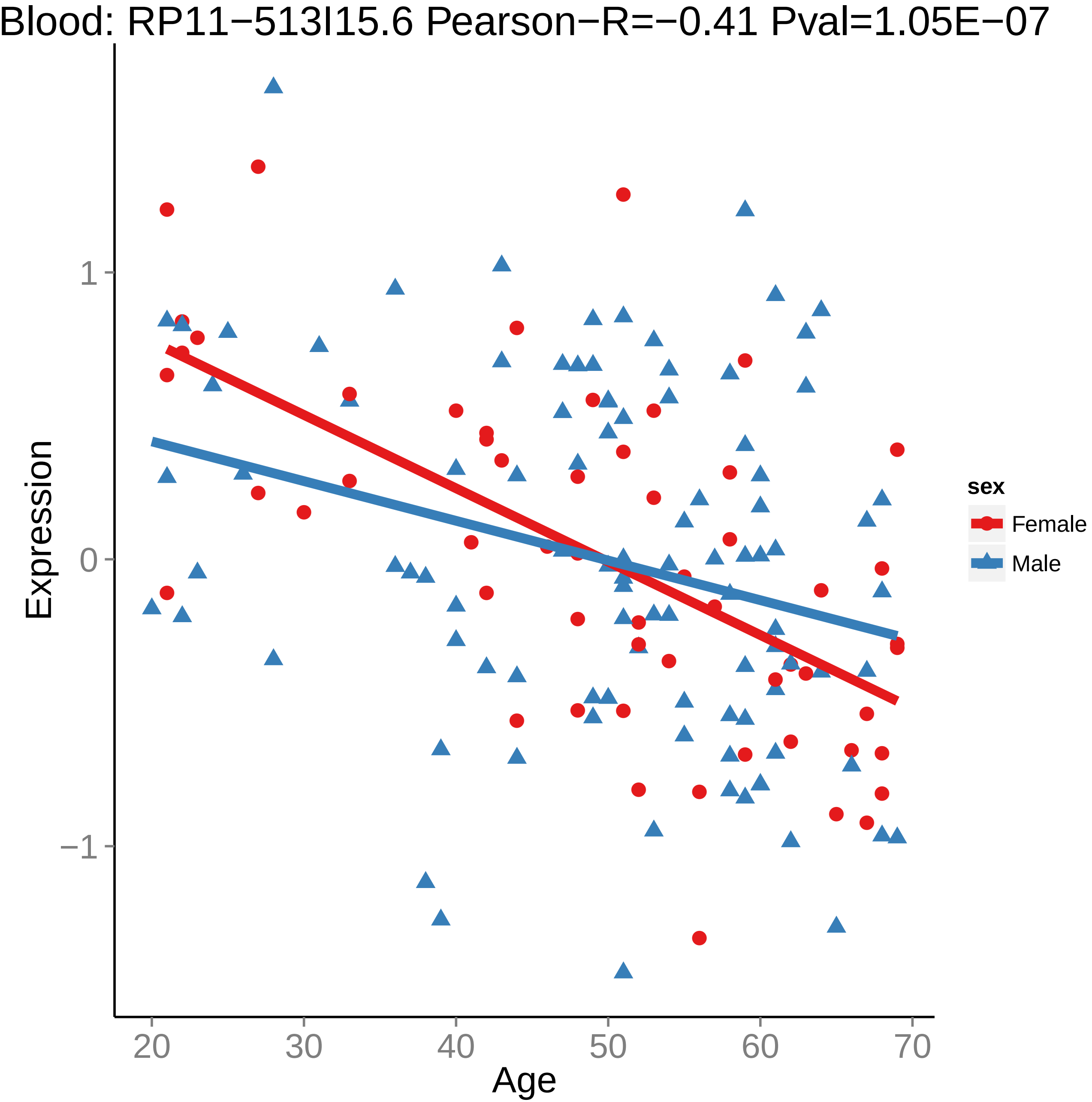

Blood: RP11−513I15.6 Pearson−R=−0.41 Pval=1.05E−07
Expression
1
0
−1
20
30
40
50
60
70
Age
sex
Female
Male

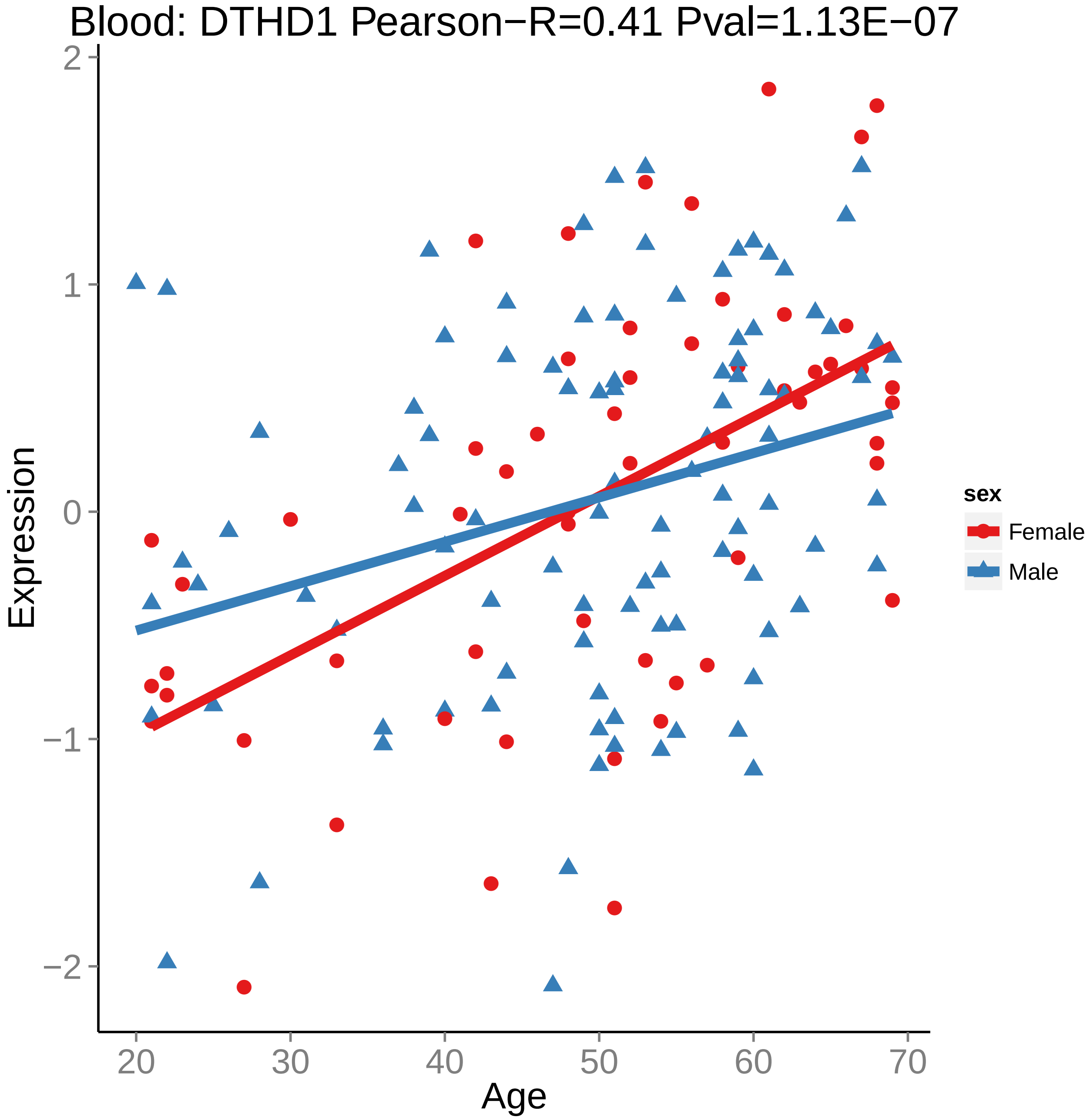

Blood: DTHD1 Pearson−R=0.41 Pval=1.13E−07
Expression
Age
sex
Female
Male

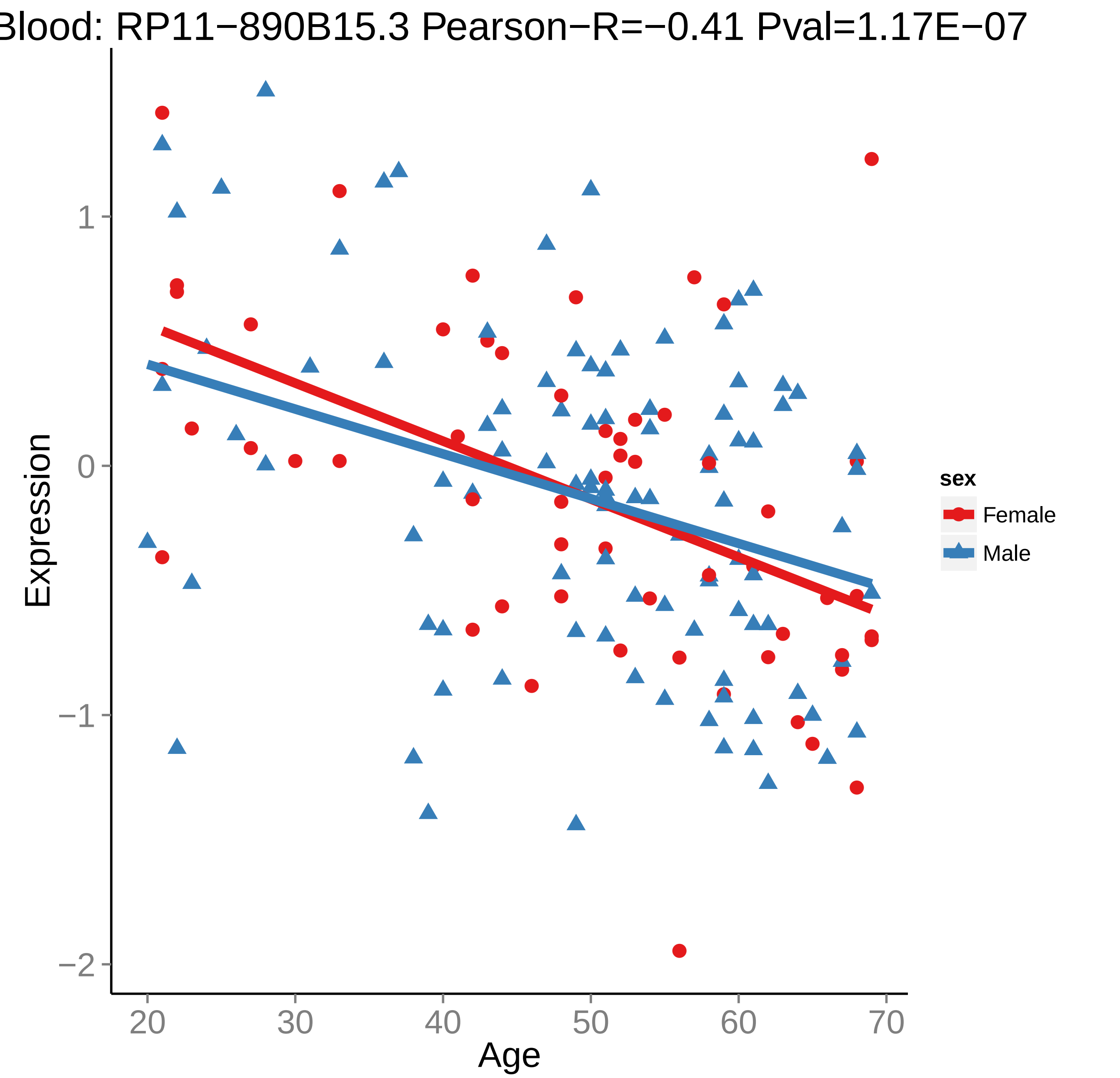
Blood: RP11−890B15.3 Pearson−R=−0.41 Pval=1.17E−07
Expression
1
0
−1
−2
20
30
40
50
60
70
Age
sex
Female
Male

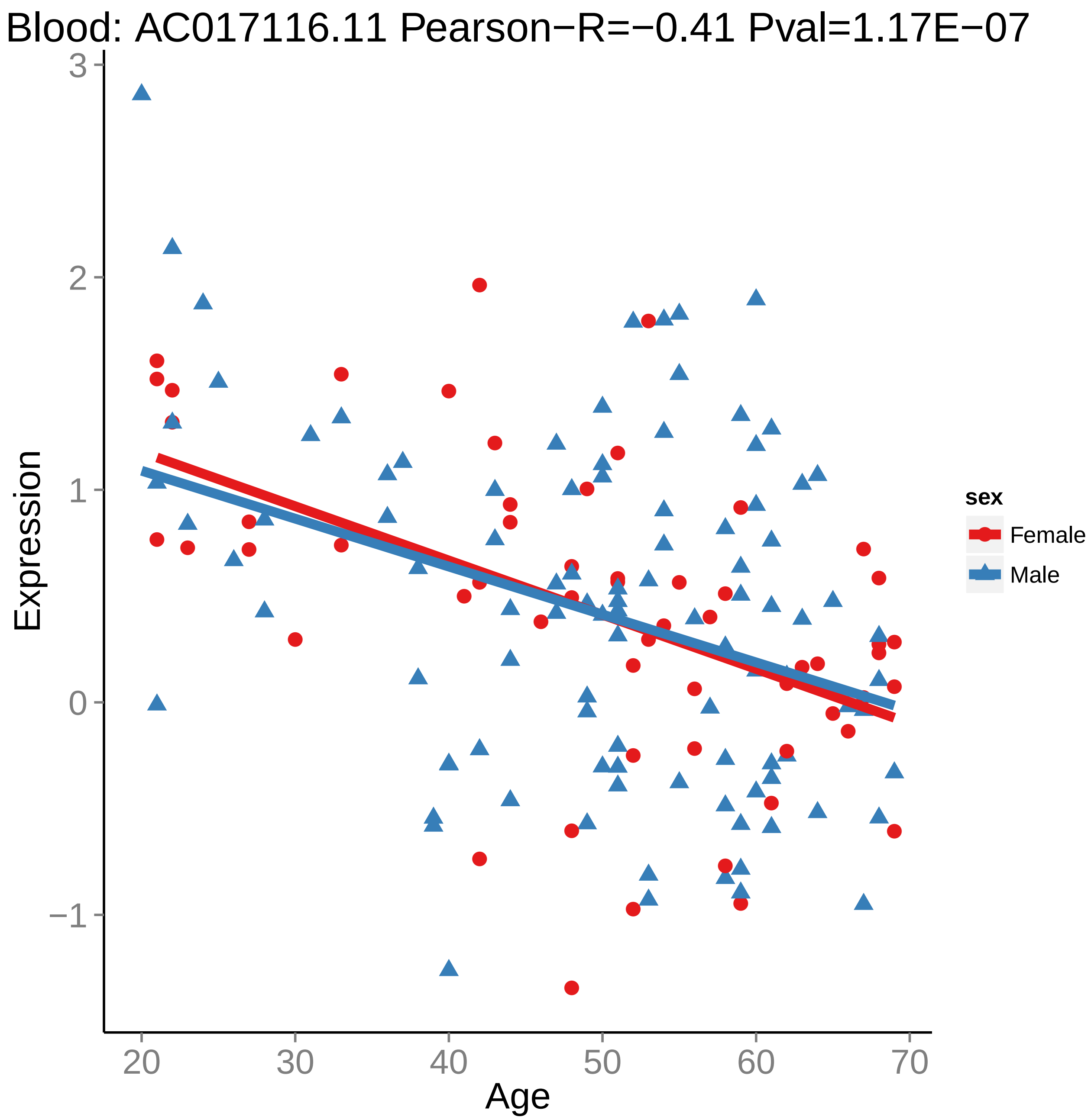

Blood: AC017116.11 Pearson−R=−0.41 Pval=1.17E−07
Expression
Age
3
2
1
0
−1
20
30
40
50
60
70
sex
Female
Male

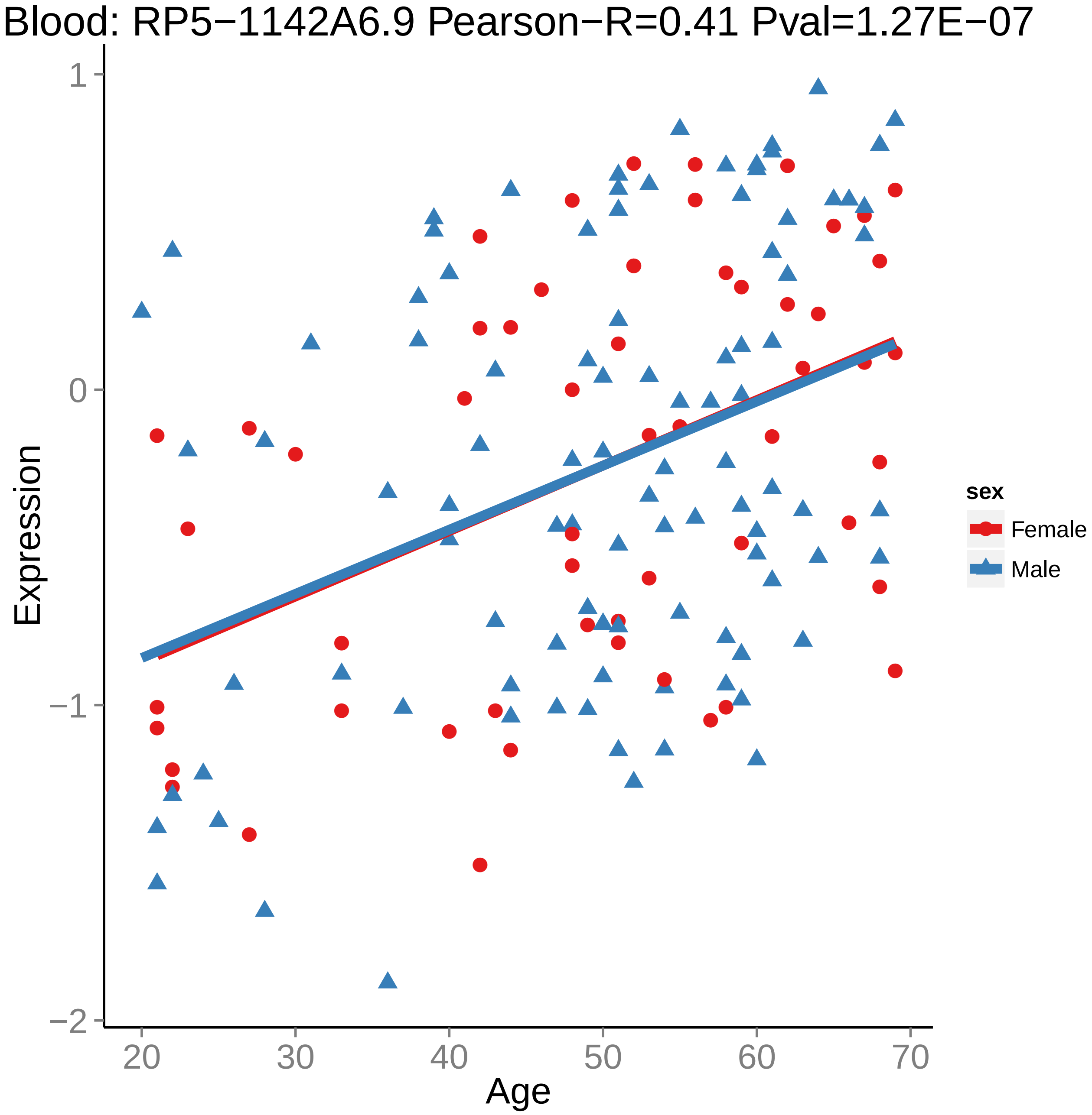

Blood: RP5−1142A6.9 Pearson−R=0.41 Pval=1.27E−07
Expression
1
0
−1
−2
20
30
40
50
60
70
Age
sex
Female
Male

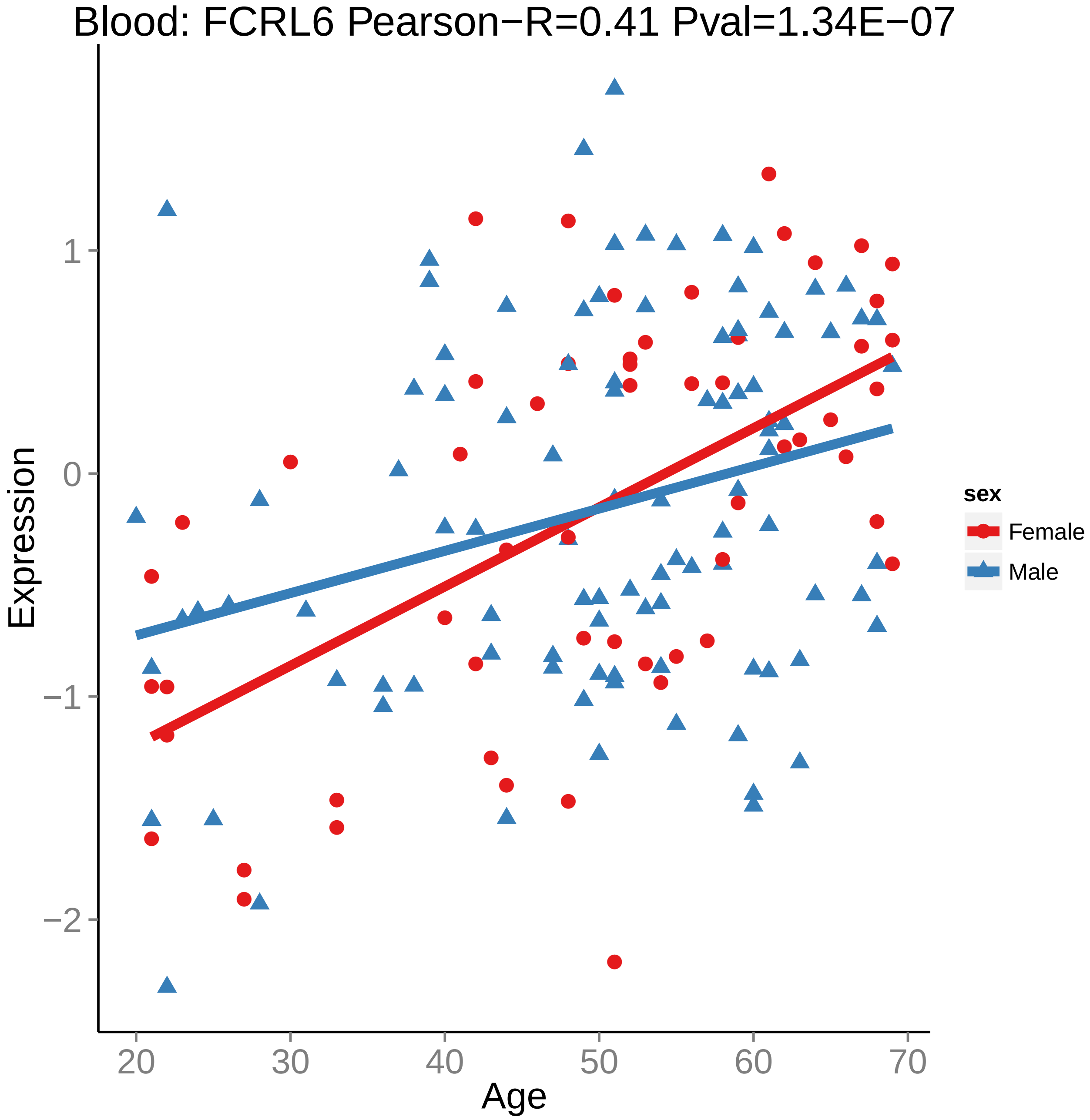

Blood: FCRL6 Pearson−R=0.41 Pval=1.34E−07
Expression
Age
1
0
−1
−2
20
30
40
50
60
70
sex
Female
Male

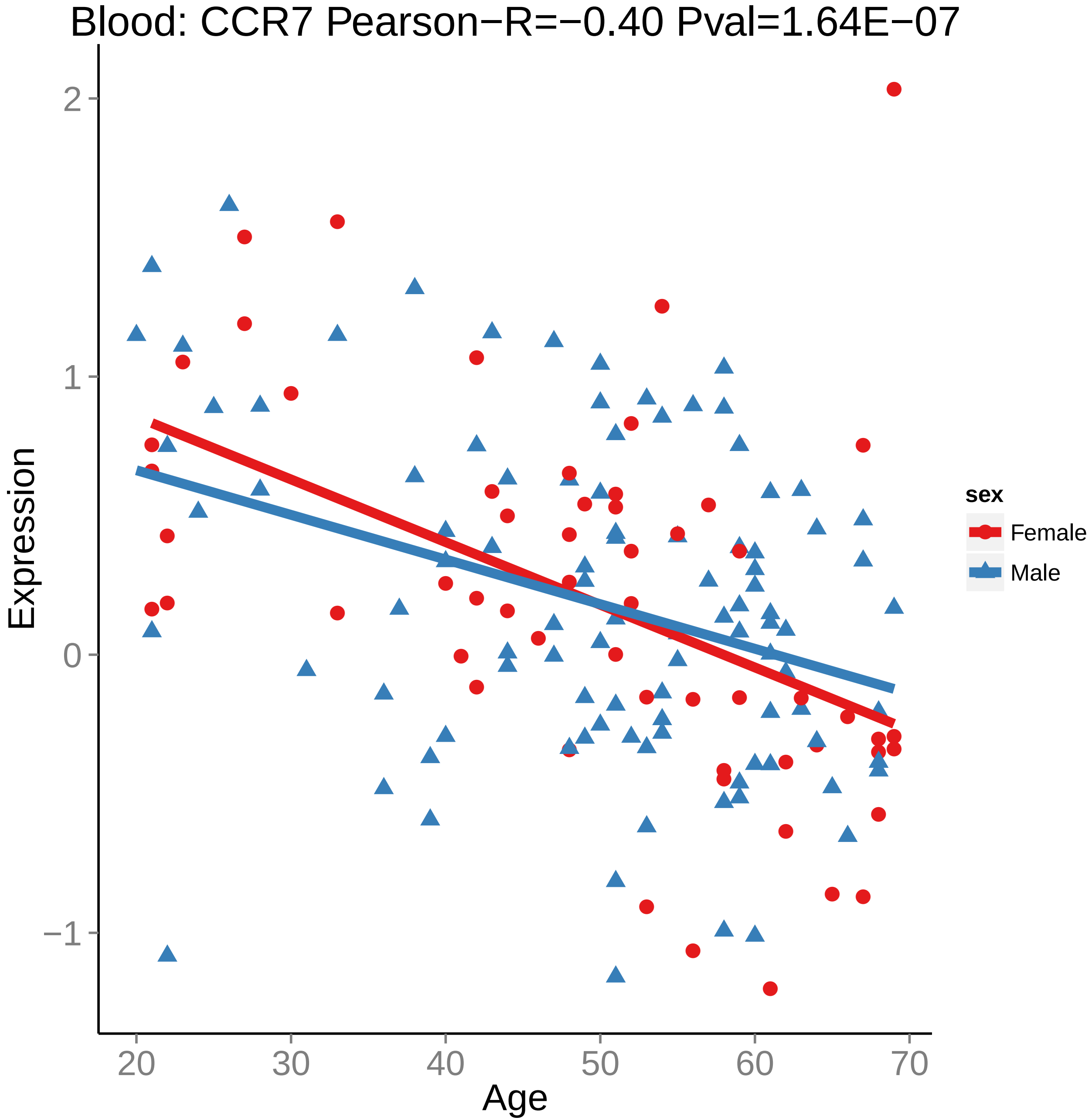

Blood: CCR7 Pearson−R=−0.40 Pval=1.64E−07
Expression
Age
2
1
0
−1
20
30
40
50
60
70
sex
Female
Male

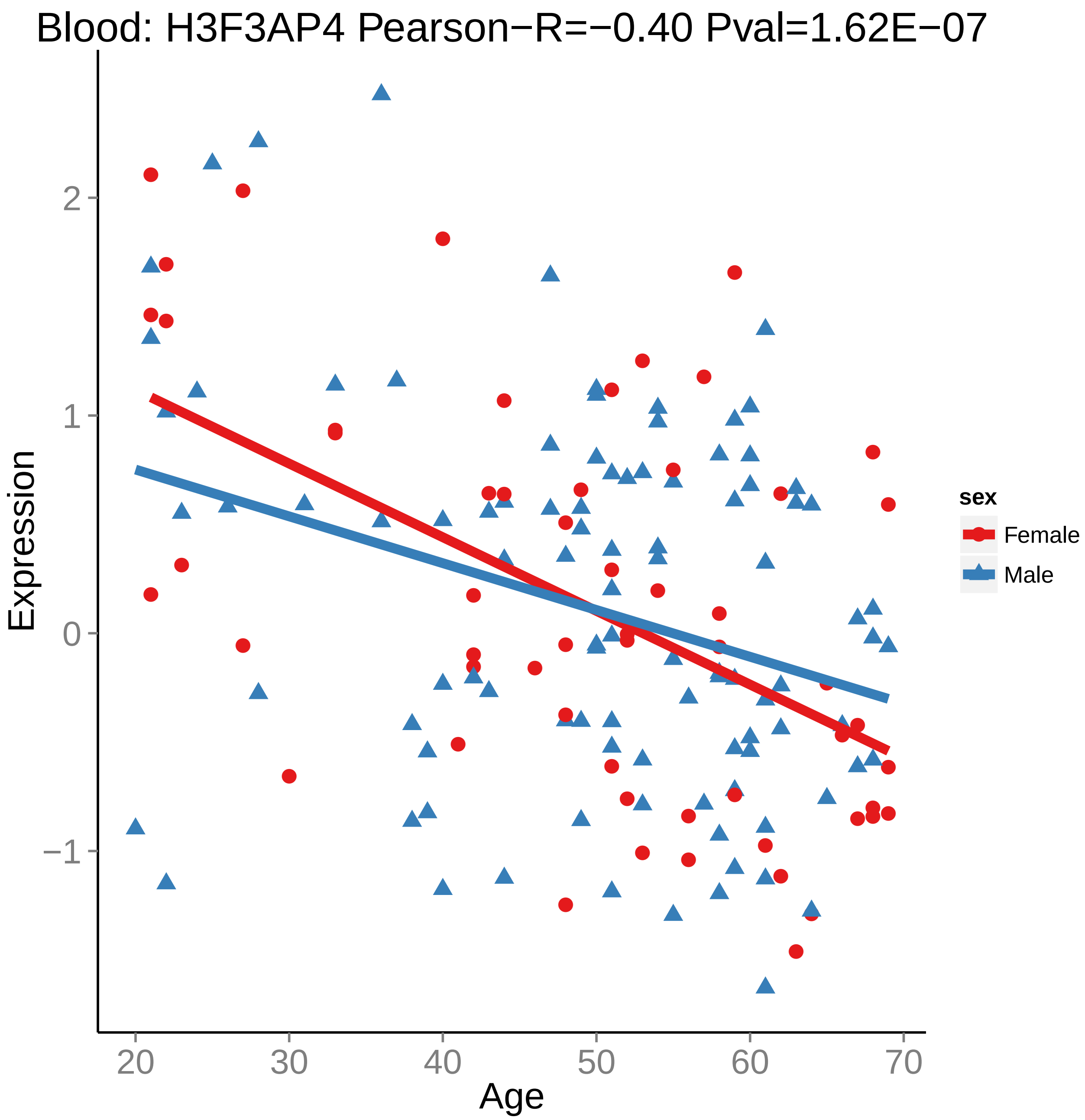

Blood: H3F3AP4 Pearson−R=−0.40 Pval=1.62E−07
Expression
Age
2
1
0
−1
20
30
40
50
60
70
sex
Female
Male

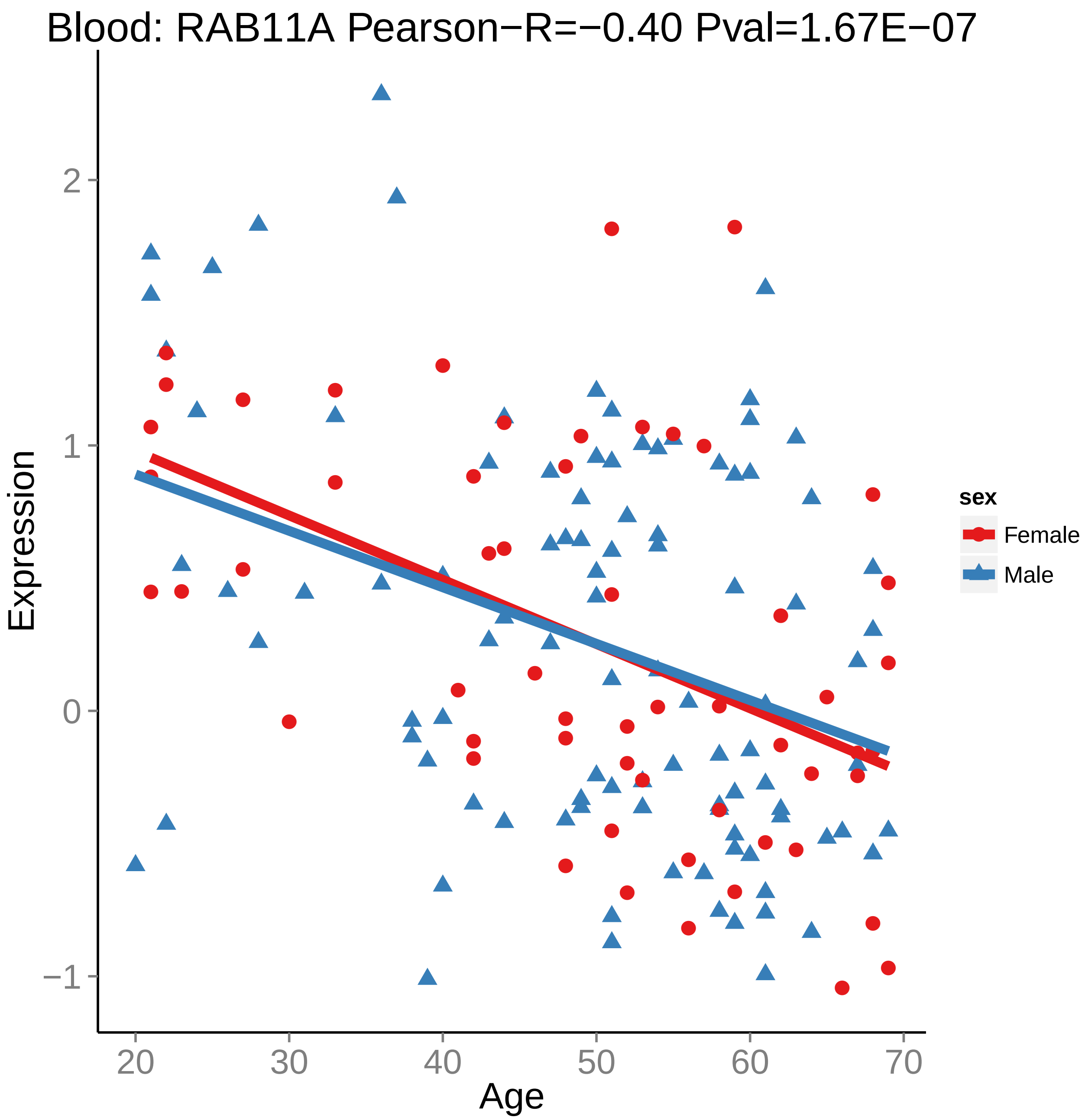
Blood: RAB11A Pearson−R=−0.40 Pval=1.67E−07
Expression
2
1
0
−1
20
30
40
50
60
70
Age
sex
Female
Male

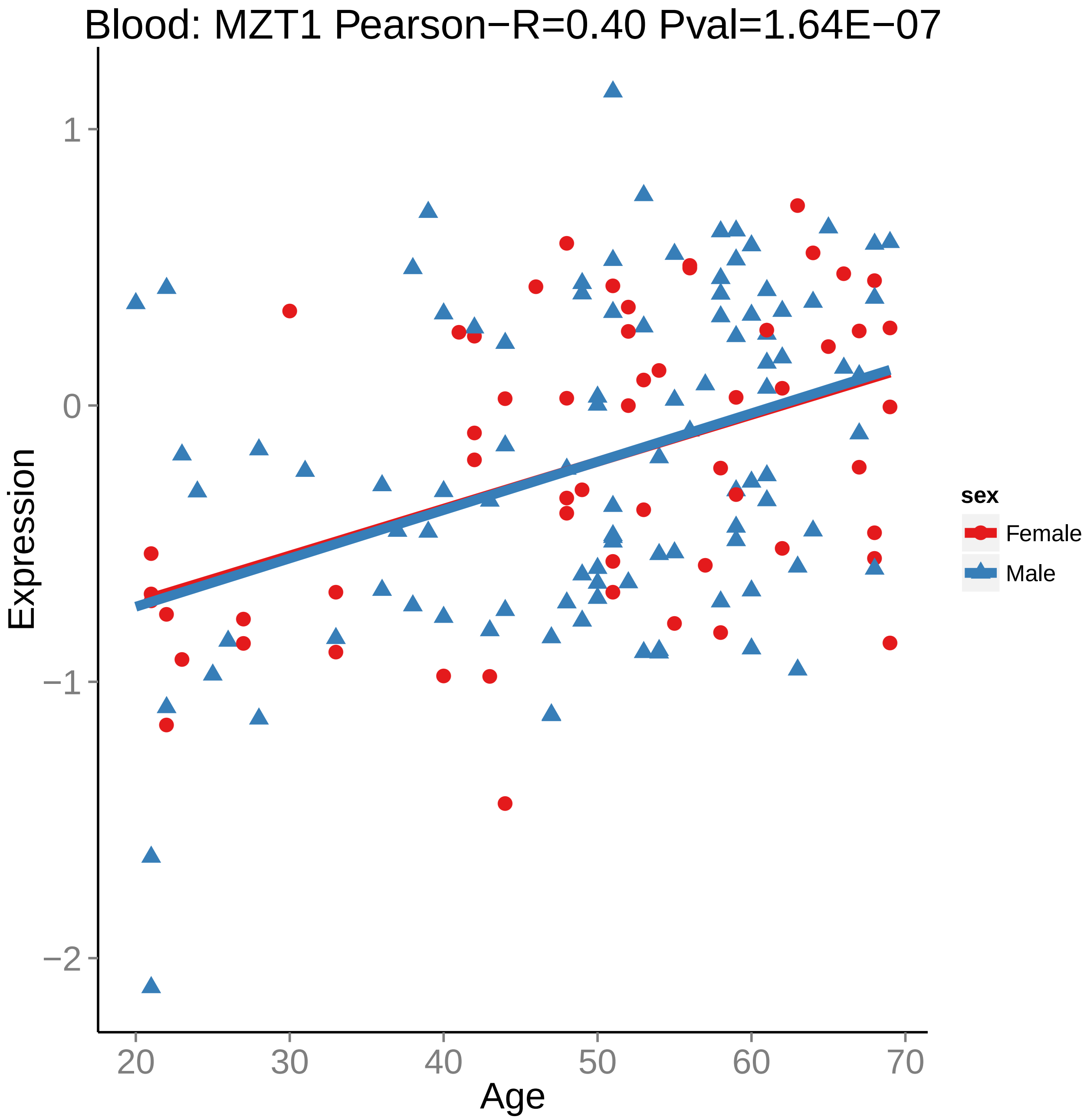

Blood: MZT1 Pearson−R=0.40 Pval=1.64E−07
Expression
Age
1
0
−1
−2
20
30
40
50
60
70
sex
Female
Male

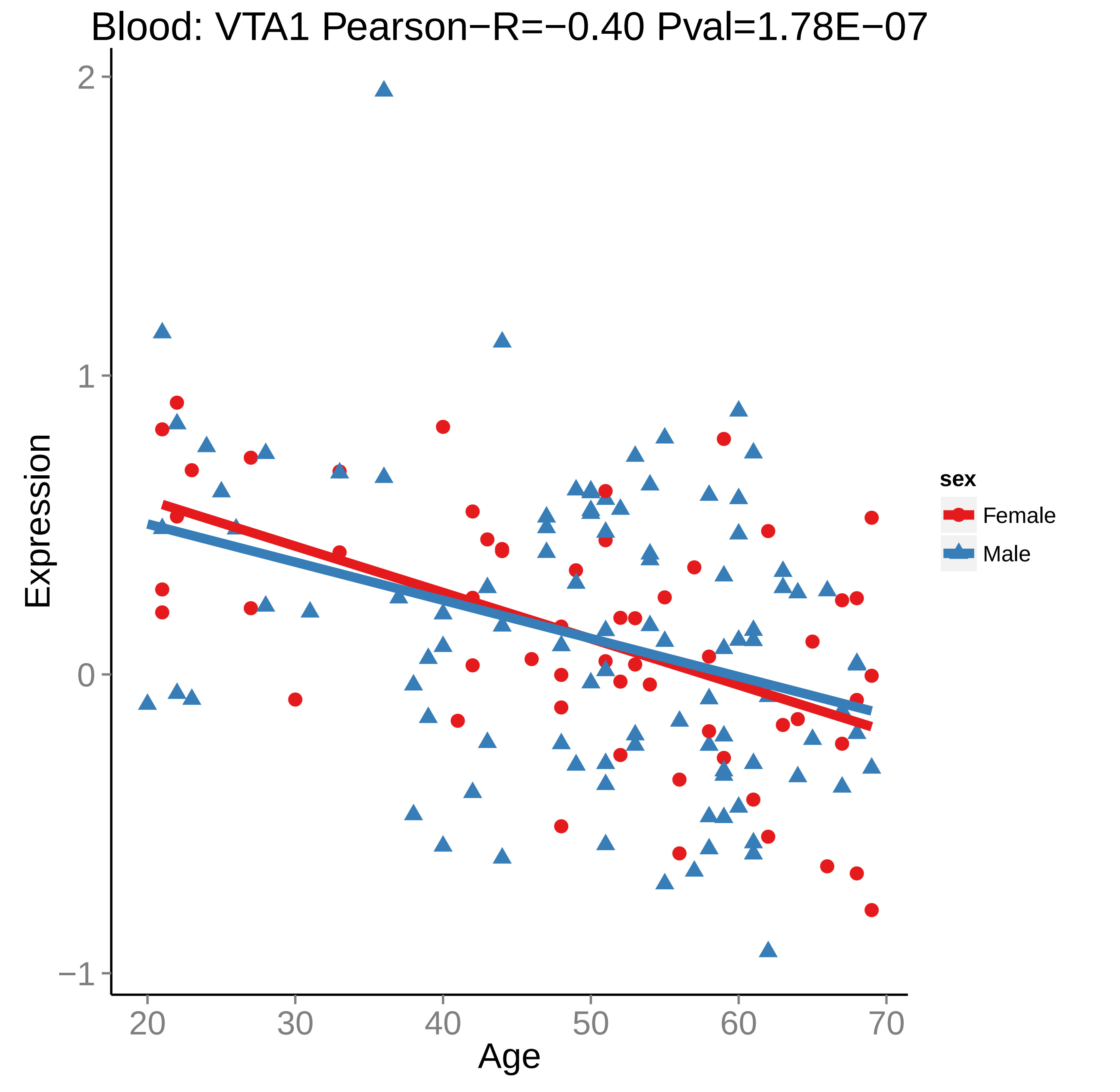

Blood: VTA1 Pearson−R=−0.40 Pval=1.78E−07
Expression
Age
2
1
0
−1
20
30
40
50
60
70
sex
Female
Male

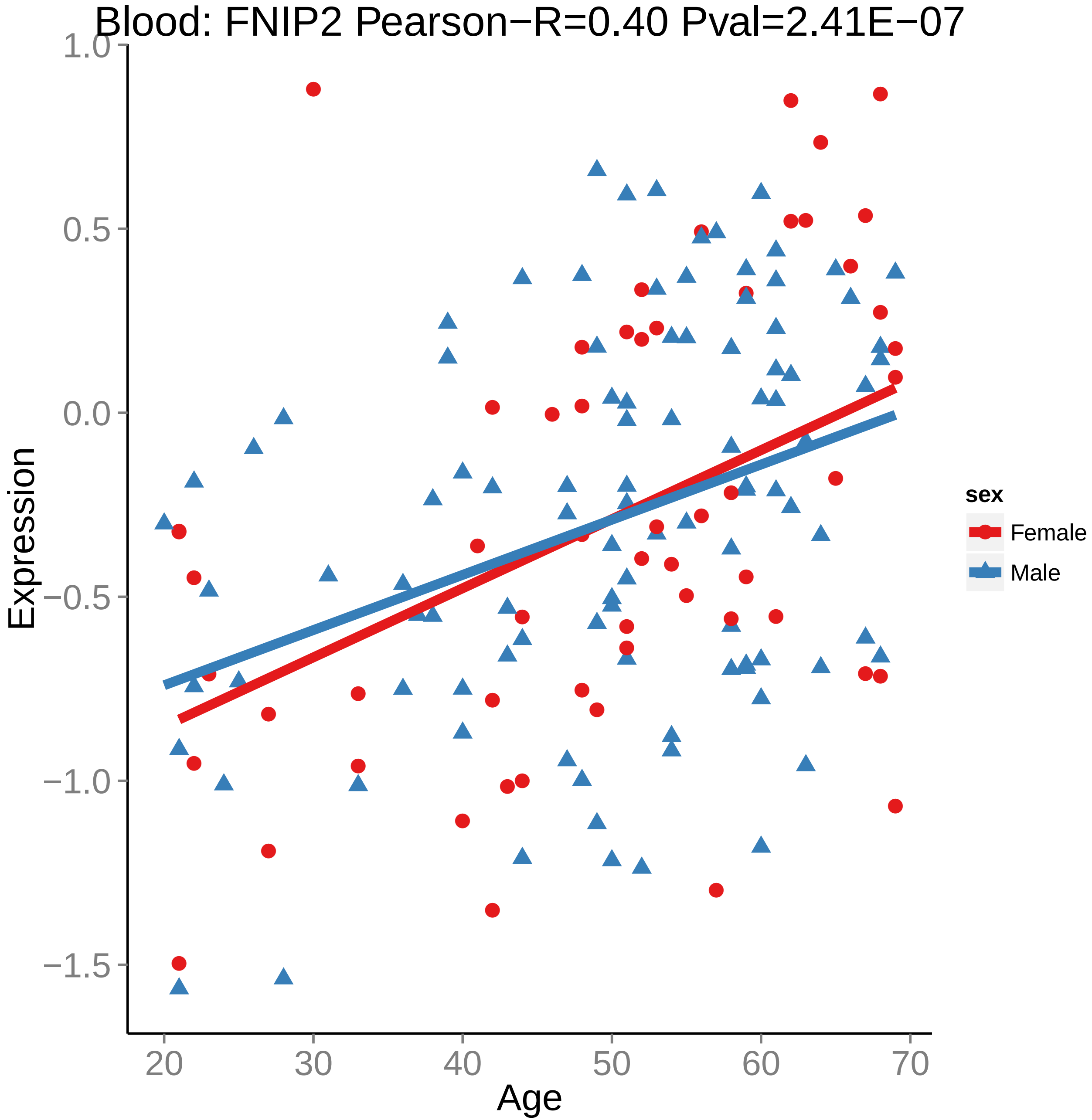

Blood: FNIP2 Pearson−R=0.40 Pval=2.41E−07
Expression
Age
1.0
0.5
0.0
−0.5
−1.0
−1.5
20
30
40
50
60
70
sex
Female
Male

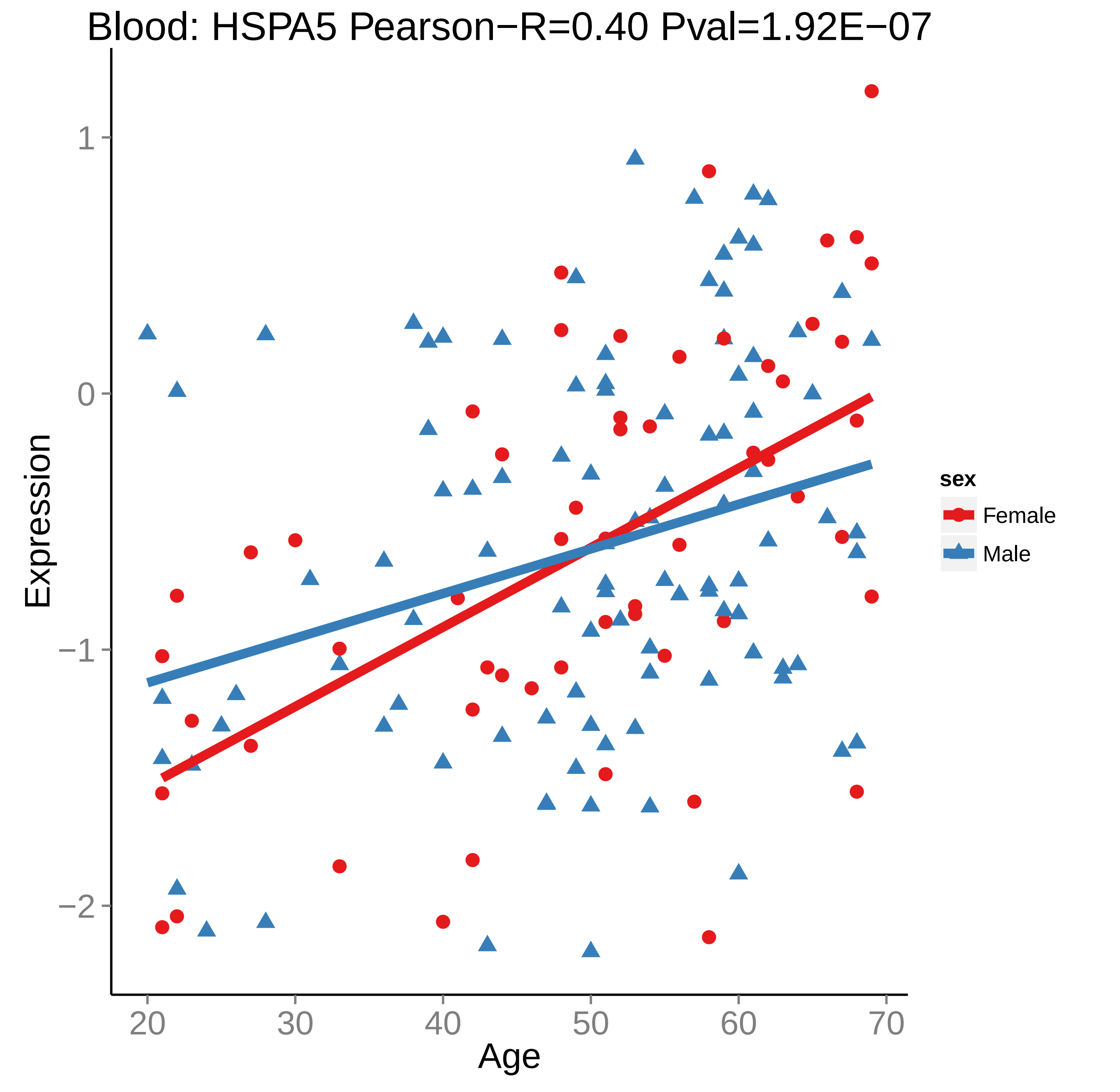

Blood: HSPA5 Pearson−R=0.40 Pval=1.92E−07
Expression
Age
1
0
−1
−2
20
30
40
50
60
70
sex
Female
Male

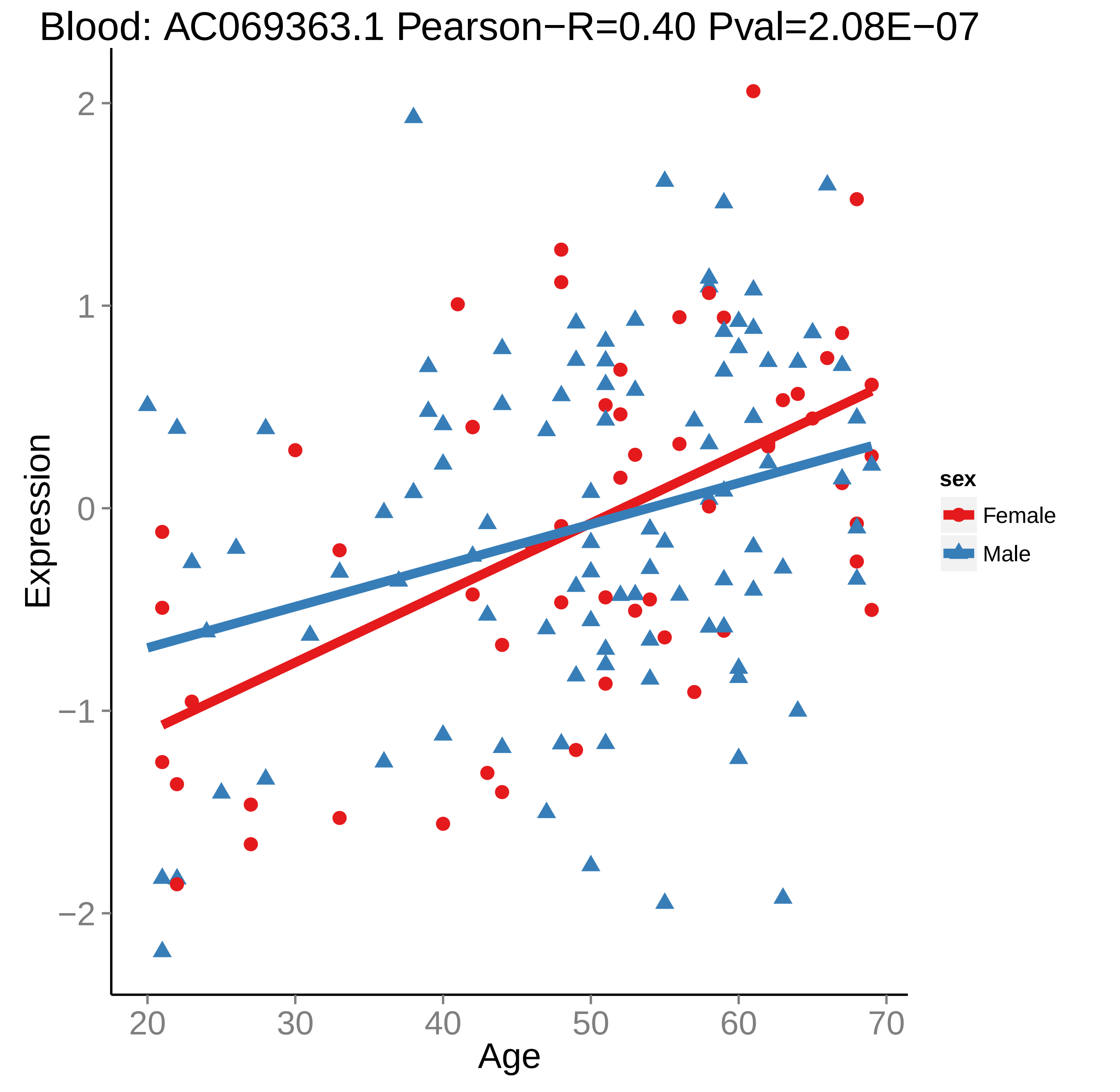

Blood: AC069363.1 Pearson−R=0.40 Pval=2.08E−07
Expression
Age
sex
Female
Male
2
1
0
−1
−2
20
30
40
50
60
70

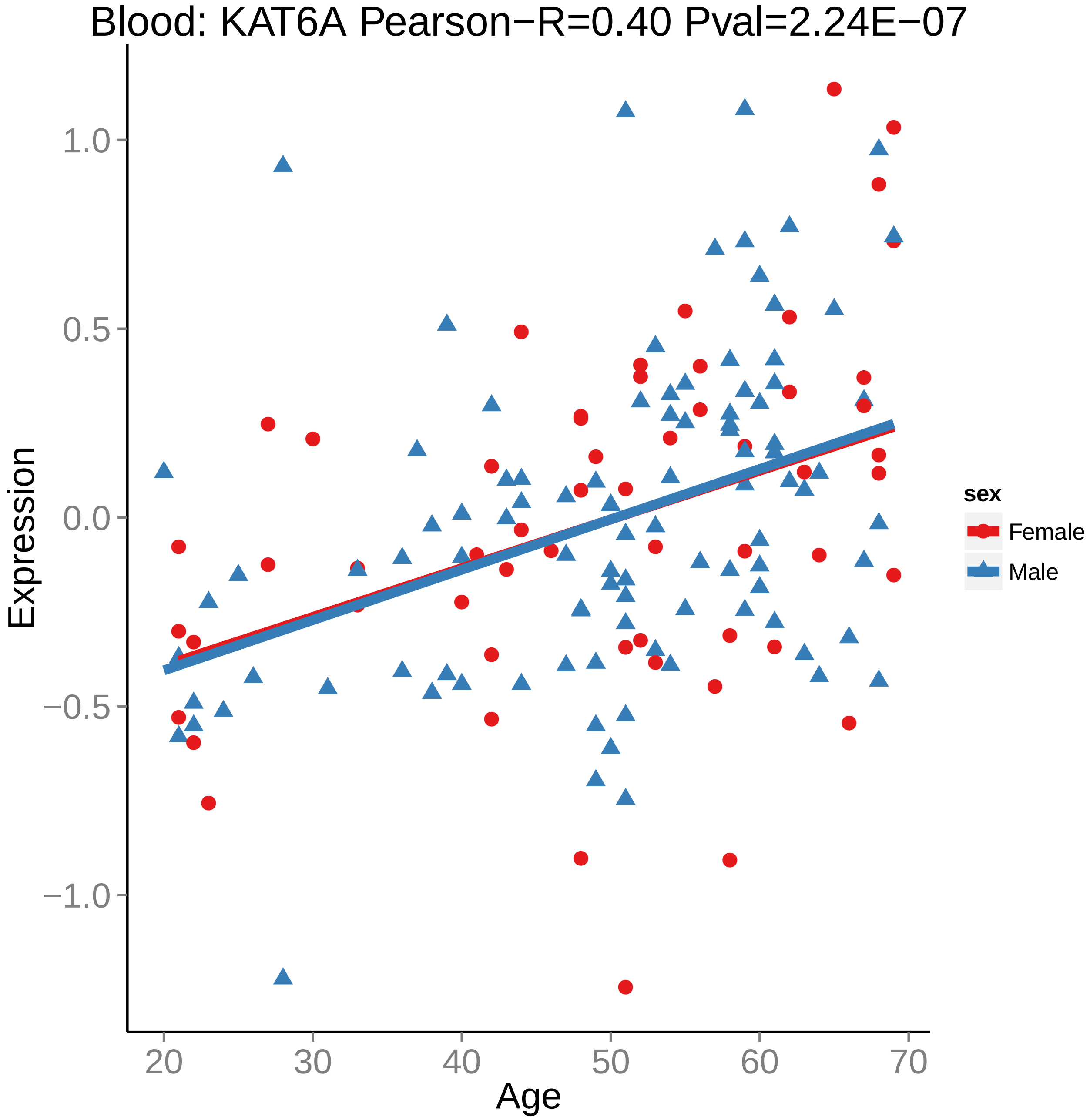
Blood: KAT6A Pearson−R=0.40 Pval=2.24E−07
Expression
1.0
0.5
0.0
−0.5
−1.0
20
30
40
50
60
70
Age
sex
Female
Male

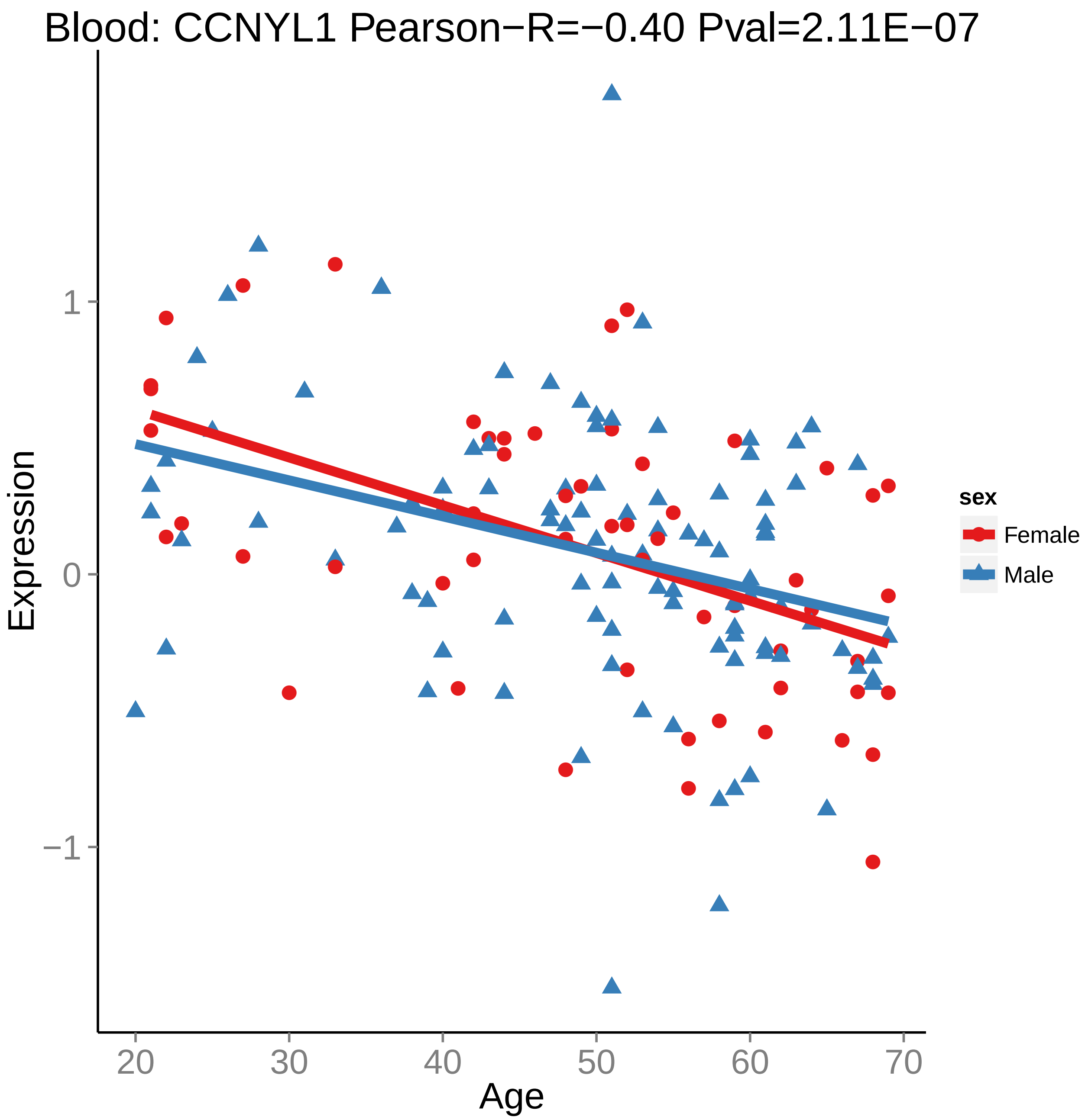

Blood: CCNYL1 Pearson−R=−0.40 Pval=2.11E−07
Expression
Age
1
0
−1
20
30
40
50
60
70
sex
Female
Male

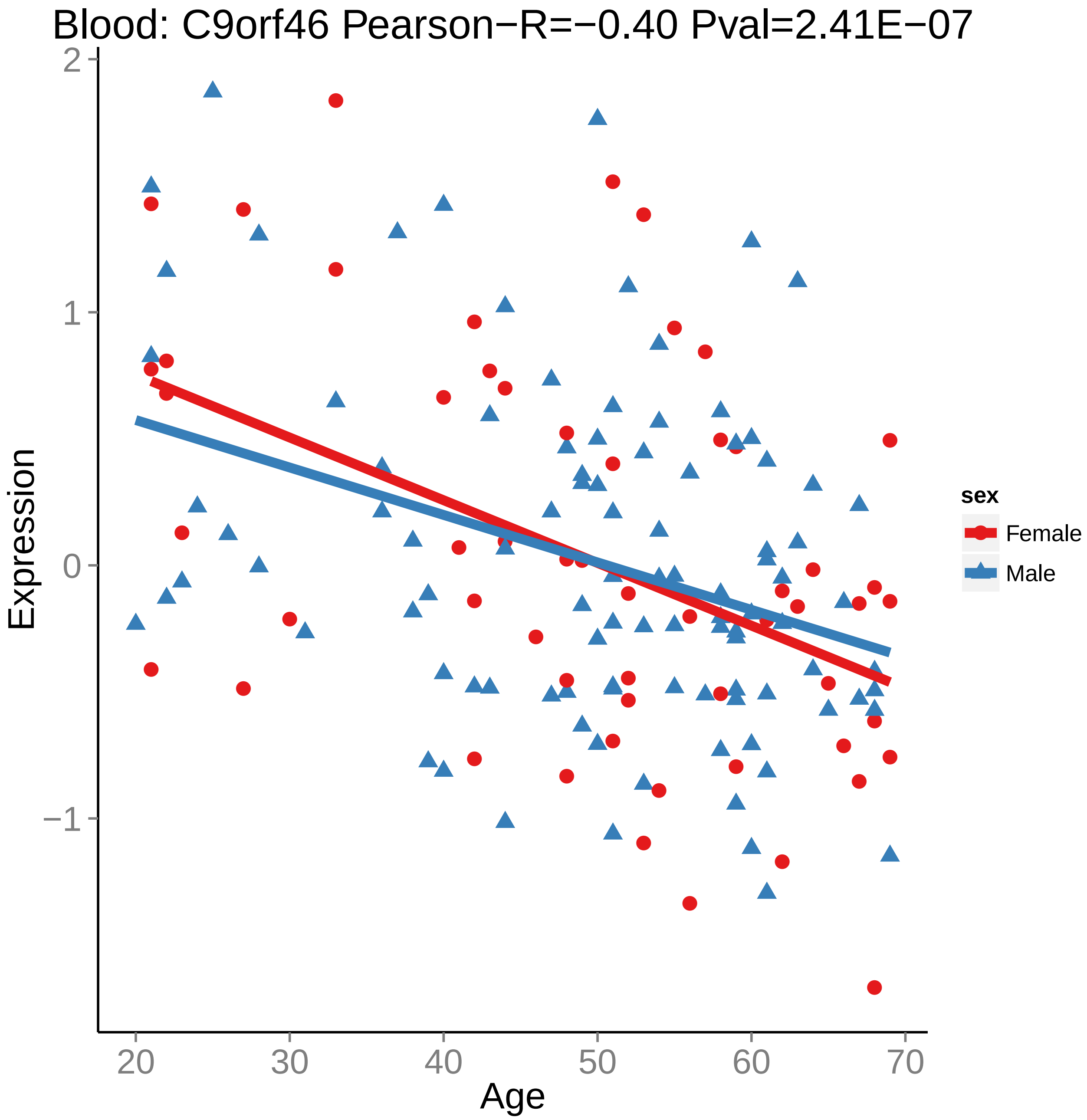

Blood: C9orf46 Pearson−R=−0.40 Pval=2.41E−07
Expression
Age
sex
Female
Male

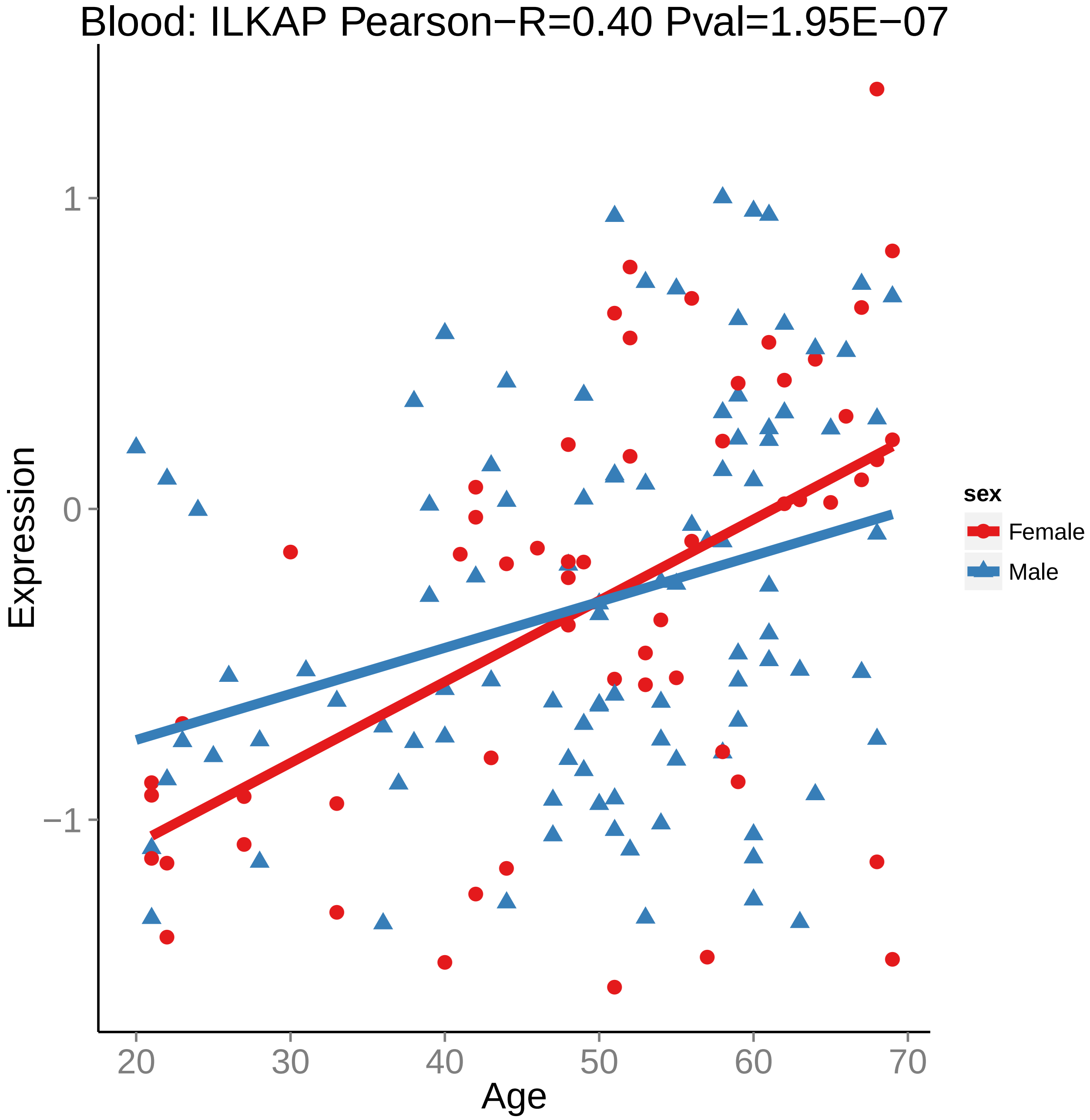

Blood: ILKAP Pearson−R=0.40 Pval=1.95E−07
Expression
Age
1
0
−1
20
30
40
50
60
70
sex
Female
Male

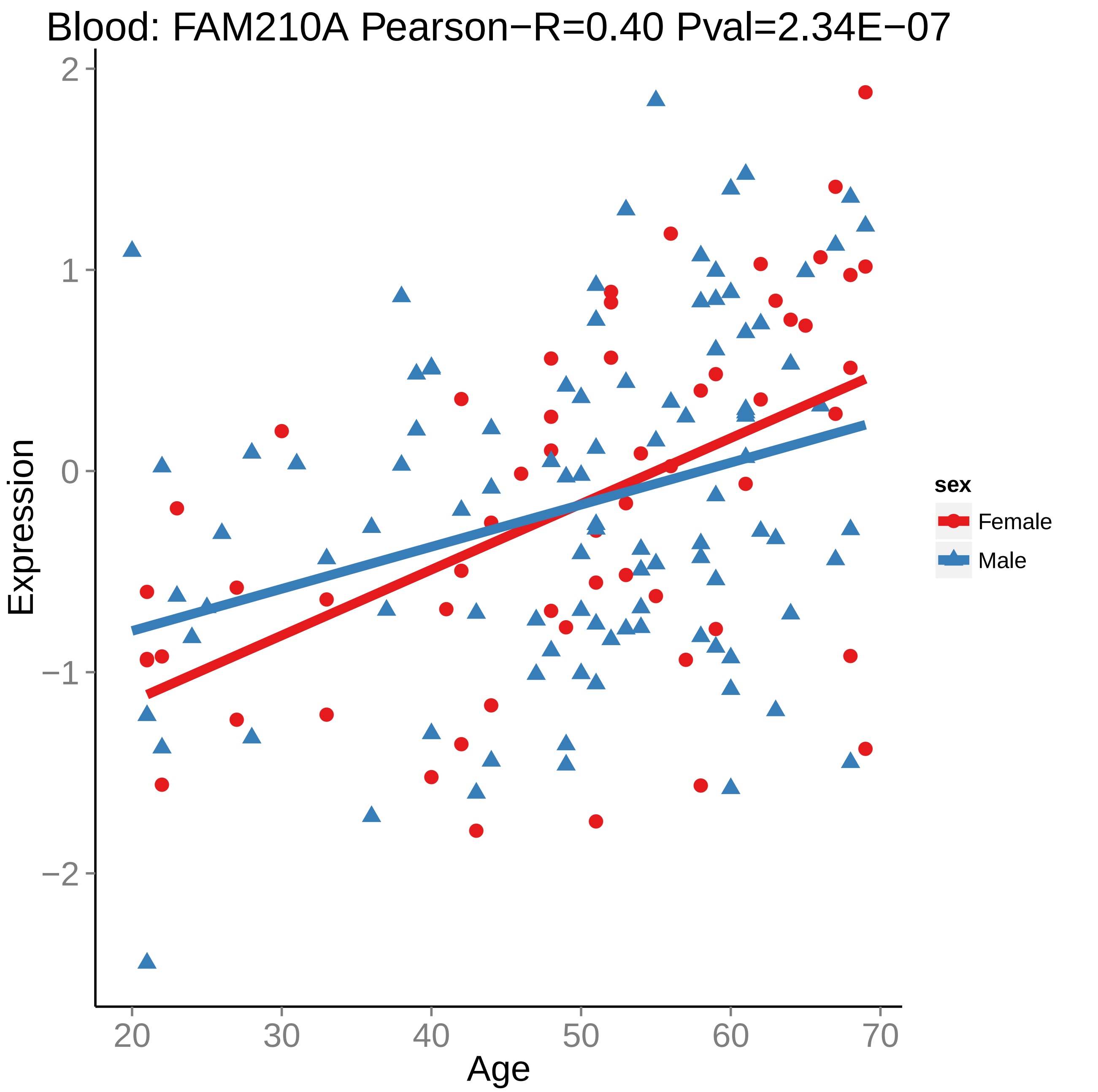
Blood: FAM210A Pearson−R=0.40 Pval=2.34E−07
Expression
Age
2
1
0
−1
−2
20
30
40
50
60
70
sex
Female
Male

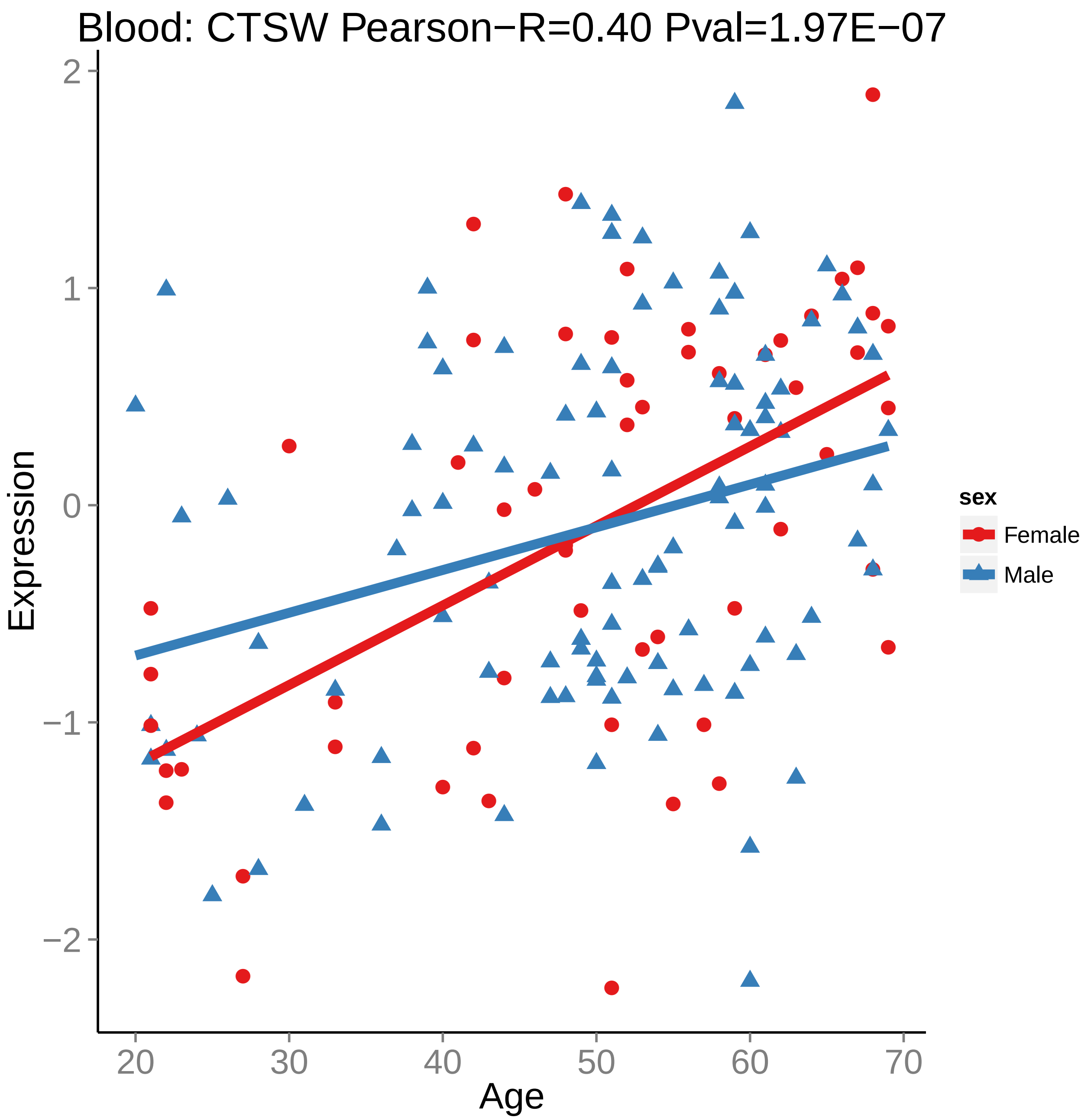

Blood: CTSW Pearson−R=0.40 Pval=1.97E−07
Expression
Age
2
1
0
−1
−2
20
30
40
50
60
70
sex
Female
Male

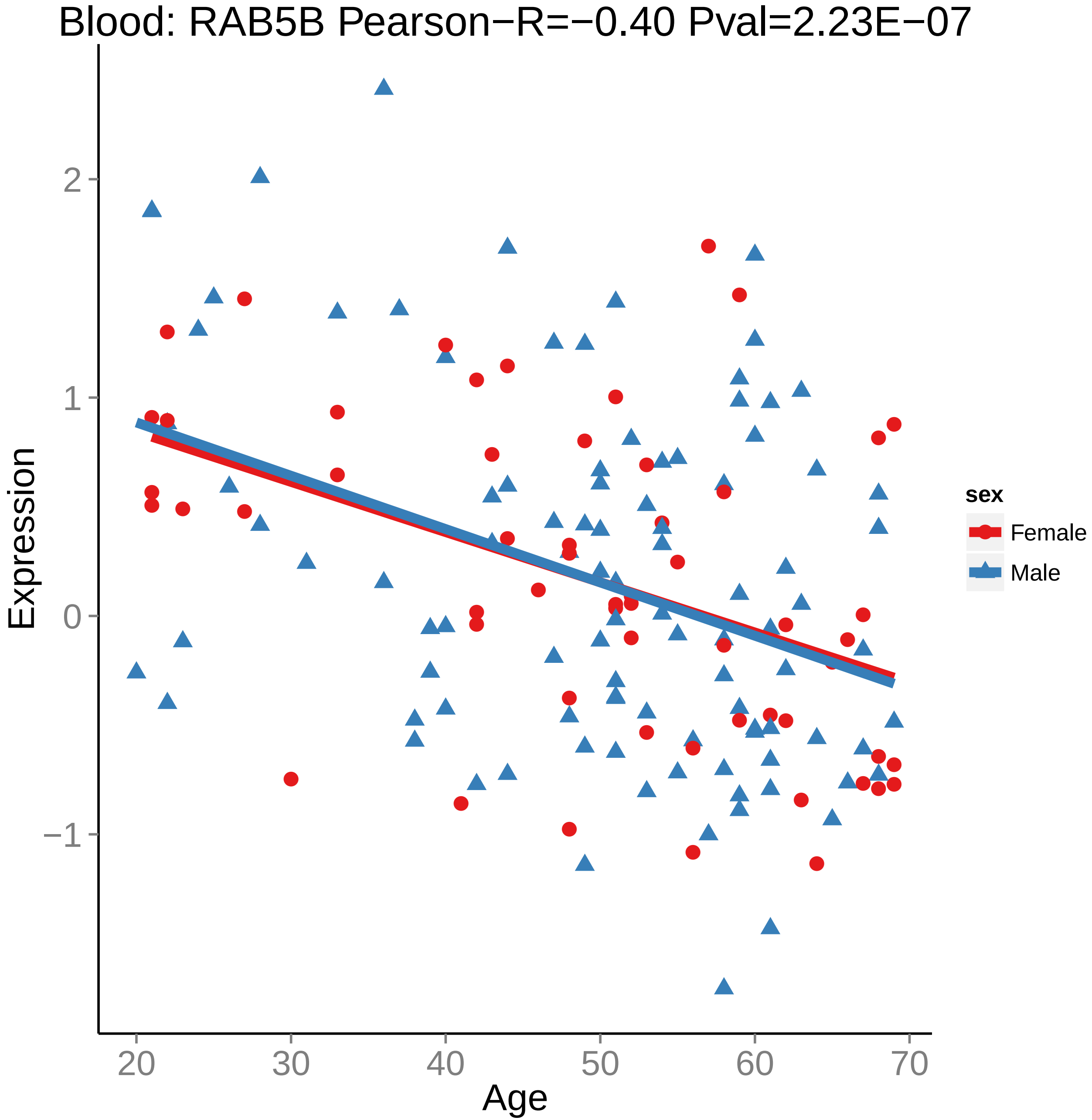
Blood: RAB5B Pearson−R=−0.40 Pval=2.23E−07
Expression
2
1
0
−1
20
30
40
50
60
70
Age
sex
Female
Male

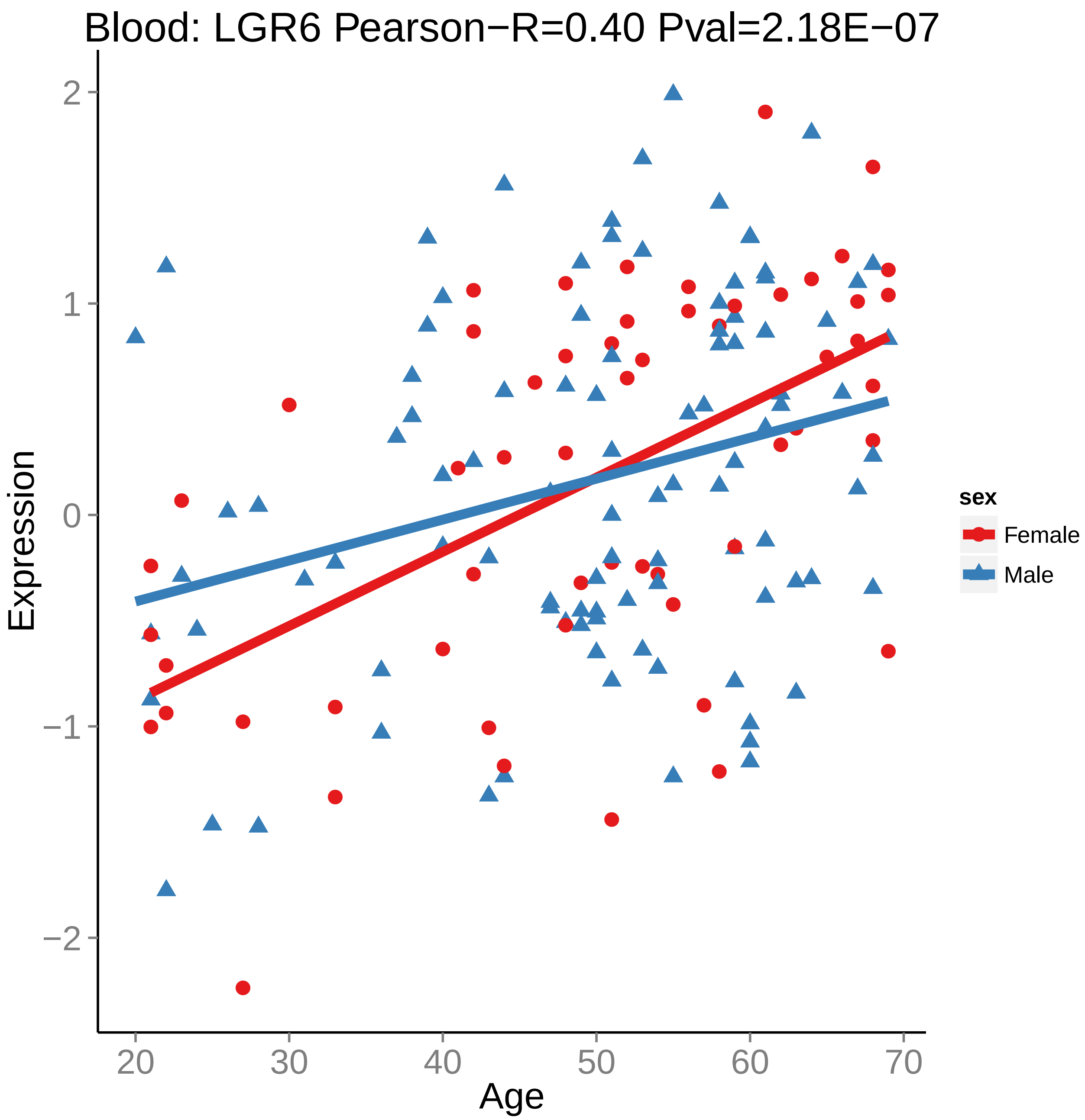

Blood: LGR6 Pearson−R=0.40 Pval=2.18E−07
Expression
Age
2
1
0
−1
−2
20
30
40
50
60
70
sex
Female
Male

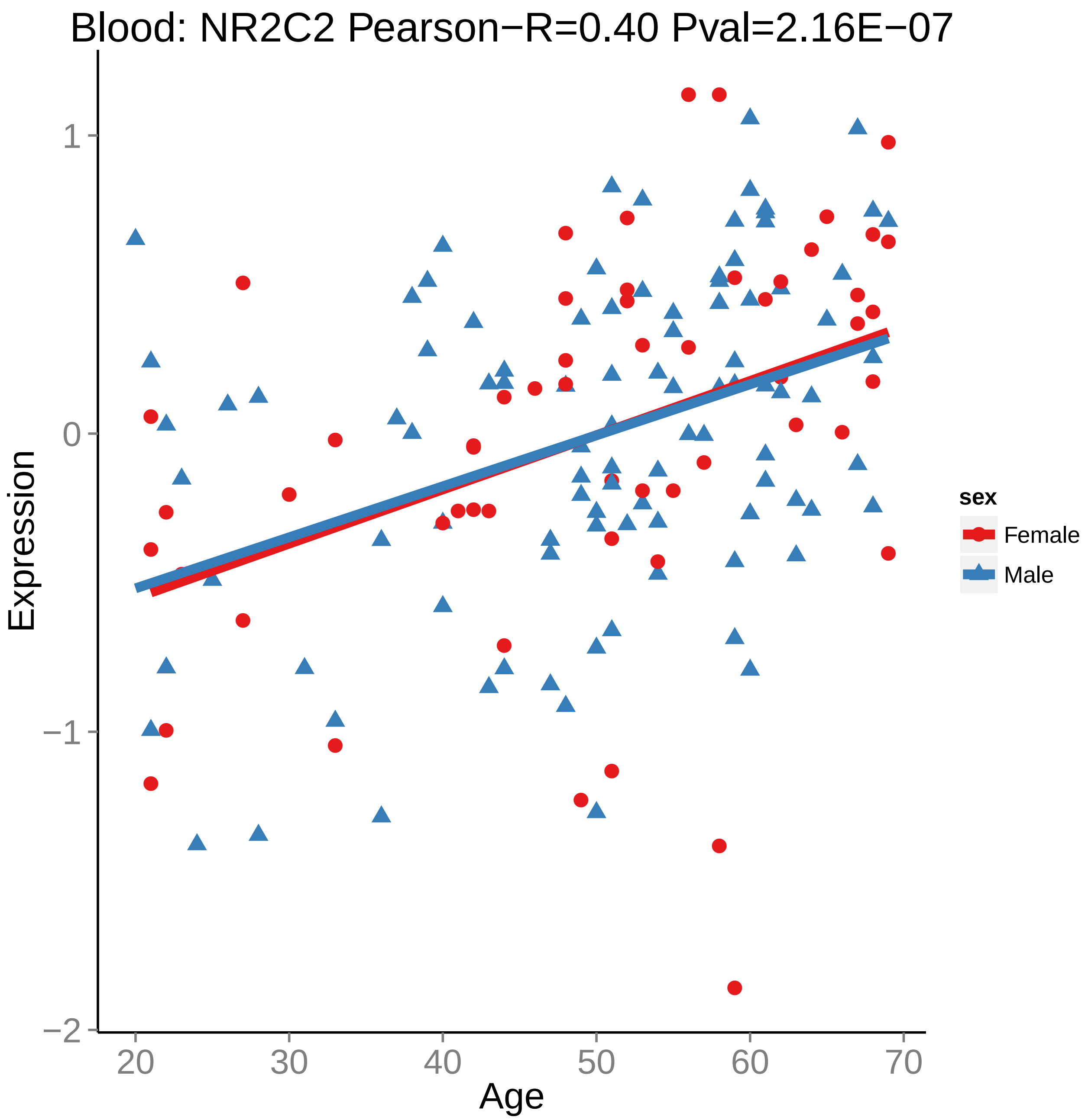

Blood: NR2C2 Pearson−R=0.40 Pval=2.16E−07
Expression
1
0
−1
−2
20
30
40
50
60
70
Age
sex
Female
Male

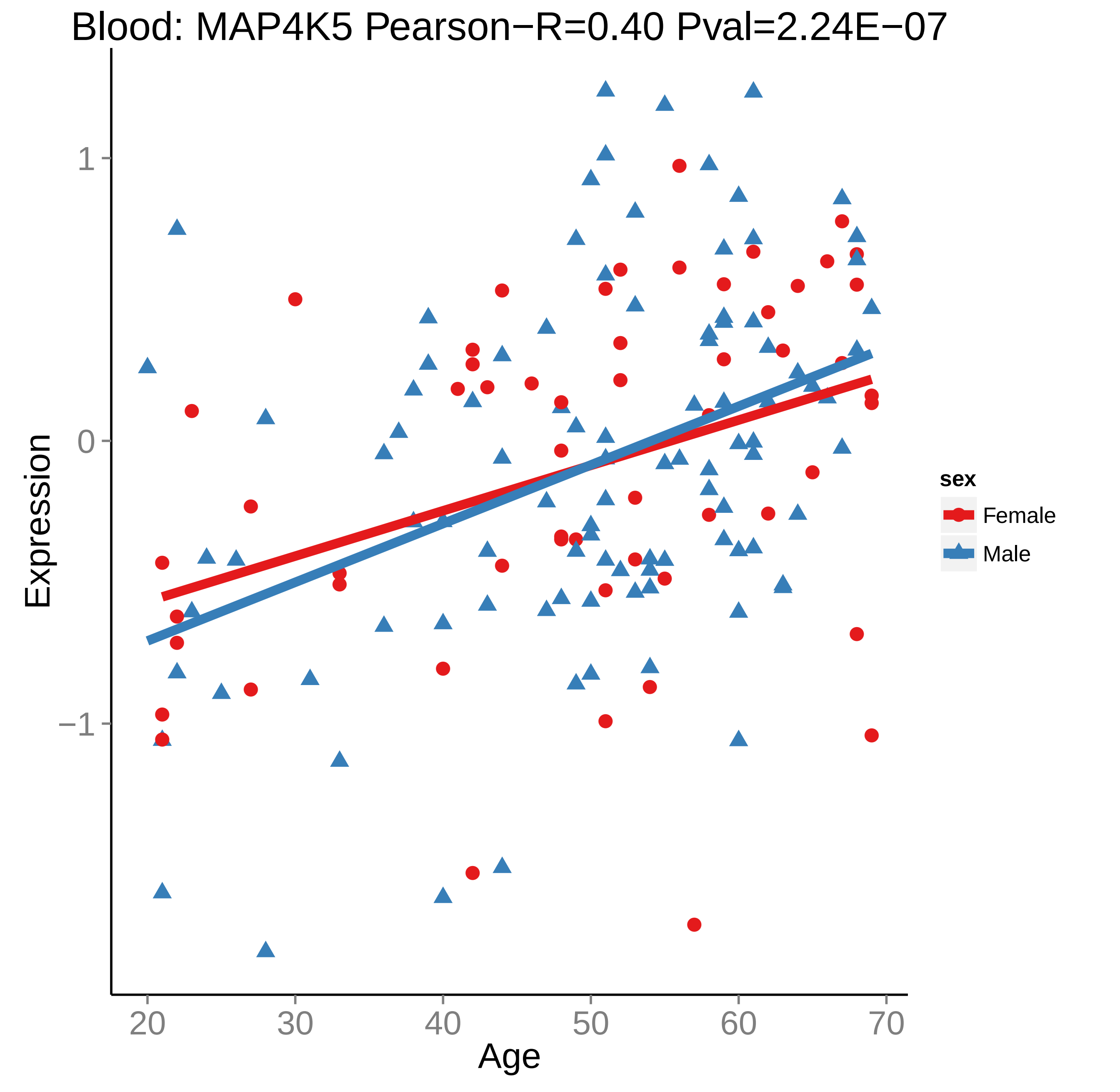

Blood: MAP4K5 Pearson−R=0.40 Pval=2.24E−07
Expression
1
0
−1
20
30
40
50
60
70
Age
sex
Female
Male

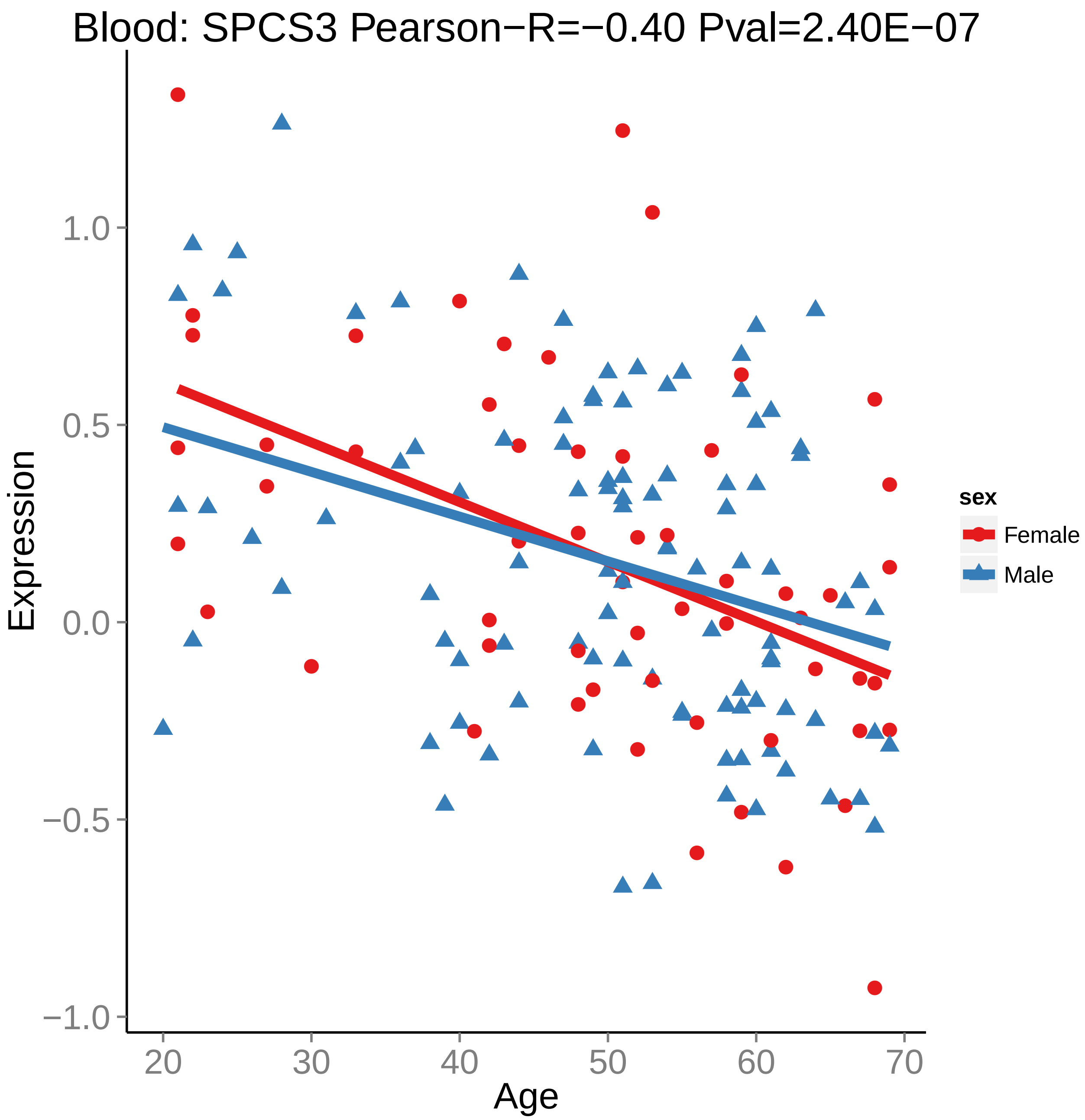

Blood: SPCS3 Pearson−R=−0.40 Pval=2.40E−07
Expression
1.0
0.5
0.0
−0.5
−1.0
20
30
40
50
60
70
Age
sex
Female
Male

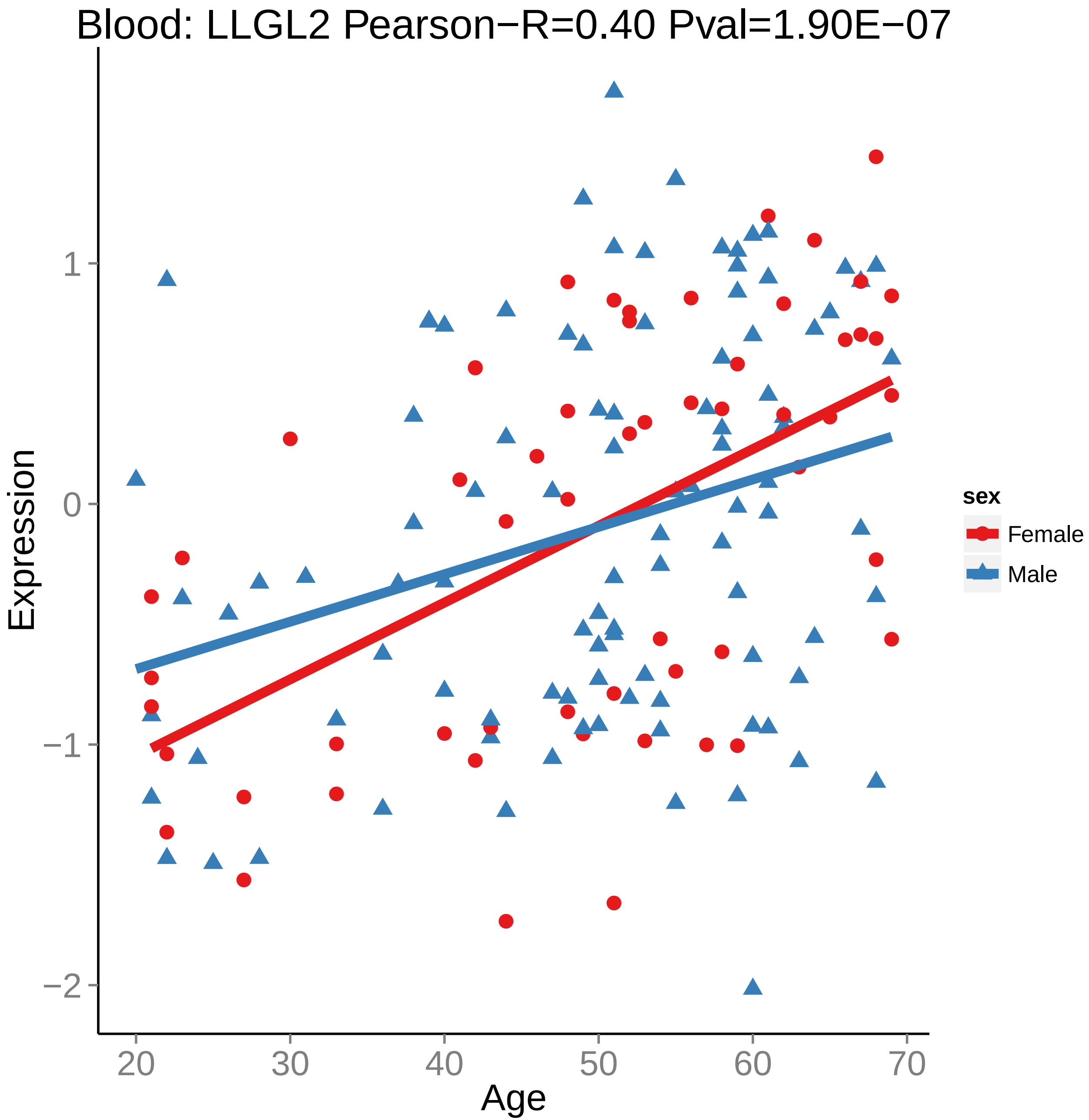

Blood: LLGL2 Pearson−R=0.40 Pval=1.90E−07
Expression
1
0
−1
−2
20
30
40
50
60
70
Age
sex
Female
Male

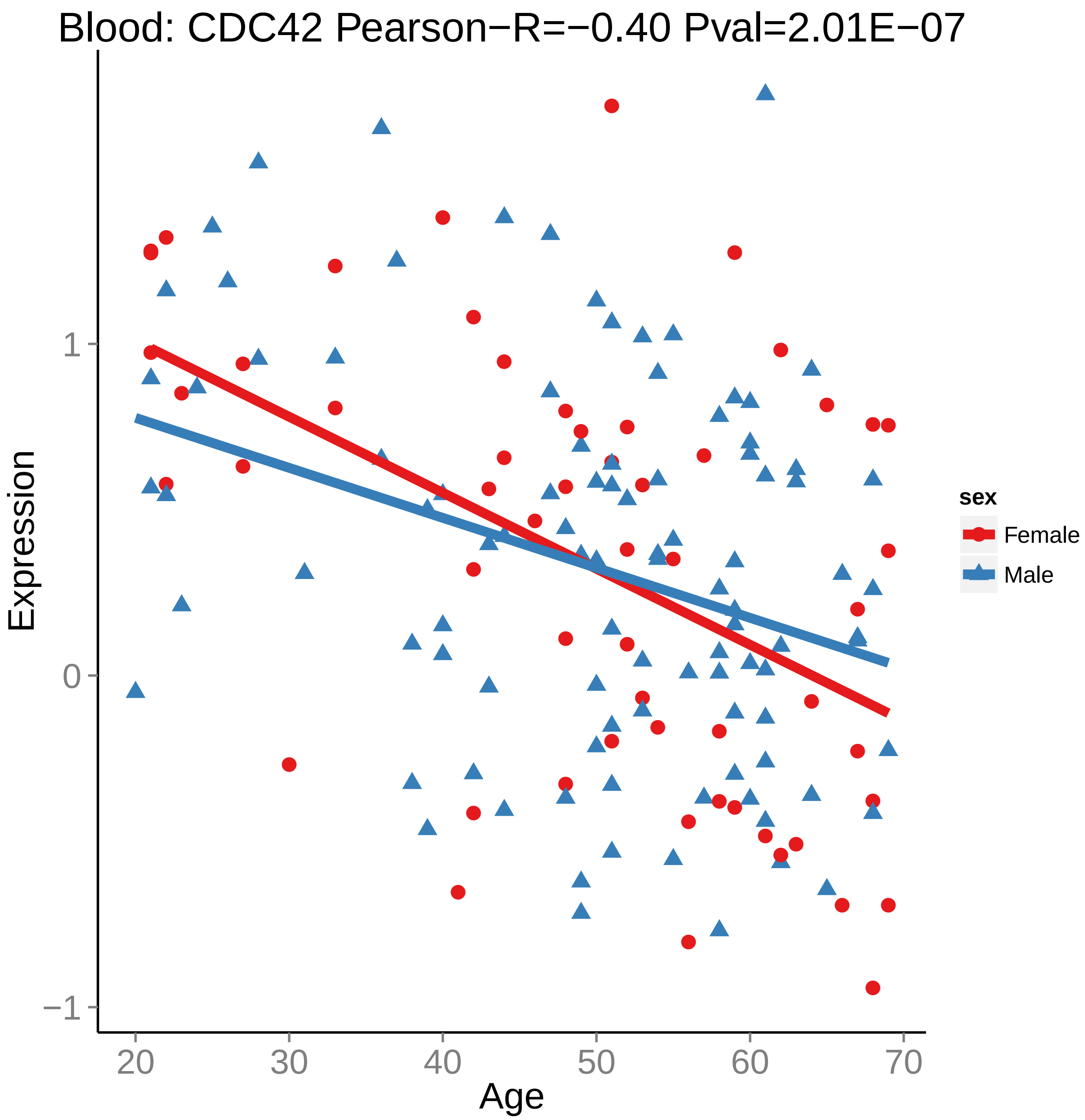

Blood: CDC42 Pearson−R=−0.40 Pval=2.01E−07
Expression
1
0
−1
20
30
40
50
60
70
Age
sex
Female
Male

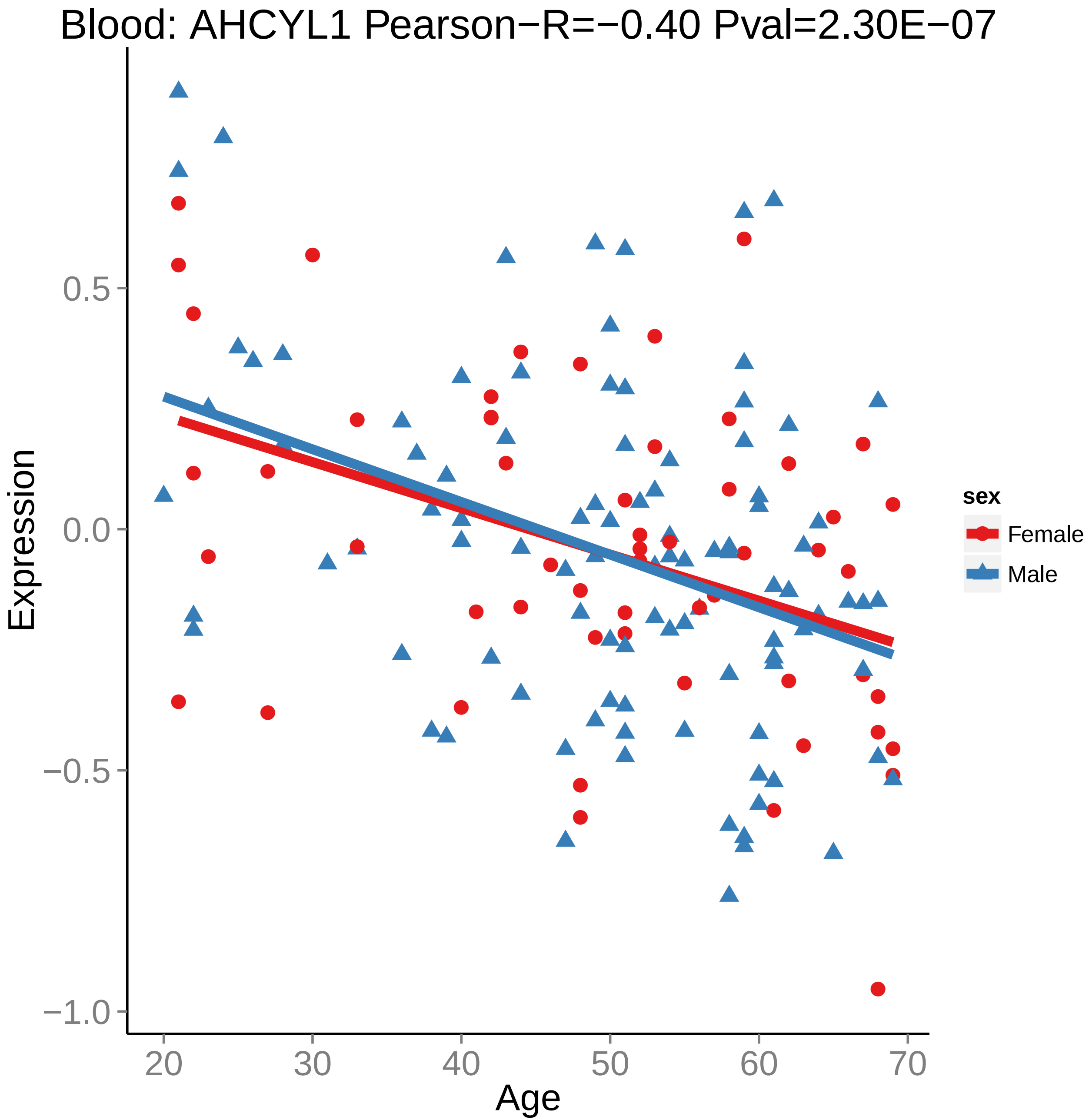

Blood: AHCYL1 Pearson−R=−0.40 Pval=2.30E−07
Expression
0.5
0.0
−0.5
−1.0
20
30
40
50
60
70
Age
sex
Female
Male

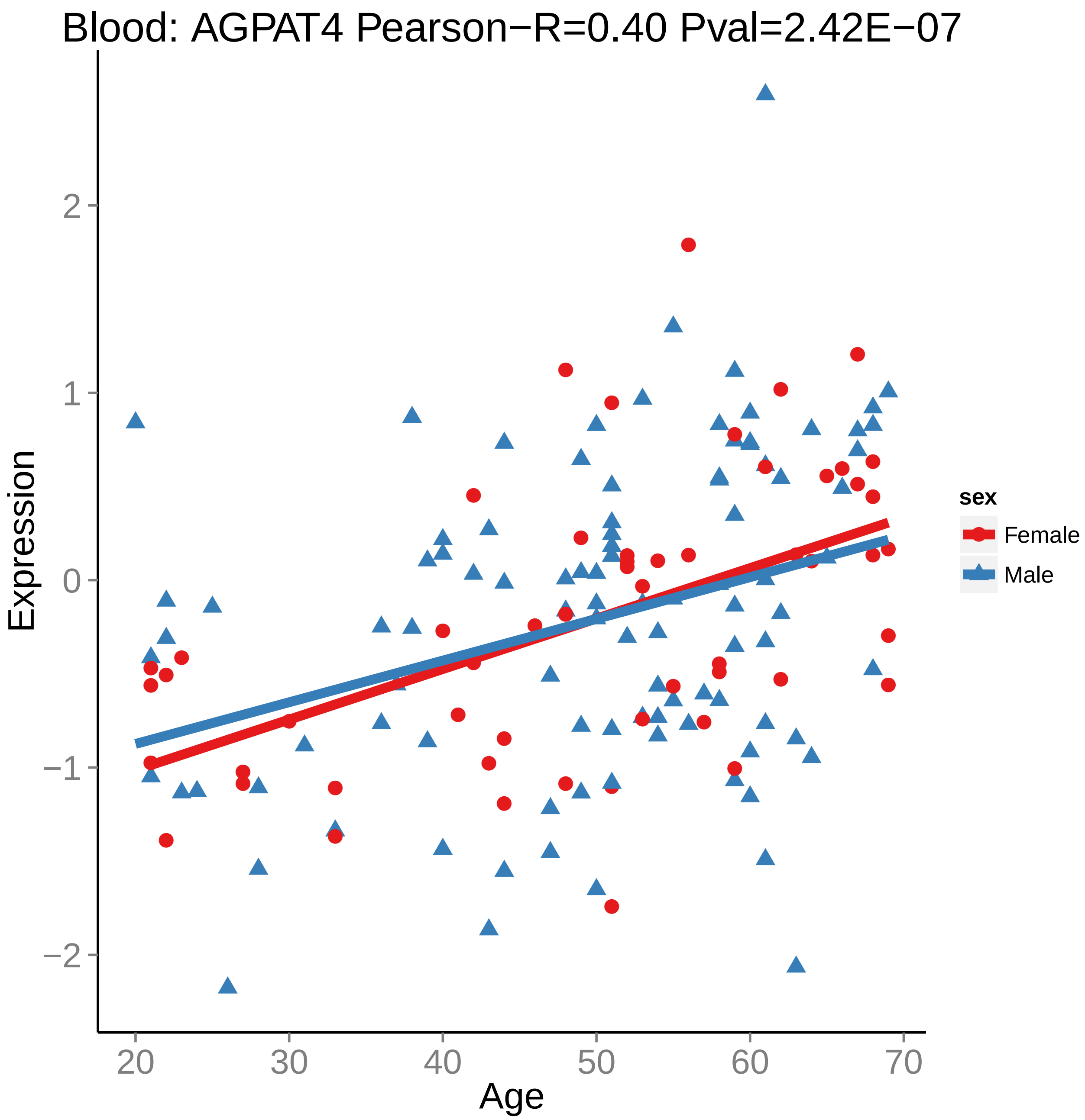

Blood: AGPAT4 Pearson−R=0.40 Pval=2.42E−07
Expression
2
1
0
−1
−2
20
30
40
50
60
70
Age
sex
Female
Male

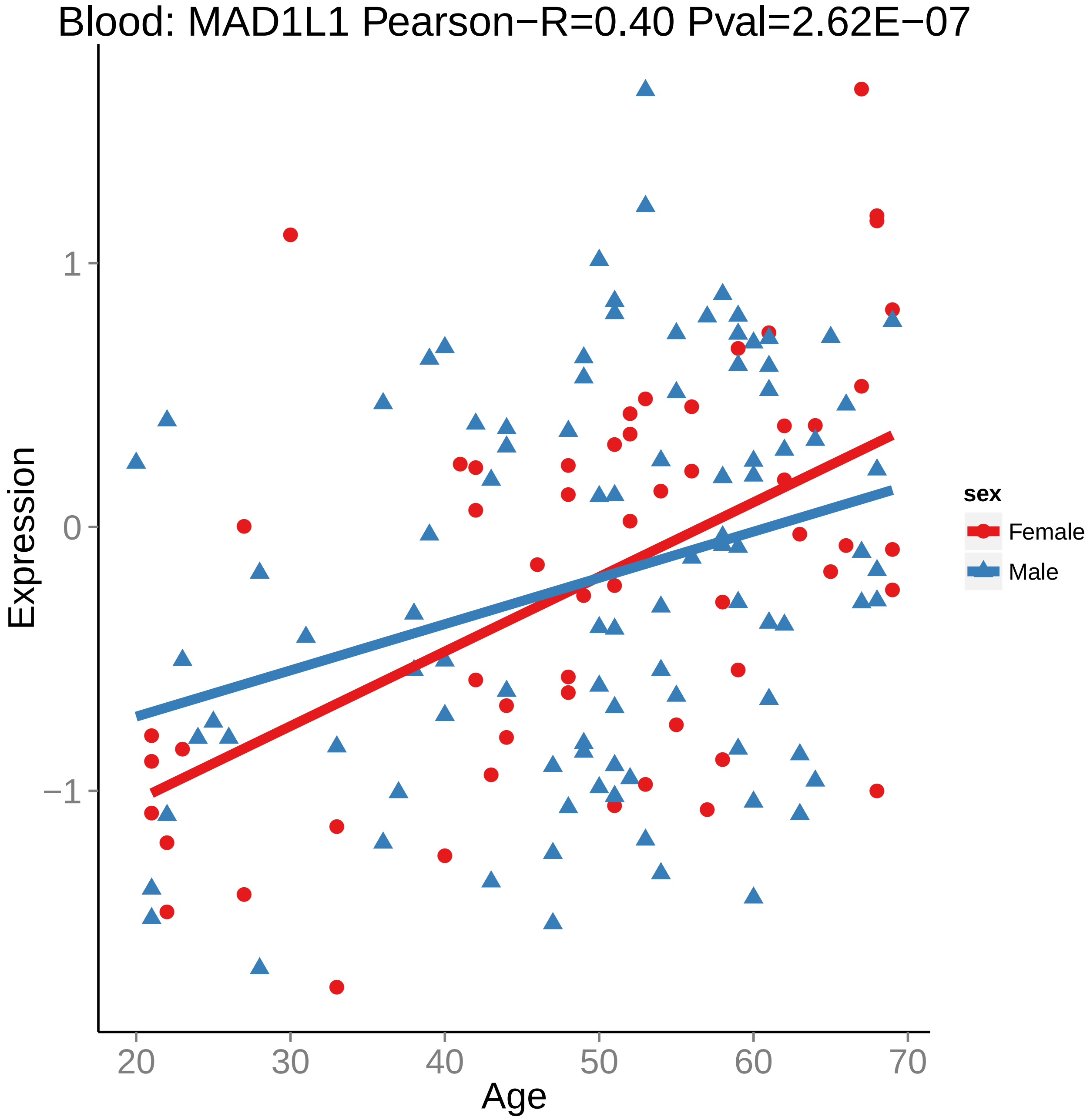

Blood: MAD1L1 Pearson−R=0.40 Pval=2.62E−07
Expression
1
0
−1
20
30
40
50
60
70
Age
sex
Female
Male

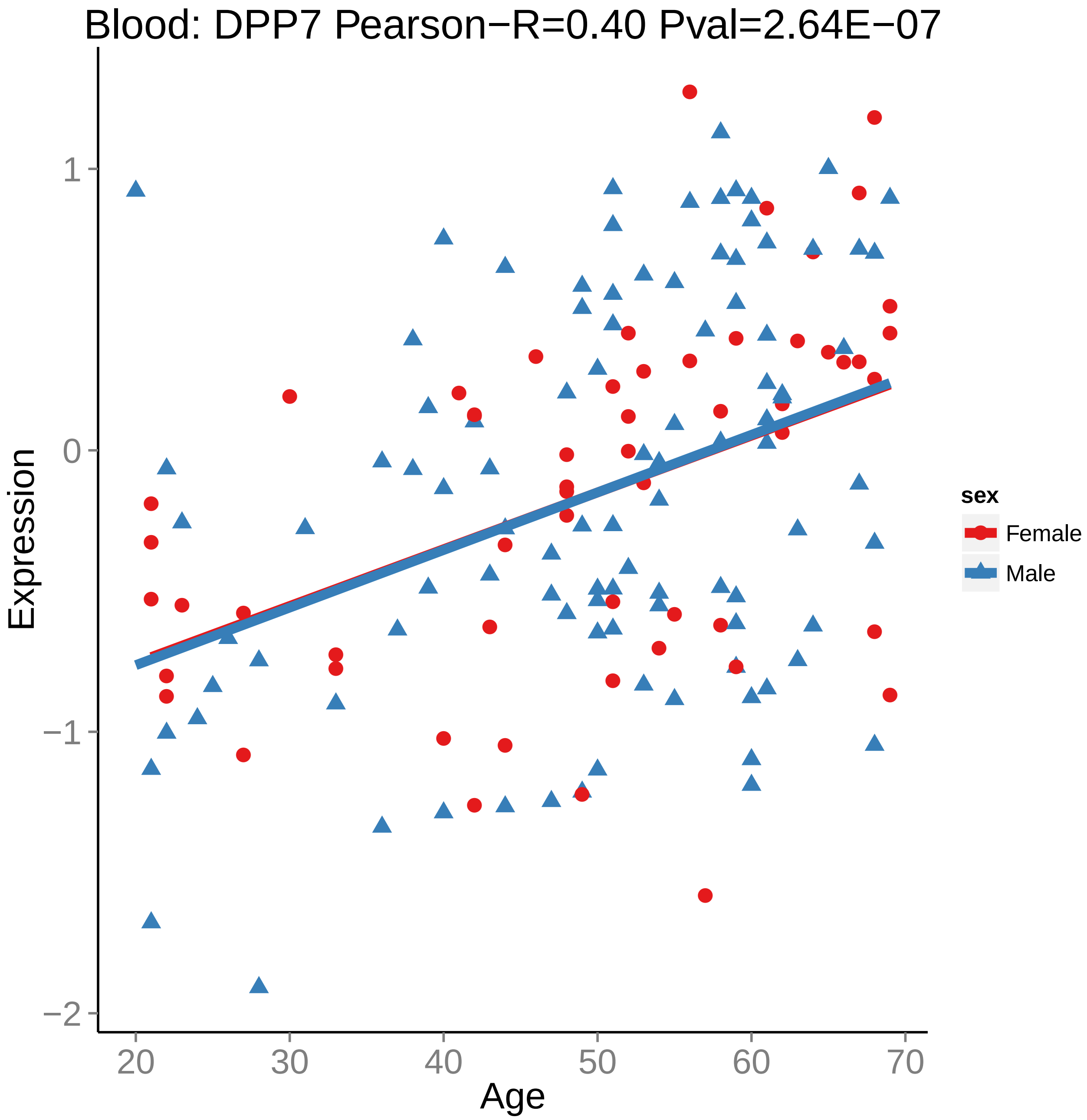

Blood: DPP7 Pearson−R=0.40 Pval=2.64E−07
Expression
1
0
−1
−2
20
30
40
50
60
70
Age
sex
Female
Male

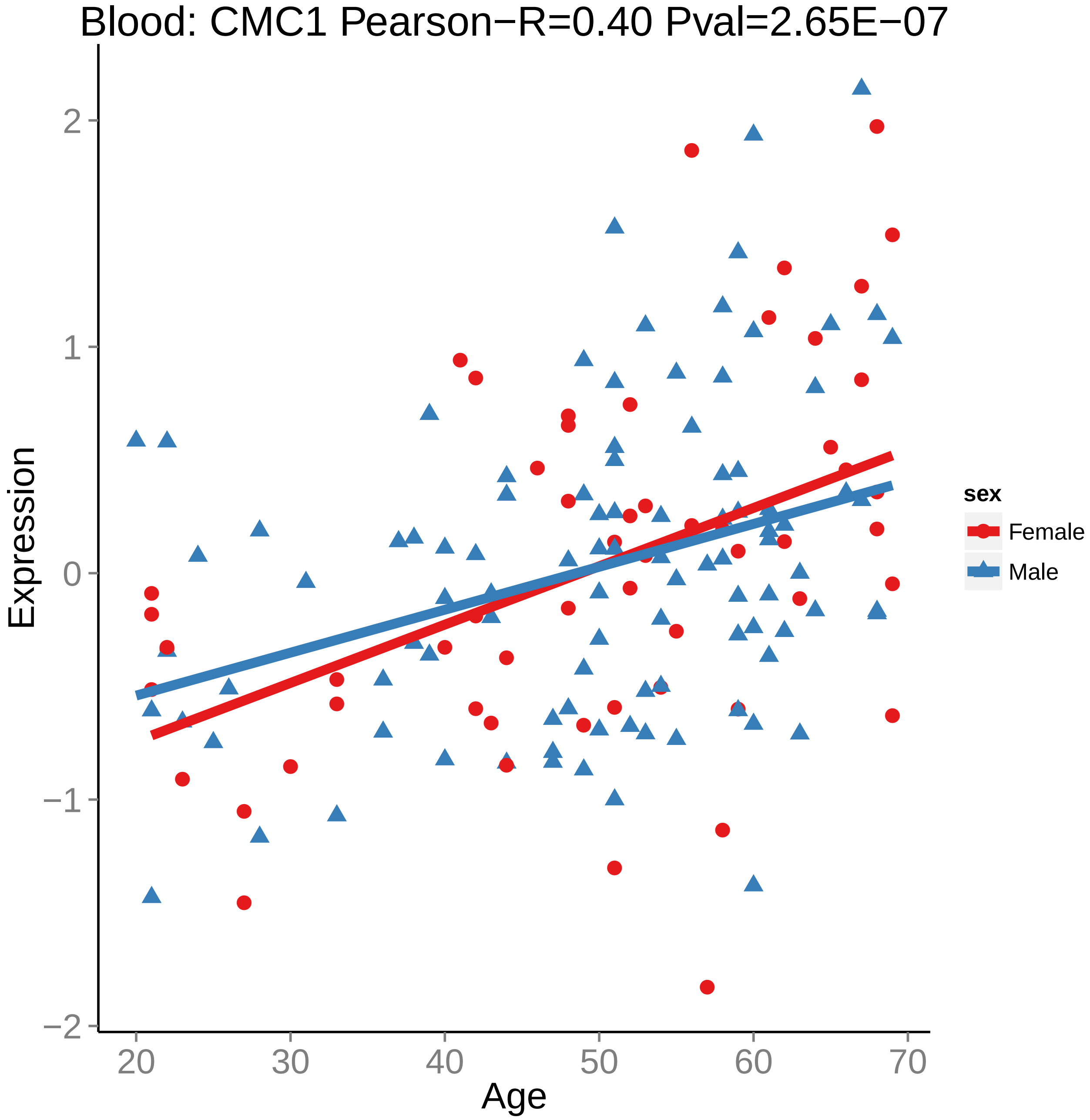

Blood: CMC1 Pearson−R=0.40 Pval=2.65E−07
Expression
Age
2
1
0
−1
−2
20
30
40
50
60
70
sex
Female
Male

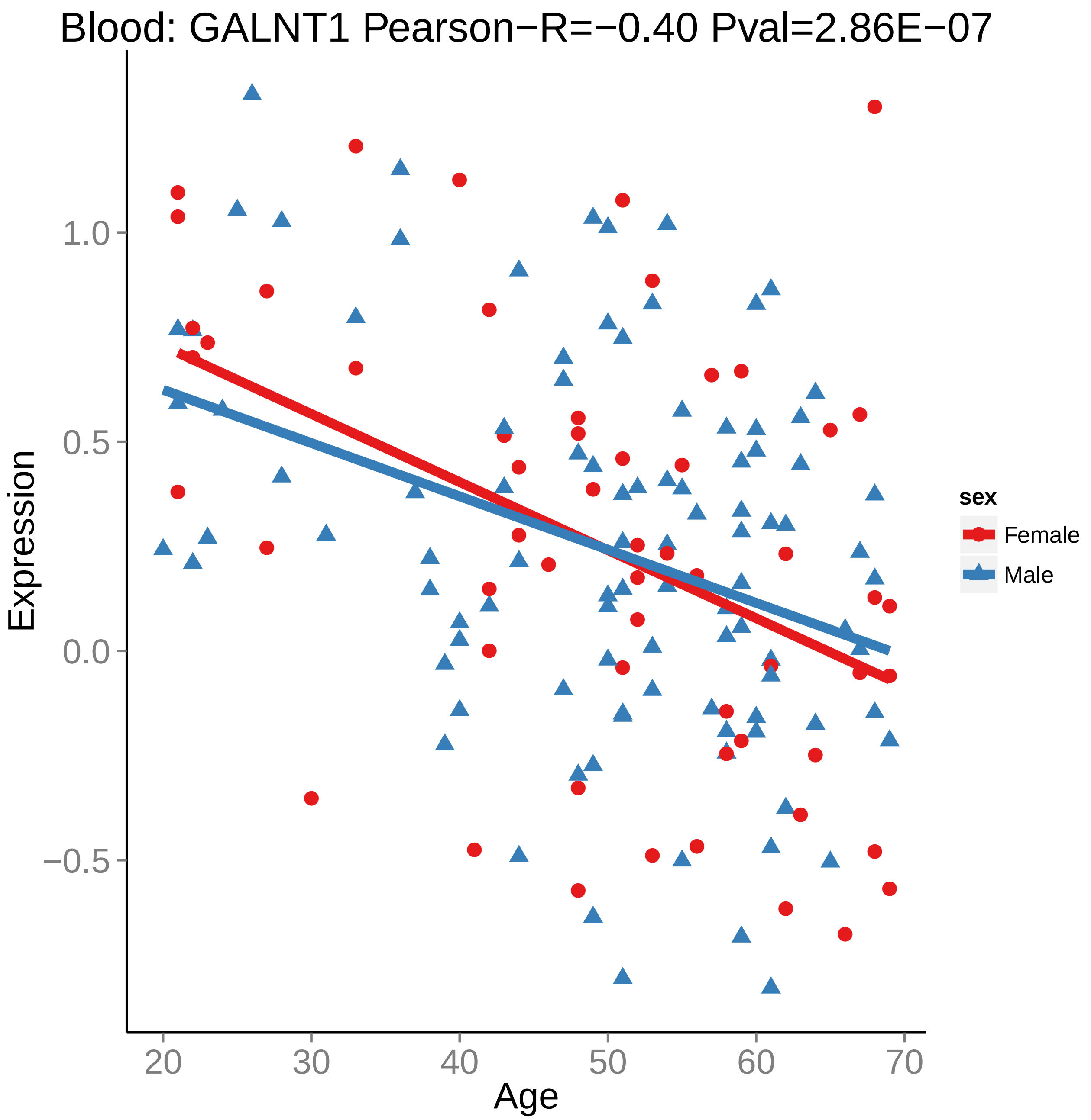

Blood: GALNT1 Pearson−R=−0.40 Pval=2.86E−07
Expression
1.0
0.5
0.0
−0.5
20
30
40
50
60
70
Age
sex
Female
Male

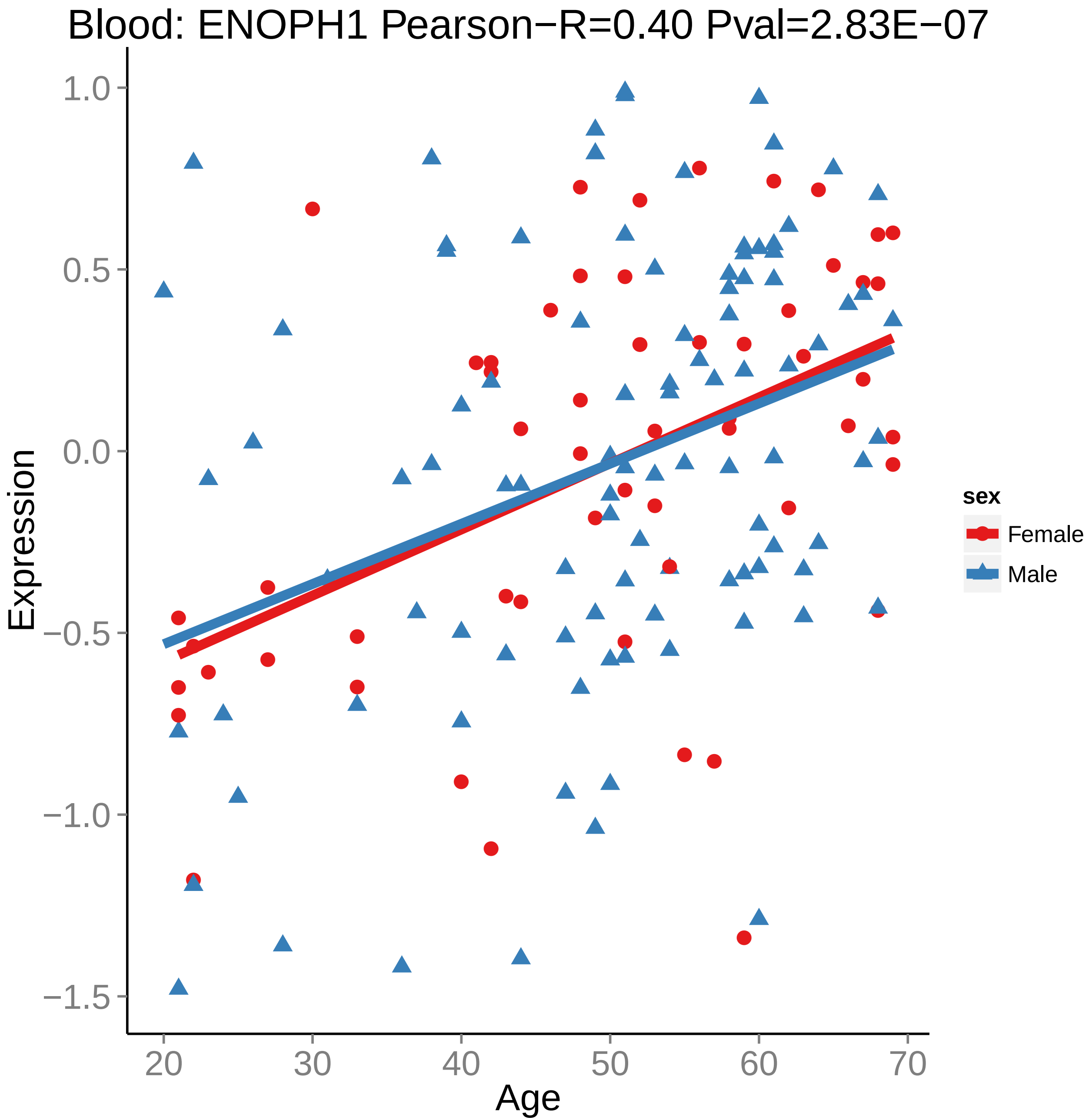
Blood: ENOPH1 Pearson−R=0.40 Pval=2.83E−07
1.0
0.5
0.0
−0.5
−1.0
−1.5
Expression
20
30
40
50
60
70
Age
sex
Female
Male

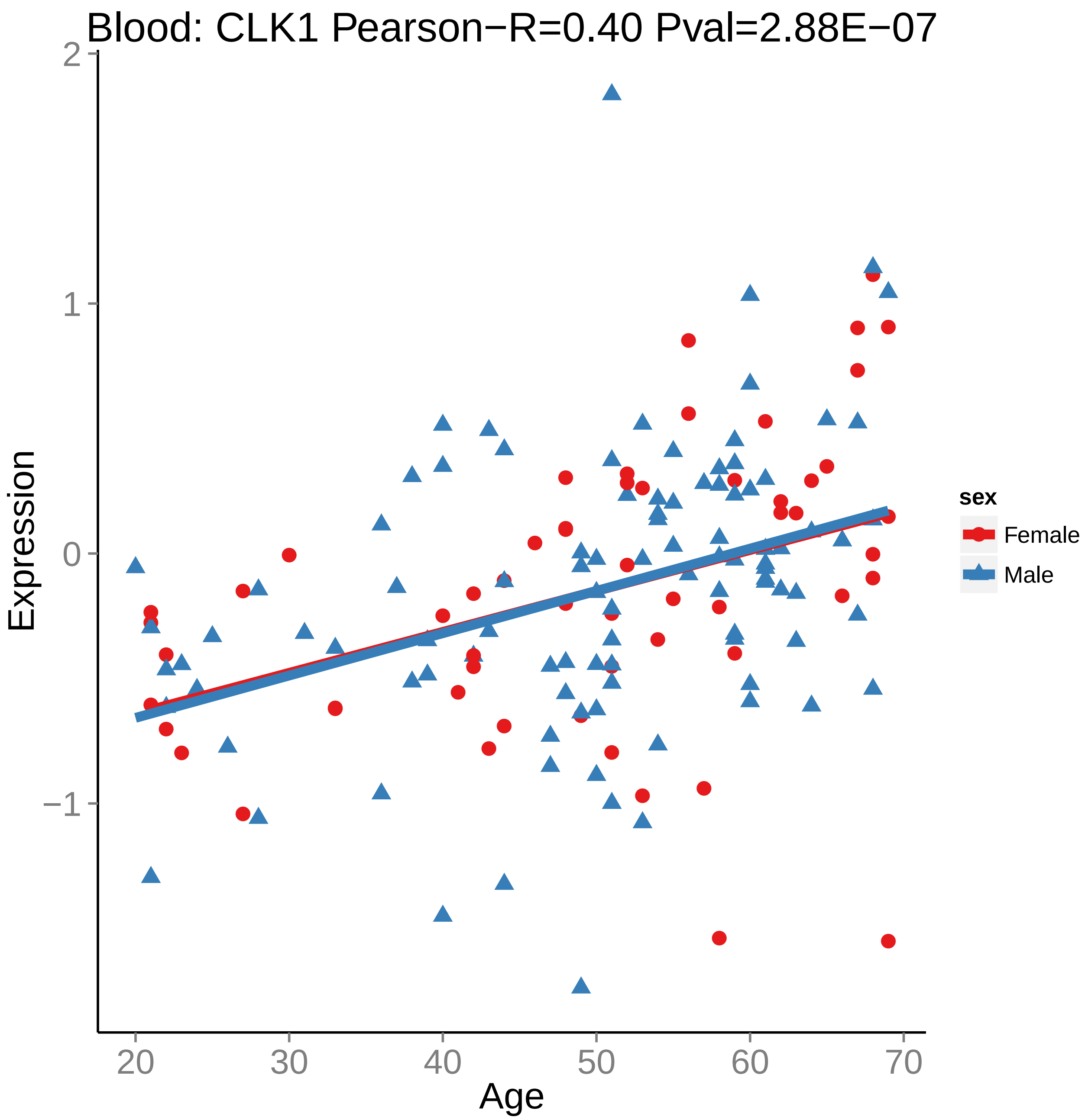
Blood: CLK1 Pearson−R=0.40 Pval=2.88E−07
Expression
Age
sex
Female
Male
2
1
0
−1
20
30
40
50
60
70

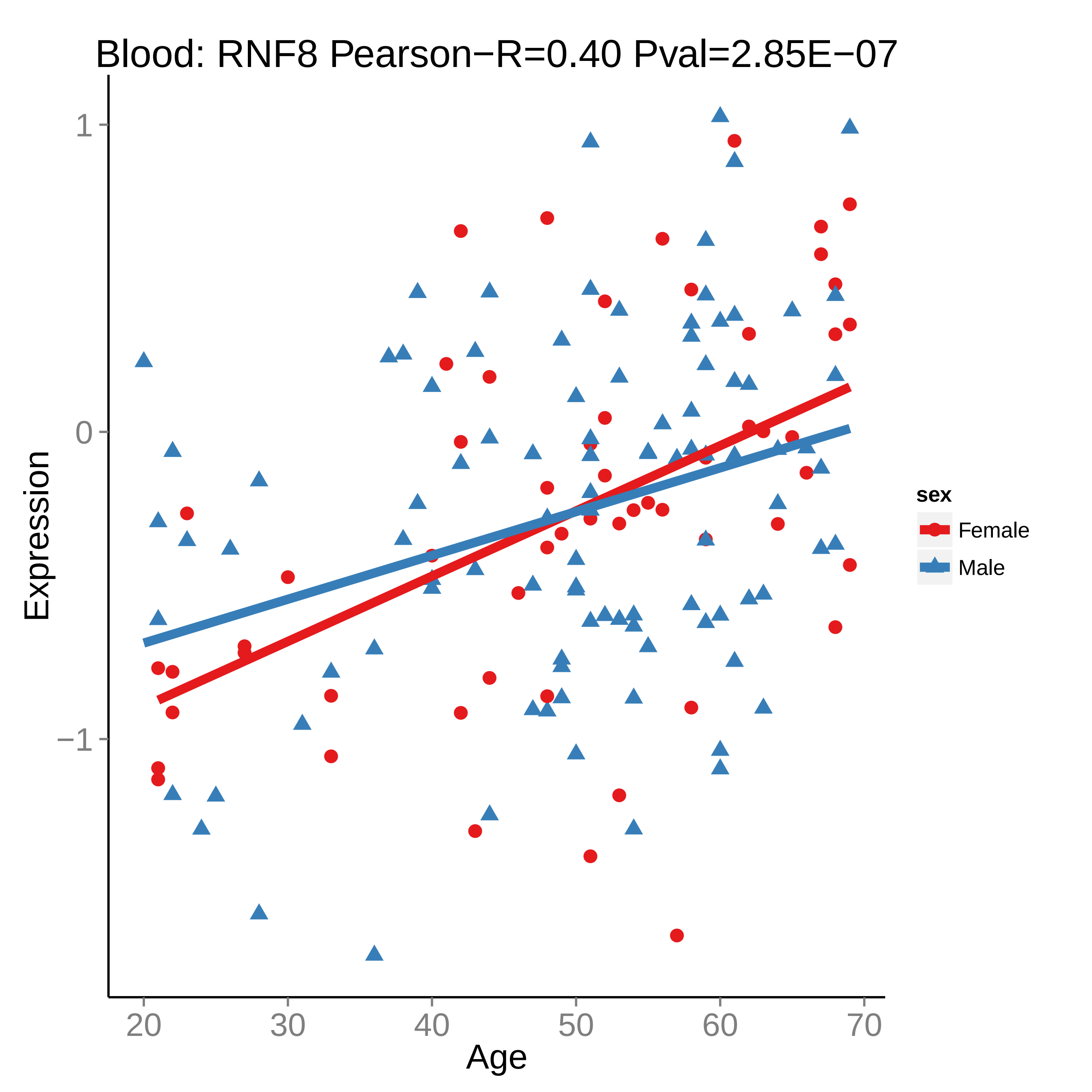
Blood: RNF8 Pearson−R=0.40 Pval=2.85E−07
1
0
−1
Expression
20
30
40
50
60
70
Age
sex
Female
Male

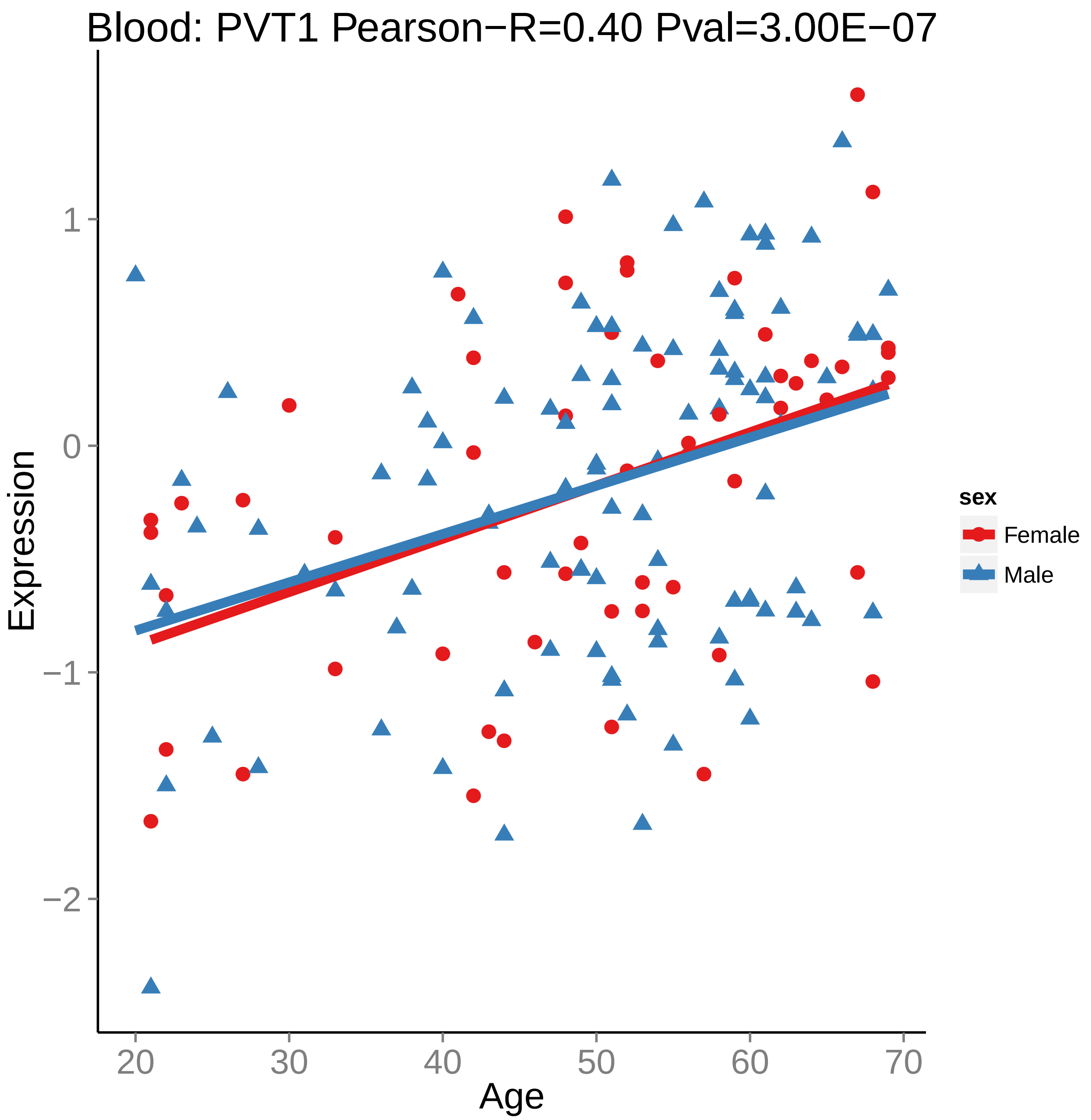

Blood: PVT1 Pearson−R=0.40 Pval=3.00E−07
Expression
1
0
−1
−2
20
30
40
50
60
70
Age
sex
Female
Male

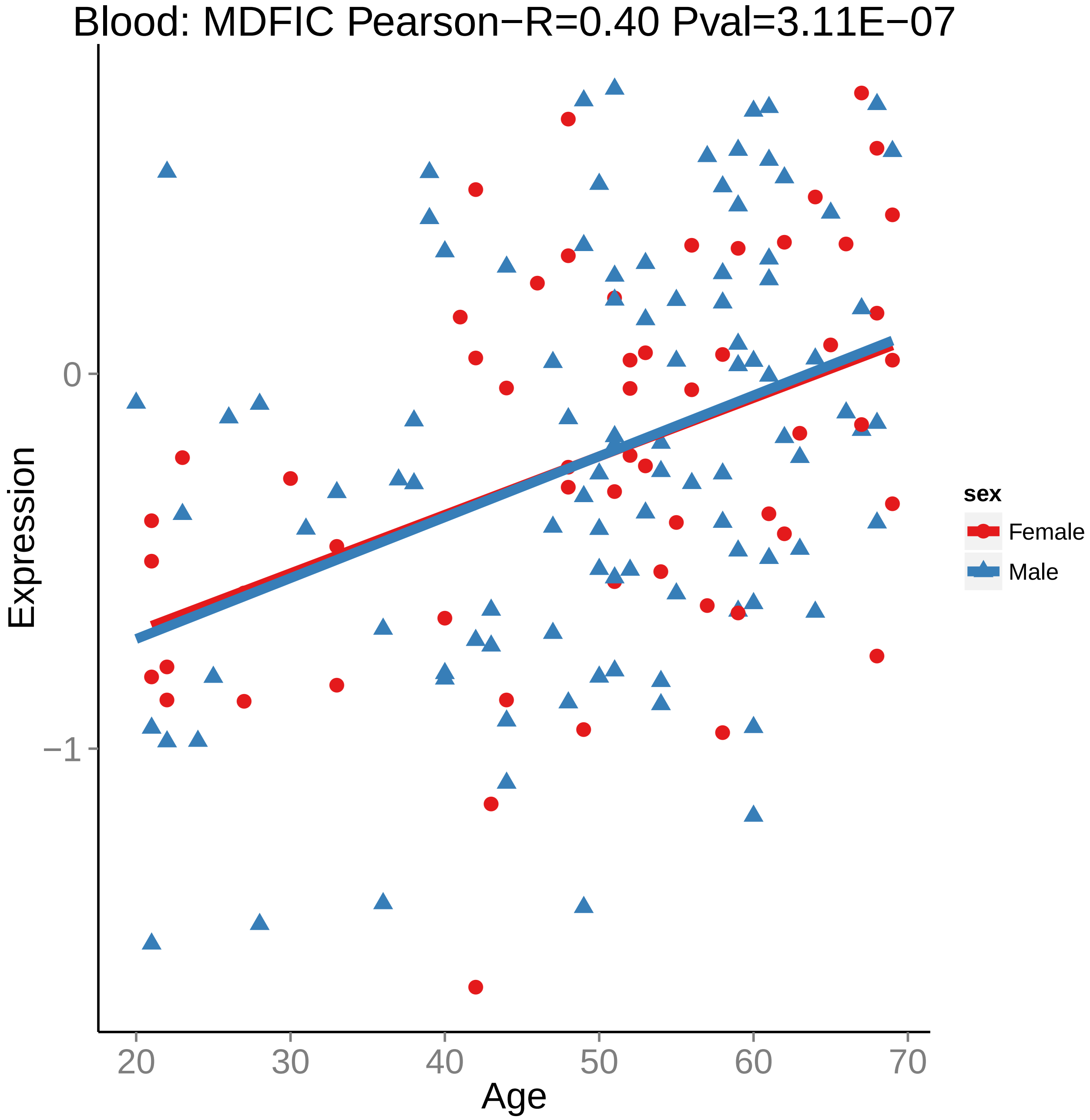

Blood: MDFIC Pearson−R=0.40 Pval=3.11E−07
Expression
0
−1
20
30
40
50
60
70
Age
sex
Female
Male

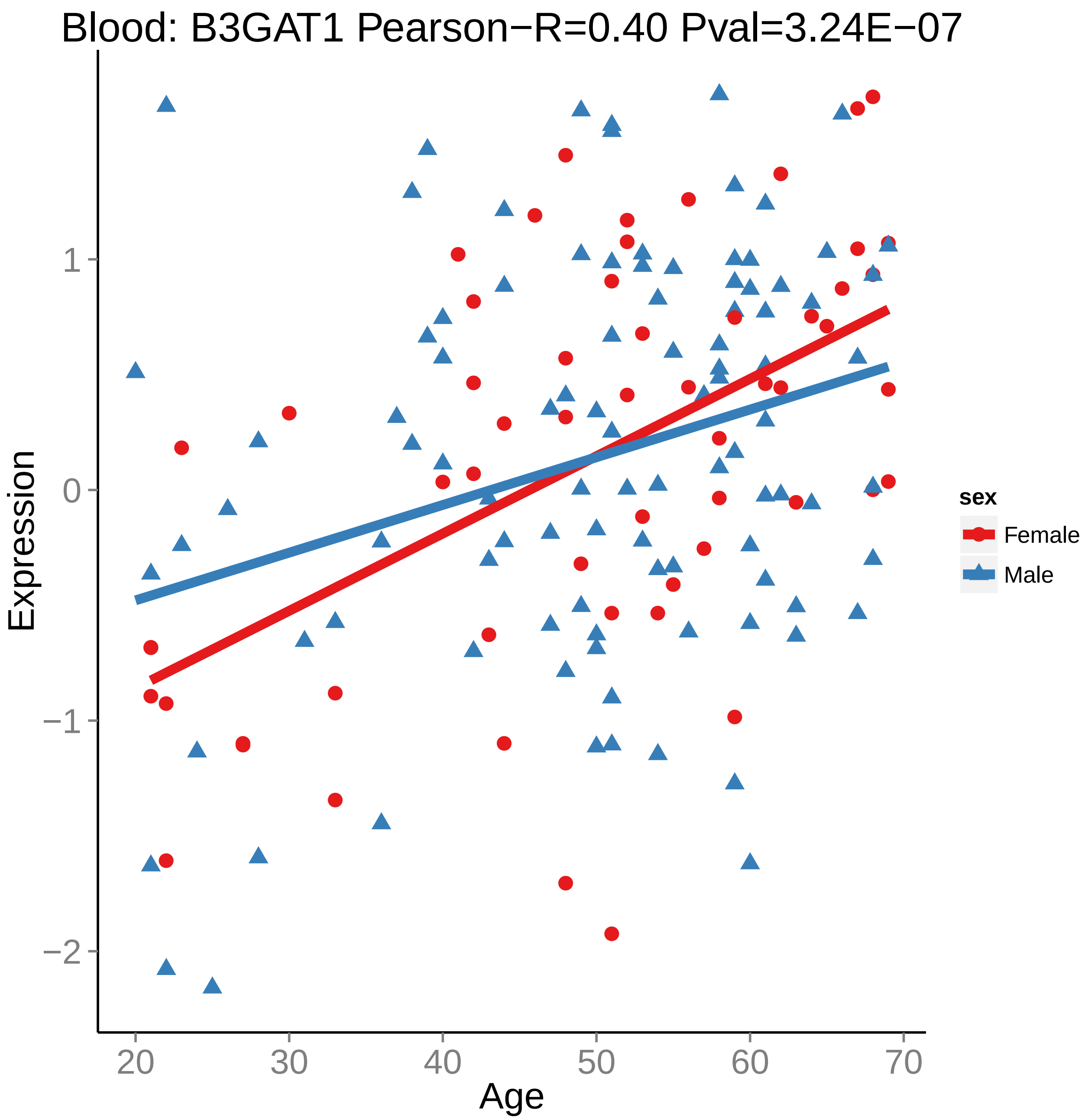

Blood: B3GAT1 Pearson−R=0.40 Pval=3.24E−07
Expression
Age
1
0
−1
−2
20
30
40
50
60
70
sex
Female
Male

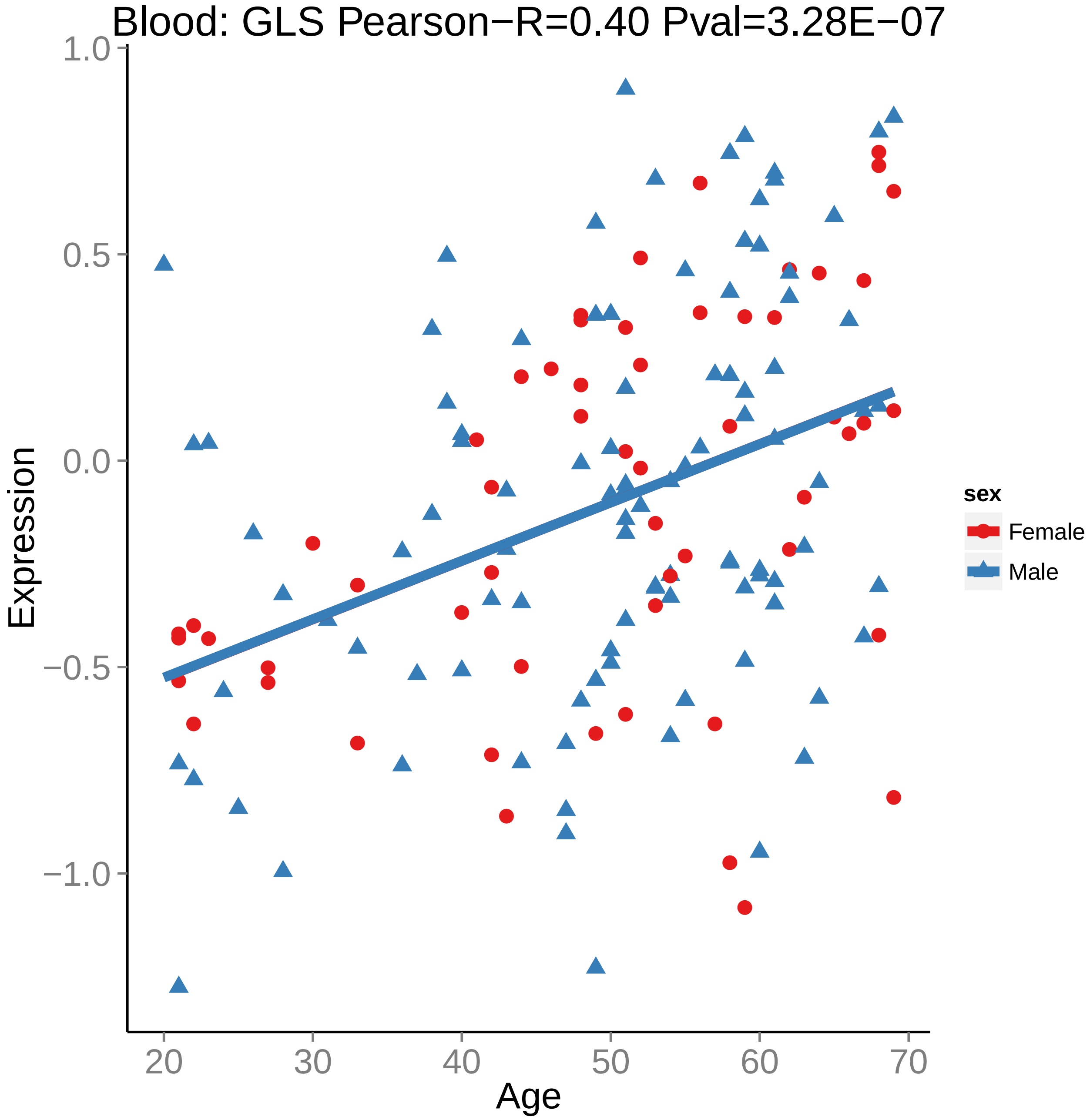

Blood: GLS Pearson−R=0.40 Pval=3.28E−07
1.0
0.5
0.0
−0.5
−1.0
Expression
20
30
40
50
60
70
Age
sex
Female
Male

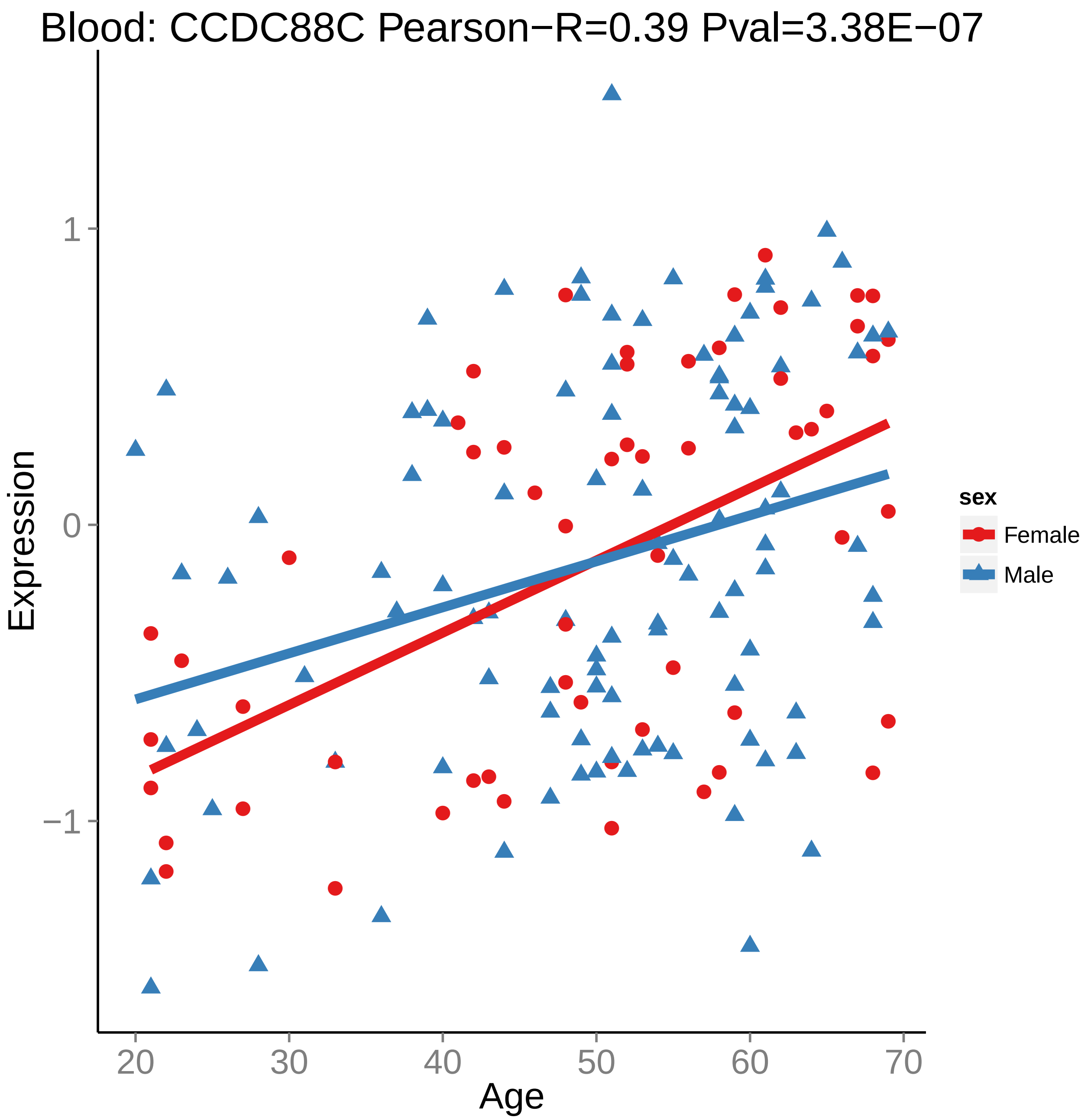

Blood: CCDC88C Pearson−R=0.39 Pval=3.38E−07
Expression
1
0
−1
20
30
40
50
60
70
Age
sex
Female
Male

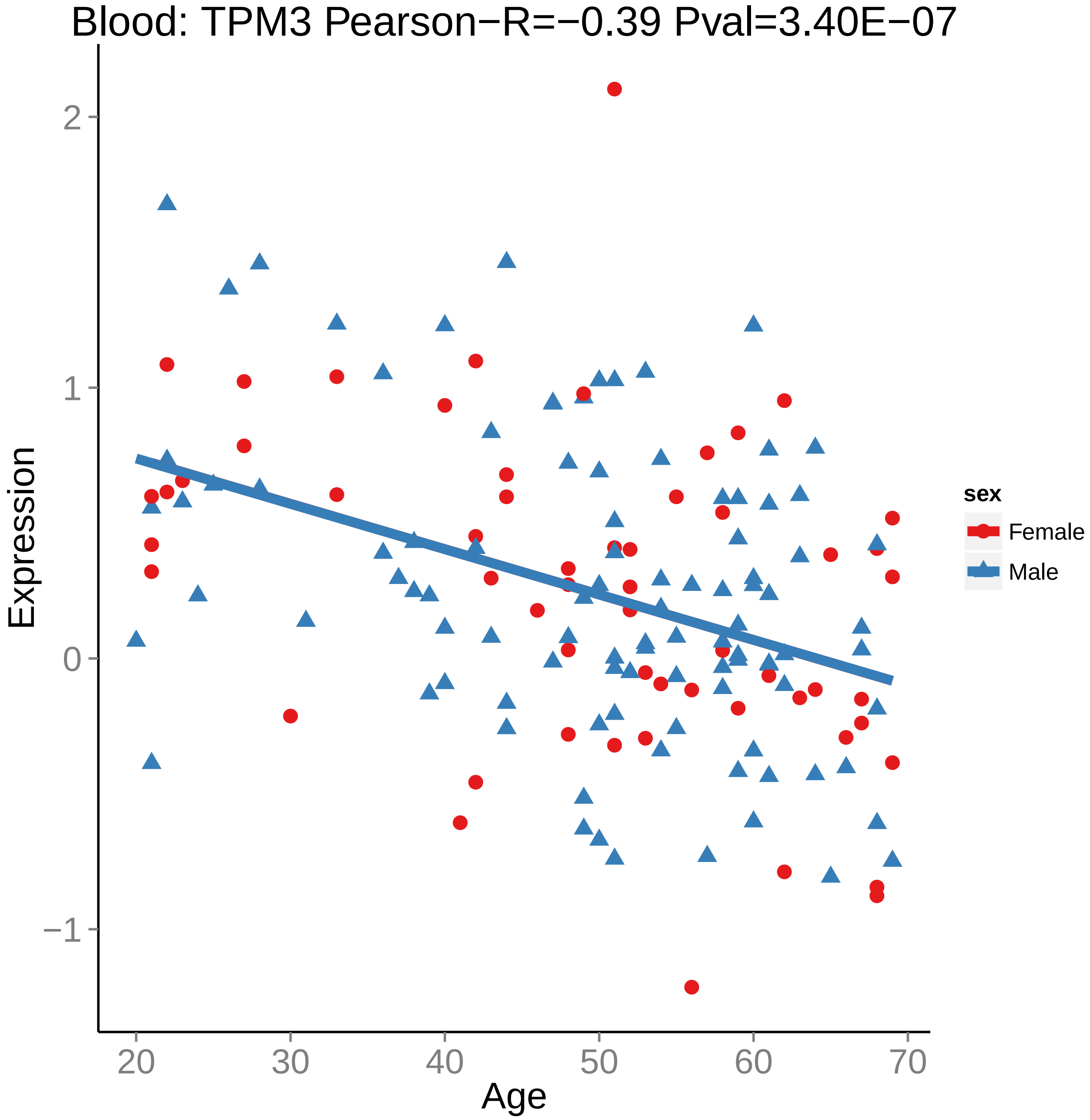

Blood: TPM3 Pearson−R=−0.39 Pval=3.40E−07
Expression
2
1
0
−1
20
30
40
50
60
70
Age
sex
Female
Male

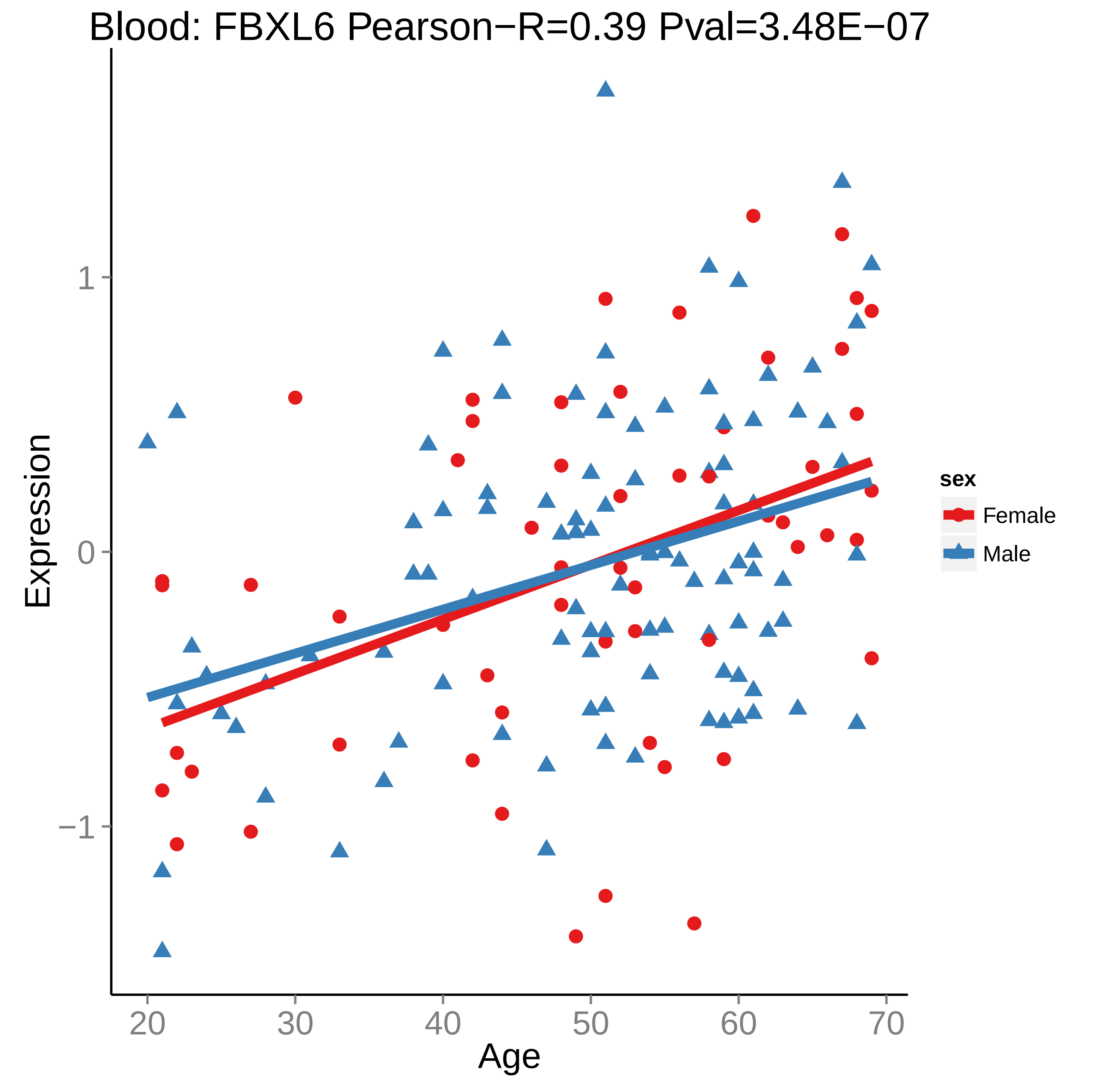

Blood: FBXL6 Pearson−R=0.39 Pval=3.48E−07
Expression
1
0
−1
20
30
40
50
60
70
Age
sex
Female
Male

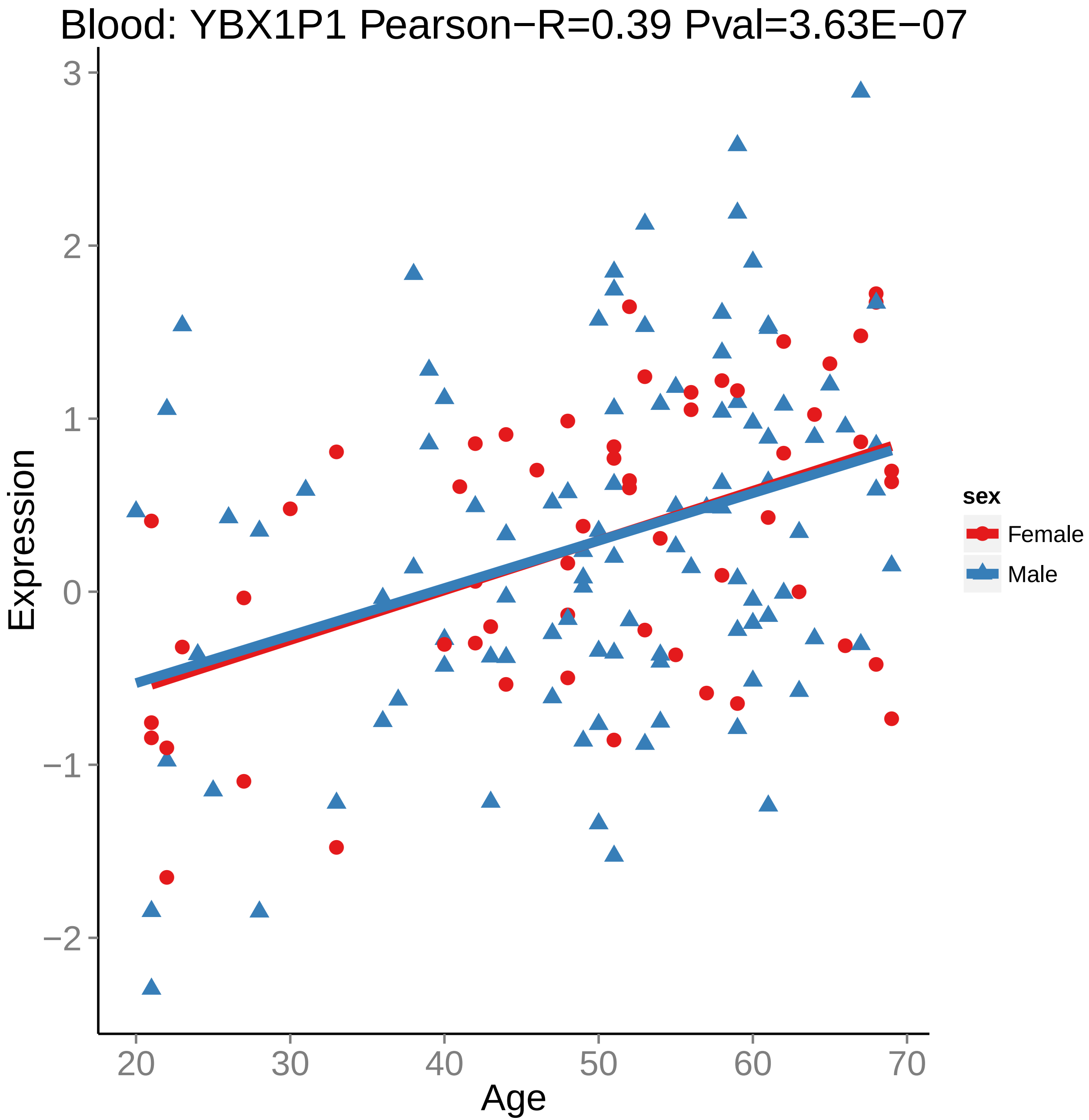
Blood: YBX1P1 Pearson−R=0.39 Pval=3.63E−07
Expression
Age
3
2
1
0
−1
−2
20
30
40
50
60
70
sex
Female
Male

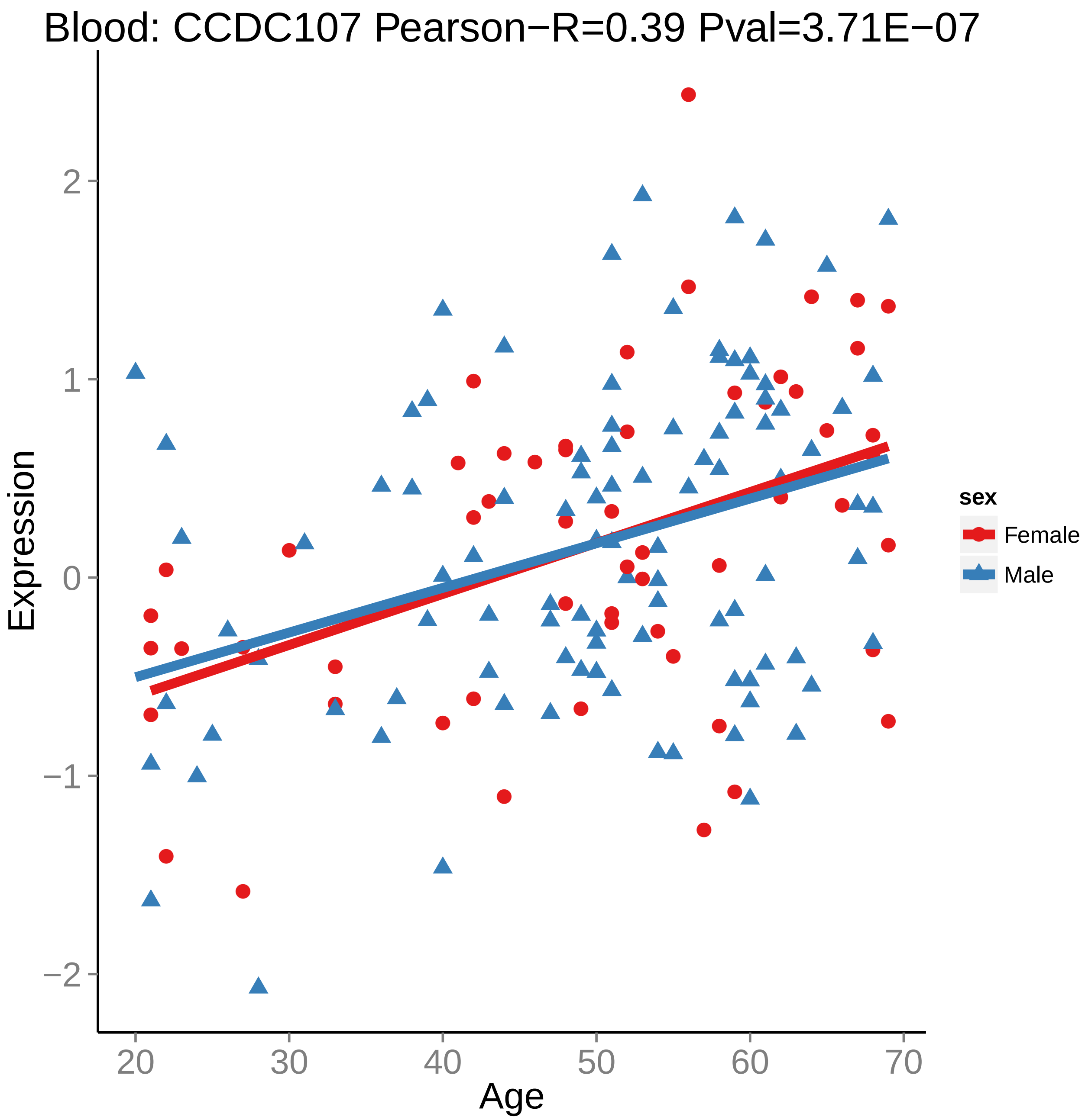
Blood: CCDC107 Pearson−R=0.39 Pval=3.71E−07
Expression
Age
2
1
0
−1
−2
20
30
40
50
60
70
sex
Female
Male

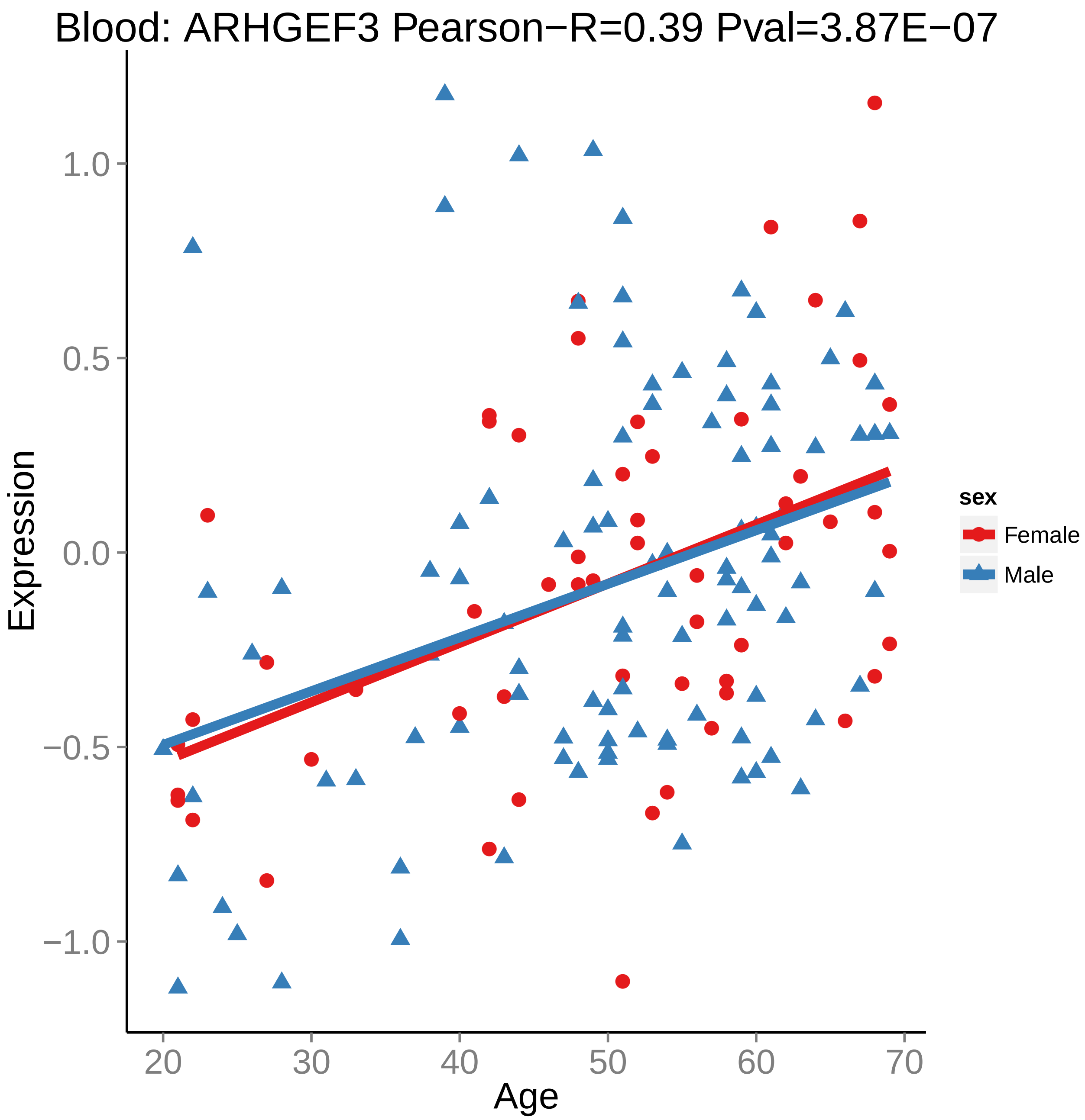

Blood: ARHGEF3 Pearson−R=0.39 Pval=3.87E−07
Expression
1.0
0.5
0.0
−0.5
−1.0
20
30
40
50
60
70
Age
sex
Female
Male

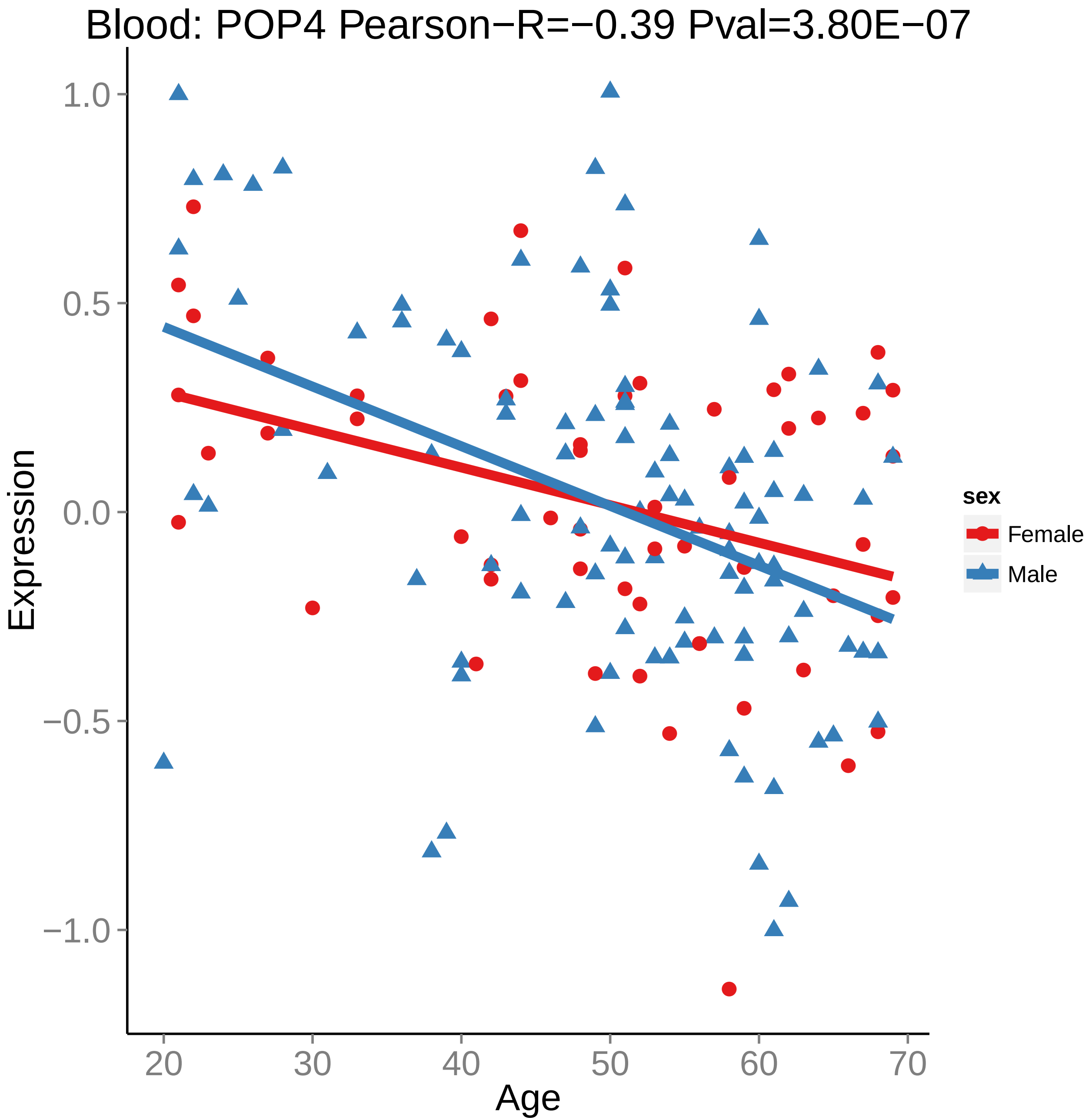

Blood: POP4 Pearson−R=−0.39 Pval=3.80E−07
Expression
1.0
0.5
0.0
−0.5
−1.0
20
30
40
50
60
70
Age
sex
Female
Male

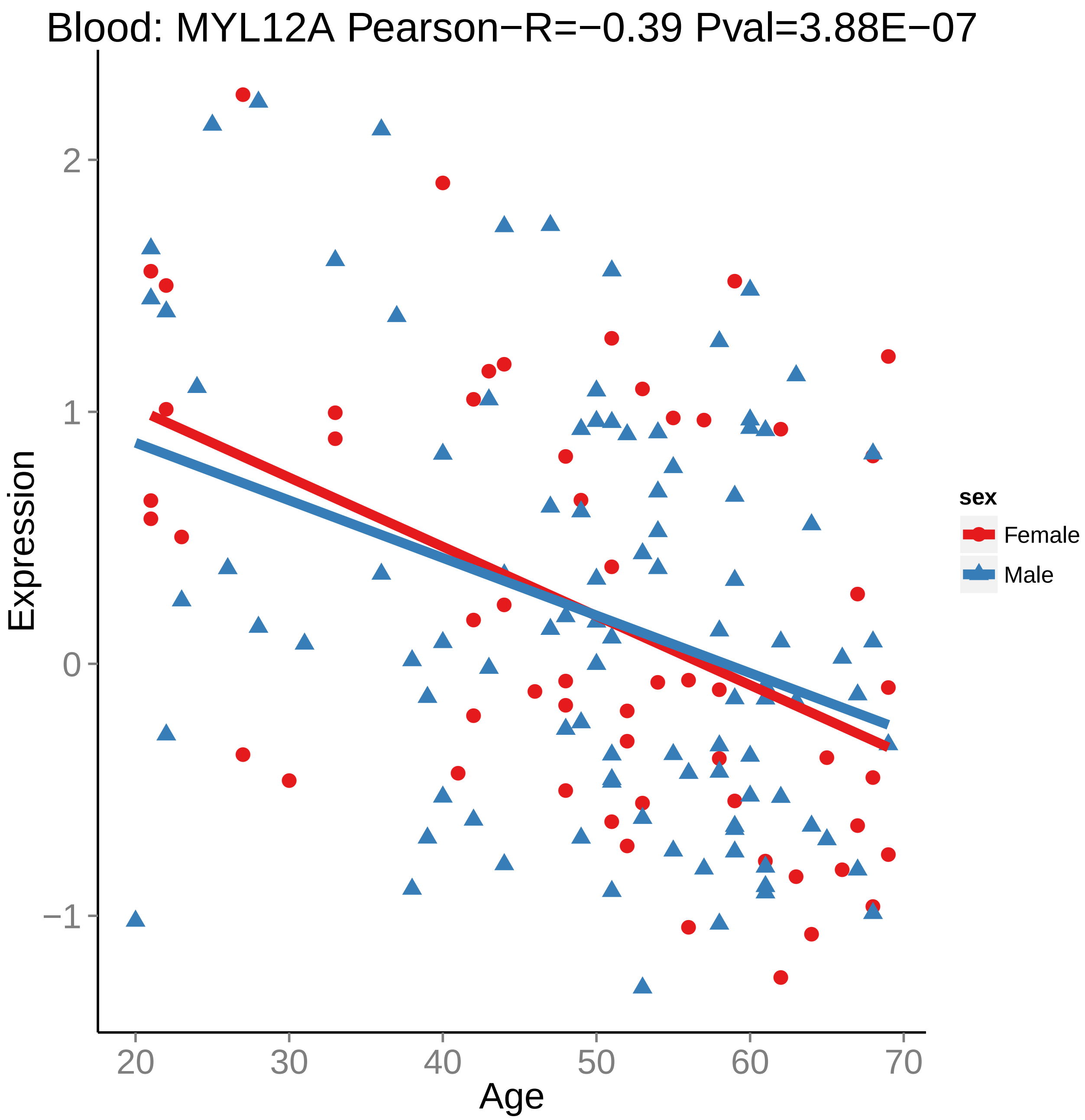

Blood: MYL12A Pearson−R=−0.39 Pval=3.88E−07
Expression
2
1
0
−1
20
30
40
50
60
70
Age
sex
Female
Male

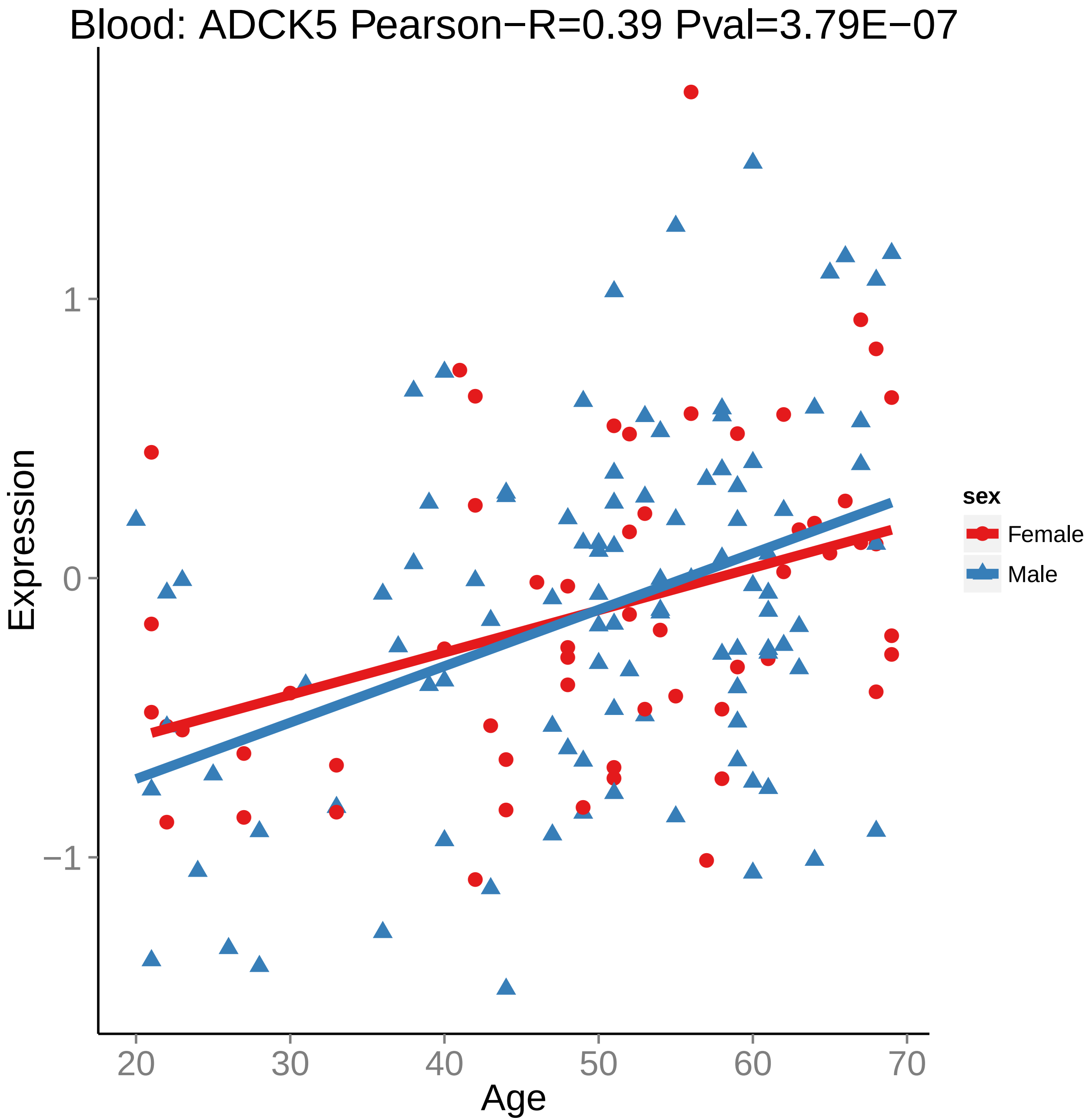

Blood: ADCK5 Pearson−R=0.39 Pval=3.79E−07
Expression
1
0
−1
20
30
40
50
60
70
Age
sex
Female
Male

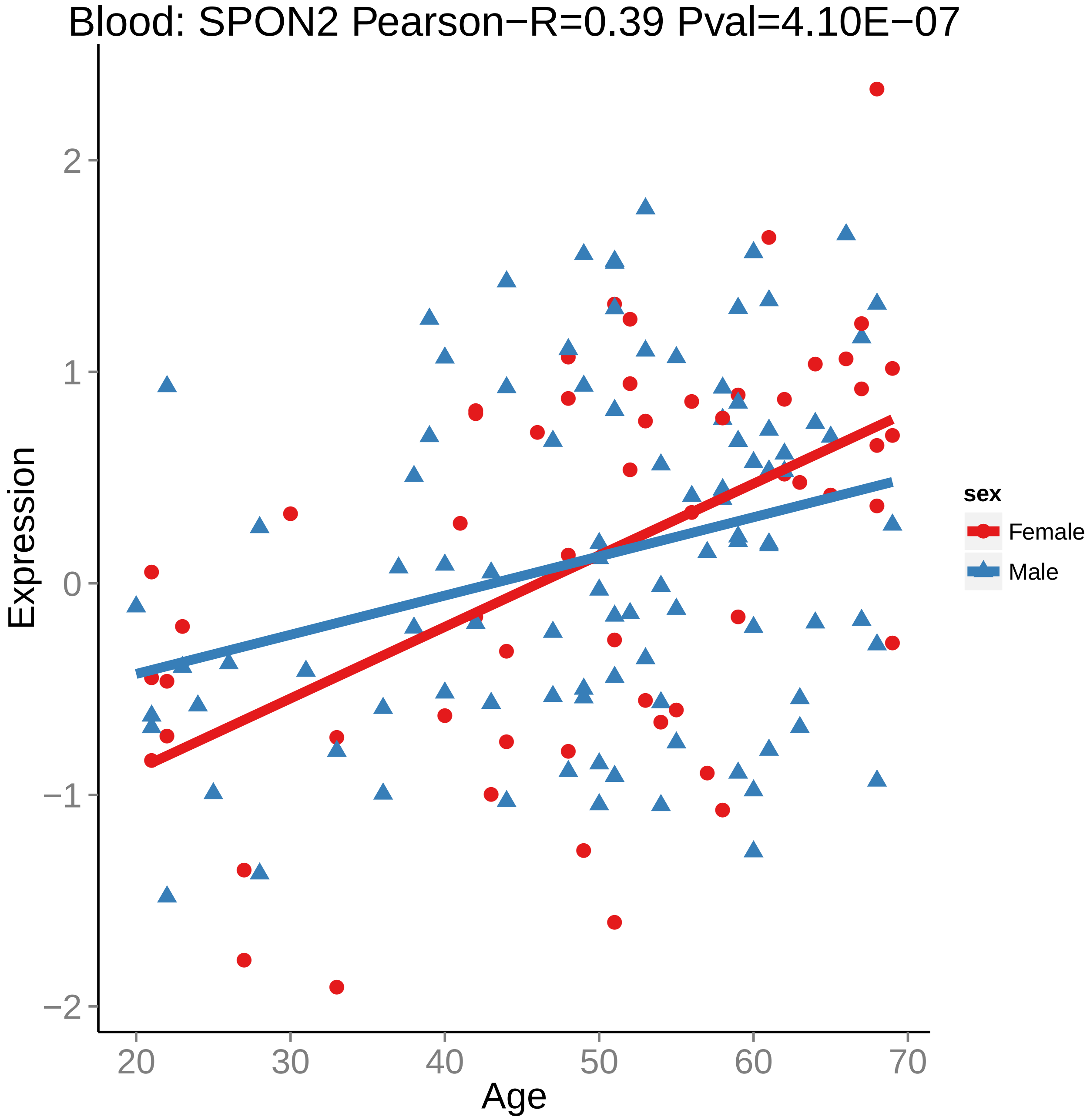

Blood: SPON2 Pearson−R=0.39 Pval=4.10E−07
Expression
2
1
0
−1
−2
20
30
40
50
60
70
Age
sex
Female
Male

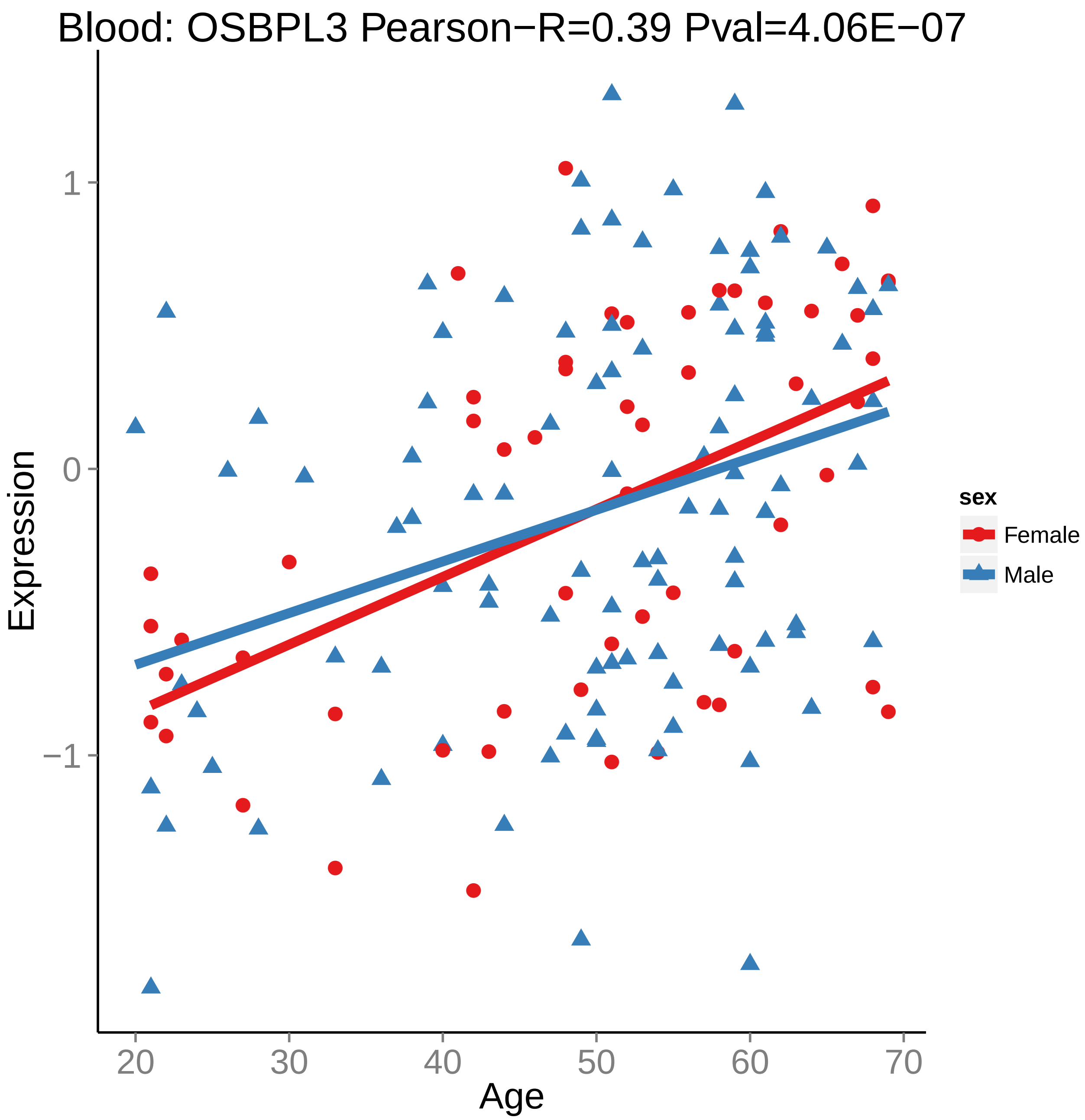
Blood: OSBPL3 Pearson−R=0.39 Pval=4.06E−07
Expression
1
0
−1
20
30
40
50
60
70
Age
sex
Female
Male

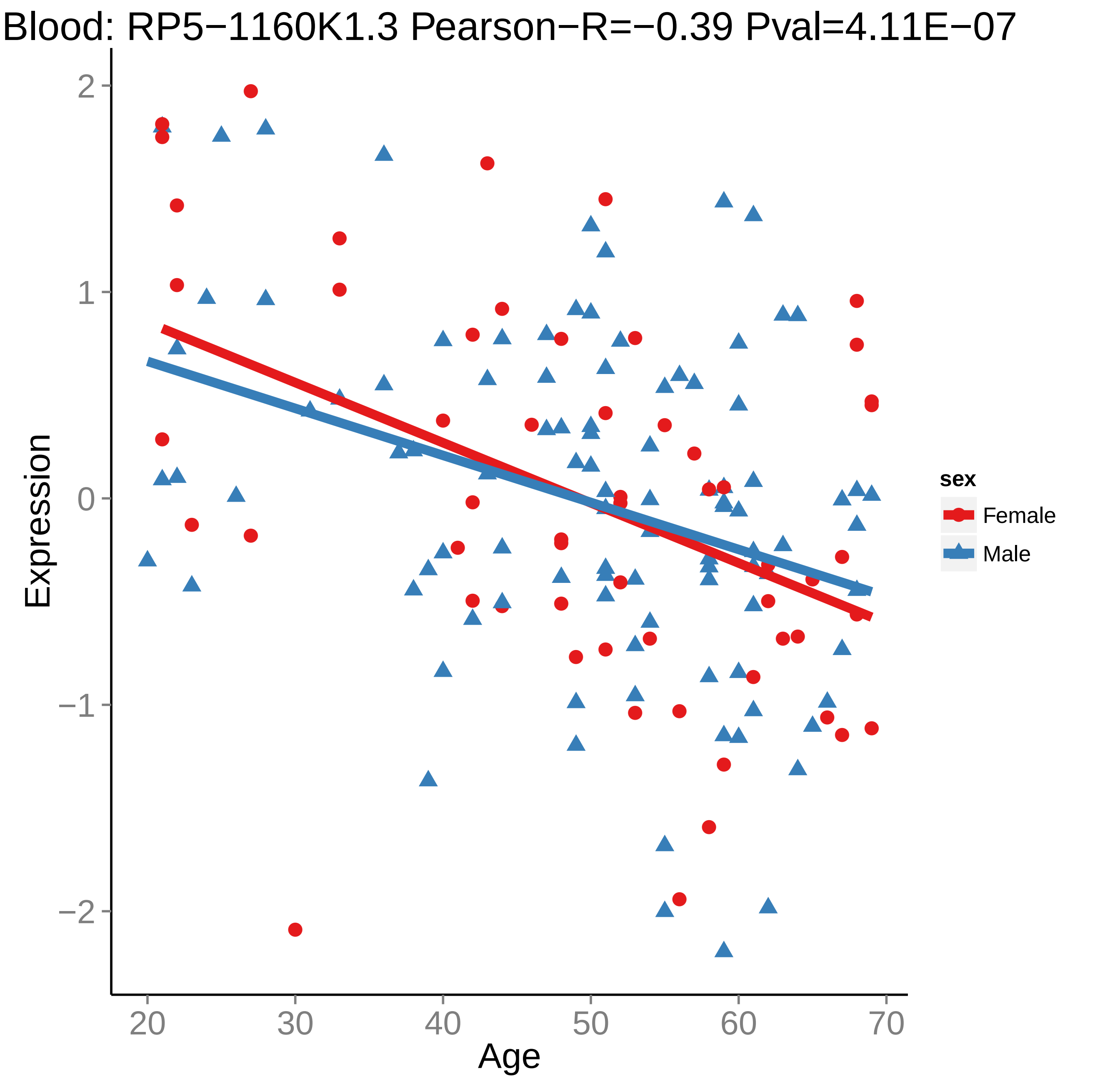

Blood: RP5−1160K1.3 Pearson−R=−0.39 Pval=4.11E−07
Expression
2
1
0
−1
−2
20
30
40
50
60
70
Age
sex
Female
Male

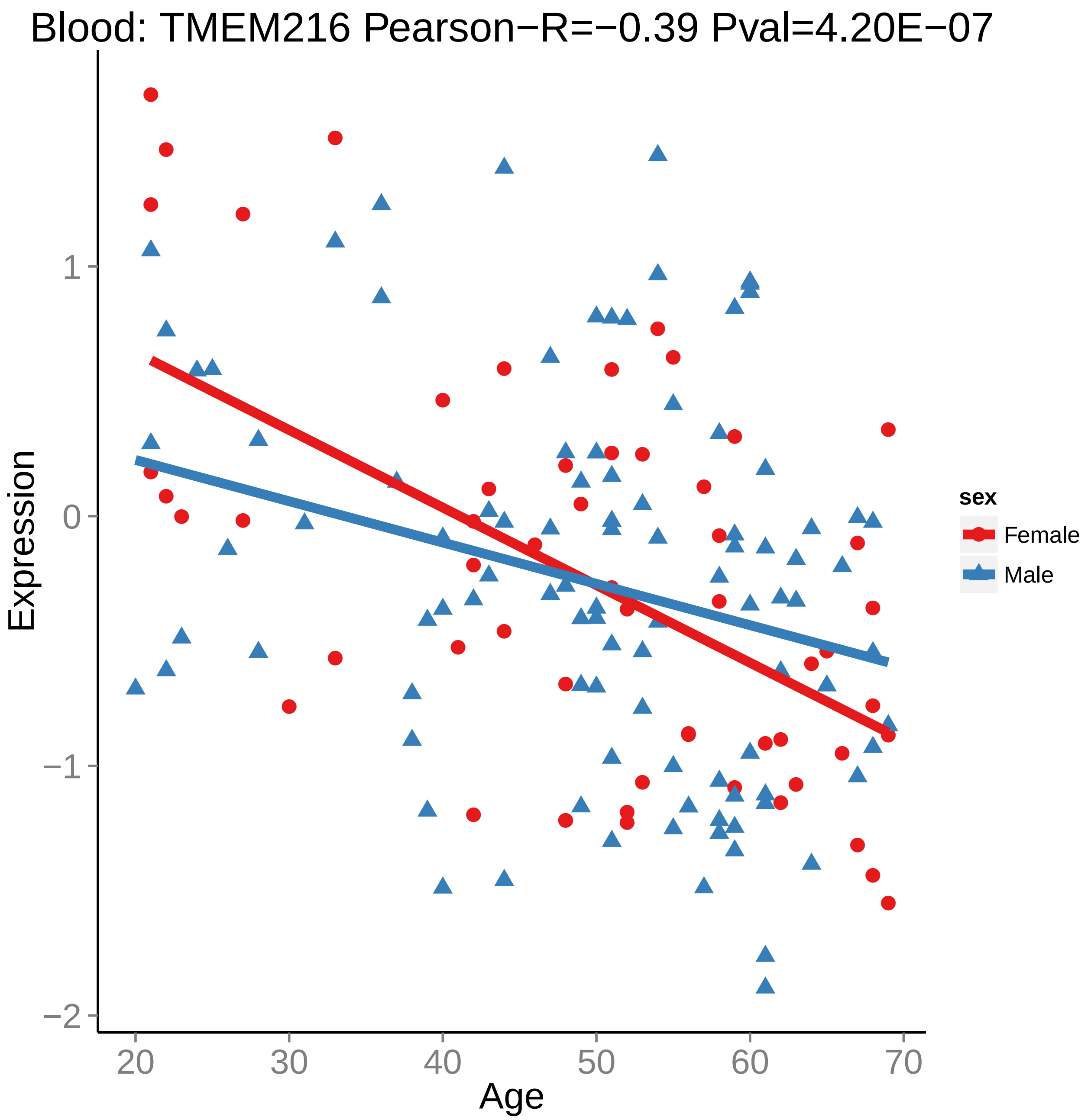
Blood: TMEM216 Pearson−R=−0.39 Pval=4.20E−07
Expression
1
0
−1
−2
20
30
40
50
60
70
Age
sex
Female
Male

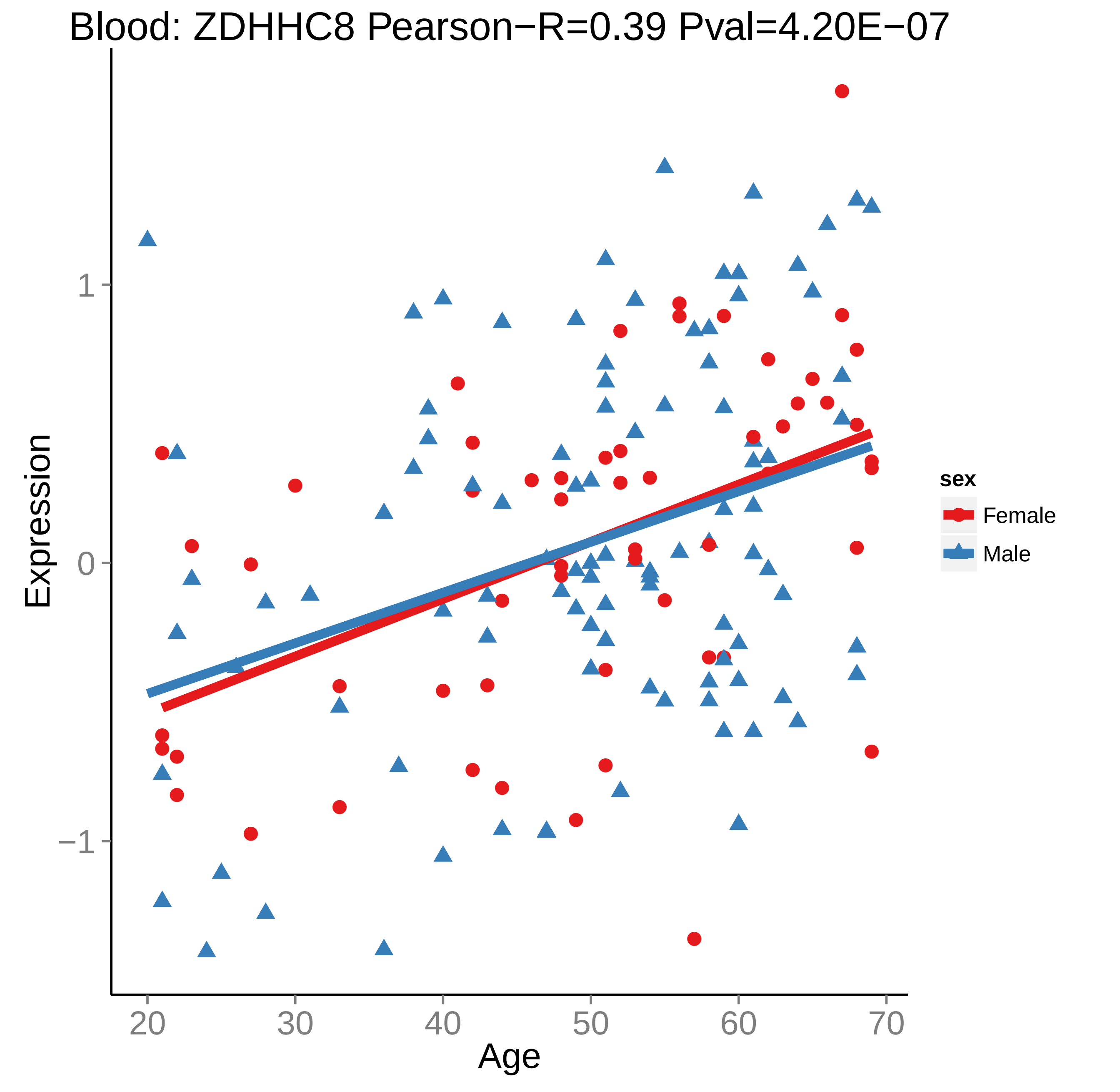
Blood: ZDHHC8 Pearson−R=0.39 Pval=4.20E−07
Expression
1
0
−1
20
30
40
50
60
70
Age
sex
Female
Male

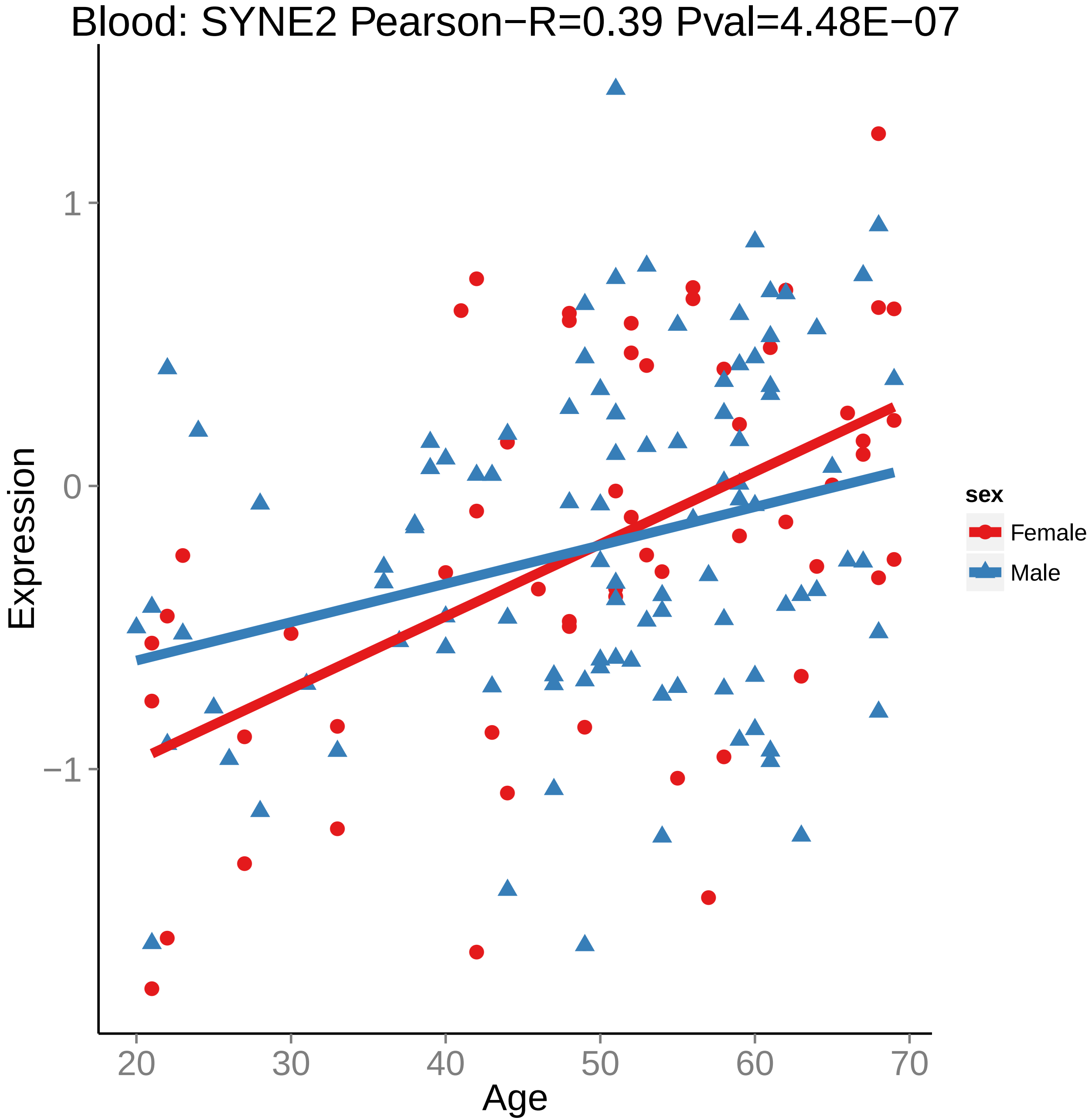
Blood: SYNE2 Pearson−R=0.39 Pval=4.48E−07
Expression
Age
1
0
−1
20
30
40
50
60
70
sex
Female
Male

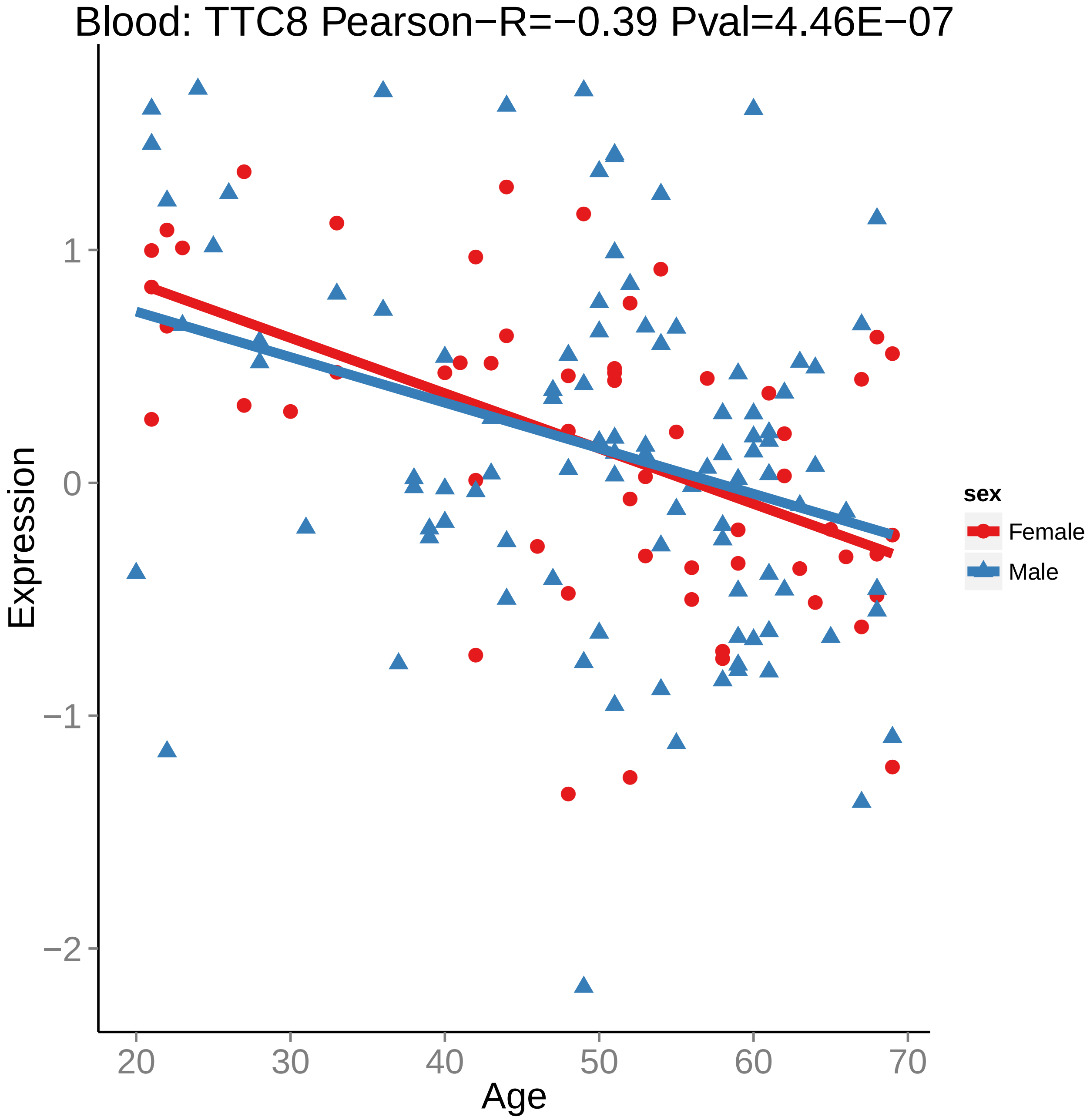

Blood: TTC8 Pearson−R=−0.39 Pval=4.46E−07
Expression
Age
1
0
−1
−2
20
30
40
50
60
70
sex
Female
Male

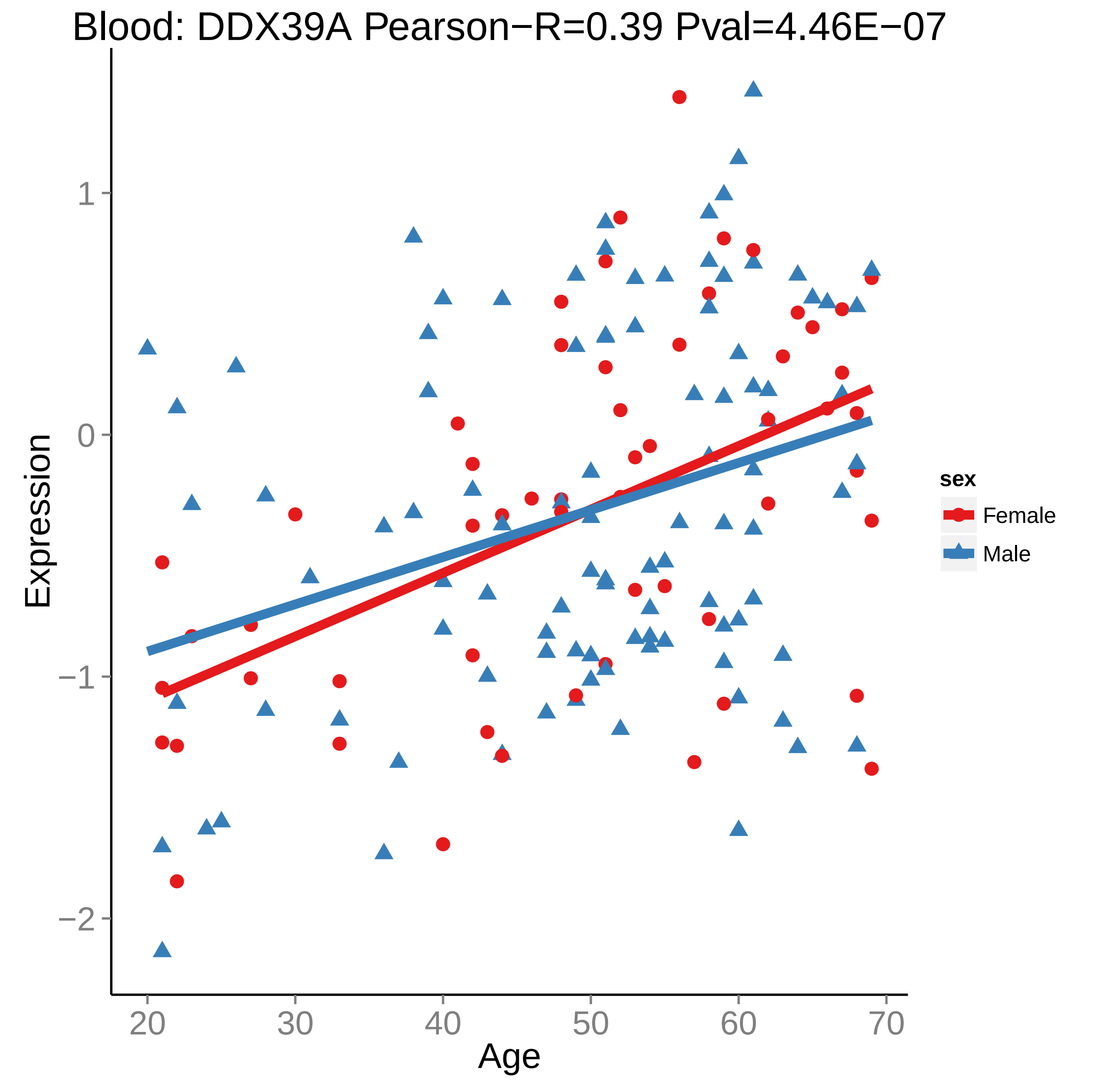

Blood: DDX39A Pearson−R=0.39 Pval=4.46E−07
Expression
Age
1
0
−1
−2
20
30
40
50
60
70
sex
Female
Male

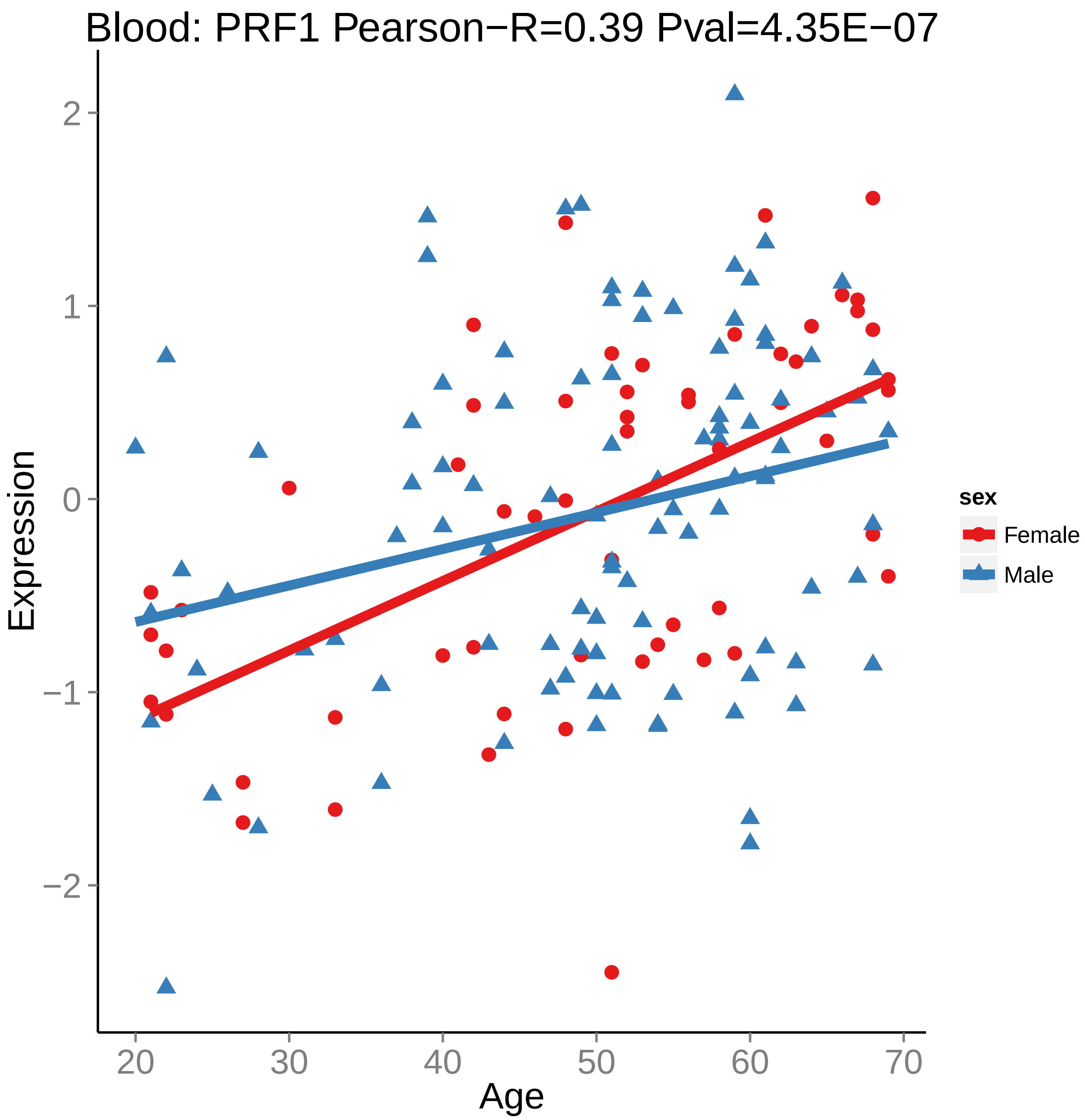
Blood: PRF1 Pearson−R=0.39 Pval=4.35E−07
Expression
2
1
0
−1
−2
20
30
40
50
60
70
Age
sex
Female
Male
